# Supplementary material for: Carbodefluorination of fluoroalkyl ketones via a carbene-initiated rearrangement strategy
Source: Nat Commun. 2022 Jul 25;13:4280. doi: 10.1038/s41467-022-31976-z (PMC9314321; doi:10.1038/s41467-022-31976-z)
Supplement: Supplementary file 1 — Supplementary Information [file 41467_2022_31976_MOESM1_ESM.pdf]

Supplementary Information for

Carbodefluorination of fluoroalkyl ketones via a carbene-initiated rearrangement strategy

Linxuan Li,†[1] Xinyu Zhang,†[1] Yongquan Ning,†[1] Xiaolong Zhang,†[1] Binbin Liu,[1] Zhansong Zhang,[1] Paramasivam Sivaguru,[1] Giuseppe Zanoni,[2] Shuang Li,[1] Edward A. Anderson[3] and Xihe Bi*[1,4]

[1] Department of Chemistry, Northeast Normal University, Changchun 130024, China. [2] Department of Chemistry, University of Pavia, Viale Taramelli 12, 27100 Pavia, Italy. [3] Chemistry Research Laboratory, University of Oxford, 12 Mansfield Road, Oxford, OX1 3TA, U.K.; [4] State Key Laboratory of Elemento-Organic Chemistry, Nankai University, Tianjin 300071, China.

E-mail: bixh507@nenu.edu.cn

Contents

Supplementary Methods........1

I. General Information........1

II. Optimization Tables........2

III. Experimental Procedure........5

IV. Substrates Ineffective for Carbodefluorination of Fluoroalkyl Ketones........13

V. X-ray Crystallographic Data and NOESY Spectrum........14

VI. Computational Details........20

VII. Characterization Data for the Products........22

VIII. Copies of ^{1}H, ^{13}C and ^{19}F Spectra........87

Supplementary References........401

Supplementary Methods

I. General information

General. All reactions dealing with air- or moisture-sensitive compounds were carried out in a flame-dried, sealed Schlenk reaction tube under an atmosphere of argon. The products were purified by column chromatography over silica gel (200-400 size). NMR spectra were recorded on a Brüker Advance 600 (^{1}H: 600 MHz, ^{13}C:150 MHz) and Brüker Advance 500 (^{1}H: 500 MHz,^{13}C: 125 MHz) at ambient temperature. The following residual solvent signals were used as references for ^{1}H and ^{13}C NMR spectra: $CDCl_3$, δH 0.00 ppm (relative to TMS), δC 77.00 ppm; DMSO-d6, δH 2.50 ppm, δC 40.45 ppm; CD_3CN, δH 1.96 ppm, δC 1.80, 118.26 ppm. ^{19}F NMR chemical shifts were determined relative to $CFCl_3$ as outside standard and low field is positive. The following abbreviations were used to explain the multiplicities: s = singlet, d = doublet, t = triplet, q = quartet, qi = quintet, m = multiplet, br = broad. Thin layer chromatographic (TLC) analysis was performed with glass-backed silica gel plates, visualizing with UV light (254 nm) and/or staining with aqueous $KMnO_4$ stain. Mass spectra were recorded on TSQ 8000 Evo by using EI method. High-resolution mass spectra (HRMS) were recorded on Bruck microTof by using ESI method (Northeast Normal University). Melting points were determined with XRC-1 and are uncorrected. IR were recorded on Nicolet 6700 spectrometer.

Materials. All reagents were purchased from commercial sources and used after waterless treatment. Catalyst $Tp^{X}Ag$ was synthesized according to the previous report[1]. Solvents were dried over CaH_2 (for DCE and $CHCl_3$) or sodium (for toluene) by refluxing for overnight and freshly distilled prior to use.

II. Optimization Tables

Supplementary Table 1. Optimization of the reaction conditions with *N*-sulfonylhydrazones and indole-3-carbinol[a]

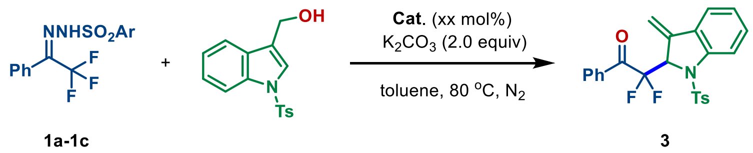

Entry	1	Cat.	Yield[b]
1	1a	AgOTf (10 mol%)	Trace
2	1a	$AgSbF_6$ (10 mol%)	15%
3	1a	$Tp^{(CF3)2}Ag$ (10 mol%)	44%
**4**	**1a**	**$Tp^{Br3}Ag$ (10 mol%)**	**84%**
5	1a	$Tp^{Br3}Cu$ (10 mol%)	30%
6	1a	CuI (10 mol%)	n.d.
7	1a	$Rh_2(OAc)_4$ (5 mol%)	n.d.
8	1a	$Pd_2(dba)_3$ (5 mol%)	n.d.
9	1a	FeTPPCl (5 mol%)	n.d.
10	1b	$Tp^{Br3}Ag$ (10 mol%)	64%
11	1c	$Tp^{Br3}Ag$ (10 mol%)	65%

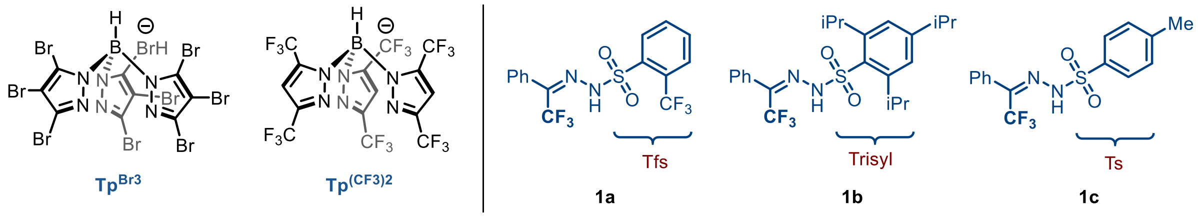

[a]Reaction conditions: **1** (0.3 mmol), indole-3-carbinol (0.6 mmol), K_2CO_3 (0.4 mmol), catalyst (10 mol%) in toluene (4 mL) at 80 °C under Ar for 16 h. [b]Isolated yields.

Supplementary Table 2. Optimization of the reaction conditions with *N*-sulfonylhydrazones and allyl alcohol[a]

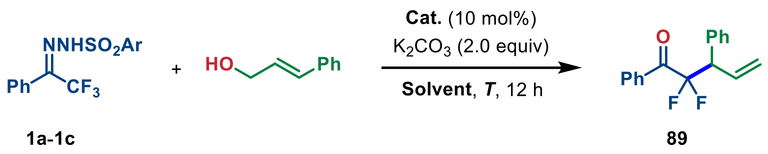

Entry	Ar	Cat.	*T*	Yield[b]
1	1a	$Tp^{Br3}Ag$	60 °C	88%
2	1b	$Tp^{Br3}Ag$	60 °C	66%
3	1c	$Tp^{Br3}Ag$	60 °C	47%
**4**	**1a**	$\mathbf{Tp^{Br3}Ag}$	**80 °C**	**99% (96%)**
5	1a	$Tp^{(CF3)2}Ag$	80 °C	65%
6	1a	AgOTf	80 °C	25%
7	1a	$Pd(OAc)_2$	80 °C	49%
8	1a	CuOAc	80 °C	trace

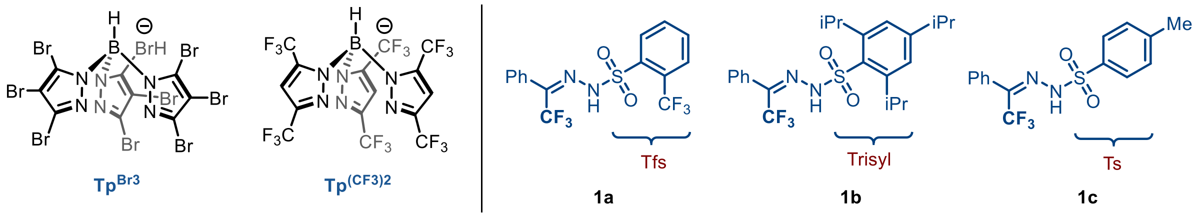

[a]Reaction conditions: **1** (0.3 mmol), allyl alcohol (0.6 mmol), K_2CO_3 (0.4 mmol) and catalyst (10 mol%) under Ar for 12 h. [b]Isolated yields.

Supplementary Table 3. Optimization of the reaction conditions with *N*-sulfonylhydrazones and propargyl alcohol[a]

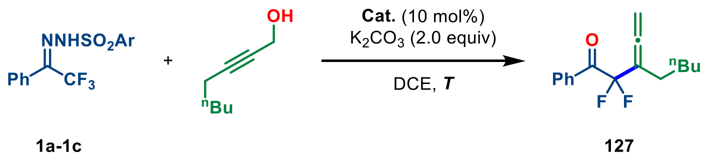

Entry	Ar	Cat. (mol%)	*T* °C	Yield (%)[b]
1	1a	$Tp^{Br3}Ag$ (10)	40 °C	73%
2	1b	$Tp^{Br3}Ag$ (10)	40 °C	61%
3	1c	$Tp^{Br3}Ag$ (10)	40 °C	35%
**4**	**1a**	**$Tp^{Br3}Ag$ (10)**	**80 °C**	**99% (95%)**
5	1a	AgOTf (10)	80 °C	70%
6	1a	$Pd(OAc)_2$ (10)	80 °C	15%
7	1a	CuOAc (10)	80 °C	34%
8	1a	$Rh_2(esp)_2$(2)	80 °C	n.d.
9	1a	FeTPPCl (2)	80 °C	n.d.

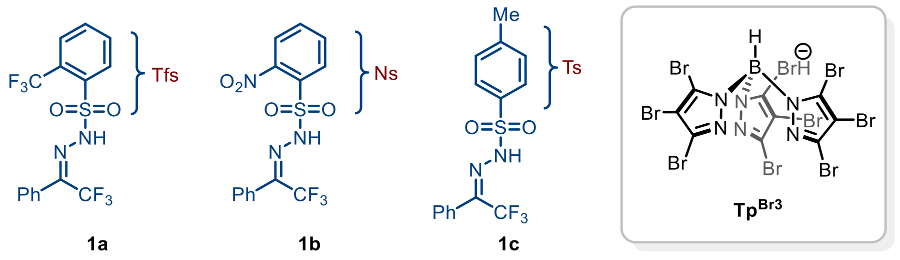

[a]Reaction conditions: **1** (0.3 mmol), propargyl alcohol (0.6 mmol), K_2CO_3 (0.6 mmol), and catalyst in DCE (4 mL) for 18 h. [b]The value in parentheses refers to the isolated yield.

III. Experimental Procedure

1. General procedure for the synthesis of products

General procedure A: Carbodefluorination reaction of indole-3-carbinols (3-18, 23-57)

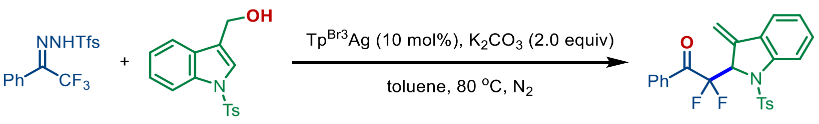

1a **3**

To a dried sealed tube was charged with *N*-tfsylhydrazone **1a** (0.3 mmol, 1.0 equiv), indole-3-carbinol (0.6 mmol, 2.0 equiv), $Tp^{Br3}Ag$ (10 mol%), K_2CO_3 (0.6 mmol, 2.0 equiv) in an argon-filled glovebox. Anhydrous toluene (4 mL) was added. The resulting mixture was stirred at 80 °C for 16 h. When the reaction was completed, the crude reaction mixture was allowed to reach room temperature, and filtered through a short pad of diatomite with ethyl acetate (EtOAc) as an eluent. The filtrate was concentrated in vacuo, and the resulting crude product was purified by column chromatography using ethyl acetate/petroleum ether (1 : 25; v : v) as the eluent to provide product **3** (84% yield).

General procedure B: Carbodefluorination reaction of 2-substituted indole-3-carbinols (19-22)

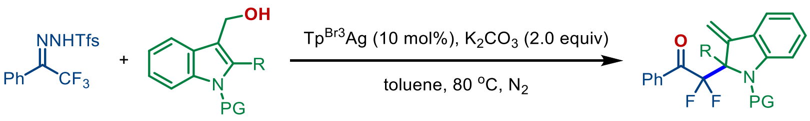

1a **19-22**

To a dried sealed tube was charged with *N*-tfsylhydrazone **1a** (0.3 mmol, 1.0 equiv), 2-substituted indole-3-carbinols (0.6 mmol, 2.0 equiv), $Tp^{Br3}Ag$ (10 mol%), K_2CO_3 (0.6 mmol, 2.0 equiv) in an argon-filled glovebox. Anhydrous toluene (4 mL) was added. The resulting mixture was stirred at 80 °C for 8 h, then the temperature was increased to 120 °C and stirring was continued for 24 h. When the reaction was completed, the crude reaction mixture was allowed to reach room temperature, and filtered through a short pad of diatomite with ethyl acetate (EtOAc) as an eluent. The filtrate was concentrated in vacuo, and the resulting crude product was purified by column chromatography using ethyl acetate/petroleum ether (1 : 25; v : v) as the eluent to provide product.

General procedure C: Carbodefluorination reaction of pyrrole and (benzo)furan carbinols (58-80)

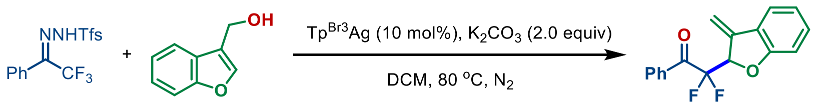

1a 79

(With 79 as an example): To a dried sealed tube was charged with *N*-tfsylhydrazone **1a** (0.3 mmol, 1.0 equiv), $Tp^{Br3}Ag$ (10 mol%), K_2CO_3 (0.6 mmol, 2.0 equiv) in an argon-filled glovebox. Anhydrous dichloromethane (DCM) (3 mL) was added, then added benzofuran-3-ylmethanol (0.9 mmol, 3.0 equiv) dissolved in DCM (1 mL). The resulting mixture was stirred at 80 °C for 12 h. When the reaction was completed, the crude reaction mixture was allowed to reach room temperature, and filtered through a short pad of diatomite with EtOAc as an eluent. The filtrate was concentrated in vacuo and then the resulting crude product was purified by column chromatography using petroleum ether as eluent to provide product **79** (90% yield). It needs to be treated in a neutral or alkaline medium, and it is easy to restore the aroma under acidic conditions.

General procedure D: Carbodefluorination reaction of (benzo)thiophene carbinols (81-85)

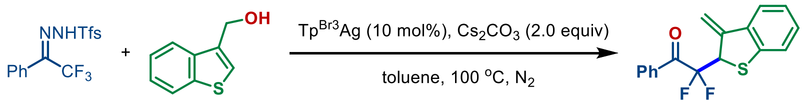

1a 85

(With 85 as an example): To a dried sealed tube was charged with *N*-tfsylhydrazone **1a** (0.3 mmol, 1.0 equiv), $Tp^{Br3}Ag$ (10 mol%), Cs_2CO_3 (0.6 mmol, 2.0 equiv) in an argon-filled glovebox. Anhydrous toluene (3 mL) was added, then added benzofuran-3-ylmethanol (0.9 mmol, 3.0 equiv) dissolved in toluene (1 mL). The resulting mixture was stirred at 100 °C for 16 h. When the reaction was completed, the crude reaction mixture was allowed to reach room temperature, and filtered through a short pad of diatomite with EtOAc as an eluent. The filtrate was concentrated in vacuo and then the resulting crude product was purified by column chromatography using petroleum ether as eluent to provide product **79** (66% yield). It needs to be treated in a neutral or alkaline medium, and it is easy to restore the aroma under acidic conditions.

General procedure E: Carbodefluorination reaction of β,γ-unsaturated alcohols (86-191)

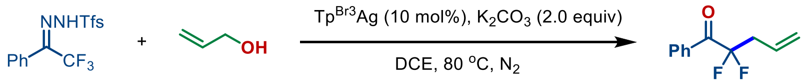

1a 86

(With 86 as an example): To a dried sealed tube was charged with *N*-tfsylhydrazone **1a** (0.3 mmol, 1.0 equiv), $Tp^{Br3}Ag$ (10 mol%), K_2CO_3 (0.6 mmol, 2.0 equiv) in an argon-filled glovebox. Anhydrous 1,2-dichloroethane (DCE) (3 mL) was added, then added allyl alcohol (0.6 mmol, 2.0 equiv) dissolved in DCE (1 mL). The resulting mixture was stirred at 80 °C for 12 h. When the reaction was completed, the crude reaction mixture was allowed to reach room temperature, and filtered through a short pad of diatomite with EtOAc as an eluent. The filtrate was concentrated in vacuo and then the resulting crude product was purified by column chromatography using petroleum ether as eluent to provide product **86** (88% yield). (The reaction time for propargyl alcohol is 18 h.)

General procedure F: Carbodefluorination reaction of alkyl-*N*-triftosylhydrazones with β,γ-unsaturated alcohols (177-182)

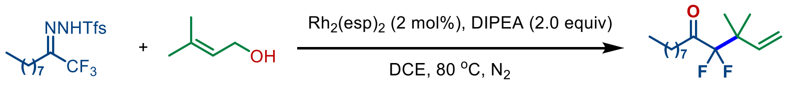

1d **177**

(With 177 as an example): To a dried sealed tube was charged with alkyl-*N*-tfsylhydrazone **1d** (0.3 mmol, 1.0 equiv), $Rh_2(esp)_2$ (2 mol%) in an argon-filled glovebox. Anhydrous 1,2-dichloroethane (DCE) (3 mL) was added, then added DIPEA (0.6 mmol, 2.0 equiv) and 3-Methyl-2-buten-1-ol (0.6 mmol, 2.0 equiv) dissolved in DCE (1 mL). The resulting mixture was stirred at 80 °C for 12 h. When the reaction was completed, the crude reaction mixture was allowed to reach room temperature, and filtered through a short pad of diatomite with EtOAc as an eluent. The filtrate was concentrated in vacuo and then the resulting crude product was purified by column chromatography using petroleum ether as eluent to provide product **177** (84% yield).

2. Gram-scale synthesis and further transformation

General procedure for the gram-scale synthesis:

For indole-3-carbinol: In a nitrogen-filled glovebox, a flame-dried screw-cap 250 mL reaction tube equipped with a Teflon-coated magnetic stir bar was charged with *N*-triftosylhydrazone **1a** (10.0 mmol), indole-3-carbinol (20.0 mmol), K_2CO_3 (20.0 mmol) and $Tp^{Br3}Ag$ (10 mol%). Anhydrous toluene (100 mL) was added and the reaction mixture was stirred at 80 °C for 16 h, until the reaction was complete as indicated by TLC. The reaction crude was filtered through a short silica gel eluting with EtOAc. The filtrate was

concentrated in vacuo, and the resulting crude product was purified by column chromatography using ethyl acetate/petroleum ether as the eluent to afford the pure product **3**.

For allyl or propargyl alcohol: In a nitrogen-filled glovebox, a flame-dried screw-cap 250 mL reaction tube equipped with a Teflon-coated magnetic stir bar was charged with *N*-triftosylhydrazone **1a** (5.0 mmol), 2-propen-1-ol or 2-octyn-1-ol (10.0 mmol), K_2CO_3 (10.0 mmol) and $Tp^{Br3}Ag$ (10 mol%). Anhydrous 1,2-dichloroethane (DCE) (40 mL) was added and the reaction mixture was stirred at 80 °C for 18 h, until the reaction was complete as indicated by TLC. The reaction crude was filtered through a short silica gel eluting with DCM. The filtrate was evaporated under reduced pressure to leave a crude mixture, which was purified by flash column chromatography to afford the pure product.

General procedure G: Synthesis of 192 and 193 (Cyclopropanation)[2]

For formyl cyclopropanation 192: A screw capped reaction vial was charged with DFHZ-Tfs (0.4 mmol, 1.0 equiv), FeTPPCl (3 mol%), then evacuated and filled with N_2 for three times, then toluene (1 mL) which dissolved with **3** (0.2 mmol, 1.0 equiv) and NaOH aq. (2 mL, 5.0 wt%) was successively added by syringe. The reaction was stirred at 60 ºC for 24 h. Then 10 mL water was added to the mixtureand layers partitioned. The aqueous layer was extracted with DCM and the organic layer was combined and dried with anhydrous Na_2SO4, then filtered through a short silica gel eluting with DCM. The filtrate was evaporated under reduced pressure to leave a crude mixture, which was purified through silica gel flash column chromatography to give the final product.

For difluormethyl cyclopropanation 193: In a nitrogen-filled glovebox, a flame-dried screw-cap reaction tube equipped with a magnetic stir bar was charged with TFHZ-Tfs (0.4 mmol, 1.0 equiv), K_2CO_3 (0.6 mmol, 3.0 equiv), FeTPPCl (3 mol%). The solution of **3** (0.2 mmol, 1.0 equiv) in dry 1,4-dioxane (3 mL) was added. The tube was sealed with a screw cap equipped with a septum, removed from the glovebox. The reaction mixture was stirred at 40 ºC for 24 h. The reaction crude was filtered through a short pad of celite and washed with EtOAc. The filtrate was evaporated under reduced pressure to leave a crude mixture, which was purified through silica gel flash column chromatography eluting with n-hexane to give the final product.

General procedure H: Synthesis of 194, 200, 205 (Carbonyl reduction)

Dissolve the ketone (0.2 mmol, 1.0 equiv) in methanol (2 mL), stir at 0°C, and then slowly add sodium borohydride (0.24 mmol, 1.2 equiv). The reaction was monitored by TLC. After the reaction, the mixture

was quenched with saturated ammonium chloride and then partitioned between water and EtOAc. The combined organic extracts were dried ($MgSO_4$) and concentrated. The crude reaction product was purified by flash column chromatography to obtain a pure product.

Synthesis of 195 (Aromatization):

Under a nitrogen atmosphere, the ketone (0.2 mmol, 1.0 equiv) was dissolved in acetonitrile (2 mL), and NaI (0.32 mmol), TMSCl (0.32 mmol) and H_2O (0.16 mmol) were added sequentially, and then the mixed system was stirred at room temperature for 10 h. After the reaction, the mixture was quenched with saturated ammonium chloride and then partitioned between water and EtOAc. The combined organic extracts were dried ($MgSO_4$) and concentrated. The crude reaction product was purified by flash column chromatography to obtain a pure product.

Synthesis of 196[3]:

In a flame-dried 8 mL reaction vial, carbodefluorination product **3** (0.2 mmol, 1.0 equiv) was dissolved in CH_2Cl_2 (2 mL) and cooled to 0°C. *N*-bromosuccinimide (0.22 mmol, 1.1 equiv) was added to the solution, allowed to warm to 23 °C and stirred for 4 h. The reaction mixture was concentrated by rotary evaporation and the crude product was purified by silica gel column chromatography (6:1 hexanes/EtOAc) to give **196** (74% yield) as a brown semi-solid.

General procedure I: Synthesis of 197, 202, 207 (Carbonyl alkenylation)

Potassium *tert*-butoxide (0.3 mmol, 1.5 equiv) in dry THF (0.5 mL) was added to a suspension of Ph_3PCH_3Br (0.3 mmol, 1.5 equiv) in 1 mL of dry THF at 0 °C. The external cooling was removed, and the mixture was stirred for 30 min. The ketone (0.2 mmol, 1.0 equiv) was added, and the solution was stirred at room temperature for 5 h. The mixture was quenched with 1 N HCl and then partitioned between water and EtOAc. The combined organic extract was dried ($MgSO_4$) and concentrated, the reaction crude was purified by flash column chromatography to afford the pure product.

General procedure for the synthesis of 198:

Potassium tert-butoxide (0.3 mmol, 1.5 equiv) in dry THF (0.5 mL) was added to a suspension of Ph_3PCH_3Br (0.3 mmol, 1.5 equiv) in 1 mL of dry THF at 0 °C. The external cooling was removed, and the mixture was stirred for 30 min. The ketone (0.2 mmol, 1.0 equiv) was added, and the solution was stirred at room temperature for 5 h. Then raise the temperature to 60°C and continue to react for 12 h. The mixture was

quenched with 1 N HCl and then partitioned between water and EtOAc. The combined organic extract was dried ($MgSO_4$) and concentrated, the reaction crude was purified by flash column chromatography to afford the pure product.

General procedure J: Synthesis of 199, 201, 204, 206 (DAST fluorination)

In a nitrogen-filled glovebox, a flame-dried reaction tube equipped with a Teflon-coated magnetic stir bar was charged with carbonyl or hydroxyl substrate (0.2 mmol) and DCM (2 mL). The mixture was cooled to -78 °C, and the DAST (0.2 mmol, 1.0 equiv) was added dropwise. Then the reaction mixture was stired at room temperature. Until the reaction was complete as indicated by TLC, the mixture was quenched with iced-water. The organic layer was washed with sat. $NaHCO_3$ solution, water and dried over $MgSO_4$. Then the solvent was evaporated under reduced pressure to leave a crude mixture, which was purified by flash column chromatography to afford the pure product.

Synthesis of 208:

To a nitrogen-filled flame-dried reaction tube equipped with a Teflon-coated magnetic stir bar was charged with **127** (0.2 mmol), AIBN (0.04 mmol), ethyl bromomethacrylate (0.4 mmol, 2.0 equiv) and toluene (2 mL). The reaction mixture was stirred at 80 °C for 1.5 h, until the reaction was complete as indicated by TLC. The reaction crude was evaporated under reduced pressure, and purified by flash column chromatography to afford the pure product.

3. General procedure for the mechanismtic investigation.

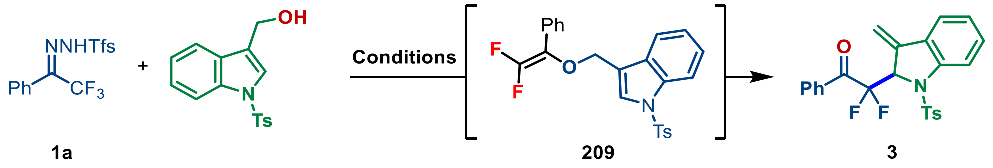

Reaction kinetics study: To a dried sealed tube was charged with *N*-tfsylhydrazone **1a** (119 mg, 0.3 mmol, 1.0 equiv), (1-tosyl-1*H*-indol-3-yl)methanol (181 mg, 0.6 mmol, 2.0 equiv), $Tp^{Br3}Ag$ (33 mg, 10 mol%), K_2CO_3 (83 mg, 0.6 mmol, 2.0 equiv) in an argon-filled glovebox. Anhydrous toluene (4 mL) were added. The resulting mixture was stirred at 80 °C. By monitoring the intermediates and products at different times of the reaction, it was found that the intermediates were produced rapidly within the first hour of the reaction. After 16 h of the reaction, the intermediate conversion was almost complete. The whole reaction kinetics process was monitored by **^{1}H NMR**.

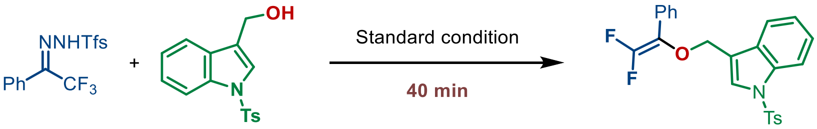

1a **209**, 62%

Capture reaction intermediates: To a dried sealed tube was charged with *N*-tfsylhydrazone **1a** (118.8 mg, 0.3 mmol, 1.0 equiv), (1-tosyl-1*H*-indol-3-yl)methanol **2** (181 mg, 0.6 mmol, 2.0 equiv), $Tp^{Br3}Ag$ (33 mg, 10 mol%), K_2CO_3 (83 mg, 0.6 mmol, 2.0 equiv) in an argon-filled glovebox. Anhydrous toluene (4 mL) were added. The resulting mixture was stirred at 80 °C for 40 min. The crude reaction mixture was allowed to reach room temperature and filtered through a short pad of celite using EtOAc as the eluent. The filtrate was concentrated in vacuo, and the resulting crude product was purified by column chromatography using EtOAc/petroleum ether (1:25; v:v) as the eluent to provide product **209** (62% yield).

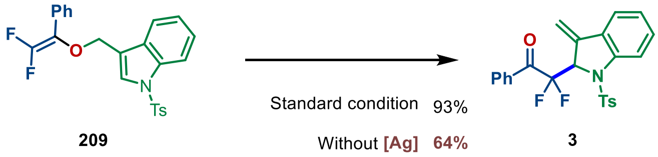

209 **3**

Control experiment: To a dried sealed tube was charged with difluoroalkenyl ether intermediate **209** (88 mg, 0.2 mmol, 1.0 equiv), $Tp^{Br3}Ag$ (22 mg, 10 mol%), K_2CO_3 (55 mg, 0.4 mmol, 2.0 equiv) in an argon-filled glovebox. Anhydrous toluene (4 mL) were added. The resulting mixture was stirred at 80 °C for 10 h. The crude reaction mixture was allowed to reach room temperature and filtered through a short pad of celite using EtOAc as the eluent. The filtrate was concentrated in vacuo, and the resulting crude product was purified by column chromatography using EtOAc/petroleum ether as the eluent to provide product **3** (93% yield).

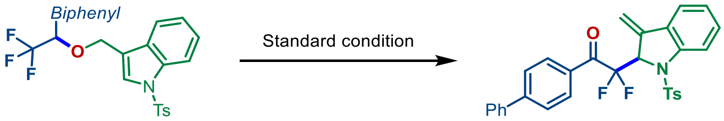

210 **44**, 0%

Intermediate verification experiment: To a dried sealed tube was charged with **210** (54 mg, 0.1 mmol, 1.0 equiv), $Tp^{Br3}Ag$ (11 mg, 10 mol%), K_2CO_3 (28 mg, 0.2 mmol, 2.0 equiv) in an argon-filled glovebox. Anhydrous toluene (2 mL) were added. The resulting mixture was stirred at 80 °C for 24 h. The target product is not detected by ^1H NMR.

[3,3] Rearrangement of *gem*-dichloroalkenyl ethers: *gem*-Dichloroalkenyl ether was synthesized according to the previous report[4].

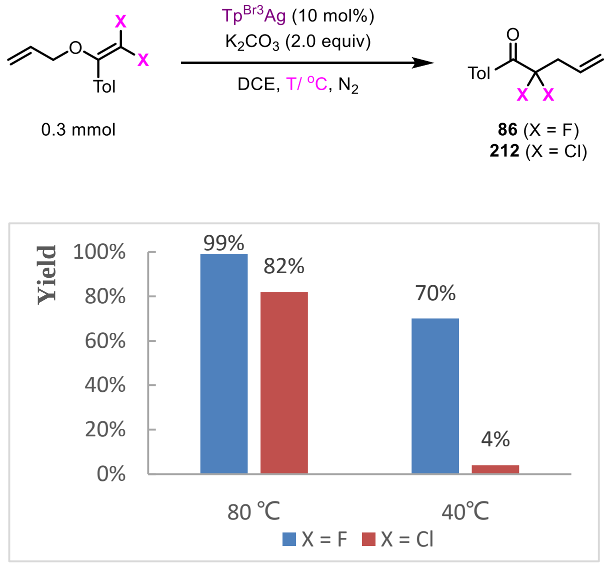

Supplementary Fig. 1. Reactivity of alkenyl ethers substituted by fluorine and chlorine atoms.

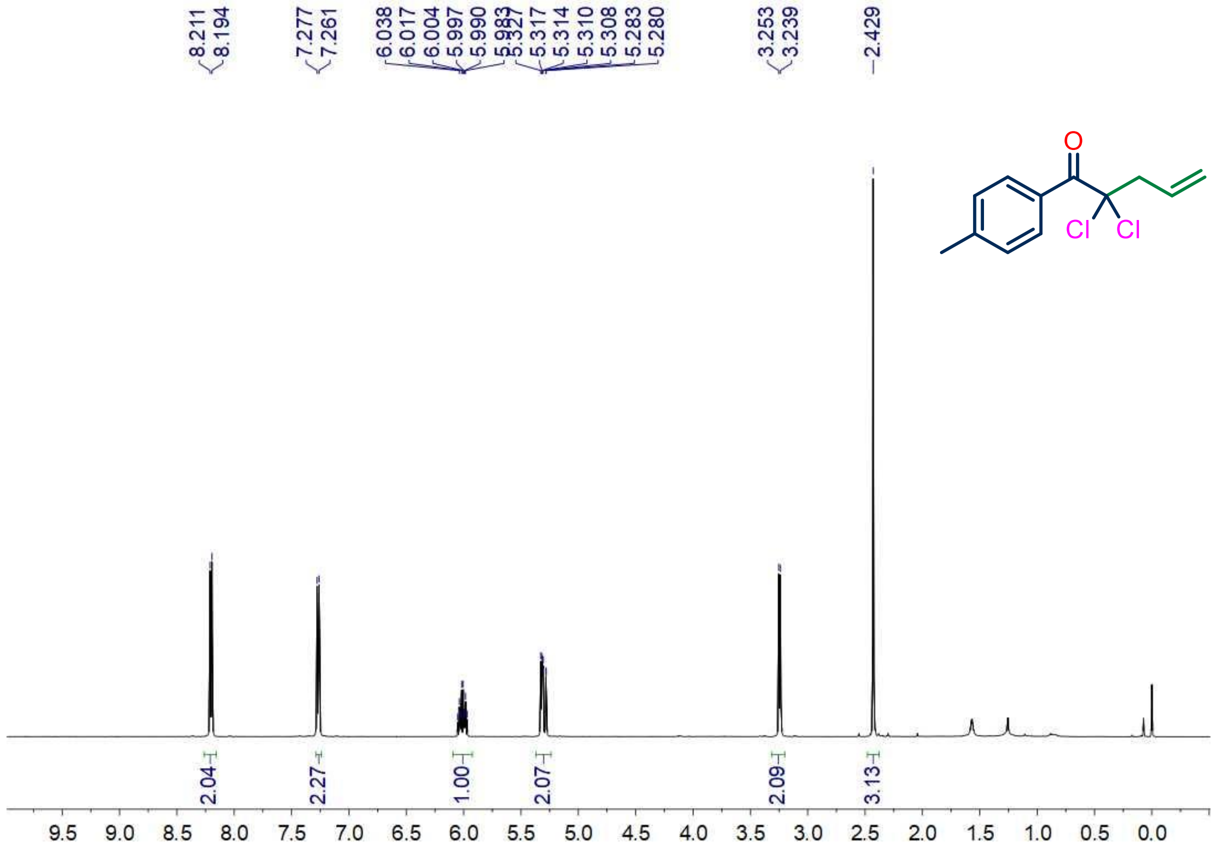

Supplementary Fig. 2. ^{1}H NMR (500 MHz, $CDCl_3$) spectrum of compound **212**

IV. Substrates Ineffective for Carbodefluorination of Fluoroalkyl Ketones

Benzyl alcohol

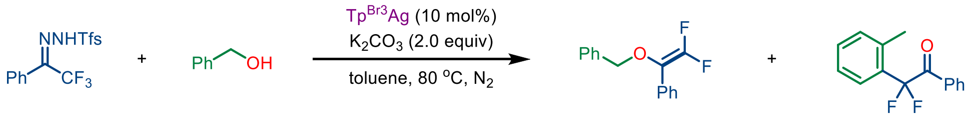

1a, 0.3 mmol 2.0 equiv **213**, 92% **214**, 0%

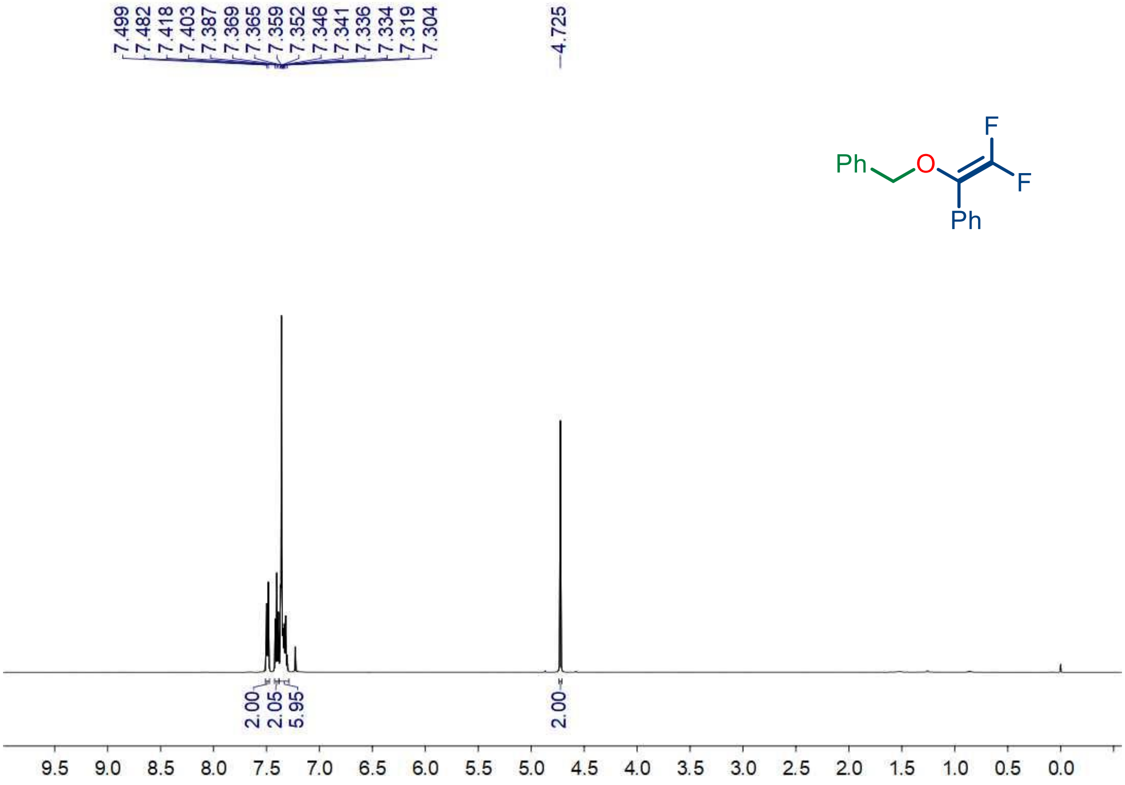

Supplementary Fig. 3. ^{1}H NMR (500 MHz, $CDCl_3$) spectrum of compound **213**

Tertiary alcohols

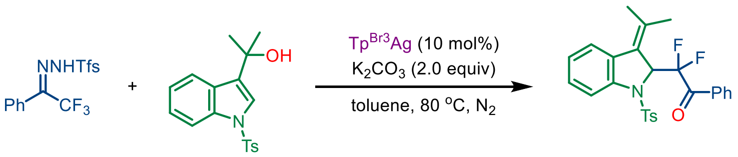

1a, 0.6 mmol 0.3 mmol **215**, <5%

Reaction of alkyl *N*-trifluoromethylhydrazones and 3-indolylcarbinols

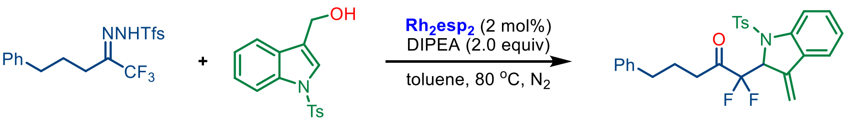

1b, 0.6 mmol 0.3 mmol **216**, 0%

V. X-ray Crystallographic data and NOESY Spectrum

1. Supplementary Table 4. X-ray Crystallographic Data of 29

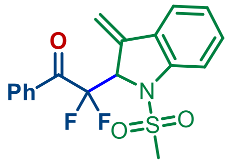

29 (CCDC 2121100)

≡

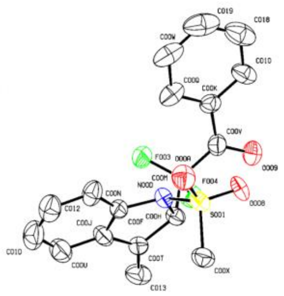

Empirical formula	$C_{18}H_{15}F_2NO_3S$
Temperature	293 K
Formula weight	363.37
Unit cell dimensions	a = 8.4172(5) Å alpha = 93.660(7) deg<br>b = 8.4704(6) Å beta = 91.349(6) deg.<br>c = 23.728(2) Å gamma = 100.577(6) deg.
Volume	1658.5(2) $Å^3$
Z	4
ρcalc	1.455 g/cm^3
μ/mm^{-1}	0.234
F(000)	752
Crystal size	0.1 x 0.1x 0.1 m
Radiation	MoKα (λ = 0.71073 Å)
2θrange for data collection	7.08 to 58.838/^{o}
Reflections collected	12195
Independent reflections	7484[R_{int} = 0.0502, R_{sigma} = 0.0962]
Data/restraints/parameters	7484/0/467
Goodness-of-fit on F^2	1.073
Final R indexes [I>=2σ (I)]	R_1 = 0.1095, wR_2 = 0.2733
Final R indexes [all data]	R_1 = 0.1509, wR_2 = 0.3110

Product **29** was crystallized as a colourless crystal via vaporization of a hexane/ EtOAc solution, and its structure was determined by x-ray structure analysis. The crystallographic data that can be obtained free of charge from The Cambridge Crystallographic Data Centre via www.ccdc.cam.ac.uk/data request/cif.

2. NOESY Spectrum

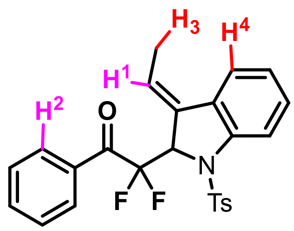

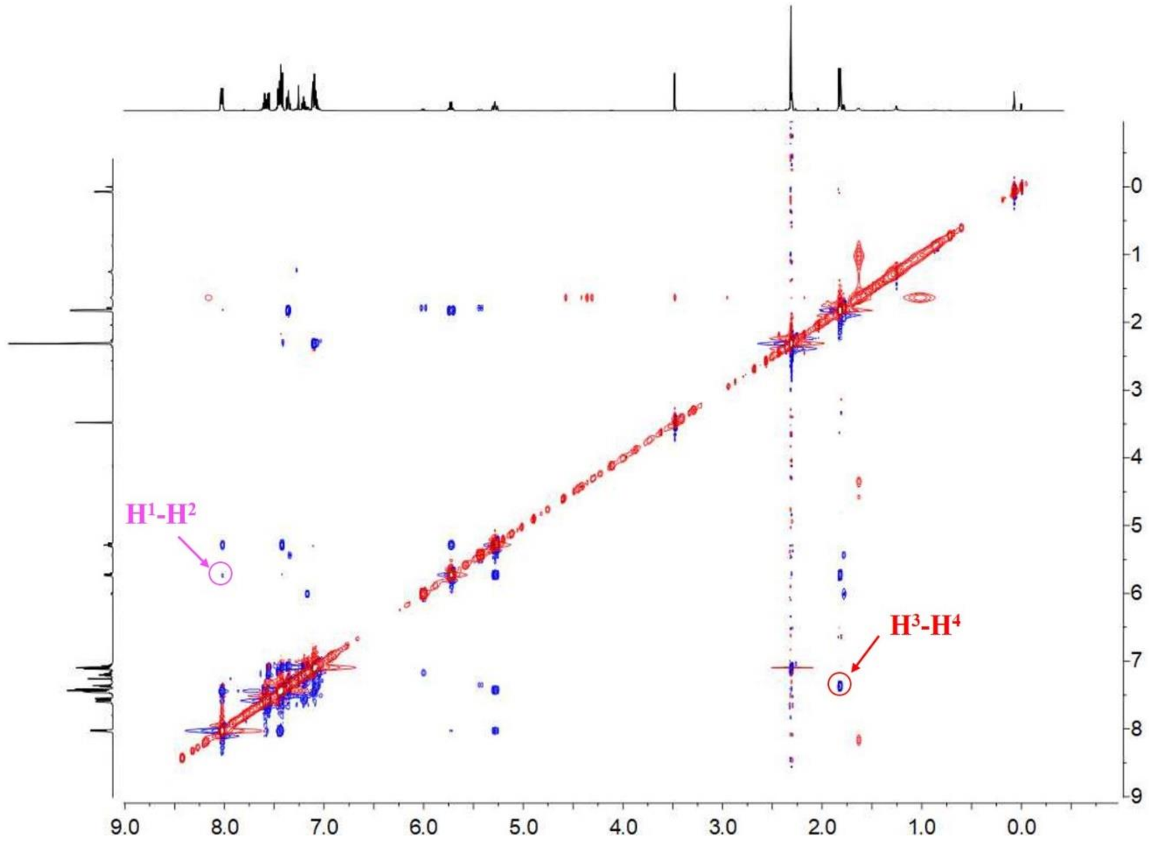

Supplementary Fig. 4. NOESY Spectrum of compound 13.

From the NOESY experiment of compound **13**, we observed the correlation peaks between H^1 proton and H^2 proton in the NOESY spectrum. Thus, indicating the H^1 and H^2 are spatially close. In addition, we also observed correlation peaks between H^3 proton and H^4 proton in the NOESY spectrum. Therefore, it shows that H^3 and H^4 are close in space. This proves that the space between the H^1 and the H^4 is far away, and the product has a trans structure.

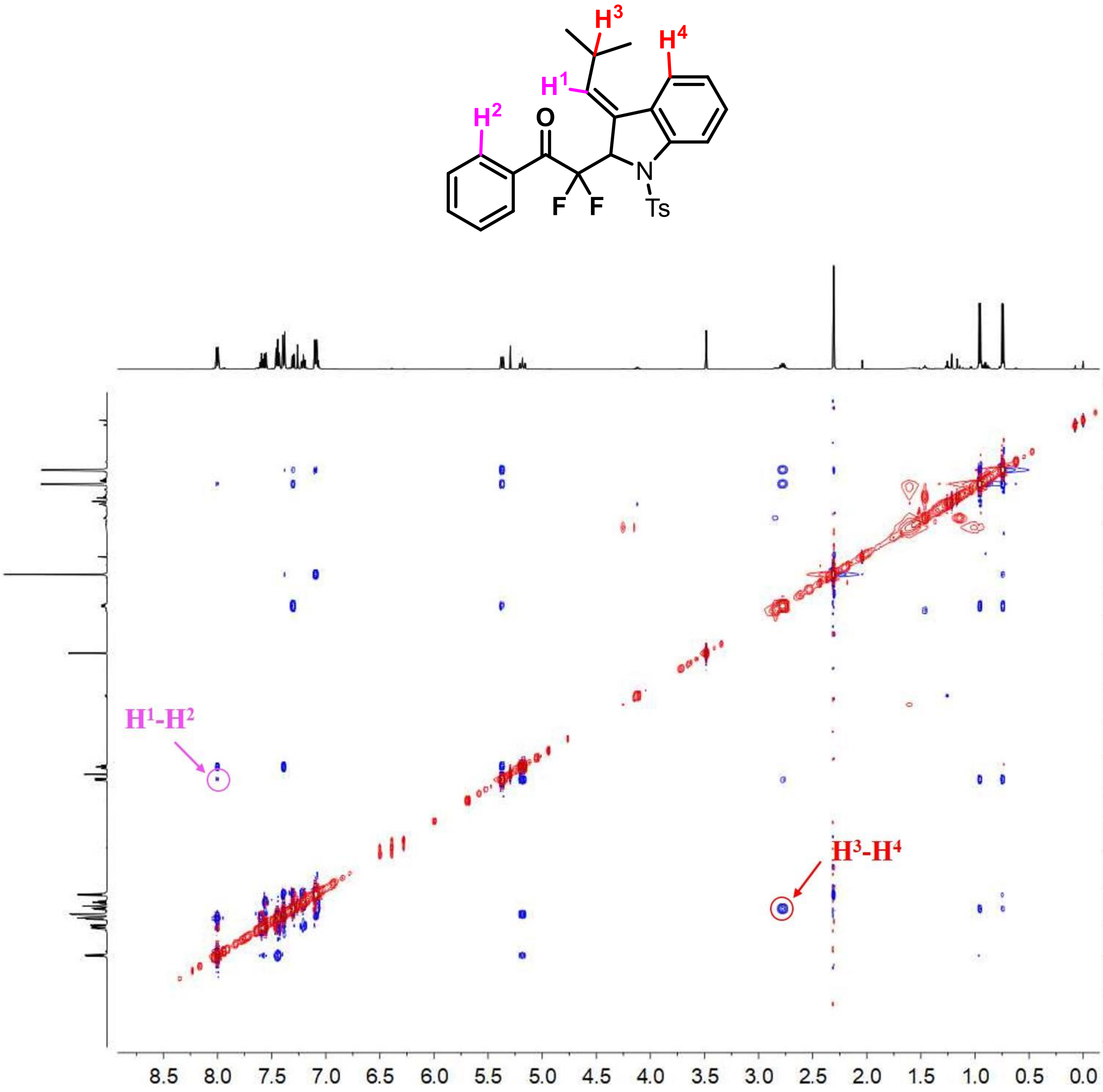

Supplementary Fig. 5. NOESY Spectrum of compound 14.

From the NOESY experiment of compound **14**, we observed the correlation peaks between H^1 proton and H^2 proton in the NOESY spectrum. Thus, indicating the H^1 and H^2 are spatially close. In addition, we also observed correlation peaks between H^3 proton and H^4 proton in the NOESY spectrum. Therefore, it shows that H^3 and H^4 are close in space. This proves that the space between the H^1 and the H^4 is far away, and the product has a trans structure.

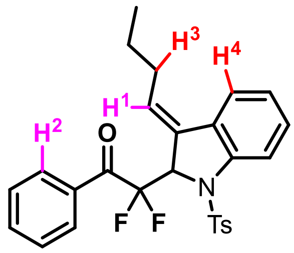

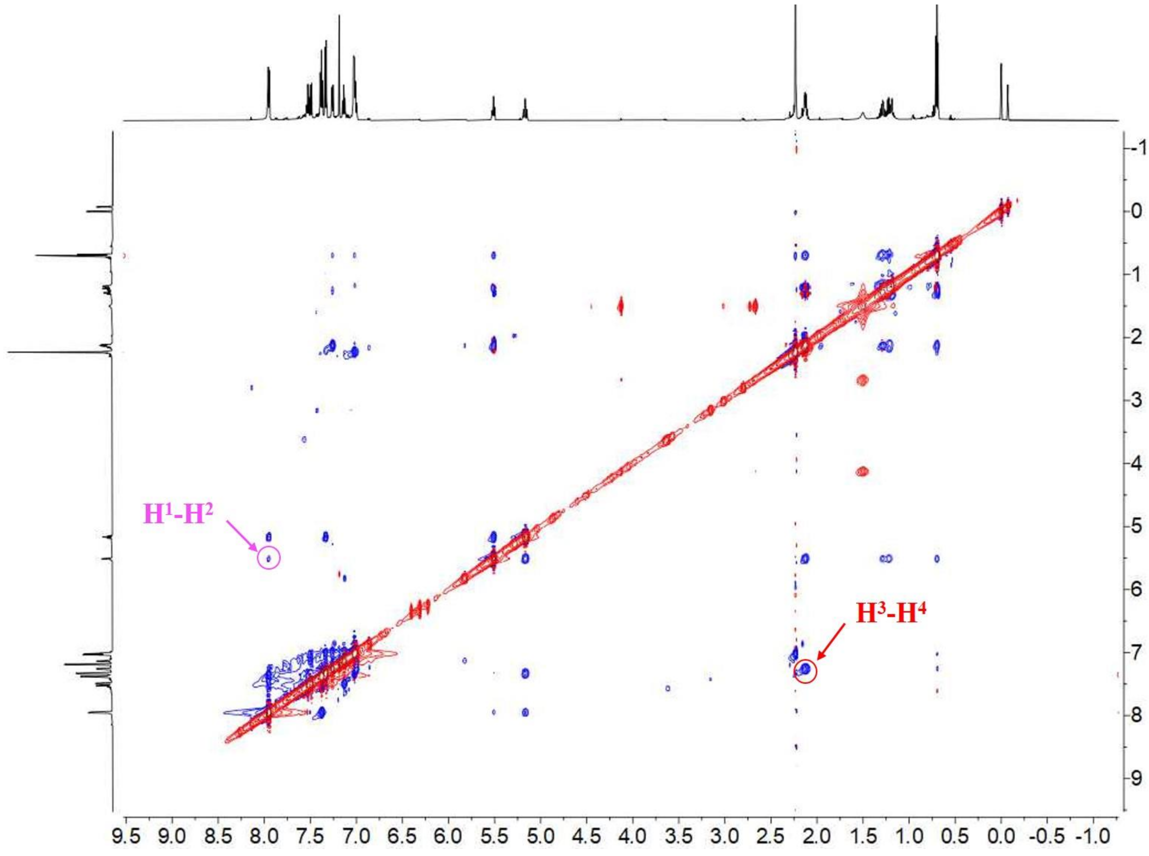

Supplementary Fig. 6. NOESY Spectrum of compound 15.

From the NOESY experiment of compound **15**, we observed the correlation peaks between H^1 proton and H^2 proton in the NOESY spectrum. Thus, indicating the H^1 and H^2 are spatially close. In addition, we also observed correlation peaks between H^3 proton and H^4 proton in the NOESY spectrum. Therefore, it shows that H^3 and H^4 are close in space. This proves that the space between the H^1 and the H^4 is far away, and the product has a trans structure.

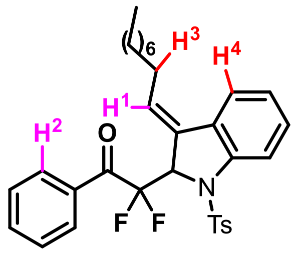

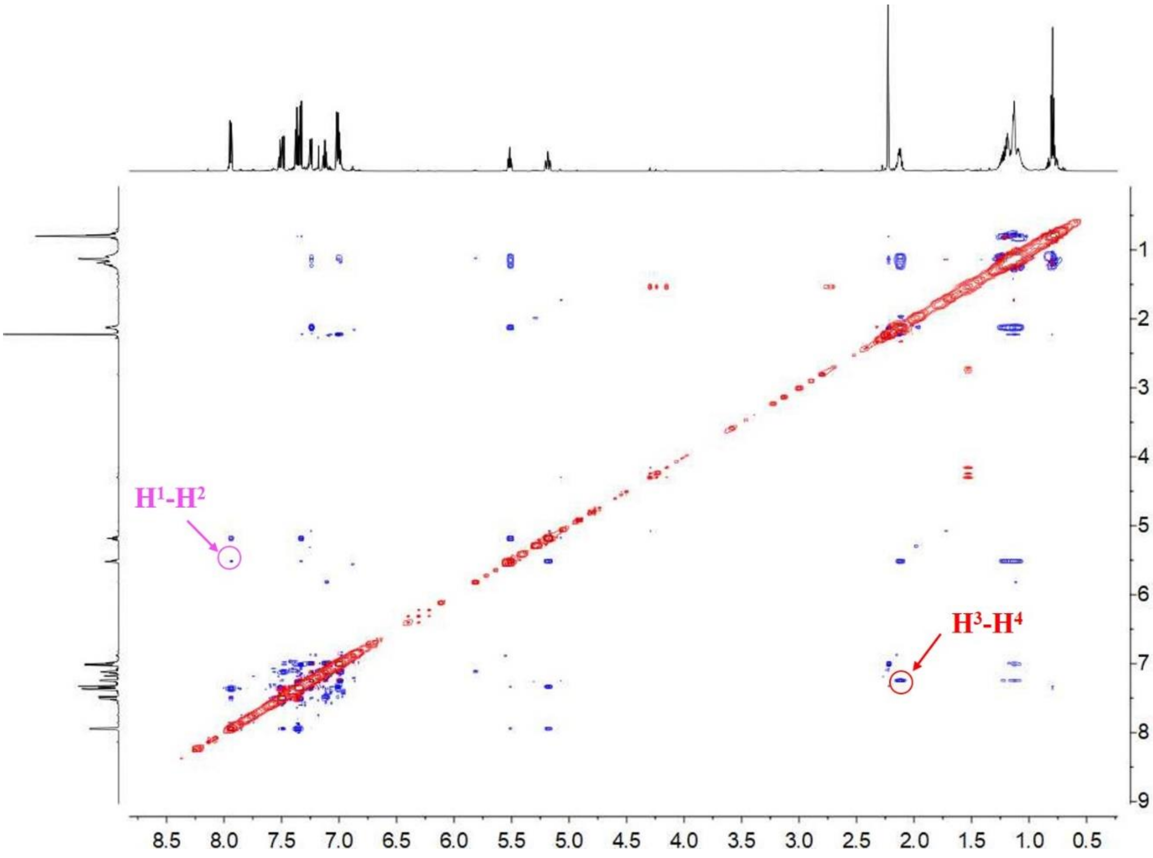

Supplementary Fig. 7. NOESY Spectrum of compound 16.

From the NOESY experiment of compound **16**, we observed the correlation peaks between H^1 proton and H^2 proton in the NOESY spectrum. Thus, indicating the H^1 and H^2 are spatially close. In addition, we also observed correlation peaks between H^3 proton and H^4 proton in the NOESY spectrum. Therefore, it shows that H^3 and H^4 are close in space. This proves that the space between the H^1 and the H^4 is far away, and the product has a trans structure.

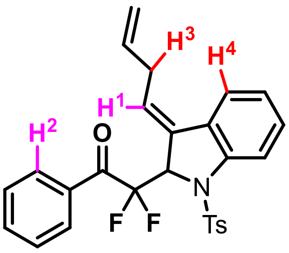

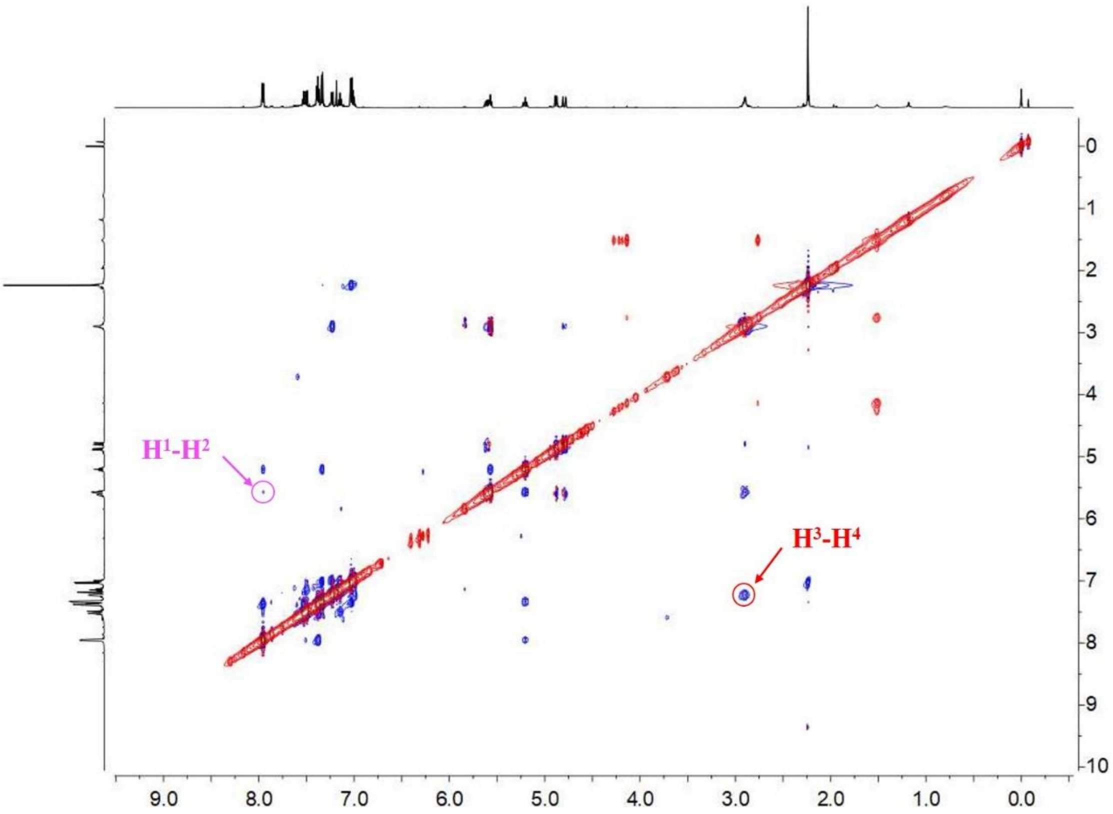

Supplementary Fig. 8. NOESY Spectrum of compound 18.

From the NOESY experiment of compound **18**, we observed the correlation peaks between H^1 proton and H^2 proton in the NOESY spectrum. Thus, indicating the H^1 and H^2 are spatially close. In addition, we also observed correlation peaks between H^3 proton and H^4 proton in the NOESY spectrum. Therefore, it shows that H^3 and H^4 are close in space. This proves that the space between the H^1 and the H^4 is far away, and the product has a trans structure.

VI. Computational details

1. Theoretical methodology:

All DFT theoretical calculations have been carried out using the Gaussian 16 program package[5]. Geometry optimizations were conducted in the framework of the density functional theory (DFT) at the B3LYP [6,7] level. The effective core potential SDD[8] basis set was used to represent Ag and Br atoms, all the other atoms (C, H, O, N etc.) were described with 6-31G(d, p) basis set.[9-11] .Final energies were retrieved from single-point calculations at the B3LYP/DEF2TZVP2 level[12] including the D3BJ dispersion correction[13] scheme developed by Grimme. All structures have been optimized considering solvent effects using the SMD[14] model for toluene. Reaction paths were traced by the intrinsic reaction coordinate [15,16] method for all transition states. All energetics reported throughout the text are in kcal/mol. 3D structures were generated using CYLview visualization software.[17]

2. HF elimination vs proton transfer

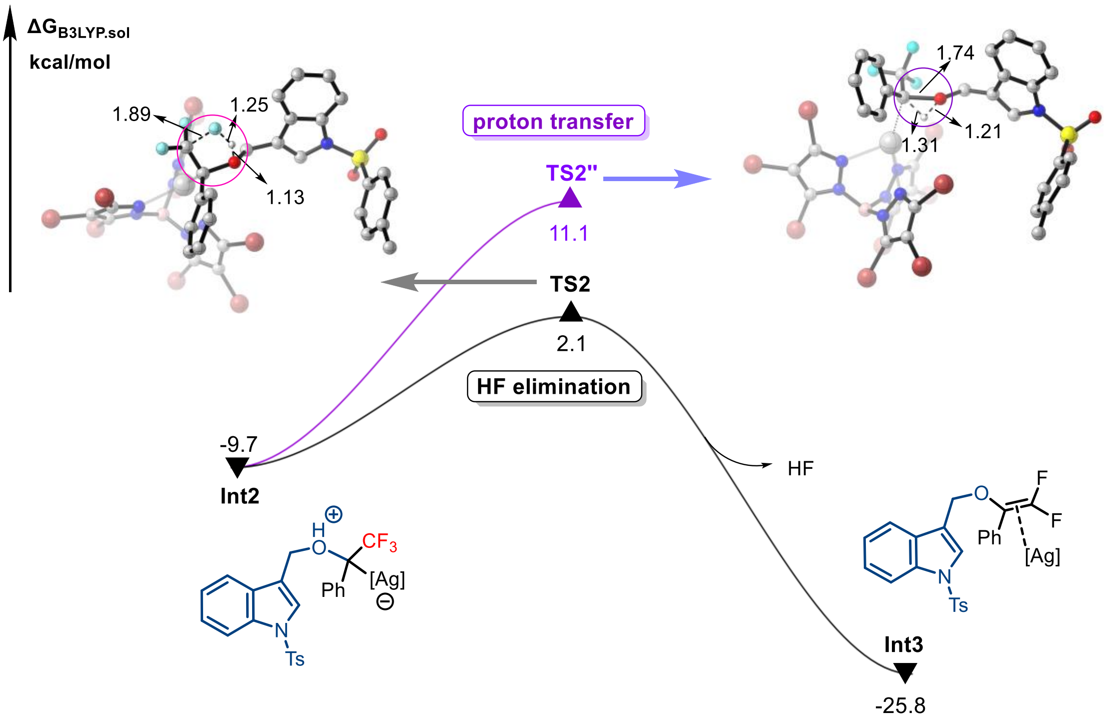

Supplementary Fig. 9. Gibbs-free energy profile for the comparision of HF elimination and proton transfer pathways, distances are given in angstroms.

3. RDG isosurface for TS3'

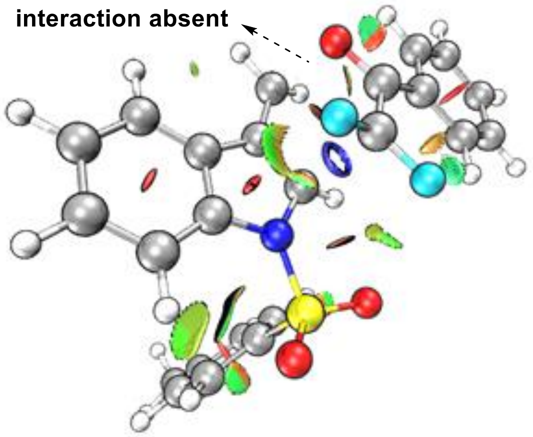

Supplementary Fig. 10. Color filled RDG isosurface for TS3' (isovalue set to 0.4): (blue) areas of attraction (covalent bonding); (green) vdW interaction; (red) areas of repulsion (steric and ring effects).

(Details of the cartesian coordinates and energies for the computed structures are provided in Supplementary Data file 1.)

VII. Characterization data for the products

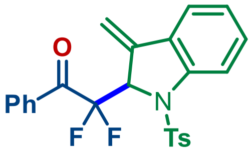

(3) According to the general procedure A, using *N*-triftosylhydrazone derived from 2,2,2-trifluoro-1-phenylethan-1-one (118.8 mg, 0.3 mmol), (1-tosyl-1*H*-indol-3-yl)methanol (180.8 mg, 0.6 mmol), K_2CO_3 (82.9 mg, 0.6 mmol), $Tp^{Br3}Ag$ (33.0 mg, 10 mol%) and toluene (4.0 mL) afforded compound **3** (110.6 mg, 84% yield) as a white solid; mp: 161-162 °C; **^{1}H NMR** (500 MHz, $CDCl_3$) δ 8.07 (d, *J* = 8.0 Hz, 2H), 7.61 (t, *J* = 8.0 Hz, 2H), 7.47 (t, *J* = 8.0 Hz, 2H), 7.43 (d, *J* = 8.5 Hz, 2H), 7.27-7.24 (m, 2H), 7.12-7.06 (m, 3H), 5.50 (s, 1H), 5.43 (dd, *J* = 12.5, 10.5 Hz, 1H), 5.17 (s, 1H), 2.30 (s, 3H); **^{13}C NMR** (125 MHz, $CDCl_3$) δ 189.1 (t, *J* = 27.5 Hz), 144.5, 143.9, 137.7, 134.3, 133.4, 133.0, 131.2, 130.2 (t, *J* = 3.8 Hz), 130.0, 129.6, 128.5, 127.4, 125.7, 120.8, 118.4, 116.1 (t, *J* = 258.8 Hz), 109.5, 66.8 (dd, *J* = 26.3, 25.0 Hz), 21.5; **^{19}F NMR** (470 MHz, $CDCl_3$) δ -106.32 (dd, *J* = 270.3, 9.9 Hz), -110.62 (dd, *J* = 270.3, 12.7 Hz); **IR** (Film): 3052, 2958, 1732, 1598, 1466, 1373, 1242, 1170, 1045, 939, 847, 734 cm^{-1}; **HRMS** (ESI^+) m/z calcd for $C_{24}H_{19}F_2NNaO_3S$ $[M+Na]^+$ 462.0946, found 462.0950.

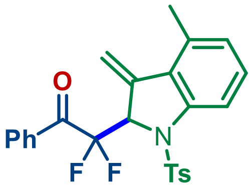

(4) According to the general procedure A, using *N*-triftosylhydrazone derived from 2,2,2-trifluoro-1-phenylethan-1-one (118.8 mg, 0.3 mmol), (4-methyl-1-tosyl-1*H*-indol-3-yl)methanol (189.2 mg, 0.6 mmol), K_2CO_3 (82.9 mg, 0.6 mmol), $Tp^{Br3}Ag$ (33.0 mg, 10 mol%) and toluene (4.0 mL) afforded compound **4** (118.2 mg, 87% yield) as a white solid; mp: 149-150 °C; **^{1}H NMR** (500 MHz, $CDCl_3$) δ 8.05 (d, *J* = 8.0 Hz, 2H), 7.60 (t, *J* = 7.0 Hz, 1H), 7.48-7.42 (m, 5H), 7.16-7.08 (m, 3H), 6.86 (d, *J* = 7.5 Hz, 1H), 5.47 (s, 1H), 5.38 (t, *J* = 11.0 Hz, 1H), 5.29 (s, 1H), 2.31 (s, 3H), 2.29 (s, 3H); **^{13}C NMR** (150 MHz, $CDCl_3$) δ 189.1 (t, *J* = 27.0 Hz), 144.5, 144.4, 138.6, 135.0, 134.2, 133.7, 133.2, 130.2 (t, *J* = 3.0 Hz), 129.5, 129.4, 128.6, 128.5, 128.1, 127.4, 116.2 (t, *J* = 259.5 Hz), 116.1, 113.4, 67.4 (t, *J* = 25.5 Hz), 21.5, 20.4; **^{19}F NMR** (564 MHz, $CDCl_3$) δ -107.42 (dd, *J* = 265.6, 10.7 Hz), -110.21 (dd, J = 265.6, 12.4 Hz); **IR** (Film): 3063, 2924, 2359, 1697, 1607, 1448, 1362, 1287, 1169, 1073, 942, 817, 734 cm^{-1}; **HRMS** (ESI^+) m/z calcd for $C_{25}H_{21}F_2NNaO_3S$ $[M+Na]^+$ 476.1102, found 476.1103.

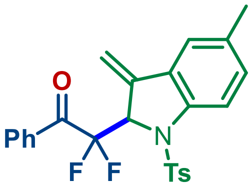

(5) According to the general procedure A, using *N*-triftosylhydrazone derived from 2,2,2-trifluoro-1-phenylethan-1-one (118.8 mg, 0.3 mmol), (5-methyl-1-tosyl-1*H*-indol-3-yl)methanol (189.2 mg, 0.6 mmol), K_2CO_3 (82.9 mg, 0.6 mmol), $Tp^{Br3}Ag$ (33.0 mg, 10 mol%) and toluene (4.0 mL) afforded compound **5** (126.4 mg, 93% yield) as a white solid; mp: 137-138 °C; **^{1}H NMR** (500 MHz, $CDCl_3$) δ 8.07 (d, *J* = 7.5 Hz, 2H), 7.60 (t, *J* = 7.5 Hz, 1H), 7.50 (d, *J* = 8.0 Hz, 1H), 7.46 (t, *J* = 8.0 Hz, 2H), 7.42 (d, *J* = 8.0 Hz, 2H), 7.14-7.04 (m, 4H), 5.46 (s, 1H), 5.38 (dd, *J* = 12.6, 10.8 Hz, 1H), 5.13 (s, 1H), 2.30 (s, 3H), 2.28 (s, 3H); **^{13}C NMR** (125 MHz, $CDCl_3$) δ 189.2 (t, *J* = 28.0 Hz), 144.4, 141.7, 137.8, 135.5, 134.2, 133.4, 133.0, 131.3, 130.9, 130.2 (t, *J* = 4.5 Hz), 129.6, 128.5, 127.4,

121.1, 118.2, 116.1 (t, J = 260.0 Hz), 109.1, 67.0 (dd, J = 27.0, 24.0 Hz), 21.5, 21.0; **^{19}F NMR** (470 MHz, $CDCl_3$) δ -106.64 (dd, J = 269.8, 10.8 Hz), -110.45 (dd, J = 269.8, 13.2 Hz); **IR** (Film): 3054, 2364, 1698, 1480, 1363, 1264, 1169, 1070, 972, 876, 732 cm^{-1}; **HRMS** (ESI$^+$) m/z calcd for $C_{25}H_{21}F_2NNaO_3S$ [M+Na]$^+$ 476.1102, found 476.1108.

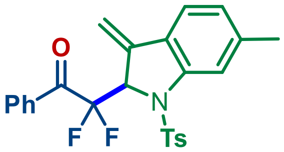

(6) According to the general procedure A, using *N*-triftosylhydrazone derived from 2,2,2-trifluoro-1-phenylethan-1-one (118.8 mg, 0.3 mmol), (6-methyl-1-tosyl-1*H*-indol-3-yl)methanol (189.2 mg, 0.6 mmol), K_2CO_3 (82.9 mg, 0.6 mmol)**,** Tp^{Br3}Ag (33.0 mg, 10 mol%) and toluene (4.0 mL) afforded compound **6** (119.6 mg, 88% yield) as a white solid; mp: 144-145 °C; **^{1}H NMR** (500 MHz, $CDCl_3$) δ 8.06 (d, J = 8.0 Hz, 2H), 7.61 (t, J = 7.0 Hz, 1H), 7.49-7.41 (m, 5H), 7.12 (t, J = 8.0 Hz, 3H), 6.89 (d, J = 8.0 Hz, 1H), 5.43-5.36 (m, 2H), 5.09 (s, 1H), 2.36 (s, 3H), 2.31 (s, 3H); **^{13}C NMR** (125 MHz, $CDCl_3$) δ 189.2 (t, J = 28.8 Hz), 144.5, 144.2, 140.7, 137.6, 134.3, 133.6, 133.2, 130.3 (t, J = 2.5 Hz), 129.7, 128.8, 128.6, 127.4, 126.7, 120.5, 119.0, 116.2 (t, J = 261.3 Hz), 108.4, 67.1 (dd, J = 26.3, 25.0 Hz), 21.8, 21.6; **^{19}F NMR** (470 MHz, $CDCl_3$) δ -106.46 (dd, J = 269.3, 9.9 Hz), -110.67 (dd, J = 269.3, 13.2 Hz); **IR** (Film): 3057, 2361,1697, 1598, 1424, 1366, 1264, 1172, 1091, 903, 830, 718 cm^{-1}; **HRMS** (ESI$^+$) m/z calcd for $C_{25}H_{21}F_2NNaO_3S$ [M+Na]$^+$ 476.1102, found 476.1101.

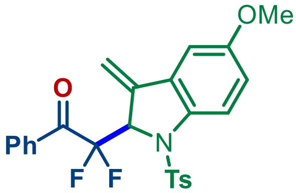

(7) According to the general procedure A, using *N*-triftosylhydrazone derived from 2,2,2-trifluoro-1-phenylethan-1-one (118.8 mg, 0.3 mmol), (5-methoxy-1-tosyl-1*H*-indol-3-yl)methanol (198.8 mg, 0.6 mmol), K_2CO_3 (82.9 mg, 0.6 mmol)**,** Tp^{Br3}Ag (33.0 mg, 10 mol%) and toluene (4.0 mL) afforded compound **7** (128.0 mg, 91% yield) as a white solid; mp: 118-119 °C; **^{1}H NMR** (500 MHz, $CDCl_3$) δ 8.07 (d, J = 8.0 Hz, 2H), 7.61 (t, J = 7.5 Hz, 1H), 7.52 (d, J = 8.5 Hz, 1H), 7.47 (t, J = 8.0 Hz, 2H), 7.40 (d, J = 8.0 Hz, 2H), 7.11 (d, J = 8.0 Hz, 2H), 6.83 (dd, J = 8.5, 3.0 Hz, 1H), 6.74 (d, J = 2.5 Hz, 1H), 5.46 (s, 1H), 5.34 (dd, J = 12.5, 11.0 Hz, 1H), 5.16 (s, 1H), 3.77 (s, 3H), 2.32 (s, 3H); **^{13}C NMR** (125 MHz, $CDCl_3$) δ 189.2 (t, J = 27.5 Hz), 158.0, 144.4, 137.9, 137.4, 134.3, 133.1, 132.7, 130.2 (t, J = 2.5 Hz), 129.6, 128.5, 127.5, 119.7, 116.6, 116.1 (t, J = 260.0 Hz), 109.7, 105.1, 67.3 (t, J = 25.0 Hz), 55.6, 21.5; **^{19}F NMR** (564 MHz, $CDCl_3$) δ -108.21 (dd, J = 267.9, 11.8 Hz), -109.38 (dd, J = 267.9, 11.3 Hz); **IR** (Film): 3066, 2938, 1698, 1481, 1361, 1284, 1168, 1069, 935, 826, 705 cm^{-1}; **HRMS** (ESI$^+$) m/z calcd for $C_{25}H_{21}F_2NNaO_4S$ [M+Na]$^+$ 492.1052, found 492.1043.

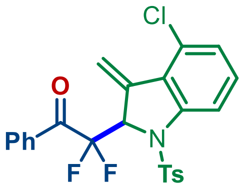

(8) According to the general procedure A, using *N*-triftosylhydrazone derived from 2,2,2-trifluoro-1-phenylethan-1-one (118.8 mg, 0.3 mmol), (4-chloro-1-tosyl-1*H*-indol-3-yl)methanol (201.5 mg, 0.6 mmol), K_2CO_3 (82.9 mg, 0.6 mmol)**,** Tp^{Br3}Ag (33.0 mg, 10 mol%) and toluene (4.0 mL) afforded compound **8** (113.5 mg, 80% yield) as a white solid; mp: 140-141 °C; **^{1}H NMR** (500 MHz, $CDCl_3$) δ 8.05 (d, J = 8.0 Hz, 2H), 7.61 (t, J = 7.5 Hz, 1H),

7.55 (d, J = 8.0 Hz, 1H), 7.49-7.44 (m, 4H), 7.19-7.12 (m, 3H), 7.05 (d, J = 8.0 Hz, 1H), 6.21 (s, 1H), 5.45 (t, J = 11.5 Hz, 1H), 5.40 (s, 1H), 2.33 (s, 3H); **^{13}C NMR** (150 MHz, $CDCl_3$) δ 188.9 (t, J = 28.5 Hz), 145.8, 144.9, 136.0, 134.4, 133.4, 132.9, 130.16, 130.13, 130.3, 129.8, 128.6, 127.39, 127.35, 116.7, 115.95, 115.93 (t, J = 260.0 Hz), 67.2 (t, J = 25.5 Hz), 21.5; **^{19}F NMR** (564 MHz, $CDCl_3$) δ -106.42 (dd, J = 271.3, 10.2 Hz), -110.83 (dd, J = 271.3, 13.0 Hz); **IR** (Film): 3055, 1689, 1448, 1366, 1264, 1171, 1089, 939, 847, 731 cm^{-1}; **HRMS** (ESI$^+$) m/z calcd for $C_{24}H_{19}ClF_2NO_3S$ [M+H]$^+$ 474.0738, found 474.0740.

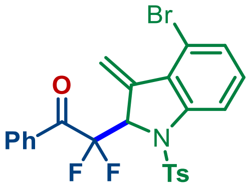

(9) According to the general procedure A, using N-triftosylhydrazone derived from 2,2,2-trifluoro-1-phenylethan-1-one (118.8 mg, 0.3 mmol), (4-bromo-1-tosyl-1H-indol-3-yl)methanol (228.2 mg, 0.6 mmol), , K_2CO_3 (82.9 mg, 0.6 mmol)**,** Tp^{Br3}Ag (33.0 mg, 10 mol%) and toluene (4.0 mL) afforded compound **9** (141.1 mg, 91% yield) as a white solid; mp: 148-149 °C; **^{1}H NMR** (500 MHz, $CDCl_3$) δ 8.05 (d, J = 8.0 Hz, 2H), 7.63-7.59 (m, 2H), 7.50-7.44 (m, 4H), 7.26 (d, J = 8.0 Hz, 1H), 7.15 (d, J = 8.0 Hz, 2H), 7.08 (t, J = 8.0 Hz, 1H), 6.35 (s, 1H), 5.44 (dd, J = 11.5, 10.0 Hz, 1H), 5.32 (s, 1H), 2.34 (s, 3H); **^{13}C NMR** (150 MHz, $CDCl_3$) δ 188.9 (t, J = 28.5 Hz), 146.0, 144.9, 136.6, 134.4, 133.4, 133.0, 130.8, 130.3, 130.2 (t, J = 3.0 Hz), 129.8, 128.8, 128.6, 127.4, 117.9, 117.3, 116.0 (t, J = 261.0 Hz), 115.8, 67.3 (t, J = 25.5 Hz), 21.6; **^{19}F NMR** (564 MHz, $CDCl_3$) δ -106.53 (dd, J = 271.3, 10.2 Hz), -110.74 (dd, J = 271.3, 13.0 Hz); **IR** (Film): 3063, 2365, 1697, 1598, 1483, 1366, 1260, 1172, 1093, 969, 815, 733 cm^{-1}; **HRMS** (ESI$^+$) m/z calcd for $C_{24}H_{18}BrF_2NNaO_3S$ [M+Na]$^+$ 540.0051, found 540.0055.

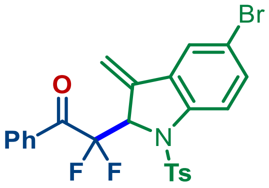

(10) According to the general procedure A, using N-triftosylhydrazone derived from 2,2,2-trifluoro-1-phenylethan-1-one (118.8 mg, 0.3 mmol), (5-bromo-1-tosyl-1H-indol-3-yl)methanol (228.2 mg, 0.6 mmol), K_2CO_3 (82.9 mg, 0.6 mmol)**,** Tp^{Br3}Ag (33.0 mg, 10 mol%) and toluene (4.0 mL) afforded compound **10** (133.4 mg, 86% yield) as a white solid; mp: 120-121 °C; **^{1}H NMR** (500 MHz, $CDCl_3$) δ 8.06 (d, J = 7.5 Hz, 2H), 7.62 (t, J = 7.5 Hz, 1H), 7.53-7.43 (m, 5H), 7.39-7.35 (m, 2H), 7.15 (d, J = 8.5 Hz, 2H), 5.50 (s, 1H), 5.43 (dd, J = 11.5, 10.0 Hz, 1H), 5.23 (s, 1H), 2.33 (s, 3H); **^{13}C NMR** (150 MHz, $CDCl_3$) δ 188.8 (t, J = 28.5 Hz), 144.9, 143.0, 136.6, 134.5, 133.22, 133.15, 132.81, 132.78, 130.2 (t, J = 3.0 Hz), 129.8, 128.6, 127.4, 123.9, 119.8, 118.9, 115.8 (t, J = 261.0 Hz), 111.1, 66.9 (t, J = 24.0 Hz), 21.5; **^{19}F NMR** (564 MHz, $CDCl_3$) δ -105.85 (dd, J = 273.5, 9.6 Hz), -110.58 (dd, J = 273.5, 13.5 Hz); **IR** (Film): 3061, 2923, 1695, 1596, 1461, 1355, 1261, 1162, 1069, 921, 890, 820, 707 cm^{-1}; **HRMS** (ESI$^+$) m/z calcd for $C_{24}H_{18}BrF_2NNaO_3S$ [M+Na]$^+$ 540.0051, found 540.0052.

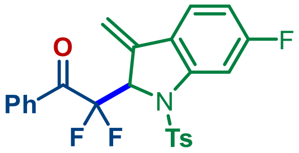

(11) According to the general procedure A, using N-triftosylhydrazone derived from 2,2,2-trifluoro-1-phenylethan-1-one (118.8 mg, 0.3 mmol), (6-fluoro-1-tosyl-1H-indol-3-yl)methanol (191.6 mg, 0.6 mmol), , K_2CO_3 (82.9 mg, 0.6 mmol)**,** Tp^{Br3}Ag (33.0 mg, 10 mol%) and toluene (4.0 mL) afforded compound **11** (116.5

mg, 85% yield) as a white solid; mp: 133-134 °C; **^{1}H NMR** (500 MHz, $CDCl_3$) δ 8.06 (d, *J* = 7.5 Hz, 2H), 7.63 (t, *J* = 7.5 Hz, 1H), 7.50-7.46 (m, 4H), 7.34 (dd, *J* = 9.5, 2.5 Hz, 1H), 7.20 (dd, *J* = 8.5, 5.5 Hz, 1H), 7.16 (d, *J* = 8.0 Hz, 2H), 6.78 (td, *J* = 8.5, 2.0 Hz, 1H), 5.50-5.42 (m, 2H), 5.14 (s, 1H), 2.34 (s, 3H); **^{13}C NMR** (150 MHz, $CDCl_3$) δ 188.8 (t, *J* = 27.0 Hz), 163.7 (d, *J* = 248.0 Hz), 162.9, 145.3 (d, *J* = 13.5 Hz), 144.9, 136.6, 134.4, 133.4, 132,9, 130.2, 129.8, 128.6, 127.4, 127.2 (d, *J* = 1.5 Hz), 121.9 (d, *J* = 10.5 Hz), 115.9 (t, *J* = 259.5 Hz), 112.9 (d, *J* = 24.0 Hz), 109.1, 106.2 (d, *J* = 27.0 Hz), 67.3 (t, *J* = 25.5 Hz), 21.6; **^{19}F NMR** (564 MHz, $CDCl_3$) δ -105.78 (dd, *J* = 273.0, 7.9 Hz), -108.97 (q, *J* = 8.5 Hz), (-110.81)-(-109.00) (m); **IR** (Film): 3055, 2982, 2306, 1700, 1591, 1493, 1356, 1264, 1175, 1069, 923, 830, 732 cm^{-1}; **HRMS** (ESI^+) m/z calcd for $C_{24}H_{18}F_3NNaO_3S$ $[M+Na]^+$ 480.0852, found 480.0857.

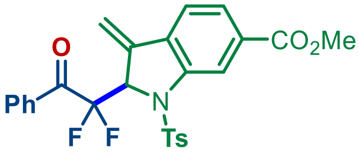

(12) According to the general procedure A, using *N*-triftosylhydrazone derived from 2,2,2-trifluoro-1-phenylethan-1-one (118.8 mg, 0.3 mmol), methyl 3-(hydroxymethyl)-1-tosyl-1*H*-indole-6-carboxylate (215.6 mg, 0.6 mmol), K_2CO_3 (82.9 mg, 0.6 mmol), $Tp^{Br3}Ag$ (33.0 mg, 10 mol%) and toluene (4.0 mL) afforded compound **12** (122.3 mg, 82% yield) as a white solid; mp: 145-146 °C; **^{1}H NMR** (500 MHz, $CDCl_3$) δ 8.26 (d, *J* = 1.0 Hz, 1H), 8.06 (d, *J* = 7.5 Hz, 2H), 7.80 (dd, *J* = 8.0, 1.5 Hz, 1H), 7.63 (t, *J* = 7.5 Hz, 1H), 7.51-7.43 (m, 4H), 7.32 (d, *J* = 8.0 Hz, 1H), 7.13 (d, *J* = 8.5 Hz, 2H), 5.64 (s, 1H), 5.50 (dd, *J* = 12.0, 10.5 Hz, 1H), 5.33 (s, 1H), 3.94 (s, 3H), 2.32 (s, 3H); **^{13}C NMR** (150 MHz, $CDCl_3$) δ 188.9 (t, *J* = 28.5 Hz), 166.1, 144.8, 144.1, 137.0, 135.4, 134.5, 133.3, 132.8, 131.8, 130.2 (t, *J* = 3.0 Hz), 129.7, 128.6, 127.4, 127.3, 120.6, 119.3, 115.9 (t, *J* = 261.0 Hz), 112.4, 66.9 (t, *J* = 25.5 Hz), 52.4, 21.5; **^{19}F NMR** (564 MHz, $CDCl_3$) δ -106.21 (dd, *J* = 274.1, 9.6 Hz), -110.33 (dd, *J* = 274.1, 13.0 Hz); **IR** (Film): 3060, 2954, 1720, 1437, 1367, 1264, 1110, 967, 815, 732 cm^{-1}; **HRMS** (ESI^+) m/z calcd for $C_{26}H_{21}F_2NNaO_5S$ $[M+Na]^+$ 520.1001, found 520.1006.

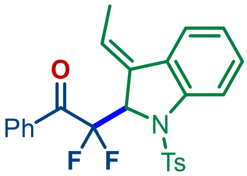

(13) According to the general procedure A, using *N*-triftosylhydrazone derived from 2,2,2-trifluoro-1-phenylethan-1-one (118.8 mg, 0.3 mmol), methyl 3-(1-hydroxyethyl)-1-tosyl-1*H*-indole-6-carboxylate (224.1 mg, 0.6 mmol), K_2CO_3 (82.9 mg, 0.6 mmol), $Tp^{Br3}Ag$ (33.0 mg, 10 mol%) and toluene (4.0 mL) afforded compound **13** (81.5 mg, 60% yield) as a white solid. mp: 123-124 ℃; **^{1}NMR** (500 MHz, $CDCl_3$) δ 7.95 (d, *J* = 7.5 Hz, 2H), 7.52 (t, *J* = 7.5 Hz, 1H), 7.49 (d, *J* = 8.0 Hz, 1H), 7.40-7.33 (m, 4H), 7.28 (t, *J* = 8.0 Hz, 1H), 7.13 (td, *J* = 8.0, 1.0 Hz, 1H), 7.05-6.98 (m, 3H), 5.65 (q, *J* = 7.5 Hz, 1H), 5.21 (t, *J* = 16.5 Hz, 1H), 2.24 (s, 3H), 1.75 (dd, *J* = 7.5, 1.0 Hz, 3H); **^{13}C NMR** (125 MHz, $CDCl_3$) δ 189.5 (t, *J* = 28.8 Hz), 144.4, 134.2, 133.8, 133.3, 131.6, 130.3 (t, *J* = 2.5 Hz), 129.5, 128.9, 128.5, 128.4, 127.5, 127.3, 125.7, 125.2, 124.5, 118.6, 116.4 (t, *J* = 258.8 Hz), 68.0 (t, *J* = 26.3 Hz), 21.5, 14.3; **^{19}F NMR** (564 MHz, $CDCl_3$) δ -108.00 (dd, *J* = 264.0, 10.7 Hz), -110.07 (dd, *J* = 264.0, 11.8 Hz); **IR** (Film): 3054, 2986, 2361, 1732, 1698, 1598, 1421, 1363, 1264, 1171, 895, 731 cm^{-1}; **HRMS** (ESI^+) m/z calcd for $C_{25}H_{21}F_2NNaO_3S$ $[M+Na]^+$ 476.1102, found 476.1110.

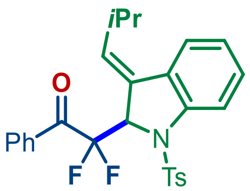

(14) According to the general procedure A, using *N*-triftosylhydrazone derived from 2,2,2-trifluoro-1-phenylethan-1-one (118.8 mg, 0.3 mmol), methyl 3-(1-hydroxy-3-methylbutyl)-1-tosyl-1*H*-indole-6-carboxylate (249.3 mg, 0.6 mmol), K_2CO_3 (83.2 mg, 0.6 mmol)**,** $Tp^{Br3}Ag$ (33.0 mg, 10 mol%) and toluene (4.0 mL) afforded compound 1**4** (79.4 mg, 55% yield) as a white solid. mp: 135-136 ℃; **^{1}H NMR** (500 MHz, $CDCl_3$) δ 8.00 (d, *J* = 8.0 Hz, 2H), 7.59 (t, *J* = 7.5 Hz, 1H), 7.56 (d, *J* = 8.0 Hz, 1H), 7.45 (t, *J* = 7.5 Hz, 2H), 7.39 (d, *J* = 8.5 Hz, 2H), 7.30 (d, *J* = 7.5 Hz, 1H), 7.21 (t, *J* = 8.0 Hz, 1H), 7.09 (t, *J* = 8.5 Hz, 3H), 5.37 (d, *J* = 9.5 Hz, 1H), 5.18 (t, *J* = 11.5 Hz, 1H), 2.85-2.70 (m, 1H), 2.31 (s, 3H), 0.96 (d, *J* = 6.5 Hz, 3H), 0.74 (d, *J* = 6.5 Hz, 3H); **^{13}C NMR** (125 MHz, $CDCl_3$) δ 189.6 (d, *J* = 28.8 Hz), 144.6, 144.4, 138.8, 134.2, 133.6, 133.5, 131.3, 130.2 (t, *J* = 2.5 Hz), 129.4, 129.0, 128.5, 127.5, 126.7, 126.0, 124.2, 119.3, 116.4 (t, *J* = 260.0 Hz), 68.3 (t, *J* = 25.0 Hz), 27.3, 22.1, 22.0, 21.5; **^{19}F NMR** (470 MHz, $CDCl_3$) δ -107.66 (dd, *J* = 262.7, 11.3 Hz), -110.52 (dd, *J* = 262.7, 12.2 Hz); **IR** (Film): 3059, 2965, 1732, 1698, 1598, 1456, 1363, 1265, 1171, 1045, 910, 814, 732 cm^{-1}; **HRMS** (ESI^+) m/z calcd for $C_{27}H_{25}F_2NNaO_3S$ $[M+Na]^+$ 504.1415, found 504.1421.

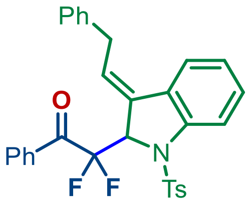

(15) According to the general procedure A, using *N*-triftosylhydrazone derived from 2,2,2-trifluoro-1-phenylethan-1-one (118.8 mg, 0.3 mmol), methyl 3-(1-hydroxy-2-phenylethyl)-1-tosyl-1*H*-indole-6-carboxylate (269.7 mg, 0.6 mmol), K_2CO_3 (82.9 mg, 0.6 mmol)**,** $Tp^{Br3}Ag$ (33.0 mg, 10 mol%) and toluene (4.0 mL) afforded compound **15** (104.7 mg, 66% yield) as a White solid; mp: 155-156 ℃; **^{1}H NMR** (500 MHz, $CDCl_3$) δ 8.02 (d, *J* = 8.0 Hz, 2H), 7.60 (t, *J* = 8.0 Hz, 2H), 7.49-7.41 (m, 4H), 7.38 (d, *J* = 8.0 Hz, 1H), 7.30-7.17 (m, 6H), 7.13 (d, *J* = 8.0 Hz, 2H), 7.08 (t, *J* = 7.5 Hz, 1H), 6.94 (d, *J* = 6.8 Hz, 2H), 5.79 (t, *J* = 7.5 Hz, 1H), 5.31 (t, *J* = 11.5 Hz, 1H), 3.70-3.48 (m, 2H), 2.35 (s, 3H); **^{13}C NMR** (125 MHz, $CDCl_3$) δ 189.4 (t, *J* = 27.5 Hz), 144.8, 144.4, 138.9, 134.2, 133.8, 133.3, 131.2, 130.3 (t, *J* = 2.5 Hz), 130.0, 129.6, 129.4, 129.0, 128.6, 128.5, 128.4, 128.1, 127.6, 126.5, 125.9, 124.3, 119.1, 116.3 (t, *J* = 258.8 Hz), 68.2 (t, *J* = 26.3 Hz), 34.4, 21.6; **^{19}F NMR** (470 MHz, $CDCl_3$) δ -107.96 (dd, *J* = 264.1, 12.2 Hz), -109.73 (dd, *J* = 264.1, 11.3 Hz); **IR** (Film): 3054, 2983, 1702, 1422, 1264, 1172, 895, 731 cm^{-1}; **HRMS** (ESI^+) m/z calcd for $C_{31}H_{25}F_2NNaO_3S$ $[M+Na]^+$ 552.1415, found 552.1417.

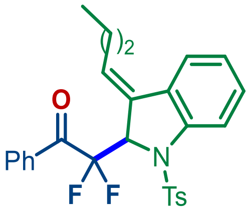

(16) According to the general procedure A, using *N*-triftosylhydrazone derived from 2,2,2-trifluoro-1-phenylethan-1-one (118.8 mg, 0.3 mmol), methyl 3-(1-hydroxybutyl)-1-tosyl-1*H*-indole-6-carboxylate (240.9 mg, 0.6 mmol), K_2CO_3 (82.9 mg, 0.6 mmol)**,** $Tp^{Br3}Ag$ (33.0 mg, 10 mol%) and toluene (4.0 mL) afforded compound **16** (93.8 mg, 65% yield) as a white solid. mp: 131-132 ℃; **^{1}H NMR** (600 MHz, $CDCl_3$) δ 8.02 (d, *J* = 7.8 Hz,

2H), 7.60 (t, J = 7.2 Hz, 1H), 7.57 (d, J = 7.8 Hz, 1H), 7.45 (t, J = 8.4 Hz, 2H), 7.41 (d, J = 8.4 Hz, 2H), 7.33 (d, J = 7.8 Hz, 1H), 7.23-7.19 (m, 1H), 7.12-7.07 (m, 3H), 5.58 (t, J = 7.2 Hz, 1H), 5.24 (t, J = 11.4 Hz, 1H), 2.31 (s, 3H), 2.23-2.17 (m, 2H), 1.40-1.33 (m, 1H), 1.32-1.27 (m, 1H), 0.77 (t, J = 7.2 Hz, 3H); **^{13}C NMR** (150 MHz, $CDCl_3$) δ 189.5 (t, J = 28.5 Hz), 144.5, 144.4, 134.2, 133.7, 133.4, 131.6, 131.5, 130.2 (d, J = 3.0 Hz), 129.5, 128.9, 128.8, 128.5, 127.5, 125.8, 124.4, 119.0, 116.1 (t, J = 294.0 Hz), 68.1 (t, J = 24.0 Hz), 30.2, 22.2, 21.5, 13.5; **^{19}F NMR** (564 MHz, $CDCl_3$) δ -108.06 (dd, J = 264.0, 11.3 Hz), -110.10 (dd, J = 264.0, 11.3 Hz); **IR** (Film): 3054, 2986, 1689, 1598, 1421, 1363, 1264, 1171, 1089, 895, 731 cm^{-1}; **HRMS** (ESI$^+$) m/z calcd for $C_{27}H_{25}F_2NNaO_3S$ [M+Na]$^+$ 504.1415, found 504.1416.

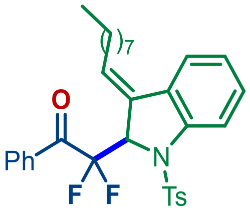

(17) According to the general procedure A, using *N*-triftosylhydrazone derived from 2,2,2-trifluoro-1-phenylethan-1-one (118.8 mg, 0.3 mmol), methyl 3-(1-hydroxynonyl)-1-tosyl-1*H*-indole-6-carboxylate (282.9 mg, 0.6 mmol), K_2CO_3 (82.9 mg, 0.6 mmol), Tp^{Br3}Ag (33.0 mg, 10 mol%) and toluene (4.0 mL) afforded compound **17** (135.5 mg, 82% yield) as a white solid. mp: 137-138 ℃; **^{1}H NMR** (600 MHz, $CDCl_3$) δ 8.02 (d, J = 7.8 Hz, 2H), 7.61-7.55 (m, 2H), 7.44 (t, J = 7.8 Hz, 2H), 7.41 (d, J = 8.4 Hz, 2H), 7.32 (d, J = 7.8 Hz, 1H), 7.20 (t, J = 7.8 Hz, 1H), 7.11-7.05 (m, 3H), 5.59 (t, J = 7.8 Hz, 1H), 5.26 (t, J = 11.4 Hz, 1H), 2.30 (s, 3H), 2.24-2.17 (m, 2H), 1.36-1.11 (m, 14H), 0.87 (t, J = 6.6 Hz, 3H); **^{13}C NMR** (150 MHz, $CDCl_3$) δ 189.5 (t, J = 27.0 Hz), 144.5, 144.3, 134.2, 133.8, 133.4, 131.8, 131.6, 130.2, 129.5, 128.9, 128.6, 128.5, 127.5, 125.8, 124.4, 118.9, 116.4 (t, J = 25.5 Hz), 68.2 (t, J = 258.0 Hz), 31.7, 29.1, 29.0, 28.3, 22.6, 21.5, 14.1; **^{19}F NMR** (564 MHz, $CDCl_3$) δ -107.80 (dd, J = 262.3, 10.7 Hz), -110.30 (dd, J = 262.3, 13.0 Hz); **IR** (Film): 3054, 2928, 1703, 1458, 1363, 1264, 1171, 1090, 940, 814, 731 cm^{-1}; **HRMS** (ESI$^+$) m/z calcd for $C_{32}H_{35}F_2NNaO_3S$ [M+Na]$^+$ 574.2198, found 574.2199.

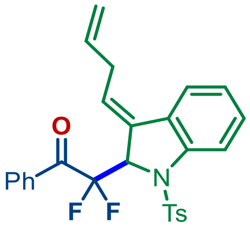

(18) According to the general procedure A, using *N*-triftosylhydrazone derived from 2,2,2-trifluoro-1-phenylethan-1-one (118.8 mg, 0.3 mmol), 1-(5-methyl-1-tosyl-1*H*-indol-3-yl)but-3-en-1-ol (213.3 mg, 0.6 mmol), K_2CO_3 (82.9 mg, 0.6 mmol), Tp^{Br3}Ag (33.0 mg, 10 mol%) and toluene (4.0 mL) afforded compound **18** (100.6 mg, 70% yield) as a white solid; mp: 151-152 °C; **^{1}H NMR** (600 MHz, $CDCl_3$) δ 8.03 (d, J = 7.2 Hz, 2H), 7.60 (t, J = 7.2 Hz, 1H), 7.57 (d, J = 7.2 Hz, 1H), 7.48-7.43 (m, 2H), 7.41 (d, J = 6.0 Hz, 2H), 7.30 (d, J = 7.8 Hz, 1H), 7.22 (t, J = 7.8 Hz, 1H), 7.13-7.03 (m, 3H), 5.72-5.62 (m, 2H), 5.28 (t, J = 12.0 Hz, 1H), 4.96 (dd, J = 10.2, 1.2 Hz, 1H), 4.87 (dd, J = 16.8, 1.8 Hz, 1H), 3.03-2.92 (m, 2H), 2.32 (s, 3H); **^{13}C NMR** (150 MHz, $CDCl_3$) δ 189.4 (t, J = 27.0 Hz), 144.6, 144.4, 134.2, 134.2, 133.7, 133.3, 131.2, 130.3 (t, J = 3.0 Hz), 130.1, 129.5, 129.3, 128.5, 127.6, 127.5, 127.3, 125.9, 124.4, 119.0, 116.3 (t, J = 259.5 Hz), 116.0, 68.1 (t, J = 25.5 Hz), 32.0, 21.5; **^{19}F NMR** (564 MHz, $CDCl_3$) δ -108.07 (dd, J = 264.5, 11.3 Hz), -109.77 (dd, J = 264.5, 12.4 Hz); **IR** (Film): 3054, 1698, 1598, 1457, 1363, 1264, 1171, 1090, 923, 895, 732 cm^{-1}; **HRMS** (ESI$^+$) m/z calcd for $C_{27}H_{23}F_2NNaO_3S$ [M+Na]$^+$

502.1259, found 502.1261.

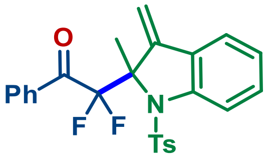

(19) According to the general procedure B, using *N*-triftosylhydrazone derived from 2,2,2-trifluoro-1-phenylethan-1-one (118.8 mg, 0.3 mmol), (2-methyl-1-tosyl-1*H*-indol-3-yl)methanol (189.2 mg, 0.6 mmol), K_2CO_3 (82.9 mg, 0.6 mmol)**,** $Tp^{Br3}Ag$ (33.0 mg, 10 mol%) and toluene (4.0 mL) afforded compound **19** (88.3 mg, 65% yield) as a white solid; mp: 162-163 °C; **^{1}H NMR** (600 MHz, $CDCl_3$) δ 7.98 (d, *J* = 7.8 Hz, 2H), 7.65 (d, *J* = 8.4 Hz, 1H), 7.55 (d, *J* = 8.4 Hz, 3H), 7.40 (t, *J* = 7.8 Hz, 2H), 7.36 (d, *J* = 7.8 Hz, 1H), 7.20 (t, *J* = 7.2 Hz, 1H), 7.12 (d, *J* = 8.4 Hz, 1H), 7.06 (t, *J* = 7.2 Hz, 1H), 5.67 (s, 1H), 5.25 (s, 1H), 2.30 (s, 3H), 1.95 (s, 3H); **^{13}C NMR** (150 MHz, $CDCl_3$) δ 189.1 (t, *J* = 27.0 Hz), 144.1, 144.1, 143.5, 138.1, 134.2, 133.8, 130.5 (t, *J* = 4.5 Hz), 130.4, 129.7, 129.2, 128.2, 126.8, 124.7, 120.7, 116.8 (t, *J* = 262.5 Hz), 116.4, 107.7, 74.5 (t, *J* = 27.0 Hz), 21.5, 18.2; **^{19}F NMR** (470 MHz, $CDCl_3$) δ -108.32 (d, *J* = 253.8 Hz), -110.02 (d, *J* = 253.8 Hz); **IR** (Film): 3054, 1695, 1598, 1461, 1361, 1264, 1171, 1046, 991, 862, 732 cm^{-1}; **HRMS** (ESI^+) m/z calcd for $C_{25}H_{21}F_2NNaO_3S$ $[M+Na]^+$ 476.1102, found 476.1105.

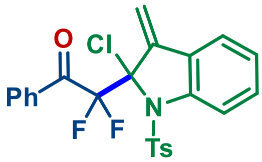

(20) According to the general procedure B, using *N*-triftosylhydrazone derived from 2,2,2-trifluoro-1-phenylethan-1-one (118.8 mg, 0.3 mmol), (2-chloro-1-tosyl-1*H*-indol-3-yl)methanol (201.5 mg, 0.6 mmol), K_2CO_3 (82.9 mg, 0.6 mmol)**,** $Tp^{Br3}Ag$ (33.0 mg, 10 mol%) and toluene (4.0 mL) afforded compound **20** (73.8 mg, 52% yield) as a white solid; mp: 138-139 °C; **^{1}H NMR** (600 MHz, $CDCl_3$) δ 8.18 (d, *J* = 7.8 Hz, 2H), 7.94 (d, *J* = 8.4 Hz, 1H), 7.71 (t, *J* = 7.8 Hz, 3H), 7.64 (t, *J* = 7.2 Hz, 1H), 7.52 (t, *J* = 7.8 Hz, 2H), 7.39 (t, *J* = 7.8 Hz, 1H), 7.33 (t, *J* = 7.8 Hz, 1H), 7.19 (d, *J* = 8.4 Hz, 2H), 4.94 (s, 2H), 2.31 (s, 3H); **^{13}C NMR** (150 MHz, $CDCl_3$) δ 187.5 (t, *J* = 30.7 Hz), 145.5, 136.4, 135.0, 134.0, 132.6, 130.0, 129.8, 128.7, 128.0, 127.4, 127.2, 124.2, 124.0, 120.1, 116.4, 114.8, 35.3 (t, *J* = 7.8 Hz), 21.6; **^{19}F NMR** (564 MHz, $CDCl_3$) δ -88.38; **IR** (Film): 3054, 2986, 1698, 1601, 1421, 1364, 1264, 1172, 895, 731 cm^{-1}; **HRMS** (ESI^+) m/z calcd for $C_{24}H_{18}ClF_2NNaO_3S$ $[M+Na]^+$ 496.0556, found 496.0558.

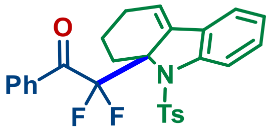

(21) According to the general procedure B, using *N*-triftosylhydrazone derived from 2,2,2-trifluoro-1-phenylethan-1-one (118.8 mg, 0.3 mmol), 9-tosyl-2,3,4,9-tetrahydro-1*H*-carbazol-4-ol (204.9 mg, 0.6 mmol), K_2CO_3 (82.9 mg, 0.6 mmol)**,** $Tp^{Br3}Ag$ (33.0 mg, 10 mol%) and toluene (4.0 mL) afforded compound **21** (66.1 mg, 46% yield) as a white solid; mp: 152-153 °C; **^{1}H NMR** (500 MHz, $CDCl_3$) δ 8.17 (d, *J* = 8.0 Hz, 1H), 8.04 (d, *J* = 8.0 Hz, 2H), 7.68-7.63 (m, 2H), 7.62-7.59 (m, 1H), 7.54 (dd, *J* = 8.0, 3.6 Hz, 1H), 7.45 (t, *J* = 8.0 Hz, 2H), 7.30-7.25 (m, 2H), 7.25-7.19 (m, 3H), 3.94 (dt, *J* = 25.0, 6.5 Hz, 1H), 3.23 (ddd, *J* = 18.0, 6.0, 2.5 Hz, 1H), 2.89 (dt, *J* = 18.0, 8.5 Hz, 1H), 2.35 (s, 3H), 2.06-1.93 (m, 1H), 1.87 (s, 1H), 1.77-1.70 (m, 1H); **^{19}F NMR** (564 MHz, $CDCl_3$) δ -90.89 (dd, *J* = 278.2, 6.6 Hz), -105.33 (dd, *J* = 278.2, 25.2 Hz); **IR** (Film): 3054, 1701, 1421, 1264, 1172, 895, 731 cm^{-1}; **HRMS** (ESI^+) m/z calcd for $C_{27}H_{23}F_2NNaO_3S$ $[M+Na]^+$ 502.1259, found 502.1258.

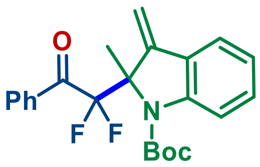

(22) According to the general procedure B, using *N*-triftosylhydrazone derived from 2,2,2-trifluoro-1-phenylethan-1-one (118.8 mg, 0.3 mmol), *tert*-butyl 3-(hydroxymethyl)-2-methyl-1*H*-indole-1-carboxylate (156.8 mg, 0.6 mmol), K_2CO_3 (82.9 mg, 0.6 mmol)**,** $Tp^{Br3}Ag$ (33.0 mg, 10 mol%) and toluene (4.0 mL) afforded compound **22** (86.2 mg, 72% yield) as a white solid; mp: 127-128 °C; **^{1}H NMR** ^{1}H NMR (500 MHz, $CDCl_3$) δ 7.88 (d, *J* = 8.0 Hz, 2H), 7.70 (d, *J* = 8.0 Hz, 1H), 7.53 (t, *J* = 7.5 Hz, 1H), 7.46 (d, *J* = 7.5 Hz, 1H), 7.38 (t, *J* = 8.0 Hz, 2H), 7.25 (t, *J* = 7.5 Hz, 1H), 7.04 (td, *J* = 7.5, 0.5 Hz, 1H), 5.79 (s, 1H), 5.42 (s, 1H), 1.99 (s, 3H), 1.24 (s, 9H); **^{13}C NMR** (150 MHz, $CDCl_3$) δ 188.9 (dd, *J* = 31.5, 25.5 Hz), 151.0, 134.3, 133.6, 130.0, 129.9 (d, *J* = 1.5 Hz), 129.9 (d, *J* = 1.5 Hz), 128.4, 123.3, 120.1, 117.6 (t, *J* = 261 Hz), 116.5, 107.0, 83.1, 71.6 (t, *J* = 28.5 Hz), 27.7, 21.0; **^{19}F NMR** (564 MHz, $CDCl_3$) δ -106.99 (d, *J* = 253.8 Hz), -109.79 (d, *J* = 253.8 Hz); **IR** (Film): 3055, 1706, 1575, 1468, 1316, 1264, 1167, 981, 896, 731 cm^{-1}; **HRMS** (ESI$^+$) m/z calcd for $C_{23}H_{23}F_2NNaO_3S$ $[M+Na]^+$ 422.1538, found 422.1544.

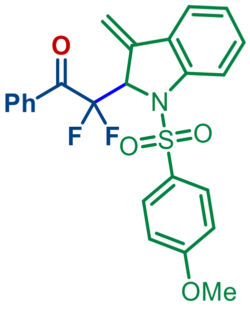

(23) According to the general procedure A, using *N*-triftosylhydrazone derived from 2,2,2-trifluoro-1-phenylethan-1-one (118.8 mg, 0.3 mmol), (1-((4-methoxyphenyl)sulfonyl)-1*H*-indol-3-yl)methanol (190.4 mg, 0.6 mmol), K_2CO_3 (82.9 mg, 0.6 mmol)**,** $Tp^{Br3}Ag$ (33.0 mg, 10 mol%) and toluene (4.0 mL) afforded compound **23** (121.5 mg, 89% yield) as a white solid; mp: 121-122 °C; **^{1}H NMR** (500 MHz, $CDCl_3$) δ 8.07 (d, *J* = 8.0 Hz, 2H), 7.63-7.6 (m, 2H), 7.50-7.45 (m, 4H), 7.30-7.24 (m, 2H), 7.09 (t, *J* = 7.5 Hz, 1H), 6.78 (d, *J* = 9.0 Hz, 2H), 5.52 (s, 1H), 5.40 (t, *J* = 10.5 Hz, 1H), 5.18 (s, 1H), 3.78 (s, 3H); **^{13}C NMR** (125 MHz, $CDCl_3$) δ 189.2 (t, *J* = 27.5 Hz), 163.5, 144.0, 137.8, 134.3, 133.0, 131.4, 130.3 (t, *J* = 3.8 Hz), 130.0, 129.6, 128.6, 127.9, 125.7, 120.8, 118.6, 116.1 (t, *J* = 260.0 Hz), 114.2, 109.5, 66.8 (t, *J* = 25.0 Hz), 55.5; **^{19}F NMR** (470 MHz, $CDCl_3$) δ -106.56 (dd, *J* = 270.3, 10.3 Hz), -110.50 (dd, *J* = 270.3, 13.2 Hz); **IR** (Film): 3055, 1698, 1596, 1497, 1363, 1263, 1181, 1091, 1024, 922, 831, 731 cm^{-1}; **HRMS** (ESI$^+$) m/z calcd for $C_{24}H_{19}F_2NNaO_4S$ $[M+Na]^+$ 478.0895, found 478.0897.

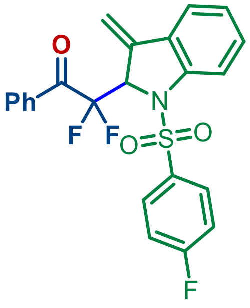

(24) According to the general procedure A, using *N*-triftosylhydrazone derived from 2,2,2-trifluoro-1-

phenylethan-1-one (118.8 mg, 0.3 mmol), (1-((4-fluorophenyl)sulfonyl)-1*H*-indol-3-yl)methanol (183.2 mg, 0.6 mmol), K_2CO_3 (82.9 mg, 0.6 mmol), $Tp^{Br3}Ag$ (33.0 mg, 10 mol%) and toluene (4.0 mL) afforded compound **24** (109.0 mg, 82% yield) as a white solid; mp: 144-145 °C; **^{1}H NMR** (500 MHz, $CDCl_3$) δ 8.06 (d, *J* = 8.0 Hz, 2H), 7.65-7.54 (m, 4H), 7.47 (t, *J* = 8.0 Hz, 2H), 7.30-7.26 (m, 2H), 7.11 (t, *J* = 7.5 Hz, 1H), 7.01 (t, *J* = 8.5 Hz, 2H), 5.53 (s, 1H), 5.47-5.38 (m, 1H), 5.20 (s, 1H); **^{13}C NMR** (125 MHz, $CDCl_3$) δ 189.0 (t, *J* = 27.5 Hz), 165.6 (d, *J* = 256.0 Hz), 143.6, 137.4, 134.4, 132.9, 132.4 (d, *J* = 3.8 Hz), 131.3, 130.2, 130.13, 130.11, 128.6, 126.0, 120.9, 118.5, 116.4, 116.2, 116.0 (t, *J* = 261.3 Hz) , 109.9, 66.8 (t, *J* = 23.8 Hz); **^{19}F NMR** (470 MHz, $CDCl_3$) δ (-101.12)-(-103.89) (m), -105.84 (dd, *J* = 272.6, 9.9 Hz), -110.86 (dd, *J* = 272.6, 13.6 Hz); **IR** (Film): 3055, 1698, 1598, 1491, 1367, 1264, 1172, 1090, 982, 898, 731 cm^{-1}; **HRMS** (ESI$^+$) m/z calcd for $C_{23}H_{16}F_3NNaO_3S$ $[M+Na]^+$ 466.0695, found 466.0690.

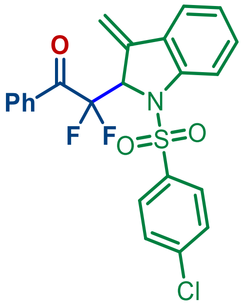

(25) According to the general procedure A, using *N*-triftosylhydrazone derived from 2,2,2-trifluoro-1-phenylethan-1-one (118.8 mg, 0.3 mmol), (1-((4-chlorophenyl)sulfonyl)-1*H*-indol-3-yl)methanol (193.1 mg, 0.6 mmol), K_2CO_3 (82.9 mg, 0.6 mmol), $Tp^{Br3}Ag$ (33.0 mg, 10 mol%) and toluene (4.0 mL) afforded compound **25** (110.2 mg, 80% yield) as a white solid; mp: 155-156 °C; **^{1}H NMR** (600 MHz, $CDCl_3$) δ 8.06 (d, *J* = 7.8 Hz, 2H), 7.64-7.60 (m, 2H), 7.53-7.45 (m, 4H), 7.33-7.24 (m, 4H), 7.14-7.09 (m, 1H), 5.54 (s, 1H), 5.47-5.39 (m, 1H), 5.21 (s, 1H); **^{13}C NMR** (150 MHz, $CDCl_3$) δ 188.9 (t, *J* = 28.5 Hz), 143.5, 140.3, 137.3, 134.9, 134.4, 132.9, 131.3, 130.19, 130.17, 129.3, 128.8, 128.6, 126.0, 121.0, 118.4, 115.9 (t, *J* = 261.0 Hz), 110.0, 66.8 (t, *J* = 24.0 Hz); **^{19}F NMR** (564 MHz, $CDCl_3$) δ -105.66 (dd, *J* = 273.0, 9.6 Hz), -110.94 (dd, *J* = 273.0, 14.1 Hz); **IR** (Film): 3054, 2986, 1697, 1421, 1373, 1264, 1175, 1085, 895, 731 cm^{-1}; **HRMS** (ESI$^+$) m/z calcd for $[M+Na]^+$ $C_{23}H_{16}ClF_2NNaO_3S$ 482.0400, found 482.0389.

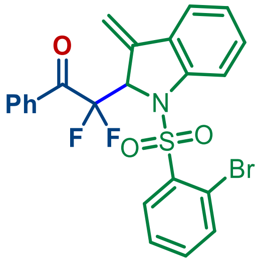

(26) According to the general procedure A, using *N*-triftosylhydrazone derived from 2,2,2-trifluoro-1-phenylethan-1-one (118.8 mg, 0.3 mmol), (1-((2-bromophenyl)sulfonyl)-1*H*-indol-3-yl)methanol (219.7 mg, 0.6 mmol), K_2CO_3 (82.9 mg, 0.6 mmol), $Tp^{Br3}Ag$ (33.0 mg, 10 mol%) and toluene (4.0 mL) afforded compound **26** (108.6 mg, 72% yield) as a white solid; mp: 141-142 °C; **^{1}H NMR** (600 MHz, $CDCl_3$) δ 8.14-8.06 (m, 3H), 7.62 (t, *J* = 7.2 Hz, 1H), 7.58 (dd, *J* = 7.8, 1.2 Hz, 1H), 7.47 (t, *J* = 7.8 Hz, 2H), 7.37 (td, *J* = 7.8, 1.2 Hz, 1H), 7.34-7.28 (m, 2H), 7.20 (d, *J* = 7.8 Hz, 1H), 7.12-7.06 (m, 1H), 6.99 (td, *J* = 7.8, 1.2 Hz, 1H), 6.20 (dd, *J* = 16.8, 6.6 Hz, 1H), 5.66 (s, 1H), 5.26 (s, 1H); **^{13}C NMR** (150 MHz, $CDCl_3$) δ 188.6 (t, *J* = 27.0 Hz), 142.9, 137.9, 137.1, 135.7, 134.5, 134.3, 133.8, 132.7, 130.3, 130.2, 129.8, 128.7, 127.4, 124.8, 121.1, 119.9, 116.2 (t, *J* = 259.5 Hz),

116.1, 109.7, 67.9 (t, J = 24.0 Hz); **^{19}F NMR** (564 MHz, $CDCl_3$) δ -102.29 (dd, J = 275.2, 6.2 Hz), -112.80 (dd, J = 275.2, 16.9 Hz); **IR** (Film): 3054, 2985, 1694, 1585, 1423, 1362, 1264, 1172, 1047, 933, 895, 731 cm^{-1}; **HRMS** (ESI$^+$) m/z calcd for $C_{23}H_{16}BrF_2NNaO_3S$ [M+Na]$^+$ 525.9895, found 525.9890.

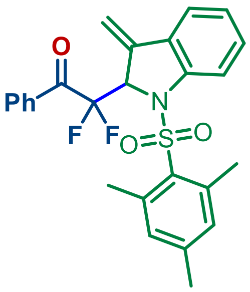

(27) According to the general procedure A, using *N*-triftosylhydrazone derived from 2,2,2-trifluoro-1-phenylethan-1-one (118.8 mg, 0.3 mmol), (1-(mesitylsulfonyl)-1*H*-indol-3-yl)methanol (197.6 mg, 0.6 mmol), K_2CO_3 (82.9 mg, 0.6 mmol)**,** Tp^{Br3}Ag (33.0 mg, 10 mol%) and toluene (4.0 mL) afforded compound **27** (126.1 mg, 90% yield) as a white solid; mp: 152-153 °C; **^{1}H NMR** (600 MHz, $CDCl_3$) δ 7.95 (d, J = 7.8 Hz, 2H), 7.59 (t, J = 7.2 Hz, 1H), 7.43 (t, J = 8.4 Hz, 2H), 7.36 (d, J = 7.2 Hz, 1H), 7.16-7.10 (m, 1H), 7.07-7.03 (m, 1H), 7.02 (d, J = 7.8 Hz, 1H), 6.92 (s, 2H), 5.66 (s, 1H), 5.62 (dd, J = 16.2, 6.0 Hz, 1H), 5.22 (s, 1H), 2.58 (s, 6H), 2.27 (s, 3H); **^{13}C NMR** (125 MHz, $CDCl_3$) δ 188.7 ((t, J = 27.5 Hz), 144.4, 143.7, 141.1, 138.6, 134.4, 132.7, 132.2, 131.3, 131.2, 130.0 (t, J = 2.5 Hz), 129.7, 128.6, 125.0, 120.9, 117.8, 116.3 (t, J = 260.0 Hz), 109.4, 66.0 (t, J = 23.8 Hz), 22.9, 21.0; **^{19}F NMR** (470 MHz, $CDCl_3$) δ -102.48 (dd, J = 274.0, 6.6 Hz), -112.36 (dd, J = 274.0, 16.5 Hz); **IR** (Film): 3058, 2924, 2861, 1701, 1464, 1355, 1267, 1151, 1069, 925, 830, 731 cm^{-1}; **HRMS** (ESI$^+$) m/z calcd for $C_{26}H_{23}F_2NNaO_3S$ [M+Na]$^+$ 490.1259, found 490.1256.

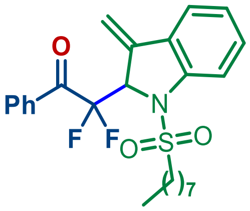

(28) According to the general procedure A, using *N*-triftosylhydrazone derived from 2,2,2-trifluoro-1-phenylethan-1-one (118.8 mg, 0.3 mmol), (1-(nonylsulfonyl)-1*H*-indol-3-yl)methanol (202.5 mg, 0.6 mmol), K_2CO_3 (82.9 mg, 0.6 mmol)**,** Tp^{Br3}Ag (33.0 mg, 10 mol%) and toluene (4.0 mL) afforded compound **28** (103.7 mg, 75% yield) as a white solid; mp: 123-124 °C; **^{1}H NMR** (500 MHz, $CDCl_3$) δ 8.05 (d, J = 7.5 Hz, 2H), 7.61 (t, J = 7.0 Hz, 1H), 7.54-7.40 (m, 4H), 7.32-7.25 (m, 1H), 7.14 (td, J = 7.5, 0.5 Hz, 1H), 5.74 (s, 1H), 5.60 (dd, J = 15.0, 8.5 Hz, 1H), 5.34 (s, 1H), 3.03-2.88 (m, 2H), 1.83-1.72 (m, 1H), 1.71-1.61 (m, 1H), 1.34-1.15 (m, 10H), 0.85 (t, J = 7.0 Hz, 3H); **^{13}C NMR** (150 MHz, $CDCl_3$) δ 188.74 (t, J = 27.0 Hz), 143.9, 138.1, 134.4, 132.9, 130.41, 130.39, 130.2 (t, J = 3.0 Hz), 128.6, 125.2, 121.1, 116.8, 116.1 (t, J = 259.5 Hz), 109.9, 67.0 (t, J = 24.0 Hz), 50.7, 31.6, 28.8, 28.7, 28.1, 22.6, 22.5, 14.0; **^{19}F NMR** (564 MHz, $CDCl_3$) δ -104.61 (d, J = 273.0 Hz), -112.03 (dd, J = 273.0, 14.7 Hz); **IR** (Film): 3068, 2927, 2857, 1698, 1599, 1463, 1358, 1286, 1160, 1071, 925, 828, 776 cm^{-1}; **HRMS** (ESI$^+$) m/z calcd for $C_{25}H_{29}F_2NNaO_3S$ [M+Na]$^+$ 484.1728, found 484.1729.

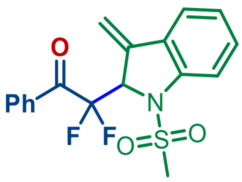

(29) According to the general procedure A, using *N*-triftosylhydrazone derived from 2,2,2-trifluoro-1-phenylethan-1-one (118.8 mg, 0.3 mmol), (1-(methylsulfonyl)-1*H*-indol-3-yl)methanol (135.2 mg, 0.6 mmol), K_2CO_3 (82.9 mg, 0.6 mmol), $Tp^{Br3}Ag$ (33.0 mg, 10 mol%) and toluene (4.0 mL) afforded compound **29** (87.1 mg, 80% yield) as a white solid; mp: 118-119 °C; **^{1}H NMR** (500 MHz, $CDCl_3$) δ 8.04 (d, *J* = 7.5 Hz, 2H), 7.61 (t, *J* = 7.5 Hz, 1H), 7.51-7.41 (m, 4H), 7.30 (t, *J* = 8.0 Hz, 1H), 7.15 (t, *J* = 7.5 Hz, 1H), 5.75 (s, 1H), 5.60 (dd, *J* = 14.5, 8.0 Hz, 1H), 5.35 (s, 1H), 2.84 (s, 3H); **^{13}C NMR** (150 MHz, $CDCl_3$) δ 188.6 (t, *J* = 28.5 Hz), 143.7, 137.8, 134.5, 132.7, 130.4, 130.1 (t, *J* = 3.0 Hz), 128.6, 125.5, 121.1, 117.1, 116.1 (t, *J* = 259.5 Hz), 110.2, 66.7 (t, *J* = 24.0 Hz), 37.2; **^{19}F NMR** (564 MHz, $CDCl_3$) δ -104.50 (dd, *J* = 273.5, 7.3 Hz), -112.09 (dd, *J* = 273.5, 14.7 Hz); **IR** (Film): 3054, 2983, 2305, 1698, 1598, 1462, 1359, 1264, 1167, 1071, 980, 895, 731 cm^{-1}; **HRMS** (ESI$^+$) m/z calcd for $C_{18}H_{15}F_2NNaO_3S$ $[M+Na]^+$ 386.0633, found 386.0642.

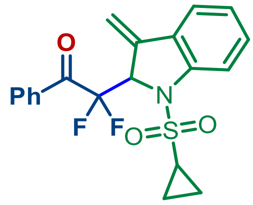

(30) According to the general procedure A, using *N*-triftosylhydrazone derived from 2,2,2-trifluoro-1-phenylethan-1-one (118.8 mg, 0.3 mmol), (1-(cyclopropylsulfonyl)-1*H*-indol-3-yl)methanol (150.8 mg, 0.6 mmol), K_2CO_3 (82.9 mg, 0.6 mmol), $Tp^{Br3}Ag$ (33.0 mg, 10 mol%) and toluene (4.0 mL) afforded compound **30** (96.9 mg, 83% yield) as a white solid; mp: 122-123 °C; **^{1}H NMR** (500 MHz, $CDCl_3$) δ 8.04 (d, *J* = 7.5 Hz, 2H), 7.61 (t, *J* = 7.5 Hz, 1H), 7.50-7.42 (m, 5H), 7.32-7.25 (m, 1H), 7.15 (td, *J* = 7.5, 1.0 Hz, 1H), 5.76 (s, 1H), 5.56 (ddt, *J* = 13.5, 9.5, 1.5 Hz, 2H), 5.37 (s, 1H), 2.27 (tt, *J* = 8.0, 5.0 Hz, 1H), 1.31-1.06 (m, 5H), 0.94-0.81 (m, 4H); **^{13}C NMR** (125 MHz, $CDCl_3$) δ 188.9 (t, *J* = 28.4 Hz), 144.1, 138.4, 134.4, 133.0, 131.1, 130.3, 130.2 (t, *J* = 3.7 Hz), 128.6, 125.5, 121.1, 117.8, 116.1 (t, *J* = 7.4 Hz), 109.7, 67.2 (t, *J* = 7.4 Hz), 27.9, 5.0, 4.9; **^{19}F NMR** (470 MHz, $CDCl_3$) δ -105.86 (dd, *J* = 272.2, 9.5 Hz), -111.59 (dd, *J* = 272.2, 14.0 Hz); **IR** (Film): 3066, 2926, 1694, 1598, 1463, 1360, 1265, 1161, 1054, 931, 828, 732 cm^{-1}; **HRMS** (ESI$^+$) m/z calcd for $C_{20}H_{18}F_2NO_3S$ $[M+H]^+$ 390.0970, found 390.0972.

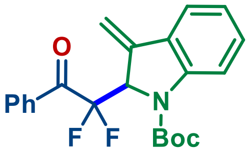

(31) According to the general procedure A, using *N*-triftosylhydrazone derived from 2,2,2-trifluoro-1-phenylethan-1-one (118.8 mg, 0.3 mmol), *tert*-butyl 3-(hydroxymethyl)-1*H*-indole-1-carboxylate (148.4 mg, 0.6 mmol), K_2CO_3 (82.9 mg, 0.6 mmol), $Tp^{Br3}Ag$ (33.0 mg, 10 mol%) and toluene (4.0 mL) afforded compound **31** (69.3 mg, 60% yield) as a white solid; mp: 110-111 °C;**^{1}H NMR** (500 MHz, $CDCl_3$) δ 8.03 (d, *J* = 7.5 Hz, 2H), 7.61 (t, *J* = 7.5 Hz, 1H), 7.51-7.42 (m, 3H), 7.28 (t, *J* = 7.5 Hz, 1H), 7.05 (t, *J* = 7.5 Hz, 1H), 5.74 (s, 1H), 5.60 (t, *J* = 12.0 Hz, 1H), 5.35 (s, 1H), 1.35 (s, 9H); **^{13}C NMR** (125 MHz, $CDCl_3$) δ 189.2, 151.5, 144.4, 138.1, 134.2, 133.2, 130.0 (t, *J* = 3.8 Hz), 130.0, 129.3, 128.6, 123.5, 120.3, 116.61, 116.56 (t, *J* = 258.8 Hz), 108.5, 82.8, 64.7 (t, *J* = 26.3 Hz), 27.8; **^{19}F NMR** (564 MHz, $CDCl_3$) δ -109.86; **IR** (Film): 3054, 1706, 1467, 1371, 1264, 1163, 904; 828, 719 cm^{-1}; **HRMS** (ESI$^+$) m/z calcd for $C_{22}H_{21}F_2NNaO_3S$ $[M+Na]^+$ 408.1382, found 408.1390.

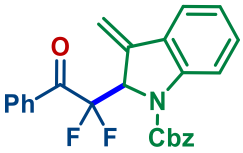

(32) According to the general procedure A, using *N*-triftosylhydrazone derived from 2,2,2-trifluoro-1-phenylethan-1-one (118.8 mg, 0.3 mmol), benzyl 3-(hydroxymethyl)-1*H*-indole-1-carboxylate (168.8 mg, 0.6 mmol), K_2CO_3 (82.9 mg, 0.6 mmol)**,** $Tp^{Br3}Ag$ (33.0 mg, 10 mol%) and toluene (4.0 mL) afforded compound **32** (96.8 mg, 77% yield) as a white solid; mp: 129-130 °C; **^{1}H NMR** (500 MHz, $CDCl_3$) δ 7.99 (d, *J* = 7.0 Hz, 2H), 7.81 (br, 1H), 7.59 (t, *J* = 7.0 Hz, 1H), 7.45-7.38 (m, 3H), 7.38-7.22 (m, 6H), 7.05 (t, *J* = 7.5 Hz, 1H), 5.71 (s, 2H), 5.28 (s, 1H), 5.17 (d, *J* = 12.0 Hz, 1H), 4.88 (br, 1H); **^{13}C NMR** (125 MHz, $CDCl_3$) δ 188.5 (t, *J* = 27.5 Hz), 137.9, 135.3, 134.3, 132.9, 130.1, 129.9 (t, *J* = 3.8 Hz), 128.7, 128.6, 128.5, 123.9, 120.4, 116.6, 116.5 (t, *J* = 257.5 Hz), 68.1, 64.8 (t, *J* = 25.0 Hz); **^{19}F NMR** (470 MHz, $CDCl_3$) δ -107.05-(-112.67) (m); **IR** (Film): 3054, 2965, 1706, 1467, 1424, 1372, 1264, 1162, 1098, 902, 718 cm^{-1}; **HRMS** (ESI$^+$) m/z calcd for $C_{25}H_{19}F_2NNaO_3S$ $[M+Na]^+$ 442.1225, found 442.1226.

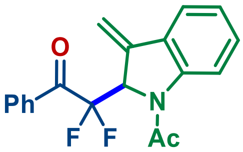

(33) According to the general procedure A, using *N*-triftosylhydrazone derived from 2,2,2-trifluoro-1-phenylethan-1-one (118.8 mg, 0.3 mmol), 1-(3-(hydroxymethyl)-1*H*-indol-1-yl)ethan-1-one (113.5 mg, 0.6 mmol), K_2CO_3 (82.9 mg, 0.6 mmol)**,** $Tp^{Br3}Ag$ (33.0 mg, 10 mol%) and toluene (4.0 mL) afforded compound **33** (63.8 mg, 65% yield) as a white solid; mp: 115-116 °C; **^{1}H NMR** (500 MHz, $CDCl_3$) δ 8.01 (s, 2H), 7.61 (t, *J* = 7.5 Hz, 1H), 7.46 (t, *J* = 8.0 Hz, 3H), 7.27 (dd, *J* = 14.5, 6.5 Hz, 1H), 7.09 (s, 1H), 5.75 (s, 2H), 5.46-5.17 (m, 1H), 2.33 (s, 3H); **^{19}F NMR** (470 MHz, $CDCl_3$) δ -97.40 (d, *J* = 280.2 Hz), -109.39-(-111.57) (m), -115.01 (d, *J* = 281.1 Hz); **IR** (Film): 3057, 2964, 1708, 1599, 1424, 1362, 1268, 1119, 1034, 903, 831, 736 718 cm^{-1}; **HRMS** (ESI$^+$) m/z calcd for $C_{19}H_{16}F_2NO_2$ $[M+H]^+$ 328.1144, found 328.1142.

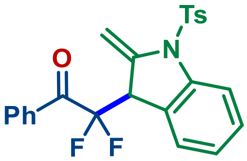

(34) According to the general procedure A, using *N*-triftosylhydrazone derived from 2,2,2-trifluoro-1-phenylethan-1-one (118.8 mg, 0.3 mmol), (1-tosyl-1*H*-indol-2-yl)methanol (180.8 mg, 0.6 mmol), K_2CO_3 (82.9 mg, 0.6 mmol)**,** $Tp^{Br3}Ag$ (33.0 mg, 10 mol%) and toluene (4.0 mL) afforded compound **34** (96.1 mg, 73% yield) as a white solid; mp: 130-131 °C; **^{1}H NMR** (500 MHz, DMSO) δ 7.83 (d, *J* = 7.5 Hz, 2H), 7.80-7.73 (m, 2H), 7.65 (d, *J* = 8.5 Hz, 2H), 7.57 (t, *J* = 7.5 Hz, 2H), 7.40 (t, *J* = 7.5 Hz, 1H), 7.31 (d, *J* = 8.5 Hz, 2H), 7.18 (d, *J* = 7.5 Hz, 1H), 7.10 (t, *J* = 7.5 Hz, 1H), 5.70 (s, 1H), 5.00 (t, *J* = 14.0 Hz, 1H), 4.84 (s, 1H), 2.32 (s, 3H); **^{13}C NMR** (125 MHz, DMSO) δ 189.2 (t, *J* = 28.8 Hz), 146.0, 143.6, 141.3, 136.0, 134.5, 132.6, 130.8, 130.7, 130.5, 130.1, 128.0, 127.2, 125.4, 124.6, 117.7 (t, *J* = 258.8 Hz), 115.6, 101.4, 49.8 (t, *J* = 23.8 Hz), 22.0; **^{19}F NMR** (470 MHz, DMSO) δ -100.92 (dd, *J* = 280.1, 13.2 Hz), -103.16 (dd, *J* = 280.1, 15.0 Hz); **IR** (Film): 2983, 1736, 1447, 1372, 1233, 1043, 938, 846, 786 cm^{-1}; **HRMS** (ESI$^+$) m/z calcd for $C_{24}H_{19}F_2NNaO_3S$ $[M+Na]^+$ 462.0946, found 462.0947.

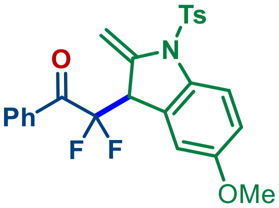

(35) According to the general procedure A, using *N*-triftosylhydrazone derived from 2,2,2-trifluoro-1-phenylethan-1-one (118.8 mg, 0.3 mmol), (5-methoxy-1-tosyl-1*H*-indol-2-yl)methanol (198.8 mg, 0.6 mmol), K_2CO_3 (82.9 mg, 0.6 mmol), $Tp^{Br3}Ag$ (33.0 mg, 10 mol%) and toluene (4.0 mL) afforded compound **35** (92.9 mg, 66% yield) as a white solid; mp: 144-145 °C; **^{1}H NMR** (500 MHz, CD_3CN) δ 7.84 (d, *J* = 8.0 Hz, 1H), 7.76 (d, *J* = 8.0 Hz, 2H), 7.65 (d, *J* = 8.5 Hz, 2H), 7.37 (t, *J* = 7.5 Hz, 1H), 7.34 (d, *J* = 8.5 Hz, 2H), 7.24 (d, *J* = 8.5 Hz, 2H), 7.19 (d, *J* = 7.5 Hz, 1H), 7.08 (t, *J* = 7.5 Hz, 1H), 5.77 (s, 1H), 4.89 (s, 1H), 4.76 (t, *J* = 13.5 Hz, 1H), 2.42 (s, 3H), 2.35 (s, 3H); **^{13}C NMR** (150 MHz, CD_3CN) δ 189.5 (t, *J* = 30.0 Hz), 157.5, 145.9, 141.7, 137.0, 135.4, 134.5, 132.9, 130.8, 130.3 (t, *J* = 3.0 Hz), 130.1, 129.5, 128.0, 117.6 (t, *J* = 256.5 Hz), 116.9, 115.5, 112.5, 101.8, 55.9, 50.0 (t, *J* = 24.0 Hz), 21.2; **^{19}F NMR** (564 MHz, CD_3CN) δ -101.43 (dd, *J* = 282.6, 13.0 Hz), -104.07 (dd, *J* = 282.6, 15.2 Hz); **HRMS** (ESI^+) m/z calcd for $C_{25}H_{21}F_2NNaO_4S$ $[M+Na]^+$ 492.1052, found 492.1062.

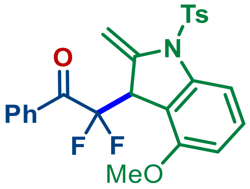

(36) According to the general procedure A, using *N*-triftosylhydrazone derived from 2,2,2-trifluoro-1-phenylethan-1-one (118.8 mg, 0.3 mmol), (4-methoxy-1-tosyl-1*H*-indol-2-yl)methanol (198.8 mg, 0.6 mmol), K_2CO_3 (82.9 mg, 0.6 mmol), $Tp^{Br3}Ag$ (33.0 mg, 10 mol%) and toluene (4.0 mL) afforded compound **36** (98.5 mg, 70% yield) as a white solid; mp: 138-139 °C; **^{1}H NMR** (500 MHz, DMSO) δ 7.88 (d, *J* = 8.0 Hz, 2H), 7.71 (d, *J* = 8.0 Hz, 3H), 7.55 (dd, *J* = 8.0, 7.5 Hz, 2H), 7.41-7.33 (m, 4H), 6.63 (dd, *J* = 8.0, 1.0 Hz, 1H), 5.80 (t, *J* = 1.5 Hz, 1H), 5.16 (d, *J* = 1.5 Hz, 1H), 4.87 (dd, *J* = 22.5, 6.5 Hz, 1H), 3.18 (s, 3H), 2.33 (s, 3H); **^{13}C NMR** (125 MHz, DMSO) δ 187.8 (t, *J* = 27.5 Hz), 156.0, 145.5, 144.3, 140.7, 134.9, 134.3, 132.5, 132.3, 130.3, 129.8, 129.3, 127.6, 116.5 (t, *J* = 256.3 Hz), 110.7 (d, *J* = 6.3 Hz), 107.9 (d, *J* = 1.3 Hz), 102.6, 54.8, 49.0 (t, *J* = 25.0 Hz), 21.5; **^{19}F NMR** (564 MHz, DMSO) δ -101.12 (dd, *J* = 251.0, 5.6 Hz), -110.09 (dd, *J* = 251.0, 22.0 Hz); **HRMS** (ESI^+) m/z calcd for $C_{25}H_{21}F_2NNaO_4S$ $[M+Na]^+$ 492.1052, found 492.1056.

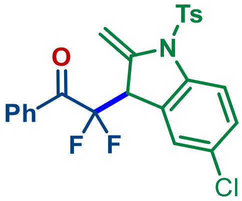

(37) According to the general procedure A, using *N*-triftosylhydrazone derived from 2,2,2-trifluoro-1-phenylethan-1-one (118.8 mg, 0.3 mmol), (5-chloro-1-tosyl-1*H*-indol-2-yl)methanol (201.5 mg, 0.6 mmol), K_2CO_3 (82.9 mg, 0.6 mmol), $Tp^{Br3}Ag$ (33.0 mg, 10 mol%) and toluene (4.0 mL) afforded compound **37** (97.9 mg, 69% yield) as a white solid; mp: 132-133 °C; **^{1}H NMR** (500 MHz, DMSO) δ 8.05 (d, *J* = 8.0 Hz, 2H), 7.79 (d, *J* = 2.0 Hz, 1H), 7.76 (t, *J* = 7.5 Hz, 1H), 7.61 (dd, *J* = 8.5, 8.0 Hz, 2H), 7.57 (d, *J* = 8.5 Hz, 2H), 7.49 (dd, *J* = 9.0, 2.0 Hz, 1H), 7.42 (d, *J* = 8.5 Hz, 1H), 7.28 (d, *J* = 8.0 Hz, 1H), 5.92 (s, 1H), 5.85 (t, *J* = 11.8 Hz, 1H), 5.38 (s, 1H), 2.29 (d, *J* = 11.5 Hz, 3H); **^{13}C NMR** (125 MHz, DMSO) δ 189.2 (t, *J* = 26.3 Hz), 146.1, 143.0, 136.7, 135.8, 134.2, 133.7, 133.5, 133.4, 125.4, 120.1, 119.3, 117.0 (t, *J* = 258.8 Hz), 114.1, 67.7 (t, *J* = 26.3 Hz), 21.9; **^{19}F NMR** (564 MHz, DMSO) δ -100.90 (dd, *J* = 258.3, 13.5 Hz), -101.45 (dd, *J* = 258.3, 10.2 Hz); **IR** (Film): 3052, 2968, 1693, 1602, 1462, 1365, 1264, 1172, 1070, 922, 896, 731 cm^{-1}.

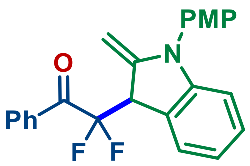

(38) According to the general procedure A, using *N*-triftosylhydrazone derived from 2,2,2-trifluoro-1-phenylethan-1-one (118.8 mg, 0.3 mmol), (1-(4-methoxyphenyl)-1*H*-indol-2-yl)methanol (151.9 mg, 0.6 mmol), K_2CO_3 (82.9 mg, 0.6 mmol)**,** $Tp^{Br3}Ag$ (33.0 mg, 10 mol%) and toluene (4.0 mL) afforded compound **38** (76.2 mg, 65% yield) as a white solid; mp: 152-153 °C; ^{1}**H NMR** (500 MHz, DMSO) δ 7.85 (d, *J* = 7.0 Hz, 2H), 7.80 (d, *J* = 8.0 Hz, 1H), 7.78-7.69 (m, 3H), 7.59-7.54 (m, 2H), 7.40 (t, *J* = 8.0 Hz, 1H), 7.18 (d, *J* = 7.5 Hz, 1H), 7.10 (t, *J* = 7.5 Hz, 1H), 7.03 (d, *J* = 9.0 Hz, 2H), 5.71 (t, *J* = 2.0 Hz, 1H), 5.00 (t, *J* = 14.3 Hz, 1H), 4.84 (t, *J* = 2.0 Hz, 1H), 3.79 (s, 3H); ^{13}**C NMR** (125 MHz, DMSO) δ 189.2 (t, *J* = 30.0 Hz), 164.7, 143.7, 141.46-141.18 (m), 136.0, 132.6, 130.8, 130.5, 130.4, 130.1, 128.9, 127.2, 125.3, 124.6 (d, *J* = 2.4 Hz), 117.7, 115.6, 115.4, 101.2, 56.7, 49.9 (t, *J* = 23.9 Hz); ^{19}**F NMR** (470 MHz, DMSO) δ -101.08 (dd, *J* = 280.6, 13.7 Hz), -103.01 (dd, *J* = 280.6, 15.3 Hz); **IR** (Film): 3015, 2984, 1736, 1447, 1372, 1233, 1043, 938, 846, 786 cm^{-1}; **HRMS** (ESI^+) m/z calcd for $C_{24}H_{19}F_2NNaO_4S$ $[M+Na]^+$ 478.0895, found 478.0890.

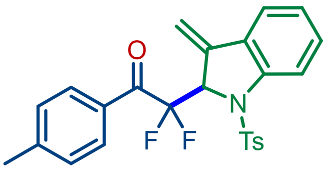

(39) According to the general procedure A, using *N*-triftosylhydrazone derived from 2,2,2-trifluoro-1-(p-tolyl)ethan-1-one (123.1 mg, 0.3 mmol), (1-tosyl-1*H*-indol-3-yl)methanol (180.8 mg, 0.6 mmol), K_2CO_3 (82.9 mg, 0.6 mmol)**,** $Tp^{Br3}Ag$ (33.0 mg, 10 mol%) and toluene (4.0 mL) afforded compound **39** (115.5 mg, 85% yield) as a white solid; mp: 148-149; ^{1}**H NMR** (600 MHz, $CDCl_3$) δ 7.99 (d, *J* = 7.8 Hz, 2H), 7.63 (d, *J* = 8.4 Hz, 1H), 7.44 (d, *J* = 8.4 Hz, 2H), 7.29-7.23 (m, 4H), 7.11 (d, *J* = 8.4 Hz, 2H), 7.08 (t, *J* = 7.8 Hz, 1H), 5.49 (s, 1H), 5.43 (dd, *J* = 12.0, 10.8 Hz, 1H), 5.15 (s, 1H), 2.41 (s, 3H), 2.30 (s, 3H); ^{13}**C NMR** (150 MHz, $CDCl_3$) δ 188.6 (t, *J* = 27.0 Hz), 145.6, 144.6, 144.0, 137.8, 133.5, 131.3, 130.5, 130.4 (t, *J* = 3.0 Hz), 130.0, 129.7, 129.4, 127.5, 125.7, 120.8, 118.5, 116.2 (t, *J* = 259.5 Hz), 109.5, 66.9 (dd, *J* = 25.5, 24.0 Hz), 21.9, 21.6; ^{19}**F NMR** (564 MHz, $CDCl_3$) δ -105.89 (dd, *J* = 270.7, 9.0 Hz), -111.10 (dd, *J* = 270.7, 13.0 Hz); **IR** (Film): 3054, 1710, 1596, 1461, 1365, 1264, 1127, 1089, 989, 896, 731 cm^{-1}; **HRMS** (ESI^+) m/z calcd for $C_{25}H_{21}F_2NNaO_3S$ $[M+Na]^+$ 476.1102, found 476.1106.

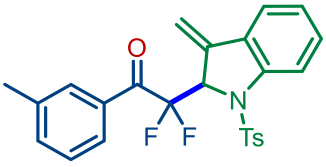

(40) According to the general procedure A, using *N*-triftosylhydrazone derived from 2,2,2-trifluoro-1-(*m*-tolyl)ethan-1-one (123.1 mg, 0.3 mmol), (1-tosyl-1*H*-indol-3-yl)methanol (180.8 mg, 0.6 mmol), K_2CO_3 (82.9 mg, 0.6 mmol)**,** $Tp^{Br3}Ag$ (33.0 mg, 10 mol%) and toluene (4.0 mL) afforded compound **40** (122.3 mg, 90% yield) as a white solid; mp: 135-136; ^{1}**H NMR** (600 MHz, $CDCl_3$) δ 7.89-7.86 (m, 2H), 7.62 (d, *J* = 7.8 Hz, 1H), 7.45-7.42 (m, 3H), 7.36 (t, *J* = 7.8 Hz, 1H), 7.29-7.24 (m, 2H), 7.12 (d, *J* = 8.4 Hz, 2H), 7.10-7.06 (m, 1H), 5.50 (s, 1H), 5.46-5.39 (m, 1H), 5.16 (s, 1H), 2.40 (s, 3H), 2.32 (s, 3H); ^{13}**C NMR** (150 MHz, $CDCl_3$) δ 189.2 (t, *J* = 28.5 Hz), 144.5, 144.0, 138.4, 137.8, 135.2, 133.6, 133.0, 131.3, 130.7 (t, *J* = 3.0 Hz), 130.1, 129.7, 128.5, 127.51,

127.47, 125.7, 120.8, 118.5, 116.1 (t, J = 259.5 Hz), 109.5, 66.8 (dd, J = 27.0, 25.5 Hz), 21.6, 21.4; **^{19}F NMR** (564 MHz, $CDCl_3$) δ -105.82 (dd, J = 270.7, 9.6 Hz), -110.86 (dd, J = 270.7, 13.0 Hz); **IR** (Film): 3054, 1719, 1597, 1422, 1369, 1264, 1173, 989, 896, 731 cm^{-1}; **HRMS** (ESI^+) m/z calcd for $C_{25}H_{21}F_2NNaO_3S$ $[M+Na]^+$ 476.1102, found 476.1097.

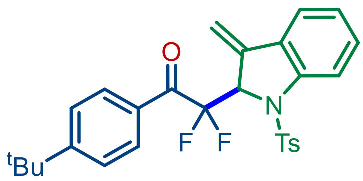

(41) According to the general procedure A, using *N*-triftosylhydrazone derived from 1-(4-(*tert*-butyl)phenyl)-2,2,2-trifluoroethan-1-one **(135.6 mg, 0.3 mmol)**, (1-tosyl-1*H*-indol-3-yl)methanol (180.8 mg, 0.6 mmol), K_2CO_3 (82.9 mg, 0.6 mmol)**,** $Tp^{Br3}Ag$ (33.0 mg, 10 mol%) and toluene (4.0 mL) afforded compound **41** (121.8 mg, 82% yield) as a white solid; mp: 149-150 °C; **^{1}H NMR** (500 MHz, $CDCl_3$) δ 8.02 (d, J = 8.0 Hz, 2H), 7.63 (d, J = 8.0 Hz, 1H), 7.50-7.44 (m, 4H), 7.28-7.23 (m, 2H), 7.12 (d, J = 8.0 Hz, 2H), 7.08 (t, J = 7.5 Hz, 1H), 5.50-5.42 (m, 2H), 5.15 (s, 1H), 2.32 (s, 3H), 1.34 (s, 9H); **^{13}C NMR** (125 MHz, $CDCl_3$) δ 188.7 (t, J = 27.5 Hz), 158.4, 144.5, 144.0, 137.8, 133.6, 131.3, 130.4, 130.3 (t, J = 2.5 Hz), 130.0, 129.6, 127.5, 125.6, 120.8, 118.5, 116.1 (t, J = 260.0 Hz), 109.5, 66.8 (dd, J = 26.3, 25.0 Hz), 35.3, 31.0, 21.6; **^{19}F NMR** (470 MHz, $CDCl_3$) δ -105.26 (dd, J = 271.2, 8.9 Hz), -111.29 (dd, J = 271.2, 13.6 Hz); **IR** (Film): 3052, 1760, 1598, 1448, 1372, 1265, 1174, 1097, 977, 813, 723 cm^{-1}; **HRMS** (ESI^+) m/z calcd for $C_{28}H_{27}F_2NNaO_3S$ $[M+Na]^+$ 518.1572, found 518.1573.

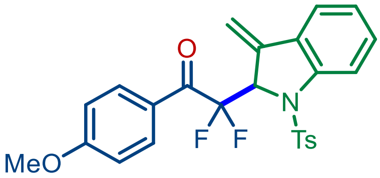

(42) According to the general procedure A, using *N*-triftosylhydrazone derived from 2,2,2-trifluoro-1-(4-methoxyphenyl)ethan-1-one (127.8 mg, 0.3 mmol), (1-tosyl-1*H*-indol-3-yl)methanol (180.8 mg, 0.6 mmol), K_2CO_3 (82.9 mg, 0.6 mmol)**,** $Tp^{Br3}Ag$ (33.0 mg, 10 mol%) and toluene (4.0 mL) afforded compound **42** (116.8 mg, 83% yield) as a white solid; mp: 155-156 °C; **^{1}H NMR** (500 MHz, $CDCl_3$) δ 8.09 (d, J = 8.5 Hz, 2H), 7.63 (d, J = 8.0 Hz, 1H), 7.44 (d, J = 8.5 Hz, 2H), 7.26 (m, 2H), 7.11 (d, J = 8.0 Hz, 2H), 7.08 (t, J = 7.5 Hz, 1H), 6.94 (d, J = 9.0 Hz, 2H), 5.49 (s, 1H), 5.45-5.38 (m, 1H), 5.16 (s, 1H), 3.89 (s, 3H), 2.32 (s, 3H); **^{13}C NMR** (150 MHz, $CDCl_3$) δ 187.1 (t, J = 28.5 Hz), 164.5, 144.5, 143.9, 137.7, 133.5, 132.9 (t, J = 3.0 Hz), 131.3, 130.0, 129.6, 127.4, 125.9, 125.6, 120.8, 118.4, 116.3 (t, J = 259.5 Hz), 113.9, 109.5, 66.9 (dd, J = 27.0, 24.0 Hz), 55.5, 21.5; **^{19}F NMR** (564 MHz, $CDCl_3$) δ -105.44 (dd, J = 269.0, 9.0 Hz), -110.89 (dd, J = 269.0, 13.5 Hz); **IR** (Film): 3054, 1705, 1594, 1463, 1352, 1264, 1160, 1070, 982, 831, 731 cm^{-1}; **HRMS** (ESI^+) m/z calcd for $C_{25}H_{21}F_2NNaO_4S$ $[M+Na]^+$ 492.1052, found 492.1060.

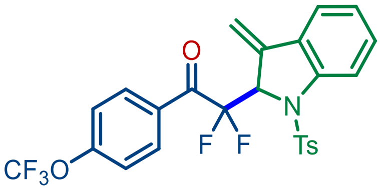

(43) According to the general procedure A, using *N*-triftosylhydrazone derived from 2,2,2-trifluoro-1-(4-(trifluoromethoxy)phenyl)ethan-1-one (144.0 mg, 0.3 mmol), (1-tosyl-1*H*-indol-3-yl)methanol (180.8 mg, 0.6 mmol), K_2CO_3 (82.9 mg, 0.6 mmol)**,** $Tp^{Br3}Ag$ (33.0 mg, 10 mol%) and toluene (4.0 mL) afforded compound **43**

(142.8 mg, 91% yield) as a white solid; mp: 137-138; **^{1}H NMR** (600 MHz, $CDCl_3$) δ 8.04 (d, *J* = 9.0 Hz, 2H), 7.51 (d, *J* = 8.4 Hz, 1H), 7.35 (d, *J* = 8.4 Hz, 2H), 7.22-7.17 (m, 4H), 7.04 (d, *J* = 8.4 Hz, 2H), 7.02 (t, *J* = 7.2 Hz, 1H), 5.45 (s, 1H), 5.27 (t, *J* = 11.4 Hz, 1H), 5.15 (s, 1H), 2.24 (s, 3H); **^{13}C NMR** (150 MHz, $CDCl_3$) δ 187.8 (t, *J* = 28.5 Hz), 153.4, 144.7, 143.7, 137.4, 133.2, 132.5 (t, *J* = 3.0 Hz), 131.3, 131.2, 130.1, 129.7, 127.4, 125.8, 120.8, 120.2 (q, *J* = 258.0 Hz), 120.0, 118.4, 116.0 (t, *J* = 259.5 Hz), 109.8, 66.8 (t, *J* = 25.5 Hz), 21.5; **^{19}F NMR** (564 MHz, $CDCl_3$) δ -57.47 (s), -108.24 (dd, *J* = 268.5, 11.8 Hz), -109.10 (dd, *J* = 268.5, 10.7 Hz); **IR** (Film): 3054, 2361, 1703, 1602, 1421, 1365, 1264, 1214, 1172, 1072, 895, 731 cm^{-1}; **HRMS** (ESI^+) m/z calcd for $C_{25}H_{18}F_5NNaO_4S$ $[M+Na]^+$ 546.0769, found 546.0770.

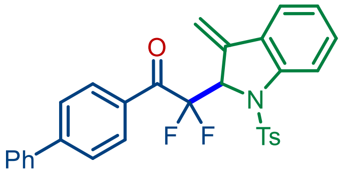

(44) According to the general procedure A, using *N*-triftosylhydrazone derived from 1-([1,1'-biphenyl]-4-yl)-2,2,2-trifluoroethan-1-one (141.6 mg, 0.3 mmol), (1-tosyl-1*H*-indol-3-yl)methanol (180.8 mg, 0.6 mmol), K_2CO_3 (82.9 mg, 0.6 mmol)**,** $Tp^{Br3}Ag$ (33.0 mg, 10 mol%) and toluene (4.0 mL) afforded compound **44** (148.3 mg, 96% yield) as a white solid; mp: 160-161 °C; **^{1}H NMR** (500 MHz, $CDCl_3$) δ 8.15 (d, *J* = 8.0 Hz, 2H), 7.69 (d, *J* = 8.5 Hz, 2H), 7.66-7.61 (m, 3H), 7.48-7.44 (m, 4H), 7.40 (t, *J* = 7.5 Hz, 1H), 7.28-7.24 (m, 2H), 7.12-7.07 (m, 3H), 5.52 (s, 1H), 5.45 (t, *J* = 11.0 Hz, 1H), 5.20 (s, 1H), 2.30 (s, 3H); **^{13}C NMR** (125 MHz, $CDCl_3$) δ 188.7 (t, *J* = 27.5 Hz), 146.9, 144.6, 144.0, 139.5, 137.7, 133.5, 131.8, 131.3, 130.9 (t, *J* = 3.8 Hz), 130.1, 129.7, 129.0, 128.6, 127.5, 127.4, 127.2, 125.8, 120.9, 118.5, 116.2 (t, *J* = 260.0 Hz), 109.7, 66.9 (t, *J* = 25.0 Hz), 21.6; **^{19}F NMR** (470 MHz, $CDCl_3$) δ -106.35 (dd, *J* = 270.3, 10.3 Hz), -110.48 (dd, *J* = 270.3, 13.2 Hz); **IR** (Film): 3054, 1689, 1596, 1497, 1364, 1264, 1165, 971, 895, 731 cm^{-1}; **HRMS** (ESI^+) m/z calcd for $C_{30}H_{23}F_2NNaO_3S$ $[M+Na]^+$ 538.1259, found 538.1287.

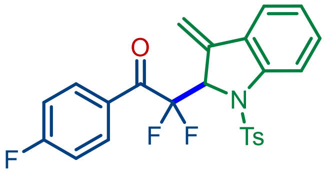

(45) According to the general procedure A, using *N*-triftosylhydrazone derived from 2,2,2-trifluoro-1-(4-fluorophenyl)ethan-1-one (124.2 mg, 0.3 mmol), (1-tosyl-1*H*-indol-3-yl)methanol (180.8 mg, 0.6 mmol), K_2CO_3 (82.9 mg, 0.6 mmol)**,** $Tp^{Br3}Ag$ (33.0 mg, 10 mol%) and toluene (4.0 mL) afforded compound **45** (123.4 mg, 90% yield) as a White solid; mp: 168-169 °C; **^{1}H NMR** (600 MHz, $CDCl_3$) δ 8.12 (dd, *J* = 8.4, 5.4 Hz, 2H), 7.60 (d, *J* = 7.8 Hz, 1H), 7.42 (d, *J* = 8.4 Hz, 2H), 7.29-7.25 (m, 2H), 7.16-7.08 (m, 5H), 5.52 (s, 1H), 5.36 (t, *J* = 11.4 Hz, 1H), 5.21 (s, 1H), 2.32 (s, 3H); **^{13}C NMR** (150 MHz, $CDCl_3$) δ 187.6 (t, *J* = 27.0 Hz), 166.4 (d, *J* = 257.0 Hz), 144.7, 143.8, 137.5, 133.34 (t, *J* = 3.5 Hz), 133.27 (t, *J* = 3.5 Hz), 131.3, 130.1, 129.7, 129.6, 127.5, 125.8, 120.9, 118.5, 116.1 (t, *J* = 259.5 Hz), 115.9 (d, *J* = 22.0 Hz), 109.8, 66.9 (dd, *J* = 27.0, 25.5 Hz), 21.6; **^{19}F NMR** (564 MHz, $CDCl_3$) δ -102.11-(-102.19) (m), -107.63 (dd, *J* = 268.5, 11.3 Hz), -109.45 (dd, *J* = 269.0, 11.8 Hz); **IR** (Film): 3054, 2986, 1692, 1599, 1462, 1360, 1264, 1170, 1070, 932, 895, 732 cm^{-1}; **HRMS** (ESI^+) m/z calcd for $C_{24}H_{18}F_3NNaO_3S$ $[M+Na]^+$ 480.0852, found 480.0872.

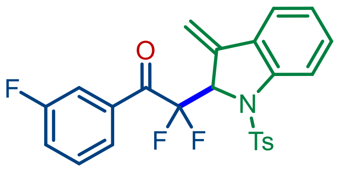

(46) According to the general procedure A, using *N*-triftosylhydrazone derived from 2,2,2-trifluoro-1-(3-fluorophenyl)ethan-1-one (124.2 mg, 0.3 mmol), (1-tosyl-1*H*-indol-3-yl)methanol (180.8 mg, 0.6 mmol), K_2CO_3 (82.9 mg, 0.6 mmol), $Tp^{Br3}Ag$ (33.0 mg, 10 mol%) and toluene (4.0 mL) afforded compound **46** (116.5 mg, 85% yield) as a white solid; mp: 146-147 °C; **^{1}H NMR** (600 MHz, $CDCl_3$) δ 7.89 (d, *J* = 7.8 Hz, 1H), 7.72 (d, *J* = 9.6 Hz, 1H), 7.62 (d, *J* = 7.8 Hz, 1H), 7.46 (td, *J* = 7.8, 5.4 Hz, 1H), 7.42 (d, *J* = 8.4 Hz, 2H), 7.32 (td, *J* = 8.4, 2.4 Hz, 1H), 7.30-7.26 (m, 2H), 7.13-7.08 (m, 3H), 5.53 (s, 1H), 5.36 (t, *J* = 12.0 Hz, 1H), 5.21 (s, 1H), 2.31 (s, 3H); **^{13}C NMR** (150 MHz, $CDCl_3$) δ 188.1 (t, *J* = 27.0 Hz), 162.4 (d, *J* = 248.0 Hz), 144.6, 143.8, 137.5, 134.9 (d, *J* = 6.0 Hz), 133.3, 131.2, 130.2 (d, *J* = 7.5 Hz), 130.1, 129.6, 127.4, 126.1 (d, *J* = 3.0 Hz), 125.8, 121.4 (d, *J* = 21.0 Hz), 120.8, 118.5, 116.94 (dt, *J* = 22.5, 3.0 Hz), 115.9 (t, *J* = 259.5 Hz), 109.7, 66.8 (dd, *J* = 27.0, 25.5 Hz), 21.5; **^{19}F NMR** (564 MHz, $CDCl_3$) δ -108.17 (dd, *J* = 269.6, 11.8 Hz), -109.60 (dd, *J* = 269.6, 11.3 Hz), -111.35-(-111.49) (m); **IR** (Film): 3056, 2969, 1703, 1589, 1461, 1356, 1263, 1163, 1052, 967, 865, 732 cm^{-1}; **HRMS** (ESI^+) m/z calcd for $C_{24}H_{18}F_3NNaO_3S$ $[M+Na]^+$ 480.0852, found 480.0860.

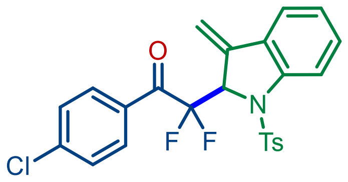

(47) According to the general procedure A, using *N*-triftosylhydrazone derived from 1-(4-chlorophenyl)-2,2,2-trifluoroethan-1-one (129 mg, 0.3 mmol), (1-tosyl-1*H*-indol-3-yl)methanol (180.8 mg, 0.6 mmol), K_2CO_3 (82.9 mg, 0.6 mmol), $Tp^{Br3}Ag$ (33.0 mg, 10 mol%) and toluene (4.0 mL) afforded compound **47** (130.5 mg, 92% yield) as a white solid; mp: 153-154 °C; **^{1}H NMR** (500 MHz, $CDCl_3$) δ 8.00 (d, *J* = 8.5 Hz, 2H), 7.60 (d, *J* = 8.5 Hz, 1H), 7.42 (dd, *J* = 13.5, 9.0 Hz, 4H), 7.26 (d, *J* = 7.5 Hz, 2H), 7.10 (m, 3H), 5.52 (s, 1H), 5.33 (t, *J* = 11.5 Hz, 1H), 5.20 (s, 1H), 2.31 (s, 3H); **^{13}C NMR** (125 MHz, $CDCl_3$) δ 188.1 (t, *J* = 27.0 Hz), 144.7, 143.7, 141.0, 137.4, 133.2, 131.7 (t, *J* = 3.0 Hz), 131.4, 131.2, 130.1, 129.6, 128.9, 127.4, 125.8, 120.8, 118.4, 116.0 (t, *J* = 259.5 Hz), 109.8, 66.8 (t, *J* = 25.5 Hz), 21.5; **^{19}F NMR** (564 MHz, $CDCl_3$) δ -108.21 (dd, *J* = 268.5, 11.8 Hz), -109.39 (dd, *J* = 268.5, 11.3 Hz); **IR** (Film): 3065, 2924, 1694, 1598, 1447, 1359, 1285, 1168, 1069, 978, 812, 713 cm^{-1}; **HRMS** (ESI^+) m/z calcd for $C_{24}H_{18}ClF_2NNaO_3S$ $[M+Na]^+$ 496.0556, found 496.0559.

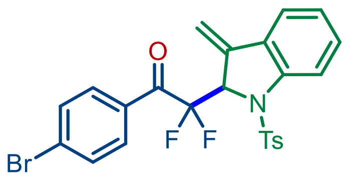

(48) According to the general procedure A, using *N*-triftosylhydrazone derived from 1-(4-bromophenyl)-2,2,2-trifluoroethan-1-one (142.2 mg, 0.3 mmol), (1-tosyl-1*H*-indol-3-yl)methanol (180.8 mg, 0.6 mmol), K_2CO_3 (82.9 mg, 0.6 mmol), $Tp^{Br3}Ag$ (33.0 mg, 10 mol%) and toluene (4.0 mL) afforded compound **48** (134.9 mg, 87% yield) as a white solid; mp: 144-145 °C; **^{1}H NMR** (500 MHz, $CDCl_3$) δ 7.93 (d, *J* = 8.5 Hz, 2H), 7.63-7.59 (m, 3H), 7.42 (d, *J* = 8.0 Hz, 2H), 7.30-7.25 (m, 2H), 7.14-7.07 (m, 3H), 5.53 (s, 1H), 5.33 (t, *J* = 11.5 Hz, 1H), 5.21 (s, 1H), 2.32 (s, 3H); **^{13}C NMR** (150 MHz, $CDCl_3$) δ 188.3 (t, *J* = 28.5 Hz), 144.7, 143.7, 137.4, 133.2, 131.92,

131.86, 131.7 (t, $J = 3.0$ Hz), 131.2, 130.1, 130.0, 129.7, 127.4, 125.8, 120.8, 118.4, 116.0 (t, $J = 259.5$ Hz), 109.8, 66.8 (t, $J = 27.0$ Hz), 21.5; **^{19}F NMR** (564 MHz, $CDCl_3$) δ -108.30 (dd, $J = 268.5$, 11.8 Hz), -109.37 (dd, $J = 268.5$, 11.3 Hz); **IR** (Film): 3054, 2986, 1701, 1584, 1460, 1364, 1264, 1171, 1071, 971, 895, 731 cm^{-1}; **HRMS** (ESI^+) m/z calcd for $C_{24}H_{18}BrF_2NNaO_3S$ $[M+Na]^+$ 540.0050, found 540.0055.

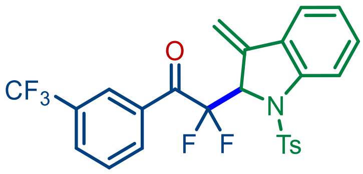

(49) According to the general procedure A, using *N*-triftosylhydrazone derived from 2,2,2-trifluoro-1-(3-(trifluoromethyl)phenyl)ethan-1-one (139.2 mg, 0.3 mmol), (1-tosyl-1*H*-indol-3-yl)methanol (180.8 mg, 0.6 mmol), K_2CO_3 (82.9 mg, 0.6 mmol)**,** $Tp^{Br3}Ag$ (33.0 mg, 10 mol%) and toluene (4.0 mL) afforded compound **49** (124.7 mg, 82% yield) as a white solid; mp: 123-124; **^{1}H NMR** (600 MHz, $CDCl_3$) δ 8.30-8.26 (m, 2H), 7.86 (d, $J = 7.8$ Hz, 1H), 7.63 (t, $J = 7.8$ Hz, 1H), 7.59 (d, $J = 8.4$ Hz, 1H), 7.41 (d, $J = 7.8$ Hz, 2H), 7.29-7.26 (m, 2H), 7.13-7.09 (m, 3H), 5.55 (s, 1H), 5.32 (dd, $J = 12.0$, 10.2 Hz, 1H), 5.26 (s, 1H), 2.31 (s, 3H); **^{13}C NMR** (125 MHz, $CDCl_3$) δ 188.3 (t, $J = 28.1$ Hz), 144.7, 143.6, 137.3, 133.7, 133.4, 133.1, 131.2, 131.1 (q, $J = 32.9$ Hz), 130.5 (q, $J = 3.1$ Hz), 130.2, 129.7, 129.2, 127.4, 127.2 (d, $J = 3.1$ Hz), 125.9, 123.5 (q, $J = 271.4$ Hz), 120.8, 118.5, 116.0 (dd, $J = 260.5$, 258.6 Hz), 109.9, 66.8 (dd, $J = 26.4$, 25.9 Hz), 21.5; **^{19}F NMR** (564 MHz, $CDCl_3$) δ -62.88 (s), -108.49 (dd, $J = 267.9$, 10.2 Hz), -109.70 (dd, $J = 267.9$, 13.0 Hz); **IR** (Film): 3054, 2985, 1708, 1600, 1421, 1365, 1264, 1172, 1074, 895, 731 cm^{-1}; **HRMS** (ESI^+) m/z calcd for $C_{25}H_{18}F_5NNaO_3S$ $[M+Na]^+$ 530.0820, found 530.0818.

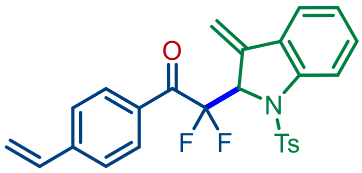

(50) According to the general procedure A, using *N*-triftosylhydrazone derived from 2,2,2-trifluoro-1-(4-vinylphenyl)ethan-1-one (126.6 mg, 0.3 mmol), (1-tosyl-1*H*-indol-3-yl)methanol (180.8 mg, 0.6 mmol), K_2CO_3 (82.9 mg, 0.6 mmol)**,** $Tp^{Br3}Ag$ (33.0 mg, 10 mol%) and toluene (4.0 mL) afforded compound **50** (106.0 mg, 76% yield) as a white solid; mp: 141-142 ℃; **^{1}H NMR** (600 MHz, $CDCl_3$) δ 8.05 (d, $J = 8.4$ Hz, 2H), 7.62 (d, $J = 7.8$ Hz, 1H), 7.49 (d, $J = 8.4$ Hz, 2H), 7.44 (d, $J = 8.4$ Hz, 2H), 7.29-7.24 (m, 2H), 7.11 (d, $J = 8.4$ Hz, 2H), 7.08 (t, $J = 7.8$ Hz, 1H), 6.75 (dd, $J = 17.4$, 10.8 Hz, 1H), 5.91 (d, $J = 17.4$ Hz, 1H), 5.50 (s, 1H), 5.44 (d, $J = 11.4$ Hz, 1H), 5.43-5.38 (m, 2H), 5.17 (s, 1H), 2.31 (s, 3H); **^{13}C NMR** (150 MHz, $CDCl_3$) δ 188.3 (t, $J = 28.5$ Hz), 144.5, 143.9, 143.2, 137.7, 135.8, 133.5, 132.1, 131.3, 130.7 (t, $J = 3.0$ Hz), 130.0, 129.6, 127.4, 126.3, 125.7, 120.8, 118.5, 117.7, 116.1 (t, $J = 259.5$ Hz), 109.5, 66.8 (t, $J = 25.5$ Hz), 21.5; **^{19}F NMR** (564 MHz, $CDCl_3$) δ -106.48 (dd, $J = 269.6$, 10.2 Hz), -110.62 (dd, $J = 269.6$, 13.0 Hz); **IR** (Film): 3054, 2963, 1694, 1598, 1458, 1359, 1263, 1166, 1070, 973, 896, 728 cm^{-1}; **HRMS** (ESI^+) m/z calcd for $C_{26}H_{21}F_2NNaO_3S$ $[M+Na]^+$ 488.1102, found 488.1120.

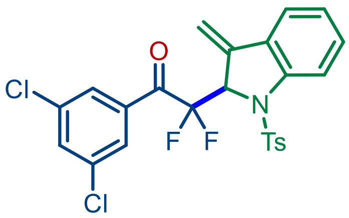

(51) According to the general procedure A, using *N*-triftosylhydrazone derived from 1-(3,5-dichlorophenyl)-2,2,2-trifluoroethan-1-one (139.2 mg, 0.3 mmol), (1-tosyl-1*H*-indol-3-yl)methanol (180.8 mg, 0.6 mmol), K_2CO_3 (82.9 mg, 0.6 mmol)**,** $Tp^{Br3}Ag$ (33.0 mg, 10 mol%) and toluene (4.0 mL) afforded compound **51** (117.1 mg, 77% yield) as a white solid; mp: 138-139; **^{1}H NMR** (600 MHz, $CDCl_3$) δ 7.91 (d, *J* = 1.8 Hz, 2H), 7.64 (d, *J* = 7.8 Hz, 1H), 7.59 (t, *J* = 1.8 Hz, 1H), 7.41 (d, *J* = 8.4 Hz, 2H), 7.33-7.27 (m, 2H), 7.14-7.11 (m, 3H), 5.56 (s, 1H), 5.30-5.25 (m, 1H), 5.25 (s, 1H), 2.32 (s, 3H); **^{13}C NMR** (150 MHz, $CDCl_3$) δ 187.3 (dd, *J* = 29.0, 27.5 Hz), 144.8, 143.6, 137.2, 135.5, 135.4, 133.9, 133.1, 131.2, 130.3, 129.7, 128.6 (t, *J* = 3.2 Hz), 127.4, 126.0, 120.9, 118.5, 115.9 (dd, *J* = 260.7, 258.5 Hz), 110.0, 66.7 (dd, *J* = 28.2, 25.8 Hz), 21.6; **^{19}F NMR** (564 MHz, $CDCl_3$) δ -108.37 (dd, *J* = 267.3, 9.0 Hz), -110.11 (dd, *J* = 267.3, 14.1 Hz); **IR** (Film): 3054, 2361, 1711, 1565, 1461, 1365, 1264, 1172, 1079, 953, 895, 732 cm^{-1}; **HRMS** (ESI^+) m/z calcd for $C_{24}H_{17}Cl_2F_2NNaO_3S$ $[M+Na]^+$ 530.0167, found 530.0170.

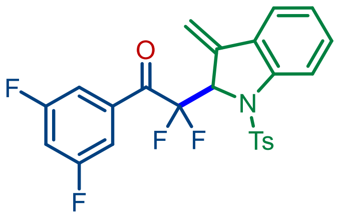

(52) According to the general procedure A, using *N*-triftosylhydrazone derived from 1-(3,5-difluorophenyl)-2,2,2-trifluoroethan-1-one (129.6 mg, 0.3 mmol), (1-tosyl-1*H*-indol-3-yl)methanol (180.8 mg, 0.6 mmol), K_2CO_3 (82.9 mg, 0.6 mmol)**,** $Tp^{Br3}Ag$ (33.0 mg, 10 mol%) and toluene (4.0 mL) afforded compound **52** (105.5 mg, 74% yield) as a white solid; mp: 129-130; **^{1}H NMR** (600 MHz, $CDCl_3$) δ 7.64 (d, *J* = 7.8 Hz, 1H), 7.60-7.57 (m, 2H), 7.41 (d, *J* = 8.4 Hz, 2H), 7.33-7.28 (m, 2H), 7.14-7.06 (m, 3H), 7.08 (m, 1H), 5.55 (s, 1H), 5.32-5.27 (m, 1H), 5.24 (s, 1H), 2.32 (s, 3H); **^{13}C NMR** (125 MHz, $CDCl_3$) δ 187.2 (dd, *J* = 28.8, 27.5 Hz), 162.7 (dd, *J* = 250.0, 12.0 Hz), 144.8, 143.7, 137.3, 133.1, 131.2, 130.3, 129.7, 127.5, 126.0, 120.9, 118.5, 115.9 (dd, *J* = 261.3, 258.8 Hz), 113.6-113.56-113.39 (m), 109.9, 109.7 (t, *J* = 25.0 Hz), 66.8 (dd, *J* = 27.5, 25.0 Hz), 21.6; **^{19}F NMR** (564 MHz, $CDCl_3$) δ -107.71 (t, *J* = 6.5 Hz), -108.58 (dd, *J* = 268.5, 9.6 Hz), -110.08 (dd, *J* = 268.5, 13.0 Hz); **IR** (Film): 3067, 2925, 2361, 1711, 1596, 1461, 1364, 1267, 1171, 1078, 989, 871, 734 cm^{-1}; **HRMS** (ESI^+) m/z calcd for $C_{24}H_{17}F_4NNaO_3S$ $[M+Na]^+$ 498.0758, found 498.0767.

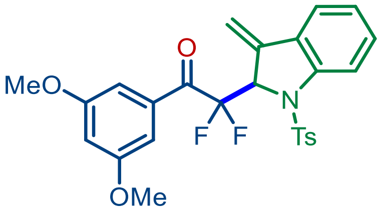

(53) According to the general procedure A, using *N*-triftosylhydrazone derived from 1-(3,5-dimethoxyphenyl)-2,2,2-trifluoroethan-1-one (136.8 mg, 0.3 mmol), (1-tosyl-1*H*-indol-3-yl)methanol (180.8 mg, 0.6 mmol), K_2CO_3 (82.9 mg, 0.6 mmol)**,** $Tp^{Br3}Ag$ (33.0 mg, 10 mol%) and toluene (4.0 mL) afforded compound **53** (134.7 mg, 90% yield) as a white solid; mp: 152-153; **^{1}H NMR** (500 MHz, $CDCl_3$) δ 7.62 (d, *J* = 8.5 Hz, 1H), 7.44 (d, *J* = 8.5 Hz, 2H), 7.30-7.23 (m, 4H), 7.12 (d, *J* = 8.0 Hz, 2H), 7.09 (t, *J* = 7.0 Hz, 1H), 6.72 (t, *J* = 2.4 Hz, 1H), 5.51 (s, 1H), 5.41 (t, *J* = 10.5 Hz, 1H), 5.15 (s, 1H), 3.84 (s, 6H), 2.32 (s, 3H); **^{13}C NMR** (125 MHz, $CDCl_3$) δ 188.5 (t, *J* = 27.6 Hz), 160.6, 144.6, 143.8, 137.6, 134.5, 133.4, 131.1, 130.0, 129.6, 127.4, 125.7, 120.8, 118.3, 115.93 (t, *J* = 260.0 Hz), 109.5, 107.6, 66.9 (t, *J* = 24.9 Hz), 55.6, 21.5; **^{19}F NMR** (564 MHz, $CDCl_3$) δ -106.04 (dd, *J* = 267.9, 10.2 Hz), -110.60 (dd, *J* = 267.9, 13.0 Hz); **IR** (Film): 3054, 1699, 1595, 1460, 1363, 1264, 1171, 1066, 936, 896, 731 cm^{-1}; **HRMS** (ESI^+) m/z calcd for $C_{26}H_{23}F_2NNaO_5S$ $[M+Na]^+$ 522.1157, found 522.1173.

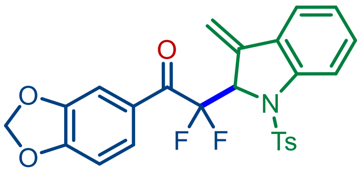

(54) According to the general procedure A, using *N*-triftosylhydrazone derived from 1-(benzo[*d*][1,3]dioxol-5-yl)-2,2,2-trifluoroethan-1-one (132.0 mg, 0.3 mmol), (1-tosyl-1*H*-indol-3-yl)methanol (180.8 mg, 0.6 mmol), K_2CO_3 (82.9 mg, 0.6 mmol), $Tp^{Br3}Ag$ (33.0 mg, 10 mol%) and toluene (4.0 mL) afforded compound **54** (115.9 mg, 80% yield) as a white solid; mp: 124-125; **^{1}H NMR** (500 MHz, $CDCl_3$) δ 7.78 (d, *J* = 8.0 Hz, 1H), 7.64 (d, *J* = 7.5 Hz, 1H), 7.52 (s, 1H), 7.44 (d, *J* = 8.0 Hz, 2H), 7.30-7.25 (m, 2H), 7.12-7.08 (m, 3H), 6.87 (d, *J* = 8.5 Hz, 1H), 6.06 (s, 2H), 5.50 (s, 1H), 5.40 (dd, *J* = 11.5, 10.5 Hz, 1H), 5.17 (s, 1H), 2.31 (s, 3H); **^{13}C NMR** (150 MHz, $CDCl_3$) δ 186.8 (t, *J* = 27.0 Hz), 153.0, 148.1, 144.5, 143.9, 137.7 133.4, 131.3, 130.0, 129.6, 127.7 (t, *J* = 4.5 Hz), 127.5, 127.4, 125.6, 120.8, 118.4, 116.2 (t, *J* = 259.5 Hz), 109.7, 109.5, 108.1, 102.1, 66.8 (dd, *J* = 27.0, 25.5 Hz), 21.5; **^{19}F NMR** (564 MHz, $CDCl_3$) δ -105.71 (dd, *J* = 269.0, 10.2 Hz), -110.28 (dd, *J* = 269.0, 13.5 Hz); **IR** (Film): 3054, 1719, 1597, 1422, 1369, 1264, 1173, 989, 896, 731 cm^{-1}; **HRMS** (ESI^+) m/z calcd for $C_{25}H_{19}F_2NNaO_5S$ $[M+Na]^+$ 506.0844, found 506.0850.

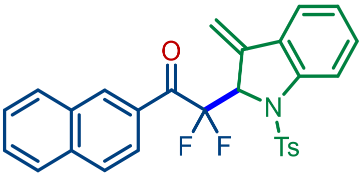

(55) According to the general procedure A, using *N*-triftosylhydrazone derived from 2,2,2-trifluoro-1-(naphthalen-2-yl)ethan-1-one (133.8 mg, 0.3 mmol), (1-tosyl-1*H*-indol-3-yl)methanol (180.8 mg, 0.6 mmol), K_2CO_3 (82.9 mg, 0.6 mmol), $Tp^{Br3}Ag$ (33.0 mg, 10 mol%) and toluene (4.0 mL) afforded compound **55** (127.6 mg, 87% yield) as a white solid; mp: 102-103 °C; **^{1}H NMR** (600 MHz, $CDCl_3$) δ 8.73 (s, 1H), 8.05 (dd, *J* = 8.4, 1.5 Hz, 1H), 7.98 (d, *J* = 7.8 Hz, 1H), 7.87 (t, *J* = 9.0 Hz, 2H), 7.66-7.62 (m, 1H), 7.59 (d, *J* = 8.4 Hz, 1H), 7.58-7.54 (m, 1H), 7.44 (d, *J* = 8.4 Hz, 2H), 7.28-7.22 (m, 2H), 7.10 (d, *J* = 8.4 Hz, 2H), 7.08 (td, *J* = 7.8, 1.2 Hz, 1H), 5.52 (s, 1H), 5.49 (dd, *J* = 12.0, 10.8 Hz, 1H), 5.21 (s, 1H), 2.30 (s, 3H); **^{13}C NMR** (150 MHz, $CDCl_3$) δ 188.8 (t, *J* = 27.5 Hz), 144.5, 143.9, 137.7, 136.0, 133.5, 133.3 (t, *J* = 4.5 Hz), 132.2, 131.2, 130.3, 130.2, 130.0, 129.6, 129.4, 128.4, 127.7, 127.4, 126.9, 125.7, 124.8, 120.8, 118.4, 116.3 (t, *J* = 259.8 Hz), 109.6, 67.0 (t, *J* = 25.2 Hz), 21.5; **^{19}F NMR** (564 MHz, $CDCl_3$) δ -105.54 (dd, *J* = 269.0, 9.6 Hz), -110.20 (dd, *J* = 269.0, 13.0 Hz); **IR** (Film): 3067, 2925, 2361, 1711, 1596, 1461, 1364, 1267, 1171, 1078, 989, 871, 734 cm^{-1}; **HRMS** (ESI^+) m/z calcd for $C_{28}H_{21}F_2NNaO_3S$ $[M+Na]^+$ 512.1102, found 512.1101.

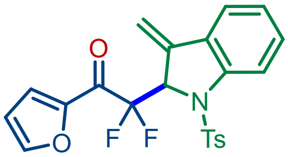

(56) According to the general procedure A, using *N*-triftosylhydrazone derived from 2,2,2-trifluoro-1-(furan-2-yl)ethan-1-one (115.8 mg, 0.3 mmol), (1-tosyl-1*H*-indol-3-yl)methanol (180.8 mg, 0.6 mmol), K_2CO_3 (82.9 mg, 0.6 mmol), $Tp^{Br3}Ag$ (33.0 mg, 10 mol%) and toluene (4.0 mL) afforded compound **56** (51.5 mg, 40% yield) as a white solid; mp: 134-135 °C; **^{1}H NMR** (600 MHz, $CDCl_3$) δ 7.68 (s, 1H), 7.55 (d, *J* = 8.4 Hz, 1H), 7.44 (s, 1H), 7.35 (d, *J* = 8.4 Hz, 2H), 7.21 (d, *J* = 7.8 Hz, 2H), 7.05-7.02 (m, 3H), 6.61-6.54 (m, 1H), 5.46 (s, 1H), 5.23 (t, *J* = 12.6 Hz, 1H), 5.15 (s, 1H), 2.25 (s, 3H); **^{13}C NMR** (150 MHz, $CDCl_3$) δ 176.5 (t, *J* = 28.5 Hz), 149.3, 149.1,

144.6, 143.8, 137.3, 133.4, 131.1, 130.1, 129.7, 127.4, 125.8, 124.1 (t, J = 6.0 Hz), 120.9, 118.4, 115.4 (t, J = 258.0 Hz), 113.0, 109.9, 66.7 (t, J = 25.5 Hz), 21.6; **^{19}F NMR** (564 MHz, $CDCl_3$) δ -112.44 (dd, J = 262.3, 8.6 Hz), -113.49 (dd, J = 262.3, 10.2 Hz); **IR** (Film): 3054, 1681, 1461, 1365, 1264, 1172, 1089, 895, 731 cm^{-1}; **HRMS** (ESI$^+$) m/z calcd for $C_{22}H_{17}F_2NNaO_4S$ [M+Na]$^+$ 452.0739, found 452.0743.

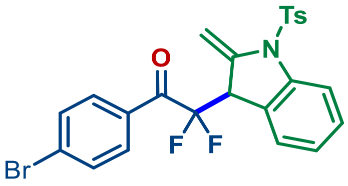

(57) According to the general procedure A, using N-triftosylhydrazone derived from 1-(4-bromophenyl)-2,2,2-trifluoroethan-1-one (142.2 mg, 0.3 mmol), (1-tosyl-1H-indol-2-yl)methanol (180.8 mg, 0.6 mmol), K_2CO_3 (82.9 mg, 0.6 mmol), Tp^{Br3}Ag (33.0 mg, 10 mol%) and toluene (4.0 mL) afforded compound **57** (111.7 mg, 72% yield) as a colorless oil; **^{1}H NMR** (500 MHz, DMSO) δ 7.79 (t, J = 8.5 Hz, 3H), 7.74 (d, J = 8.5 Hz, 2H), 7.64 (d, J = 8.5 Hz, 2H), 7.40 (t, J = 8.5 Hz, 1H), 7.30 (d, J = 8.5 Hz, 2H), 7.17 (d, J = 7.5 Hz, 1H), 7.10 (t, J = 7.5 Hz, 1H), 5.69 (s, 1H), 5.00 (t, J = 14.0 Hz, 1H), 4.83 (s, 1H), 2.32 (s, 3H); **^{13}C NMR** (125 MHz, DMSO) δ 188.5 (t, J = 30.0 Hz), 146.0, 143.5, 141.1, 134.5, 133.2, 132.3, 131.6, 130.8, 130.7, 130.6, 128.0, 127.2, 125.4, 124.4, 117.6 (t, J = 256.3 Hz), 115.5, 101.5, 49.7 (t, J = 23.8 Hz), 22.0; **^{19}F NMR** (470 MHz, DMSO) δ -101.01 (dd, J = 279.7, 13.2 Hz), -103.30 (dd, J = 279.7, 15.0 Hz); **IR** (Film): 3054, 2986, 1698, 1601, 1421, 1364, 1264, 1172, 895, 731 cm^{-1}; **HRMS** (ESI$^+$) m/z calcd for $C_{24}H_{18}BrF_2NNaO_3S$ [M+Na]$^+$ 540.0051, found 540.0054.

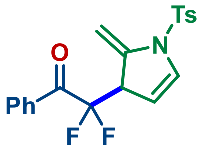

(58) According to the general procedure C, using N-triftosylhydrazone derived from 2,2,2-trifluoro-1-phenylethan-1-one (118.8 mg, 0.3 mmol), (1-tosyl-1H-pyrrol-2-yl)methanol (225.9 mg, 0.9 mmol), K_2CO_3 (82.9 mg, 0.6 mmol), Tp^{Br3}Ag (33.0 mg, 10 mol%) and DCM (4.0 mL) afforded compound **58** (107.4 mg, 92% yield) as a colorless oil; **^{1}H NMR** (500 MHz, DMSO) δ 7.88 (d, J = 7.5 Hz, 2H), 7.75 (t, J = 7.5 Hz, 1H), 7.70 (d, J = 8.0 Hz, 2H), 7.59-7.55 (m, 2H), 7.42 (d, J = 8.0 Hz, 2H), 6.99 (dd, J = 4.5, 1.5 Hz, 1H), 5.41 (s, 1H), 5.27-5.23 (m, 1H), 4.74 (s, 1H), 4.56-4.46 (m, 1H), 2.38 (s, 3H); **^{13}C NMR** (125 MHz, DMSO) δ 189.3 (t, J = 30.0 Hz), 145.7, 140.5, 136.0, 135.6, 133.7, 132.5, 130.8, 130.6, 130.1, 128.4, 117.3 (t, J = 255.0 Hz), 106.4, 99.4, 52.5 (t, J = 23.8 Hz), 22.0; **^{19}F NMR** (470 MHz, DMSO) δ -104.46 (dd, J = 275.4, 12.7 Hz), -105.35 (dd, J = 275.4, 16.5 Hz); **IR** (Film): 2984, 1736, 1447, 1372, 1234, 1043, 938, 917, 846, 811 cm^{-1}; **HRMS** (ESI$^+$) m/z calcd for $C_{20}H_{17}F_2NNaO_3S$ [M+Na]$^+$ 412.0789, found 412.0778.

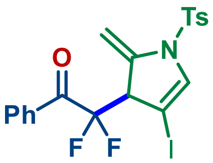

(59) According to the general procedure C, using N-triftosylhydrazone derived from 2,2,2-trifluoro-1-phenylethan-1-one (118.8 mg, 0.3 mmol), (4-iodo-1-tosyl-1H-pyrrol-2-yl)methanol (339.3 mg, 0.9 mmol), K_2CO_3 (82.9 mg, 0.6 mmol), Tp^{Br3}Ag (33.0 mg, 10 mol%) and DCM (4.0 mL) afforded compound **59** (131.3 mg, 85% yield) as a colorless oil; **^{1}H NMR** (500 MHz, DMSO) δ 7.85 (d, J = 7.5 Hz, 2H), 7.78-7.71 (m, 3H), 7.58 (t, J = 8.0 Hz, 2H), 7.43 (d, J = 8.0 Hz, 2H), 7.29 (s, 1H), 5.75 (s, 1H), 5.45 (s, 1H), 4.76 (s, 1H), 4.65 (t, J = 12.5 Hz, 1H), 2.40 (s, 3H); **^{13}C NMR** (150 MHz, DMSO) δ 188.6 (t, J = 30.0 Hz), 145.5, 140.0, 139.6, 135.4, 133.1, 132.6,

131.2, 130.4, 130.2, 129.6, 128.0, 116.7 (t, J = 258.0 Hz), 100.2, 56.8 (t, J = 24.0 Hz), 21.6; **^{19}F NMR** (564 MHz, DMSO) δ -102.17 (dd, J = 274.1, 12.4 Hz), -103.52 (dd, J = 274.1, 14.7 Hz).

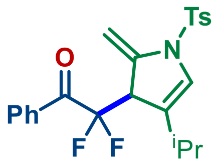

(60) According to the general procedure C, using N-triftosylhydrazone derived from 2,2,2-trifluoro-1-phenylethan-1-one (118.8 mg, 0.3 mmol), (4-isopropyl-1-tosyl-1H-pyrrol-2-yl)methanol (263.7 mg, 0.9 mmol), K_2CO_3 (82.9 mg, 0.6 mmol)**,** Tp^{Br3}Ag (33.0 mg, 10 mol%) and DCM (4.0 mL) afforded compound **60** (116.4 mg, 90% yield) as a colorless oil; **^{1}H NMR** (500 MHz, DMSO) δ 7.81 (d, J = 7.5 Hz, 2H), 7.75 (t, J = 7.5 Hz, 1H), 7.68 (d, J = 8.5 Hz, 2H), 7.56 (t, J = 7.5 Hz, 2H), 7.38 (d, J = 8.5 Hz, 2H), 6.57 (s, 1H), 5.32 (s, 1H), 4.60 (s, 1H), 4.55-4.41 (m, 1H), 2.37 (s, 3H), 2.25-2.18 (m, 1H), 1.08 (d, J = 6.5 Hz, 3H), 0.89 (d, J = 7.0 Hz, 3H); **^{13}C NMR** (125 MHz, DMSO) δ 189.8 (t, J = 28.8 Hz), 145.5, 141.3 (t, J = 3.8 Hz), 135.8, 133.6, 133.0, 130.6, 130.5, 130.0, 128.4, 127.5, 118.0 (t, J = 257.5 Hz), 99.9, 53.0 (t, J = 23.8 Hz), 27.1, 22.9, 22.0, 21.2; **^{19}F NMR** (470 MHz, DMSO) δ -100.30 (dd, J = 271.2, 12.2 Hz), -103.45 (dd, J = 271.2, 15.5 Hz); **IR** (Film): 2983, 1736, 1447, 1372, 1273, 1233, 1043, 938, 917, 846 cm^{-1};

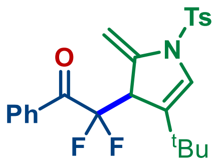

(61) According to the general procedure C, using N-triftosylhydrazone derived from 2,2,2-trifluoro-1-phenylethan-1-one (118.8 mg, 0.3 mmol), (4-(*tert*-butyl)-1-tosyl-1H-pyrrol-2-yl)methanol (274.5 mg, 0.9 mmol), K_2CO_3 (82.9 mg, 0.6 mmol)**,** Tp^{Br3}Ag (33.0 mg, 10 mol%) and DCM (4.0 mL) afforded compound **61** (121.5 mg, 91% yield) as a colorless oil; **^{1}H NMR** (500 MHz, DMSO) δ 7.80 (d, J = 8.0 Hz, 2H), 7.75 (t, J = 7.0 Hz, 1H), 7.67 (d, J = 8.5 Hz, 2H), 7.59-7.55 (m, 2H), 7.38 (d, J = 8.0 Hz, 2H), 6.77 (s, 1H), 5.21 (s, 1H), 4.45 (dd, J = 21.0, 5.5 Hz, 1H), 4.36 (s, 1H), 2.38 (s, 3H), 1.11 (s, 9H); **^{13}C NMR** (125 MHz, DMSO) δ 190.0 (t, J = 30.0 Hz), 145.6, 142.1 (dd, J = 8.8, 3.8 Hz), 135.7, 133.6, 133.3, 130.6, 130.5, 130.4, 130.1, 129.7, 128.4, 118.1 (t, J = 256.3 Hz), 99.5, 53.1 (t, J = 25.0 Hz), 32.4, 31.1, 22.0; **^{19}F NMR** (470 MHz, DMSO) δ -92.08 (dd, J = 271.7, 5.2 Hz), -104.34 (dd, J = 271.7, 20.7 Hz).

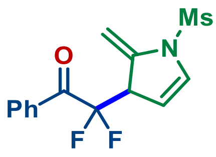

(62) According to the general procedure C, using N-triftosylhydrazone derived from 2,2,2-trifluoro-1-phenylethan-1-one (118.8 mg, 0.3 mmol), (1-(methylsulfonyl)-1H-pyrrol-2-yl)methanol (157.5 mg, 0.9 mmol), K_2CO_3 (82.9 mg, 0.6 mmol)**,** Tp^{Br3}Ag (33.0 mg, 10 mol%) and DCM (4.0 mL) afforded compound **62** (84.5 mg, 90% yield) as a colorless oil; **^{1}H NMR** (600 MHz, DMSO) δ 8.06 (d, J = 7.8 Hz, 2H), 7.78 (t, J = 7.2 Hz, 1H), 7.62 (t, J = 7.8 Hz, 2H), 6.74 (d, J = 4.2 Hz, 1H), 5.33 (s, 1H), 5.31-5.28 (m, 1H), 4.85 (s, 1H), 4.71 (dd, J = 14.4, 12.6 Hz, 1H), 3.11 (s, 3H). **^{13}C NMR** (150 MHz, DMSO) δ 189.1 (t, J = 30.0 Hz), 141.3, 136.1, 135.4, 132.4, 130.8, 130.2, 117.7 (t, J = 255.6 Hz), 105.7 (d, J = 4.5 Hz), 98.6, 52.7 (t, J = 24.0 Hz), 37.1; **^{19}F NMR** (564 MHz, DMSO) δ -103.48 (dd, J = 278.6, 11.8 Hz), -105.04 (dd, J = 278.6, 16.9 Hz).

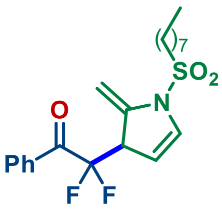

(63) According to the general procedure C, using *N*-triftosylhydrazone derived from 2,2,2-trifluoro-1-phenylethan-1-one (118.8 mg, 0.3 mmol), (1-(octylsulfonyl)-1*H*-pyrrol-2-yl)methanol (245.9 mg, 0.9 mmol), K_2CO_3 (82.9 mg, 0.6 mmol)**,** $Tp^{Br3}Ag$ (33.0 mg, 10 mol%) and DCM (4.0 mL) afforded compound **63** (107.3 mg, 87% yield) as a colorless oil; **^{1}H NMR** (500 MHz, DMSO) δ 8.05 (d, *J* = 7.5 Hz, 2H), 7.79 (t, *J* = 7.5 Hz, 1H), 7.63 (t, *J* = 8.0 Hz, 2H), 6.78 (dd, *J* = 4.5, 1.5 Hz, 1H), 5.27 (s, 1H), 5.24-5.21 (m, 1H), 4.80 (s, 1H), 4.74 (dd, *J* = 14.5, 12.0 Hz, 1H), 3.33-3.28 (m, 2H), 1.68-1.58 (m, 2H), 1.40-1.30 (m, 2H), 1.26-1.15 (m, 8H), 0.83 (t, *J* = 6.5 Hz, 3H); **^{13}C NMR** (150 MHz, $CDCl_3$) δ 189.2 (t, *J* = 30.0 Hz), 141.2, 136.2, 136.1, 135.9, 135.8, 132.4, 130.8, 130.24, 130.19, 130.1, 117.8 (t, *J* = 255.0 Hz), 104.2, 97.8, 52.8 (t, *J* = 22.5 Hz), 50.2, 32.0, 29.26, 29.24, 28.3, 23.5, 23.0, 14.9; **^{19}F NMR** (564 MHz, $CDCl_3$) δ -98.79 (dd, *J* = 288.1, 11.8 Hz), -100.36 (dd, *J* = 278.1, 17.5 Hz); **IR** (Film): 2983, 1736, 1447, 1372, 1273, 1223, 1043, 938, 846 cm^{-1}.

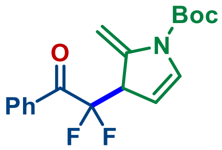

(64) According to the general procedure C, using *N*-triftosylhydrazone derived from 2,2,2-trifluoro-1-phenylethan-1-one (118.8 mg, 0.3 mmol), *tert*-butyl 2-(hydroxymethyl)-1*H*-pyrrole-1-carboxylate (177.5 mg, 0.9 mmol), K_2CO_3 (82.9 mg, 0.6 mmol)**,** $Tp^{Br3}Ag$ (33.0 mg, 10 mol%) and DCM (4.0 mL) afforded compound **64** (94.5 mg, 93% yield) as a colorless oil; **^{1}H NMR** (500 MHz, DMSO) δ 8.04 (d, *J* = 8.0 Hz, 2H), 7.78 (t, *J* = 7.0 Hz, 1H), 7.62 (t, *J* = 7.5 Hz, 2H), 6.93 (dd, *J* = 4.5, 1.5 Hz, 1H), 5.70 (s, 1H), 5.14 (dd, *J* = 4.5, 3.0 Hz, 1H), 4.77 (s, 1H), 4.59 (t, *J* = 14.0 Hz, 1H), 1.47 (s, 9H); **^{13}C NMR** (150 MHz, DMSO) δ 188.9 (t, *J* = 30.0 Hz), 149.8, 140.2, 135.5, 134.8, 132.2, 130.2, 129.7, 117.6 (t, *J* = 255.0 Hz), 102.9 (t, *J* = 3.0 Hz), 98.3, 82.3, 52.0 (t, *J* = 24.0 Hz), 28.2; **^{19}F NMR** (564 MHz, DMSO) δ -103.92 (dd, *J* = 274.1, 10.2 Hz), -105.28 (d, *J* = 274.1 Hz); **IR** (Film): 2983, 1736, 1447, 1372, 1233, 1043, 938, 846, 786 cm^{-1}.

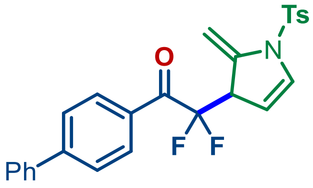

(65) According to the general procedure C, using *N*-triftosylhydrazone derived from 1-([1,1'-biphenyl]-4-yl)-2,2,2-trifluoroethan-1-one (141.6 mg, 0.3 mmol), (1-tosyl-1*H*-pyrrol-2-yl)methanol (225.9 mg, 0.9 mmol), K_2CO_3 (82.9 mg, 0.6 mmol)**,** $Tp^{Br3}Ag$ (33.0 mg, 10 mol%) and DCM (4.0 mL) afforded compound **65** (111.6 mg, 80% yield) as a colorless oil; **^{1}H NMR** (600 MHz, DMSO) δ 7.98 (d, *J* = 8.4 Hz, 2H), 7.88 (d, *J* = 8.4 Hz, 2H), 7.77 (d, *J* = 7.8 Hz, 2H), 7.71 (d, *J* = 8.4 Hz, 2H), 7.53 (t, *J* = 7.8 Hz, 2H), 7.47 (t, *J* = 7.8 Hz, 1H), 7.44 (d, *J* = 7.8 Hz, 2H), 7.00 (dd, *J* = 4.2, 1.5 Hz, 1H), 5.42 (s, 1H), 5.30-5.23 (m, 1H), 4.77 (s, 1H), 4.53 (dd, *J* = 14.4, 13.2 Hz, 1H), 2.39 (s, 3H); **^{13}C NMR** (150 MHz, DMSO) δ 188.2 (t, *J* = 30.0 Hz), 146.7, 145.3, 140.0, 138.8, 135.1, 133.2, 131.0, 130.7, 130.3, 129.7, 129.4, 127.9, 127.7, 127.6, 117.0 (t, *J* = 256.5 Hz), 106.0, 99.0, 52.0 (t, *J* = 24.0 Hz), 21.6; **^{19}F NMR** (564 MHz, DMSO) δ -104.44 (dd, *J* = 274.7, 12.4 Hz), -105.36 (dd, *J* = 274.7, 16.4 Hz).

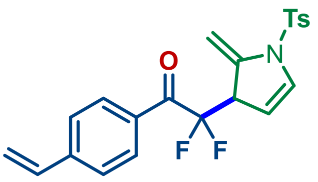

(66) According to the general procedure C, using *N*-triftosylhydrazone derived from 2,2,2-trifluoro-1-(4-vinylphenyl)ethan-1-one (126.6 mg, 0.3 mmol), (1-tosyl-1*H*-pyrrol-2-yl)methanol (225.9 mg, 0.9 mmol), K_2CO_3 (82.9 mg, 0.6 mmol)**,** $Tp^{Br3}Ag$ (33.0 mg, 10 mol%) and DCM (4.0 mL) afforded compound **66** (108.3 mg, 87% yield) as a colorless oil; **^{1}H NMR** (500 MHz, DMSO) δ 7.86 (d, *J* = 8.0 Hz, 2H), 7.70 (d, *J* = 8.5 Hz, 2H), 7.65 (d, *J* = 8.5 Hz, 2H), 7.42 (d, *J* = 8.0 Hz, 2H), 6.99 (d, *J* = 3.0 Hz, 1H), 6.83 (dd, *J* = 17.5, 11.0 Hz, 1H), 6.07 (d, *J* = 17.5 Hz, 1H), 5.50 (d, *J* = 11.5 Hz, 1H), 5.41 (s, 1H), 5.27-5.20 (m, 1H), 4.74 (s, 1H), 4.49 (dd, *J* = 15.0, 13.5 Hz, 1H), 2.38 (s, 3H); **^{13}C NMR** (125 MHz, DMSO) δ 188.5 (t, *J* = 30.0 Hz), 145.7, 144.2, 140.5, 136.4, 135.5, 133.7, 131.5, 131.2, 130.7, 128.4, 127.6, 119.8, 117.4 (t, *J* = 256.3 Hz), 106.4, 99.4, 52.5 (t, *J* = 23.8 Hz), 22.0; **^{19}F NMR** (470 MHz, DMSO) δ -104.37 (dd, *J* = 275.4, 12.7 Hz), -105.30 (dd, *J* = 275.4, 16.5 Hz).

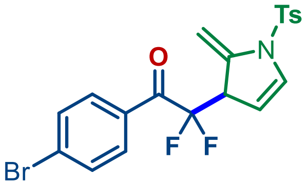

(67) According to the general procedure C, using *N*-triftosylhydrazone derived from 1-(4-bromophenyl)-2,2,2-trifluoroethan-1-one (142.2 mg, 0.3 mmol), (1-tosyl-1*H*-pyrrol-2-yl)methanol (225.9 mg, 0.9 mmol), K_2CO_3 (82.9 mg, 0.6 mmol)**,** $Tp^{Br3}Ag$ (33.0 mg, 10 mol%) and DCM (4.0 mL) afforded compound **67** (120.5 mg, 86% yield) as a colorless oil; **^{1}H NMR** (500 MHz, DMSO) δ 7.79 (s, 4H), 7.70 (d, *J* = 8.5 Hz, 1H), 7.42 (d, *J* = 8.0 Hz, 1H), 7.02-6.94 (m, 1H), 5.40 (s, 1H), 5.29-5.14 (m, 1H), 4.74 (s, 1H), 4.56-4.40 (m, 1H), 2.39 (s, 1H); **^{13}C NMR** (125 MHz, DMSO) δ 188.0 (t, *J* = 30.0 Hz), 145.3, 139.9, 135.1, 133.2, 132.8, 132.0, 131.0, 130.3, 130.1, 127.9, 116.8 (t, *J* = 265.0 Hz), 105.8, 99.0, 52.0 (t, *J* = 23.8 Hz), 21.5; **^{19}F NMR** (470 MHz, DMSO) δ -104.32 (dd, *J* = 275.4, 12.7 Hz), -105.64 (dd, *J* = 275.4, 16.9 Hz); **IR** (Film): 3061, 2983, 1736, 1447, 1372, 1234, 1043, 938, 846 cm^{-1}; **HRMS** (ESI^+) m/z calcd for $C_{20}H_{16}BrF_2NNaO_3S$ $[M+Na]^+$ 489.9895, found 489.9893.

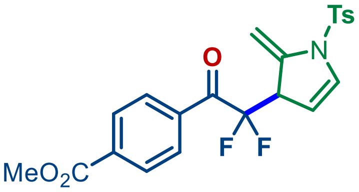

(68) According to the general procedure C, using *N*-triftosylhydrazone derived from methyl 4-(2,2,2-trifluoroacetyl)benzoate (172.5 mg, 0.3 mmol), (1-tosyl-1*H*-pyrrol-2-yl)methanol (225.9 mg, 0.9 mmol), K_2CO_3 (82.9 mg, 0.6 mmol)**,** $Tp^{Br3}Ag$ (33.0 mg, 10 mol%) and DCM (4.0 mL) afforded compound **68** (100.6 mg, 75% yield) as a colorless oil; **^{1}H NMR** (600 MHz, DMSO) δ 8.09 (d, *J* = 8.4 Hz, 2H), 7.99 (d, *J* = 8.4 Hz, 2H), 7.70 (d, *J* = 7.8 Hz, 2H), 7.41 (d, *J* = 7.8 Hz, 2H), 6.99 (d, *J* = 4.8 Hz, 1H), 5.41 (s, 1H), 5.28-5.22 (m, 1H), 4.75 (s, 1H), 4.53 (dd, *J* = 14.4, 12.6 Hz, 1H), 3.90 (s, 3H), 2.38 (s, 3H); **^{13}C NMR** (150 MHz, DMSO) δ 188.7 (t, *J* = 30.0 Hz), 165.7, 145.3, 139.8, 135.4, 135.2, 135.0, 133.2, 130.5, 130.3, 130.1, 127.9, 116.7 (t, *J* = 255.0 Hz), 105.7, 99.1, 53.2, 51.9 (t, *J* = 22.5 Hz), 21.5; **^{19}F NMR** (564 MHz, DMSO) δ -104.44 (dd, *J* = 276.4, 11.8 Hz), -105.74 (dd, *J* = 276.4, 16.9 Hz).

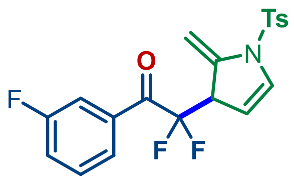

(69) According to the general procedure C, using *N*-triftosylhydrazone derived from 2,2,2-trifluoro-1-(3-fluorophenyl)ethan-1-one (124.2 mg, 0.3 mmol), (1-tosyl-1*H*-pyrrol-2-yl)methanol (225.9 mg, 0.9 mmol), K_2CO_3 (82.9 mg, 0.6 mmol), $Tp^{Br3}Ag$ (33.0 mg, 10 mol%) and DCM (4.0 mL) afforded compound **69** (87.9 mg, 72% yield) as a colorless oil; **^{1}H NMR** (500 MHz, DMSO) δ 7.73 (d, *J* = 4.5 Hz, 1H), 7.70 (d, *J* = 8.0 Hz, 2H), 7.65-7.61 (m, 2H), 7.58 (d, *J* = 9.5 Hz, 1H), 7.41 (d, *J* = 8.0 Hz, 2H), 6.99 (d, *J* = 4.5 Hz, 1H), 5.41 (s, 1H), 5.27-5.22 (m, 1H), 4.76 (s, 1H), 4.56-4.47 (m, 1H), 2.38 (s, 3H); **^{13}C NMR** (125 MHz, DMSO) δ 187.8 (td, *J* = 31.3, 2.5 Hz), 163.3, 161.4, 145.3, 139.8, 135.2, 133.2, 131.9 (d, *J* = 9 Hz), 130.3, 127.9, 126.5, 122.6 (d, *J* = 20.0 Hz), 116.7 (t, *J* = 255.0 Hz), 116.5 (d, *J* = 23.8 Hz), 105.8, 99.1, 51.9 (t, *J* = 23.8 Hz), 21.5; **^{19}F NMR** (470 MHz, DMSO) δ -104.45 (dd, *J* = 275.0, 12.2 Hz), -105.63 (dd, *J* = 275.0, 16.9 Hz), -111.14 (dd, *J* = 16.0, 8.0 Hz); **IR** (Film): 2983, 1735, 1447, 1372, 1234, 1043, 938, 846, 786 cm^{-1}.

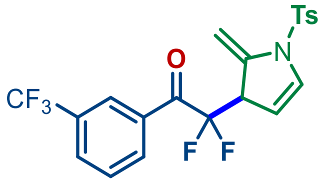

(70) According to the general procedure C, using *N*-triftosylhydrazone derived from 2,2,2-trifluoro-1-(3-(trifluoromethyl)phenyl)ethan-1-one (139.2 mg, 0.3 mmol), (1-tosyl-1*H*-pyrrol-2-yl)methanol (225.9 mg, 0.9 mmol), K_2CO_3 (82.9 mg, 0.6 mmol), $Tp^{Br3}Ag$ (33.0 mg, 10 mol%) and DCM (4.0 mL) afforded compound **70** (106.9 mg, 78% yield) as a colorless oil; **^{1}H NMR** (500 MHz, DMSO) δ 8.19-8.12 (m, 2H), 8.08 (s, 1H), 7.84 (t, *J* = 8.0 Hz, 1H), 7.70 (d, *J* = 8.0 Hz, 2H), 7.42 (d, *J* = 8.5 Hz, 2H), 6.99 (d, *J* = 4.5 Hz, 1H), 5.42 (s, 1H), 5.30-5.24 (m, 1H), 4.79 (s, 1H), 4.64-4.50 (m, 1H), 2.38 (s, 3H); **^{13}C NMR** (150 MHz, DMSO) δ 188.2 (t, *J* = 30.0 Hz), 145.7, 140.3, 135.7, 134.6, 133.7, 133.2, 132.2, 131.5, 130.7 (q, *J* = 31.5 Hz), 130.7, 128.4, 126.8, 124.5 (t, *J* = 271.5 Hz), 117.2 (t, *J* = 256.5 Hz), 106.3, 99.7, 52.3 (t, *J* = 22.5 Hz), 21.9; **^{19}F NMR** (564 MHz, DMSO) δ -61.70 (s), -103.96 (dd, *J* = 277.5, 11.3 Hz), -106.06 (dd, *J* = 277.5, 18.0 Hz).

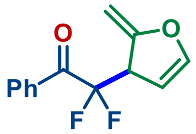

(71) According to the general procedure C, using *N*-triftosylhydrazone derived from 2,2,2-trifluoro-1-phenylethan-1-one (118.8 mg, 0.3 mmol), furan-2-ylmethanol (88.2 mg, 0.9 mmol), K_2CO_3 (82.9 mg, 0.6 mmol), $Tp^{Br3}Ag$ (33.0 mg, 10 mol%) and DCM (4.0 mL) afforded compound **71** (50.1 mg, 82% yield) as a colorless oil; **^{1}H NMR** (600 MHz, $CDCl_3$) δ 8.11 (d, *J* = 7.2 Hz, 2H), 7.64 (t, *J* = 7.2 Hz, 1H), 7.50 (t, *J* = 8.4 Hz, 2H), 6.59 (t, *J* = 2.5 Hz, 2H), 5.18 (t, *J* = 2.5 Hz, 2H), 4.84 (t, *J* = 2.5 Hz, 2H), 4.52 (t, *J* = 2.5 Hz, 2H), 4.50-4.42 (m, 1H); **^{13}C NMR** (125 MHz, $CDCl_3$) δ 188.7 (t, *J* = 31.3 Hz), 156.8 (t, *J* = 2.5 Hz), 147.5, 134.5, 132.1, 130.2 (t, *J* = 3.8 Hz), 128.7, 116.7 (t, *J* = 256.3 Hz), 99.5, 90.0, 48.7 (t, *J* = 25.0 Hz); **^{19}F NMR** (564 MHz, $CDCl_3$) δ -104.57 (dd, *J* = 288.8, 13.0 Hz), -105.59 (dd, *J* = 288.8, 16.4 Hz); **IR** (Film): 3065, 2983, 1736, 1447, 1372, 1233, 1043, 938, 846, 786 cm^{-1}; **HRMS** (ESI^+) m/z calcd for $C_{13}H_{10}F_2NaO_2$ $[M+Na]^+$ 259.0541, found 259.0535.

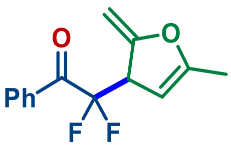

(72) According to the general procedure C, using *N*-triftosylhydrazone derived from 2,2,2-trifluoro-1-phenylethan-1-one (118.8 mg, 0.3 mmol), (5-methylfuran-2-yl)methanol (100.8 mg, 0.9 mmol), K_2CO_3 (82.9 mg, 0.6 mmol)**,** $Tp^{Br3}Ag$ (33.0 mg, 10 mol%) and DCM (4.0 mL) afforded compound **72** (56.3 mg, 75% yield) as a colorless oil; **^{1}H NMR** (500 MHz, $CDCl_3$) δ 8.10 (d, *J* = 7.5 Hz, 2H), 7.64 (t, *J* = 7.5 Hz, 1H), 7.50 (t, *J* = 8.0 Hz, 2H), 4.80-4.73 (m, 2H), 4.46-4.39 (m, 2H), 1.90 (t, *J* = 1.5 Hz, 3H); **^{13}C NMR** (125 MHz, $CDCl_3$) δ 189.0 (t, *J* = 31.3 Hz), 157.3, 134.4, 132.3, 130.2 (t, *J* = 3.8 Hz), 128.7, 116.9 (t, *J* = 256.3 Hz), 94.0 (t, *J* = 3.8 Hz), 89.0, 50.0 (t, *J* = 25.0 Hz), 13.4; **^{19}F NMR** (470 MHz, $CDCl_3$) δ -104.52 (dd, *J* = 288.1, 12.7 Hz), -105.61 (dd, *J* = 288.1, 15.5 Hz).

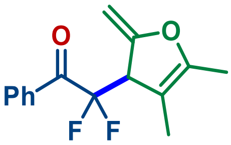

(73) According to the general procedure C, using *N*-triftosylhydrazone derived from 2,2,2-trifluoro-1-phenylethan-1-one (118.8 mg, 0.3 mmol), (4,5-dimethylfuran-2-yl)methanol (113.4 mg, 0.9 mmol), K_2CO_3 (82.9 mg, 0.6 mmol)**,** $Tp^{Br3}Ag$ (33.0 mg, 10 mol%) and DCM (4.0 mL) afforded compound **73** (54.6 mg, 69% yield) as a colorless oil; **^{1}H NMR** (500 MHz, $CDCl_3$) δ 8.07 (d, *J* = 7.5 Hz, 2H), 7.63 (t, *J* = 7.0 Hz, 1H), 7.49 (t, *J* = 7.5 Hz, 2H), 4.64 (s, 1H), 4.34-4.25 (m, 2H), 1.83 (s, 3H), 1.66 (s, 3H); **^{13}C NMR** (125 MHz, DMSO) δ 189.3 (t, *J* = 28.8 Hz), 156.6, 150.7, 135.4, 132.6, 130.1, 129.6, 118.2 (t, *J* = 256.3 Hz), 101.9, 88.9, 53.1 (t, *J* = 23.8 Hz), 11.2, 11.1; **^{19}F NMR** (470 MHz, DMSO) δ -100.75 (dd, *J* = 272.1, 11.8 Hz), -103.79 (dd, *J* = 272.1, 15.5 Hz); **IR** (Film): 3050, 2983, 1736, 1447, 1372, 1233, 1043, 938, 846, 786 cm^{-1},

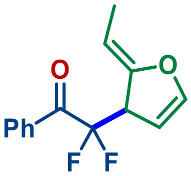

(74) According to the general procedure C, using *N*-triftosylhydrazone derived from 2,2,2-trifluoro-1-phenylethan-1-one (118.8 mg, 0.3 mmol), 1-(furan-2-yl)ethan-1-ol (100.8 mg, 0.9 mmol), K_2CO_3 (82.9 mg, 0.6 mmol)**,** $Tp^{Br3}Ag$ (33.0 mg, 10 mol%) and DCM (4.0 mL) afforded compound **74** (45.0 mg, 60% yield) as a colorless oil; **^{1}H NMR** (500 MHz, $CDCl_3$) δ 8.09 (d, *J* = 7.5 Hz, 2H), 7.64 (t, *J* = 7.5 Hz, 1H), 7.50 (t, *J* = 8.0 Hz, 2H), 6.62 (s, 1H), 5.14 (t, *J* = 2.5 Hz, 1H), 4.91-4.80 (m, 1H), 4.47-4.33 (m, 1H), 1.68 (dd, *J* = 7.0, 2.5 Hz, 3H); **^{13}C NMR** (125 MHz, $CDCl_3$) δ 189.2 (t, *J* = 30.0 Hz), 149.6, 147.5, 134.4, 132.4, 130.2 (t, *J* = 3.8 Hz), 128.7, 116.9 (t, *J* = 256.3 Hz), 101.3, 99.0 (t, *J* = 5.0 Hz), 48.5 (t, *J* = 25.0 Hz), 10.7; **^{19}F NMR** (470 MHz, $CDCl_3$) δ -104.66 (dd, *J* = 286.2, 12.7 Hz), -105.40 (dd, *J* = 286.2, 15.0 Hz).

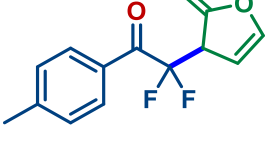

(75) According to the general procedure C, using *N*-triftosylhydrazone derived from 2,2,2-trifluoro-1-(p-tolyl)ethan-1-one (123.1 mg, 0.3 mmol), furan-2-ylmethanol (88.2 mg, 0.9 mmol), K_2CO_3 (82.9 mg, 0.6 mmol)**,**

$Tp^{Br3}Ag$ (33.0 mg, 10 mol%) and DCM (4.0 mL) afforded compound **75** (63.0 mg, 84% yield) as a colorless oil; **^{1}H NMR** (500 MHz, DMSO) δ 7.95 (d, J = 8.0 Hz, 1H), 7.41 (d, J = 8.2 Hz, 1H), 6.91 (s, 1H), 5.22 (s, 1H), 40.78 (s, 1H), 4.60 (t, J = 14.5 Hz, 1H), 4.41 (s, 1H), 3.36 (s, 1H), 2.40 (s, 1H); **^{13}C NMR** (150 MHz, DMSO) δ 188.2 (t, J = 30.0 Hz), 157.2, 148.5, 146.5, 130.4, 130.2, 129.5, 117.3 (t, J = 256.7 Hz), 99.9 (t, J = 4.0 Hz), 90.1, 49.0 (t, J = 24.7 Hz), 21.8; **^{19}F NMR** (564 MHz, DMSO) δ -104.30 (dd, J = 277.6, 13.6 Hz), -104.86 (dd, J = 277.6, 15.5 Hz); **HRMS** (ESI$^+$) m/z calcd for $C_{14}H_{12}F_2NaO_2$ [M+Na]$^+$ 273.0698, found 273.0698.

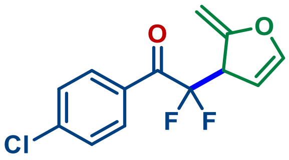

(76) According to the general procedure C, using *N*-triftosylhydrazone derived from 1-(4-chlorophenyl)-2,2,2-trifluoroethan-1-one (129 mg, 0.3 mmol), furan-2-ylmethanol (88.2 mg, 0.9 mmol), K_2CO_3 (82.9 mg, 0.6 mmol)**,** $Tp^{Br3}Ag$ (33.0 mg, 10 mol%) and DCM (4.0 mL) afforded compound **76** (62.4 mg, 77% yield) as a colorless oil; **^{1}H NMR** (500 MHz, $CDCl_3$) δ 8.05 (d, J = 8.3 Hz, 1H), 7.70 (d, J = 8.4 Hz, 1H), 6.92 (s, 1H), 5.24 (s, 1H), 4.79 (s, 1H), 4.62 (t, J = 14.7 Hz, 1H), 4.42 (s, 1H); **^{13}C NMR** (150 MHz, DMSO) δ 187.79 (t, J = 30.4 Hz), 156.98, 148.65, 140.70, 132.17, 130.64, 129.91, 117.15 (t, J = 256.5 Hz), 99.82, 90.31, 48.87 (t, J = 24.5 Hz); **^{19}F NMR** (564 MHz, DMSO) δ -104.32 (dd, J = 277.7, 12.4 Hz), -105.19 (dd, J = 277.7, 16.1 Hz); **IR** (Film): 2984, 1740, 1443, 1373, 1232, 1038, 939, 840, 787 cm^{-1}.

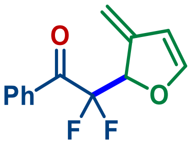

(77) According to the general procedure C, using *N*-triftosylhydrazone derived from 2,2,2-trifluoro-1-phenylethan-1-one (118.8 mg, 0.3 mmol), furan-3-ylmethanol (88.2 mg, 0.9 mmol), K_2CO_3 (82.9 mg, 0.6 mmol)**,** $Tp^{Br3}Ag$ (33.0 mg, 10 mol%) and DCM (4.0 mL) afforded compound **77** (54.5 mg, 77% yield) as a colorless oil; **^{1}H NMR** (500 MHz, DMSO) δ 8.03 (d, J = 7.8 Hz, 2H), 7.78 (t, J = 7.4 Hz, 1H), 7.62 (t, J = 7.7 Hz, 2H), 7.05 (s, 1H), 5.87-5.77 (m, 1H), 5.06 (s, 1H), 4.82 (s, 1H); **^{13}C NMR** (125 MHz, DMSO) δ 189.02-188.5 (t, J = 30.4 Hz), 154.6, 144.0, 135.5, 132.5, 130.2 (t, J = 3.4 Hz), 129.6, 115.8 (dd, J = 260.8, 256.1 Hz), 108.8, 103.0, 80.8 (dd, J = 29.1, 26.3 Hz); **^{19}F NMR** (470 MHz, DMSO) δ -111.26 (dd, J = 275.0, 8.1 Hz), -115.59 (dd, J = 275.0, 16.7 Hz); **IR** (Film): 3058, 2928, 2254, 1704, 1076, 903, 881, 717, 684, 649 cm^{-1}.

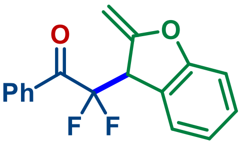

(78) According to the general procedure C, using *N*-triftosylhydrazone derived from 2,2,2-trifluoro-1-phenylethan-1-one (118.8 mg, 0.3 mmol), benzofuran-2-ylmethanol (133.2 mg, 0.9 mmol), K_2CO_3 (82.9 mg, 0.6 mmol)**,** $Tp^{Br3}Ag$ (33.0 mg, 10 mol%) and DCM (4.0 mL) afforded compound **78** (55.8 mg, 65% yield) as a colorless oil; **^{1}H NMR** (500 MHz, $CDCl_3$) δ 7.99 (d, J = 7.5 Hz, 2H), 7.54 (t, J = 7.5 Hz, 1H), 7.39 (t, J = 8.0 Hz, 2H), 7.34 (d, J = 7.5 Hz, 1H), 7.12 (td, J = 7.5, 1.5 Hz, 1H), 6.87 (t, J = 7.5 Hz, 1H), 6.75 (d, J = 8.0 Hz, 2H), 5.75-5.66 (m, 1H), 5.62 (s, 1H), 5.26 (s, 1H); **^{13}C NMR** (150 MHz, DMSO) δ 189.1 (dd, J = 28.5, 27.0 Hz), 162.2, 139.9, 136.0, 132.8, 131.9, 130.6, 130.0, 126.3, 122.8, 122.0, 116.5 (t, J = 255.0 Hz), 111.4, 107.5, 82.6 (dd, J = 28.5, 25.5 Hz); **^{19}F NMR** (470 MHz, $CDCl_3$) δ -109.96 (dd, J = 289.1, 6.1 Hz), -116.59 (dd, J = 289.1, 15.5 Hz); **IR** (Film): 3051, 2984, 2258, 1704, 1599, 1479, 1094, 911, 717, 650 cm^{-1}.

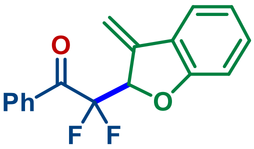

(79) According to the general procedure C, using *N*-triftosylhydrazone derived from 2,2,2-trifluoro-1-phenylethan-1-one (118.8 mg, 0.3 mmol), benzofuran-3-ylmethanol (133.2 mg, 0.9 mmol), K_2CO_3 (82.9 mg, 0.6 mmol)**,** $Tp^{Br3}Ag$ (33.0 mg, 10 mol%) and DCM (4.0 mL) afforded compound **79** (77.2 mg, 90% yield) as a colorless oil; **^{1}H NMR** (500 MHz, DMSO) δ 8.03 (d, *J* = 8.0 Hz, 2H), 7.77 (t, *J* = 7.0 Hz, 1H), 7.64-7.58 (m, 3H), 7.25 (t, *J* = 7.5 Hz, 1H), 6.99 (t, *J* = 7.5 Hz, 1H), 6.91 (d, *J* = 8.5 Hz, 1H), 6.09 (dd, *J* = 17.0, 7.0 Hz, 1H), 5.86 (s, 1H), 5.30 (s, 1H); **^{13}C NMR** (150 MHz, DMSO) δ 188.6 (dd, *J* = 28.5, 27.0 Hz), 161.7, 139.4, 135.6, 132.3, 131.4, 130.2, 129.7, 125.8, 122.4, 121.9, 116.0 (t, *J* = 255.0 Hz), 110.9, 107.1, 82.2 (dd, *J* = 28.5, 25.5 Hz); **^{19}F NMR** (564 MHz, DMSO) δ -109.83 (dd, *J* = 277.5, 6.8 Hz), -115.51 (dd, *J* = 277.5, 16.9 Hz); **IR** (Film): 3077, 2976, 1694, 1591, 1461, 1366, 1235, 1182, 1038, 935, 826, 731 cm^{-1}.

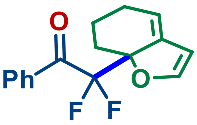

(80) According to the general procedure C, using *N*-triftosylhydrazone derived from 2,2,2-trifluoro-1-phenylethan-1-one (118.8 mg, 0.3 mmol), 3a,4,5,6,7,7a-hexahydrobenzofuran-4-ol (126 mg, 0.9 mmol), K_2CO_3 (82.9 mg, 0.6 mmol)**,** $Tp^{Br3}Ag$ (33.0 mg, 10 mol%) and DCM (4.0 mL) afforded compound **80** (36.4 mg, 44% yield) as a colorless oil; **^{1}H NMR** (500 MHz, $CDCl_3$) δ 8.10 (d, *J* = 7.5 Hz, 2H), 7.59 (t, *J* = 7.5 Hz, 1H), 7.45 (t, *J* = 8.0 Hz, 2H), 6.24 (d, *J* = 2.0 Hz, 1H), 5.73 (dd, *J* = 6.0, 3.5 Hz, 1H), 5.58 (d, *J* = 2.5 Hz, 1H), 2.77-2.68 (m, 1H), 2.39-2.25 (m, 2H), 2.03 (qd, *J* = 14.0, 8.4 Hz, 1H), 1.76-1.66 (m, 2H); **^{13}C NMR** (150 MHz, $CDCl_3$) δ 189.5 (dd, *J* = 28.5, 27.0 Hz), 148.7, 137.4 (d, *J* = 3.0 Hz), 134.0, 130.6 (t, *J* = 3.0 Hz), 128.2, 119.9, 118.1 (t, *J* = 262.5 Hz), 107.6, 85.4 (t, *J* = 27.0 Hz), 27.2 (d, *J* = 3.0 Hz), 22.8, 15.6; **^{19}F NMR** (470 MHz, $CDCl_3$) δ -106.92 (d, *J* = 254.7 Hz), -109.88 (d, *J* = 254.7 Hz).

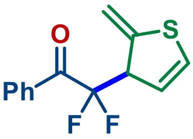

(81) According to the general procedure D, using *N*-triftosylhydrazone derived from 2,2,2-trifluoro-1-phenylethan-1-one (118.8 mg, 0.3 mmol), thiophen-2-ylmethanol (102.8 mmol, 0.9 mmol), and toluene (4.0 mL) afforded compound **81** (48.4 mg, 64% yield) as a colorless oil; **^{1}H NMR** (500 MHz, DMSO) δ 8.03 (d, *J* = 7.8 Hz, 2H), 7.78 (t, *J* = 7.4 Hz, 1H), 7.62 (t, *J* = 7.7 Hz, 2H), 7.05 (s, 1H), 5.87-5.77 (m, 1H), 5.06 (s, 1H), 4.82 (s, 1H); **^{13}C NMR** (125 MHz, DMSO) δ 189.02-188.5 (t, *J* = 30.4 Hz), 154.6, 144.0, 135.5, 132.5, 130.2 (t, *J* = 3.4 Hz), 129.6, 115.8 (dd, *J* = 260.8, 256.1 Hz), 108.8, 103.0, 80.8 (dd, *J* = 29.1, 26.3 Hz); **^{19}F NMR** (470 MHz, DMSO) δ -97.45 (d, *J* = 280.1), -108.06 (d, *J* = 280.1); **IR** (Film): 2929, 1701, 1379, 1097, 906, 724 cm^{-1}.

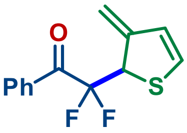

(82) According to the general procedure D, using *N*-triftosylhydrazone derived from 2,2,2-trifluoro-1-phenylethan-1-one (118.8 mg, 0.3 mmol), thiophen-3-ylmethanol **(**102.8 mmol, 0.9 mmol**)**, and toluene (4.0 mL)

afforded compound **82** (52.9 mg, 70% yield) as a colorless oil; **^{1}H NMR** (500 MHz, DMSO) δ 8.04 (d, *J* = 8.0 Hz, 2H), 7.78 (t, *J* = 7.5 Hz, 1H), 7.62 (t, *J* = 8.0 Hz, 2H), 6.73 (d, *J* = 6.0 Hz, 1H), 6.23 (d, *J* = 6.0 Hz, 1H), 5.41 (s, 1H), 5.23 (dd, *J* = 20.0, 10.0 Hz, 1H), 5.15 (s, 1H); **^{13}C NMR** (125 MHz, DMSO) δ 189.5 (t, *J* = 31.3 Hz), 147.9 (d, *J* = 2.5 Hz), 136.1, 133.7, 132.3, 130.7 (t, *J* = 2.8 Hz), 130.2, 127.4, 118.1 (t, *J* = 257.5 Hz), 113.4 (d, *J* = 1.3 Hz), 51.87 (t, *J* = 23.8 Hz); **^{19}F NMR** (470 MHz, DMSO) δ -103.26 (dd, *J* = 280.2, 10.3 Hz), -110.97 (dd, *J* = 280.2, 20.2 Hz); **IR** (Film): 3065, 2984, 1736, 1447, 1372, 1233, 1043, 938, 846, 785 cm^{-1}.

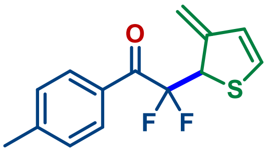

(83) According to the general procedure D, using *N*-triftosylhydrazone derived from 2,2,2-trifluoro-1-(p-tolyl)ethan-1-one (123.1 mg, 0.3 mmol), thiophen-3-ylmethanol (102.8 mmol, 0.9 mmol), and toluene (4.0 mL) afforded compound **83** (58.3 mg, 73% yield) as a colorless oil; **^{1}H NMR** (600 MHz, DMSO) δ 7.96 (d, *J* = 7.8 Hz, 2H), 7.43 (d, *J* = 7.8 Hz, 2H), 6.74 (d, *J* = 6.0 Hz, 1H), 6.23 (d, *J* = 6.0 Hz, 1H), 5.41 (s, 1H), 5.21 (dd, *J* = 20.4, 9.6 Hz, 1H), 5.14 (s, 1H), 2.42 (s, 3H); **^{13}C NMR** (150 MHz, DMSO) δ 188.8 (t, *J* = 31.5 Hz), 147.9, 147.1, 133.7, 130.9, 130.8, 129.8, 127.4, 118.2 (t, *J* = 258.0 Hz), 113.3, 51.9 (t, *J* = 24.0 Hz), 22.3; **^{19}F NMR** (564 MHz, DMSO) δ -103.27 (d, *J* = 279.2 Hz), -110.79 (dd, *J* = 279.2, 18.6 Hz); **IR** (Film): 3061, 2983, 1736, 1447, 1372, 1233, 1043,938, 846, 785 cm^{-1}.

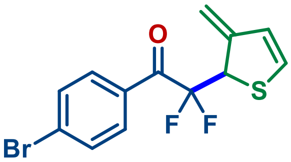

(84) According to the general procedure D, using *N*-triftosylhydrazone derived from 1-(4-bromophenyl)-2,2,2-trifluoroethan-1-one (142.2 mg, 0.3 mmol), **thiophen-3-ylmethanol (**102.8 mmol, 0.9 mmol**)**, and toluene (4.0 mL) afforded compound **84** (66.3 mg, 67% yield) as a colorless oil; **^{1}H NMR** (500 MHz, DMSO) δ 8.05 (d, *J* = 8.5 Hz, 2H), 7.69 (d, *J* = 8.5 Hz, 2H), 6.92 (s, 1H), 5.24 (s, 1H), 4.79 (s, 1H), 4.62 (t, *J* = 14.5 Hz, 1H), 4.42 (s, 1H); **^{13}C NMR** (150 MHz, DMSO) δ 187.8 (t, *J* = 30.0 Hz), 157.0, 148.6, 140.7, 132.2, 130.6, 129.9, 117.2 (t, *J* = 265.0 Hz), 98.8, 48.9 (t, *J* = 23.8 Hz); **^{19}F NMR** (470 MHz, DMSO) δ -100.97 (dd, *J* = 280.4, 13.6 Hz), -103.19 (dd, *J* = 280.4, 15.3 Hz).

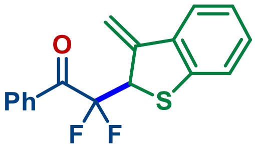

(85) According to the general procedure D, using *N*-triftosylhydrazone derived from 2,2,2-trifluoro-1-phenylethan-1-one (118.8 mg, 0.3 mmol), benzo[b]thiophen-3-ylmethanol (147.8 mmol, 0.9 mmol), and toluene (4.0 mL) afforded compound **85** (60.0 mg, 66% yield) as a colorless oil; **^{1}H NMR** (500 MHz, DMSO) δ 8.03 (d, *J* = 7.5 Hz, 2H), 7.77 (t, *J* = 7.5 Hz, 1H), 7.65-7.58 (m, 3H), 7.30 (d, *J* = 7.5 Hz, 1H), 7.26-7.21 (m, 1H), 7.16-7.11 (m, 1H), 6.04 (d, *J* = 1.2 Hz, 1H), 5.44-5.35 (m, 2H); **^{13}C NMR** (125 MHz, DMSO) δ 189.5 (t, *J* = 31.3 Hz), 143.2, 141.9, 137.0, 136.1, 132.4, 130.8, 130.7, 130.2, 126.1, 123.4, 123.0, 118.2 (t, *J* = 258.8 Hz), 114.3, 52.9 (t, *J* = 23.8 Hz); **^{19}F NMR** (470 MHz, DMSO) δ -101.38 (dd, *J* = 278.2, 9.4 Hz), -111.57 (dd, *J* = 278.2, 20.2 Hz); **^{19}F NMR** (564 MHz, DMSO) δ -103.27 (d, *J* = 279.8 Hz), -110.79 (dd, *J* = 279.8, 18.4 Hz); **IR**

(Film): 3059, 2982, 2250, 1732, 1447, 1373, 1238, 1042, 938, 820, 758 cm^{-1}; **HRMS** (ESI^{+}) m/z calcd for $C_{17}H_{12}F_2NaOS$ [M+Na]$^{+}$ 325.0469, found 325.0468.

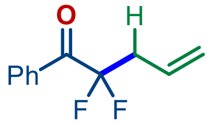

(86) According to the general procedure E, using phenyl trifluoromethyl ketone *N*-triftosylhydrazone (118.8 mg, 0.3 mmol), allyl alcohol (34.8 mg, 0.6 mmol), and DCE (4.0 mL) afforded compound **86** (51.7 mg, 88% yield) as a colorless oil. **^{1}H-NMR** (600 MHz, $CDCl_3$) δ 8.14-8.07 (m, 2H), 7.68-7.59 (m, 1H), 7.54-7.46 (m, 2H), 5.90-5.80 (m, 1H), 5.32-5.23 (m, 2H), 2.98 (td, *J* = 17.4, 6.6 Hz, 2H). **^{13}C-NMR** (125 MHz, $CDCl_3$) δ 189.1 (t, *J* = 31.3 Hz), 134.3, 132.0 (t, *J* = 2.5 Hz), 130.1 (t, *J* = 2.5 Hz), 128.7, 127.6 (t, *J* = 5.0 Hz), 121.5, 118.6 (t, *J* = 252.5 Hz), 38.6 (t, *J* = 23.8 Hz). **^{19}F-NMR** (470 MHz, $CDCl_3$) δ -99.38 (t, *J* = 17.4 Hz). **IR** (Film): 3059, 2985, 1648, 1345, 1267, 908, 722, 649 cm^{-1}. **HRMS** (ESI) m/z calcd. for $C_{11}H_{11}F_2O^{+}$ [M+H]$^{+}$: 197.0772; Found: 197.0772.

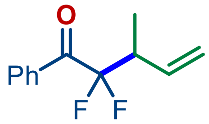

(87) According to the general procedure E, using phenyl trifluoromethyl ketone *N*-triftosylhydrazone (118.8 mg, 0.3 mmol), 2-buten-1-ol (43.2 mg, 0.6 mmol), and DCE (4.0 mL) afforded compound **87** (62.4 mg, 99% yield) as a colorless oil; **^{1}H-NMR** (500 MHz, $CDCl_3$) δ 8.08-8.04 (m, 2H), 7.65-7.60 (m, 1H), 7.53-7.46 (m, 2H), 5.86-5.77 (m, 1H), 5.22-5.10 (m, 2H), 3.24-3.07 (m, 1H), 1.23 (d, *J* = 7.0 Hz, 3H). **^{13}C-NMR** (125 MHz, $CDCl_3$) δ 189.8 (t, *J* = 30.0 Hz), 134.2 (t, *J* = 5.0 Hz), 134.1, 132.8 (t, *J* = 2.5 Hz), 130.0 (t, *J* = 3.8 Hz), 128.6, 119.5 (t, *J* = 255.0 Hz), 118.9, 42.4 (t, *J* = 22.5 Hz), 12.8 (t, *J* = 5.0 Hz). **^{19}F-NMR** (470 MHz, $CDCl_3$) δ -106.10 (dd, *J* = 274.5, 14.0 Hz, 1F), -107.66 (dd, *J* = 274.5, 16.5 Hz, 1F). **IR** (Film): 3090, 2966, 1688, 1603, 1257, 1095, 1061, 740, 720, 700 cm^{-1}. **HRMS** (ESI) m/z calcd. for $C_{12}H_{13}F_2O^{+}$ [M+H]$^{+}$: 211.0929; Found: 211.0928.

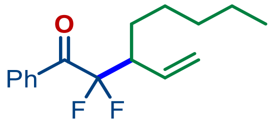

(88) According to the general procedure E, using phenyl trifluoromethyl ketone *N*-triftosylhydrazone (118.8 mg, 0.3 mmol), oct-2-en-1-ol (76.8 mg, 0.6 mmol), and DCE (4.0 mL) afforded compound **88** (76.6 mg, 96% yield) as a colorless oil; **^{1}H-NMR** (500 MHz, $CDCl_3$) δ 8.05 (d, *J* = 8.0 Hz, 2H), 7.66-7.58 (m, 1H), 7.52-7.45 (m, 2H), 5.64 (dt, *J* = 17.0, 10.0 Hz, 1H), 5.23 (d, *J* = 10.0 Hz, 1H), 5.11 (d, *J* = 17.0 Hz, 1H), 3.00-2.85 (m, 1H), 1.76-1.65 (m, 1H), 1.54-1.34 (m, 2H), 1.34-1.15 (m, 5H), 0.87 (t, *J* = 5.0 Hz, 3H). **^{13}C-NMR** (150 MHz, $CDCl_3$) δ 190.0 (t, *J* = 30.0 Hz), 134.0, 133.0 (dd, *J* = 9.0, 4.5 Hz), 129.9 (t, *J* = 3.0 Hz), 128.6, 120.7, 119.5 (t, *J* = 255.0 Hz), 48.8 (t, *J* = 21.0 Hz), 31.4, 26.6 (t, *J* = 3.0 Hz), 26.4, 22.4, 14.0. **^{19}F-NMR** (564 MHz, $CDCl_3$) δ -105.14 (dd, *J* = 271.8, 13.5 Hz), -106.19 (dd, *J* = 271.8, 16.4 Hz). **IR** (Film): 3055, 2931, 1703, 1598, 1449, 1259, 904, 723 cm^{-1}. **HRMS** (ESI) m/z calcd. for $C_{16}H_{21}F_2O^{+}$ [M+H]$^{+}$: 266.1483; Found: 266.1482.

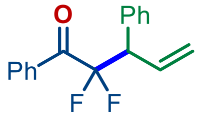

(89) According to the general procedure E, using phenyl trifluoromethyl ketone *N*-triftosylhydrazone (118.8 mg, 0.3 mmol), 3-phenylprop-2-en-1-ol (80.4 mg, 0.6 mmol), and DCE (4.0 mL) afforded compound **89** (71.8 mg, 88% yield) as a colorless oil; **^{1}H-NMR** (500 MHz, $CDCl_3$) δ 8.00-7.94 (m, 2H), 7.63-7.57 (m, 1H), 7.49-7.43 (m,

2H), 7.37-7.27 (m, 5H), 6.30-6.18 (m, 1H), 5.32 (d, J = 10.0 Hz, 1H), 5.24 (d, J = 17.0 Hz, 1H), 4.34 (td, J = 16.5, 8.0 Hz, 1H). **^{13}C-NMR** (150 MHz, $CDCl_3$) δ 189.8 (t, J = 30.0 Hz), 135.1, 134.0, 132.9, 132.4 (t, J = 4.5 Hz), 129.8 (t, J = 4.5 Hz), 129.6, 128.6, 128.5, 127.8, 120.5, 118.6 (t, J = 256.5 Hz), 53.9 (t, J = 21.0 Hz). **^{19}F-NMR** (564 MHz, $CDCl_3$) δ -102.89 (dd, J = 274.1, 17.0 Hz), -103.57 (dd, J = 274.1, 16.5 Hz). **IR** (Film): 3065, 2930, 1702, 1597, 1448, 1171, 1047, 732 cm^{-1}.

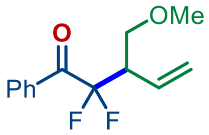

(90) According to the general procedure E, using phenyl trifluoromethyl ketone *N*-triftosylhydrazone (118.8 mg, 0.3 mmol), 4-methoxybut-2-en-1-ol (61.2 mg, 0.6 mmol), and DCE (4.0 mL) afforded compound **90** (65.5 mg, 91% yield) as a colorless oil; **^{1}H-NMR** (500 MHz, $CDCl_3$) δ 8.07-7.98 (m, 2H), 7.64-7.56 (m, 1H), 7.52-7.44 (m, 2H), 5.85-5.75 (m, 1H), 5.36-5.26 (m, 2H), 3.65 (dd, J = 9.5, 7.0 Hz, 1H), 3.57-3.51 (m, 1H), 3.47-3.34 (m, 1H), 3.21 (s, 3H). **^{13}C-NMR** (150 MHz, $CDCl_3$) δ 189.4 (t, J = 28.5 Hz), 133.8, 133.0, 130.1 (t, J = 3.0 Hz), 129.7 (t, J = 3.0 Hz), 128.5, 121.6, 118.7 (dd, J = 258.0, 253.5 Hz), 70.3 (t, J = 4.5 Hz), 58.7, 49.0 (t, J = 7.5 Hz). **^{19}F-NMR** (564 MHz, $CDCl_3$) δ -102.94 (dd, J = 273.0, 13.0 Hz), -108.75 (dd, J = 273.0, 18.0 Hz).

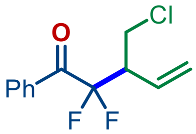

(91) According to the general procedure E, using phenyl trifluoromethyl ketone *N*-triftosylhydrazone (118.8 mg, 0.3 mmol), 4-chlorobut-2-en-1-ol (63.6 mg, 0.6 mmol), and DCE (4.0 mL) afforded compound **91** (45.5 mg, 62% yield) as a colorless oil; **^{1}H-NMR** (500 MHz, $CDCl_3$) δ 8.10-8.00 (m, 2H), 7.69-7.61 (m, 1H), 7.55-7.46 (m, 2H), 5.78-5.66 (m, 1H), 5.38 (d, J = 10.5 Hz, 1H), 5.30 (d, J = 17.0 Hz, 1H), 3.92 (dd, J = 11.0, 3.5 Hz, 1H), 3.66 (dd, J = 11.0, 9.0 Hz, 1H), 3.46-3.32 (m, 1H). **^{13}C-NMR** (150 MHz, $CDCl_3$) δ 188.6 (t, J = 30.0 Hz), 134.5, 132.2 (t, J = 3.0 Hz), 130.0 (t, J = 3.0 Hz), 129.8 (t, J = 3.0 Hz), 128.8, 123.2, 118.2 (t, J = 258.0 Hz), 51.1 (t, J = 21.0 Hz), 41.0 (t, J = 6.0 Hz). **^{19}F-NMR** (564 MHz, $CDCl_3$) δ -102.26 (dd, J = 283.0, 11.8 Hz), -105.31 (dd, J = 283.0, 17.5 Hz). **IR** (Film): 3059, 2926, 1702, 1449, 1264, 733, 704 cm^{-1}. **HRMS** (ESI) m/z calcd. for $C_{12}H_{12}ClF_2O^+$ $[M+H]^+$: 245.0539; Found: 245.0536.

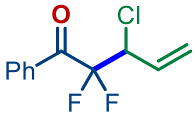

(92) According to the general procedure E, using phenyl trifluoromethyl ketone *N*-triftosylhydrazone (118.8 mg, 0.3 mmol), 3-chloroprop-2-en-1-ol (55.2 mg, 0.6 mmol), and DCE (4.0 mL) afforded compound **92** (42.8 mg, 62% yield) as a colorless oil; **^{1}H-NMR** (500 MHz, $CDCl_3$) δ 8.14-8.05 (m, 2H), 7.71-7.63 (m, 1H), 7.59-7.47 (m, 2H), 6.07-5.96 (m, 1H), 5.60-5.44 (m, 2H), 5.10-4.98 (dt, J = 13.5, 9.3 Hz, 1H). **^{13}C-NMR** (125 MHz, $CDCl_3$) δ 188.3 (dd, J = 30.0, 28.8 Hz), 134.5, 132.4 (t, J = 2.5 Hz), 130.0 (t, J = 3.5 Hz), 129.4 (t, J = 2.5 Hz), 128.8, 123.0, 115.5 (dd, J = 262.5, 257.5 Hz), 59.1 (dd, J = 28.8, 25.0 Hz). **^{19}F-NMR** (470 MHz, $CDCl_3$) δ -102.88 (dd, J = 278.0, 9.3 Hz, 1F), -109.11 (dd, J = 278.0, 13.5 Hz, 1F).

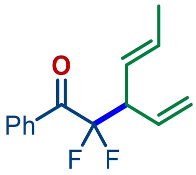

(93) According to the general procedure E, using phenyl trifluoromethyl ketone *N*-triftosylhydrazone (118.8 mg, 0.3 mmol), hexa-2,4-dien-1-ol (58.8 mg, 0.6 mmol), and DCE (4.0 mL) afforded compound **93** (69.9 mg, 92% yield) as a colorless oil; **^{1}H-NMR** (500 MHz, $CDCl_3$) δ 8.07-8.01 (m, 2H), 7.65-7.59 (m, 1H), 7.52-7.45 (m, 2H), 5.94-5.84 (m, 1H), 5.66-5.56 (m, 1H), 5.53-5.44 (m, 1H), 5.27-5.15 (m, 2H), 3.73-3.60 (m, 1H), 1.68 (d, *J* = 6.0 Hz, 3H). **^{13}C-NMR** (150 MHz, $CDCl_3$) δ 189.8 (t, *J* = 29.8 Hz), 134.0, 133.0, 132.0 (t, *J* = 4.3 Hz), 131.6, 129.9 (t, *J* = 3.6 Hz), 128.6, 123.8 (t, *J* = 4.3 Hz), 119.7, 118.6 (t, *J* = 256.5 Hz), 51.7 (t, *J* = 22.0 Hz), 18.1. **^{19}F-NMR** (564 MHz, $CDCl_3$) δ -104.32 (dd, *J* = 272.3, 14.8 Hz), -105.41 (dd, *J* = 272.0, 16.1 Hz). **HRMS** (ESI) m/z calcd. for $C_{14}H_{15}F_2O^+$ $[M+H]^+$: 237.1085; Found: 237.1082.

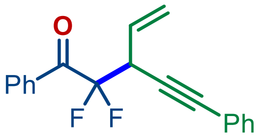

(94) According to the general procedure E, using phenyl trifluoromethyl ketone *N*-triftosylhydrazone (118.8 mg, 0.3 mmol), 5-phenylpent-2-en-4-yn-1-ol (94.8 mg, 0.6 mmol), and DCE (4.0 mL) afforded compound **94** (68.4 mg, 77% yield) as a colorless oil; **^{1}H-NMR** (500 MHz, $CDCl_3$) δ 8.13 (t, *J* = 7.0 Hz, 2H), 7.65-7.62 (m, 1H), 7.51-7.48 (m, 2H), 7.36-7.34 (m, 2H), 7.30-7.25 (m, 3H), 6.05-5.98 (m, 1H), 5.65 (d, *J* = 17.5 Hz 1H), 5.46 (d, *J* = 10.0 Hz, 1H), 4.38-4.31 (m, 1H) **^{13}C-NMR** (150 MHz, $CDCl_3$) δ 189.0 (t, *J* = 30.0 Hz), 134.3, 132.7, 131.7, 130.2 (t, *J* = 3.0 Hz), 128.7, 128.5 (t, *J* = 4.5 Hz), 128.2, 122.4, 121.4, 117.1 (t, *J* = 259.5 Hz), 87.0, 81.7 (t, *J* = 4.5 Hz), 41.8 (dd, *J* = 27.0, 24.0 Hz). **^{19}F-NMR** (564 MHz, $CDCl_3$) δ -102.15 (dd, *J* = 273.0, 11.3 Hz, 1F), -104.89 (dd, *J* = 273.0, 17.5 Hz). **IR** (Film): 3063, 2989, 1700, 1598, 1264, 1059, 736, 688 cm^{-1}. **HRMS** (ESI) m/z calcd. for $C_{19}H_{15}F_2O^+$ $[M+H]^+$: 297.1085; Found: 297.1085.

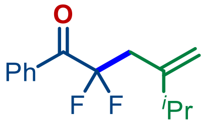

(95) According to the general procedure E, using phenyl trifluoromethyl ketone *N*-triftosylhydrazone (118.8 mg, 0.3 mmol), 3-methyl-2-methylenebutan-1-ol (60 mg, 0.6 mmol), and DCE (4.0 mL) afforded compound **95** (67.8 mg, 95% yield) as a colorless oil; **^{1}H-NMR** (600 MHz, $CDCl_3$) δ 8.03-7.99 (m, 2H), 7.57-7.52 (m, 1H), 7.44-7.40 (m, 2H), 4.95 (s, 1H), 4.85 (s, 1H), 2.89 (t, *J* = 18.0 Hz, 2H), 2.30-2.22 (m, 1H), 0.96 (d, *J* = 6.8 Hz, 6H). **^{13}C-NMR** (125 MHz, $CDCl_3$) δ 189.6 (t, *J* = 31.3 Hz), 146.0 (t, *J* = 2.5 Hz), 134.1, 130.1 (t, *J* = 3.8 Hz), 128.6, 121.9, 119.0 (t, *J* = 253.8 Hz), 113.6, 38.8 (t, *J* = 22.5 Hz), 34.0, 21.5. **^{19}F-NMR** (470 MHz, $CDCl_3$) δ -98.13 (t, *J* = 18.0 Hz). **HRMS** (ESI) m/z calcd. for $C_{14}H_{17}F_2O^+$ $[M+H]^+$: 239.1242; Found: 239.1242.

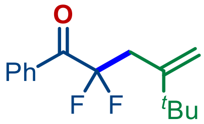

(96) According to the general procedure E, using phenyl trifluoromethyl ketone *N*-triftosylhydrazone (118.8 mg, 0.3 mmol), 3,3-dimethyl-2-methylenebutan-1-ol (68.4 mg, 0.6 mmol), and DCE (4.0 mL) afforded compound **96** (69.6 mg, 92% yield) as a colorless oil; **^{1}H-NMR** (500 MHz, $CDCl_3$) δ 8.13-8.07 (m, 2H), 7.65-7.60 (m, 1H),

7.52-7.46 (m, 2H), 5.12 (s, 1H), 5.00 (s, 1H), 3.00 (t, *J* = 18.0 Hz, 2H), 1.06 (s, 9H). **^{13}C-NMR** (125 MHz, $CDCl_3$) δ 189.7 (t, *J* = 30.0 Hz), 147.6, 134.1, 132.3, 130.1 (t, *J* = 3.8 Hz), 128.6, 119.1 (t, *J* = 252.5 Hz), 112.1, 36.3, 34.8 (t, *J* = 22.5 Hz), 28.8. **^{19}F-NMR** (470 MHz, $CDCl_3$) δ -97.09 (t, *J* = 18.0 Hz). **IR** (Film): 3061, 2967, 1702, 1449, 1264, 1173, 734, 704 cm^{-1}. **HRMS** (ESI) m/z calcd. for $C_{15}H_{19}F_2O^+$ $[M+H]^+$: 253.1398; Found: 253.1399.

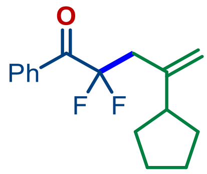

(97) According to the general procedure E, using phenyl trifluoromethyl ketone *N*-triftosylhydrazone (118.8 mg, 0.3 mmol), 2-cyclopentylprop-2-en-1-ol (75.6 mg, 0.6 mmol), and DCE (4.0 mL) afforded compound **97** (71.3 mg, 90% yield) as a colorless oil; **^{1}H-NMR** (500 MHz, $CDCl_3$) δ 8.16-8.06 (m, 2H), 7.66-7.60 (m, 1H), 7.53-7.46 (m, 2H), 5.04 (s, 1H), 4.91 (s, 1H), 2.98 (t, *J* = 18.0 Hz, 2H), 2.55-2.46 (m, 1H), 1.90-1.81 (m, 2H), 1.75-1.64 (m, 2H), 1.63-1.52 (m, 2H), 1.42-1.29 (m, 2H). **^{13}C-NMR** (125 MHz, $CDCl_3$) δ 189.6 (t, *J* = 30.0 Hz), 143.4 (t, *J* = 2.5 Hz), 134.1, 132.3, 130.1 (t, *J* = 3.8 Hz), 128.6, 119.0 (t, *J* = 252.5 Hz), 113.8, 46.4, 39.8 (t, *J* = 22.5 Hz), 31.4, 24.7. **^{19}F-NMR** (470 MHz, $CDCl_3$) δ -98.11 (t, *J* = 18.0 Hz). **IR** (Film): 3295, 2953, 2869, 1702, 1449, 1173, 1044, 905, 737 cm^{-1}. **HRMS** (ESI) m/z calcd. for $C_{16}H_{19}F_2O^+$ $[M+H]^+$: 265.1398; Found: 265.1395.

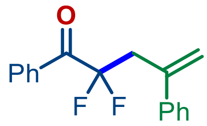

(98) According to the general procedure E, using phenyl trifluoromethyl ketone *N*-triftosylhydrazone (118.8 mg, 0.3 mmol), 2-phenylprop-2-en-1-ol (80.4 mg, 0.6 mmol), and DCE (4.0 mL) afforded compound **98** (66.1 mg, 81% yield) as a colorless oil; **^{1}H-NMR** (500 MHz, $CDCl_3$) δ 8.04-7.99 (m, 2H), 7.62-7.56 (m, 1H), 7.46-7.40 (m, 2H), 7.39-7.34 (m, 2H), 7.32-7.23 (m, 3H), 5.53 (s, 1H), 5.31 (s, 1H), 3.51-3.37 (t, *J* = 17.5 Hz, 2H). **^{13}C-NMR** (150 MHz, $CDCl_3$) δ 189.4 (t, *J* = 31.5 Hz), 140.8, 139.1 (t, *J* = 4.5 Hz), 134.2, 132.2 (t, *J* = 3.0 Hz), 130.1 (t, *J* = 3.0 Hz), 128.6, 128.3, 127.7, 126.3, 119.3, 118.6 (t, *J* = 253.5 Hz), 39.3 (t, *J* = 24.0 Hz). **^{19}F-NMR** (564 MHz, $CDCl_3$) δ -98.01 (t, *J* = 17.5 Hz). **IR** (Film): 3057, 2987, 1700, 1597, 1264, 1038, 733, 701 cm^{-1}. **HRMS** (ESI) m/z calcd. for $C_{17}H_{15}F_2O^+$ $[M+H]^+$: 273.1085; Found: 273.1083.

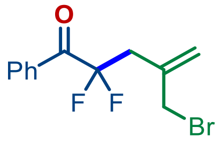

(99) According to the general procedure E, using phenyl trifluoromethyl ketone *N*-triftosylhydrazone (118.8 mg, 0.3 mmol), 2-(bromomethyl)prop-2-en-1-ol (89.4 mg, 0.6 mmol), and DCE (4.0 mL) afforded compound **99** (73.7 mg, 85% yield) as a colorless oil; **^{1}H-NMR** (500 MHz, $CDCl_3$) δ 8.14-8.07 (m, 2H), 7.68-7.62 (m, 1H), 7.54-7.48 (m, 2H), 5.46 (s, 1H), 5.20 (s, 1H), 4.11 (s, 2H), 3.25 (t, *J* = 18.0 Hz, 2H). **^{13}C-NMR** (125 MHz, $CDCl_3$) δ 188.7 (t, *J* = 31.3 Hz), 136.4 (t, *J* = 3.8 Hz), 134.4, 131.8 (t, *J* = 3.0 Hz), 130.1 (t, *J* = 3.8 Hz), 128.7, 122.1, 118.6 (t, *J* = 253.8 Hz), 37.2 (t, *J* = 22.5 Hz), 37.0. **^{19}F-NMR** (470 MHz, $CDCl_3$) δ -98.41 (t, *J* = 18.0 Hz). **IR** (Film): 3064, 2925, 1701, 1597, 1271, 1171, 1093, 901 cm^{-1}. **HRMS** (ESI) m/z calcd. for $C_{12}H_{12}BrF_2O^+$ $[M+H]^+$: 289.0034; Found: 289.0031.

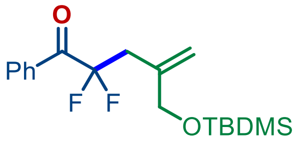

(100) According to the general procedure E, using phenyl trifluoromethyl ketone *N*-triftosylhydrazone (118.8 mg, 0.3 mmol), 2-(((*tert*-butyldimethylsilyl)oxy)methyl)prop-2-en-1-ol (121.2 mg, 0.6 mmol), and DCE (4.0 mL) afforded compound **100** (95.9 mg, 94% yield) as a colorless oil; **^{1}H-NMR** (600 MHz, $CDCl_3$) δ 8.15-8.03 (m, 2H), 7.66-7.60 (m, 1H), 7.53-7.44 (m, 2H), 5.34 (s, 1H), 5.07 (s, 1H), 4.16 (s, 2H), 2.96 (t, *J* = 18.0 Hz, 2H), 0.91 (s, 9H), 0.07 (s, 6H). **^{13}C-NMR** (150 MHz, $CDCl_3$) δ 189.2 (t, *J* = 30.0 Hz), 139.1 (t, *J* = 3.0 Hz), 134.2, 132.1, 130.1(t, *J* = 3.0 Hz), 128.7, 118.7 (t, *J* = 252.0 Hz), 115.8, 109.0, 66.0, 63.9, 36.8 (t, *J* = 22.5 Hz), 25.9, 18.3, -5.5. **^{19}F-NMR** (564 MHz, $CDCl_3$) δ -98.18 (t, *J* = 18.0 Hz). **IR** (Film): 3069, 2929, 1703, 1252, 1077, 834, 775 cm^{-1}. **HRMS** (ESI) m/z calcd. for $C_{18}H_{27}F_2O_2Si^+$ $[M+H]^+$: 341.1742; Found: 341.1738.

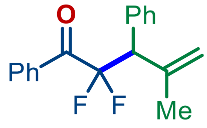

(101) According to the general procedure E, using phenyl trifluoromethyl ketone *N*-triftosylhydrazone (118.8 mg, 0.3 mmol), 2-methyl-3-phenylprop-2-en-1-ol (88.8 mg, 0.6 mmol), and DCE (4.0 mL) afforded compound **101** (78.1 mg, 91% yield) as a colorless oil; **^{1}H-NMR** (500 MHz, $CDCl_3$) δ 8.03-7.96 (m, 2H), 7.65-7.57 (m, 1H), 7.50-7.44 (m, 2H), 7.42-7.37 (m, 2H), 7.37-7.27 (m, 3H), 5.07 (s, 1H), 5.04 (s, 1H), 4.36-4.26 (m, 1H), 1.74 (s, 3H). **^{13}C-NMR** (150 MHz, $CDCl_3$) δ 190.0 (t, *J* = 30.0 Hz), 140.6, 134.3, 133.9, 133.0, 130.0, 129.8 (t, *J* = 4.5 Hz), 128.6, 128.4, 127.8, 118.9 (t, *J* = 257.3 Hz), 115.3, 55.5 (t, *J* = 21.0 Hz), 23.2. **^{19}F-NMR** (564 MHz, $CDCl_3$) δ -97.20 (dd, *J* = 282.0, 14.5 Hz), -104.14 (dd, *J* = 282.0, 21.5 Hz). **HRMS** (ESI) m/z calcd. for $C_{18}H_{17}F_2O^+$ $[M+H]^+$: 288.1242; Found: 288.1239.

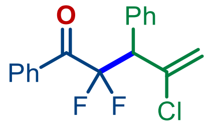

(102) According to the general procedure E, using phenyl trifluoromethyl ketone *N*-triftosylhydrazone (118.8 mg, 0.3 mmol), 2-chloro-3-phenylprop-2-en-1-ol (100.8 mg, 0.6 mmol), and DCE (4.0 mL) afforded compound **102** (85.4 mg, 93% yield) as a colorless oil; **^{1}H-NMR** (500 MHz, $CDCl_3$) δ 8.00 (d, *J* = 8.0 Hz, 2H), 7.66-7.60 (m, 1H), 7.52-7.42 (m, 4H), 7.41-7.35 (m, 3H), 5.62 (s, 1H), 5.55 (s, 1H), 4.71 (dd, *J* = 17.5, 14.7 Hz, 1H). **^{13}C-NMR** (150 MHz, $CDCl_3$) δ 189.0 (t, *J* = 28.5 Hz), 136.8, 136.7, 134.2, 132.3 (t, *J* = 4.5 Hz), 130.0, 129.9 (t, *J* = 3.0 Hz), 128.7, 128.6, 128.5, 117.6 (dd, *J* = 261.0, 258.0 Hz), 116.9, 56.9 (t, *J* = 22.5 Hz). **^{19}F-NMR** (564 MHz, $CDCl_3$) δ -99.98 (dd, *J* = 285.3, 14.7 Hz), -102.85 (dd, *J* = 285.3, 17.5 Hz). **IR** (Film): 3066, 2925, 1701, 1597, 1448, 1199, 1049, 907 cm^{-1}. **HRMS** (ESI) m/z calcd. for $C_{17}H_{14}ClF_2O^+$ $[M+H]^+$: 307.0695; Found: 307.0690.

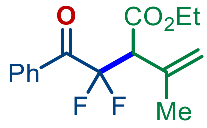

(103) According to the general procedure E, using phenyl trifluoromethyl ketone *N*-triftosylhydrazone (118.8 mg, 0.3 mmol), ethyl (E)-4-hydroxy-3-methylbut-2-enoate (86.4 mg, 0.6 mmol), and DCE (4.0 mL) afforded compound **103** (68.5 mg, 81% yield) as a colorless oil; **^{1}H-NMR** (500 MHz, $CDCl_3$) δ 8.08 (d, *J* = 7.5 Hz, 2H), 7.65-7.60 (m, 1H), 7.52-7.46 (m, 2H), 5.24 (s, 1H), 5.12 (s, 1H), 4.18 (q, *J* = 7.0 Hz, 2H), 1.94 (s, 3H), 1.23 (t, *J* = 7.0 Hz, 3H). **^{13}C-NMR** (150 MHz, $CDCl_3$) δ 189.4 (dd, *J* = 33.0, 30.0 Hz), 168.1 (d, *J* = 12.0 Hz), 135.3, 134.2, 132.2, 130.1 (dd, *J* = 4.5, 1.5 Hz), 128.6, 119.9, 117.2 (dd, *J* = 265.5, 250.5 Hz), 61.4, 55.5 (dd, *J* = 24.0, 19.5 Hz), 22.2 (d, *J* = 1.5 Hz), 13.9. **^{19}F-NMR** (564 MHz, $CDCl_3$) δ -98.97 (dd, *J* = 295.0, 7.9 Hz), -107.30 (dd, *J* =

295.0, 23.7 Hz). **IR** (Film): 3061, 2983, 1732, 1372, 1236, 1644, 911, 729 cm^{-1}. **HRMS** (ESI) m/z calcd. for $C_{15}H_{17}F_2O_3^+$ [M+H]$^+$: 283.1140; Found: 283.1135.

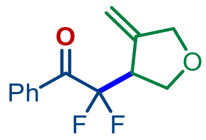

(104) According to the general procedure E, using phenyl trifluoromethyl ketone *N*-triftosylhydrazone (118.8 mg, 0.3 mmol), (2,5-dihydrofuran-3-yl)methanol (60.0 mg, 0.6 mmol), and DCE (4.0 mL) afforded compound **104** (53.6 mg, 75% yield) as a colorless oil; **^{1}H-NMR** (500 MHz, $CDCl_3$) δ 8.16-8.07 (m, 2H), 7.69-7.61 (m, 1H), 7.56-7.47 (m, 2H), 5.26 (s, 1H), 5.21 (s, 1H), 4.39-4.29 (m, 2H), 4.24-4.18 (m, 1H), 4.06 (dd, *J* = 9.5, 5.0 Hz, 1H), 3.74-3.62 (m, 1H). **^{13}C-NMR** (150 MHz, $CDCl_3$) δ 188.9 (t, *J* = 31.5 Hz), 143.1, 134.5, 132.1 (t, *J* = 3.0 Hz), 130.2 (t, *J* = 3.0 Hz), 128.7, 118.8 (t, *J* = 255.0 Hz), 109.8, 72.1, 68.7 (t, *J* = 4.5 Hz), 46.7 (t, *J* = 22.5 Hz). **^{19}F-NMR** (564 MHz, $CDCl_3$) δ -101.72 (dd, *J* = 296.1, 14.1 Hz), -104.59 (dd, *J* = 296.1, 18.6 Hz). **IR** (Film): 3053, 2987, 1704, 1600, 1448, 1265, 908, 732, 706 cm^{-1}. **HRMS** (ESI) m/z calcd. for $C_{13}H_{13}F_2O_2^+$ [M+H]$^+$: 239.0878; Found: 239.0875.

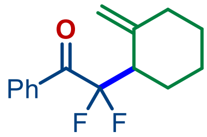

(105) According to the general procedure E, using phenyl trifluoromethyl ketone *N*-triftosylhydrazone (118.8 mg, 0.3 mmol), cyclohex-1-en-1-ylmethanol (67.2 mg, 0.6 mmol), and DCE (4.0 mL) afforded compound **105** (73.5 mg, 98% yield) as a colorless oil; **^{1}H-NMR** (500 MHz, $CDCl_3$) δ 8.08-8.02 (d, *J* = 7.8 Hz, 2H), 7.65-7.59 (m, 1H), 7.52-7.46 (m, 2H), 4.86 (s, 1H), 4.60 (s, 1H), 3.20-3.08 (m, 1H), 2.35-2.25 (m, 1H), 2.20-2.10 (m, 1H), 1.95-1.86 (m, 1H), 1.85-1.74 (m, 2H), 1.72-1.62 (m, 1H), 1.60-1.47 (m, 2H). **^{13}C-NMR** (125 MHz, $CDCl_3$) δ 190.1 (t, *J* = 30.0 Hz), 143.6, 133.9, 133.0, 129.9 (t, *J* = 2.5 Hz), 128.6, 120.3 (dd, *J* = 257.5, 255.0 Hz), 112.4, 46.2 (t, *J* = 21.3 Hz), 34.7, 27.6, 26.1 (t, *J* = 2.5 Hz), 23.4. **^{19}F-NMR** (470 MHz, $CDCl_3$) δ -101.15 (dd, *J* = 272.6, 14.1 Hz), -103.93 (dd, *J* = 272.6, 21.2 Hz). **IR** (Film): 3074, 2937, 2859, 1703, 1448, 1029, 687 cm^{-1}. **HRMS** (ESI) m/z calcd. for $C_{15}H_{17}F_2O^+$ [M+H]$^+$: 251.1242; Found: 251.1240.

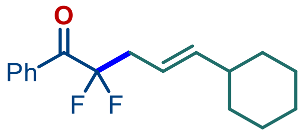

(106) According to the general procedure E, using phenyl trifluoromethyl ketone *N*-triftosylhydrazone (118.8 mg, 0.3 mmol), 1-cyclohexylprop-2-en-1-ol (84.0 mg, 0.6 mmol), and DCE (4.0 mL) afforded compound **106** (73.4 mg, 88% yield) as a colorless oil; **^{1}H-NMR** (500 MHz, $CDCl_3$) δ 8.11-8.05 (m, 2H), 7.66-7.58 (m, 1H), 7.51-7.45 (m, 2H), 5.56 (dd, *J* = 15.5, 7.0 Hz, 1H), 5.41-5.31 (m, 1H), 2.88 (td, *J* = 17.0, 7.0 Hz, 2H), 2.00-1.88 (m, 1H), 1.74-1.59 (m, 5H), 1.30-1.18 (m, 2H), 1.18-1.09 (m, 1H), 1.08-0.97 (m, 2H). **^{13}C-NMR** (125 MHz, $CDCl_3$) δ 189.6 (t, *J* = 30.0 Hz), 143.7, 134.1, 132.3, 130.1 (t, *J* = 2.5 Hz), 128.6, 118.8 (t, *J* = 251.3 Hz), 116.1 (t, *J* = 5.0 Hz), 40.7, 37.9 (t, *J* = 22.5 Hz), 32.6, 26.1, 25.9. **^{19}F-NMR** (470 MHz, $CDCl_3$) δ -99.72 (t, *J* = 17.0 Hz). **HRMS** (ESI) m/z calcd. for $C_{17}H_{21}F_2O^+$ [M+H]$^+$: 279.1555; Found: 279.1556.

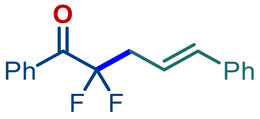

(107) According to the general procedure E, using phenyl trifluoromethyl ketone *N*-triftosylhydrazone (118.8 mg, 0.3 mmol), 1-phenylprop-2-en-1-ol (80.4 mg, 0.6 mmol), and DCE (4.0 mL) afforded compound **107** (58.8 mg, 72% yield) as a colorless oil; **^{1}H-NMR** (500 MHz, $CDCl_3$) δ 8.16-8.08 (m, 2H), 7.68-7.60 (m, 1H), 7.54-7.46 (m, 2H), 7.38-7.34 (m, 2H), 7.33-7.28 (m, 2H), 7.25-7.21 (m, 1H), 6.58 (d, *J* = 15.5 Hz, 1H), 6.20 (dt, *J* = 15.5, 7.0 Hz, 1H), 3.13 (tdd, *J* = 17.0, 7.0, 1.5 Hz, 2H). **^{13}C-NMR** (125 MHz, $CDCl_3$) δ 189.2 (t, *J* = 31.3 Hz), 136.7, 136.2, 134.3, 132.0 (t, *J* = 2.5 Hz), 130.2 (t, *J* = 2.5 Hz), 128.7, 128.5, 127.8, 126.4, 118.8 (t, *J* = 5.0 Hz), 118.7 (t, *J* = 253.8 Hz), 37.8 (t, *J* = 23.8 Hz). **^{19}F-NMR** (470 MHz, $CDCl_3$) δ -98.92 (t, *J* = 17.0 Hz). **IR** (Film): 3061, 3027, 2923, 1265, 1171, 966, 733, 701 cm^{-1}. **HRMS** (ESI) m/z calcd. for $C_{17}H_{15}F_2O^+$ $[M+H]^+$: 273.1085; Found: 273.1082.

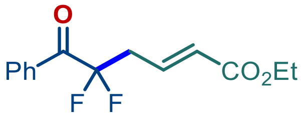

(108) According to the general procedure E, using phenyl trifluoromethyl ketone *N*-triftosylhydrazone (118.8 mg, 0.3 mmol), ethyl 2-hydroxybut-3-enoate (78.0 mg, 0.6 mmol), and DCE (4.0 mL) afforded compound **108** (45.8 mg, 57% yield) as a colorless oil; **^{1}H-NMR** (500 MHz, $CDCl_3$) δ 8.15-8.08 (m, 2H), 7.69-7.62 (m, 1H), 7.54-7.48 (m, 2H), 6.95 (dt, *J* = 16.0, 7.0 Hz, 1H), 6.04 (d, *J* = 16.0 Hz, 1H), 4.20 (q, *J* = 7.0 Hz, 2H), 3.12 (tdd, *J* = 17.0, 7.0, 1.5 Hz, 2H), 1.29 (t, *J* = 7.0 Hz, 3H). **^{13}C-NMR** (150 MHz, $CDCl_3$) δ 188.3 (t, *J* = 31.5 Hz), 165.6, 137.2 (t, *J* = 6.0 Hz), 134.6, 131.4 (t, *J* = 3.0 Hz), 130.2 (t, *J* = 3.0 Hz), 128.8, 127.2, 118.2 (t, *J* = 253.5 Hz), 60.6, 36.5 (t, *J* = 24.0 Hz), 14.2. **^{19}F-NMR** (564 MHz, $CDCl_3$) δ -98.20 (t, *J* = 17.0 Hz). **IR** (Film): 3074, 2981, 1720, 1702, 1265, 1183, 731, 703 cm^{-1}. **HRMS** (ESI) m/z calcd. for $C_{14}H_{15}F_2O_3^+$ $[M+H]^+$: 269.0983; Found: 269.0980.

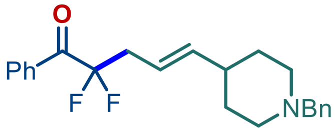

(109) According to the general procedure E, using phenyl trifluoromethyl ketone *N*-triftosylhydrazone (118.8 mg, 0.3 mmol), 1-(1-benzylpiperidin-4-yl)prop-2-en-1-ol (138.6 mg, 0.6 mmol), and DCE (4.0 mL) afforded compound **109** (104.0 mg, 94% yield) as a colorless oil; **^{1}H-NMR** (500 MHz, $CDCl_3$) δ 8.07 (d, *J* = 8.0 Hz, 2H), 7.64-7.59 (m, 1H), 7.52-7.46 (m, 2H), 7.33-7.28 (m, 4H), 7.25-7.21 (m, 1H), 5.57 (dd, *J* = 16.0, 7.0 Hz, 1H), 5.44-5.36 (m, 1H), 3.47 (s, 2H), 3.00-2.80 (m, 4H), 2.00-1.89 (m, 3H), 1.65-1.57 (m, 2H), 1.45-1.33 (m, 2H). **^{13}C-NMR** (150 MHz, $CDCl_3$) δ 189.4 (t, *J* = 30.0 Hz), 142.3, 138.5, 134.2, 132.2, 130.1 (t, *J* = 3.0 Hz), 129.2, 128.6, 128.1, 126.9, 118.8 (t, *J* = 252.0 H), 117.1, 63.4, 53.4, 38.9, 37.7 (t, *J* = 24.0 Hz), 31.8. **^{19}F-NMR** (564 MHz, $CDCl_3$) δ -99.67 (t, *J* = 17.0 Hz).

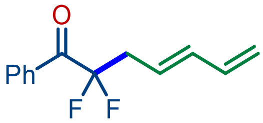

(110) According to the general procedure E, using phenyl trifluoromethyl ketone *N*-triftosylhydrazone (118.8 mg, 0.3 mmol), penta-1,4-dien-3-ol (50.4 mg, 0.6 mmol), and DCE (4.0 mL) afforded compound **110** (30.6 mg, 46% yield) as a colorless oil; **^{1}H-NMR** (500 MHz, $CDCl_3$) δ 8.16-8.06 (m, 2H), 7.68-7.60 (m, 1H), 7.54-7.45 (m, 2H), 6.39-6.19 (m, 2H), 5.75-5.65 (m, 1H), 5.19 (d, *J* = 16.5 Hz, 1H), 5.09 (d, *J* = 10.5 Hz, 1H), 3.00 (td, *J* = 17.0, 7.0 Hz, 2H). **^{13}C-NMR** (125 MHz, $CDCl_3$) δ 189.2 (t, *J* = 30.0 Hz), 137.0, 136.2, 134.3, 132.0, 130.2 (t, *J* = 2.5 Hz), 128.7, 122.7 (t, *J* = 5.0 Hz), 118.6 (t, *J* = 252.5 Hz), 117.5, 37.4 (t, *J* = 23.8 Hz). **^{19}F-NMR** (470 MHz, $CDCl_3$) δ -99.03 (t, *J* = 17.0 Hz). **IR** (Film): 3093, 2926, 1701, 1264, 733, 703 cm^{-1}.

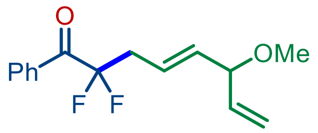

(111) According to the general procedure E, using phenyl trifluoromethyl ketone *N*-triftosylhydrazone (118.8 mg, 0.3 mmol), 4-methoxyhexa-1,5-dien-3-ol (76.8 mg, 0.6 mmol), and DCE (4.0 mL) afforded compound **111** (68.6 mg, 86% yield) as a colorless oil; **^{1}H-NMR** (500 MHz, $CDCl_3$) δ 8.12-8.06 (m, 2H), 7.65-7.58 (m, 1H), 7.52-7.44 (m, 2H), 5.78-5.67 (m, 2H), 5.62 (dd, *J* = 15.0, 6.5 Hz, 1H), 5.25-5.15 (m, 2H), 4.05 (t, *J* = 6.5 Hz, 1H), 3.27 (s, 3H), 2.97 (td, *J* = 17.5, 7.0 Hz, 2H). **^{13}C-NMR** (150 MHz, $CDCl_3$) δ 189.0 (t, *J* = 31.5 Hz), 137.2, 136.2, 134.3, 131.9 (t, *J* = 3.0 Hz), 130.1 (t, *J* = 3.0 Hz), 128.7, 122.1 (t, *J* = 252.0 Hz), 116.9, 82.4, 55.9, 37.2 (t, *J* = 24.0 Hz). **^{19}F-NMR** (564 MHz, $CDCl_3$) δ -99.29 (t, *J* = 17.5 Hz). **IR** (Film): 3057, 2938, 1702, 1597, 1453, 1261, 908, 736, 717, 649 cm^{-1}. **HRMS** (ESI) m/z calcd. for $C_{15}H_{17}F_2O_2^+$ [M+H]$^+$: 267.1191; Found: 267.1196.

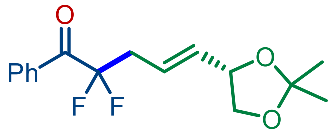

(112) According to the general procedure E, using phenyl trifluoromethyl ketone *N*-triftosylhydrazone (118.8 mg, 0.3 mmol), 1-((*S*)-2,2-dimethyl-1,3-dioxolan-4-yl)but-3-en-2-ol (103.2 mg, 0.6 mmol), and DCE (4.0 mL) afforded compound **112** (47.1 mg, 53% yield) as a colorless oil; **^{1}H-NMR** (500 MHz, $CDCl_3$) δ 8.13-8.05 (m, 2H), 7.66-7.60 (m, 1H), 7.53-7.45 (m, 2H), 5.88-5.77 (m, 1H), 5.69 (dd, *J* = 15.5, 7.5 Hz, 1H), 4.50 (q, *J* = 7.0 Hz, 1H), 4.07 (dd, *J* = 8.5, 6.5 Hz, 1H), 3.56 (t, *J* = 8.0 Hz, 1H), 3.05-2.89 (m, 1H), 1.41 (s, 3H), 1.37 (s, 3H). **^{13}C-NMR** (125 MHz, $CDCl_3$) δ 188.9 (t, *J* = 31.3 Hz), 134.6, 134.4, 131.8 (t, *J* = 2.5 Hz), 130.2, 128.7, 123.5 (t, *J* = 6.3 Hz), 118.4 (t, *J* = 252.5 Hz), 109.4, 76.5, 69.3, 37.0 (t, *J* = 25.0 Hz), 26.6, 25.8. **^{19}F-NMR** (470 MHz, $CDCl_3$) δ -99.15 (q, *J* = 17.0 Hz). **IR** (Film): 3063, 2987, 2927, 1702, 1380, 1059, 734 cm^{-1}. **HRMS** (ESI) m/z calcd. for $C_{16}H_{19}F_2O_3^+$ [M+H]$^+$: 297.1302; Found: 297.1318.

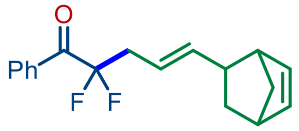

(113) According to the general procedure E, using phenyl trifluoromethyl ketone *N*-triftosylhydrazone (118.8 mg, 0.3 mmol), 1-(bicyclo[2.2.1]hept-5-en-2-yl)prop-2-en-1-ol (90.0 mg, 0.6 mmol) and DCE (4.0 mL) afforded compound **113** (69.9 mg, 81% yield) as a colorless oil; **^{1}H-NMR** (500 MHz, $CDCl_3$) δ 8.12-8.05 (m, 2H), 7.67-7.59 (m, 1H), 7.53-7.46 (m, 2H), 6.12 (dd, *J* = 6.0, 3.5 Hz, 1H), 5.86 (dd, *J* = 6.0, 3.0 Hz, 1H), 5.44-5.34 (m, 1H), 5.25 (dd, *J* = 15.0, 8.5 Hz, 1H), 2.85 (td, *J* = 16.5, 7.0 Hz, 2H), 2.81-2.78 (m, 1H), 2.77-2.73 (m, 1H), 2.71-2.64 (m, 1H), 1.93-1.85 (m, 1H), 1.40-1.34 (m, 1H), 1.25-1.21 (m, 1H), 0.77-0.69 (m, 1H). **^{13}C-NMR** (150 MHz, $CDCl_3$) δ 189.6 (t, *J* = 30.0 Hz), 142.7, 137.3, 134.1, 132.6, 130.1 (t, *J* = 3.0 Hz), 128.6, 118.8 (t, *J* = 252.0 Hz), 117.5 (t, *J* = 6.0 Hz), 49.5, 47.9, 42.8, 41.9, 37.7 (t, *J* = 24.0 Hz), 32.6. **^{19}F-NMR** (564 MHz, $CDCl_3$) δ -99.66 (dt, *J* = 27.1, 17.0 Hz). **HRMS** (ESI) m/z calcd. for $C_{18}H_{19}F_2O^+$ [M+H]$^+$: 289.1398; Found: 289.1393.

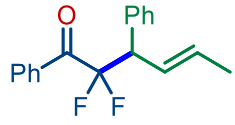

(114) According to the general procedure E, using phenyl trifluoromethyl ketone *N*-triftosylhydrazone (118.8 mg, 0.3 mmol), 4-phenylbut-3-en-2-ol (88.8 mg, 0.6 mmol), and DCE (4.0 mL) afforded compound **115** (61.8 mg, 72%

yield) as a colorless oil; ^{1}**H-NMR** (500 MHz, $CDCl_3$) δ 7.98-7.93 (m, 2H), 7.62-7.57 (m, 1H), 7.48-7.42 (m, 2H), 7.35-7.25 (m, 5H), 5.88-5.80 (m, 1H), 5.69-5.60 (m, 1H), 4.26 (td, *J* = 16.5, 8.5 Hz, 1H), 1.69 (dd, *J* = 6.5, 1.5 Hz, 3H). ^{13}**C-NMR** (150 MHz, $CDCl_3$) δ 190.2 (t, *J* = 28.5 Hz), 135.9, 133.9, 133.1, 131.7, 129.8 (t, *J* = 4.5 Hz), 129.4, 128.5, 127.6, 125.0 (t, *J* = 4.5 Hz), 118.7 (t, *J* = 258.0 Hz), 53.2 (t, *J* = 22.5 Hz), 18.1. ^{19}**F-NMR** (564 MHz, $CDCl_3$) δ -103.21 (dd, *J* = 270.8, 15.8 Hz, 1F), -104.03 (dd, *J* = 270.8, 17.0 Hz, 1F). **IR** (Film): 3057, 2918, 1703, 1264, 1172, 733, 699 cm^{-1}. **HRMS** (ESI) m/z calcd. for $C_{18}H_{17}F_2O^+$ $[M+H]^+$: 287.1242; Found: 287.1230.

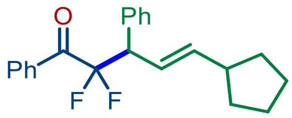

(115) According to the general procedure E, using phenyl trifluoromethyl ketone *N*-triftosylhydrazone (118.8 mg, 0.3 mmol), 1-cyclopentyl-3-phenylprop-2-en-1-ol (121.2 mg, 0.6 mmol), and DCE (4.0 mL) afforded compound **115**(68.3 mg, 67% yield) as a colorless oil; ^{1}**H-NMR** (500 MHz, $CDCl_3$) δ 7.95 (d, *J* = 7.5 Hz, 2H), 7.63-7.56 (m, 1H), 7.49-7.42 (m, 2H), 7.37-7.26 (m, 5H), 5.75 (dd, *J* = 15.5, 9.0 Hz, 1H), 5.55 (dd, *J* = 15.5, 7.5 Hz, 1H), 4.27-4.15 (m, 1H), 2.48-2.33 (m, 1H), 1.77-1.63 (m, 2H), 1.63-1.45 (m, 4H), 1.24-1.11 (m, 2H). ^{13}**C-NMR** (150 MHz, $CDCl_3$) δ 190.4 (t, *J* = 28.5 Hz), 141.7, 135.9, 133.9, 133.3, 129.8 (t, *J* = 4.5 Hz), 129.5, 128.5, 128.5, 127.6, 121.5 (dd, *J* = 6.0, 1.5 Hz), 118.8 (t, *J* = 258.0 Hz), 53.3 (t, *J* = 21.0 Hz), 43.2, 32.7, 25.0. ^{19}**F-NMR** (564 MHz, $CDCl_3$) δ -102.31 (dd, *J* = 266.8, 13.5 Hz), -105.75 (dd, *J* = 266.8, 19.2 Hz). **IR** (Film): 3271, 2951, 1704, 1172, 1051, 713 cm^{-1}. **HRMS** (ESI) m/z calcd. for $C_{22}H_{23}F_2O^+$ $[M+H]^+$: 341.1711; Found: 341.1709.

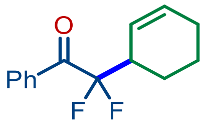

(116) According to the general procedure E, using phenyl trifluoromethyl ketone *N*-triftosylhydrazone (118.8 mg, 0.3 mmol), cyclohex-2-en-1-ol (58.8 mg, 0.6 mmol), and DCE (4.0 mL) afforded compound **116** (66.5 mg, 94% yield) as a colorless oil; ^{1}**H-NMR** (500 MHz, $CDCl_3$) δ 8.14-8.03 (m, 2H), 7.65-7.59 (m, 1H), 7.53-7.45 (m, 2H), 6.00-5.91 (m, 1H), 5.72-5.64 (m, 1H), 3.24-3.06 (m, 1H), 2.10-1.98 (m, 2H), 1.90-1.79 (m, 2H), 1.69-1.48 (m, 2H). ^{13}**C-NMR** (150 MHz, $CDCl_3$) δ 189.9 (t, *J* = 30.0 Hz), 134.2, 132.8, 131.8, 130.1 (t, *J* = 4.5 Hz), 128.7, 121.4 (t, *J* = 4.5 Hz), 119.8 (t, *J* = 253.5 Hz), 40.0 (t, *J* = 21.0 Hz), 24.7, 21.8 (t, *J* = 3.0 Hz), 20.8. ^{19}**F-NMR** (564 MHz, $CDCl_3$) δ -105.65 (dd, *J* = 276.4, 17.0 Hz), -107.77 (dd, *J* = 276.4, 17.0 Hz). **IR** (Film): 3034, 2937, 1704, 1597, 1449, 1265, 915, 730, 697 cm^{-1}. **HRMS** (ESI) m/z calcd. for $C_{14}H_{15}F_2O^+$ $[M+H]^+$: 237.1085; Found: 237.1081.

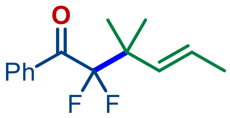

(117) According to the general procedure E, using phenyl trifluoromethyl ketone *N*-triftosylhydrazone (118.8 mg, 0.3 mmol), 4-methylpent-3-en-2-ol (60.0 mg, 0.6 mmol), and DCE (4.0 mL) afforded compound **117** (59.3 mg, 83% yield) as a colorless oil; ^{1}**H-NMR** (500 MHz, $CDCl_3$) δ 8.04-7.98 (m, 2H), 7.61- 7.53 (m, 1H), 7.47-7.41 (m, 2H), 5.57-5.47 (m, 2H), 1.59 (d, *J* = 4.5 Hz, 3H), 1.24 (s, 6H). ^{13}**C-NMR** (150 MHz, $CDCl_3$) δ 190.8 (t, *J* = 30.0 Hz), 134.5, 133.6, 133.0 (t, *J* = 4.5 Hz), 130.3 (t, *J* = 4.5 Hz), 128.3, 126.4, 121.0 (t, *J* = 258.0 Hz), 42.6 (t, *J* = 21.0 Hz), 21.6 (t, *J* = 4.5 Hz), 18.0. ^{19}**F-NMR** (564 MHz, $CDCl_3$) δ -107.84 (s).

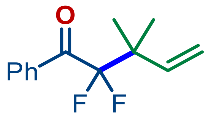

(118) According to the general procedure E, using phenyl trifluoromethyl ketone *N*-triftosylhydrazone (118.8 mg, 0.3 mmol), 3-methylbut-2-en-1-ol (51.6 mg, 0.6 mmol), and DCE (4.0 mL) afforded compound **118** (65.8 mg, 98% yield) as a colorless oil; ^{1}**H-NMR** (500 MHz, $CDCl_3$) δ 8.04 (d, *J* = 8.0 Hz, 2H), 7.62-7.56 (m, 1H), 7.48-

7.42 (m, 2H), 6.01 (dd, *J* = 17.5, 11.0 Hz, 1H), 5.18-5.11 (m, 2H), 1.27 (s, 6H). **^{13}C-NMR** (125 MHz, $CDCl_3$) δ 190.3 (t, *J* = 31.3 Hz), 140.0 (t, *J* = 3.8 Hz), 134.2 (t, *J* = 2.5 Hz), 133.8, 130.2 (t, *J* = 5.0 Hz), 128.4, 120.7 (t, *J* = 258.8 Hz), 115.3, 43.5 (t, *J* = 21.3 Hz), 21.2 (t, *J* = 3.8 Hz). **^{19}F-NMR** (470 MHz, $CDCl_3$) δ -107.41 (s). **IR** (Film): 3289, 2946, 2253, 1646, 1271, 1037, 732 cm^{-1}.

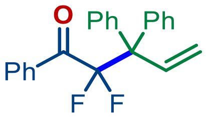

(119) According to the general procedure E, using phenyl trifluoromethyl ketone *N*-triftosylhydrazone (118.8 mg, 0.3 mmol), 3,3-diphenylprop-2-en-1-ol (126.0 mg, 0.6 mmol), and DCE (4.0 mL) afforded compound **119** (55.3 mg, 53% yield) as a colorless oil; **^{1}H-NMR** (500 MHz, $CDCl_3$) δ 7.79-7.73 (m, 2H), 7.55-7.49 (m, 1H), 7.42-7.26 (m, 12H), 7.00 (dd, *J* = 17.0, 10.5 Hz, 1H), 5.56 (d, *J* = 10.5 Hz, 1H), 4.82 (d, *J* = 17.0 Hz, 1H). **^{13}C-NMR** (125 MHz, $CDCl_3$) δ 190.5 (t, *J* = 31.3 Hz), 139.8, 138.7 (t, *J* = 3.8 Hz), 134.2 (t, *J* = 2.5 Hz), 133.3, 130.3 (t, *J* = 2.5 Hz), 129.7 (t, *J* = 3.8 Hz), 128.1, 127.8, 127.2, 120.5, 119.8 (t, *J* = 265.0 Hz), 62.3 (t, *J* = 20.0 Hz). **^{19}F-NMR** (470 MHz, $CDCl_3$) δ -94.70 (s). **IR** (Film): 3051, 2987, 1708, 1598, 1447, 1259, 906, 724, 649 cm^{-1}. **HRMS** (ESI) m/z calcd. for $C_{23}H_{19}F_2O^+$ [M+H]$^+$: 349.1398; Found: 349.1393.

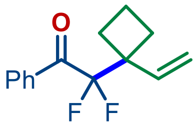

(120) According to the general procedure E, using phenyl trifluoromethyl ketone *N*-triftosylhydrazone (118.8 mg, 0.3 mmol), 2-cyclobutylideneethan-1-ol (58.8 mg, 0.6 mmol) and DCE (4.0 mL) afforded compound **120** (67.3 mg, 95% yield) as a colorless oil; **^{1}H-NMR** (500 MHz, $CDCl_3$) δ 8.07-8.00 (m, 2H), 7.62-7.56 (m, 1H), 7.49-7.42 (m, 2H), 6.01 (dd, *J* = 17.5, 11.0 Hz, 1H), 5.33 (d, *J* = 17.5 Hz, 1H), 5.28 (d, *J* = 11.0 Hz, 1H), 2.66-2.55 (m, 2H), 2.13-2.03 (m, 2H), 2.02-1.85 (m, 2H). **^{13}C-NMR** (150 MHz, $CDCl_3$) δ 189.7 (t, *J* = 31.5 Hz), 137.7 (t, *J* = 4.5 Hz), 133.8, 133.5 (t, *J* = 3.0 Hz), 130.0 (t, *J* = 4.5 Hz), 128.4, 119.1 (t, *J* = 256.5 Hz), 116.7, 48.7 (t, *J* = 22.5 Hz), 26.7 (t, *J* = 4.5 Hz), 15.6. **^{19}F-NMR** (564 MHz, $CDCl_3$) δ -108.19 (s). **IR** (Film): 3053, 2952, 1702, 1598, 1448, 1265, 1141, 908, 724, 649 cm^{-1}. **HRMS** (ESI) m/z calcd. for $C_{14}H_{15}F_2O^+$ [M+H]$^+$: 237.1085; Found: 237.1082.

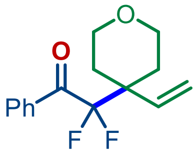

(121) According to the general procedure E, using phenyl trifluoromethyl ketone *N*-triftosylhydrazone (118.8 mg, 0.3 mmol), 2-(tetrahydro-4*H*-pyran-4-ylidene)ethan-1-ol (76.8 mg, 0.6 mmol), and DCE (4.0 mL) afforded compound **121** (64.6 mg, 94% yield) as a colorless oil; **^{1}H-NMR** (500 MHz, $CDCl_3$) δ 8.01 (d, *J* = 8.0 Hz, 2H), 7.62-7.55 (m, 1H), 7.48-7.42 (m, 2H), 5.76 (dd, *J* = 18.0, 11.0 Hz, 1H), 5.47 (d, *J* = 11.0 Hz, 1H), 5.26 (d, *J* = 18.0 Hz, 1H), 3.84 (dd, *J* = 11.5, 4.5 Hz, 2H), 3.54 (td, *J* = 12.5, 2.0 Hz, 2H), 2.15 (td, *J* = 13.5, 5.0 Hz, 2H), 1.91-1.83 (m, 2H). **^{13}C-NMR** (150 MHz, $CDCl_3$) δ 190.0 (t, *J* = 30.0 Hz), 136.1, 133.9, 133.9, 130.2 (t, *J* = 4.5 Hz), 128.5, 121.2, 119.2 (t, *J* = 259.5 Hz), 63.3, 45.1 (t, *J* = 21.0 Hz), 28.7. **^{19}F-NMR** (564 MHz, $CDCl_3$) δ -108.48 (s). **HRMS** (ESI) m/z calcd. for $C_{15}H_{17}F_2O_2^+$ [M+H]$^+$: 267.1191; Found: 267.1190.

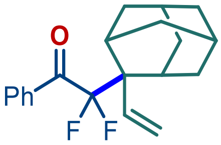

(122) According to the general procedure E, using phenyl trifluoromethyl ketone *N*-triftosylhydrazone (118.8 mg, 0.3 mmol), 2-(adamantan-2-ylidene)ethan-1-ol (106.8 mg, 0.6 mmol), and DCE (4.0 mL) afforded compound **122** (91 mg, 96% yield) as a colorless oil; **^{1}H-NMR** (500 MHz, $CDCl_3$) δ 7.99-7.91 (m, 2H), 7.58-7.51 (m, 1H), 7.45-7.39 (m, 2H), 5.62 (dd, *J* = 18.0, 11.0 Hz, 1H), 5.29 (d, *J* = 11.0 Hz, 1H), 5.11 (d, *J* = 18.0 Hz, 1H), 2.58-2.46 (m, 4H), 2.11-2.02 (m, 2H), 2.00-1.94 (m, 1H), 1.83-1.71 (m, 5H), 1.62-1.54 (m, 2H). **^{13}C-NMR** (150 MHz, $CDCl_3$) δ 192.0 (t, *J* = 30.0 Hz), 140.8 (t, *J* = 4.5 Hz), 135.0 (d, *J* = 3.0 Hz), 133.1, 130.1 (t, *J* = 4.5 Hz), 128.1, 121.6 (t, *J* = 265.5 Hz), 120.0, 50.5 (t, *J* = 19.5 Hz), 39.2, 34.2, 33.4 (t, *J* = 3.0 Hz), 31.1, 27.7, 26.9. **^{19}F-NMR** (564 MHz, $CDCl_3$) δ -97.14 (s). **HRMS** (ESI) m/z calcd. for $C_{20}H_{23}F_2O^+$ $[M+H]^+$: 317.1711; Found: 317.1707.

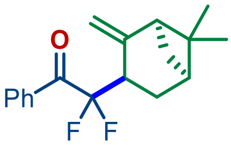

(123) According to the general procedure E, using phenyl trifluoromethyl ketone *N*-triftosylhydrazone (118.8 mg, 0.3 mmol), ((1*R*,5*S*)-6,6-dimethylbicyclo[3.1.1]hept-2-en-2-yl)methanol (91.2 mg, 0.6 mmol), and DCE (4.0 mL) afforded compound **123** (86.1 mg, 99% yield) as a colorless oil; **^{1}H-NMR** (500 MHz, $CDCl_3$) δ 8.13-8.05 (m, 2H), 7.66-7.59 (m, 1H), 7.54-7.46 (m, 2H), 4.95 (s, 1H), 4.92 (s, 1H), 3.71-3.57 (m, 1H), 2.49 (t, *J* = 5.5 Hz, 1H), 2.38-2.30 (m, 1H), 2.15-2.06 (m, 1H), 2.01-1.95 (m, 1H), 1.90-1.82 (m, 1H), 1.49 (d, *J* = 10.5 Hz, 1H), 1.27 (s, 3H), 0.77 (s, 3H). **^{13}C-NMR** (150 MHz, $CDCl_3$) δ 190.9 (t, *J* = 30.0 Hz), 145.5, 134.0, 133.1, 130.1(t, *J* = 4.5 Hz), 128.6, 119.7 (t, *J* = 255.0 Hz), 113.6 (d, *J* = 3.0 Hz), 52.3, 40.7, 39.6, 37.7 (dd, *J* = 24.0, 19.5 Hz), 27.5-27.3 (m), 25.8, 25.6-25.4 (m), 21.7. **^{19}F-NMR** (564 MHz, $CDCl_3$) δ -98.78 (dd, *J* = 270.7, 15.3 Hz), -106.14 (dd, *J* = 270.7, 24.3 Hz). **IR** (Film): 3061, 2970, 1702, 1598, 1265, 1173, 737, 716 cm^{-1}. **HRMS** (ESI) m/z calcd. for $C_{18}H_{21}F_2O^+$ $[M+H]^+$: 291.1555; Found: 291.1552.

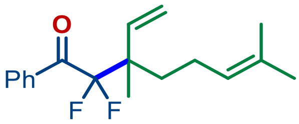

(124) According to the general procedure E, using phenyl trifluoromethyl ketone *N*-triftosylhydrazone (118.8 mg, 0.3 mmol), 3,7-dimethylocta-2,6-dien-1-ol (92.4 mg, 0.6 mmol), and DCE (4.0 mL) afforded compound **124** (70.9 mg, 81% yield) as a colorless oil; **^{1}H-NMR** (500 MHz, $CDCl_3$) δ 8.03 (d, *J* = 7.9 Hz, 2H), 7.63-7.55 (m, 1H), 7.50-7.41 (m, 2H), 5.90 (dd, *J* = 17.5, 11.0 Hz, 1H), 5.23 (d, *J* = 11.0 Hz, 1H), 5.14 (d, *J* = 17.5 Hz, 1H), 5.10-5.04 (m, 1H), 1.97-1.84 (m, 2H), 1.73-1.68 (m, 2H), 1.67 (s, 3H), 1.58 (s, 3H), 1.26 (s, 3H). **^{13}C-NMR** (125 MHz, $CDCl_3$) δ 190.5 (t, *J* = 31.3 Hz), 138.3 (t, *J* = 3.8 Hz), 134.4, 133.7, 131.8, 130.3 (t, *J* = 3.8 Hz), 128.4, 124.0, 120.8 (t, *J* = 260.0 Hz), 117.1, 46.9 (t, *J* = 20.0 Hz), 33.6, 25.6, 22.2, 17.6, 16.1 (t, *J* = 4.5 Hz). **^{19}F-NMR** (470 MHz, $CDCl_3$) δ -106.58 (s). **IR** (Film): 3093, 2927, 1693, 1448, 1265, 726, 697 cm^{-1}. **HRMS** (ESI) m/z calcd. for $C_{18}H_{23}F_2O^+$ $[M+H]^+$: 291.1566; Found: 291.1570.

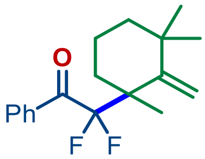

(125) According to the general procedure E, using phenyl trifluoromethyl ketone *N*-triftosylhydrazone (118.8 mg, 0.3 mmol), (2,6,6-trimethylcyclohex-1-en-1-yl)methanol (92.4 mg, 0.6 mmol), and DCE (4.0 mL) afforded compound **125** (85.8 mg, 98% yield) as a colorless oil; **^{1}H-NMR** (500 MHz, $CDCl_3$) δ 8.09-8.01 (m, 2H), 7.62-7.54 (m, 1H), 7.49-7.43 (m, 2H), 5.11 (d, *J* = 3.5 Hz, 1H), 5.06 (d, *J* = 2.0 Hz, 1H), 2.18-2.09 (m, 1H), 1.72-1.54 (m, 4H), 1.48-1.40 (m, 4H), 1.20 (s, 3H), 1.17 (s, 3H). **^{13}C-NMR** (125 MHz, $CDCl_3$) δ 190.1 (dd, *J* = 33.8, 31.3 Hz), 156.5 (d, *J* = 3.8 Hz), 134.6 (t, *J* = 2.5 Hz), 133.4, 130.0 (t, *J* = 3.8 Hz), 128.3, 121.3 (dd, *J* = 262.5, 256.3

Hz), 112.3 (d, J = 6.3 Hz), 45.4 (dd, J = 20.0, 18.8 Hz), 38.6, 36.2, 33.3, 32.8 (dd, J = 6.3, 3.8 Hz), 31.7, 25.9 (dd, J = 8.8, 3.8 Hz), 17.9. **^{19}F-NMR** (470 MHz, $CDCl_3$) δ -98.12 (d, J = 290.1 Hz), -101.44 (d, J = 290.1 Hz). **HRMS** (ESI) m/z calcd. for $C_{18}H_{23}F_2O^+$ $[M+H]^+$: 293.1711; Found: 293.1709.

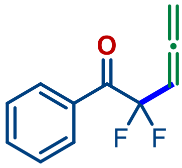

(126) According to the general procedure E, using phenyl trifluoromethyl ketone *N*-triftosylhydrazone (118.8 mg, 0.3 mmol), prop-2-yn-1-ol (33.6 mg, 0.6 mmol), and DCE (4.0 mL) afforded compound **126** (57.6 mg, 99% yield) as a colorless oil; **^{1}H-NMR** (500 MHz, $CDCl_3$) δ 8.08 (d, J = 8.0 Hz, 2H), 7.64 (t, J = 7.5 Hz, 1H), 7.49 (t, J = 7.8 Hz, 2H), 5.71 (tt, J = 9.3, 6.8 Hz, 1H), 5.12 (q, J = 6.5 Hz, 2H). **^{13}C-NMR** (125 MHz, $CDCl_3$) δ 209.4 (t, J = 9.0 Hz), 188.1 (t, J = 30.3 Hz), 134.3, 131.9, 130.2 (t, J = 2.9 Hz), 128.6, 115.3 (t, J = 251.3 Hz), 88.1 (t, J = 30.4 Hz), 81.3. **^{19}F NMR** (470 MHz, $CDCl_3$) δ -94.4 (dt, J = 8.5, 6.8 Hz). **IR** (Film): 3055, 2255, 1983, 1703, 1595, 1453, 1269, 1122, 903, 719, 649 cm^{-1}. **HRMS** (ESI) *m/z* calculated $C_{11}H_8F_2NaO$ 217.0433, found 217.0435.

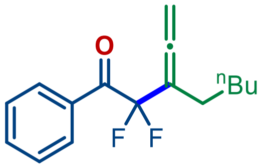

(127) According to the general procedure E, using phenyl trifluoromethyl ketone *N*-triftosylhydrazone (118.8 mg, 0.3 mmol), hept-2-yn-1-ol (67.2 mg, 0.6 mmol), and DCE (4.0 mL) afforded compound **127** (75.2 mg, 95% yield) as a colorless oil; **^{1}H-NMR** (600 MHz, $CDCl_3$) δ 8.03 (d, J = 7.8 Hz, 2H), 7.60 (t, J = 7.4 Hz, 1H), 7.47 (t, J = 7.9 Hz, 2H), 5.03-4.91 (m, 2H), 2.23-2.17 (m, 2H), 1.54-1.47 (m, 2H), 1.35-1.30 (m, 4H), 0.91-0.86 (m, 3H). **^{13}C-NMR** (150 MHz, $CDCl_3$) δ 207.4 (t, J = 8.1 Hz), 188.6 (t, J = 29.1 Hz), 134.0, 132.4, 130.1 (d, J = 2.1 Hz), 128.5, 116.8 (t, J = 252.6 Hz), 101.3 (t, J = 27.9 Hz), 81.8, 31.2, 26.9, 25.0, 22.3, 13.9. **^{19}F NMR** (564 MHz, $CDCl_3$) δ -98.8 (t, J = 6.0 Hz). **IR** (Film): 3055, 2957, 2257, 1961, 1711, 1621, 1450, 1264, 1251, 1130, 902, 711, 647 cm^{-1}. **HRMS** (ESI) *m/z* calculated $C_{16}H_{18}F_2NaO$ 287.1218, found 287.1212.

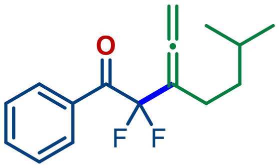

(128) According to the general procedure E, using phenyl trifluoromethyl ketone *N*-triftosylhydrazone (118.8 mg, 0.3 mmol), 5-methylhex-2-yn-1-ol (67.2 mg, 0.6 mmol), and DCE (4.0 mL) afforded compound **128** (76.8 mg, 97% yield) as a colorless oil; **^{1}H-NMR** (600 MHz, $CDCl_3$) δ 8.03 (d, J = 7.7 Hz, 2H), 7.62-7.58 (m, 1H), 7.46 (t, J = 7.8 Hz, 2H), 4.99-4.91 (m, 2H), 2.27-2.14 (m, 2H), 1.67-1.54 (m, 1H), 1.43-1.34 (m, 2H), 0.91 (d, J = 6.7 Hz, 6H). **^{13}C-NMR** (150 MHz, $CDCl_3$) δ 207.3 (t, J = 8.1 Hz), 188.6 (t, J = 29.2 Hz), 134.0, 132.5, 130.1 (t, J = 1.8 Hz), 128.5, 116.9 (t, J = 252.6 Hz), 101.5 (t, J = 27.9 Hz), 81.9, 36.3, 27.6, 23.0, 22.4. **^{19}F NMR** (564 MHz, $CDCl_3$) δ -98.7 (t, J = 5.6 Hz). **IR** (Film): 3055, 2955, 2257, 1955, 1705, 1600, 1451, 1261, 1200, 1131, 906, 725, 649 cm^{-1}. **HRMS** (ESI) *m/z* calculated $C_{16}H_{18}F_2NaO$ 287.1223, found 287.1227.

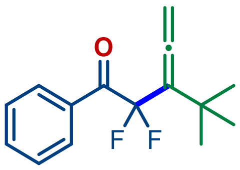

(129) According to the general procedure E, using phenyl trifluoromethyl ketone *N*-triftosylhydrazone (118.8 mg, 0.3 mmol), 4,4-dimethylpent-2-yn-1-ol (67.2 mg, 0.6 mmol), and DCE (4.0 mL) afforded compound **129** (58.5 mg, 78% yield) as a colorless oil; ^{1}**H-NMR** (500 MHz, $CDCl_3$) δ 8.02 (d, *J* = 7.5 Hz, 2H), 7.61-7.57 (m, 1H), 7.49-7.43 (m, 2H), 4.82 (t, *J* = 6.5 Hz, 2H), 1.30 (s, 9H). ^{13}**C-NMR** (125 MHz, $CDCl_3$) δ 208.4 (t, *J* = 10.0 Hz), 188.4 (t, *J* = 29.3 Hz), 133.7, 132.8, 130.1 (t, *J* = 2.3 Hz), 128.4, 118.7 (t, *J* = 254.3 Hz), 108.7 (t, *J* = 26.8 Hz), 81.7, 33.6, 30.4. ^{19}**F NMR** (470 MHz, $CDCl_3$) δ -93.77 (t, *J* = 6.3 Hz). **IR** (Film): 3055, 2969, 2255, 1948, 1707, 1598, 1367, 1267, 1121, 1072, 906, 721, 649 cm^{-1}. **HRMS** (ESI) *m/z* calculated $C_{15}H_{16}F_2NaO$ 273.1061, found 273.1052.

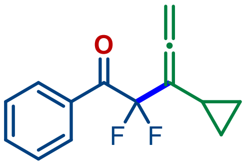

(130) According to the general procedure E, using phenyl trifluoromethyl ketone *N*-triftosylhydrazone (118.8 mg, 0.3 mmol), 3-cyclopropylprop-2-yn-1-ol (57.6 mg, 0.6 mmol), and DCE (4.0 mL) afforded compound **130** (63.9 mg, 91% yield) as a colorless oil; ^{1}**H-NMR** (500 MHz, $CDCl_3$) δ 8.04 (d, *J* = 7.5 Hz, 2H), 7.63-7.58 (m, 1H), 7.50-7.44 (m, 2H), 5.02 (td, *J* = 5.7, 2.5 Hz, 2H), 1.49-1.35 (m, 1H), 0.82-0.73 (m, 2H), 0.49-0.40 (m, 2H). ^{13}**C-NMR** (125 MHz, $CDCl_3$) δ 205.8 (t, *J* = 7.4 Hz), 188.5 (t, *J* = 29.0 Hz), 134.1, 132.4, 130.0 (t, *J* = 2.8 Hz), 128.5, 116.5 (t, *J* = 253.3 Hz), 104.8 (t, *J* = 27.2 Hz), 82.9, 6.7, 6.5. ^{19}**F NMR** (470 MHz, $CDCl_3$) δ -98.5 (t, *J* = 5.3 Hz). **IR** (Film): 3055, 2254, 1951, 1705, 1598, 1449, 1263, 1185, 1134, 903, 719, 649 cm^{-1}. **HRMS** (ESI) *m/z* calculated $C_{14}H_{12}F_2NaO$ 257.0756, found 257.0748.

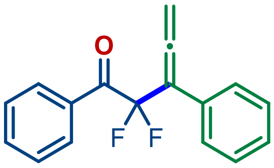

(131) According to the general procedure E, using phenyl trifluoromethyl ketone *N*-triftosylhydrazone (118.8 mg, 0.3 mmol), 3-phenylprop-2-yn-1-ol (79.2 mg, 0.6 mmol), and DCE (4.0 mL) afforded compound **131** (55.1 mg, 68% yield) as a colorless oil; ^{1}**H-NMR** (500 MHz, $CDCl_3$) δ 8.05 (d, *J* = 7.6 Hz, 2H), 7.62-7.57 (m, 1H), 7.51 (d, *J* = 7.6 Hz, 2H), 7.48-7.43 (m, 2H), 7.39-7.34 (m, 2H), 7.32-7.28 (m, 1H), 5.26 (t, *J* = 5.7 Hz, 2H).^{13}**C-NMR** (125 MHz, $CDCl_3$) δ 209.6 (t, *J* = 8.0 Hz), 188.0 (t, *J* = 28.9 Hz), 134.1, 132.3, 130.2 (t, *J* = 2.3 Hz), 129.7, 128.7, 128.6, 128.2, 127.8, 116.6 (t, *J* = 254.5 Hz), 103.7 (t, *J* = 27.7 Hz), 83.1. ^{19}**F NMR** (470 MHz, $CDCl_3$) δ -94.9 (t, *J* = 5.8 Hz). **HRMS** (ESI) *m/z* calculated $C_{17}H_{12}F_2NaO$ 293.0754, found 293.0759.

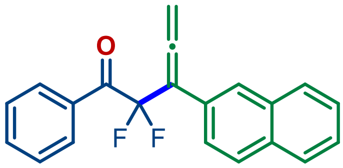

(132) According to the general procedure E, using phenyl trifluoromethyl ketone *N*-triftosylhydrazone (118.8 mg, 0.3 mmol), 3-(naphthalen-2-yl)prop-2-yn-1-ol (109.2 mg, 0.6 mmol), and DCE (4.0 mL) afforded compound **132**

(57.6 mg, 60% yield) as a colorless oil; **^{1}H-NMR** (600 MHz, $CDCl_3$) δ 8.08 (d, *J* = 7.9 Hz, 2H), 8.01 (s, 1H), 7.85-7.78 (m, 3H), 7.61-7.55 (m, 2H), 7.50-7.43 (m, 4H), 5.34 (t, *J* = 5.5 Hz, 2H). **^{13}C-NMR** (150 MHz, $CDCl_3$) δ 210.0 (t, *J* = 7.9 Hz), 188.0 (t, *J* = 28.9 Hz), 134.1, 133.3, 132.8, 132.3, 130.2, 128.6, 128.32, 128.31, 127.5, 126.91, 126.85, 126.5, 126.4, 125.4, 116.6 (t, *J* = 254.6 Hz), 104.03 (t, *J* = 27.7 Hz), 83.5. **^{19}F NMR** (564 MHz, $CDCl_3$) δ -94.63 (t, *J* = 6.0 Hz). **IR** (Film): 3055, 2985, 2253, 1940, 1707, 1263, 1128, 903, 717, 649 cm^{-1}. **HRMS** (ESI) *m/z* calculated $C_{21}H_{14}F_2NaO$ 343.0905, found 343.0897.

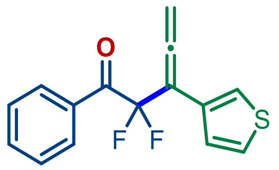

(133) According to the general procedure E, using phenyl trifluoromethyl ketone *N*-triftosylhydrazone (118.8 mg, 0.3 mmol), 3-(thiophen-3-yl)prop-2-yn-1-ol (82.8 mg, 0.6 mmol), and DCE (4.0 mL) afforded compound **133** (62.1 mg, 75% yield) as a light yellow oil; **^{1}H-NMR** (500 MHz, $CDCl_3$) δ 8.06 (d, *J* = 7.5 Hz, 2H), 7.64-7.58 (m, 1H), 7.50-7.43 (m, 3H), 7.30 (dd, *J* = 5.0, 3.0 Hz, 1H), 7.12 (d, *J* = 5.0 Hz, 1H), 5.29 (t, *J* = 5.0 Hz, 2H). **^{13}C-NMR** (125 MHz, $CDCl_3$) δ 209.2 (t, *J* = 7.6 Hz), 188.0 (t, *J* = 28.9 Hz), 134.2, 132.2, 130.2 (t, *J* = 2.7 Hz), 129.2, 128.6, 127.1, 125.7, 123.0, 116.2 (t, *J* = 254.4 Hz), 100.3 (t, *J* = 28.3 Hz), 83.4. **^{19}F NMR** (470 MHz, $CDCl_3$) δ -96.4 (t, *J* = 5.1 Hz). **IR** (Film): 3055, 2987, 2257, 1938, 1706, 1265, 1134, 903, 740, 717, 649 cm^{-1}. **HRMS** (ESI) *m/z* calculated $C_{15}H_{10}F_2NaOS$ 299.0313, found 299.0316.

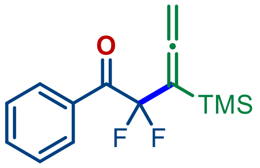

(134) According to the general procedure E, using phenyl trifluoromethyl ketone *N*-triftosylhydrazone (118.8 mg, 0.3 mmol), 3-(trimethylsilyl)prop-2-yn-1-ol (76.8 mg, 0.6 mmol), and DCE (4.0 mL) afforded compound **134** (64.6 mg, 81% yield) as a colorless oil; **^{1}H-NMR** (500 MHz, $CDCl_3$) δ 8.02 (d, *J* = 7.5 Hz, 2H), 7.61 (t, *J* = 7.5 Hz, 1H), 7.46 (t, *J* = 7.5 Hz, 2H), 4.59 (t, *J* = 6.5 Hz, 2H), 0.28 (s, 9H). **^{13}C-NMR** (125 MHz, $CDCl_3$) δ 211.7 (t, *J* = 13.5 Hz), 188.6 (t, *J* = 30.1 Hz), 133.9, 132.6, 130.2 (t, *J* = 2.4 Hz), 128.4, 118.8 (t, *J* = 249.8 Hz), 95.2 (t, *J* = 35.9 Hz), 74.0, -0.8. **^{19}F NMR** (470 MHz, $CDCl_3$) δ -89.5 (t, *J* = 6.4 Hz). **IR** (Film): 3055, 2957, 2261, 1930, 1704, 1598, 1449, 1263, 1251, 1227, 1155, 946, 904, 844, 722, 649 cm^{-1}. **HRMS** (ESI) *m/z* calculated $C_{14}H_{16}F_2NaOSi$ 289.0831, found 289.0839.

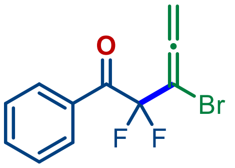

(135) According to the general procedure E, using phenyl trifluoromethyl ketone *N*-triftosylhydrazone (118.8 mg, 0.3 mmol), 3-bromoprop-2-yn-1-ol (79.8 mg, 0.6 mmol), and DCE (4.0 mL) afforded compound **135** (38.2 mg, 47% yield) as a colorless oil; **^{1}H-NMR** (500 MHz, $CDCl_3$) δ 8.04 (d, *J* = 8.0 Hz, 2H), 7.65 (t, *J* = 7.5 Hz, 1H), 7.50 (t, *J* = 8.0 Hz, 2H), 5.14 (t, *J* = 5.0 Hz, 2H). **^{13}C-NMR** (150 MHz, $CDCl_3$) δ 206.2 (t, *J* = 6.4 Hz), 186.1 (t, *J* = 28.4 Hz), 134.6, 131.8, 130.1 (t, *J* = 3.3 Hz), 128.7, 113.0 (t, *J* = 254.1 Hz), 87.2, 84.6 (t, *J* = 34.7 Hz). **^{19}F NMR** (564 MHz, $CDCl_3$) δ -95.4 (t, *J* = 5.2 Hz). **IR** (Film): 3055, 2257, 1975, 1709, 1597, 1449, 1261, 1096, 905, 725, 649 cm^{-1}. **HRMS** (ESI) *m/z* calculated $C_{11}H_7BrF_2NaO$ 294.9541, found 294.9530.

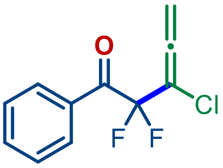

(136) According to the general procedure E, using phenyl trifluoromethyl ketone *N*-triftosylhydrazone (118.8 mg, 0.3 mmol), 3-chloroprop-2-yn-1-ol (53.4 mg, 0.6 mmol), and DCE (4.0 mL) afforded compound **136** (43.8 mg, 64% yield) as a colorless oil; **^{1}H-NMR** (500 MHz, $CDCl_3$) δ 8.05 (d, *J* = 8.2 Hz, 2H), 7.65 (t, *J* = 7.4 Hz, 1H), 7.50 (t, *J* = 7.8 Hz, 2H), 5.43 (t, *J* = 4.9 Hz, 2H). **^{13}C-NMR** (150 MHz, $CDCl_3$) δ 205.7 (t, *J* = 5.3 Hz), 186.3 (t, *J* = 28.7 Hz), 134.6, 131.8, 130.1 (t, *J* = 2.8 Hz), 128.7, 113.3 (t, *J* = 256.2 Hz), 99.1 (t, *J* = 33.4 Hz), 89.3. **^{19}F NMR** (470 MHz, $CDCl_3$) δ -98.49 (t, *J* = 4.2 Hz). **HRMS** (ESI) *m/z* calculated $C_{11}H_7ClF_2NaO$ 251.0051, found 251.0053.

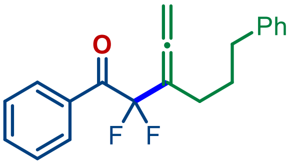

(137) According to the general procedure E, using phenyl trifluoromethyl ketone *N*-triftosylhydrazone (118.8 mg, 0.3 mmol), 6-phenylhex-2-yn-1-ol (104.4 mg, 0.6 mmol), and DCE (4.0 mL) afforded compound **137** (79.6 mg, 85% yield) as a colorless oil; **^{1}H-NMR** (500 MHz, $CDCl_3$) δ 8.02 (d, *J* = 7.5 Hz, 2H), 7.61-7.56 (m, 1H), 7.48-7.42 (m, 2H), 7.29-7.24 (m, 2H), 7.19-7.14 (m, 3H), 5.01-4.91 (m, 2H), 2.70-2.63 (m, 2H), 2.30-2.22 (m, 2H), 1.88-1.77 (m, 2H). **^{13}C-NMR** (125 MHz, $CDCl_3$) δ 207.3 (t, *J* = 8.0 Hz), 188.4 (t, *J* = 29.3 Hz), 141.7, 134.0, 132.4, 130.1 (t, *J* = 2.3 Hz), 128.5, 128.4, 128.3, 125.8, 116.8 (t, *J* = 252.8 Hz), 101.0 (t, *J* = 27.9 Hz), 82.1, 35.2, 29.1, 24.7. **^{19}F NMR** (470 MHz, $CDCl_3$) δ -98.6 (t, *J* = 5.7 Hz). **IR** (Film): 3055, 2936, 2253, 1953, 1704, 1598, 1496, 1460, 1265, 1201, 1131, 1074, 904, 723, 649 cm^{-1}. **HRMS** (ESI) *m/z* calculated $C_{20}H_{18}F_2NaO$ 335.1218, found 335.1214.

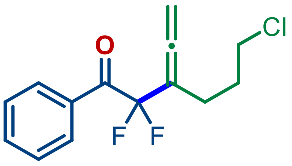

(138) According to the general procedure E, using phenyl trifluoromethyl ketone *N*-triftosylhydrazone (118.8 mg, 0.3 mmol), 6-chlorohex-2-yn-1-ol (79.2 mg, 0.6 mmol), and DCE (4.0 mL) afforded compound **138** (67.2mg, 83% yield) as a colorless oil; **^{1}H-NMR** (600 MHz, $CDCl_3$) δ 8.03 (d, *J* = 7.9 Hz, 2H), 7.62 (t, *J* = 7.4 Hz, 1H), 7.48 (t, *J* = 7.8 Hz, 2H), 5.11-4.95 (m, 2H), 3.59 (t, *J* = 6.5 Hz, 2H), 2.46-2.32 (m, 2H), 2.11-1.94 (m, 2H). **^{13}C-NMR** (150 MHz, $CDCl_3$) δ 207.1 (t, *J* = 8.0 Hz), 188.3 (t, *J* = 29.1 Hz), 134.2, 132.3, 130.1 (t, *J* = 2.6 Hz), 128.6, 116.6 (t, *J* = 253.4 Hz), 100.0 (t, *J* = 28.2 Hz), 82.5, 43.9, 30.2, 22.7. **^{19}F NMR** (564 MHz, $CDCl_3$) δ -98.52 (t, *J* = 5.4 Hz). **IR** (Film): 3055, 2959, 2255, 1953, 1704, 1598, 1449, 1265, 1132, 1075, 904, 720, 685, 649 cm^{-1}. **HRMS** (ESI) *m/z* calculated $C_{14}H_{13}ClF_2NaO$ 293.0518, found 293.0515.

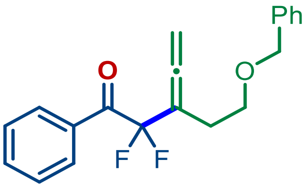

(139) According to the general procedure E, using phenyl trifluoromethyl ketone *N*-triftosylhydrazone (118.8 mg, 0.3 mmol), 5-(benzyloxy)pent-2-yn-1-ol (114.0 mg, 0.6 mmol), and DCE (4.0 mL) afforded compound **139**

(70.8mg, 72% yield) as a colorless oil; **^{1}H-NMR** (500 MHz, $CDCl_3$) δ 8.03 (d, *J* = 7.5 Hz, 2H), 7.62-7.57 (m, 1H), 7.44 (t, *J* = 7.5 Hz, 2H), 7.36-7.26 (m, 5H), 5.02-4.89 (m, 2H), 4.50 (s, 2H), 3.66 (t, *J* = 7.0 Hz, 2H), 2.63-2.53 (m, 2H). **^{13}C-NMR** (125 MHz, $CDCl_3$) δ 207.7 (t, *J* = 8.3 Hz), 188.3 (t, *J* = 29.3 Hz), 138.2, 134.1, 132.4, 130.2 (t, *J* = 2.7 Hz), 128.5, 128.3, 127.57, 127.56, 116.7 (t, *J* = 252.8 Hz), 98.1 (t, *J* = 28.4 Hz), 81.9, 72.9, 67.9, 25.9. **^{19}F NMR** (470 MHz, $CDCl_3$) δ -98.4 (t, *J* = 6.0 Hz). **IR** (Film): 3057, 2864, 2253, 1954, 1705, 1598, 1450, 1263, 1202, 1067, 905, 723, 649 cm^{-1}. **HRMS** (ESI) *m/z* calculated $C_{20}H_{18}F_2NaO_2$ 351.1167, found 351.1177.

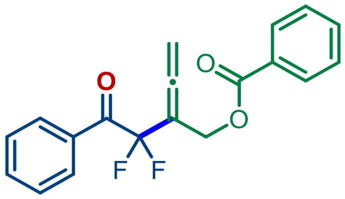

(140) According to the general procedure E, using phenyl trifluoromethyl ketone *N*-triftosylhydrazone (118.8 mg, 0.3 mmol), 4-hydroxybut-2-yn-1-yl benzoate (114.0 mg, 0.6 mmol), and DCE (4.0 mL) afforded compound **140** (75.8mg, 77% yield) as a colorless oil; **^{1}H-NMR** (600 MHz, $CDCl_3$) δ 8.05 (d, *J* = 7.7 Hz, 2H), 7.96-7.93 (m, 2H), 7.60 (t, *J* = 7.4 Hz, 1H), 7.57-7.53 (m, 1H), 7.45 (t, *J* = 7.9 Hz, 2H), 7.40 (t, *J* = 7.8 Hz, 2H), 5.23-5.19 (m, 2H), 5.08 (t, *J* = 2.0 Hz, 2H). **^{13}C NMR** (150 MHz, $CDCl_3$) δ 208.9 (t, *J* = 7.2 Hz), 187.6 (t, *J* = 29.3 Hz), 165.8, 134.3, 133.1, 132.0, 130.2, 129.7, 129.5, 128.6, 128.3, 115.7 (t, *J* = 254.4 Hz), 97.6 (t, *J* = 28.3 Hz), 82.6, 18.4 (t, *J* = 644.0 Hz). **^{19}F NMR** (564 MHz, $CDCl_3$) δ -98.18 (t, *J* = 5.2 Hz). **HRMS** (ESI) *m/z* calculated $C_{19}H_{14}F_2NaO_3$ 351.0811, found 351.0817.

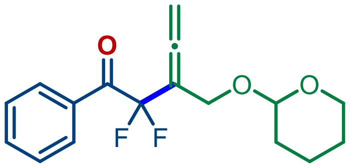

(141) According to the general procedure E, using phenyl trifluoromethyl ketone *N*-triftosylhydrazone (118.8 mg, 0.3 mmol), 4-((tetrahydro-2*H*-pyran-2-yl)oxy)but-2-yn-1-ol (102.0 mg, 0.6 mmol), and DCE (4.0 mL) afforded compound **141** (50.8 mg, 55% yield) as a colorless oil; **^{1}H-NMR** (600 MHz, $CDCl_3$) δ 8.06 (d, *J* = 7.8 Hz, 2H), 7.61 (t, *J* = 7.4 Hz, 1H), 7.47 (t, *J* = 7.8 Hz, 2H), 5.18-5.12 (m, 2H), 4.64 (t, *J* = 3.2 Hz, 1H), 4.45 (dt, *J* = 12.0, 2.2 Hz, 1H), 4.21 (dt, *J* = 12.0, 1.9 Hz, 1H), 3.83-3.75 (m, 1H), 3.53-3.46 (m, 1H), 1.72-1.63 (m, 1H), 1.62-1.51 (m, 2H), 1.50-1.40 (m, 3H). **^{13}C-NMR** (150 MHz, $CDCl_3$) δ 208.3 (t, *J* = 7.4 Hz), 187.8 (t, *J* = 29.1 Hz), 134.0, 132.3, 130.1 (d, *J* = 2.1 Hz), 128.5, 115.7 (t, *J* = 253.8 Hz), 98.8 (t, *J* = 27.6 Hz), 97.6, 81.7, 62.5, 61.8, 29.9, 25.3, 18.8. **^{19}F NMR** (564 MHz, $CDCl_3$) δ -99.0 (t, *J* = 4.4 Hz). **IR** (Film): 3055, 2944, 2255, 1981, 1707, 1263, 1025, 905, 721, 649 cm^{-1}. **HRMS** (ESI) *m/z* calculated $C_{17}H_{18}F_2NaO_3$ 331.1116, found 331.1128.

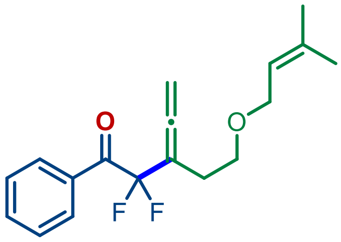

(142) According to the general procedure E, using phenyl trifluoromethyl ketone *N*-triftosylhydrazone (118.8 mg, 0.3 mmol), 5-((3-methylbut-2-en-1-yl)oxy)pent-2-yn-1-ol (100.8 mg, 0.6 mmol), and DCE (4.0 mL) afforded compound **142** (67.9 mg, 74% yield) as a colorless oil; **^{1}H-NMR** (600 MHz, $CDCl_3$) δ 8.04 (d, *J* = 7.7 Hz, 2H), 7.63-7.58 (m, 1H), 7.50-7.44 (m, 2H), 5.35-5.23 (m, 1H), 5.03-4.93 (m, 2H), 3.95 (d, *J* = 6.9 Hz, 2H), 3.59 (t, *J* = 6.9 Hz, 2H), 2.58-2.48 (m, 2H), 1.74 (s, 3H), 1.66 (s, 3H). **^{13}C-NMR** (150 MHz, $CDCl_3$) δ 207.7 (t, *J* = 8.2 Hz),

188.3 (t, J = 29.1 Hz), 136.9, 134.0, 132.4, 130.2 (t, J = 2.1 Hz), 128.5, 120.9, 116.7 (t, J = 253.0 Hz), 98.2 (t, J = 28.1 Hz), 81.8, 67.6, 67.2, 25.9, 25.8, 17.9. **^{19}F NMR** (564 MHz, $CDCl_3$) δ -98.4 (t, J = 5.6 Hz). **IR** (Film): 3055, 2961, 2255, 1951, 1706, 1448, 1263, 1076, 906, 725, 649 cm^{-1}. **HRMS** (ESI) *m/z* calculated $C_{18}H_{20}F_2NaO_2$ 329.1324, found 329.1333.

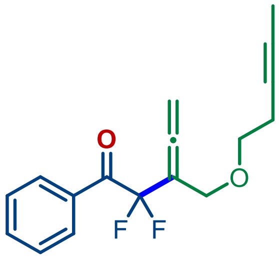

(143) According to the general procedure E, using phenyl trifluoromethyl ketone *N*-triftosylhydrazone (118.8 mg, 0.3 mmol), 4-(pent-3-yn-1-yloxy)but-2-yn-1-ol (91.2 mg, 0.6 mmol), and DCE (4.0 mL) afforded compound **143** (73.9 mg, 85% yield) as a colorless oil; **^{1}H NMR** (600 MHz, $CDCl_3$) δ 8.05 (d, J = 7.7 Hz, 2H), 7.61 (t, J = 10.6 Hz, 1H), 7.48 (t, J = 7.9 Hz, 2H), 5.18-5.13 (m, 2H), 4.26 (t, J = 2.1 Hz, 2H), 3.48 (t, J = 7.2 Hz, 2H), 2.32-2.28 (m, 2H), 1.75 (t, J = 2.5 Hz, 3H). **^{13}C NMR** (150 MHz, $CDCl_3$) δ 208.2 (t, J = 7.5 Hz), 187.8 (t, J = 29.2 Hz), 134.1, 132.3, 130.1 (t, J = 2.6 Hz), 128.5, 115.8 (t, J = 254.0 Hz), 98.5 (t, J = 27.7 Hz), 81.9, 76.8, 75.4, 69.0, 66.5, 19.7. **^{19}F NMR** (564 MHz, $CDCl_3$) δ (-97.28)-(-101.72) (m). **HRMS** (ESI) *m/z* calculated $C_{17}H_{16}F_2NaO_2$ 313.1018, found 313.1011.

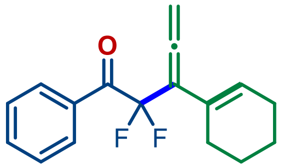

(144) According to the general procedure E, using phenyl trifluoromethyl ketone *N*-triftosylhydrazone (118.8 mg, 0.3 mmol), 3-(cyclohex-1-en-1-yl)prop-2-yn-1-ol (81.6 mg, 0.6 mmol), and DCE (4.0 mL) afforded compound **144** (65.8 mg, 80% yield) as a colorless oil; **^{1}H-NMR** (500 MHz, $CDCl_3$) δ 8.03 (d, J = 8.0 Hz, 2H), 7.59 (t, J = 7.5 Hz, 1H), 7.46 (t, J = 8.0 Hz, 2H), 6.08-6.03 (m, 1H), 5.15 (t, J = 5.0 Hz, 2H), 2.15-2.09 (m, 2H), 2.06-2.02 (m, 2H), 1.67-1.62 (m, 2H), 1.59-1.52 (m, 2H). **^{13}C-NMR** (125 MHz, $CDCl_3$) δ 209.0 (t, J = 7.5 Hz), 188.4 (t, J = 29.2 Hz), 133.9, 132.5, 130.0 (t, J = 2.3 Hz), 128.4, 128.1, 125.9, 116.3 (t, J = 254.7 Hz), 105.7 (t, J = 26.5 Hz), 83.1, 27.3, 25.8, 22.5, 21.7. **^{19}F NMR** (470 MHz, $CDCl_3$) δ -94.8 (t, J = 3.7 Hz). **HRMS** (ESI) *m/z* calculated $C_{17}H_{16}F_2NaO$ 297.1067, found 297.1064.

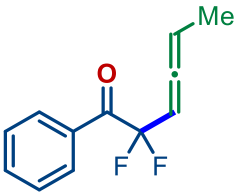

(145) According to the general procedure E, using phenyl trifluoromethyl ketone *N*-triftosylhydrazone (118.8 mg, 0.3 mmol), but-3-yn-2-ol (42.0 mg, 0.6 mmol), and DCE (4.0 mL) afforded compound **145** (44.3 mg, 71% yield) as a colorless oil; **^{1}H-NMR** (500 MHz, $CDCl_3$) 8.07 (d, J = 7.5 Hz, 2H), 7.62 (t, J = 7.5 Hz, 1H), 7.49 (t, J = 8.0 Hz, 2H), 5.66-5.59 (m, 1H), 5.50-5.42 (m, 1H), 1.55 (dd, J = 7.5, 3.0 Hz, 3H). **^{13}C-NMR** (125 MHz, $CDCl_3$) δ 206.6 (t, J = 9.5 Hz), 188.4 (t, J = 30.0 Hz), 134.1, 132.1, 130.2 (t, J = 3.0 Hz), 128.5, 115.6 (t, J = 250.0 Hz), 92.6, 88.1 (t, J = 31.0 Hz), 12.7 (t, J = 2.4 Hz). **^{19}F NMR** (470 MHz, $CDCl_3$) δ (-92.41)-(-95.88) (m). **IR** (Film):

3055, 2253, 1970, 1704, 1598, 1449, 1265, 1126, 1077, 903, 722, 649 cm^{-1}. **HRMS** (ESI) *m/z* calculated $C_{12}H_{10}F_2NaO$ 231.0586, found 231.0592.

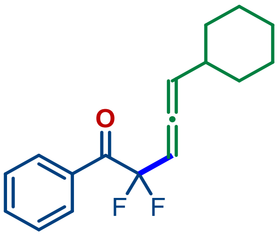

(146) According to the general procedure E, using phenyl trifluoromethyl ketone *N*-triftosylhydrazone (118.8 mg, 0.3 mmol), 1-cyclohexylprop-2-yn-1-ol (82.8 mg, 0.6 mmol), and DCE (4.0 mL) afforded compound **146** (60.4 mg, 73% yield) as a colorless oil; **^{1}H-NMR** (500 MHz, $CDCl_3$) 8.08 (d, *J* = 8.0 Hz, 2H), 7.62 (t, *J* = 7.4 Hz, 1H), 7.48 (t, *J* = 8.0 Hz, 2H), 5.71-5.66 (m, 1H), 5.46-5.41 (m, 1H), 1.96-1.88 (m, 1H), 1.67-1.52 (m, 5H), 1.26-1.04 (m, 3H), 1.00-0.91 (m, 2H). **^{13}C-NMR** (125 MHz, $CDCl_3$) δ 204.9 (t, *J* = 10.0 Hz), 188.4 (t, *J* = 29.6 Hz), 134.1, 132.2, 130.3 (t, *J* = 2.8 Hz), 128.5, 115.7 (t, *J* = 248.8 Hz), 103.5, 89.3 (t, *J* = 30.8 Hz), 36.8, 32.4, 32.3, 25.8. **^{19}F NMR** (470 MHz, $CDCl_3$) δ (-92.72)-(-95.25) (m). **IR** (Film): 3055, 2928, 2253, 1967, 1704, 1449, 1265, 1120, 903, 718, 649 cm^{-1}. **HRMS** (ESI) *m/z* calculated $C_{17}H_{18}F_2NaO$ 299.1225, found 299.1218.

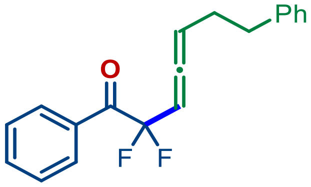

(147) According to the general procedure E, using phenyl trifluoromethyl ketone *N*-triftosylhydrazone (118.8 mg, 0.3 mmol), 5-phenylpent-1-yn-3-ol (96.0 mg, 0.6 mmol), and DCE (4.0 mL) afforded compound **147** (72.4 mg, 81% yield) as a colorless oil; **^{1}H-NMR** (500 MHz, $CDCl_3$) 8.08 (d, *J* = 7.5 Hz, 2H), 7.62 (t, *J* = 7.5 Hz, 1H), 7.49 (t, *J* = 8.0 Hz, 2H), 7.25 (t, *J* = 7.5 Hz, 2H), 7.18 (t, *J* = 7.5 Hz, 1H), 7.08 (d, *J* = 7.0 Hz, 2H), 5.70-5.64 (m, 1H), 5.54-5.48 (m, 1H), 2.60-2.50 (m, 2H), 2.26-2.21 (m, 2H). **^{13}C-NMR** (125 MHz, $CDCl_3$) δ 205.8 (t, *J* = 9.6 Hz), 188.4 (t, *J* = 29.4 Hz), 140.7, 134.2, 132.1, 130.3 (t, *J* = 2.6 Hz), 128.6, 128.36, 128.35, 126.1, 115.6 (t, *J* = 249.1 Hz), 97.1, 89.0 (t, *J* = 30.9 Hz), 34.8, 29.2. **^{19}F NMR** (470 MHz, $CDCl_3$) δ -94.0 (t, *J* = 6.1 Hz). **IR** (Film): 3055, 2253, 1973, 1704, 1598, 1422, 1263, 1123, 902, 721, 649 cm^{-1}. **HRMS** (ESI) *m/z* calculated $C_{19}H_{16}F_2NaO$ 321.1059, found 321.1061.

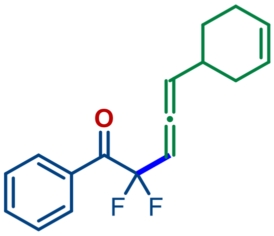

(148) According to the general procedure E, using phenyl trifluoromethyl ketone *N*-triftosylhydrazone (118.8 mg, 0.3 mmol), 1-(cyclohex-3-en-1-yl)prop-2-yn-1-ol (81.6 mg, 0.6 mmol), and DCE (4.0 mL) afforded compound **148** (60.8 mg, 79% yield) as a colorless oil; **^{1}H-NMR** (600 MHz, $CDCl_3$) δ 8.08 (d, *J* = 7.9 Hz, 2H), 7.64-7.59 (m, 1H), 7.51-7.45 (m, 2H), 5.75-5.68 (m, 1H), 5.65-5.60 (m, 1H), 5.60-5.54 (m, 1H), 5.54-5.49 (m, 1H), 2.29-2.18 (m, 1H), 2.06-1.93 (m, 3H), 1.78-1.65 (m, 1H), 1.64-1.52 (m, 1H), 1.32-1.19 (m, 1H). **^{13}C NMR** (150 MHz, $CDCl_3$) δ 205.0 (t, *J* = 6.9 Hz), 188.4 (t, *J* = 29.1 Hz), 134.2, 132.2, 130.3 (t, *J* = 2.3 Hz), 128.6 126.7, 125.4, 115.6 (t, *J* = 250.2 Hz), 102.7, 89.6 (td, *J* = 30.9 Hz), 32.8 (d, *J* = 16.2 Hz), 30.7 (d, *J* = 4.3 Hz), 28.2 (d, *J* = 5.9

Hz), 24.5 (d, J = 3.7 Hz). **^{19}F NMR** (564 MHz, $CDCl_3$) δ (-92.88)-(-94.68) (m). **HRMS** (ESI) *m/z* calculated $C_{17}H_{16}FNaO$ 297.1067, found 297.1064.

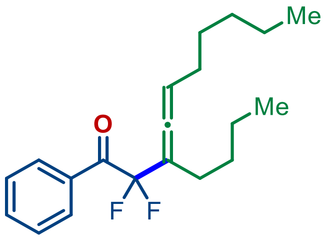

(149) According to the general procedure E, using phenyl trifluoromethyl ketone *N*-triftosylhydrazone (118.8 mg, 0.3 mmol), dodec-7-yn-6-ol (109.2 mg, 0.6 mmol), and DCE (4.0 mL) afforded compound **149** (65.3 mg, 68% yield) as a colorless oil; **^{1}H-NMR** (600 MHz, $CDCl_3$) 8.03 (d, J = 7.8 Hz, 2H), 7.60-7.57 (m, 1H), 7.45 (t, J = 7.8 Hz, 2H), 5.33-5.28 (m, 1H), 2.25-2.21 (m, 2H), 1.79-1.72 (m, 2H), 1.50-1.45 (m, 2H), 1.41-1.35 (m, 2H), 1.21-1.18 (m, 2H), 1.16-1.09 (m, 4H), 0.92 (t, J = 7.2 Hz, 3H), 0.83 (t, J = 7.2 Hz, 3H). **^{13}C NMR** (150 MHz, $CDCl_3$) δ 203.5 (t, J = 8.4 Hz), 188.9 (t, J = 28.8 Hz), 133.9, 132.6, 130.2, 128.4, 117.2 (t, J = 252.0 Hz), 101.4 (t, J = 28.2 Hz), 98.5, 31.1, 29.6, 28.4, 27.9, 25.1, 22.3, 22.2, 13.9, 13.8. **^{19}F NMR** (564 MHz, $CDCl_3$) δ (-97.8)-(-98.9) (m). **IR** (Film): 3055, 2930, 2255, 1969, 1705, 1422, 1263, 1133, 905, 724, 649 cm^{-1}. **HRMS** (ESI) *m/z* calculated $C_{20}H_{26}F_2NaO$ 343.1844, found 343.1845.

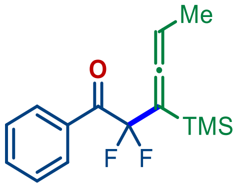

(150) According to the general procedure E, using phenyl trifluoromethyl ketone *N*-triftosylhydrazone (118.8 mg, 0.3 mmol), 4-(trimethylsilyl)but-3-yn-2-ol (85.2 mg, 0.6 mmol), and DCE (4.0 mL) afforded compound **150** (68.0 mg, 81% yield) as a colorless oil; **^{1}H-NMR** (500 MHz, $CDCl_3$) 8.02 (d, J = 7.5 Hz, 2H), 7.60-7.57 (m, 1H), 7.48-7.45 (m, 2H), 5.02-4.95 (m, 1H), 1.33 (d, J = 7.0 Hz, 3H), 0.26 (s, 9H). **^{13}C NMR** (125 MHz, $CDCl_3$) δ 210.4 (t, J = 12.9 Hz), 189.1 (t, J = 29.9 Hz), 133.7, 132.6, 130.3 (t, J = 2.4 Hz), 128.3, 118.9 (t, J = 249.1 Hz), 96.2 (t, J = 36.4 Hz), 85.4, 11.8, -0.7. **^{19}F NMR** (470 MHz, $CDCl_3$) δ (-88.87)-(-90.40) (m). **IR** (Film): 3057, 2253, 1945, 1700, 1610, 1383, 1265, 1197, 1053, 904, 722, 649 cm^{-1}. **HRMS** (ESI) *m/z* calculated $C_{12}H_{19}F_3NaO_2Si$ 303.0999, found 303.0993.

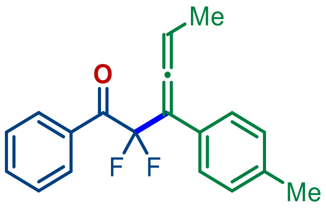

(151) According to the general procedure E, using phenyl trifluoromethyl ketone *N*-triftosylhydrazone (118.8 mg, 0.3 mmol), 4-(*p*-tolyl)but-3-yn-2-ol (96.0 mg, 0.6 mmol), and DCE (4.0 mL) afforded compound **151** (74.2 mg, 83% yield) as a colorless oil; **^{1}H-NMR** (600 MHz, $CDCl_3$) 8.06 (d, J = 7.8 Hz, 2H), 7.59-7.56 (m, 1H), 7.46-7.44 (m, 2H), 7.41 (d, J = 8.4 Hz, 2H), 7.17 (d, J = 8.4 Hz, 2H), 5.61-5.56 (m, 1H), 2.35 (s, 3H), 1.44 (d, J = 7.8 Hz, 3H).**^{13}C-NMR** (150 MHz, $CDCl_3$) δ 206.5 (t, J = 8.0 Hz), 188.3 (t, J = 28.3 Hz), 137.9, 133.9, 132.4, 130.2, 129.3, 128.4, 127.7, 127.6, 116.9 (t, J = 252.2 Hz), 103.3 (t, J = 27.6 Hz), 94.4, 21.1, 12.6. **^{19}F NMR** (564 MHz, $CDCl_3$) δ -94.8 (qd, J = 263.6, 4.9 Hz). **IR** (Film): 3055, 2924, 2257, 1953, 1706, 1598, 1513, 1449, 1267, 1133, 904, 722,649 cm^{-1}. **HRMS** (ESI) *m/z* calculated $C_{19}H_{16}F_2NaO$ 321.1061, found 321.1068.

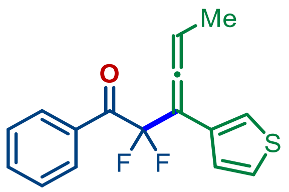

(152) According to the general procedure E, using phenyl trifluoromethyl ketone *N*-triftosylhydrazone (118.8 mg, 0.3 mmol), 4-(thiophen-3-yl)but-3-yn-2-ol (91.2 mg, 0.6 mmol), and DCE (4.0 mL) afforded compound **152** (45.2 mg, 52% yield) as a light yellow oil; **^{1}H-NMR** (500 MHz, $CDCl_3$) δ 8.08 (d, J = 7.8 Hz, 2H), 7.60 (t, J = 7.4 Hz, 1H), 7.49-7.44 (m, 3H), 7.30 (dd, J = 5.0, 2.9 Hz, 1H), 7.11 (d, J = 5.0 Hz, 1H), 5.69-5.57 (m, 1H), 1.49 (d, J = 7.3 Hz, 3H). **^{13}C NMR** (125 MHz, $CDCl_3$) δ 205.9 (t, J = 8.0 Hz), 188.2 (t, J = 28.4 Hz), 134.0, 132.3, 130.3, 130.2 (t, J = 2.7 Hz), 128.5, 127.2, 125.5, 122.7 (d, J = 2.5 Hz), 116.6 (t, J = 254.1 Hz), 99.9 (t, J = 28.5 Hz), 94.7, 12.7. **^{19}F NMR** (470 MHz, $CDCl_3$) δ -96.3 (qd, J = 262.7, 4.5 Hz). **IR** (Film): 3055, 2253, 2001, 1708, 1598, 1265, 1134, 1076, 905, 725, 649 cm^{-1}. **HRMS** (ESI) *m/z* calculated $C_{16}H_{12}F_2NaOS$ 313.0477, found 313.0469.

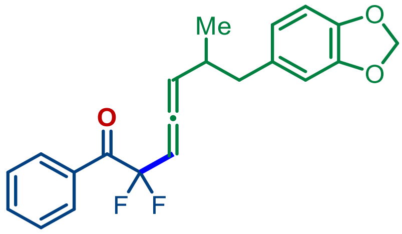

(153) According to the general procedure E, using phenyl trifluoromethyl ketone *N*-triftosylhydrazone (118.8 mg, 0.3 mmol), 5-(benzo[d][1,3]dioxol-5-yl)-4-methylpent-1-yn-3-ol (130.8 mg, 0.6 mmol), and DCE (4.0 mL) afforded compound **153** (57.7 mg, 54% yield). **^{1}H-NMR** (600 MHz, $CDCl_3$) **major:** δ 8.07 (d, J = 7.8 Hz, 2H), 7.65-7.60 (m, 1H), 7.51-7.46 (m, 2H), 6.70-6.67 (m, 1H), 6.53-6.45 (m, 2H), 5.89 (s, 2H), 5.73-5.64 (m, 1H), 5.52-5.48(m, 1H), 2.50-2.47 (m, 1H), 2.41-2.31 (m, 2H), 0.90 (d, J = 6.4 Hz, 3H). **minor:** δ 8.07 (d, J = 7.8 Hz, 2H), 7.65-7.60 (m, 1H), 7.51-7.46 (m, 2H), 6.70-6.67 (m, 1H), 6.53-6.45 (m, 2H), 5.90 (s, 2H), 5.73-5.64 (m, 1H), 5.46-5.43 (m, 1H), 2.50-2.47 (m, 1H), 2.41-2.31 (m, 2H), 0.87 (d, J = 6.7 Hz, 3H). **^{13}C NMR** (150 MHz, $CDCl_3$) **major:** δ 204.7 (d, J = 9.4 Hz), 188.4 (t, J = 29.6 Hz), 147.5, 145.8, 134.2, 133.3, 132.1, 130.3 (t, J = 3.0 Hz), 128.6, 121.9, 115.6 (t, J = 250.8 Hz), 109.3, 108.0, 103.0, 100.8, 89.8 (t, J = 30.7 Hz), 42.5, 34.6, 19.0. **^{19}F NMR** (564 MHz, $CDCl_3$) δ (-93.07)-(-94.57) (m). **IR** (Film): 3055, 2963, 2255, 1967, 1704, 1489, 1263, 1040, 905, 722, 649 cm^{-1}. **HRMS** (ESI) *m/z* calculated $C_{21}H_{18}F_2NaO_3$ 379.1116, found 379.1122.

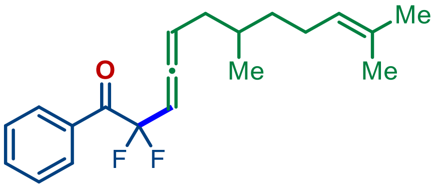

(154) According to the general procedure E, using phenyl trifluoromethyl ketone *N*-triftosylhydrazone (118.8 mg, 0.3 mmol), 5,9-dimethyldec-8-en-1-yn-3-ol (108.0 mg, 0.6 mmol), and DCE (4.0 mL) afforded compound **154** (71.6 mg, 75% yield). **^{1}H-NMR** (500 MHz, $CDCl_3$) δ 8.08 (d, J = 8.0 Hz, 2H), 7.62 (t, J = 8.0 Hz, 1H), 7.48 (t, J = 8.0 Hz, 2H), 5.68-5.59 (m, 1H), 5.48-5.40 (m, 1H), 5.07-5.01 (m, 1H), 2.01-1.85 (m, 3H), 1.83-1.73 (m, 1H), 1.67 (s, 3H), 1.58 (s, 3H), 1.47-1.36 (m, 1H), 1.31-1.21 (m, 1H), 1.13-1.04 (m, 1H), 0.81 (d, J = 10.0, 3H). **^{13}C NMR** (125 MHz, $CDCl_3$) δ 206.0 (t, J = 9.4 Hz), 188.5 (t, J = 29.9 Hz), 134.1, 132.1, 131.4 (d, J = 1.7 Hz), 130.3 (t, J = 2.7 Hz), 128.5, 124.4, 115.6 (t, J = 250.4 Hz), 96.2 (d, J = 15.0 Hz), 88.0 (t, J = 30.8 Hz), 36.3 (d, J = 4.9 Hz), 34.9 (d, J = 10.9 Hz), 32.4 (d, J = 5.1 Hz), 25.7, 25.4 (d, J = 9.1 Hz), 19.1 (d, J = 2.5 Hz), 17.6. **^{19}F NMR**

(470 MHz, $CDCl_3$) δ (-93.89)-(-94.08) (m). **IR** (Film): 3057, 2253, 1971, 1705, 1261, 1094, 904,806, 724, 649 cm^{-1}. **HRMS** (ESI) *m/z* calculated $C_{20}H_{24}F_2NaO$ 341.1687, found 341.1697.

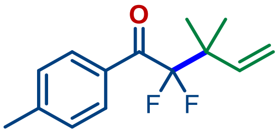

(155) According to the general procedure E, using tolyl trifluoromethyl ketone *N*-triftosylhydrazone (123.0 mg, 0.3 mmol), 3-methylbut-2-en-1-ol (51.6 mg, 0.6 mmol), and DCE (4.0 mL) afforded compound **155** (70.7 mg, 99% yield) as a colorless oil; **^{1}H-NMR** (500 MHz, $CDCl_3$) δ 7.95 (d, *J* = 8.5 Hz, 2H), 7.24 (d, *J* = 8.5 Hz, 2H), 6.01 (dd, *J* = 17.0, 10.5 Hz, 1H), 5.16-5.10 (m, 2H), 2.41 (s, 3H), 1.26 (s, 6H). **^{13}C-NMR** (125 MHz, $CDCl_3$) δ 189.8 (t, *J* = 30.0 Hz), 144.9, 140.1 (t, *J* = 5.0 Hz), 131.6, 130.5 (t, *J* = 3.8 Hz), 129.2, 120.8 (t, *J* = 257.5 Hz), 115.2, 43.5 (t, *J* = 21.3 Hz), 21.66, 21.24 (t, *J* = 3.8 Hz). **^{19}F-NMR** (470 MHz, $CDCl_3$) δ -107.32 (s). **IR** (Film): 3090, 2985, 1691, 1606, 1095, 1058, 899, 735 cm^{-1}. **HRMS** (ESI) m/z calcd. for $C_{14}H_{17}F_2O^+$ $[M+H]^+$: 239.1242; Found: 239.1241.

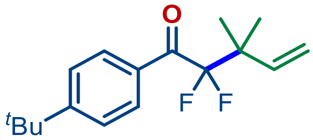

(156) According to the general procedure E, using 4-(*tert*-butyl)phenyl trifluoromethyl ketone *N*-triftosylhydrazone (135.6 mg, 0.3 mmol), 3-methylbut-2-en-1-ol (51.6 mg, 0.6 mmol), and DCE (4.0 mL) afforded compound **156** (69.7 mg, 83% yield) as a colorless oil; **^{1}H-NMR** (600 MHz, $CDCl_3$) δ 7.99 (d, *J* = 8.4 Hz, 2H), 7.47 (d, *J* = 8.4 Hz, 2H), 6.03 (dd, *J* = 17.4, 10.8 Hz, 1H), 5.21-5.10 (m, 2H), 1.34 (s, 9H), 1.27 (s, 6H). **^{13}C-NMR** (150 MHz, $CDCl_3$) δ 189.9 (t, *J* = 30.0 Hz), 157.73, 140.15 (t, *J* = 3.0 Hz), 131.45, 130.34 (t, *J* = 4.5 Hz), 125.43, 120.72 (t, *J* = 258.0 Hz), 115.18, 43.47 (t, *J* = 21.0 Hz), 35.16, 30.95, 21.28 (t, *J* = 4.5 Hz). **^{19}F-NMR** (564 MHz, $CDCl_3$) δ -107.22 (s). **IR** (Film): 3089, 2989, 1703, 1510, 1411, 1317, 1261, 1069, 724, 700 cm^{-1}. **HRMS** (ESI) m/z calcd. for $C_{17}H_{23}F_2O^+$ $[M+H]^+$: 281.1711; Found: 281.1709.

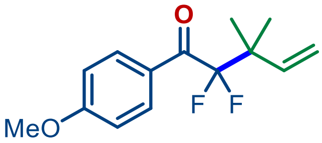

(157) According to the general procedure E, using 4-methoxyphenyl trifluoromethyl ketone *N*-triftosylhydrazone (127.8 mg, 0.3 mmol), 3-methylbut-2-en-1-ol (51.6 mg, 0.6 mmol), and DCE (4.0 mL) afforded compound **157** (66.3 mg, 87% yield) as a colorless oil; **^{1}H-NMR** (600 MHz, $CDCl_3$) δ 8.05 (d, *J* = 9.0 Hz, 2H), 6.92 (d, *J* = 9.0 Hz, 2H), 6.01 (dd, *J* = 17.5, 10.8 Hz, 1H), 5.17-5.10 (m, 2H), 3.87 (s, 3H), 1.26 (s, 6H). **^{13}C-NMR** (150 MHz, $CDCl_3$) δ 188.5 (t, *J* = 30.0 Hz), 164.1, 140.2 (t, *J* = 3.0 Hz), 132.9 (t, *J* = 4.5 Hz), 126.9, 120.9 (t, *J* = 258.0 Hz), 115.1, 113.7, 55.5, 43.5 (t, *J* = 21.0 Hz), 21.3 (t, *J* = 4.5 Hz). **^{19}F-NMR** (564 MHz, $CDCl_3$) δ -106.98(s). **IR** (Film): 3089, 2989, 1703, 1510, 1411, 1317, 1261, 1069, 724, 700 cm^{-1}. **HRMS** (ESI) m/z calcd. for $C_{14}H_{17}F_2O_2^+$ $[M+H]^+$: 255.1191; Found: 255.1187.

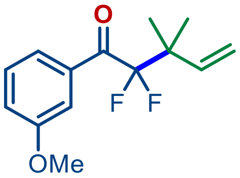

(158) According to the general procedure E, using 3-methoxyphenyl trifluoromethyl ketone *N*-triftosylhydrazone (127.8 mg, 0.3 mmol), 3-methylbut-2-en-1-ol (51.6 mg, 0.6 mmol), and DCE (4.0 mL) afforded compound **158** (73.9 mg, 97% yield) as a colorless oil; **^{1}H-NMR** (500 MHz, $CDCl_3$) δ 7.65 (d, *J* = 8.0 Hz, 1H), 7.54 (s, 1H), 7.35 (t, *J* = 8.0 Hz, 1H), 7.13 (dd, *J* = 8.0, 2.5 Hz, 1H), 6.01 (dd, *J* = 17.5, 11.0 Hz, 1H), 5.17-5.11 (m, 2H), 3.84 (s, 3H), 1.26 (s, 6H). **^{13}C-NMR** (150 MHz, $CDCl_3$) δ 190.0 (t, *J* = 30.0 Hz), 159.5, 140.0 (t, *J* = 4.5 Hz), 135.3, 129.4, 122.9 (t, *J* = 4.5 Hz), 120.6 (t, *J* = 258.0 Hz), 120.3, 115.3, 114.5 (t, *J* = 3.0 Hz), 55.3, 43.5 (t, *J* = 19.5 Hz), 21.2 (t, *J* = 3.0 Hz). **^{19}F-NMR** (564 MHz, $CDCl_3$) δ -107.29 (s); **IR** (Film): 3089, 2984, 1692, 1580, 1281, 1096, 756 cm^{-1}. **HRMS** (ESI) m/z calcd. for $C_{14}H_{17}F_2O_2^+$ $[M+H]^+$: 255.1191; Found: 255.1187.

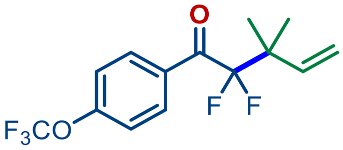

(159) According to the general procedure E, using 4-(trifluoromethoxy)phenyl trifluoromethyl ketone *N*-triftosylhydrazone (144.0 mg, 0.3 mmol), 3-methylbut-2-en-1-ol (51.6 mg, 0.6 mmol), and DCE (4.0 mL) afforded compound **159** 85.0 mg, 92% yield) as a colorless oil; **^{1}H-NMR** (500 MHz, $CDCl_3$) δ 8.10 (d, *J* = 8.5 Hz, 2H), 7.27 (d, *J* = 8.5 Hz, 2H), 5.98 (dd, *J* = 17.5, 11.0 Hz, 1H), 5.18-5.11 (m, 2H), 1.27 (s, 6H). **^{13}C-NMR** (150 MHz, $CDCl_3$) δ 188.8 (t, *J* = 31.5 Hz), 153.1, 139.8 (t, *J* = 4.5 Hz), 132.5 (t, *J* = 4.5 Hz), 132.2, 120.6 (t, *J* = 258.0 Hz), 120.3 (q, *J* = 258.0 Hz), 120.0, 115.6, 43.5 (t, *J* = 6.0 Hz), 21.2 (t, *J* = 4.5 Hz). **^{19}F-NMR** (564 MHz, $CDCl_3$) δ -57.62 (s, 3F), -107.28 (s, 2F). **IR** (Film): 3093, 2988, 1712, 1698, 1252, 1210, 1168, 879, 741 cm^{-1}.

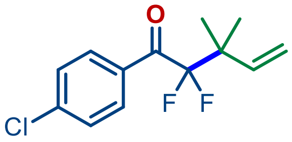

(160) According to the general procedure E, using 4-chlorophenyl trifluoromethyl ketone *N*-triftosylhydrazone (129.0 mg, 0.3 mmol), 3-methylbut-2-en-1-ol (51.6 mg, 0.6 mmol), and DCE (4.0 mL) afforded compound **160** (67.3 mg, 87% yield) as a colorless oil; **^{1}H-NMR** (500 MHz, $CDCl_3$) δ 7.98 (d, *J* = 8.5 Hz, 2H), 7.43 (d, *J* = 8.5 Hz, 2H), 5.98 (dd, *J* = 17.5, 10.5 Hz, 1H), 5.17-5.11 (m, 2H), 1.26 (s, 6H). **^{13}C-NMR** (150 MHz, $CDCl_3$) δ 189.1 (t, *J* = 31.5 Hz), 140.5, 139.8 (t, *J* = 3.0 Hz), 132.4 (t, *J* = 3.0 Hz), 131.7 (t, *J* = 4.5 Hz), 128.8, 120.6 (t, *J* = 258.0 Hz), 115.6, 43.4 (t, *J* = 21.0 Hz), 21.2 (t, *J* = 4.5 Hz). **^{19}F-NMR** (564 MHz, $CDCl_3$) δ -107.34 (s). **IR** (Film): 3287, 2983, 1703, 1602, 1096, 899, 880 cm^{-1}. **HRMS** (ESI) m/z calcd. for $C_{13}H_{12}ClF_2O^-$ $[M-H]^-$: 257.0550; Found: 257.0550.

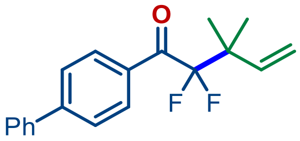

(161) According to the general procedure E, using (1,1'-biphenyl)-4-yl trifluoromethyl ketone *N*-triftosylhydrazone (141.6 mg, 0.3 mmol), 3-methylbut-2-en-1-ol (51.6 mg, 0.6 mmol), and DCE (4.0 mL) afforded compound **161** (88.2 mg, 98% yield) as a colorless oil; **^{1}H-NMR** (500 MHz, $CDCl_3$) δ 8.14 (d, *J* = 8.0 Hz, 2H), 7.69 (d, *J* = 8.5 Hz, 2H), 7.64 (d, *J* = 7.0 Hz, 2H), 7.48 (t, *J* = 7.0 Hz, 2H), 7.43 (d, *J* = 7.5 Hz, 1H), 6.05 (dd, *J* = 17.5, 11.0 Hz, 1H), 5.21-5.14 (m, 2H), 1.31 (s, 6H). **^{13}C-NMR** (125 MHz, $CDCl_3$) δ 189.8 (t, *J* = 30.0 Hz), 146.4, 140.1 (t, *J* = 5.0 Hz), 139.6, 132.8, 130.9 (t, *J* = 5.0 Hz), 129.0, 128.5, 127.3, 127.0, 120.8 (t, *J* = 258.8 Hz), 115.4, 43.5 (t, *J* = 21.3 Hz), 21.3 (t, *J* = 5.0 Hz). **^{19}F-NMR** (470 MHz, $CDCl_3$) δ -107.26 (s). **IR** (Film): 3287, 2983, 1703, 1602, 1096, 899, 880 cm^{-1}.

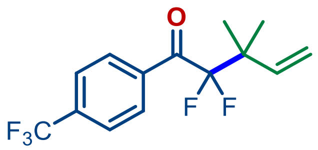

(162) According to the general procedure E, using 4-(trifluoromethyl)phenyl trifluoromethyl ketone *N*-triftosylhydrazone (139.2 mg, 0.3 mmol), 3-methylbut-2-en-1-ol (51.6 mg, 0.6 mmol), and DCE (4.0 mL) afforded compound **162** (64.8 mg, 74% yield) as a colorless oil; **^{1}H-NMR** (600 MHz, $CDCl_3$) δ 8.13 (d, *J* = 8.4 Hz, 2H), 7.72 (d, *J* = 8.4 Hz, 2H), 5.98 (dd, *J* = 17.4, 10.2 Hz, 1H), 5.19-5.13 (m, 2H), 1.28 (s, 6H). **^{13}C-NMR** (125 MHz, $CDCl_3$) δ 189.6 (t, *J* = 32.5 Hz), 139.7 (t, *J* = 3.8 Hz), 136.9, 134.9 (q, *J* = 32.5 Hz), 130.5 (t, *J* = 3.8 Hz), 125.5 (q, *J* = 3.8 Hz), 123.5 (q, *J* = 272.5 Hz), 120.4 (t, *J* = 258.8 Hz), 115.9, 43.5 (t, *J* = 20.0 Hz), 21.1 (t, *J* = 3.8 Hz). **^{19}F-NMR** (470 MHz, $CDCl_3$) δ -63.40 (s, 3F), -107.63 (s, 2F). **IR** (Film): 3073, 2928, 1693, 1446, 1259, 724, 704 cm^{-1}. **HRMS** (ESI) m/z calcd. for $C_{14}H_{14}F_5O^+$ $[M+H]^+$: 291.0813; Found: 291.0818.

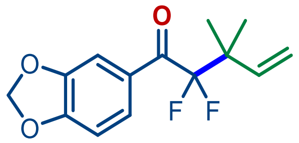

(163) According to the general procedure E, using piperonyl trifluoromethyl ketone *N*-triftosylhydrazone (132.0 mg, 0.3 mmol), allyl alcohol (34.8 mg, 0.6 mmol), and DCE (4.0 mL) afforded compound **164** (77.2 mg, 96% yield) as a colorless oil; **^{1}H-NMR** (500 MHz, $CDCl_3$) δ 7.71 (d, *J* = 8.5 Hz, 1H), 7.49 (s, 1H), 6.83 (d, *J* = 8.0 Hz, 1H), 6.04 (s, 2H), 5.99 (dd, *J* = 17.5, 11.0 Hz, 1H), 5.16-5.10 (m, 2H), 1.25 (s, 6H). **^{13}C-NMR** (125 MHz, $CDCl_3$) δ 188.0 (t, *J* = 30.0 Hz), 152.4, 147.9, 140.1 (t, *J* = 3.8 Hz), 128.4, 127.4 (t, *J* = 5.0 Hz), 120.8 (t, *J* = 258.8 Hz), 115.1, 109.9 (t, *J* = 3.8 Hz), 107.9, 102.0, 43.5 (t, *J* = 22.5 Hz), 21.2 (t, *J* = 5.0 Hz). **^{19}F-NMR** (470 MHz, $CDCl_3$) δ -106.64 (s). **IR** (Film): 3089, 2985, 2907, 1683, 1440, 1245, 1037, 924 cm^{-1}.

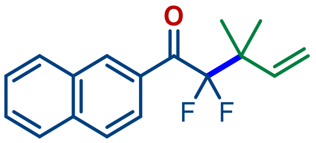

(164) According to the general procedure E, using 2-naphthyl trifluoromethyl ketone *N*-triftosylhydrazone (133.8 mg, 0.3 mmol), 3-methylbut-2-en-1-ol (51.6 mg, 0.6 mmol), and DCE (4.0 mL) afforded compound **164** (74.0 mg, 90% yield) as a colorless oil; **^{1}H-NMR** (500 MHz, $CDCl_3$) δ 8.66 (s, 1H), 8.06 (dd, *J* = 9.0, 1.5 Hz, 1H), 7.97 (d, *J* = 8.0 Hz, 1H), 7.90-7.84 (m, 2H), 7.66-7.60 (m, 1H), 7.59-7.53 (m, 1H), 6.08 (dd, *J* = 17.5, 10.5 Hz, 1H), 5.23-5.13 (m, 2H), 1.33 (s, 6H). **^{13}C-NMR** (150 MHz, $CDCl_3$) δ 190.1 (t, *J* = 31.5 Hz), 140.1 (t, *J* = 3.0 Hz), 135.7, 132.9 (t, *J* = 6.0 Hz), 132.2, 131.3 (t, *J* = 1.5 Hz), 130.1, 129.1, 128.2, 127.6, 126.8, 125.1 (t, *J* = 3.0 Hz), 120.9 (t, *J* = 258.0 Hz), 115.3, 43.6 (t, *J* = 22.5 Hz), 21.3 (t, *J* = 4.5 Hz). **^{19}F-NMR** (564 MHz, $CDCl_3$) δ -106.82 (s). **HRMS** (ESI) m/z calcd. for $C_{17}H_{17}F_2O^+$ $[M+H]^+$: 275.1242; Found: 275.1212.

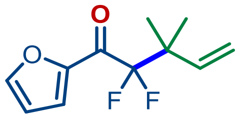

(165) According to the general procedure E, using furan-2-yl trifluoromethyl ketone *N*-triftosylhydrazone (115.8 mg, 0.3 mmol), 3-methylbut-2-en-1-ol (51.6 mg, 0.6 mmol), and DCE (4.0 mL) afforded compound **165** (30.8 mg, 48% yield) as a colorless oil; **^{1}H-NMR** (500 MHz, $CDCl_3$) δ 7.70 (d, *J* = 1.5 Hz, 1H), 7.39-7.34 (m, 1H), 6.57 (dd, *J* = 3.5, 1.5 Hz, 1H), 5.95 (dd, *J* = 17.5, 10.0 Hz, 1H), 5.16-5.08 (m, 2H), 1.23 (s, 6H). **^{13}C-NMR** (125 MHz, $CDCl_3$) δ 178.4 (t, *J* = 31.3 Hz), 149.6, 148.5, 139.4 (t, *J* = 3.8 Hz), 123.3 (t, *J* = 7.5 Hz), 119.8 (t, *J* = 257.5 Hz), 115.6, 112.6, 43.4 (t, *J* = 21.3 Hz), 20.9 (t, *J* = 3.8 Hz). **^{19}F-NMR** (470 MHz, $CDCl_3$) δ -111.33 (s). **HRMS** (ESI)

m/z calcd. for $C_{11}H_{13}F_2O_2^+$ $[M+H]^+$: 215.0878; Found: 215.0876.

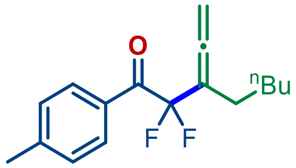

(**166**) According to the general procedure E, using 4-(methyl)phenyl trifluoromethyl ketone *N*-triftosylhydrazone (123.0 mg, 0.3 mmol), hept-2-yn-1-ol (67.2 mg, 0.6 mmol), and DCE (4.0 mL) afforded compound **166** (79.2 mg, 95% yield) as a colorless oil; **^{1}H-NMR** (500 MHz, $CDCl_3$) δ 7.93 (d, *J* = 8.0 Hz, 2H), 7.26 (d, *J* = 8.0 Hz, 2H), 4.99-4.92 (m, 2H), 2.42 (s, 3H), 2.22-2.14 (m, 2H), 1.55-1.45 (m, 2H), 1.36-1.27 (m, 4H), 0.94-0.83(m, 3H). **^{13}C-NMR** (125 MHz, $CDCl_3$) δ 207.3 (t, *J* = 8.8 Hz), 188.1 (t, *J* = 28.8 Hz), 145.1, 130.2 (t, *J* = 2.5 Hz), 129.9, 129.2, 116.9 (t, *J* = 251.3 Hz), 101.4 (t, *J* = 27.5 Hz), 81.7, 31.2, 27.0, 25.1, 22.3, 21.7, 13.9. **^{19}F NMR** (470 MHz, $CDCl_3$) δ -98.74 (t, *J* = 6.0 Hz). **HRMS** (ESI) *m/z* calculated $C_{17}H_{20}F_2NaO$ 301.1380, found 301.1385.

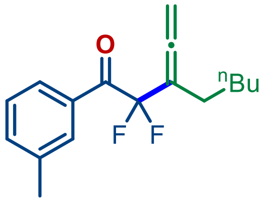

(**167**) According to the general procedure E, using 3-(methyl)phenyl trifluoromethyl ketone *N*-triftosylhydrazone (123.0 mg, 0.3 mmol), hept-2-yn-1-ol (67.2 mg, 0.6 mmol), and DCE (4.0 mL) afforded compound **167** (80.9 mg, 97% yield) as a colorless oil; **^{1}H-NMR** (500 MHz, $CDCl_3$) δ 7.85 (s, 1H), 7.82 (d, *J* = 8.0 Hz, 1H), 7.41 (d, *J* = 8.0 Hz, 1H), 7.34 (t, *J* = 8.0 Hz, 1H), 5.00-4.92 (m, 2H), 2.41 (s, 3H), 2.22-2.14 (m, 2H), 1.55-1.45 (m, 2H), 1.36-1.27 (m, 4H), 0.94-0.83(m, 3H). **^{13}C-NMR** (125 MHz, $CDCl_3$) δ 207.4 (t, *J* = 7.5 Hz), 188.8 (t, *J* = 28.8 Hz), 138.4, 134.9, 132.5, 130.5 (t, *J* = 2.5 Hz), 128.4, 127.5 (t, *J* = 2.5 Hz), 116.9 (t, *J* = 251.3 Hz), 101.4 (t, *J* = 27.5 Hz), 81.8, 31.3, 27.0, 25.1, 22.4, 21.3, 14.0. **^{19}F NMR** (470 MHz, $CDCl_3$) δ -98.71 (t, *J* = 6.0 Hz). **IR** (Film): 3053, 2957, 2930, 2861, 2256, 1954, 1703, 1603, 1584, 1466, 1380, 1265, 1124, 1070, 904, 726, 649 cm^{-1}. **HRMS** (ESI) *m/z* calculated $C_{17}H_{20}F_2NaO$ 301.1384, found 301.1374.

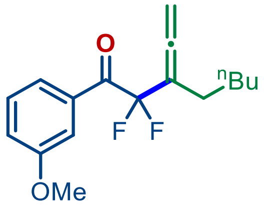

(**168**) According to the general procedure E, using 3-methoxyphenyl trifluoromethyl ketone *N*-triftosylhydrazone (127.8 mg, 0.3 mmol), hept-2-yn-1-ol (67.2 mg, 0.6 mmol), and DCE (4.0 mL) afforded compound **168** (86.4 mg, 98% yield) as a colorless oil; **^{1}H-NMR** (500 MHz, $CDCl_3$) δ 7.61 (d, *J* = 8.0 Hz, 1H), 7.55 (t, *J* = 2.0 Hz, 1H), 7.37 (t, *J* = 8.0 Hz, 1H), 7.15 (dd, *J* = 8.0, 2.5 Hz, 1H), 5.00-4.95 (m, 2H), 3.85 (s, 3H), 2.22-2.16 (m, 2H), 1.54-1.46 (m, 2H), 1.36-1.27 (m, 4H), 0.92-0.85 (m, 3H). **^{13}C-NMR** (125 MHz, $CDCl_3$) δ 207.3 (t, *J* = 8.8 Hz), 188.3 (t, *J* = 30.0 Hz), 159.5, 133.6, 129.5, 122.8 (t, *J* = 3.0 Hz), 120.5, 116.8 (t, *J* = 251.3 Hz), 114.2 (t, *J* = 2.0 Hz), 101.3 (t, *J* = 27.5 Hz), 81.8, 55.3, 31.2, 27.0, 25.1, 22.3, 13.9. **^{19}F NMR** (470 MHz, $CDCl_3$) δ -98.58 (t, *J* = 6.0 Hz). **HRMS** (ESI) *m/z* calculated $C_{17}H_{20}F_2NaO_2$ 317.1329, found 317.1333.

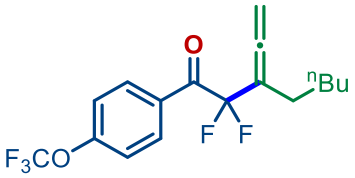

(**169**) According to the general procedure E, using 4-(trifluoromethoxy)phenyl trifluoromethyl ketone *N*-triftosylhydrazone (144.0 mg, 0.3 mmol), hept-2-yn-1-ol (67.2 mg, 0.6 mmol), and DCE (4.0 mL) afforded compound **169** (97.1 mg, 93% yield) as a colorless oil; **^{1}H-NMR** (500 MHz, $CDCl_3$) δ 8.10 (d, *J* = 8.5 Hz, 2H), 7.30 (d, *J* = 8.5 Hz, 2H), 5.04-4.94 (m, 2H), 2.26-2.15 (m, 2H), 1.58-1.47 (m, 2H), 1.38-1.22 (m, 4H), 0.96-0.85 (m, 3H). **^{13}C-NMR** (125 MHz, $CDCl_3$) δ 207.4 (t, *J* = 7.5 Hz), 187.1 (t, *J* = 30.0 Hz), 153.23 (t, *J* = 2.5 Hz), 132.3 (t, *J* = 3.8 Hz), 130.6, 120.3 (q, *J* = 258.0 Hz), 120.1, 116.8 (t, *J* = 251.3 Hz), 101.2 (t, *J* = 27.5 Hz), 82.0, 31.2, 27.0, 25.1, 22.3, 13.9. **^{19}F NMR** (470 MHz, $CDCl_3$) δ -57.67 (s), -98.93 (t, *J* = 5.6 Hz). **IR** (Film): 3055, 2959, 2932, 2861, 2254, 1954, 1709, 1604, 1506, 1254, 1213, 1173, 1132, 906, 728, 649 cm^{-1}. **HRMS** (ESI) *m/z* calculated $C_{17}H_{17}F_5NaO_2$ 371.1039, found 371.1041.

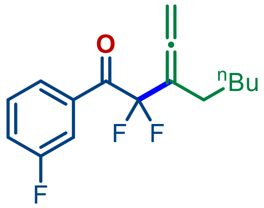

(**170**) According to the general procedure E, using 3-fluorophenyl trifluoromethyl ketone *N*-triftosylhydrazone (124.2 mg, 0.3 mmol), hept-2-yn-1-ol (67.2 mg, 0.6 mmol), and DCE (4.0 mL) afforded compound **170** (82.1 mg, 97% yield) as a colorless oil; **^{1}H-NMR** (500 MHz, $CDCl_3$) δ 7.86-7.79 (m, 1H), 7.71 (dt, *J* = 9.5, 2.5 Hz, 1H), 7.46 (td, *J* = 8.5, 5.5 Hz, 1H), 7.32 (tdd, *J* = 8.0, 2.5, 1.0 Hz, 1H), 5.01-4.95 (m, 2H), 2.24-2.15 (m, 2H), 1.56-1.46 (m, 2H), 1.40-1.26 (m, 4H), 0.92-0.84 (m, 3H). **^{13}C-NMR** (125 MHz, $CDCl_3$) δ 207.4 (t, *J* = 7.5 Hz), 187.46 (td, *J* = 30.0, 2.5 Hz), 162.5 (d, *J* = 246.0 Hz), 134.3 (d, *J* = 6.3 Hz), 130.2 (d, *J* = 7.5 Hz), 125.9 (q, *J* = 2.5 Hz), 121.2 (d, *J* = 21.3 Hz), 117.0-116.7 (m), 116.7 (t, *J* = 251.3 Hz), 101.1 (t, *J* = 27.5 Hz), 82.1, 31.2, 27.0, 25.0, 22.3, 14.0. **^{19}F NMR** (470 MHz, $CDCl_3$) δ (-98.88)-(-98.95) (m), (-111.39)-(-111.47) (m). **IR** (Film): 3053, 2932, 2857, 2255, 1957, 1710, 1588, 1444, 1263, 1117, 903, 721, 649 cm^{-1}. **HRMS** (ESI) *m/z* calculated $C_{16}H_{17}F_3NaO$ 305.1133, found 305.1124.

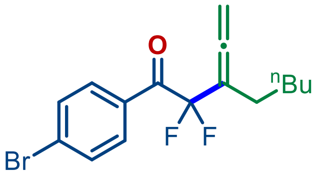

(171) According to the general procedure E, using 4-bromophenyl trifluoromethyl ketone *N*-triftosylhydrazone (141.9 mg, 0.3 mmol), hept-2-yn-1-ol (67.2 mg, 0.6 mmol), and DCE (4.0 mL) afforded compound **171** (98.8 mg, 96% yield) as a colorless oil; **^{1}H-NMR** (600 MHz, $CDCl_3$) δ 7.89 (d, *J* = 9.0 Hz, 2H), 7.64-7.60 (m, 2H), 4.99-4.95 (m, 2H), 2.22-2.14 (m, 2H), 1.54-1.45 (m, 2H), 1.36-1.29 (m, 4H), 0.92-0.85 (m, 3H). **^{13}C-NMR** (150 MHz, $CDCl_3$) δ 207.4 (t, *J* = 8.0 Hz), 187.7 (t, *J* = 30.0 Hz), 131.9, 131.6 (t, *J* = 3.0 Hz), 131.2, 129.6, 116.8 (t, *J* = 250.5 Hz), 101.2 (t, *J* = 27.0 Hz), 82.0, 31.2, 27.0, 25.0, 22.3, 14.0. **^{19}F NMR** (564 MHz, $CDCl_3$) δ -98.89 (t, *J* = 6.0 Hz). **HRMS** (ESI) *m/z* calculated $C_{16}H_{17}BrF_2NaO$ 365.0329, found 365.0333.

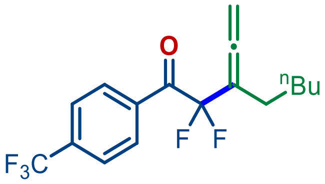

(172) According to the general procedure E, using 4-(trifluoromethyl)phenyl trifluoromethyl ketone *N*-triftosylhydrazone (139.2 mg, 0.3 mmol), hept-2-yn-1-ol (67.2 mg, 0.6 mmol), and DCE (4.0 mL) afforded

compound **172** (88.6 mg, 89% yield) as a colorless oil; **^{1}H-NMR** (600 MHz, $CDCl_3$) δ 8.14 (d, *J* = 7.8 Hz, 2H), 7.75 (d, *J* = 8.4 Hz, 2H), 5.04-4.94 (m, 2H), 2.25-2.17 (m, 2H), 1.55-1.48 (m, 2H), 1.37-1.30 (m, 4H), 0.93–0.87 (m, 3H). **^{13}C-NMR** (150 MHz, $CDCl_3$) δ 207.4 (t, *J* = 9.0 Hz), 187.8 (t, *J* = 30.0 Hz), 135.2, 135.1 (q, *J* = 31.5 Hz), 130.5 (t, *J* = 3.0 Hz), 125.6 (q, *J* = 3.0 Hz), 123.4 (q, *J* = 271.5 Hz), 116.7 (t, *J* = 250.5 Hz), 101.1 (t, *J* = 27.0 Hz), 82.2, 31.2, 27.0, 25.0, 22.4, 14.0. **^{19}F NMR** (564 MHz, $CDCl_3$) δ -63.40, -99.09 (t, *J* = 5.7 Hz). **IR** (Film): 3055, 2932, 2253, 1954, 1715, 1326, 1265, 1136, 1067, 903, 718, 649 cm^{-1}. **HRMS** (ESI) *m/z* calculated $C_{17}H_{17}F_5NaO$ 355.1097, found 355.1099.

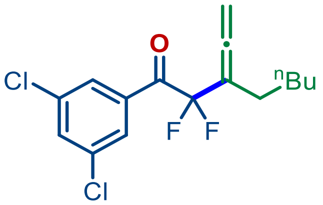

(173) According to the general procedure E, using 3,5-dichlorophenyl trifluoromethyl ketone *N*-triftosylhydrazone (138.9 mg, 0.3 mmol), hept-2-yn-1-ol (67.2 mg, 0.6 mmol), and DCE (4.0 mL) afforded compound **173** (92.0 mg, 93% yield) as a colorless oil; **^{1}H-NMR** (600 MHz, $CDCl_3$) δ 7.89 (d, *J* = 1.8 Hz, 2H), 7.59 (t, *J* = 1.8 Hz, 1H), 5.05-5.00 (m, 2H), 2.20-2.15 (m, 2H), 1.54-1.47 (m, 2H), 1.36-1.29 (m, 4H), 0.92-0.87 (m, 3H). **^{13}C-NMR** (150 MHz, $CDCl_3$) δ 207.4 (t, *J* = 7.5 Hz), 186.4 (t, *J* = 30.0 Hz), 135.6, 134.7, 133.7, 128.4 (t, *J* = 3.0 Hz), 116.5 (t, *J* = 250.5 Hz), 100.9 (t, *J* = 28.5 Hz), 82.3, 31.2, 26.9, 25.0, 22.3, 14.0. **^{19}F NMR** (564 MHz, $CDCl_3$) δ -99.03 (t, *J* = 5.8 Hz). **HRMS** (ESI) *m/z* calculated $C_{16}H_{16}Cl_2F_2NaO$ 355.0444, found 355.0447.

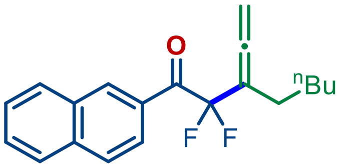

(174) According to the general procedure E, using 2-naphthyl trifluoromethyl ketone *N*-triftosylhydrazone (133.8 mg, 0.3 mmol), hept-2-yn-1-ol (67.2 mg, 0.6 mmol), and DCE (4.0 mL) afforded compound **174** (85.7 mg, 91% yield) as a colorless oil; **^{1}H NMR** (600 MHz, $CDCl_3$) δ 8.61 (s, 1H), 8.05 (dd, *J* = 8.6, 1.4 Hz, 1H), 7.96 (d, *J* = 8.1 Hz, 1H), 7.89-7.85 (m, 2H), 7.64-7.60 (m, 1H), 7.57-7.54 (m, 1H), 4.99-4.93 (m, 2H), 2.28-2.20 (m, 2H), 1.57-1.48 (m, 2H), 1.37-1.28 (m, 4H), 0.92-0.85 (m, 3H). **^{13}C NMR** (150 MHz, $CDCl_3$) δ 207.4 (t, *J* = 8.1 Hz), 188.5 (t, *J* = 29.1 Hz), 135.8, 132.7 (t, *J* = 3.3 Hz), 132.2, 130.0, 129.6, 129.2, 128.3, 127.7, 126.9, 124.9, 117.0 (t, *J* = 252.9 Hz), 101.5 (t, *J* = 27.8 Hz), 81.9, 31.2, 27.0, 25.1, 22.4, 14.0. **^{19}F NMR** (564 MHz, $CDCl_3$) δ -98.3 (t, *J* = 4.9 Hz). **IR** (Film): 3055, 2932, 2255, 1953, 1699, 1627, 1422, 1263, 1112, 903, 718, 649 cm^{-1}. **HRMS** (ESI) *m/z* calculated $C_{20}H_{20}F_2NaO$ 337.1374, found 337.1379.

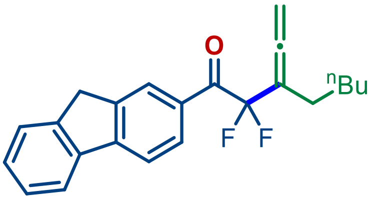

(175) According to the general procedure E, using 2-fluorenyl trifluoromethyl ketone *N*-triftosylhydrazone (145.2 mg, 0.3 mmol), hept-2-yn-1-ol (67.2 mg, 0.6 mmol), and DCE (4.0 mL) afforded compound **175** (99.3 mg, 94% yield) as a colorless oil; **^{1}H-NMR** (600 MHz, $CDCl_3$) δ 8.21 (s, 1H), 8.08 (d, *J* = 8.4 Hz, 1H), 7.87-7.78 (m, 2H), 7.60-7.51 (m, 1H), 7.45-7.36 (m, 2H), 5.10-4.91 (m, 2H), 3.94 (s, 2H), 2.26-2.19 (m, 2H), 1.56-1.48 (m, 2H), 1.37-1.30 (m, 4H), 0.92-0.86 (m, 3H). **^{13}C-NMR** (150 MHz, $CDCl_3$) δ 207.4 (t, *J* = 7.5 Hz), 188.3 (t, *J* = 28.5 Hz), 147.4, 144.7, 143.1, 140.2, 130.7, 129.7 (t, *J* = 3.0 Hz), 128.4, 127.1, 126.7 (t, *J* = 3.0 Hz), 125.3. 121.1,

119.6, 117.0 (t, J = 252.0 Hz), 101.5 (t, J = 27.0 Hz), 81.8, 36.9, 31.2, 27.0, 25.1, 22.4, 14.0. **^{19}F NMR** (564 MHz, $CDCl_3$) δ -98.27 (t, J = 5.5 Hz). **IR** (Film): 3055, 2928, 2255, 1955, 1695, 1610, 1265, 1111, 903, 721, 649 cm^{-1}. **HRMS** (ESI) *m/z* calculated $C_{23}H_{22}F_2NaO$ 375.1531, found 375.1528.

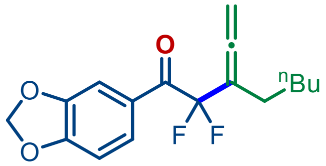

(176) According to the general procedure E, using piperonyl trifluoromethyl ketone *N*-triftosylhydrazone (132.0 mg, 0.3 mmol), hept-2-yn-1-ol (67.2 mg, 0.6 mmol), and DCE (4.0 mL) afforded compound **176** (83.2 mg, 90% yield) as a light yellow oil; **^{1}H-NMR** (500 MHz, $CDCl_3$) δ 7.62-7.57 (m, 1H), 7.40 (d, J = 2.0 Hz, 1H), 6.78 (d, J = 8.0 Hz, 1H), 5.98 (s, 2H), 4.95-4.86 (m, 2H), 2.13-2.04 (m, 2H), 1.45-1.36 (m, 2H), 1.24 (dd, J = 7.0, 3.5 Hz, 4H), 0.86-0.78 (m, 3H). **^{13}C-NMR** (125 MHz, $CDCl_3$) δ 207.2 (t, J = 7.5 Hz), 186.6 (t, J = 28.8 Hz), 152.6, 148.0, 127.2 (t, J = 3.8 Hz), 126.9, 116.8 (t, J = 251.3 Hz), 109.6 (t, J = 2.5 Hz), 108.0, 102.0, 101.5 (t, J = 27.5 Hz), 81.8, 31.2, 26.9, 25.1, 22.3, 13.9. **^{19}F NMR** (470 MHz, $CDCl_3$) δ -98.13 (t, J = 5.8 Hz). **IR** (Film): 3055, 2931, 2255, 1953, 1093, 1606, 1488, 1445, 1354, 1265, 1240, 1083, 1040, 903, 721, 649 cm^{-1}. **HRMS** (ESI) *m/z* calculated $C_{17}H_{18}F_2NaO_3$ 331.1116, found 331.1106.

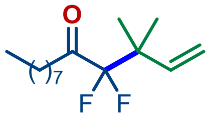

(177) According to the general procedure F, using 1,1,1-trifluorodecan-2-one *N*-triftosylhydrazone (129.6 mg, 0.3 mmol), 3-methylbut-2-en-1-ol (51.6 mg, 0.6 mmol), and DCE (4.0 mL) afforded compound **177** (44.6 mg, 84% yield) as a colorless oil; **^{1}H-NMR** (500 MHz, $CDCl_3$) δ 6.00-5.90 (m, 1H), 5.20-5.10 (m, 2H), 2.59 (t, J = 7.5 Hz, 2H), 1.60-1.52 (m, 2H), 1.34-1.23 (m, 10H), 1.18 (s, 6H), 0.88 (t, J = 6.5 Hz, 3H). **^{13}C-NMR** (150 MHz, $CDCl_3$) δ 202.2 (t, J = 30.0 Hz), 139.7, 119.1 (t, J = 258.0 Hz), 115.6, 42.7 (t, J = 21.0 Hz), 39.1, 31.79, 29.3, 29.1, 28.9, 22.6, 20.6 (t, J = 4.5 Hz), 14.1. **^{19}F-NMR** (564 MHz, $CDCl_3$) δ -114.96 (s). **HRMS** (ESI) m/z calcd. for $C_9H_{15}F_2O^+$ $[M+H]^+$: 177.1085; Found: 177.1022.

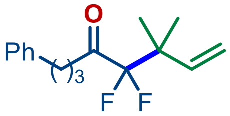

(178) According to the general procedure F, using 1,1,1-trifluoro-5-phenylpentan-2-one *N*-triftosylhydrazone (131.4 mg, 0.3 mmol), 3-methylbut-2-en-1-ol (51.6 mg, 0.6 mmol), and DCE (4.0 mL) afforded compound **178** (65.7 mg, 92% yield) as a colorless oil; **^{1}H-NMR** (500 MHz, $CDCl_3$) δ 7.30 (t, J = 7.5 Hz, 2H), 7.23-7.15 (m, 3H), 5.92 (dd, J = 17.5, 11.0 Hz, 1H), 5.17-5.10 (m, 2H), 2.63 (t, J = 7.5 Hz, 4H), 1.92 (p, J = 7.5 Hz, 2H), 1.19 (s, 6H). **^{13}C-NMR** (125 MHz, $CDCl_3$) δ201.8 (t, J = 31.3 Hz), 141.3, 139.6 (t, J = 3.8 Hz), 128.4, 126.0, 119.0 (t, J = 257.5 Hz), 115.7, 42.7 (t, J = 21.3 Hz), 38.2, 34.7, 24.1, 20.5 (t, J = 5.0 Hz). **^{19}F-NMR** (470 MHz, $CDCl_3$) δ -114.81 (s).

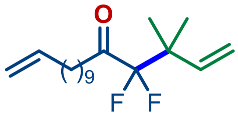

(179) According to the general procedure F, using 1,1,1-trifluorotridec-12-en-2-one *N*-triftosylhydrazone (141.6 mg, 0.3 mmol), 3-methylbut-2-en-1-ol (51.6 mg, 0.6 mmol), and DCE (4.0 mL) afforded compound **179** (44.0 mg, 78% yield) as a colorless oil; **^{1}H-NMR** (500 MHz, $CDCl_3$) δ 5.94 (dd, J = 17.5, 11.0 Hz, 1H), 5.86-5.75 (m, 1H), 5.20-5.08 (m, 2H), 5.03-4.89 (m, 2H), 2.59 (t, J = 7.5 Hz, 2H), 2.07-2.00 (m, 2H), 1.59-1.53 (m, 3H), 1.40-1.34 (m, 2H), 1.31-1.24 (m, 10H), 1.18 (s, 6H). **^{13}C-NMR** (125 MHz, $CDCl_3$) δ 202.2 (t, J = 31.3 Hz), 139.7 (t, J

= 3.8 Hz), 139.2, 119.1 (t, J = 257.5 Hz), 115.6, 114.1, 42.7 (t, J = 21.3 Hz), 39.0, 33.8, 29.4, 29.3, 29.3, 29.1, 28.9, 22.6, 20.6 (t, J = 3.8 Hz). **^{19}F-NMR** (470 MHz, $CDCl_3$) δ -114.91 (s). **HRMS** (ESI) m/z calcd. for $C_{18}H_{29}F_2O^-$ [M-H]$^-$: 299.2192; Found: 299.2193.

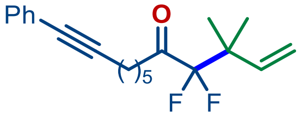

(180) According to the general procedure F, using 1,1,1-trifluoro-9-phenylnon-8-yn-2-one *N*-triftosylhydrazone (147.0 mg, 0.3 mmol), 3-methylbut-2-en-1-ol (51.6 mg, 0.6 mmol), and DCE (4.0 mL) afforded compound **180** (48.7 mg, 62% yield) as a colorless oil; **^{1}H-NMR** (500 MHz, $CDCl_3$) δ 7.41-7.35 (m, 2H), 7.30-7.24 (m, 3H), 5.94 (dd, J = 17.5, 10.5 Hz, 1H), 5.21-5.09 (m, 2H), 2.63 (t, J = 7.0 Hz, 2H), 2.41 (t, J = 7.0 Hz, 2H), 1.66-1.56 (M, 4H), 1.51-1.41 (m, 2H), 1.18 (s, 6H). **^{13}C-NMR** (125 MHz, $CDCl_3$) δ 201.9 (t, J = 31.3 Hz), 139.6 (t, J = 3.8 Hz), 131.5, 128.2, 127.5, 123.9, 119.0 (t, J = 257.5 Hz), 115.7, 89.9, 80.8, 42.7 (t, J = 22.5 Hz), 38.9, 28.4, 28.1, 22.2, 20.6 (t, J = 5.0 Hz), 19.2. **^{19}F-NMR** (470 MHz, $CDCl_3$) δ -114.86 (s). **HRMS** (ESI) m/z calcd. for $C_{16}H_{17}F_2O^+$ [M+H]$^+$: 263.1242; Found: 263.1248.

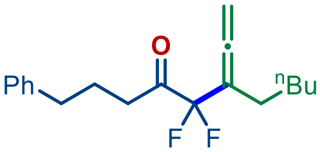

(181) According to the general procedure E, using 1,1,1-trifluoro-5-phenylpentan-2-one *N*-triftosylhydrazone (131.4 mg, 0.3 mmol), hept-2-yn-1-ol (67.2 mg, 0.6 mmol), and DCE (4.0 mL) afforded compound **181** (46.8 mg, 51% yield) as a colorless oil; **^{1}H-NMR** (600 MHz, $CDCl_3$) δ 7.29 (t, J = 7.8 Hz, 2H), 7.22-7.19 (m, 1H), 7.18-7.15 (m, 2H), 5.05-4.97 (m, 2H), 2.64 (t, J = 7.2 Hz, 4H), 2.09-2.02 (m, 2H), 1.95 (p, J = 7.2 Hz, 2H), 1.47-1.39 (m, 2H), 1.35-1.27 (m, 4H), 0.92-0.85 (m, 3H). **^{13}C-NMR** (150 MHz, $CDCl_3$) δ 206.7 (t, J = 9.0 Hz), 199.5 (t, J = 30.0 Hz), 141.2, 128.4, 126.1, 116.0 (t, J = 252.0Hz), 99.88 (t, J = 28.5 Hz), 81.4, 36.2, 34.7, 31.2, 26.9, 25.1, 24.3, 22.4, 14.0. **^{19}F NMR** (564 MHz, $CDCl_3$) δ -105.86 (d, J = 6.0 Hz). **HRMS** (ESI) *m/z* calculated $C_{19}H_{24}F_2NaO$ 329.1693, found 329.1687.

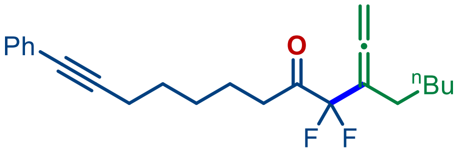

(182) According to the general procedure E, using 1,1,1-trifluoro-9-phenylnon-8-yn-2-one *N*-triftosylhydrazone (147.0 mg, 0.3 mmol), hept-2-yn-1-ol (67.2 mg, 0.6 mmol), and DCE (4.0 mL) afforded compound **182** (50.5 mg, 47% yield) as a colorless oil; **^{1}H-NMR** (600 MHz, $CDCl_3$) δ 7.41-7.35 (m, 2H), 7.30-7.24 (m, 3H), 5.11-5.05 (m, 2H), 2.66 (t, J = 7.2 Hz, 2H), 2.42 (t, J = 7.2 Hz, 2H), 2.10-2.03 (m, 2H), 1.71-1.58 (m, 4H), 1.52-1.42 (m, 4H), 1.34–1.27 (m, 4H), 0.92-0.86 (m, 3H). **^{13}C-NMR** (150 MHz, $CDCl_3$) δ 206.8 (t, J = 9.0 Hz), 199.6 (t, J = 30.0 Hz), 131.5, 128.2, 127.5, 123.9, 116.0 (t, J = 252.0 Hz), 99.9 (t, J = 28.5 Hz), 89.8, 81.4, 80.8, 36.9, 31.2, 28.4, 28.2, 26.9, 25.1, 22.4, 19.2, 14.0. **^{19}F NMR** (564 MHz, $CDCl_3$) δ -105.81 (d, J = 6.0 Hz). **IR** (Film): 3055, 2934, 2253, 1953, 1743, 1265, 1093, 903, 721, 649 cm^{-1}. **HRMS** (ESI) *m/z* calculated $C_{23}H_{28}F_2NaO$ 381.2001, found 381.2000.

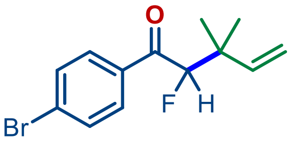

(183) According to the general procedure E, using 1-(4-bromophenyl)-2,2-difluoroethan-1-one *N*-

triftosylhydrazone (136.5 mg, 0.3 mmol), 3-methylbut-2-en-1-ol (51.6 mg, 0.6 mmol), and DCE (4.0 mL) afforded compound **183** (80.4 mg, 94% yield) as a colorless oil; **^{1}H-NMR** (500 MHz, $CDCl_3$) δ 7.83 (d, *J* = 8.5 Hz, 2H), 7.60 (d, *J* = 8.5 Hz, 2H), 5.86 (dd, *J* = 17.5, 11.0 Hz, 1H), 5.17 (d, *J* = 48.5 Hz, 1H), 5.08-5.02 (m, 2H), 1.18 (s, 3H), 1.17 (s, 3H). **^{13}C-NMR** (150 MHz, $CDCl_3$) δ 195.74 (d, *J* = 21.0 Hz), 142.10 (d, *J* = 4.5 Hz), 135.07, 131.79, 130.91 (d, *J* = 6.0 Hz), 128.77, 114.05, 99.15 (d, *J* = 192.0 Hz), 41.10 (d, *J* = 19.5 Hz), 23.39 (d, *J* = 4.5 Hz), 22.86 (d, *J* = 4.5 Hz). **^{19}F-NMR** (564 MHz, $CDCl_3$) δ -189.9 (d, *J* = 48.5 Hz);.**IR** (Film): 3086, 2970, 2932, 1692, 1582, 1070, 1009, 734, 703 cm^{-1}.

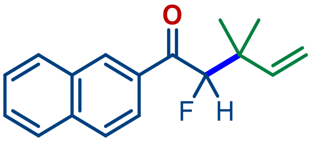

(184) According to the general procedure E, using 2,2-difluoro-1-(naphthalen-2-yl)ethan-1-one *N*-triftosylhydrazone (128.4 mg, 0.3 mmol), 3-methylbut-2-en-1-ol (51.6 mg, 0.6 mmol), and DCE (4.0 mL) afforded compound **184** (66.8 mg, 87% yield) as a colorless oil; **^{1}H-NMR** (500 MHz, $CDCl_3$) δ 8.50 (s, 1H), 8.01-7.93 (m, 2H), 7.90-7.84 (m, 2H), 7.64-7.58 (m, 1H), 7.57-7.52 (m, 1H), 5.91 (dd, *J* = 17.5, 11.0 Hz, 1H), 5.41 (d, *J* = 48.5 Hz, 1H), 5.09 -5.00 (m, 2H), 1.20 (s, 3H), 1.19 (s, 3H). **^{13}C-NMR** (125 MHz, $CDCl_3$) δ 196.4 (d, *J* = 20.0 Hz), 142.4 (d, *J* = 3.8 Hz), 135.6, 133.8, 132.3, 131.4 (d, *J* = 7.5 Hz), 129.8, 128.8, 128.4, 127.7, 126.8, 124.6 (d, *J* = 2.5 Hz), 113.8, 99.3, 97.8, 41.2 (d, J = 21.3 Hz), 23.4 (d, *J* = 5.0 Hz), 23.0 (d, *J* = 5.0 Hz). **^{19}F-NMR** (470 MHz, $CDCl_3$) δ -189.96 (d, *J* = 48.5 Hz). **HRMS** (ESI) m/z calcd. for $C_{17}H_{16}FO^-$ [M-H]$^-$: 255.1190; Found: 255.1190.

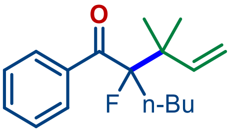

(185) According to the general procedure E, using 2,2-difluoro-1-phenylhexan-1-one *N*-triftosylhydrazone (130.2 mg, 0.3 mmol), 3-methylbut-2-en-1-ol (51.6 mg, 0.6 mmol), and DCE (4.0 mL) afforded compound **185** (54.2 mg, 69% yield) as a colorless oil; **^{1}H-NMR** (500 MHz, $CDCl_3$) δ 7.99-7.90 (m, 2H), 7.55-7.48 (m, 1H), 7.46-7.39 (m, 2H), 6.04 (dd, *J* = 11.0, 10.5 Hz, 1H), 5.10-5.03 (m, 2H), 2.25-2.05 (m, 1H), 1.87-1.76 (m, 1H), 1.52.-1.40 (m, 1H), 1.35-1.23 (m, 2H), 1.15 (s, 6H), 1.13-1.04 (m, 1H), 0.84 (t, *J* = 7.0 Hz, 3H). **^{13}C-NMR** (150 MHz, $CDCl_3$) δ 203.4 (d, *J* = 28.5 Hz), 142.9 (d, *J* = 4.5 Hz), 138.6 (d, *J* = 4.5 Hz), 132.3, 129.7 (d, *J* = 9.0 Hz), 128.1 (d, *J* = 1.5 Hz), 113.6, 108.5 (d, *J* = 195.0 Hz), 44.2 (d, *J* = 21.0 Hz), 34.0 (d, *J* = 22.5 Hz), 25.8 (d, *J* = 4.5 Hz), 23.0, 22.7 (d, *J* = 4.5 Hz), 22.4 (d, *J* = 4.5 Hz), 13.9. **^{19}F-NMR** (564 MHz, $CDCl_3$) δ -168.12 (dd, *J* = 40.6, 8.5 Hz). **IR** (Film): 3057, 2969, 1674, 1469, 1259, 906, 726, 649 cm^{-1}. **HRMS** (ESI) m/z calcd. for $C_{17}H_{22}FO^-$ [M-H]$^-$: 261.1660; Found: 261.1660.

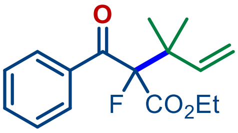

(186) According to the general procedure E, using ethyl 2,2-difluoro-3-oxo-3-phenylpropanoate *N*-triftosylhydrazone (135.0 mg, 0.3 mmol), 3-methylbut-2-en-1-ol (51.6 mg, 0.6 mmol), and DCE (4.0 mL) afforded compound **186** 58.4 mg, 70% yield) as a colorless oil; **^{1}H-NMR** (600 MHz, $CDCl_3$) δ 7.91 (d, *J* = 8.4 Hz, 2H), 7.54 (t, *J* = 7.2 Hz, 1H), 7.42 (t, *J* = 7.8 Hz, 2H), 6.27 (dd, *J* = 17.4, 10.8 Hz, 1H), 5.15-5.07 (m, 2H), 5.30-5.20 (m, 2H), 1.33 (d, *J* = 8.4 Hz, 6H), 1.21 (t, *J* = 7.2 Hz, 3H); **^{13}C-NMR** (150 MHz, $CDCl_3$) δ 192.4 (d, *J* = 28.5 Hz), 166.3 (d, J = 25.5 Hz), 142.1 (d, J = 4.5 Hz), 135.6 (d, J = 4.4 Hz), 133.2, 129.3 (d, J = 6.0 Hz), 128.3, 113.9, 101.4 (d, J = 205.5 Hz), 62.3, 44.3 (d, J = 21.0 Hz), 22.8 (d, J = 6.0 Hz), 22.6 (d, J = 4.5 Hz), 13.9; **^{19}F-NMR** (564

MHz, $CDCl_3$) δ -195.25 (s); **IR** (Film): 3089, 2982, 1751, 1701, 1230, 736, 691, 654 cm^{-1}.

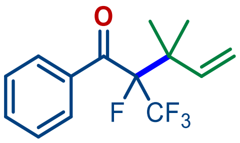

(187) According to the general procedure E, using 2,2,3,3,3-pentafluoro-1-phenylpropan-1-one *N*-triftosylhydrazone (133.8 mg, 0.3 mmol), 3-methylbut-2-en-1-ol (51.6 mg, 0.6 mmol), and DCE (4.0 mL) afforded compound **187** (75.6 mg, 92% yield) as a colorless oil; **^{1}H-NMR** (600 MHz, $CDCl_3$) δ 7.92-7.87 (m, 2H), 7.60-7.55 (m, 1H), 7.47-7.42 (m, 2H), 6.04 (dd, *J* = 18.0 10.8 Hz, 1H), 5.16-5.10 (m, 2H), 1.33 (s, 3H), 1.30 (s, 3H). **^{13}C-NMR** (150 MHz, $CDCl_3$) δ 195.0 (d, *J* = 27.0 Hz), 140.5, 136.6 (d, *J* = 4.5Hz), 133.2, 129.7 (d, *J* = 9.0 Hz), 128.3, 121.4 (td, *J* = 286.5, 30.0 Hz), 115.0, 43.5 (d, *J* = 21.0 Hz), 22.8 (d, *J* = 4.5 Hz), 22.5 (d, *J* = 4.5 Hz). **^{19}F-NMR** (564 MHz, $CDCl_3$) δ -69.28 (d, *J* = 6.5 Hz, 3F), -171.92 (q, *J* = 6.5 Hz, 1F). **HRMS** (ESI) m/z calcd. for $C_{14}H_{13}F_4O^-$ [M-H]$^-$: 273.0908; Found: 273.0910.

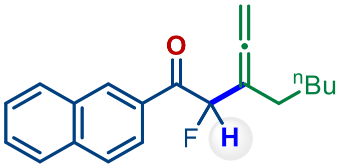

(188) According to the general procedure E, using 2,2-difluoro-1-(naphthalen-2-yl)ethan-1-one *N*-triftosylhydrazone (128.4 mg, 0.3 mmol), hept-2-yn-1-ol (67.2 mg, 0.6 mmol), and DCE (4.0 mL) afforded compound **188** (64.8 mg, 73% yield) as a colorless oil; **^{1}H-NMR** (500 MHz, $CDCl_3$) δ 8.50 (s, 1H), 8.00 (dd, *J* = 8.5, 1.5 Hz, 1H), 7.95 (d, *J* = 8.0 Hz, 1H), 7.92-7.86 (m, 2H), 7.65-7.60 (m, 1H), 7.56 (t, *J* = 7.5 Hz, 1H), 6.18 (d, *J* = 49.0 Hz, 1H), 4.90-4.86 (m, 2H), 2.23-1.98 (m, 2H), 1.53-1.40 (m, 2H), 1.30-1.24 (m, 4H), 0.87-0.81 (m, 3H). **^{13}C-NMR** (125 MHz, $CDCl_3$) δ 208.3 (d, *J* = 8.8 Hz), 193.7 (d, *J* = 20.5 Hz), 166.2 (d, *J* = 28.8 Hz), 135.8, 132.3, 131.3, 130.9 (d, *J* = 2.5 Hz), 129.7, 128.9, 128.5, 127.8, 126.9, 124.1 (d, *J* = 1.8 Hz), 100.9 (d, *J* = 21.8 Hz), 93.3 (d, *J* = 188.8 Hz), 78.7 (d, *J* = 2.8 Hz), 31.3, 27.1, 27.0, 22.4, 14.0. **^{19}F NMR** (470 MHz, $CDCl_3$) δ -184.05 (dt, *J* = 49.0, 10.2 Hz). **HRMS** (ESI) *m/z* calculated $C_{20}H_{21}FNaO$ 319.1474, found 319.1477.

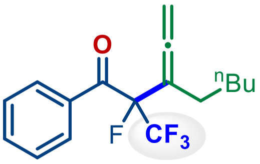

(189) According to the general procedure E, using 2,2,3,3,3-pentafluoro-1-phenylpropan-1-one *N*-triftosylhydrazone (133.8 mg, 0.3 mmol), hept-2-yn-1-ol (67.2 mg, 0.6 mmol), and DCE (4.0 mL) afforded compound **189** (66.9 mg, 71% yield) as a colorless oil; **^{1}H-NMR** (500 MHz, $CDCl_3$) δ 7.97 (d, *J* = 7.5 Hz, 2H), 7.60 (t, *J* = 7.5 Hz, 1H), 7.46 (t, *J* = 7.5 Hz, 2H), 5.13-5.06 (m, 1H), 4.98-4.91 (m, 1H), 2.33-2.20 (m, 1H), 2.19-2.10 (m, 1H), 1.55-1.45 (m, 2H), 1.38-1.26 (m, 4H), 0.93-0.83 (m, 3H). **^{13}C-NMR** (125 MHz, $CDCl_3$) δ 207.4 (d, *J* = 8.0 Hz), 189.9 (d, *J* = 25.0 Hz), 133.9, 133.7-133.5 (m), 129.8 (d, *J* = 5.0 Hz), 128.5, 122.0 (qd, *J* = 285.0, 31.3 Hz), 98.6 (d, *J* = 25.0 Hz), 95.9 (dq, *J* = 202.6, 29.5 Hz), 81.9, 31.2, 27.0, 25.6, 22.4, 13.9. **^{19}F NMR** (470 MHz, $CDCl_3$) δ -74.63 (d, *J* = 7.5 Hz), (-166.00)-(-166.22) (m). **HRMS** (ESI) *m/z* calculated $C_{17}H_{18}F_4NaO$ 337.1191, found 337.1194.

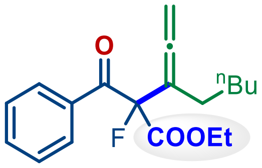

(190) According to the general procedure E, using ethyl 2,2-difluoro-3-oxo-3-phenylpropanoate *N*-triftosylhydrazone (135.0 mg, 0.3 mmol), hept-2-yn-1-ol (67.2 mg, 0.6 mmol), and DCE (4.0 mL) afforded compound **190** (57.2 mg, 60% yield) as a colorless oil; **^{1}H-NMR** (500 MHz, $CDCl_3$) δ 8.00 (d, *J* = 8.0 Hz, 2H), 7.58 (t, *J* = 7.0 Hz, 1H), 7.45 (t, *J* = 7.5 Hz, 2H), 5.03-4.95 (m, 1H), 4.92-4.85 (m, 1H), 4.35-4.24 (m, 2H), 2.20-2.03 (m, 2H), 1.56-1.45 (m, 2H), 1.36-1.29 (m, 4H), 1.27 (t, *J* = 7.0 Hz, 3H), 0.92-0.86 (m, 3H). **^{13}C-NMR** (125 MHz, $CDCl_3$) δ 207.2 (d, *J* = 8.8 Hz), 191.2 (d, *J* = 27.5 Hz), 166.2 (d, *J* = 28.8 Hz), 133.8 (d, *J* = 2.5 Hz), 133.7, 129.9 (d, *J* = 5.0 Hz), 128.4, 101.9 (d, *J* = 25.0 Hz), 99.7 (d, *J* = 198.8 Hz), 80.8 (d, *J* = 1.3 Hz), 62.6, 31.3, 27.1, 26.7, 22.4, 13.98, 13.94. **^{19}F NMR** (470 MHz, $CDCl_3$) δ -152.29 (t, *J* = 7.5 Hz). **HRMS** (ESI) *m/z* calculated $C_{19}H_{23}FNaO_3$ 341.1529, found 341.1531.

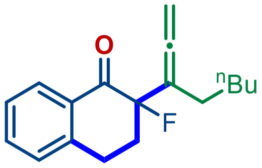

(191) According to the general procedure E, using 2,2-difluoro-3,4-dihydronaphthalen-1(2*H*)-one *N*-triftosylhydrazone (121.2 mg, 0.3 mmol), hept-2-yn-1-ol (67.2 mg, 0.6 mmol), and DCE (4.0 mL) afforded compound **191** (47.8 mg, 61% yield) as a colorless oil; **^{1}H-NMR** (500 MHz, $CDCl_3$) δ 8.02 (dd, *J* = 8.0, 1.5 Hz, 1H), 7.49 (td, *J* = 7.5, 1.5 Hz, 1H), 7.32 (t, *J* = 7.5 Hz, 1H), 7.21 (d, *J* = 8.0 Hz, 1H), 4.86-4.78 (m, 1H), 4.68-4.61 (m, 1H), 3.15-3.05 (m, 1H), 3.05-2.97 (m, 1H), 2.66-2.58 (m, 1H), 2.46-2.37 (m, 1H), 2.16-1.99 (m, 2H), 1.50-1.41 (m, 2H), 1.33-1.27 (m, 4H), 0.90-0.83 (m, 3H). **^{13}C-NMR** (125 MHz, $CDCl_3$) δ 206.9 (d, *J* = 7.5 Hz), 193.6 (d, *J* = 17.5 Hz), 142.4, 133.7, 131.8, 128.4, 127.85 (d, *J* = 1.3 Hz), 126.9, 100.9 (d, *J* = 25.0 Hz), 96.2 (d, *J* = 187.5 Hz), 79.4 (d, *J* = 2.5 Hz), 32.2 (d, *J* = 23.8 Hz), 31.4, 27.2, 26.8 (d, *J* = 10.0 Hz), 25.8, 22.4, 14.0. **^{19}F NMR** (470 MHz, $CDCl_3$) δ -150.87 (p, *J* = 9.4 Hz). **IR** (Film): 3059, 2931, 2257, 1953, 1700, 1603, 1456, 1263, 1067, 903, 722, 648 cm^{-1}. **HRMS** (ESI) *m/z* calculated $C_{18}H_{21}FNaO$ 295.1469, found 295.1469.

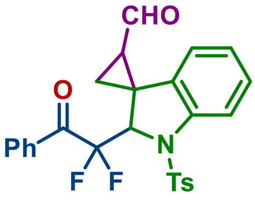

(192) According to the general procedure G, using **3** (87.8 mg, 0.2 mmol), DFHZ-Tfs (120.8 mg, 0.4 mmol), FeTPPCl (4.2 mg, 3 mol%), toluene (1.0 mL), and NaOH aq. (2 mL, 5.0 wt%) afforded compound **192** (86.6 mg, 90% yield) as a brown oil; **^{1}H NMR** (600 MHz, $CDCl_3$) δ 9.03 (d, *J* = 2.2 Hz, 1H), 8.08 (d, *J* = 7.8 Hz, 2H), 7.61 (t, *J* = 7.4 Hz, 1H), 7.51 (d, *J* = 7.9 Hz, 1H), 7.47 (t, *J* = 7.9 Hz, 2H), 7.28 (d, *J* = 8.1 Hz, 2H), 7.21 (t, *J* = 7.8 Hz, 1H), 7.15-7.07 (m, 3H), 6.64 (d, *J* = 1.1 Hz, 1H), 4.88 (dd, *J* = 13.9, 7.8 Hz, 1H), 2.35 (s, 3H), 2.29 (t, *J* = 6.2 Hz, 1H), 1.86 (dd, *J* = 8.7, 6.1 Hz, 1H), 1.64 (ddd, *J* = 8.6, 6.3, 2.2 Hz, 1H); **^{13}C NMR** (150 MHz, $CDCl_3$) δ 197.06,

188.39 (t, J = 26.6 Hz), 144.86, 141.82, 135.19, 134.26, 133.11, 130.31 (t, J = 3.5 Hz), 129.79, 128.89, 128.51, 127.28, 126.42, 118.79, 118.75, 117.09, 63.08 (t, J = 26.6 Hz), 38.57, 36.95, 21.56, 18.02; **^{19}F NMR** (564 MHz, $CDCl_3$) δ -103.76 (d, J = 262.9 Hz), -108.63 (d, J = 262.9 Hz); **IR** (Film): 3054, 2963, 1700, 1598, 1427, 1367, 1265, 1172, 1091, 903, 814, 743, 718 cm^{-1}.

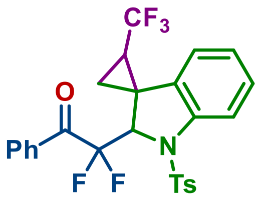

(193) According to the general procedure G, using **3** (87.8 mg, 0.2 mmol), TFHZ-Tfs (128.0 mg, 0.4 mmol), FeTPPCl (4.2 mg, 3 mol%), K_2CO_3 (82.8 mg, 0.6 mmol), and 1,4-dioxane (3.0 mL) afforded compound **193** (85.4 mg, 82% yield) as a light yellow oil; **^{1}H NMR** (500 MHz, $CDCl_3$) δ 7.98 (d, J = 8.0 Hz, 2H), 7.63-7.54 (m, 3H), 7.47 (d, J = 8.0 Hz, 1H), 7.43 (dd, J = 8.5, 7.5 Hz, 2H), 7.19 (d, J = 8.0 Hz, 2H), 7.15 (td, J = 8.0, 1.5 Hz, 1H), 7.00 (td, J = 7.5, 1.0 Hz, 1H), 6.74 (d, J = 8.0 Hz, 1H), 4.68 (dd, J = 13.0, 9.0 Hz, 1H), 2.33 (s, 3H), 1.92 (t, J = 8.5 Hz, 1H), 1.65 (t, J = 7.0 Hz, 1H), 1.59 (dd, J = 9.0, 7.0 Hz, 1H); **^{13}C NMR** (125 MHz, $CDCl_3$) δ 189.30 (t, J = 27.9 Hz), 145.10, 142.69, 134.60, 134.31, 132.55, 130.38 (t, J = 3.9 Hz), 130.05, 129.89, 128.84, 128.55, 127.27, 125.38, 122.36 (q, J = 3.7 Hz), 118.16, 116.42, 69.33 (t, J = 25.5 Hz), 30.69 (q, J = 37.2 Hz), 28.91, 21.44, 11.59; **^{19}F NMR** (564 MHz, $CDCl_3$) δ -60.20 (d, J = 7.6 Hz), -103.08 (dd, J = 278.3, 9.0 Hz), -106.64 (dd, J = 278.3, 12.3 Hz); **IR** (Film): 3054, 2971, 1696, 1597, 1424, 1365, 1264, 1171, 1092, 905, 814, 721 cm^{-1}.

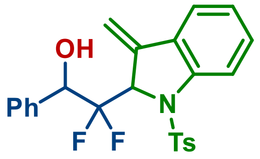

(194) According to the general procedure H, using **3** (87.8 mg, 0.2 mmol), sodium borohydride (9.1 mg, 0.24 mmol), and methanol (2.0 mL) afforded compound **194** (75.0 mg, 85% yield) as a colorless oil; **^{1}H NMR** (500 MHz, $CDCl_3$) δ 7.61 (d, J = 8.0 Hz, 1H), 7.43 (d, J = 8.5 Hz, 4H), 7.36-7.31 (m, 3H), 7.29 (t, J = 7.5 Hz, 2H), 7.13-7.08 (m, 3H), 5.54 (s, 1H), 5.32 (s, 1H), 5.27 (dd, J = 14.5, 9.0 Hz, 1H), 5.15 (dt, J = 19.5, 5.0 Hz, 1H), 2.89 (d, J = 5.5 Hz, 1H), 2.32 (s, 3H); **^{13}C NMR** (125 MHz, $CDCl_3$) δ 144.4, 144.2, 139.19, 139.16, 135.9, 133.5, 131.6, 129.9, 129.5, 128.7, 128.1, 127.5, 125.7, 120.7, 119.2 (dd, J = 256.3, 248.8 Hz), 118.9, 108.8, 72.2 (dd, J = 31.3, 22.5 Hz), 67.6 (t, J = 26.3 Hz), 21.5; **^{19}F NMR** (470 MHz, $CDCl_3$) δ -113.19 (dd, J = 258.0, 13.6 Hz), -121.13 (ddd, J = 258.0, 19.3, 8.9 Hz).

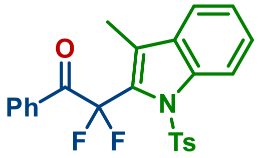

(195) Using **3** (87.8 mg, 0.2 mmol), NaI (48 mg, 0.32 mmol), TMSCl (34.8 mg, 0.32 mmol) and H_2O (2.9 mg, 0.16 mmol), and acetonitrile (2.0 mL) afforded compound **195** (84.3 mg, 96% yield) as a white solid; mp: 153-154; **^{1}H NMR** (600 MHz, $CDCl_3$) δ 7.95 (d, J = 8.3 Hz, 1H), 7.67 (d, J = 8.5 Hz, 1H), 7.61 (t, J = 7.4 Hz, 1H), 7.53 (d, J = 7.7 Hz, 1H), 7.50 (t, J = 7.8 Hz, 1H), 7.34 (dd, J = 8.5, 7.2 Hz, 1H), 7.26 (t, J = 7.1 Hz, 1H), 7.14 (d, J = 8.2 Hz, 1H), 2.47 (t, J = 3.9 Hz, 1H), 2.28 (s, 1H); **^{13}C NMR** (150 MHz, $CDCl_3$) δ 188.1 (t, J = 31.7 Hz), 136.6, 135.1, 133.6, 133.1, 130.5, 129.9 (d, J = 3.4 Hz), 129.6, 128. 6, 127.23, 126.7, 126.5, 125.5, 123.8, 120.1, 116.6, 114. 9, 21.6, 10.1; **^{19}F NMR** (564 MHz, $CDCl_3$) δ -87.39; **IR** (Film): 3069, 2961, 1707, 1598, 1449, 1365, 1244, 1175, 1064, 903, 877, 724 cm^{-1}.

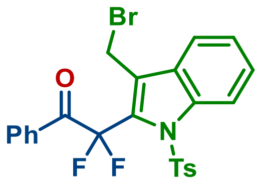

(196) Using **3** (87.8 mg, 0.2 mmol), *N*-bromosuccinimide (39.2 mg, 0.22 mmol), and DCM (2.0 mL) afforded compound **196** (76.5 mg, 74% yield) as a yellow oil; **^{1}H NMR** (500 MHz, $CDCl_3$) δ 8.18 (d, *J* = 7.5 Hz, 2H), 7.94 (d, *J* = 8.5 Hz, 1H), 7.71 (t, *J* = 8.5 Hz, 3H), 7.64 (t, *J* = 7.5 Hz, 1H), 7.53 (t, *J* = 7.5 Hz, 2H), 7.39 (t, *J* = 7.5 Hz, 1H), 7.34 (t, *J* = 7.5 Hz, 1H), 7.20 (d, *J* = 8.5 Hz, 2H), 4.83 (s, 2H), 2.32 (s, 3H); **^{13}C NMR** (150 MHz, $CDCl_3$) δ 187.4 (t, *J* = 31.5 Hz), 145.4, 136.4, 134.9, 133.9, 132.5, 129.9, 129.8, 128.6, 127.8, 127.3, 127.1, 126.8, 124.3, 124.1, 120.0, 116.4 (t, *J* = 253.5 Hz), 114.8, 21.6, 21.28 (t, *J* = 9.0 Hz); **^{19}F NMR** (564 MHz, $CDCl_3$) δ -88.70 (s).

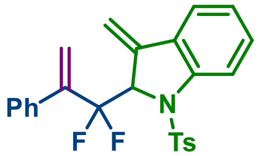

(197) According to the general procedure I, using **3** (87.8 mg, 0.2 mmol), potassium *tert*-butoxide (33.7 mg, 0.3 mmol), Ph_3PCH_3Br (107.1 mg, 0.3 mmol), and THF (1.5 mL) afforded compound **197** (69.9 mg, 80% yield) as a colorless oil; **^{1}H NMR** (500 MHz, $CDCl_3$) δ 7.46 (d, *J* = 8.0 Hz, 1H), 7.36-7.31 (m, 4H), 7.30-7.25 (m, 3H), 7.23-7.18 (m, 1H), 7.13 (d, *J* = 7.5 Hz, 1H), 7.03 (dd, *J* = 14.0, 7.5 Hz, 3H), 5.72 (d, *J* = 2.0 Hz, 1H), 5.58 (d, *J* = 2.5 Hz, 1H), 5.34 (s, 1H), 5.05 (dd, *J* = 13.0, 7.0 Hz, 1H), 4.97 (s, 1H), 2.27 (s, 3H); **^{13}C NMR** (125 MHz, $CDCl_3$) δ 144.2, 144.2, 140.9 (t, *J* = 21.9 Hz), 138.7 (d, *J* = 3.0 Hz), 136.3, 134.0, 131.4, 129.8, 129.5, 128.4, 128.2, 128.2, 127.4, 125.3, 122.1 (t, *J* = 9.1 Hz), 120.6, 119.6, 118.6, 108.5, 70.44-66.14 (m), 21.5; **^{19}F NMR** (470 MHz, $CDCl_3$) δ -99.62 (d, *J* = 249.8 Hz), -104.83 (d, *J* = 250.6 Hz); **IR** (Film): 3066, 1731, 1599, 1447, 1365, 1234, 1172, 975, 813, 734, 717 cm^{-1}.

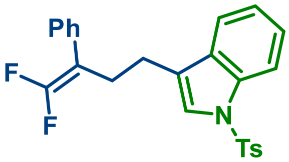

(198) Using **3** (87.8 mg, 0.2 mmol), potassium *tert*-butoxide (33.7 mg, 0.3 mmol), Ph_3PCH_3Br (107.1 mg, 0.3 mmol), and THF (1.5 mL) afforded compound **198** (66.4 mg, 76% yield) as a white solid; mp: 140-141; **^{1}H NMR** (600 MHz, $CDCl_3$) δ 7.98 (d, *J* = 8.4 Hz, 1H), 7.73 (d, *J* = 8.4 Hz, 2H), 7.39-7.34 (m, 3H), 7.32-7.26 (m, 5H), 7.22-7.19 (m, 3H), 2.78-2.74 (m, 2H), 2.73-2.68 (m, 2H), 2.33 (s, 3H); **^{13}C NMR** (150 MHz, $CDCl_3$) δ 153.7 (dd, *J* = 289.5, 286.5 Hz), 144.8, 135.3 (d, *J* = 6.0 Hz), 133.1 (t, *J* = 4.5 Hz), 130.7, 129.8, 128.6, 128.2 (t, *J* = 3.0 Hz), 127.5, 126.7, 124.7, 123.0, 122.9, 121.8, 119.2, 113.8, 91.7 (dd, *J* = 21.0, 13.5 Hz), 27.4, 23.1, 21.5; **^{19}F NMR** (564 MHz, $CDCl_3$) δ -90.80 (d, *J* = 41.7 Hz), -91.14 (d, *J* = 41.7 Hz); **IR** (Film): 3057, 1733, 1447, 1366, 1263, 1173, 973, 896, 731 cm^{-1}.

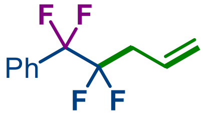

(199) According to the general procedure J, using **86** (39.2 mg, 0.2 mmol), DAST (32.2 mg, 0.2 mmol), and DCM (2.0 mL) afforded compound **199** (33.6 mg, 77% yield) as a colorless oil; **^{1}H-NMR** (500 MHz, $CDCl_3$) δ 7.63-7.58 (m, 2H), 7.56-7.51 (m, 1H), 7.51-7.45 (m, 2H), 5.96-5.80 (m, 1H), 5.36-5.24 (m, 2H), 2.93-2.80 (m, 2H). **^{13}C-NMR** (125 MHz, $CDCl_3$) δ 131.0 (t, *J* = 2.5 Hz), 128.3, 127.0 (t, *J* = 5.0 Hz), 126.7 (t, *J* = 6.3 Hz), 121.2, 117.8 (t, *J* = 250.0, 37.5 Hz), 116.7 (t, *J* = 250.0, 33.8 Hz), 35.5 (t, *J* = 23.8 Hz). **^{19}F-NMR** (470 MHz, $CDCl_3$) δ

-111.78 - -111.88 (m, 2F), -113.96 - -114.10 (m, 2F). **HRMS** (ESI) m/z calcd. for $C_{11}H_{11}F_4^+$ $[M+H]^+$: 219.0797; Found: 219.0795.

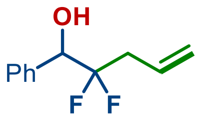

(200) According to the general procedure H, using **86** (39.2 mg, 0.2 mmol), sodium borohydride (9.1 mg, 0.24 mmol), and methanol (2.0 mL) afforded compound **200** (36.4 mg, 92% yield) as a colorless oil; **^{1}H-NMR** (600 MHz, $CDCl_3$) δ 7.48-7.43 (m, 2H), 7.41-7.35 (m, 3H), 5.87-5.78 (m, 1H), 5.23 (d, *J* = 10.2, 1H), 5.18 (d, *J* = 17.4, 1H), 4.87 (td, *J* = 10.2, 3.6 Hz, 1H), 2.79-2.67 (m, 1H), 2.54-2.42 (m, 2H). **^{13}C-NMR** (150 MHz, $CDCl_3$) δ 136.4 (t, *J* = 3.0 Hz), 128.7, 128.7 (t, *J* = 6.0 Hz), 128.4, 127.5, 122.4 (t, *J* = 244.5 Hz), 120.5, 75.0 (t, *J* = 27.0 Hz), 37.1 (t, *J* = 24.0 Hz). **^{19}F-NMR** (564 MHz, $CDCl_3$) δ -108.33 - -109.47 (m).

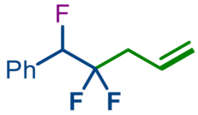

(201) According to the general procedure J, using **200** (40.0 mg, 0.2 mmol), DAST (32.2 mg, 0.2 mmol), and DCM (2.0 mL) afforded compound **201** (34.0 mg, 85% yield) as a colorless oil; **^{1}H-NMR** (600 MHz, $CDCl_3$) δ 7.45-7.37 (m, 5H), 5.88-5.79 (m, 1H), 5.47 (ddd, *J* = 44.4, 13.0, 7.2 Hz, 1H), 5.30-5.22 (m, 2H), 2.84-2.56 (m, 2H); **^{13}C-NMR** (150 MHz, $CDCl_3$) δ 132.6 (d, *J* = 21.0 Hz), 129.5, 128.4, 128.0 (dd, *J* = 7.5, 4.5 Hz), 127.1 (d, *J* = 7.5 Hz), 121.2, 12.01 (td, *J* = 244.5, 28.5 Hz), 90.8 (ddd, *J* = 181.5, 33.0, 30.0 Hz), 37.4 (t, *J* = 24.0 Hz). **^{19}F-NMR** (564 MHz, $CDCl_3$) δ -108.48 - -111.40 (m, 2F), -193.68 - -193.86 (m, 1F). **HRMS** (ESI) m/z calcd. for $C_{11}H_{10}F_3^-$ $[M-H]^-$: 199.0740; Found: 199.0730.

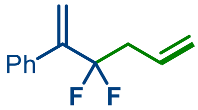

(202) According to the general procedure I, using **86** (39.2 mg, 0.2 mmol), potassium *tert*-butoxide (33.7 mg, 0.3 mmol), Ph_3PCH_3Br (107.1 mg, 0.3 mmol), and THF (1.5 mL) afforded compound **202** (33.0 mg, 85% yield) as a colorless oil; **^{1}H-NMR** (500 MHz, $CDCl_3$) δ 7.44-7.40 (m, 2H), 7.39-7.34 (m, 3H), 5.80-5.67 (m, 2H), 5.51-5.747 (m, 1H), 5.20-5.14 (m, 1H), 5.10-5.04 (m, 1H), 2.69 (td, *J* = 16.0, 7.0 Hz, 2H). **^{13}C-NMR** (150 MHz, $CDCl_3$) δ 144.4 (t, *J* = 22.5 Hz), 137.0, 129.0 (t, *J* = 4.5 Hz), 128.3, 128.22, 128.21, 121.3 (t, *J* = 243.0 Hz), 120.2, 118.4 (t, *J* = 9.0 Hz), 41.0 (t, *J* = 27.0 Hz). **^{19}F-NMR** (564 MHz, $CDCl_3$) δ -95.59 (t, *J* = 16.0 Hz).

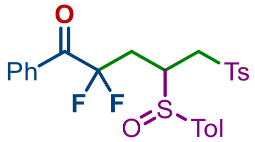

(203) Using **86** (39.2 mg, 0.2 mmol), $TolSO_2Na$ (213.6 mg, 1.2 mmol), CH_3COCl (62.8 mg, 0.8 mmol) and $CHCl_3$ (2.0 mL) afforded compound **203** (55.1 mg, 69% yield) as a colorless oil; dr = 5:4; **^{1}H-NMR** (500 MHz, $CDCl_3$) δ 8.14 (d, *J* = 8.0 Hz, major, 2H), 8.01 (d, *J* = 8.0 Hz, minor, 2H), 7.81 (d, *J* = 8.5 Hz, minor, 2H), 7.70-7.62 (m, major, 1H, minor, 1H), 7.53 (t, *J* = 8.0 Hz, major, 2H), 7.48 (t, *J* = 7.5 Hz, minor, 2H), 7.46-7.42 (m, major, 2H, minor, 2H), 7.37 (d, *J* = 8.0 Hz, minor, 2H), 7.32 (d, *J* = 8.0 Hz, major, 2H), 7.26 (d, *J* = 8.0 Hz, minor, 2H), 7.20 (t, *J* = 8.0 Hz, major, 4H), 4.03 (dd, *J* = 15.0, 5.0 Hz, minor, 1H), 3.60-3.53 (m, minor, 1H), 3.23-3.14 (m, major, 2H, minor, 1H), 3.14-3.00 (m, major, 2H), 2.96 (dd, *J* = 14.9, 8.0 Hz, minor, 1H), 2.92-2.77 (m, minor, 1H), 2.46-2.38 (m, major, 6H, minor, 6H), 2.34-2.17 (m, major, 1H). **^{13}C-NMR** (125 MHz, $CDCl_3$) δ187.2 (t, *J* = 30.0 Hz), 186.8 (t, *J* = 30.0 Hz), 144.4, 144.0, 141.6, 140.8, 136.1, 134.4, 134.2, 133.8, 133.8, 133.7, 130.4, 130.1, 129.3-129.2 (m), 129.2, 129.1, 129.0, 128.9, 127.9, 127.8, 127.2, 126.9, 124.2, 123.0, 117.5 (t, *J* = 254.5 Hz), 51.9, 50.6

(t, J = 6.9 Hz), 31.8 (t, J = 22.6 Hz), 29.9 (t, J = 22.6 Hz), 20.7, 20.6, 20.5. **^{19}F NMR** (470 MHz, $CDCl_3$) δ -95.07 (ddd, J = 290.0, 27.7,10.8 Hz, minor, 1F), -97.36 (ddd, J = 284.8, 23.0, 12.2 Hz, major, 1F), -97.55 (ddd, J = 290.0, 24.4, 11.3 Hz, minor, 1F), -98.33 (ddd, J = 284.8, 21.2, 14.6 Hz, major, 1F).

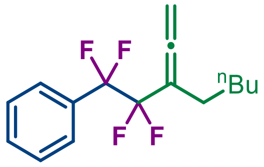

(204) According to the general procedure J, using **127** (52.8 mg, 0.2 mmol), DAST (32.2 mg, 0.2 mmol), and DCM (2.0 mL) afforded compound **204** (55.5 mg, 97% yield) as a colorless oil; **^{1}H NMR** (600 MHz, CDCl3) δ 7.55 (d, J = 7.7 Hz, 2H), 7.49 (t, J = 7.4 Hz, 1H), 7.43 (t, J = 7.6 Hz, 2H), 4.96-4.91 (m, 2H), 2.09-2.04 (m, 2H), 1.49-1.40 (m, 2H), 1.34-1.26 (m, 4H), 0.92-0.84 (m, 3H). **^{13}C NMR** (150 MHz, $CDCl_3$) δ 208.2 (t, J = 7.1 Hz), 131.2 (t, J = 24.8 Hz), 130.8, 128.1, 126.9 (t, J = 6.4 Hz), 117.2 (tt, J = 254.0, 36.3 Hz), 115.8 (t, J = 254.0, 36.0 Hz), 98.9 (t, J = 26.8 Hz), 80.6, 31.2, 27.2, 26.2, 22.4, 14.0. **^{19}F NMR** (564 MHz, $CDCl_3$) δ -110.0 (d, J = 2.9 Hz), -111.3 (d, J = 3.2 Hz). **IR** (Film): 3055, 2958, 2931, 2861, 2253, 1953, 1607, 1453, 1263, 1131, 1077, 905, 725, 649 cm^{-1}. **HRMS** (ESI) *m/z* calculated $C_{16}H_{18}F_4Na$ 309.1241, found 309.1245.

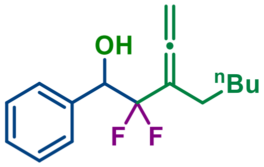

(205) According to the general procedure H, using **127** (52.8 mg, 0.2 mmol), sodium borohydride (9.1 mg, 0.24 mmol), and methanol (2.0 mL) afforded compound **205** (48.4 mg, 91% yield) as a colorless oil; **^{1}H NMR** (600 MHz, $CDCl_3$) δ 7.44-7.39 (m, 2H), 7.37-7.30 (m, 3H), 5.00-4.84 (m, 3H), 2.66 (s, 1H), 1.99-1.89 (m, 2H), 1.43-1.33 (m, 2H), 1.32-1.19 (m, 4H), 0.86 (t, J = 7.0 Hz, 3H). **^{13}C NMR** (150 MHz, $CDCl_3$) δ 206.9 (t, J = 7.8 Hz), 136.2, 128.6, 128.0, 127.9, 120.3 (t, J = 249.1 Hz), 101.4 (t, J = 28.4 Hz), 80.4, 75.3 (t, J = 29.2 Hz), 31.2, 27.0, 26.2, 22.3, 14.0. **^{19}F NMR** (564 MHz, $CDCl_3$) δ (-104.22)-(-108.06) (m). **HRMS** (ESI) *m/z* calculated $C_{16}H_{20}F_2NaO$ 289.1384, found 289.1387.

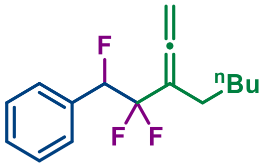

(206) According to the general procedure J, using **205** (53.2 mg, 0.2 mmol), DAST (32.2 mg, 0.2 mmol), and DCM (2.0 mL) afforded compound **206** (46.6 mg, 87% yield) as a colorless oil; **^{1}H NMR** (500 MHz, $CDCl_3$) δ 7.48-7.34 (m, 5H), 5.58 (ddd, J = 44.0, 12.5, 7.5 Hz, 1H), 5.07-4.90 (m, 2H), 2.08-1.94 (m, 2H), 1.47-1.37 (m, 2H), 1.33-1.20 (m, 4H), 0.88 (t, J = 7.0 Hz, 3H). **^{13}C NMR** (125 MHz, $CDCl_3$) δ 207.3 (t, J = 8.0 Hz), 132.8 (d, J = 20.5 Hz), 129.4, 128.1, 127.6 (d, J = 6.9 Hz), 118.3 (td, J = 248.9, 27.6 Hz), 100.8 (t, J = 27.9 Hz), 91.8 (ddd, J = 184.3, 34.2, 30.3 Hz), 80.8, 31.3, 27.1, 26.1, 22.4, 14.0. **^{19}F NMR** (470 MHz, $CDCl_3$) δ (-103.84)-(-110.33) (m), (-189.58)-(-190.91) (m). **IR** (Film): 3057, 2933, 2357, 2253, 1966, 1456, 1265, 1087, 906, 725, 649 cm^{-1}. **HRMS** (ESI) *m/z* calculated $C_{16}H_{19}F_3Na$ 291.1334, found 291.1339.

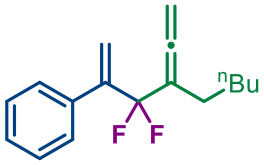

(207) According to the general procedure I, using **127** (52.8 mg, 0.2 mmol), potassium *tert*-butoxide (33.7 mg, 0.3 mmol), Ph_3PCH_3Br (107.1 mg, 0.3 mmol), and THF (1.5 mL) afforded compound **207** (41.9 mg, 80% yield) as a colorless oil; **^{1}H NMR** (600 MHz, $CDCl_3$) δ 7.37-7.31 (m, 2H), 7.28-7.20 (m, 3H), 5.68 (s, 1H), 5.53 (s, 1H), 4.85-4.76 (m, 2H), 2.01-1.91 (m, 2H), 1.39-1.29 (m, 2H), 1.25-1.13 (m, 4H), 0.79 (t, *J* = 7.0 Hz, 3H). **^{13}C NMR** (150 MHz, $CDCl_3$) δ 207.3 (t, *J* = 7.4 Hz), 144.1 (t, *J* = 24.9 Hz), 136.7, 128.01, 127.99, 127.98, 120.1 (t, *J* = 243.9 Hz), 118.7 (t, *J* = 8.1 Hz), 103.3 (t, *J* = 30.0 Hz), 80.4, 31.3, 27.0, 25. 9, 22.4, 14.0. **^{19}F NMR** (564 MHz, $CDCl_3$) δ -92.3 (t, *J* = 5.0 Hz). **IR** (Film): 3055, 2930, 2253, 1955,1700, 1422, 1265,904,720,649 cm^{-1}. **HRMS** (ESI) *m/z* calculated $C_{17}H_{20}F_2Na$ 285.1431, found 285.1433.

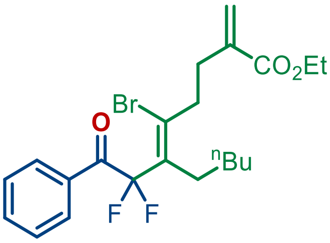

(208) Using **127** (52.8 mg, 0.2 mmol), AIBN (6.7 mg, 0.04 mmol), ethyl bromomethacrylate (77.2 mg, 0.4 mmol) and toluene (2.0 mL) afforded compound **208** (69.3 mg, 76% yield) as a colorless oil; **^{1}H NMR** (500 MHz, $CDCl_3$) **major:** δ 8.08 (d, *J* = 7.7 Hz, 2H), 7.68 (t, *J* = 7.5 Hz, 1H), 7.54 (t, *J* = 7.8 Hz, 2H), 6.19 (s, 1H), 5.55 (s, 1H), 4.18 (q, *J* = 7.1 Hz, 2H), 2.77-2.72 (m, 2H), 2.65-2.57 (m, 2H), 2.48-2.38 (m, 2H), 1.55-1.45 (m, 2H), 1.43-1.32 (m, 4H), 1.28 (t, 7.3 Hz, 3H), 0.94 (m, 3H). **minor:** δ 8.05 (d, *J* = 7.8 Hz, 2H), 7.64 (t, *J* = 7.5 Hz, 1H), 7.51 (t, *J* = 7.9 Hz, 2H), 6.18 (s, 1H), 5.56 (s, 1H), 4.27 (q, *J* = 7.1 Hz, 2H), 2.83 (t, *J* = 7.4 Hz, 2H), 2.61-2.57 (m, 2H), 2.43-2.38 (m, 2H), 1.61-1.49 (m, 2H), 1.43-1.32 (m, 4H), 1.31-1.23 (m, 3H), 0.99-0.86 (m, 3H). **^{13}C NMR** (125 MHz, $CDCl_3$) **major:** δ 187.9 (t, *J* = 32.7 Hz), 166.6, 138.8, 135.7 (t, *J* = 6.4 Hz), 134.4, 133.6, 133.3 (t, *J* = 22.0 Hz), 130.0 (t, *J* = 2.7 Hz), 128.7, 125.9, 117.3 (t, *J* = 257.9 Hz), 60.6, 38.6, 34.0 (t, *J* = 4.3 Hz), 31.7, 31.2, 27.6, 22.2, 14.1, 13.9. **minor:** δ 188.4 (t, *J* = 32.7 Hz), 166.7, 138.1, 135.7 (t, *J* = 6.4 Hz), 133.6, 132.0, 129.6, 128.4, 126.8, 116.8 (t, *J* = 254.8 Hz), 60.8, 37.7, 31.6, 30.8, 30.0 (t, *J* = 4.9 Hz), 28.8, 22.3, 14.2, 14.1. **^{19}F NMR** (600 MHz, $CDCl_3$) **major:** δ -91.26 (s, 1H). **minor:** -94.04 (s, 1H). **IR** (Film): 3055, 2963, 2359, 2257, 1709, 1449, 1263, 1204, 1092, 905, 725, 649 cm^{-1}. **HRMS** (ESI) *m/z* calculated $C_{22}H_{27}BrF_2NaO_3$ 479.1004, found 479.1005.

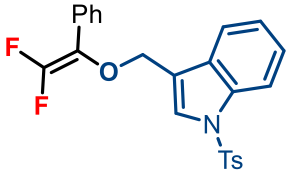

(209) Using *N*-triftosylhydrazone derived from 2,2,2-trifluoro-1-phenylethan-1-one (118.8 mg, 0.3 mmol), (1-tosyl-1*H*-indol-3-yl)methanol (180.8 mg, 0.6 mmol), K_2CO_3 (82.9 mg, 0.6 mmol), $Tp^{Br3}Ag$ (33.0 mg, 10 mol%) and toluene (4.0 mL) (40 minutes of reaction) afforded compound **209** (81.7 mg, 62% yield) as a colorless oil; **^{1}H NMR** (500 MHz, $CDCl_3$) δ 7.98 (d, *J* = 8.5 Hz, 1H), 7.75 (d, *J* = 8.0 Hz, 2H), 7.58 (d, *J* = 7.5 Hz, 1H), 7.52 (s, 1H), 7.44 (d, *J* = 8.0 Hz, 2H), 7.40 (t, *J* = 7.5 Hz, 2H), 7.36-7.31 (m, 2H), 7.28-7.24 (m, 1H), 7.22 (d, *J* = 8.0 Hz, 2H), 4.85 (s, 2H), 2.33 (s, 3H); **^{13}C NMR** (150 MHz, $CDCl_3$) δ 155.5 (t, *J* = 289.5 Hz), 145.1, 135.2, 135.1, 129.9, 129.7, 128.7, 128.3, 126.8, 126.6 (dd, *J* = 6.0, 4.5 Hz), 125.6, 125.0, 123.4, 119.8, 117.9, 117.5 (dd, *J* = 33.0, 18.0 Hz), 113.6, 64.9 (t, *J* = 3.0 Hz), 21.5; **^{19}F NMR** (564 MHz, $CDCl_3$) δ -96.74 (d, *J* = 55.8 Hz), -107.27 (d, *J* = 54.7

Hz); **IR** (Film): 3054, 1701, 1598, 1449, 1372, 1264, 1174, 1093, 903, 812, 719 cm^{-1}; **HRMS** (ESI^+) m/z calcd for $C_{24}H_{19}F_2NNaO_3S$ $[M+Na]^+$ 462.0946, found 462.0948.

VIII. Copies of ^{1}H, ^{13}C and ^{19}F Spectra

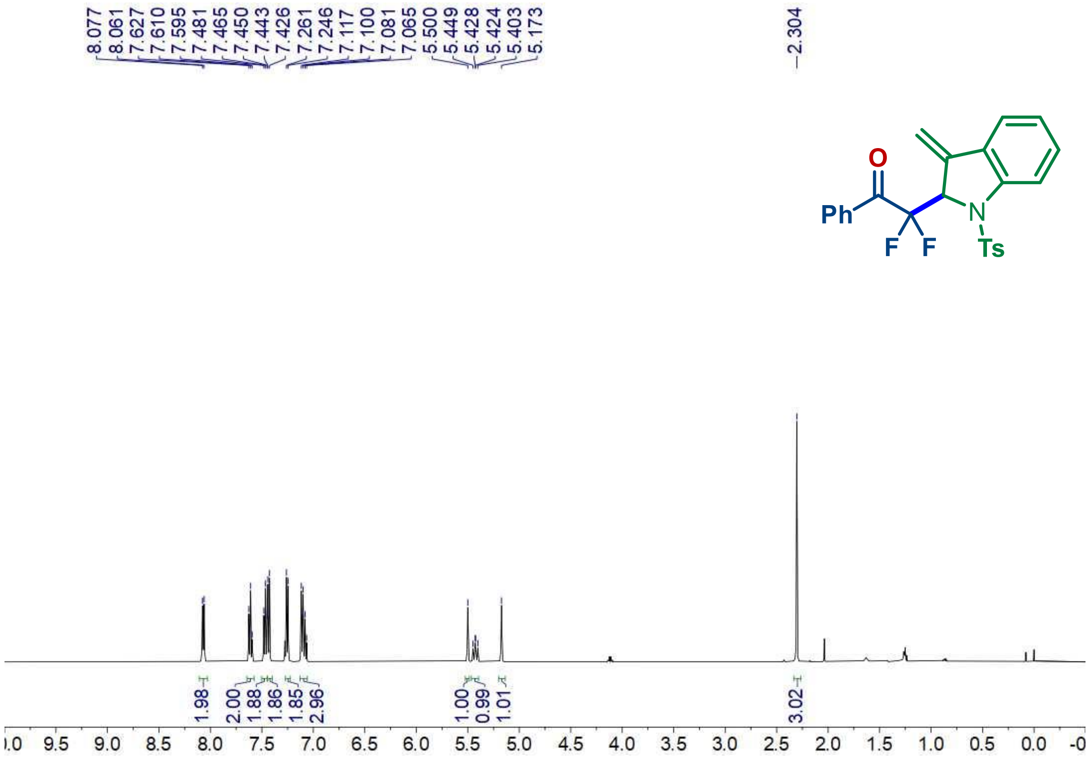

Supplementary Fig. 11 ^{1}H NMR (500 MHz, $CDCl_3$) spectrum of compound **3**

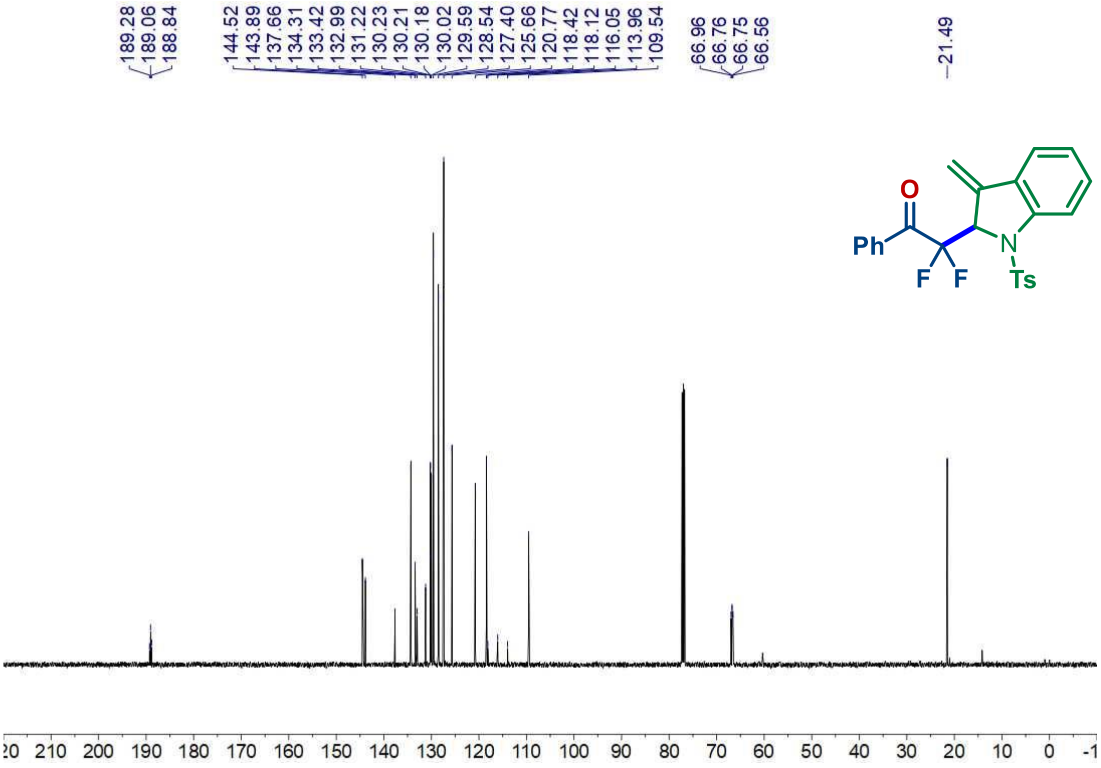

Supplementary Fig. 12 ^{13}C NMR (125 MHz, $CDCl_3$) spectrum of compound **3**

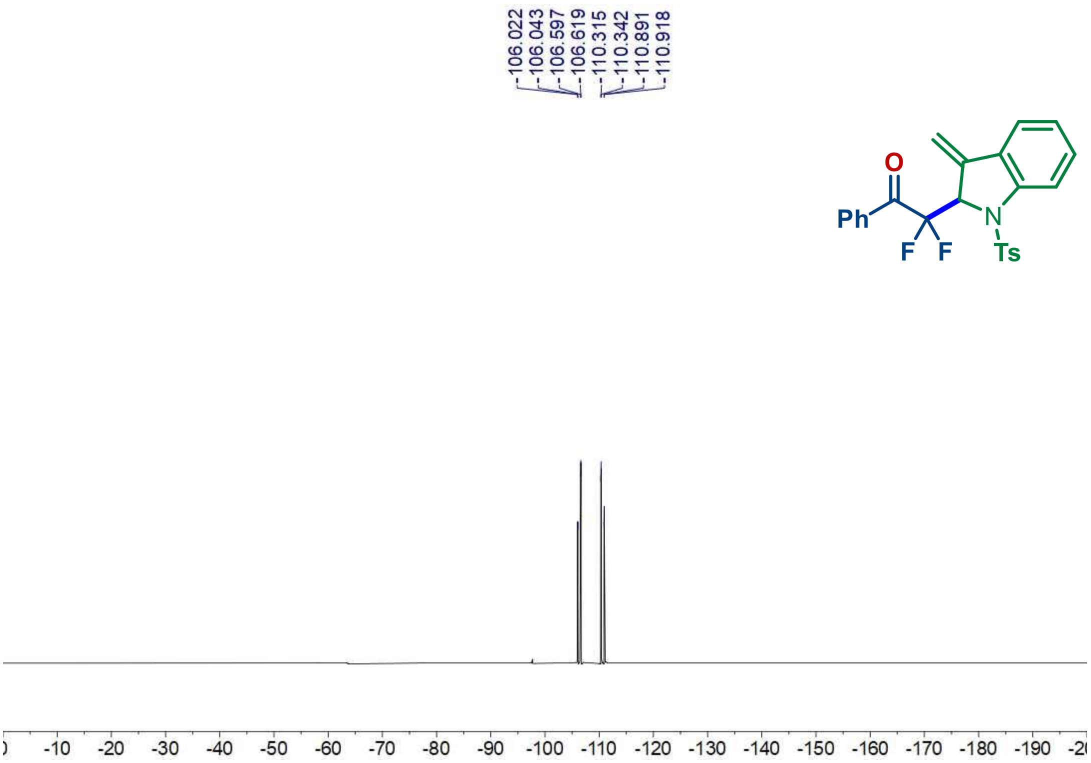

Supplementary Fig. 13 ^{19}F NMR (470 MHz, $CDCl_3$) spectrum of compound **3**

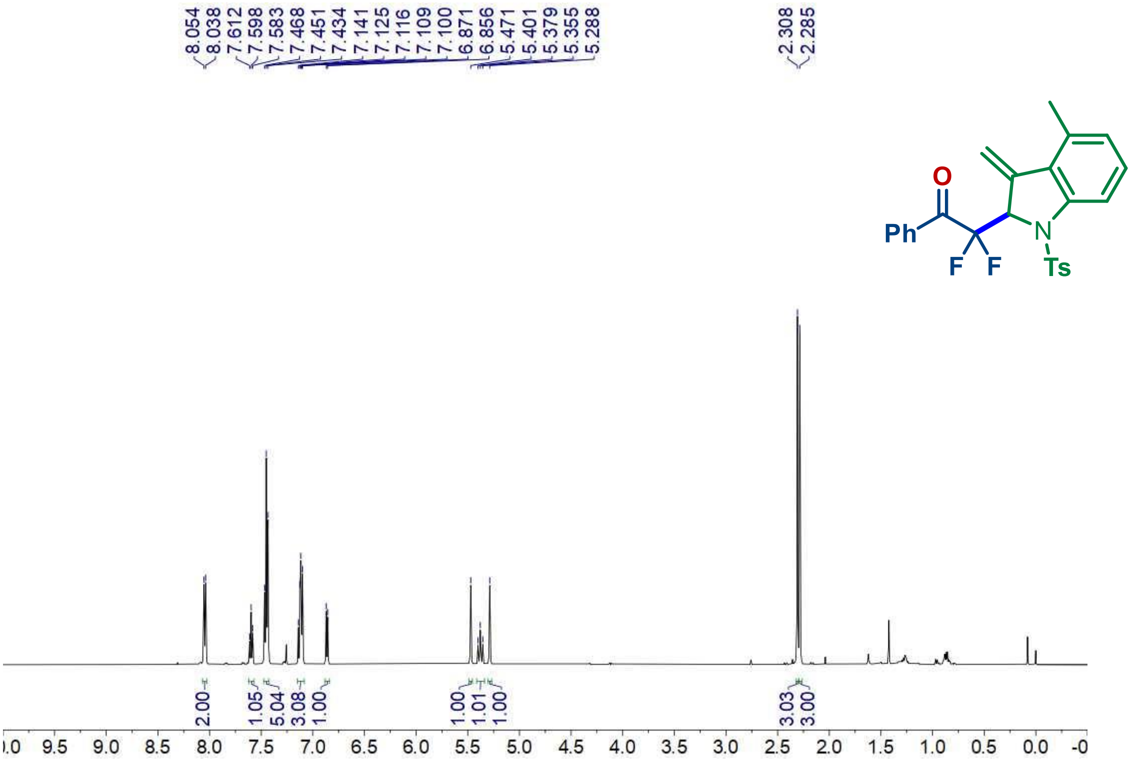

Supplementary Fig. 14 ^{1}H NMR (500 MHz, $CDCl_3$) spectrum of compound **4**

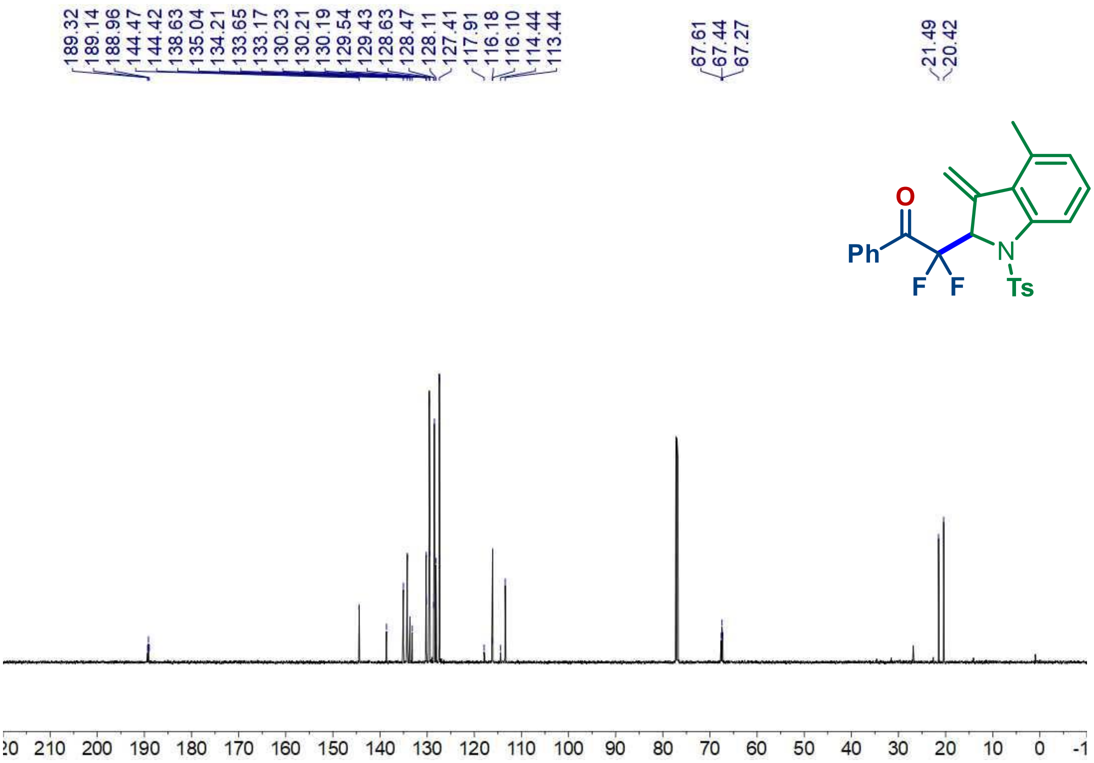

Supplementary Fig. 15 ^{13}C NMR (150 MHz, $CDCl_3$) spectrum of compound **4**

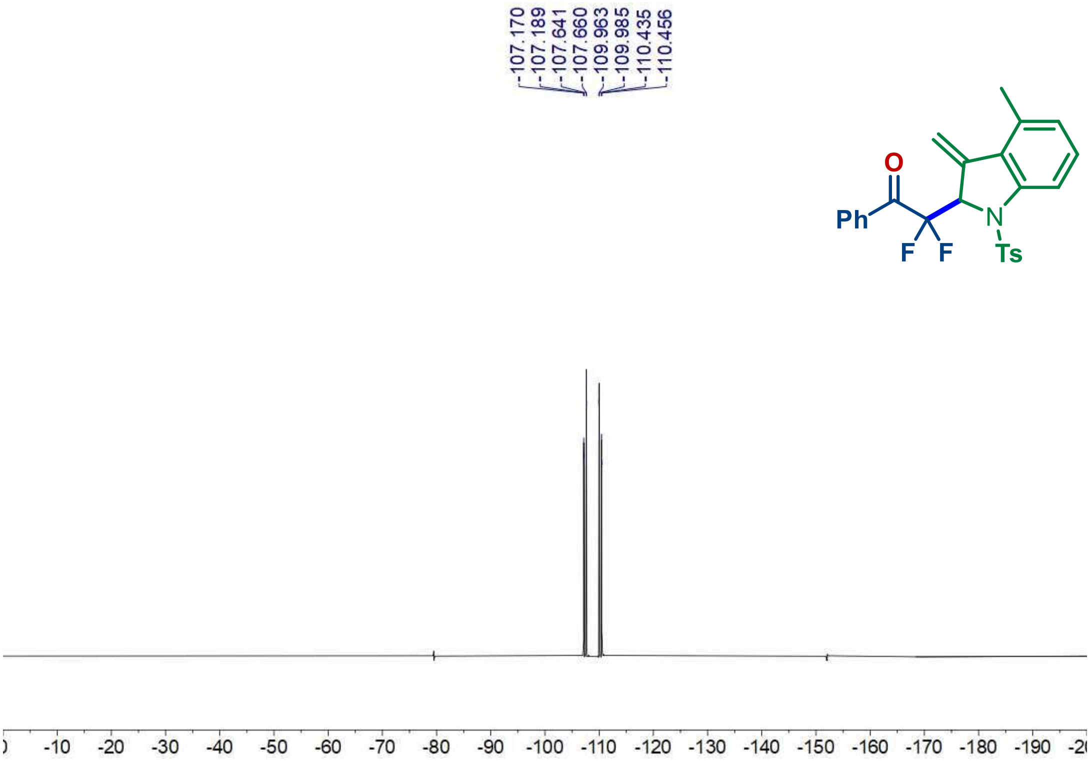

Supplementary Fig. 16 ^{19}F NMR (564 MHz, $CDCl_3$) spectrum of compound **4**

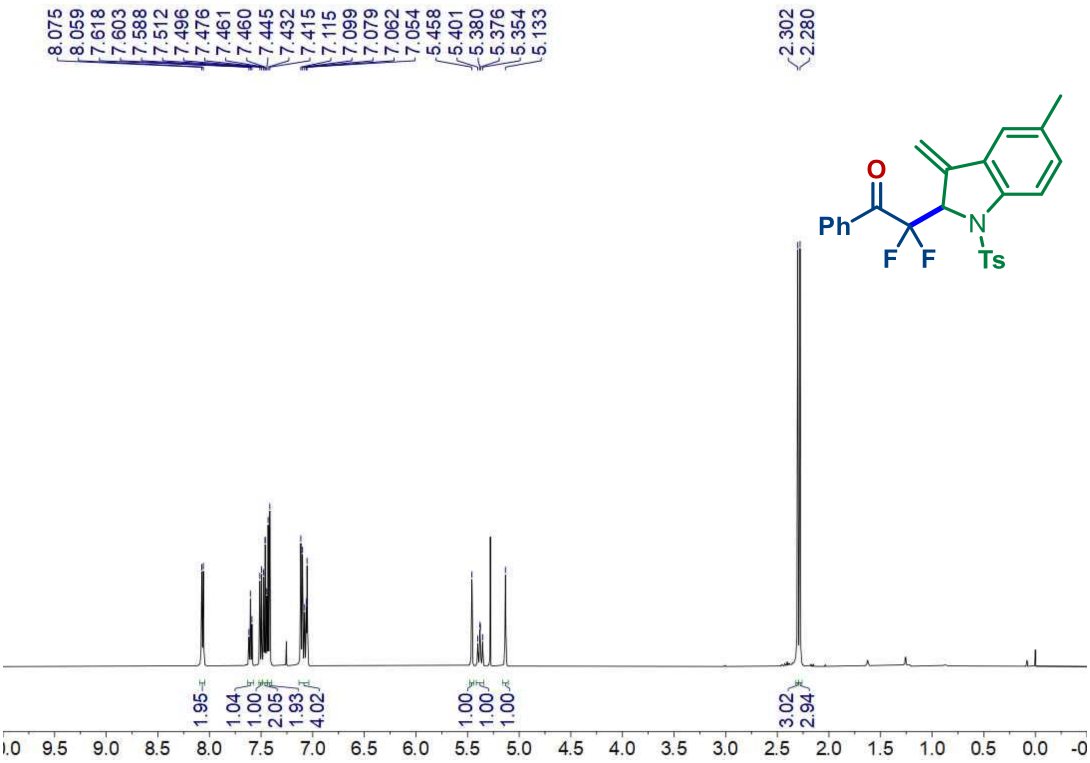

Supplementary Fig. 17 ^{1}H NMR (500 MHz, $CDCl_3$) spectrum of compound **5**

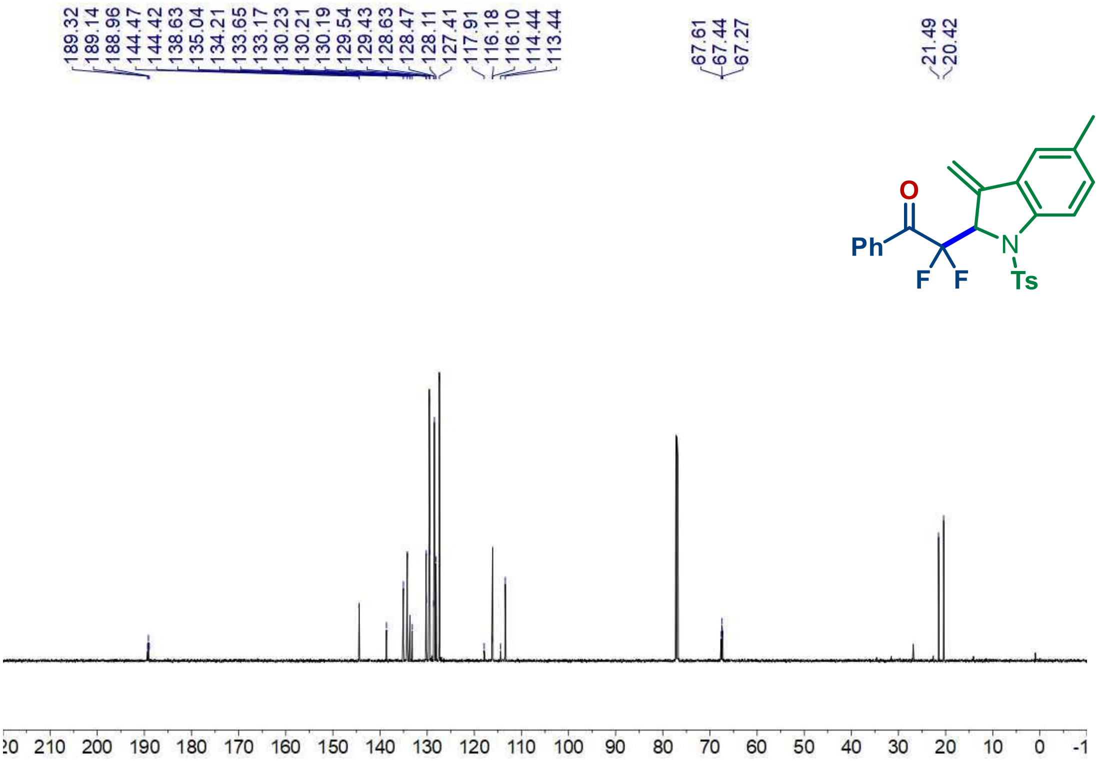

Supplementary Fig. 18 ^{13}C NMR (125 MHz, $CDCl_3$) spectrum of compound **5**

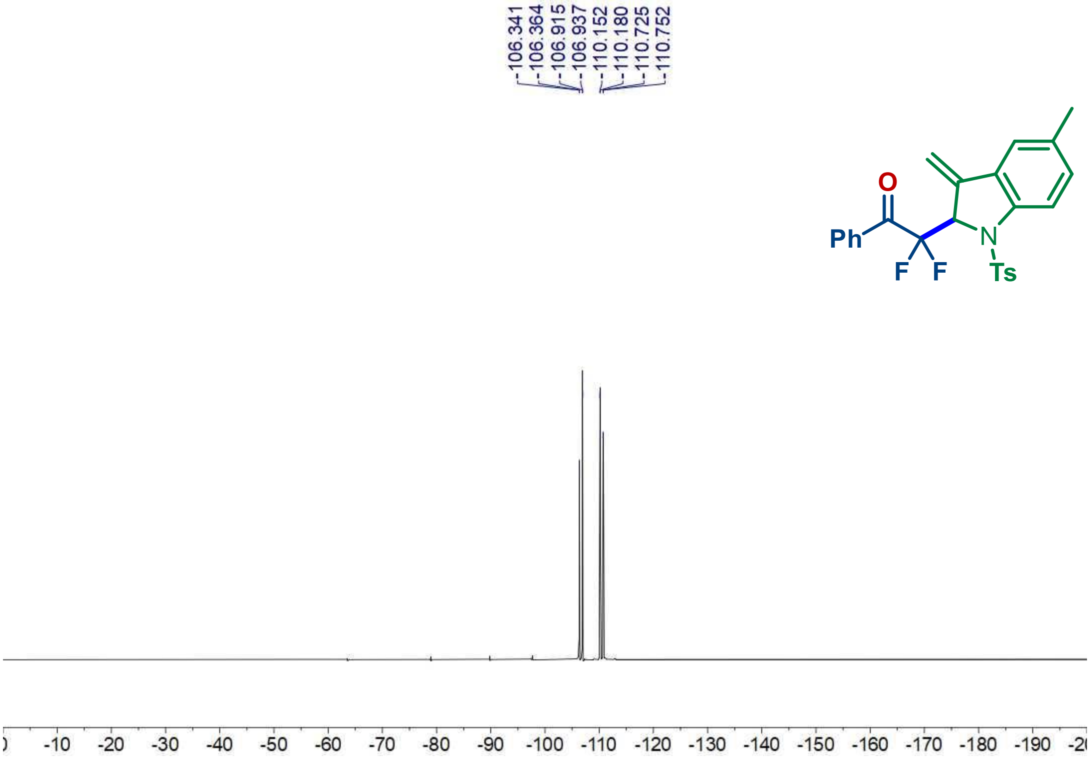

Supplementary Fig. 19 ^{19}F NMR (470 MHz, $CDCl_3$) spectrum of compound **5**

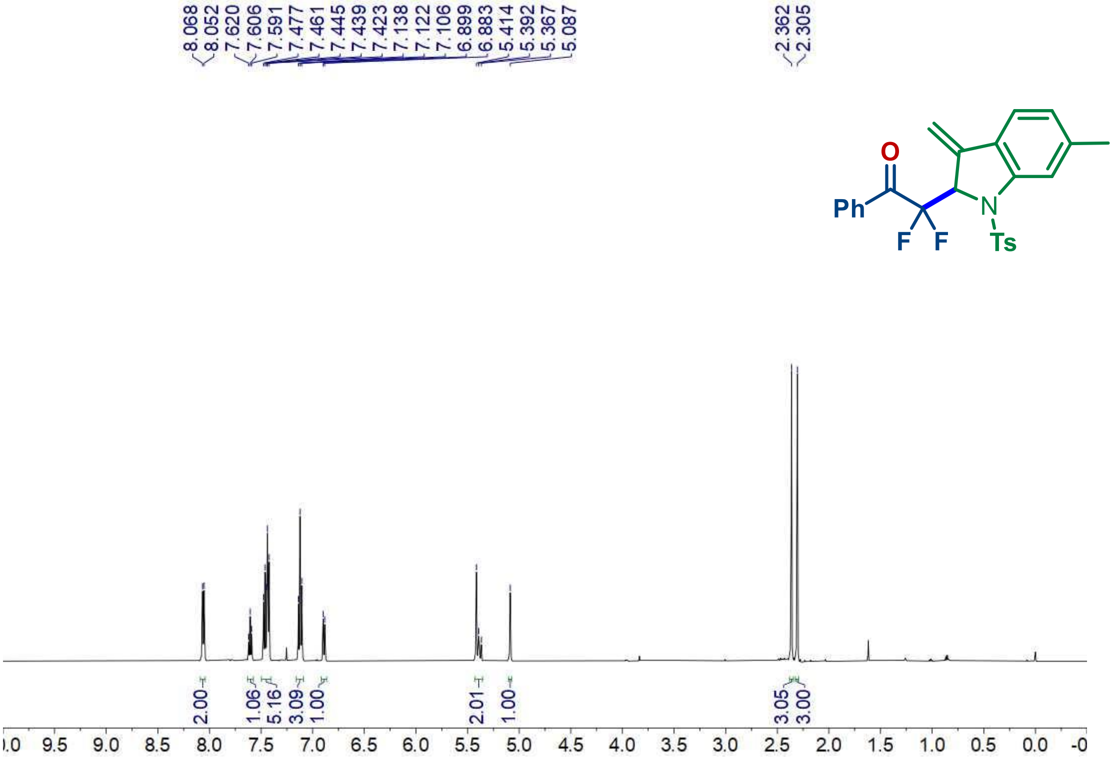

Supplementary Fig. 20 ^{1}H NMR (500 MHz, $CDCl_3$) spectrum of compound **6**

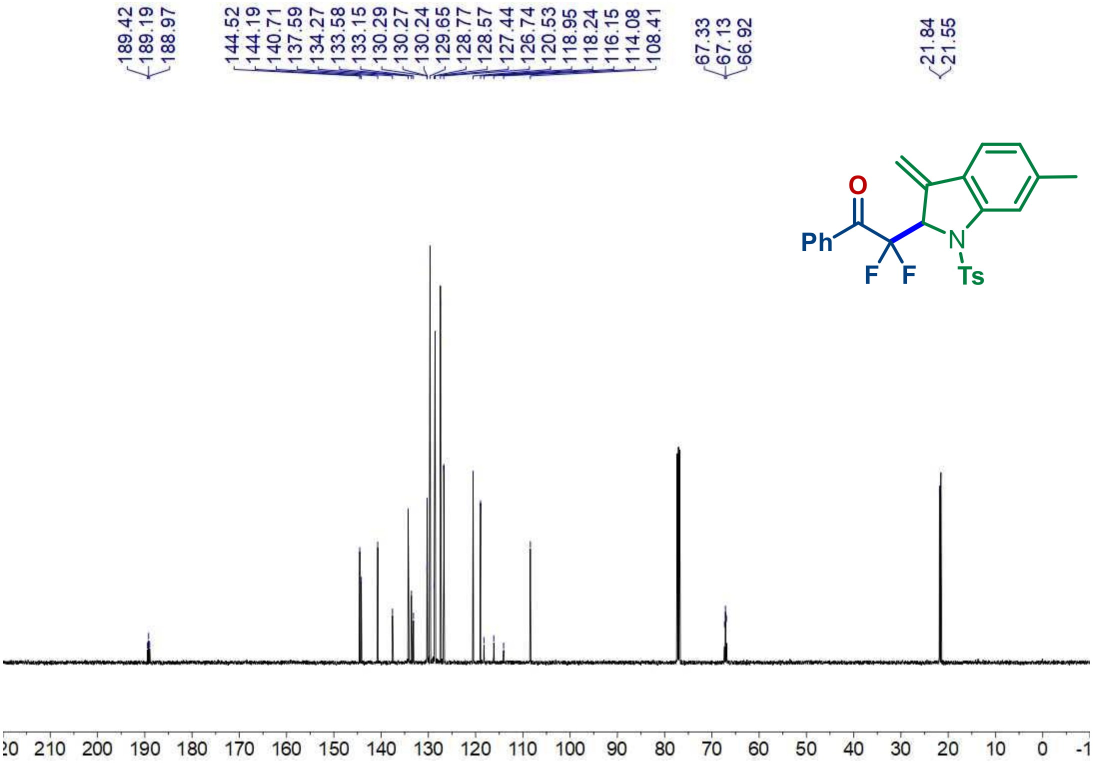

Supplementary Fig. 21 ^{13}C NMR (125 MHz, $CDCl_3$) spectrum of compound **6**

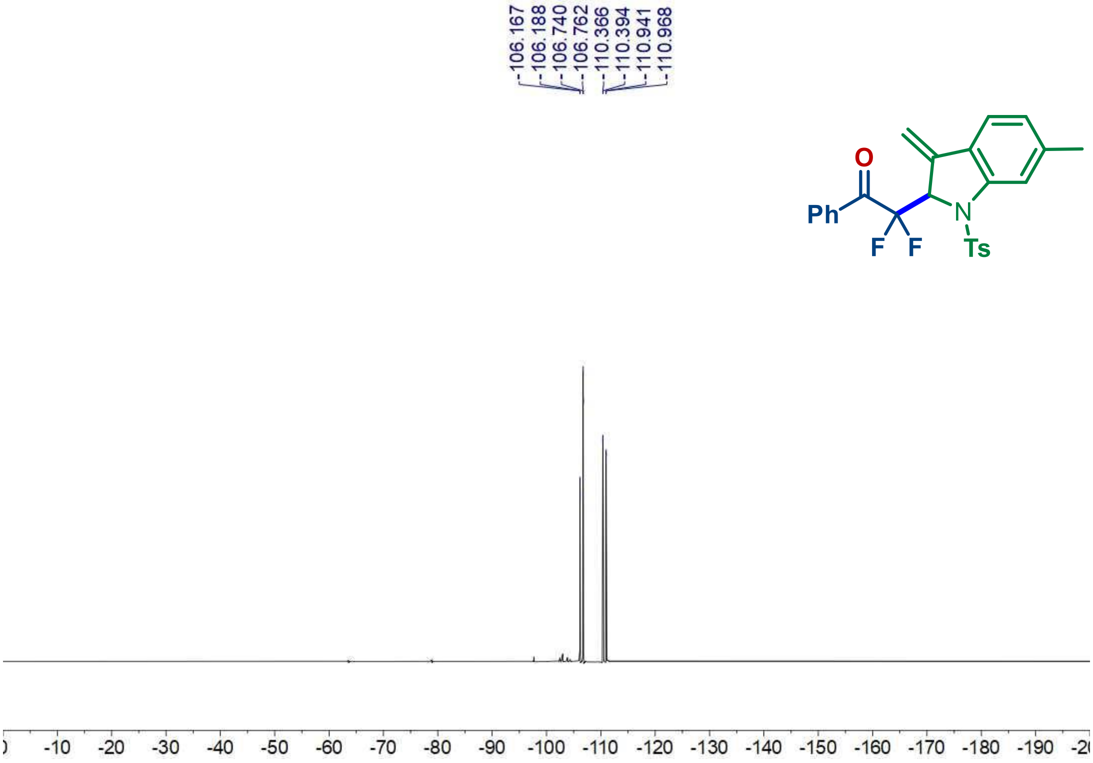

Supplementary Fig. 22 ^{19}F NMR (470 MHz, $CDCl_3$) spectrum of compound **6**

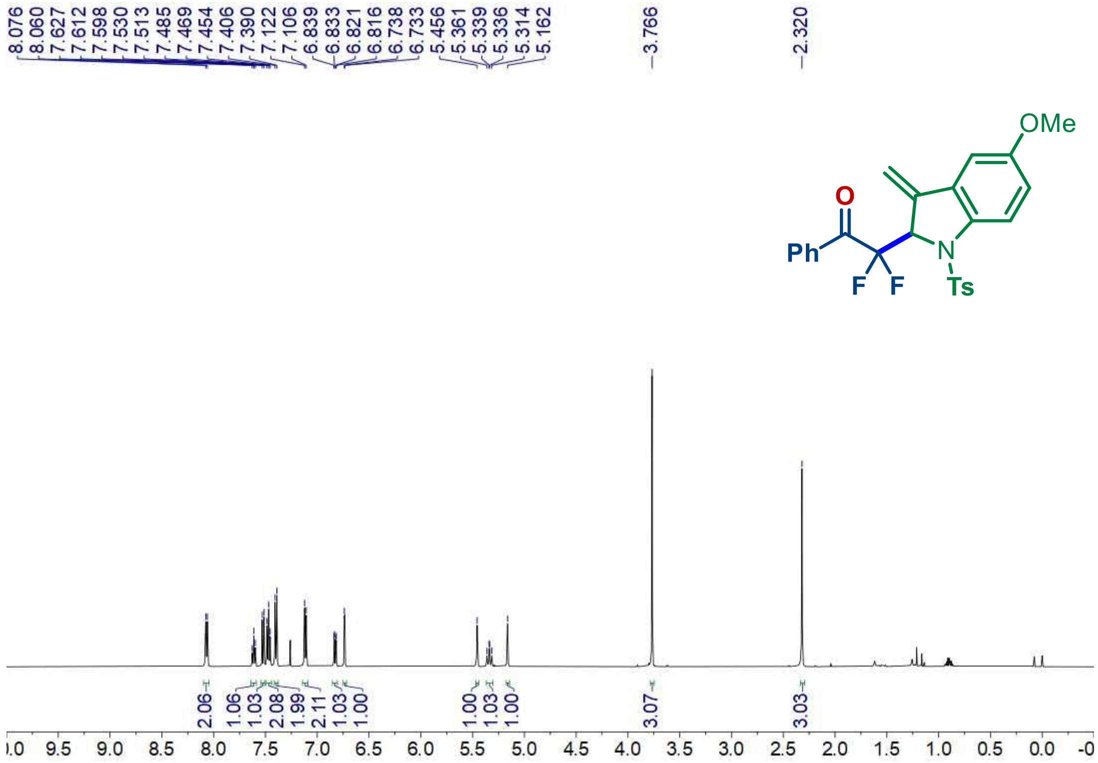

Supplementary Fig. 23 ^{1}H NMR (500 MHz, $CDCl_3$) spectrum of compound **7**

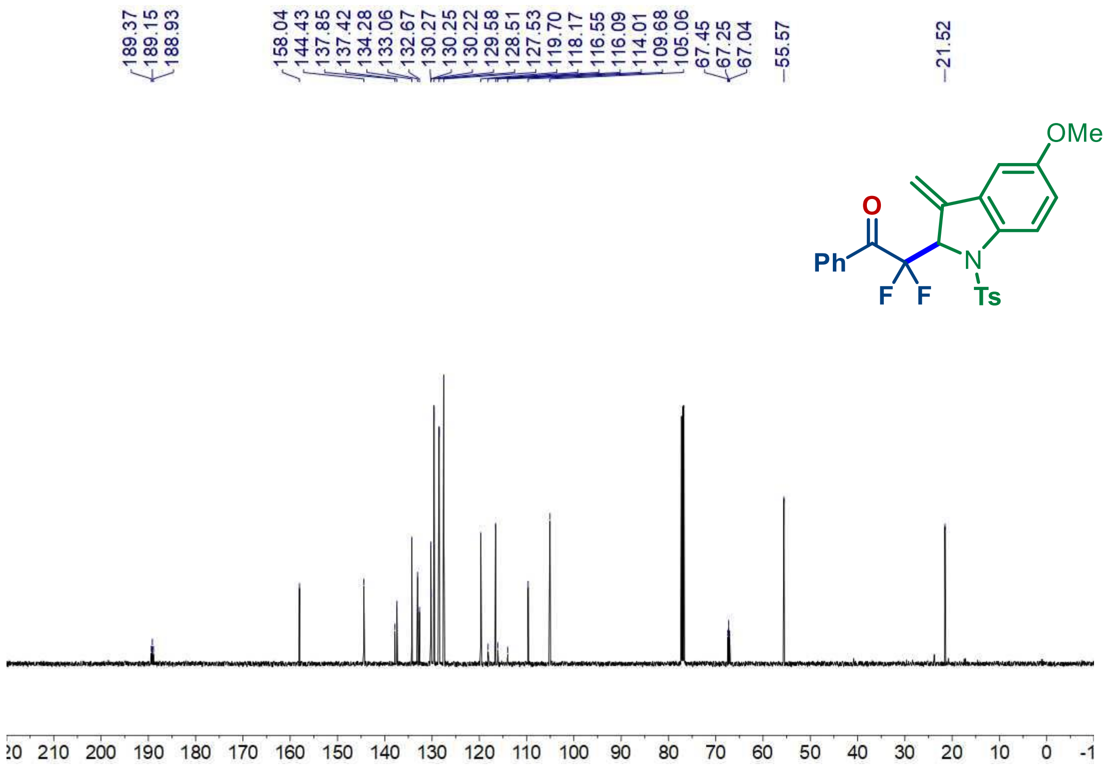

Supplementary Fig. 24 ^{13}C NMR (125 MHz, $CDCl_3$) spectrum of compound **7**

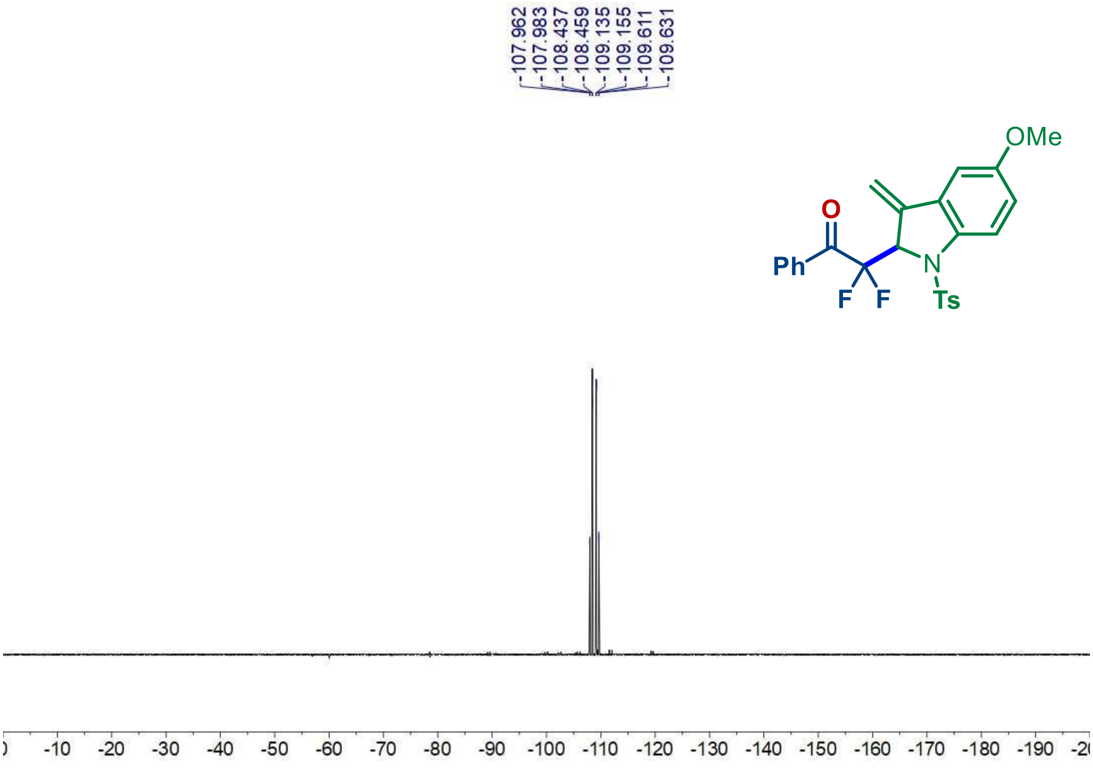

Supplementary Fig. 25 ^{19}F NMR (564 MHz, $CDCl_3$) spectrum of compound **7**

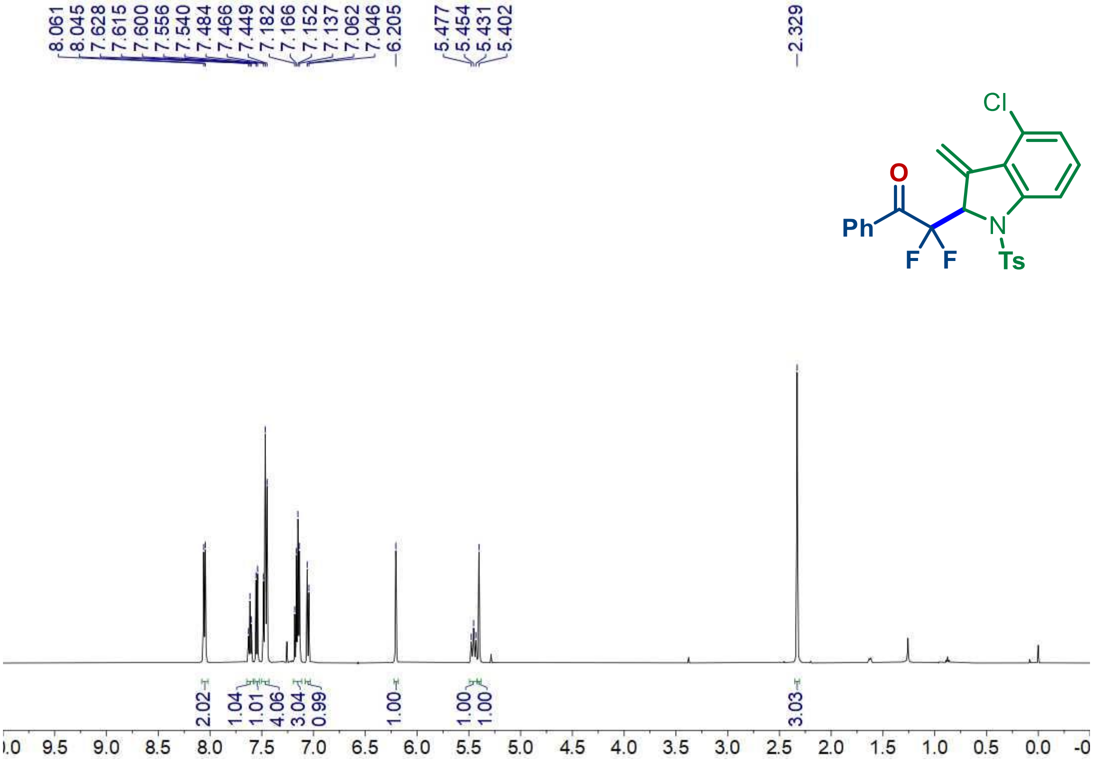

Supplementary Fig. 26 ^{1}H NMR (500 MHz, $CDCl_3$) spectrum of compound **8**

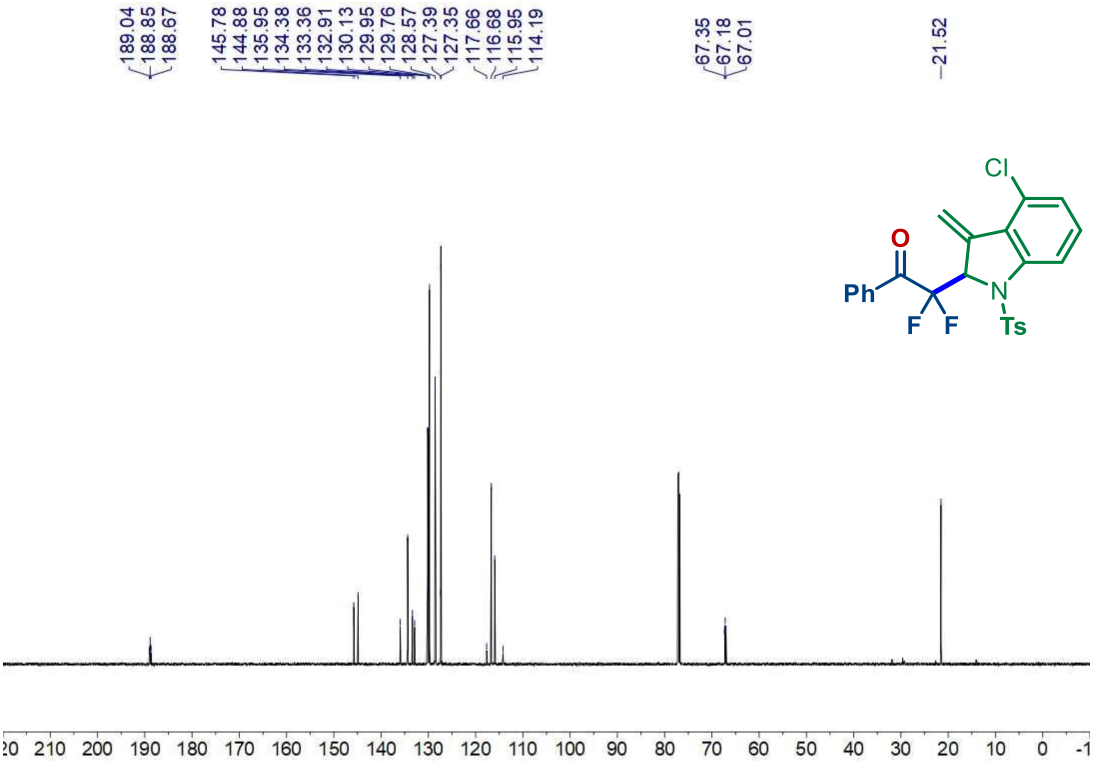

Supplementary Fig. 27 ^{13}C NMR (150 MHz, $CDCl_3$) spectrum of compound **8**

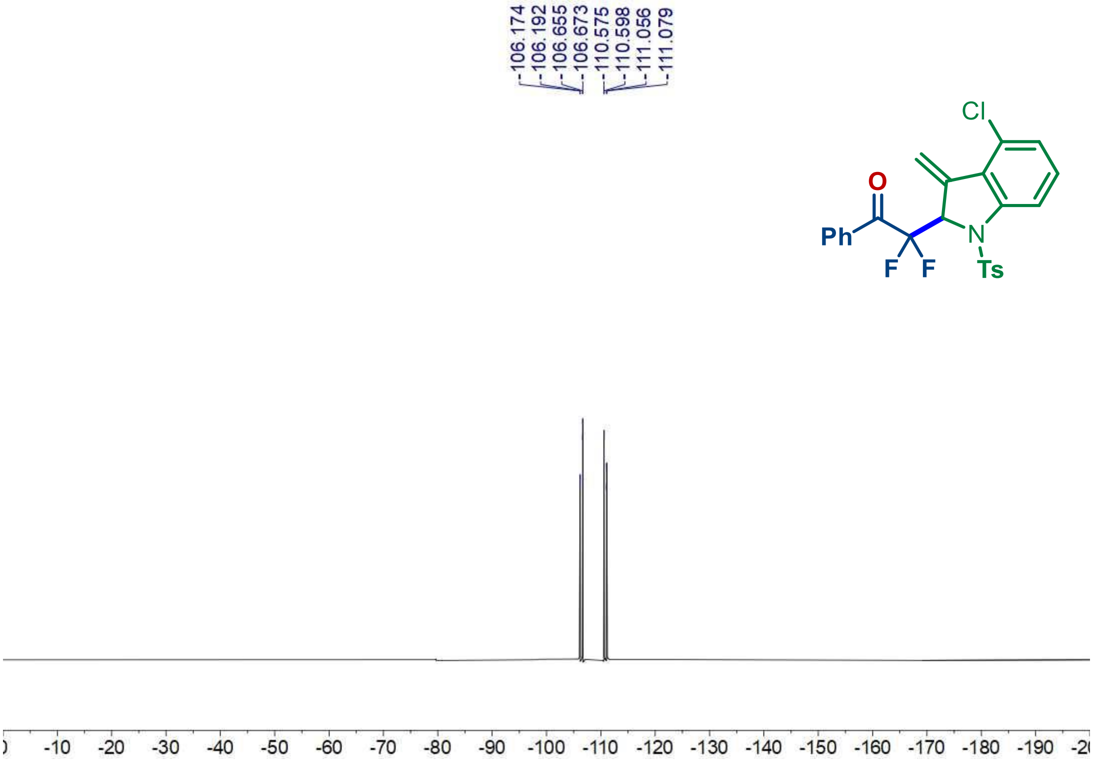

Supplementary Fig. 28 ^{19}F NMR (564 MHz, $CDCl_3$) spectrum of compound **8**

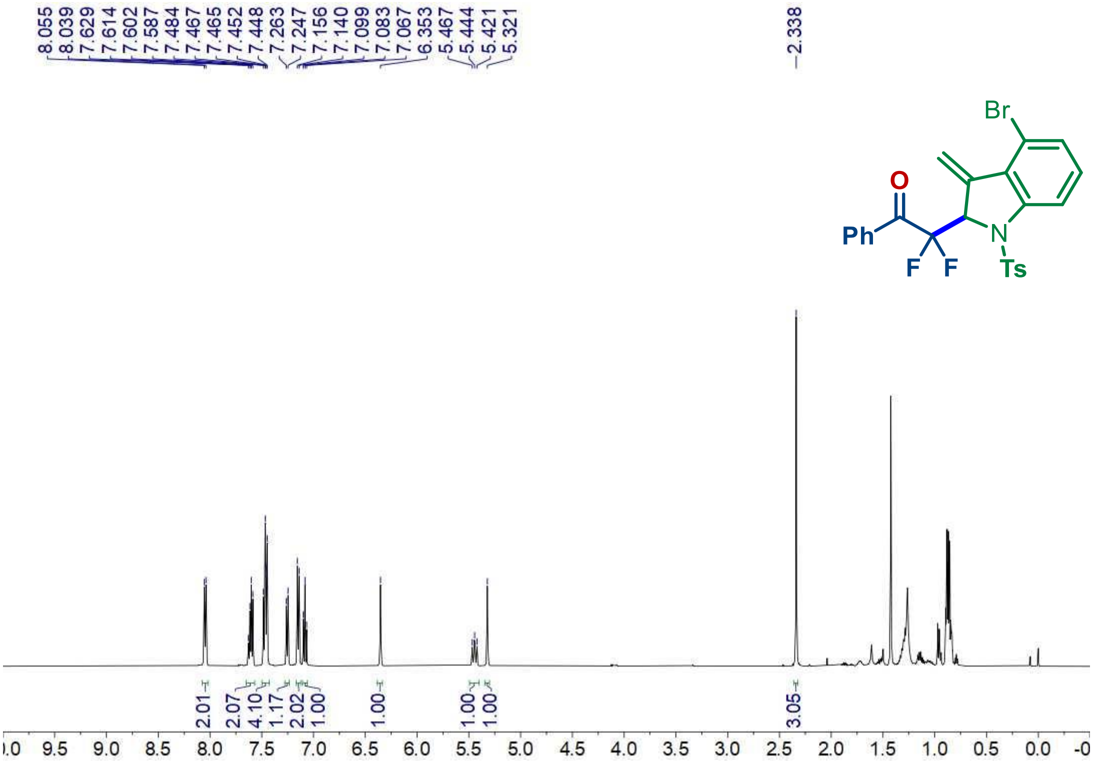

Supplementary Fig. 29 ^{1}H NMR (500 MHz, $CDCl_3$) spectrum of compound **9**

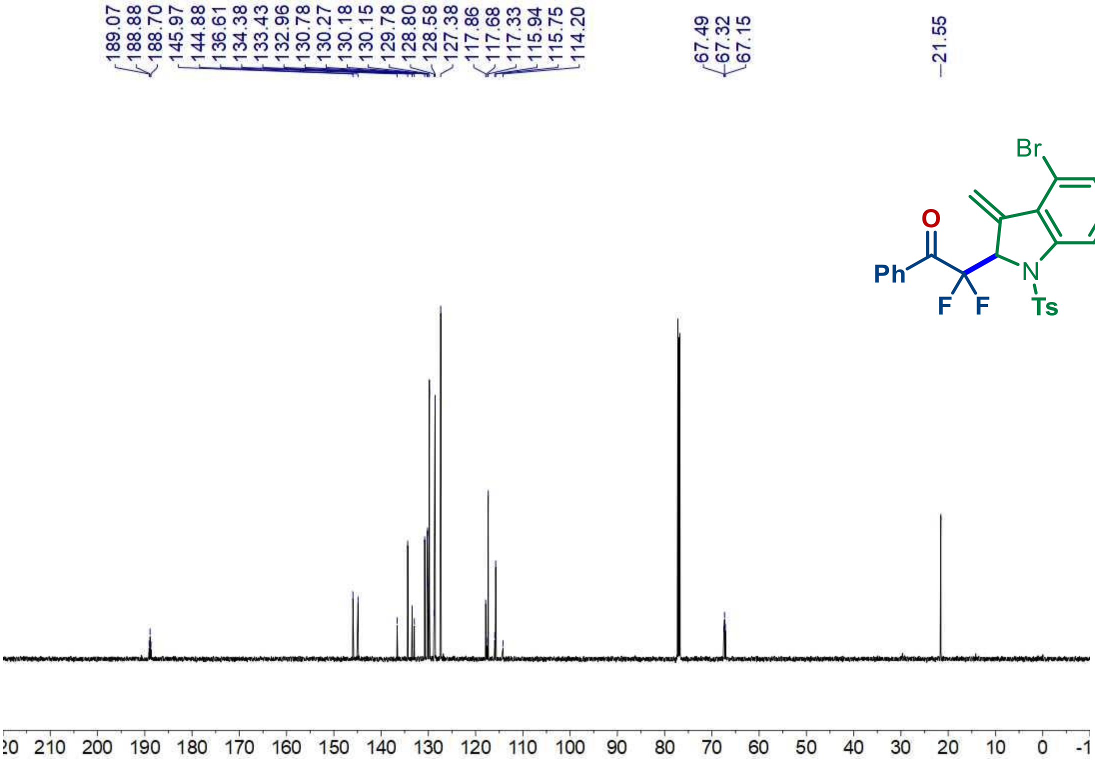

Supplementary Fig. 30 ^{13}C NMR (150 MHz, $CDCl_3$) spectrum of compound **9**

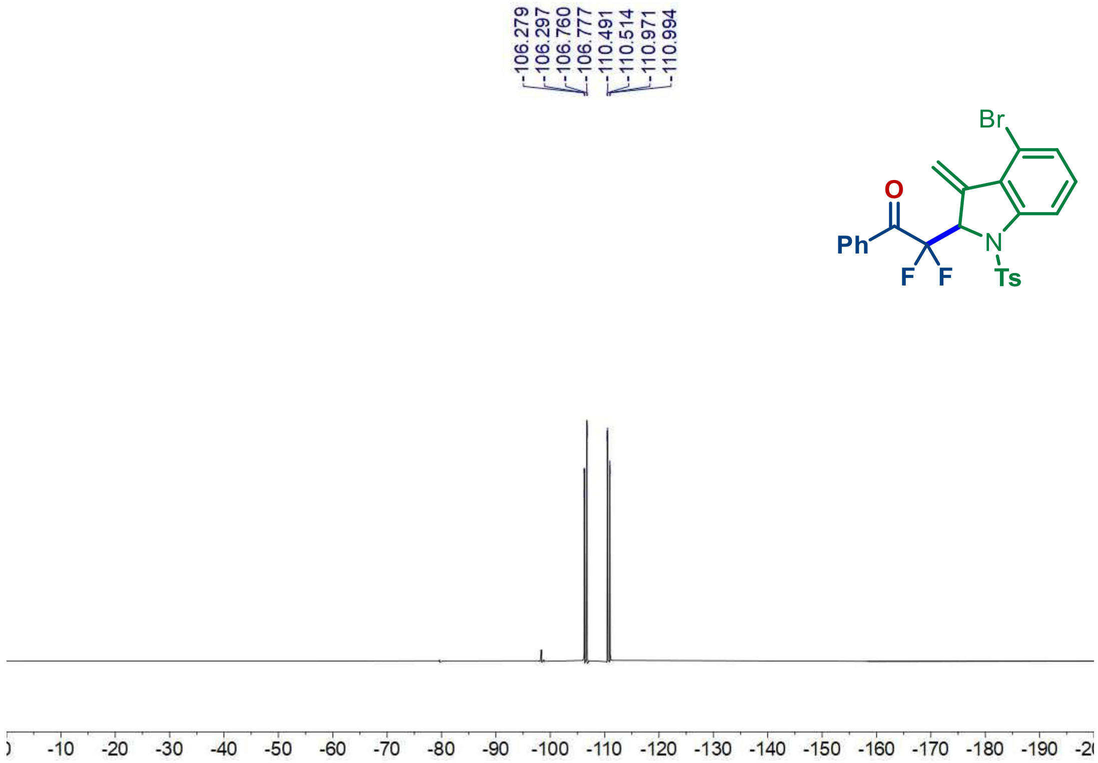

Supplementary Fig. 31 ^{19}F NMR (564 MHz, $CDCl_3$) spectrum of compound **9**

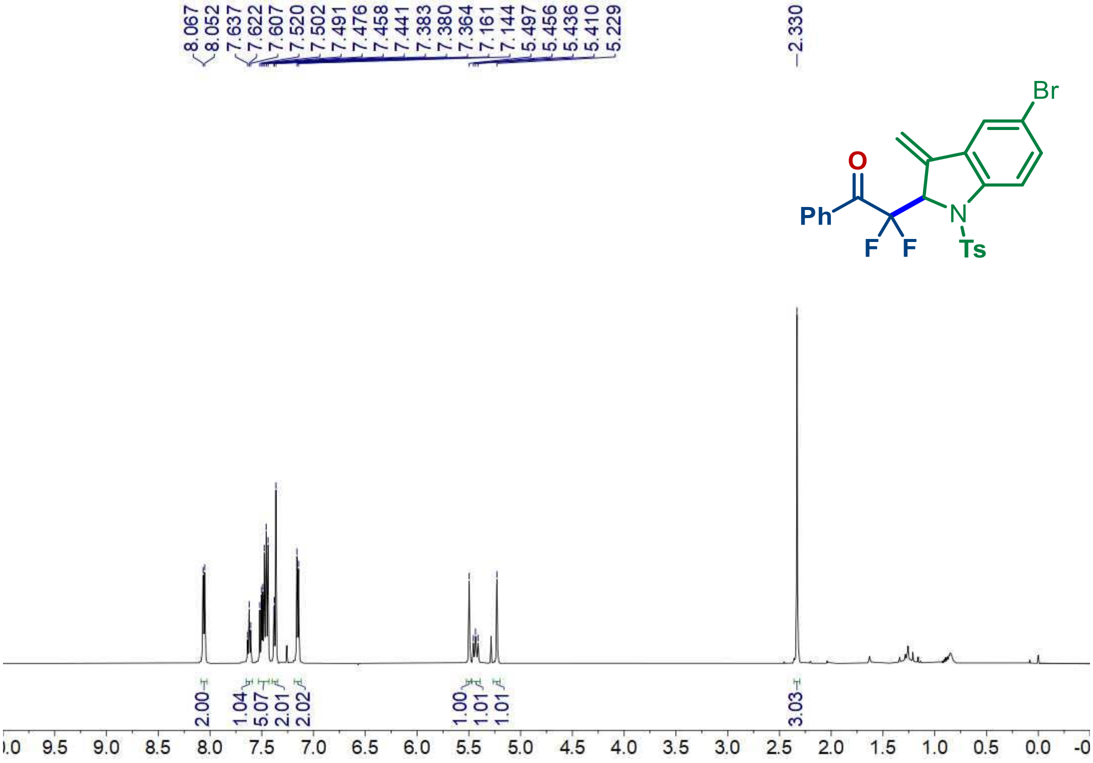

Supplementary Fig. 32 ^{1}H NMR (500 MHz, $CDCl_3$) spectrum of compound **10**

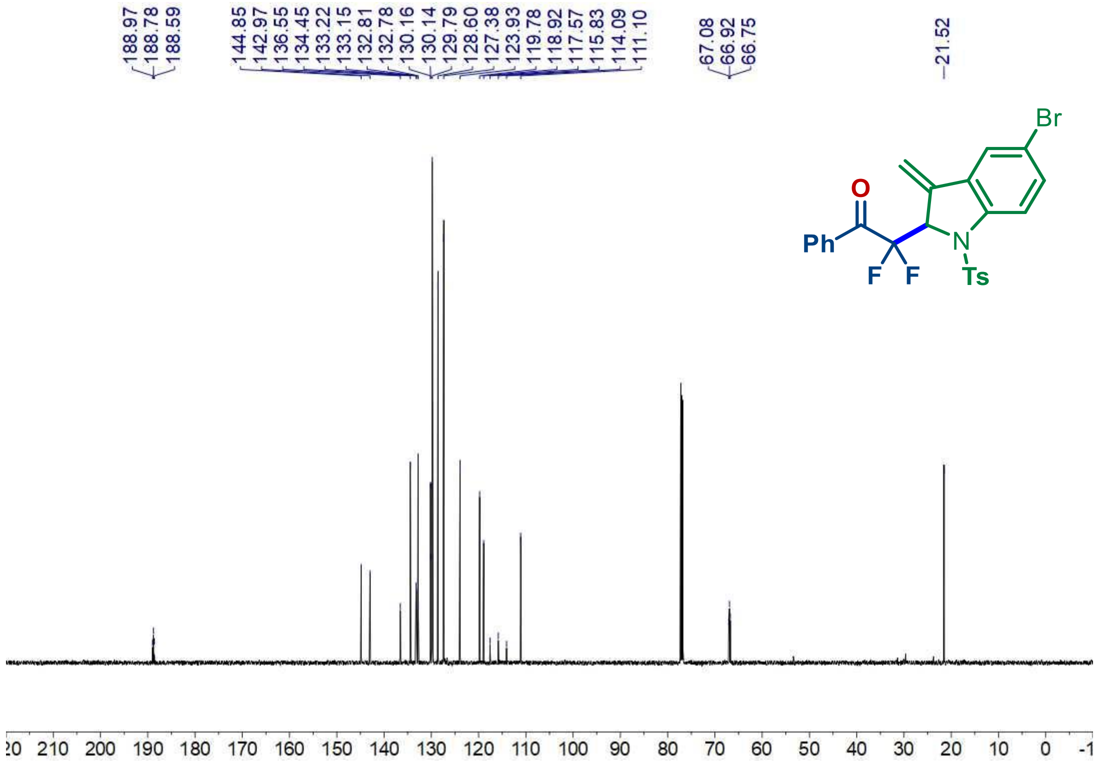

Supplementary Fig. 33 ^{13}C NMR (150 MHz, $CDCl_3$) spectrum of compound **10**

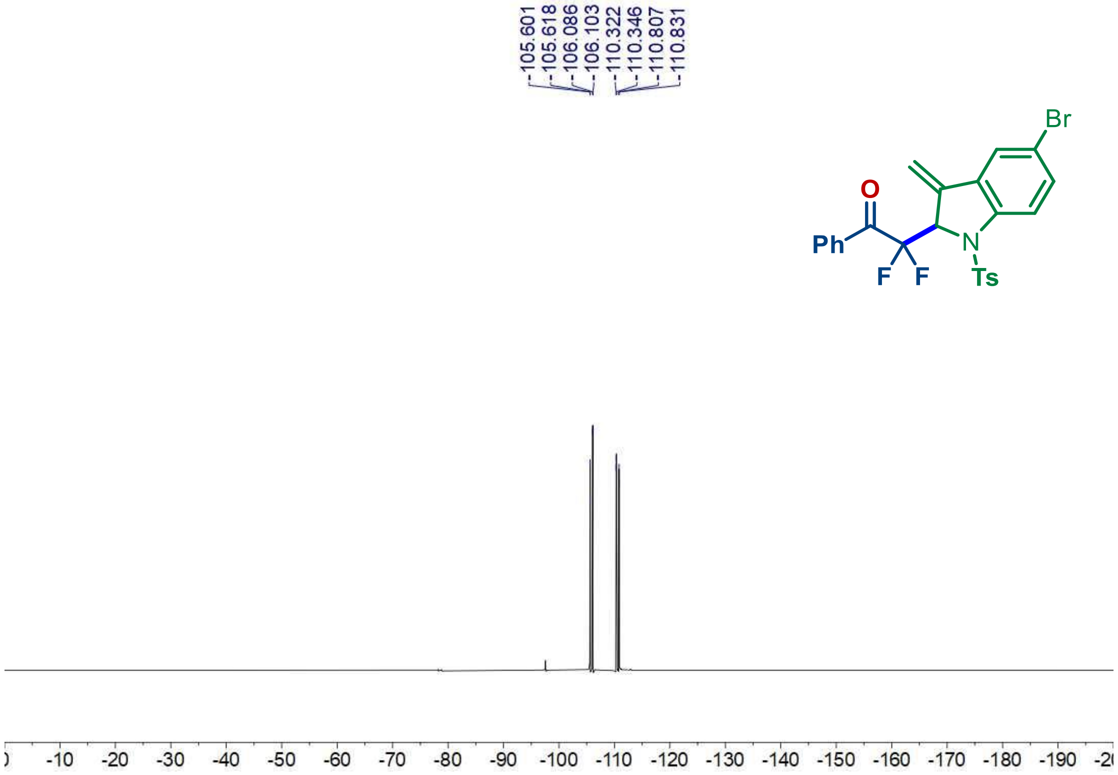

Supplementary Fig. 34 ^{19}F NMR (564 MHz, $CDCl_3$) spectrum of compound **10**

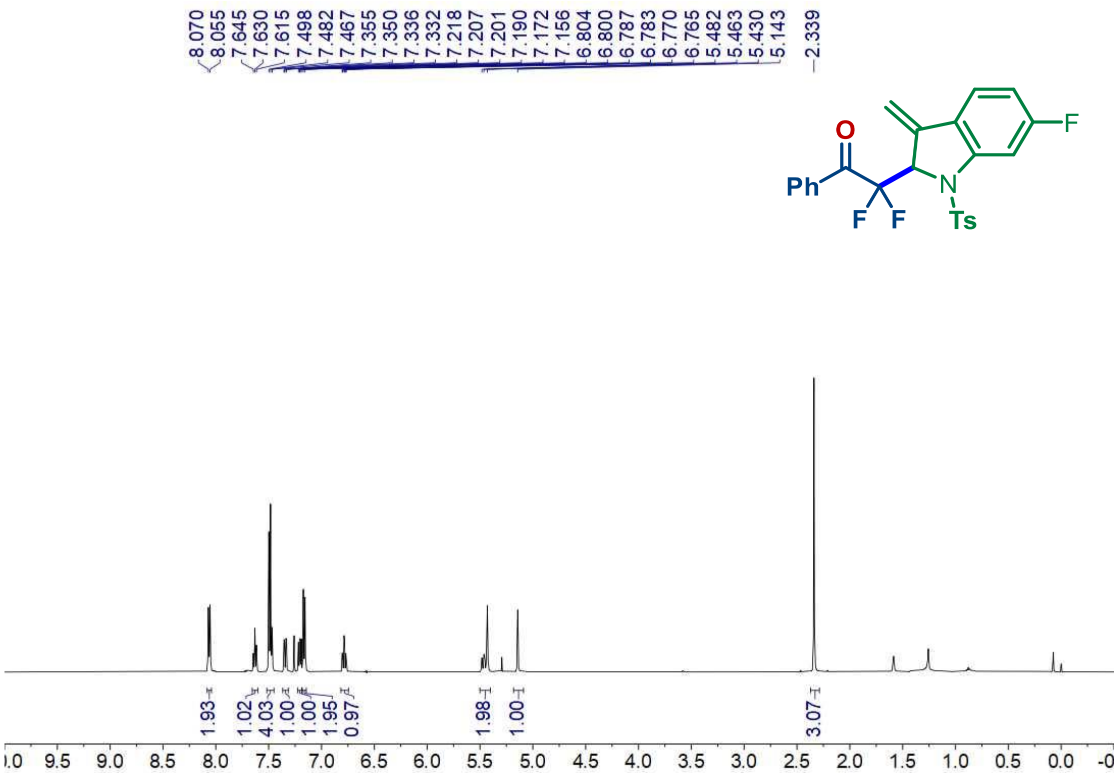

Supplementary Fig. 35 ^{1}H NMR (500 MHz, $CDCl_3$) spectrum of compound **11**

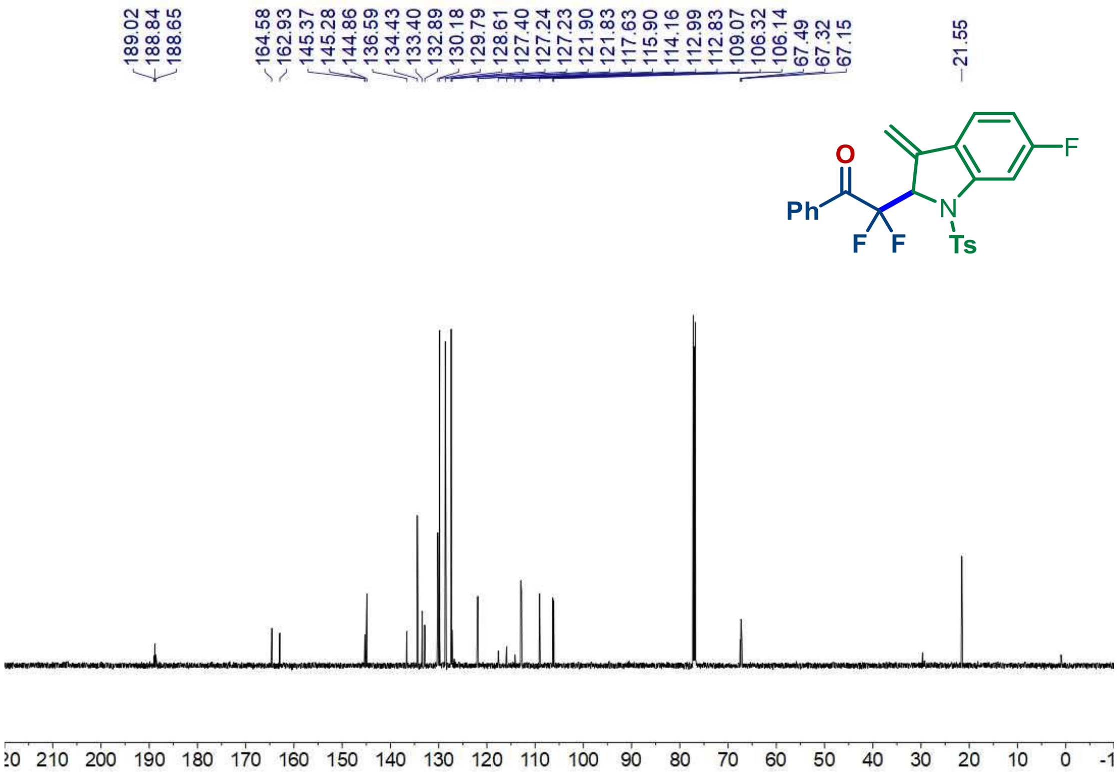

Supplementary Fig. 36 ^{13}C NMR (150 MHz, $CDCl_3$) spectrum of compound **11**

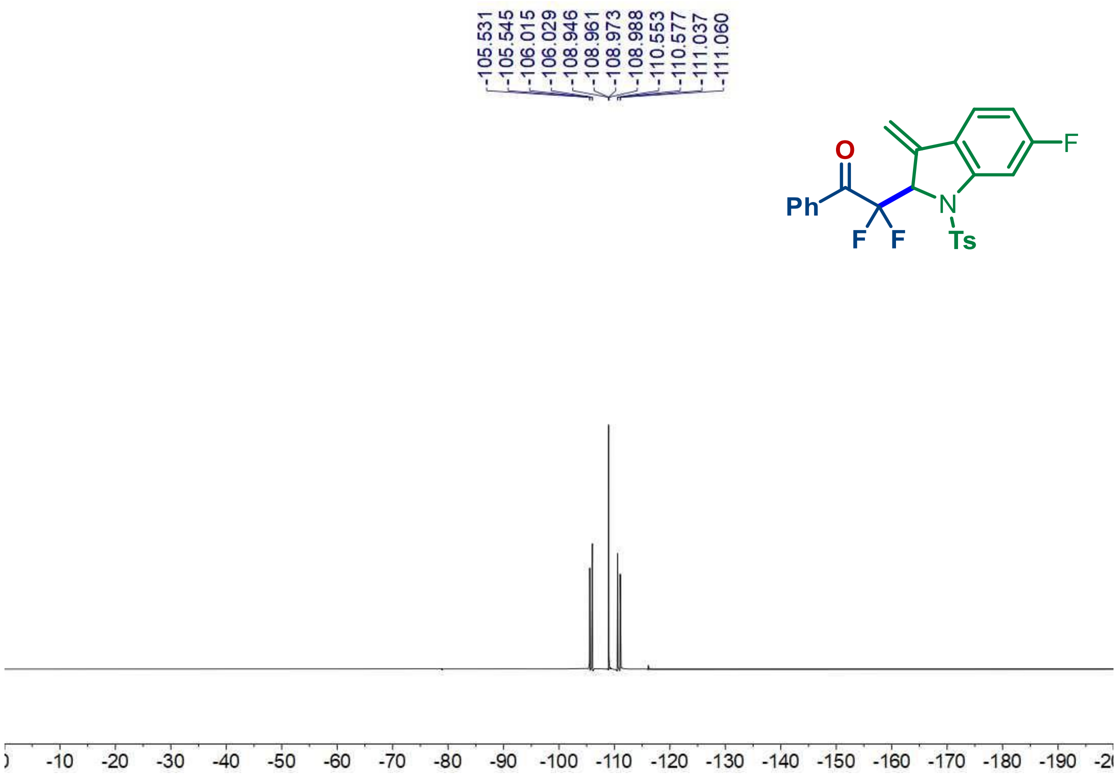

Supplementary Fig. 37 ^{19}F NMR (564 MHz, $CDCl_3$) spectrum of compound **11**

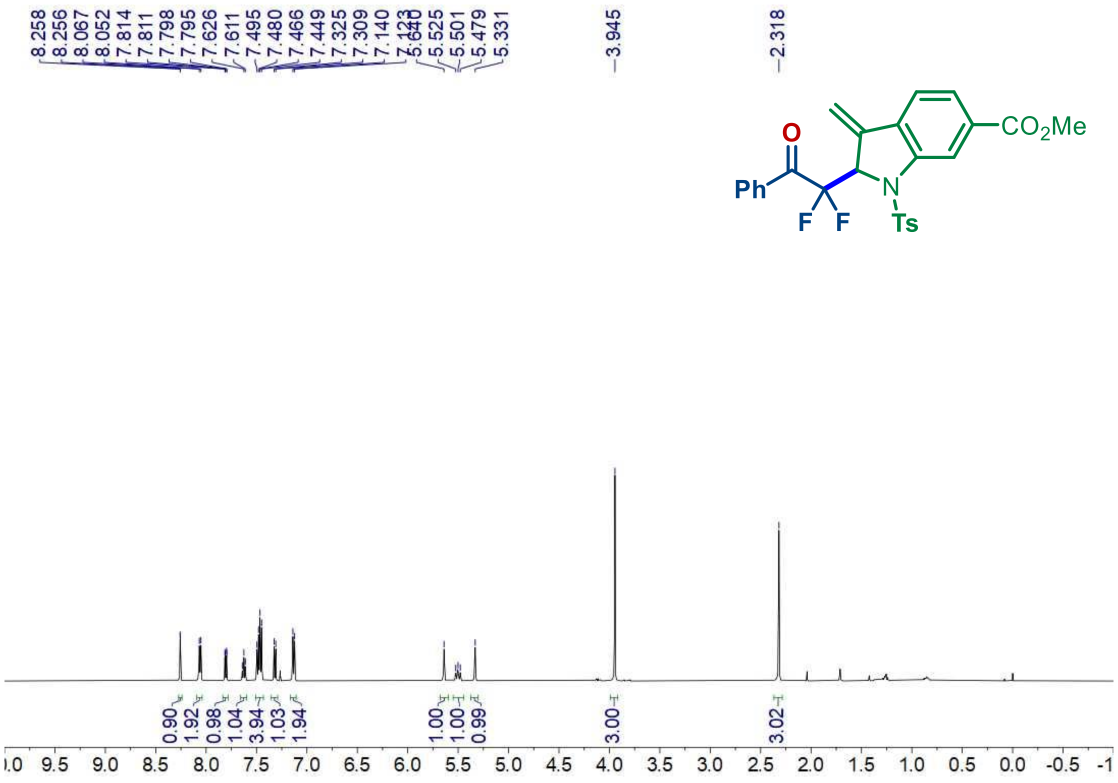

Supplementary Fig. 38 ^{1}H NMR (500 MHz, $CDCl_3$) spectrum of compound **12**

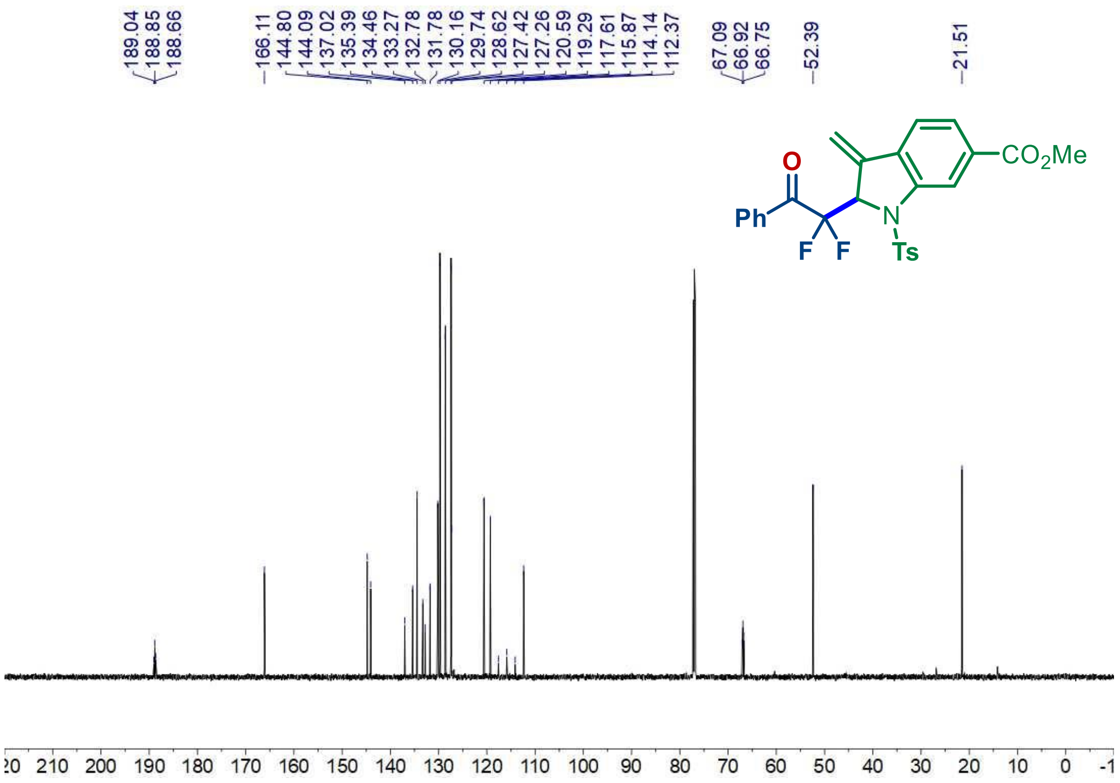

Supplementary Fig. 39 ^{13}C NMR (150 MHz, $CDCl_3$) spectrum of compound **12**

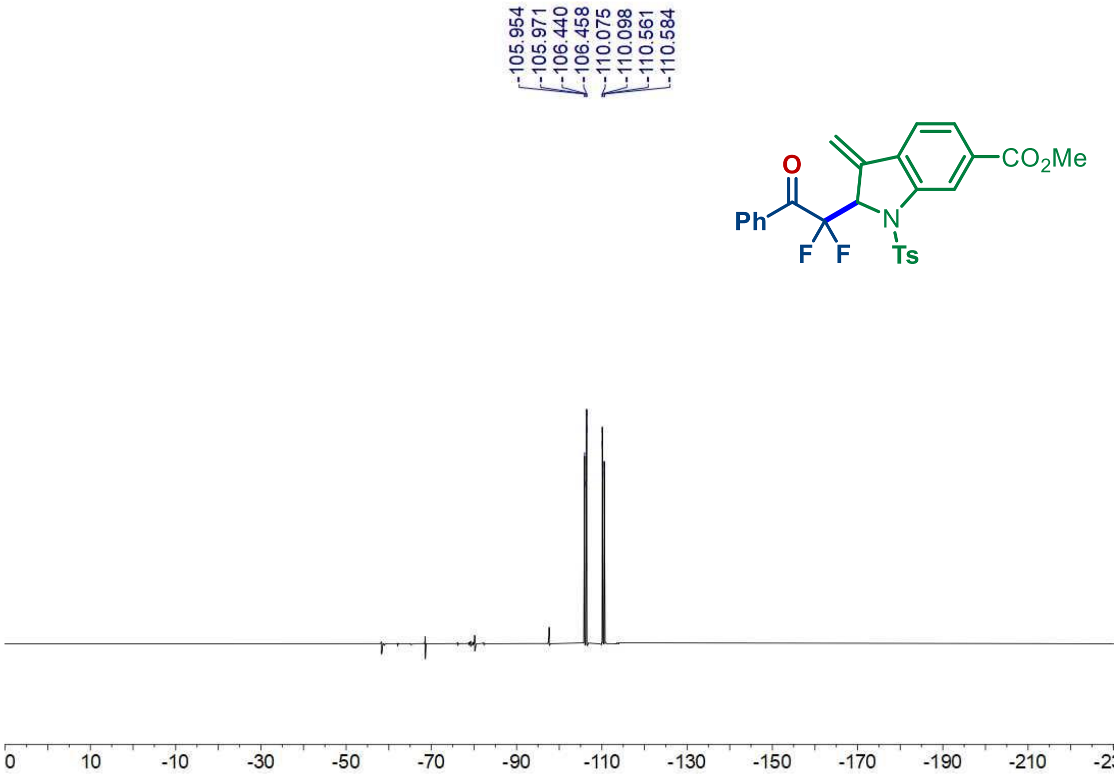

Supplementary Fig. 40 ^{19}F NMR (564 MHz, $CDCl_3$) spectrum of compound **12**

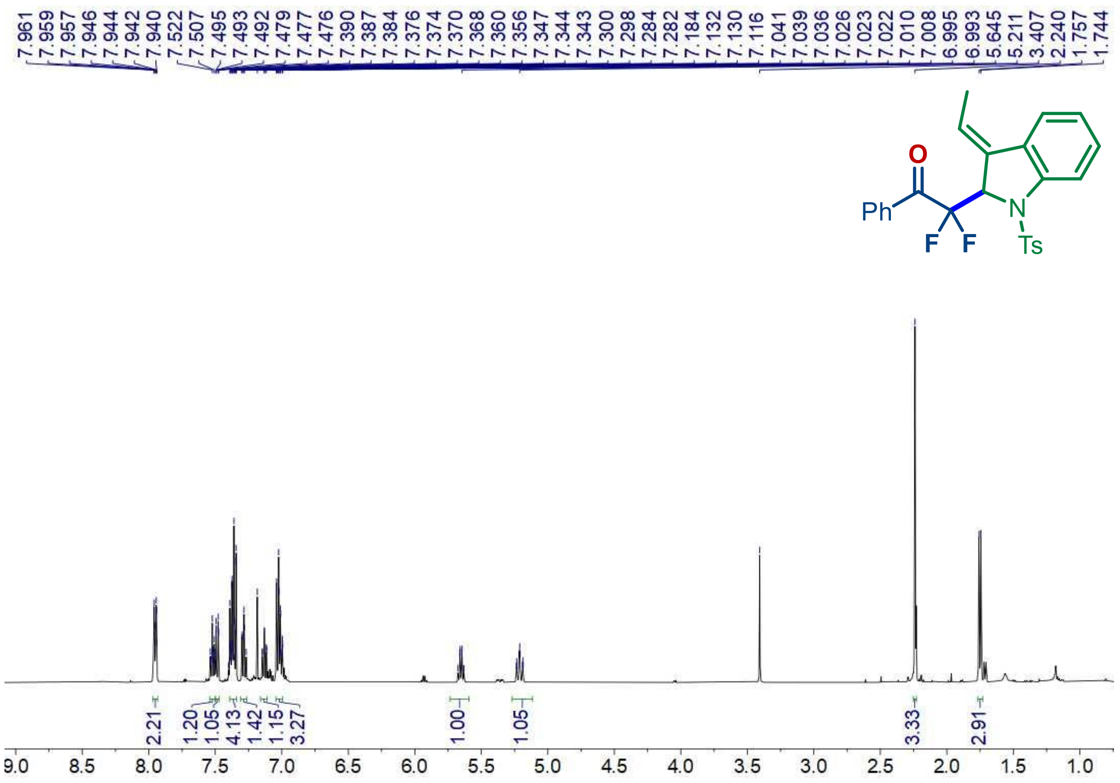

Supplementary Fig. 41 ^{1}H NMR (500 MHz, $CDCl_3$) spectrum of compound **13**

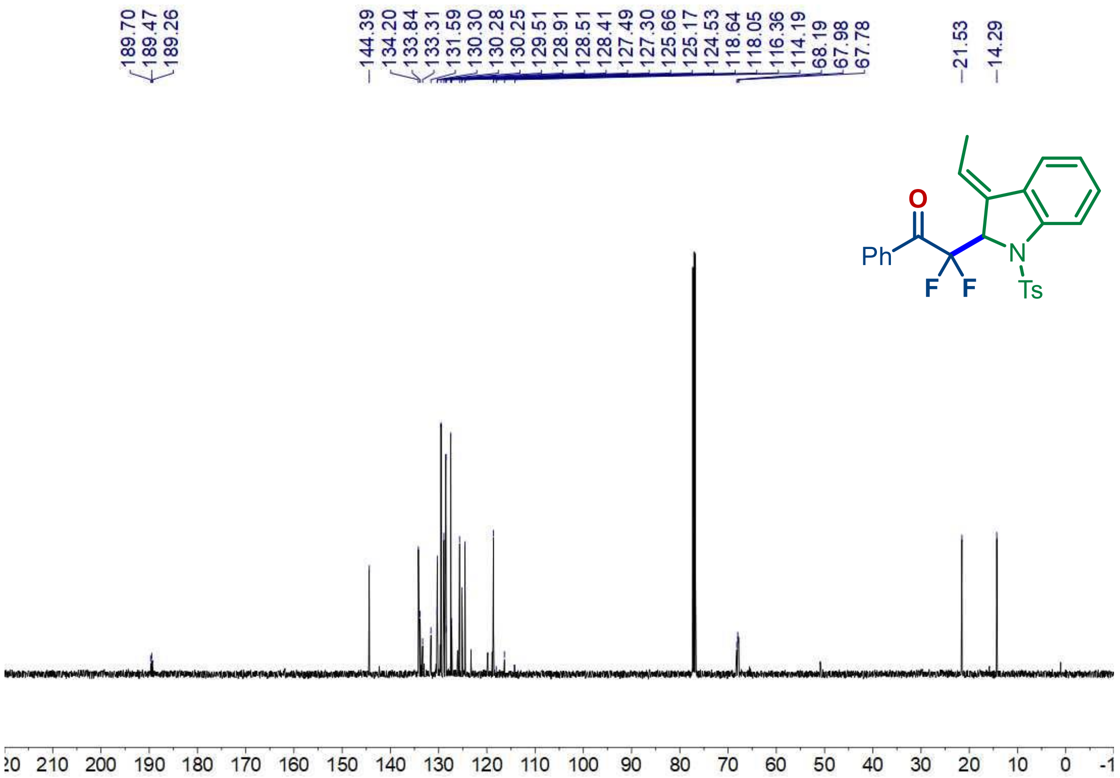

Supplementary Fig. 42 ^{13}C NMR (125 MHz, $CDCl_3$) spectrum of compound **13**

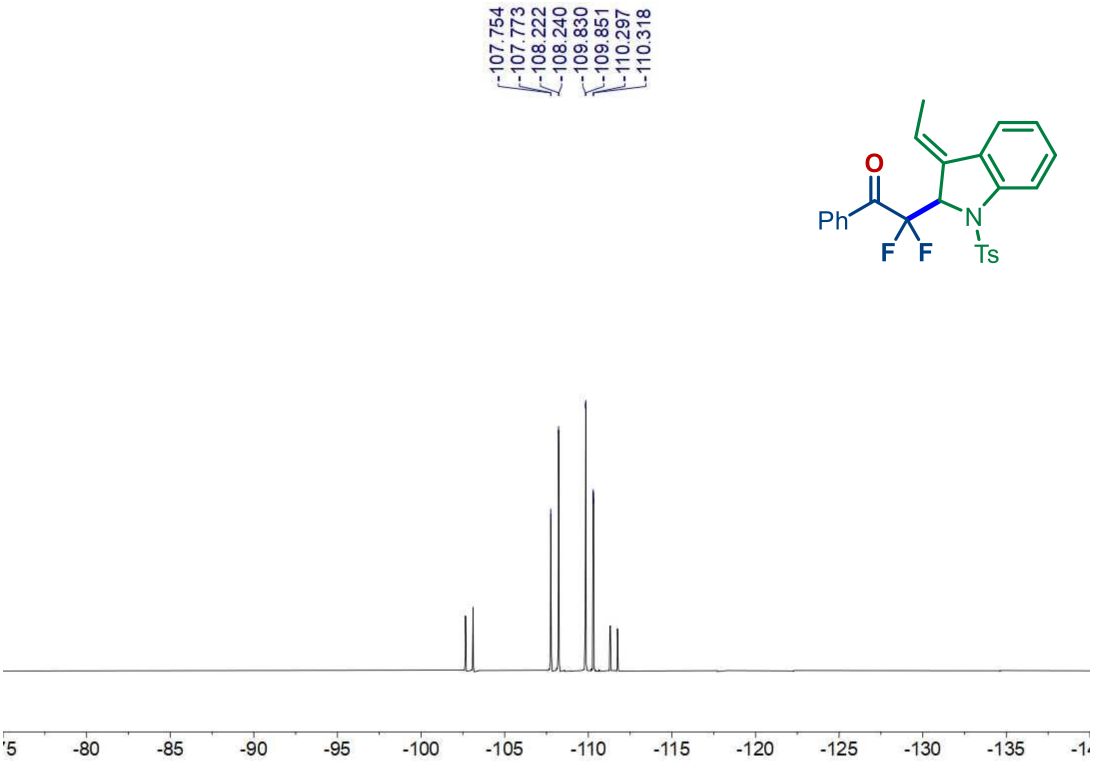

Supplementary Fig. 43 ^{19}F NMR (564 MHz, $CDCl_3$) spectrum of compound **13**

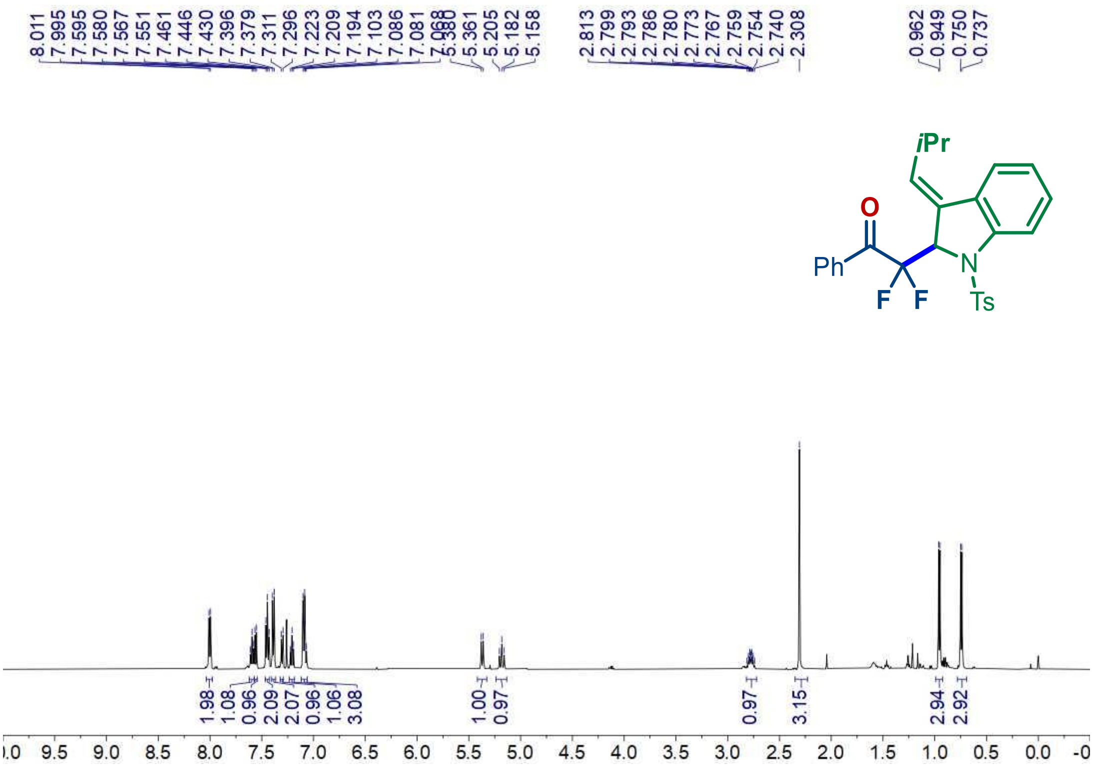

Supplementary Fig. 44 ^{1}H NMR (500 MHz, $CDCl_3$) spectrum of compound **14**

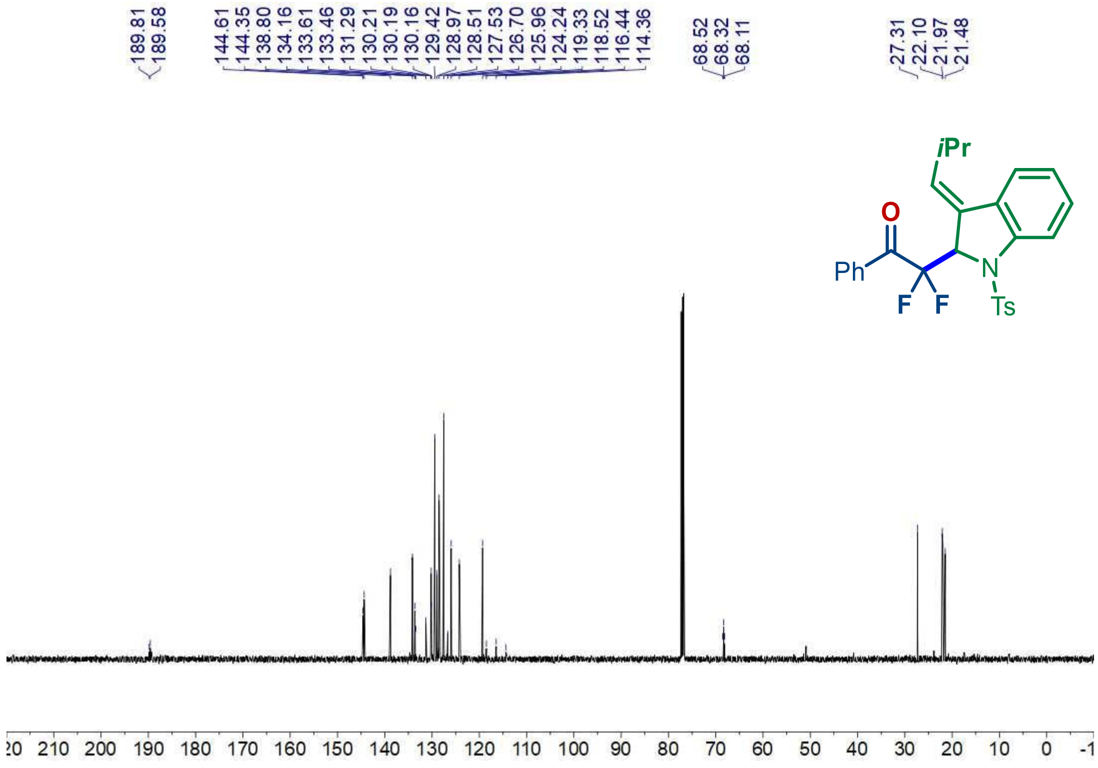

Supplementary Fig. 45 ^{13}C NMR (125 MHz, $CDCl_3$) spectrum of compound **14**

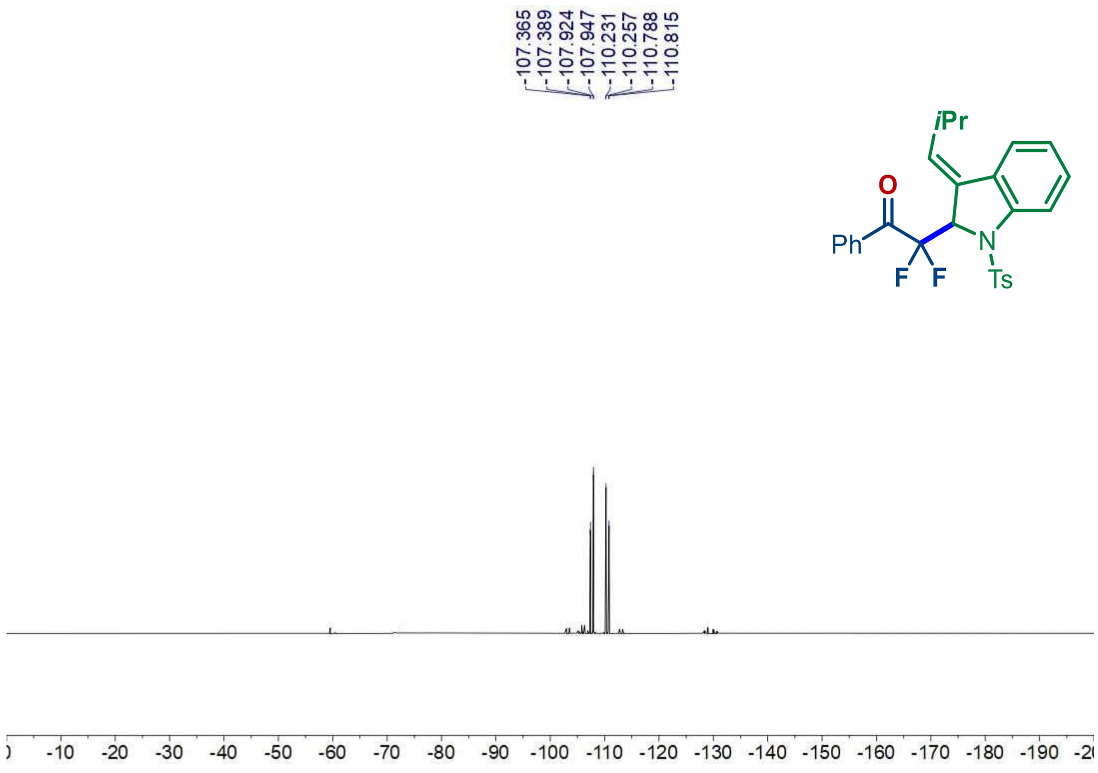

Supplementary Fig. 46 ^{19}F NMR (470 MHz, $CDCl_3$) spectrum of compound **14**

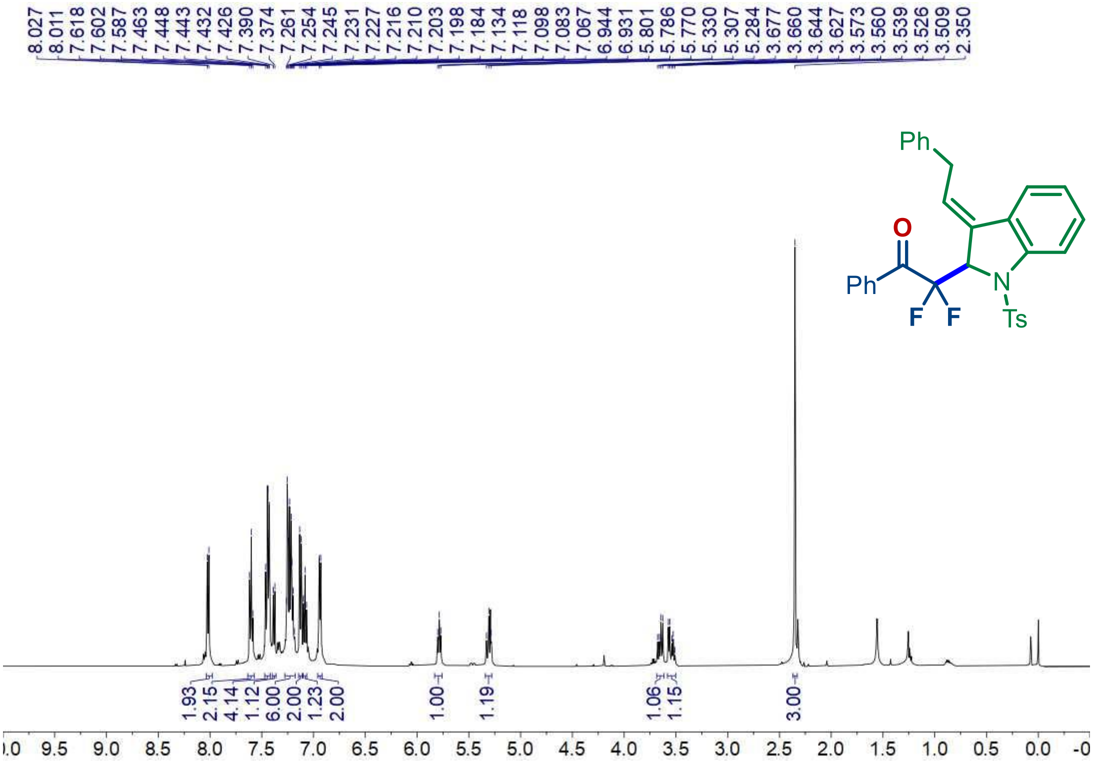

Supplementary Fig. 47 ^{1}H NMR (600 MHz, $CDCl_3$) spectrum of compound **15**

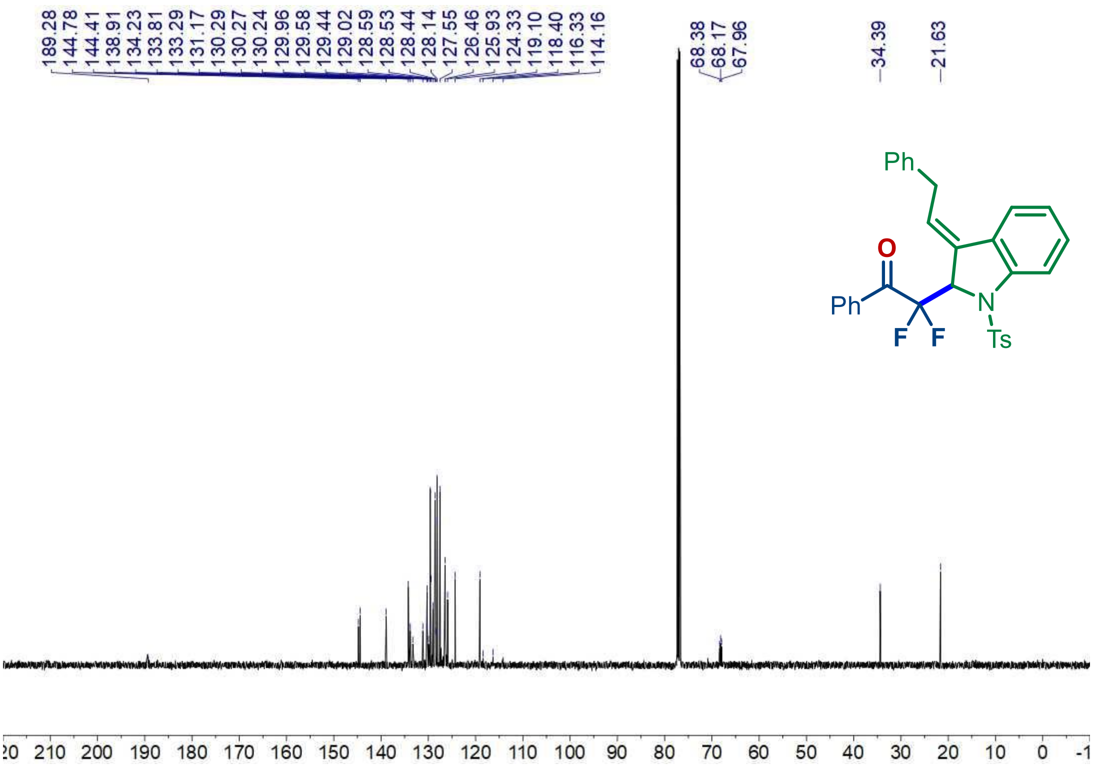

Supplementary Fig. 48 ^{13}C NMR (125 MHz, $CDCl_3$) spectrum of compound **15**

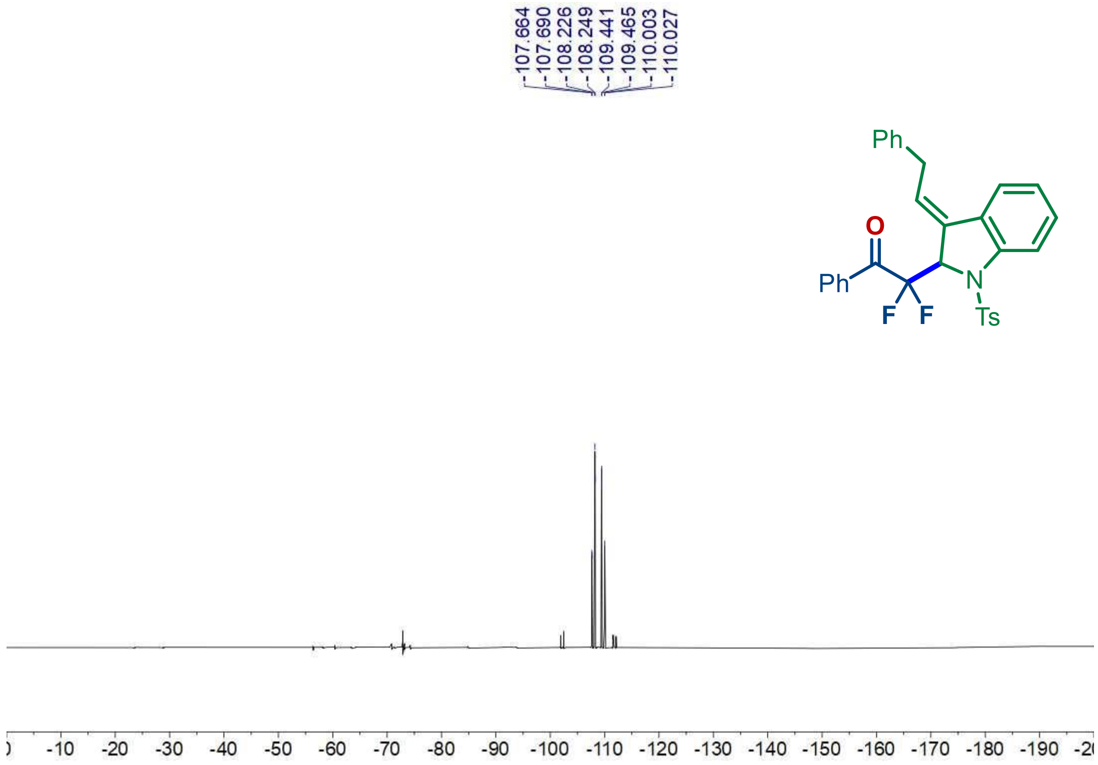

Supplementary Fig. 49 ^{19}F NMR (470 MHz, $CDCl_3$) spectrum of compound **15**

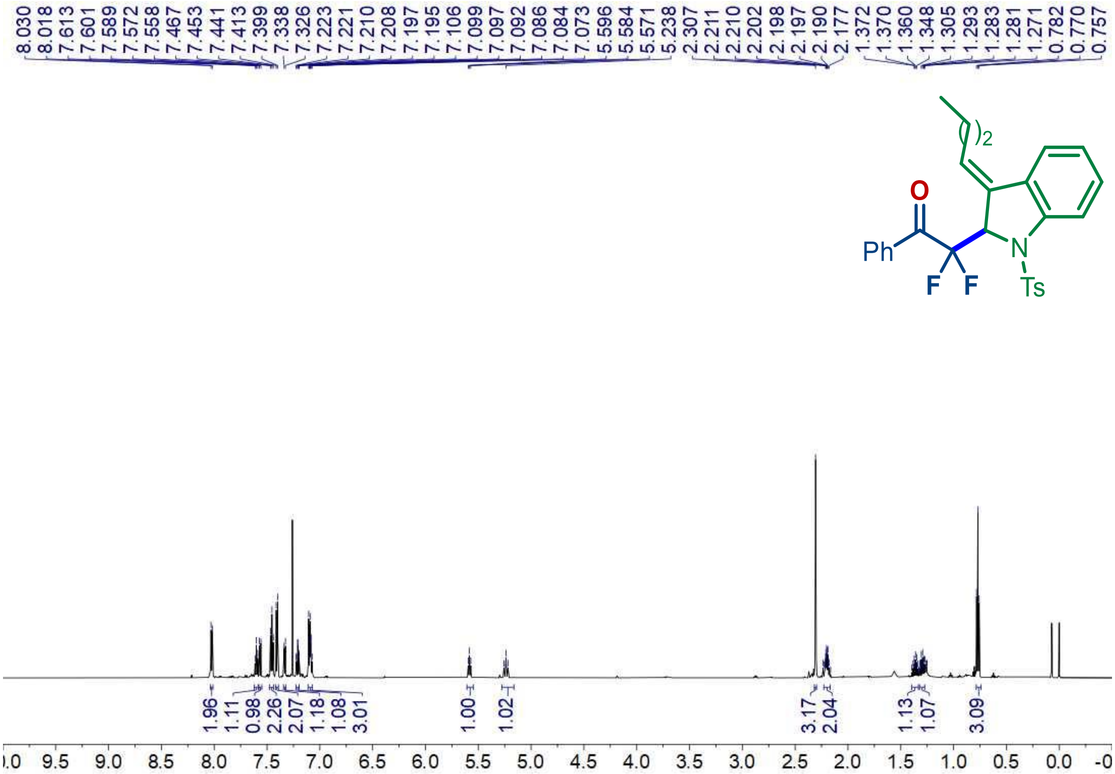

Supplementary Fig. 50 ^{1}H NMR (600 MHz, $CDCl_3$) spectrum of compound **16**

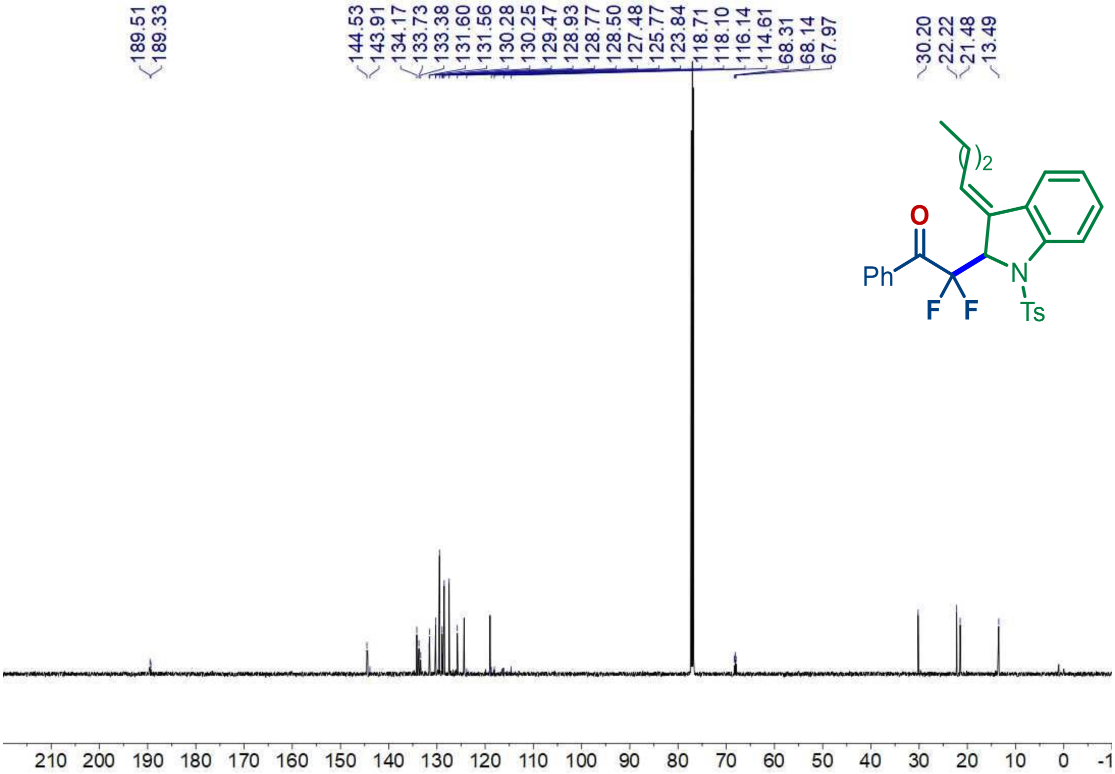

Supplementary Fig. 51 ^{13}C NMR (150 MHz, $CDCl_3$) spectrum of compound **16**

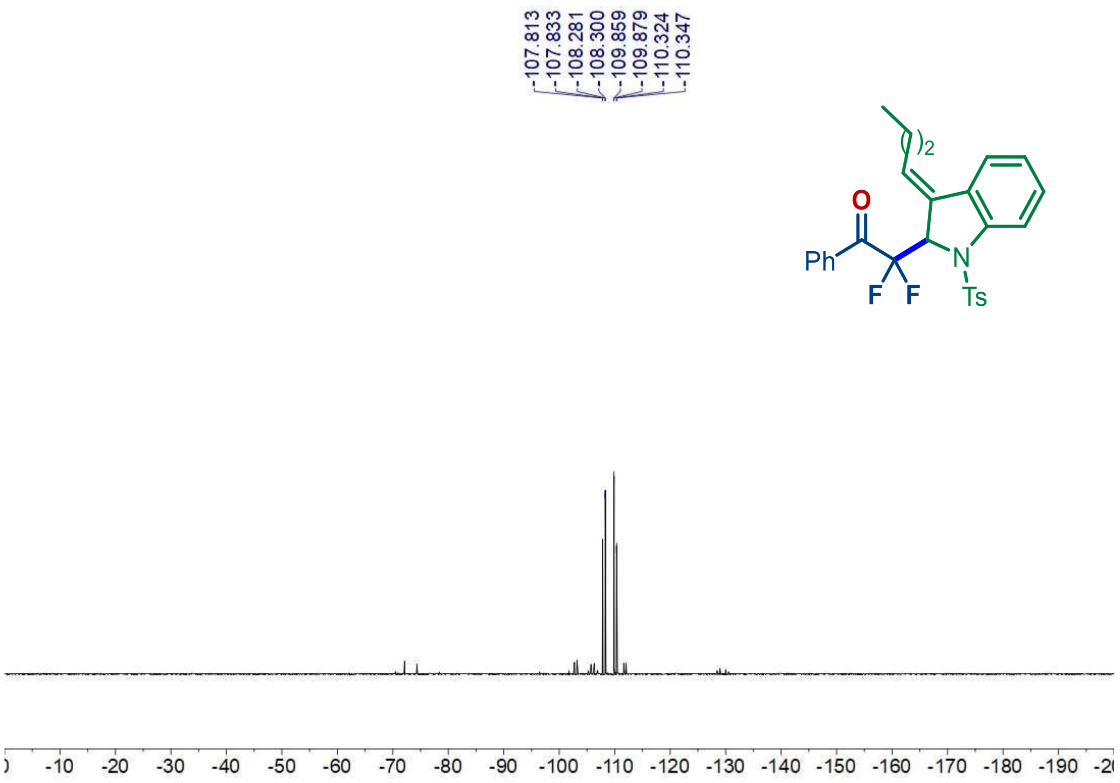

Supplementary Fig. 52 ^{19}F NMR (564 MHz, $CDCl_3$) spectrum of compound **16**

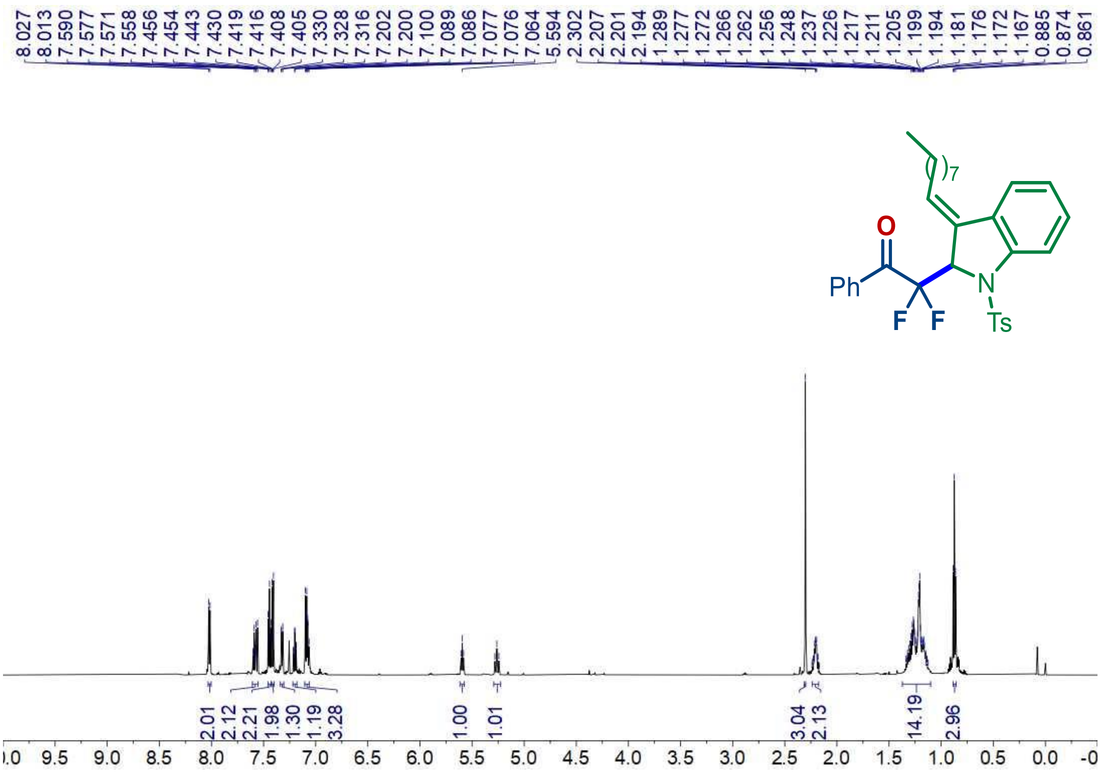

Supplementary Fig. 53 ^{1}H NMR (600 MHz, $CDCl_3$) spectrum of compound **17**

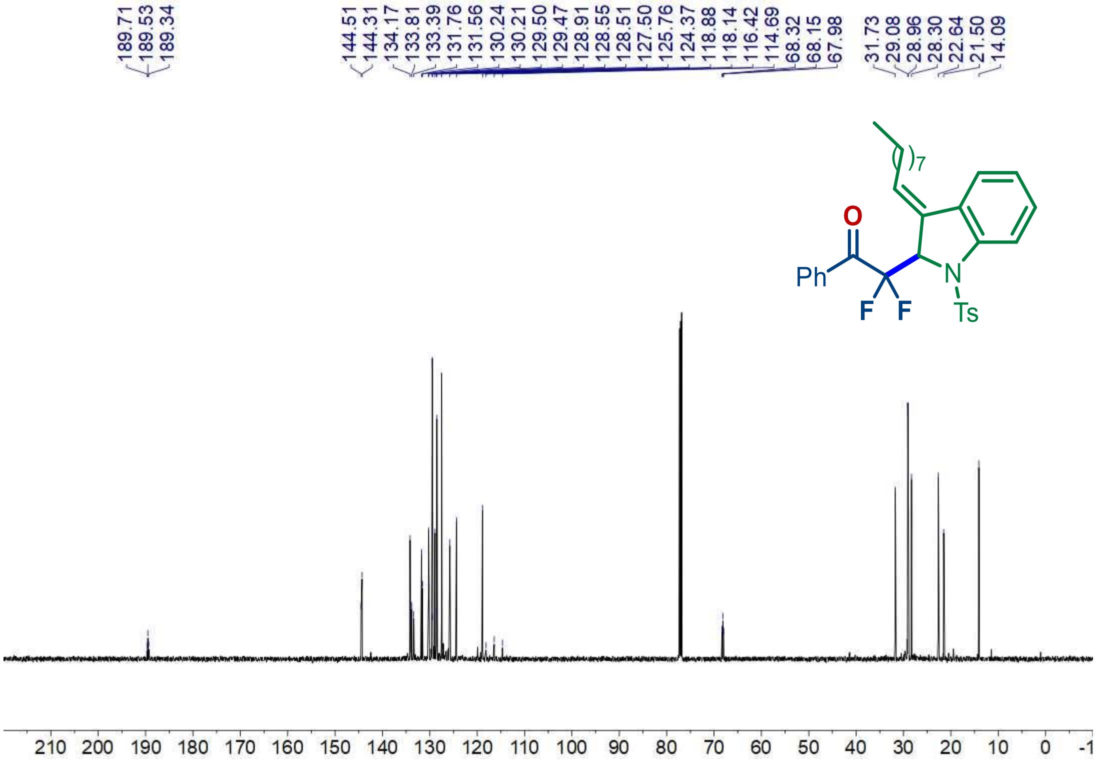

Supplementary Fig. 54 ^{13}C NMR (150 MHz, $CDCl_3$) spectrum of compound **17**

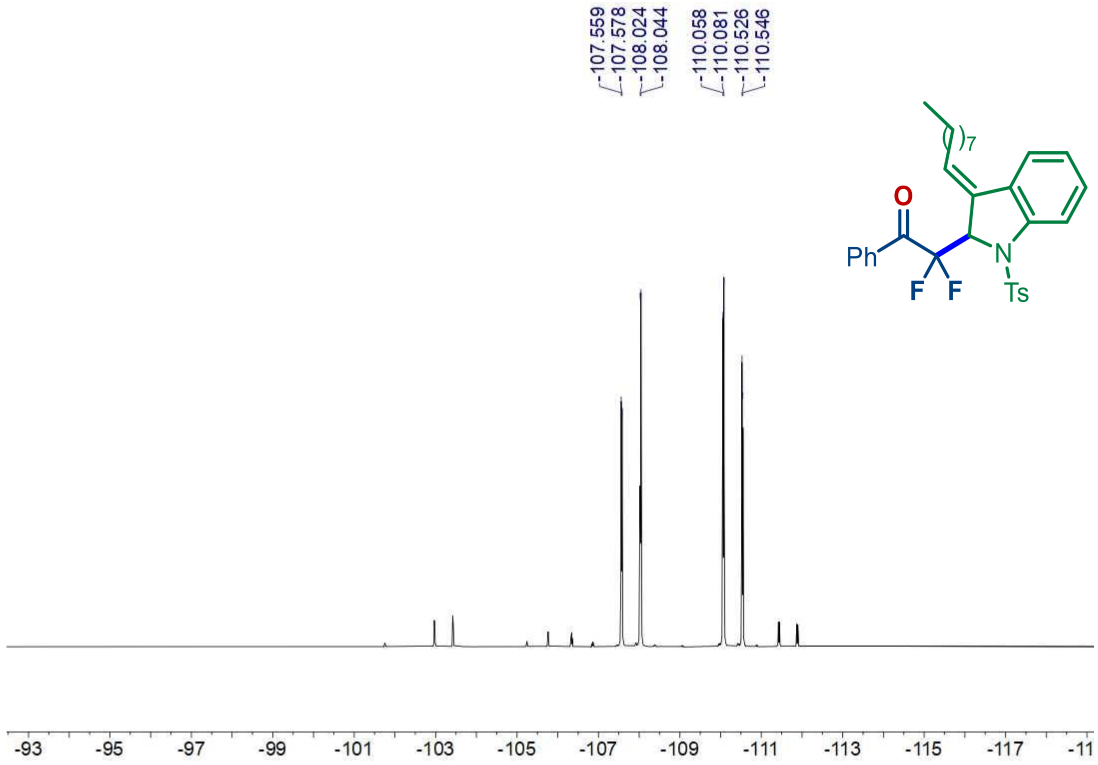

Supplementary Fig. 55 ^{19}F NMR (564 MHz, $CDCl_3$) spectrum of compound **17**

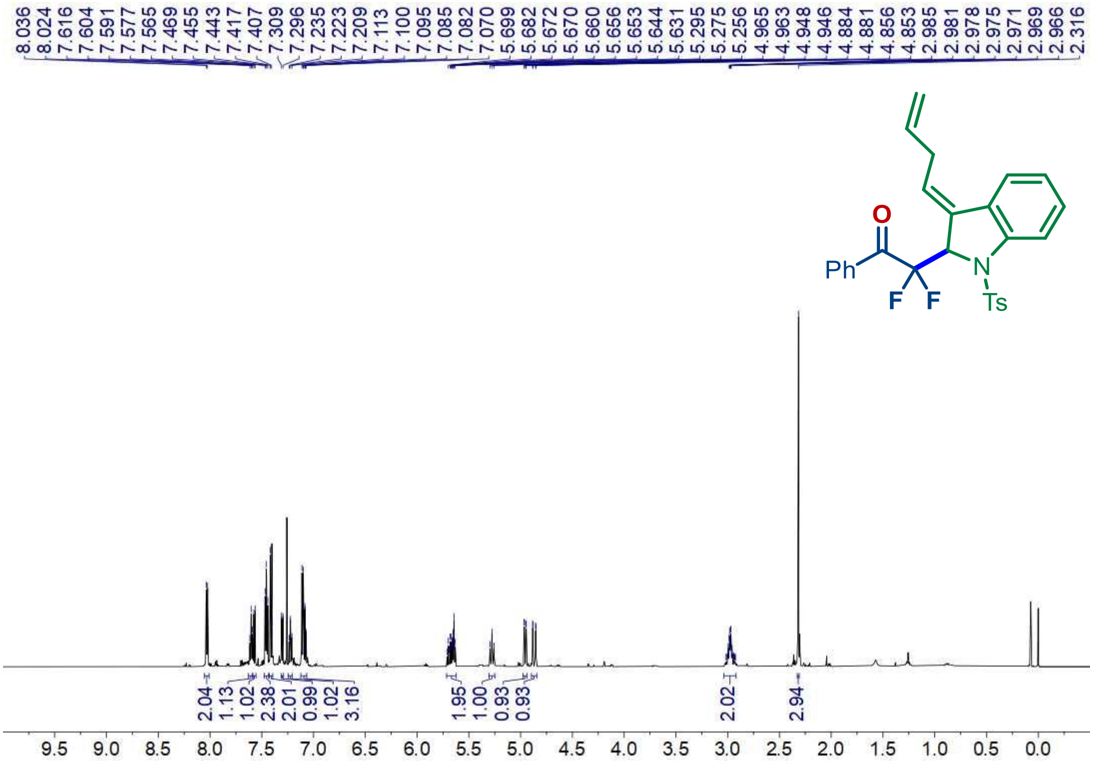

Supplementary Fig. 56 ^{1}H NMR (600 MHz, $CDCl_3$) spectrum of compound **18**

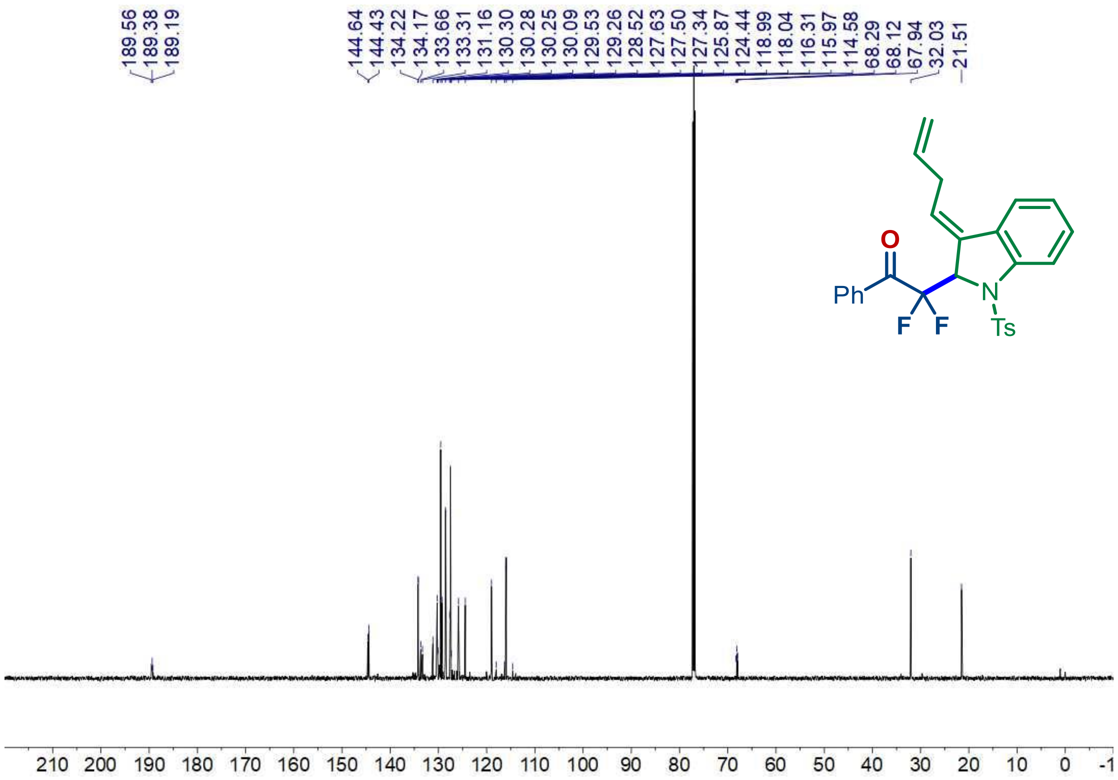

Supplementary Fig. 57 ^{13}C NMR (150 MHz, $CDCl_3$) spectrum of compound **18**

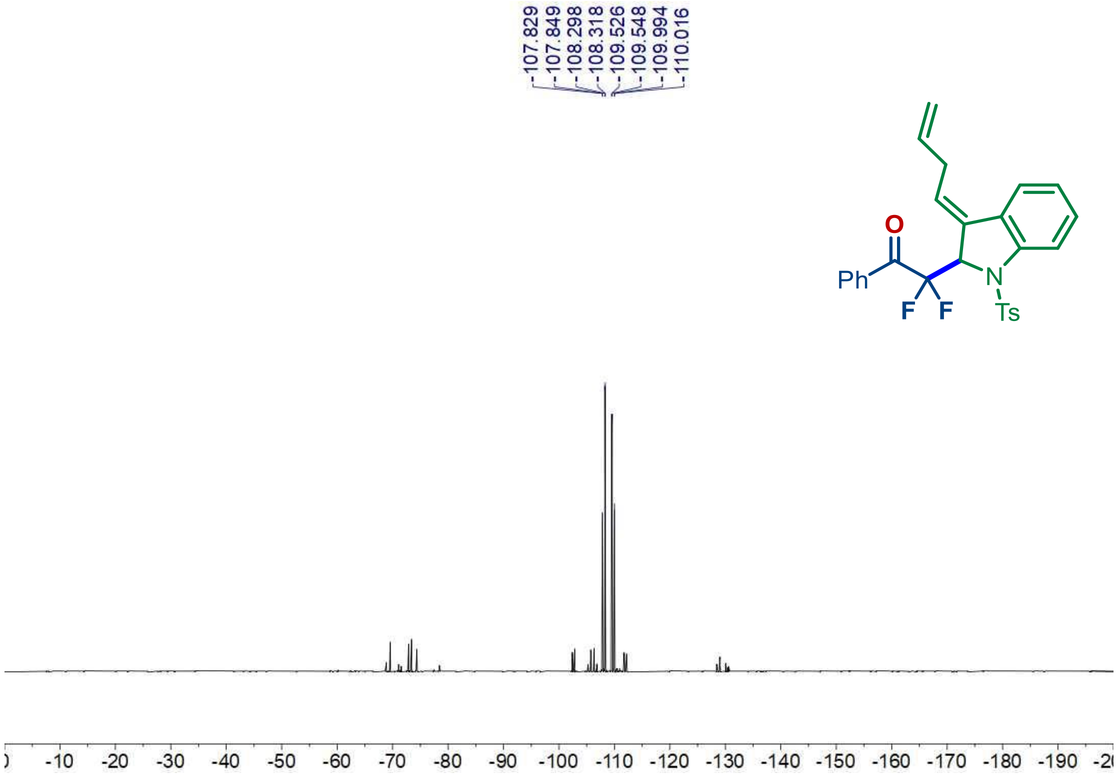

Supplementary Fig. 58 ^{19}F NMR (564 MHz, $CDCl_3$) spectrum of compound **18**

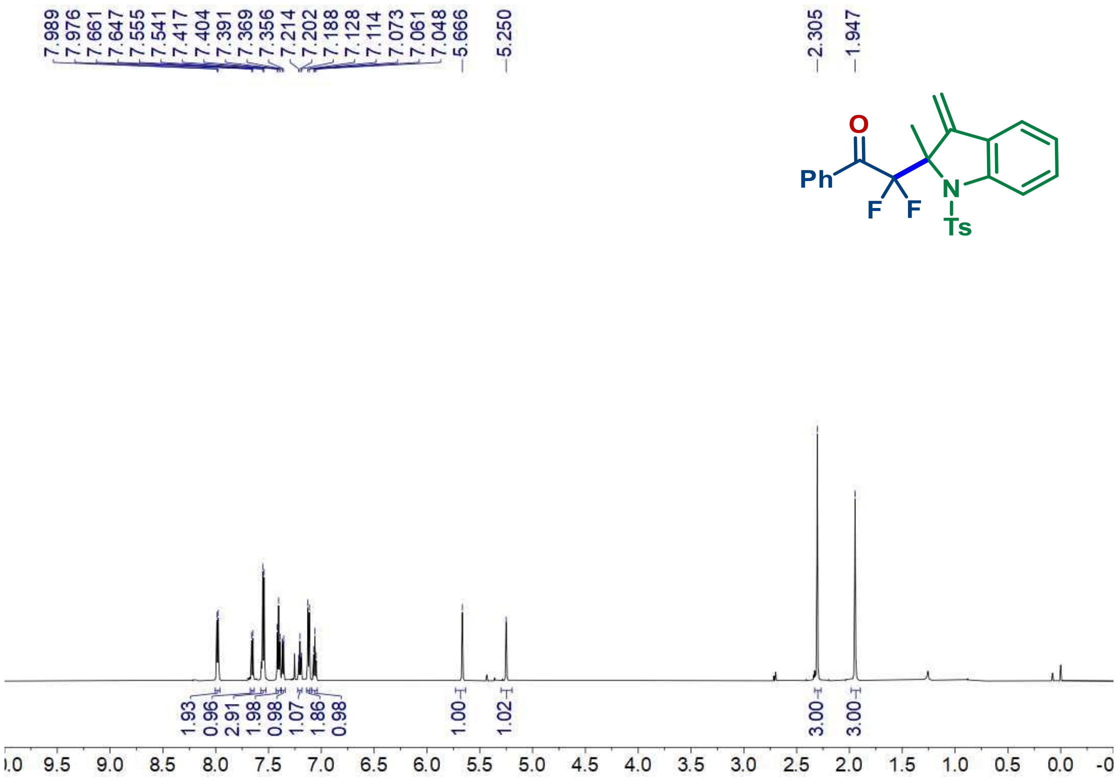

Supplementary Fig. 59 ^{1}H NMR (600 MHz, $CDCl_3$) spectrum of compound **19**

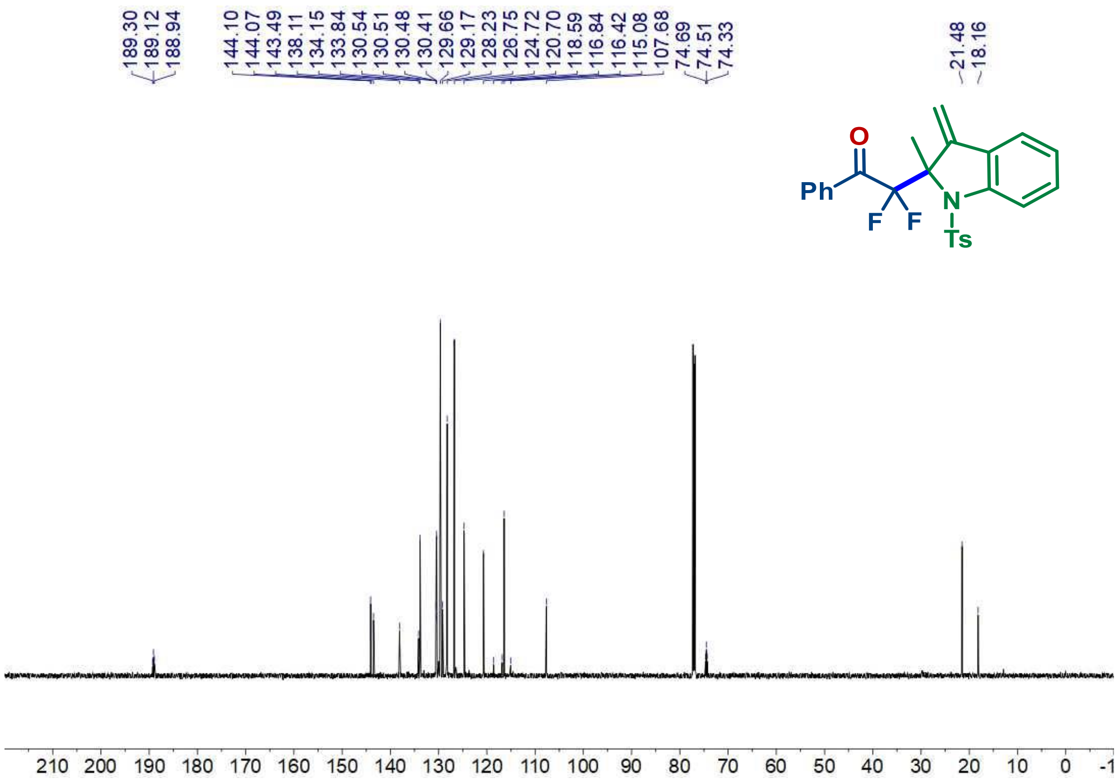

Supplementary Fig. 60 ^{13}C NMR (150 MHz, $CDCl_3$) spectrum of compound **19**

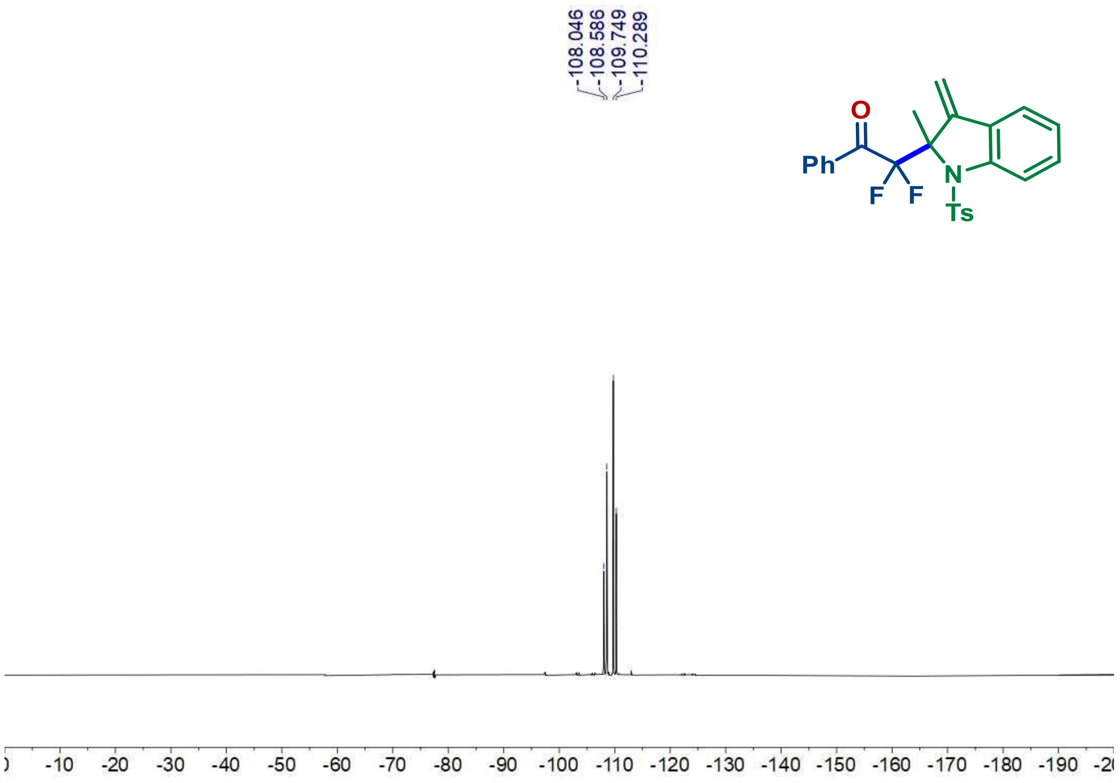

Supplementary Fig. 61 ^{19}F NMR (470 MHz, $CDCl_3$) spectrum of compound **19**

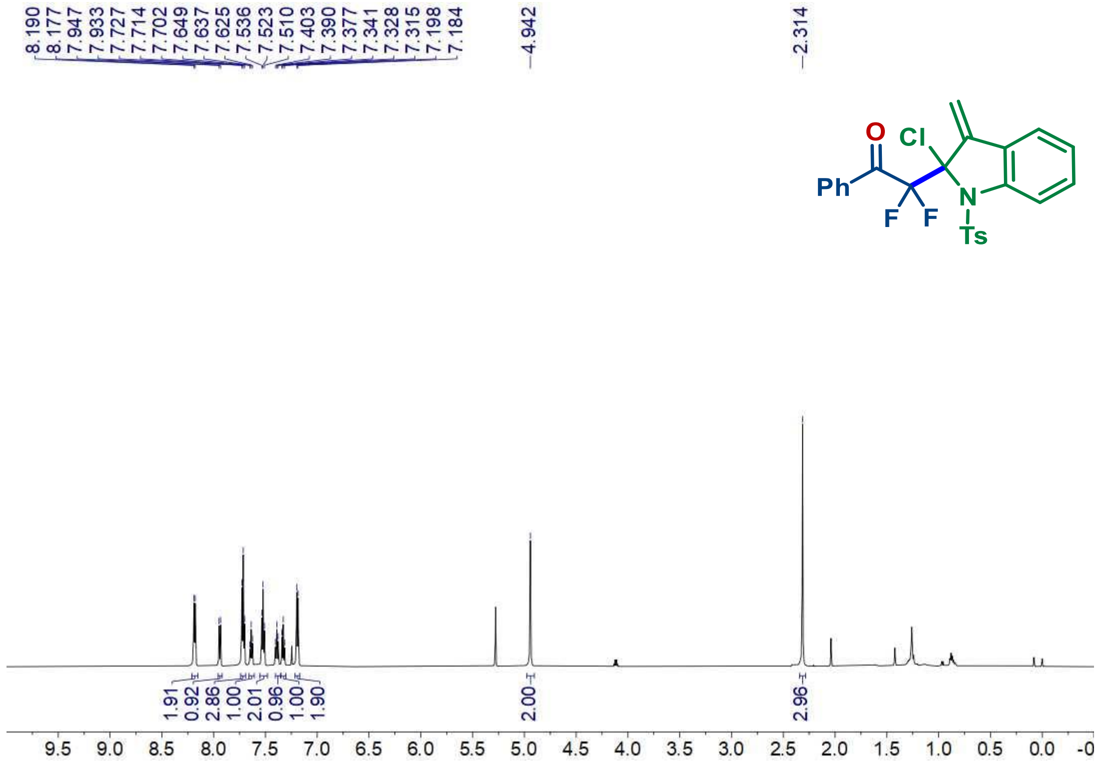

Supplementary Fig. 62 ^{1}H NMR (600 MHz, $CDCl_3$) spectrum of compound **20**

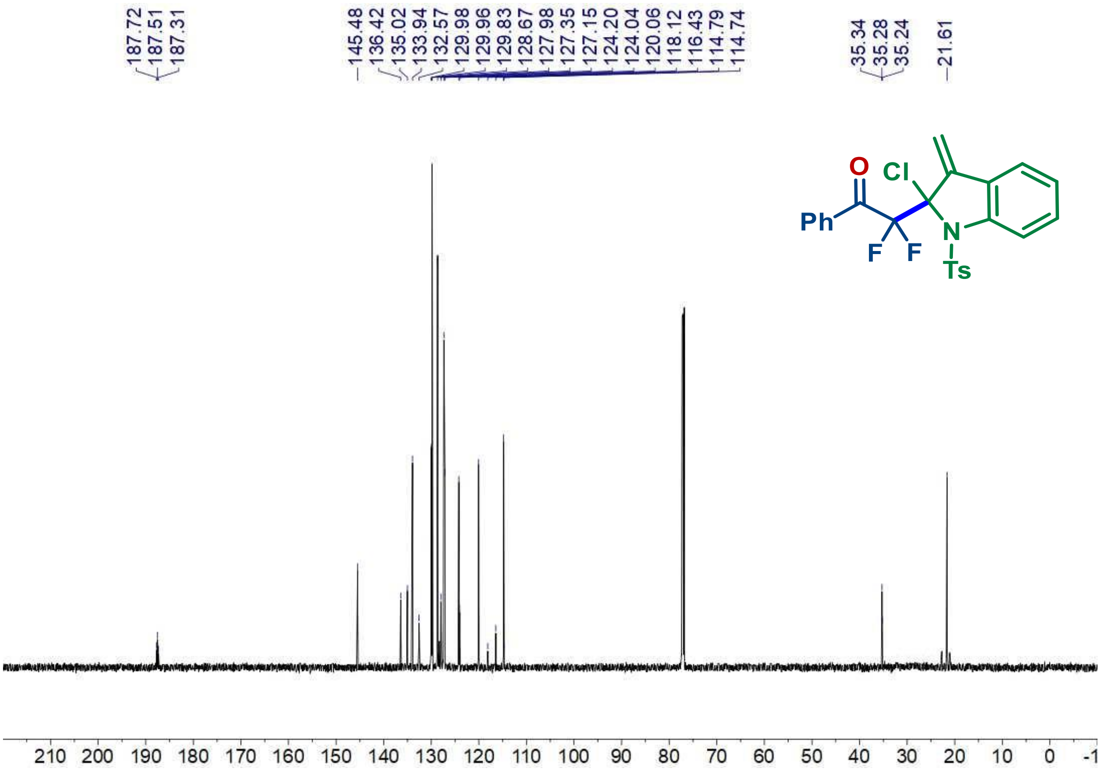

Supplementary Fig. 63 ^{13}C NMR (150 MHz, $CDCl_3$) spectrum of compound **20**

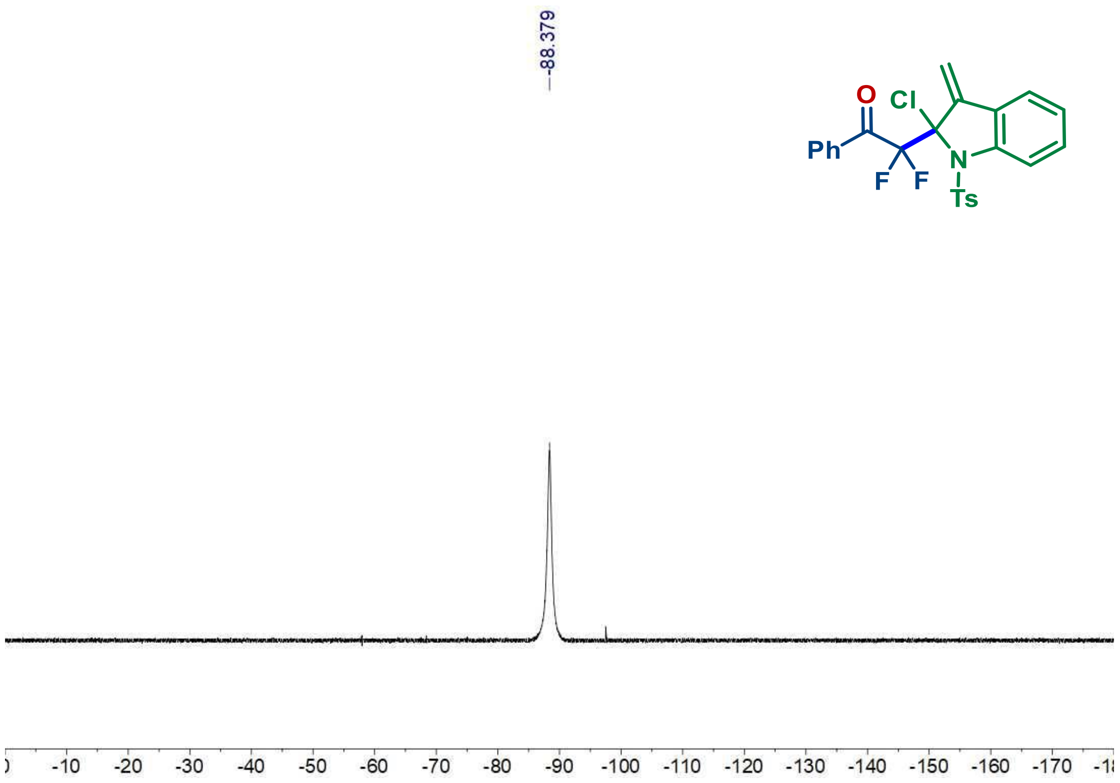

Supplementary Fig. 64 ^{19}F NMR (564 MHz, $CDCl_3$) spectrum of compound **20**

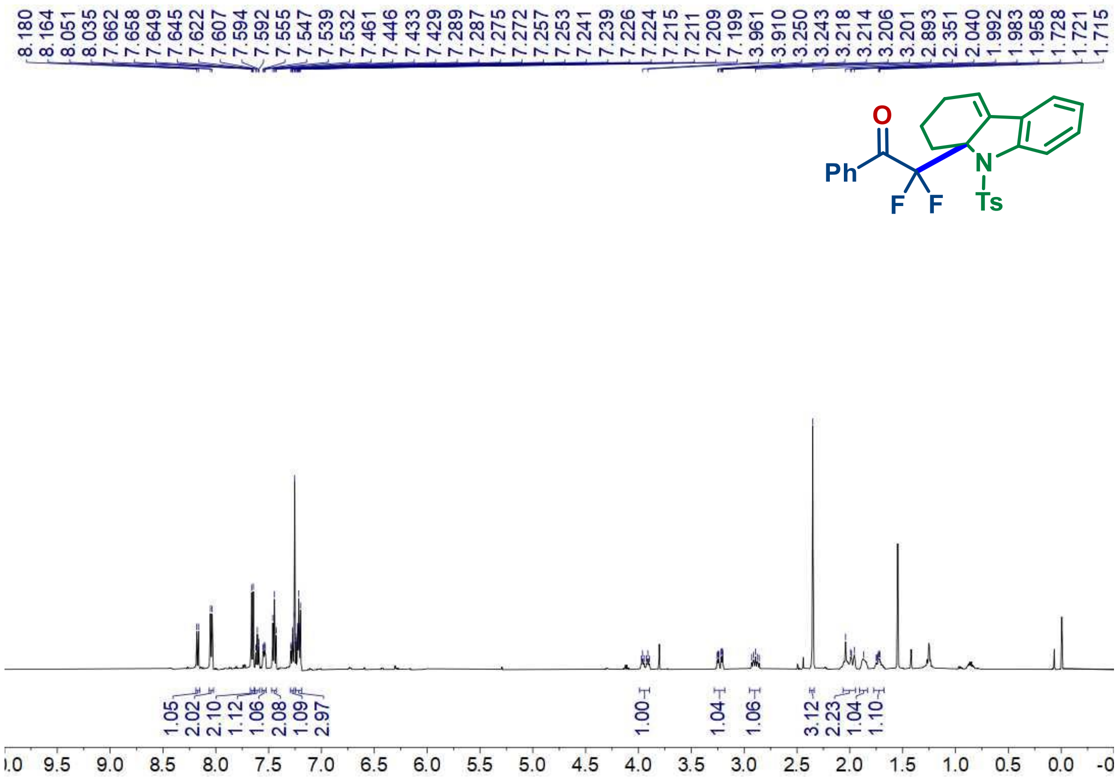

Supplementary Fig. 65 ^{1}H NMR (500 MHz, $CDCl_3$) spectrum of compound **21**

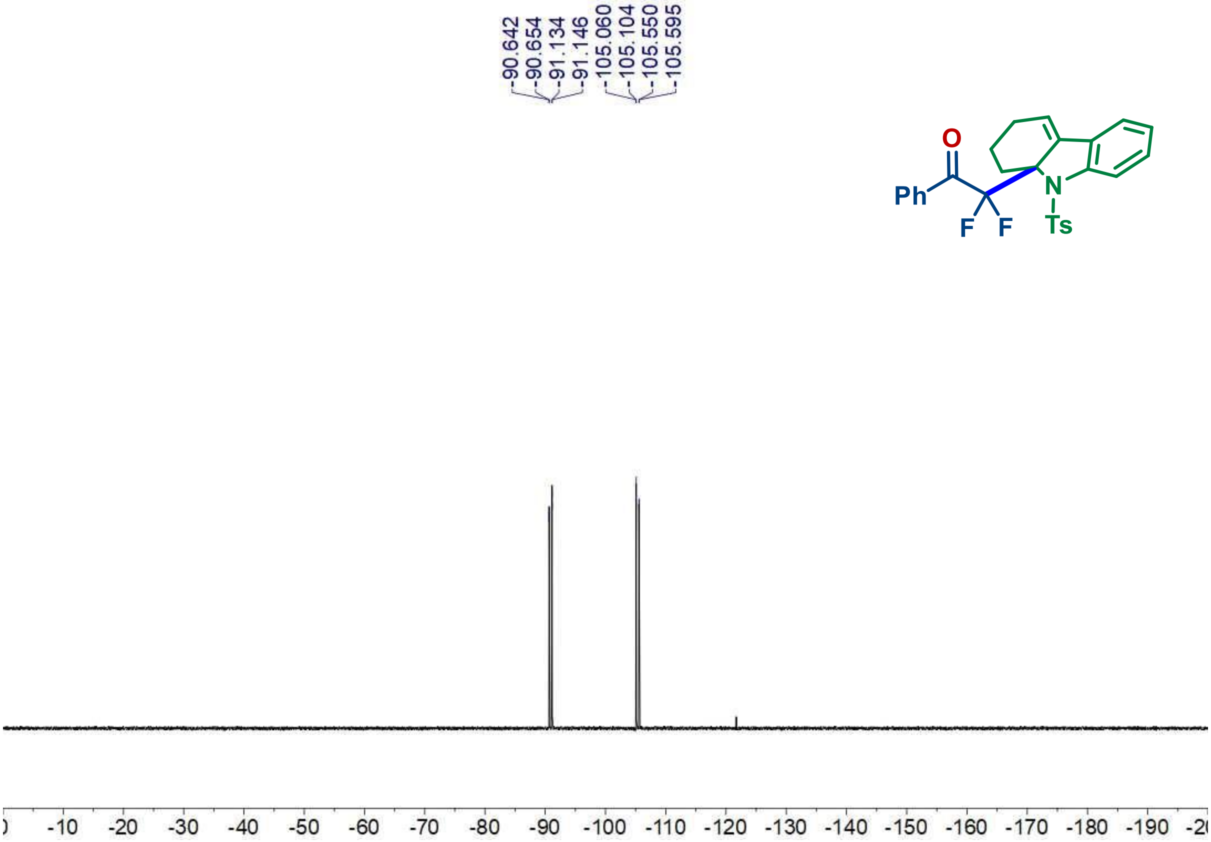

Supplementary Fig. 66 ^{19}F NMR (564 MHz, $CDCl_3$) spectrum of compound **21**

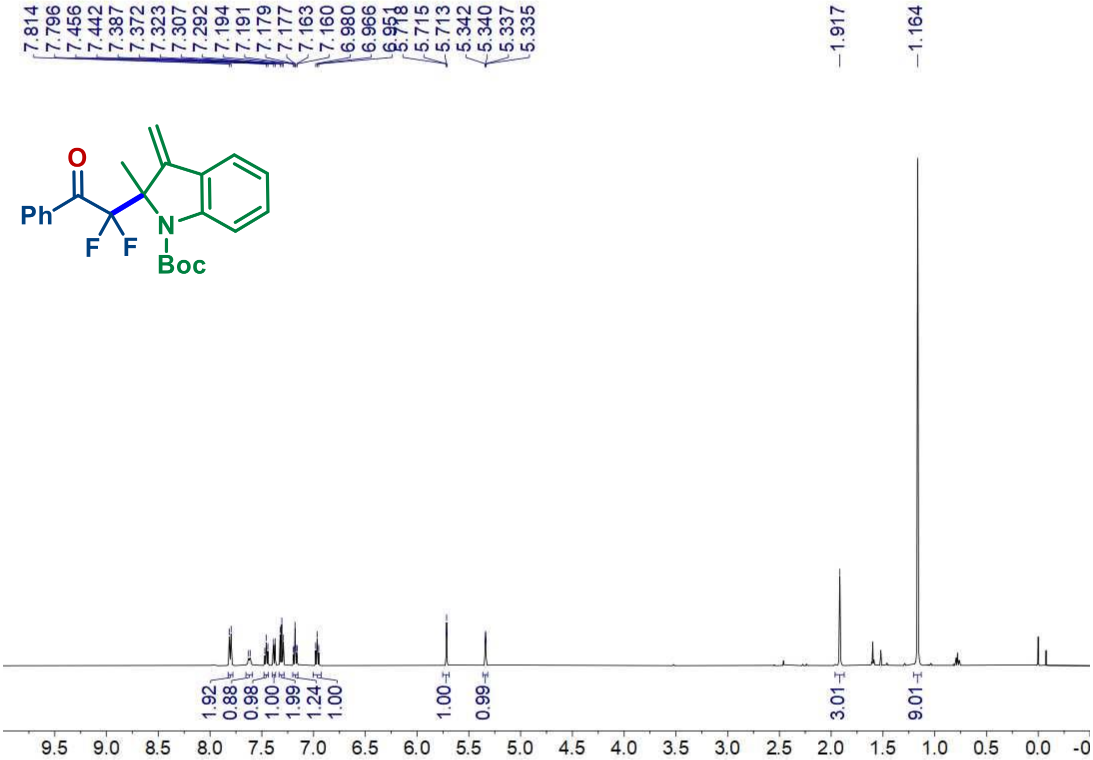

Supplementary Fig. 67 ^{1}H NMR (500 MHz, $CDCl_3$) spectrum of compound **22**

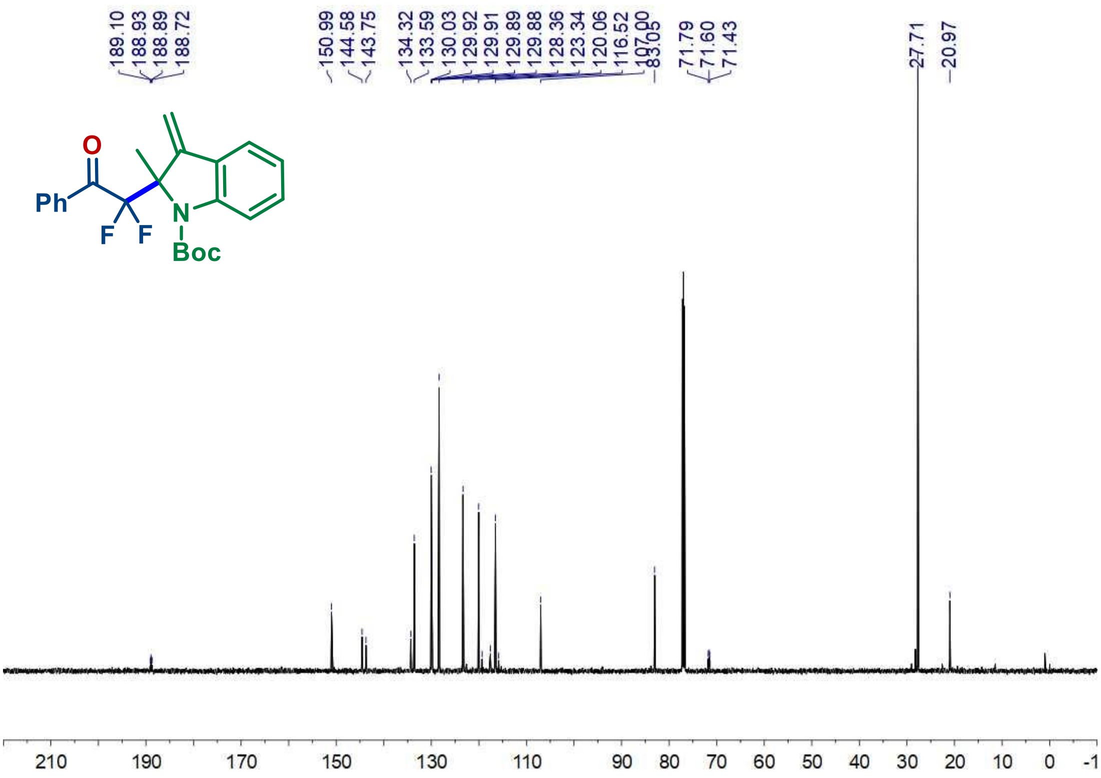

Supplementary Fig. 68 ^{13}C NMR (150 MHz, $CDCl_3$) spectrum of compound **22**

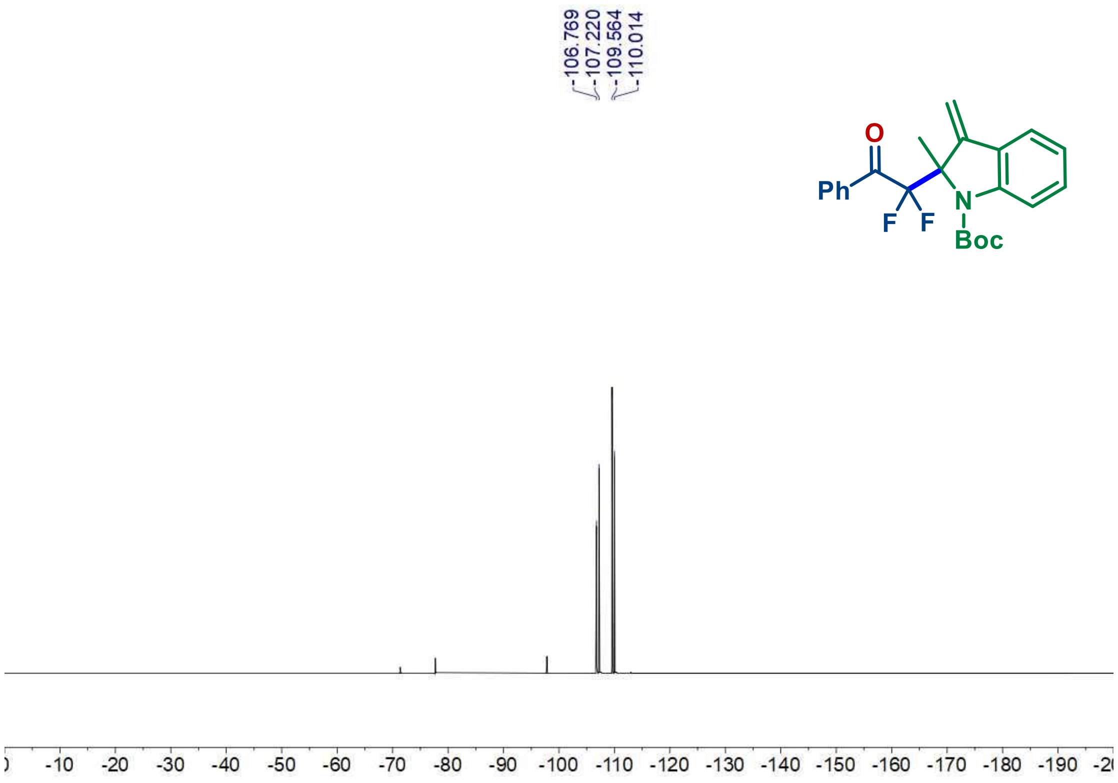

Supplementary Fig. 69 ^{19}F NMR (564 MHz, $CDCl_3$) spectrum of compound **22**

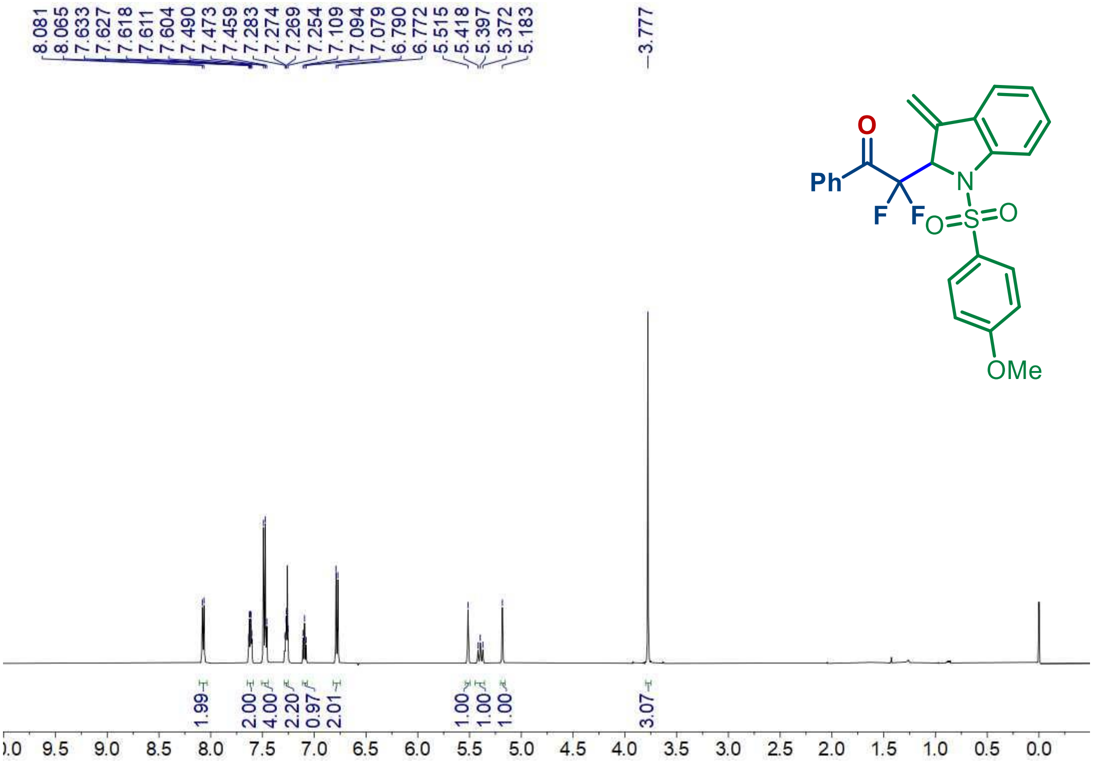

Supplementary Fig. 70 ^{1}H NMR (500 MHz, $CDCl_3$) spectrum of compound **23**

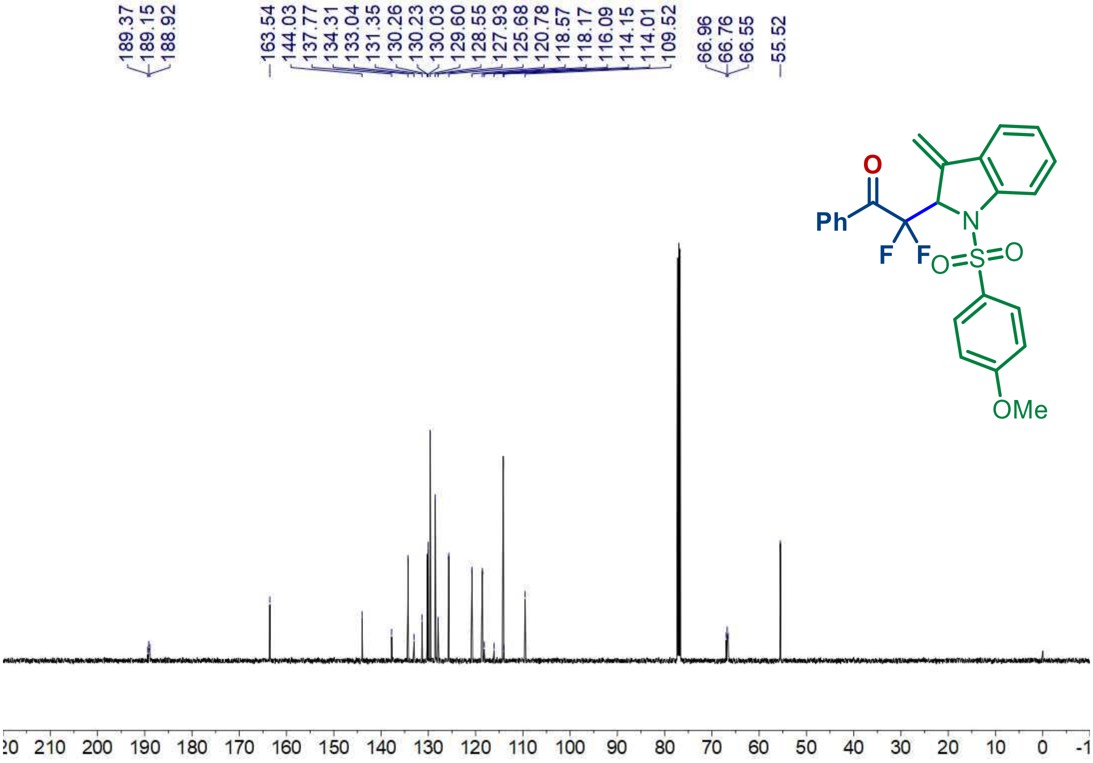

Supplementary Fig. 71 ^{13}C NMR (125 MHz, $CDCl_3$) spectrum of compound **23**

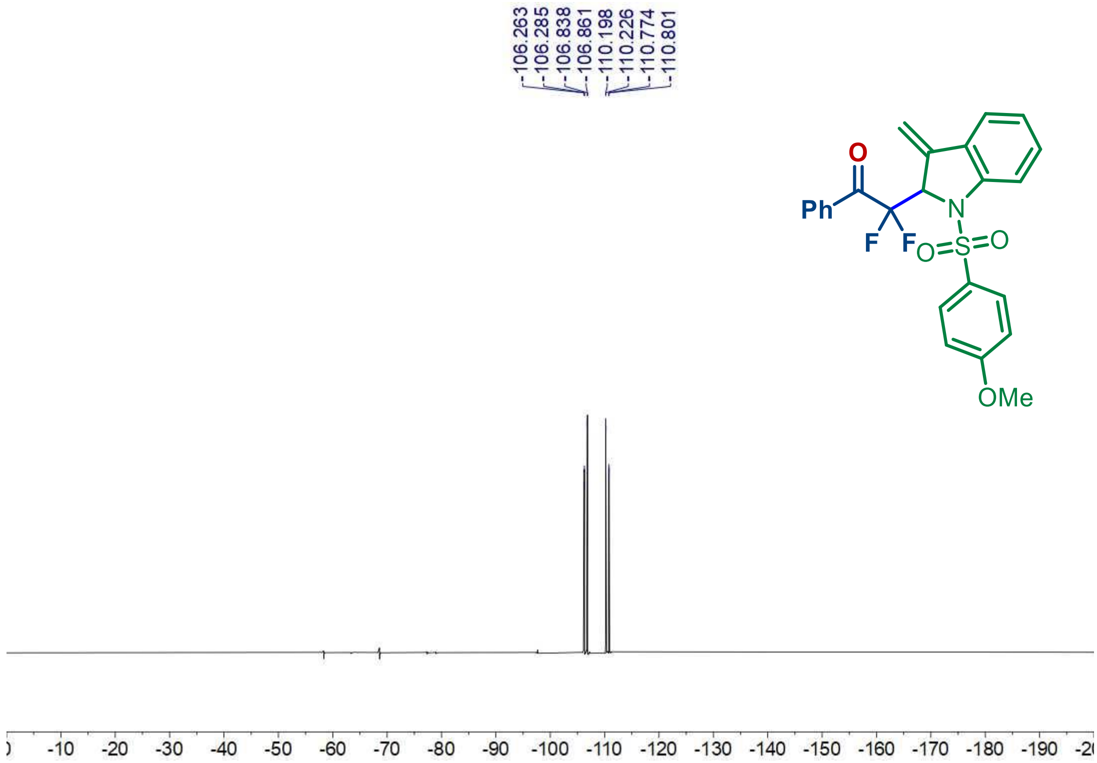

Supplementary Fig. 72 ^{19}F NMR (470 MHz, $CDCl_3$) spectrum of compound **23**

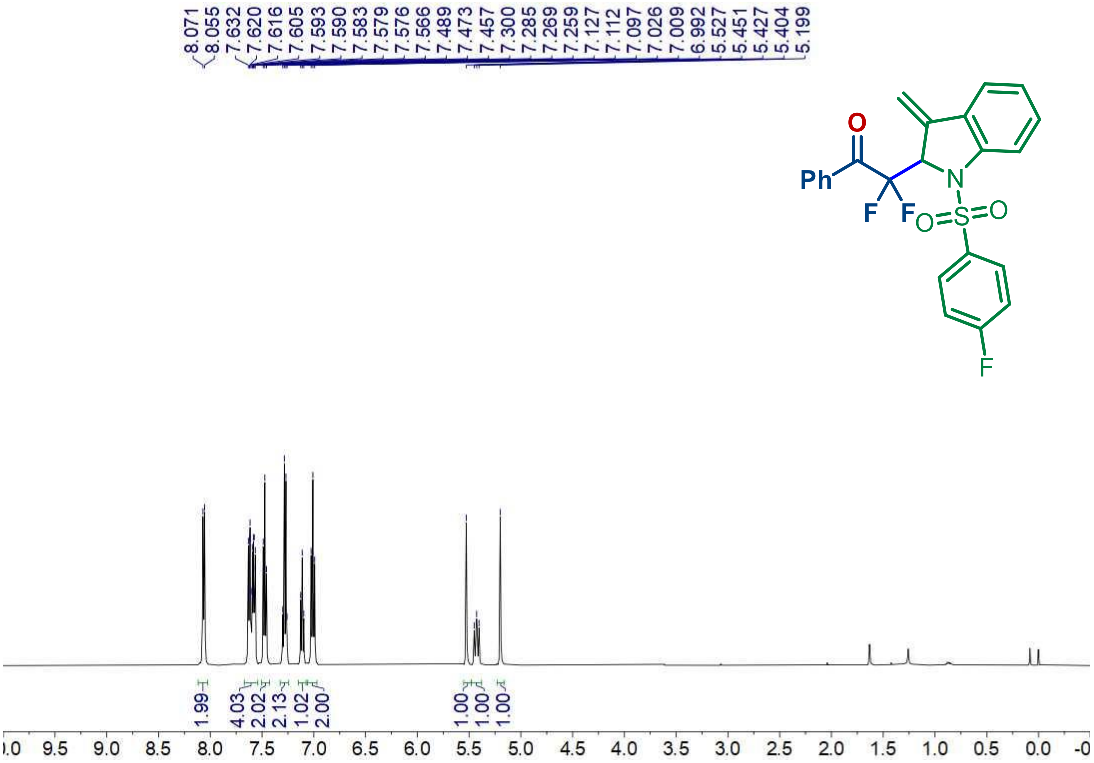

Supplementary Fig. 73 ^{1}H NMR (500 MHz, $CDCl_3$) spectrum of compound **24**

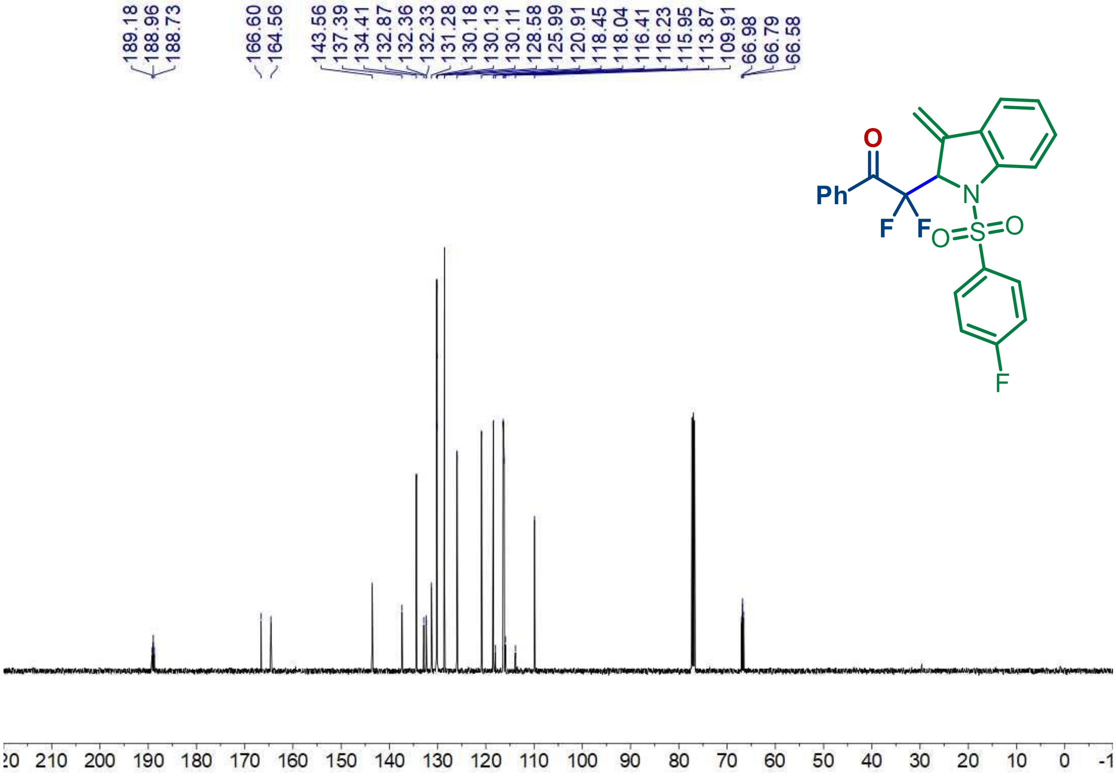

Supplementary Fig. 74 ^{13}C NMR (125 MHz, $CDCl_3$) spectrum of compound **24**

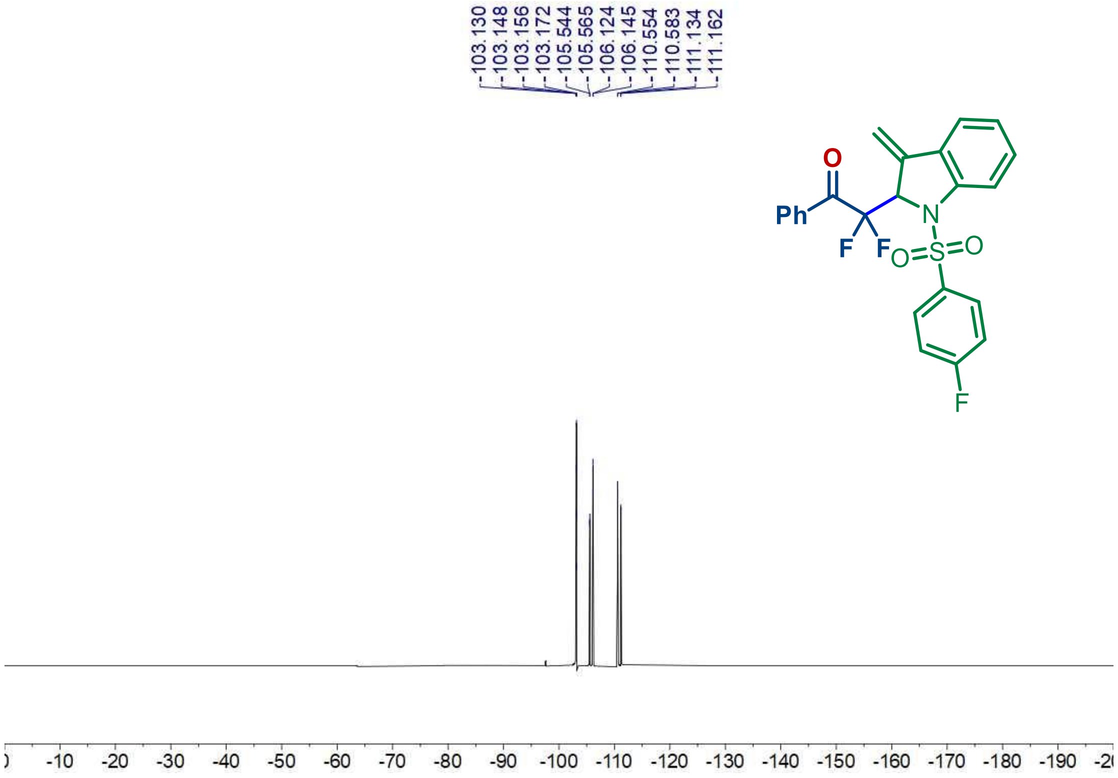

Supplementary Fig. 75 ^{19}F NMR (470 MHz, $CDCl_3$) spectrum of compound **24**

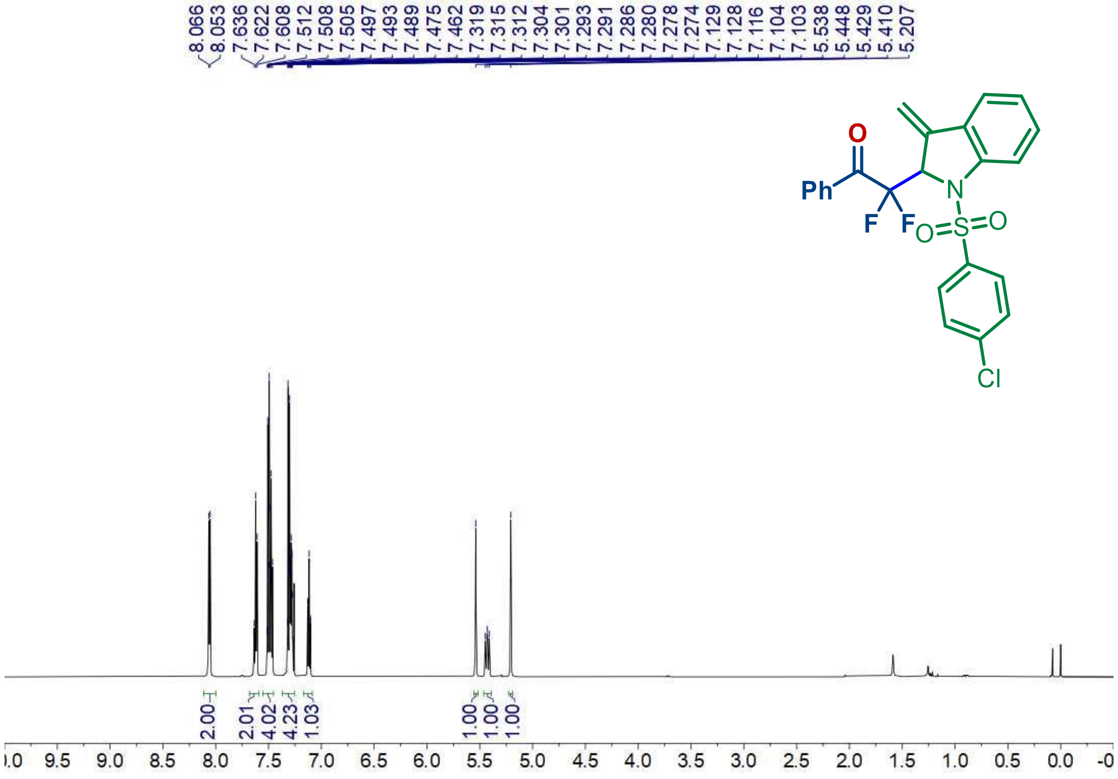

Supplementary Fig. 76 ^{1}H NMR (600 MHz, $CDCl_3$) spectrum of compound **25**

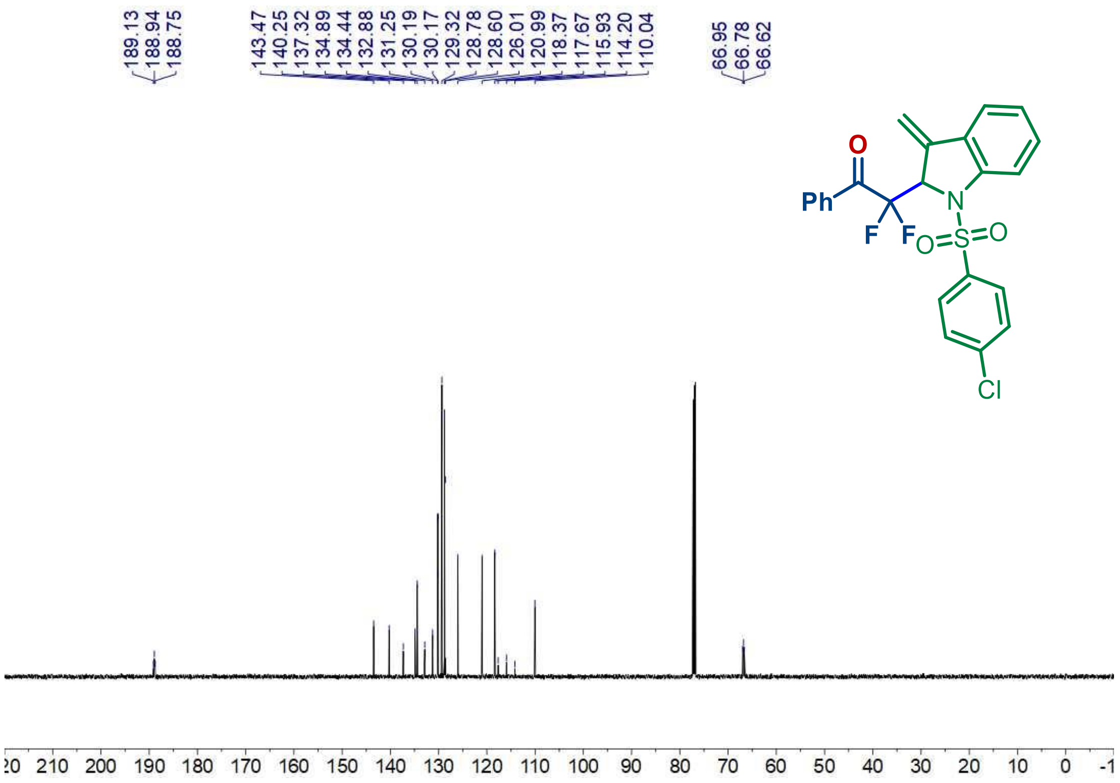

Supplementary Fig. 77 ^{13}C NMR (150 MHz, $CDCl_3$) spectrum of compound **25**

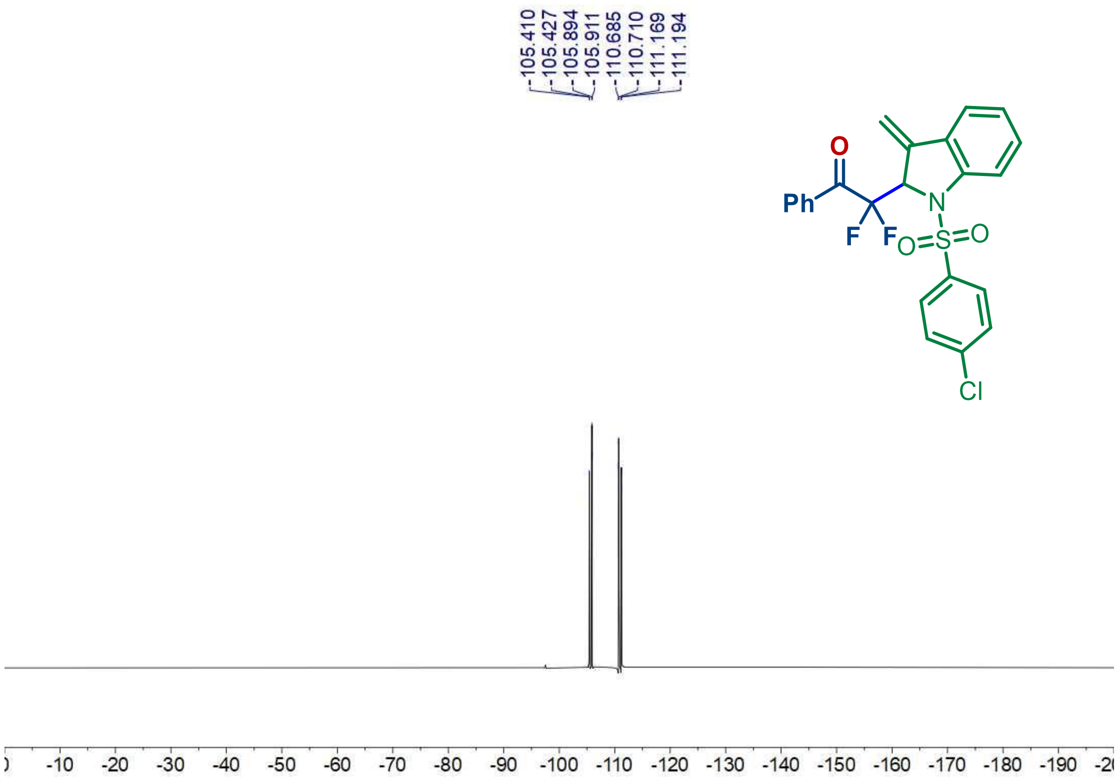

Supplementary Fig. 78 ^{19}F NMR (564 MHz, $CDCl_3$) spectrum of compound **25**

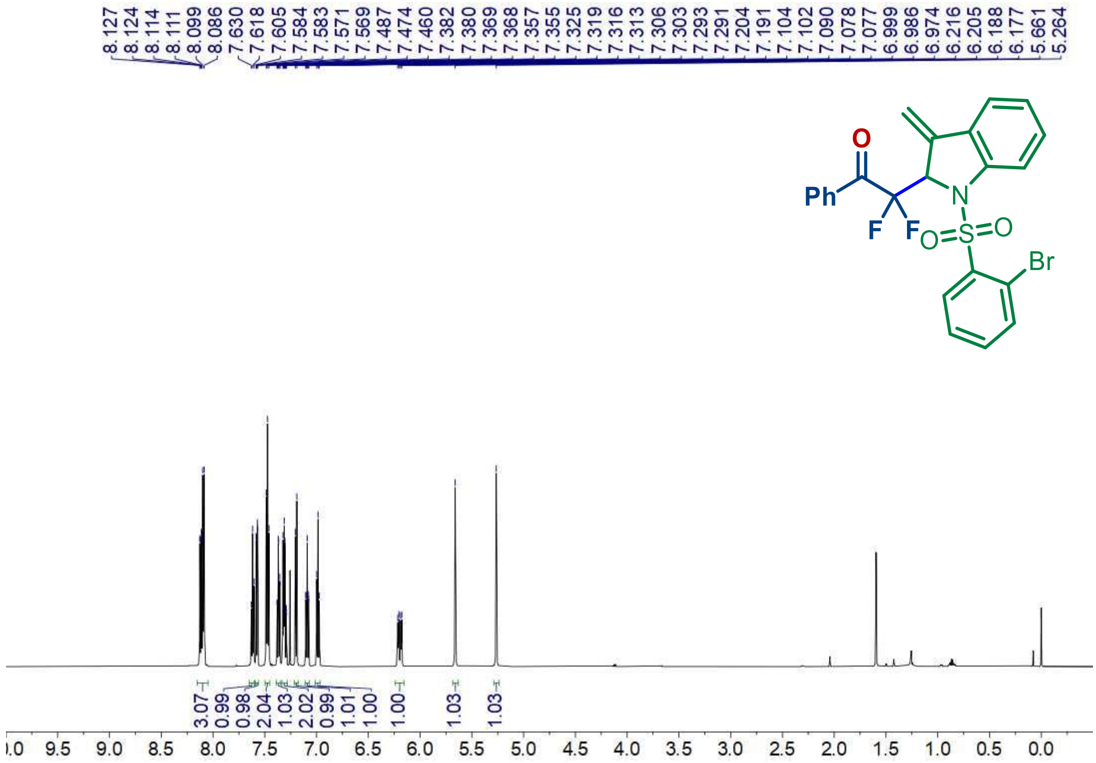

Supplementary Fig. 79 ^{1}H NMR (600 MHz, $CDCl_3$) spectrum of compound **26**

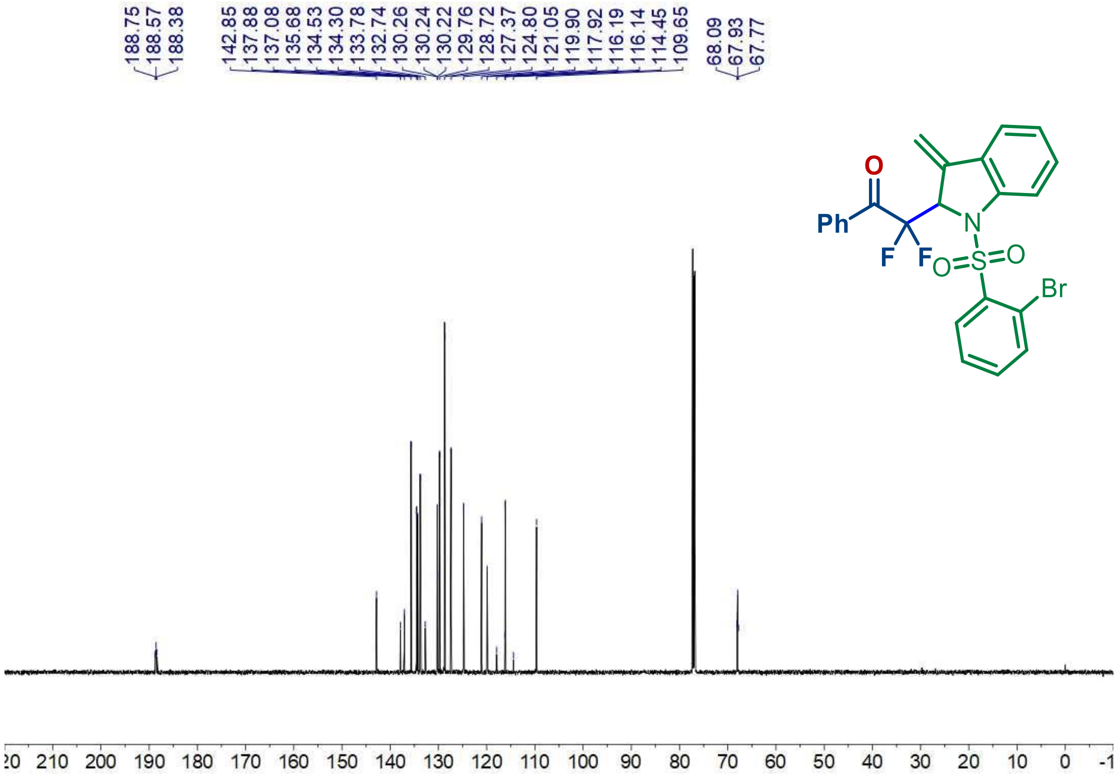

Supplementary Fig. 80 ^{13}C NMR (150 MHz, $CDCl_3$) spectrum of compound **26**

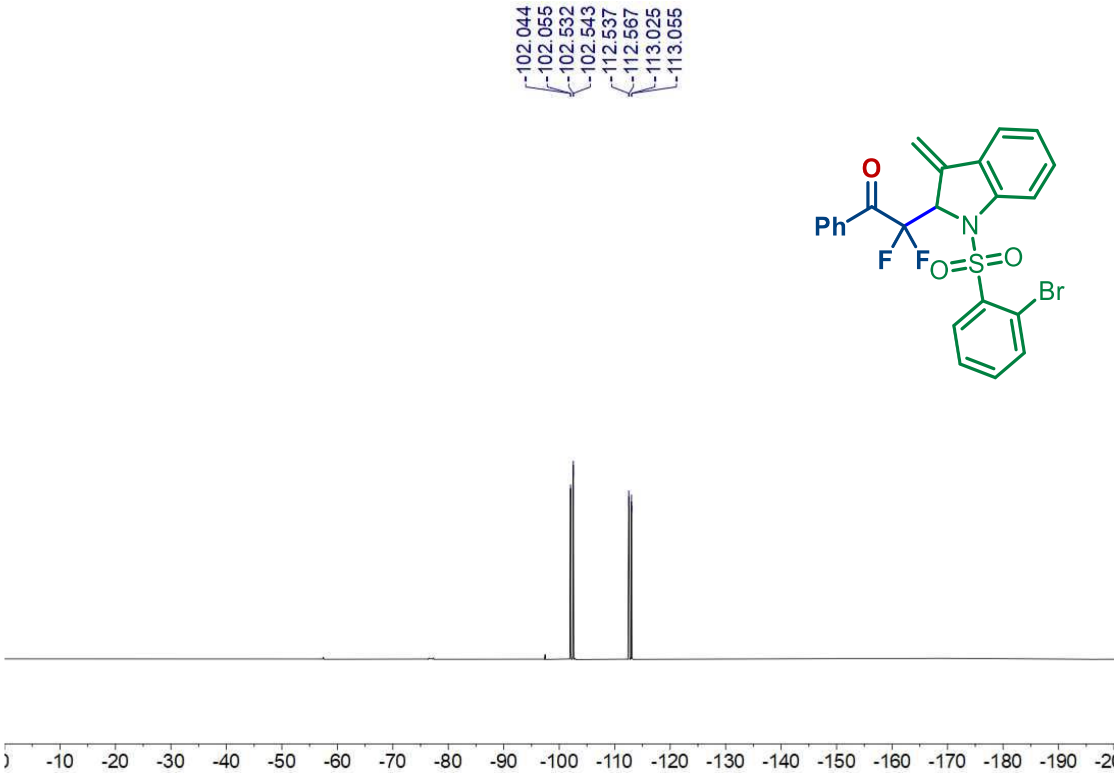

Supplementary Fig. 81 ^{19}F NMR (564 MHz, $CDCl_3$) spectrum of compound **26**

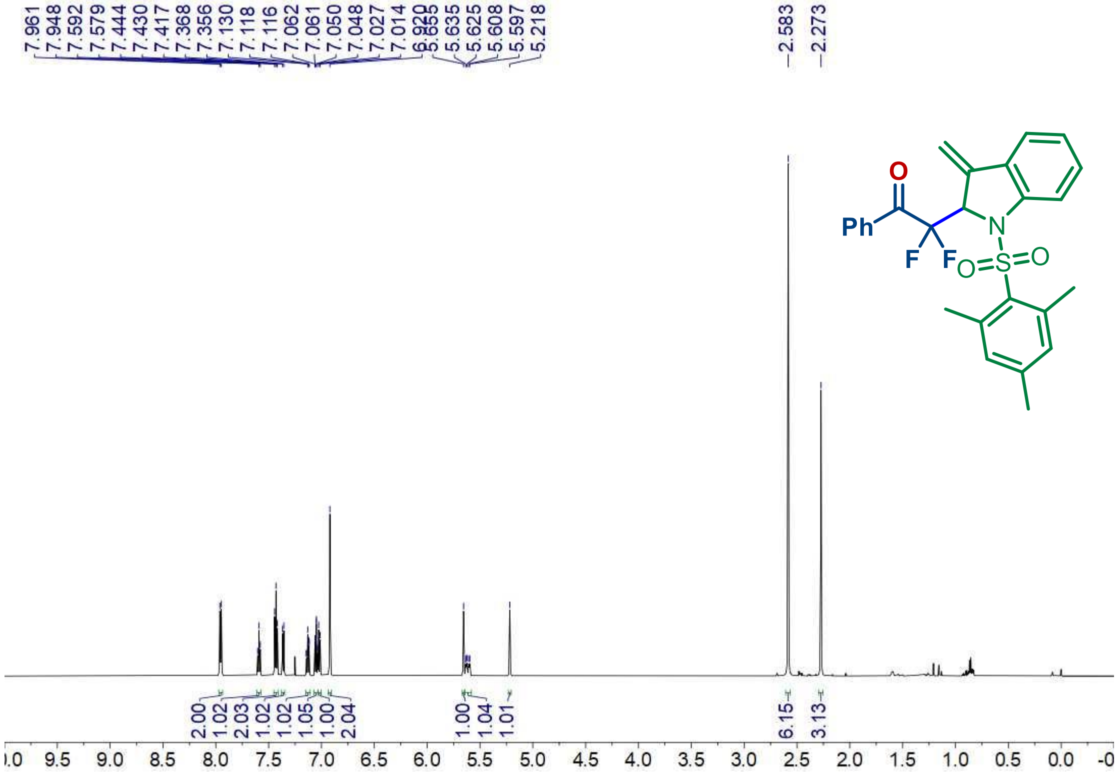

Supplementary Fig. 82 ^{1}H NMR (600 MHz, $CDCl_3$) spectrum of compound **27**

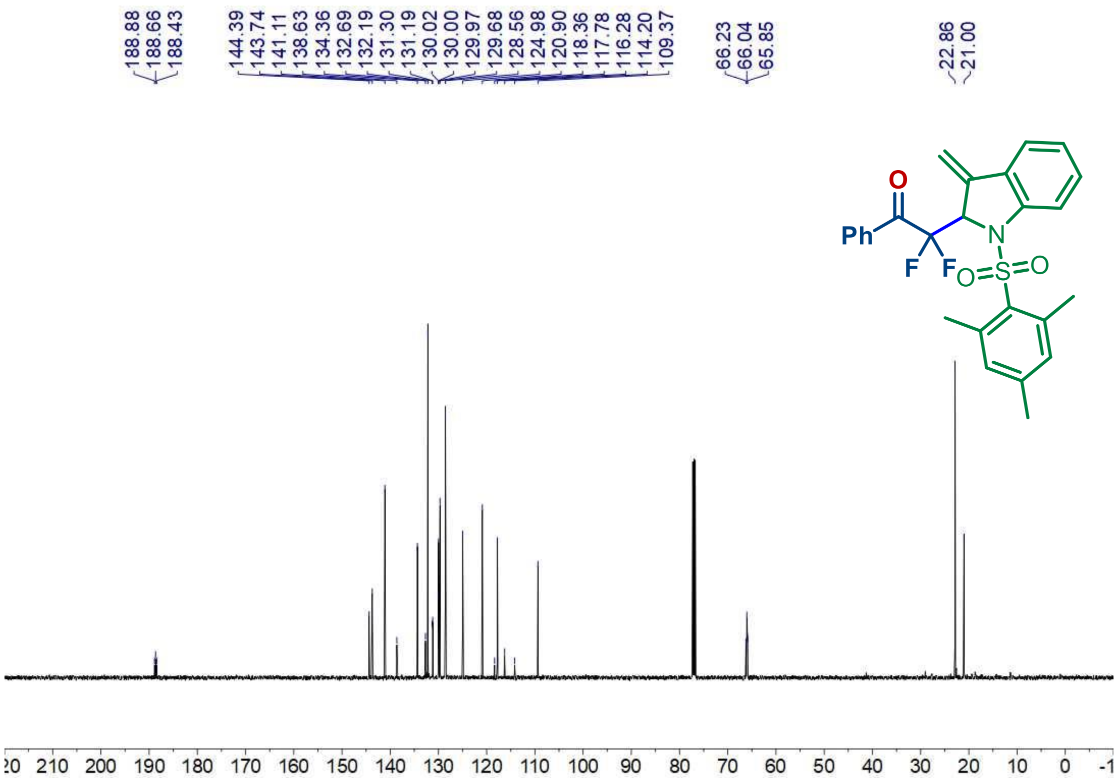

Supplementary Fig. 83 ^{13}C NMR (125 MHz, $CDCl_3$) spectrum of compound **27**

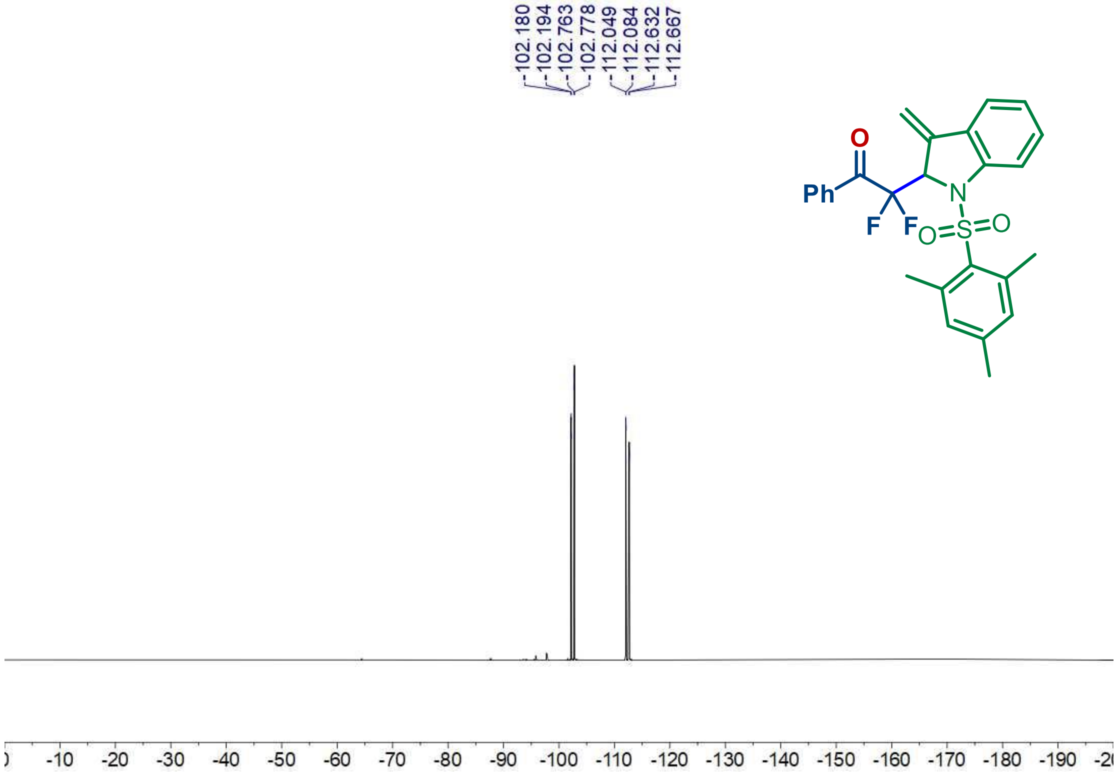

Supplementary Fig. 84 ^{19}F NMR (470 MHz, $CDCl_3$) spectrum of compound **27**

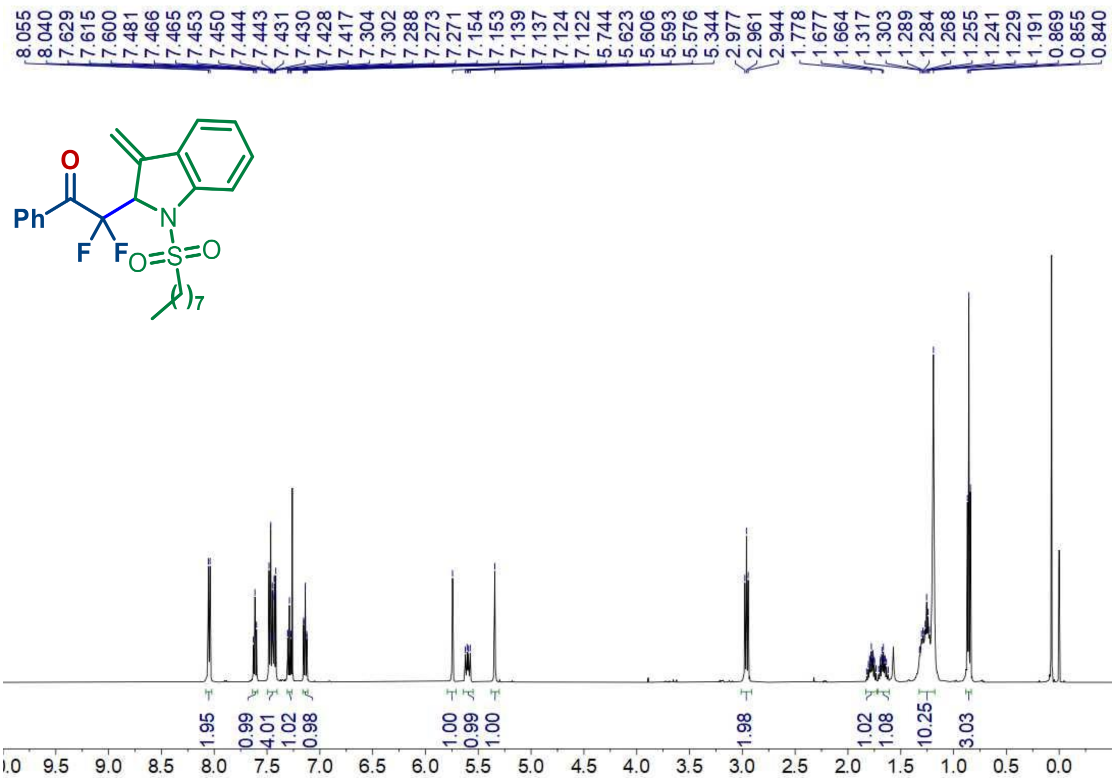

Supplementary Fig. 85 ^{1}H NMR (500 MHz, $CDCl_3$) spectrum of compound **28**

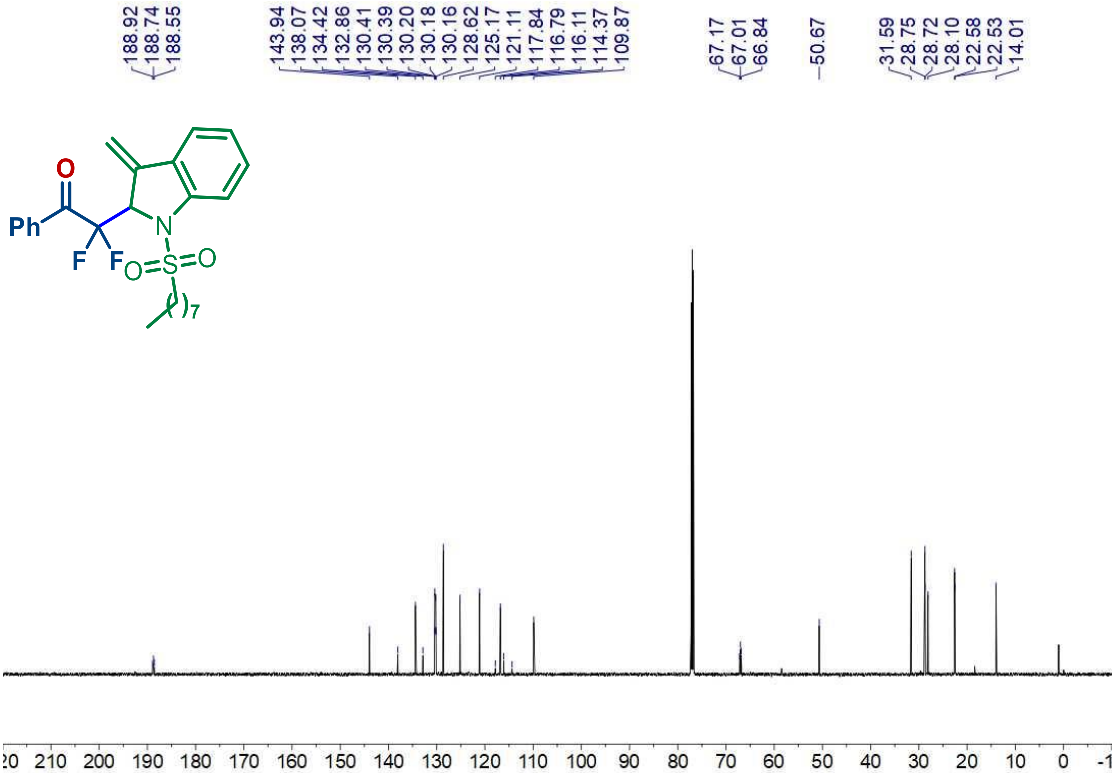

Supplementary Fig. 86 ^{13}C NMR (150 MHz, $CDCl_3$) spectrum of compound **28**

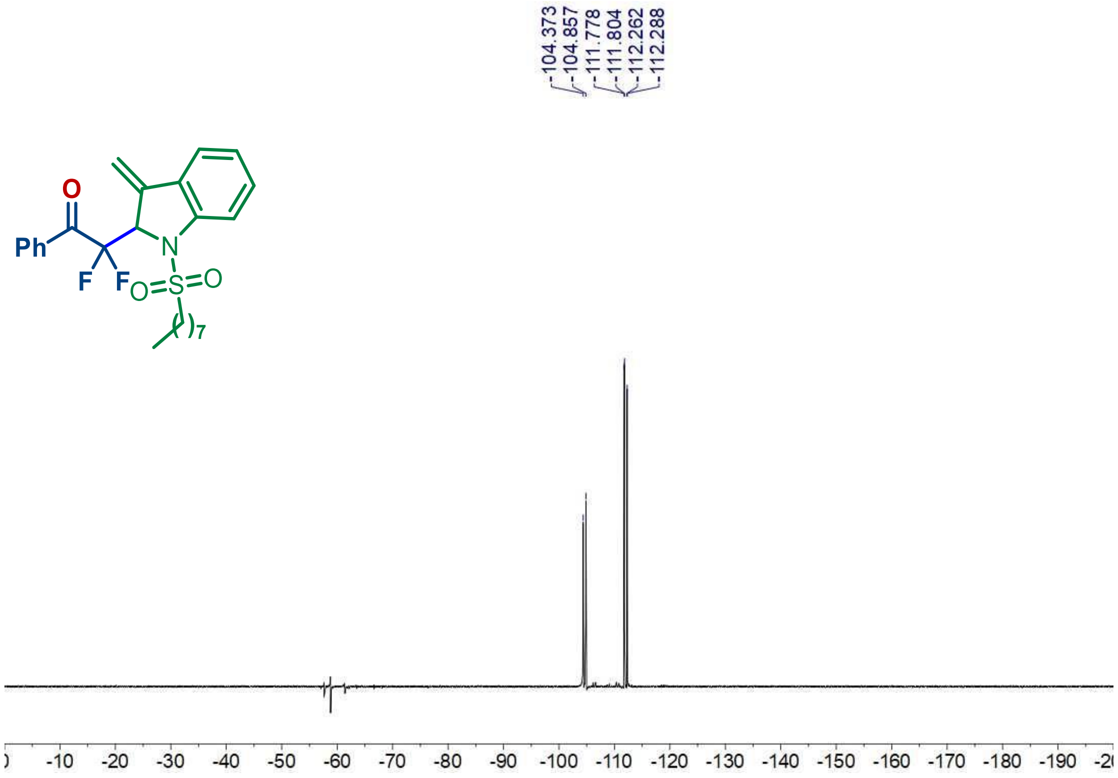

Supplementary Fig. 87 ^{19}F NMR (564 MHz, $CDCl_3$) spectrum of compound **28**

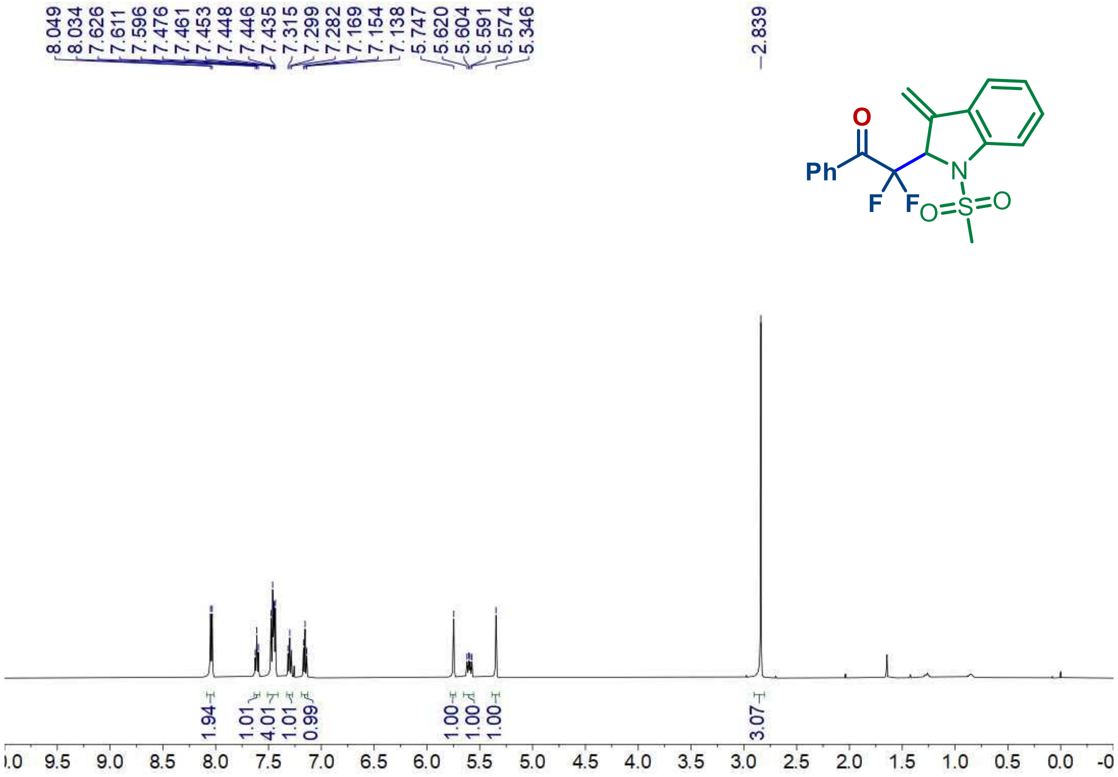

Supplementary Fig. 88 ^{1}H NMR (500 MHz, $CDCl_3$) spectrum of compound **29**

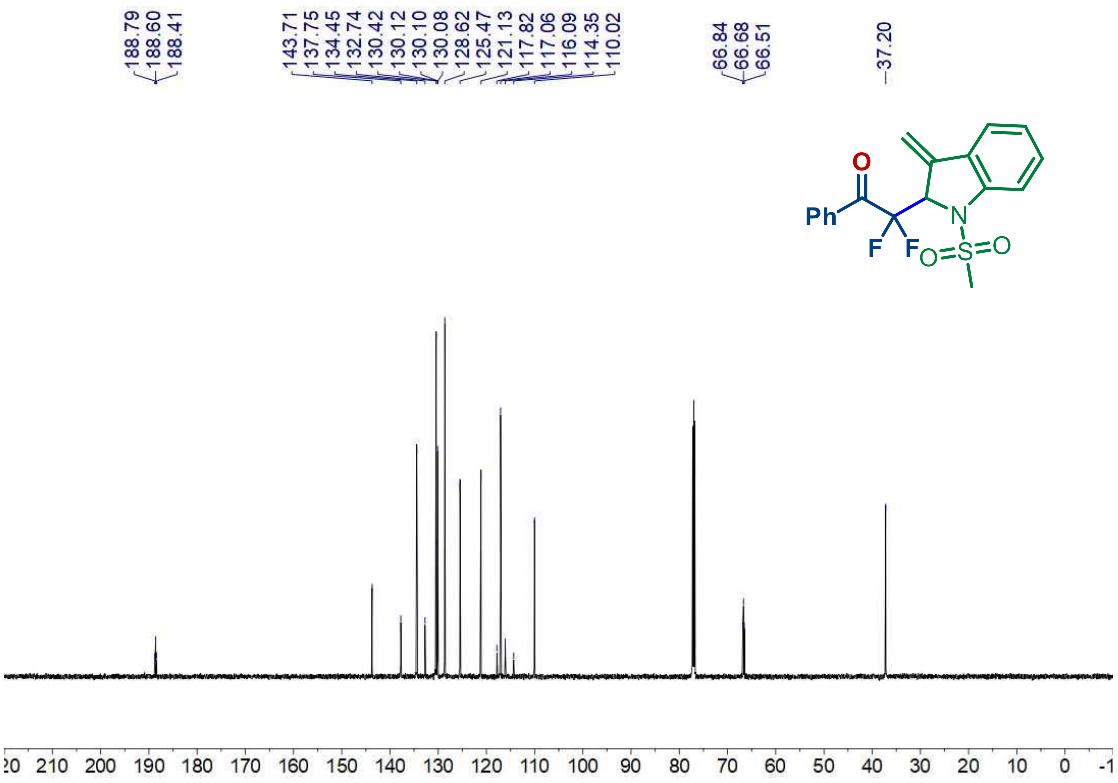

Supplementary Fig. 89 ^{13}C NMR (150 MHz, $CDCl_3$) spectrum of compound **29**

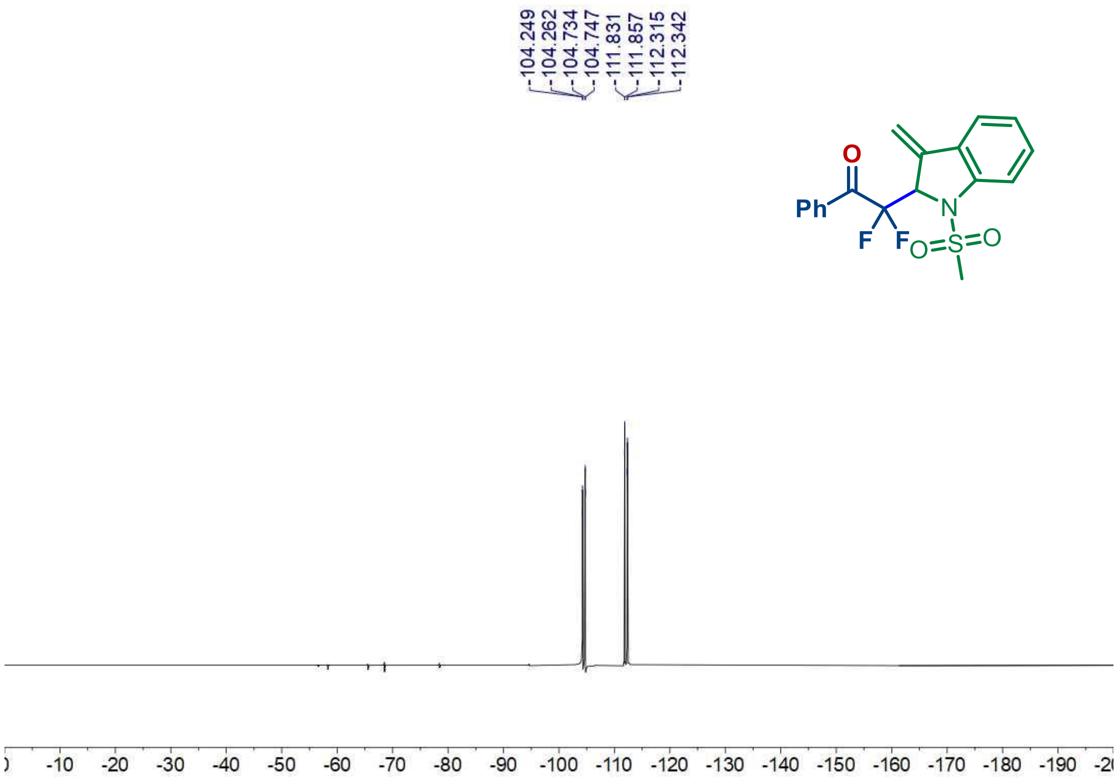

Supplementary Fig. 90 ^{19}F NMR (564 MHz, $CDCl_3$) spectrum of compound **29**

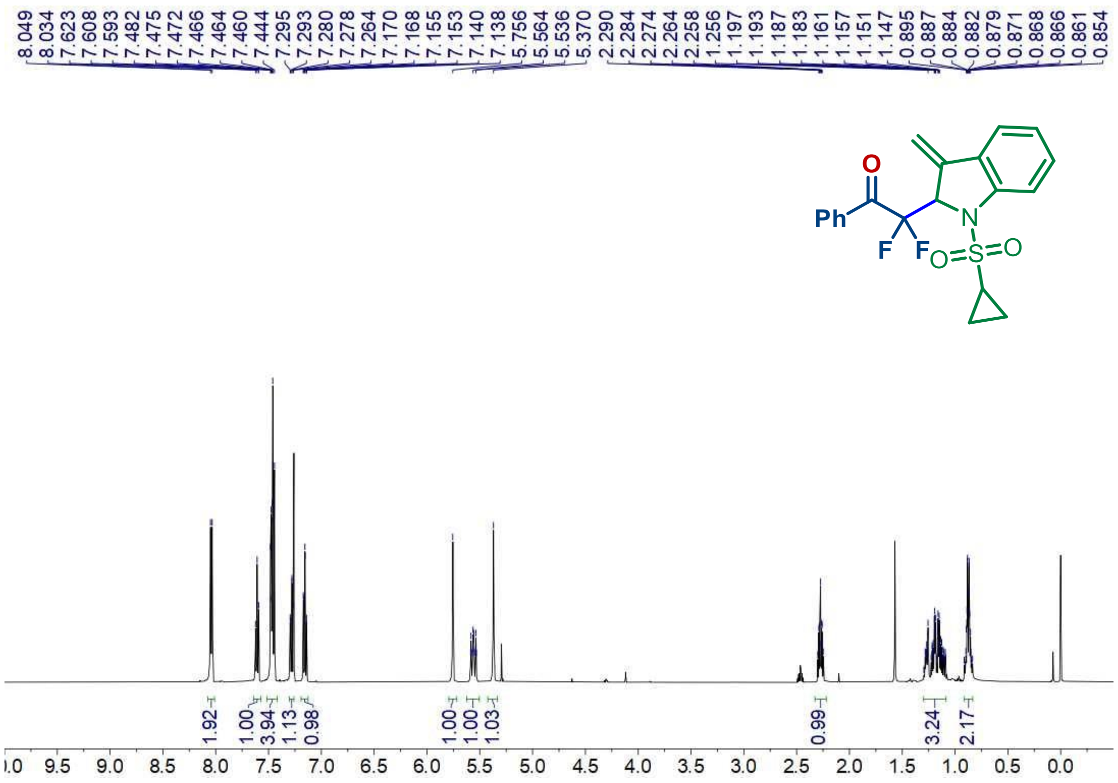

Supplementary Fig. 91 ^{1}H NMR (500 MHz, $CDCl_3$) spectrum of compound **30**

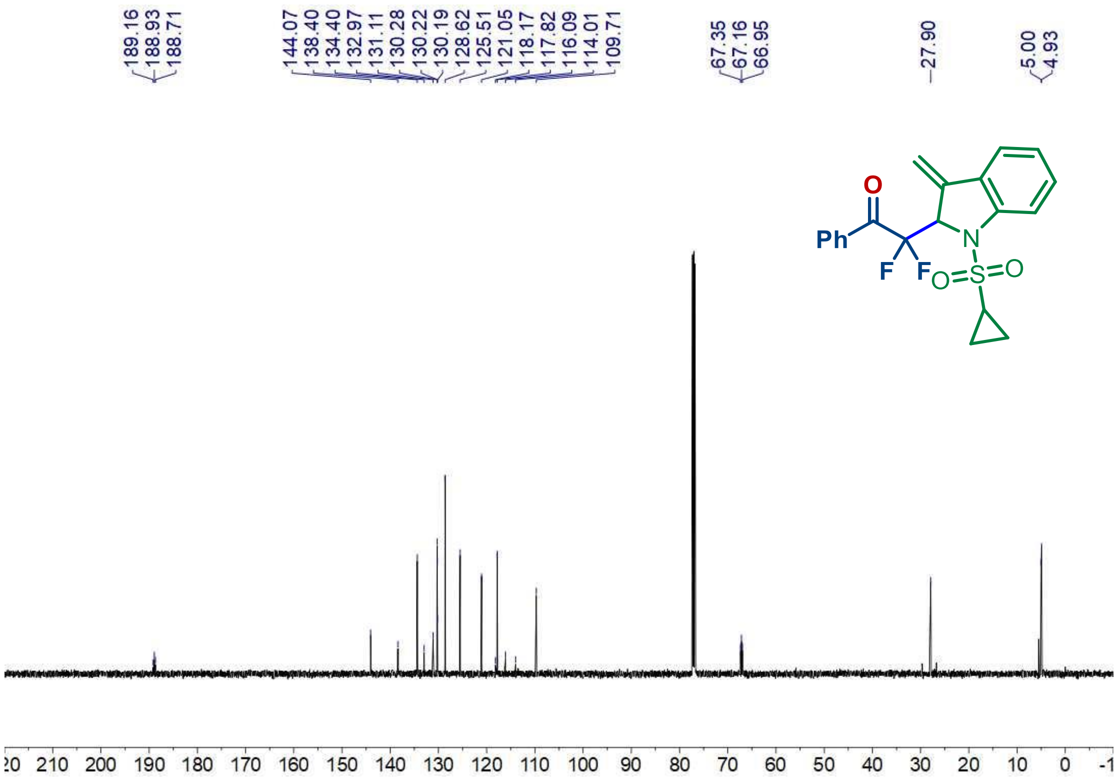

Supplementary Fig. 92 ^{13}C NMR (125 MHz, $CDCl_3$) spectrum of compound **30**

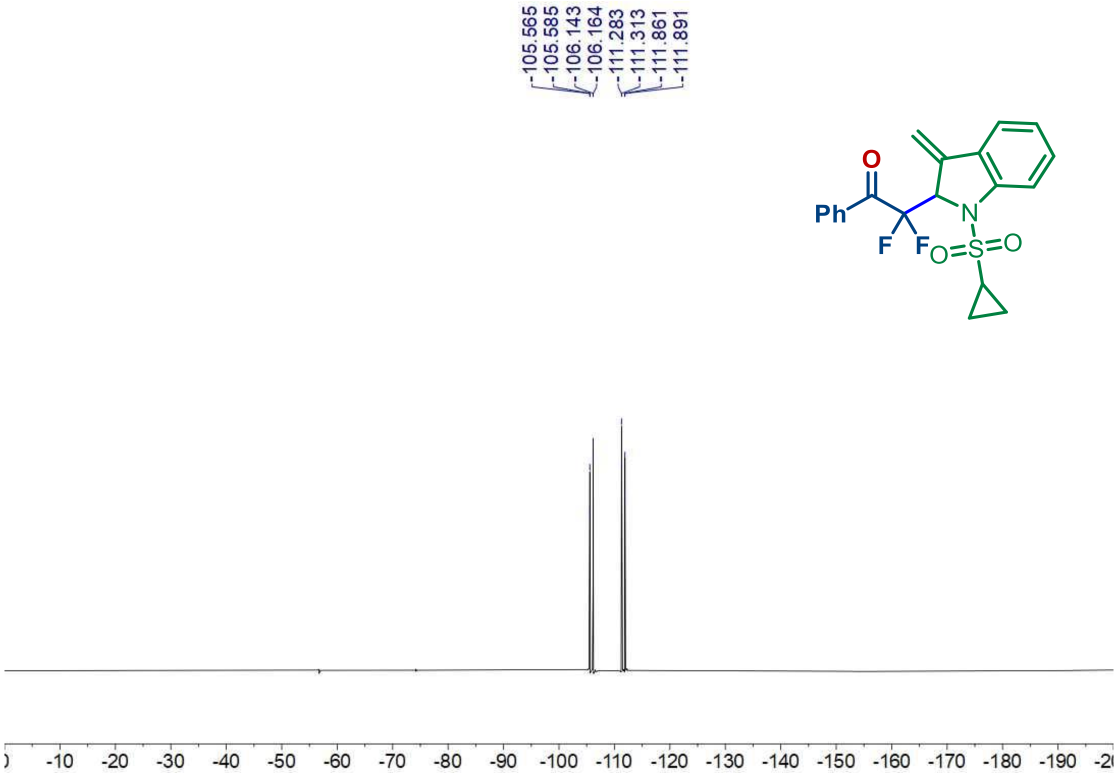

Supplementary Fig. 93 ^{19}F NMR (470 MHz, $CDCl_3$) spectrum of compound **30**

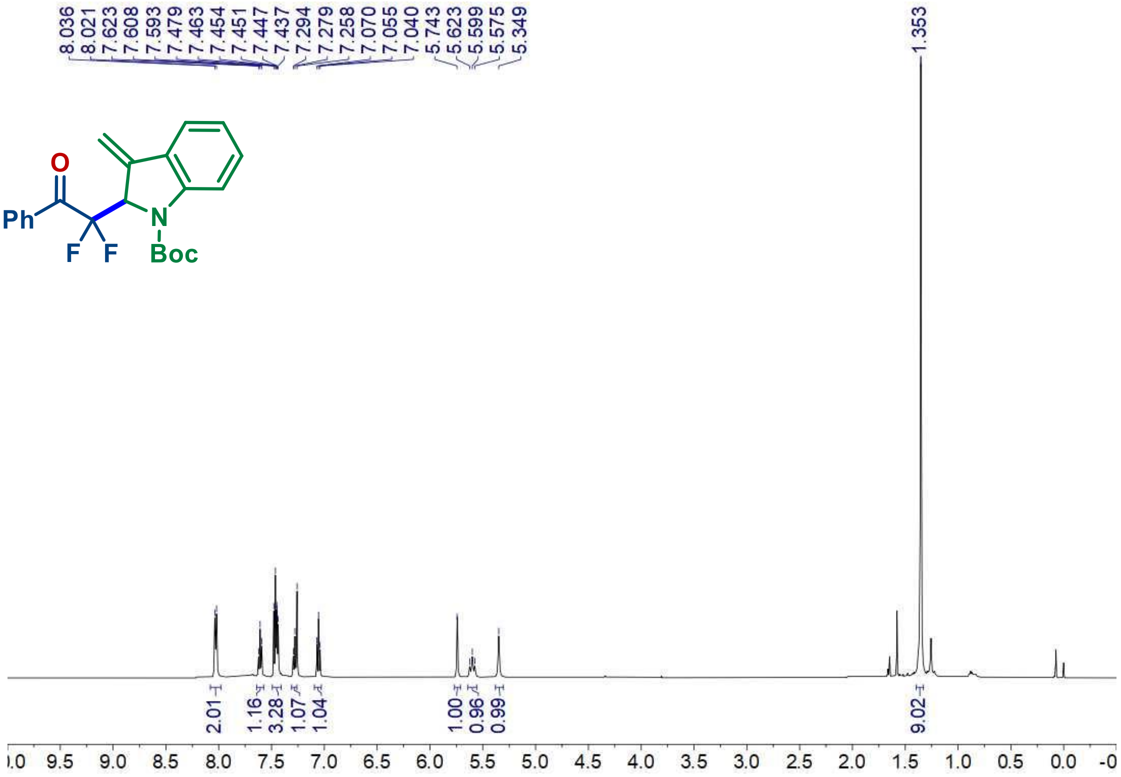

Supplementary Fig. 94 ^{1}H NMR (500 MHz, $CDCl_3$) spectrum of compound **31**

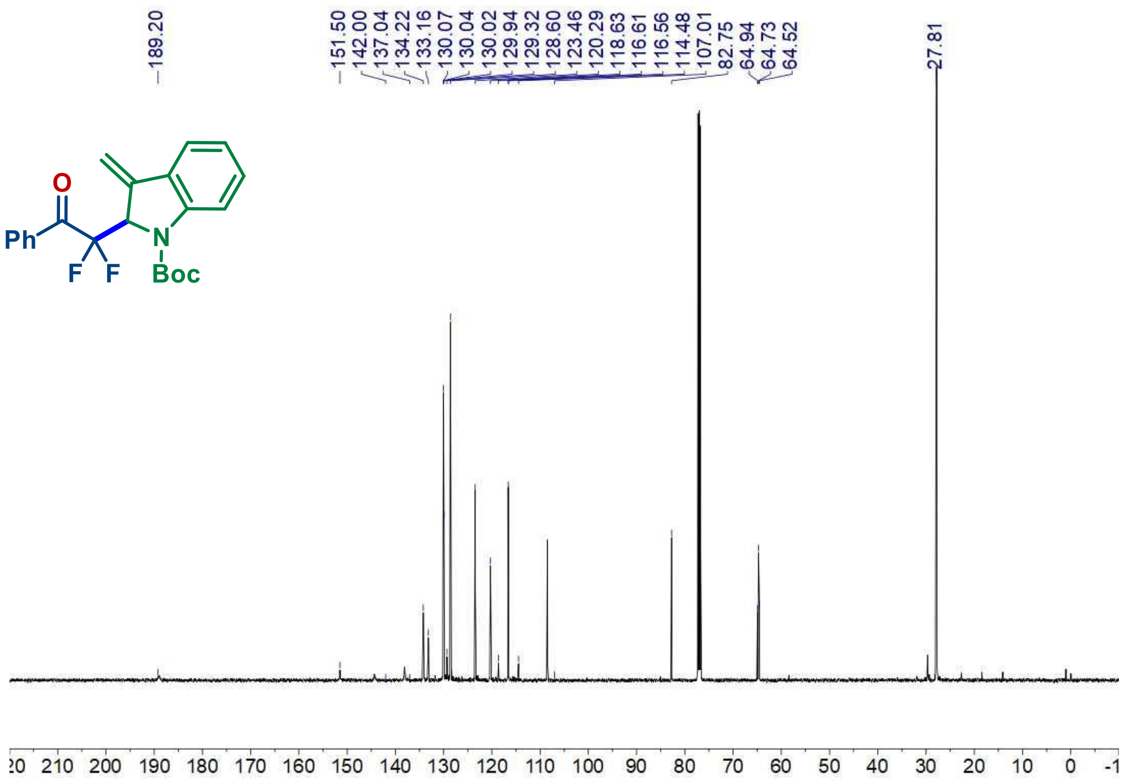

Supplementary Fig. 95 ^{13}C NMR (125 MHz, $CDCl_3$) spectrum of compound **31**

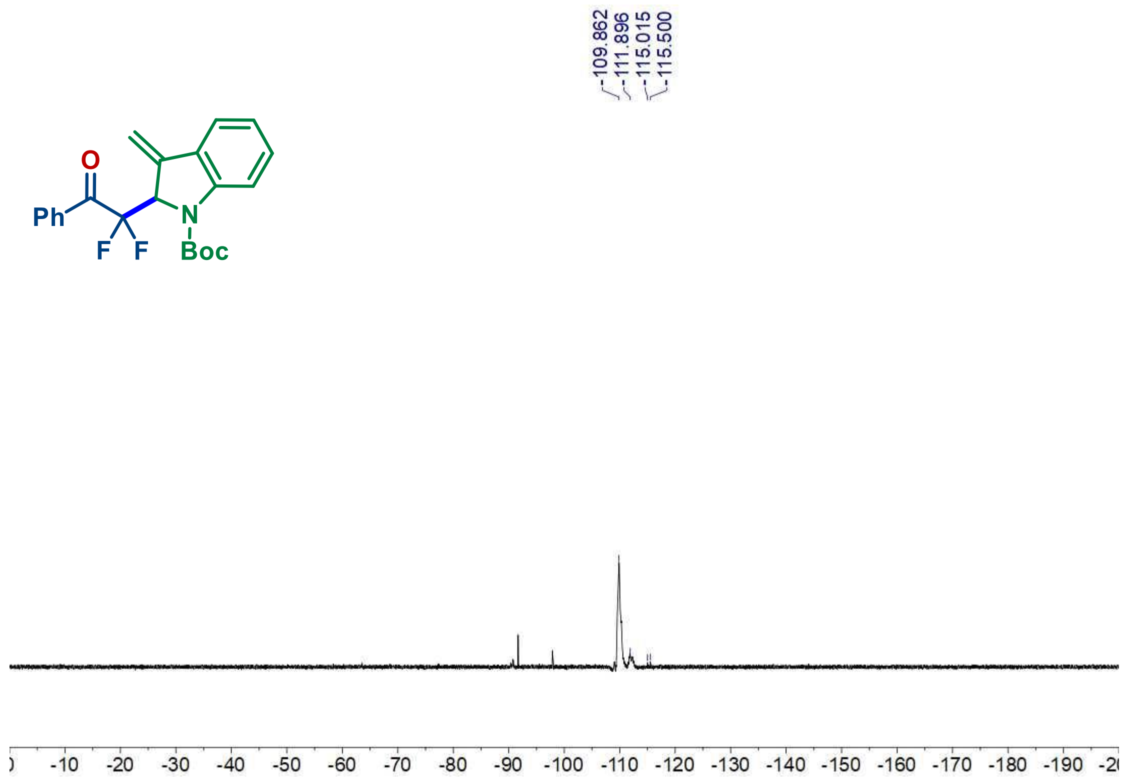

Supplementary Fig. 96 ^{19}F NMR (564 MHz, $CDCl_3$) spectrum of compound **31**

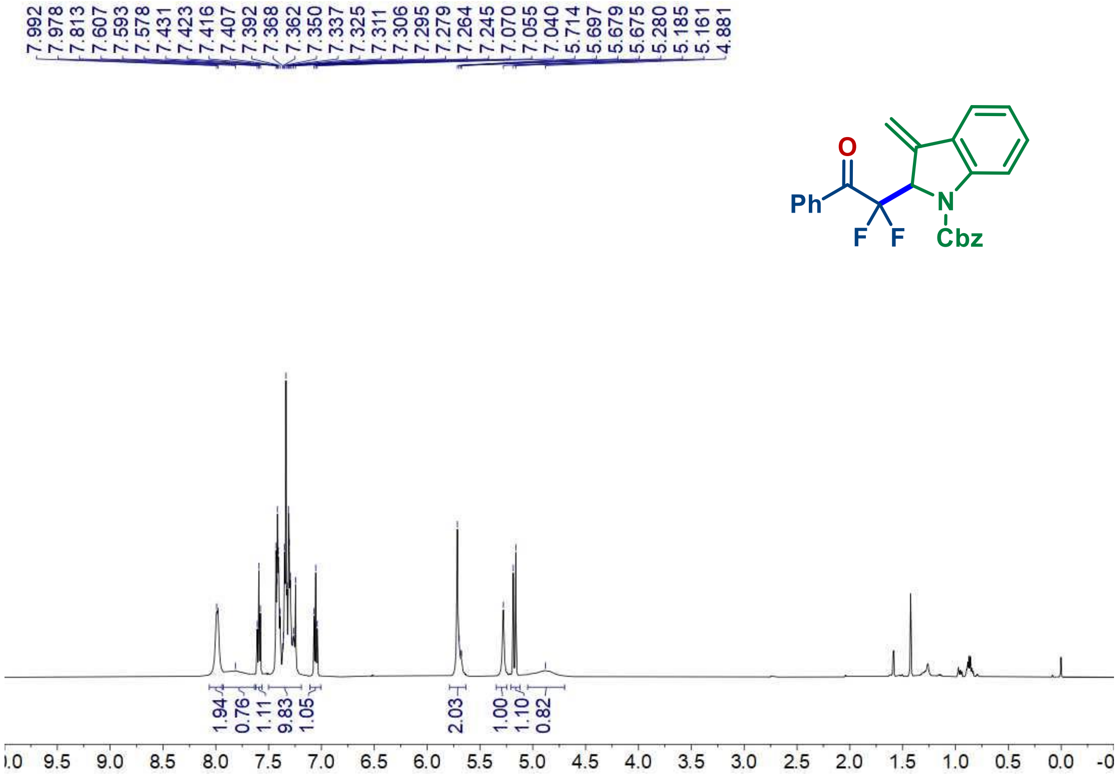

Supplementary Fig. 97 ^{1}H NMR (500 MHz, $CDCl_3$) spectrum of compound **32**

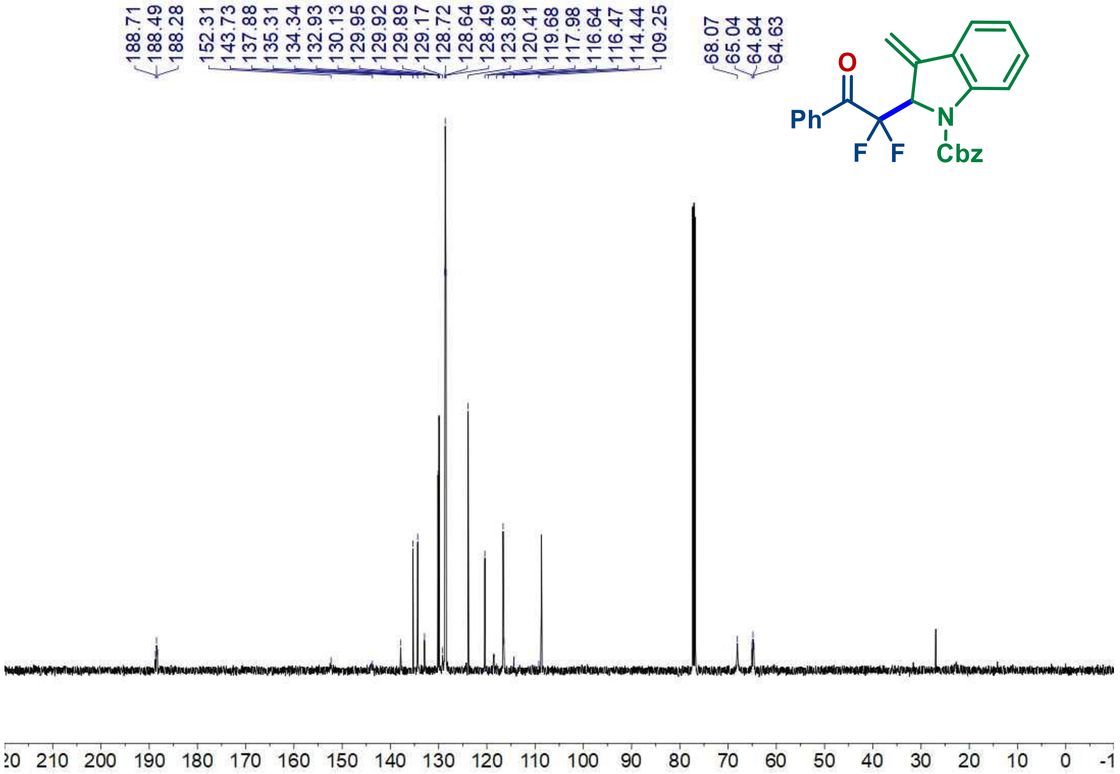

Supplementary Fig. 98 ^{13}C NMR (125 MHz, $CDCl_3$) spectrum of compound **32**

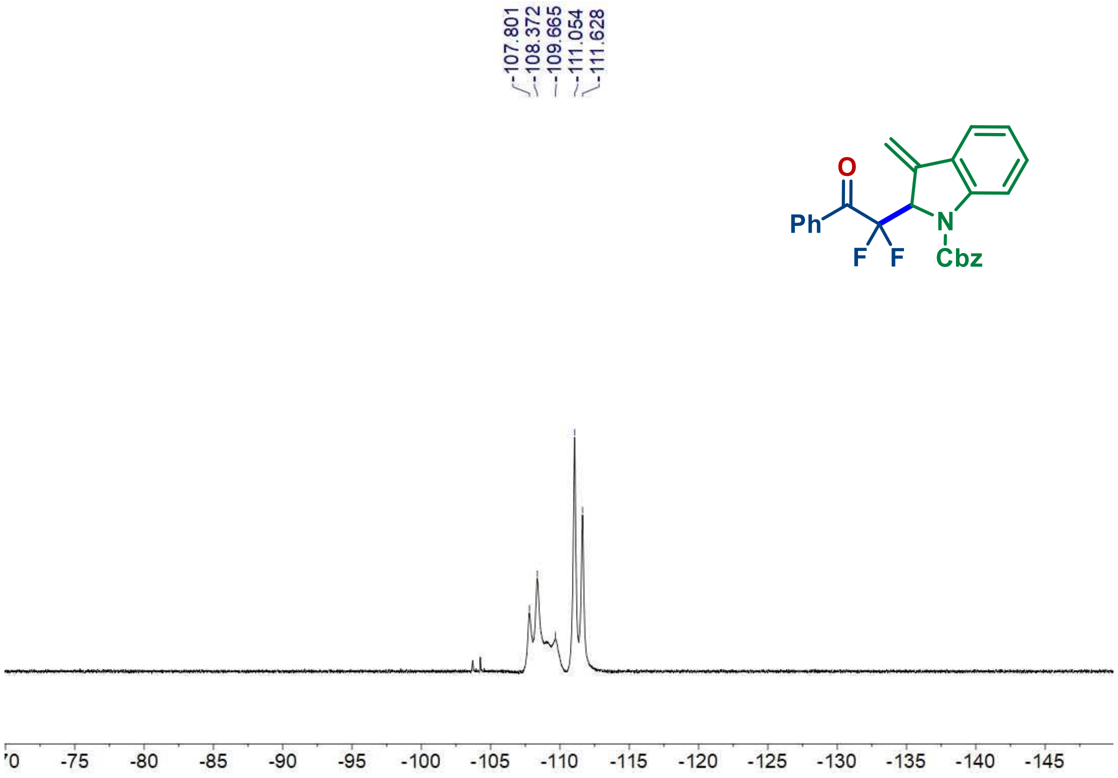

Supplementary Fig. 99 ^{19}F NMR (470 MHz, $CDCl_3$) spectrum of compound **32**

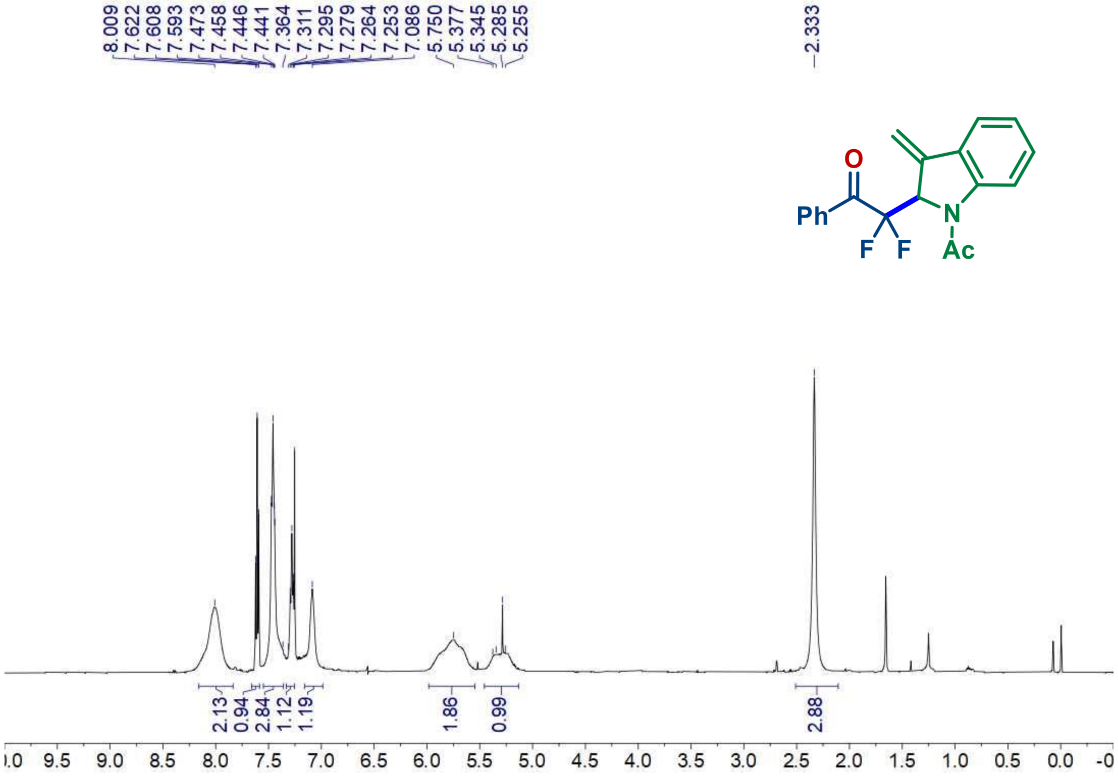

Supplementary Fig. 100 ^{1}H NMR (500 MHz, $CDCl_3$) spectrum of compound **33**

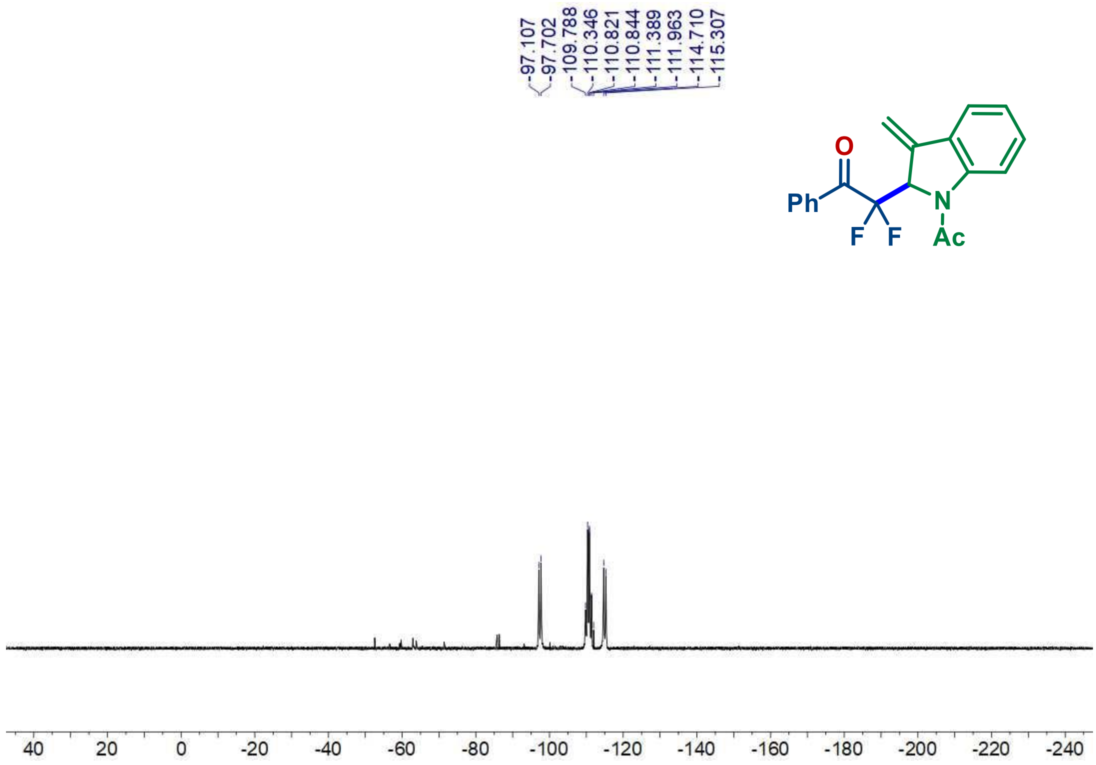

Supplementary Fig. 101 ^{19}F NMR (470 MHz, $CDCl_3$) spectrum of compound **33**

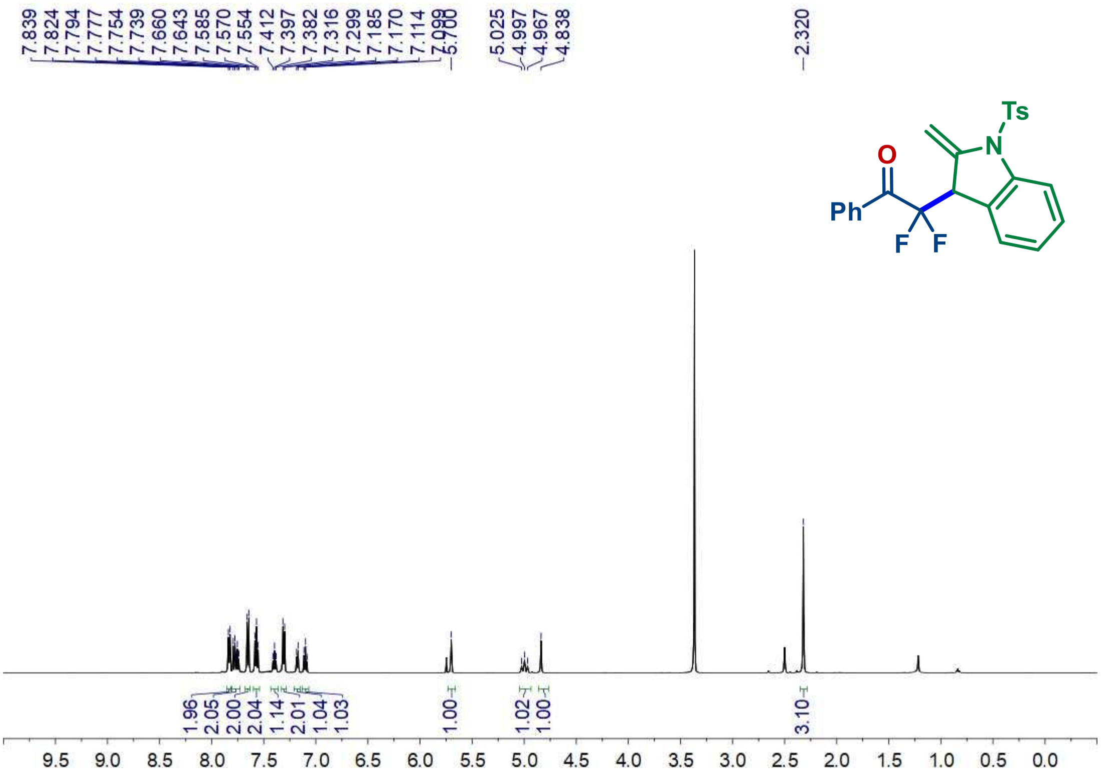

Supplementary Fig. 102 ^{1}H NMR (500 MHz, DMSO) spectrum of compound **34**

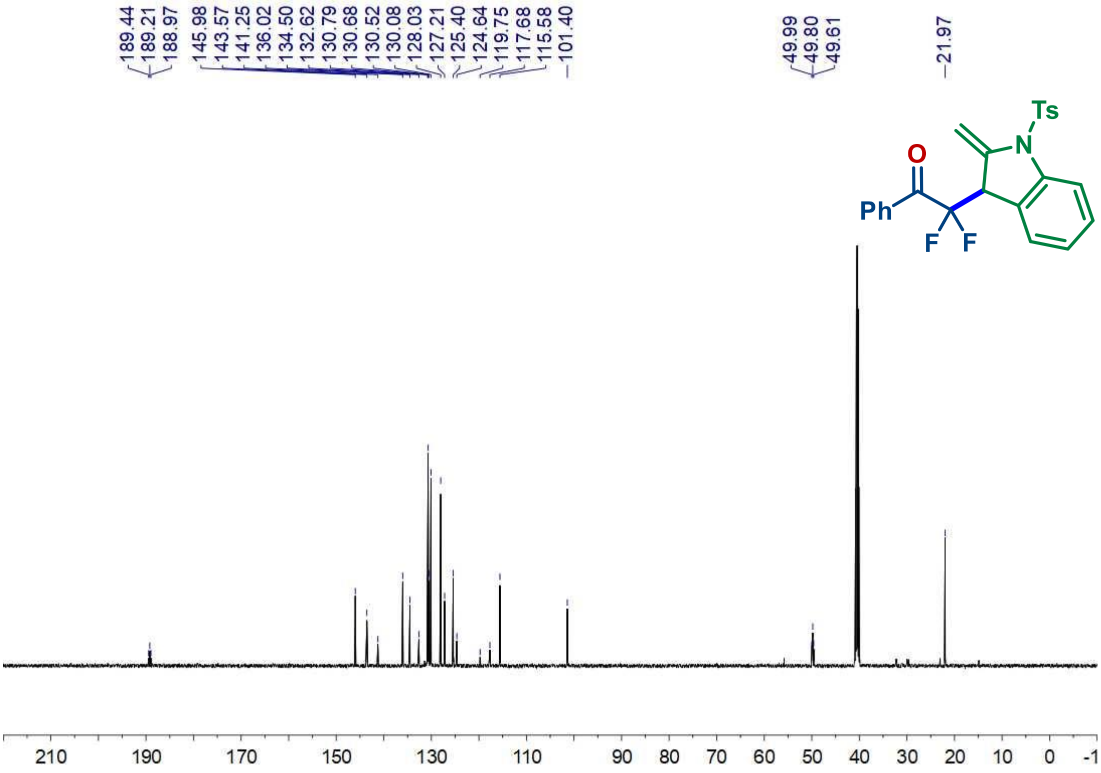

Supplementary Fig. 103 ^{13}C NMR (125 MHz, DMSO) spectrum of compound **34**

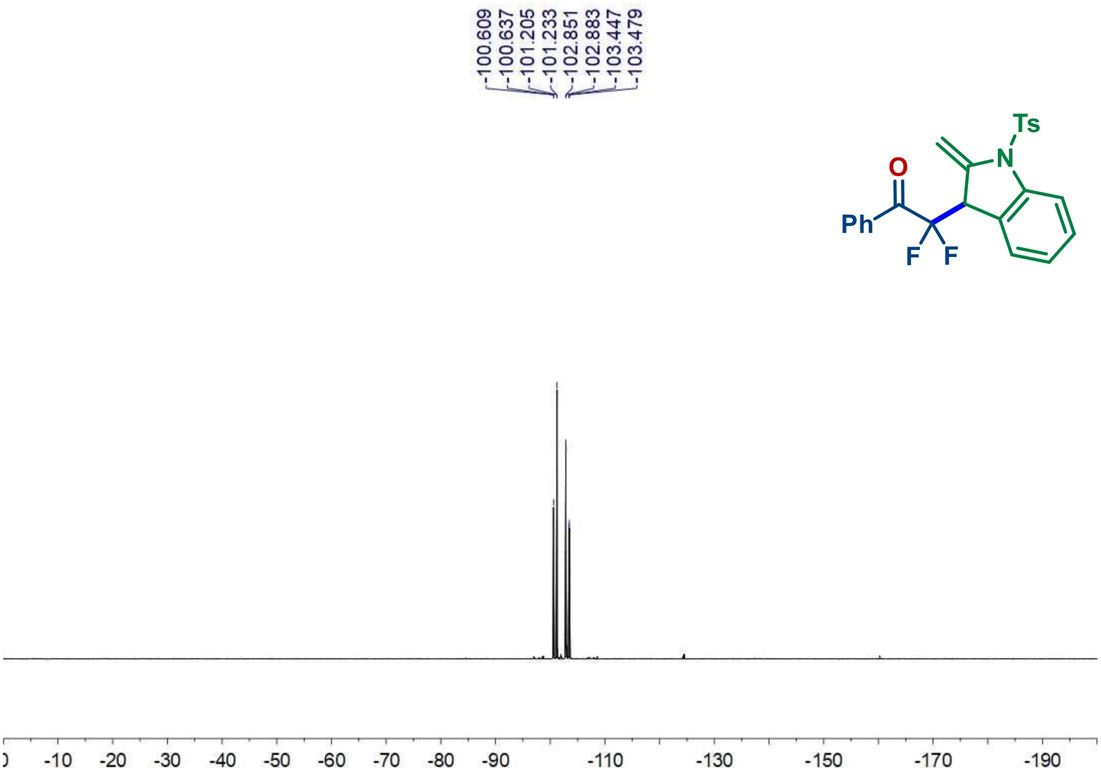

Supplementary Fig. 104 ^{19}F NMR (470 MHz, DMSO) spectrum of compound **34**

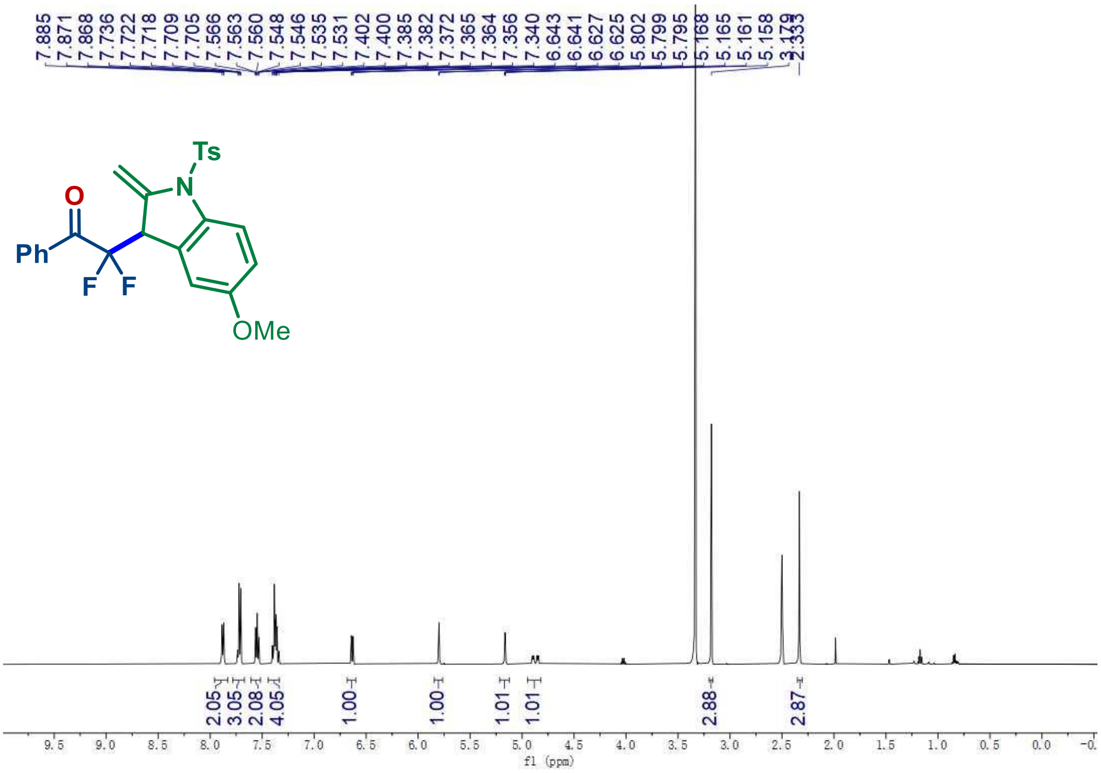

Supplementary Fig. 105 ^{1}H NMR (500 MHz, CD_3CN) spectrum of compound **35**

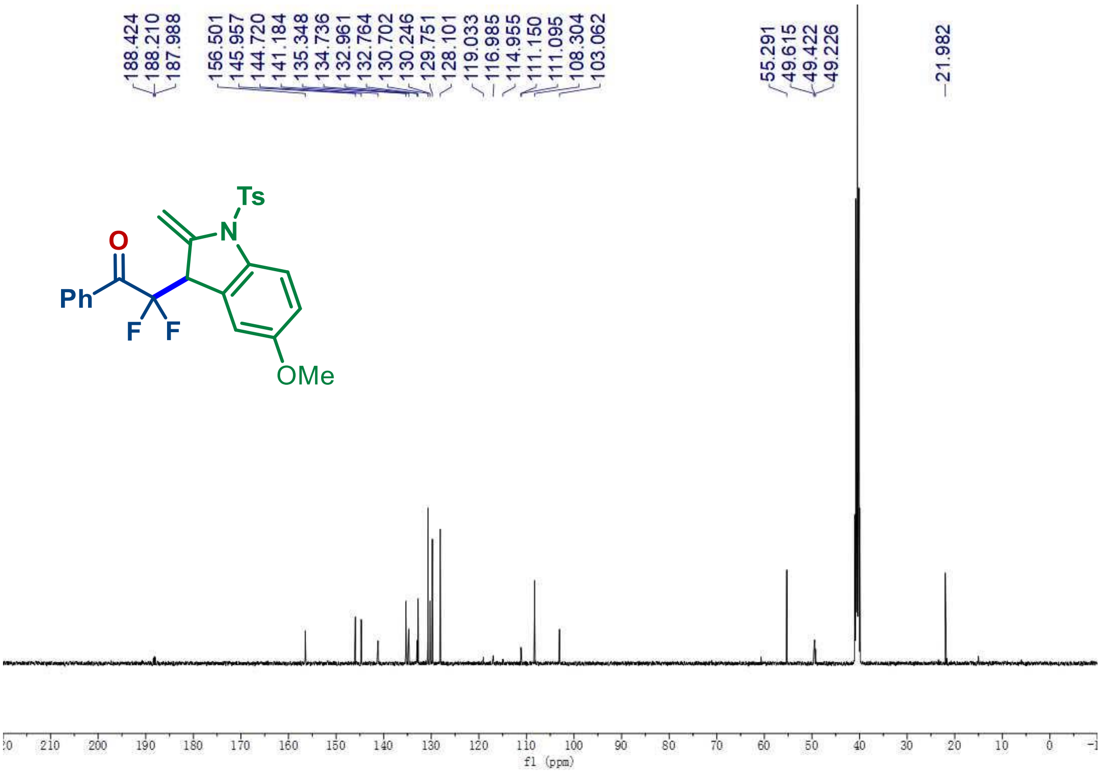

Supplementary Fig. 106 ^{13}C NMR (150 MHz, CD_3CN) spectrum of compound **35**

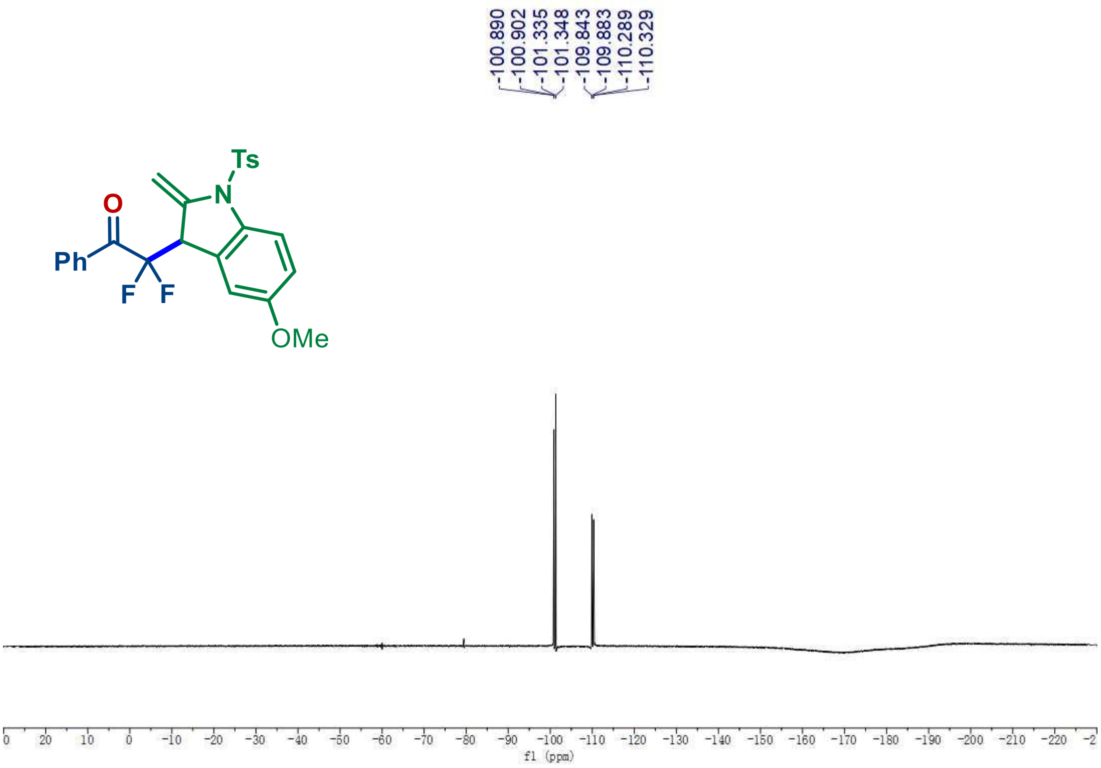

Supplementary Fig. 107 ^{19}F NMR (564 MHz, CD_3CN) spectrum of compound **35**

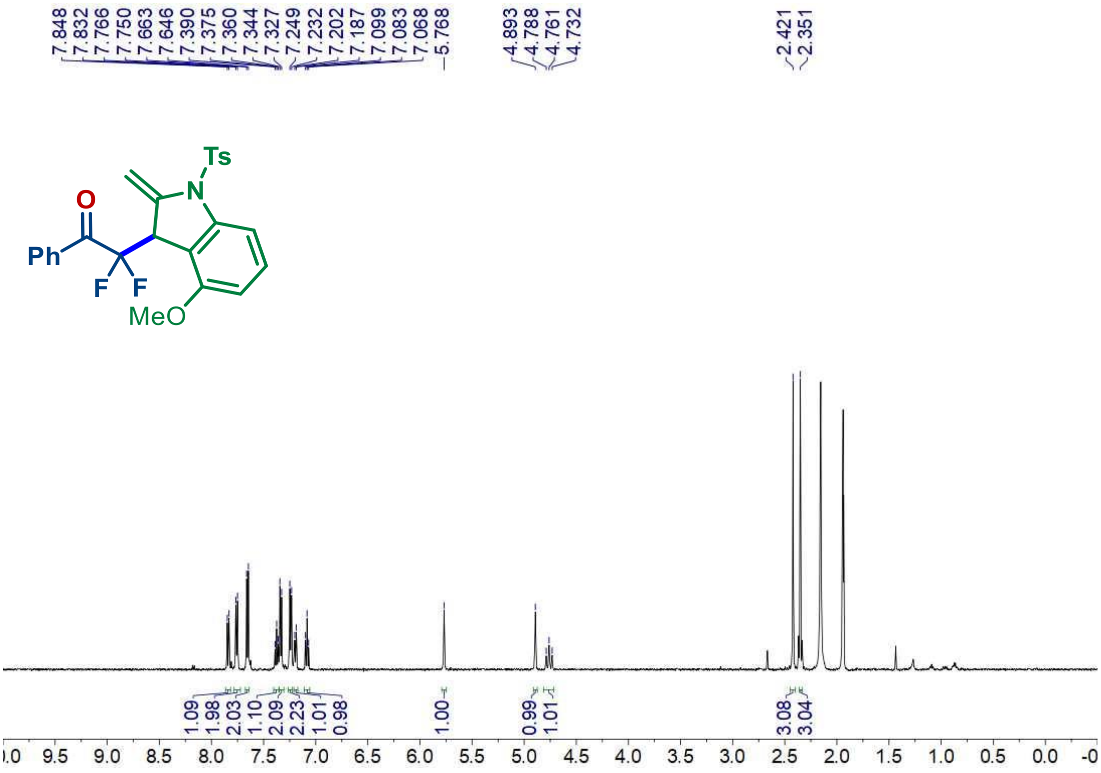

Supplementary Fig. 108 ^{1}H NMR (500 MHz, DMSO) spectrum of compound **36**

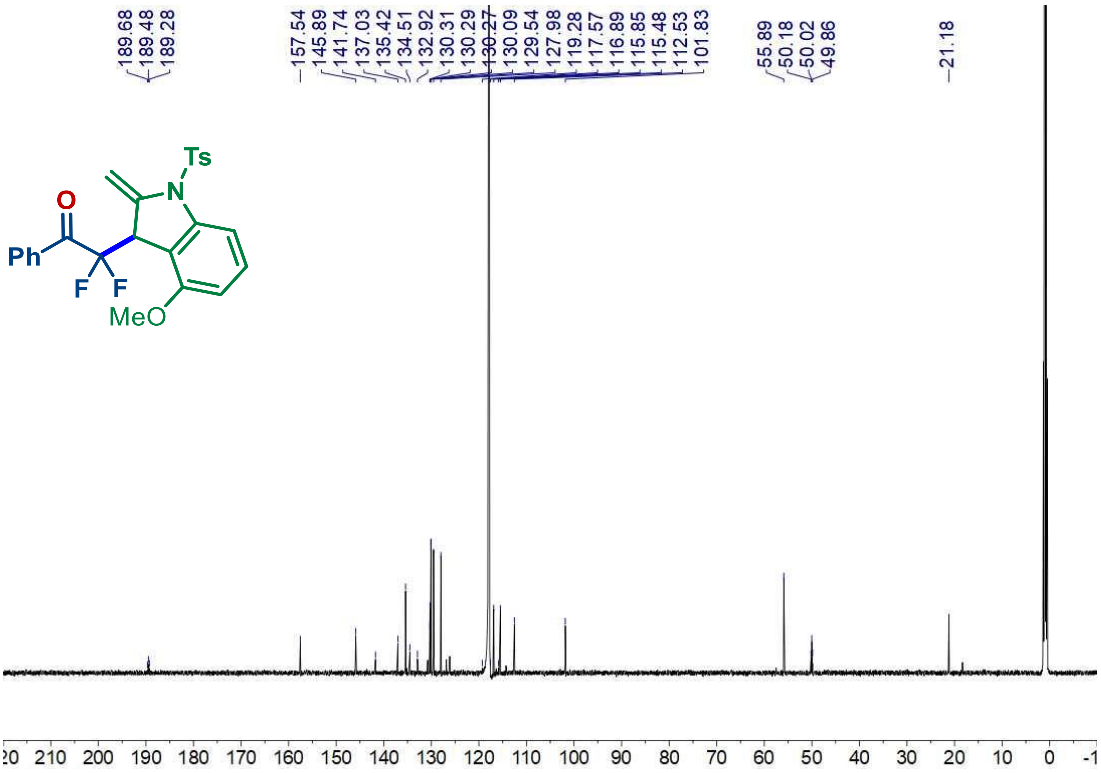

Supplementary Fig. 109 ^{13}C NMR (125 MHz, DMSO) spectrum of compound **36**

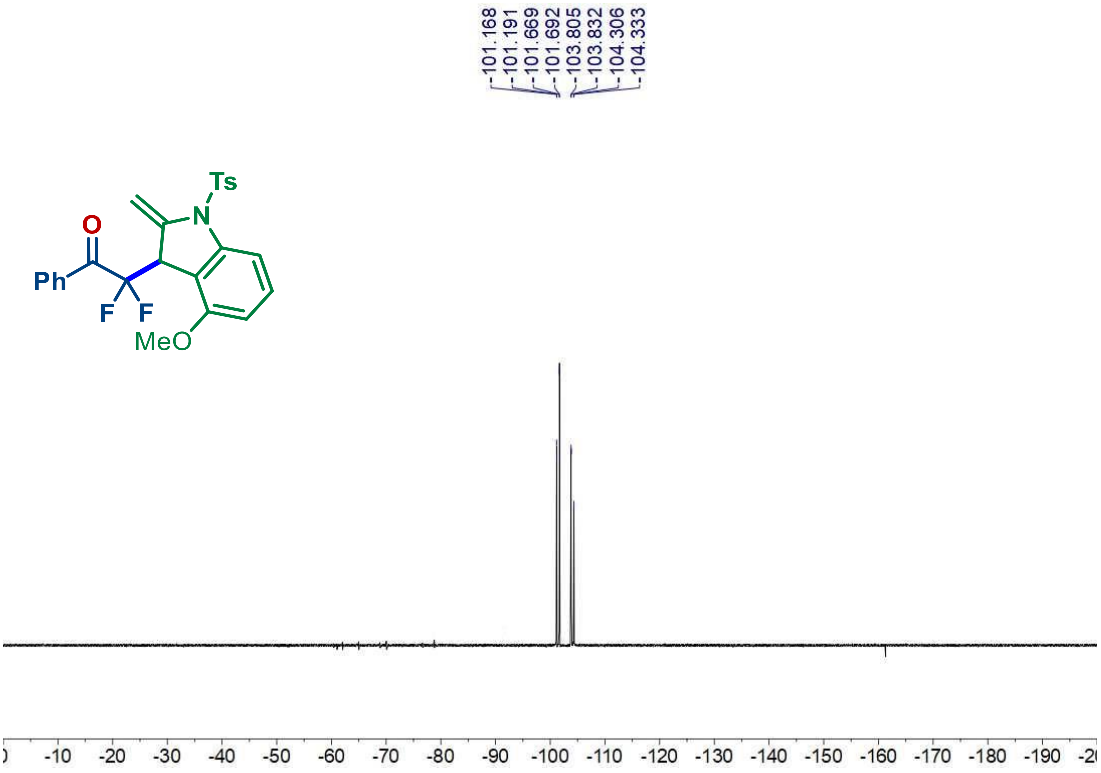

Supplementary Fig. 110 ^{19}F NMR (564 MHz, DMSO) spectrum of compound **36**

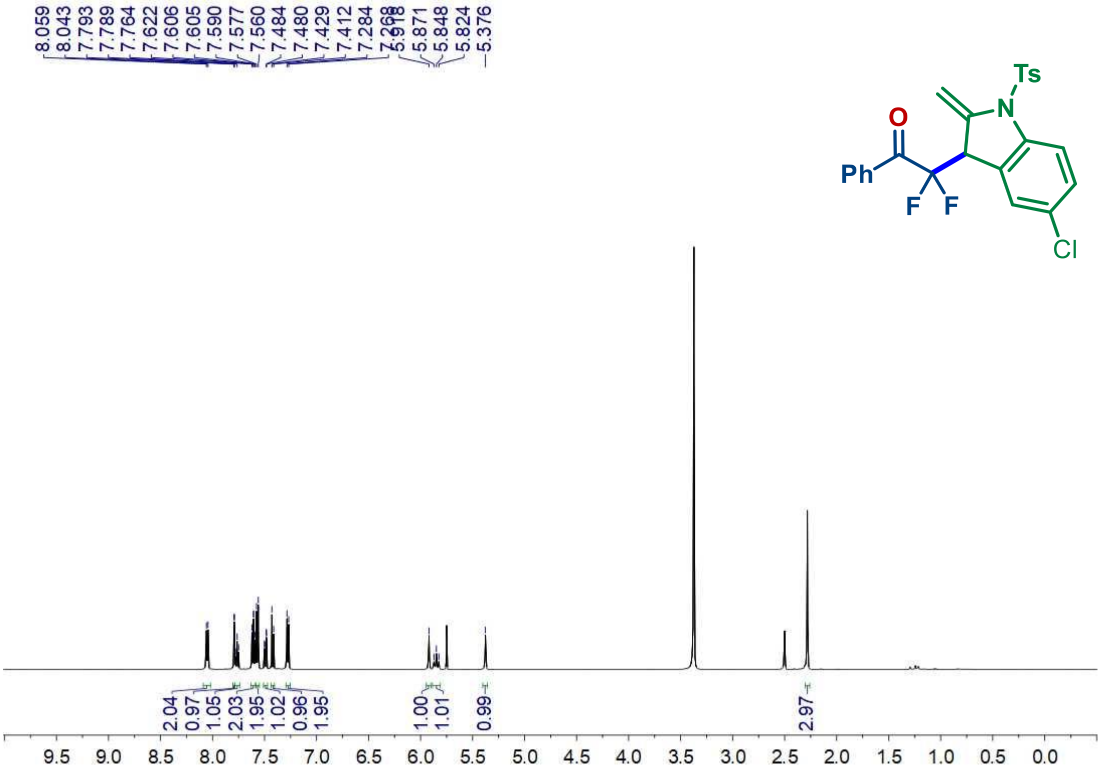

Supplementary Fig. 111 ^{1}H NMR (500 MHz, DMSO) spectrum of compound **37**

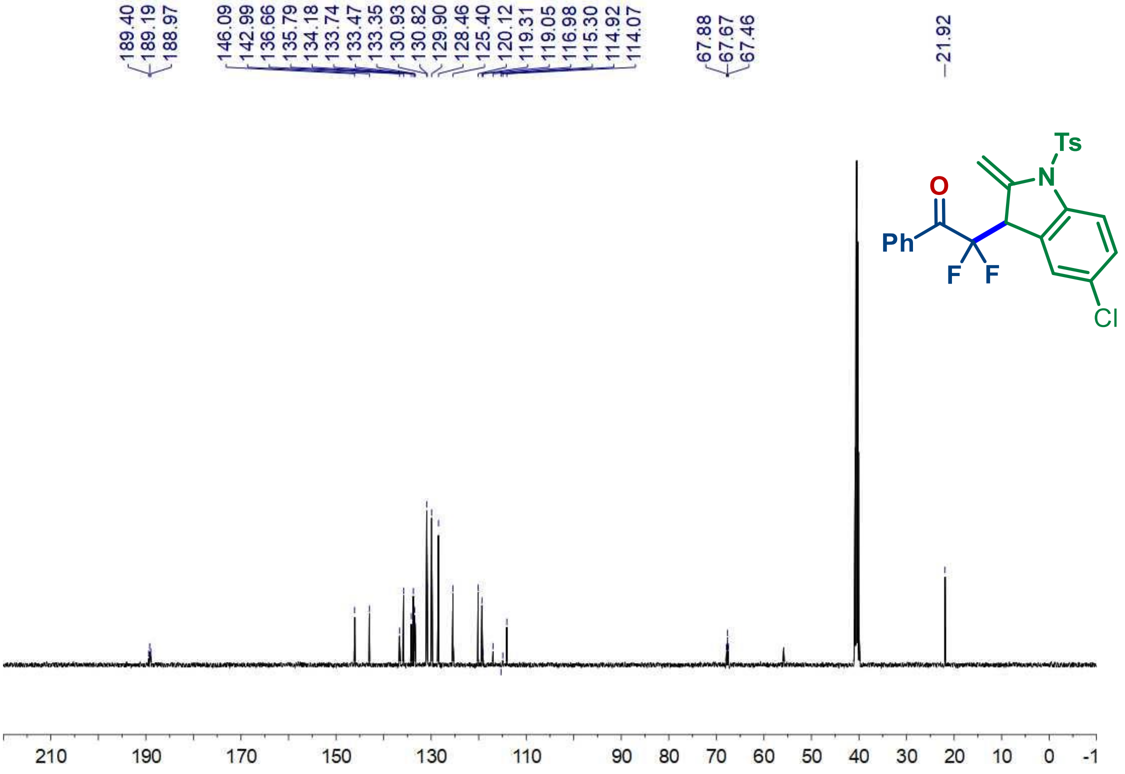

Supplementary Fig. 112 ^{13}C NMR (125 MHz, DMSO) spectrum of compound **37**

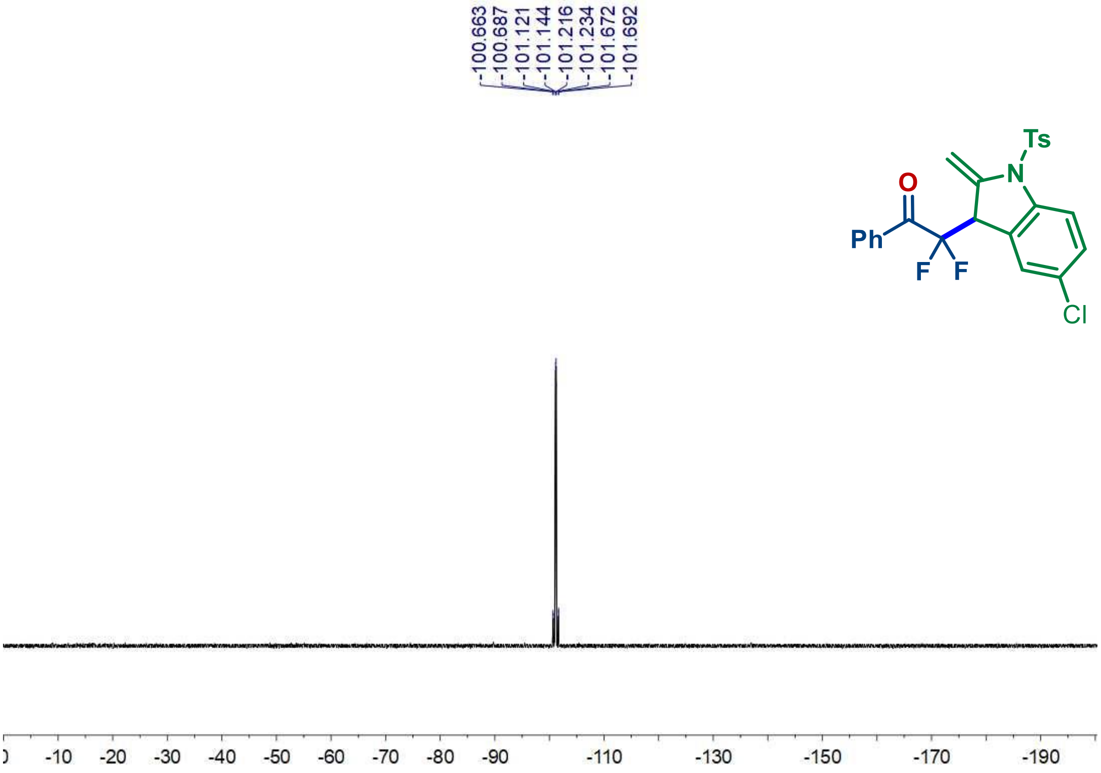

Supplementary Fig. 113 ^{19}F NMR (564 MHz, DMSO) spectrum of compound **37**

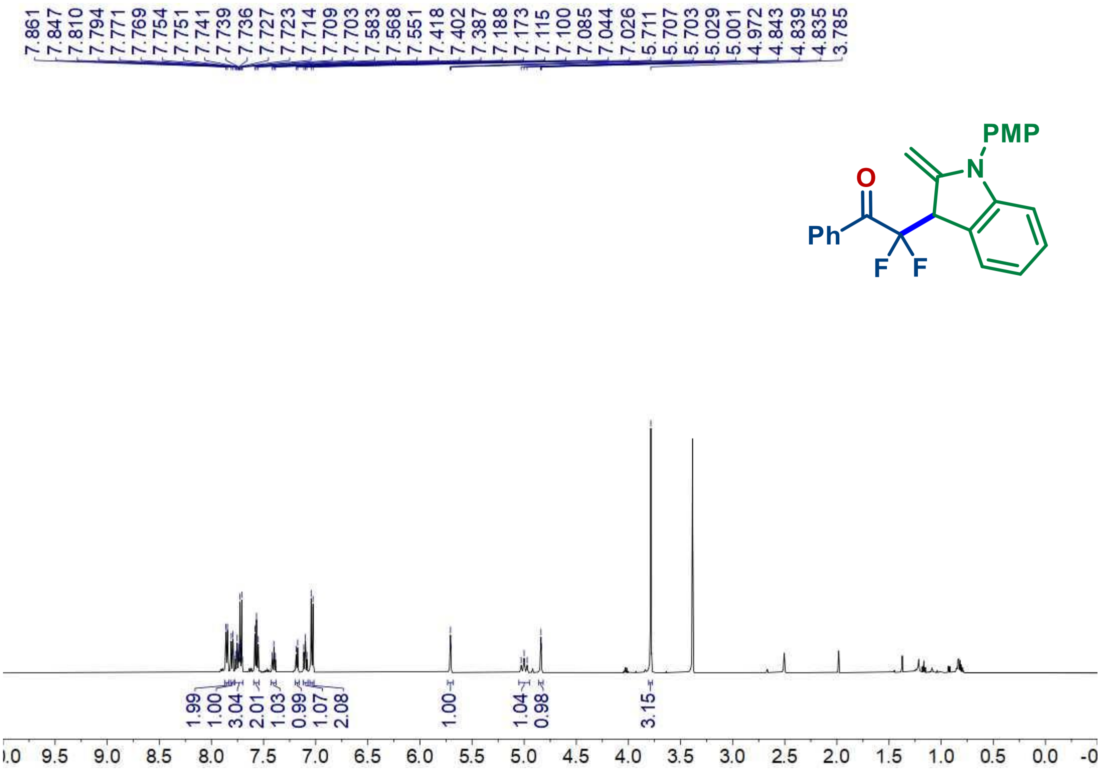

Supplementary Fig. 114 ^{1}H NMR (500 MHz, DMSO) spectrum of compound **38**

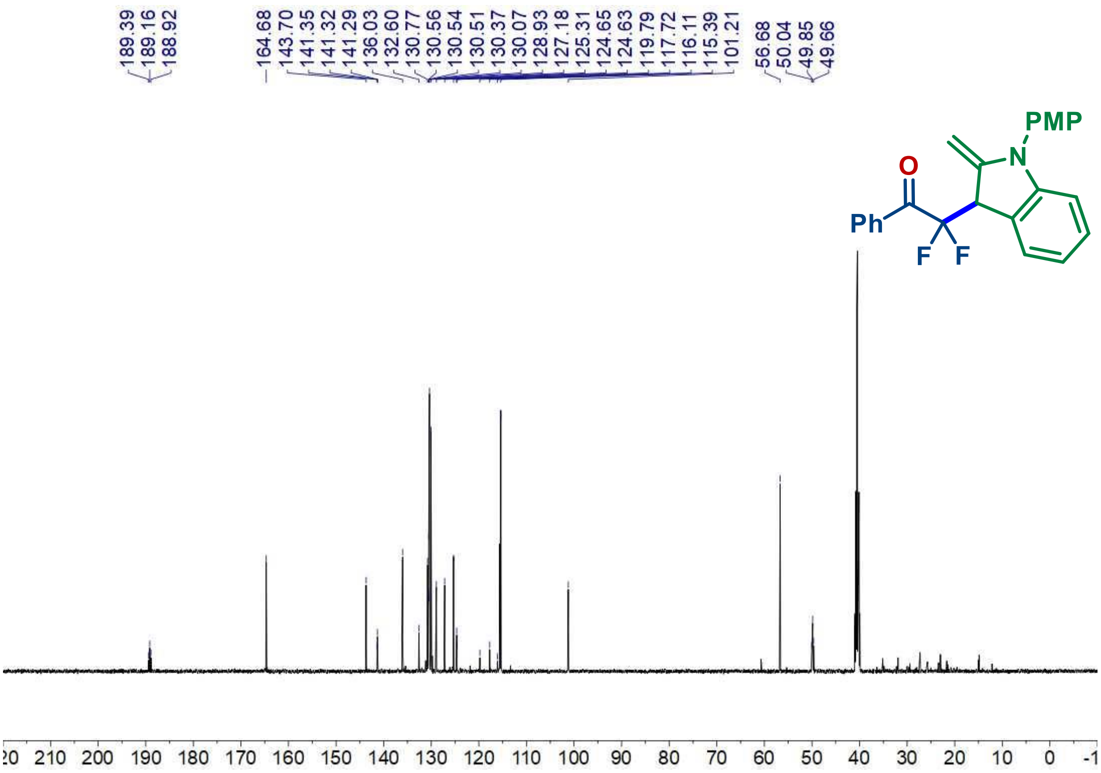

Supplementary Fig. 115 ^{13}C NMR (125 MHz, DMSO) spectrum of compound **38**

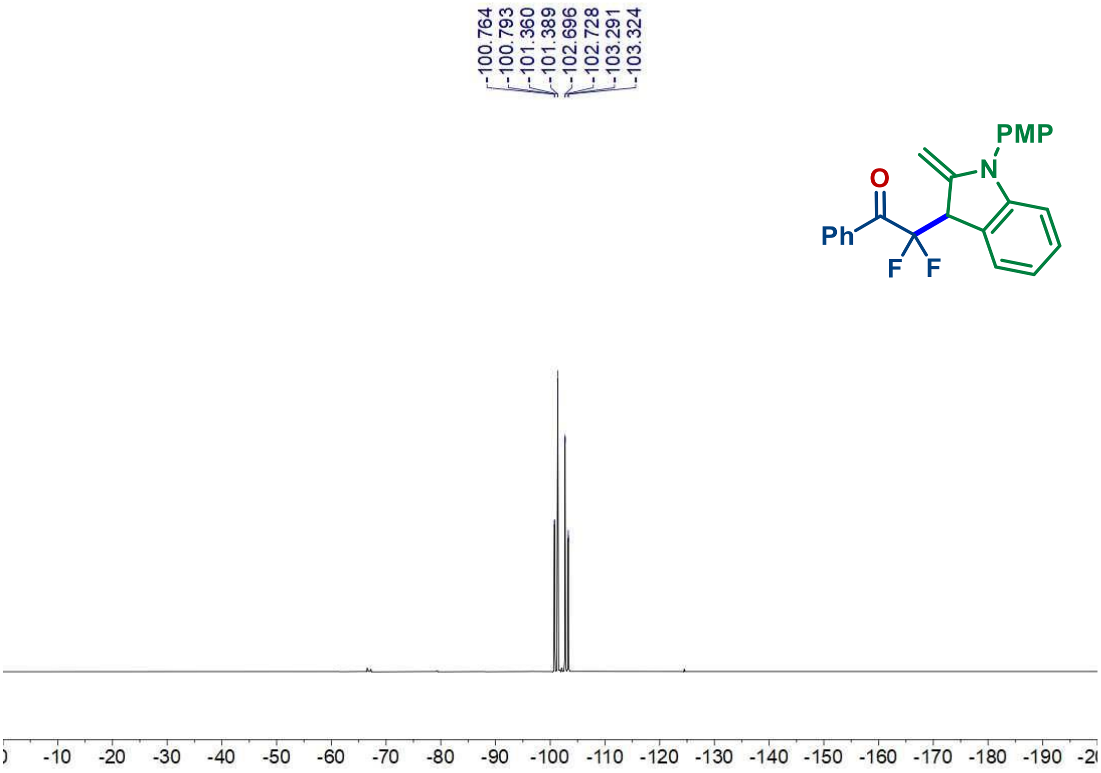

Supplementary Fig. 116 ^{19}F NMR (470 MHz, DMSO) spectrum of compound **38**

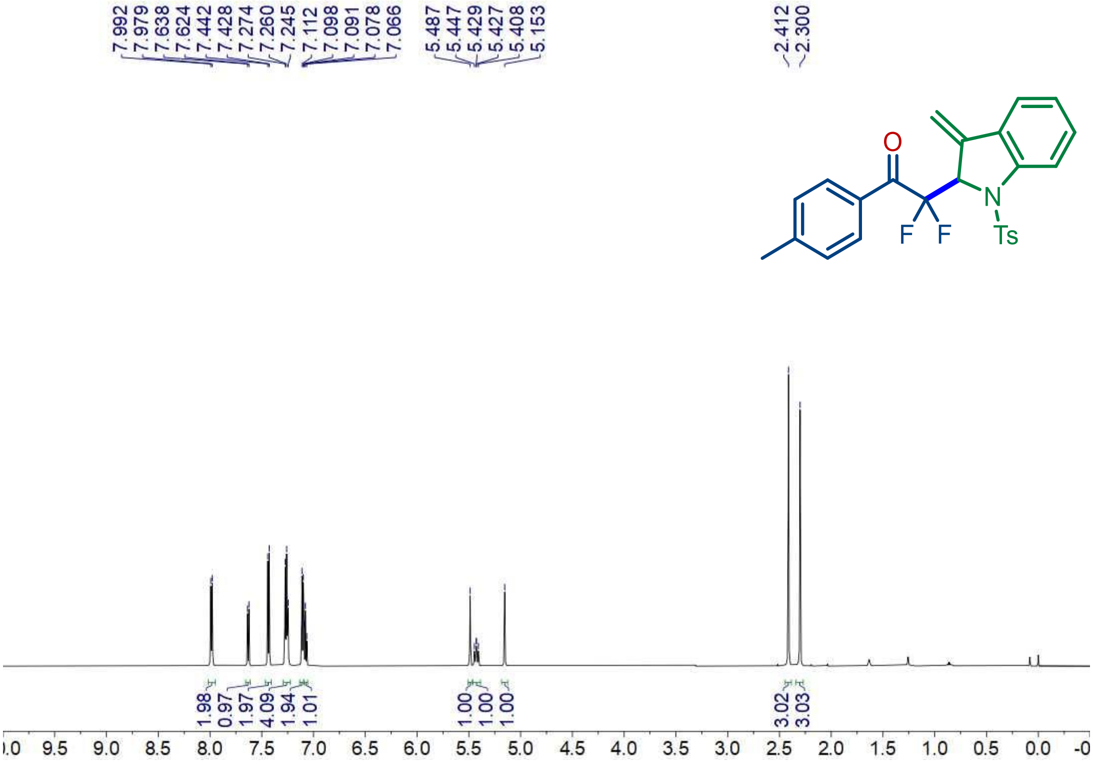

Supplementary Fig. 117 ^{1}H NMR (600 MHz, $CDCl_3$) spectrum of compound **39**

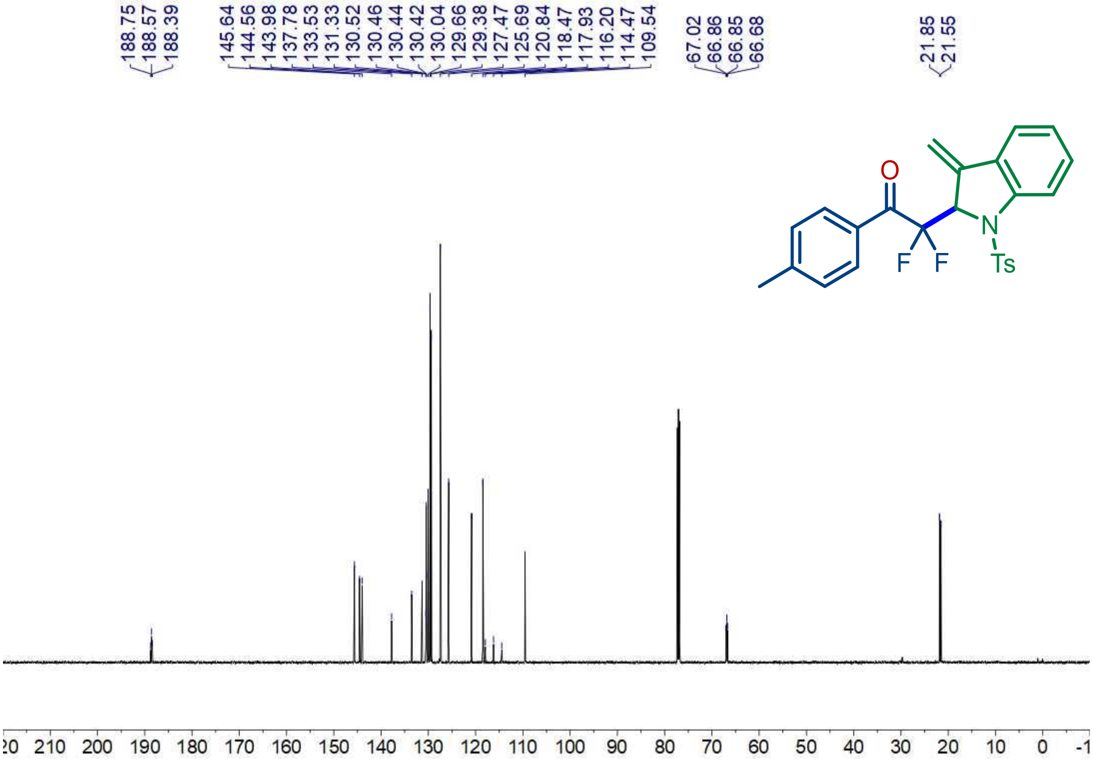

Supplementary Fig. 118 ^{13}C NMR (150 MHz, $CDCl_3$) spectrum of compound **39**

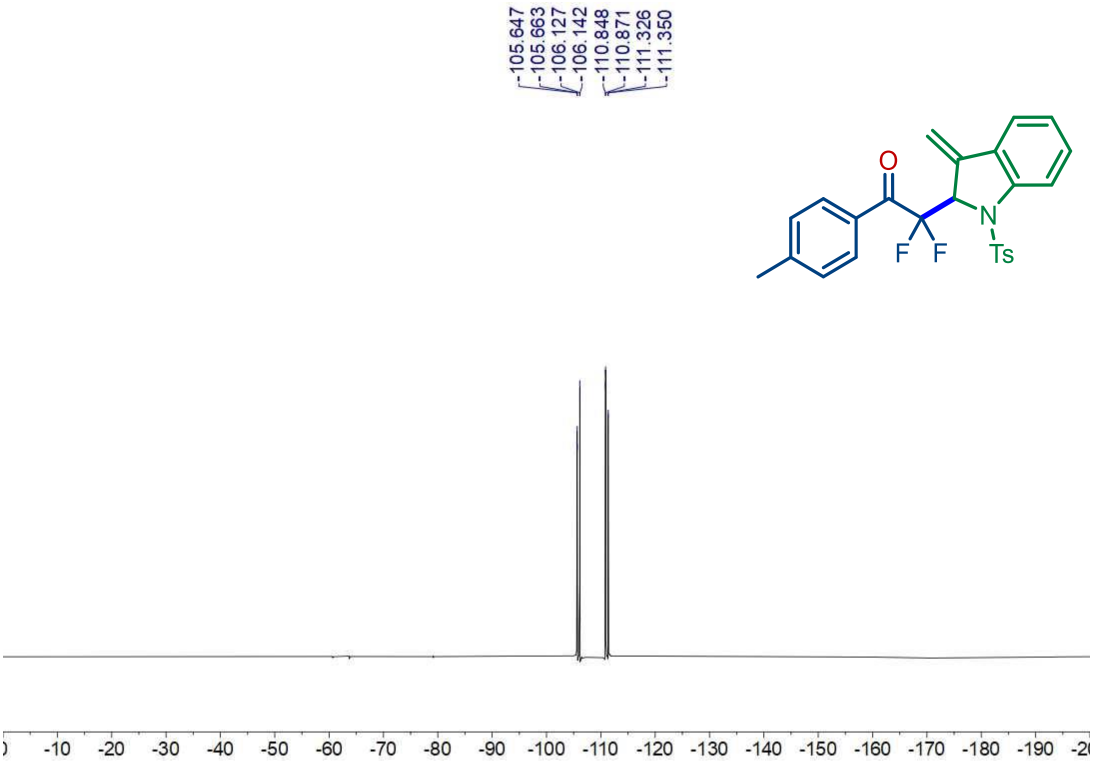

Supplementary Fig. 119 ^{19}F NMR (564 MHz, $CDCl_3$) spectrum of compound **39**

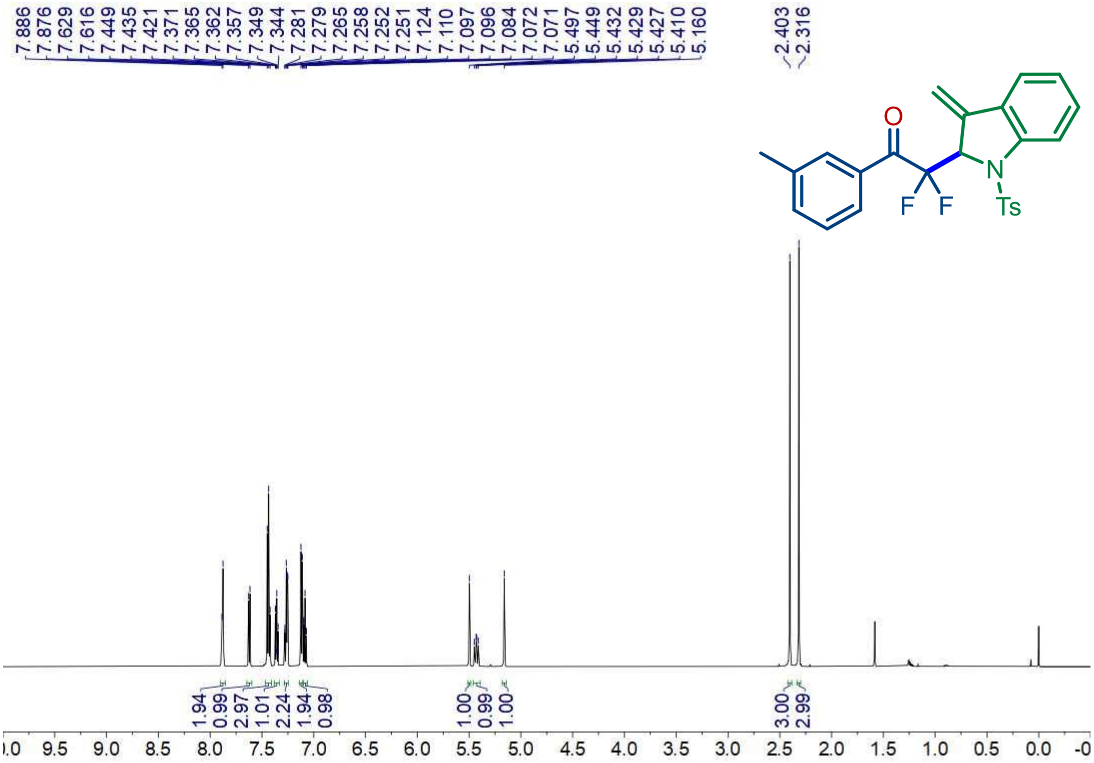

Supplementary Fig. 120 ^{1}H NMR (600 MHz, $CDCl_3$) spectrum of compound **40**

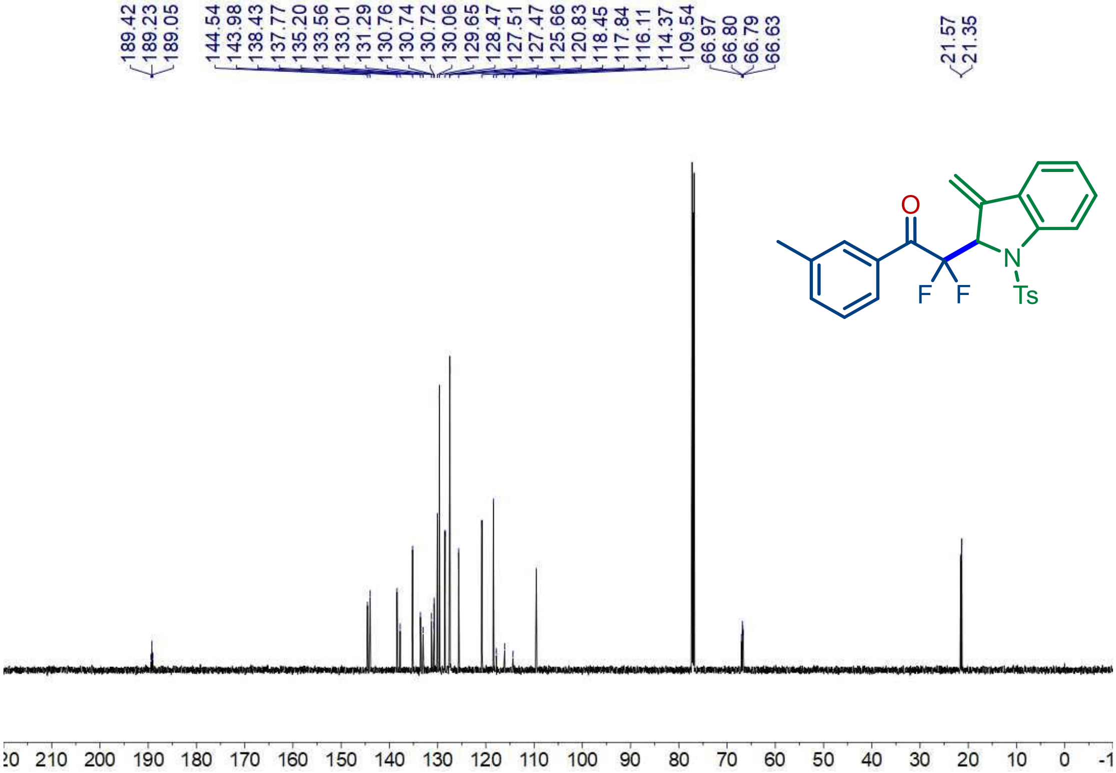

Supplementary Fig. 121 ^{13}C NMR (150 MHz, $CDCl_3$) spectrum of compound **40**

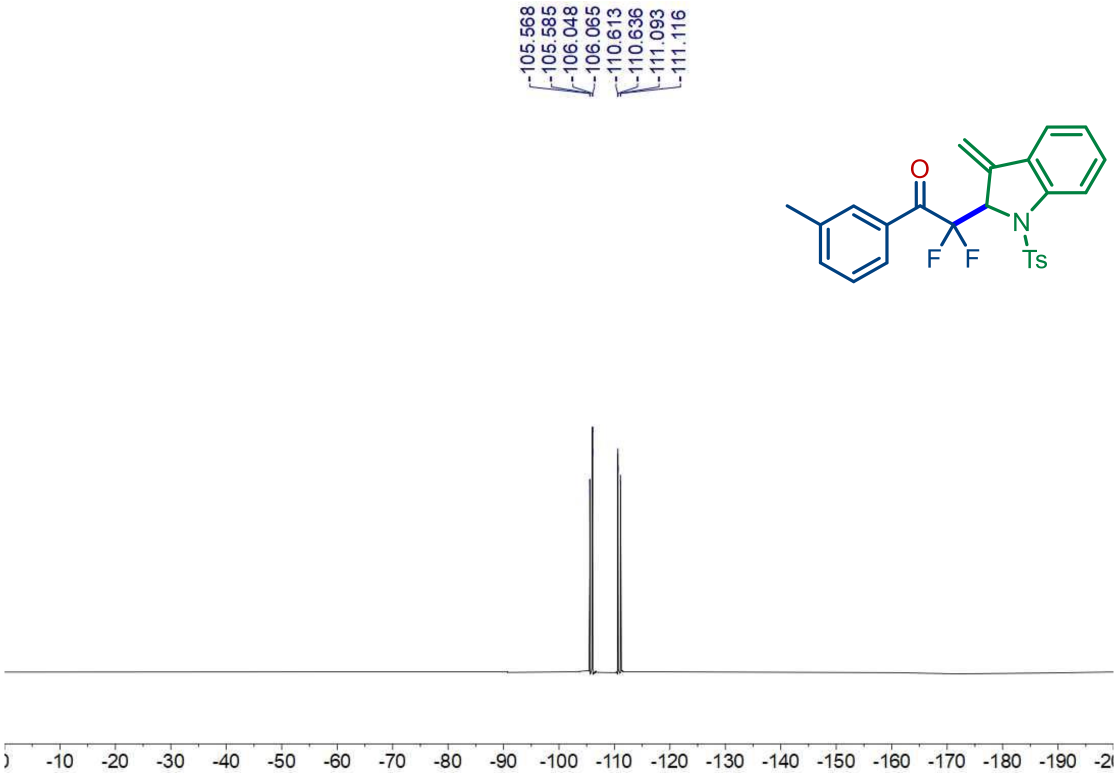

Supplementary Fig. 122 ^{19}F NMR (564 MHz, $CDCl_3$) spectrum of compound **40**

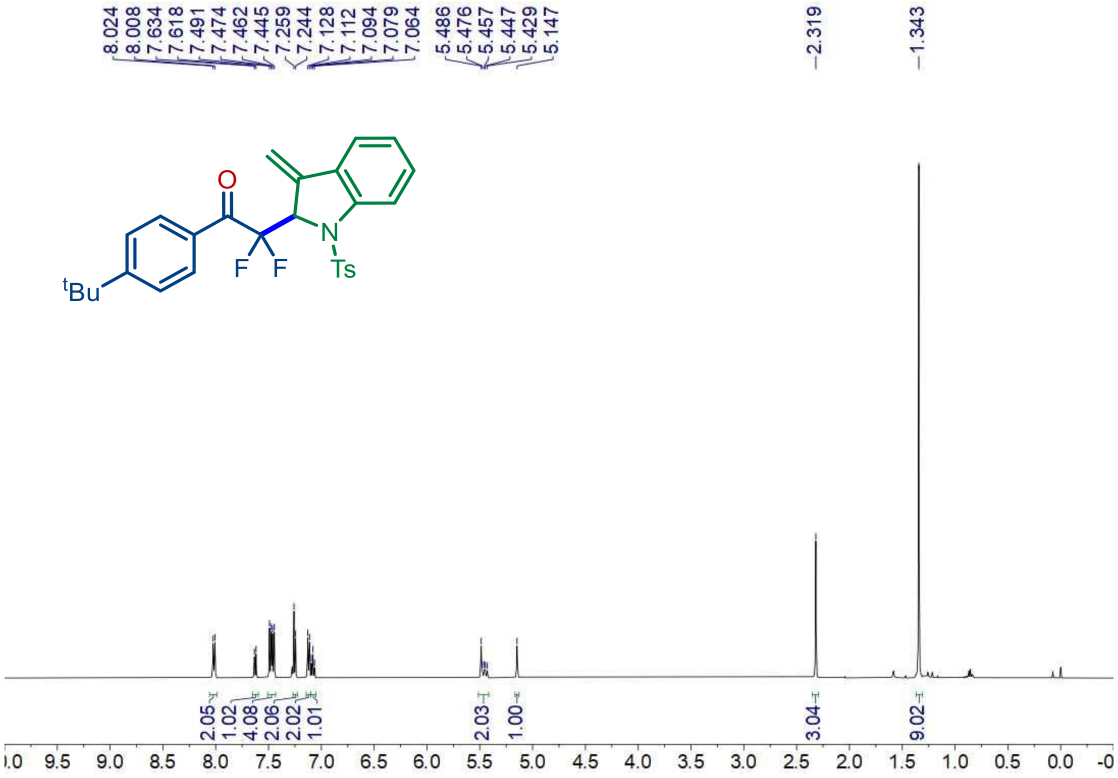

Supplementary Fig. 123 ^{1}H NMR (500 MHz, $CDCl_3$) spectrum of compound **41**

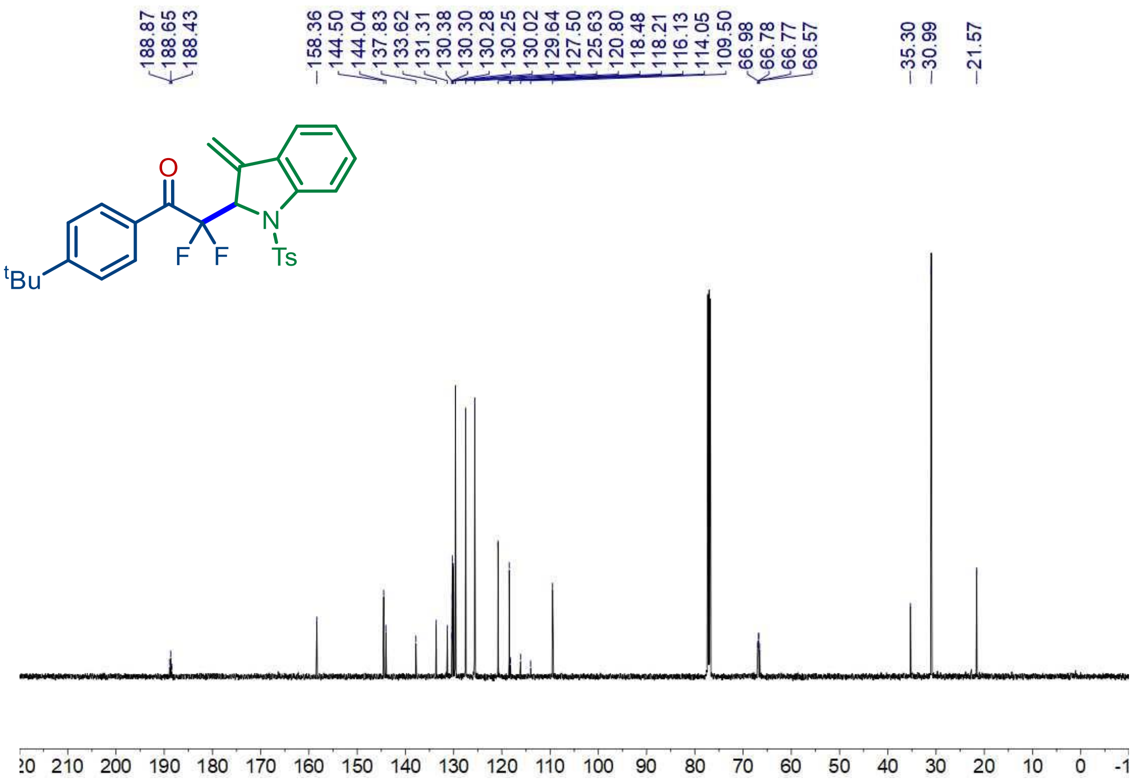

Supplementary Fig. 124 ^{13}C NMR (125 MHz, $CDCl_3$) spectrum of compound **41**

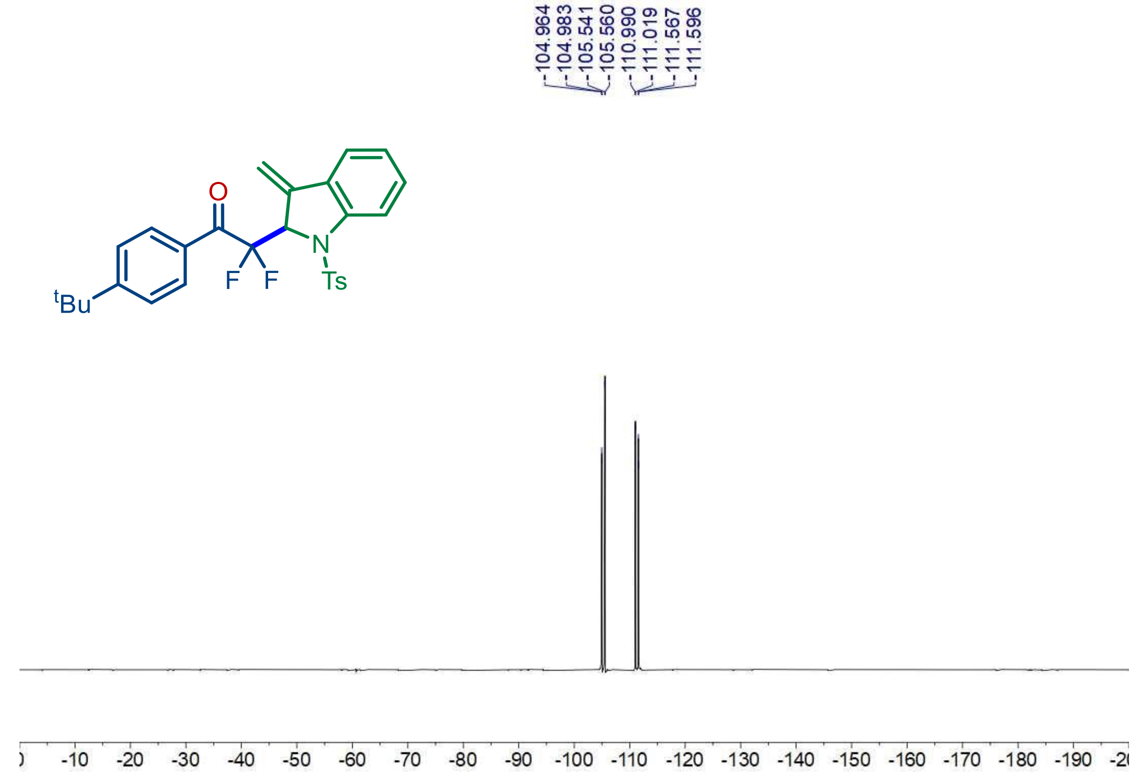

Supplementary Fig. 125 ^{19}F NMR (470 MHz, $CDCl_3$) spectrum of compound **41**

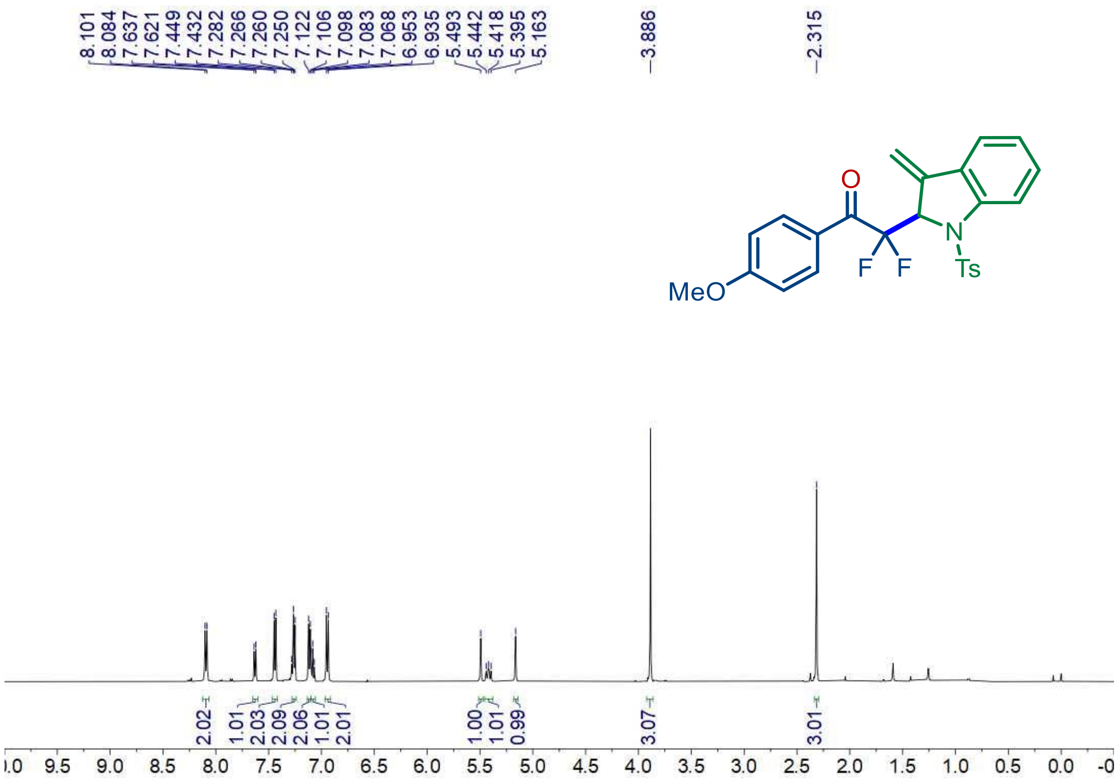

Supplementary Fig. 126 ^{1}H NMR (500 MHz, $CDCl_3$) spectrum of compound **42**

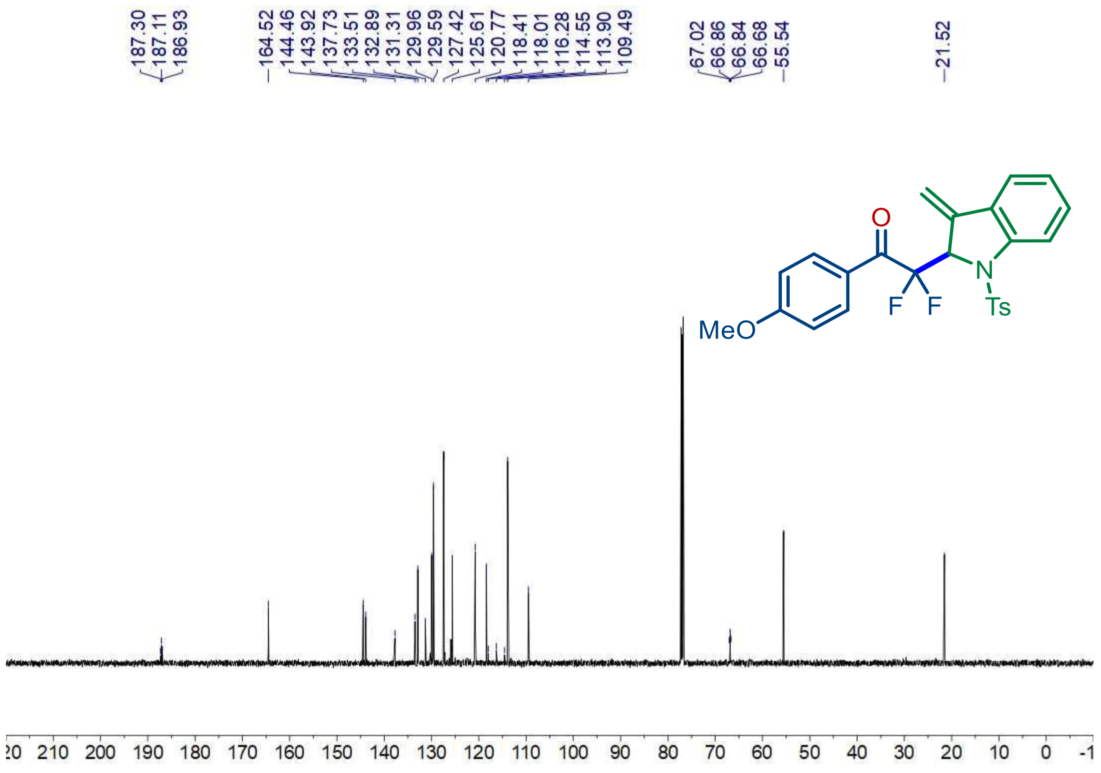

Supplementary Fig. 127 ^{13}C NMR (150 MHz, $CDCl_3$) spectrum of compound **42**

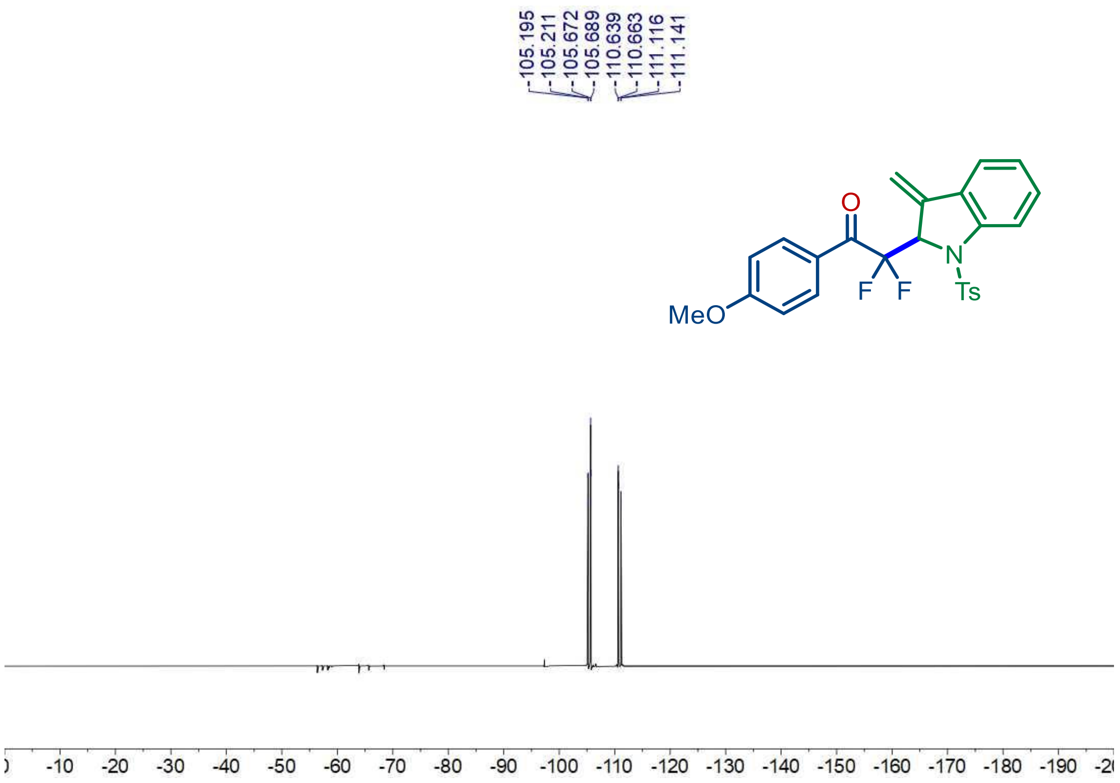

Supplementary Fig. 128 ^{19}F NMR (564 MHz, $CDCl_3$) spectrum of compound **42**

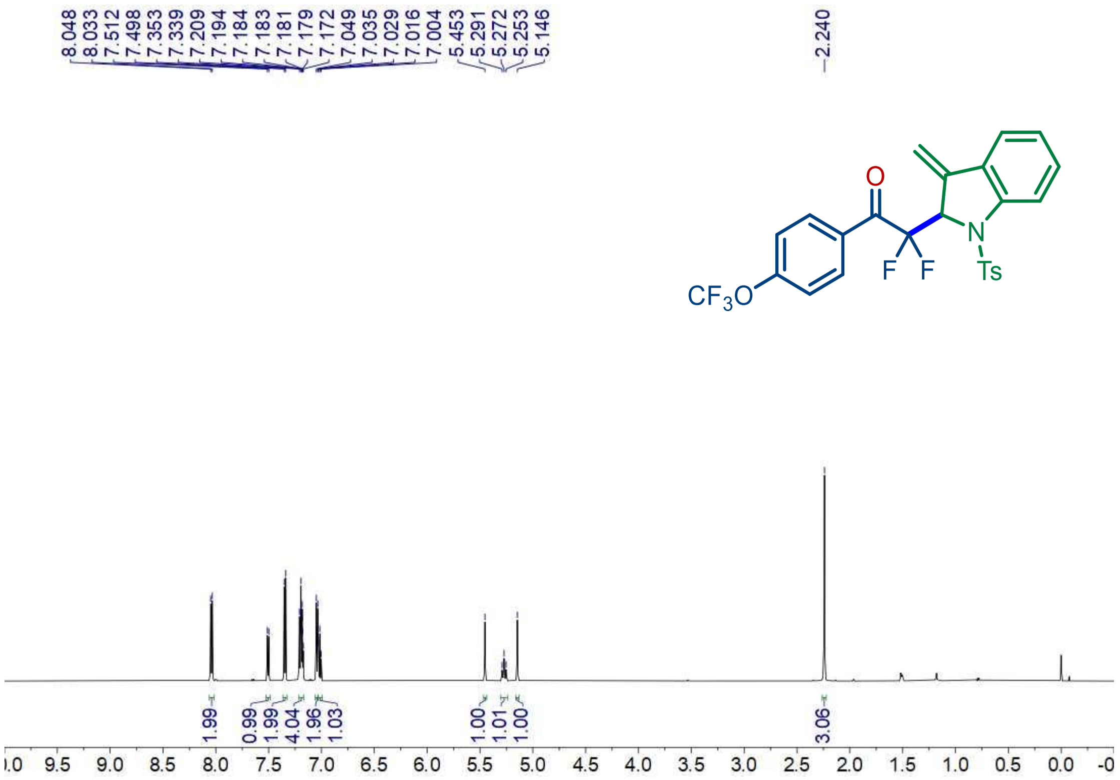

Supplementary Fig. 129 ^{1}H NMR (600 MHz, $CDCl_3$) spectrum of compound **43**

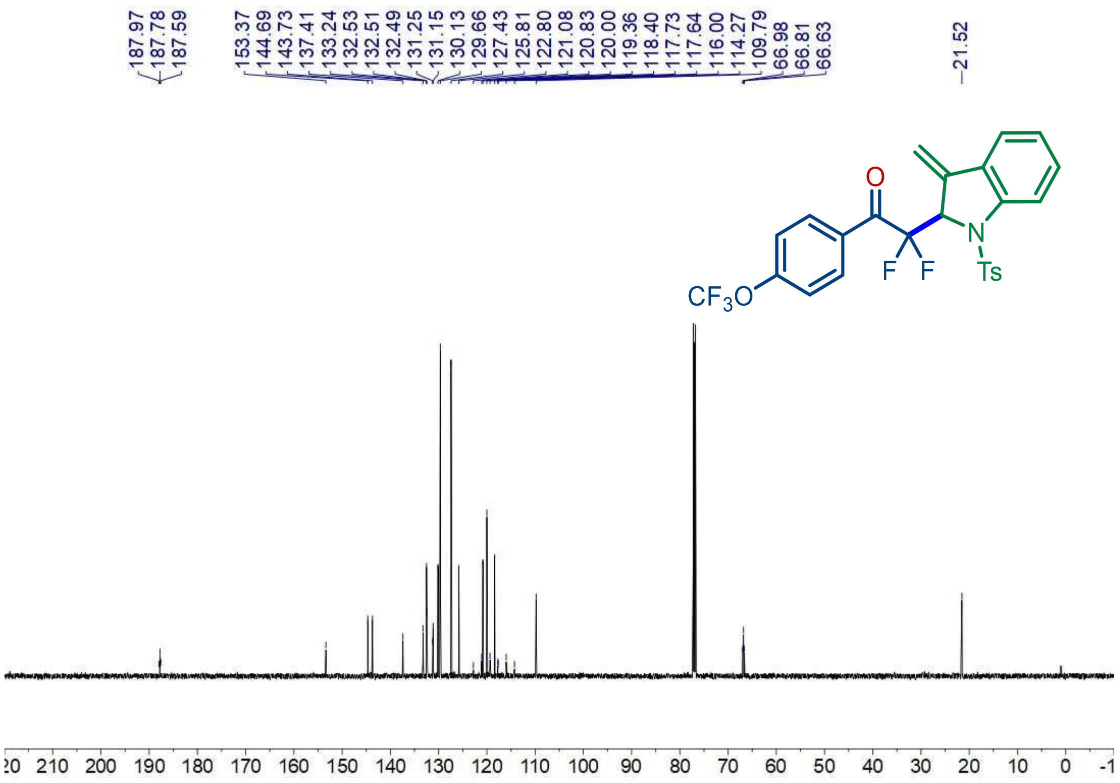

Supplementary Fig. 130 ^{13}C NMR (150 MHz, $CDCl_3$) spectrum of compound **43**

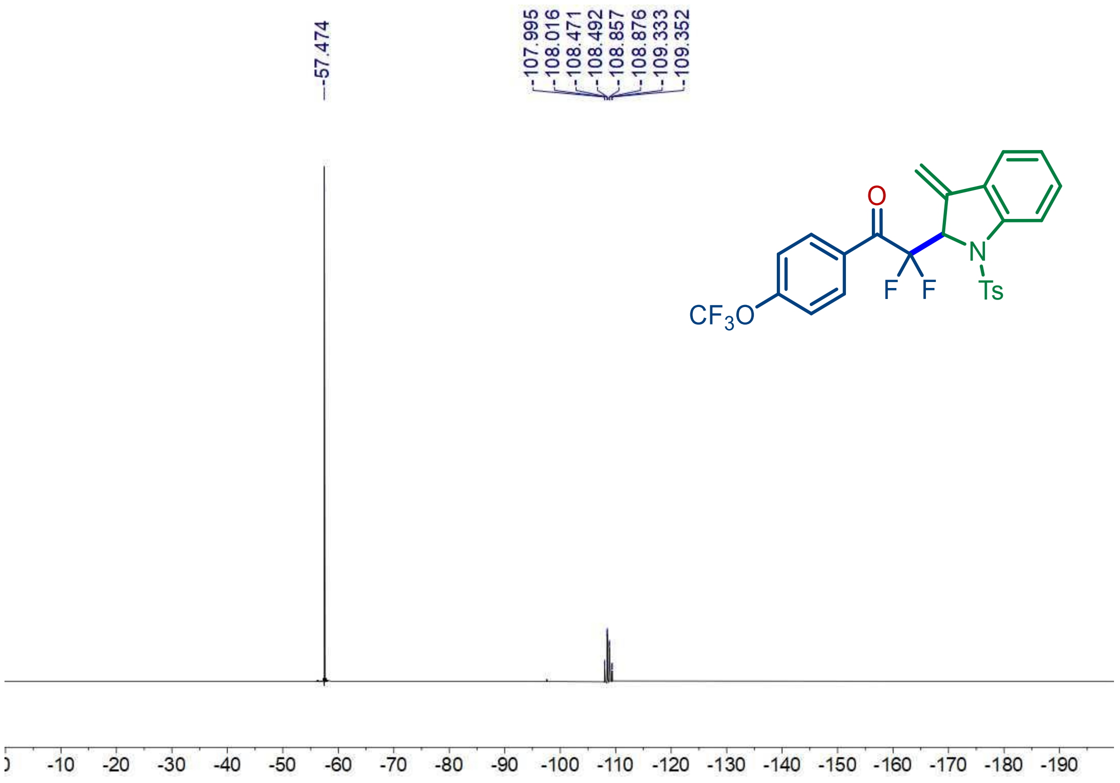

Supplementary Fig. 131 ^{19}F NMR (564 MHz, $CDCl_3$) spectrum of compound **43**

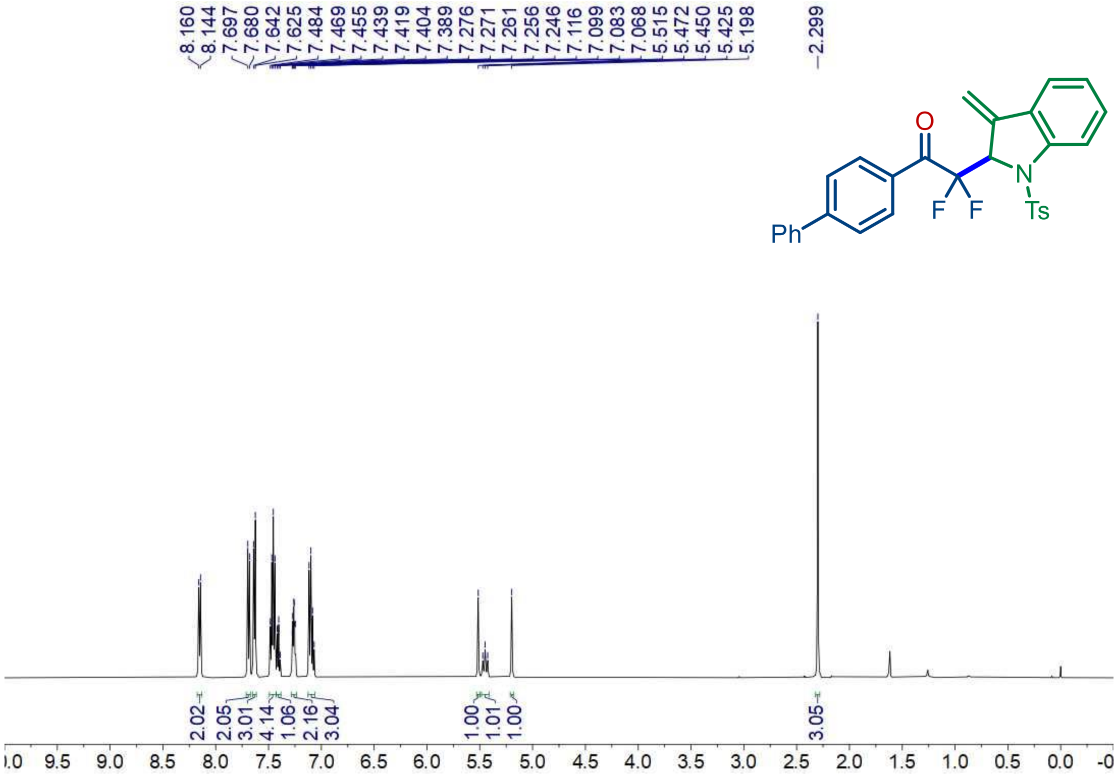

Supplementary Fig. 132 ^{1}H NMR (500 MHz, $CDCl_3$) spectrum of compound **44**

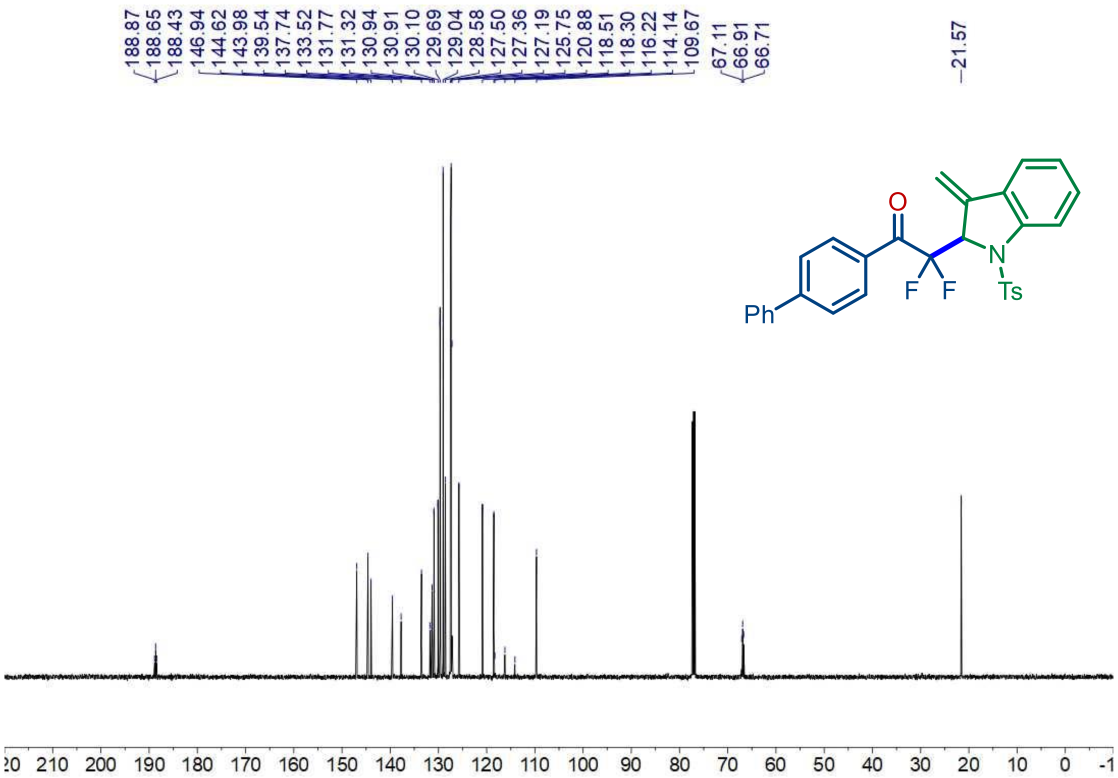

Supplementary Fig. 133 ^{13}C NMR (125 MHz, $CDCl_3$) spectrum of compound **44**

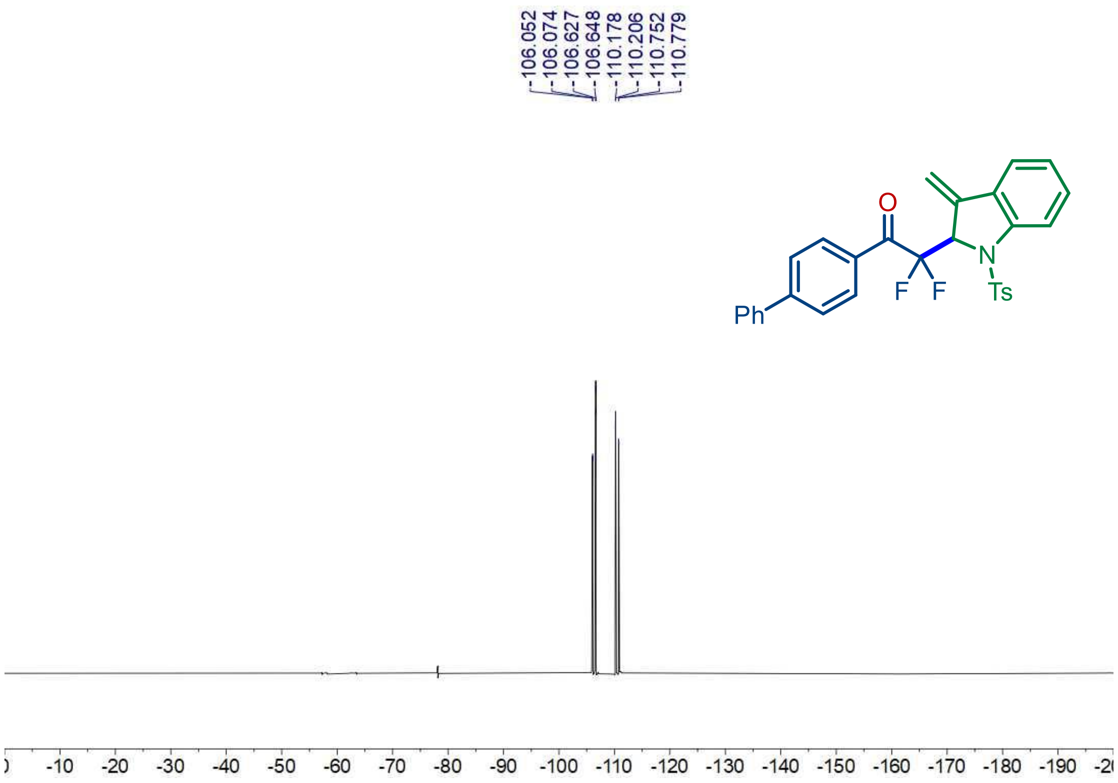

Supplementary Fig. 134 ^{19}F NMR (470 MHz, $CDCl_3$) spectrum of compound **44**

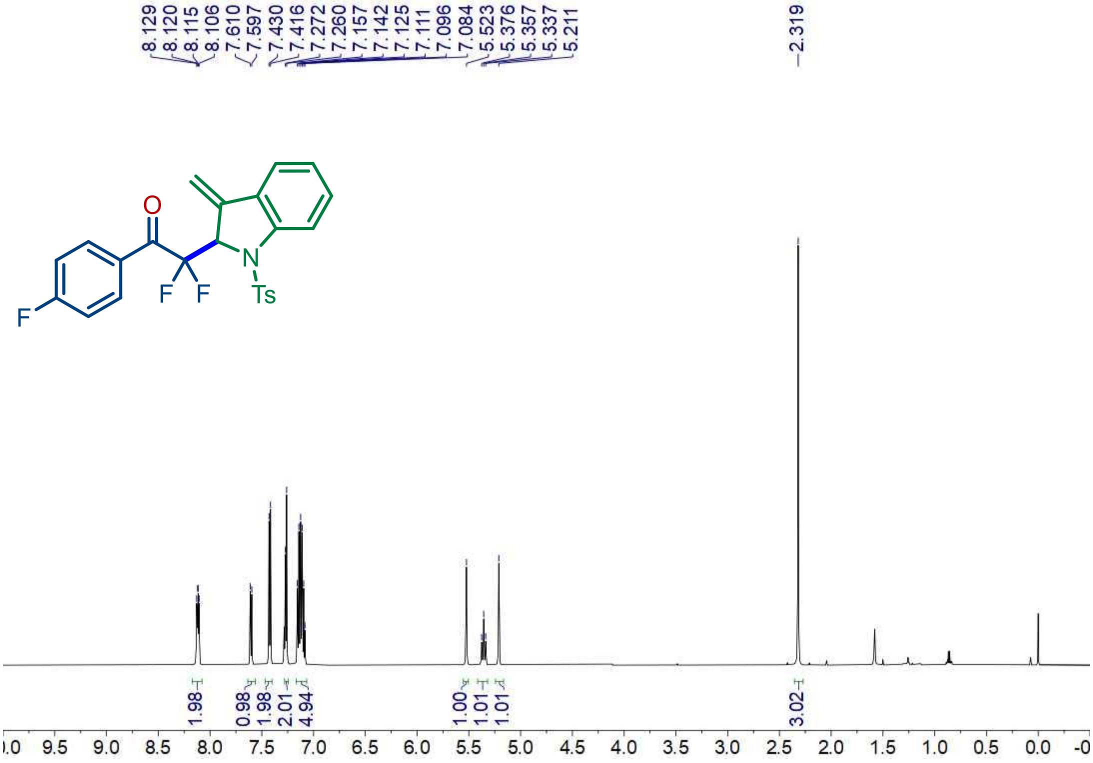

Supplementary Fig. 135 ^{1}H NMR (600 MHz, $CDCl_3$) spectrum of compound **45**

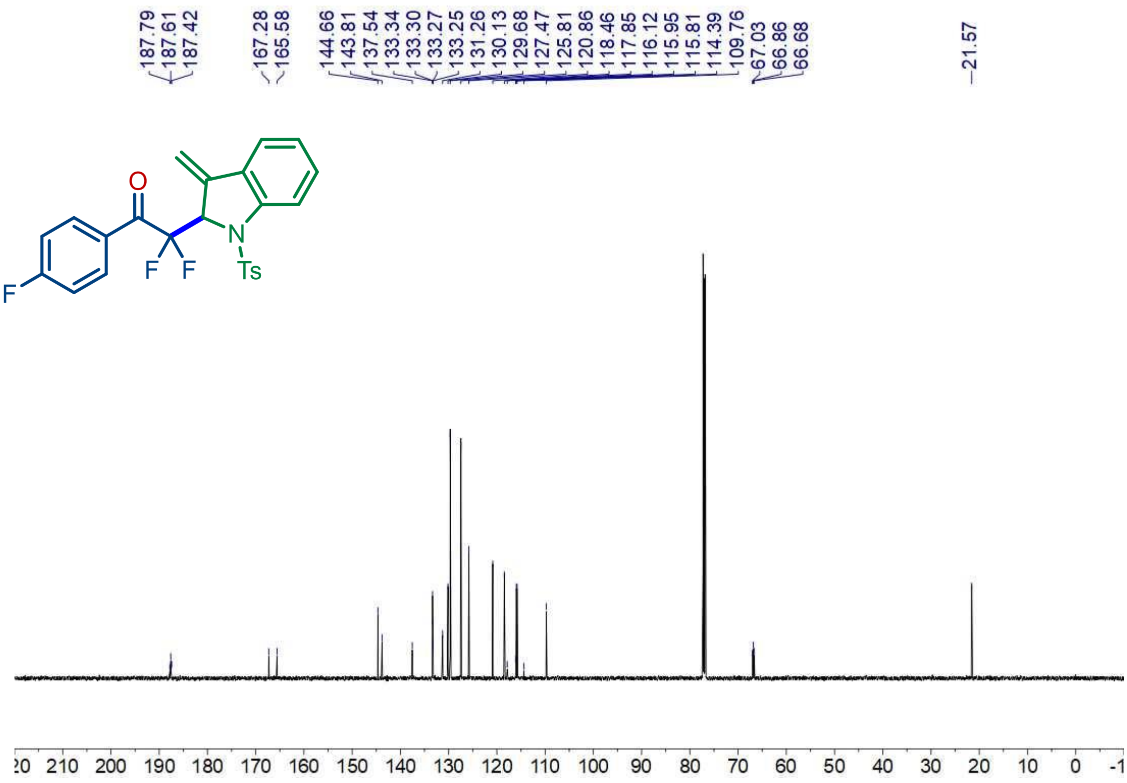

Supplementary Fig. 136 ^{13}C NMR (150 MHz, $CDCl_3$) spectrum of compound **45**

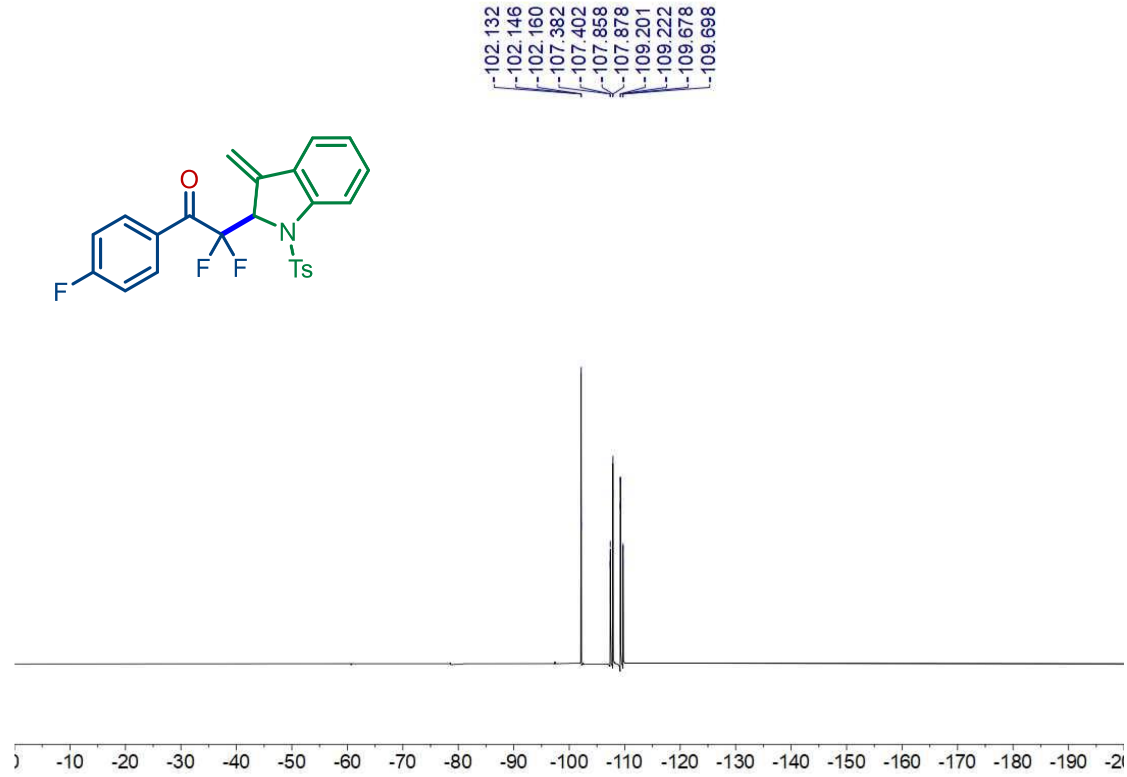

Supplementary Fig. 137 ^{19}F NMR (564 MHz, $CDCl_3$) spectrum of compound **45**

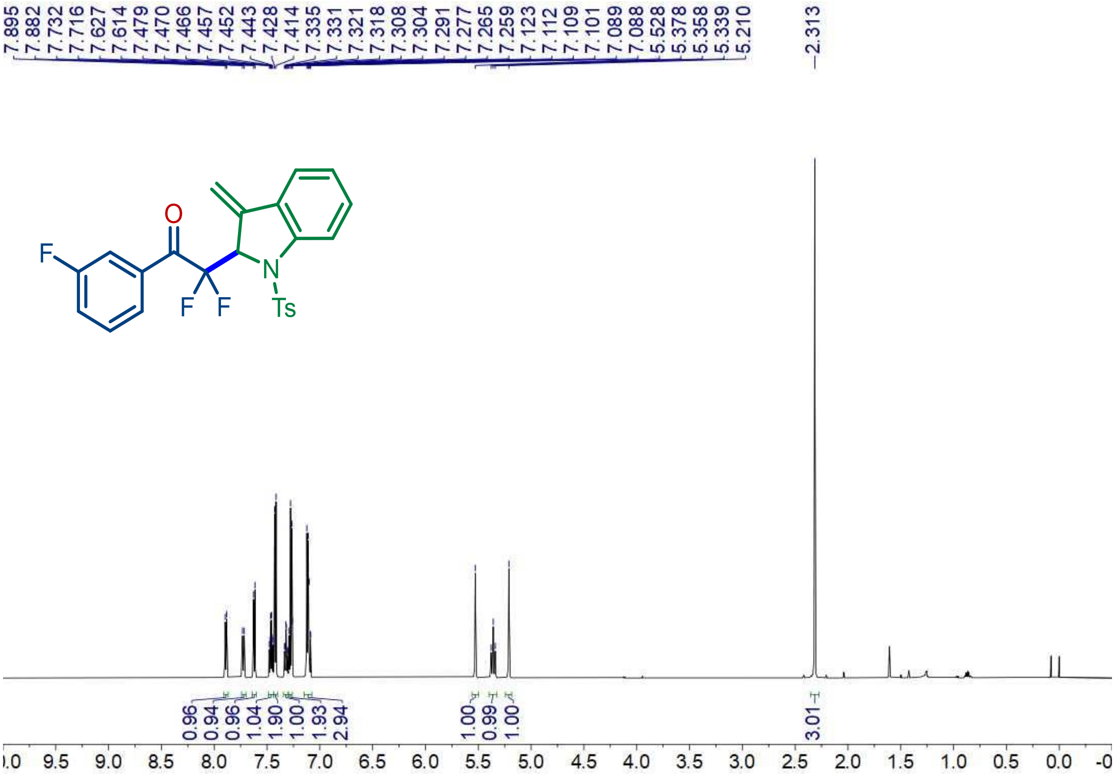

Supplementary Fig. 138 ^{1}H NMR (600 MHz, $CDCl_3$) spectrum of compound **46**

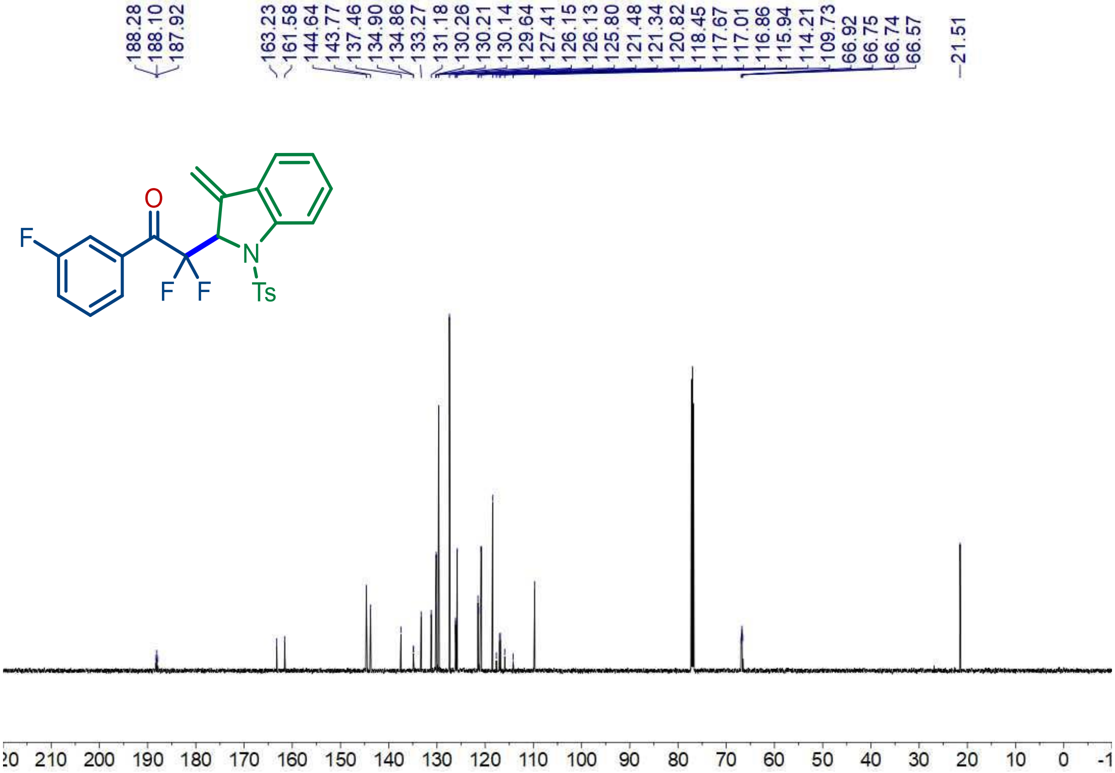

Supplementary Fig. 139 ^{13}C NMR (150 MHz, $CDCl_3$) spectrum of compound **46**

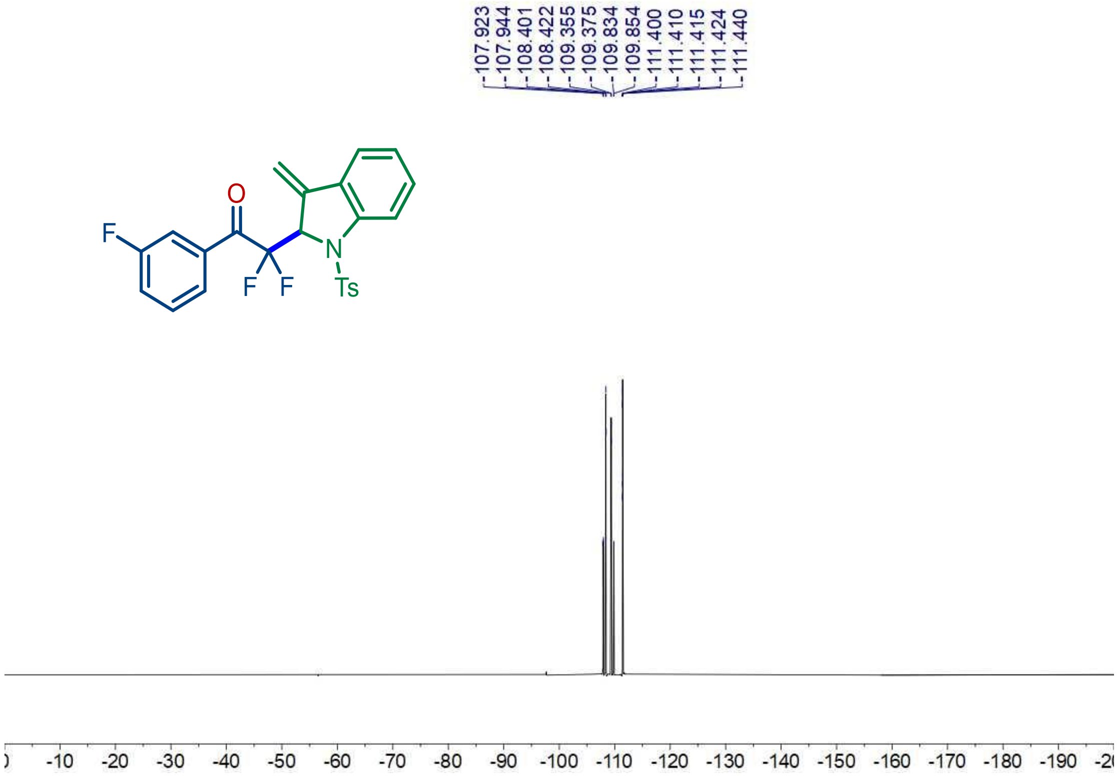

Supplementary Fig. 140 ^{19}F NMR (564 MHz, $CDCl_3$) spectrum of compound **46**

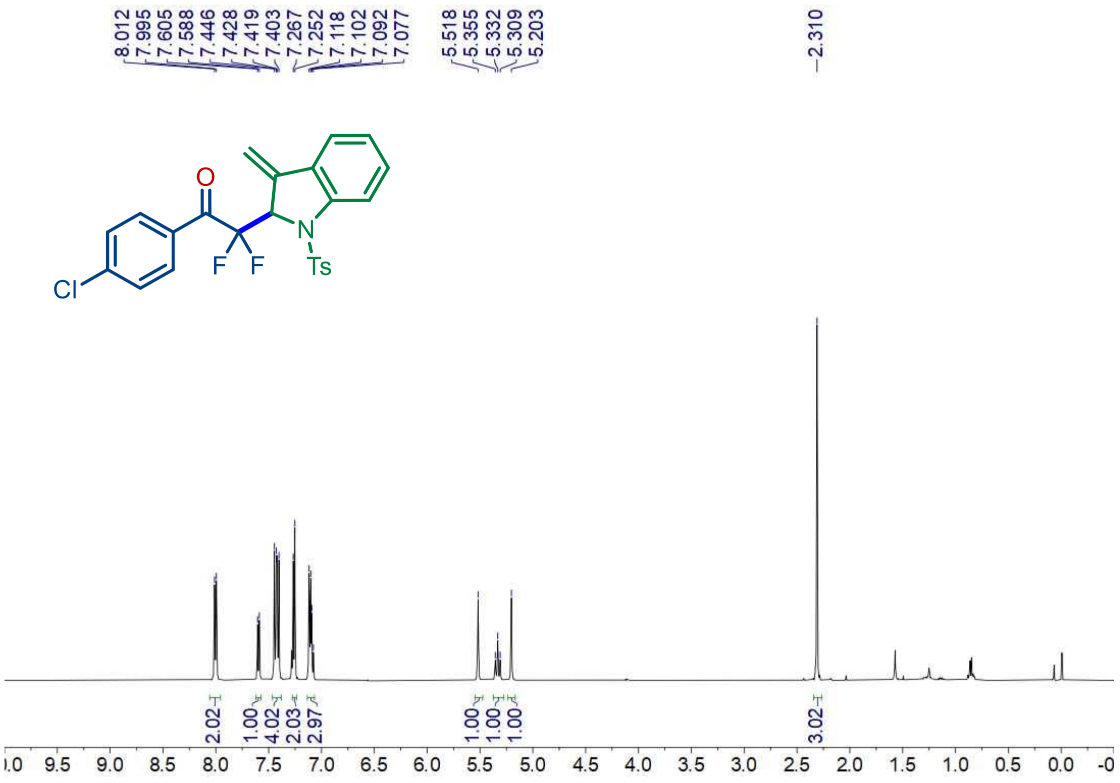

Supplementary Fig. 141 ^{1}H NMR (500 MHz, $CDCl_3$) spectrum of compound **47**

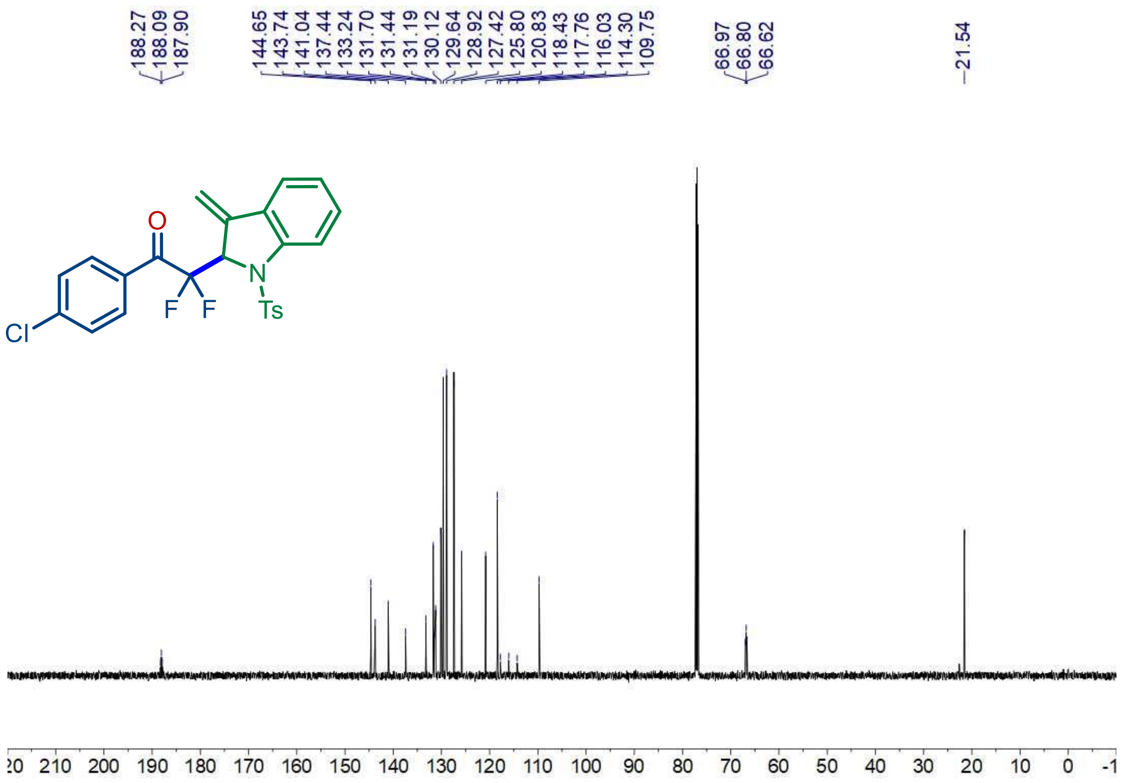

Supplementary Fig. 142 ^{13}C NMR (125 MHz, $CDCl_3$) spectrum of compound **47**

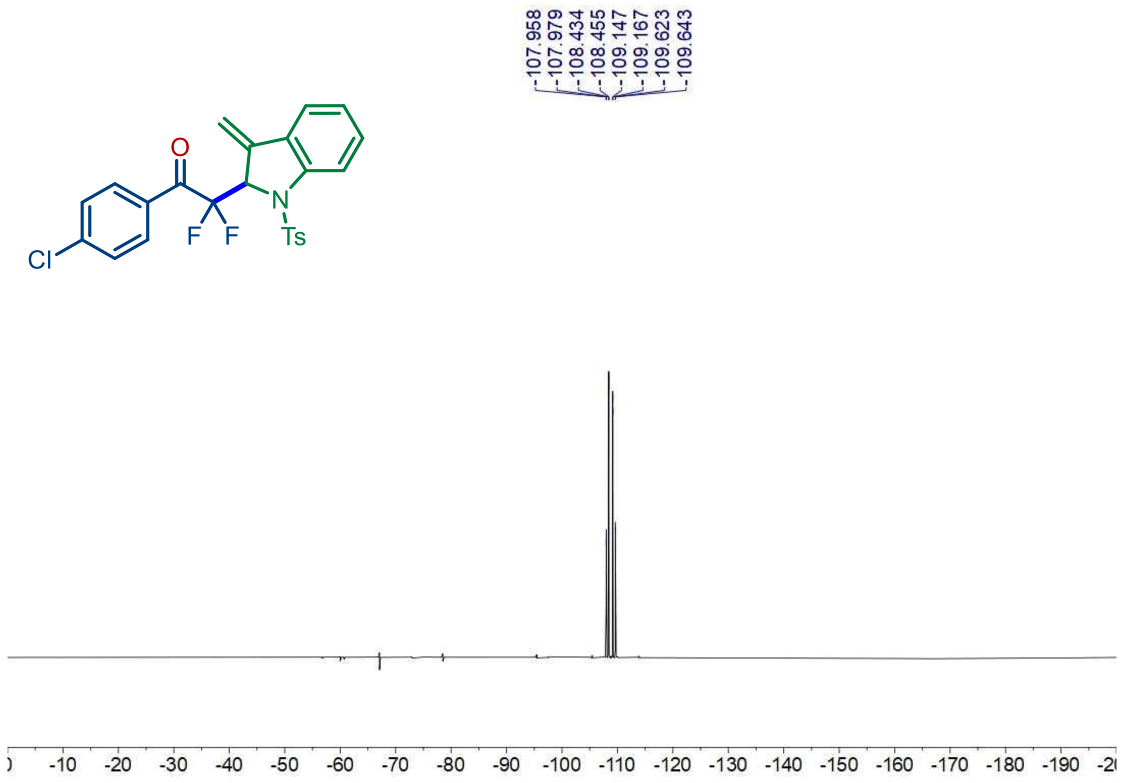

Supplementary Fig. 143 ^{19}F NMR (564 MHz, $CDCl_3$) spectrum of compound **47**

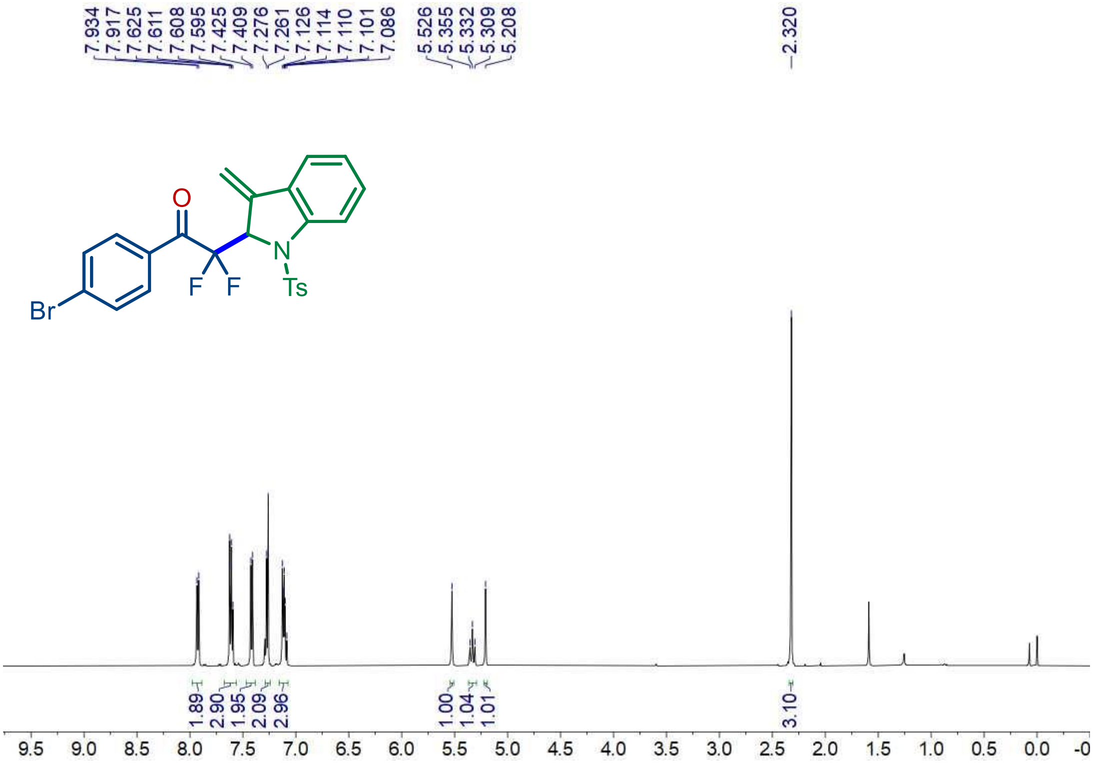

Supplementary Fig. 144 ¹H NMR (500 MHz, $CDCl_3$) spectrum of compound **48**

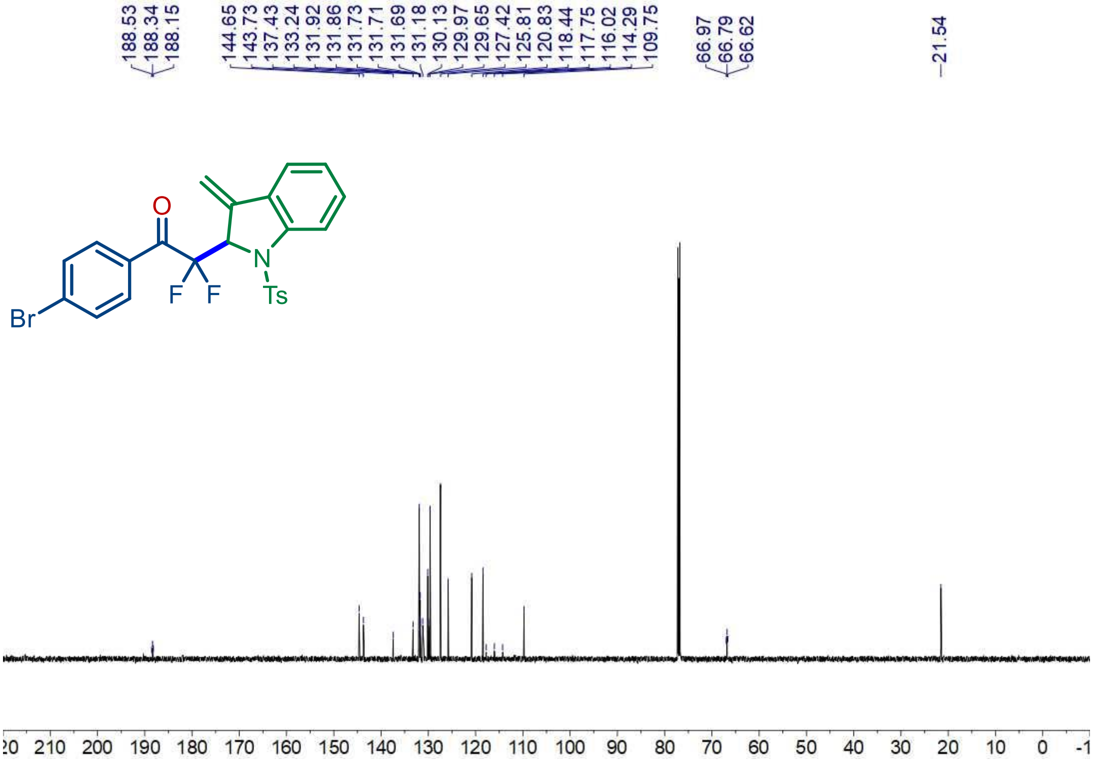

Supplementary Fig. 145 ¹³C NMR (150 MHz, $CDCl_3$) spectrum of compound **48**

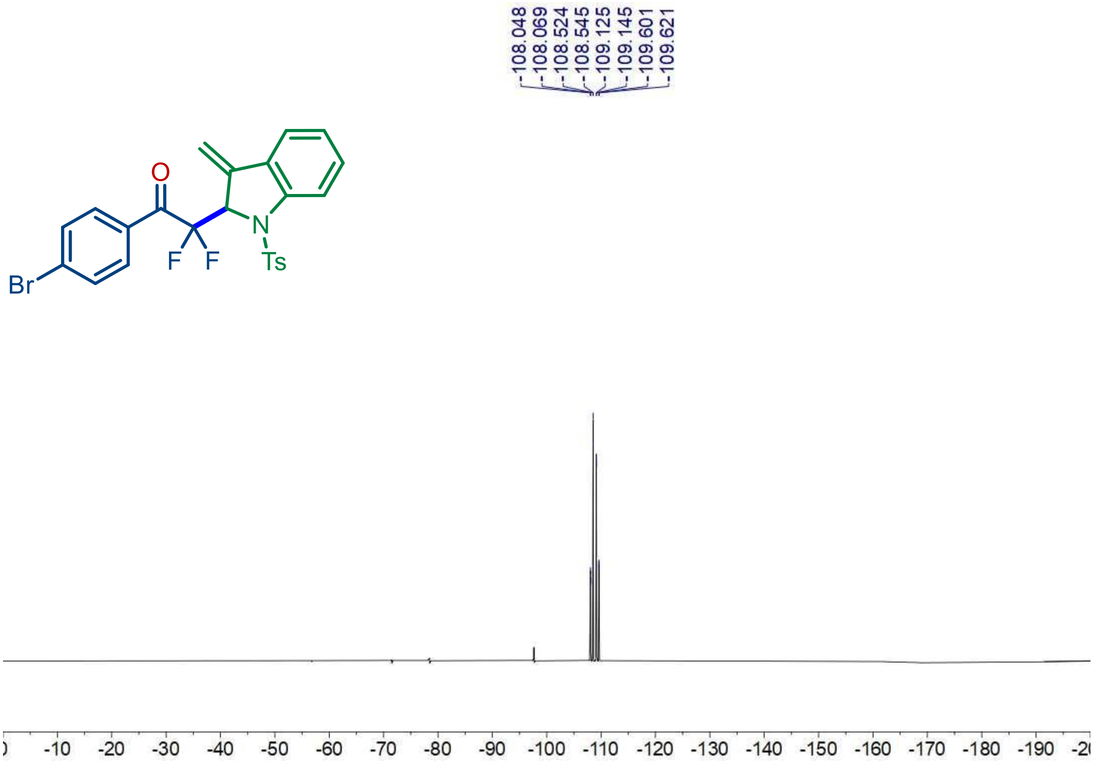

Supplementary Fig. 146 ^{19}F NMR (564 MHz, $CDCl_3$) spectrum of compound **48**

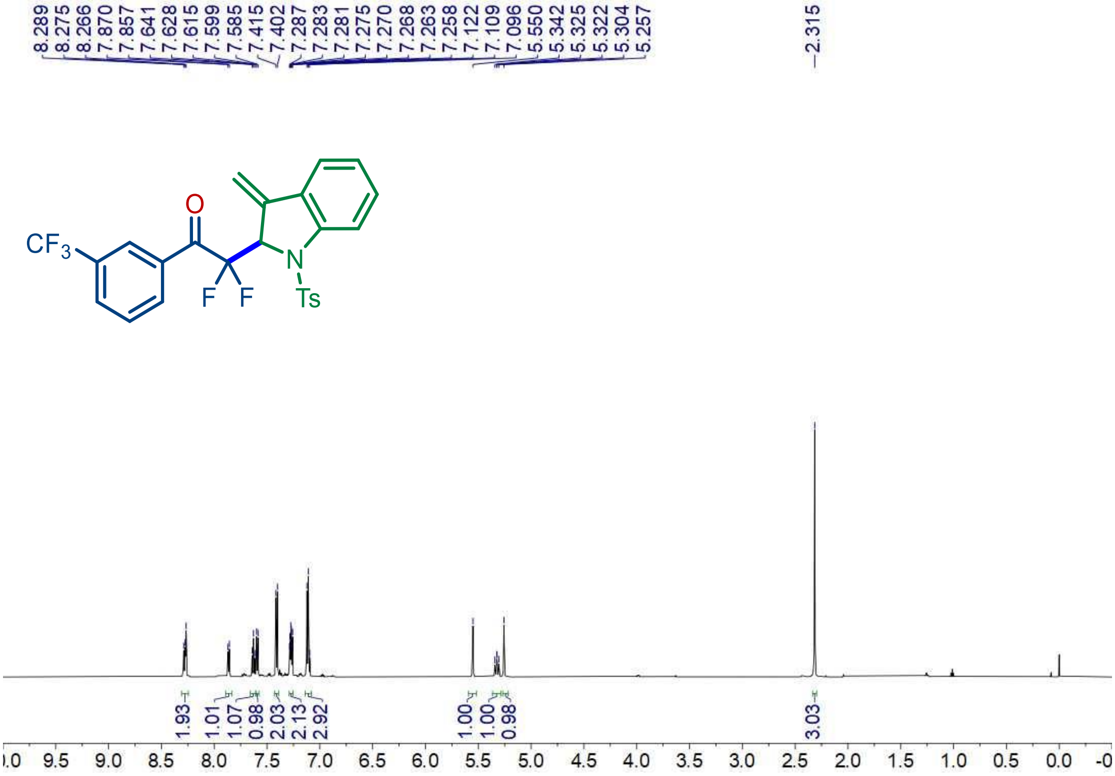

Supplementary Fig. 147 ^{1}H NMR (600 MHz, $CDCl_3$) spectrum of compound **49**

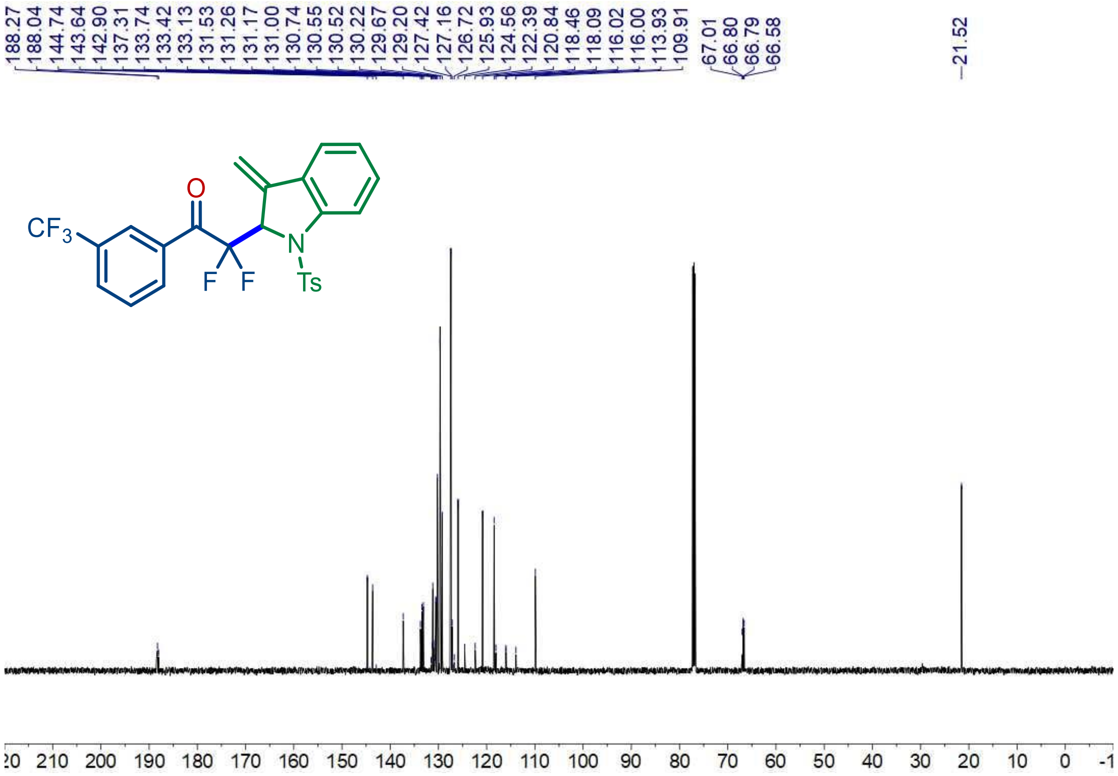

Supplementary Fig. 148 ^{13}C NMR (125 MHz, $CDCl_3$) spectrum of compound **49**

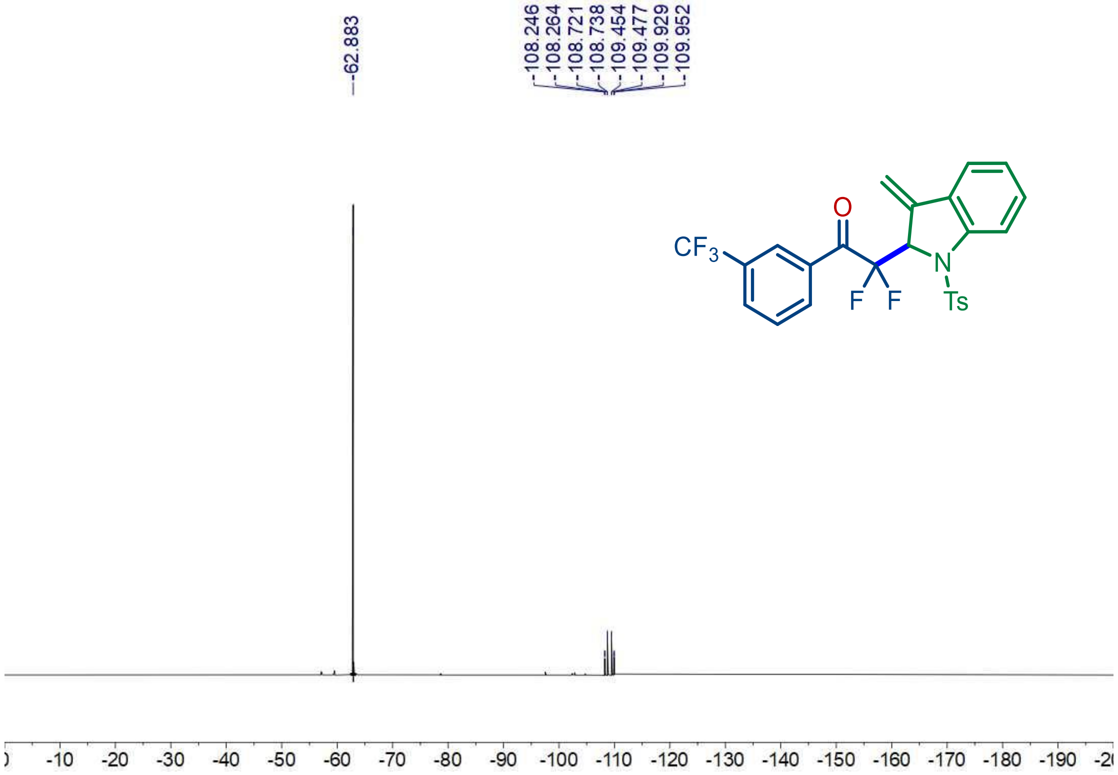

Supplementary Fig. 149 ^{19}F NMR (564 MHz, $CDCl_3$) spectrum of compound **49**

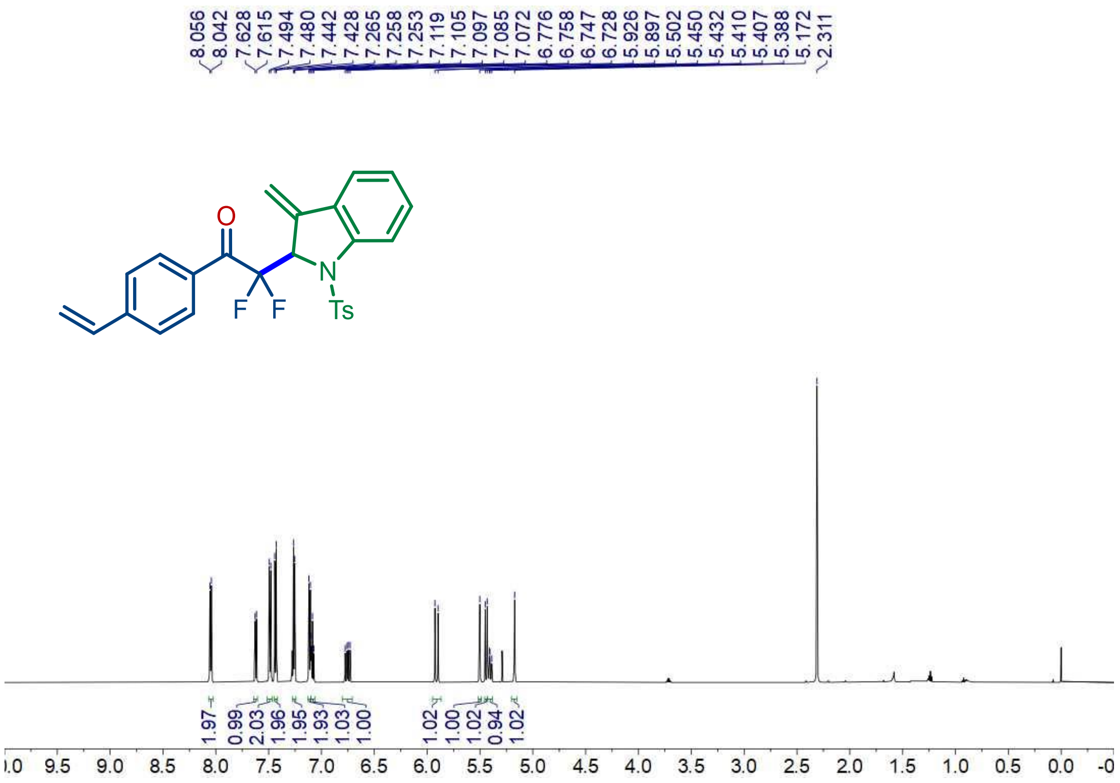

Supplementary Fig. 150 ^{1}H NMR (600 MHz, $CDCl_3$) spectrum of compound **50**

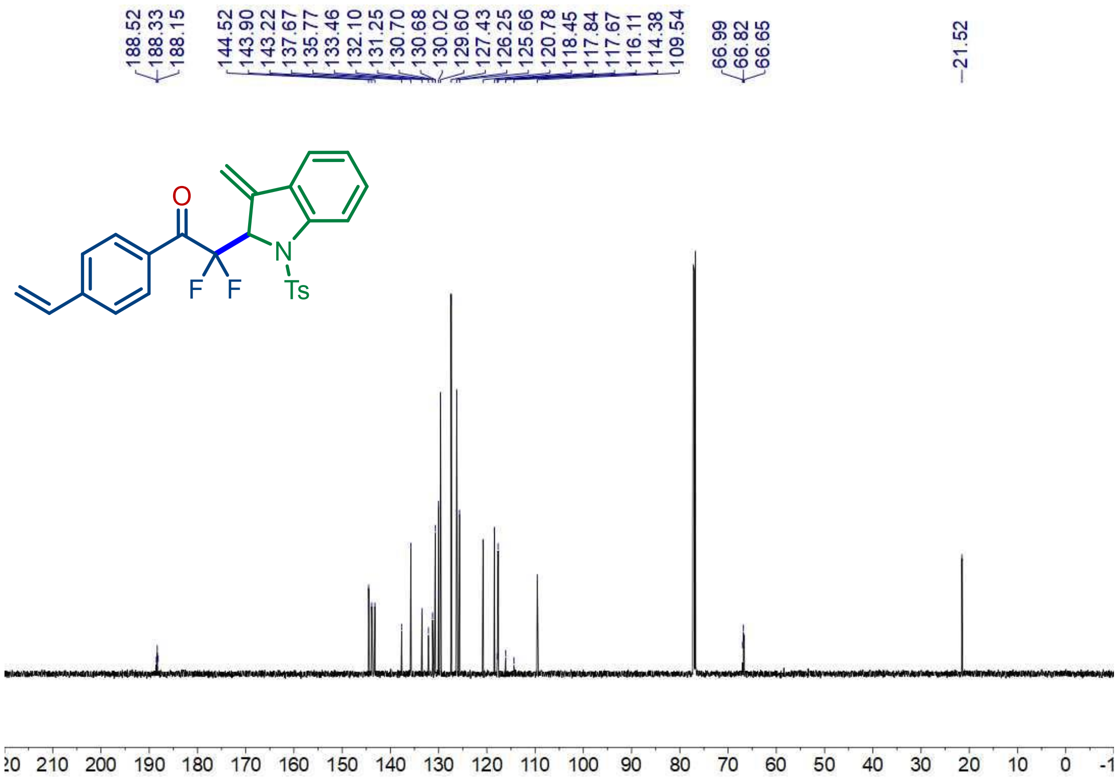

Supplementary Fig. 151 ^{13}C NMR (150 MHz, $CDCl_3$) spectrum of compound **50**

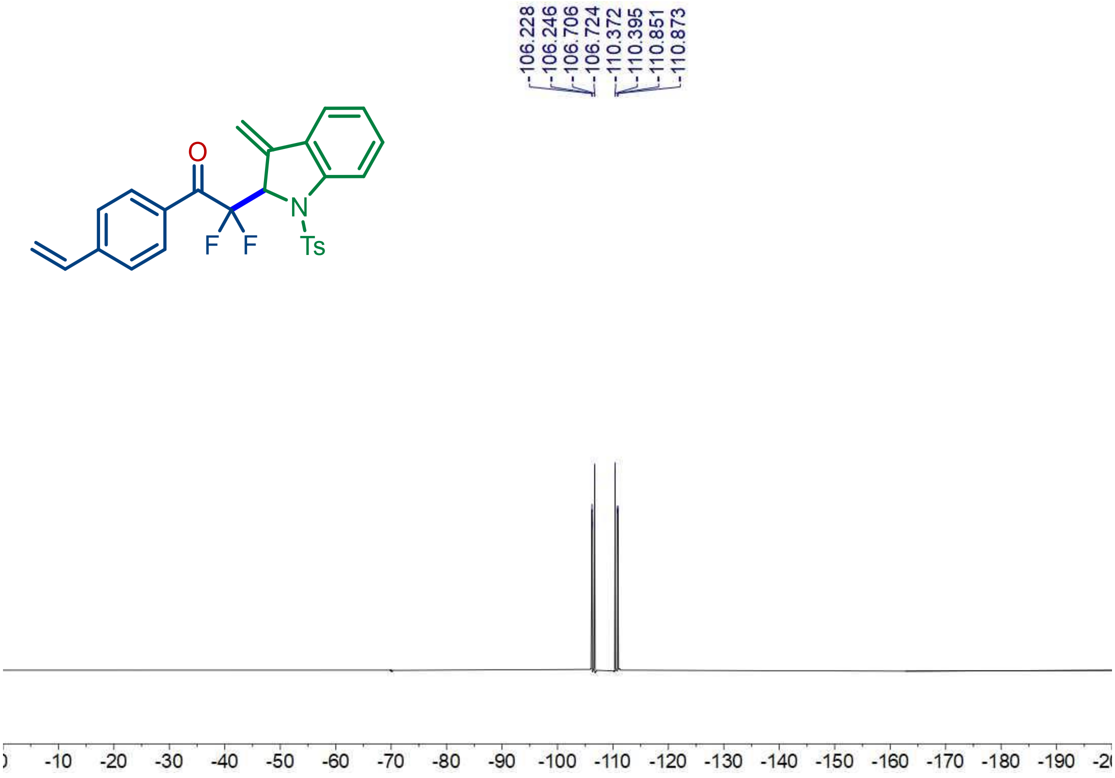

Supplementary Fig. 152 ^{19}F NMR (564 MHz, $CDCl_3$) spectrum of compound **50**

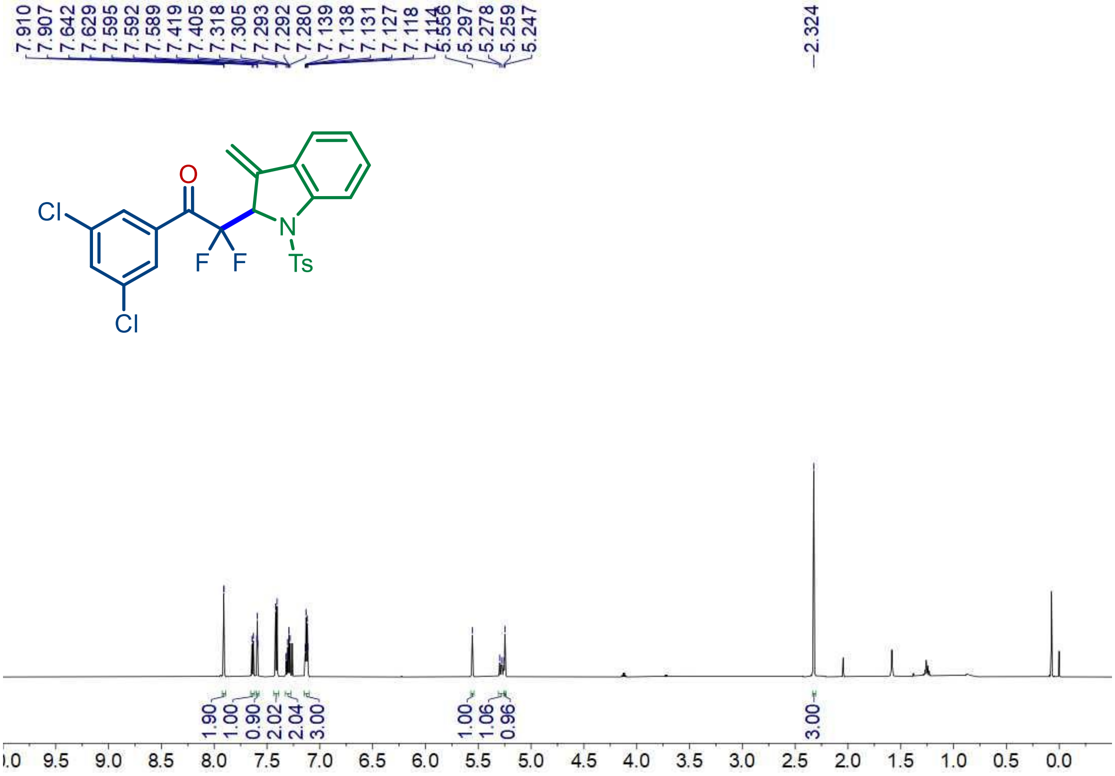

Supplementary Fig. 153 ^{1}H NMR (600 MHz, $CDCl_3$) spectrum of compound **51**

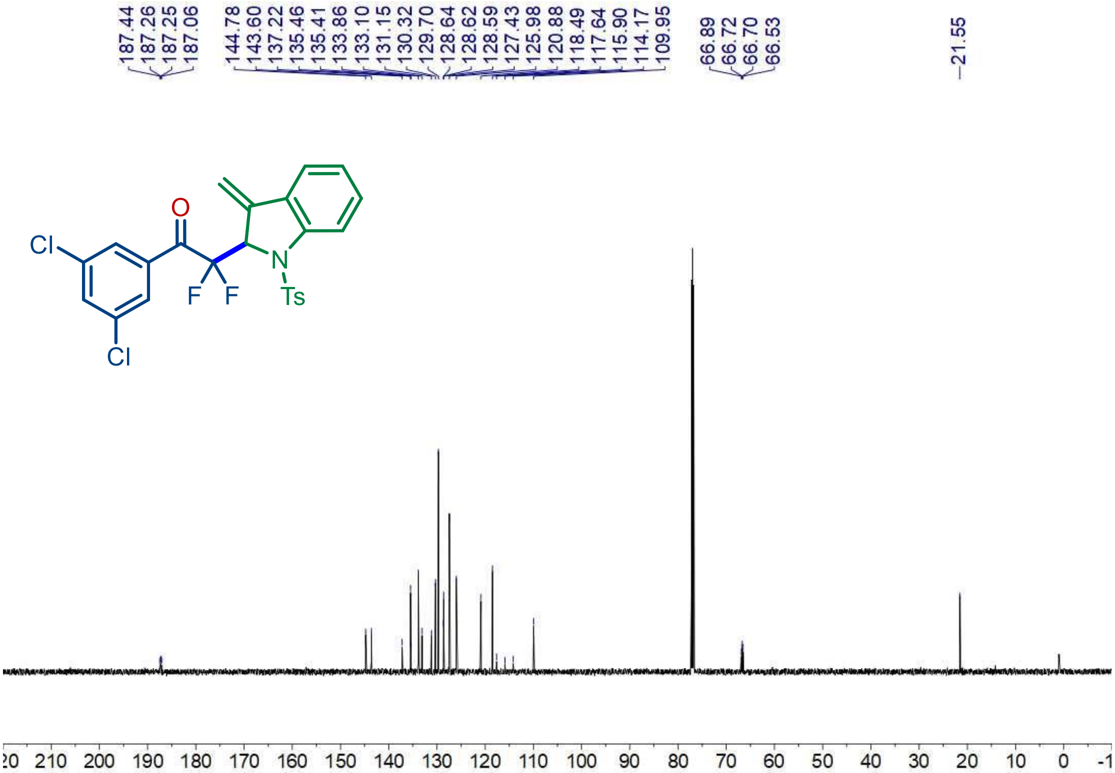

Supplementary Fig. 154 ^{13}C NMR (150 MHz, $CDCl_3$) spectrum of compound **51**

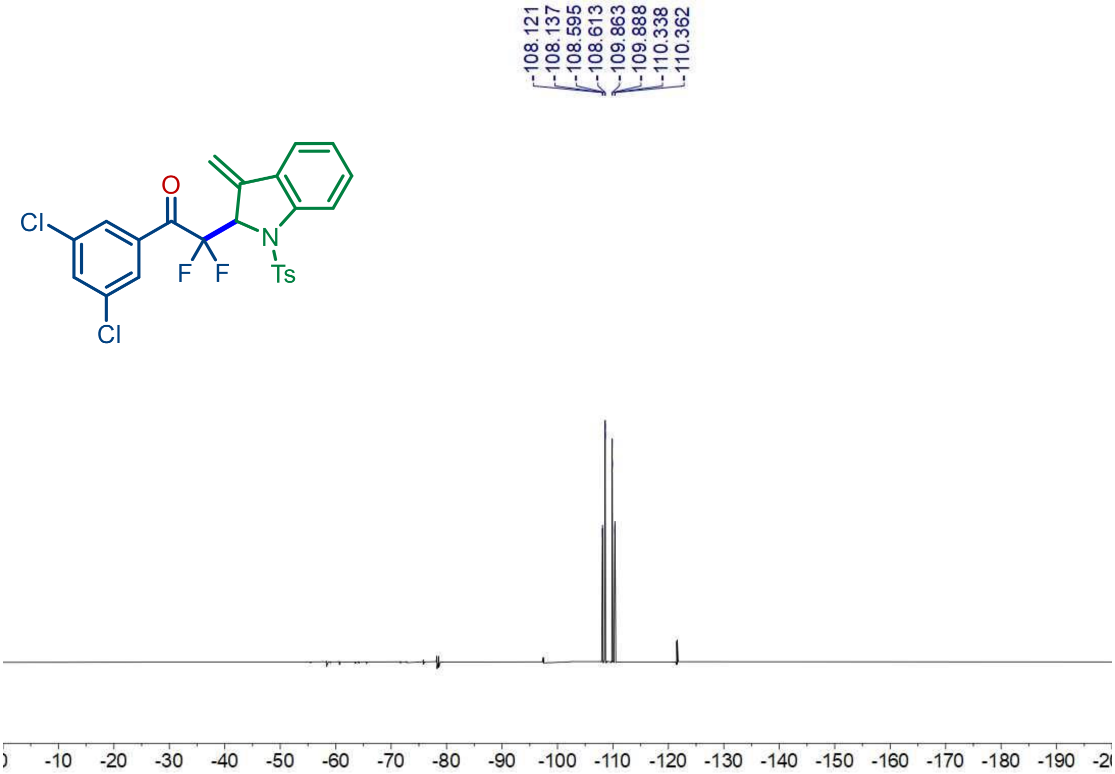

Supplementary Fig. 155 ^{19}F NMR (564 MHz, $CDCl_3$) spectrum of compound **51**

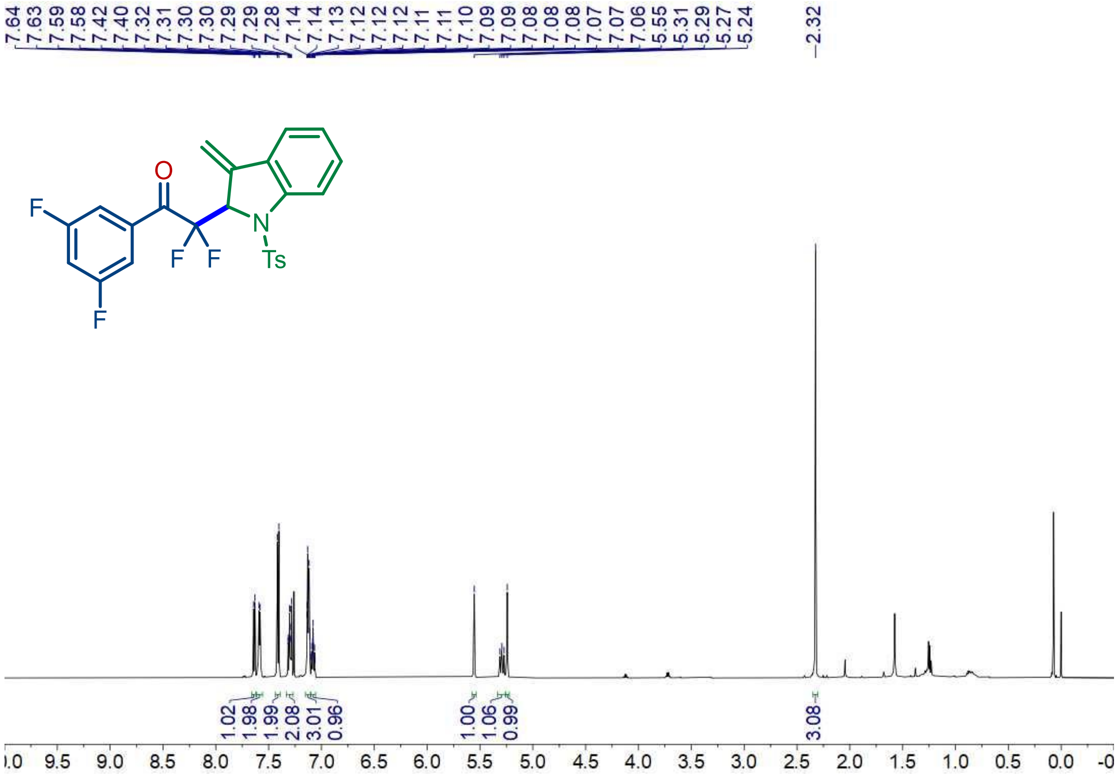

Supplementary Fig. 156 ^{1}H NMR (600 MHz, $CDCl_3$) spectrum of compound **52**

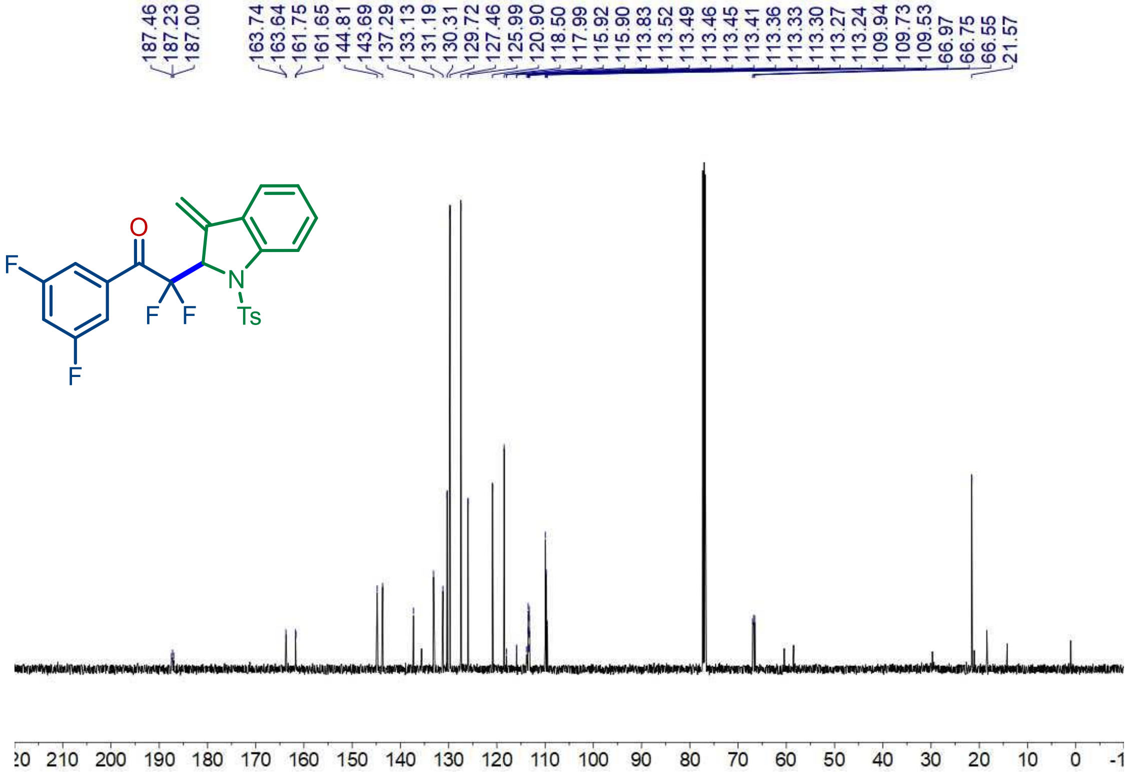

Supplementary Fig. 157 ^{13}C NMR (125 MHz, $CDCl_3$) spectrum of compound **52**

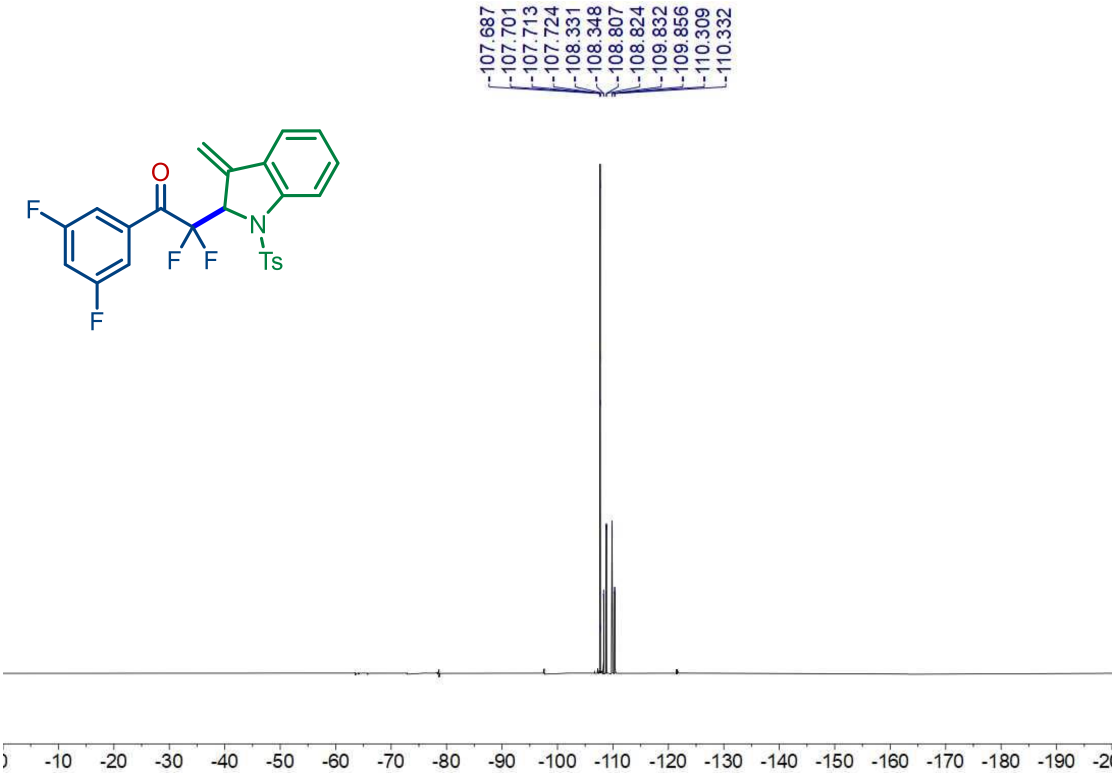

Supplementary Fig. 158 ^{19}F NMR (564 MHz, $CDCl_3$) spectrum of compound **52**

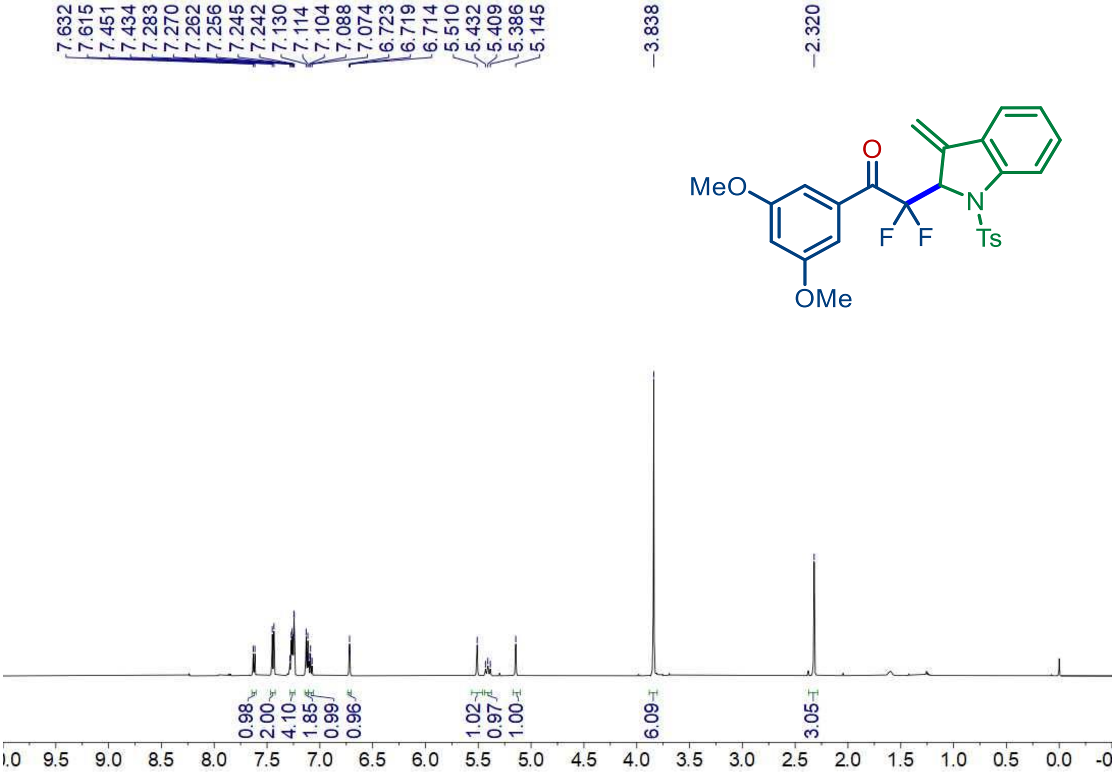

Supplementary Fig. 159 ^{1}H NMR (500 MHz, $CDCl_3$) spectrum of compound **53**

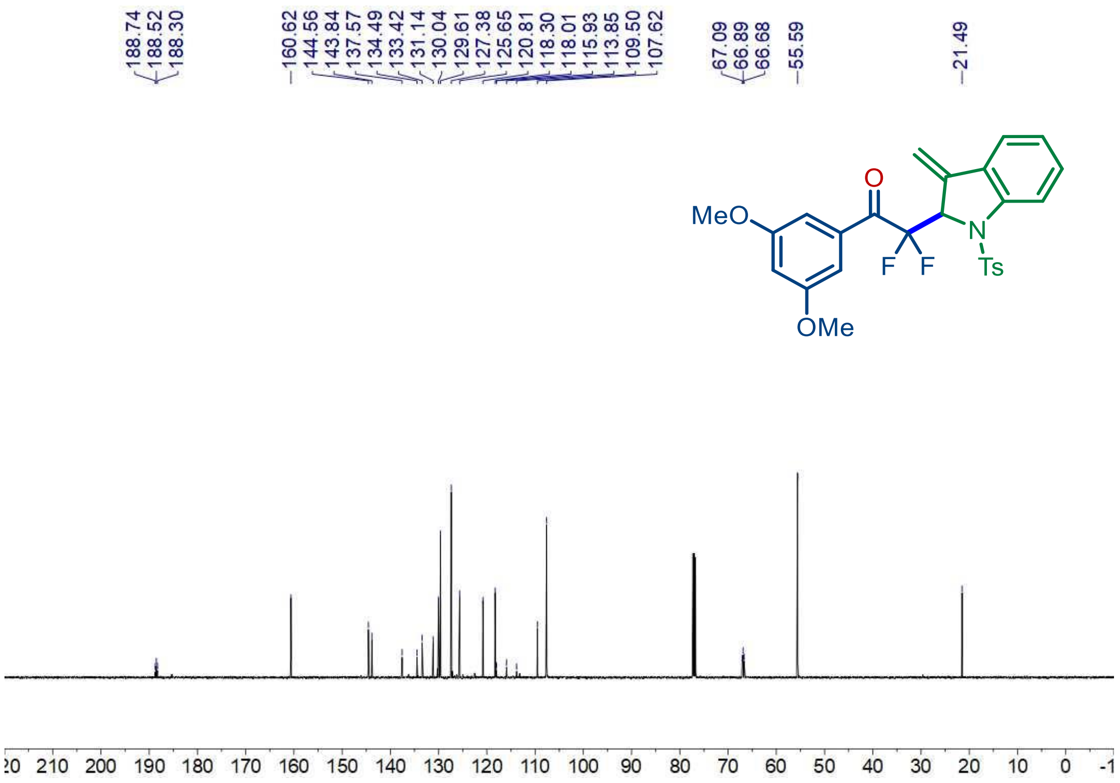

Supplementary Fig. 160 ^{13}C NMR (125 MHz, $CDCl_3$) spectrum of compound **53**

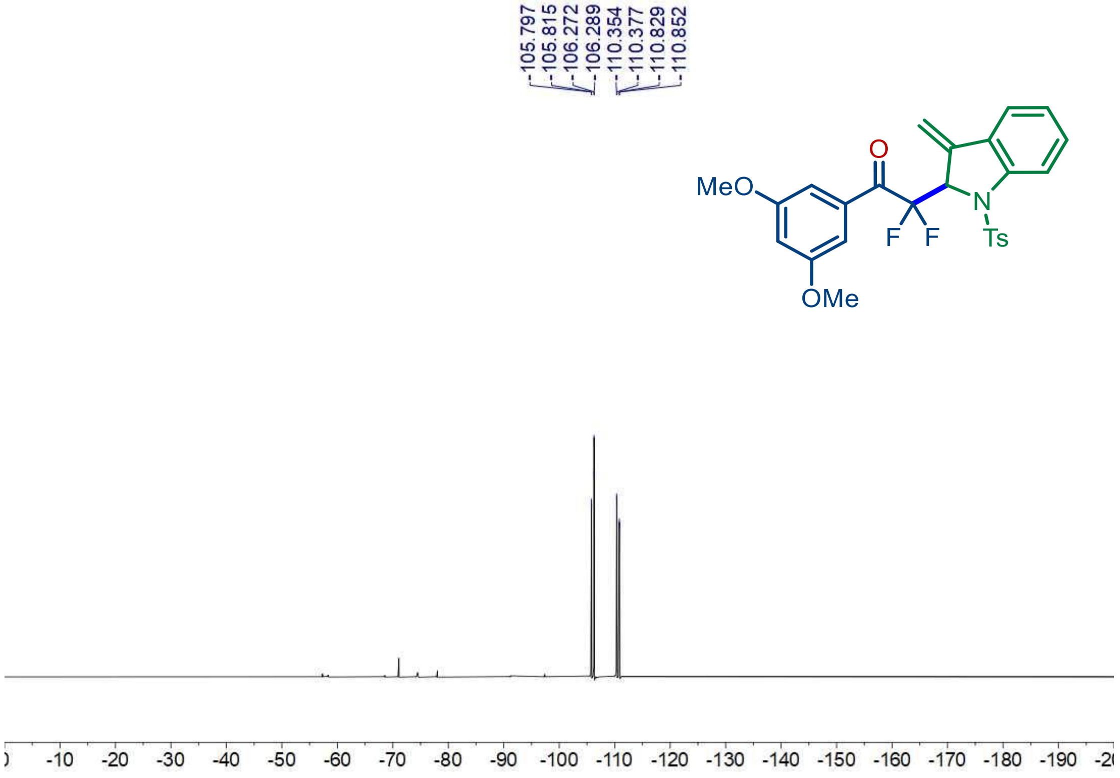

Supplementary Fig. 161 ^{19}F NMR (564 MHz, $CDCl_3$) spectrum of compound **53**

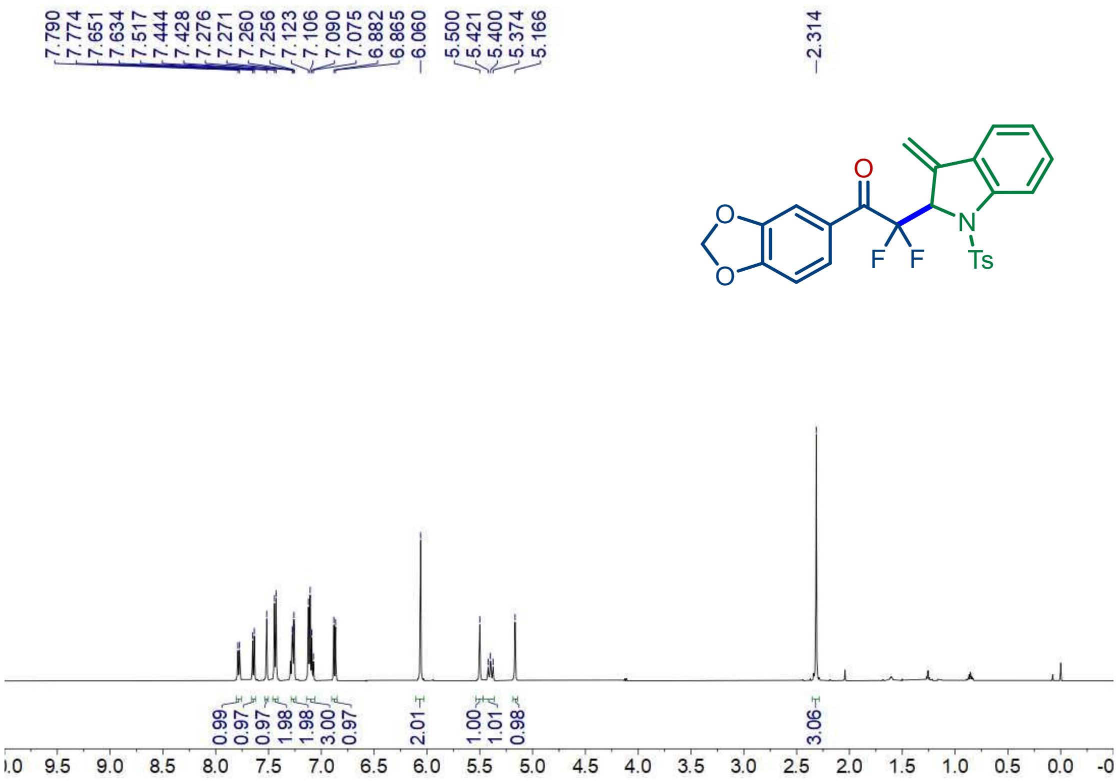

Supplementary Fig. 162 ^{1}H NMR (500 MHz, $CDCl_3$) spectrum of compound **54**

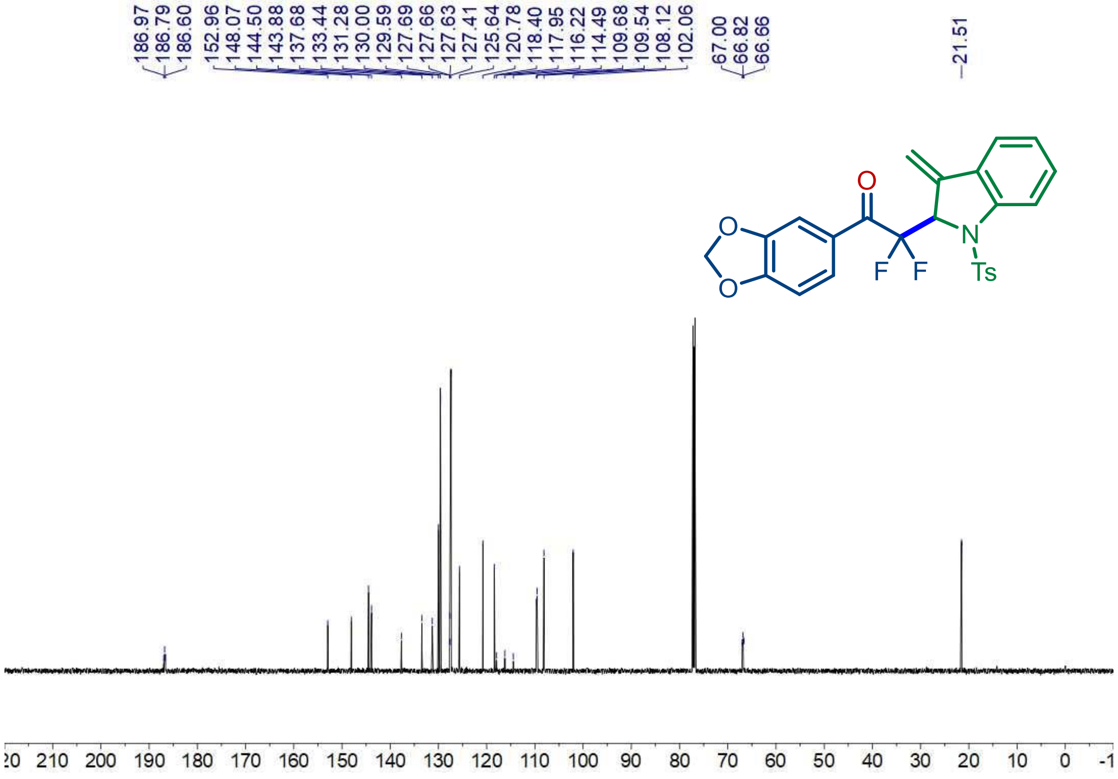

Supplementary Fig. 163 ^{13}C NMR (150 MHz, $CDCl_3$) spectrum of compound **54**

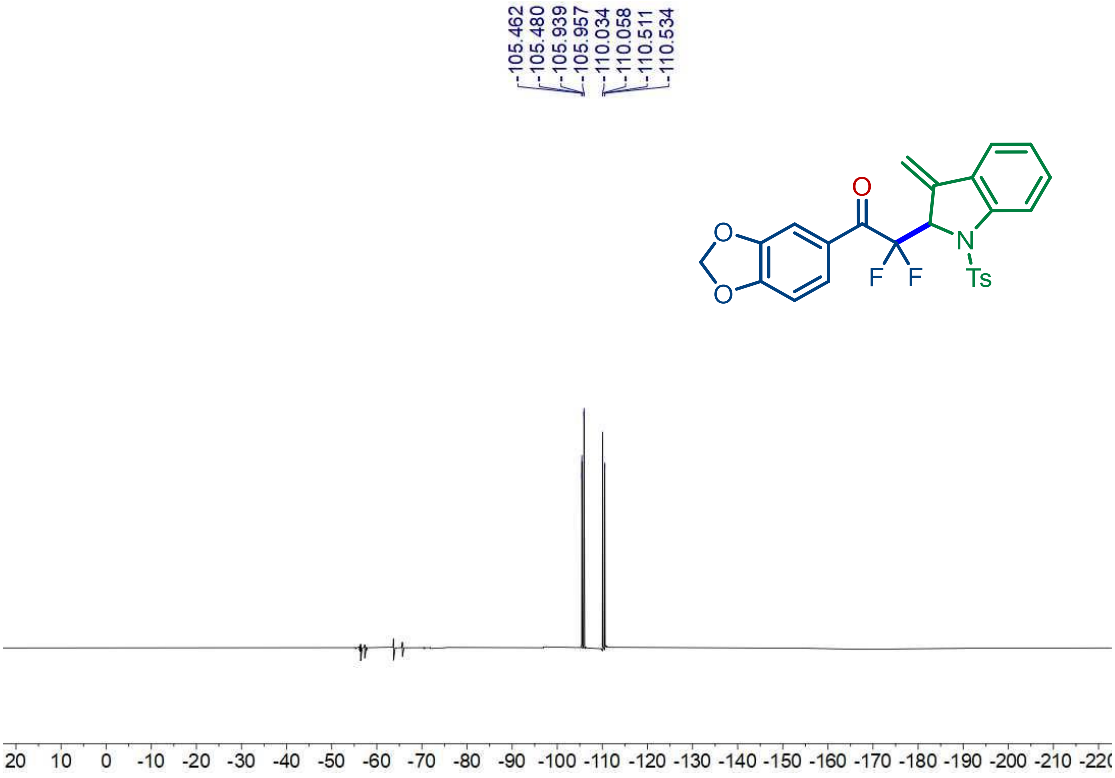

Supplementary Fig. 164 ^{19}F NMR (564 MHz, $CDCl_3$) spectrum of compound **54**

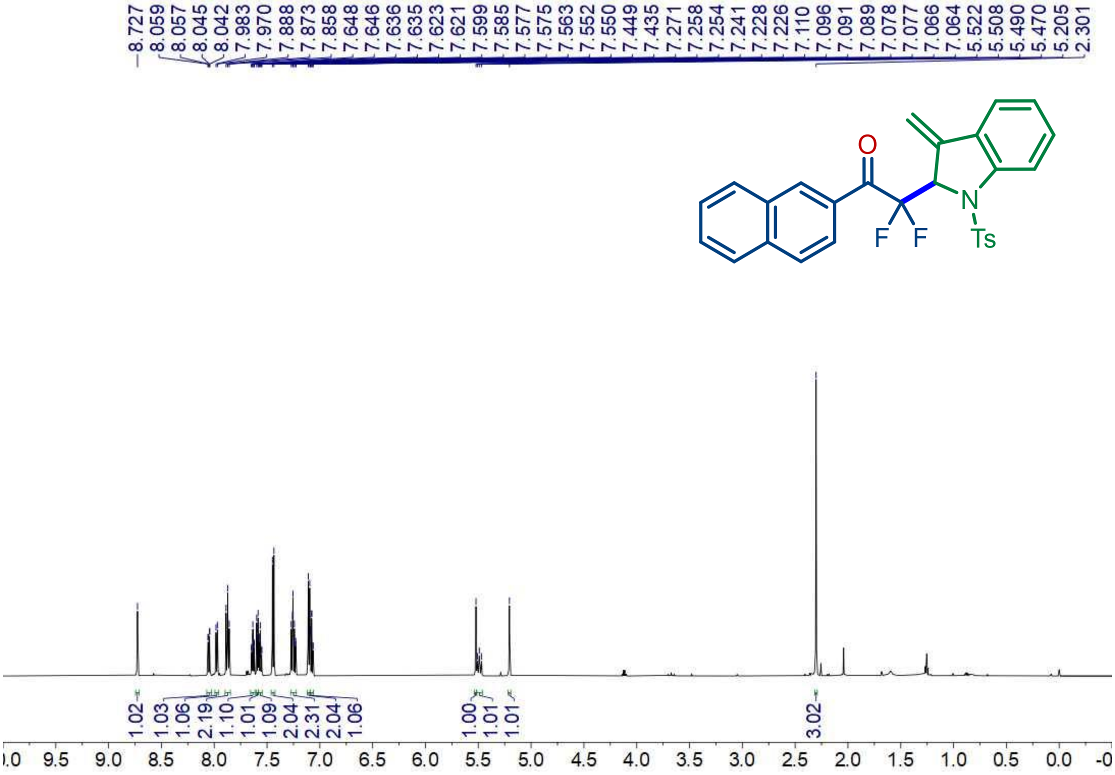

Supplementary Fig. 165 ^{1}H NMR (600 MHz, $CDCl_3$) spectrum of compound **55**

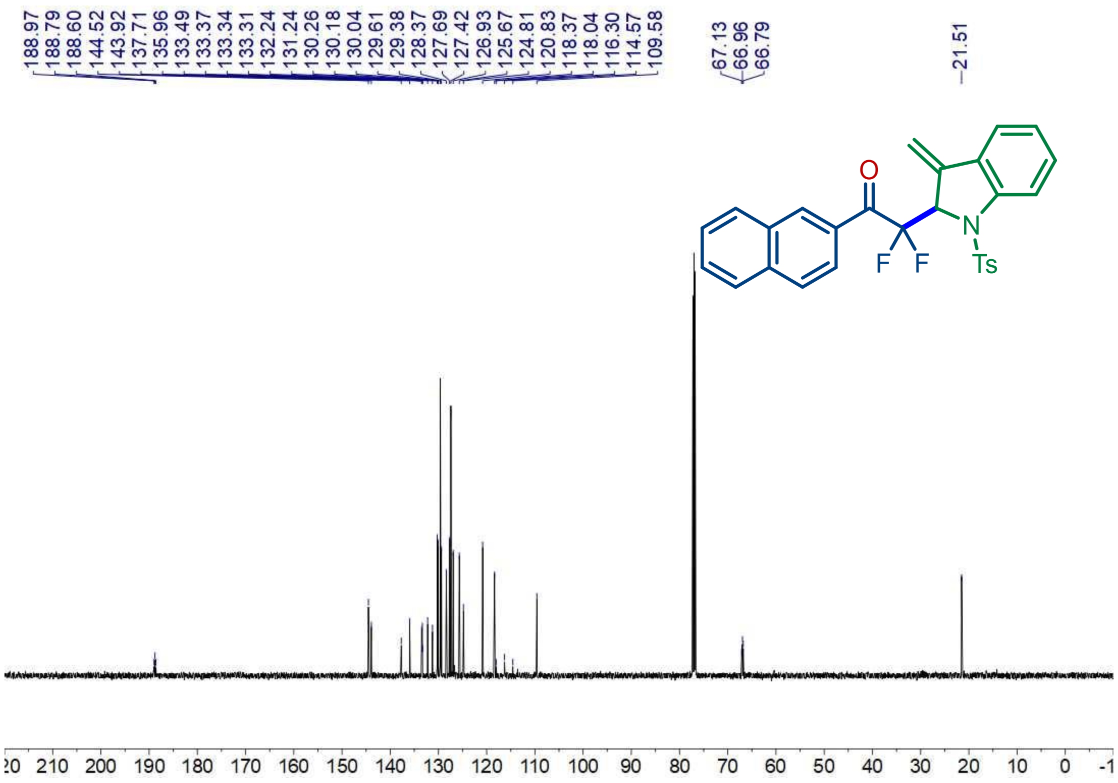

Supplementary Fig. 166 ^{13}C NMR (150 MHz, $CDCl_3$) spectrum of compound **55**

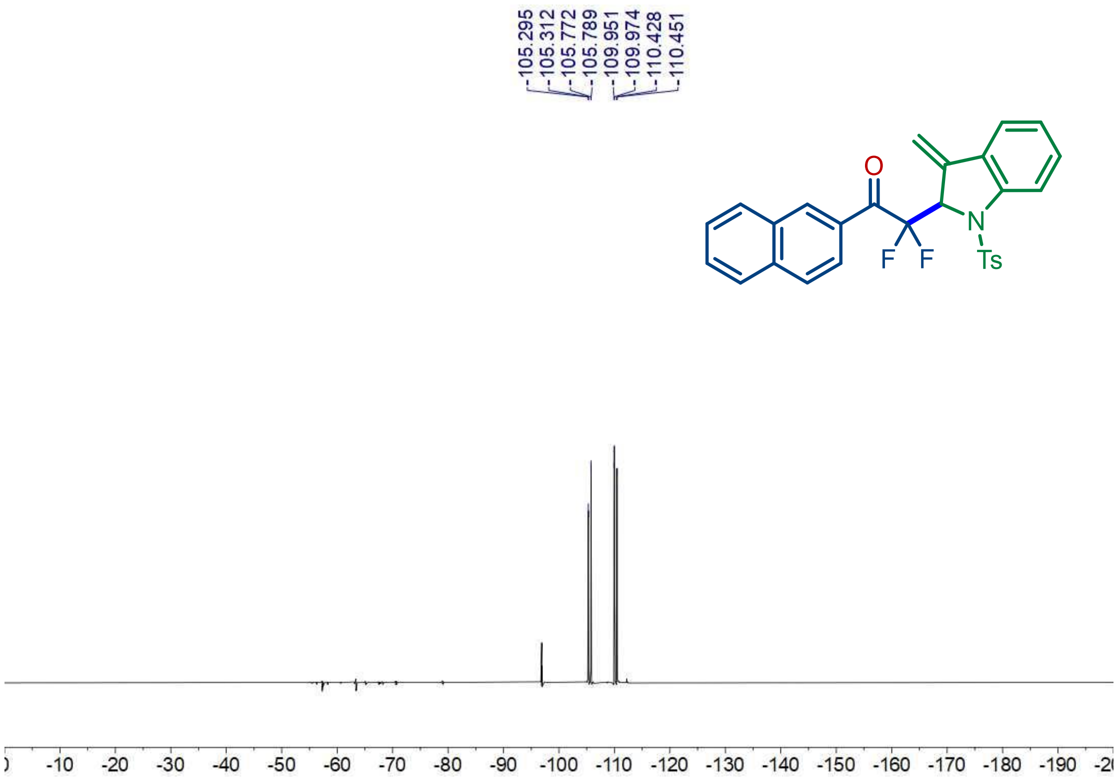

Supplementary Fig. 167 ^{19}F NMR (564 MHz, $CDCl_3$) spectrum of compound **55**

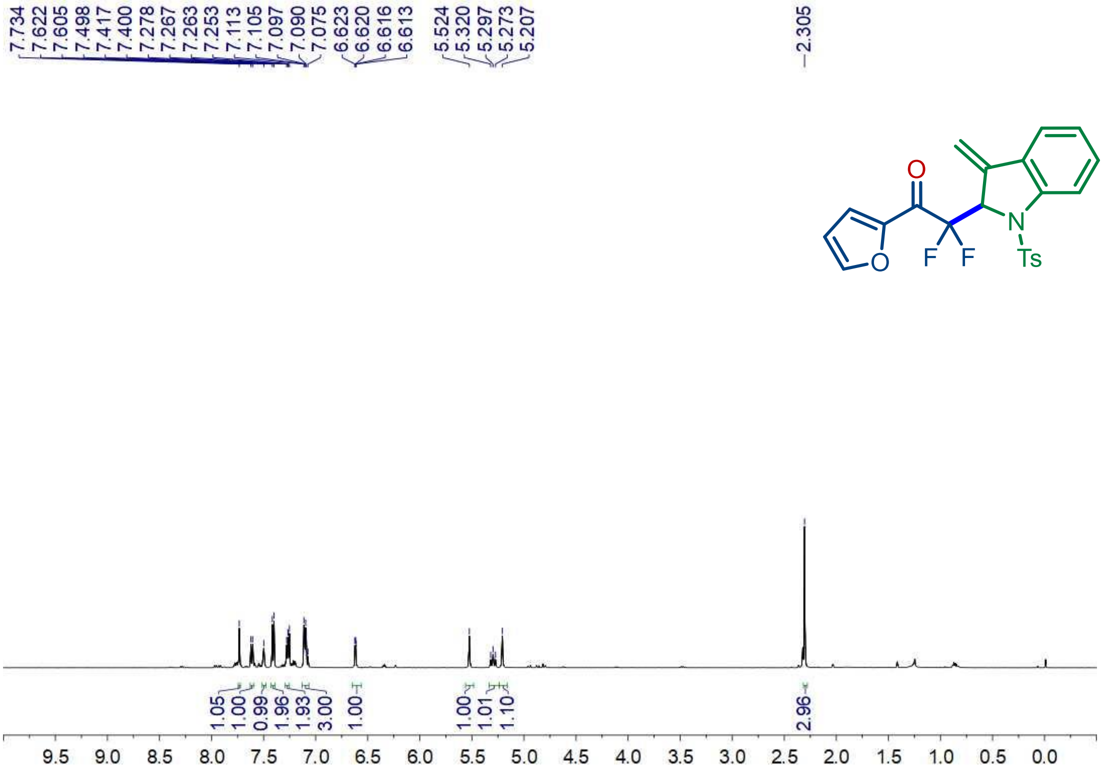

Supplementary Fig. 168 ^{1}H NMR (600 MHz, $CDCl_3$) spectrum of compound **56**

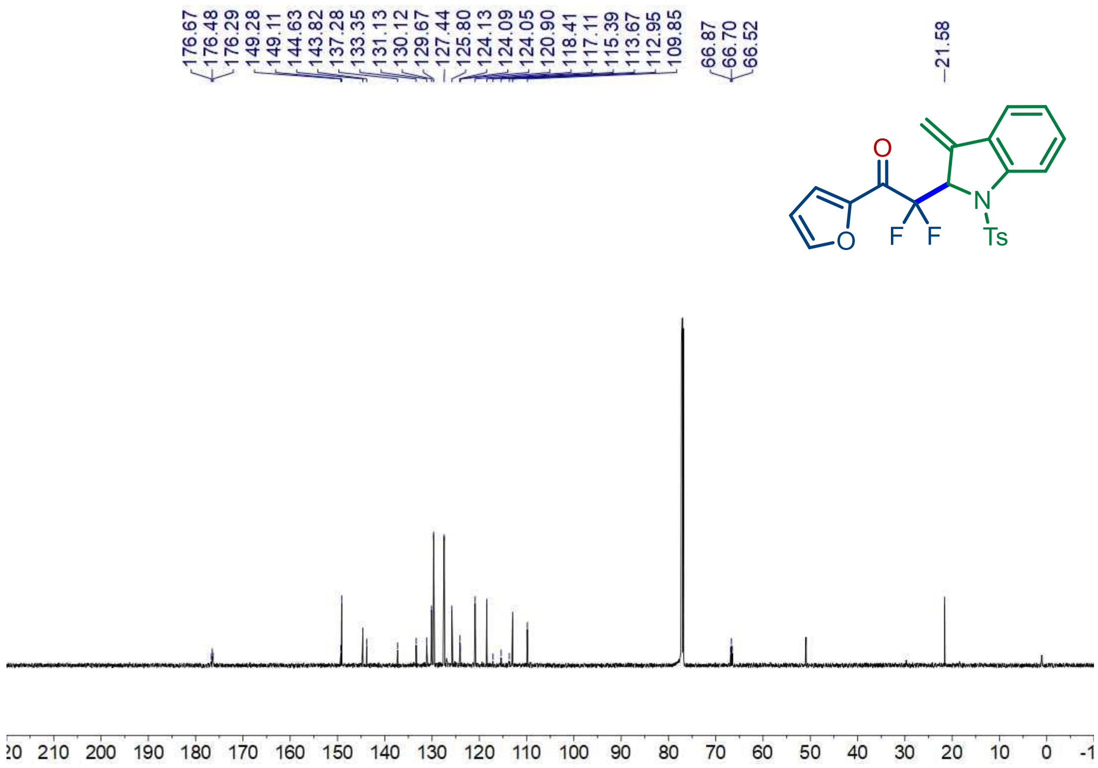

Supplementary Fig. 169 ^{13}C NMR (150 MHz, $CDCl_3$) spectrum of compound **56**

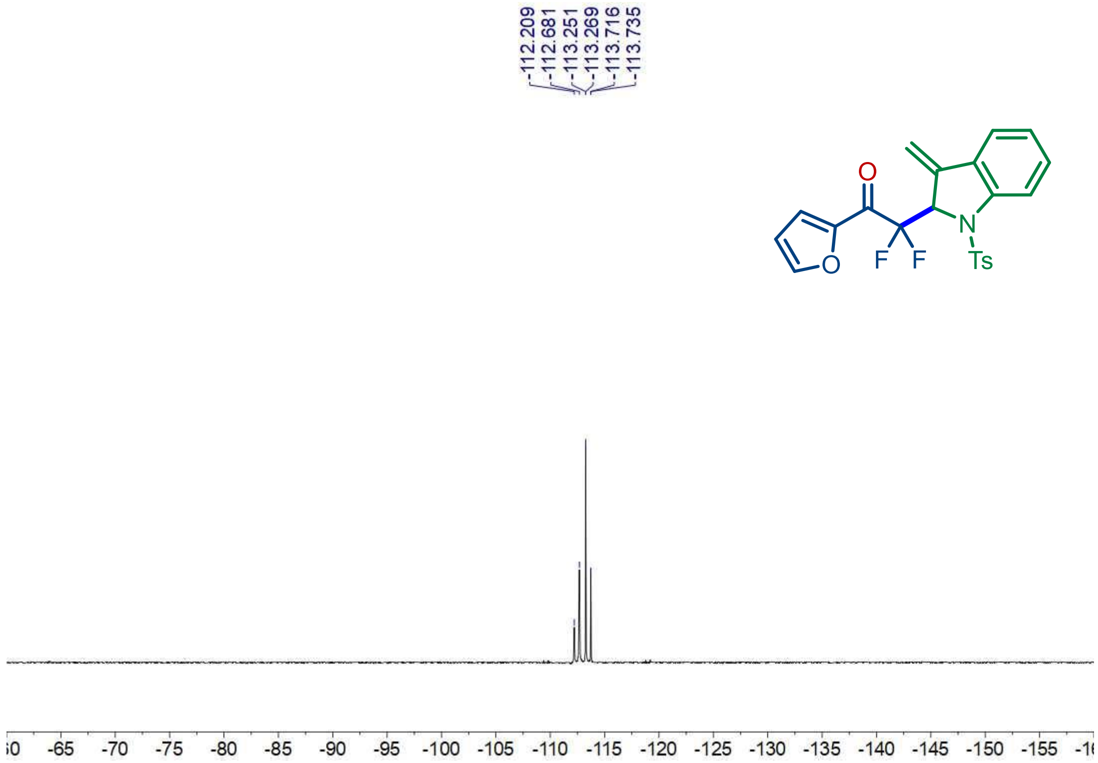

Supplementary Fig. 170 ^{19}F NMR (564 MHz, $CDCl_3$) spectrum of compound **56**

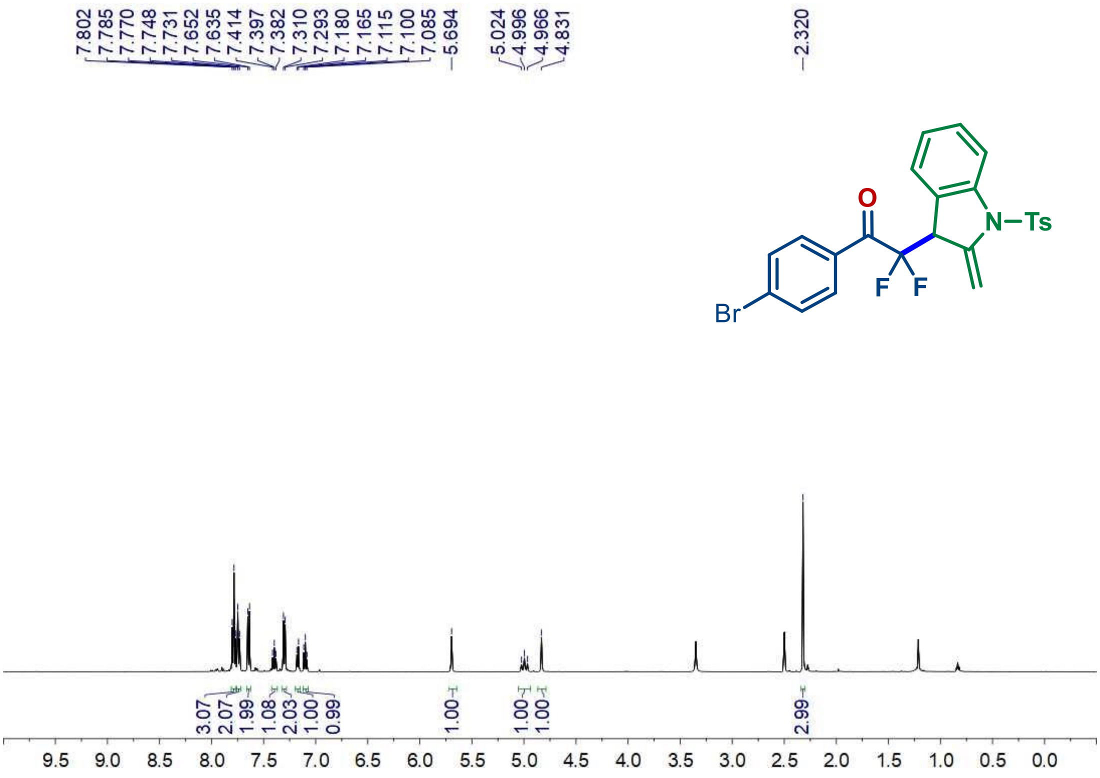

Supplementary Fig. 171 ^{1}H NMR (500 MHz, DMSO) spectrum of compound **57**

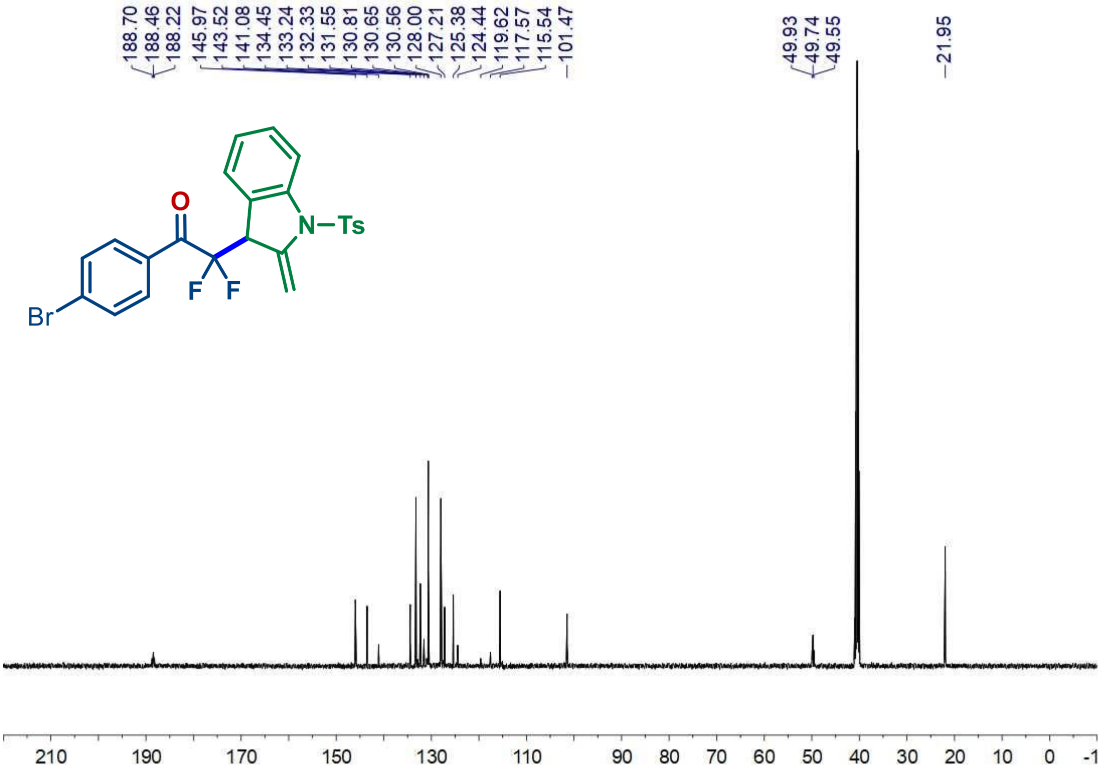

Supplementary Fig. 172 ^{13}C NMR (125 MHz, DMSO) spectrum of compound **57**

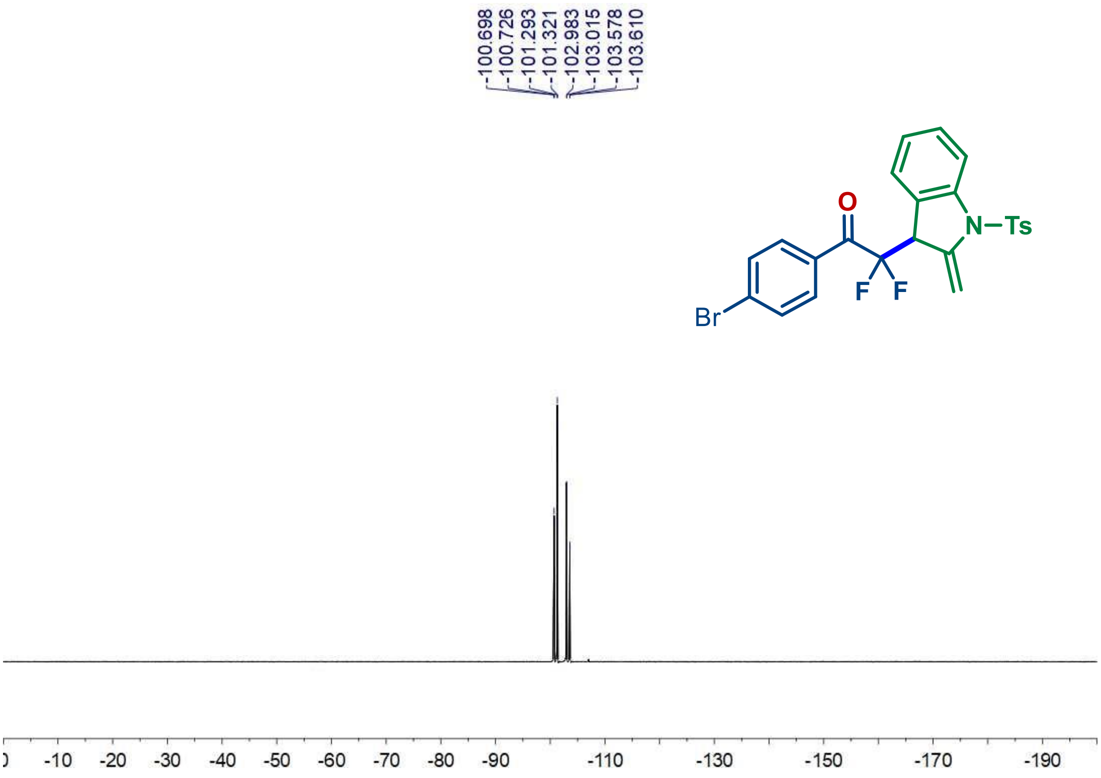

Supplementary Fig. 173 ^{19}F NMR (470 MHz, DMSO) spectrum of compound **57**

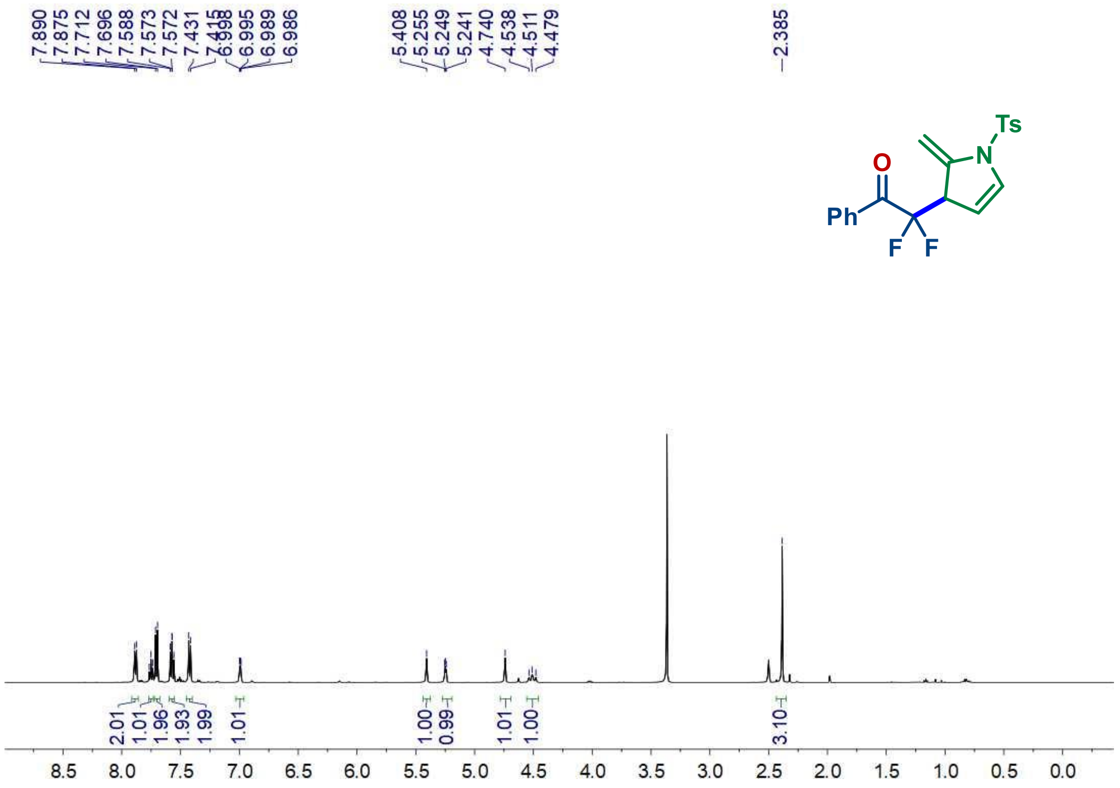

Supplementary Fig. 174 ^{1}H NMR (500 MHz, DMSO) spectrum of compound **58**

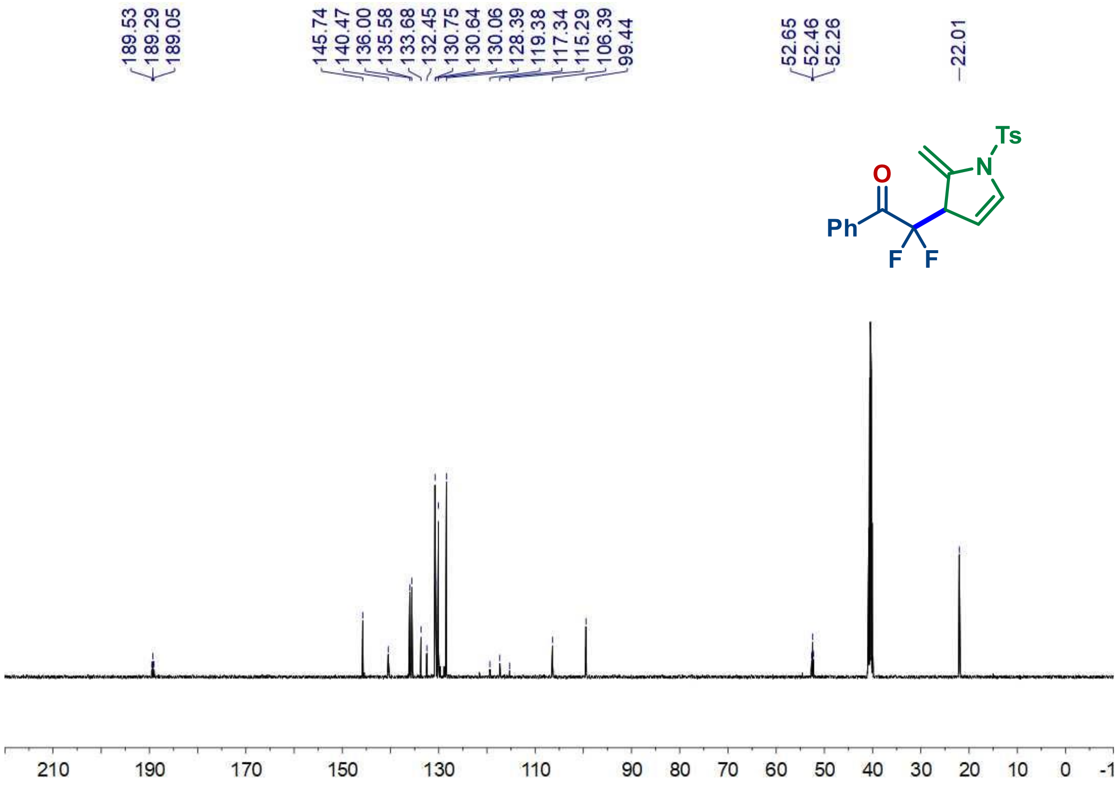

Supplementary Fig. 175 ^{13}C NMR (125 MHz, DMSO) spectrum of compound **58**

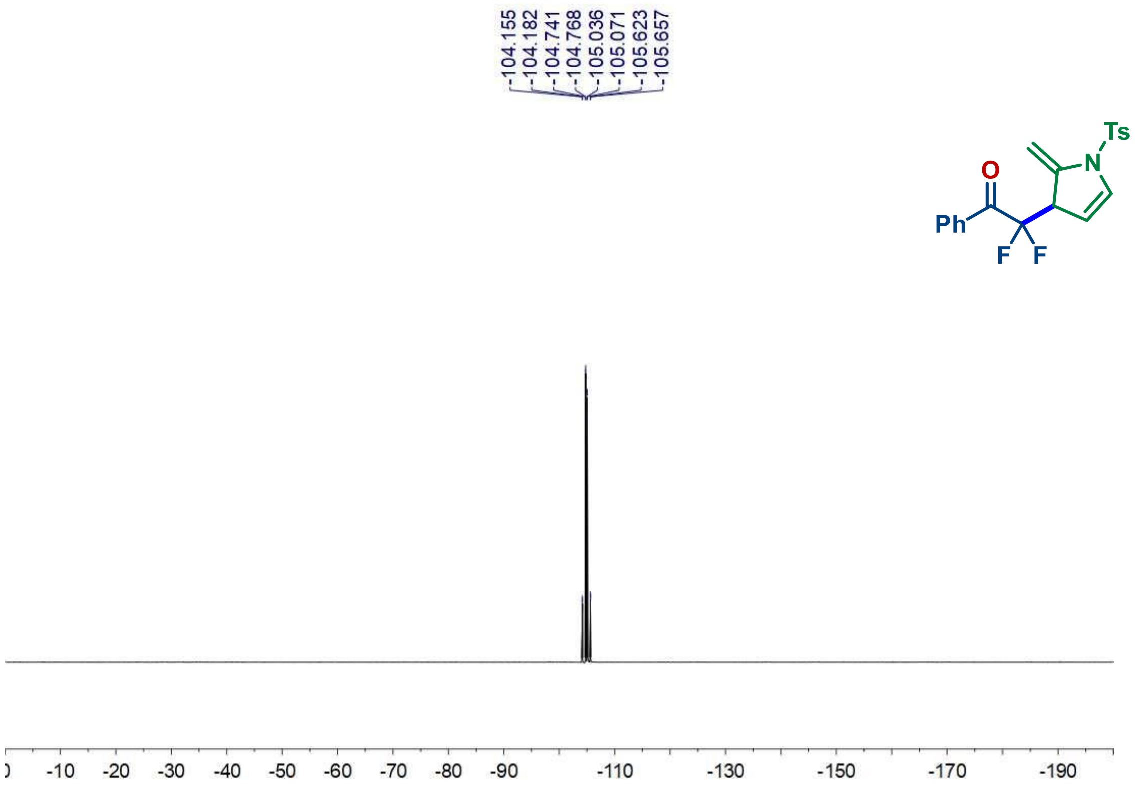

Supplementary Fig. 176 ^{19}F NMR (470 MHz, DMSO) spectrum of compound **58**

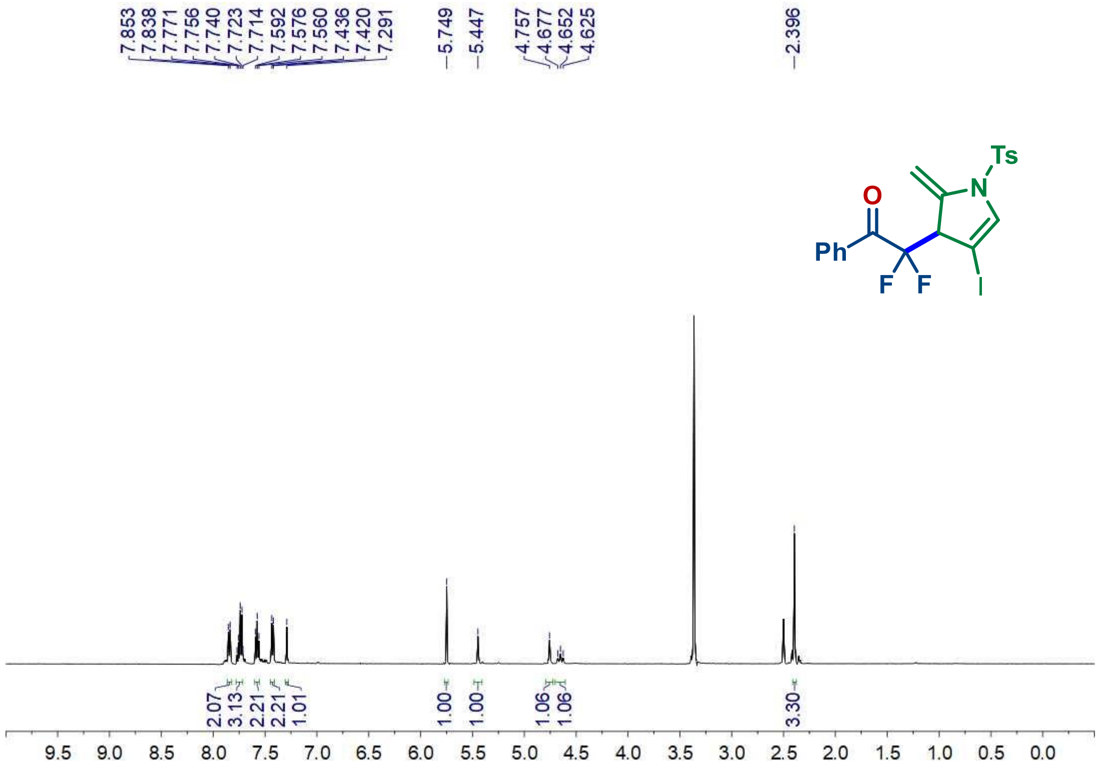

Supplementary Fig. 177 ^{1}H NMR (500 MHz, DMSO) spectrum of compound **59**

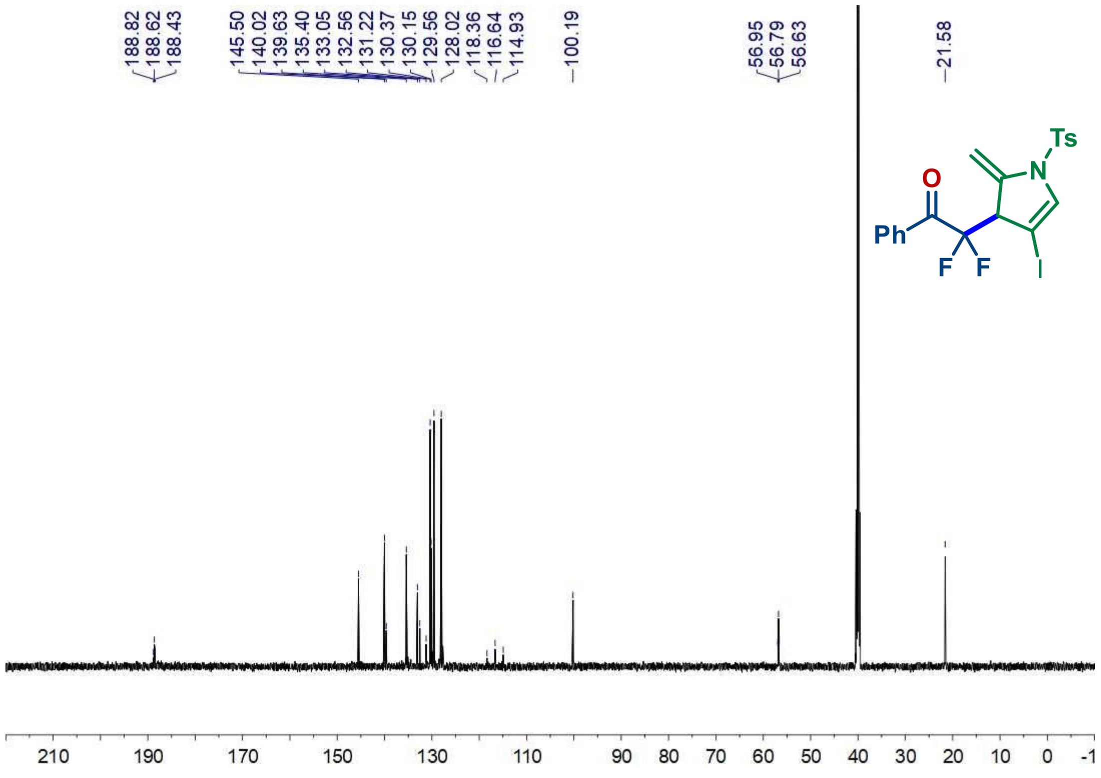

Supplementary Fig. 178 ^{13}C NMR (150 MHz, DMSO) spectrum of compound **59**

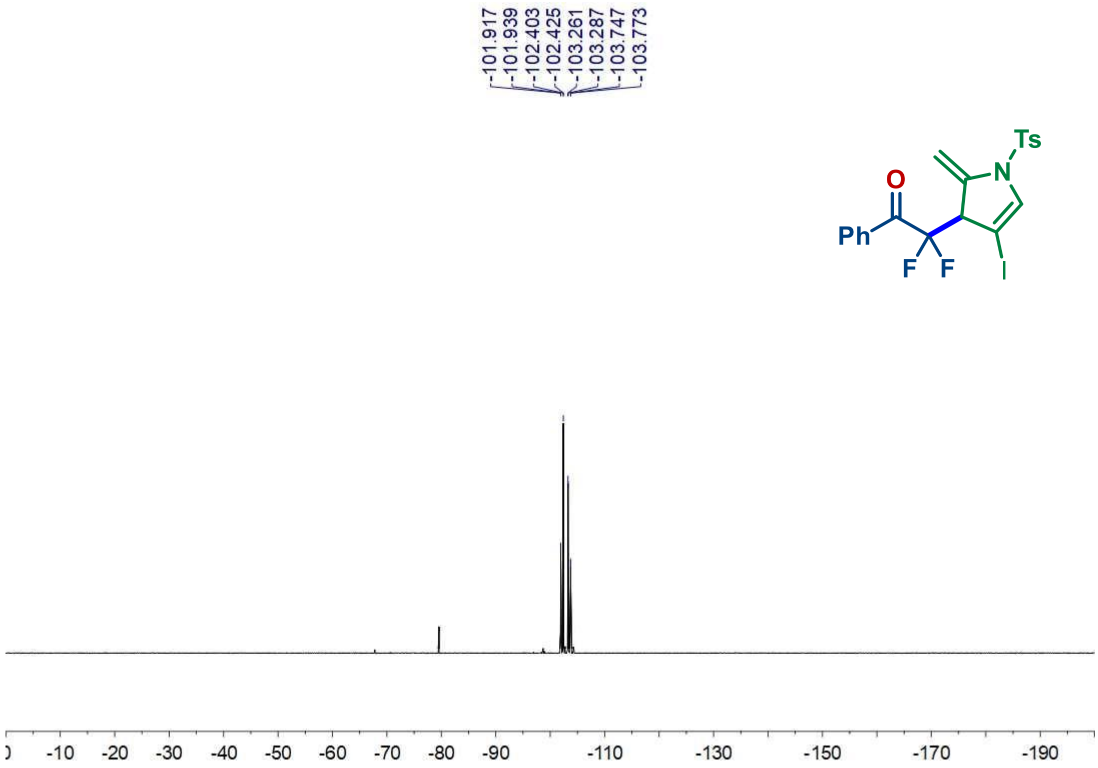

Supplementary Fig. 179 ^{19}F NMR (564 MHz, DMSO) spectrum of compound **59**

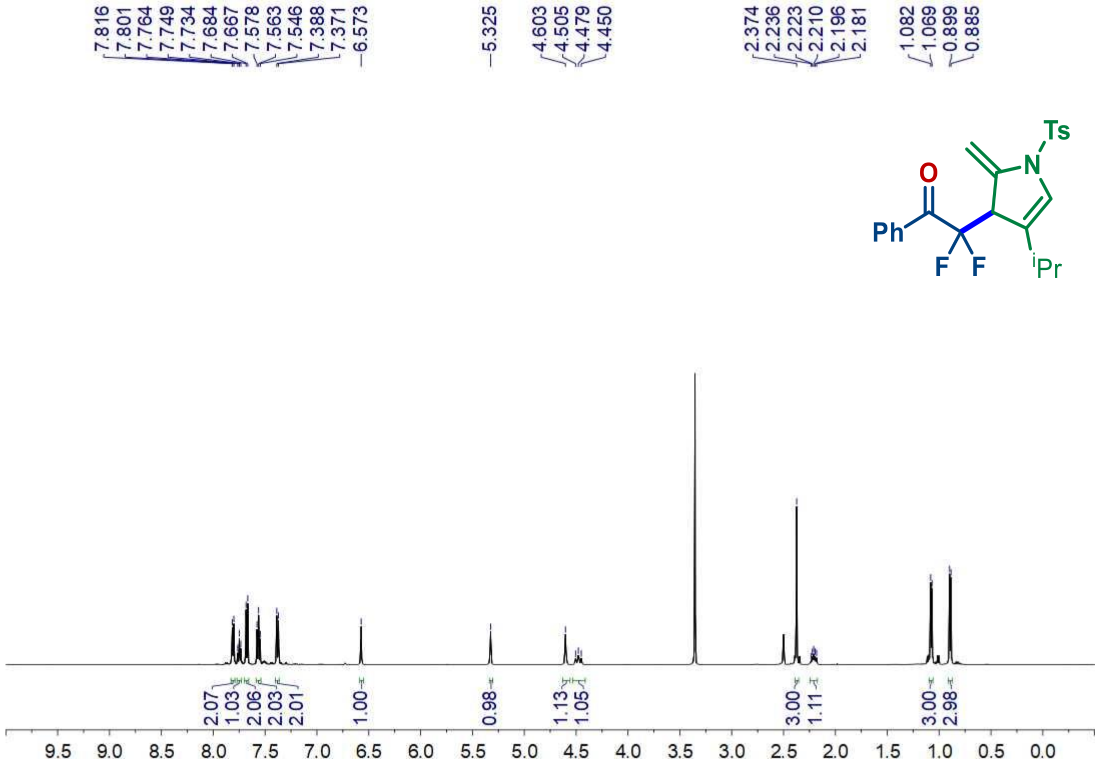

Supplementary Fig. 180 ^{1}H NMR (500 MHz, DMSO) spectrum of compound **60**

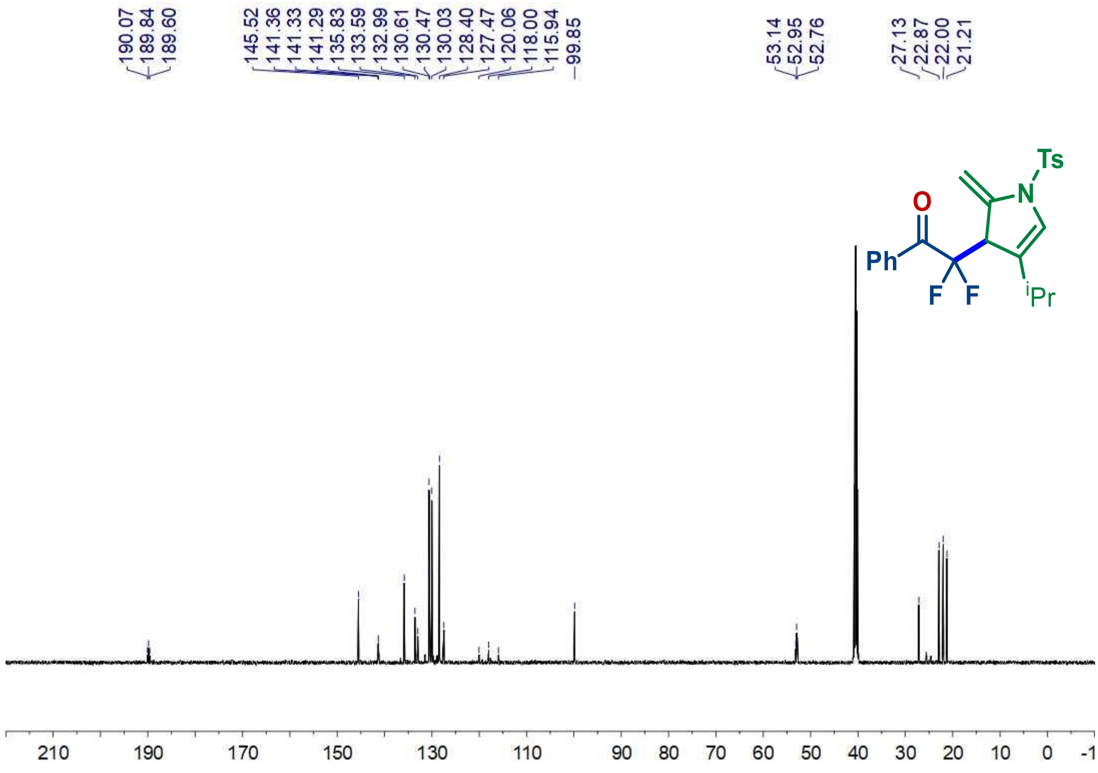

Supplementary Fig. 181 ^{13}C NMR (125 MHz, DMSO) spectrum of compound **60**

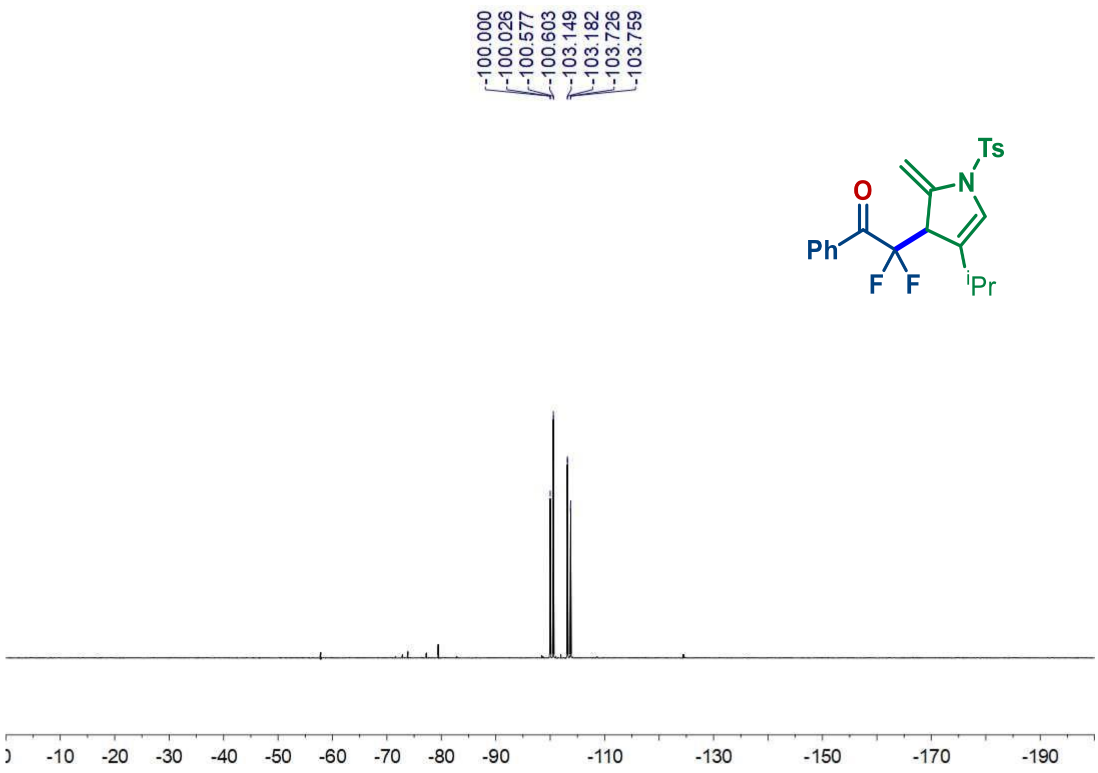

Supplementary Fig. 182 ^{19}F NMR (470 MHz, DMSO) spectrum of compound **60**

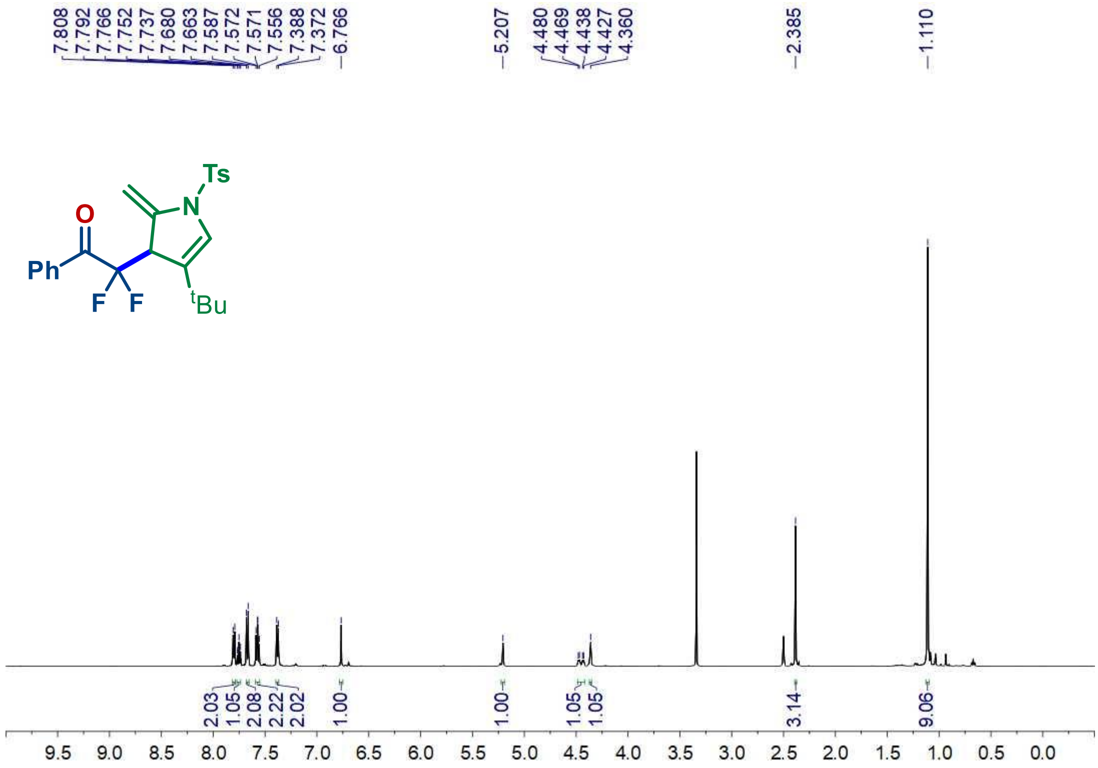

Supplementary Fig. 183 ^{1}H NMR (500 MHz, DMSO) spectrum of compound **61**

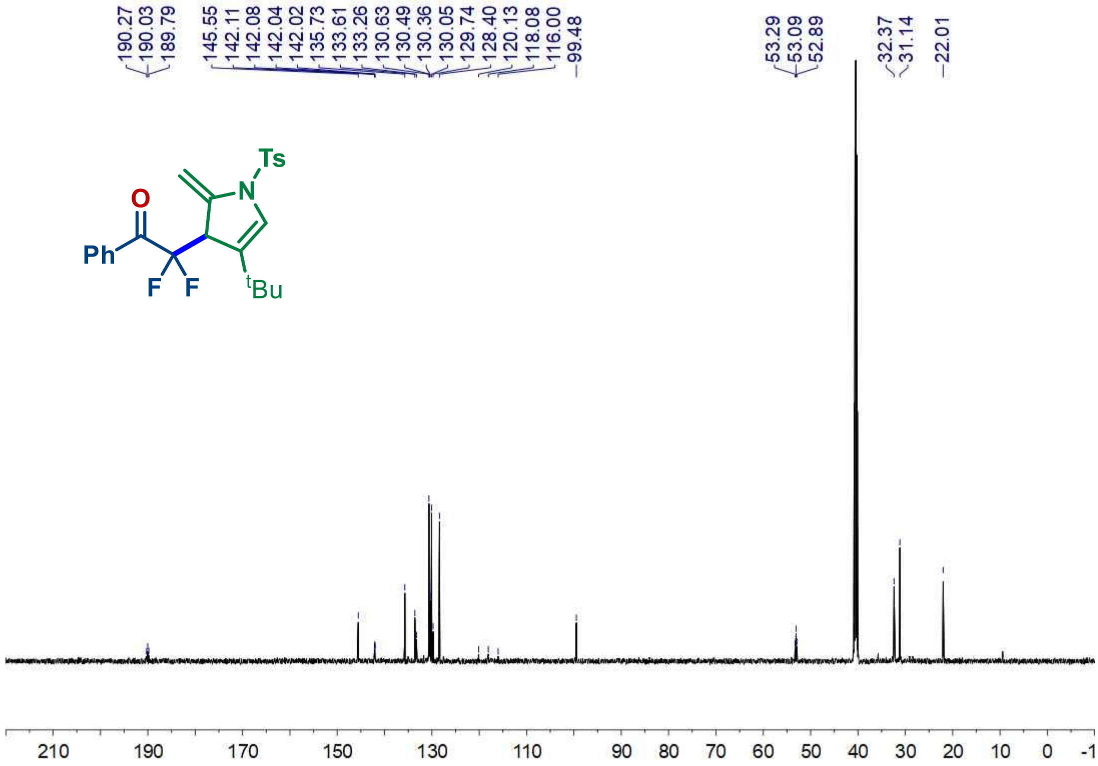

Supplementary Fig. 184 ^{13}C NMR (125 MHz, DMSO) spectrum of compound **61**

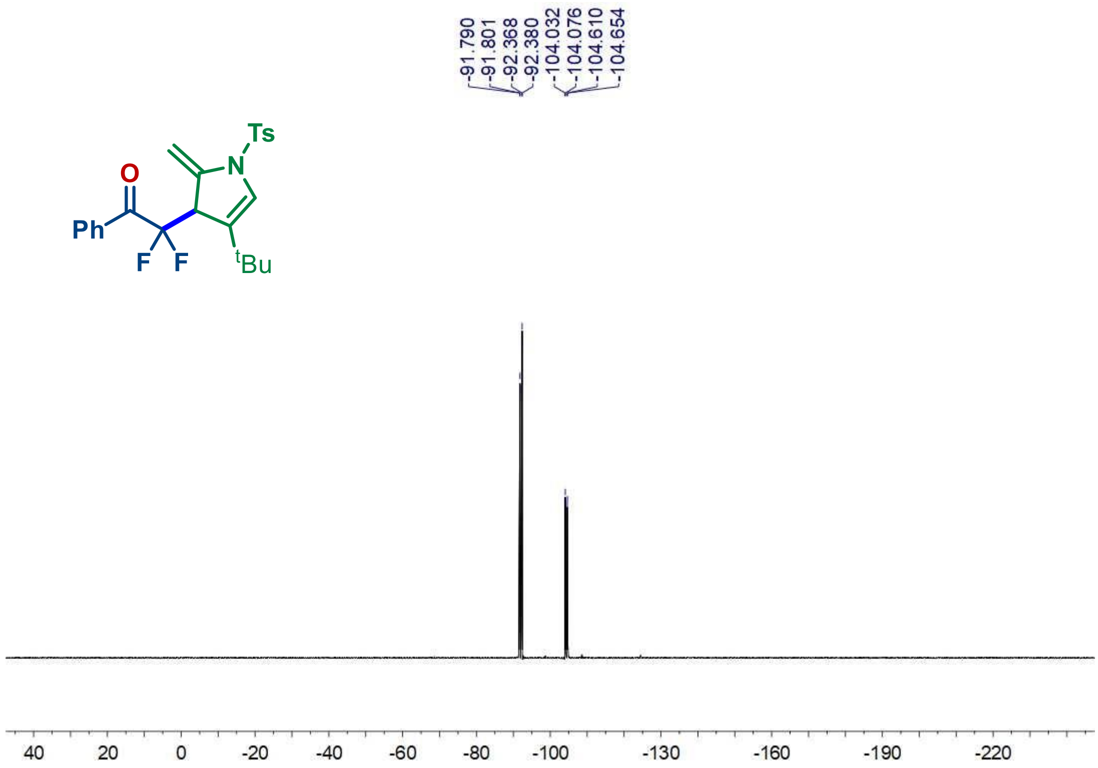

Supplementary Fig. 185 ^{19}F NMR (470 MHz, DMSO) spectrum of compound **61**

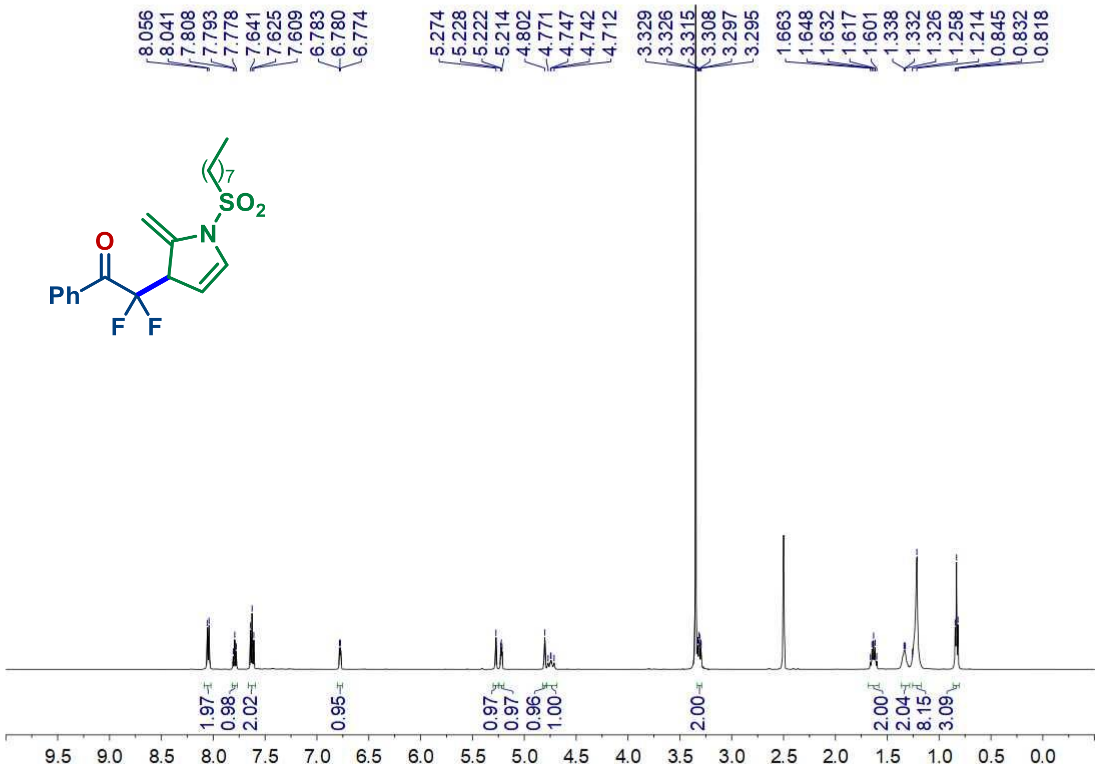

Supplementary Fig. 186 ^{1}H NMR (500 MHz, DMSO) spectrum of compound **63**

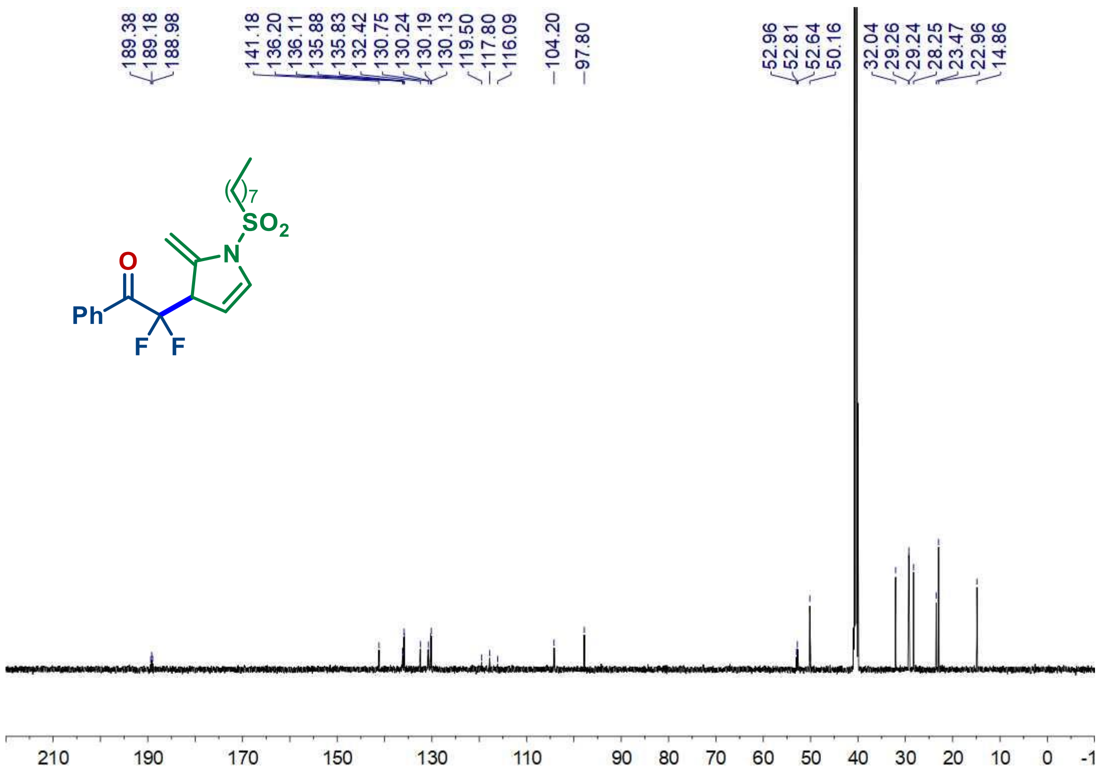

Supplementary Fig. 187 ^{13}C NMR (150 MHz, DMSO) spectrum of compound **63**

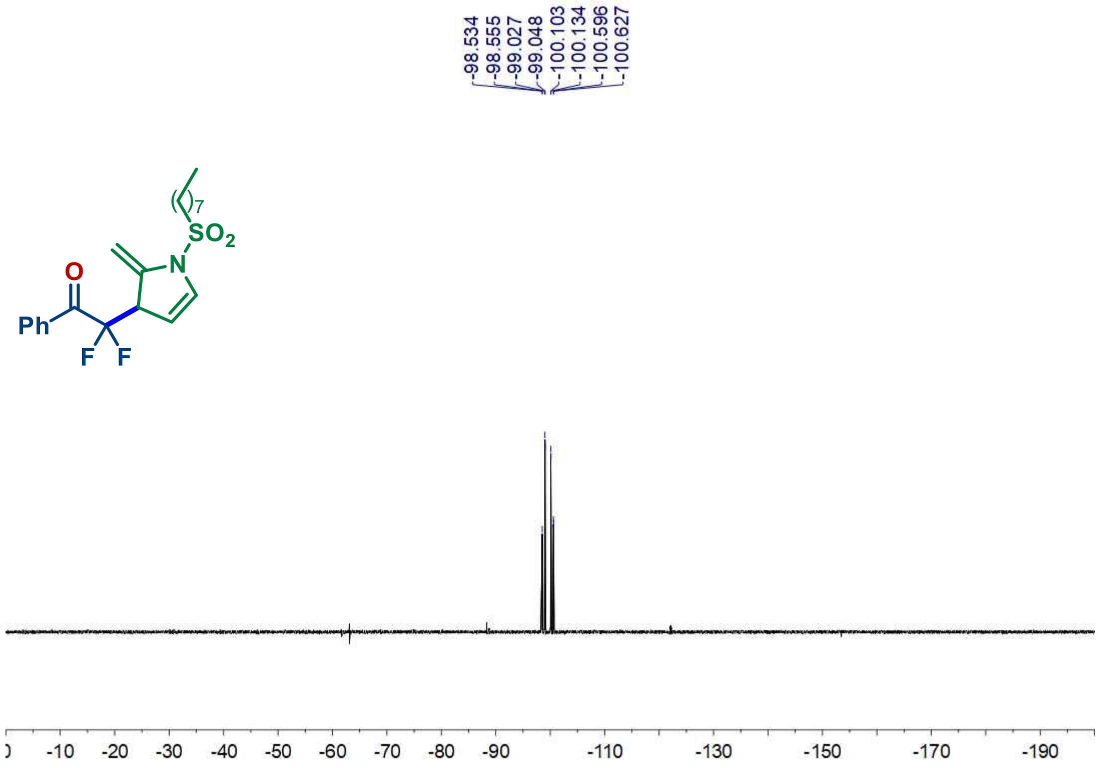

Supplementary Fig. 188 ^{19}F NMR (564 MHz, DMSO) spectrum of compound **63**

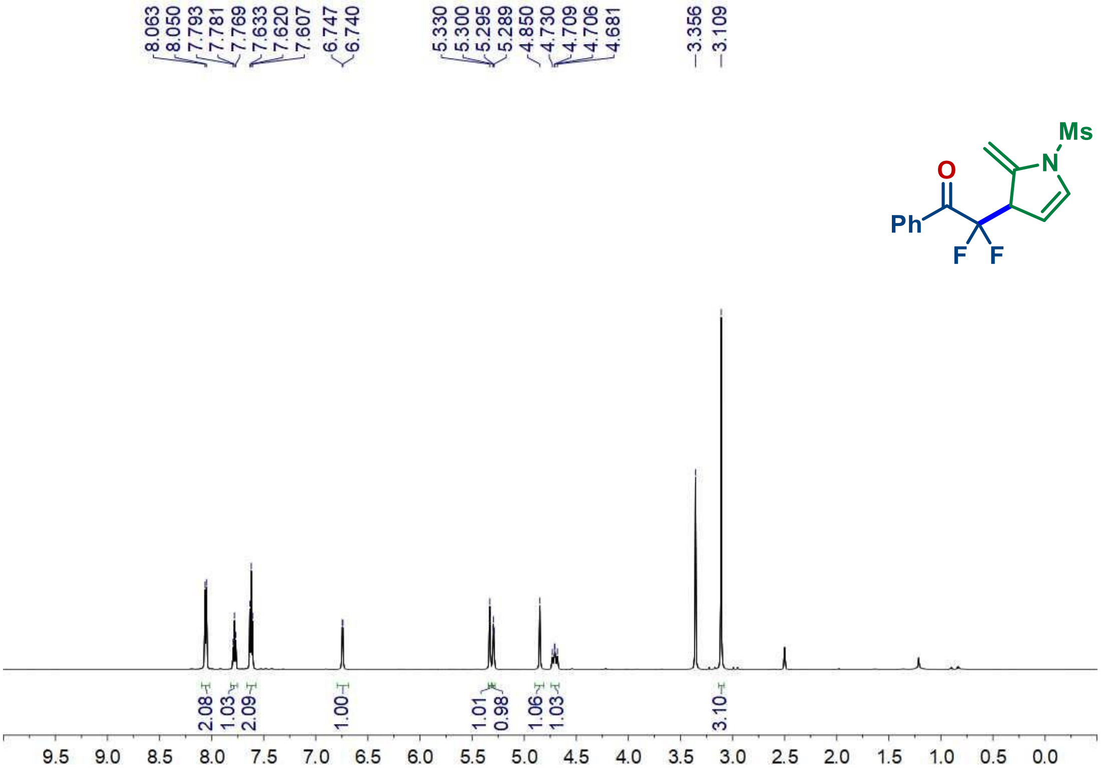

Supplementary Fig. 189 ^{1}H NMR (600 MHz, DMSO) spectrum of compound **62**

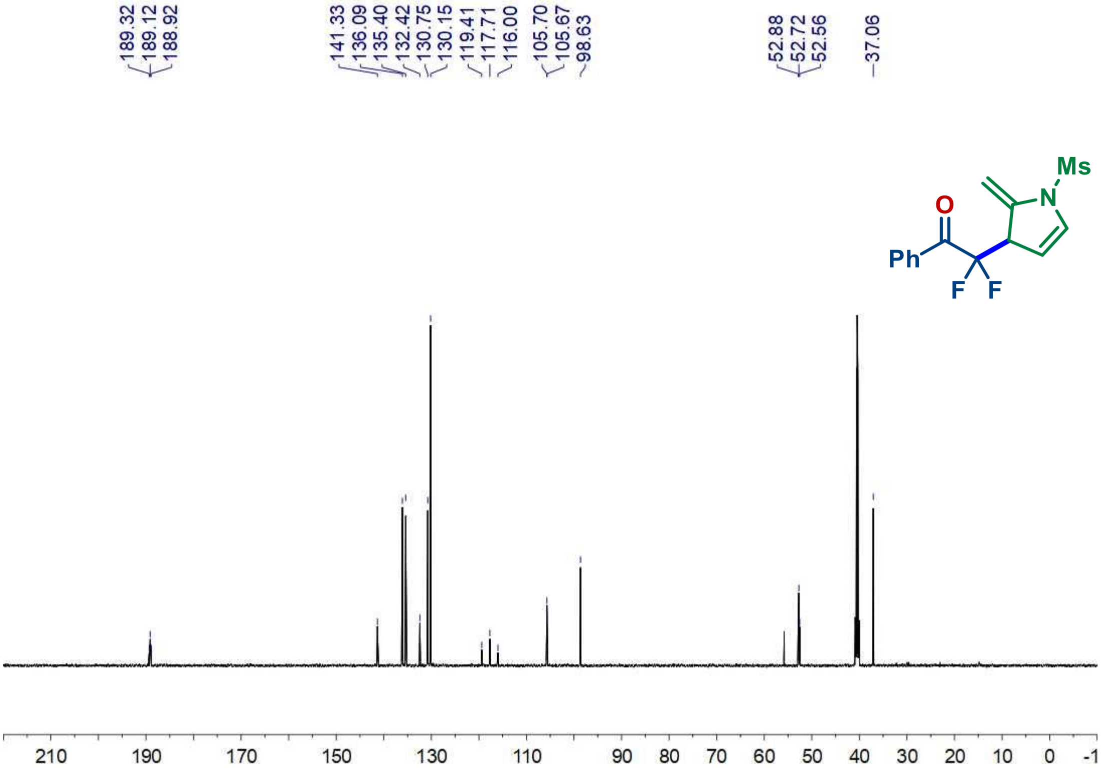

Supplementary Fig. 190 ^{13}C NMR (150 MHz, DMSO) spectrum of compound **62**

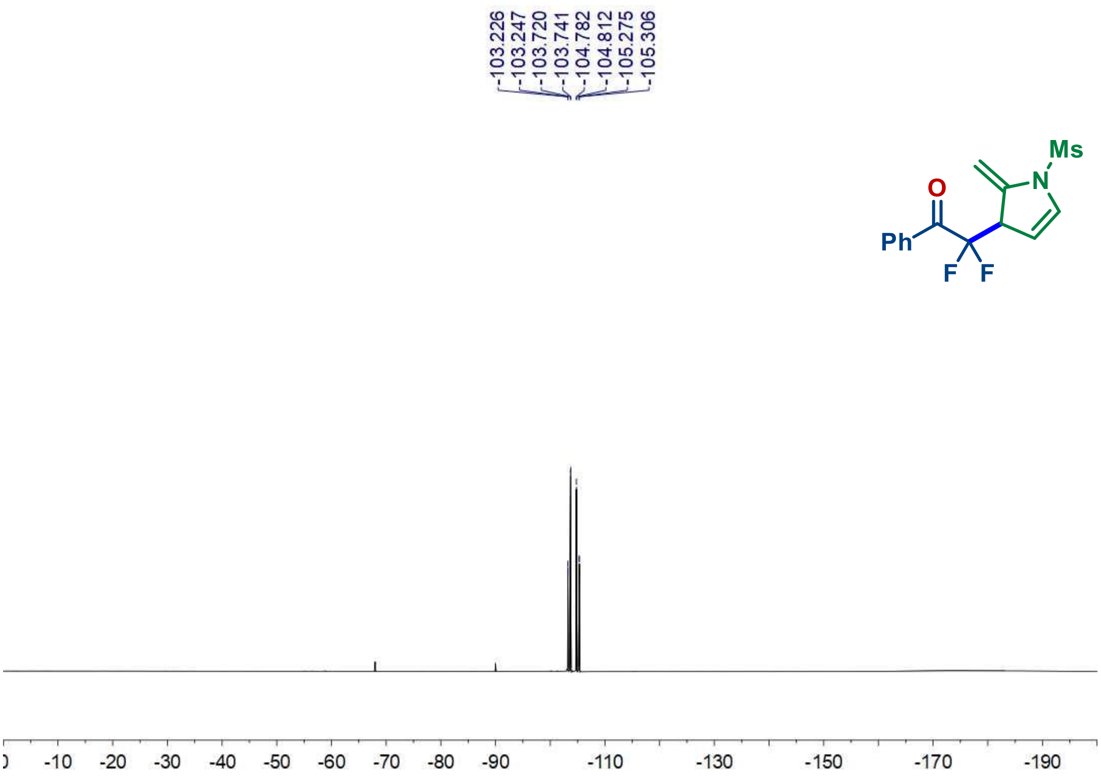

Supplementary Fig. 191 ^{19}F NMR (564 MHz, DMSO) spectrum of compound **62**

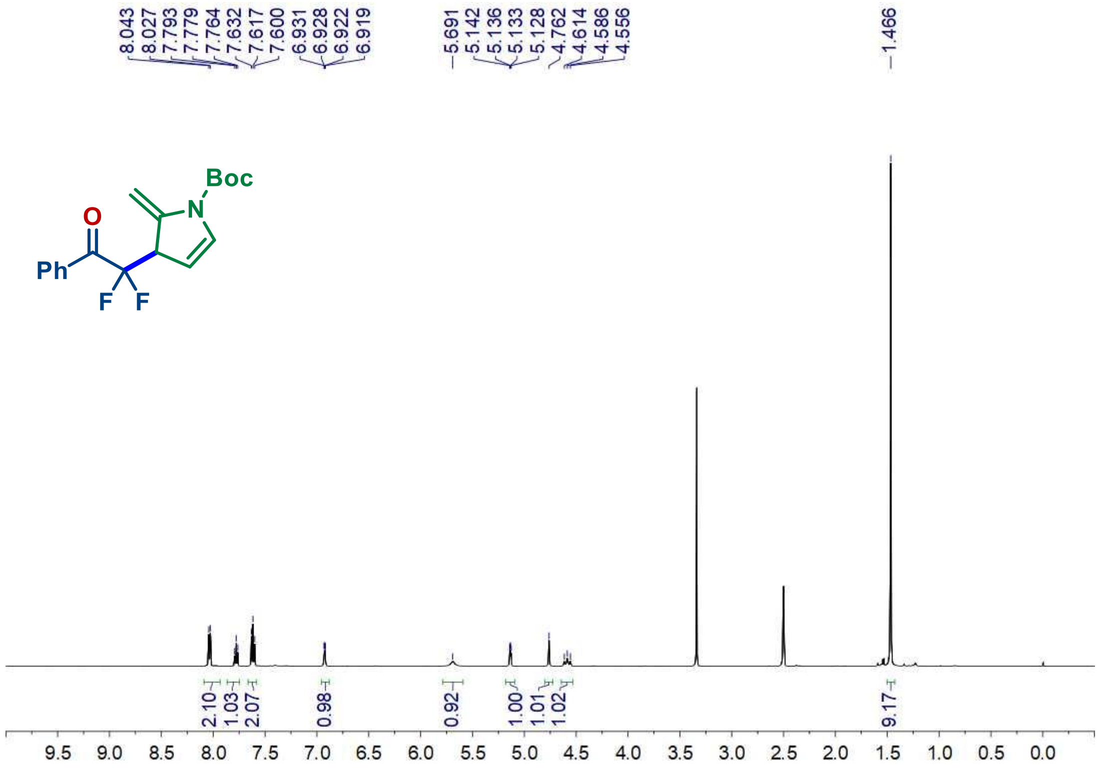

Supplementary Fig. 192 ^{1}H NMR (500 MHz, DMSO) spectrum of compound **64**

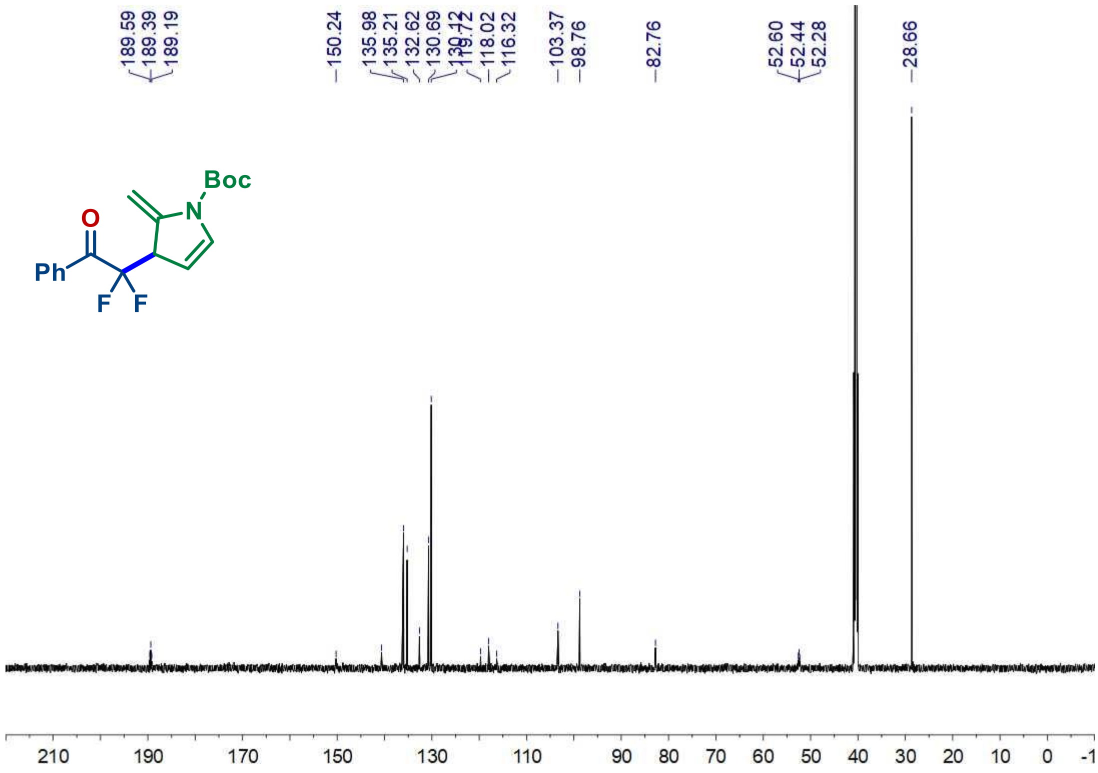

Supplementary Fig. 193 ^{13}C NMR (150 MHz, DMSO) spectrum of compound **64**

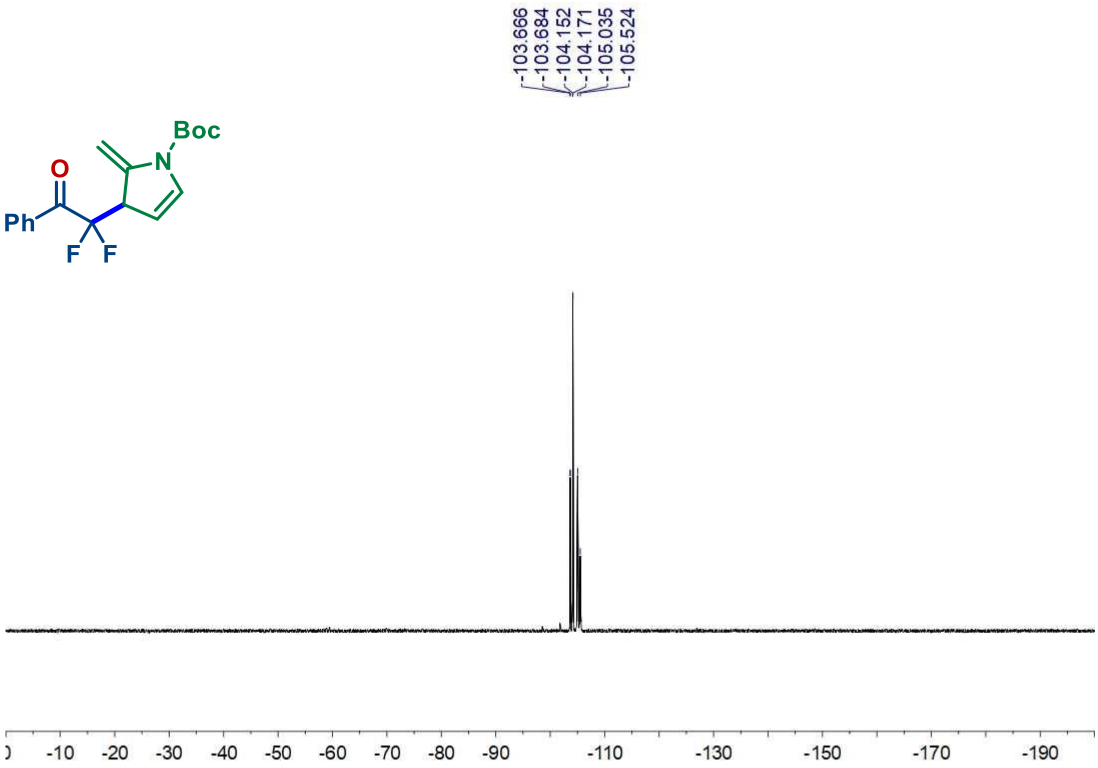

Supplementary Fig. 194 ^{19}F NMR (564 MHz, DMSO) spectrum of compound **64**

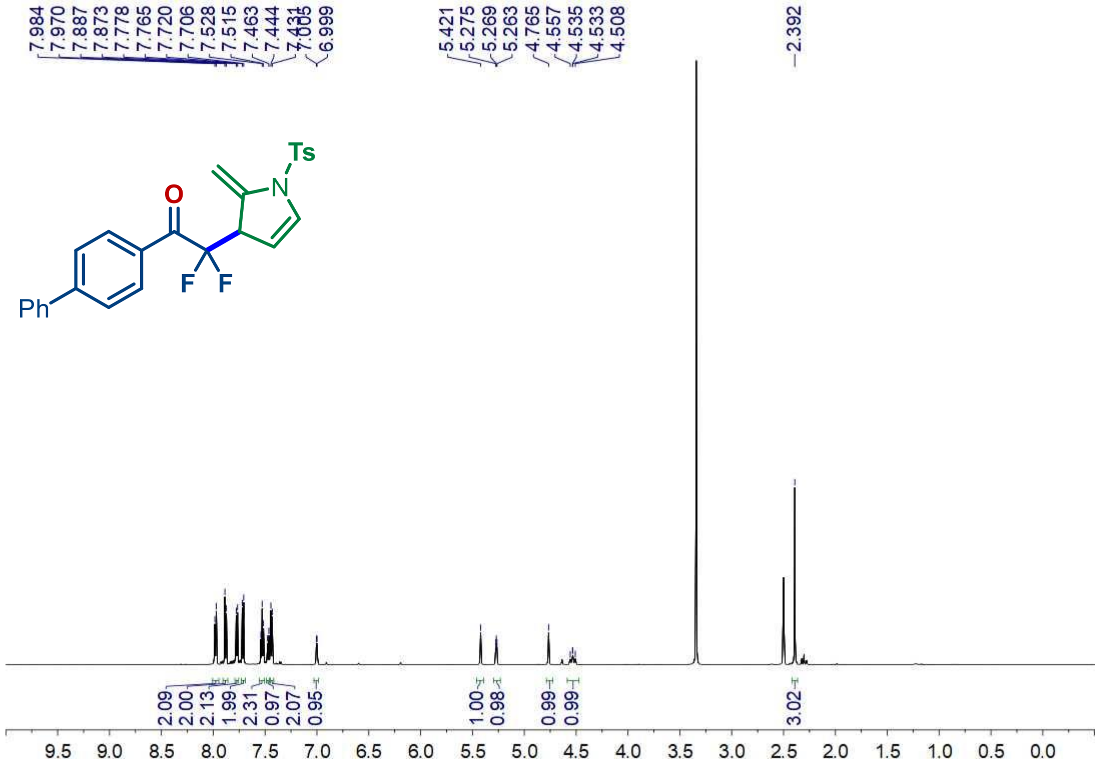

Supplementary Fig. 195 ^{1}H NMR (600 MHz, DMSO) spectrum of compound **65**

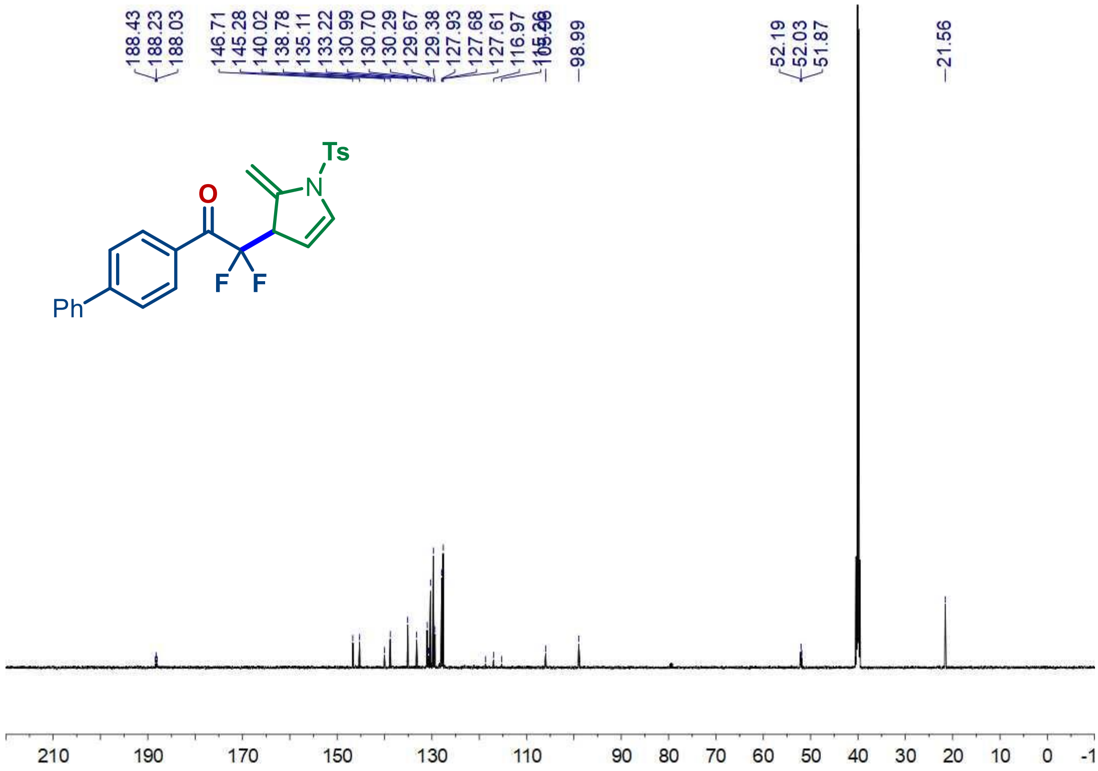

Supplementary Fig. 196 ^{13}C NMR (150 MHz, DMSO) spectrum of compound **65**

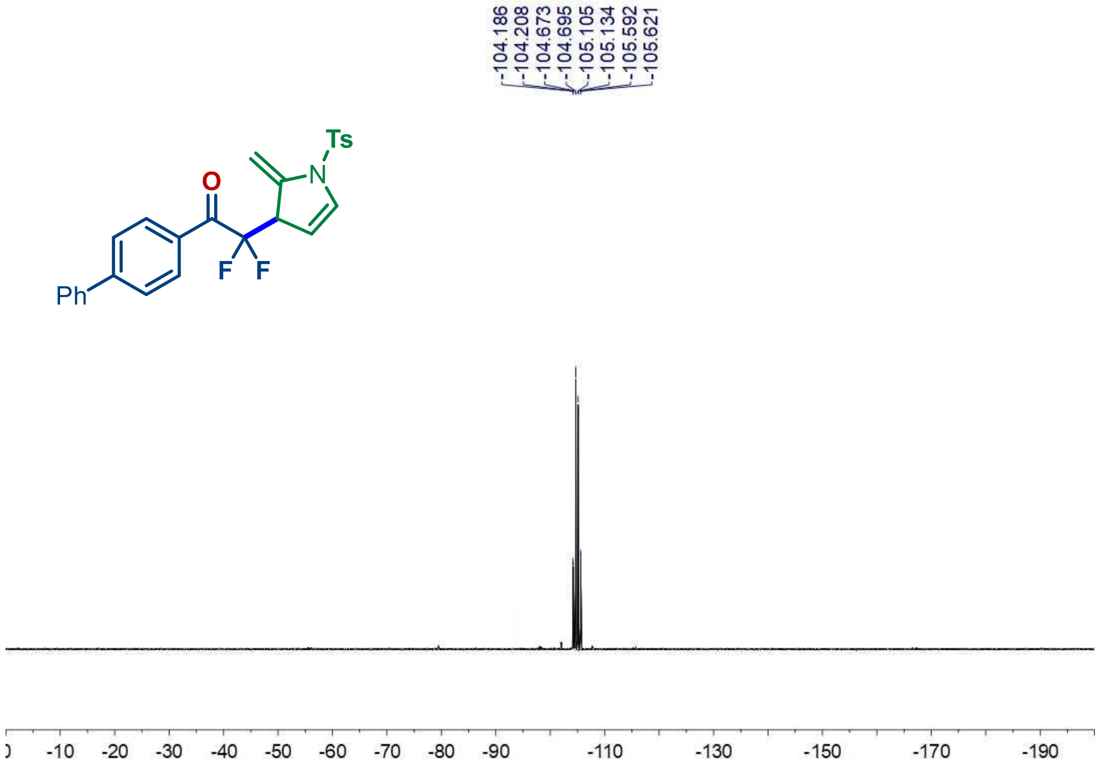

Supplementary Fig. 197 ^{19}F NMR (564 MHz, DMSO) spectrum of compound **65**

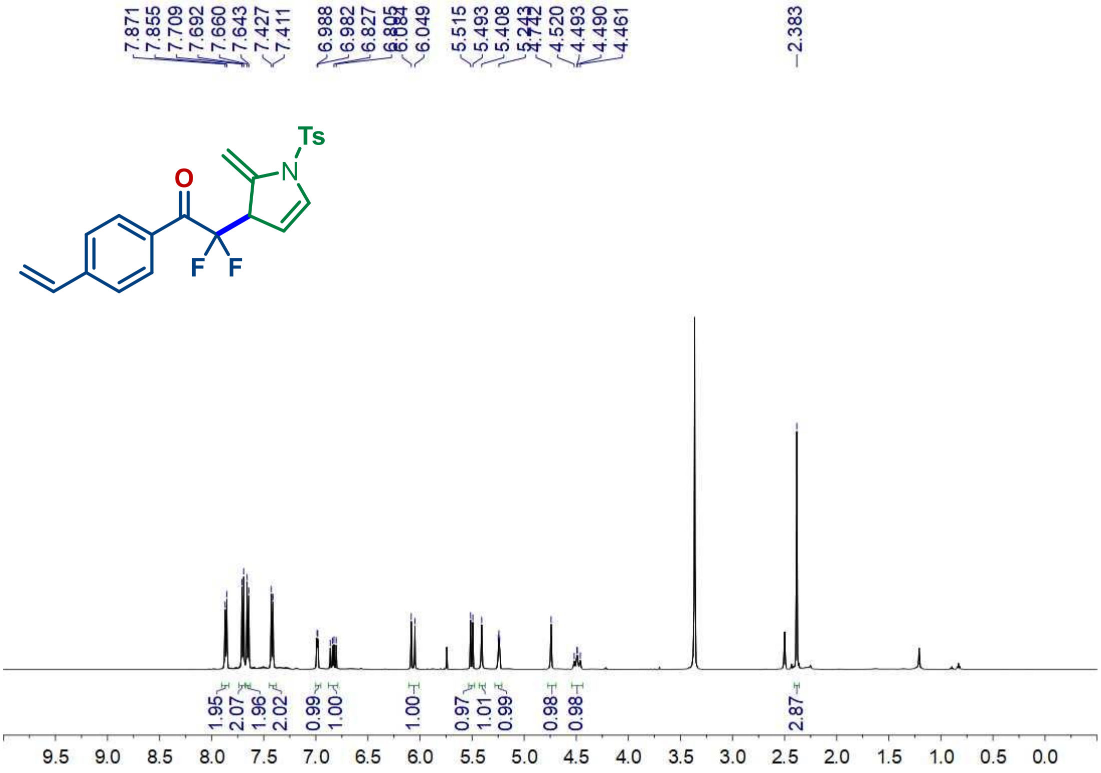

Supplementary Fig. 198 ^{1}H NMR (500 MHz, DMSO) spectrum of compound **66**

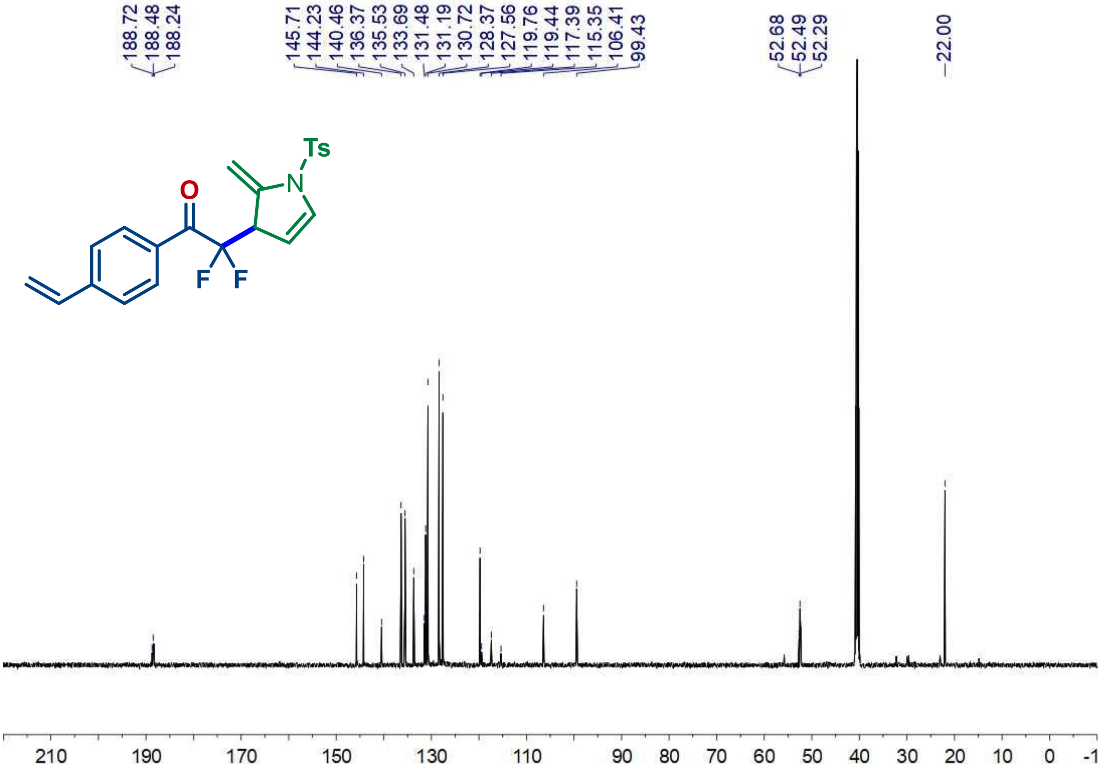

Supplementary Fig. 199 ^{13}C NMR (125 MHz, DMSO) spectrum of compound **66**

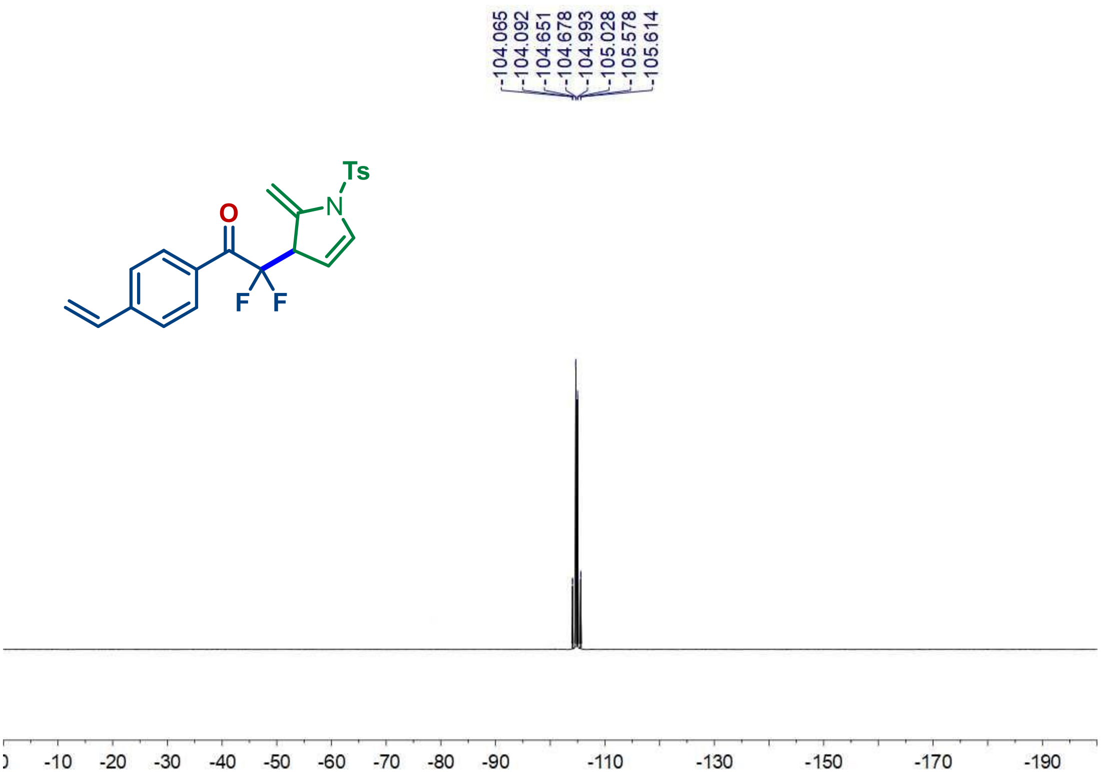

Supplementary Fig. 200 ^{19}F NMR (470 MHz, DMSO) spectrum of compound **66**

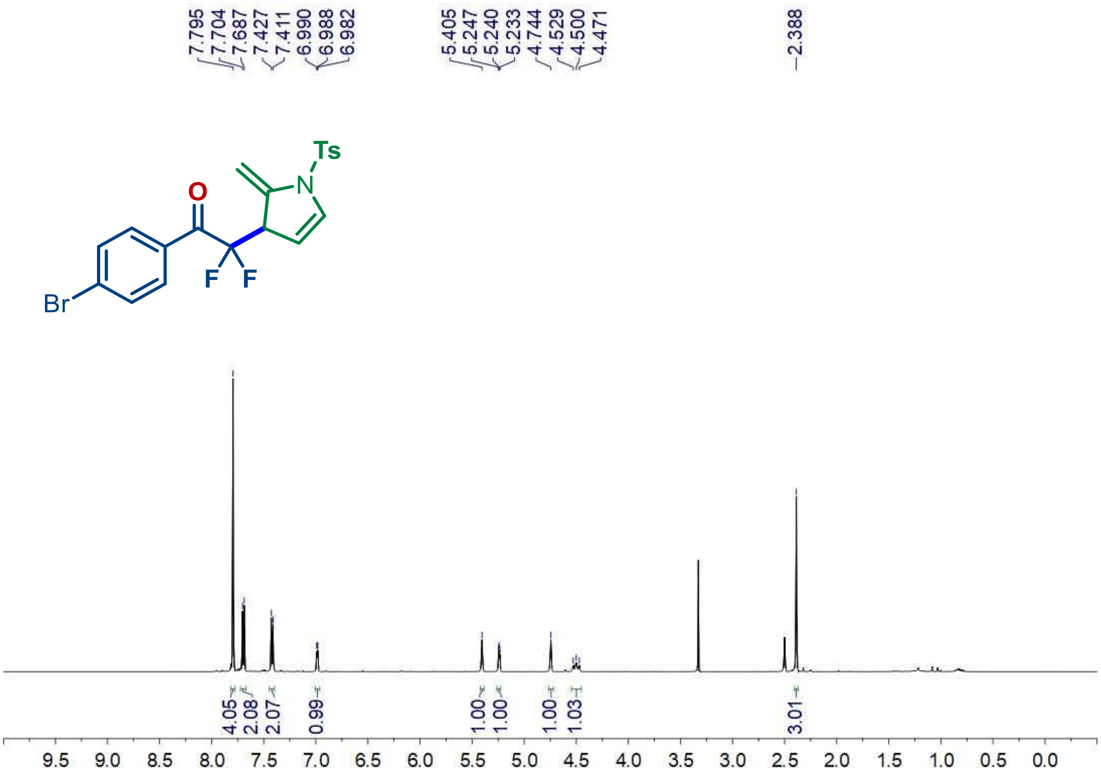

Supplementary Fig. 201 ^{1}H NMR (500 MHz, DMSO) spectrum of compound **67**

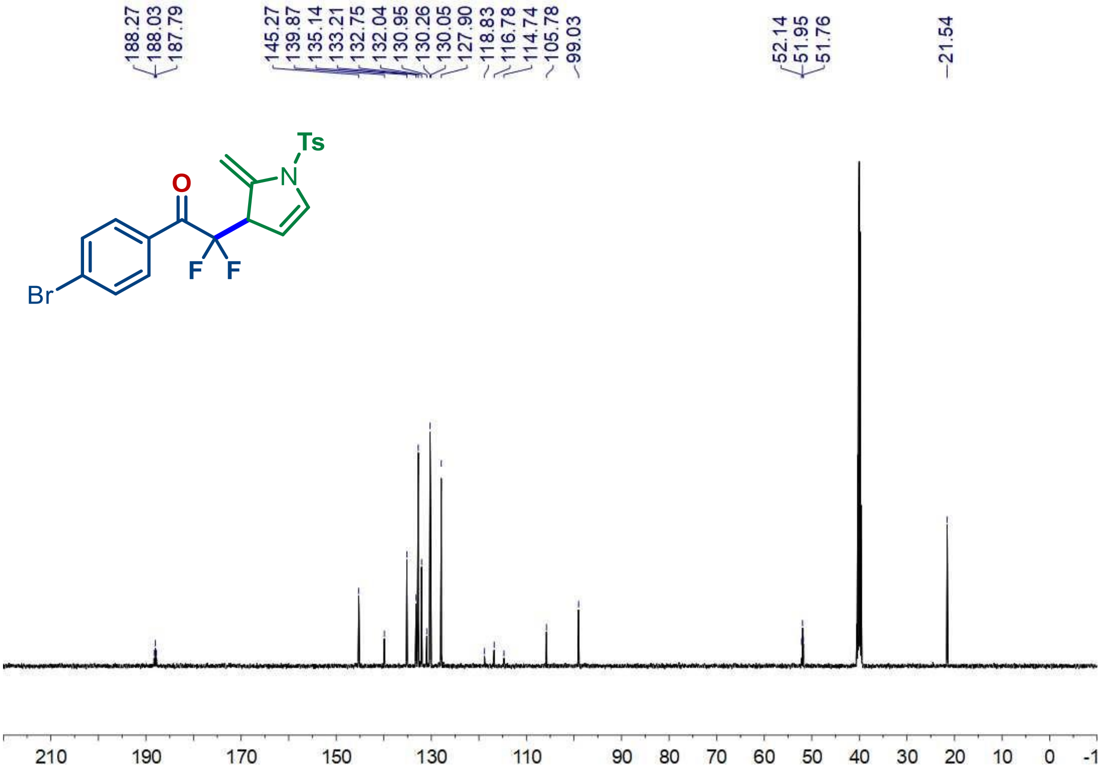

Supplementary Fig. 202 ^{13}C NMR (125 MHz, DMSO) spectrum of compound **67**

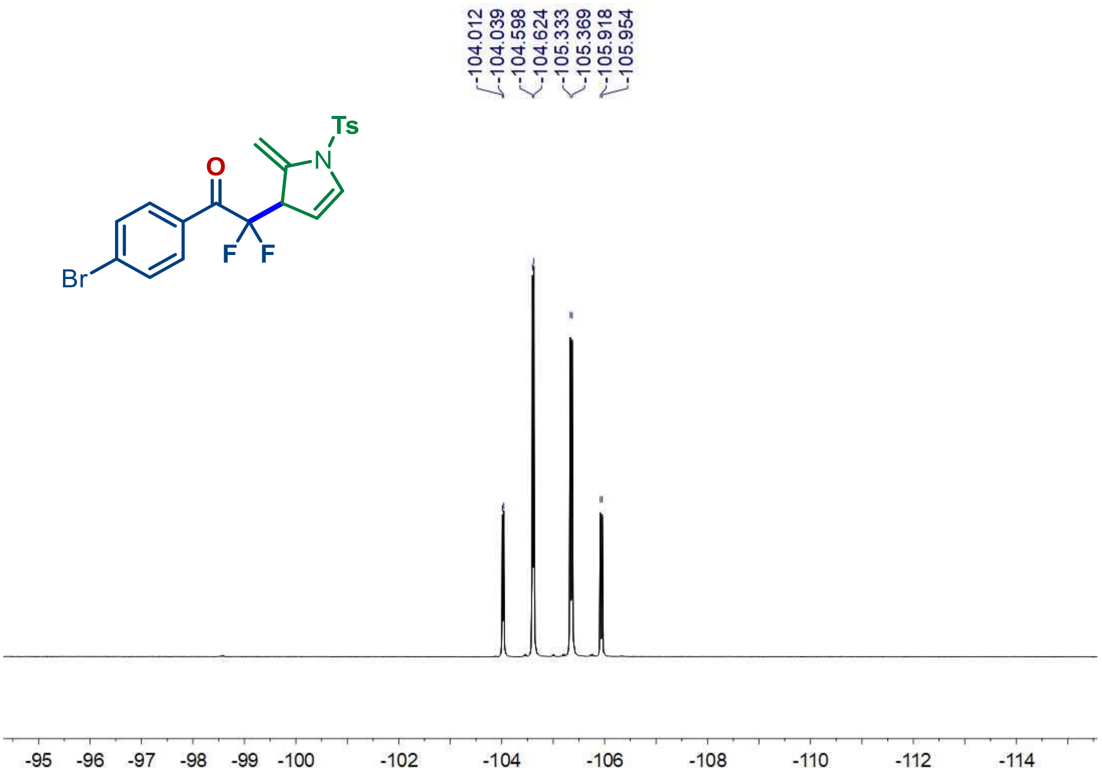

Supplementary Fig. 203 ^{19}F NMR (470 MHz, DMSO) spectrum of compound **67**

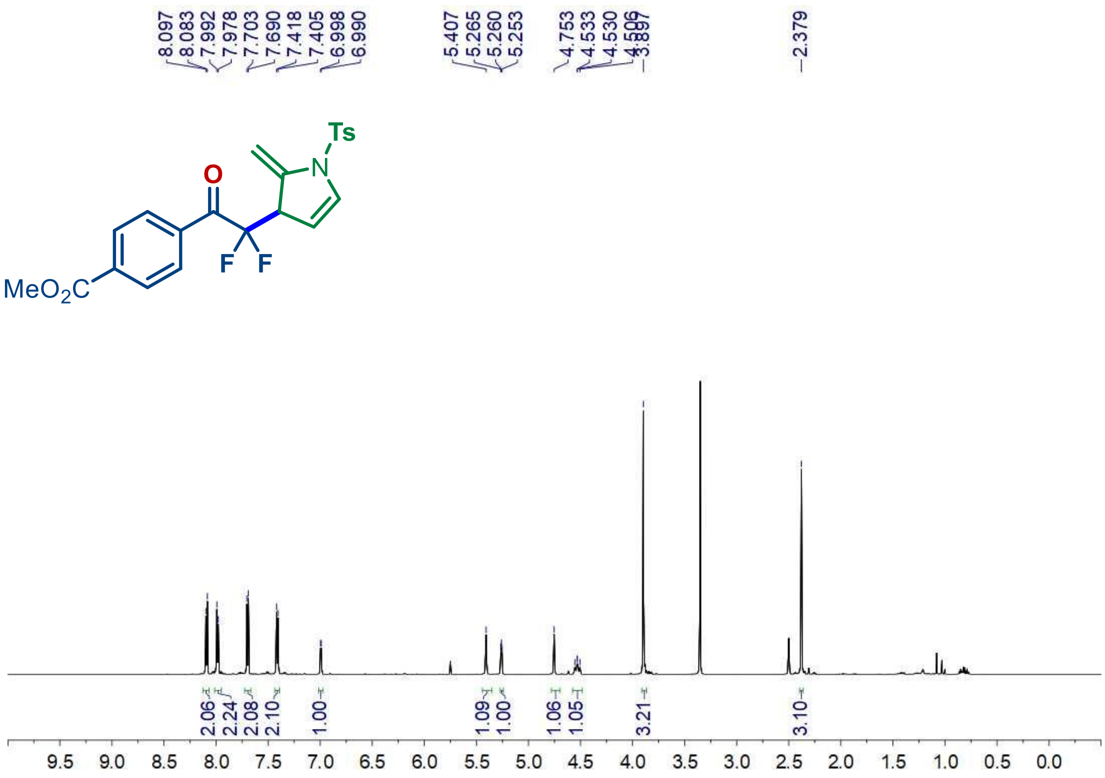

Supplementary Fig. 204 ^{1}H NMR (600 MHz, DMSO) spectrum of compound **68**

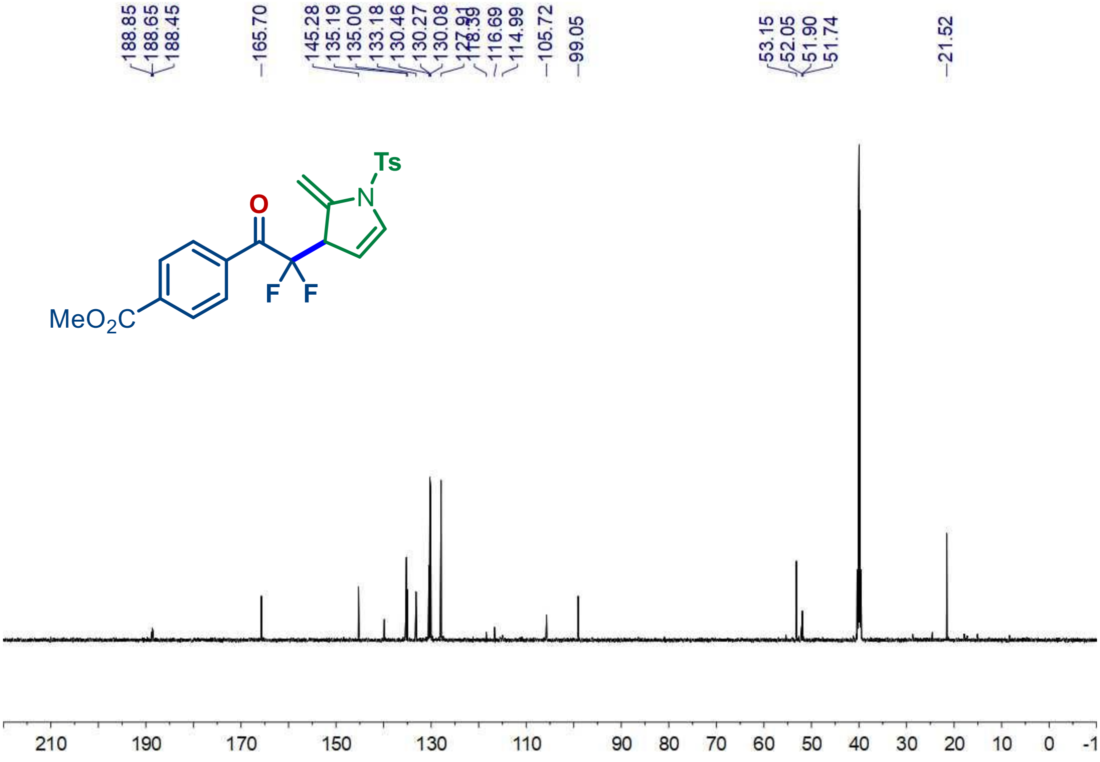

Supplementary Fig. 205 ^{13}C NMR (150 MHz, DMSO) spectrum of compound **68**

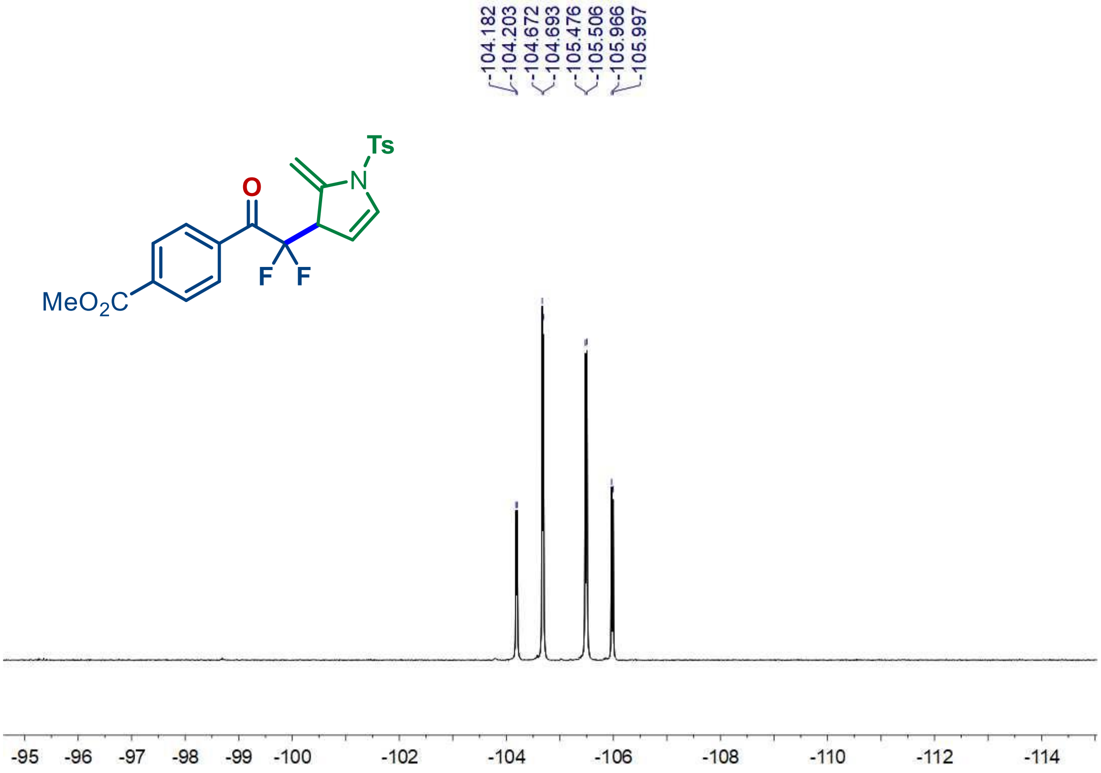

Supplementary Fig. 206 ^{19}F NMR (564 MHz, DMSO) spectrum of compound **68**

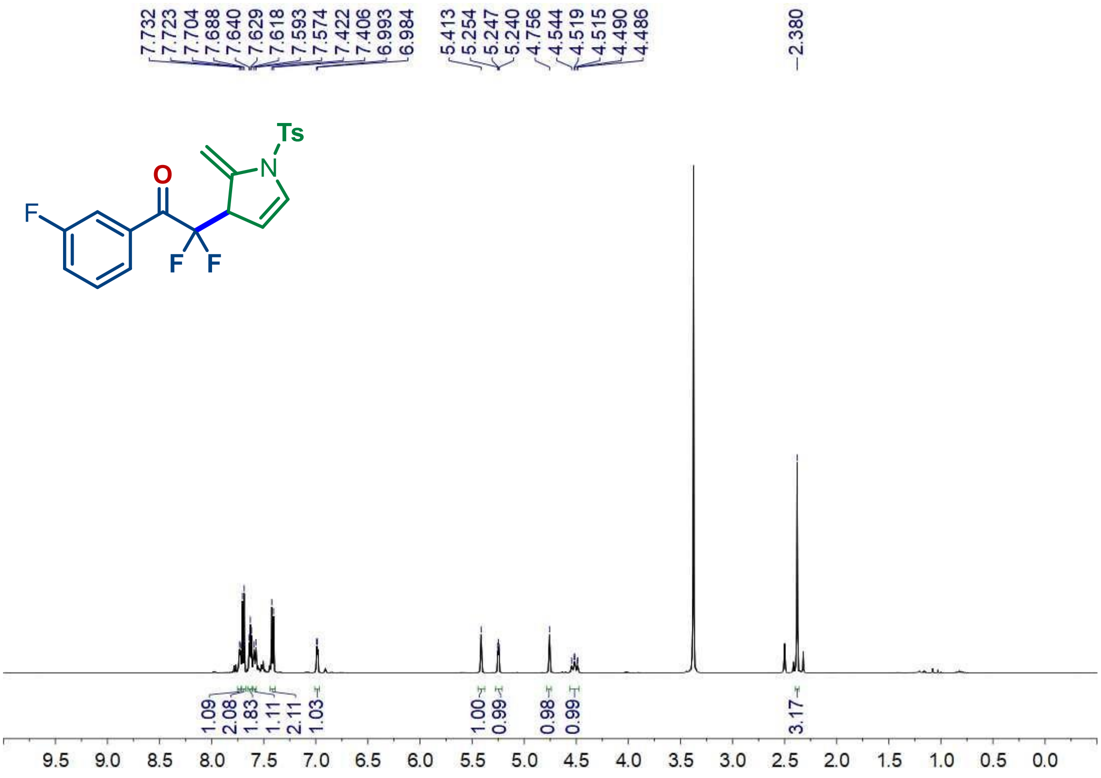

Supplementary Fig. 207 ^{1}H NMR (500 MHz, DMSO) spectrum of compound **69**

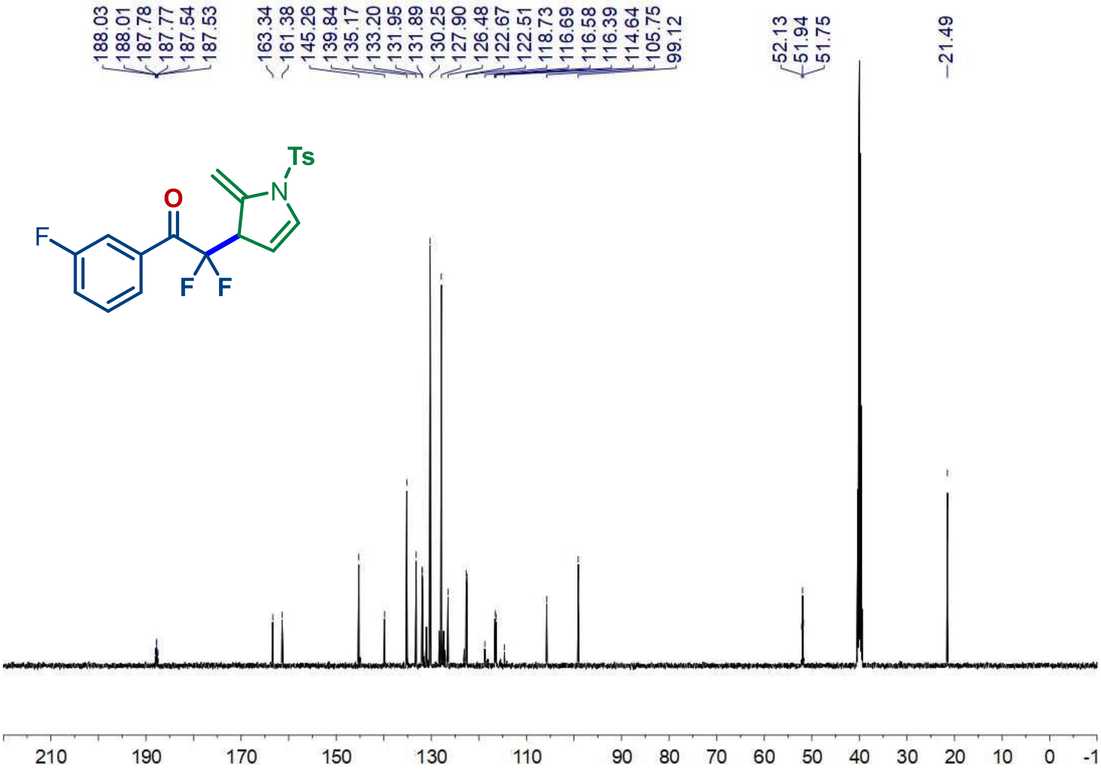

Supplementary Fig. 208 ^{13}C NMR (125 MHz, DMSO) spectrum of compound **69**

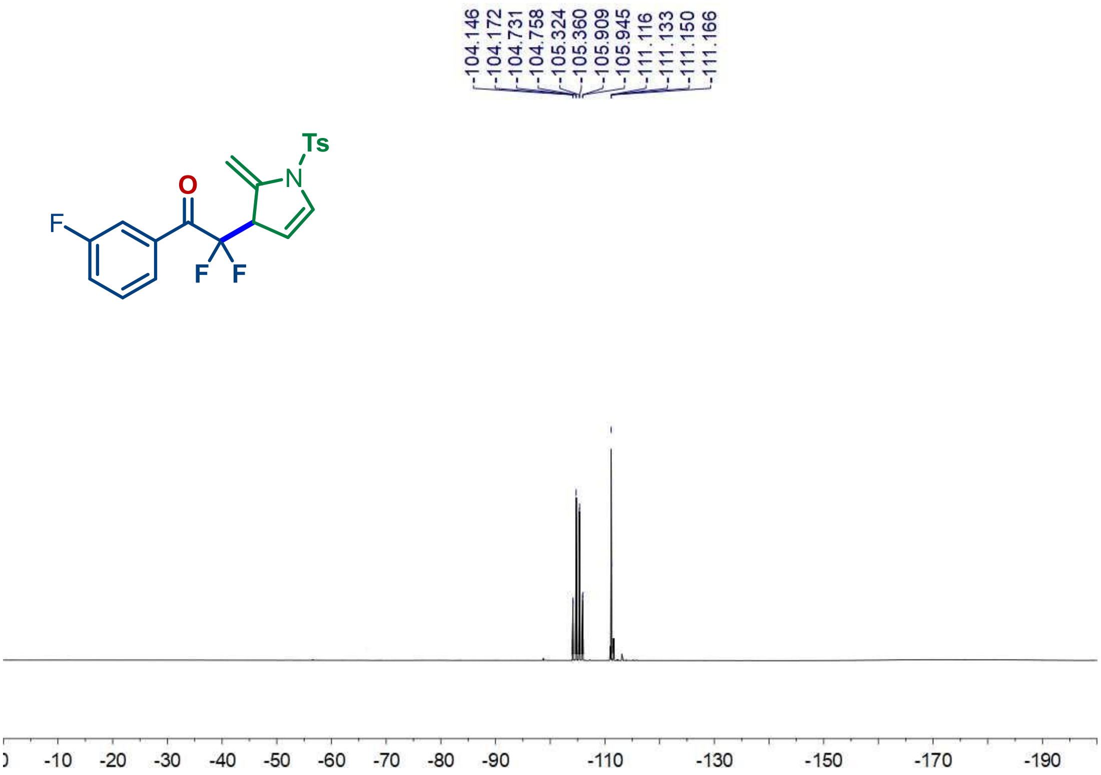

Supplementary Fig. 209 ^{19}F NMR (470 MHz, DMSO) spectrum of compound **69**

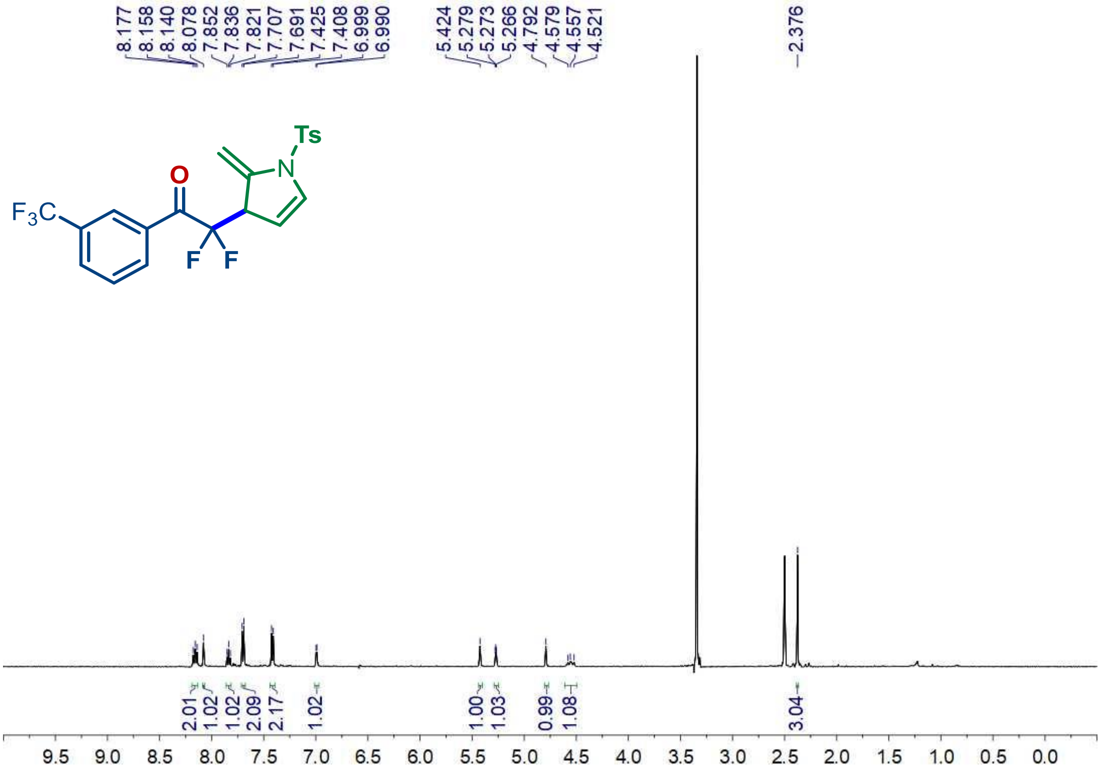

Supplementary Fig. 210 ^{1}H NMR (500 MHz, DMSO) spectrum of compound **70**

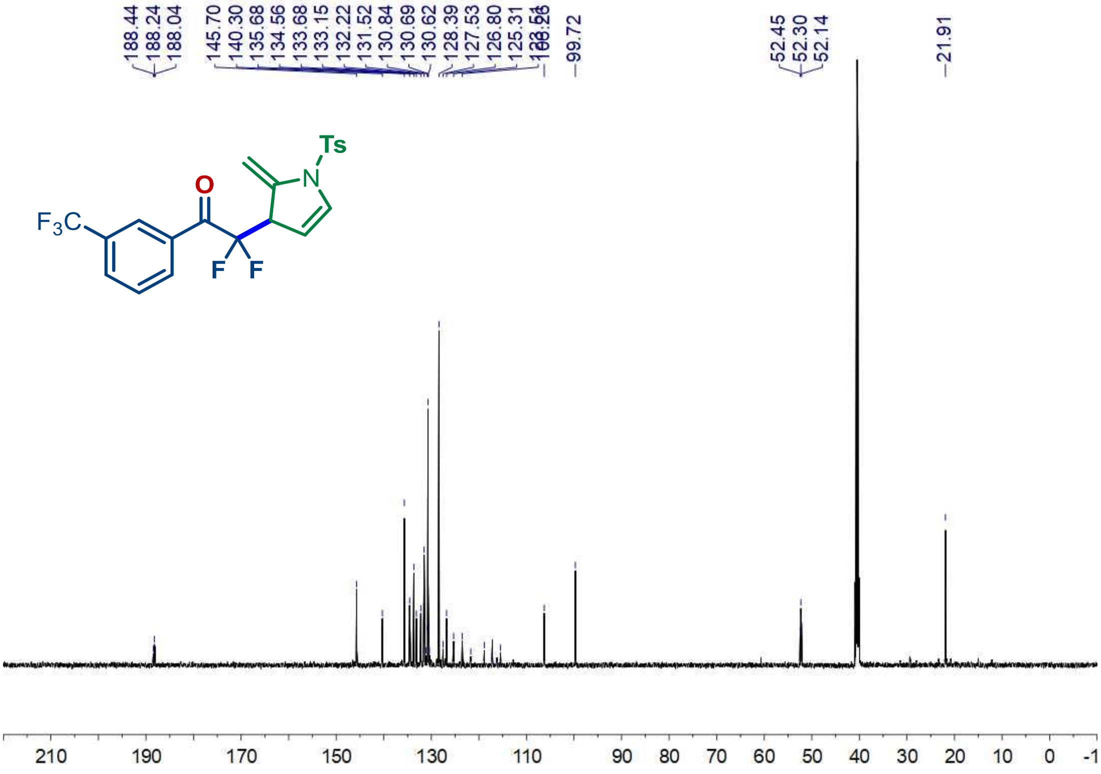

Supplementary Fig. 211 ^{13}C NMR (150 MHz, DMSO) spectrum of compound **70**

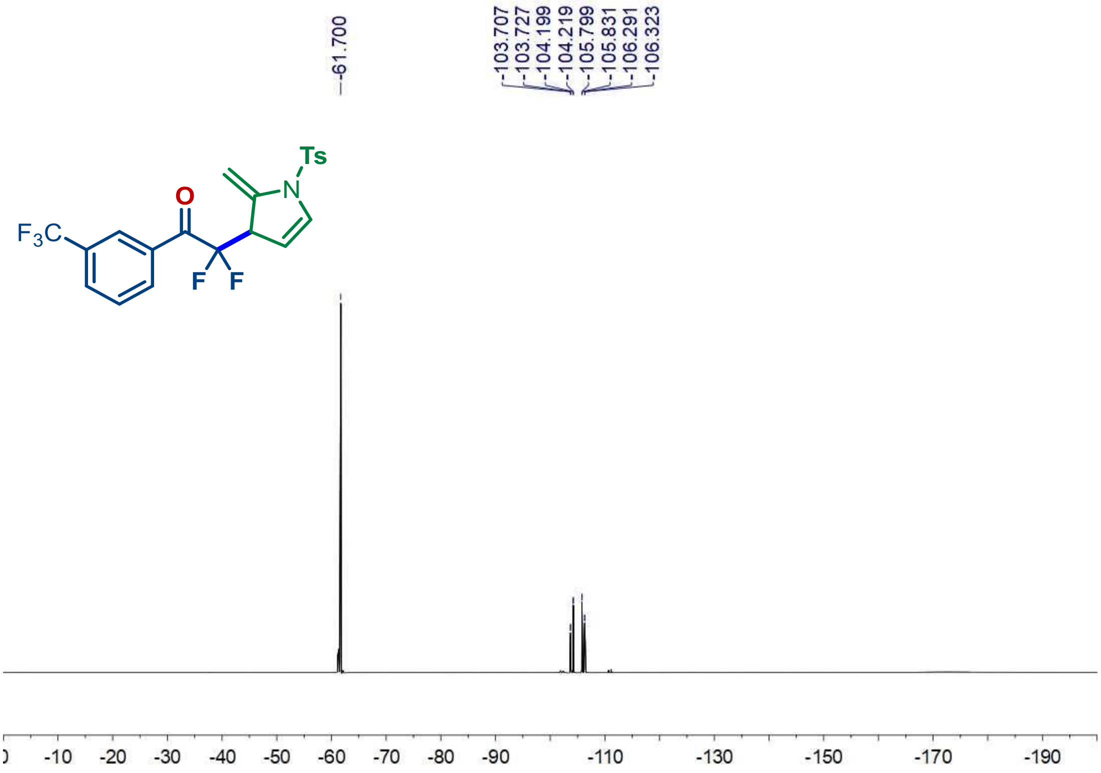

Supplementary Fig. 212 ^{19}F NMR (564 MHz, DMSO) spectrum of compound **70**

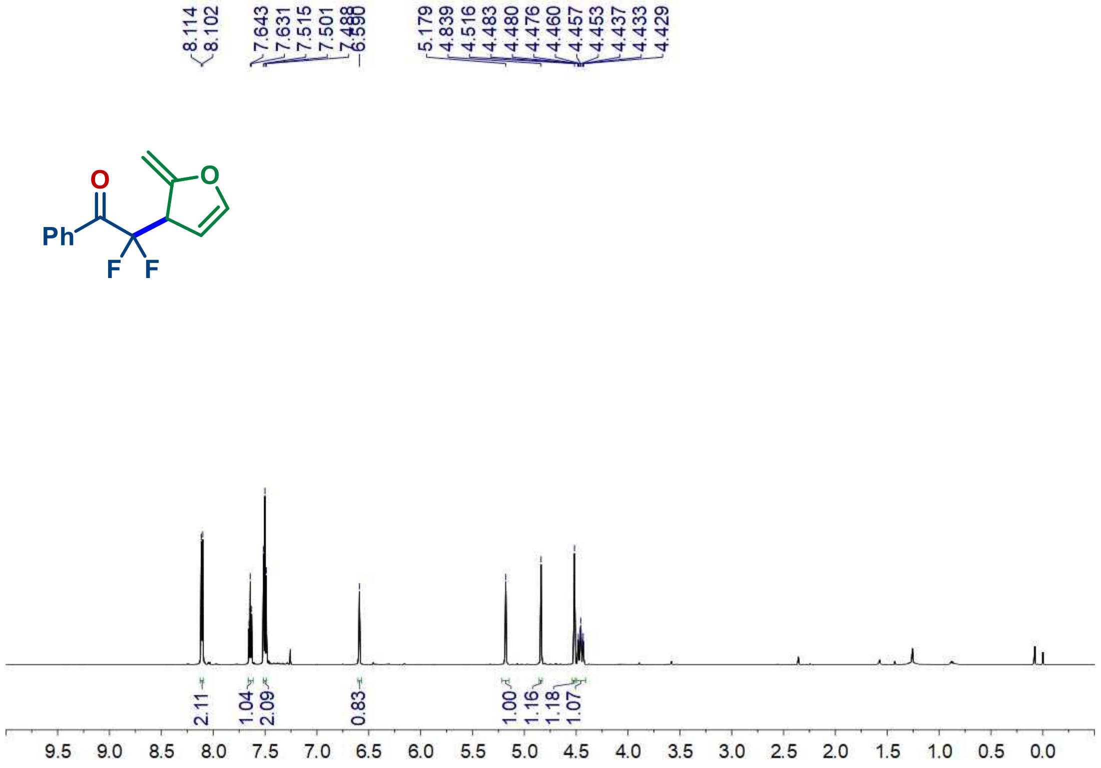

Supplementary Fig. 213 ^{1}H NMR (600 MHz, $CDCl_3$) spectrum of compound **71**

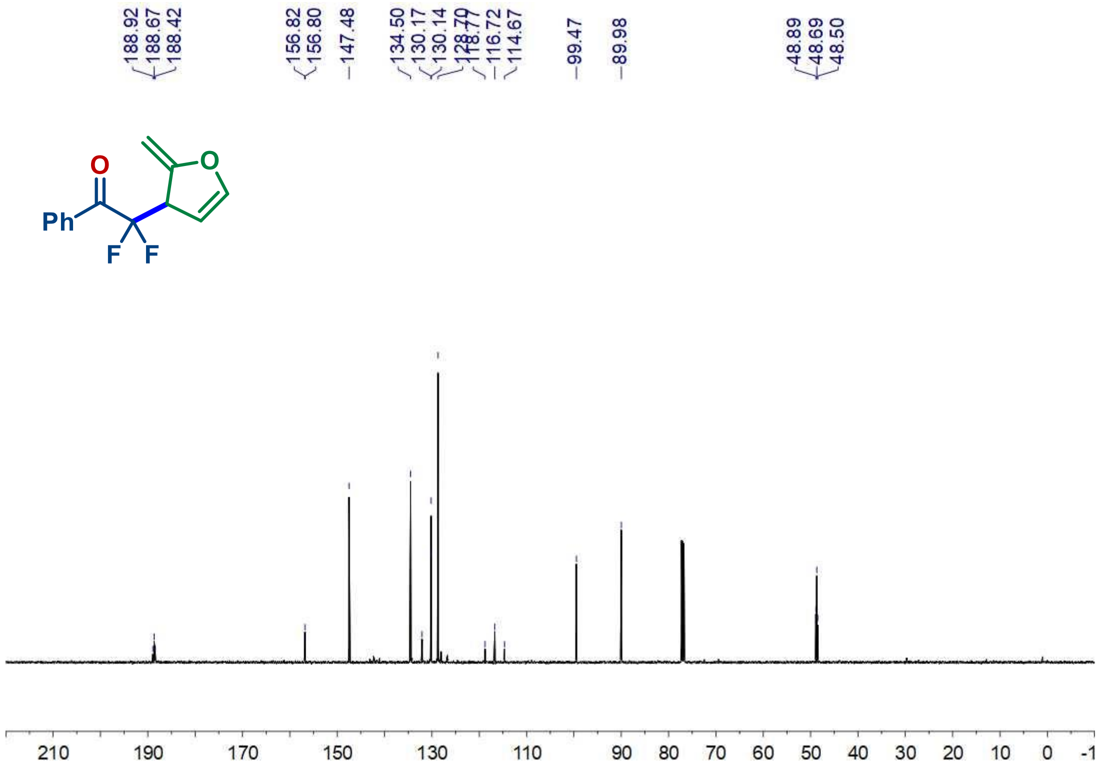

Supplementary Fig. 214 ^{13}C NMR (125 MHz, CDCl$_3$) spectrum of compound **71**

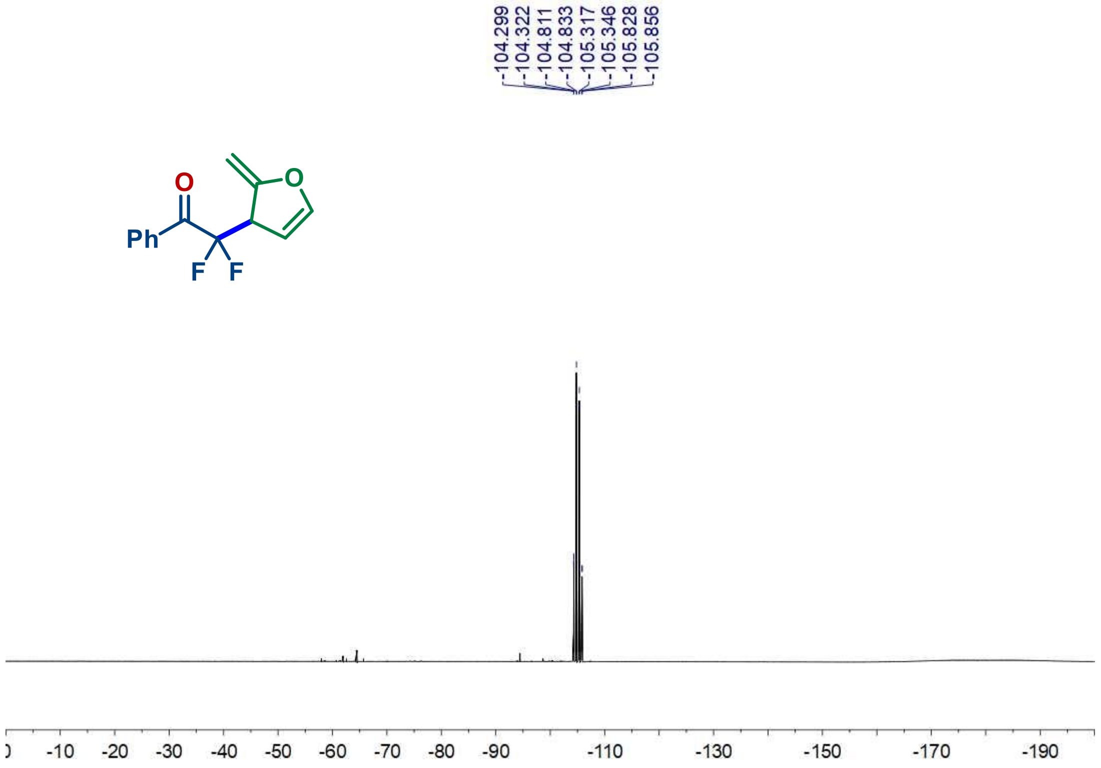

Supplementary Fig. 215 ^{19}F NMR (564 MHz, CDCl$_3$) spectrum of compound **71**

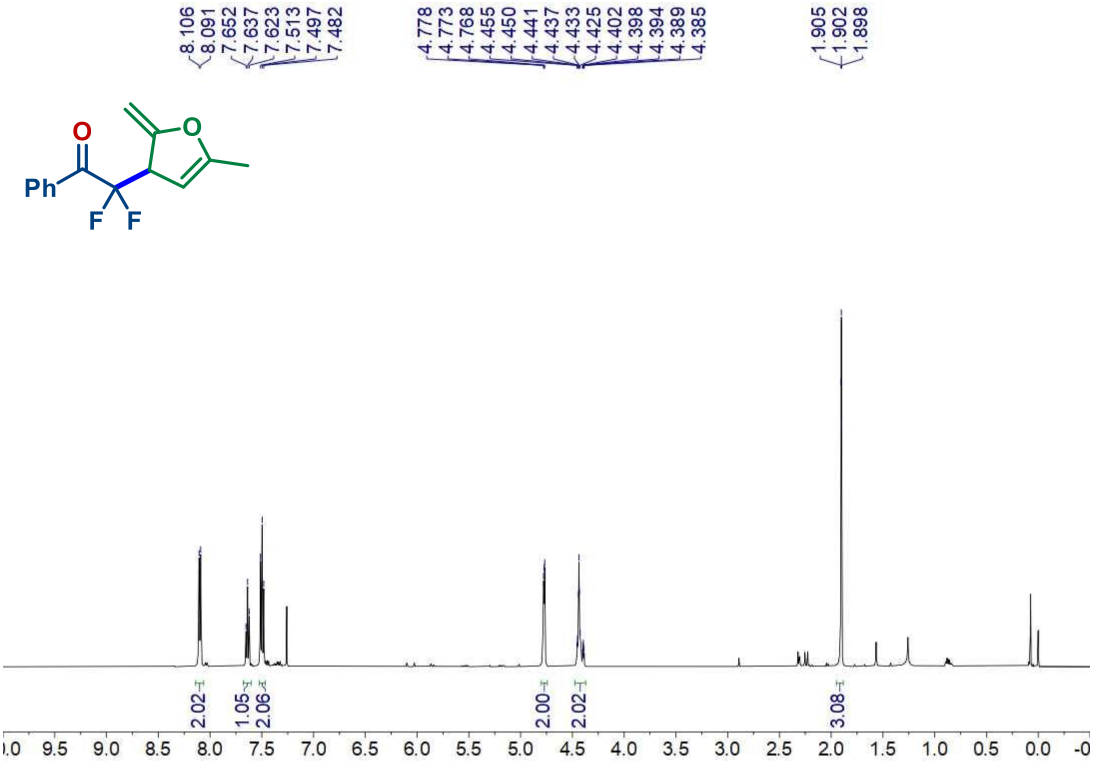

Supplementary Fig. 216 ^{1}H NMR (500 MHz, $CDCl_3$) spectrum of compound **72**

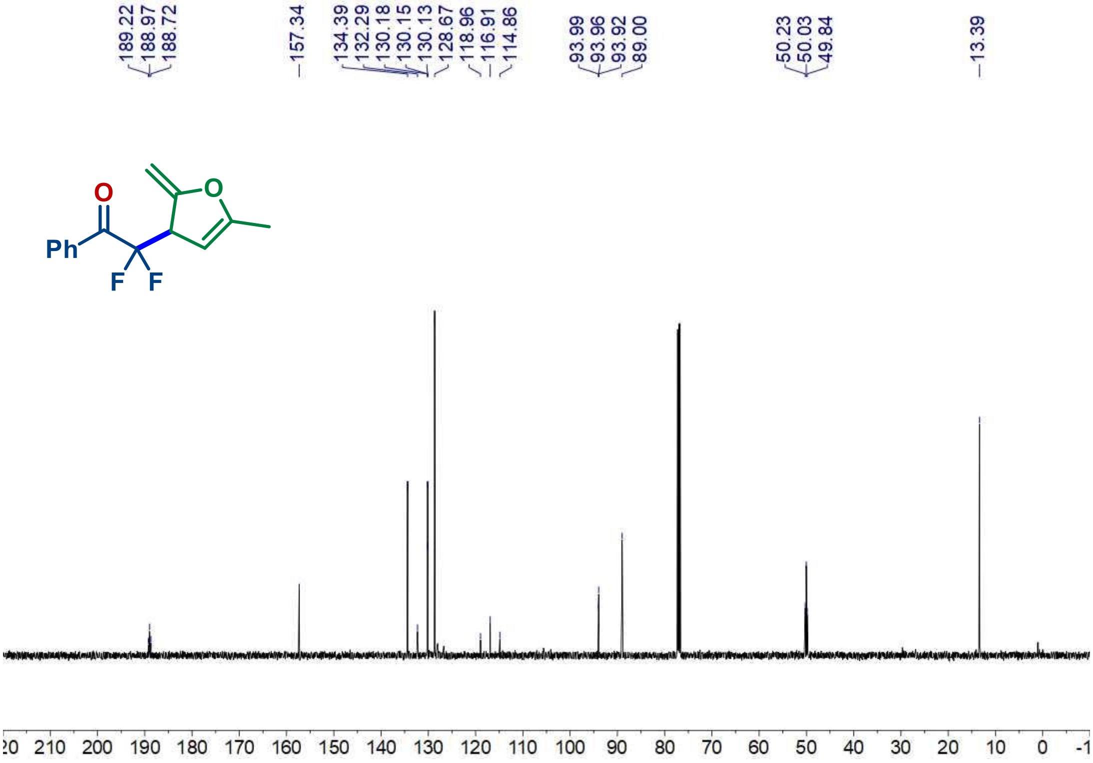

Supplementary Fig. 217 ^{13}C NMR (125 MHz, $CDCl_3$) spectrum of compound **72**

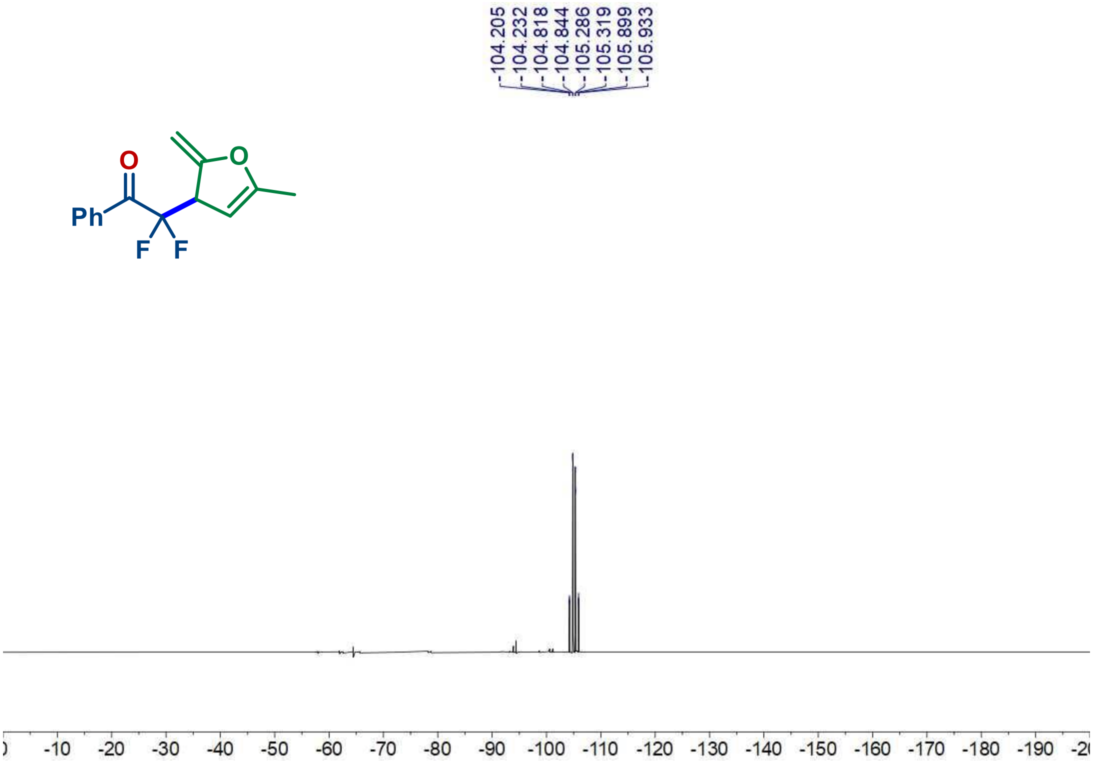

Supplementary Fig. 218 ^{19}F NMR (470 MHz, $CDCl_3$) spectrum of compound **72**

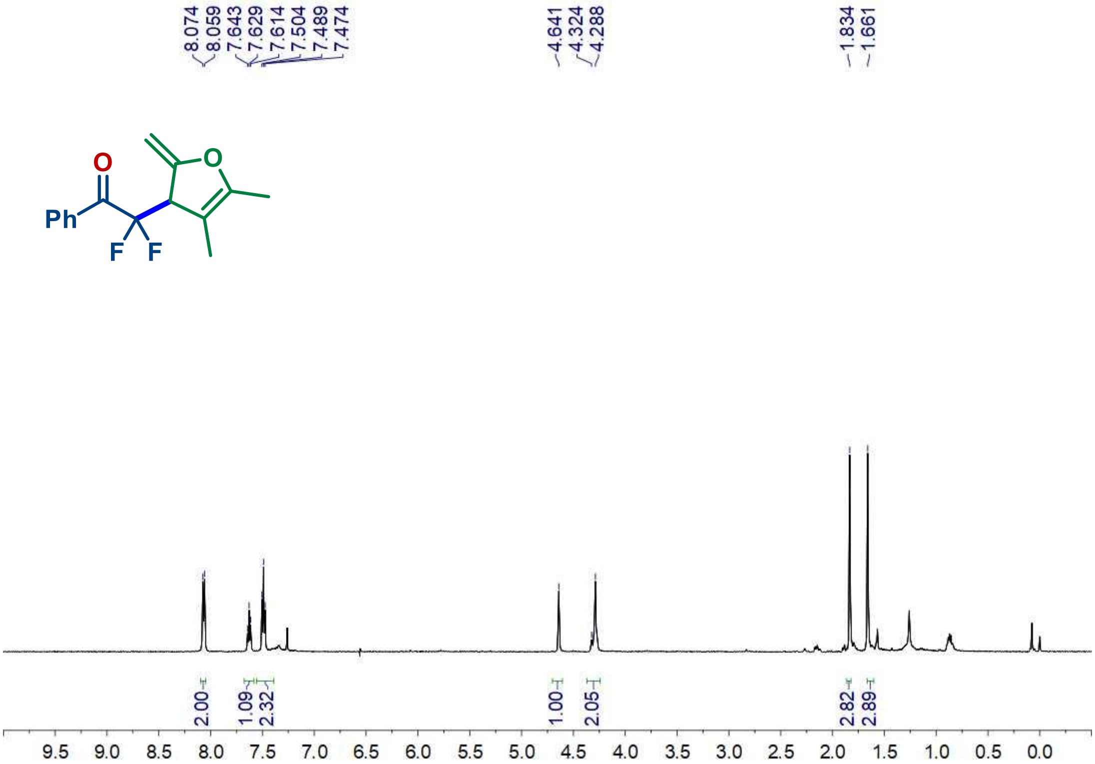

Supplementary Fig. 219 ^{1}H NMR (500 MHz, $CDCl_3$) spectrum of compound **73**

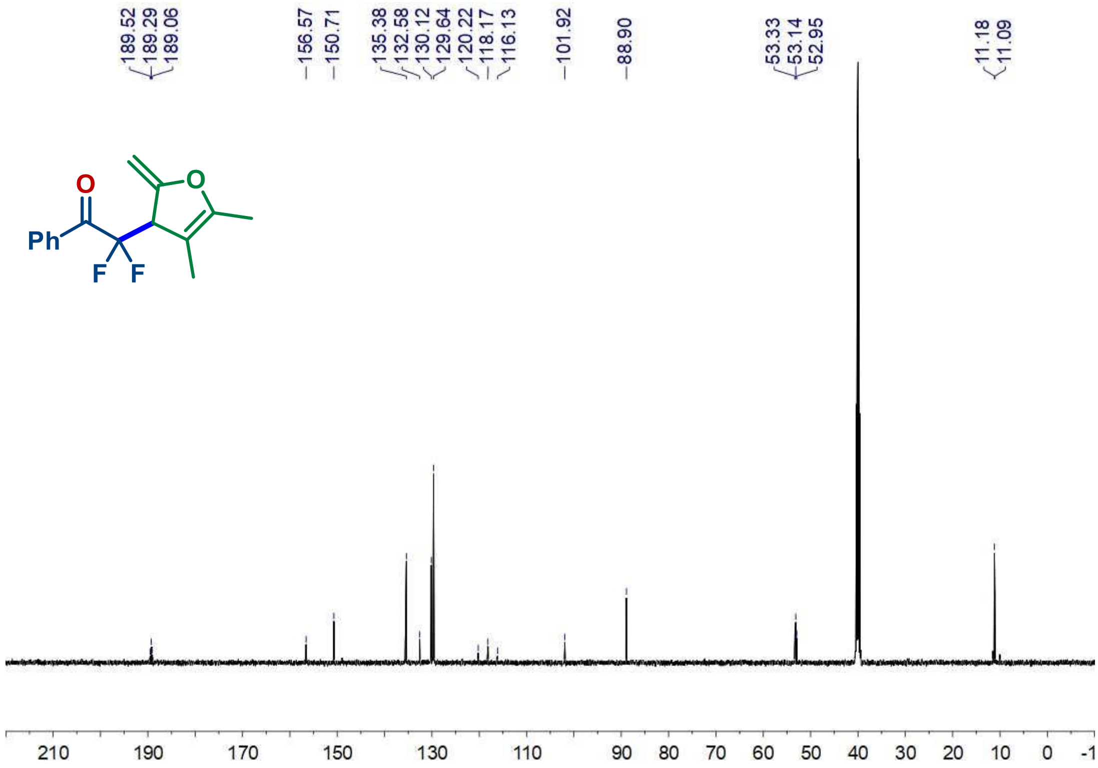

Supplementary Fig. 220 ^{13}C NMR (125 MHz, DMSO) spectrum of compound **73**

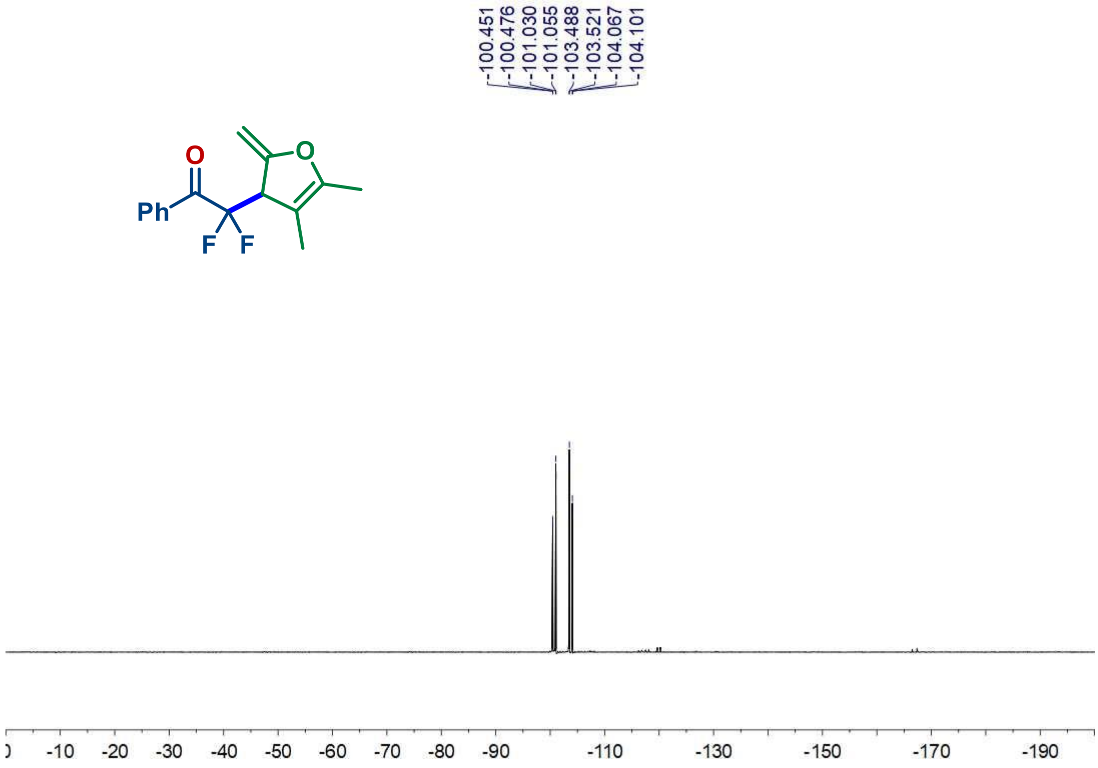

Supplementary Fig. 221 ^{19}F NMR (470 MHz, DMSO) spectrum of compound **73**

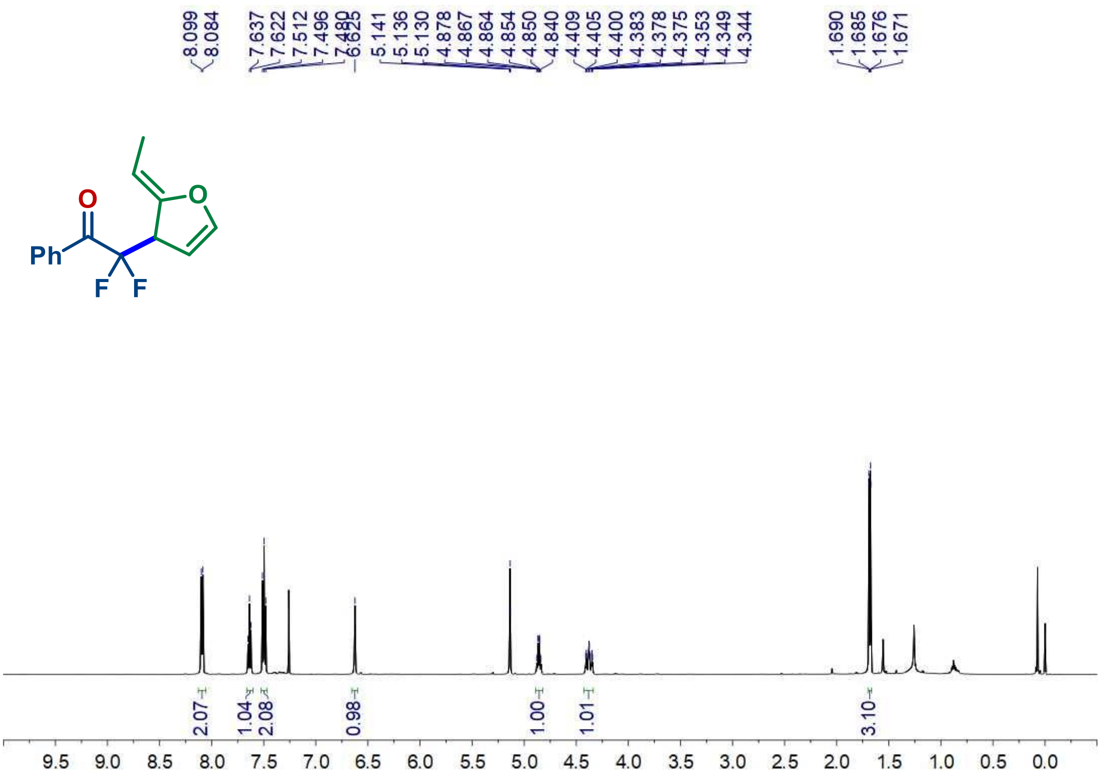

Supplementary Fig. 222 ^{1}H NMR (500 MHz, $CDCl_3$) spectrum of compound **74**

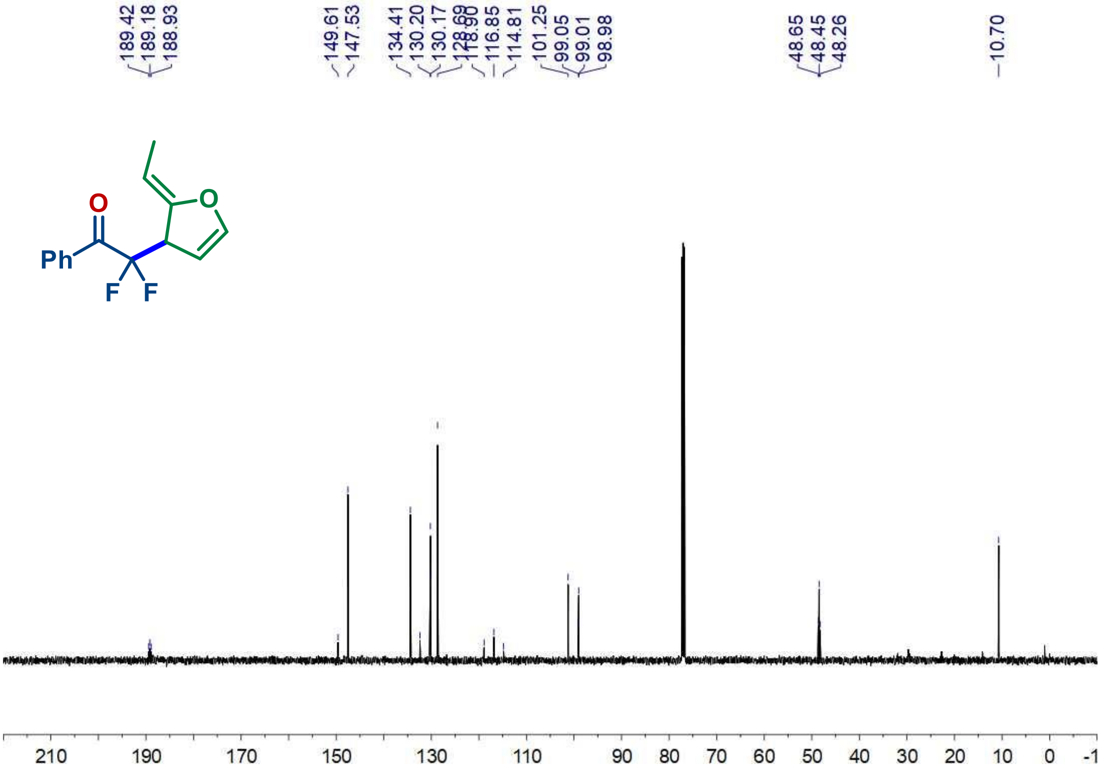

Supplementary Fig. 223 ^{13}C NMR (125 MHz, $CDCl_3$) spectrum of compound **74**

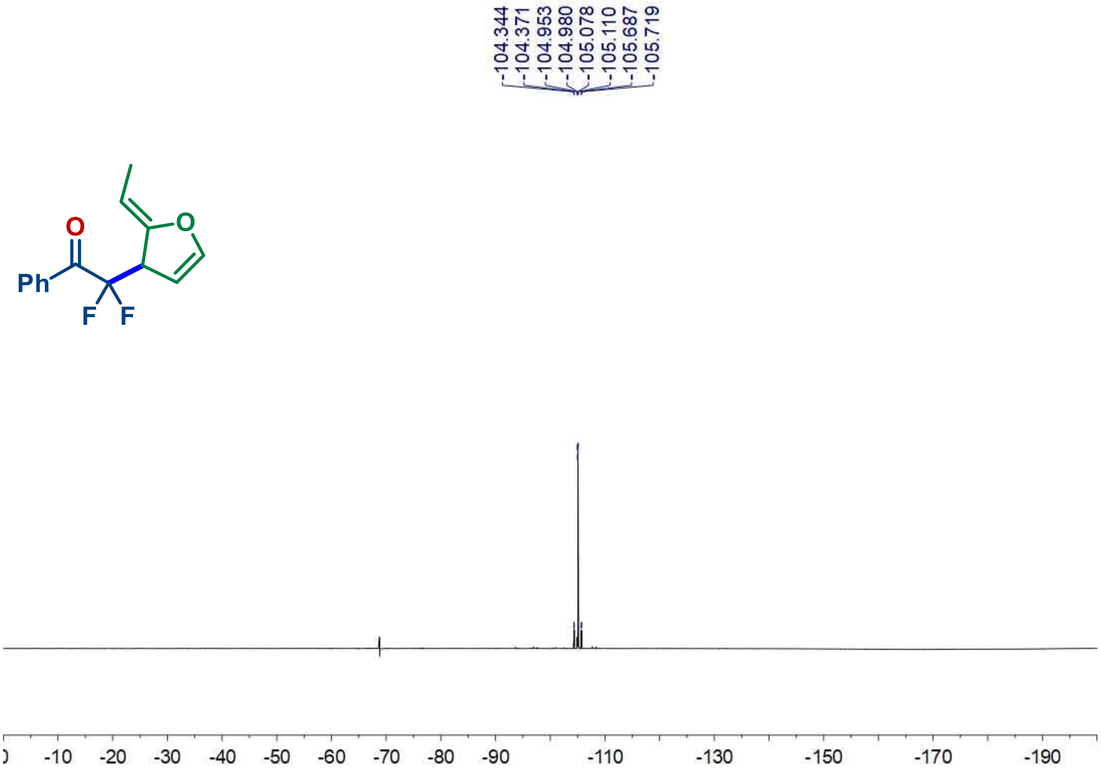

Supplementary Fig. 224 ^{19}F NMR (470 MHz, $CDCl_3$) spectrum of compound **74**

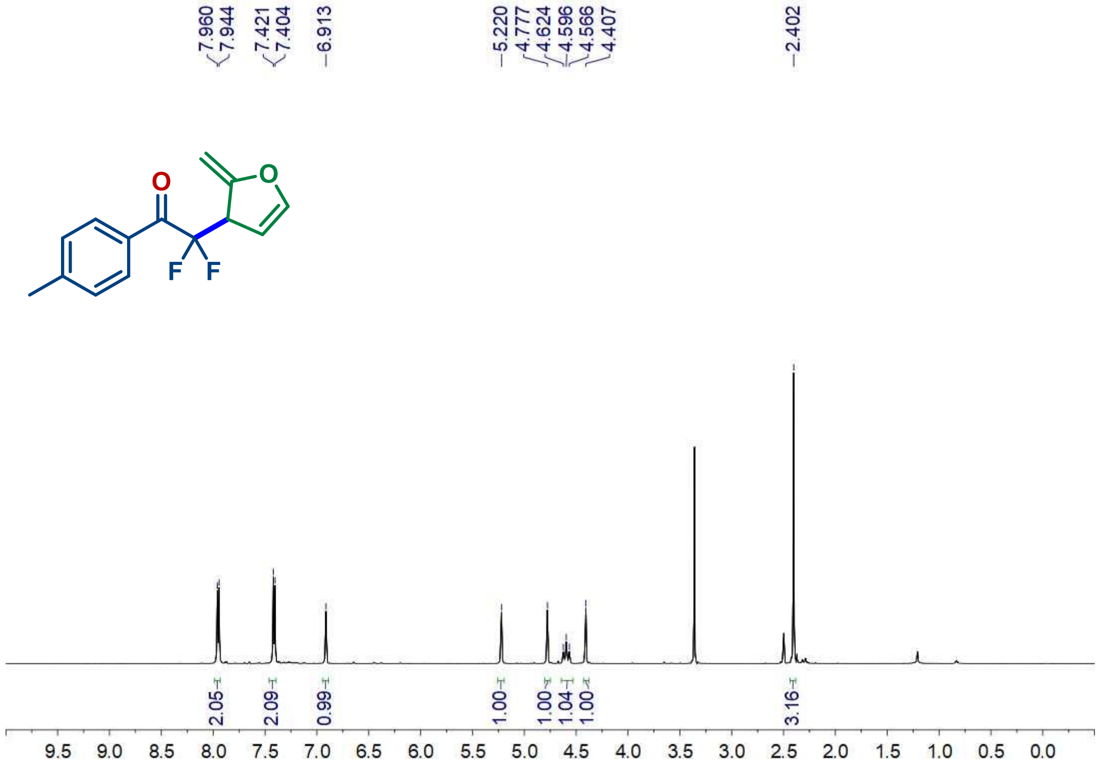

Supplementary Fig. 225 ^{1}H NMR (500 MHz, DMSO) spectrum of compound **75**

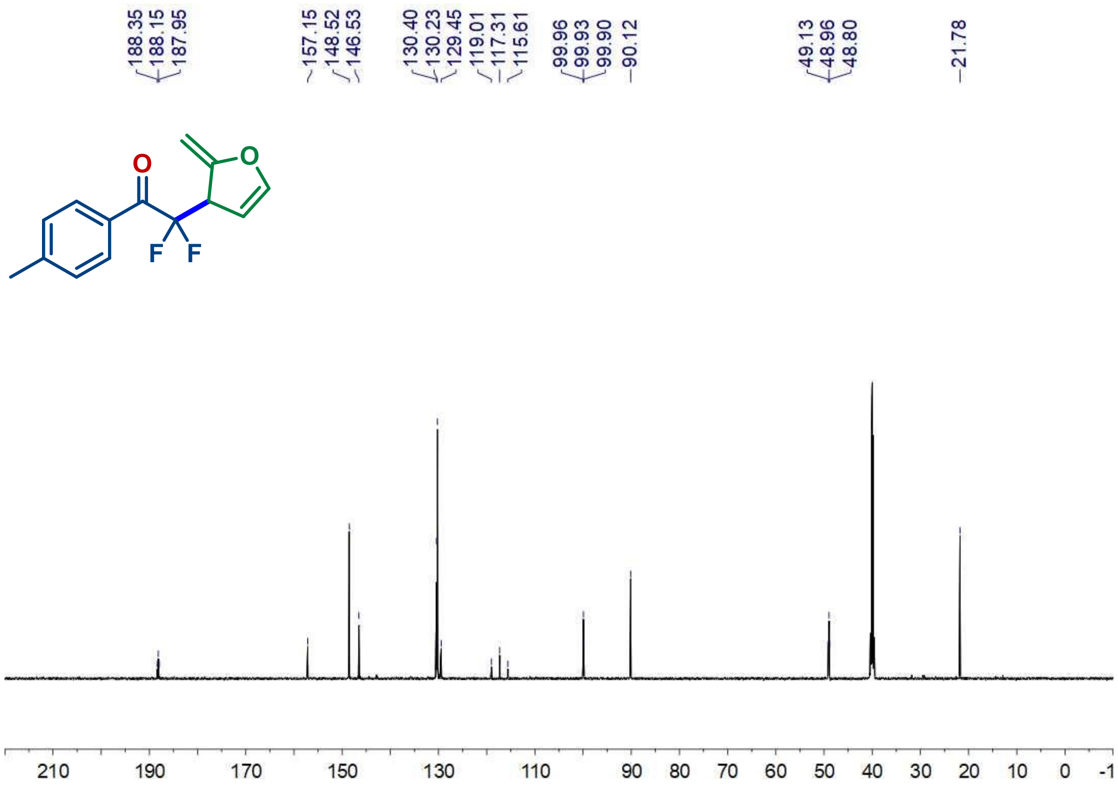

Supplementary Fig. 226 ^{13}C NMR (150 MHz, DMSO) spectrum of compound **75**

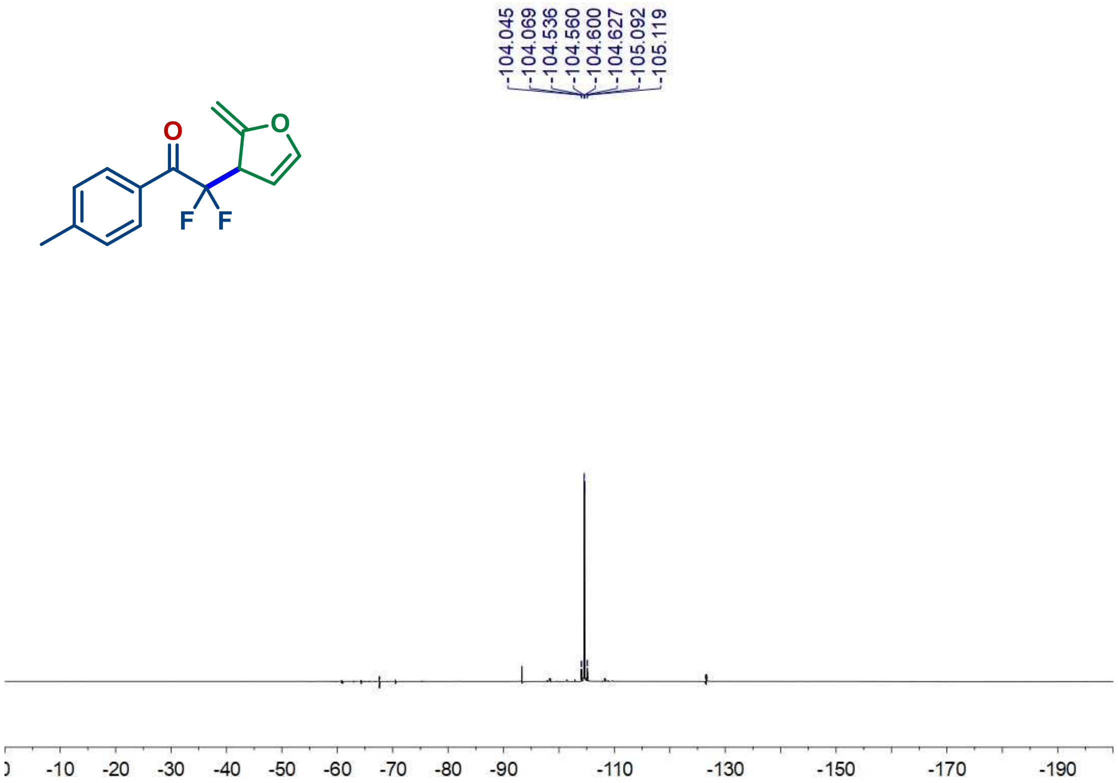

Supplementary Fig. 227 ^{19}F NMR (564 MHz, DMSO) spectrum of compound **75**

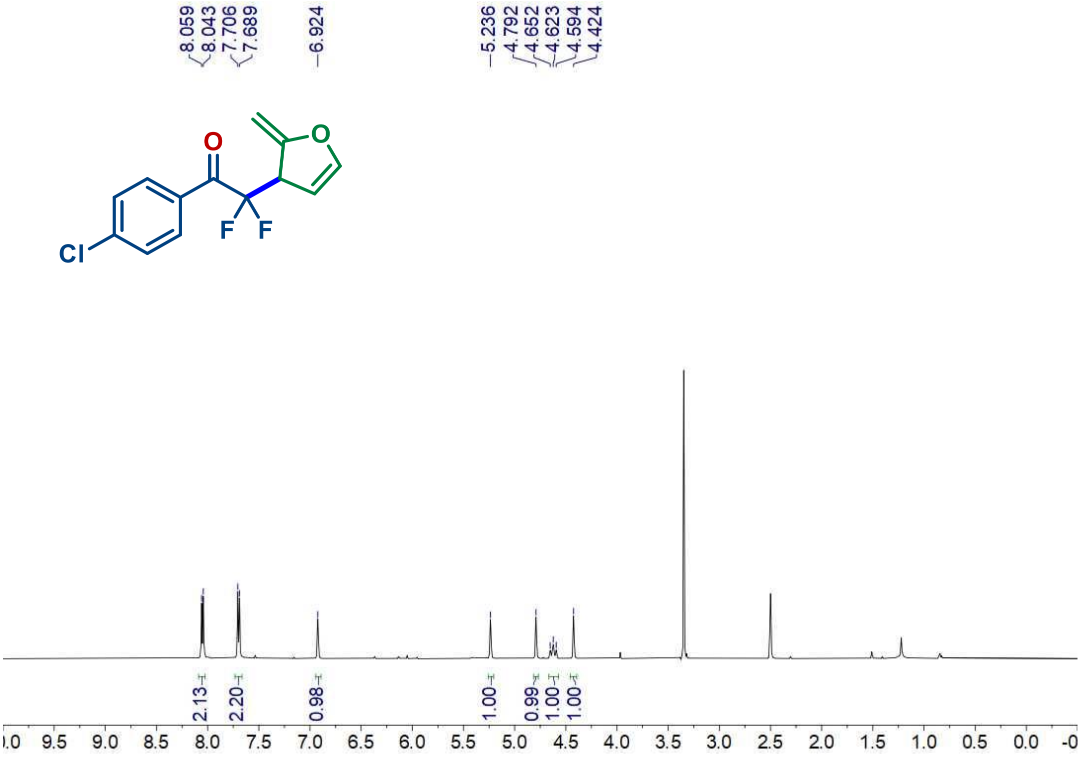

Supplementary Fig. 228 ^{1}H NMR (500 MHz, $CDCl_3$) spectrum of compound **76**

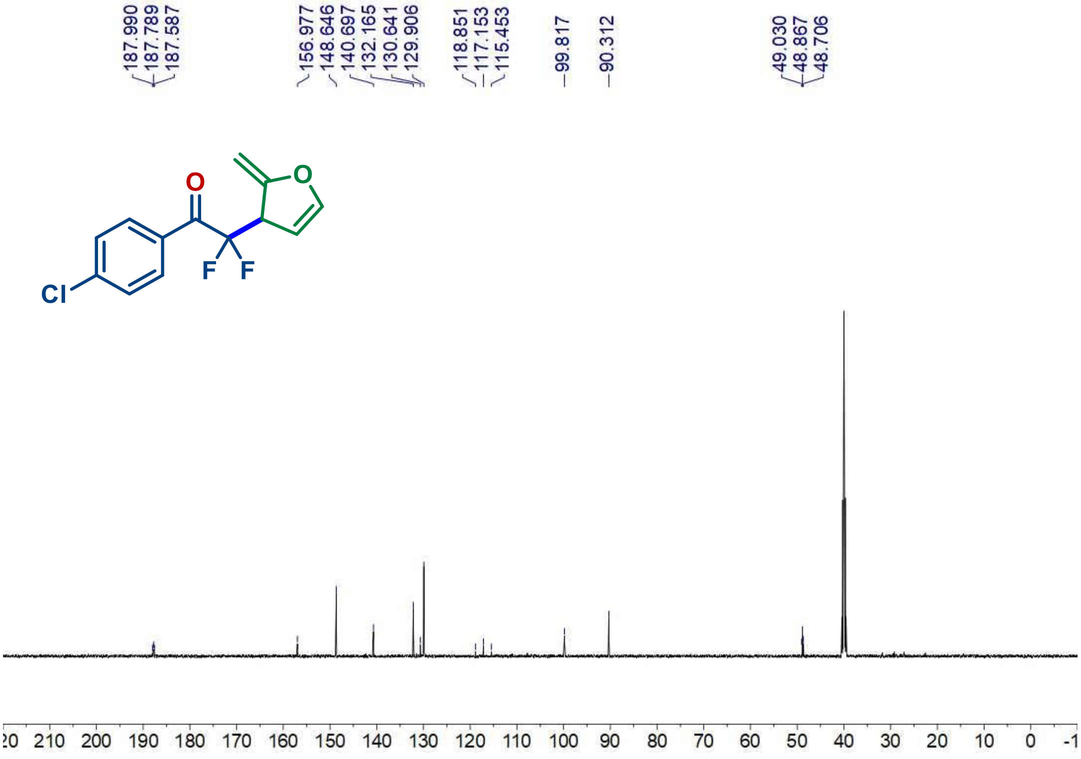

Supplementary Fig. 229 ^{13}C NMR (150 MHz, DMSO) spectrum of compound **76**

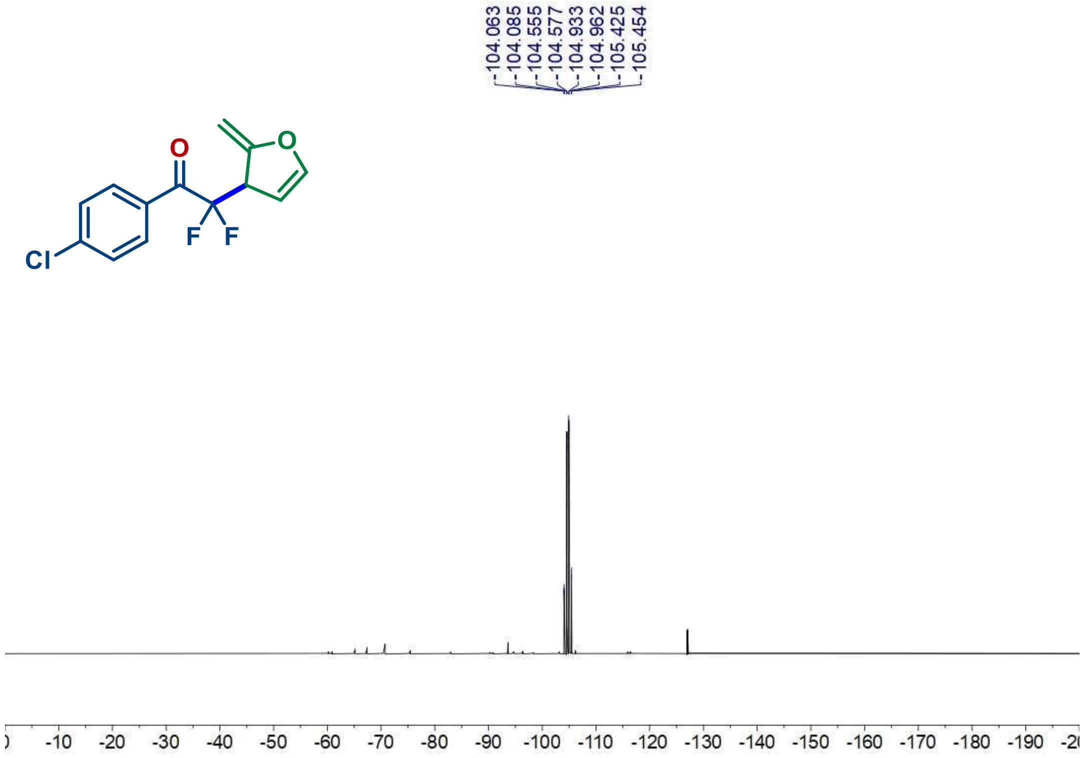

Supplementary Fig. 230 ^{19}F NMR (564 MHz, DMSO) spectrum of compound **76**

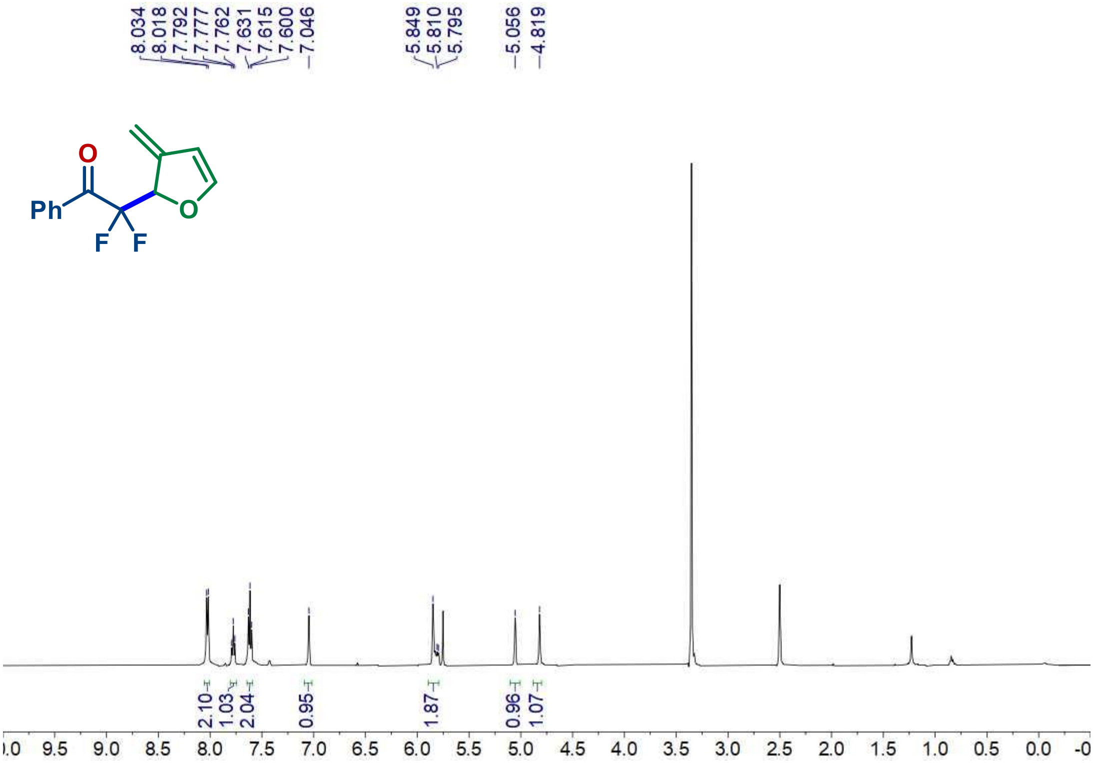

Supplementary Fig. 231 ^{1}H NMR (500 MHz, DMSO) spectrum of compound **77**

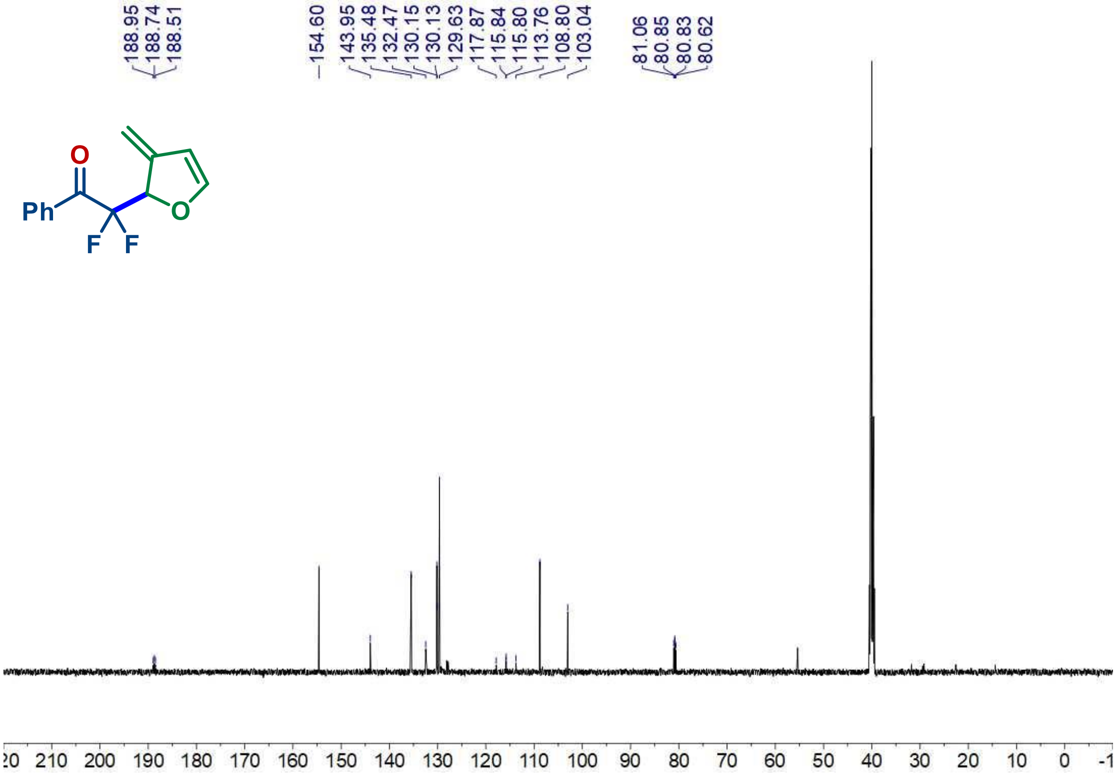

Supplementary Fig. 232 ^{13}C NMR (125 MHz, DMSO) spectrum of compound **77**

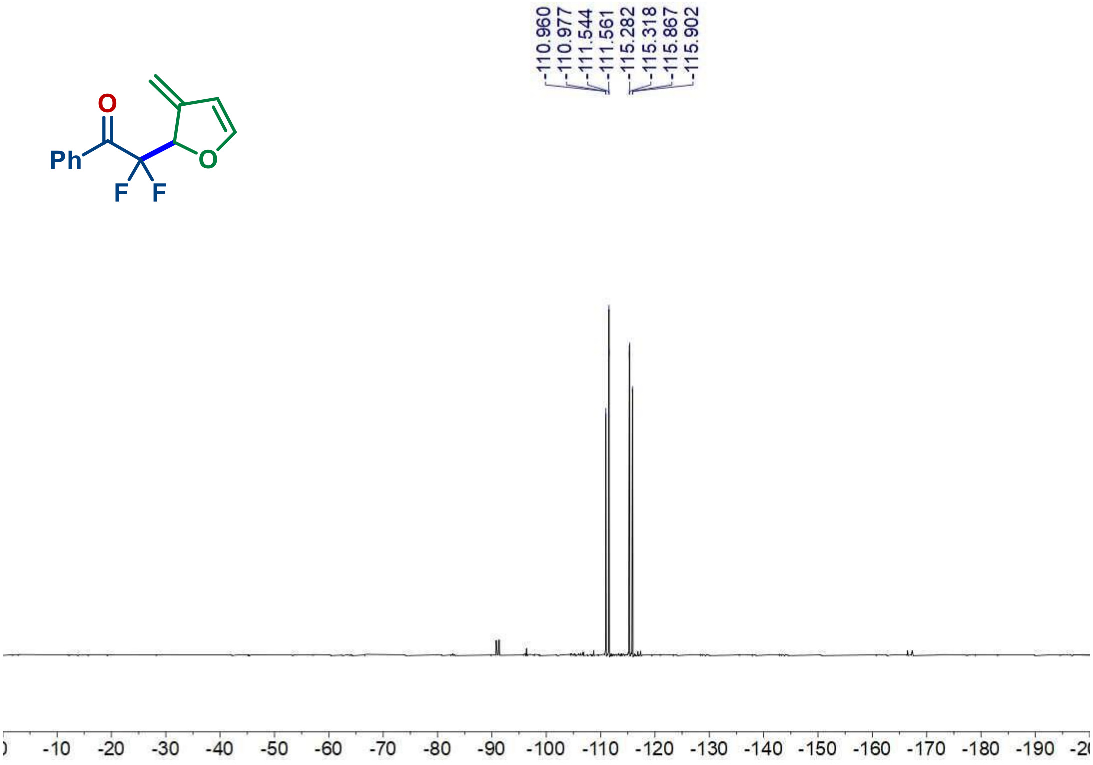

Supplementary Fig. 233 ^{19}F NMR (470 MHz, DMSO) spectrum of compound **77**

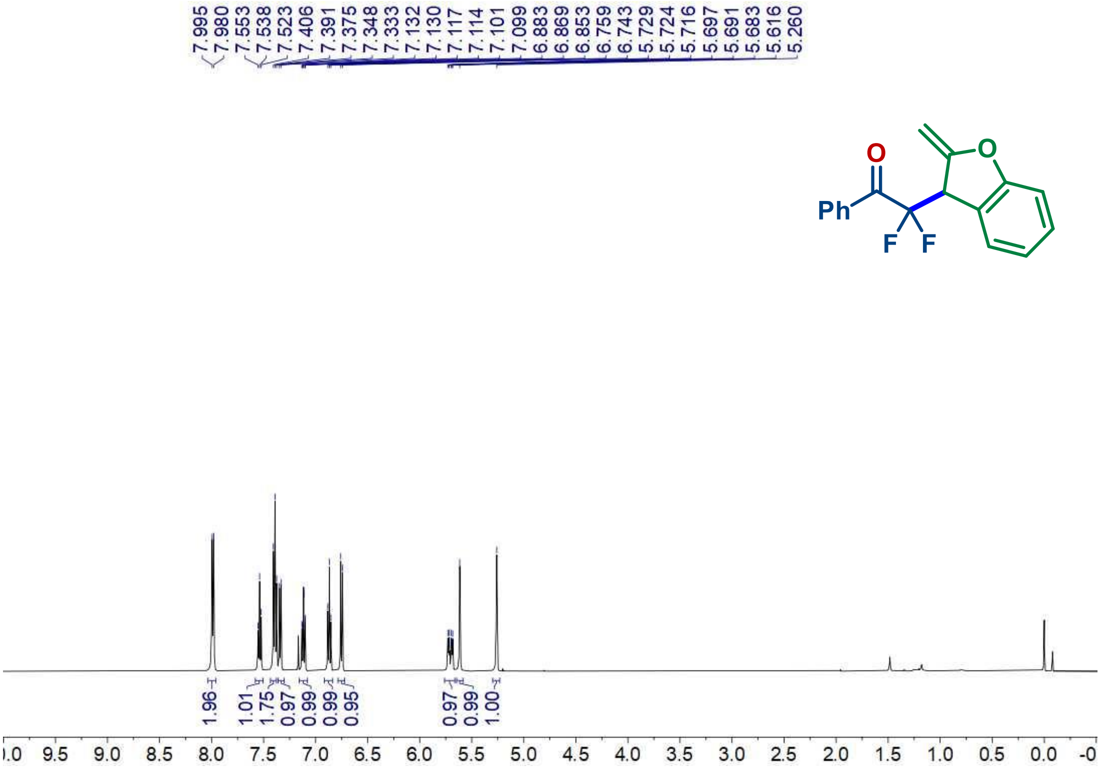

Supplementary Fig. 234 ^{1}H NMR (500 MHz, $CDCl_3$) spectrum of compound **78**

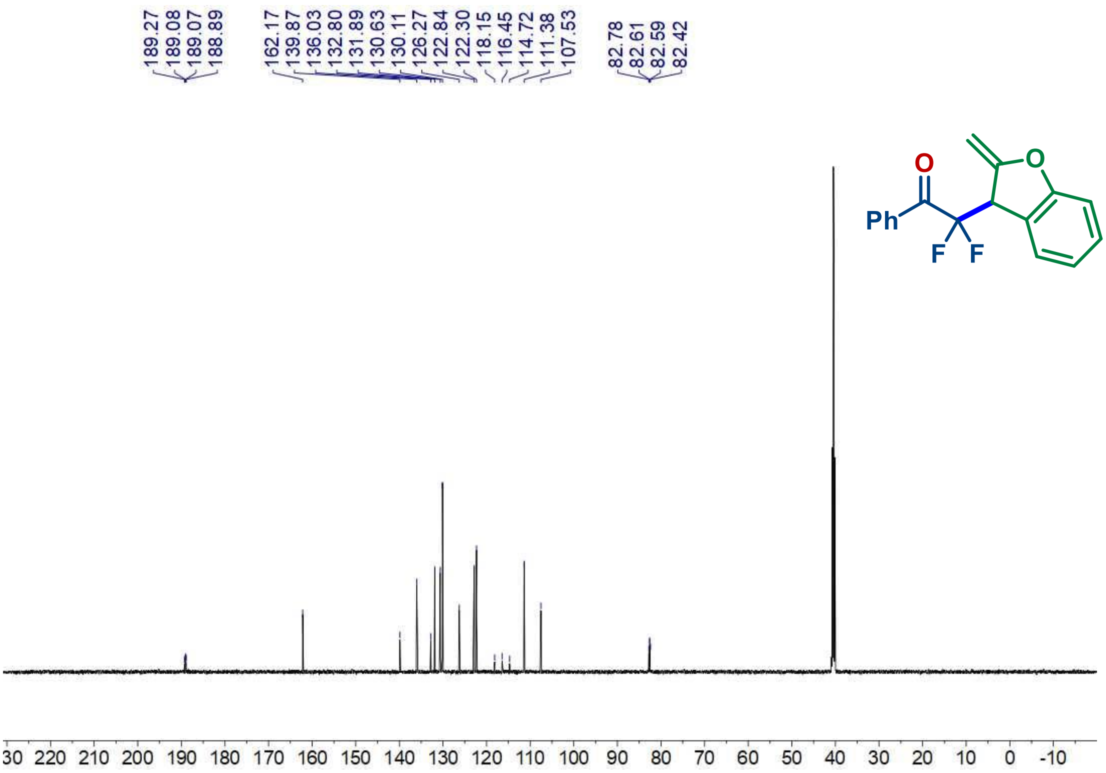

Supplementary Fig. 235 ^{13}C NMR (150 MHz, DMSO) spectrum of compound **78**

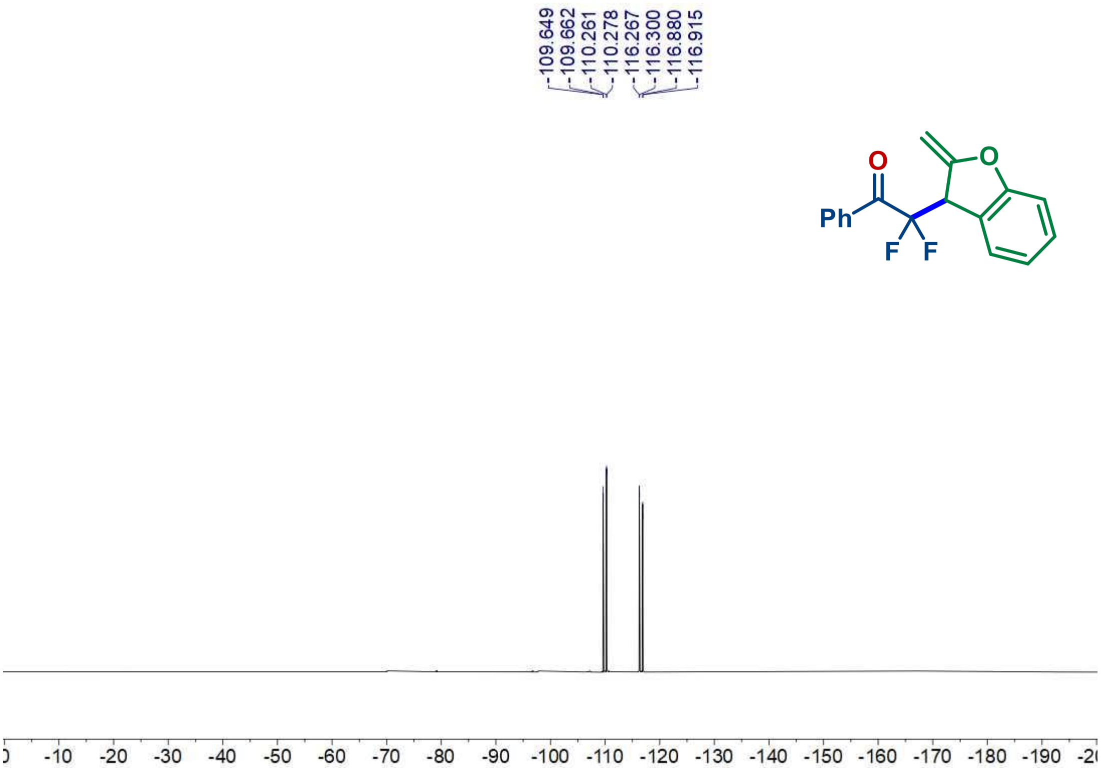

Supplementary Fig. 236 ^{19}F NMR (470 MHz, $CDCl_3$) spectrum of compound **78**

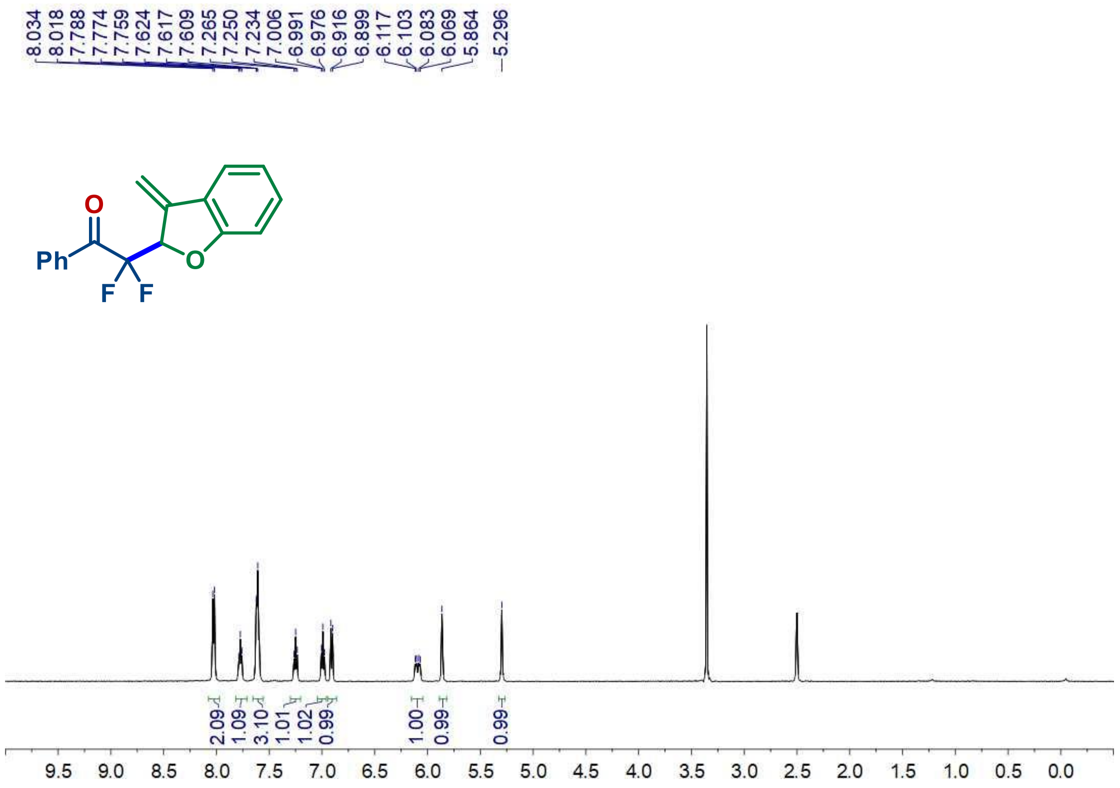

Supplementary Fig. 237 ^{1}H NMR (500 MHz, DMSO) spectrum of compound **79**

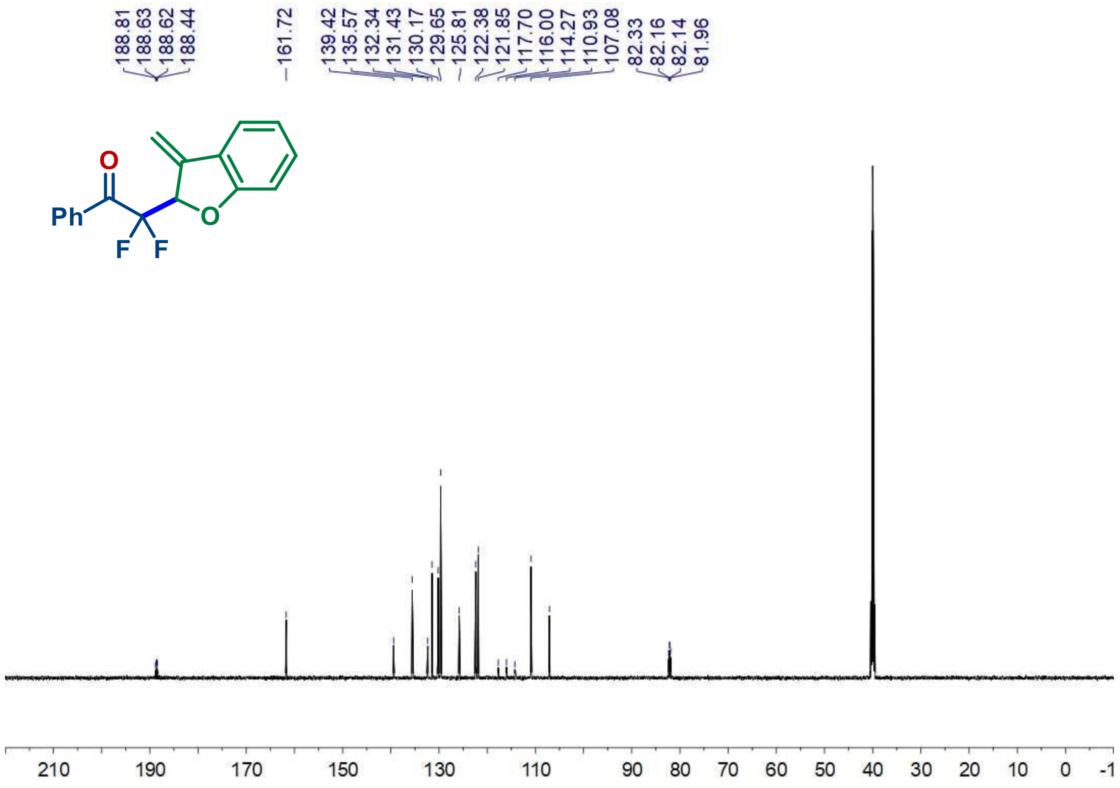

Supplementary Fig. 238 ^{13}C NMR (150 MHz, DMSO) spectrum of compound **79**

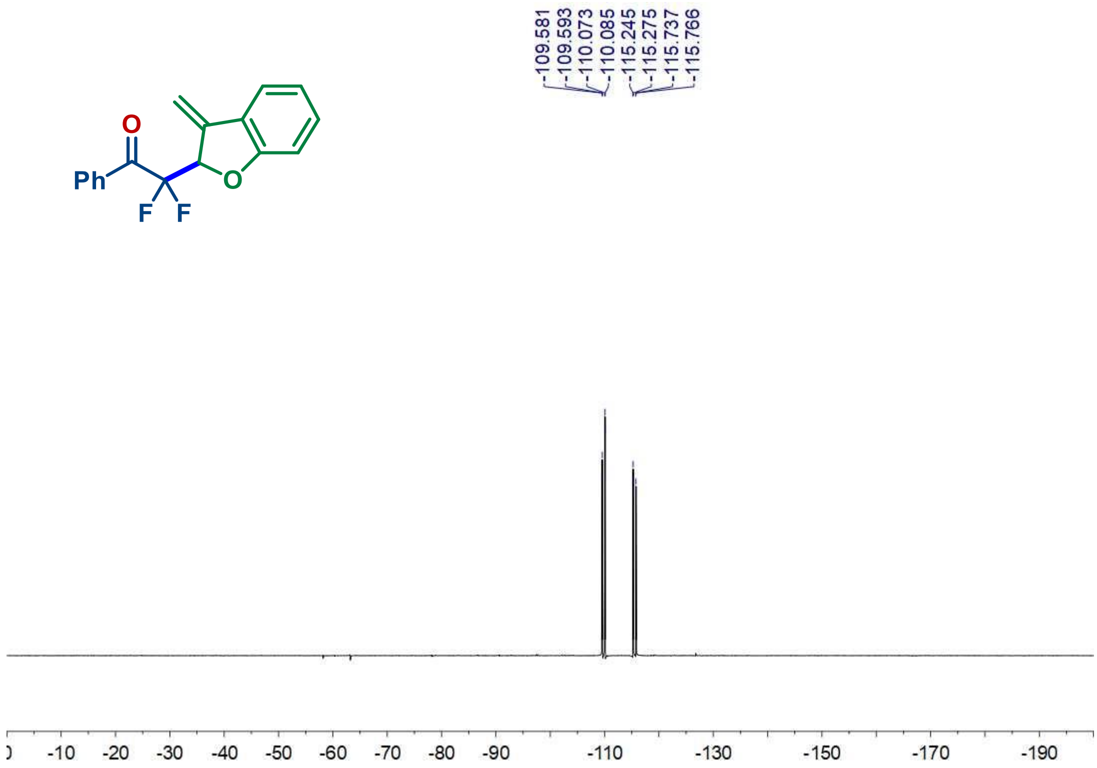

Supplementary Fig. 239 ^{19}F NMR (564 MHz, DMSO) spectrum of compound **79**

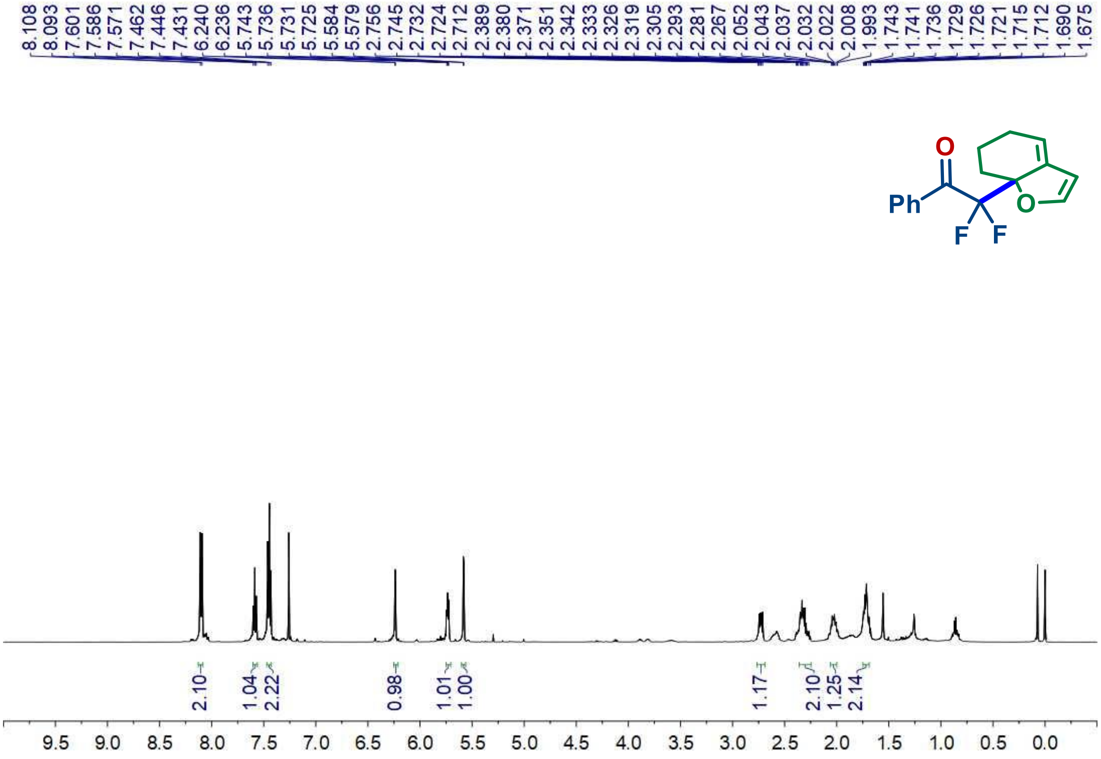

Supplementary Fig. 240 ^{1}H NMR (500 MHz, $CDCl_3$) spectrum of compound **80**

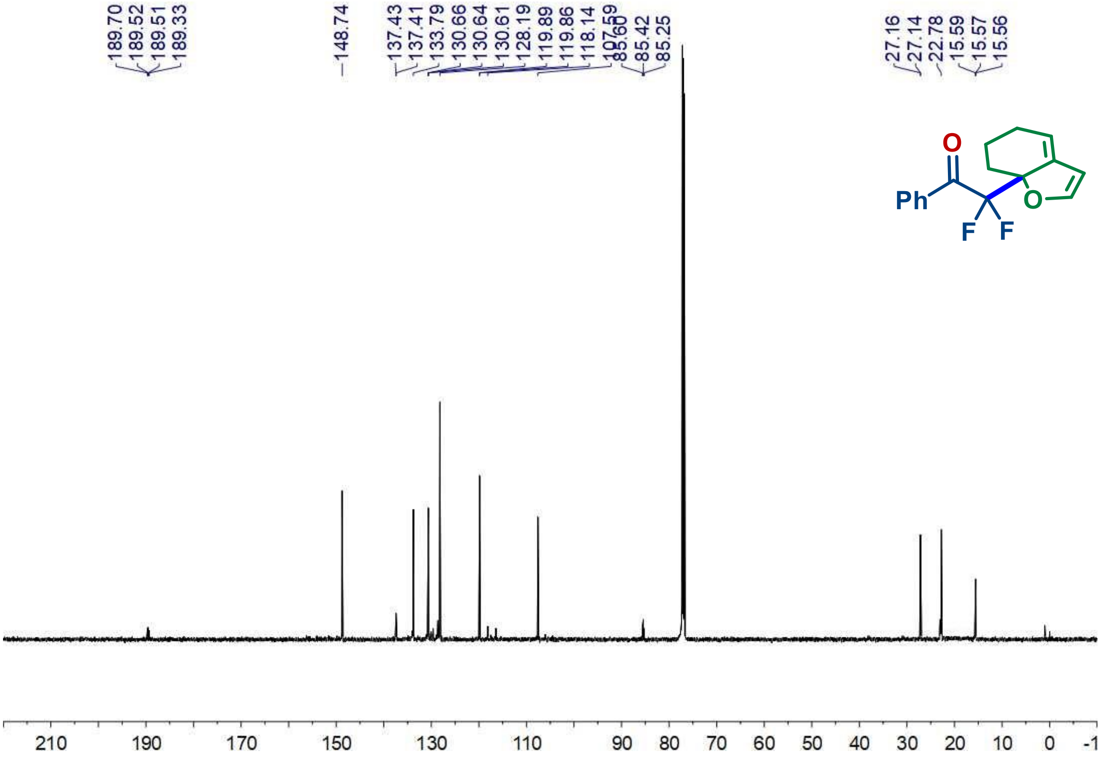

Supplementary Fig. 241 ^{13}C NMR (150 MHz, $CDCl_3$) spectrum of compound **80**

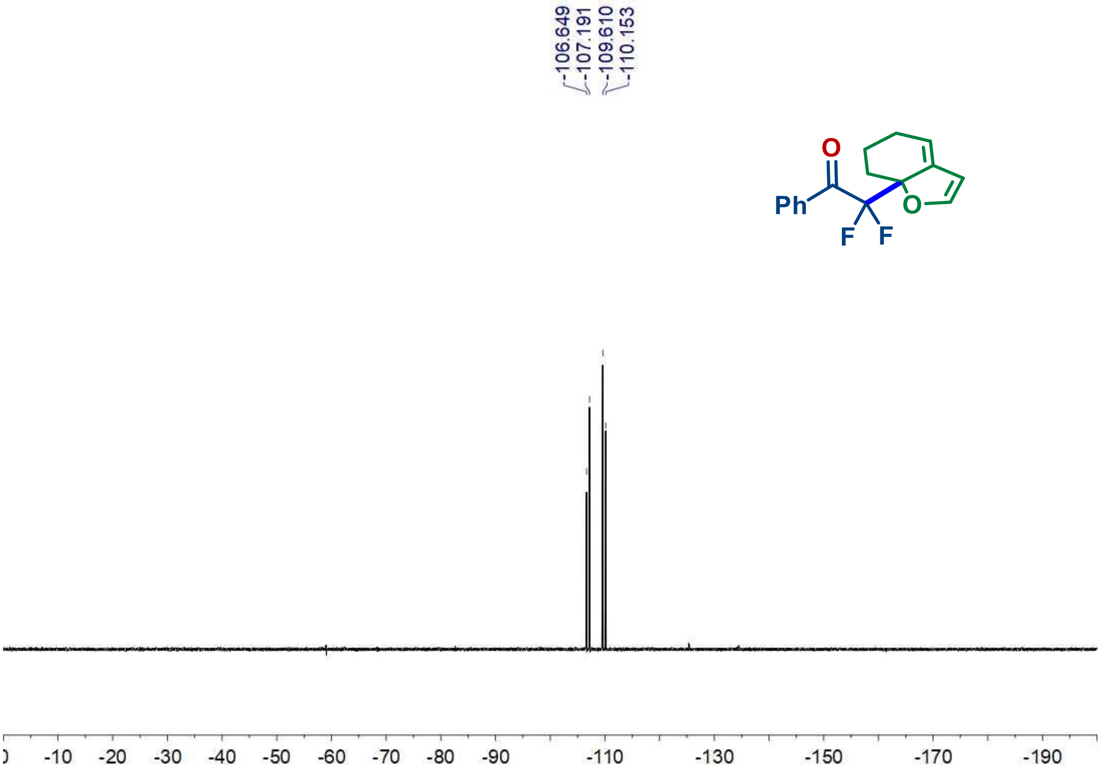

Supplementary Fig. 242 ^{19}F NMR (470 MHz, $CDCl_3$) spectrum of compound **80**

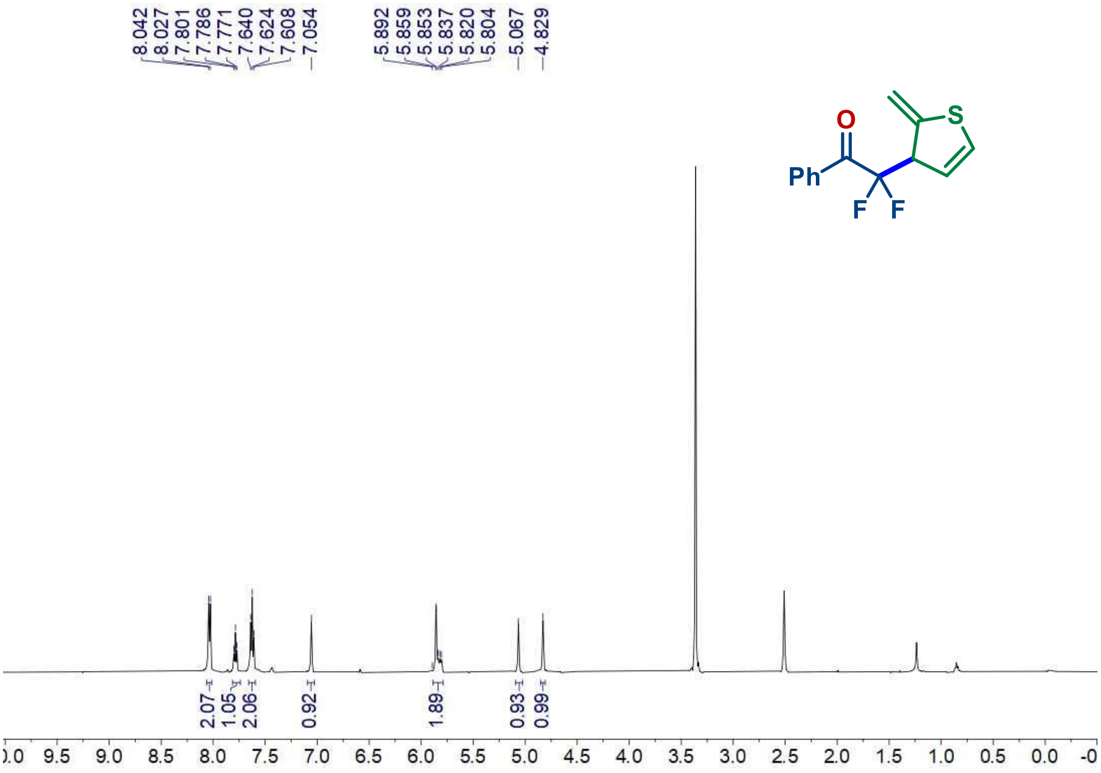

Supplementary Fig. 243 ^{1}H NMR (500 MHz, DMSO) spectrum of compound **81**

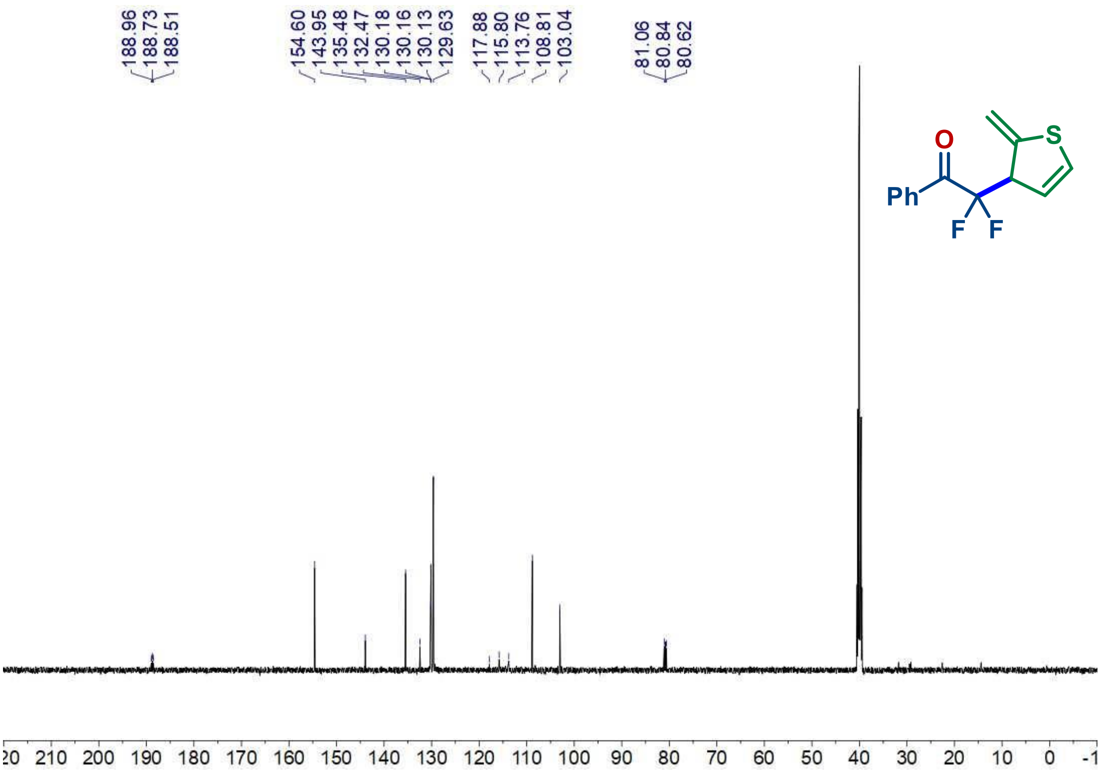

Supplementary Fig. 244 ^{13}C NMR (125 MHz, DMSO) spectrum of compound **81**

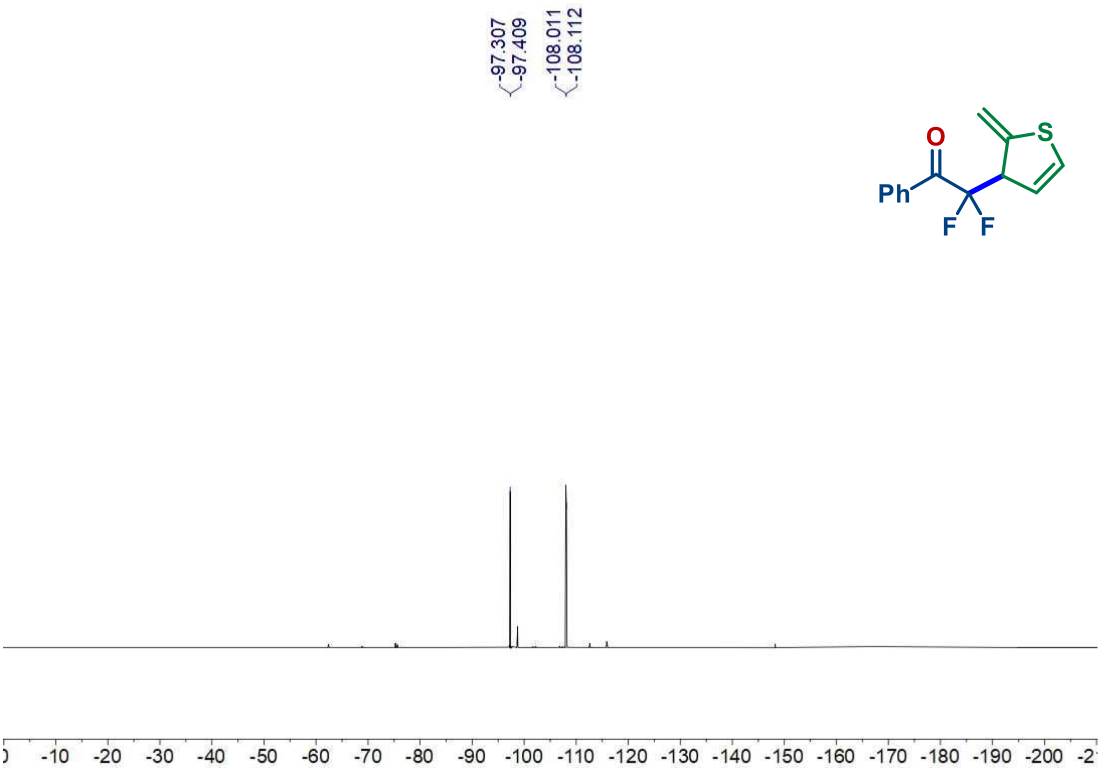

Supplementary Fig. 245 ^{19}F NMR (470 MHz, DMSO) spectrum of compound **81**

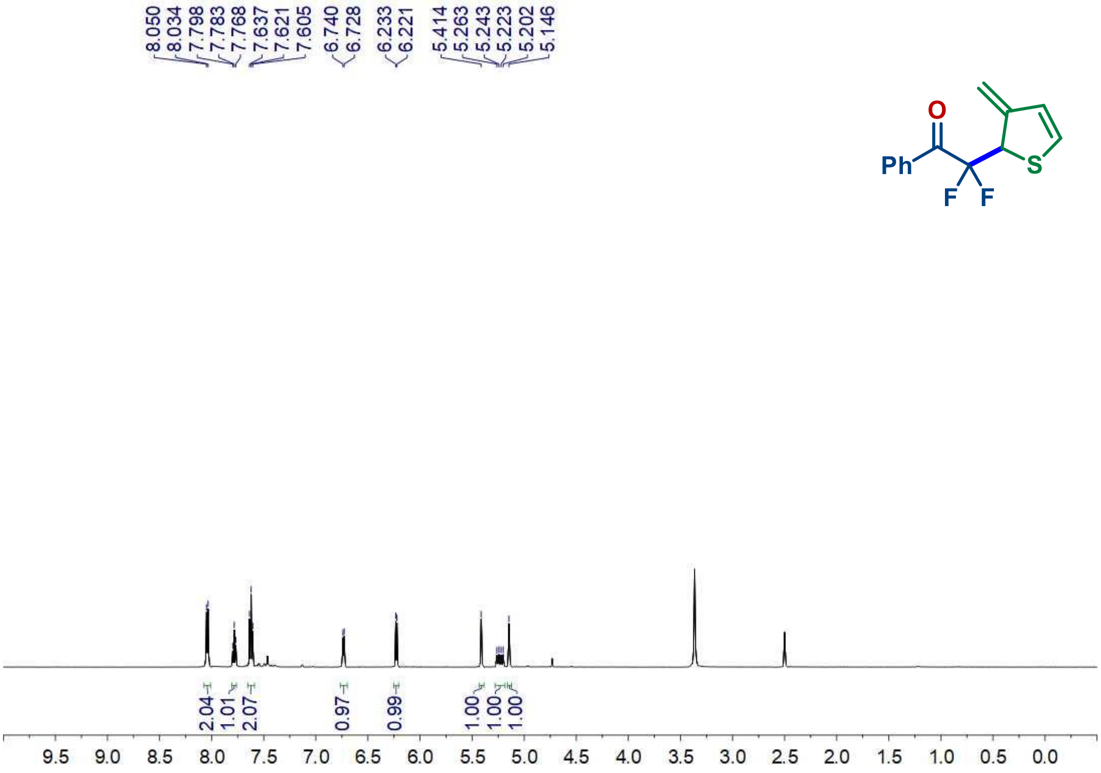

Supplementary Fig. 246 ^{1}H NMR (500 MHz, DMSO) spectrum of compound **82**

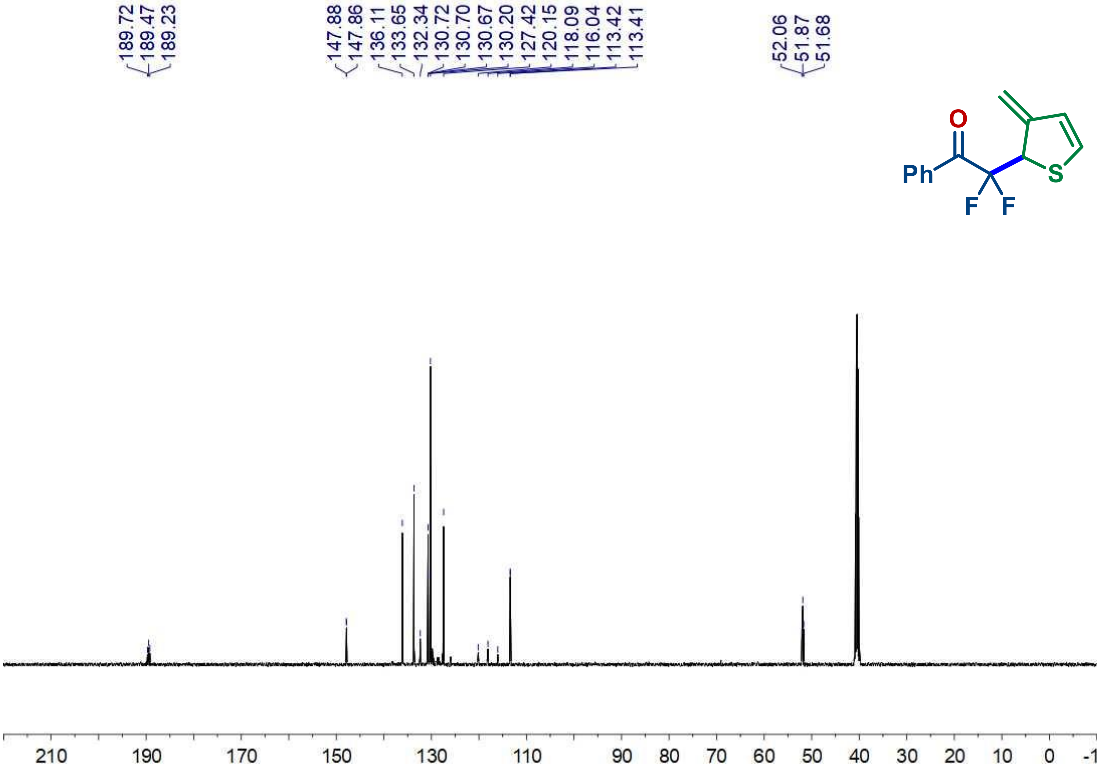

Supplementary Fig. 247 ^{13}C NMR (125 MHz, DMSO) spectrum of compound **82**

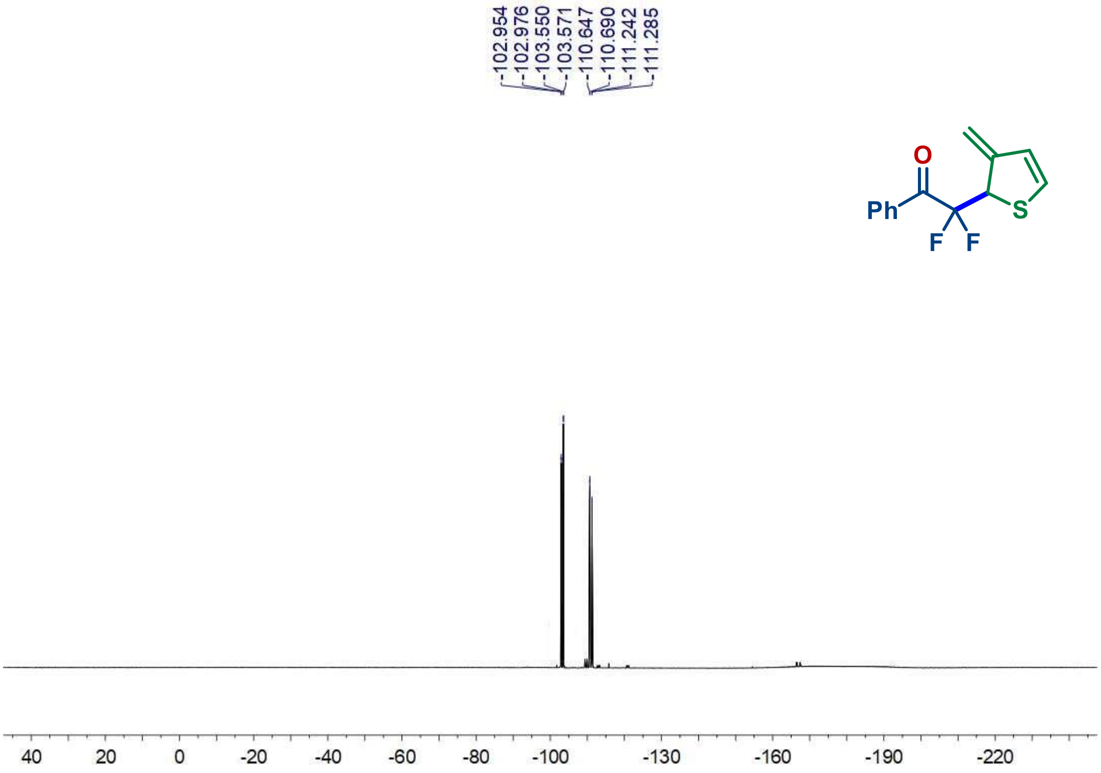

Supplementary Fig. 248 ^{19}F NMR (470 MHz, DMSO) spectrum of compound **82**

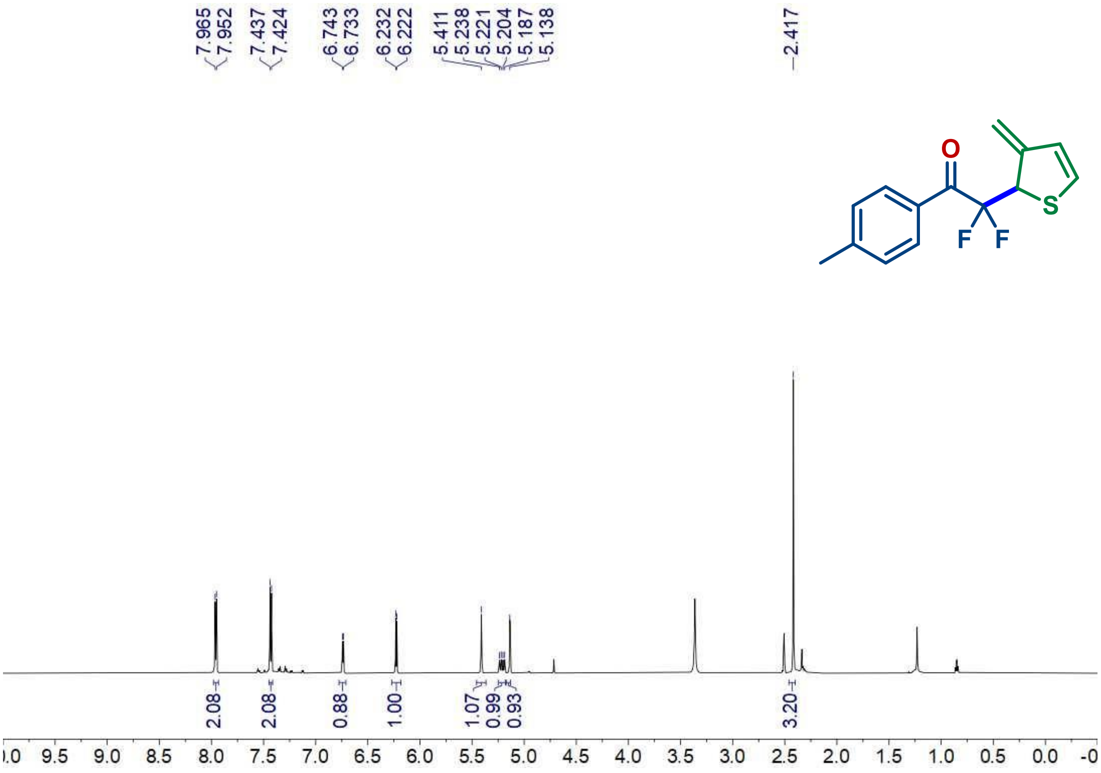

Supplementary Fig. 249 ^{1}H NMR (600 MHz, DMSO) spectrum of compound **83**

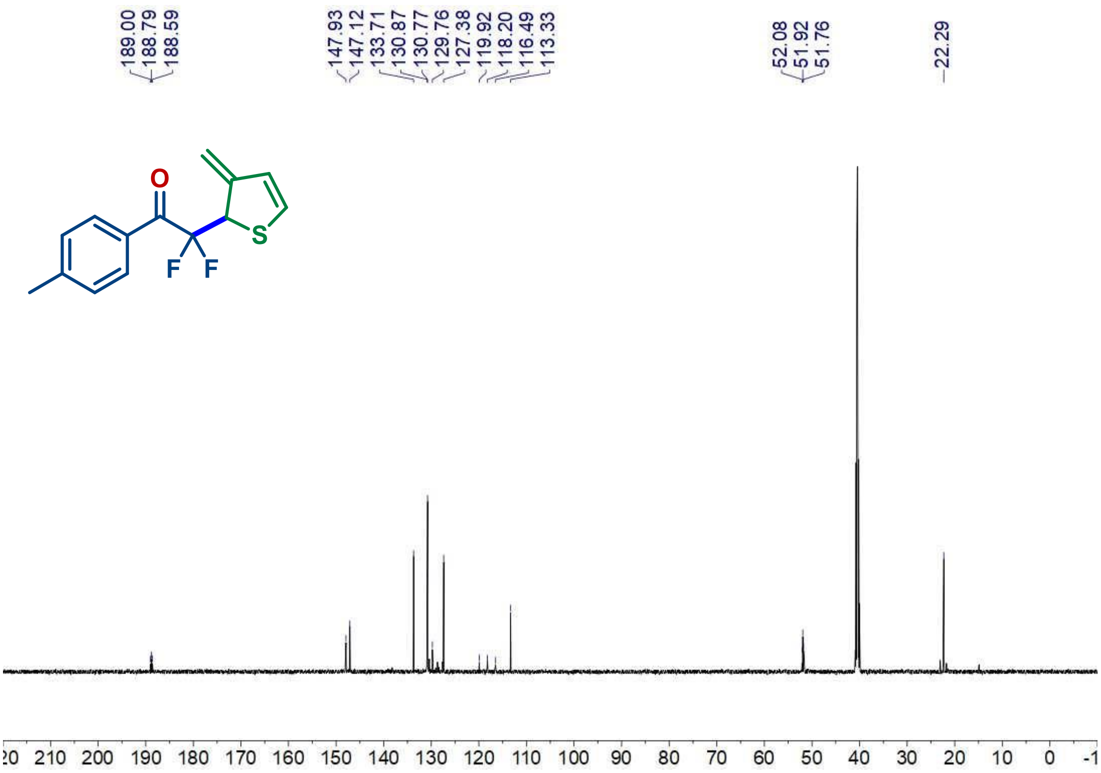

Supplementary Fig. 250 ^{13}C NMR (150 MHz, DMSO) spectrum of compound **83**

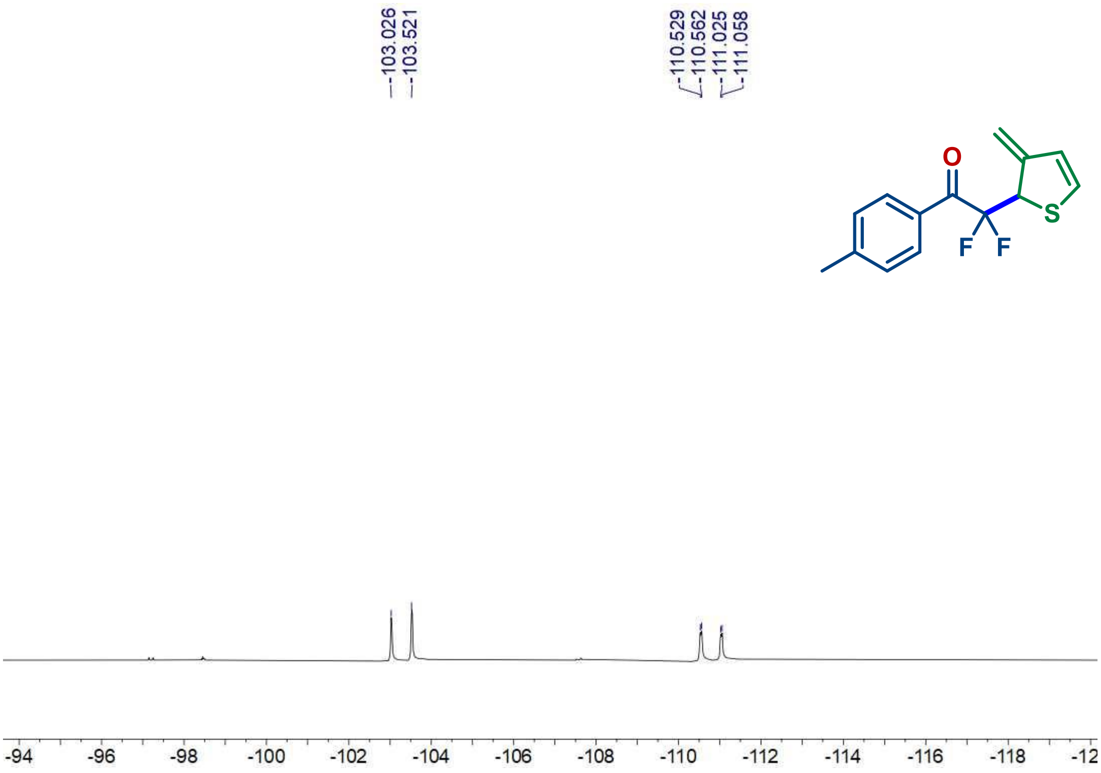

Supplementary Fig. 251 ^{19}F NMR (564 MHz, DMSO) spectrum of compound **83**

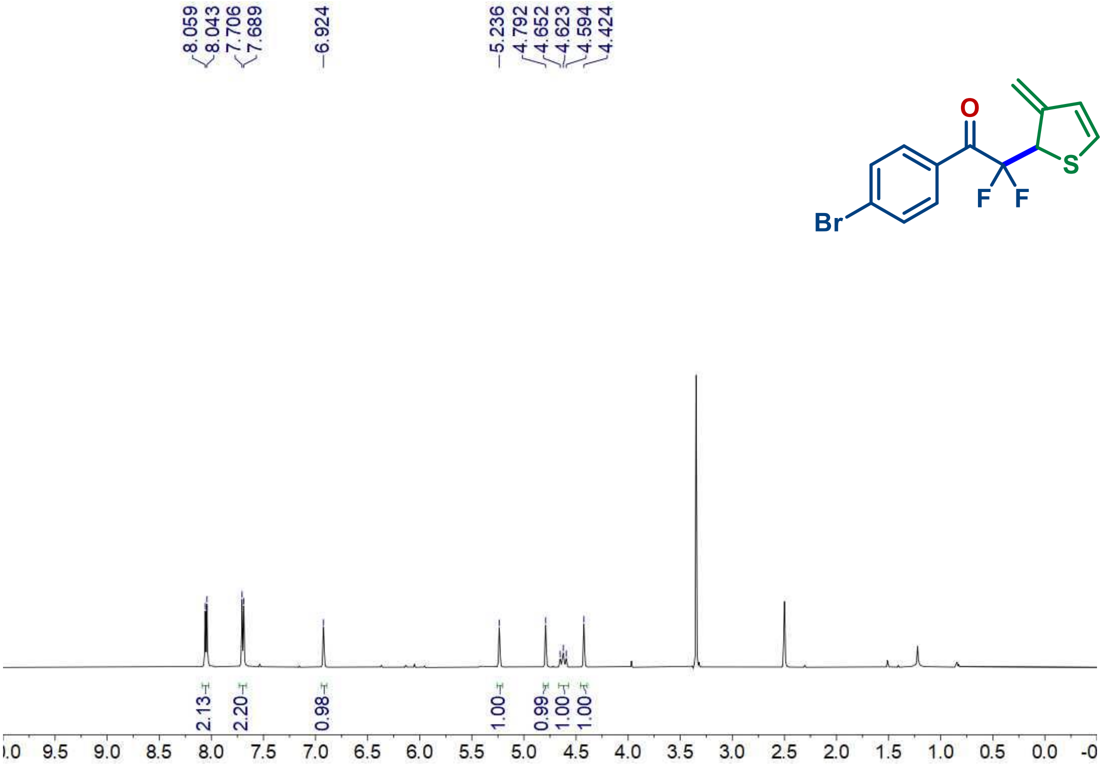

Supplementary Fig. 252 ^{1}H NMR (500 MHz, DMSO) spectrum of compound **84**

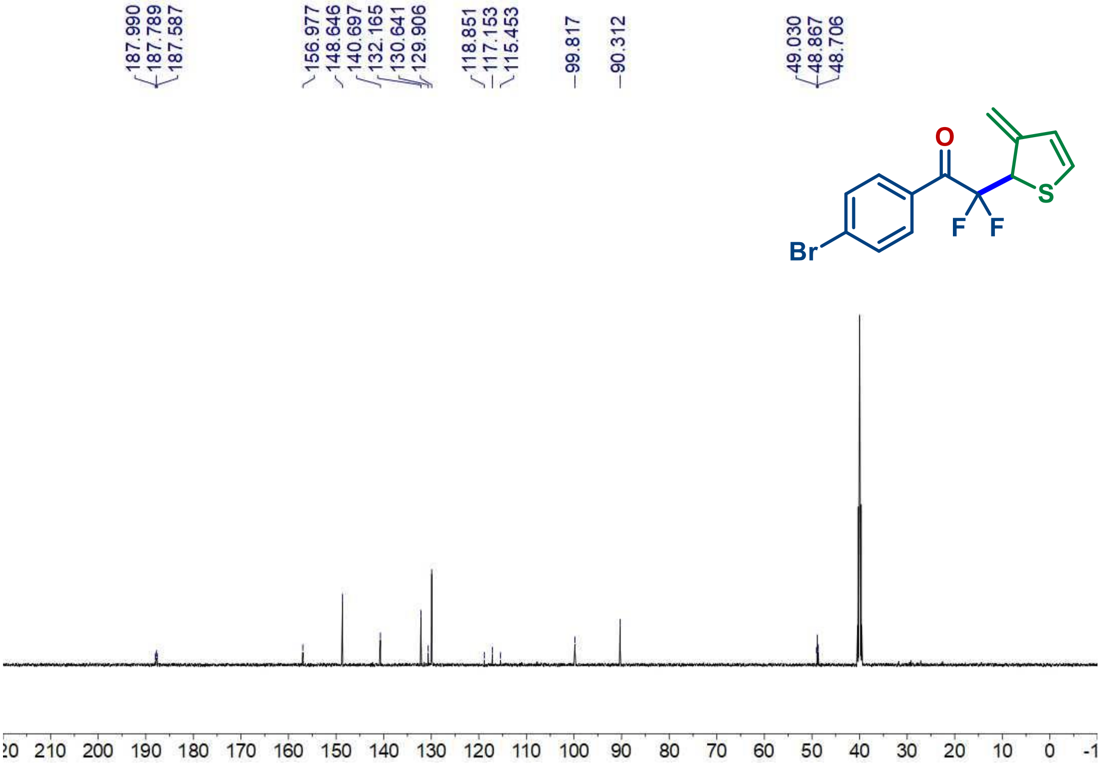

Supplementary Fig. 253 ^{13}C NMR (150 MHz, DMSO) spectrum of compound **84**

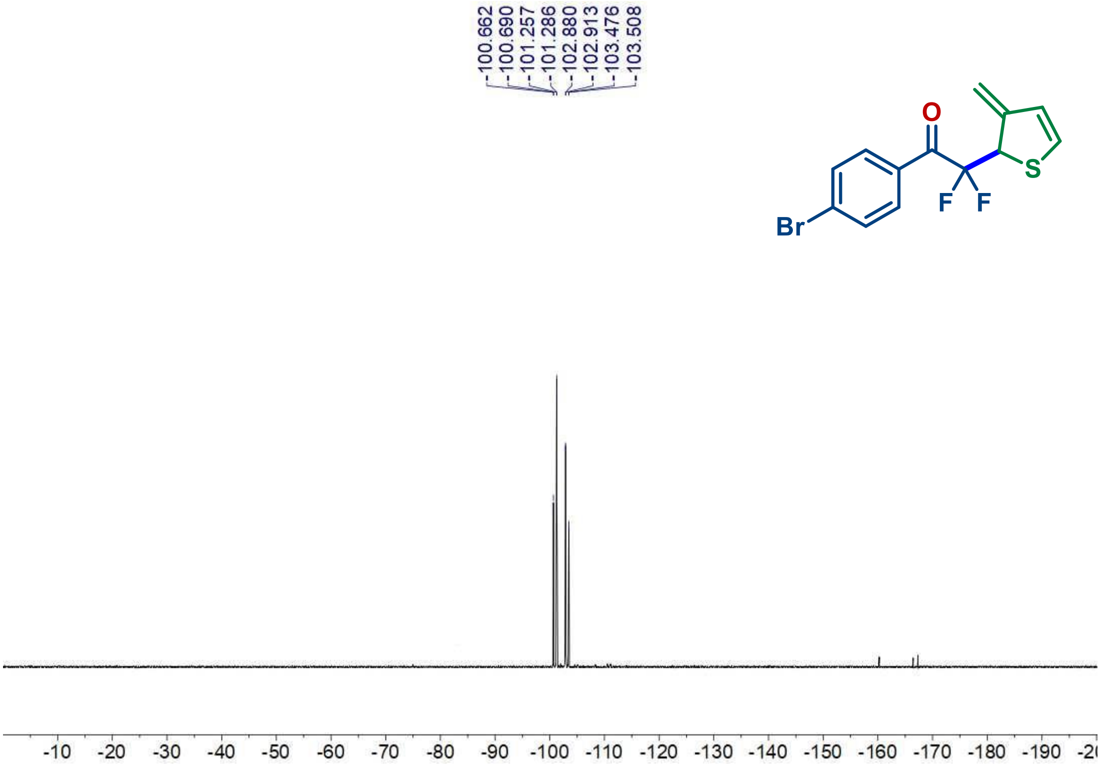

Supplementary Fig. 254 ^{19}F NMR (470 MHz, DMSO) spectrum of compound **84**

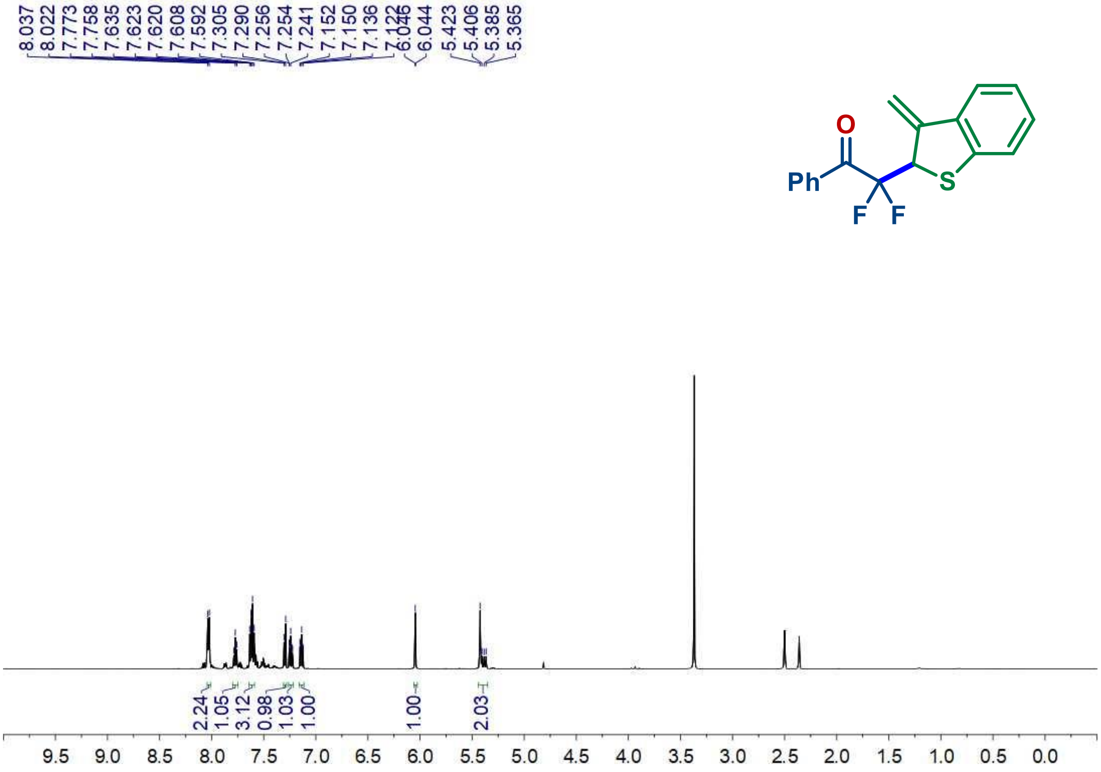

Supplementary Fig. 255 ^{1}H NMR (500 MHz, DMSO) spectrum of compound **85**

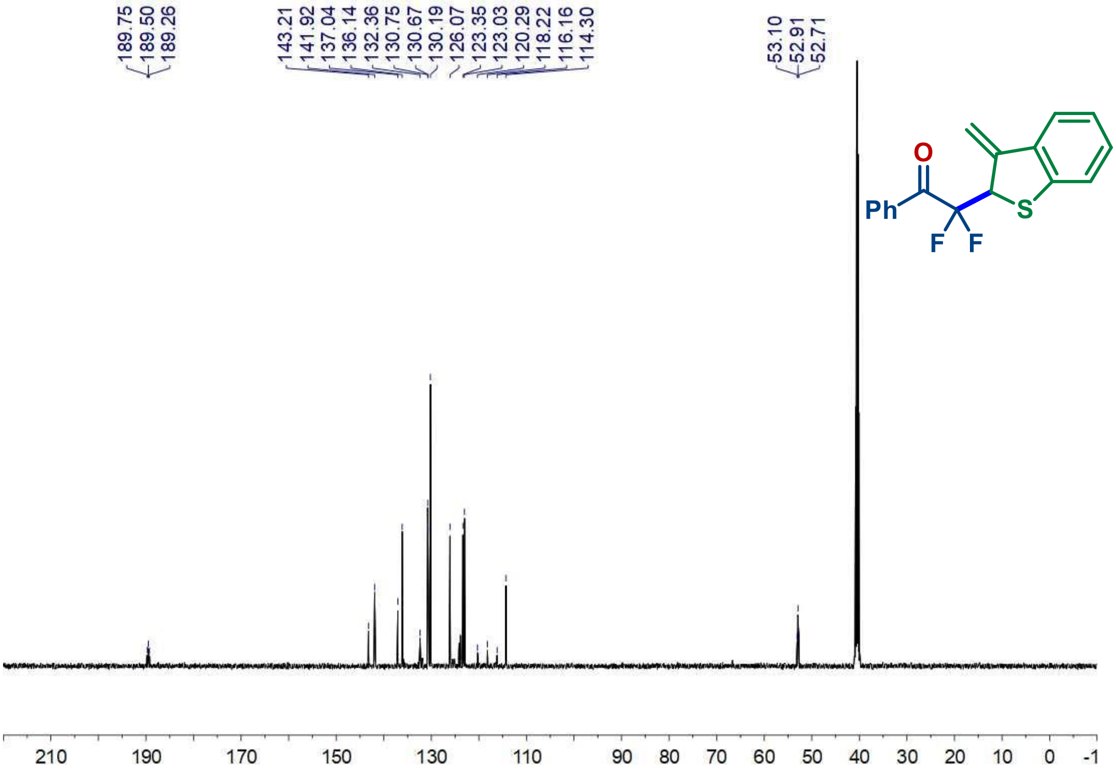

Supplementary Fig. 256 ^{13}C NMR (125 MHz, DMSO) spectrum of compound **85**

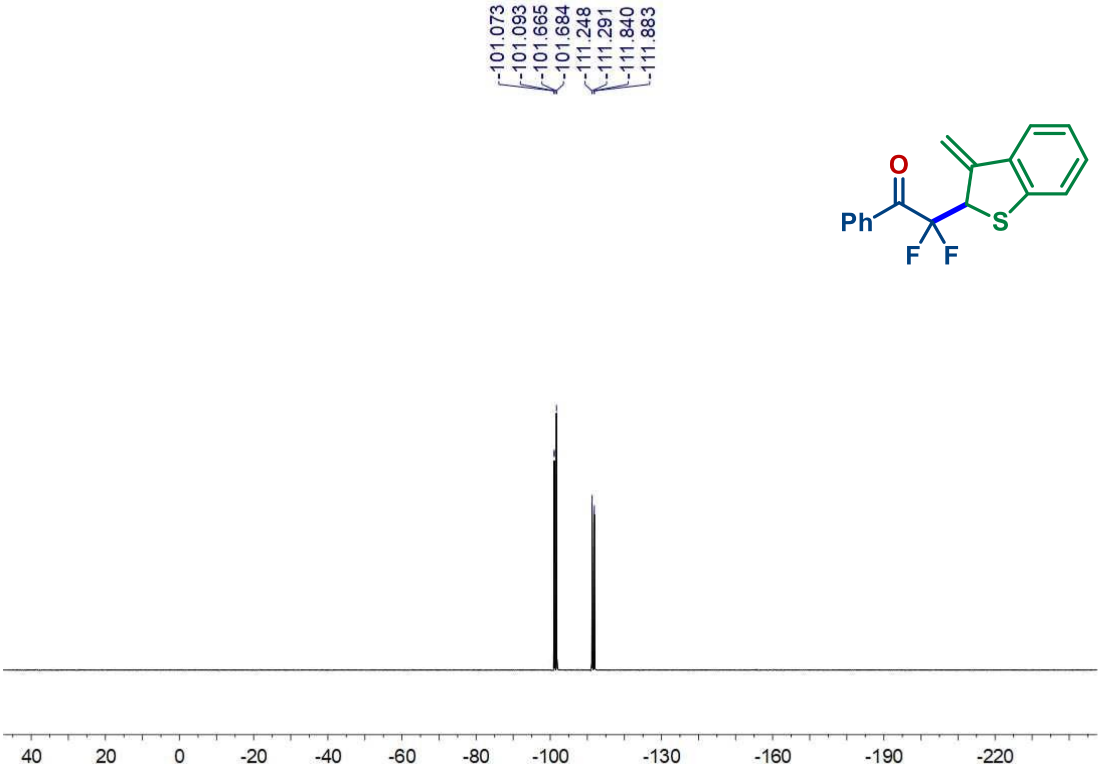

Supplementary Fig. 257 ^{19}F NMR (470 MHz, DMSO) spectrum of compound **85**

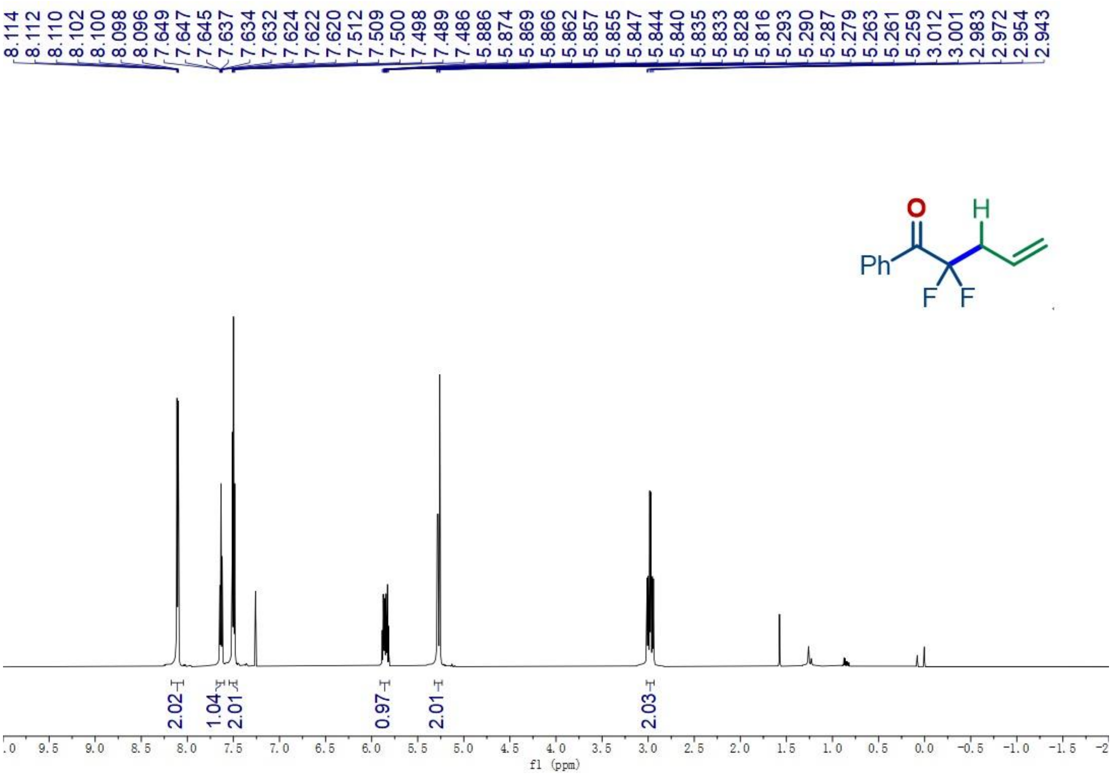

Supplementary Fig. 258 ^{1}H NMR (600 MHz, $CDCl_3$) spectrum of compound **86**

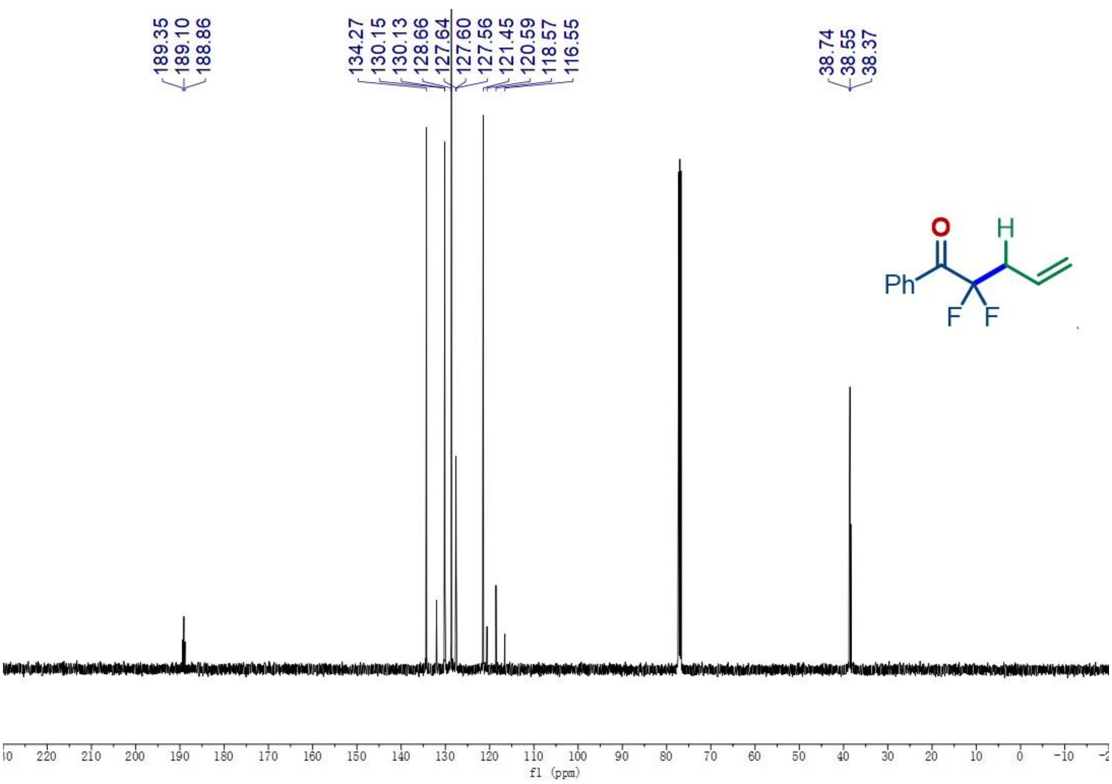

Supplementary Fig. 259 ^{13}C NMR (125 MHz, $CDCl_3$) spectrum of compound **86**

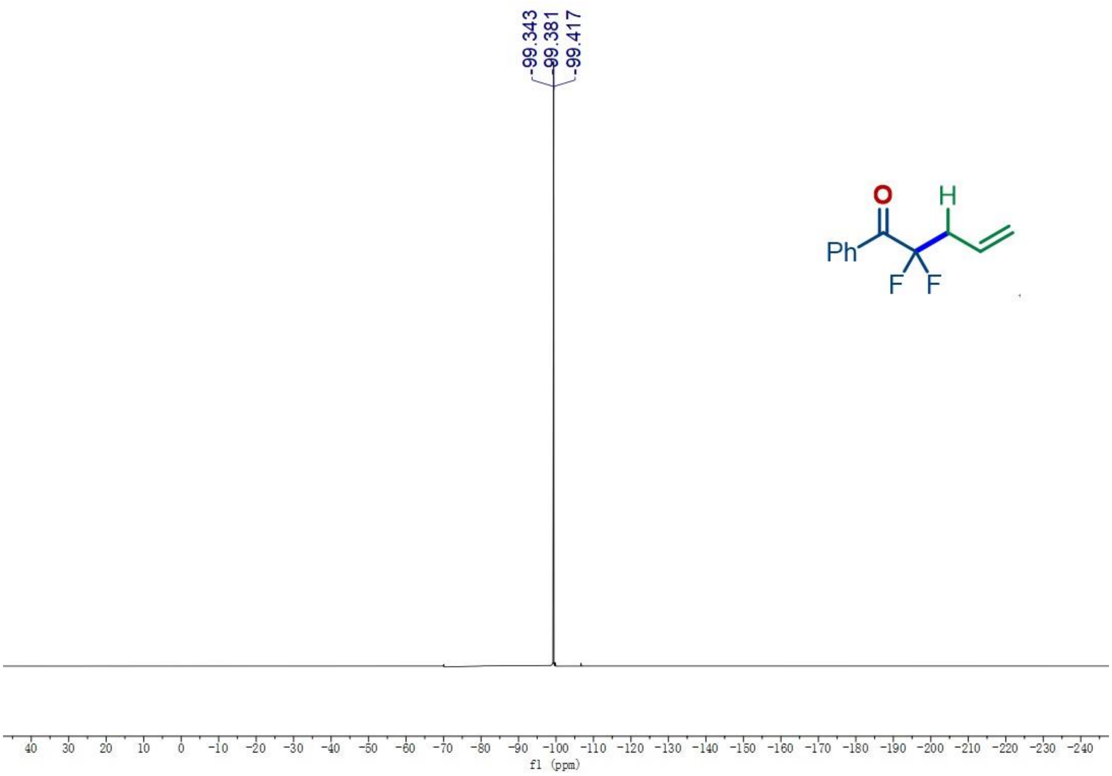

Supplementary Fig. 260 ^{19}F NMR (470 MHz, $CDCl_3$) spectrum of compound **86**

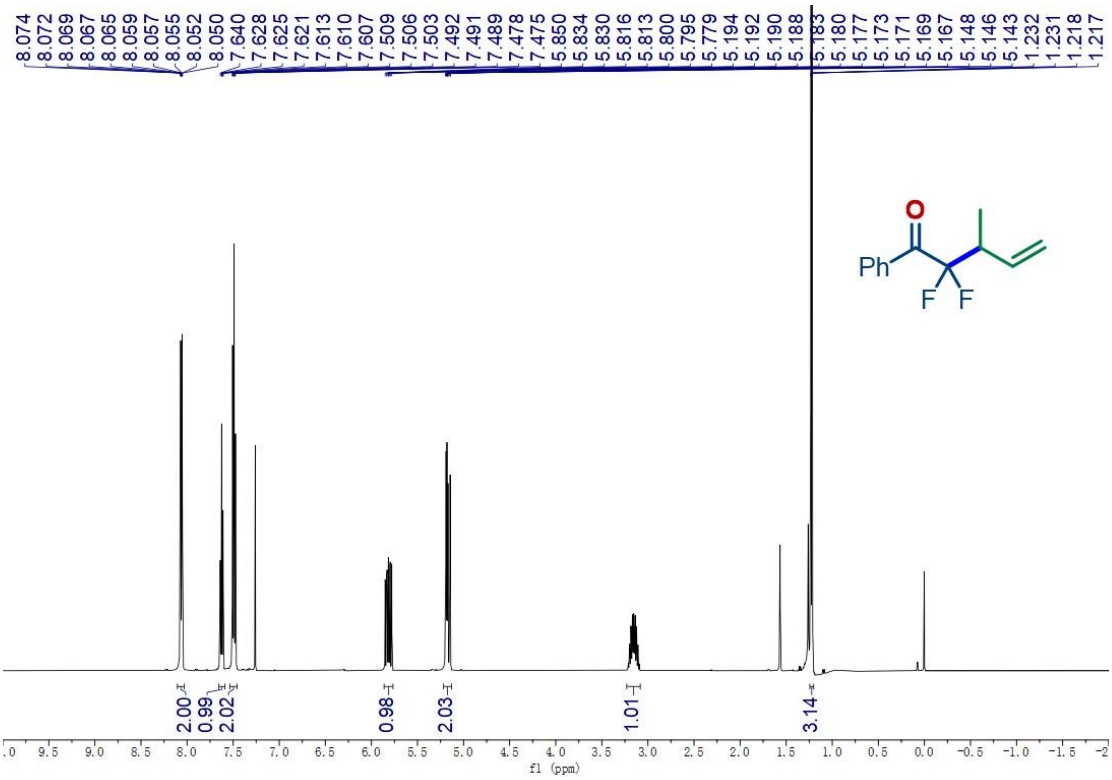

Supplementary Fig. 261 ^{1}H NMR (500 MHz, $CDCl_3$) spectrum of compound **87**

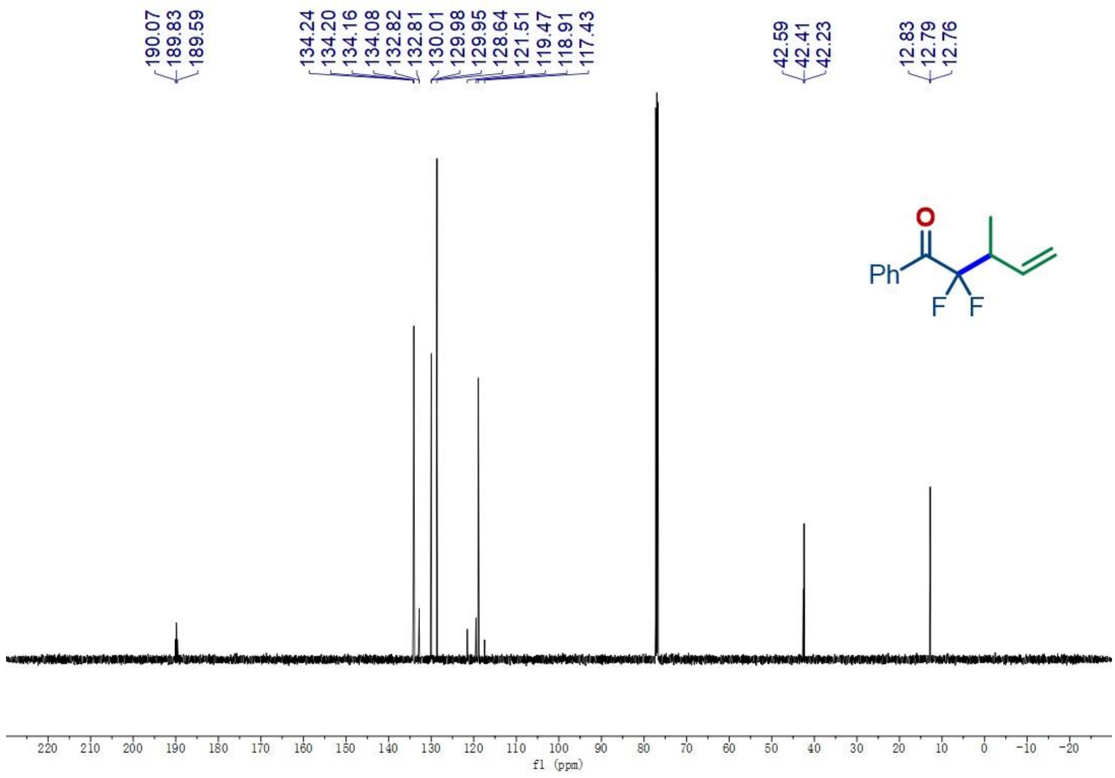

Supplementary Fig. 262 ^{13}C NMR (125 MHz, $CDCl_3$) spectrum of compound **87**

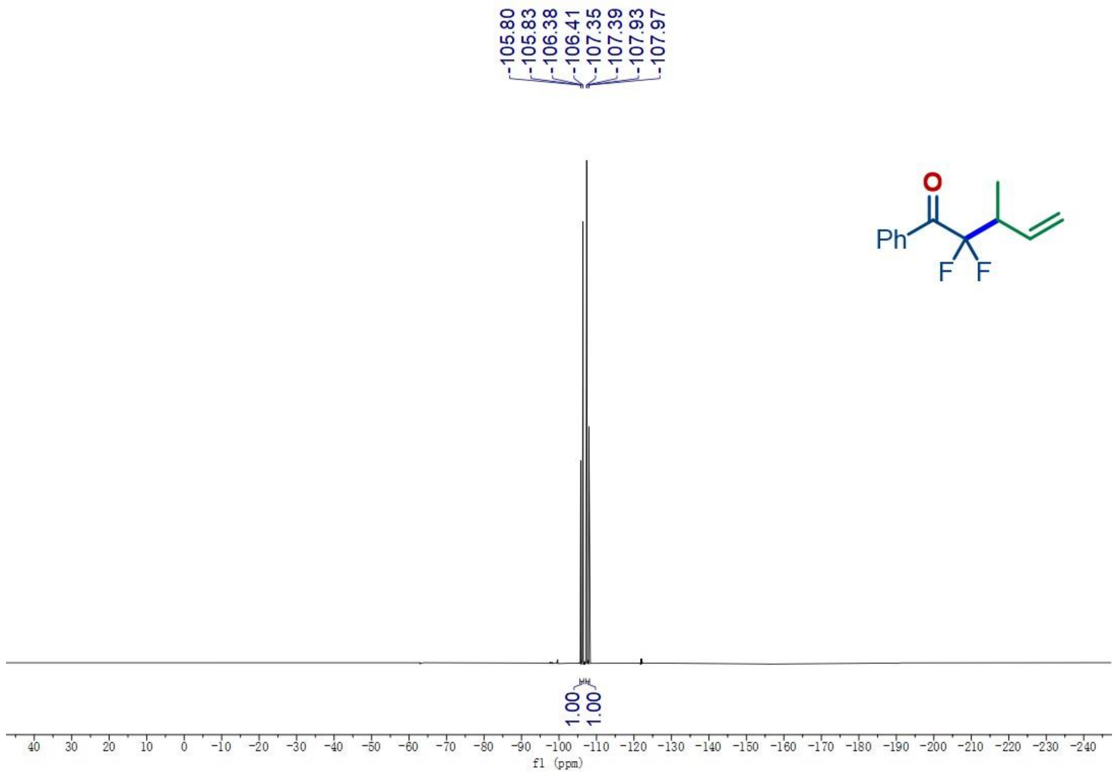

Supplementary Fig. 263 ^{19}F NMR (470 MHz, $CDCl_3$) spectrum of compound **87**

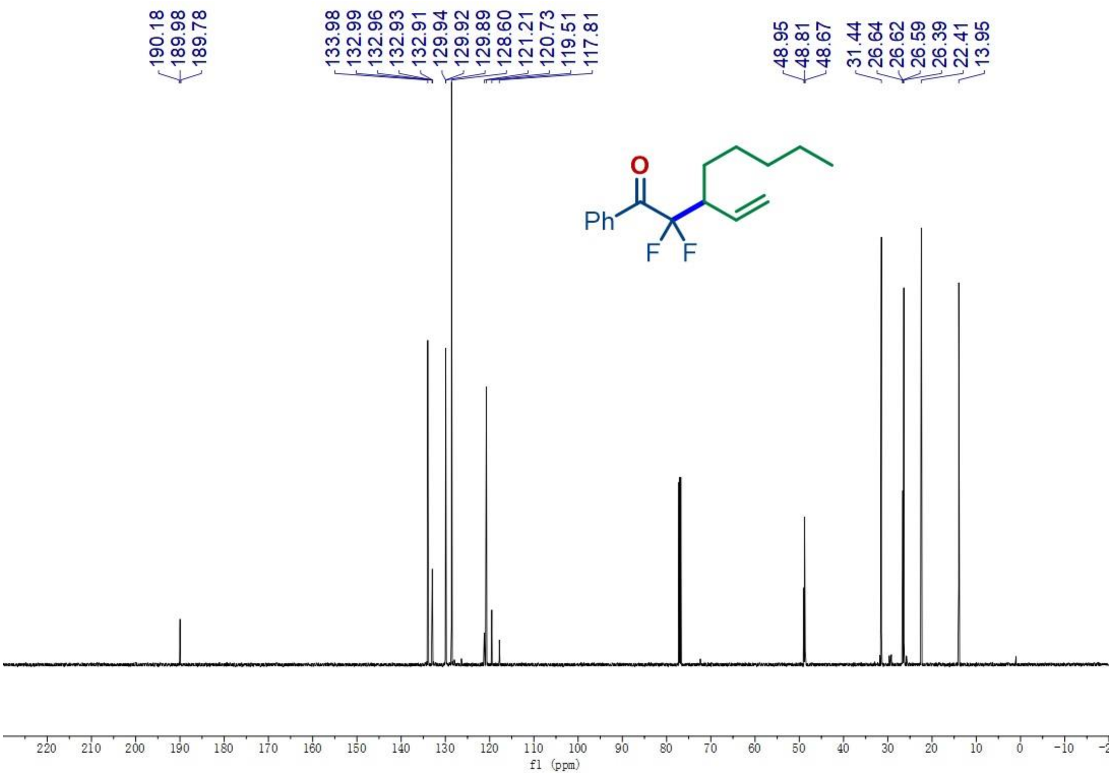

Supplementary Fig. 264 ^{1}H NMR (500 MHz, $CDCl_3$) spectrum of compound **88**

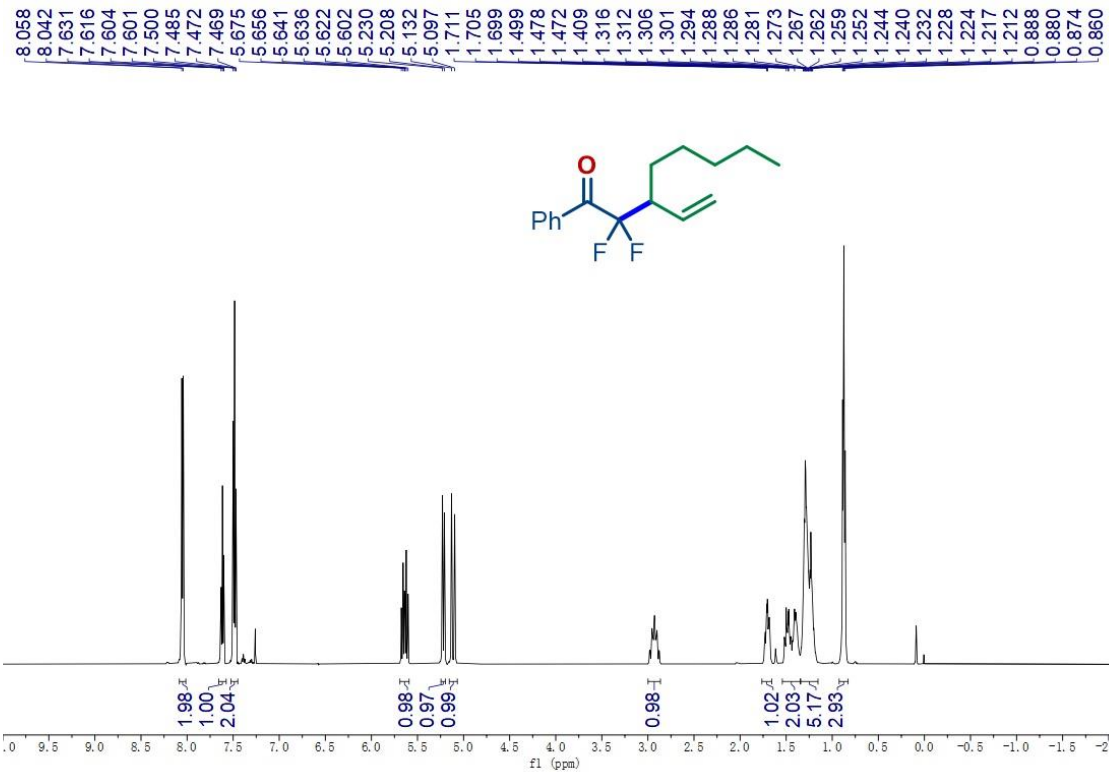

Supplementary Fig. 265 ^{13}C NMR (150 MHz, $CDCl_3$) spectrum of compound **88**

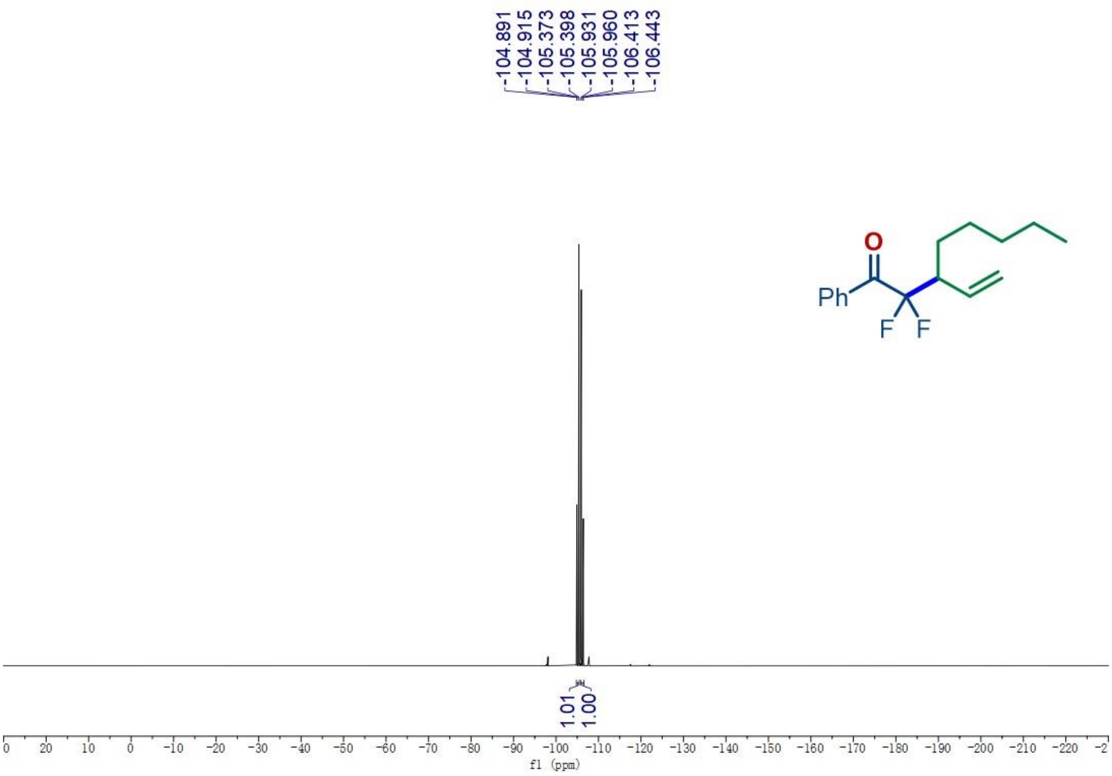

Supplementary Fig. 266 ^{19}F NMR (564 MHz, $CDCl_3$) spectrum of compound **88**

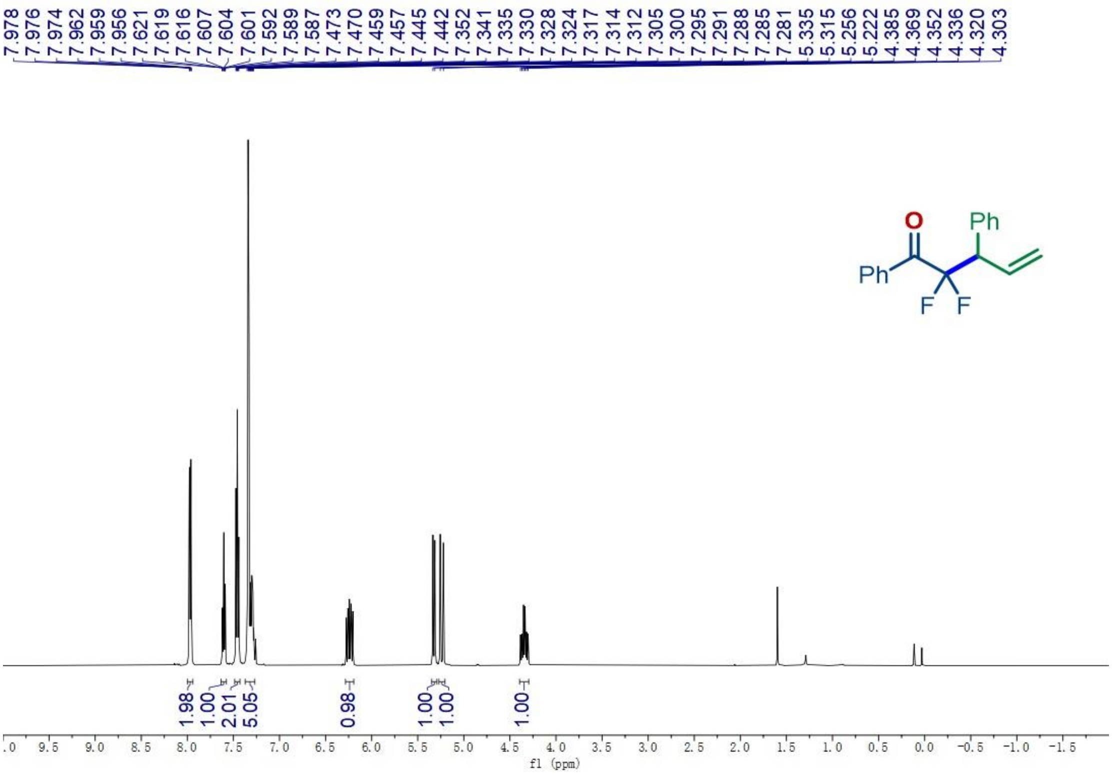

Supplementary Fig. 267 ^{1}H NMR (500 MHz, $CDCl_3$) spectrum of compound **89**

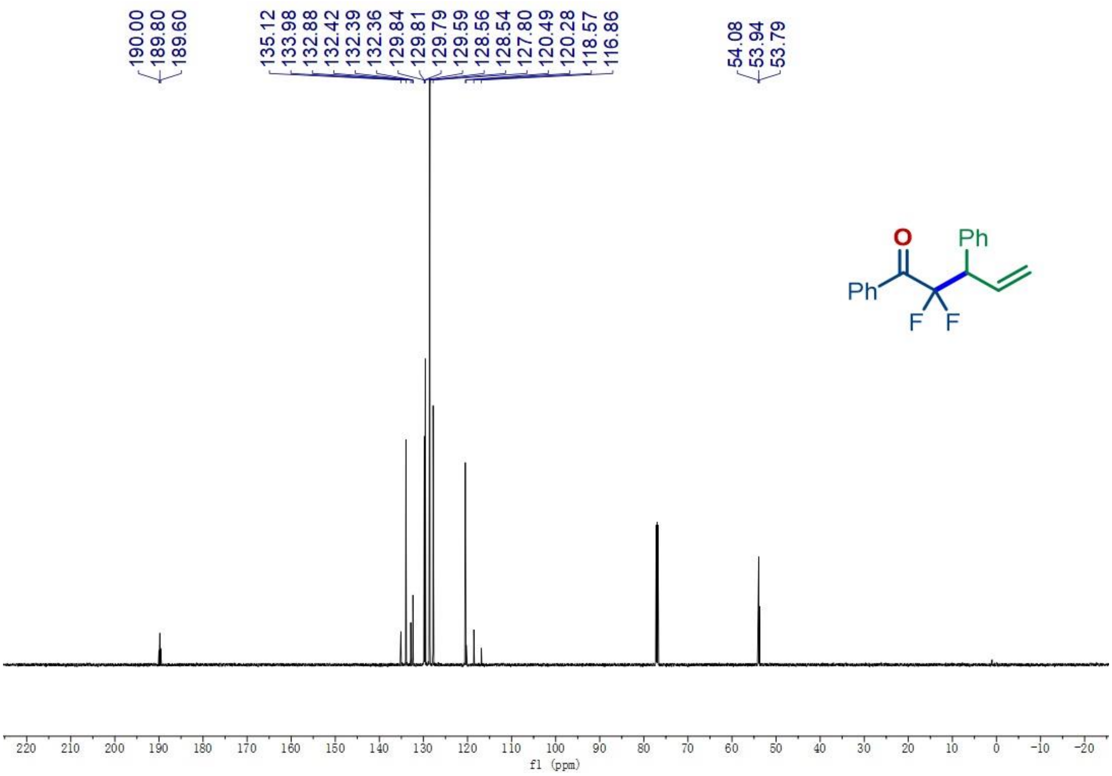

Supplementary Fig. 268 ^{13}C NMR (150 MHz, $CDCl_3$) spectrum of compound **89**

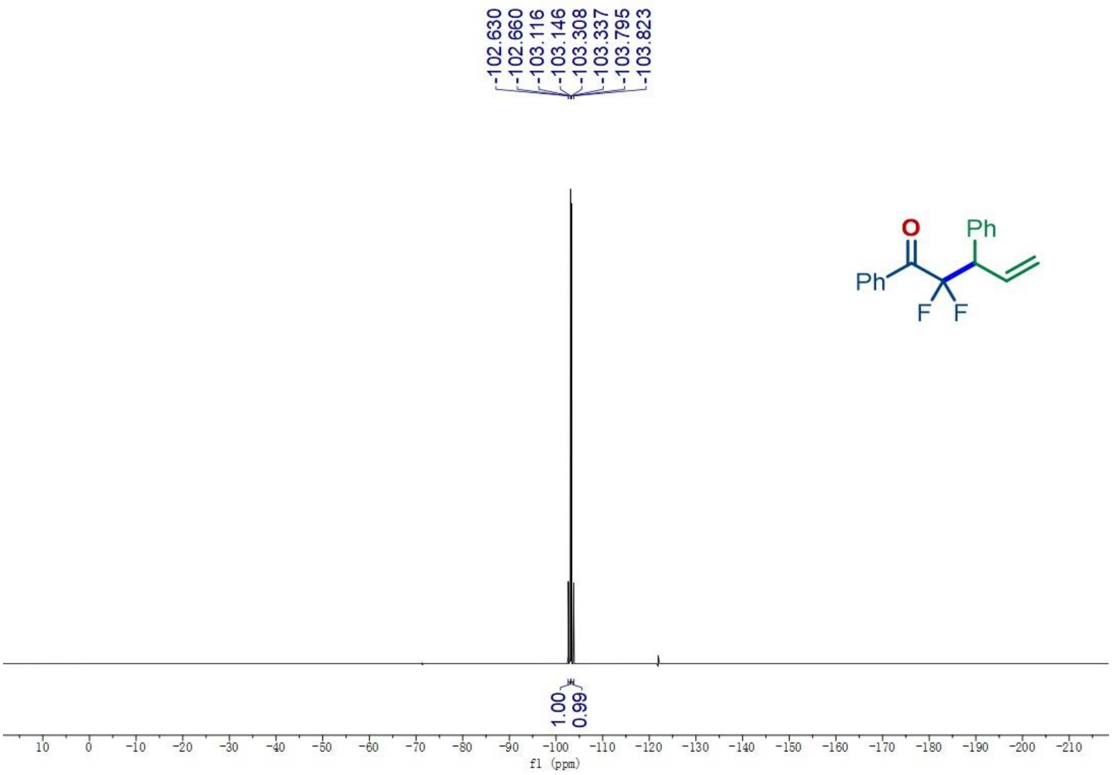

Supplementary Fig. 269 ^{19}F NMR (564 MHz, $CDCl_3$) spectrum of compound **89**

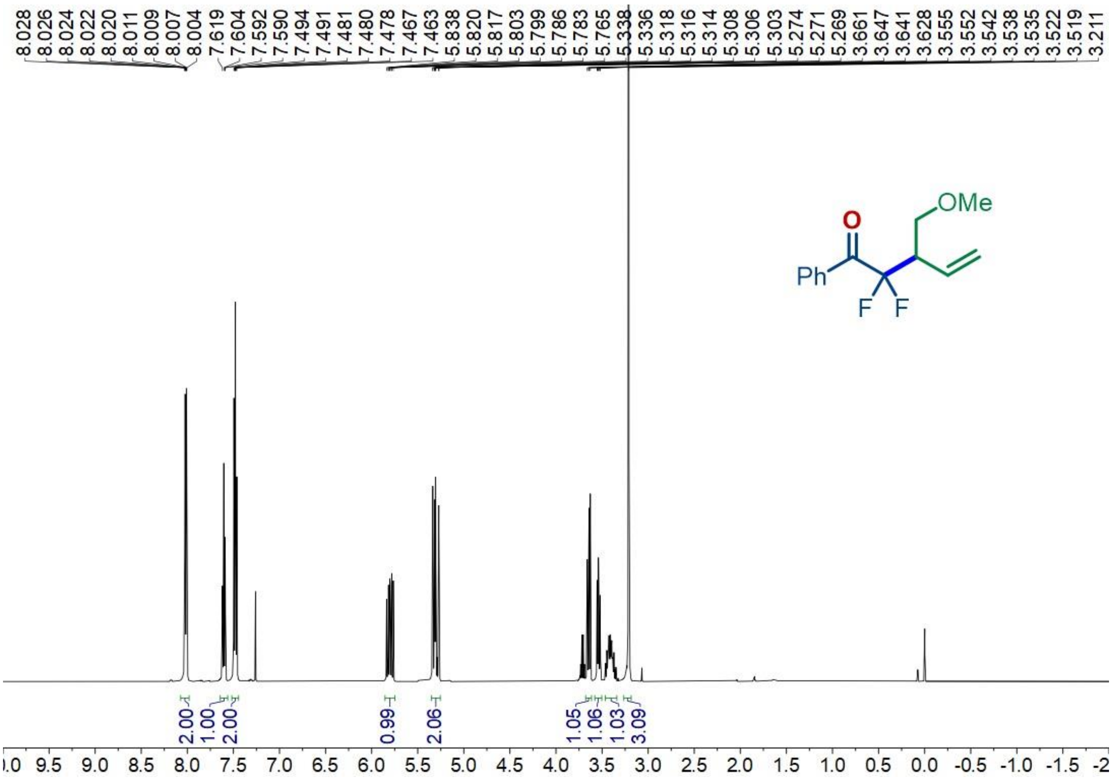

Supplementary Fig. 270 ^{1}H NMR (500 MHz, $CDCl_3$) spectrum of compound **90**

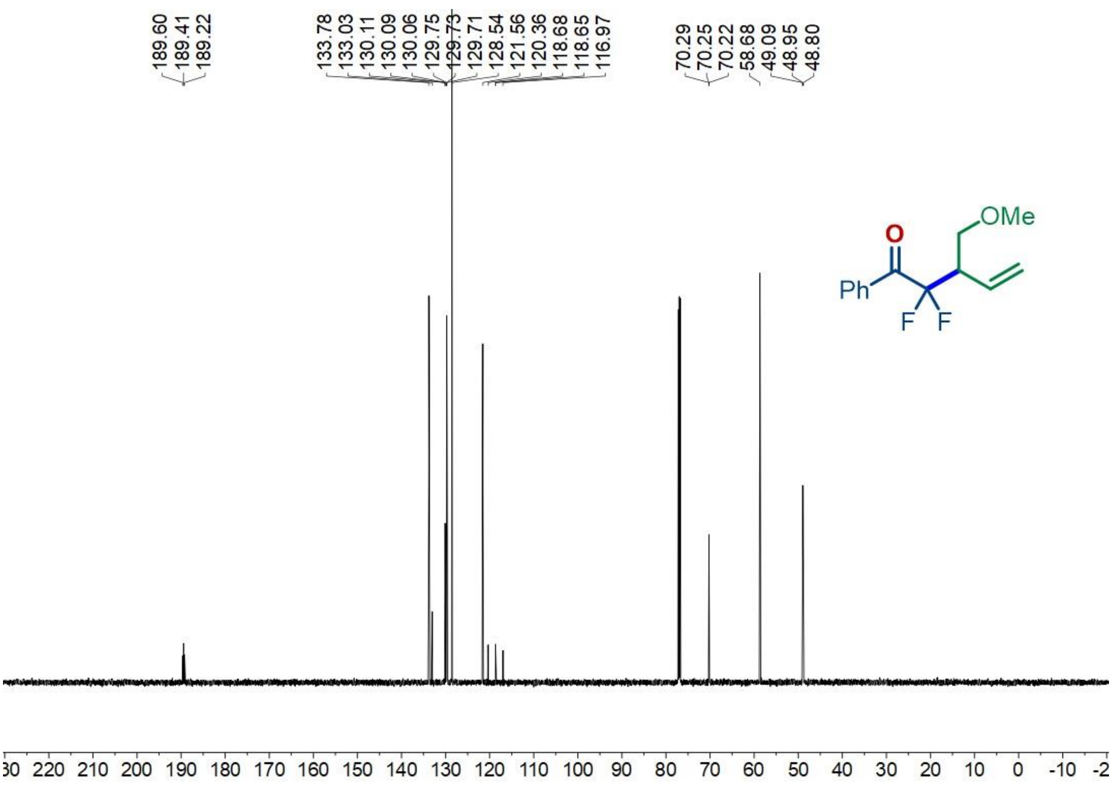

Supplementary Fig. 271 ^{13}C NMR (150 MHz, $CDCl_3$) spectrum of compound **90**

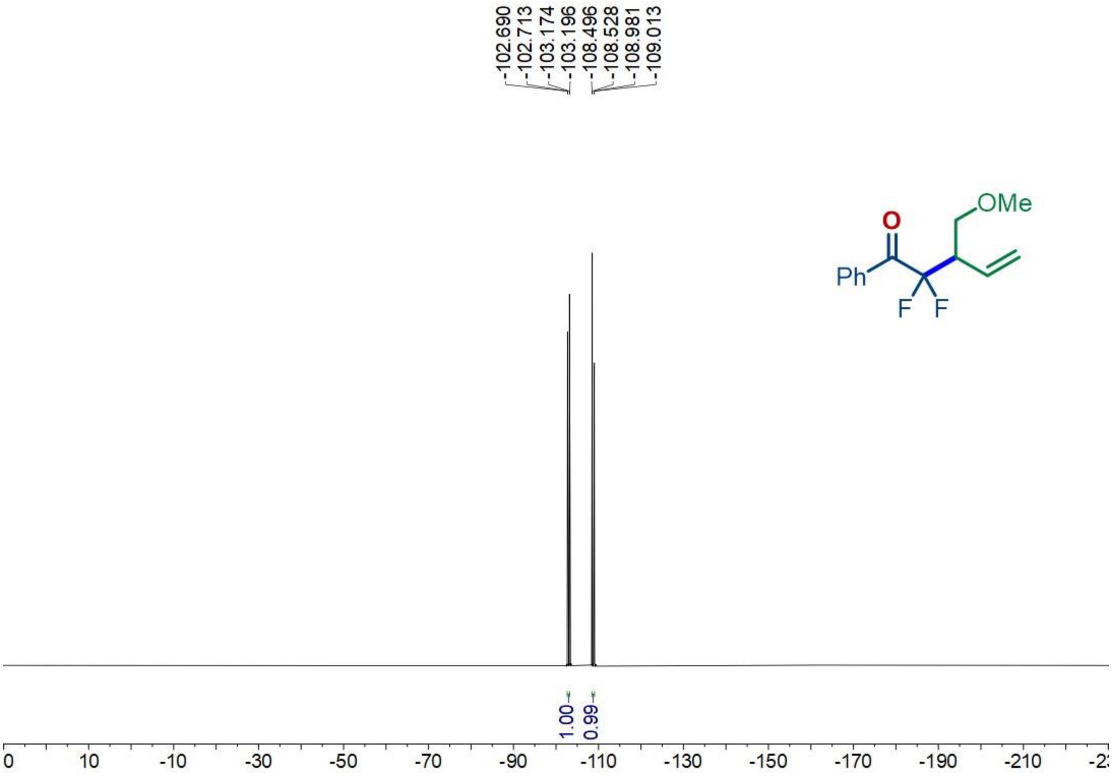

Supplementary Fig. 272 ^{19}F NMR (564 MHz, $CDCl_3$) spectrum of compound **90**

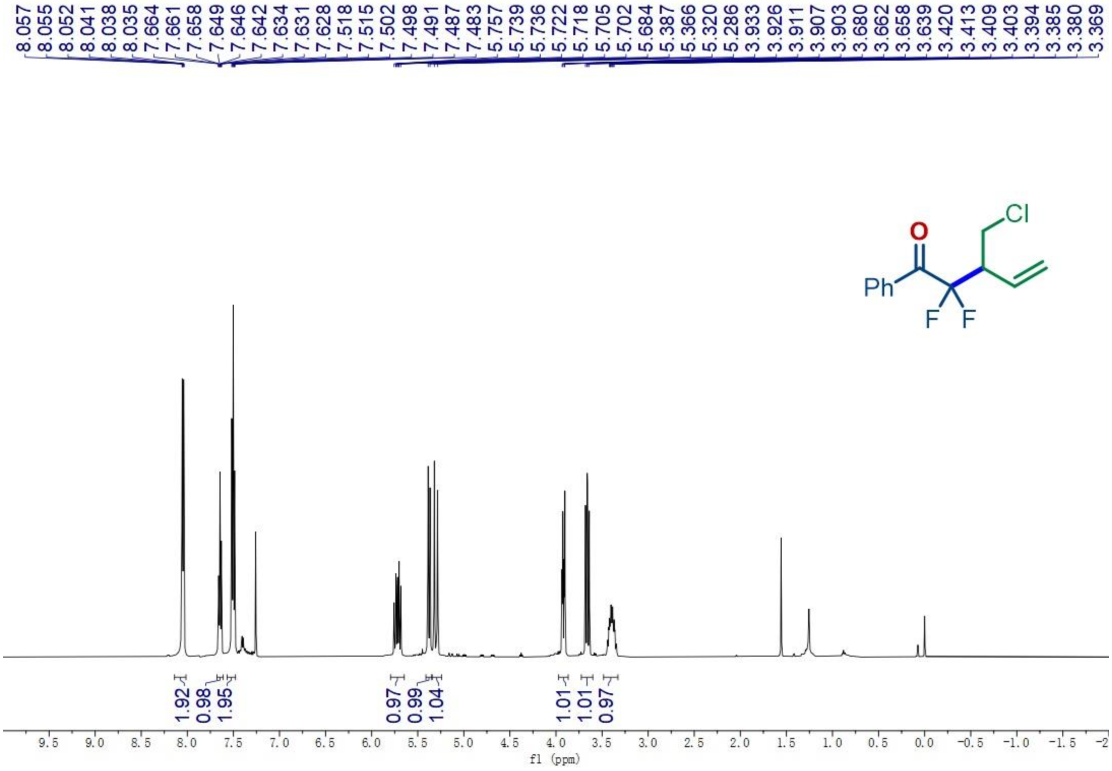

Supplementary Fig. 273 ^{1}H NMR (500 MHz, $CDCl_3$) spectrum of compound **91**

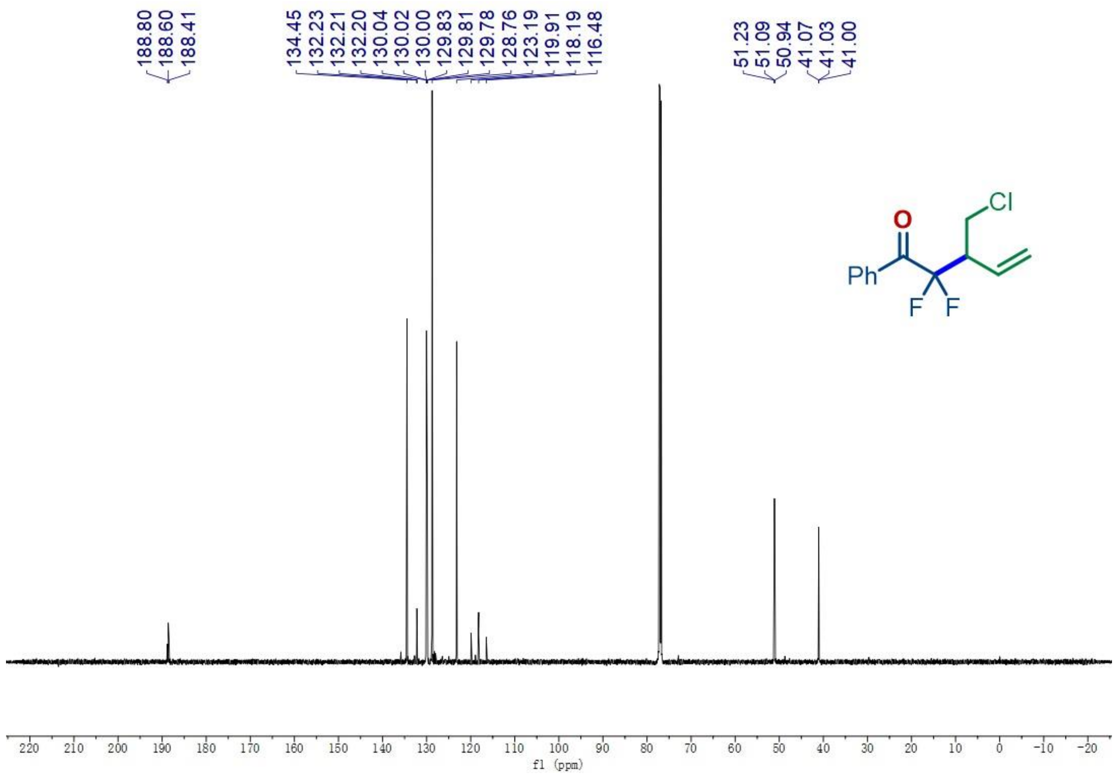

Supplementary Fig. 274 ^{13}C NMR (150 MHz, $CDCl_3$) spectrum of compound **91**

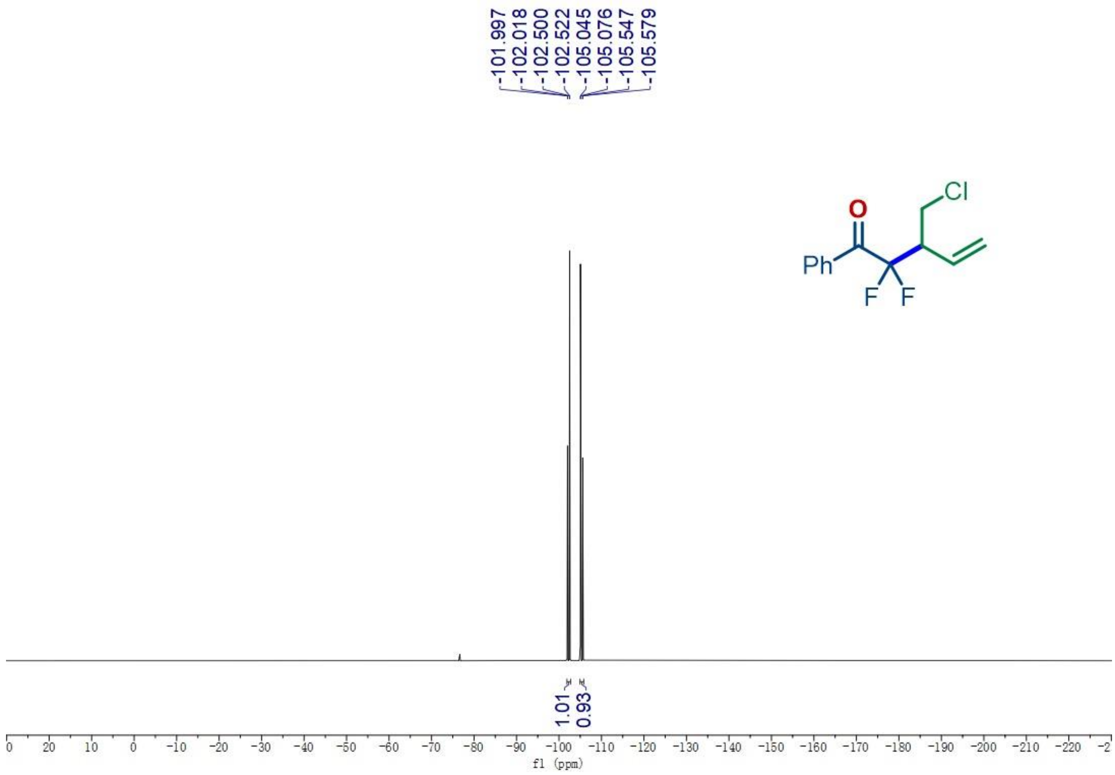

Supplementary Fig. 275 ^{19}F NMR (564 MHz, $CDCl_3$) spectrum of compound **91**

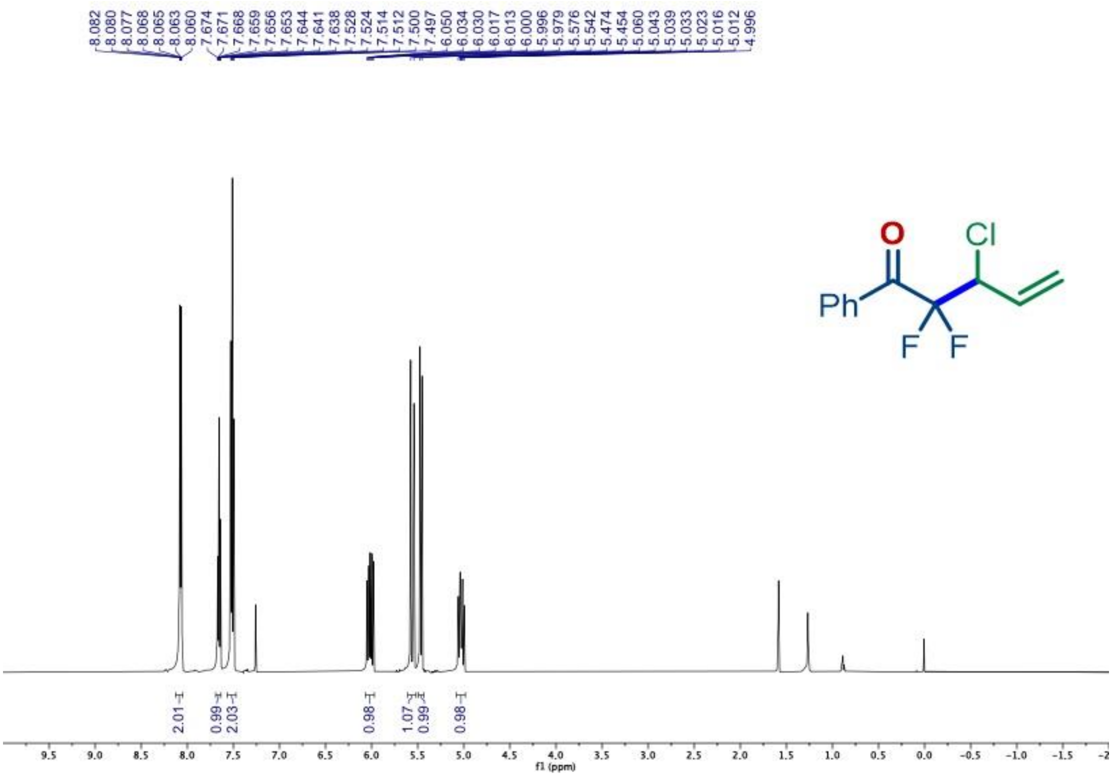

Supplementary Fig. 276 ^{1}H NMR (500 MHz, $CDCl_3$) spectrum of compound **92**

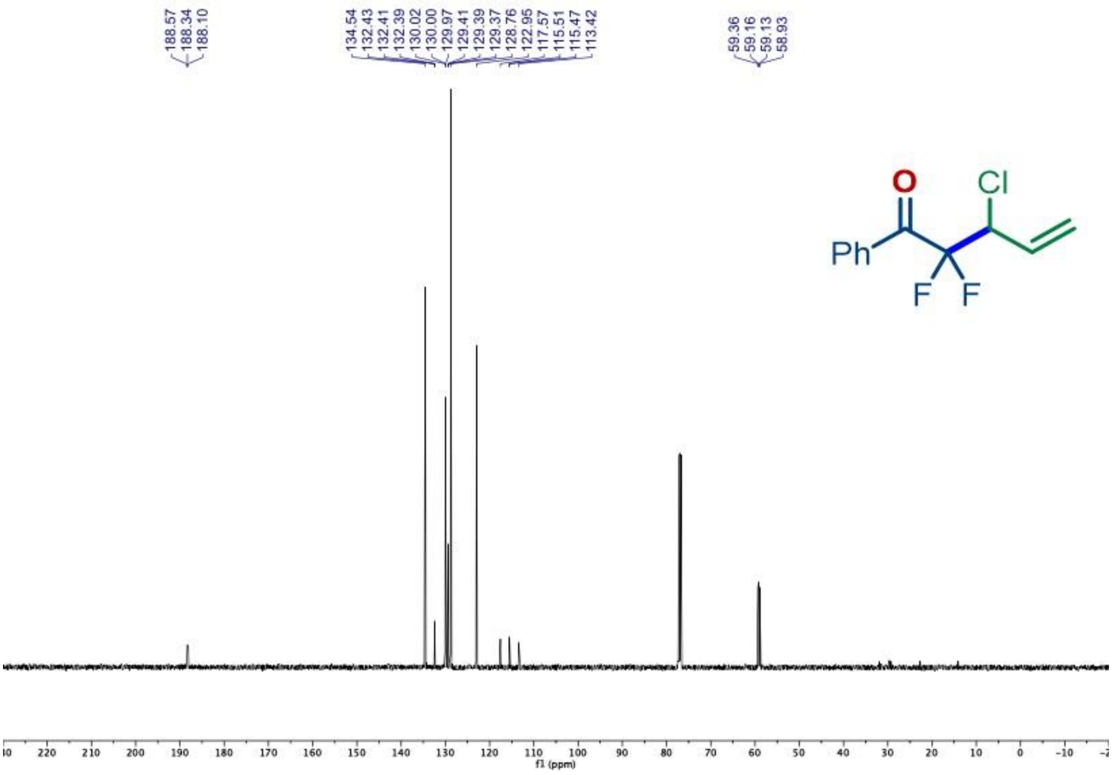

Supplementary Fig. 277 ^{13}C NMR (125 MHz, $CDCl_3$) spectrum of compound **92**

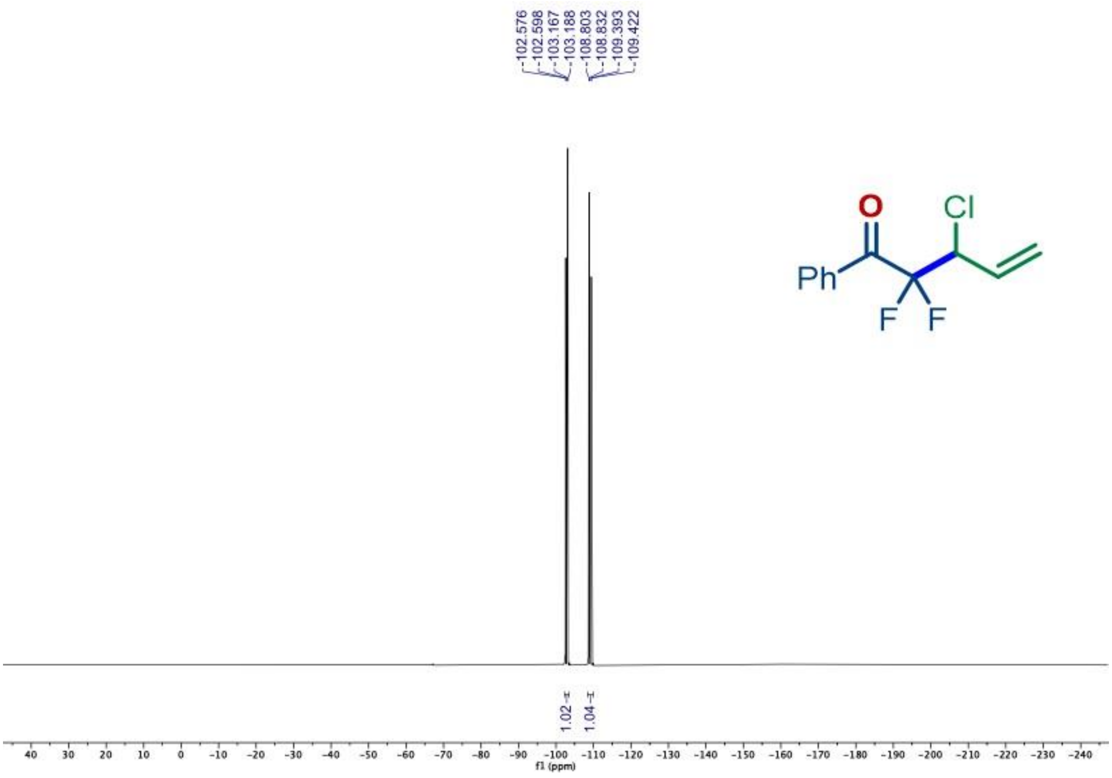

Supplementary Fig. 278 ^{19}F NMR (470 MHz, $CDCl_3$) spectrum of compound **92**

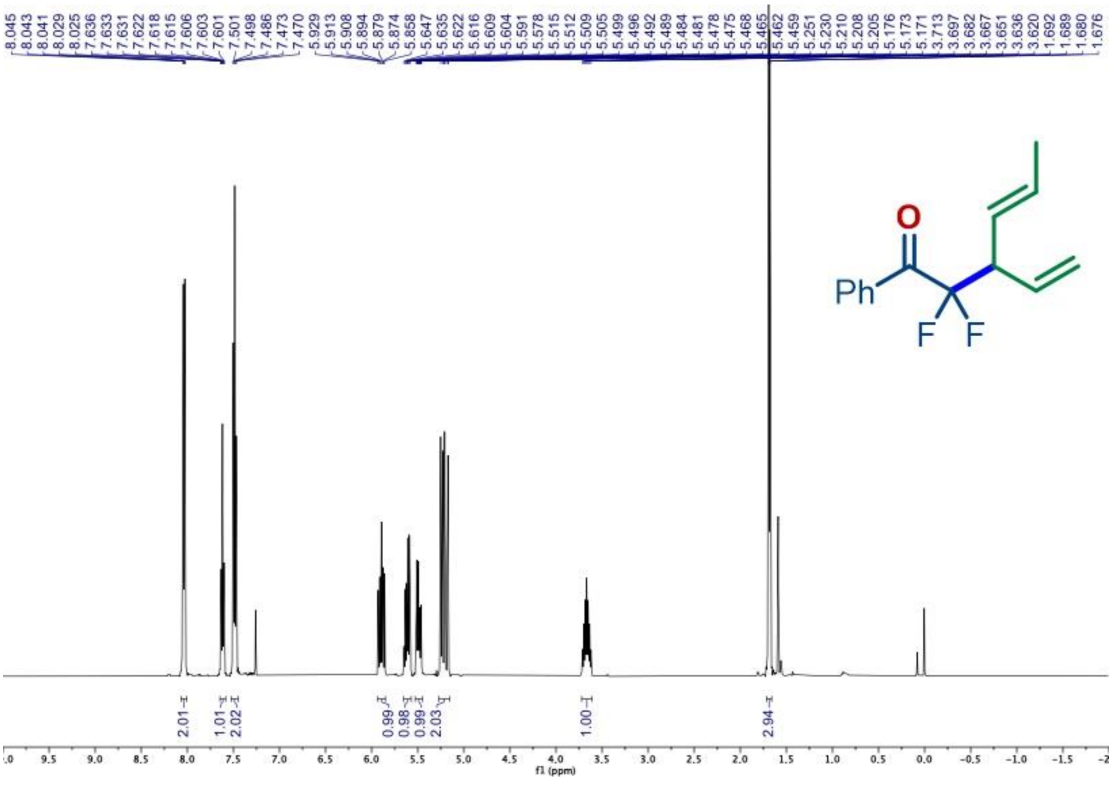

Supplementary Fig. 279 ^{1}H NMR (500 MHz, $CDCl_3$) spectrum of compound **93**

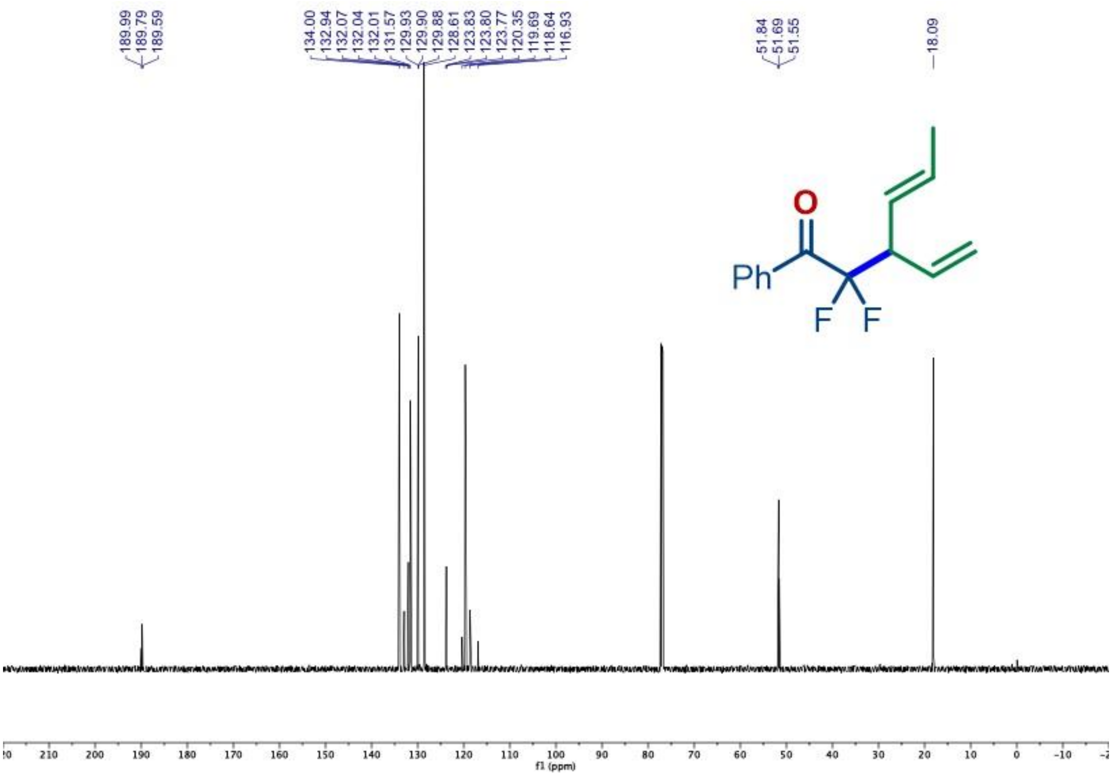

Supplementary Fig. 280 ^{13}C NMR (150 MHz, $CDCl_3$) spectrum of compound **93**

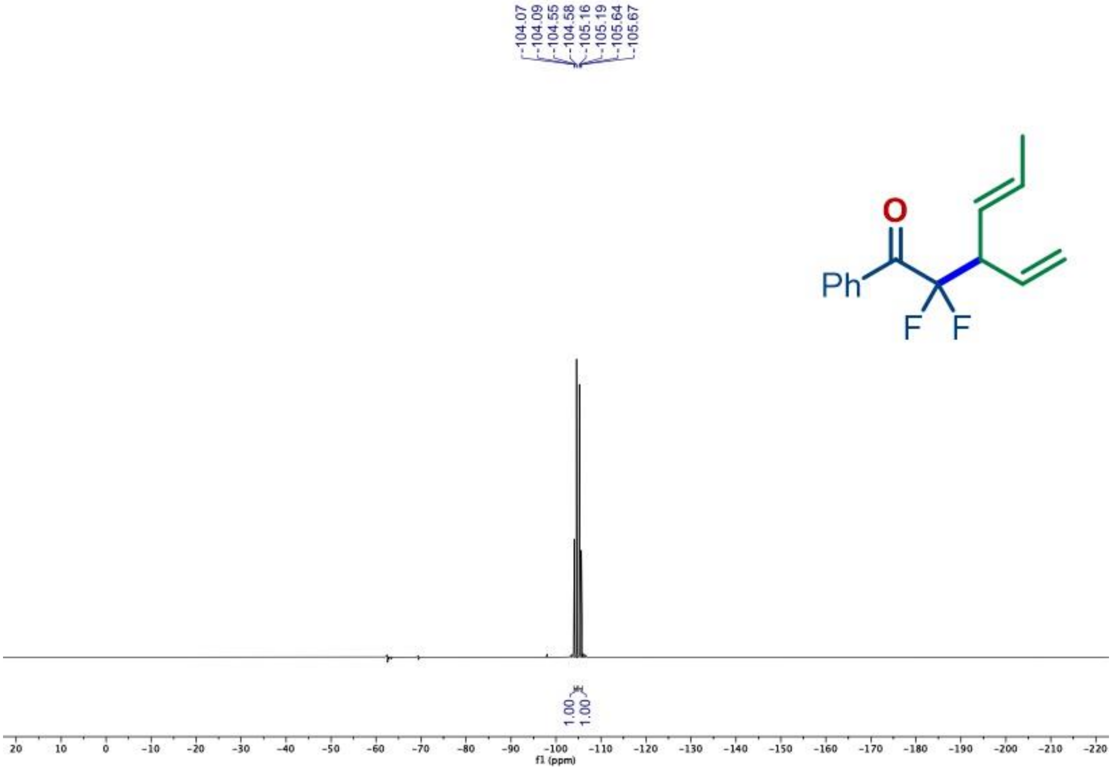

Supplementary Fig. 281 ^{19}F NMR (564 MHz, $CDCl_3$) spectrum of compound **93**

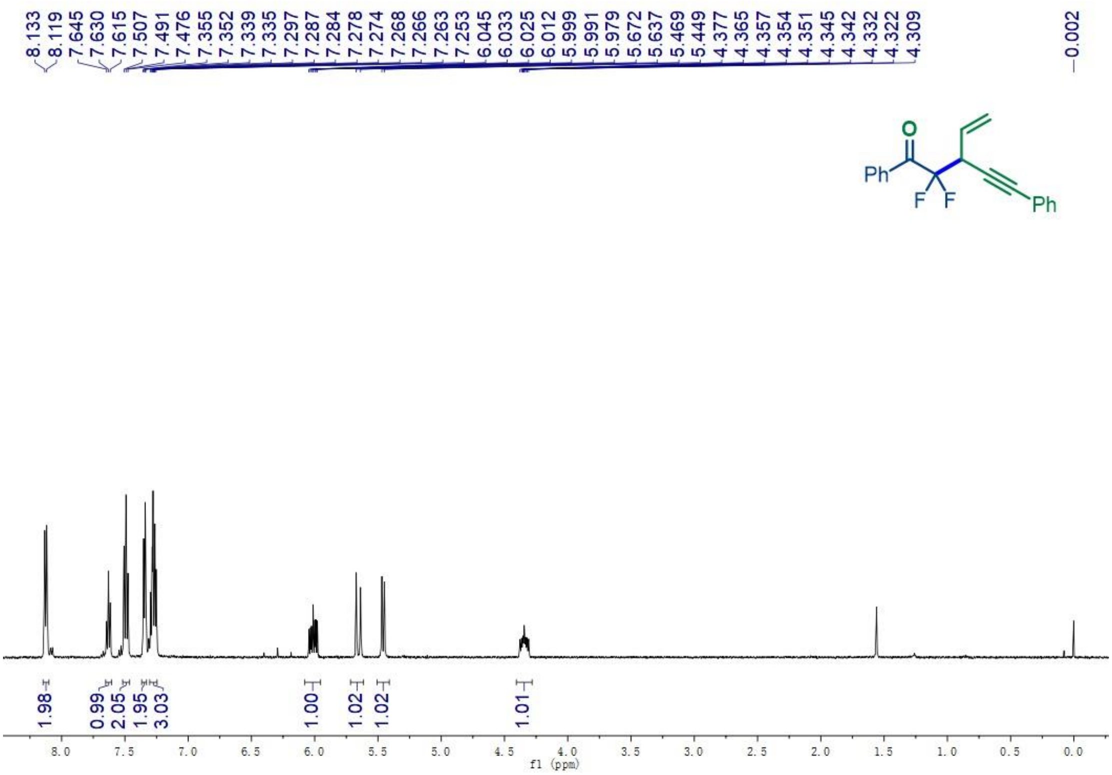

Supplementary Fig. 282 ^{1}H NMR (500 MHz, $CDCl_3$) spectrum of compound **94**

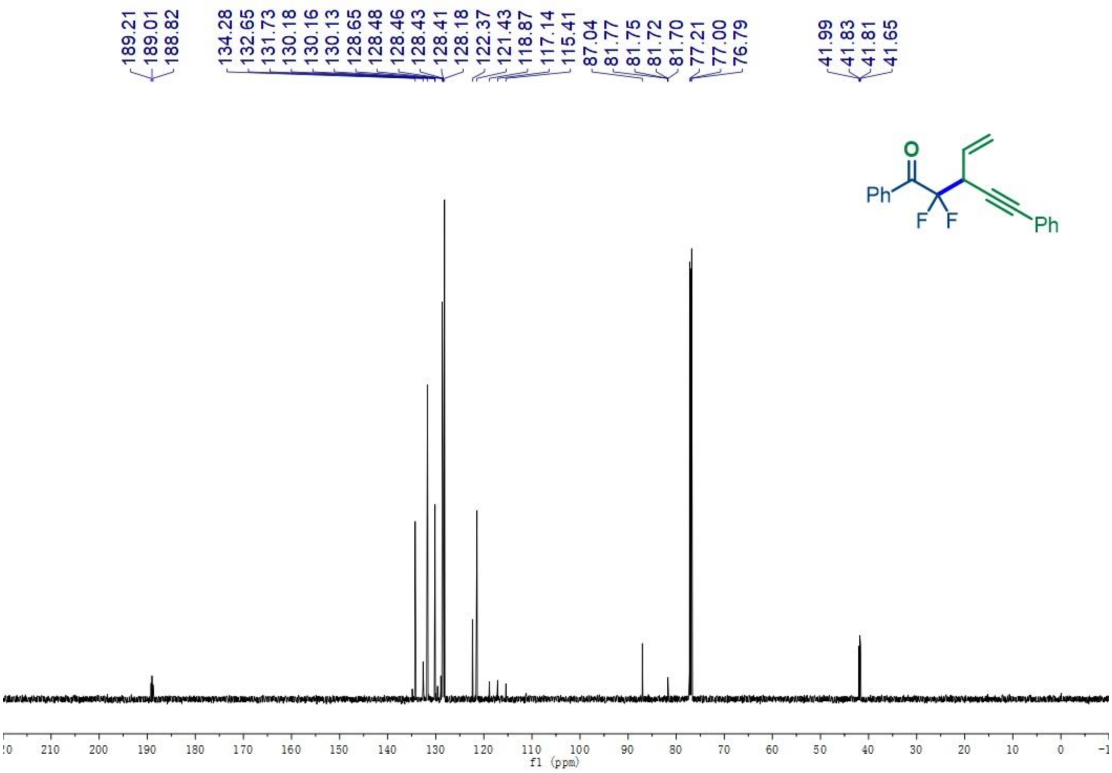

Supplementary Fig. 283 ^{13}C NMR (150 MHz, $CDCl_3$) spectrum of compound **94**

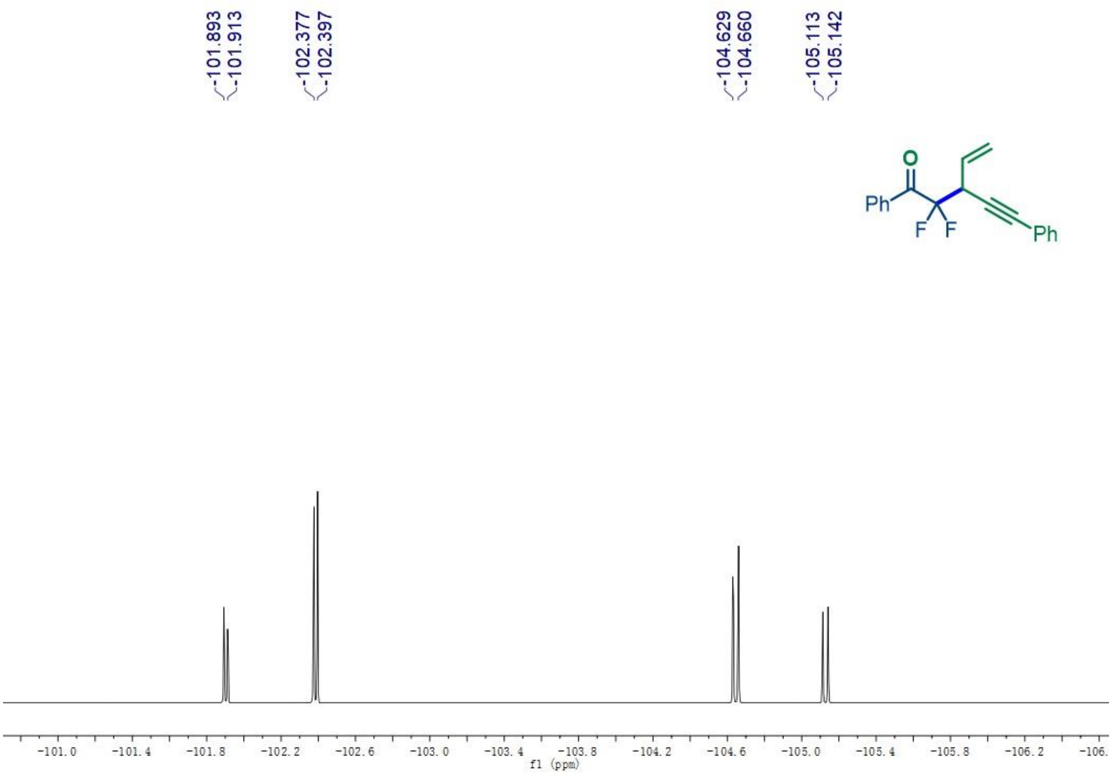

Supplementary Fig. 284 ^{19}F NMR (564 MHz, $CDCl_3$) spectrum of compound **94**

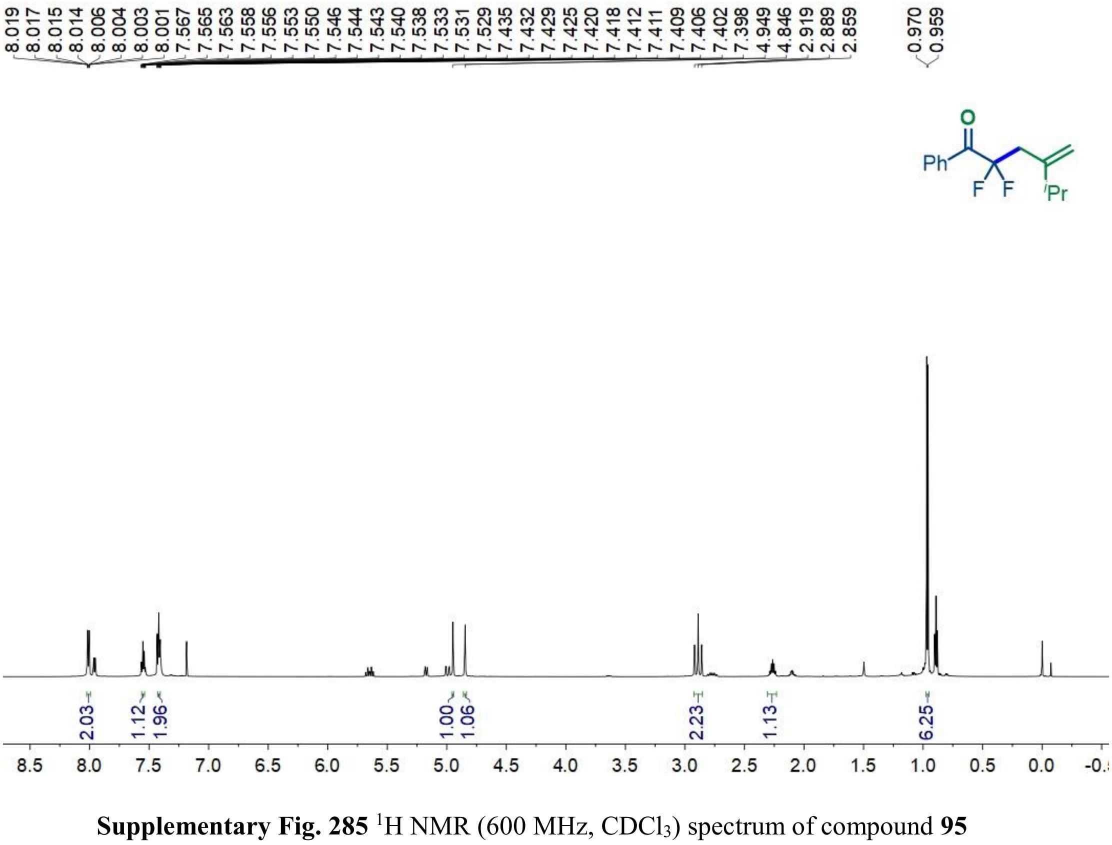

Supplementary Fig. 285 ^{1}H NMR (600 MHz, $CDCl_3$) spectrum of compound **95**

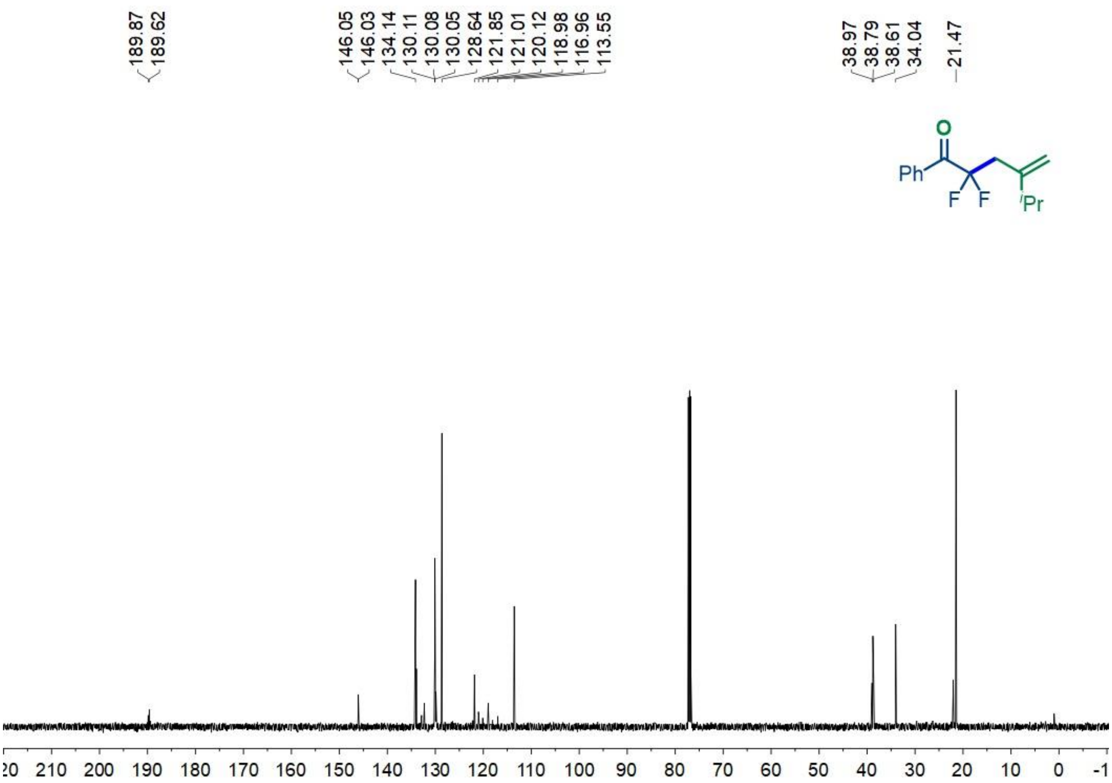

Supplementary Fig. 286 ^{13}C NMR (125 MHz, $CDCl_3$) spectrum of compound **95**

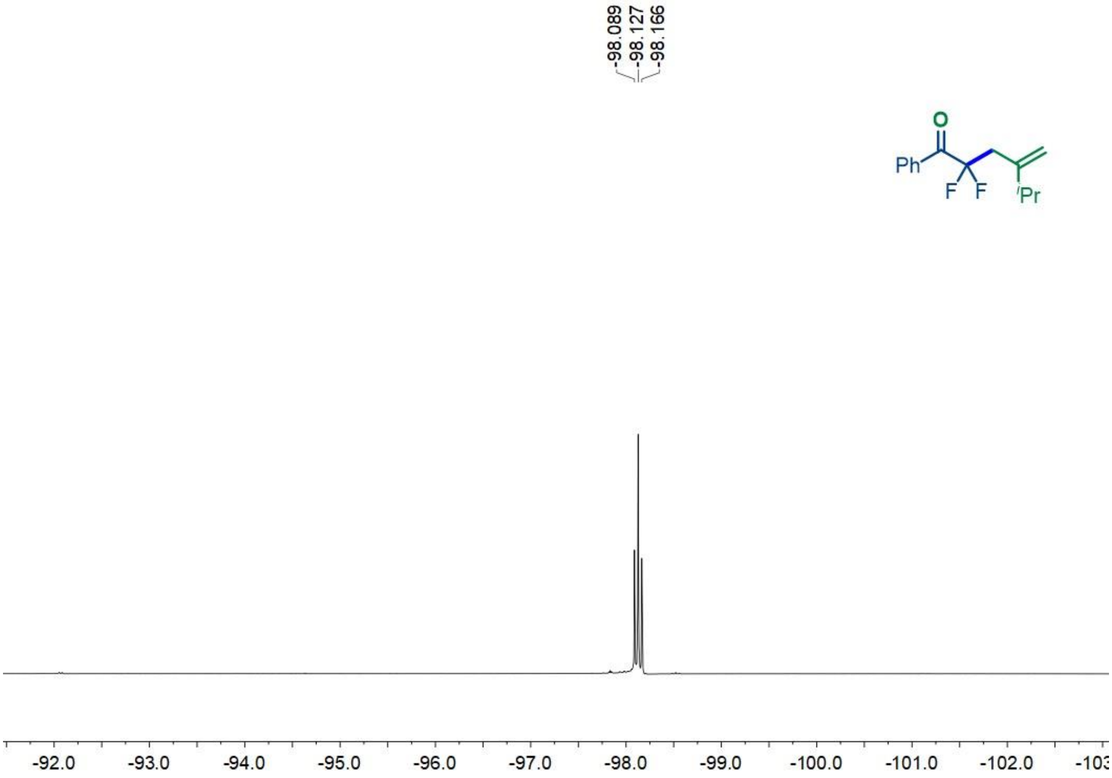

Supplementary Fig. 287 ^{19}F NMR (470 MHz, $CDCl_3$) spectrum of compound **95**

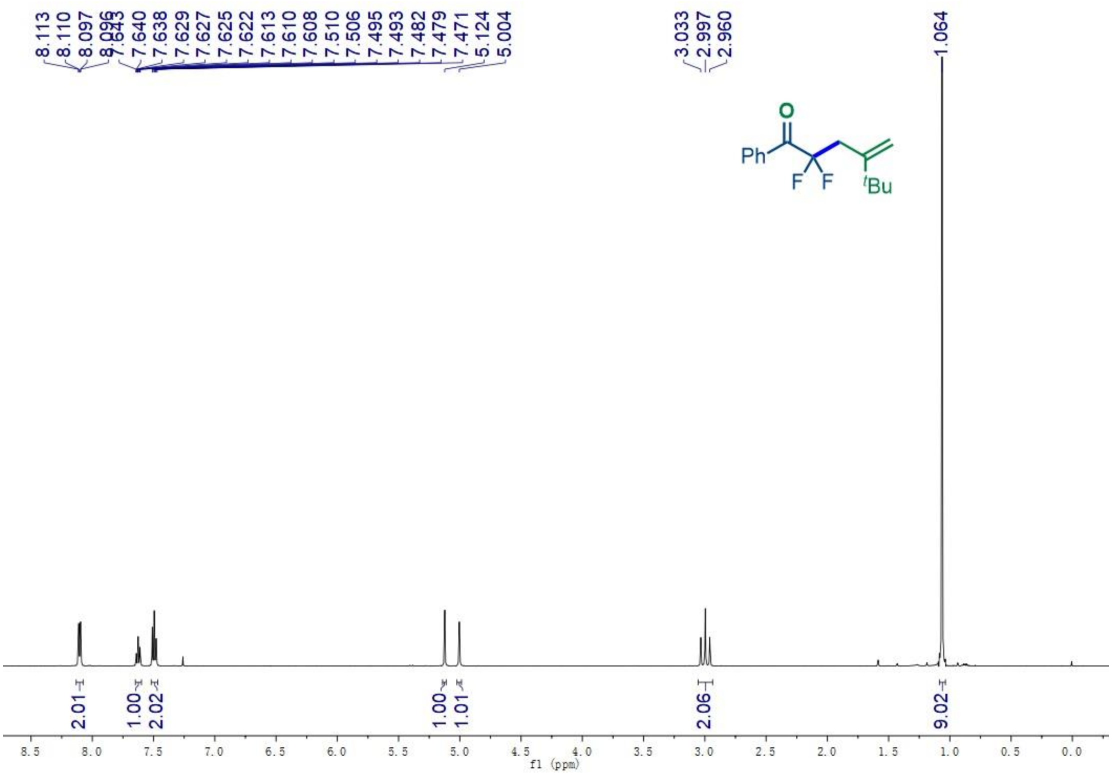

Supplementary Fig. 288 ^{1}H NMR (500 MHz, $CDCl_3$) spectrum of compound **96**

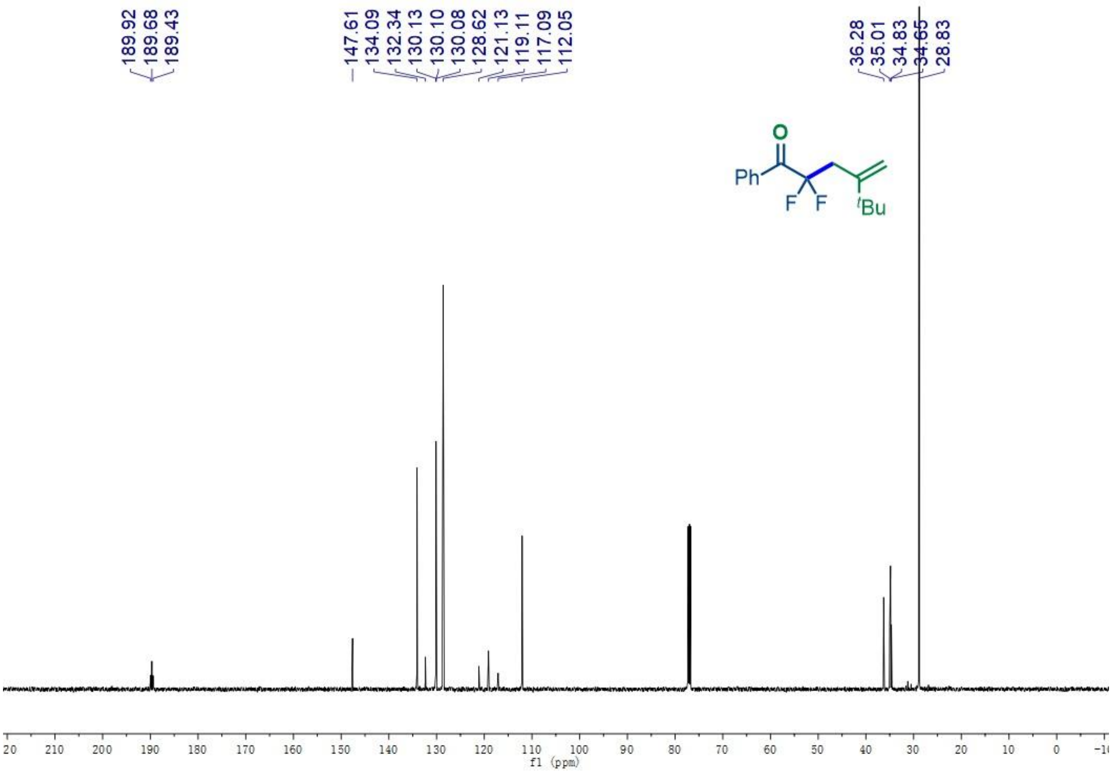

Supplementary Fig. 289 ^{13}C NMR (125 MHz, $CDCl_3$) spectrum of compound **96**

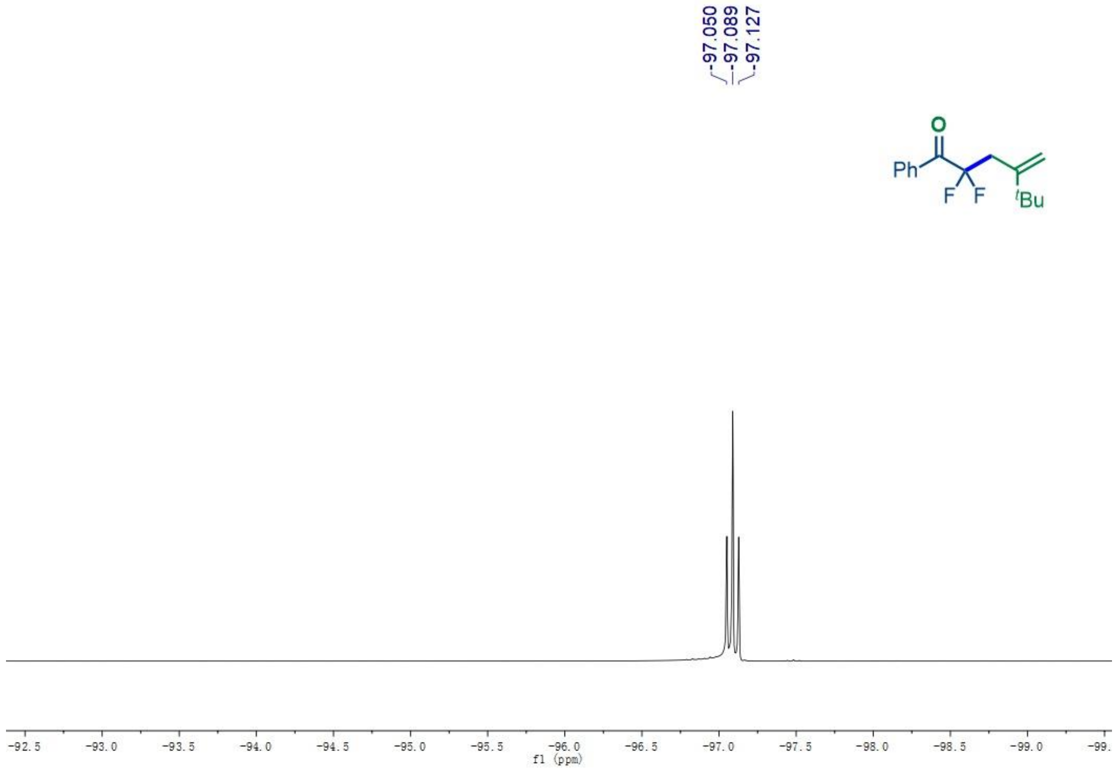

Supplementary Fig. 290 ^{19}F NMR (470 MHz, $CDCl_3$) spectrum of compound **96**

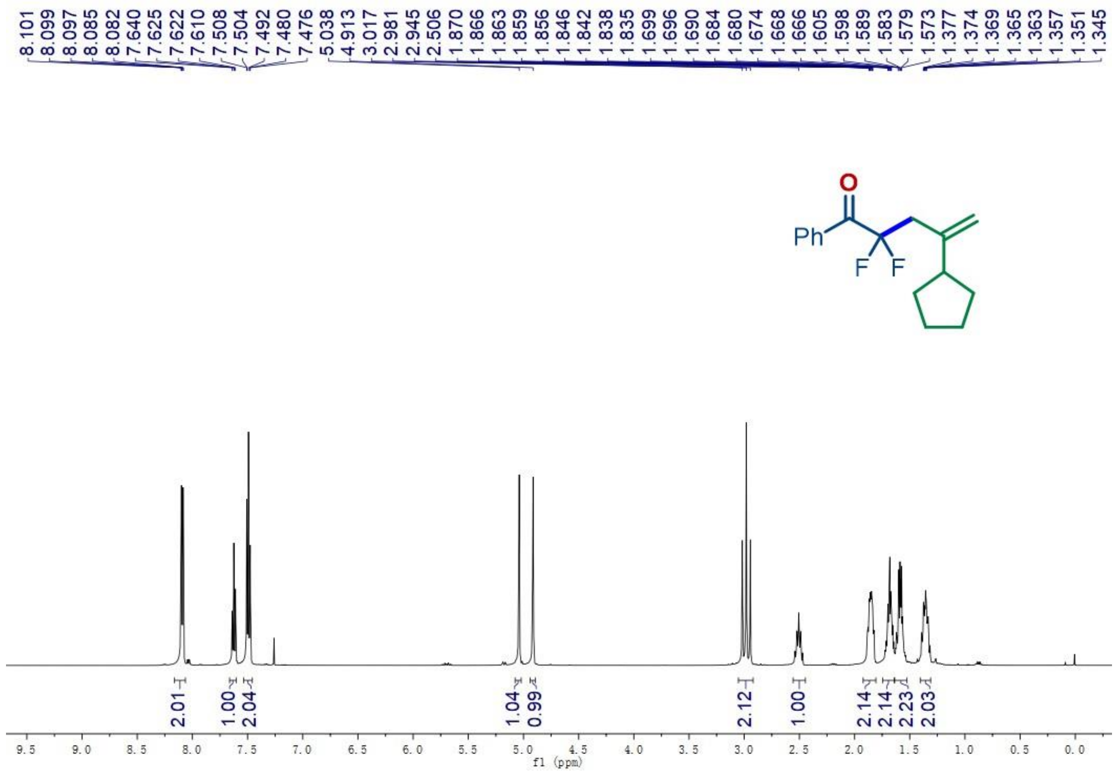

Supplementary Fig. 291 ^{1}H NMR (500 MHz, $CDCl_3$) spectrum of compound **97**

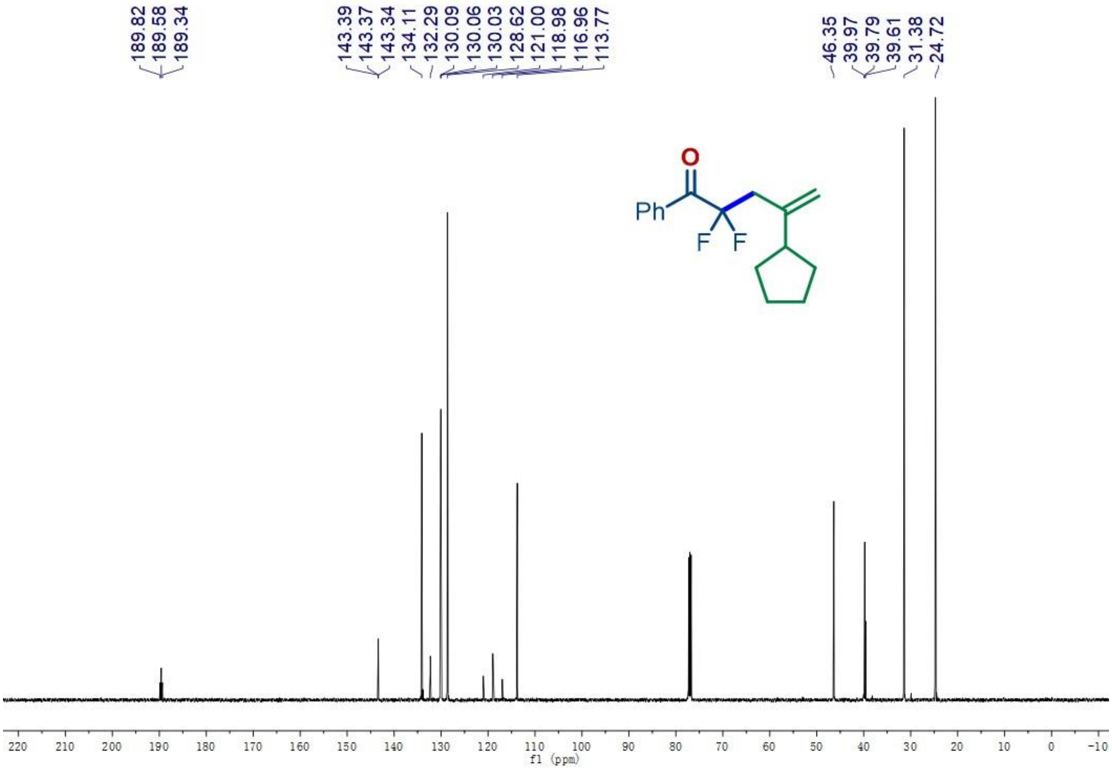

Supplementary Fig. 292 ^{13}C NMR (125 MHz, $CDCl_3$) spectrum of compound **97**

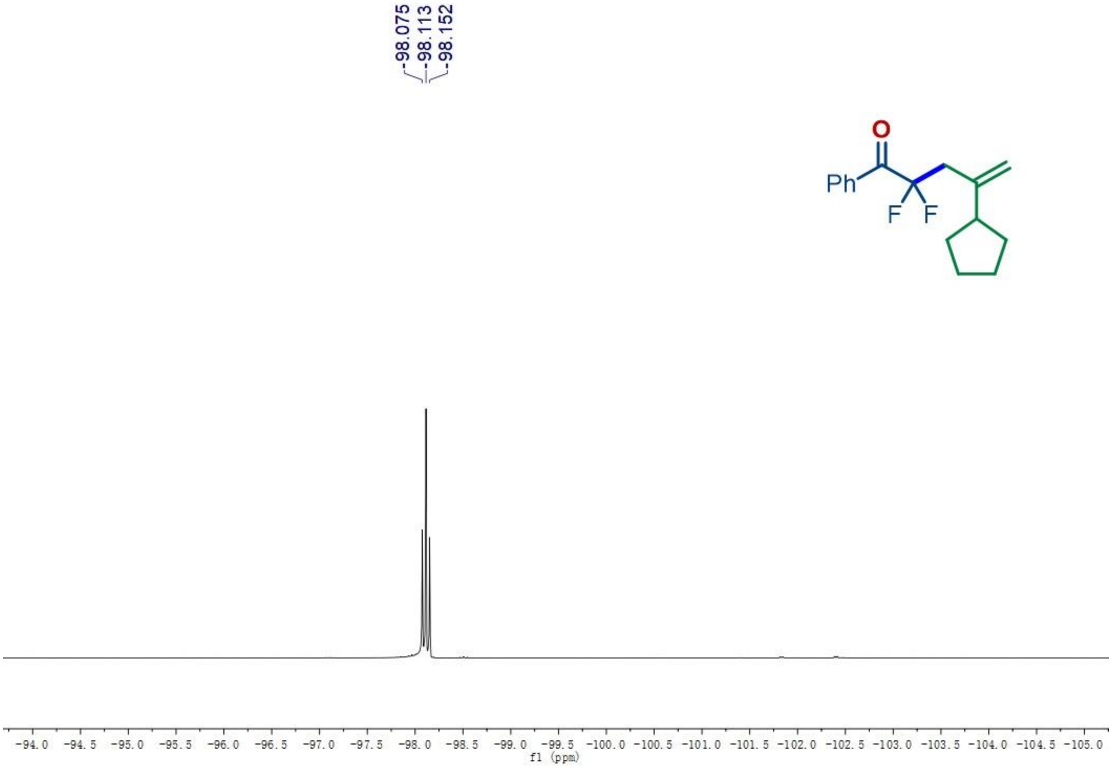

Supplementary Fig. 293 ^{19}F NMR (470 MHz, $CDCl_3$) spectrum of compound **97**

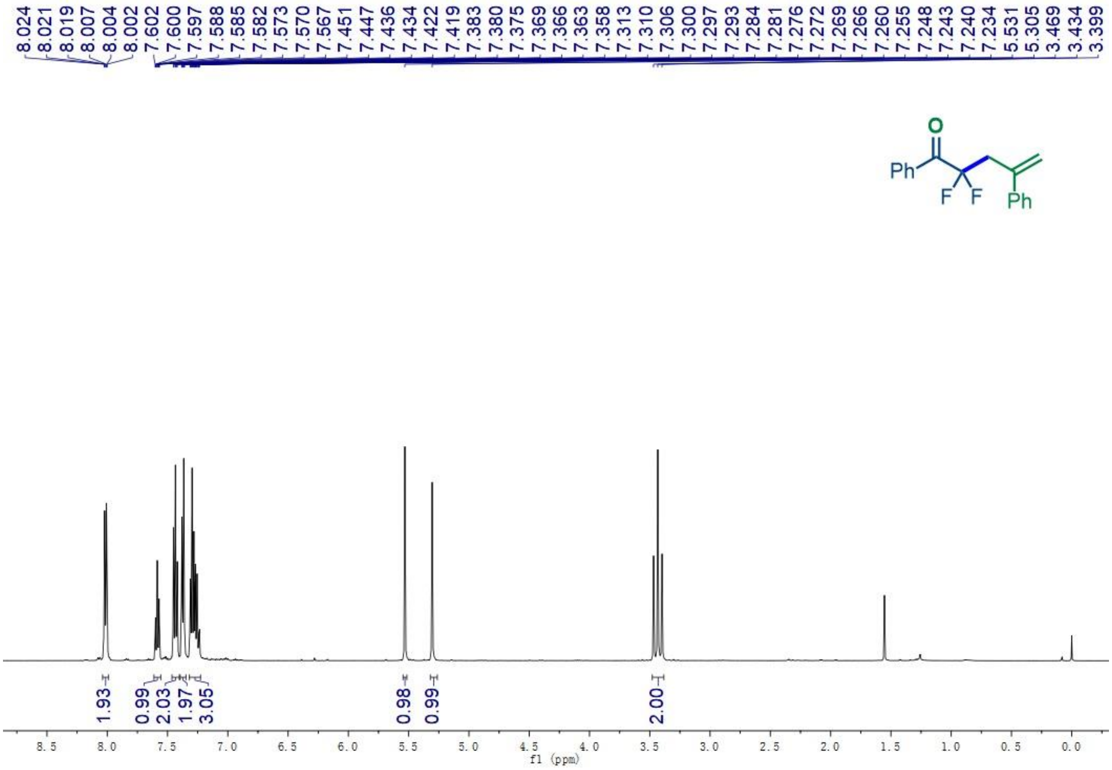

Supplementary Fig. 294 ^{1}H NMR (500 MHz, $CDCl_3$) spectrum of compound **98**

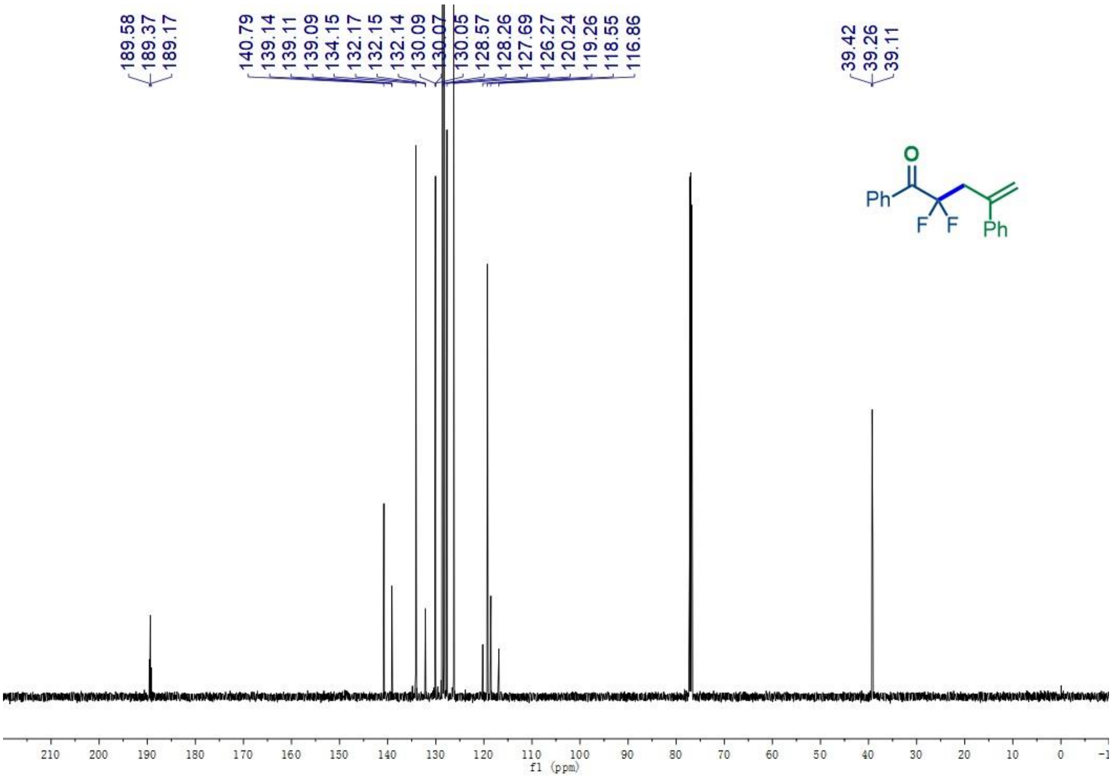

Supplementary Fig. 295 ^{13}C NMR (150 MHz, $CDCl_3$) spectrum of compound **98**

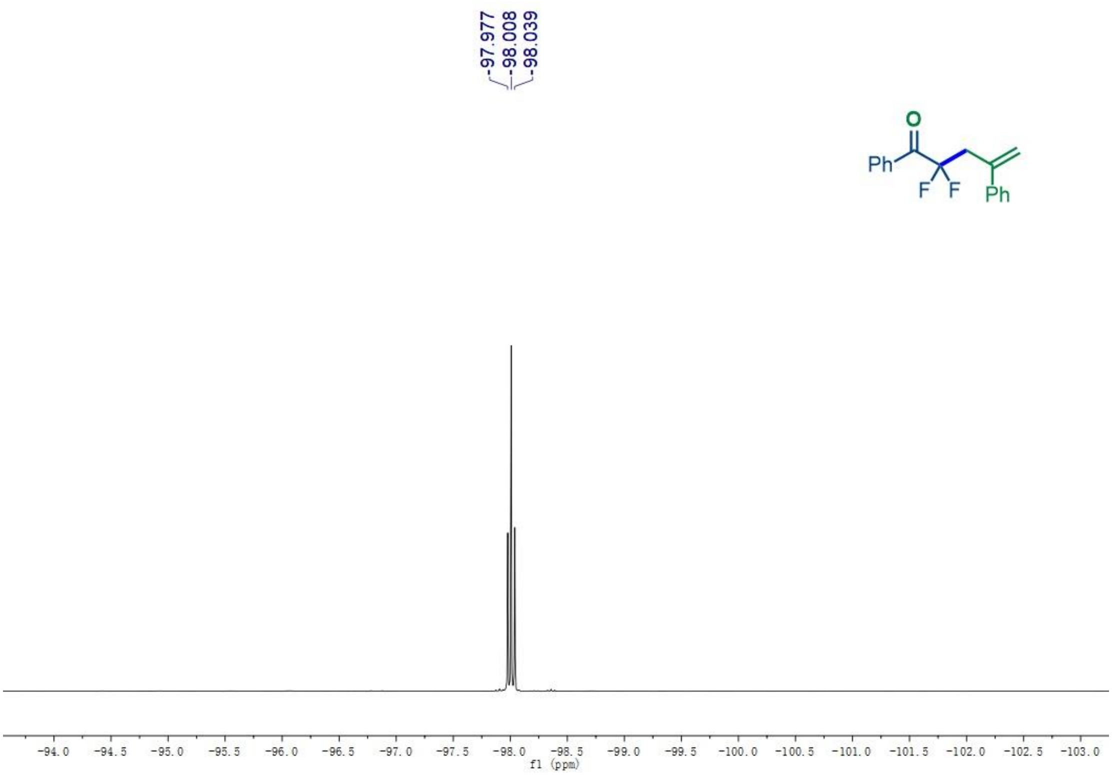

Supplementary Fig. 296 ^{19}F NMR (564 MHz, $CDCl_3$) spectrum of compound **98**

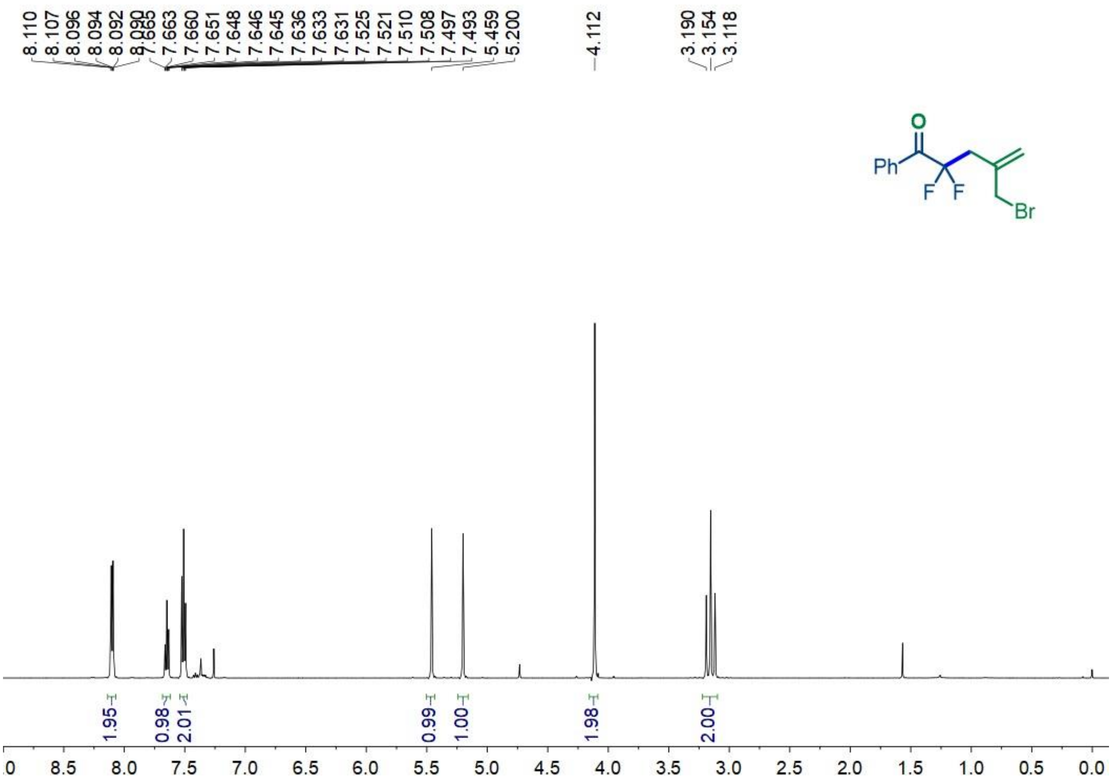

Supplementary Fig. 297 ^{1}H NMR (500 MHz, $CDCl_3$) spectrum of compound **99**

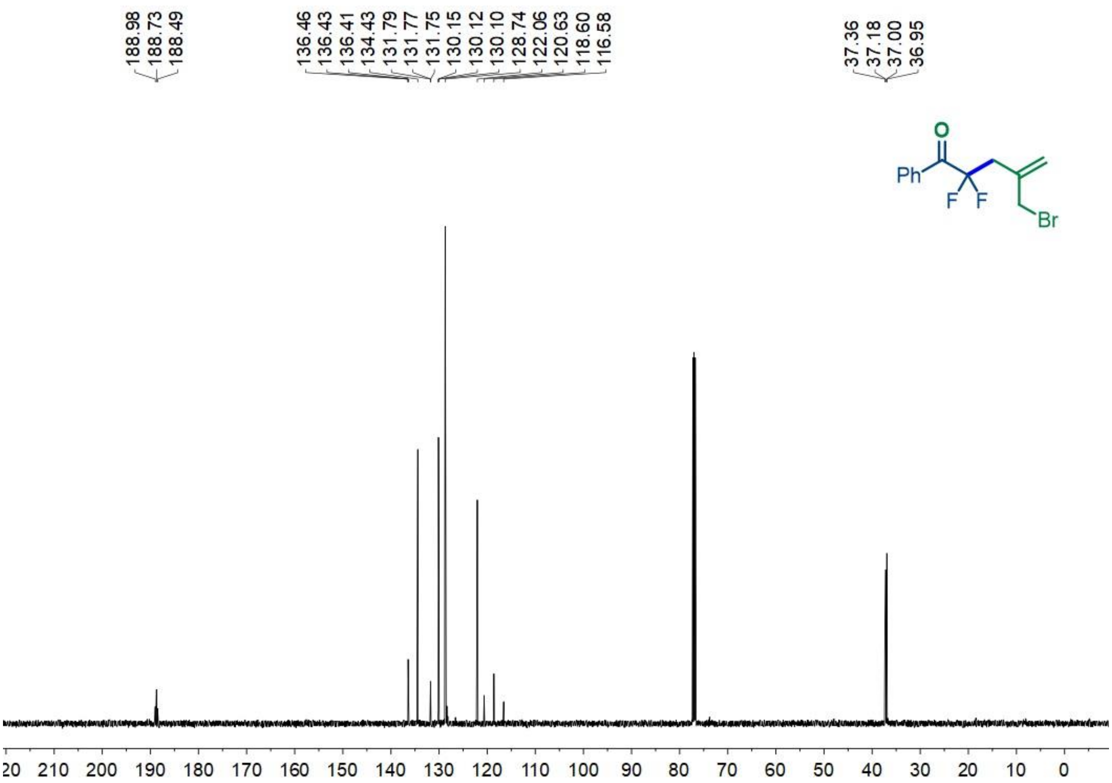

Supplementary Fig. 298 ^{13}C NMR (125 MHz, $CDCl_3$) spectrum of compound **99**

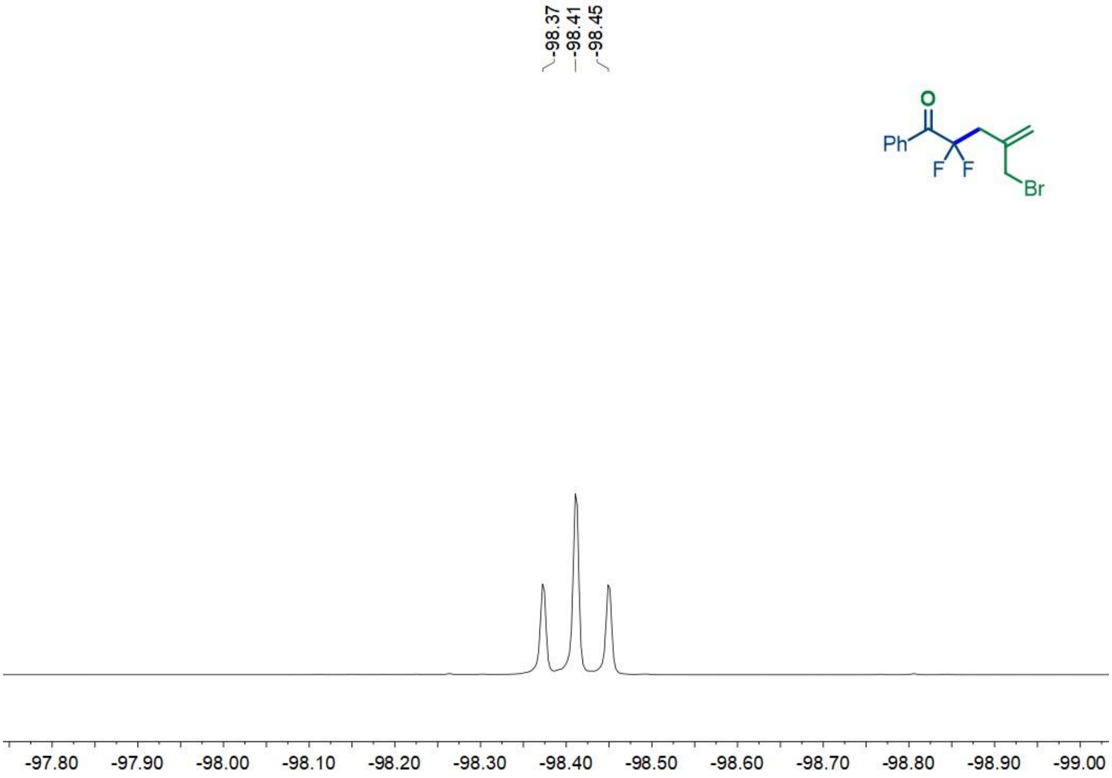

Supplementary Fig. 299 ^{19}F NMR (470 MHz, $CDCl_3$) spectrum of compound **99**

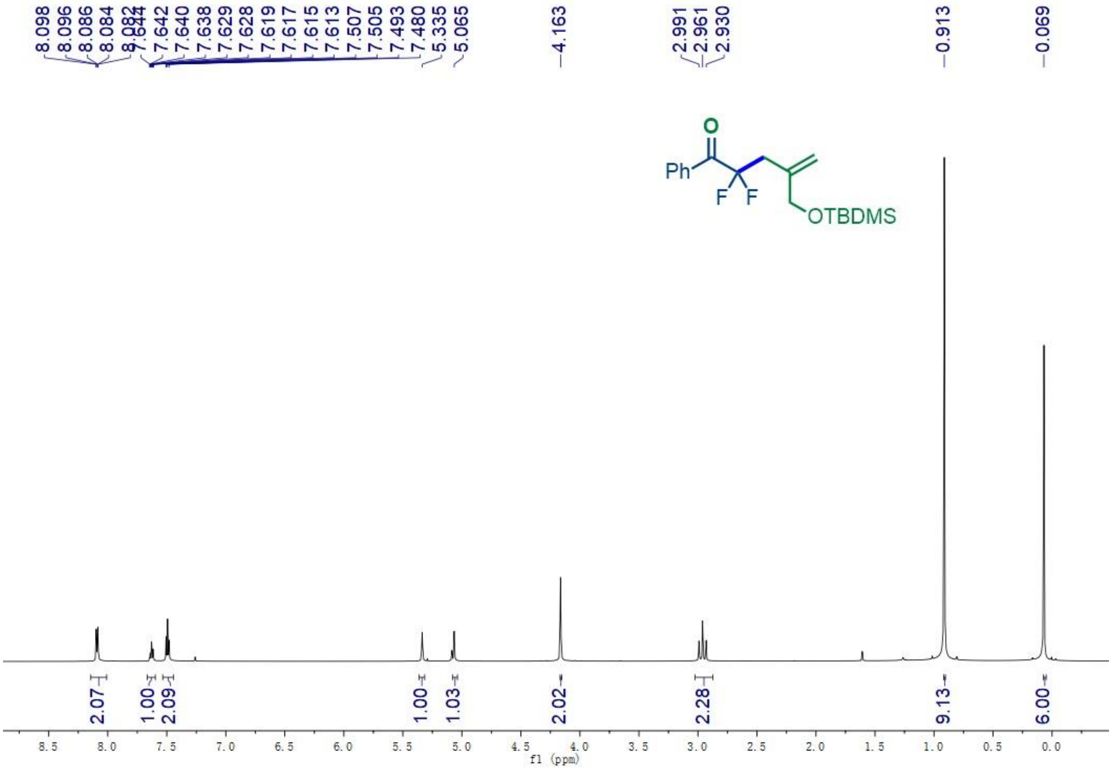

Supplementary Fig. 300 ^{1}H NMR (600 MHz, $CDCl_3$) spectrum of compound **100**

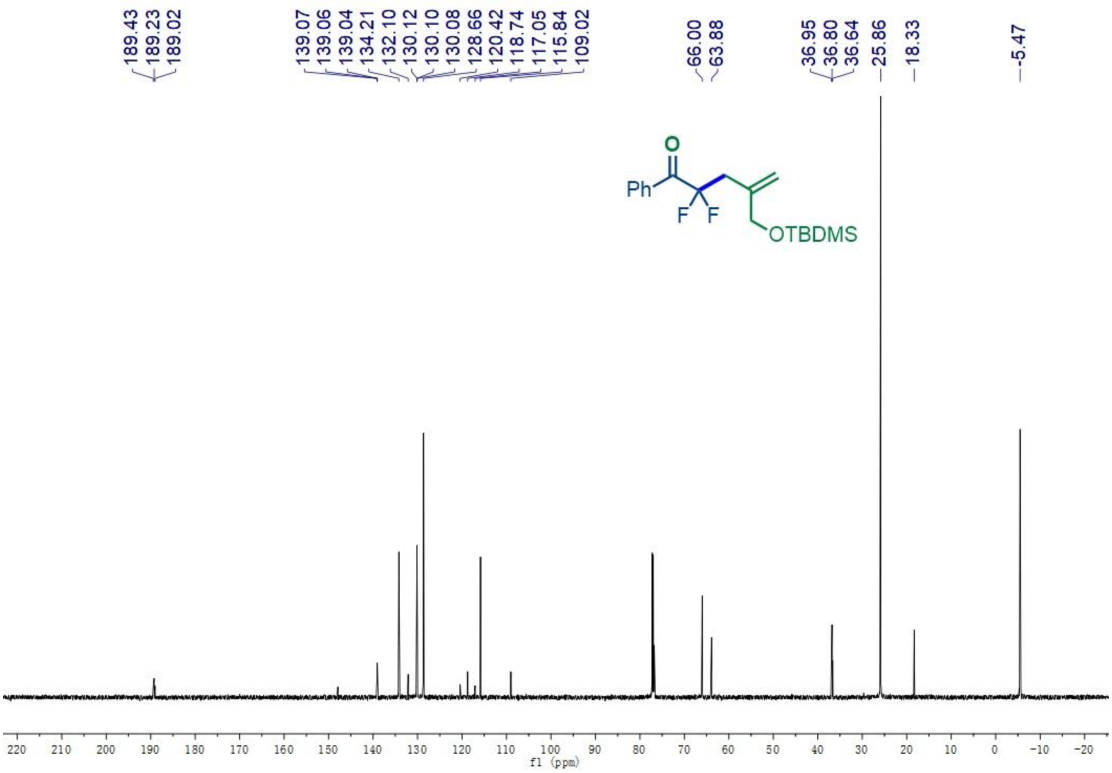

Supplementary Fig. 301 ^{13}C NMR (150 MHz, $CDCl_3$) spectrum of compound **100**

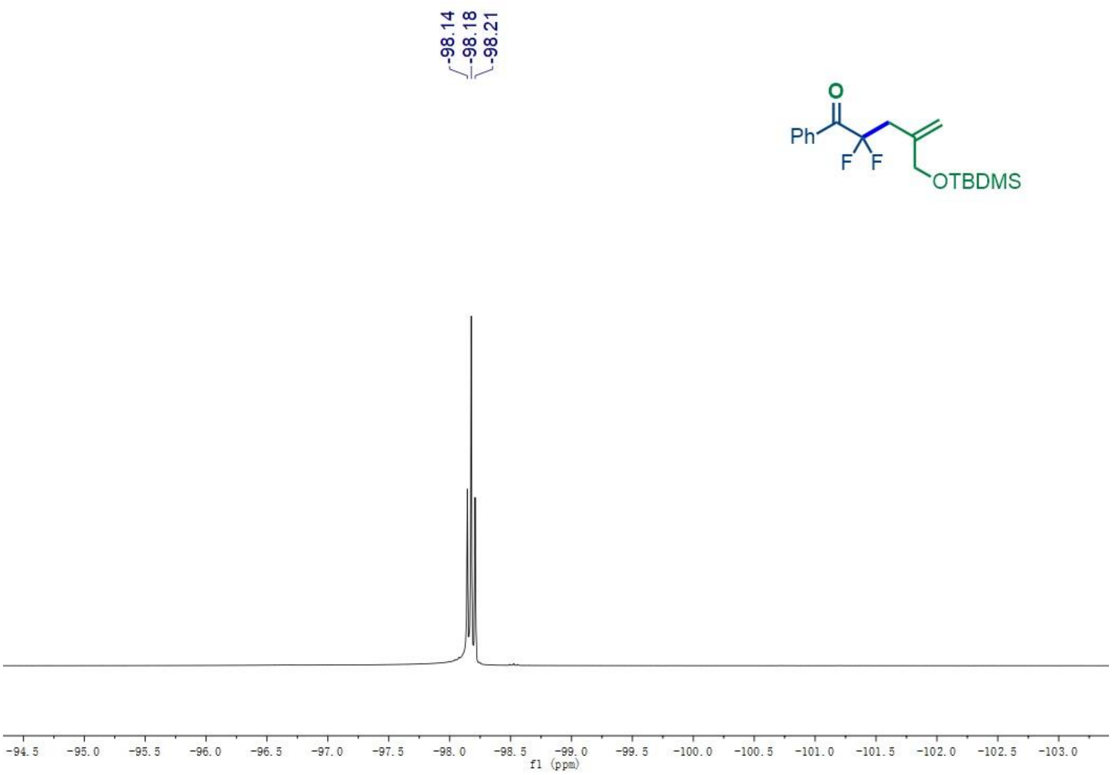

Supplementary Fig. 302 ^{19}F NMR (564 MHz, $CDCl_3$) spectrum of compound **100**

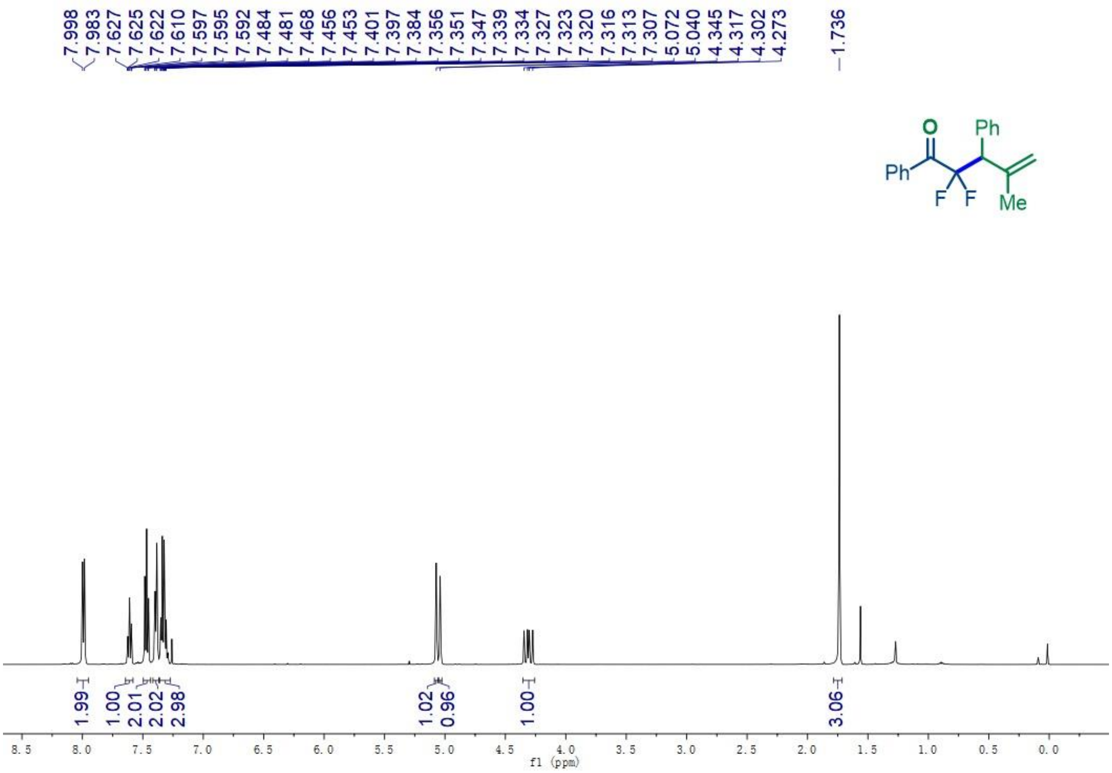

Supplementary Fig. 303 ^{1}H NMR (500 MHz, $CDCl_3$) spectrum of compound **101**

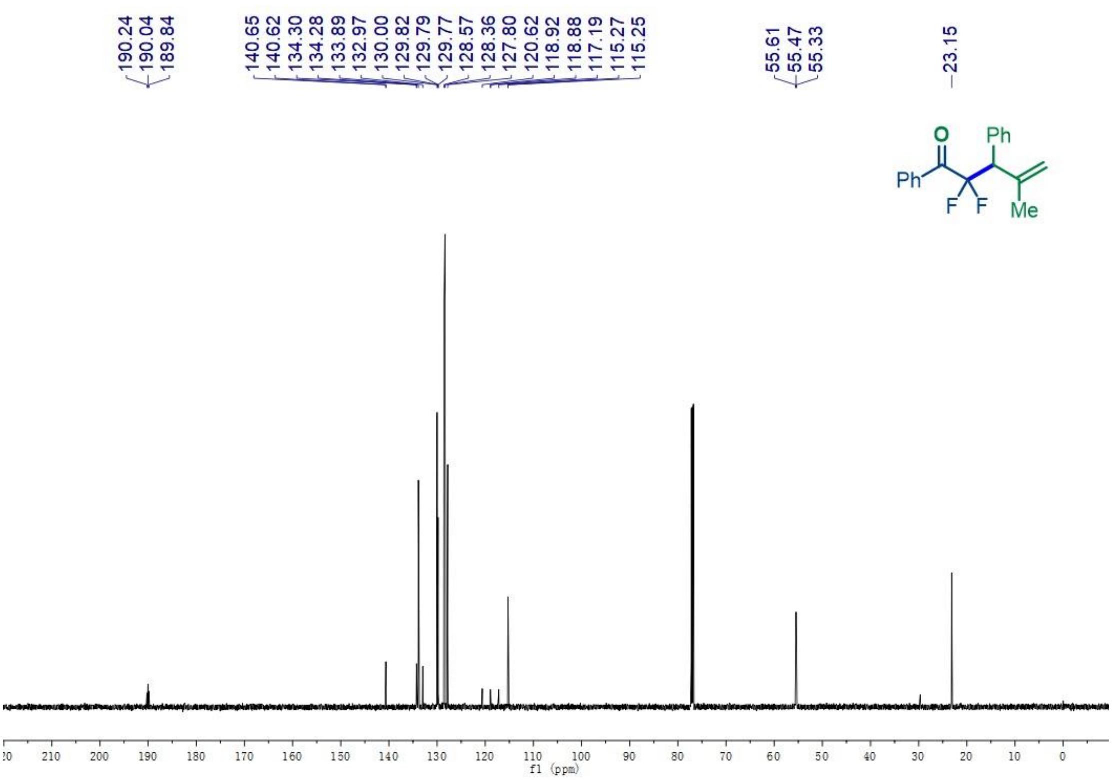

Supplementary Fig. 304 ^{13}C NMR (150 MHz, $CDCl_3$) spectrum of compound **101**

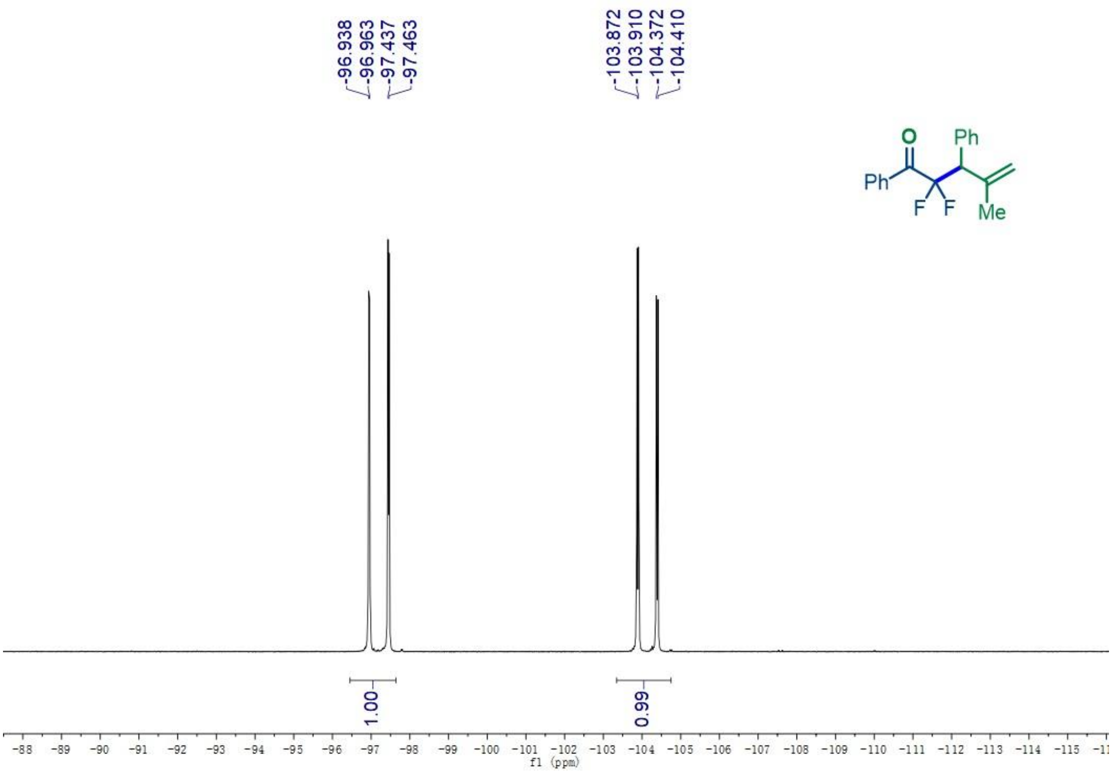

Supplementary Fig. 305 ^{19}F NMR (564 MHz, $CDCl_3$) spectrum of compound **101**

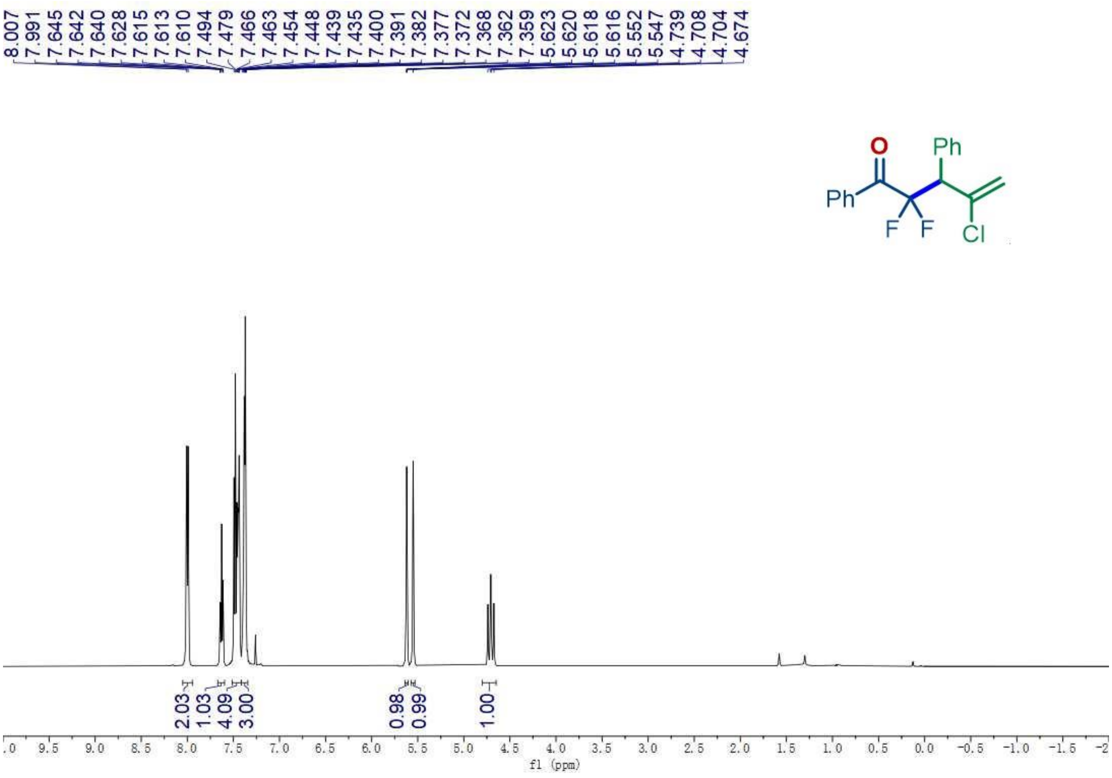

Supplementary Fig. 306 ^{1}H NMR (500 MHz, $CDCl_3$) spectrum of compound **102**

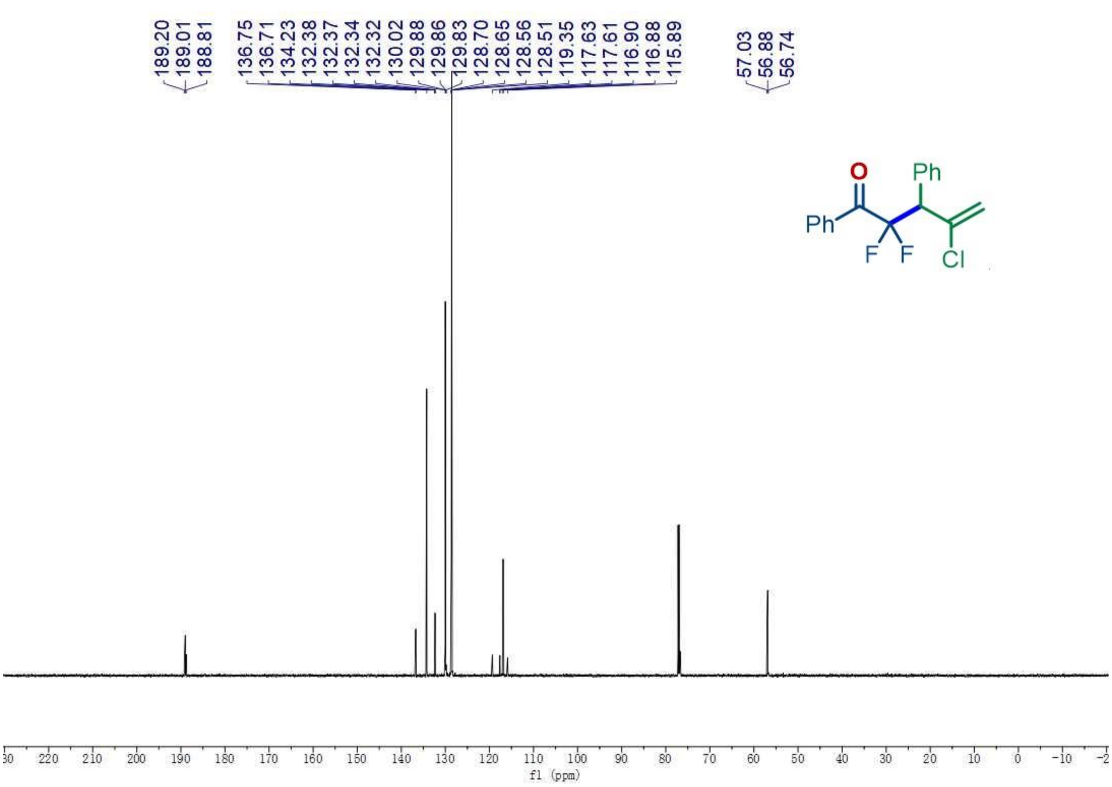

Supplementary Fig. 307 ^{13}C NMR (150 MHz, $CDCl_3$) spectrum of compound **102**

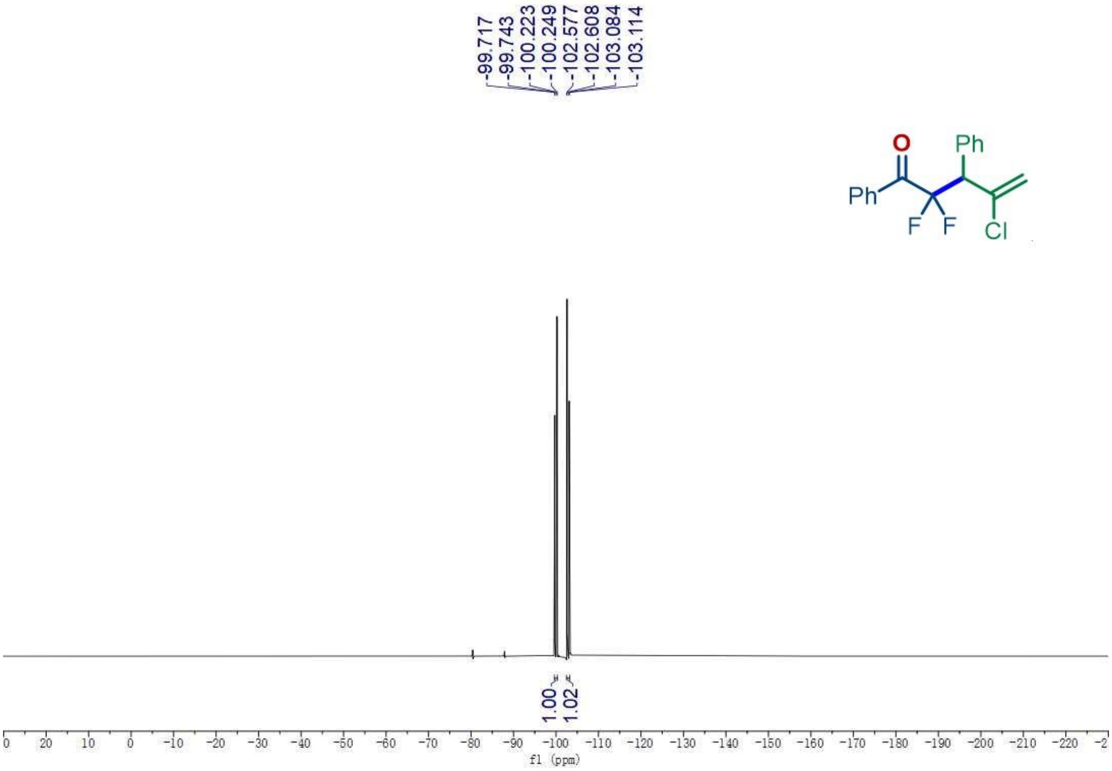

Supplementary Fig. 308 ^{19}F NMR (564 MHz, $CDCl_3$) spectrum of compound **102**

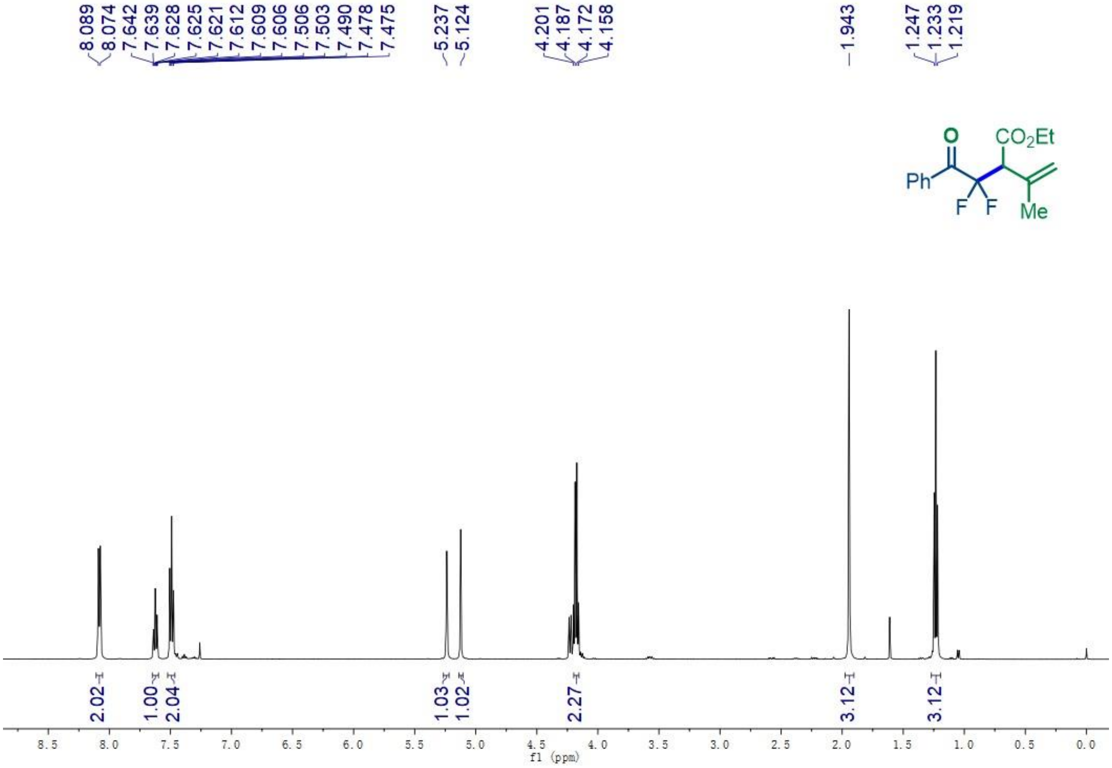

Supplementary Fig. 309 ^{1}H NMR (500 MHz, $CDCl_3$) spectrum of compound **103**

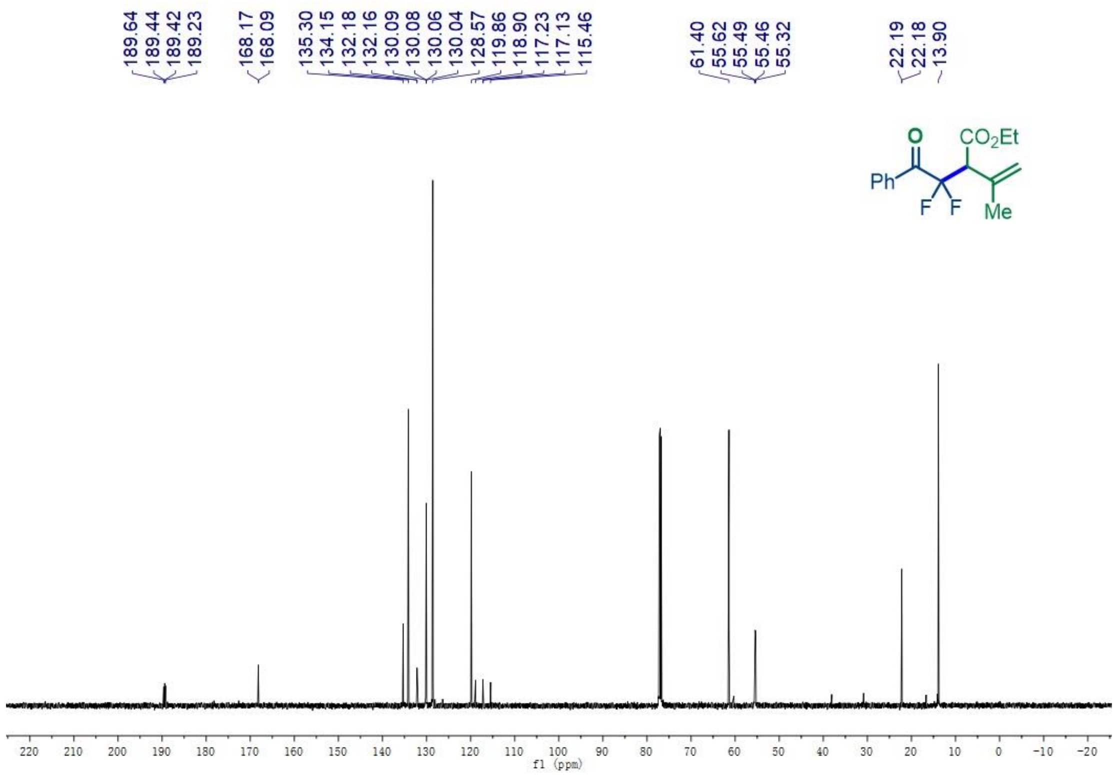

Supplementary Fig. 310 ^{13}C NMR (150 MHz, $CDCl_3$) spectrum of compound **103**

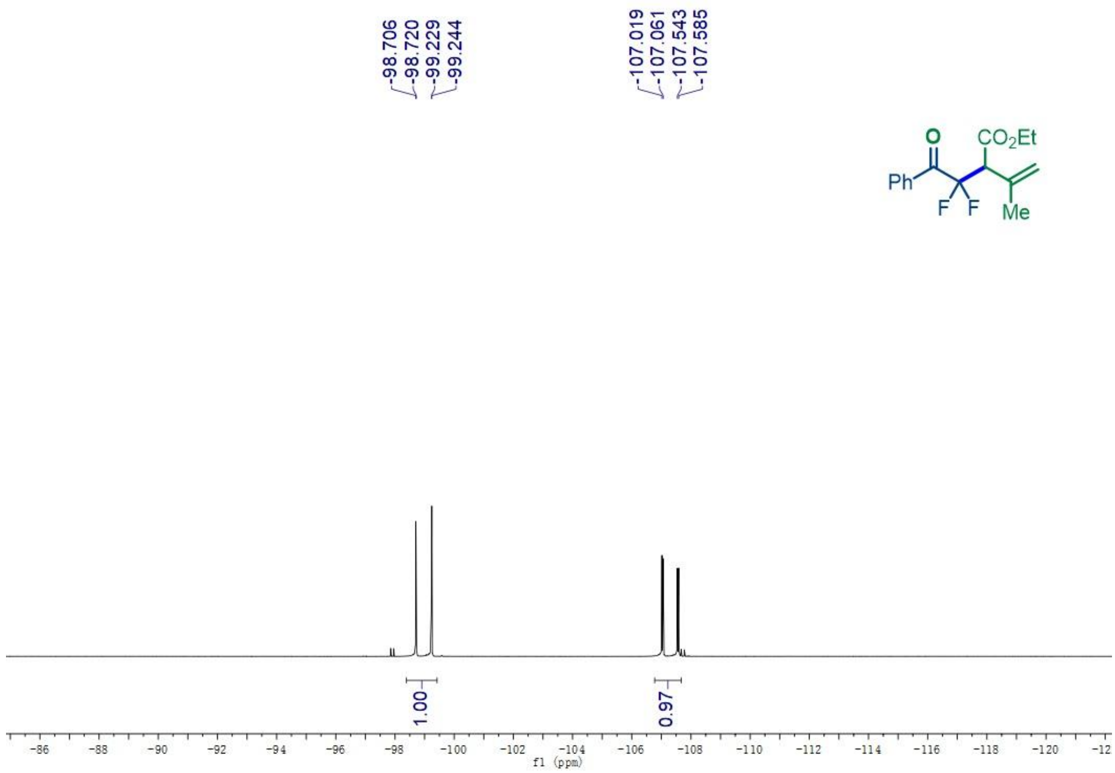

Supplementary Fig. 311 ^{19}F NMR (564 MHz, $CDCl_3$) spectrum of compound **103**

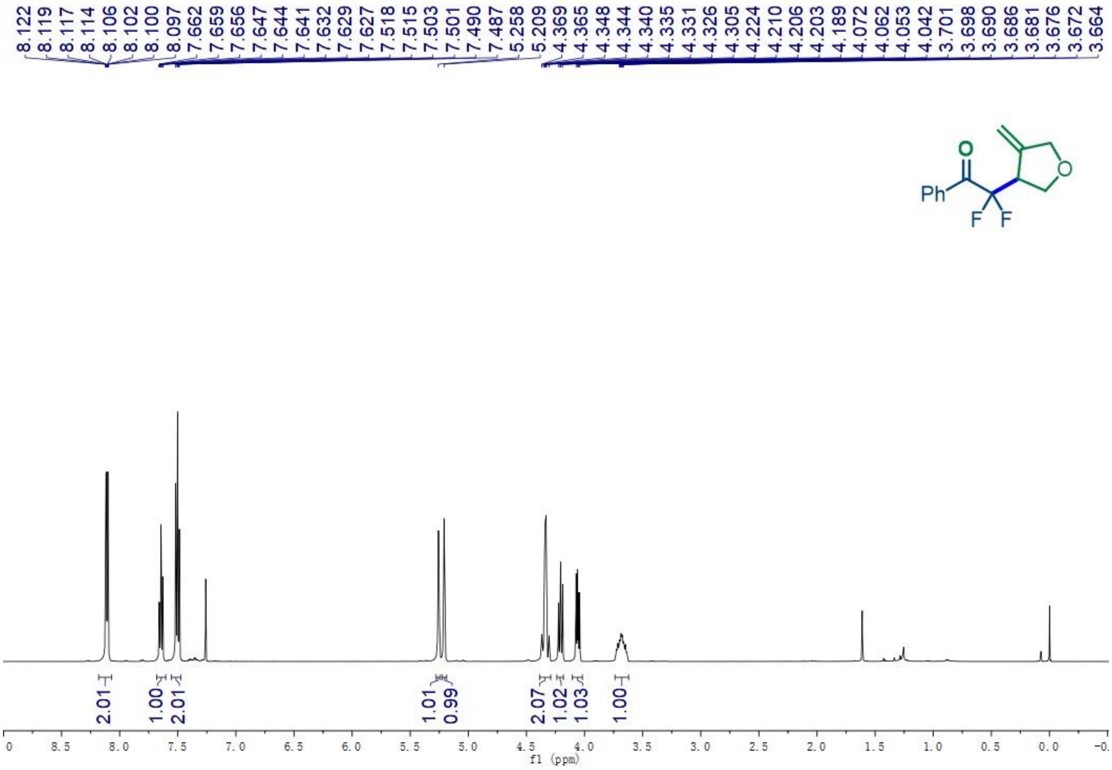

Supplementary Fig. 312 ^{1}H NMR (500 MHz, $CDCl_3$) spectrum of compound **104**

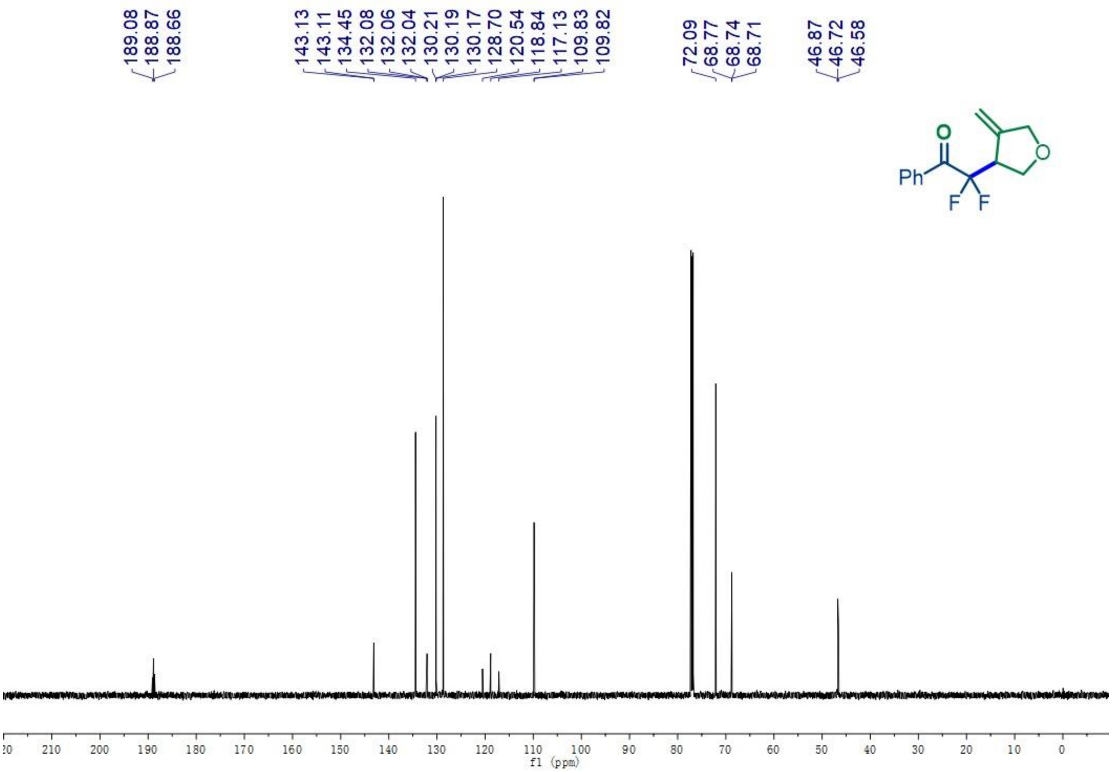

Supplementary Fig. 313 ^{13}C NMR (150 MHz, $CDCl_3$) spectrum of compound **104**

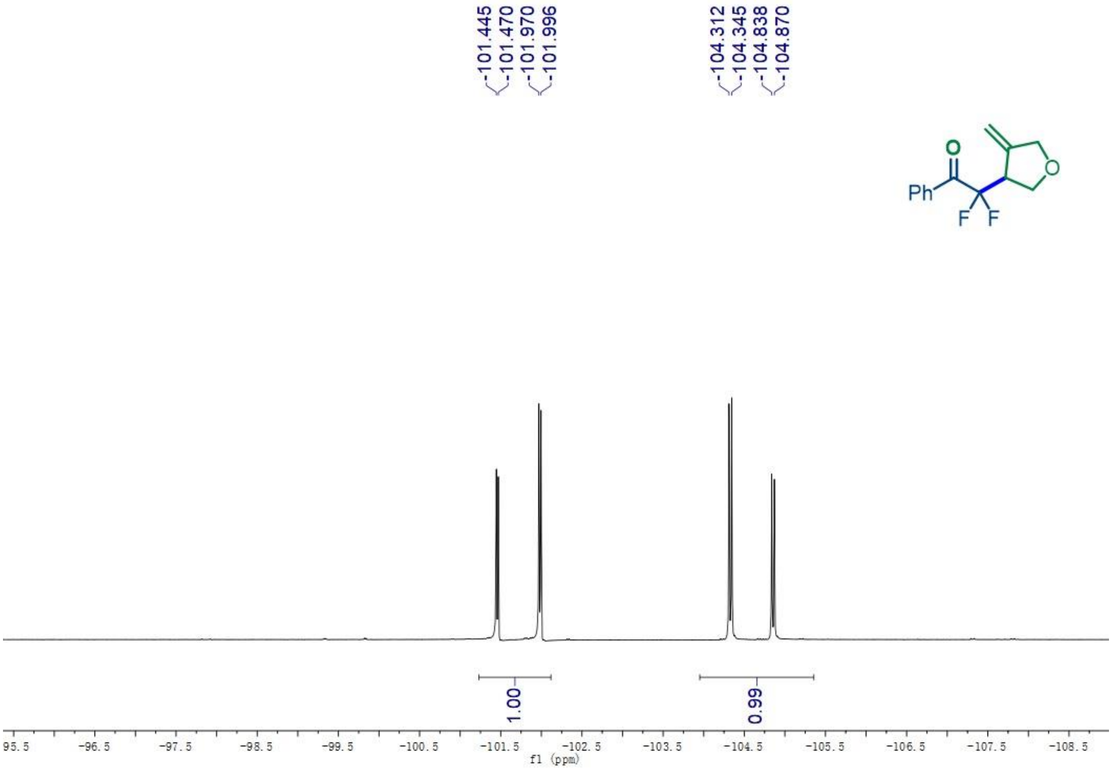

Supplementary Fig. 314 ^{19}F NMR (564 MHz, $CDCl_3$) spectrum of compound **104**

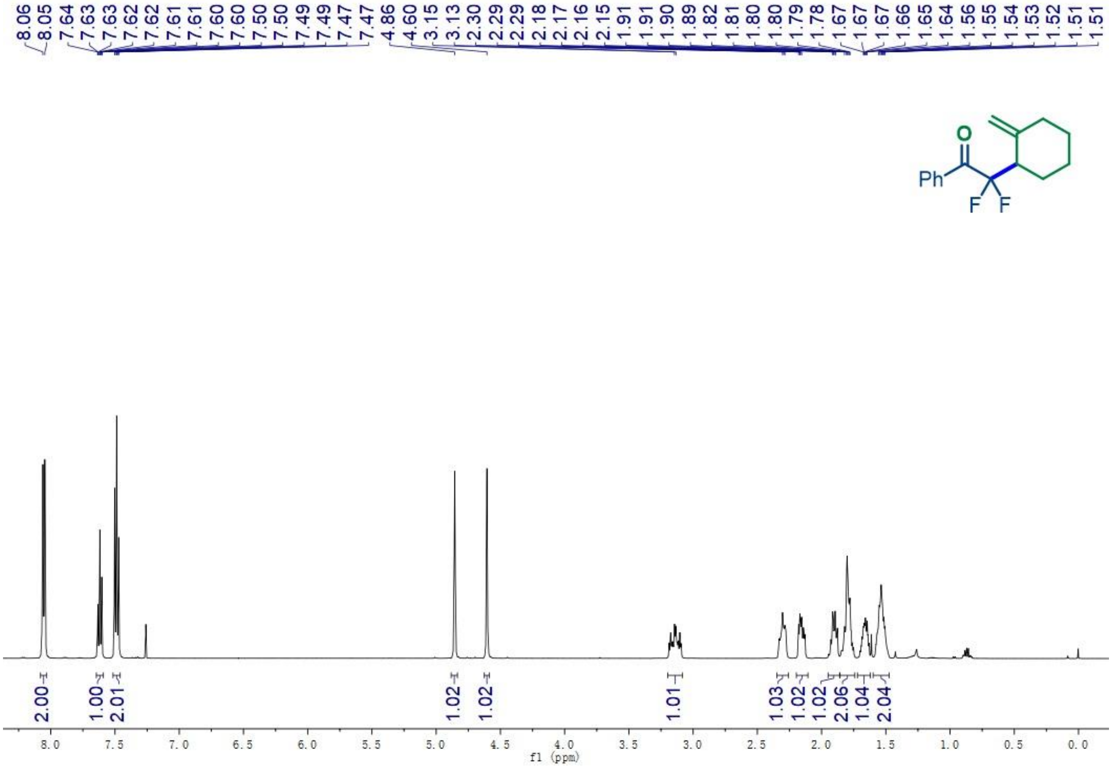

Supplementary Fig. 315 ^{1}H NMR (500 MHz, $CDCl_3$) spectrum of compound **105**

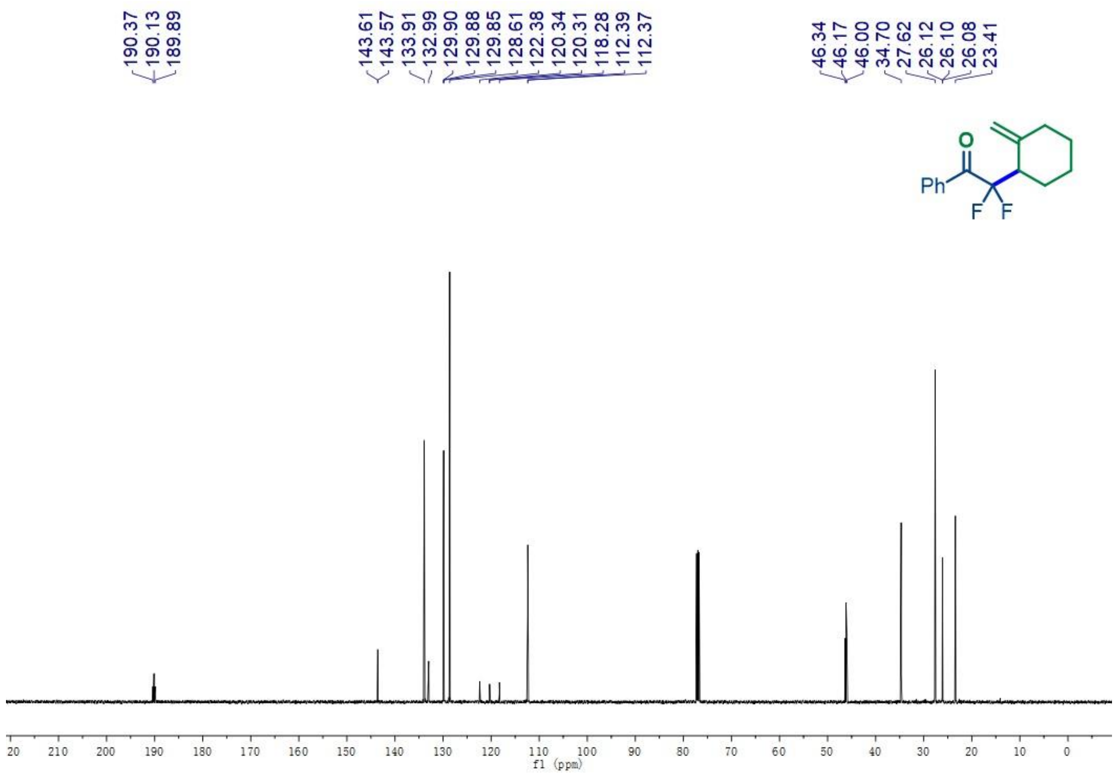

Supplementary Fig. 316 ^{13}C NMR (125 MHz, $CDCl_3$) spectrum of compound **105**

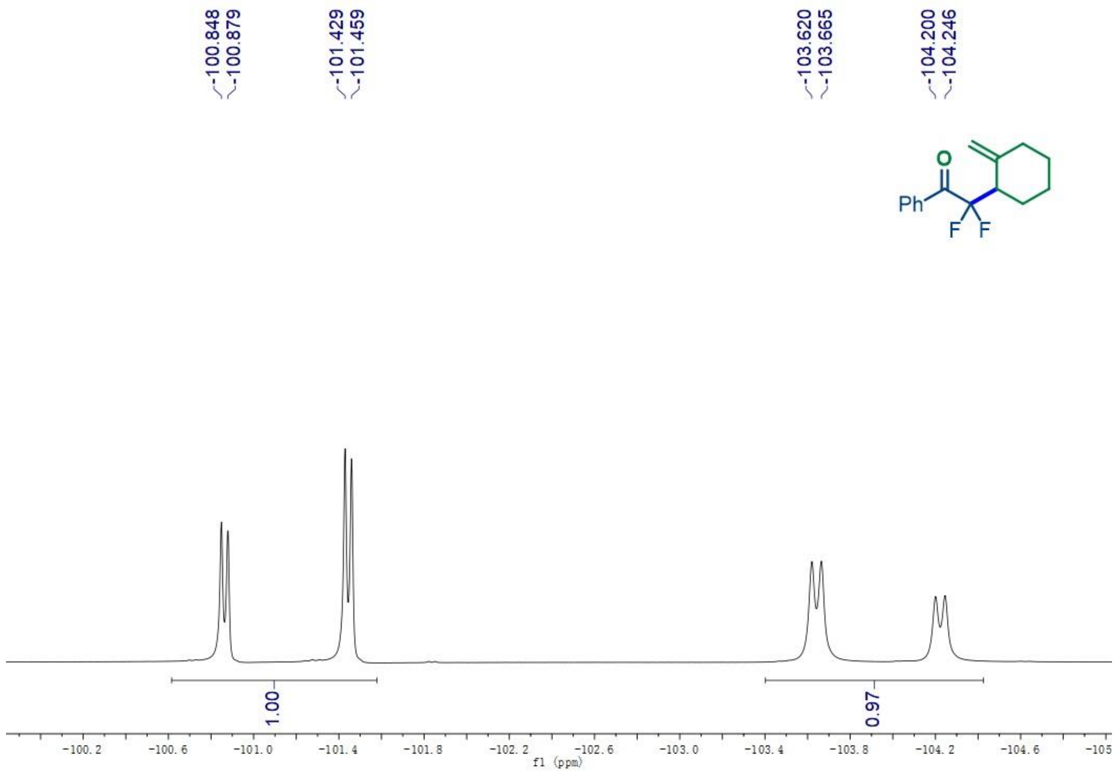

Supplementary Fig. 317 ^{19}F NMR (470 MHz, $CDCl_3$) spectrum of compound **105**

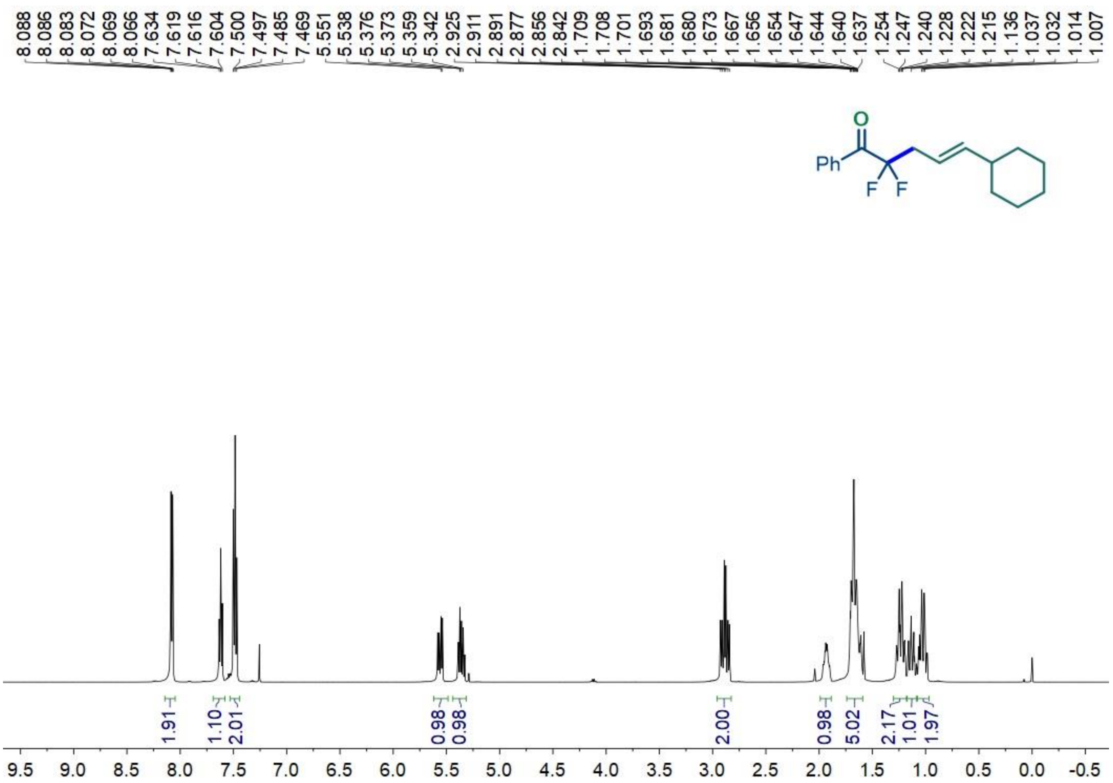

Supplementary Fig. 318 ^{1}H NMR (500 MHz, $CDCl_3$) spectrum of compound **106**

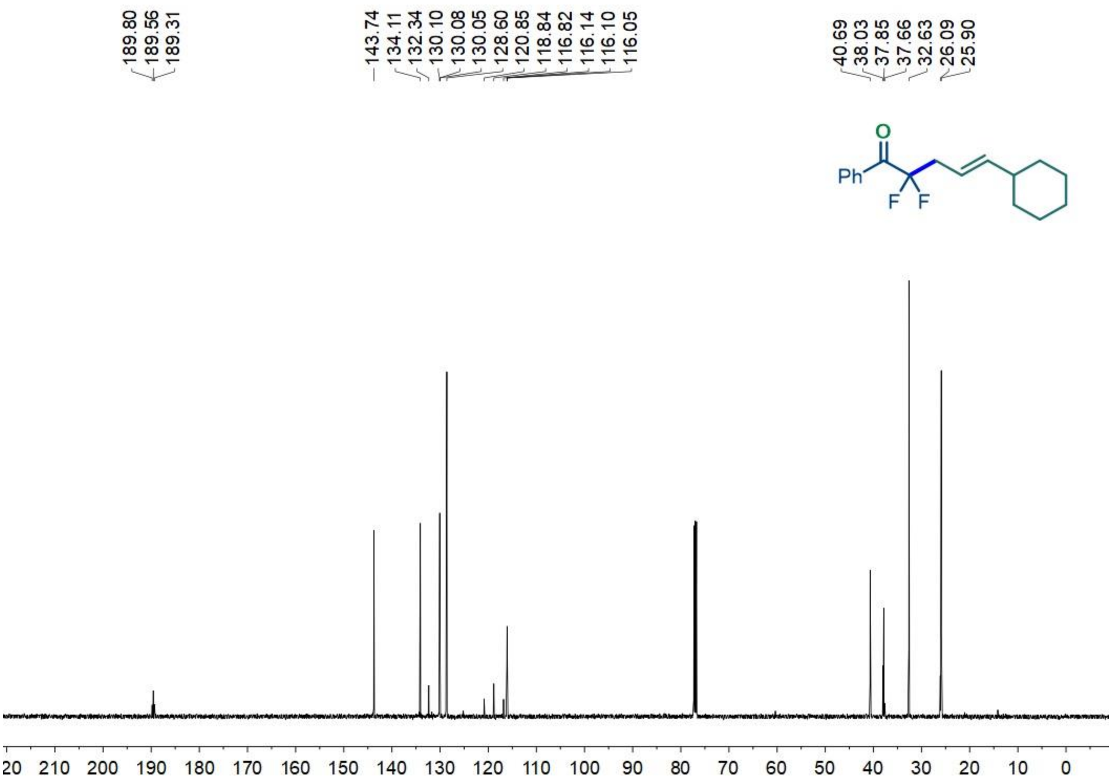

Supplementary Fig. 319 ^{13}C NMR (125 MHz, $CDCl_3$) spectrum of compound **106**

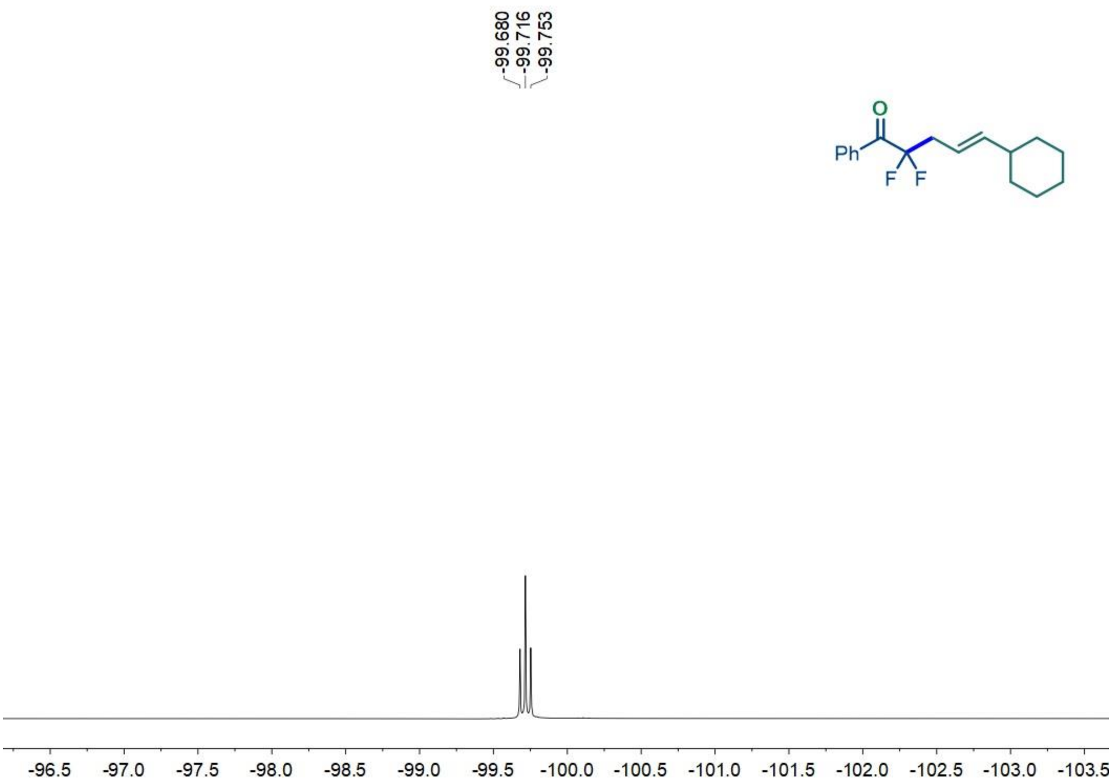

Supplementary Fig. 320 ^{19}F NMR (470 MHz, $CDCl_3$) spectrum of compound **106**

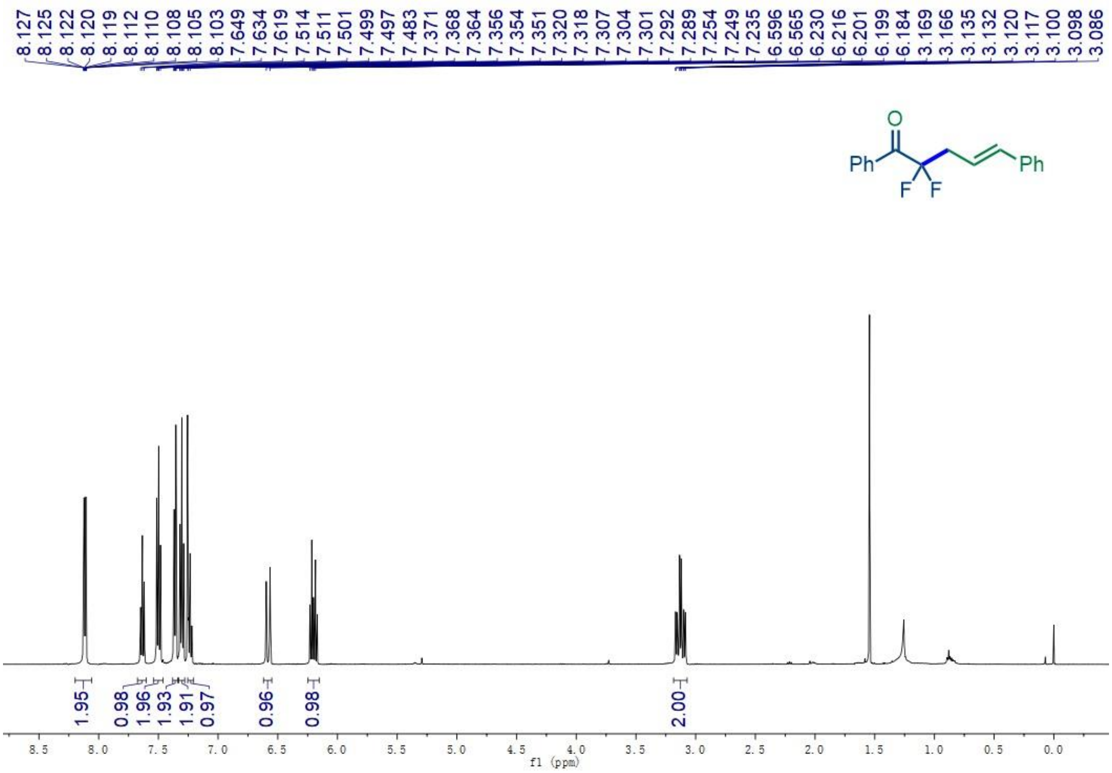

Supplementary Fig. 321 ^{1}H NMR (500 MHz, $CDCl_3$) spectrum of compound **107**

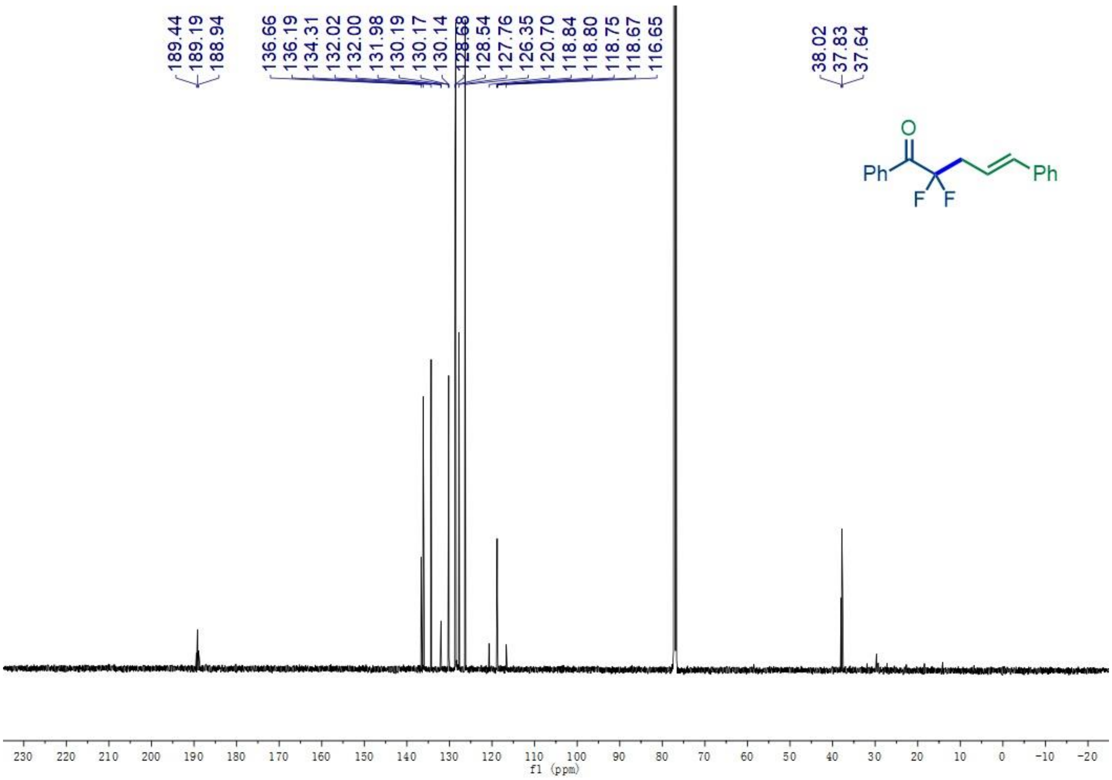

Supplementary Fig. 322 ^{13}C NMR (125 MHz, $CDCl_3$) spectrum of compound **107**

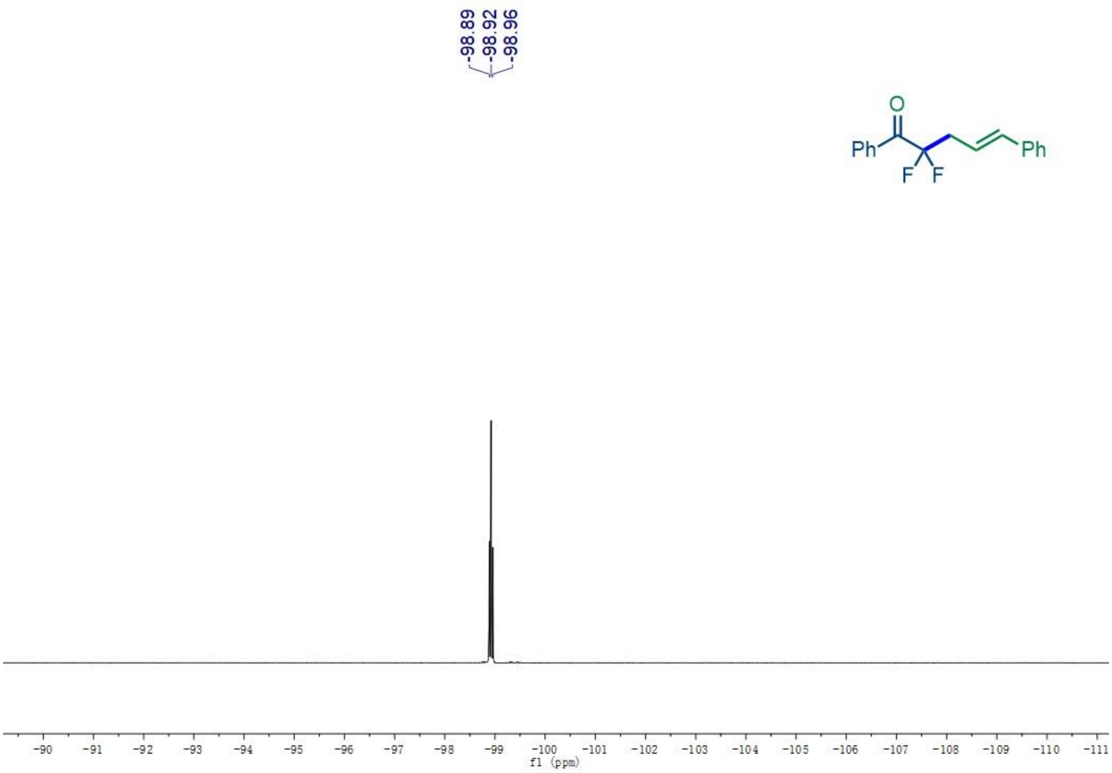

Supplementary Fig. 323 ^{19}F NMR (470 MHz, $CDCl_3$) spectrum of compound **107**

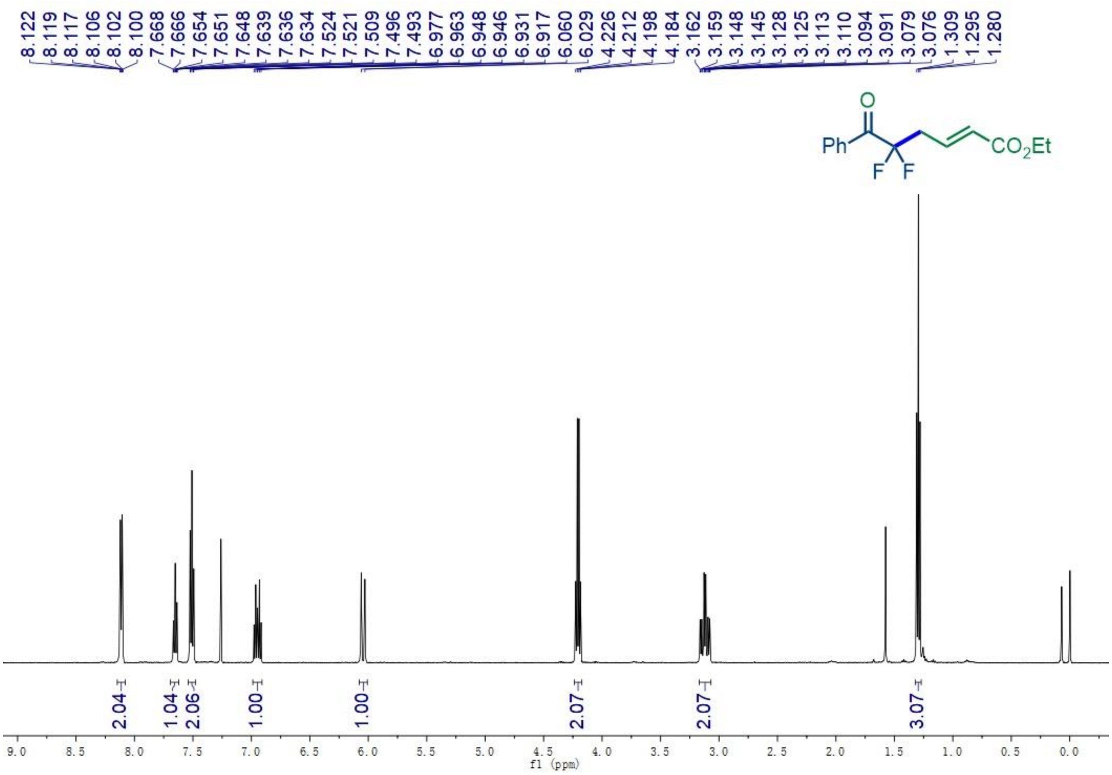

Supplementary Fig. 324 ^{1}H NMR (500 MHz, $CDCl_3$) spectrum of compound **108**

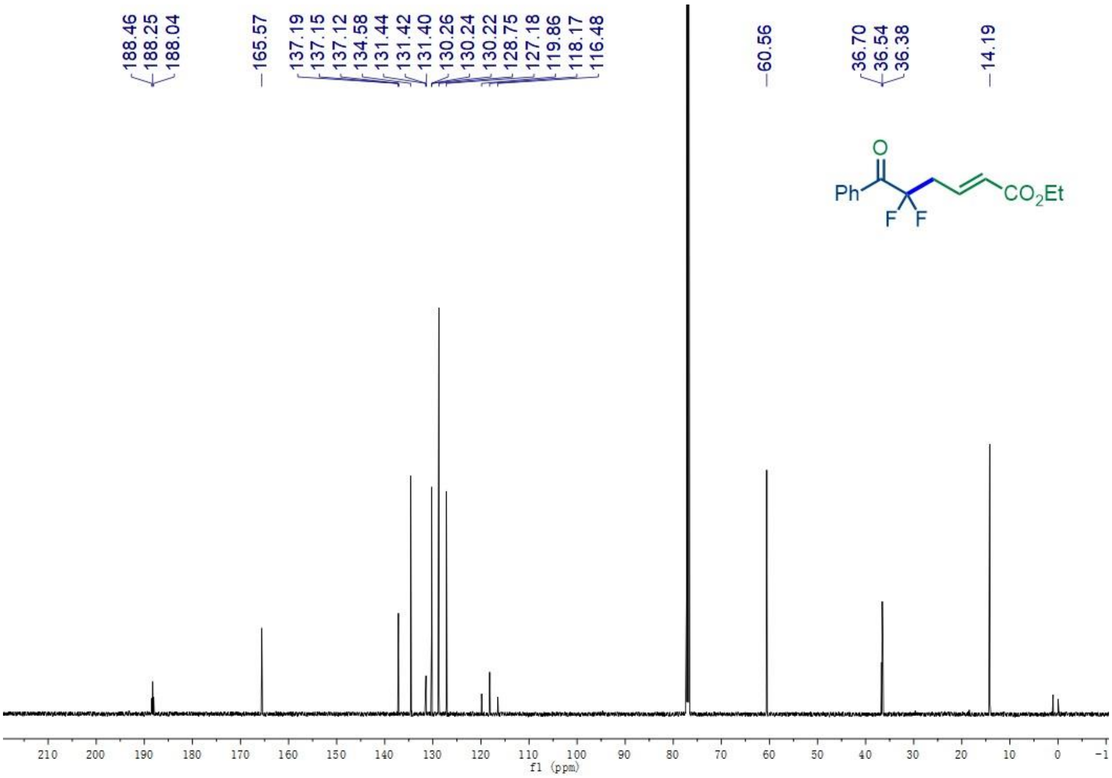

Supplementary Fig. 325 ^{13}C NMR (150 MHz, $CDCl_3$) spectrum of compound **108**

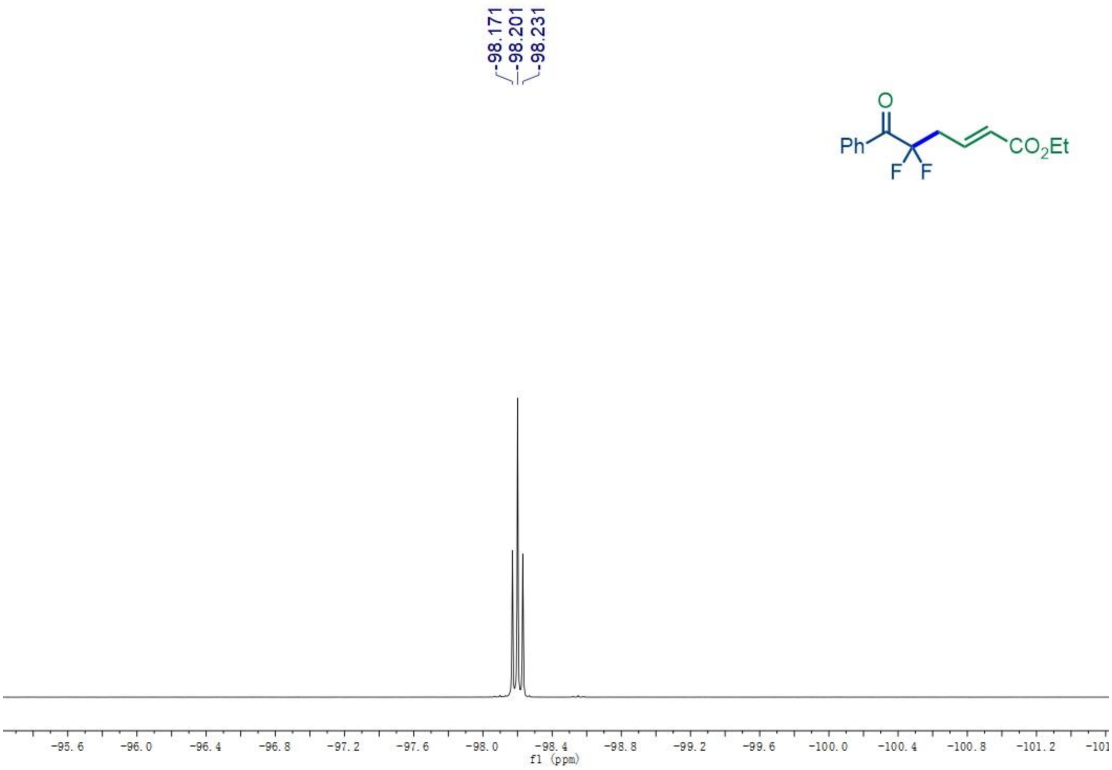

Supplementary Fig. 326 ^{19}F NMR (564 MHz, $CDCl_3$) spectrum of compound **108**

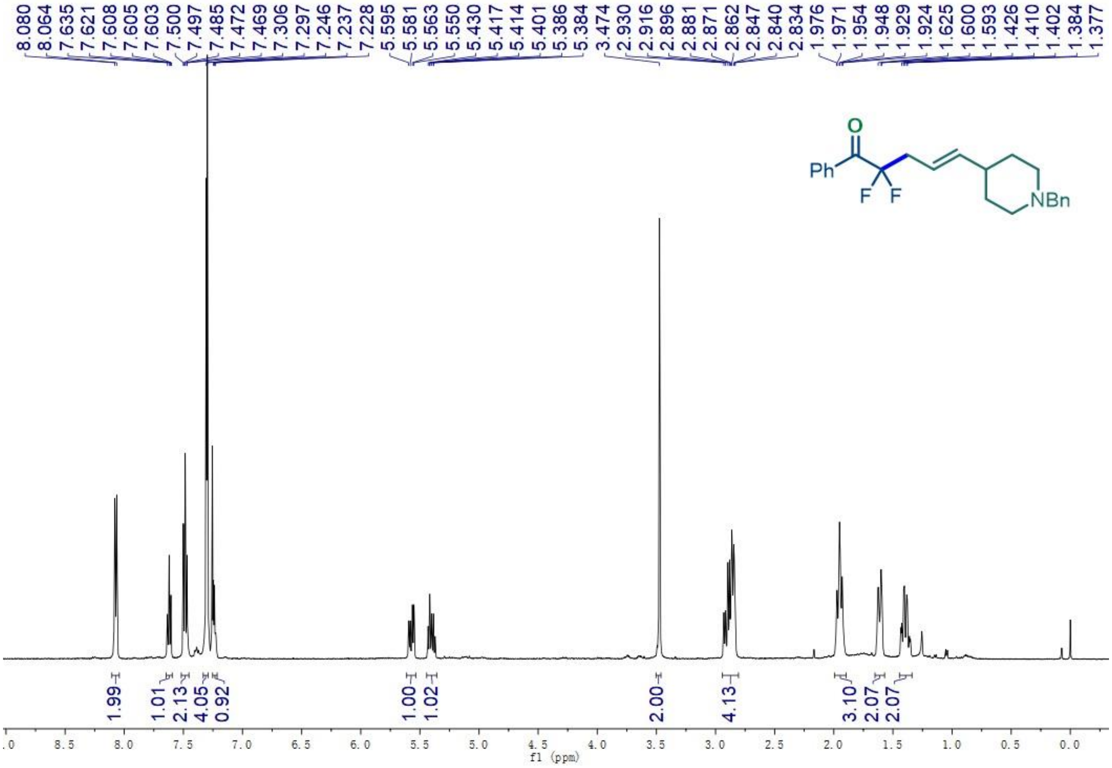

Supplementary Fig. 327 ^{1}H NMR (500 MHz, $CDCl_3$) spectrum of compound **109**

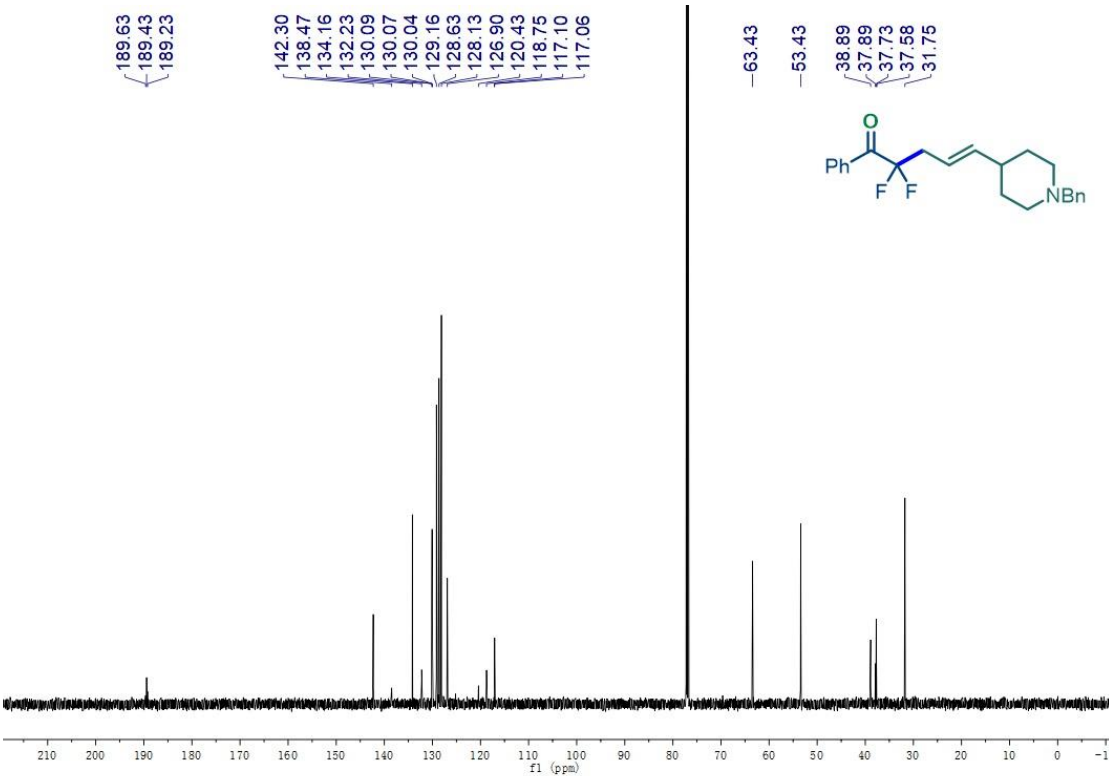

Supplementary Fig. 328 ^{13}C NMR (150 MHz, $CDCl_3$) spectrum of compound **109**

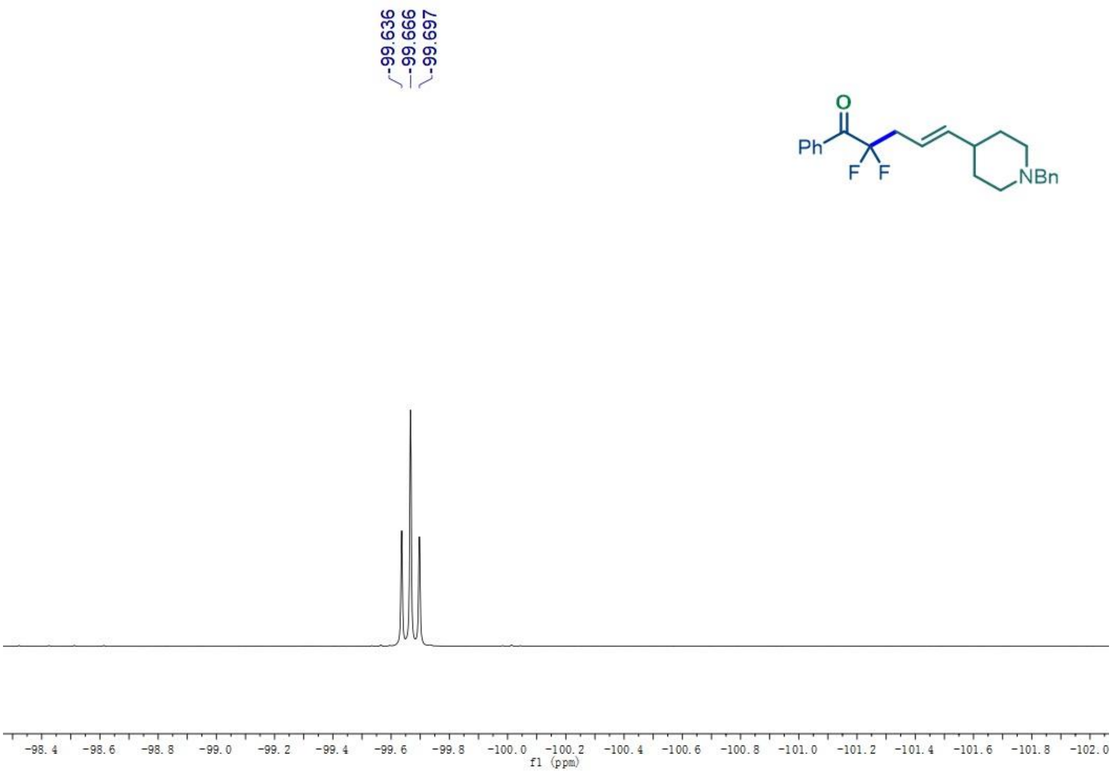

Supplementary Fig. 329 ^{19}F NMR (564 MHz, $CDCl_3$) spectrum of compound **109**

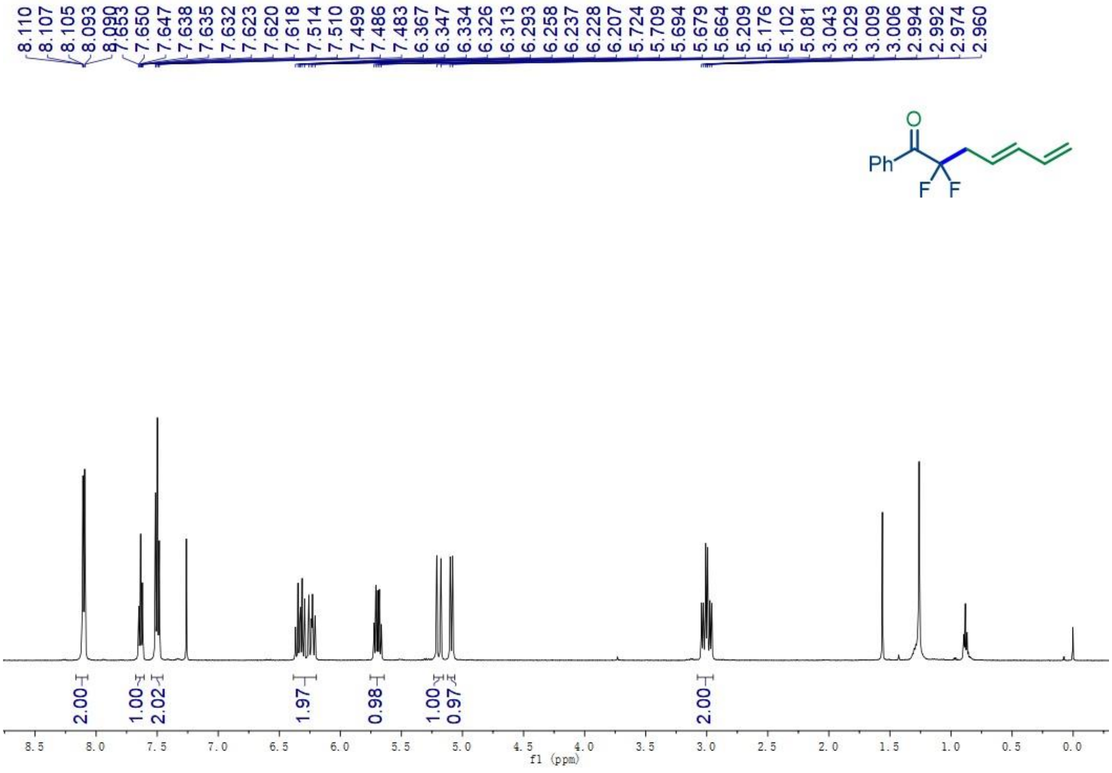

Supplementary Fig. 330 ^{1}H NMR (500 MHz, $CDCl_3$) spectrum of compound **110**

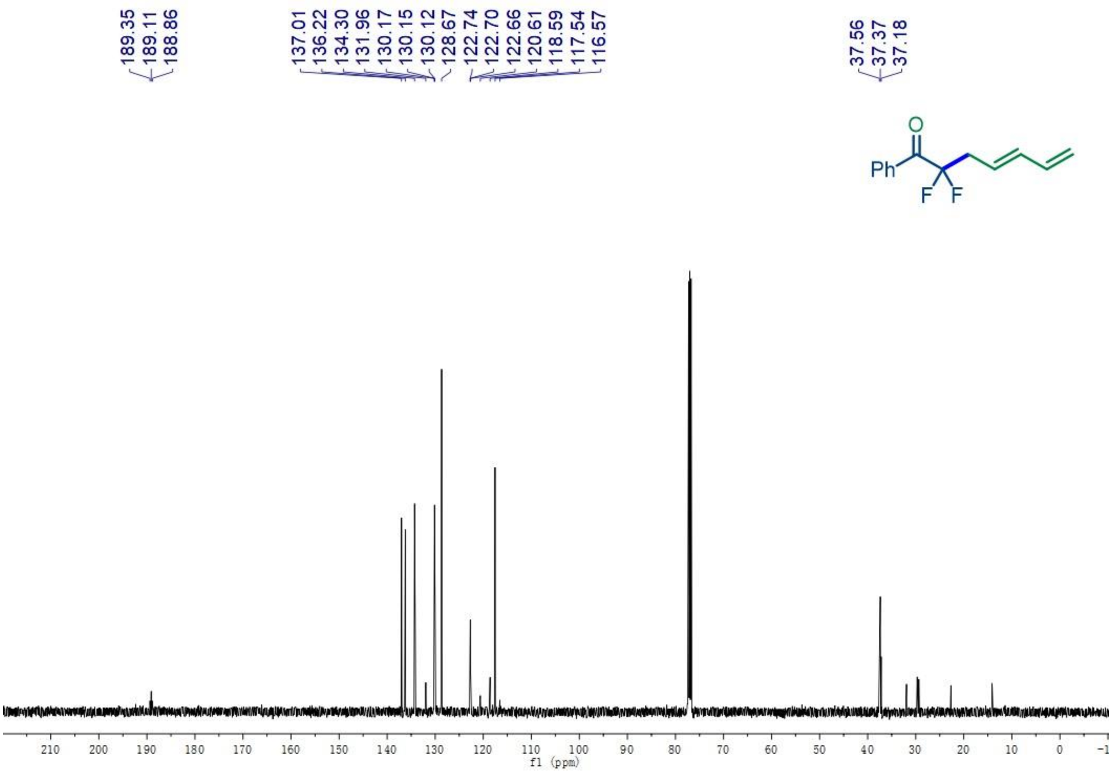

Supplementary Fig. 331 ^{13}C NMR (125 MHz, $CDCl_3$) spectrum of compound **110**

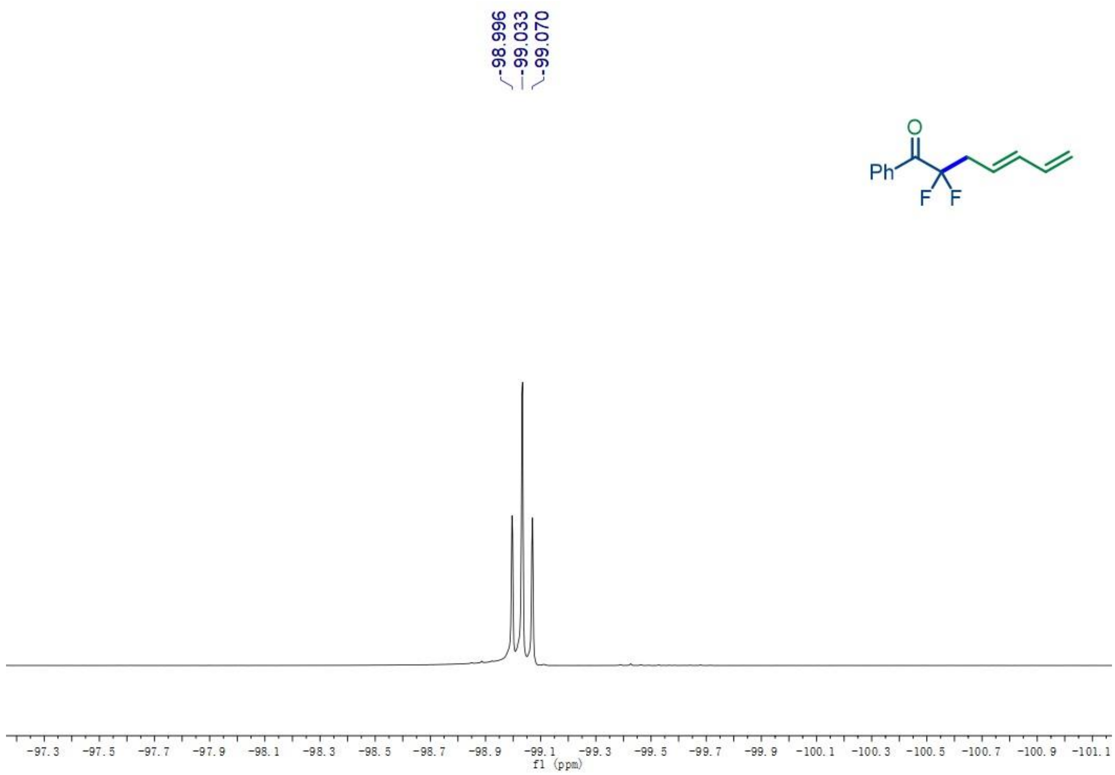

Supplementary Fig. 332 ^{19}F NMR (470 MHz, $CDCl_3$) spectrum of compound **110**

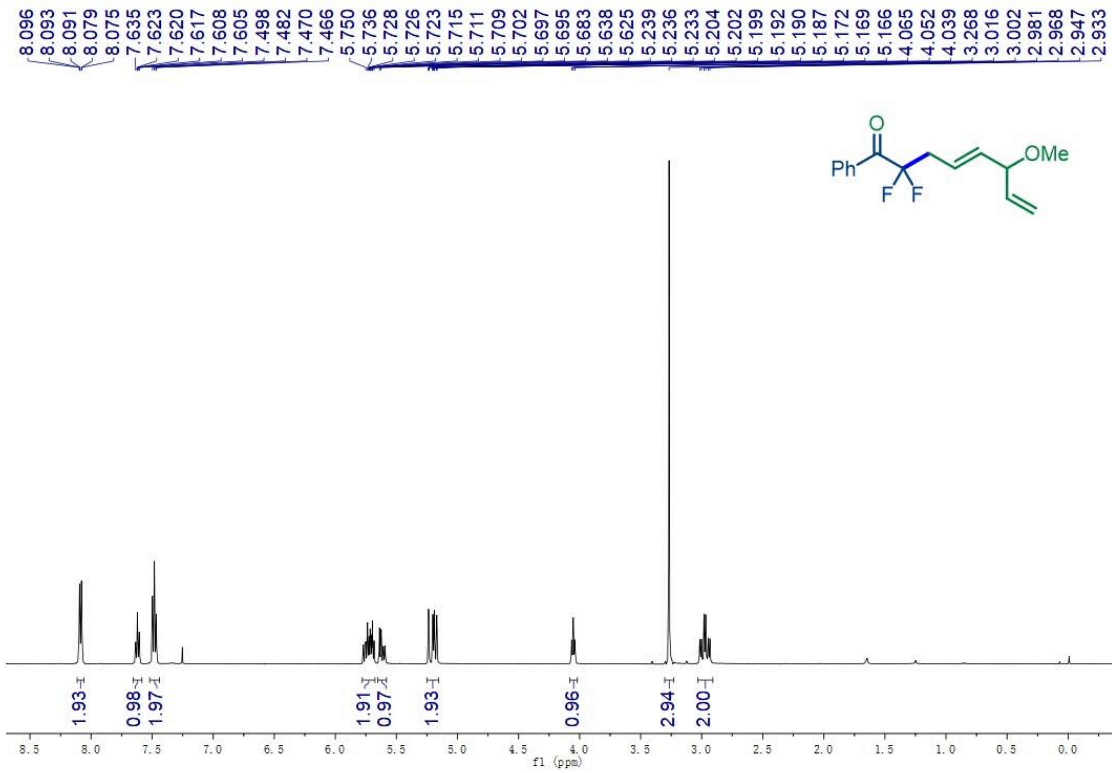

Supplementary Fig. 333 ^{1}H NMR (500 MHz, $CDCl_3$) spectrum of compound **111**

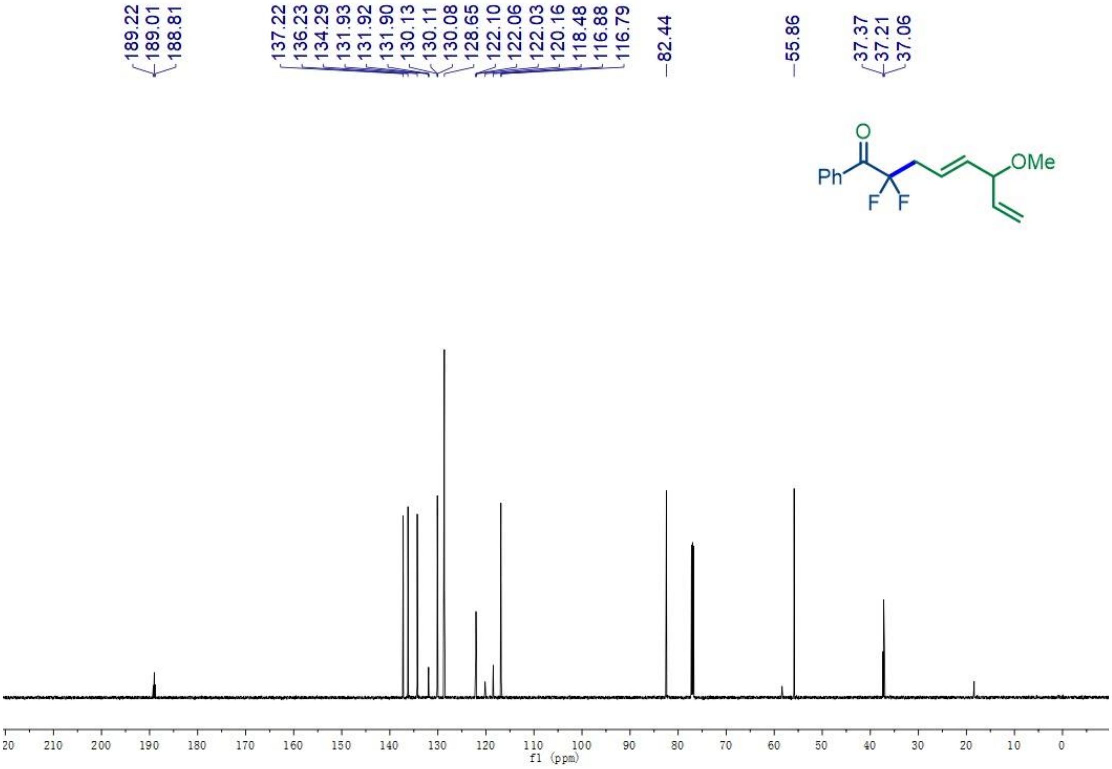

Supplementary Fig. 334 ^{13}C NMR (150 MHz, $CDCl_3$) spectrum of compound **111**

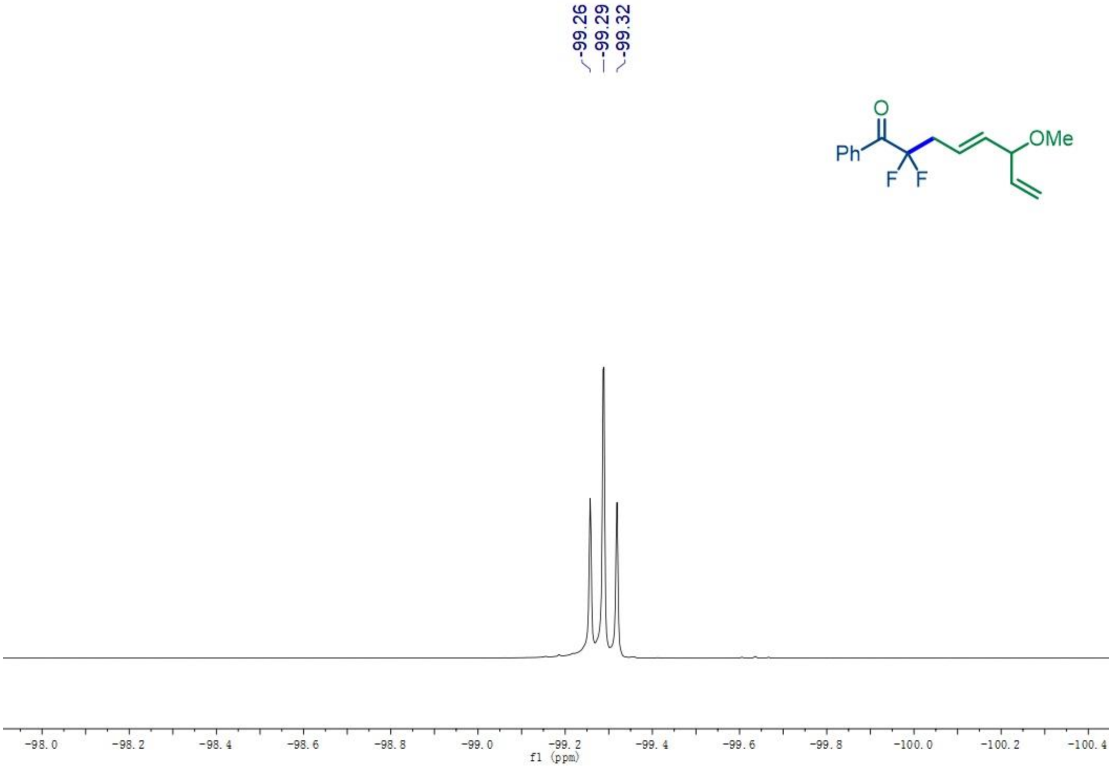

Supplementary Fig. 335 ^{19}F NMR (564 MHz, $CDCl_3$) spectrum of compound **111**

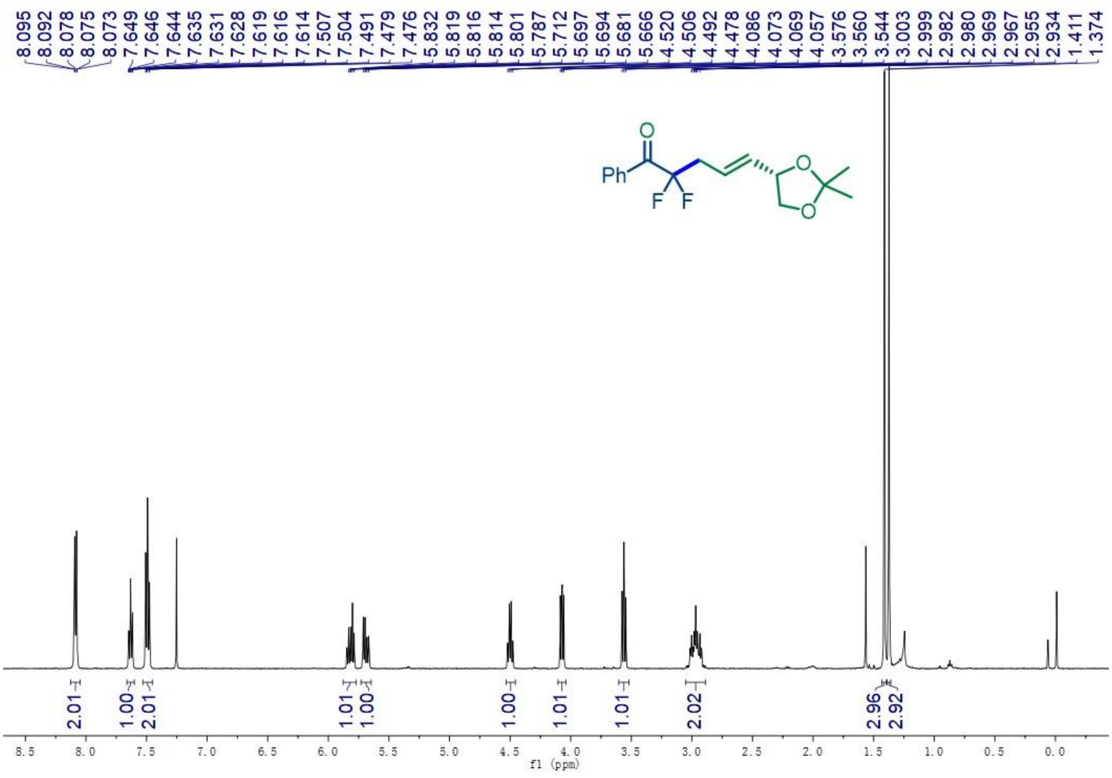

Supplementary Fig. 336 ^{1}H NMR (500 MHz, $CDCl_3$) spectrum of compound **112**

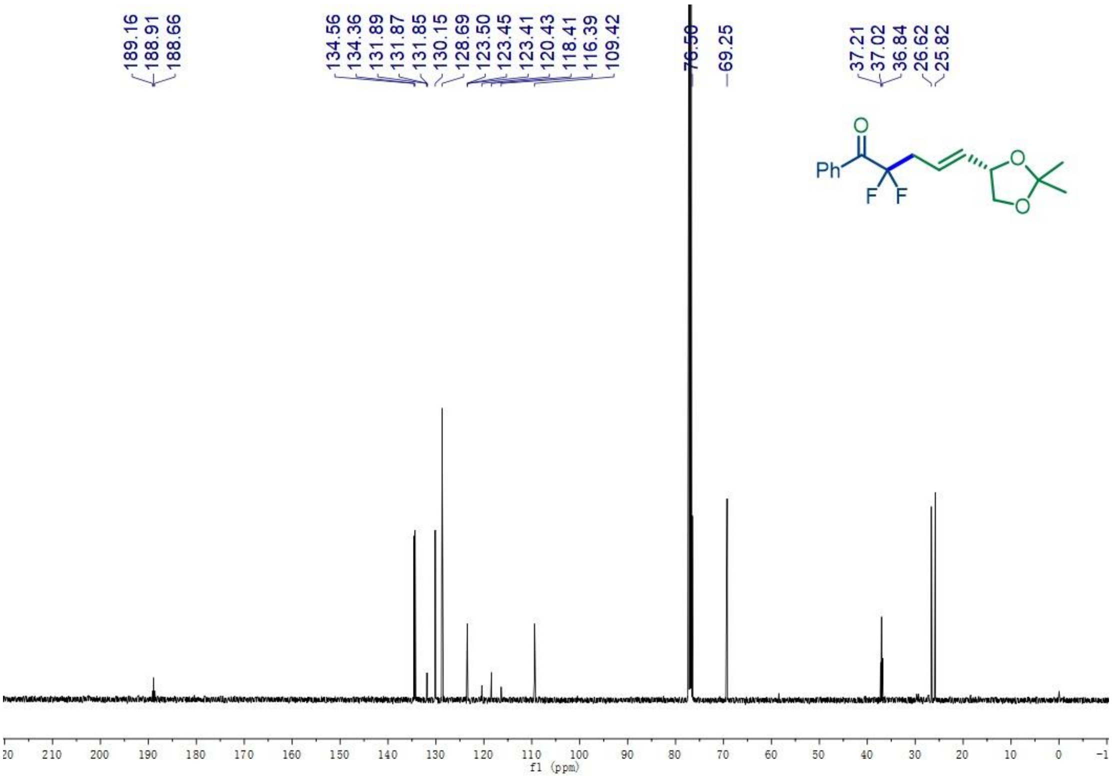

Supplementary Fig. 337 ^{13}C NMR (125 MHz, $CDCl_3$) spectrum of compound **112**

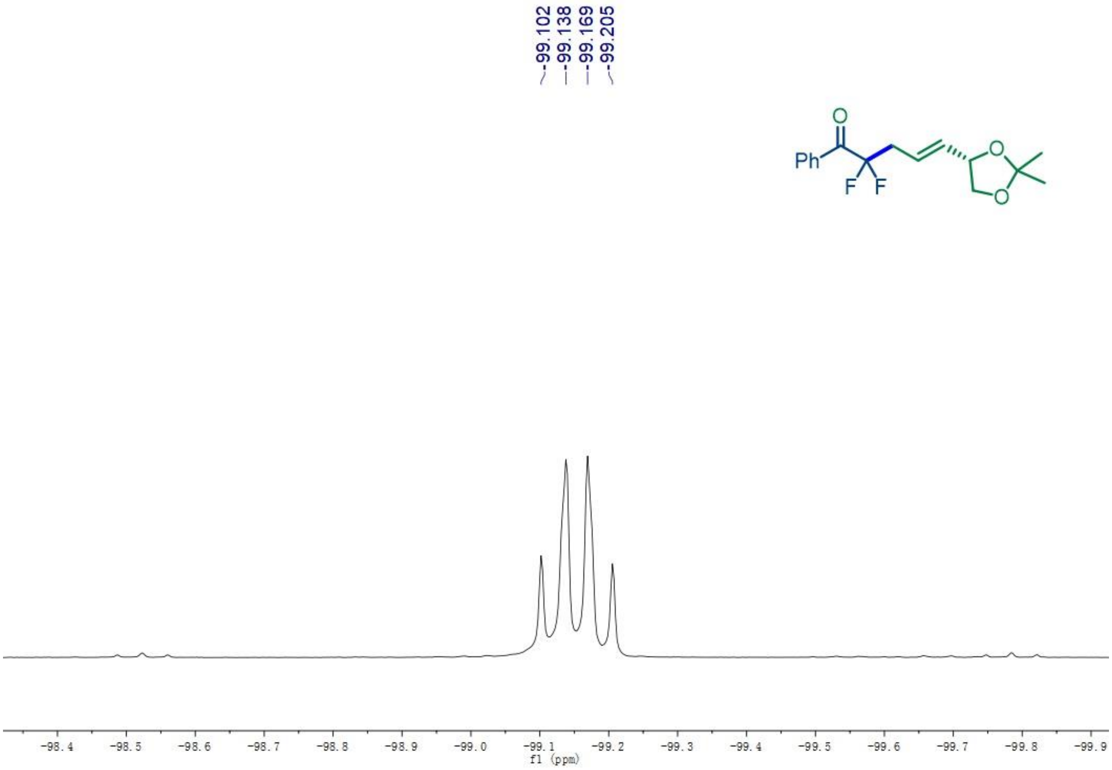

Supplementary Fig. 338 ^{19}F NMR (470 MHz, $CDCl_3$) spectrum of compound **112**

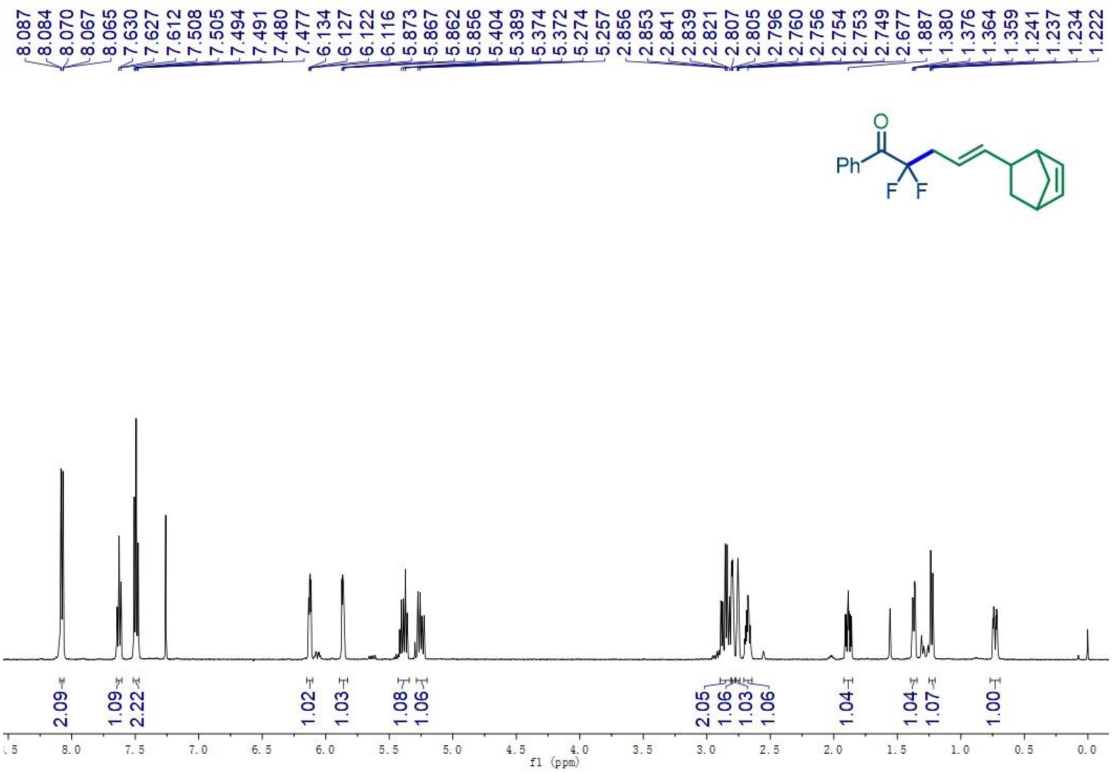

Supplementary Fig. 339 ^{1}H NMR (500 MHz, $CDCl_3$) spectrum of compound **113**

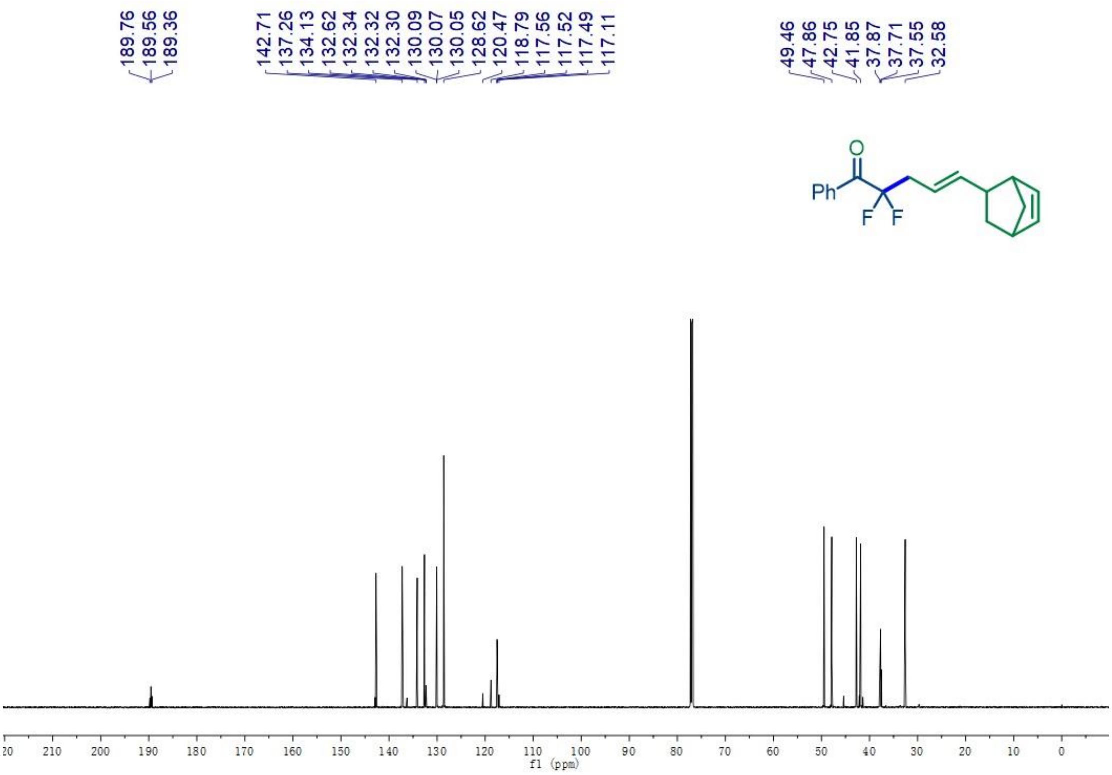

Supplementary Fig. 340 ^{13}C NMR (150 MHz, $CDCl_3$) spectrum of compound **113**

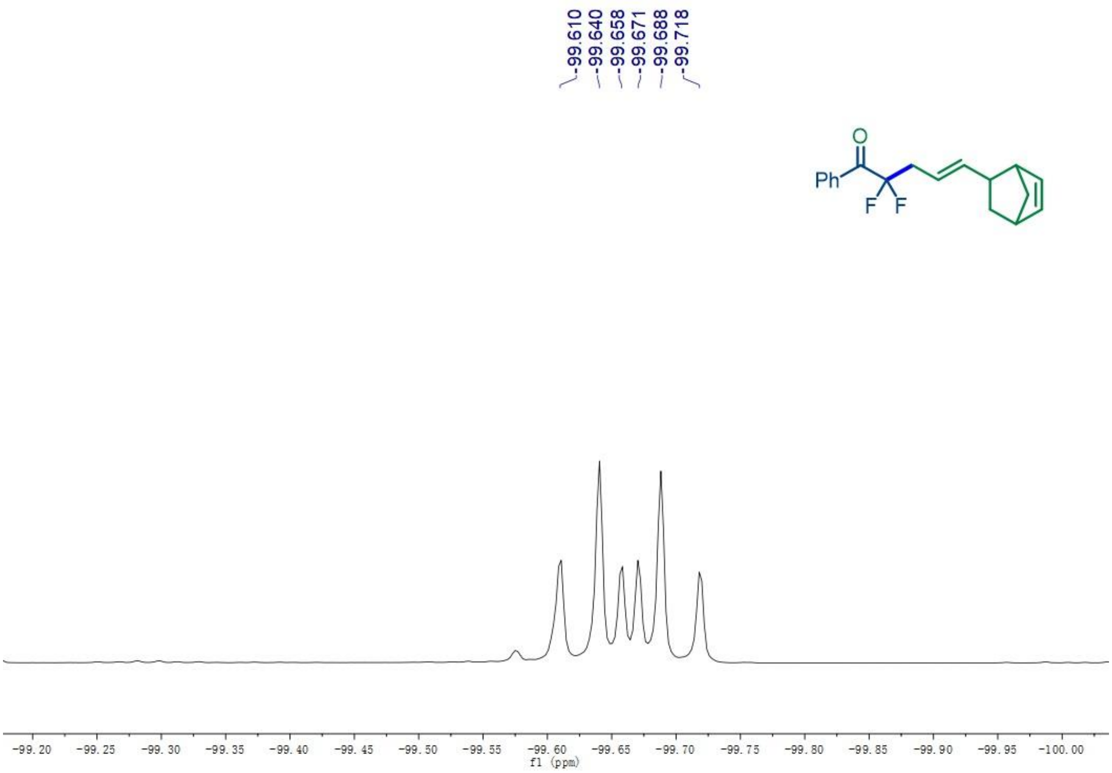

Supplementary Fig. 341 ^{19}F NMR (564 MHz, $CDCl_3$) spectrum of compound **113**

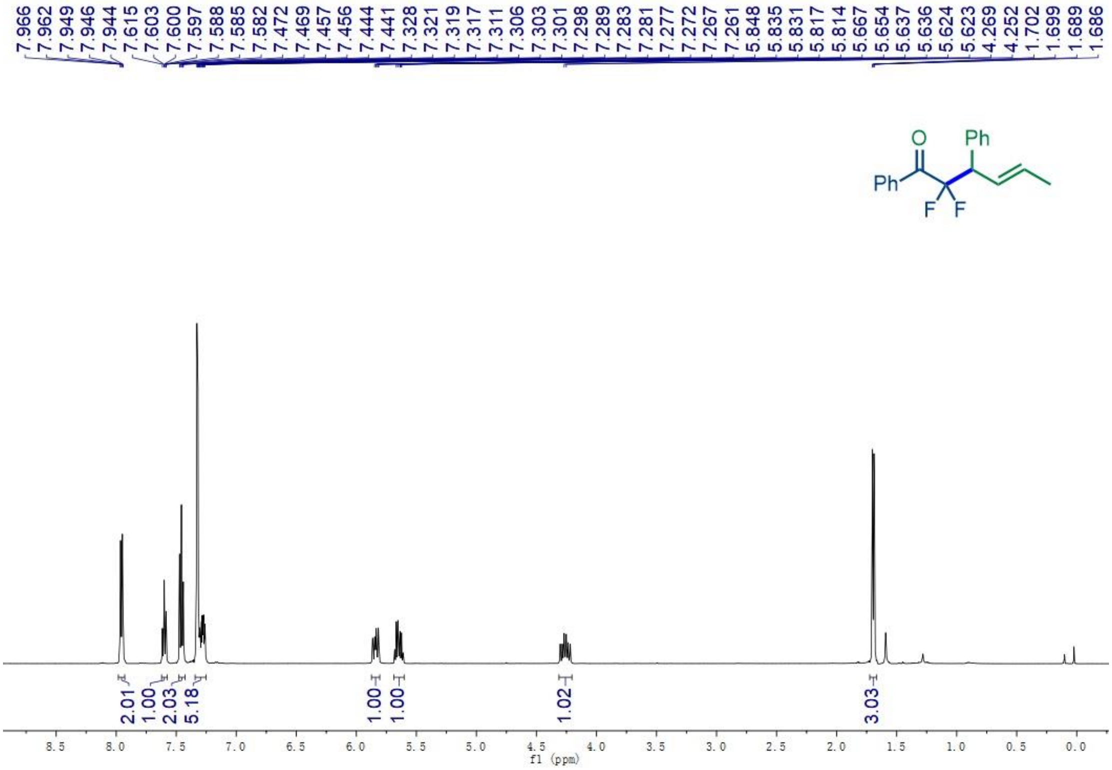

Supplementary Fig. 342 ^{1}H NMR (500 MHz, $CDCl_3$) spectrum of compound **114**

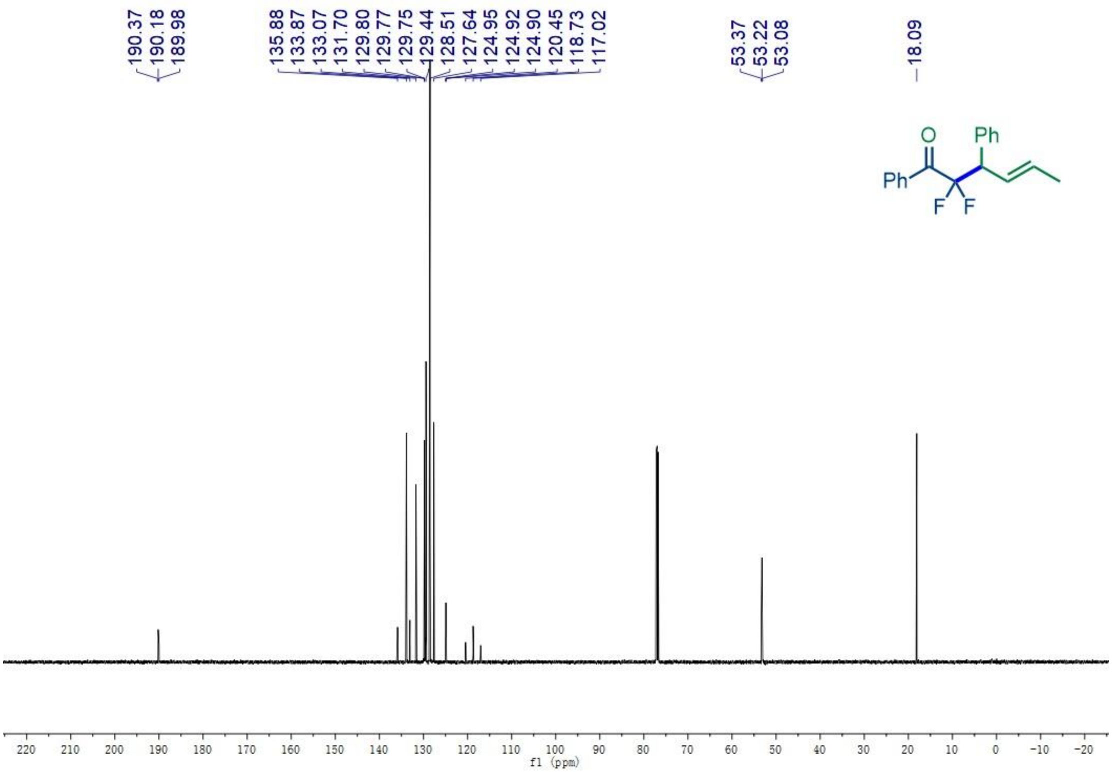

Supplementary Fig. 343 ^{13}C NMR (150 MHz, $CDCl_3$) spectrum of compound **114**

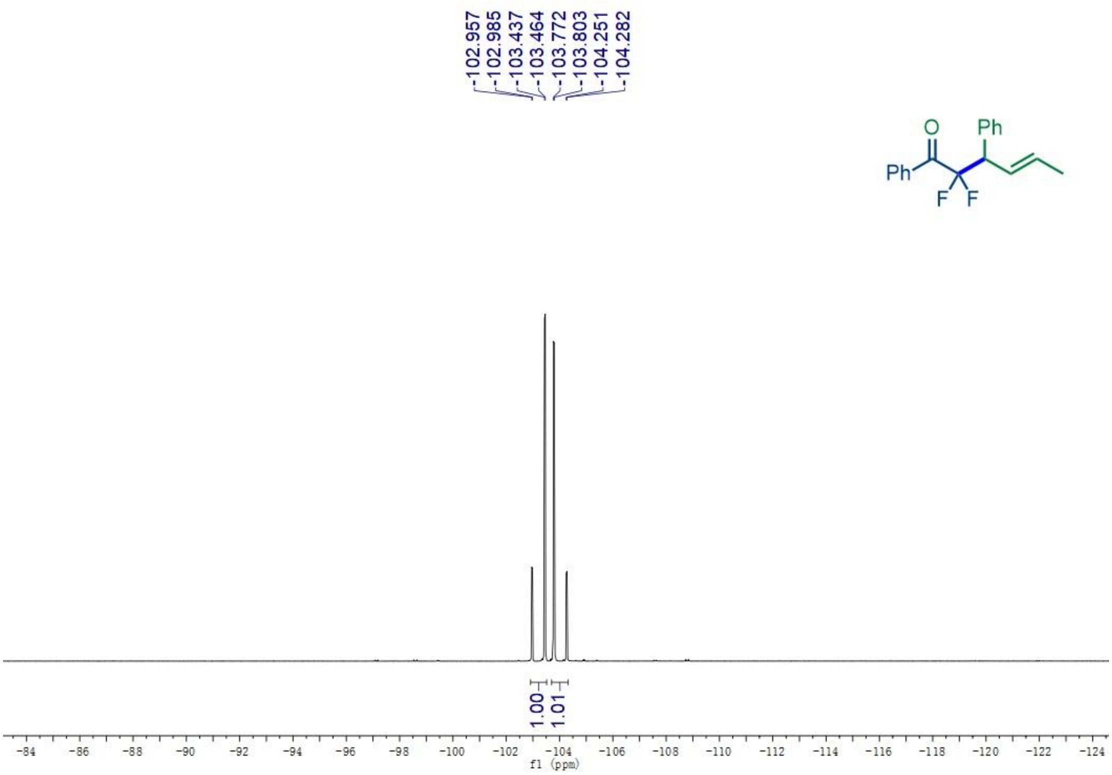

Supplementary Fig. 344 ^{19}F NMR (564 MHz, $CDCl_3$) spectrum of compound **114**

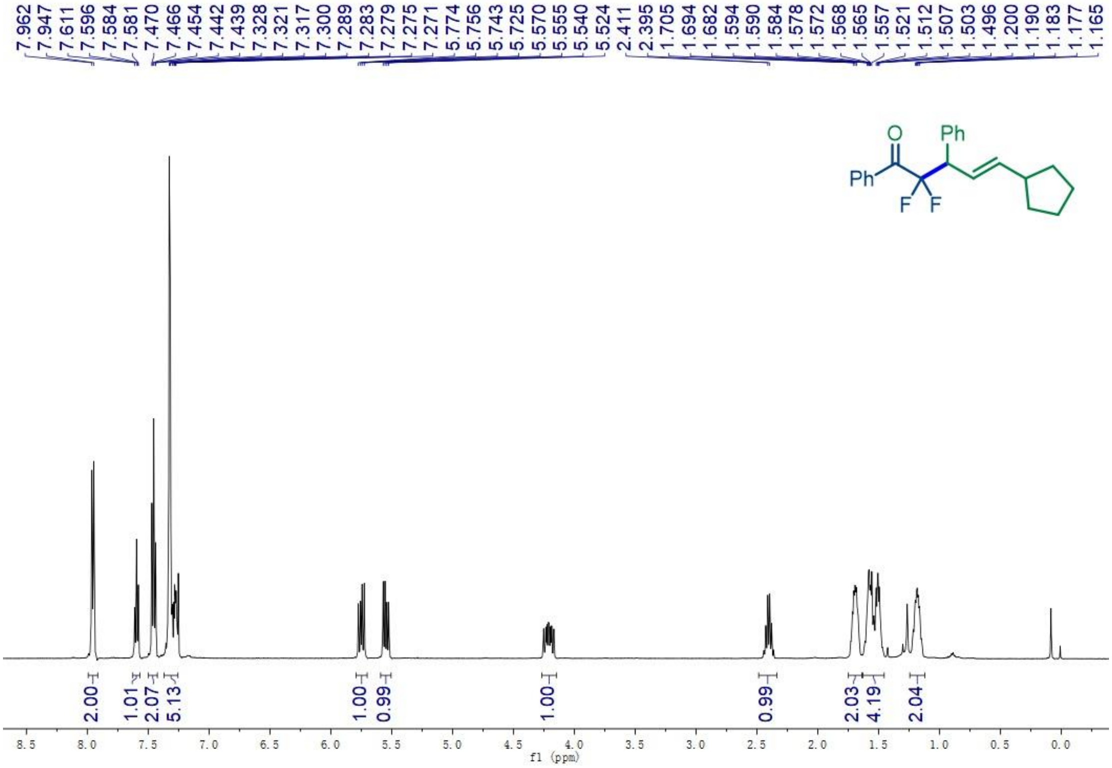

Supplementary Fig. 345 ^{1}H NMR (500 MHz, $CDCl_3$) spectrum of compound **115**

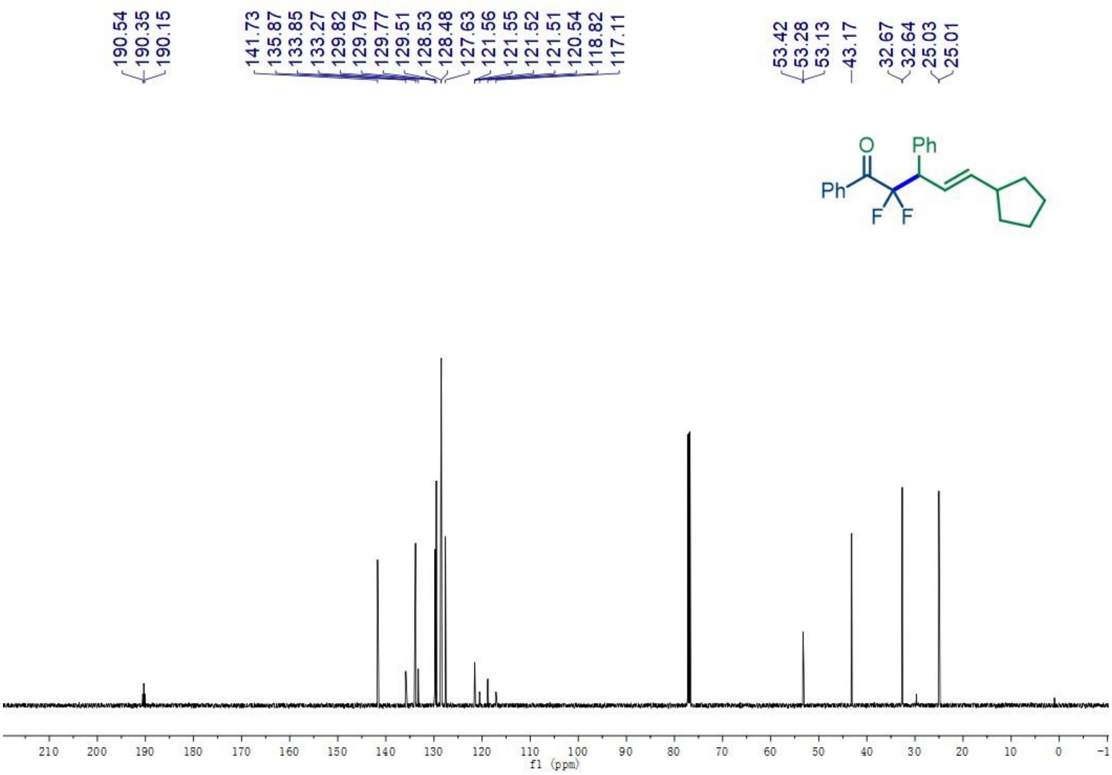

Supplementary Fig. 346 ^{13}C NMR (150 MHz, $CDCl_3$) spectrum of compound **115**

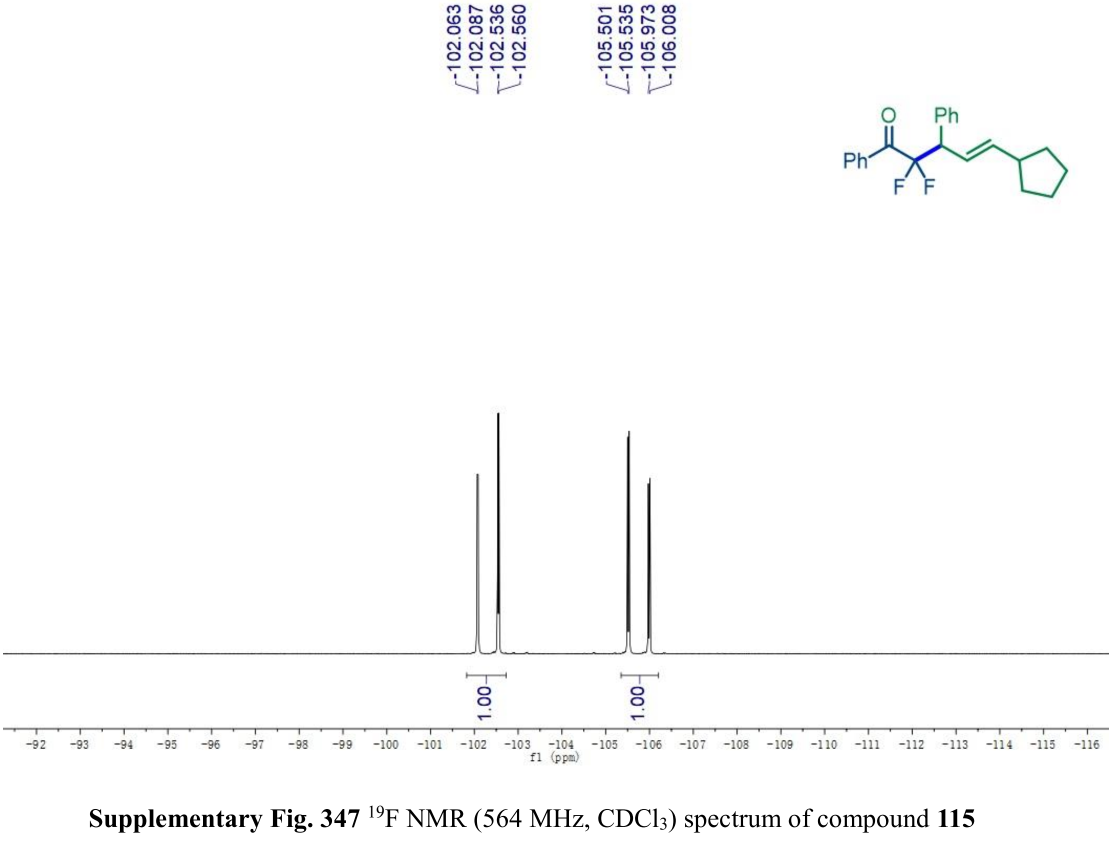

Supplementary Fig. 347 ^{19}F NMR (564 MHz, $CDCl_3$) spectrum of compound **115**

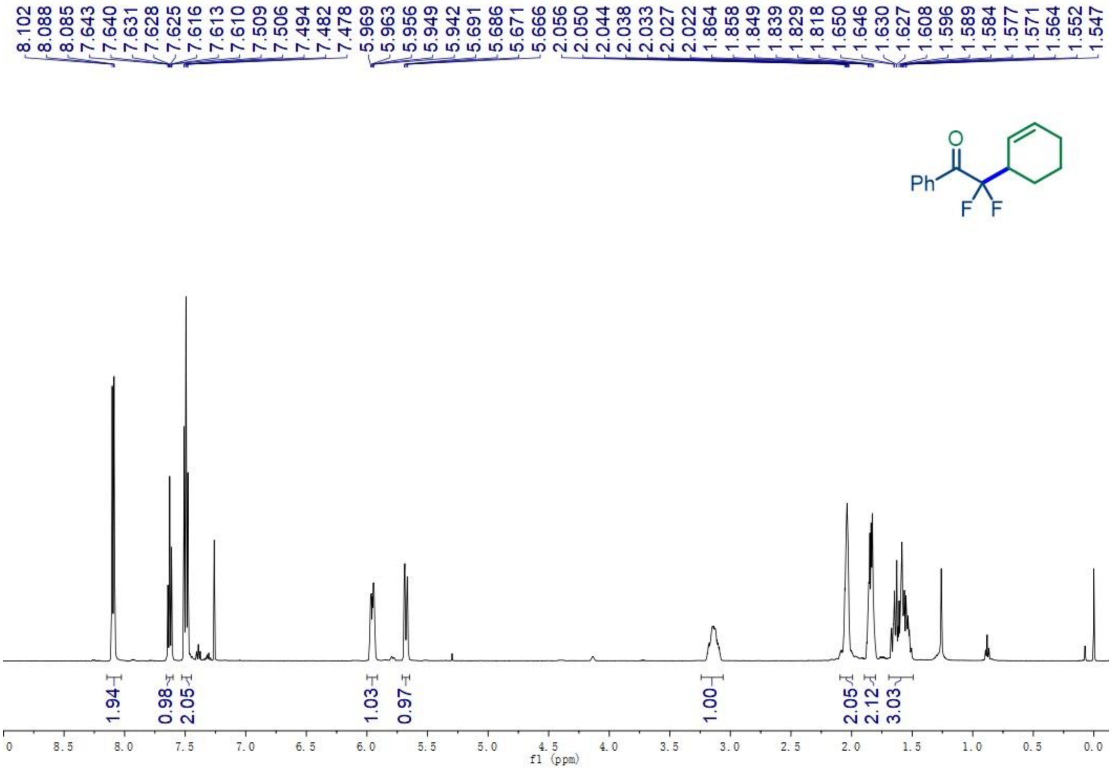

Supplementary Fig. 348 ^{1}H NMR (500 MHz, $CDCl_3$) spectrum of compound **116**

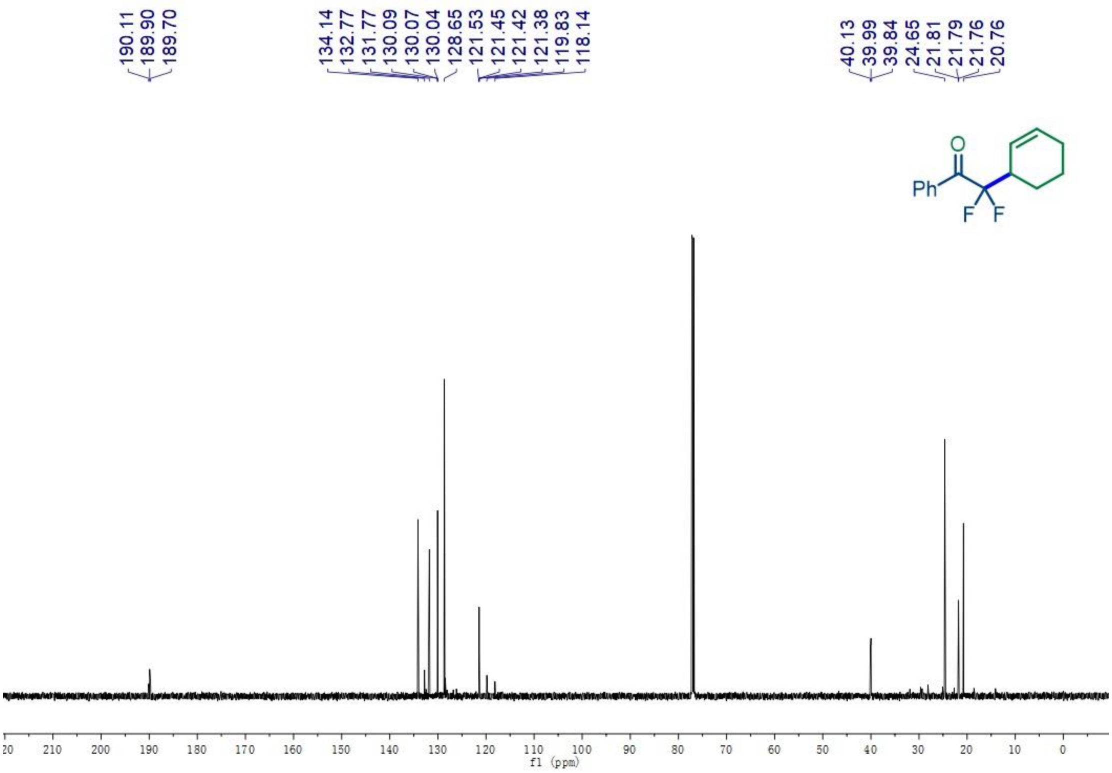

Supplementary Fig. 349 ^{13}C NMR (150 MHz, $CDCl_3$) spectrum of compound **116**

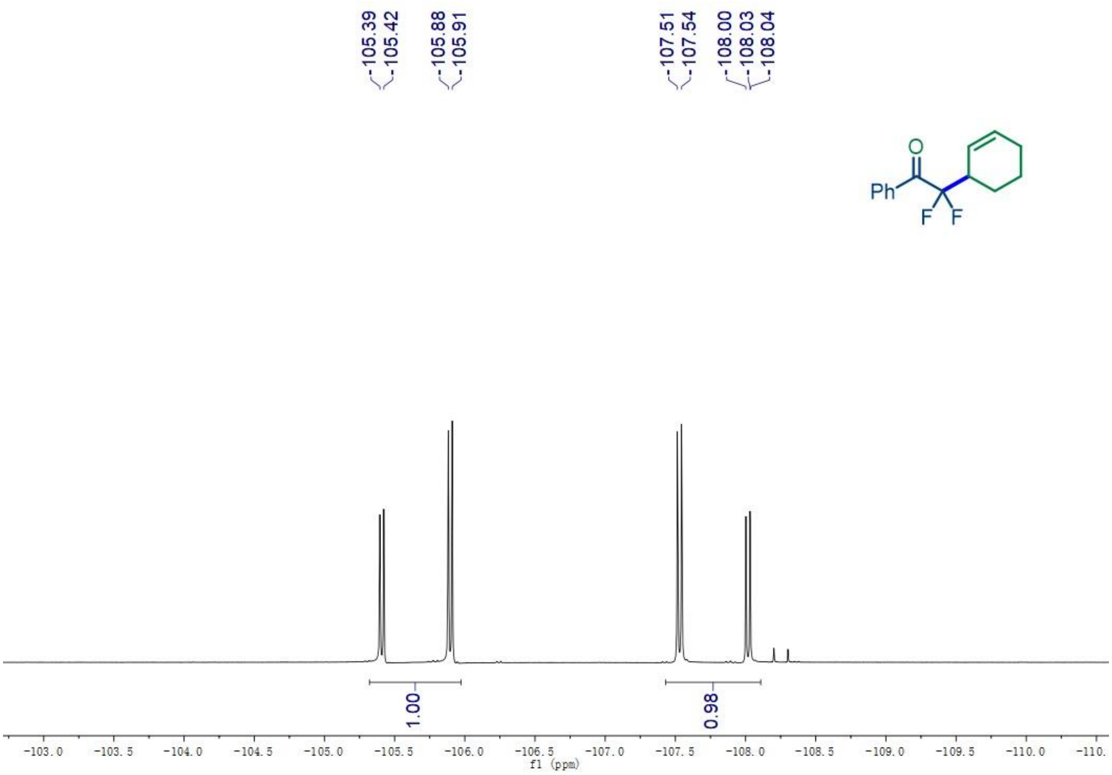

Supplementary Fig. 350 ^{19}F NMR (564 MHz, $CDCl_3$) spectrum of compound **116**

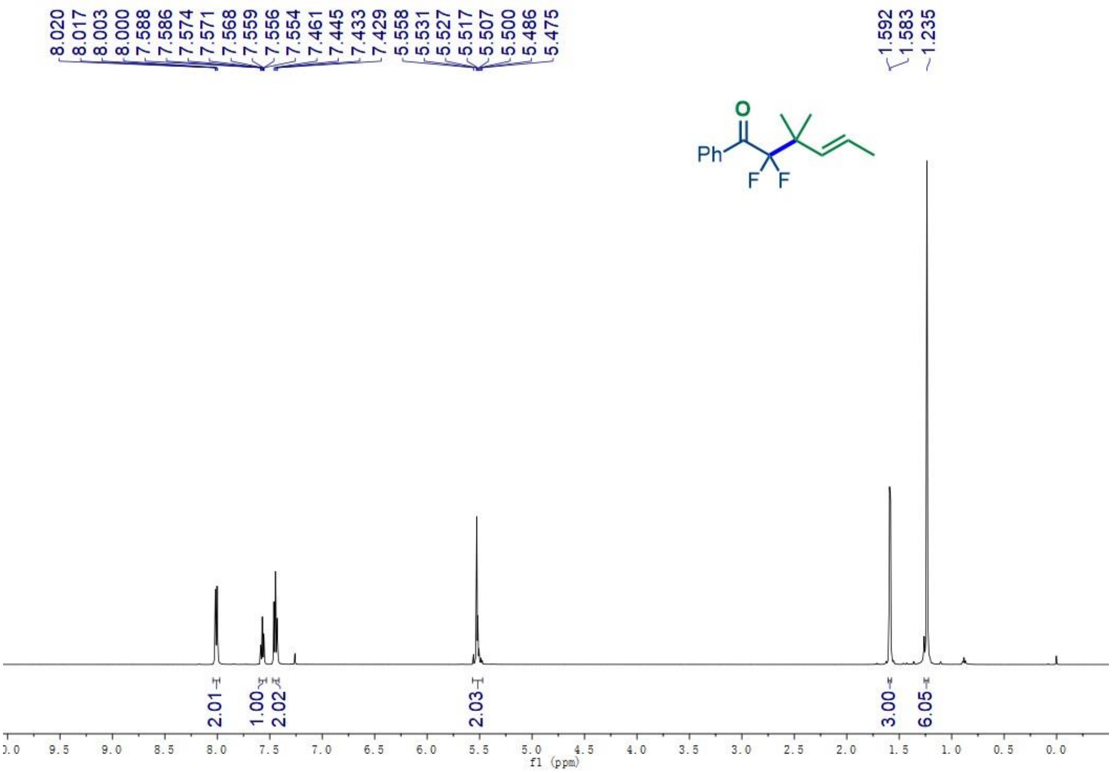

Supplementary Fig. 351 ^{1}H NMR (500 MHz, $CDCl_3$) spectrum of compound **117**

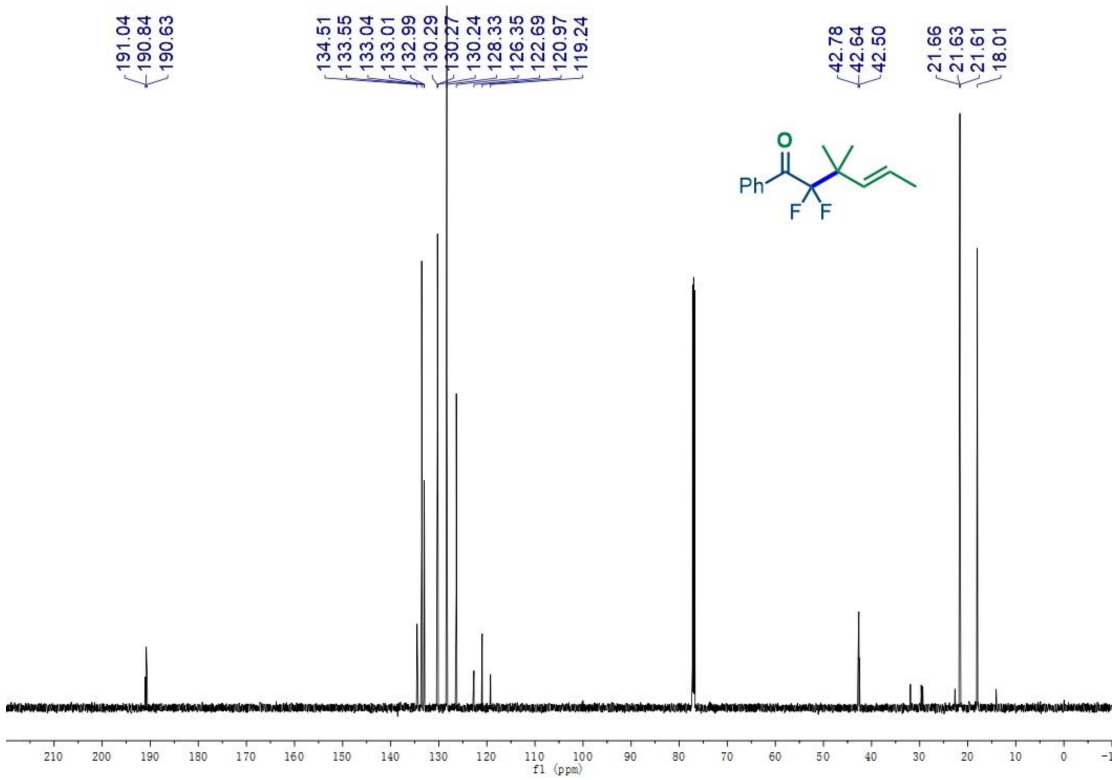

Supplementary Fig. 352 ^{13}C NMR (150 MHz, $CDCl_3$) spectrum of compound **117**

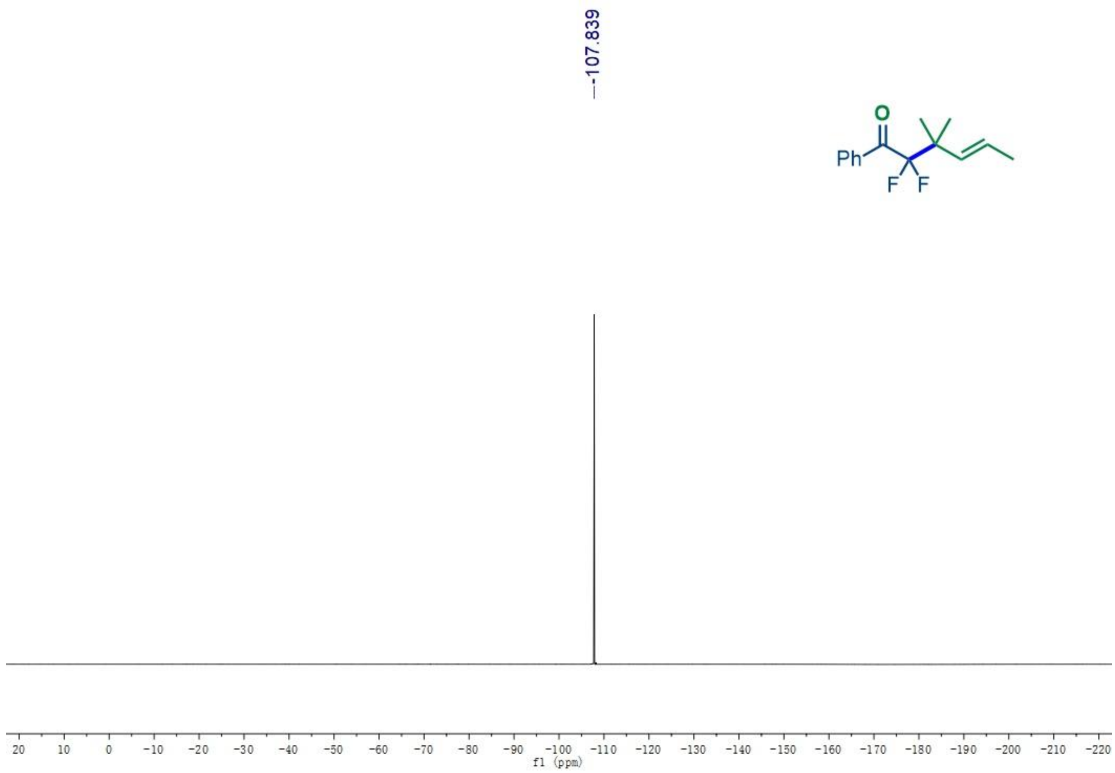

Supplementary Fig. 353 ^{19}F NMR (564 MHz, $CDCl_3$) spectrum of compound **117**

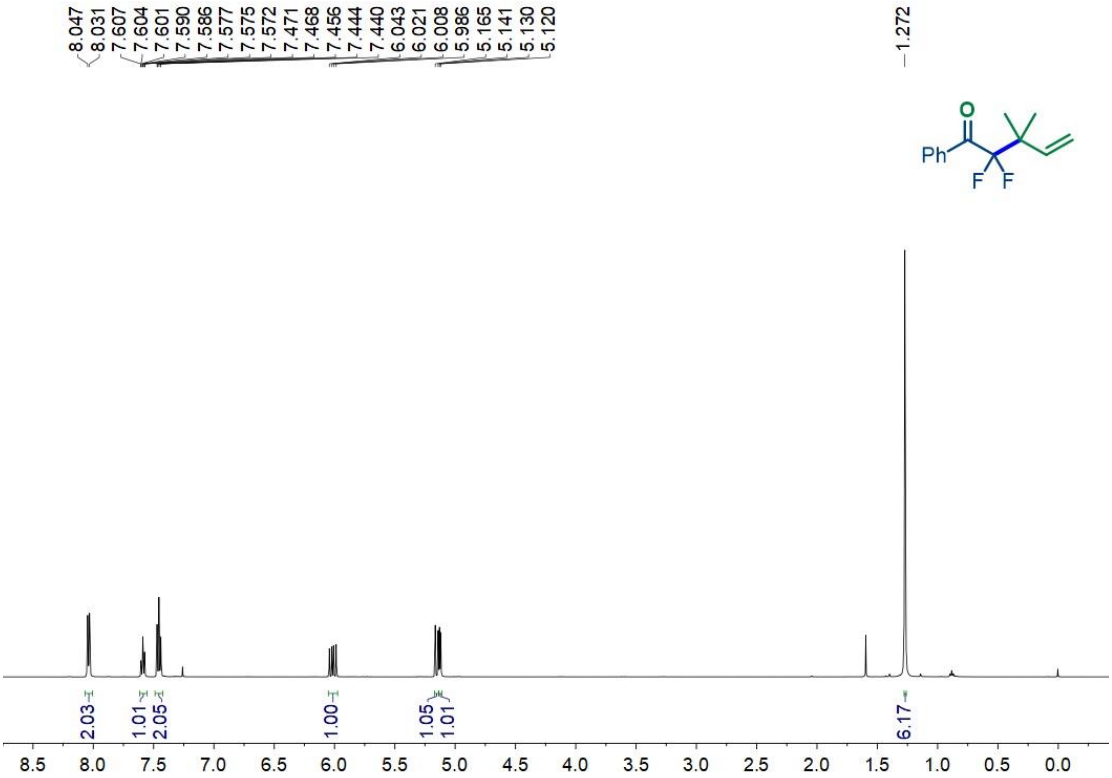

Supplementary Fig. 354 ^{1}H NMR (500 MHz, $CDCl_3$) spectrum of compound **118**

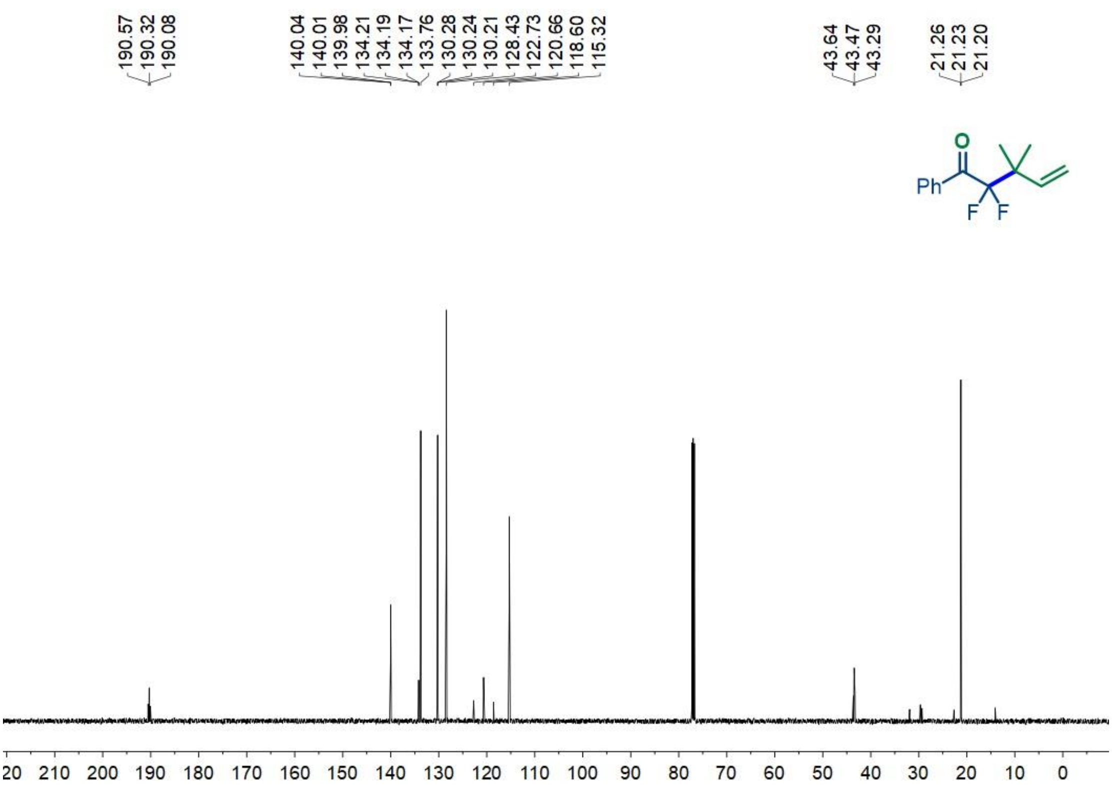

Supplementary Fig. 355 ^{13}C NMR (125 MHz, $CDCl_3$) spectrum of compound **118**

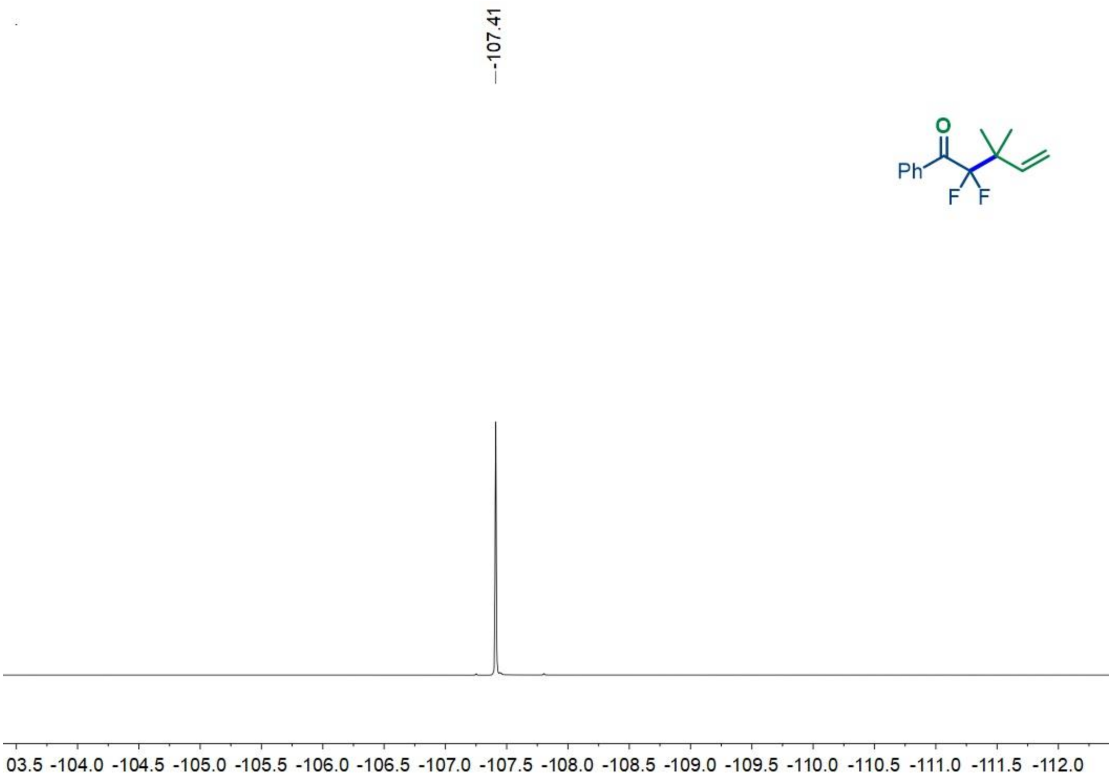

Supplementary Fig. 356 ^{19}F NMR (470 MHz, $CDCl_3$) spectrum of compound **118**

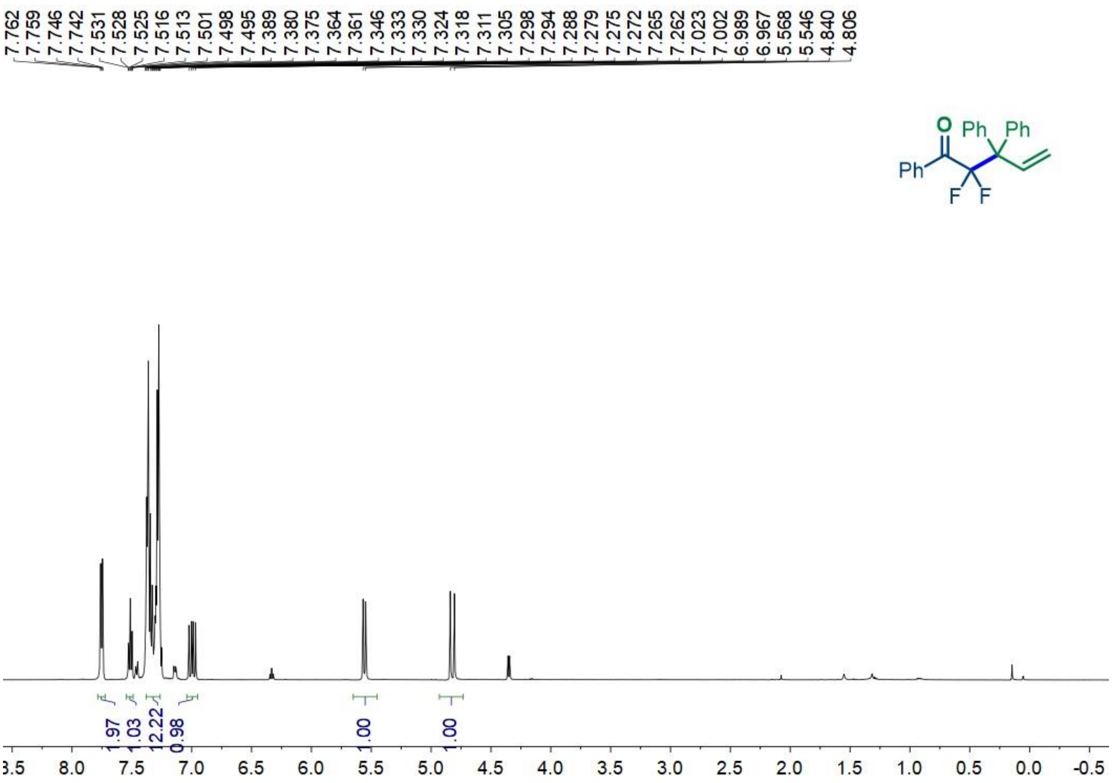

Supplementary Fig. 357 ^{1}H NMR (500 MHz, $CDCl_3$) spectrum of compound **119**

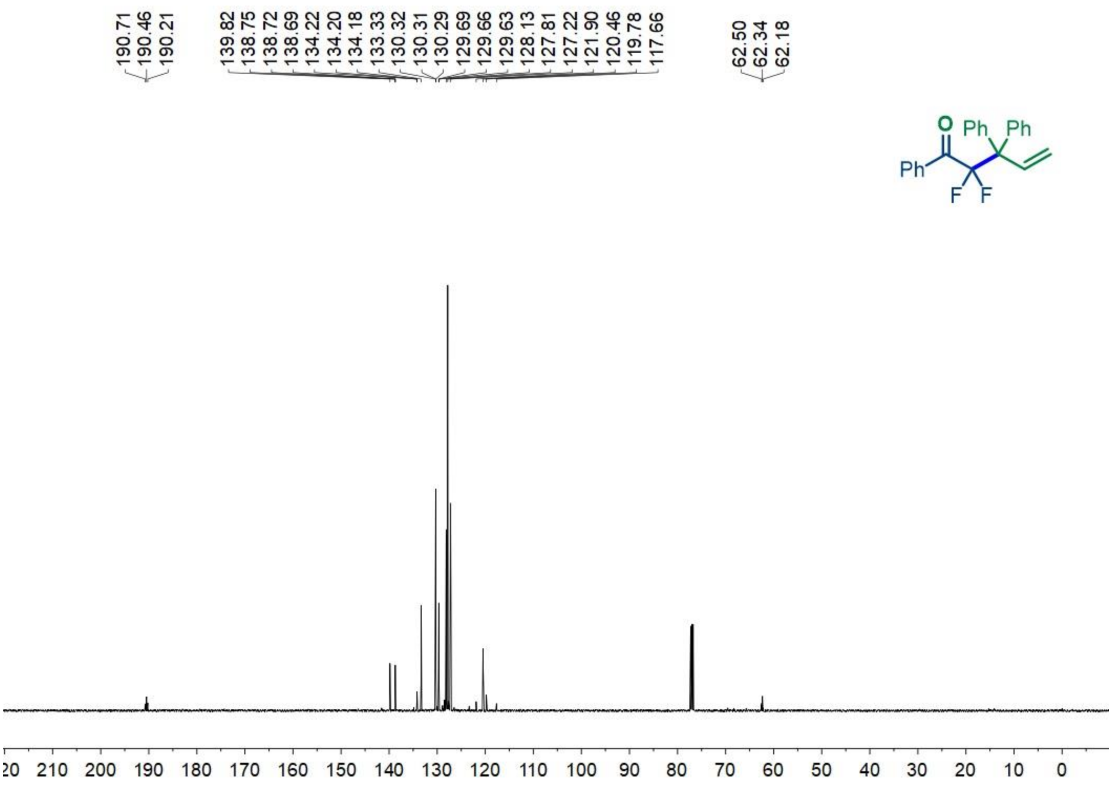

Supplementary Fig. 358 ^{13}C NMR (125 MHz, $CDCl_3$) spectrum of compound **119**

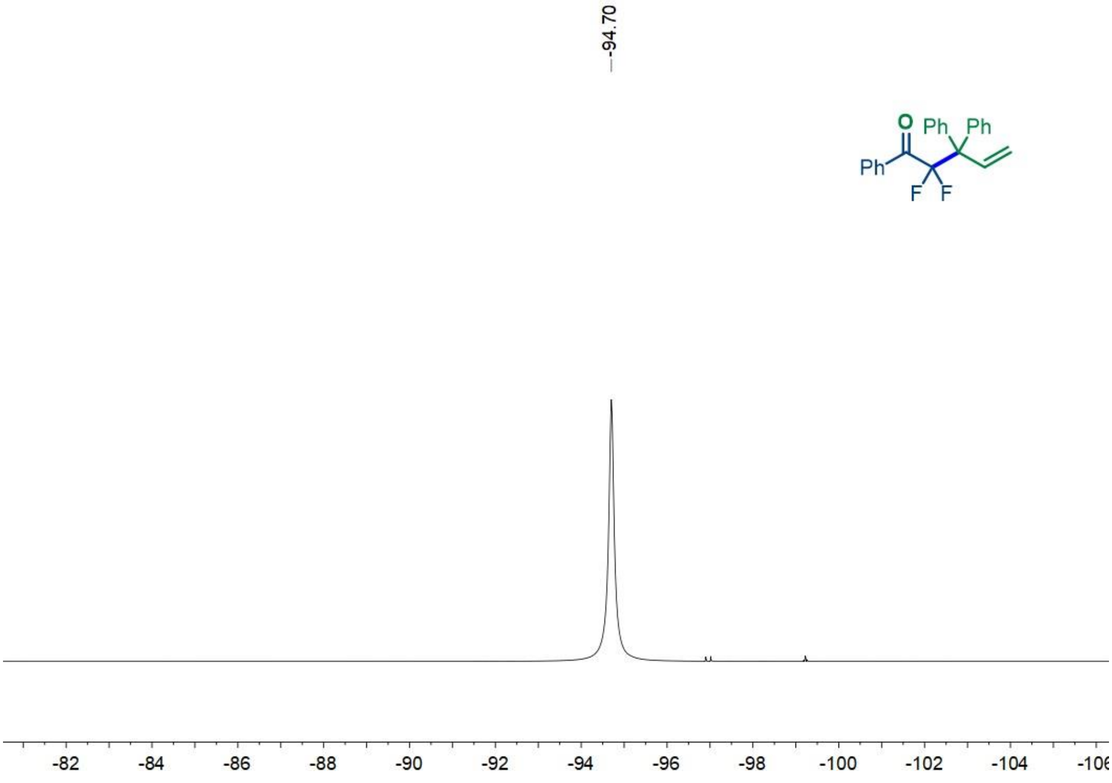

Supplementary Fig. 359 ^{19}F NMR (470 MHz, $CDCl_3$) spectrum of compound **119**

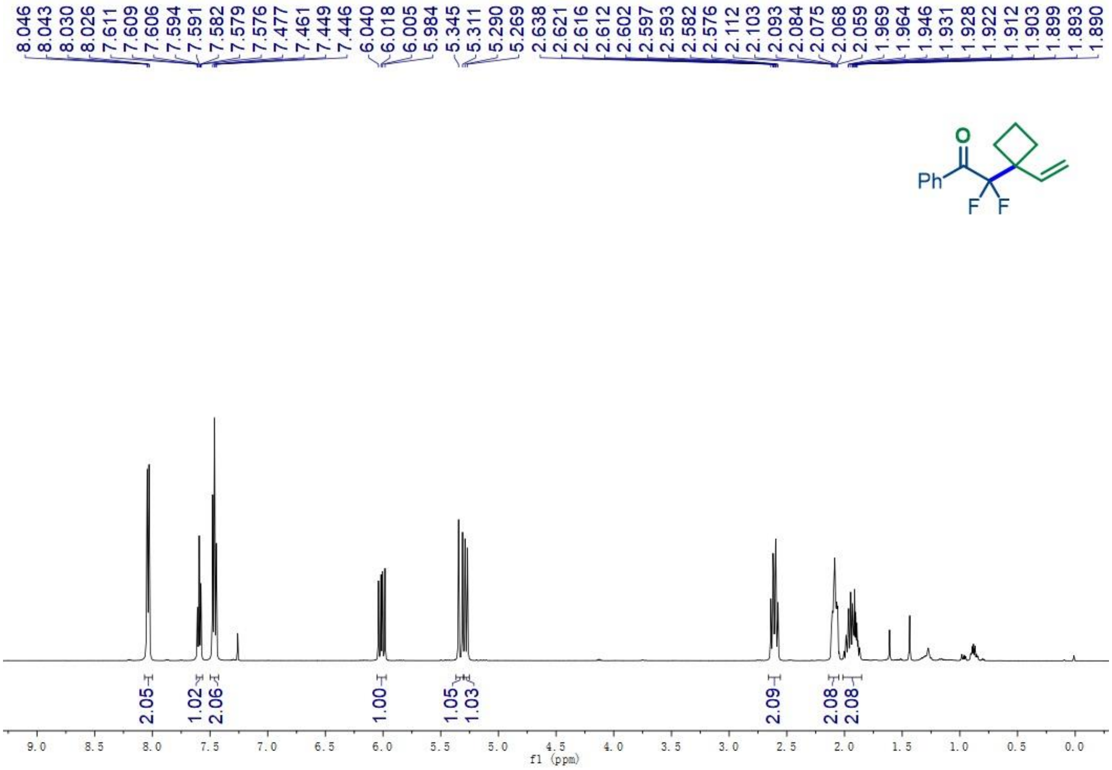

Supplementary Fig. 360 ^{1}H NMR (500 MHz, $CDCl_3$) spectrum of compound **120**

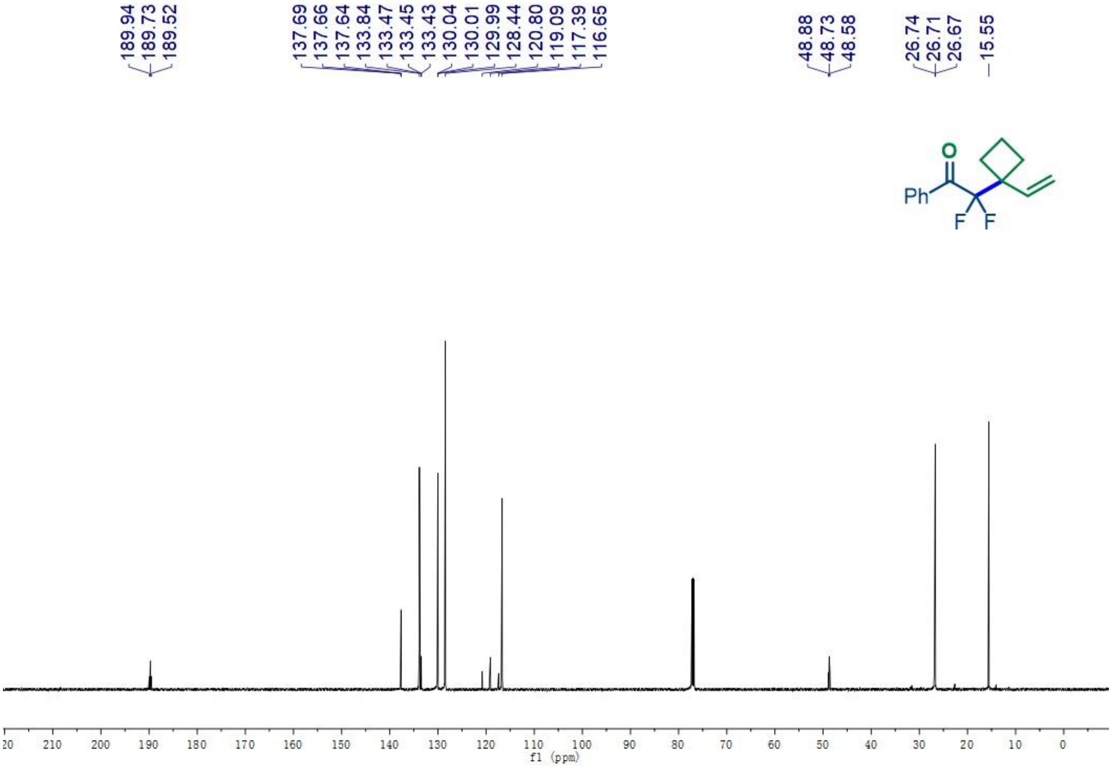

Supplementary Fig. 361 ^{13}C NMR (150 MHz, $CDCl_3$) spectrum of compound **120**

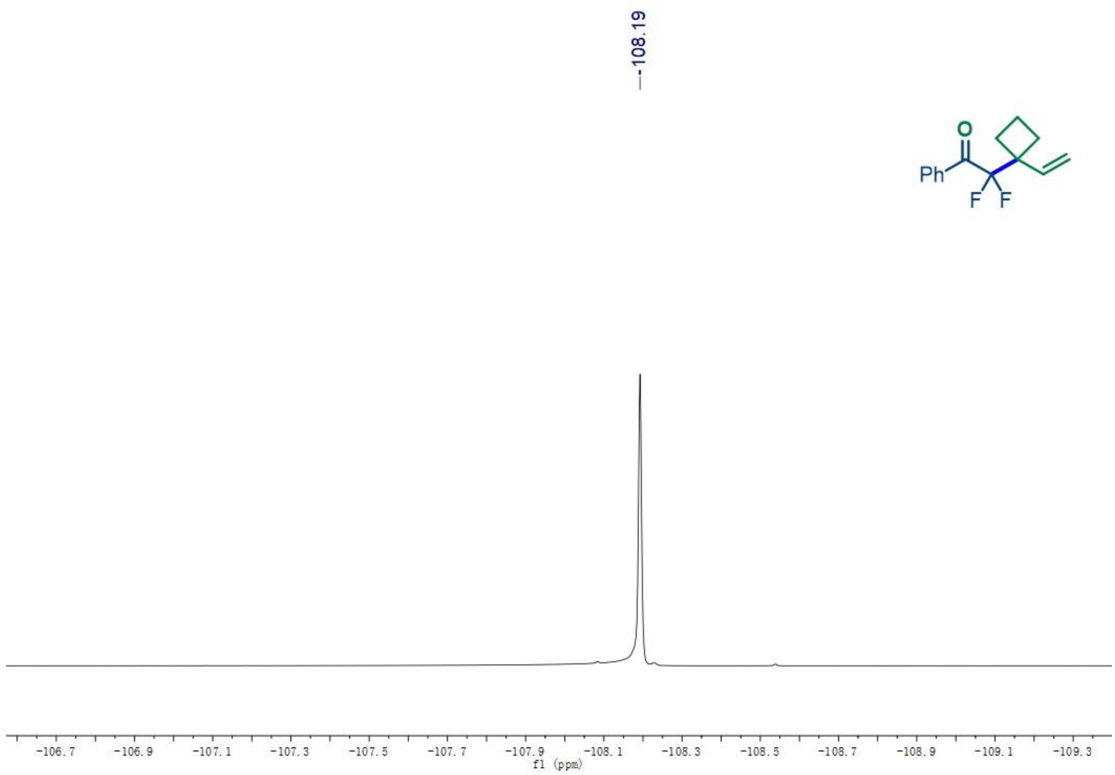

Supplementary Fig. 362 ^{19}F NMR (564 MHz, $CDCl_3$) spectrum of compound **120**

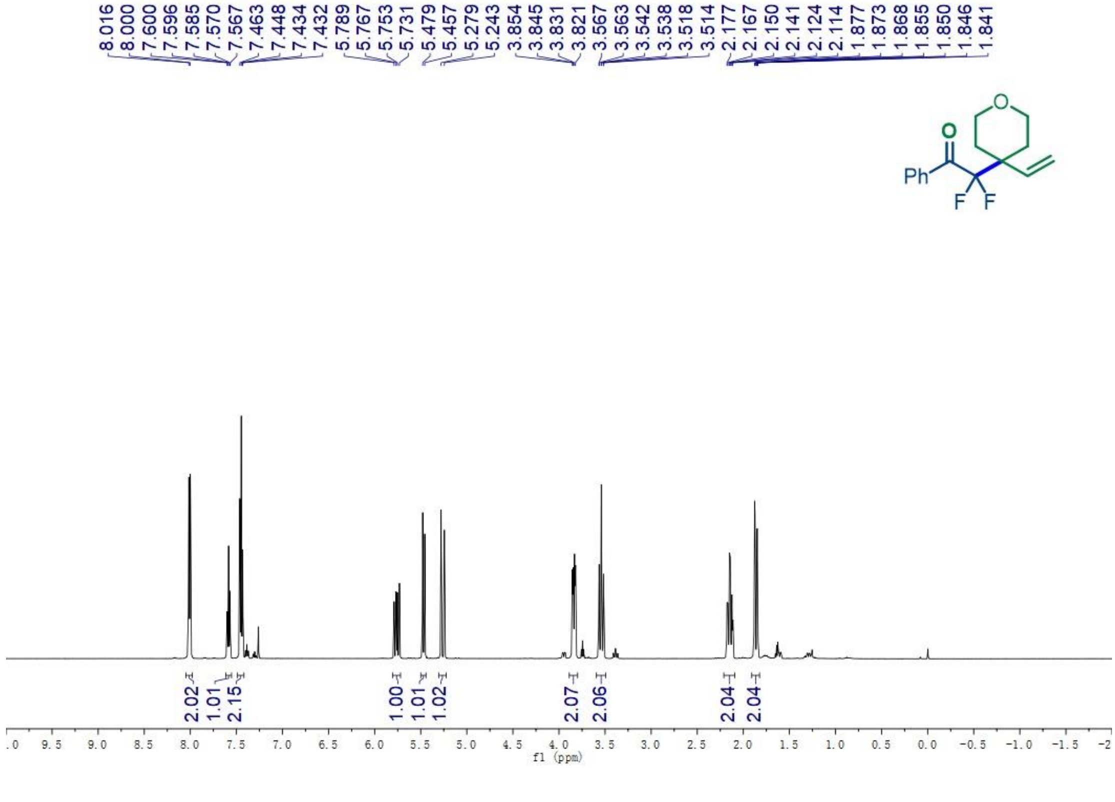

Supplementary Fig. 363 ^{1}H NMR (500 MHz, $CDCl_3$) spectrum of compound **121**

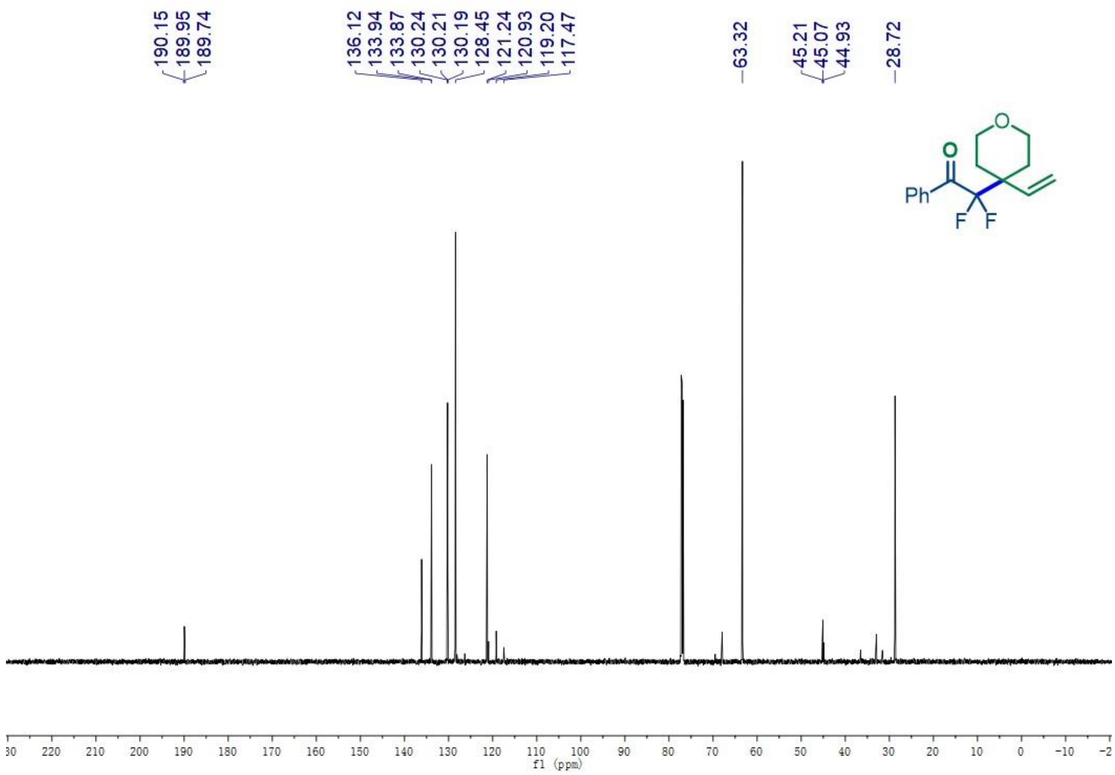

Supplementary Fig. 364 ^{13}C NMR (150 MHz, $CDCl_3$) spectrum of compound **121**

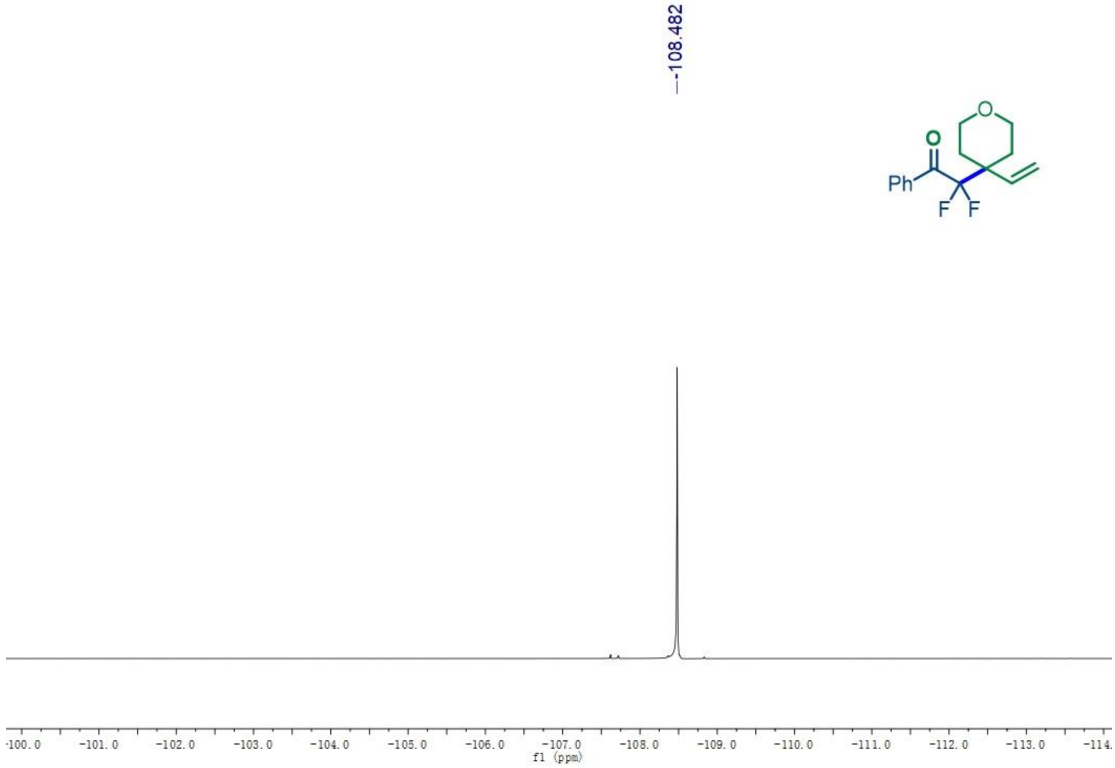

Supplementary Fig. 365 ^{19}F NMR (564 MHz, $CDCl_3$) spectrum of compound **121**

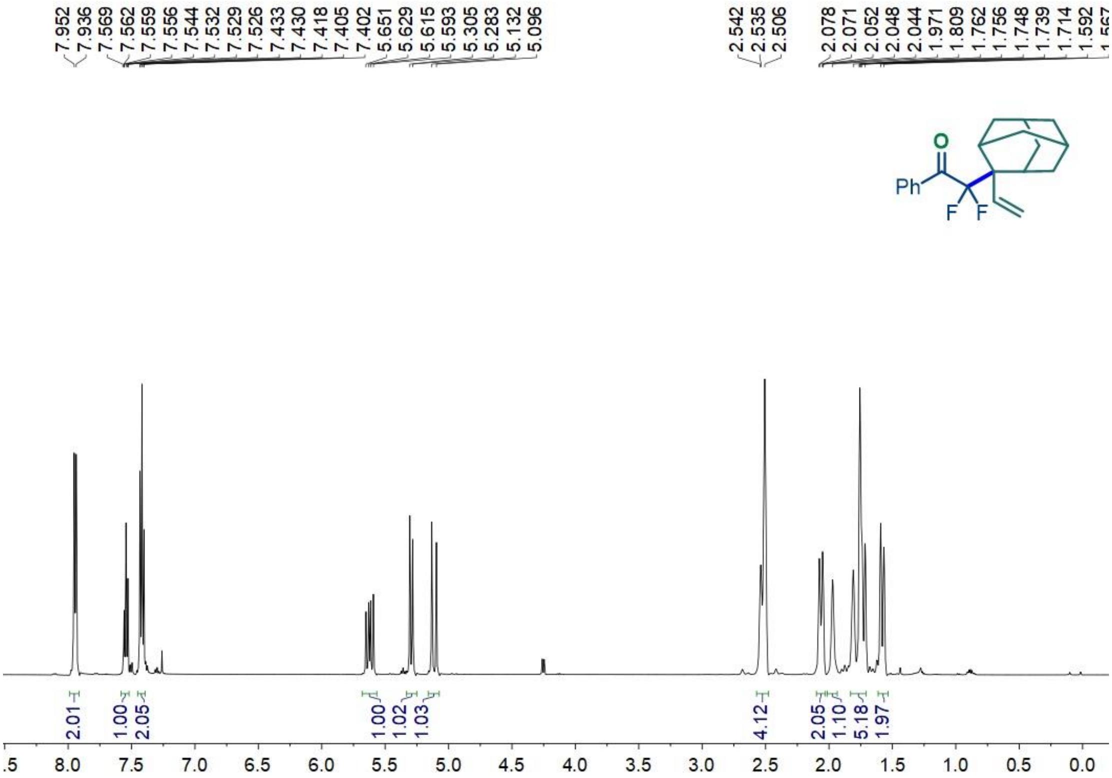

Supplementary Fig. 366 ^{1}H NMR (500 MHz, $CDCl_3$) spectrum of compound **122**

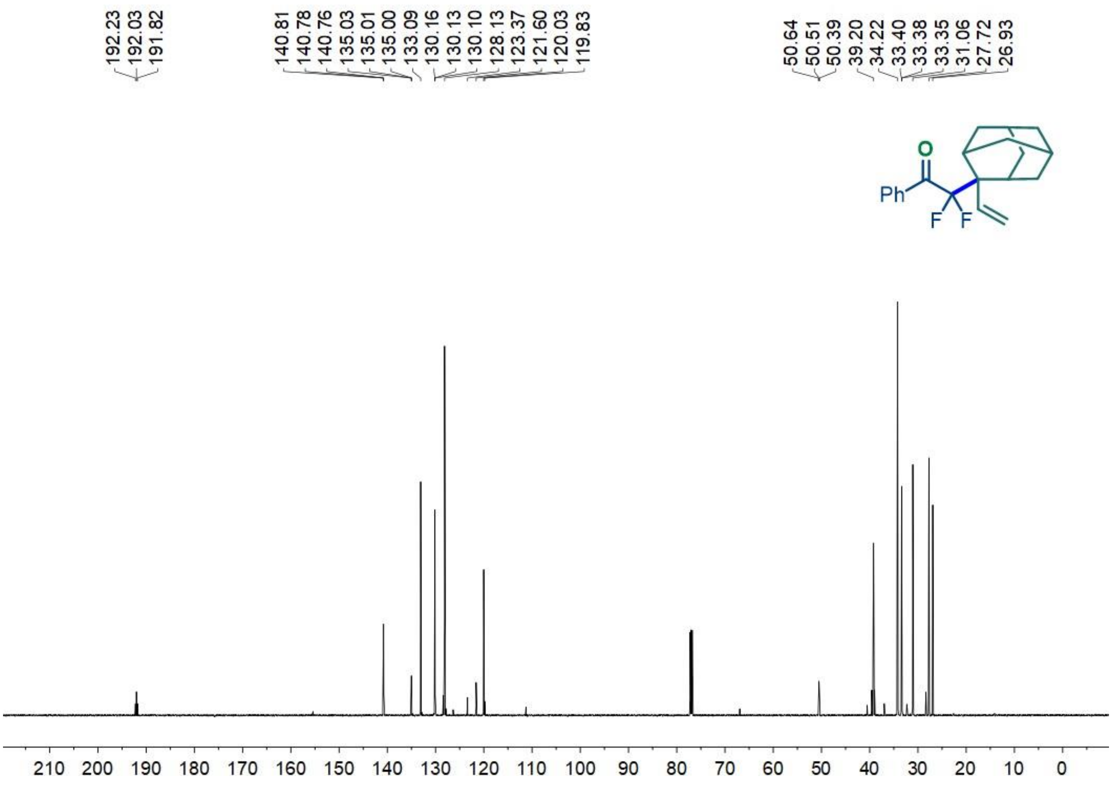

Supplementary Fig. 367 ^{13}C NMR (150 MHz, $CDCl_3$) spectrum of compound **122**

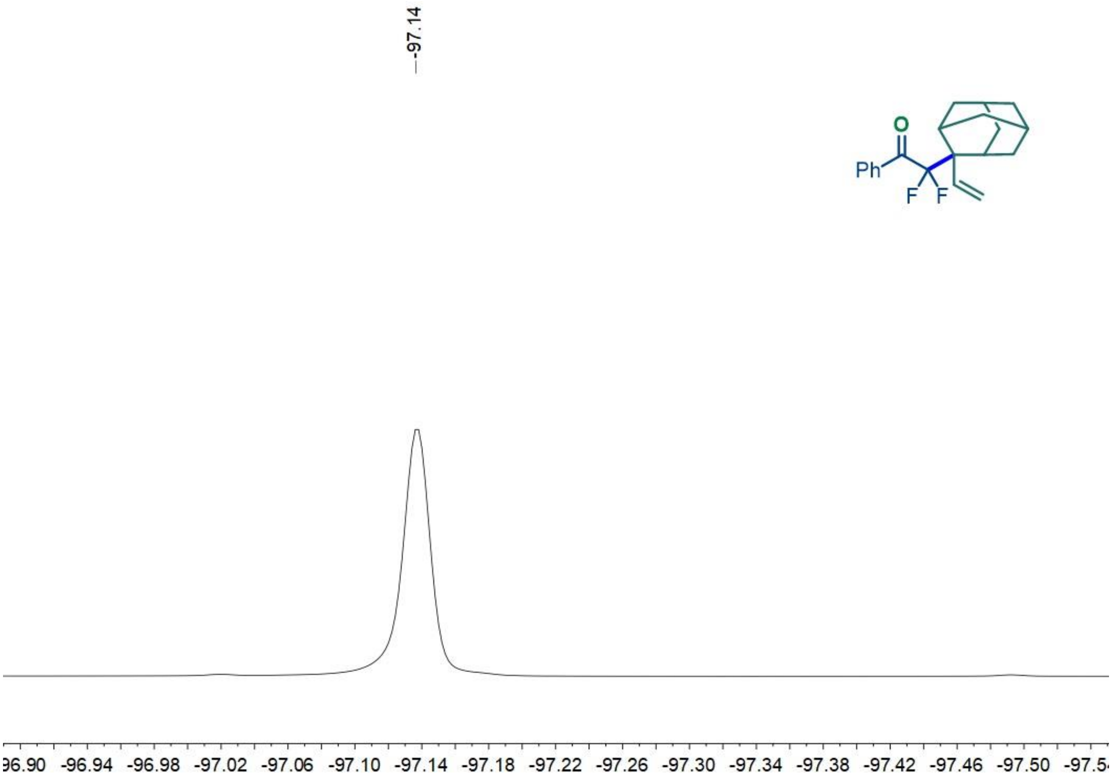

Supplementary Fig. 368 ^{19}F NMR (564 MHz, $CDCl_3$) spectrum of compound **122**

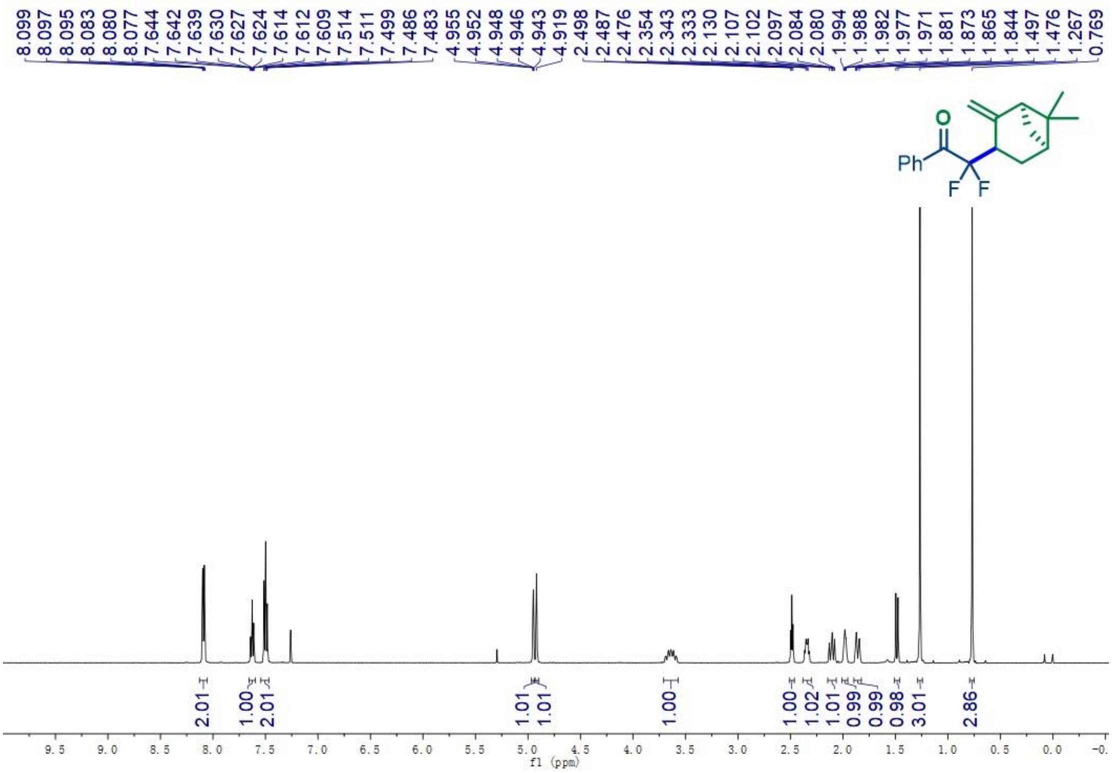

Supplementary Fig. 369 ^{1}H NMR (500 MHz, $CDCl_3$) spectrum of compound **123**

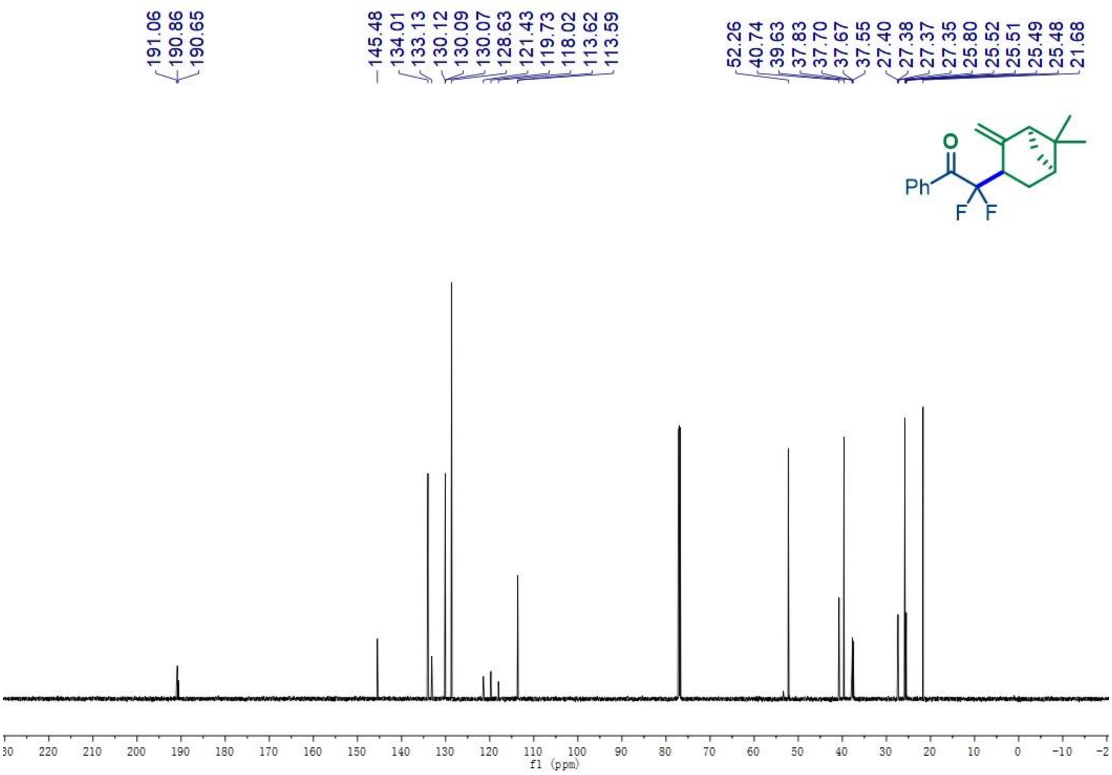

Supplementary Fig. 370 ^{13}C NMR (150 MHz, $CDCl_3$) spectrum of compound **123**

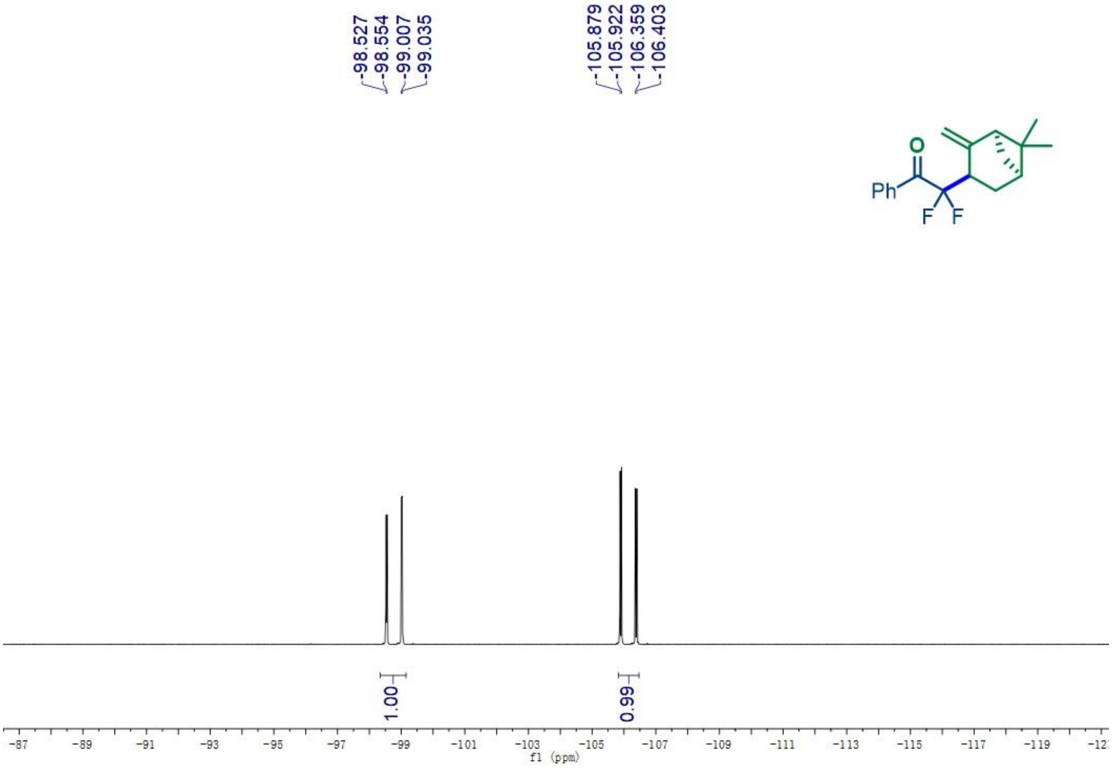

Supplementary Fig. 371 ^{19}F NMR (564 MHz, $CDCl_3$) spectrum of compound **123**

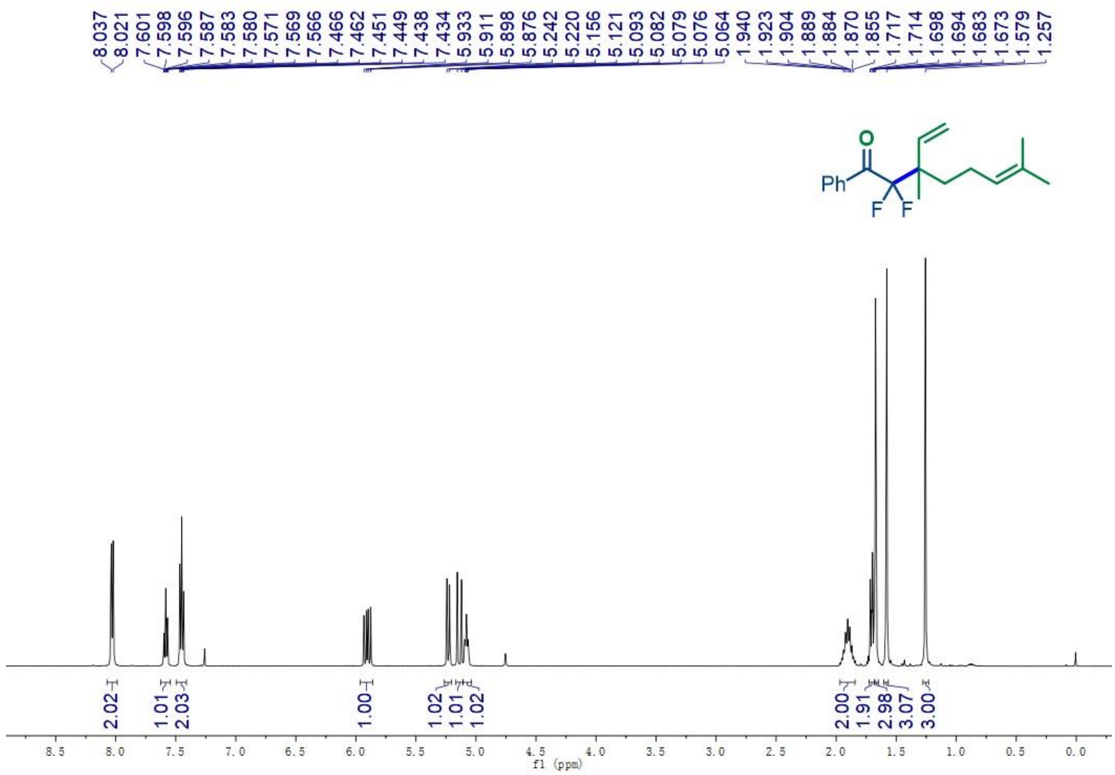

Supplementary Fig. 372 ^{1}H NMR (500 MHz, $CDCl_3$) spectrum of compound **124**

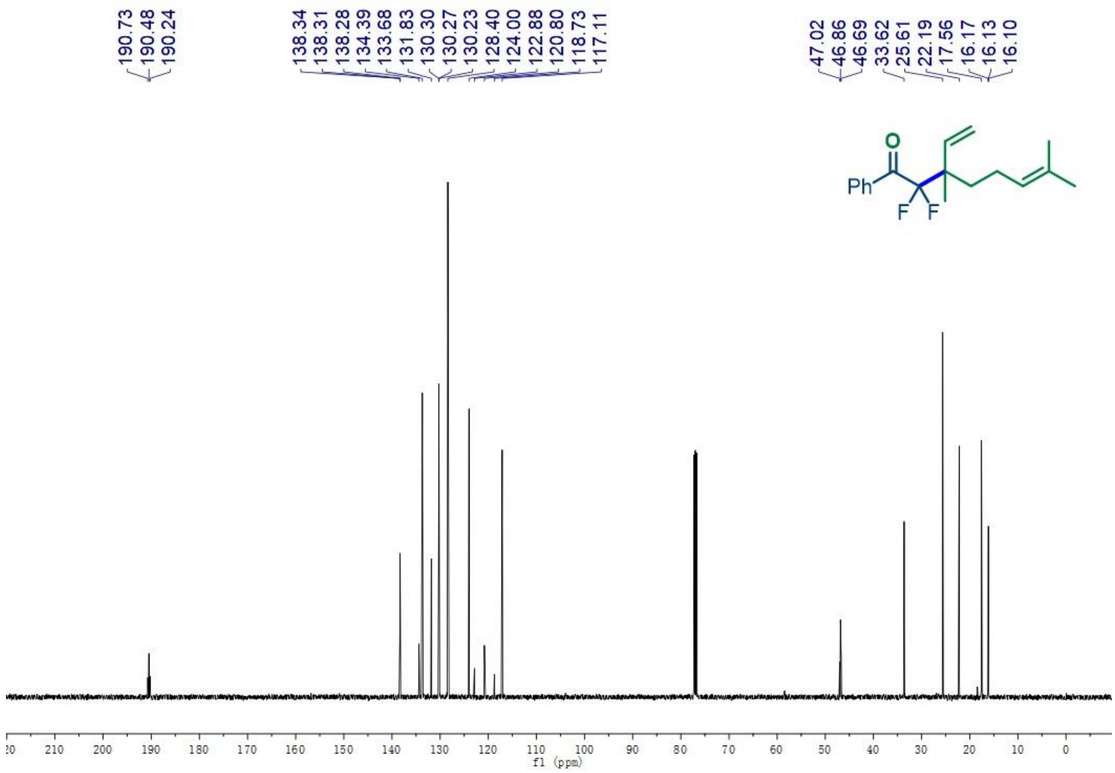

Supplementary Fig. 373 ^{13}C NMR (125 MHz, $CDCl_3$) spectrum of compound **124**

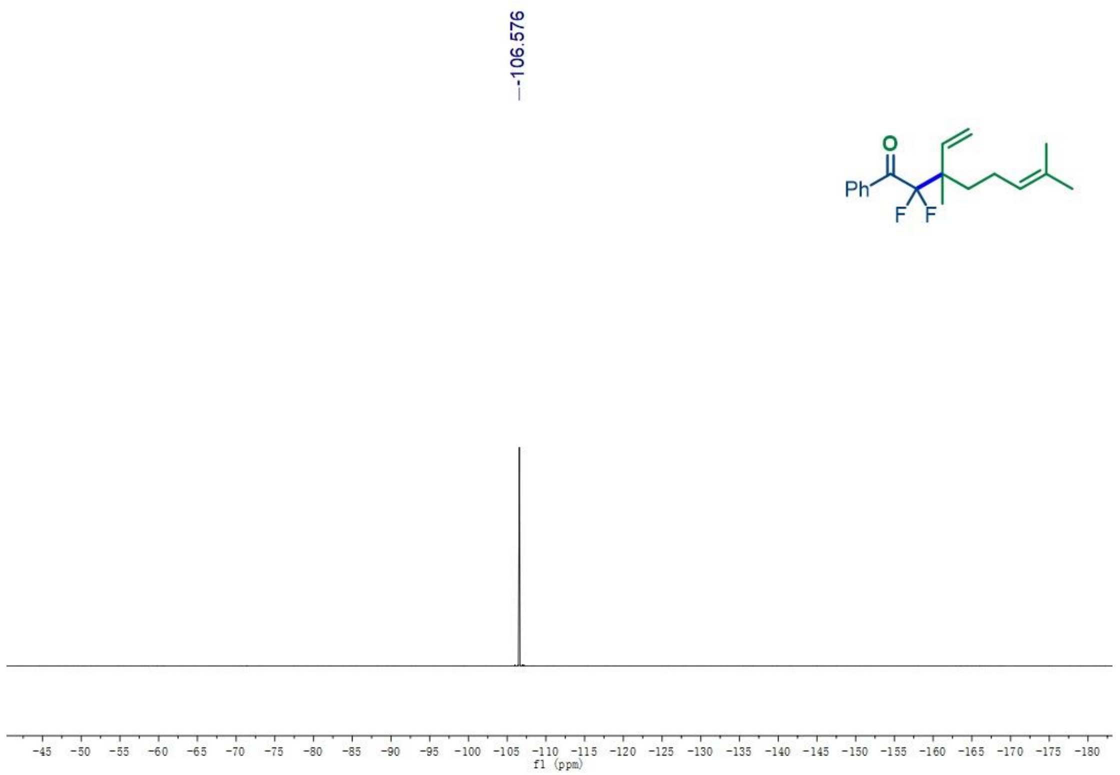

Supplementary Fig. 374 ^{19}F NMR (470 MHz, $CDCl_3$) spectrum of compound **124**

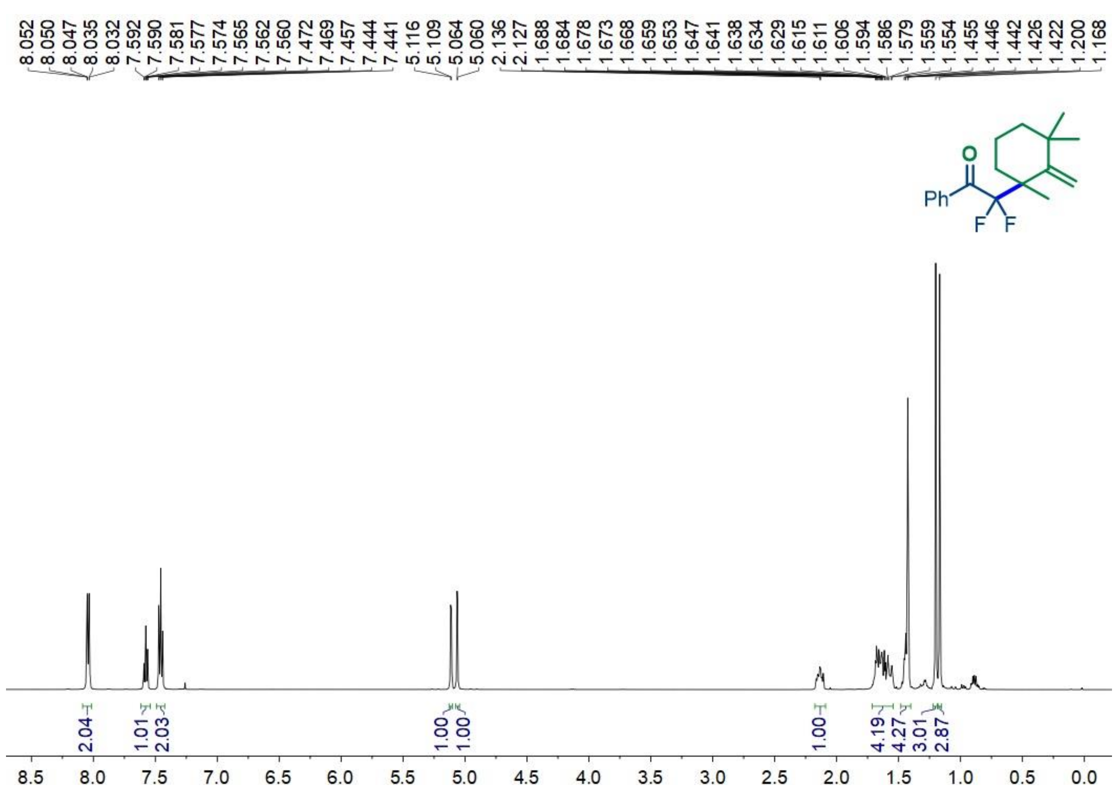

Supplementary Fig. 375 ^{1}H NMR (500 MHz, $CDCl_3$) spectrum of compound **125**

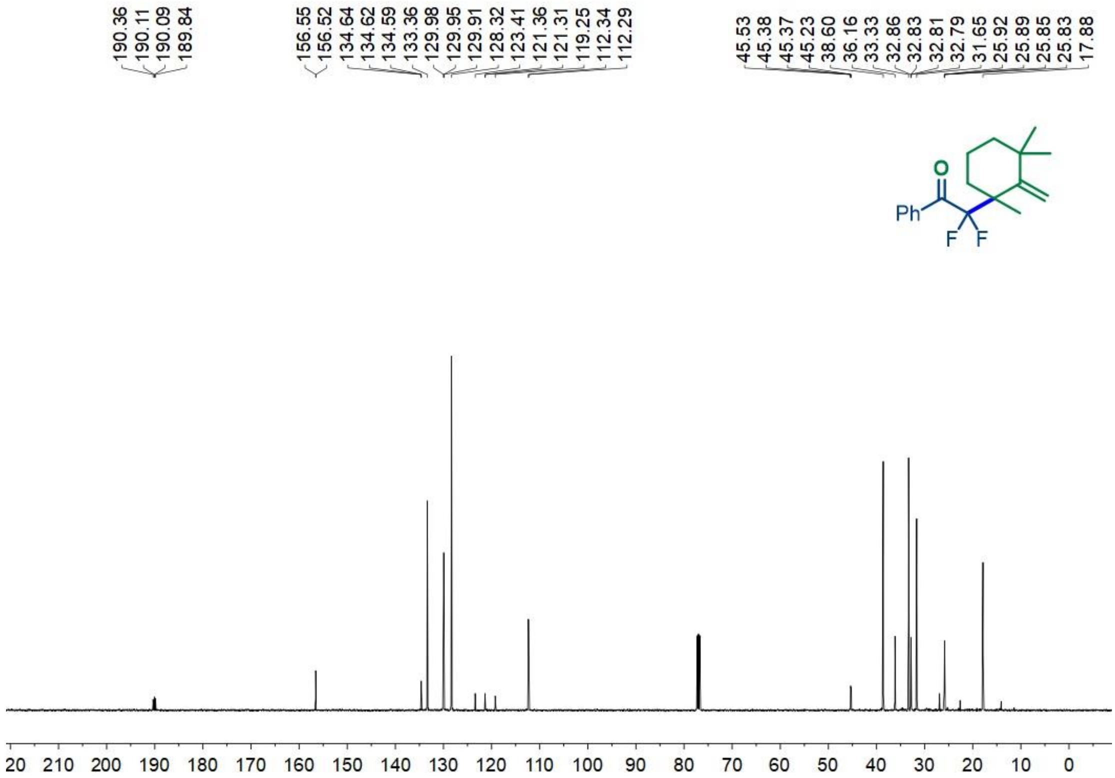

Supplementary Fig. 376 ^{13}C NMR (125 MHz, $CDCl_3$) spectrum of compound **125**

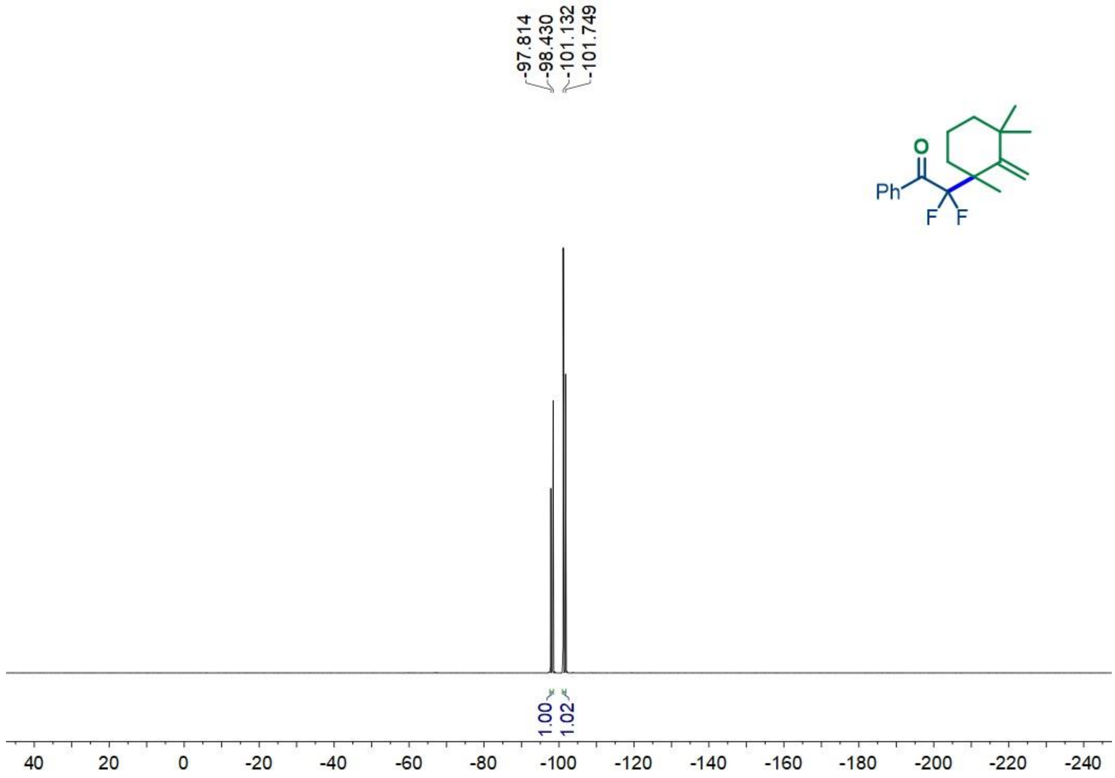

Supplementary Fig. 377 ^{19}F NMR (470 MHz, $CDCl_3$) spectrum of compound **125**

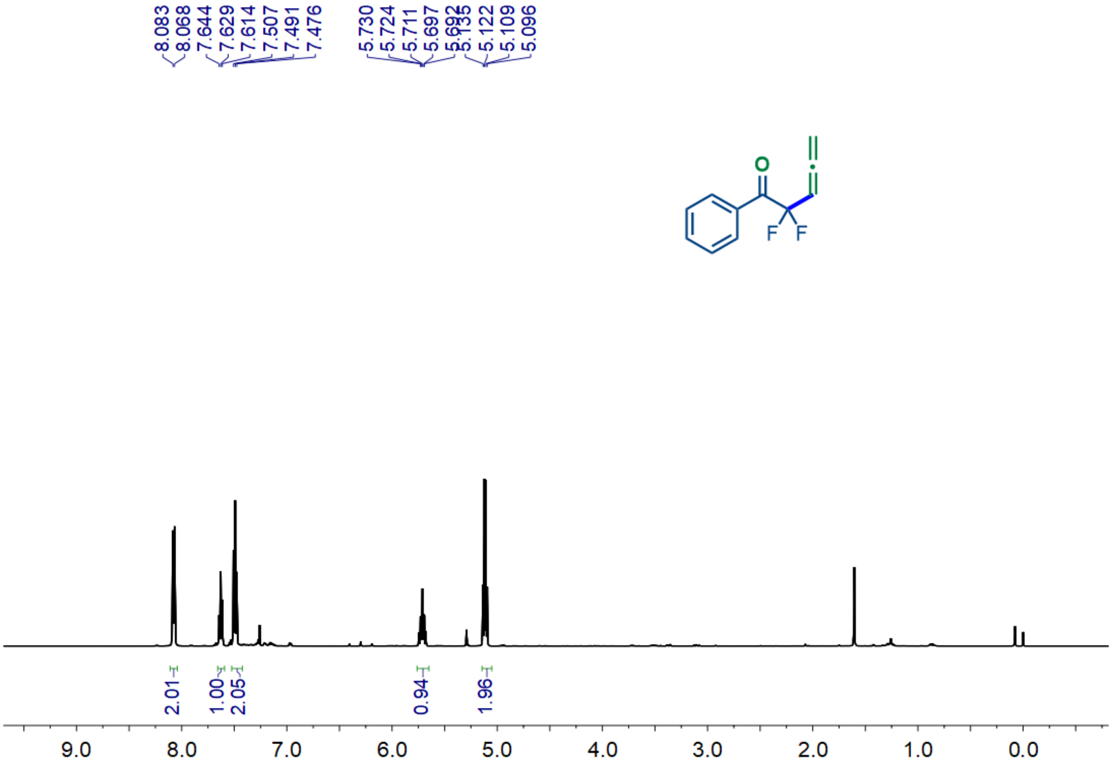

Supplementary Fig. 378 ^{1}H NMR (500 MHz, $CDCl_3$) spectrum of compound **126**

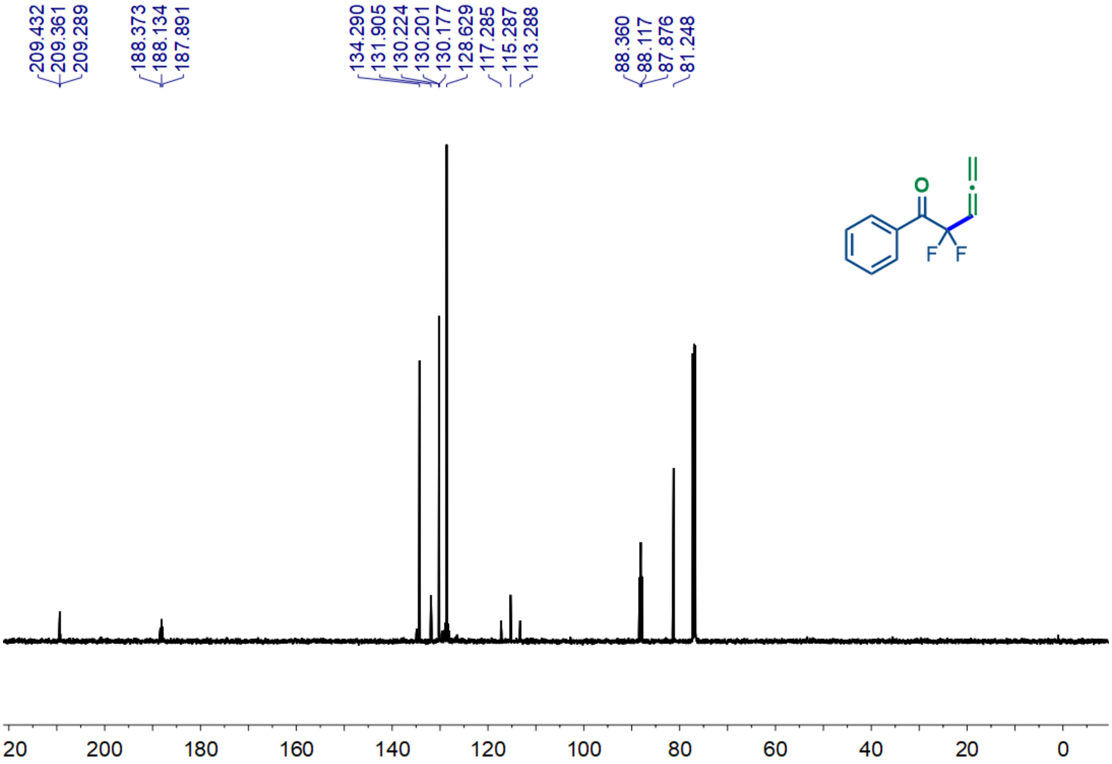

Supplementary Fig. 379 ^{13}C NMR (125 MHz, $CDCl_3$) spectrum of compound **126**

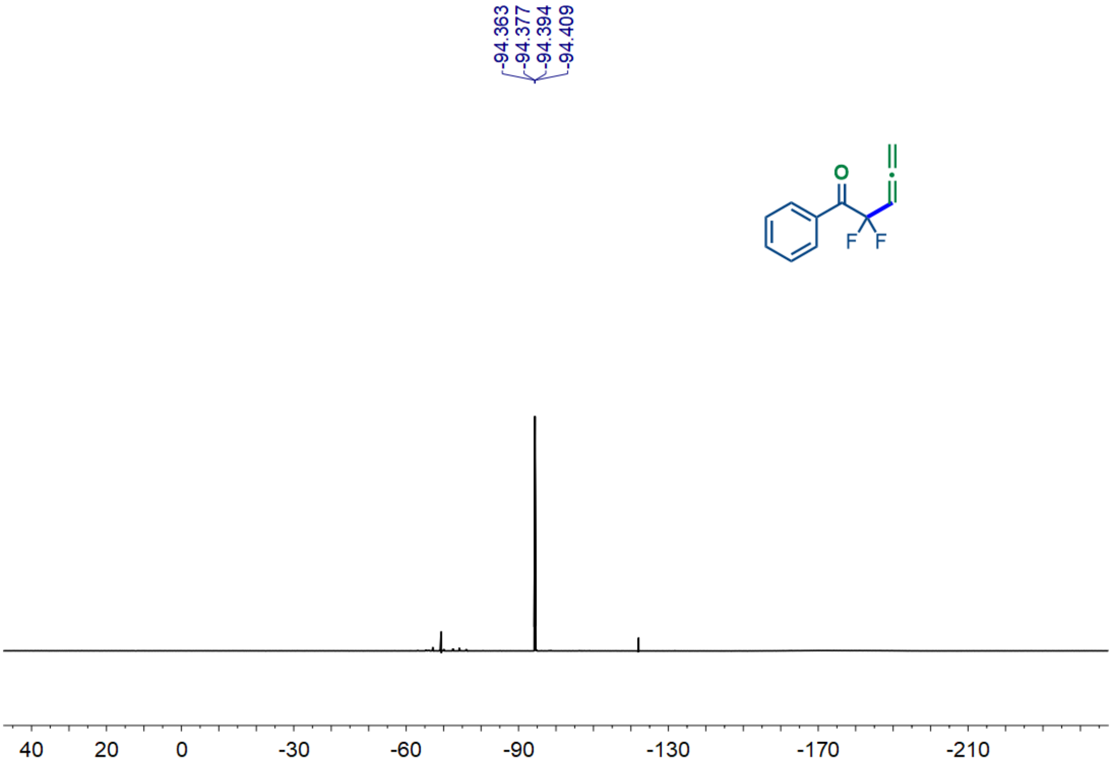

Supplementary Fig. 380 ^{19}F NMR (470 MHz, $CDCl_3$) spectrum of compound **126**

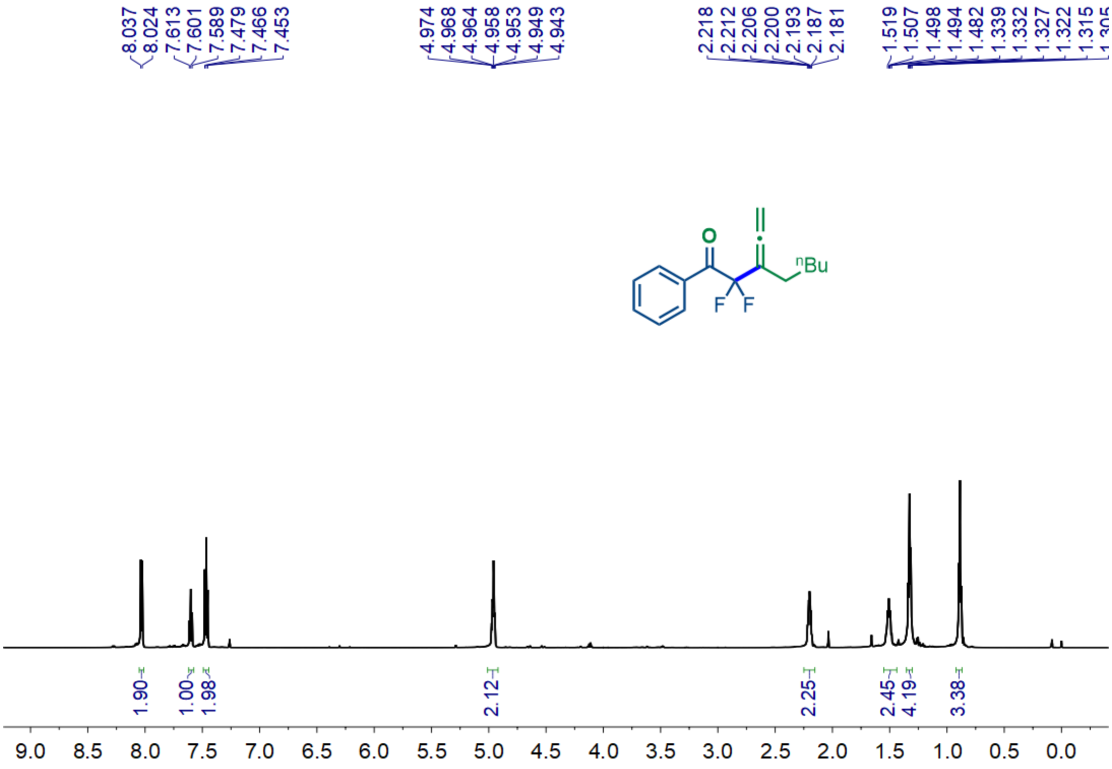

Supplementary Fig. 381 ^{1}H NMR (600 MHz, $CDCl_3$) spectrum of compound **127**

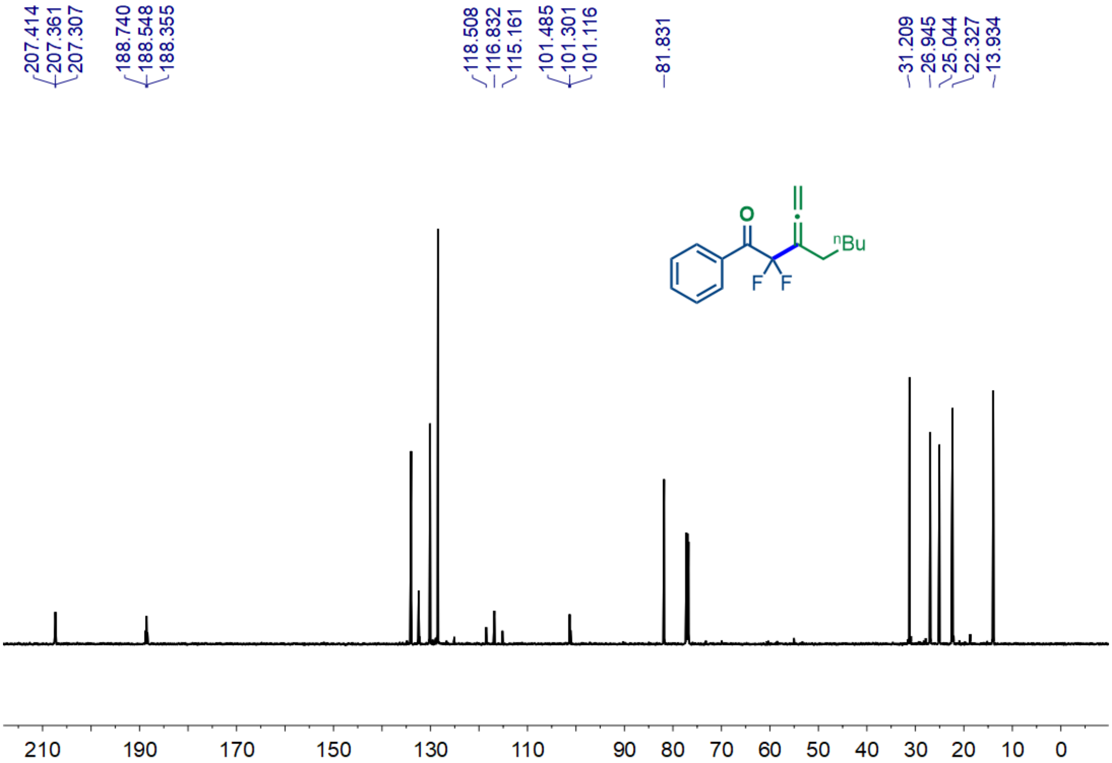

Supplementary Fig. 382 ^{13}C NMR (150 MHz, $CDCl_3$) spectrum of compound **127**

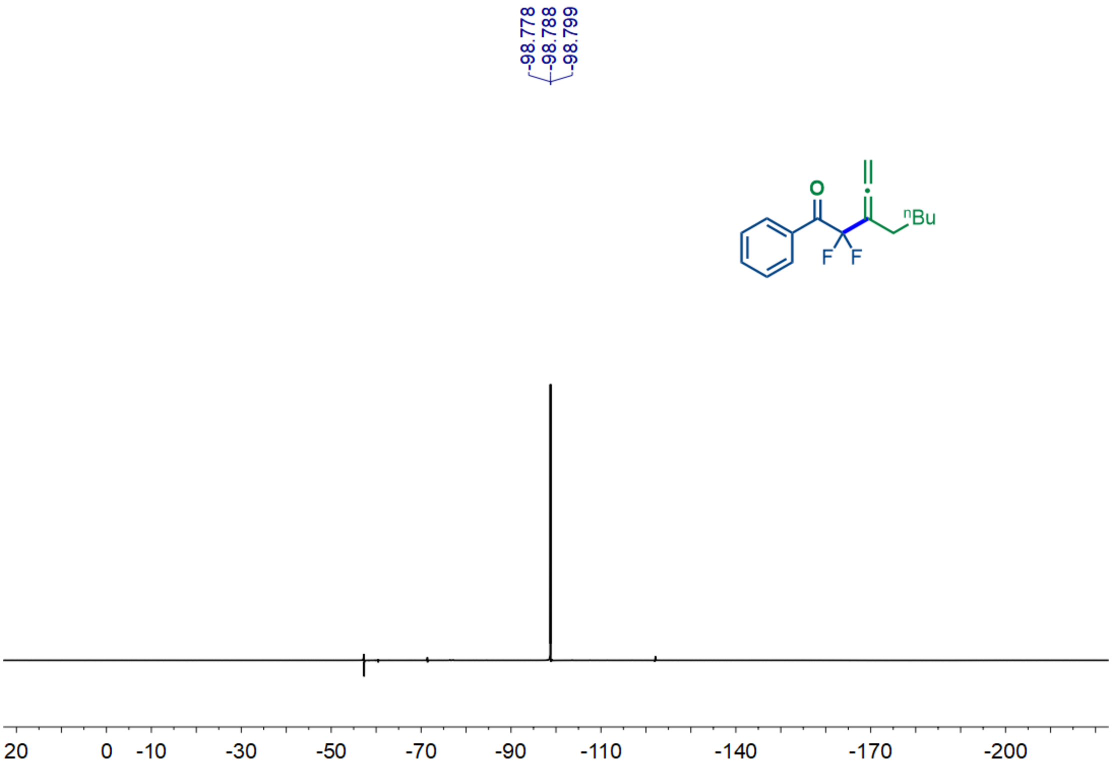

Supplementary Fig. 383 ^{19}F NMR (564 MHz, $CDCl_3$) spectrum of compound **127**

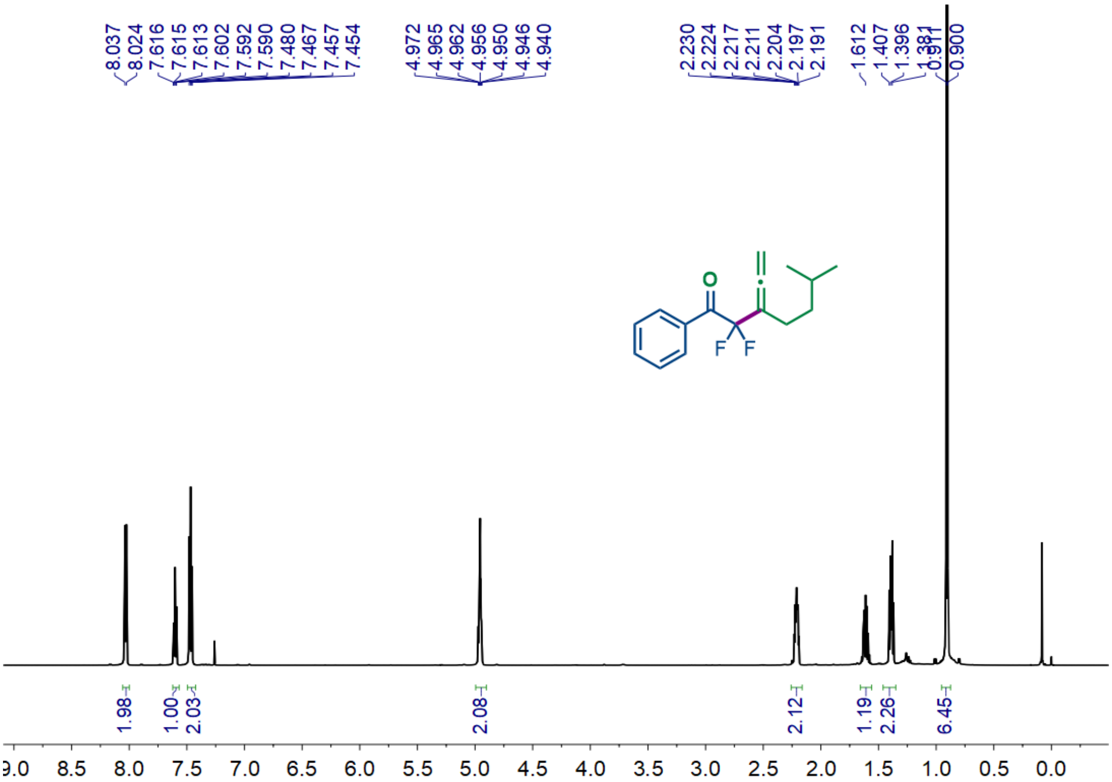

Supplementary Fig. 384 ^{1}H NMR (600 MHz, $CDCl_3$) spectrum of compound **128**

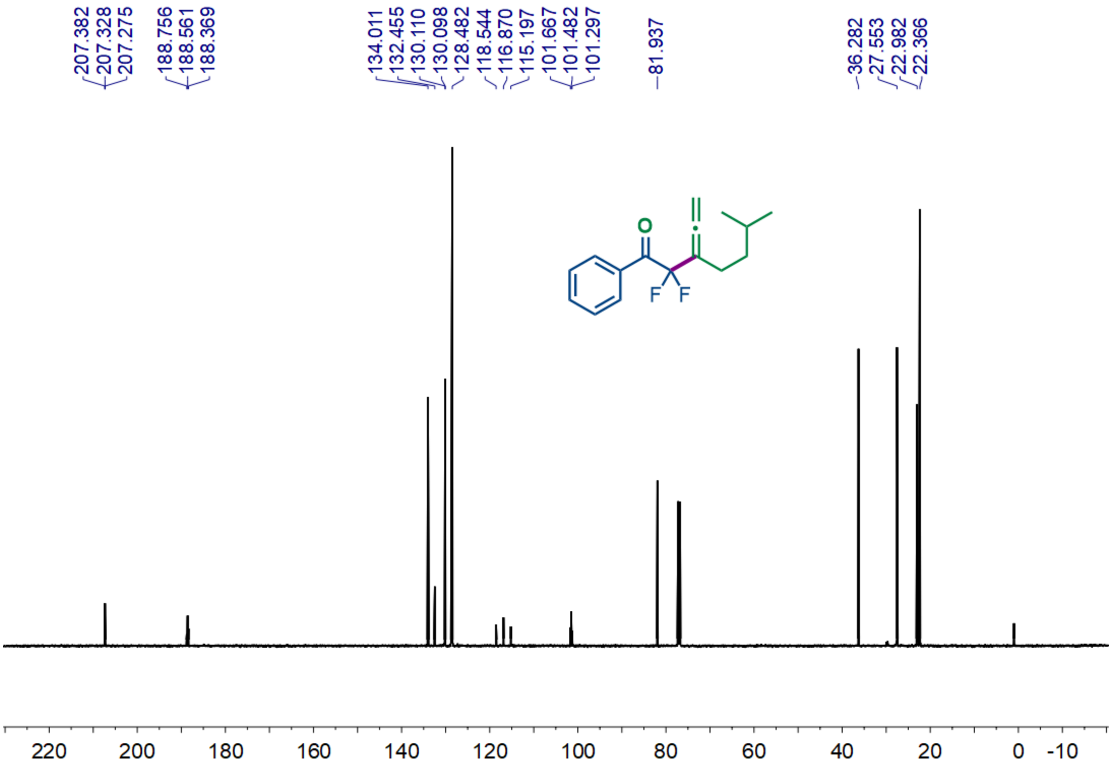

Supplementary Fig. 385 ^{13}C NMR (150 MHz, $CDCl_3$) spectrum of compound **128**

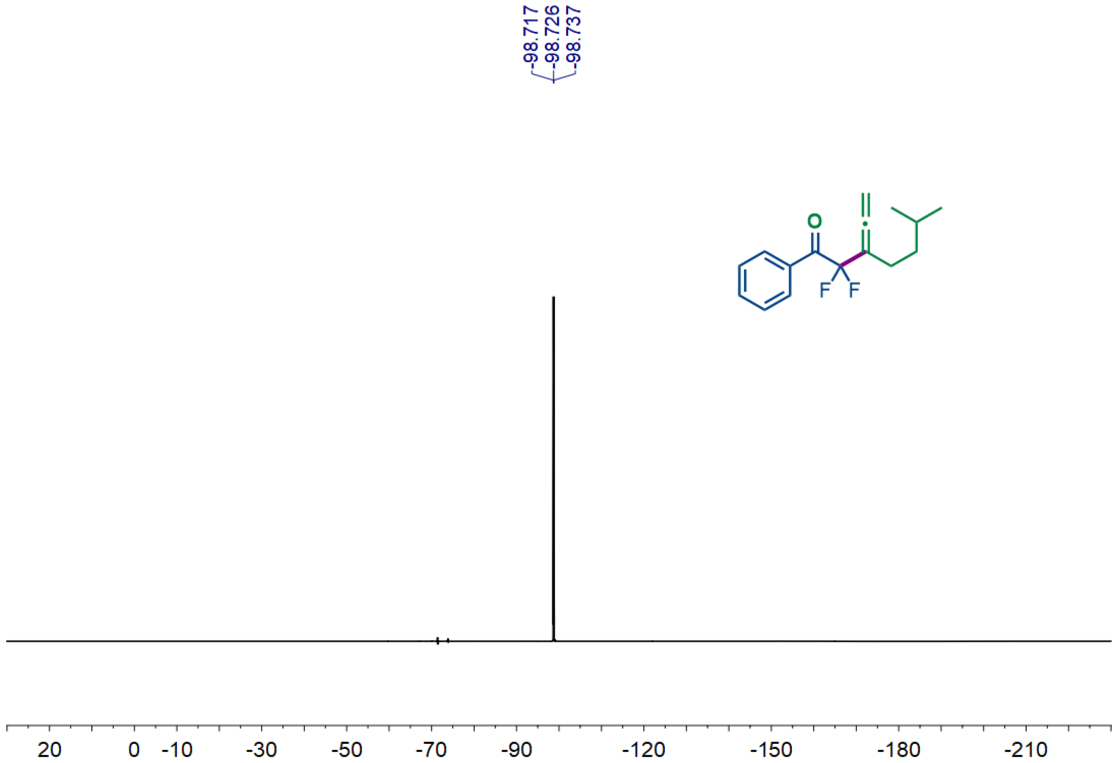

Supplementary Fig. 386 ^{19}F NMR (564 MHz, $CDCl_3$) spectrum of compound **128**

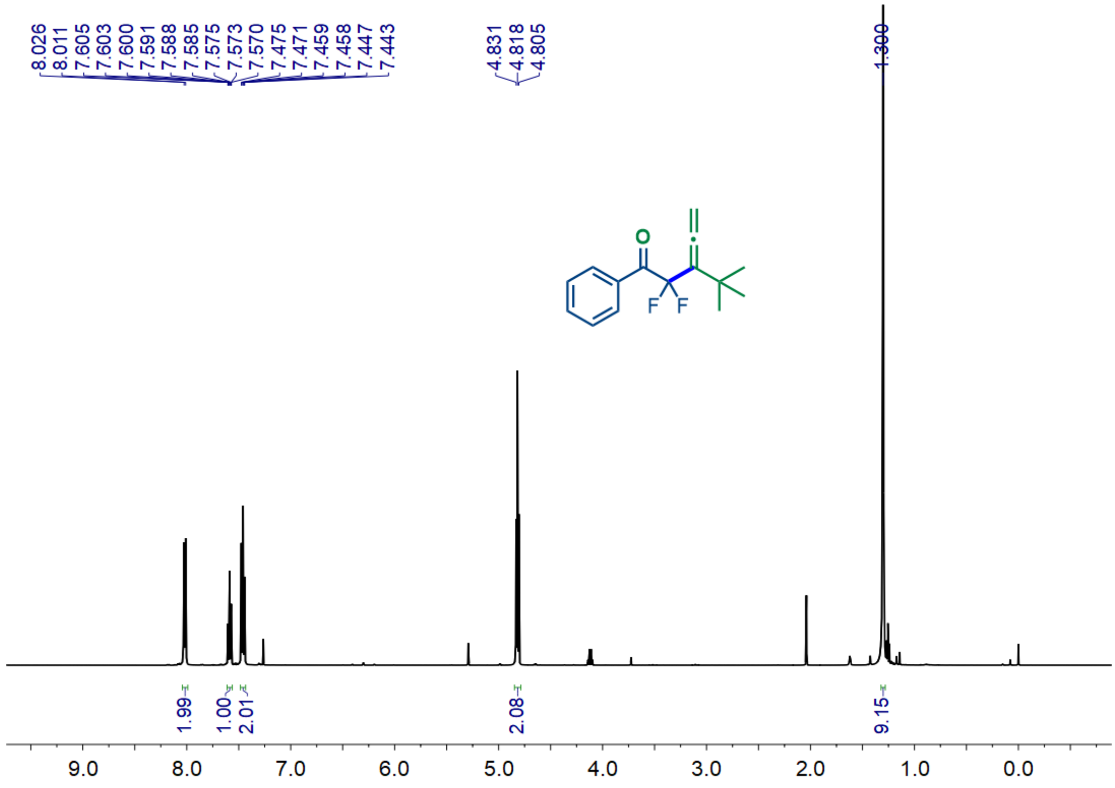

Supplementary Fig. 387 ^{1}H NMR (500 MHz, $CDCl_3$) spectrum of compound **129**

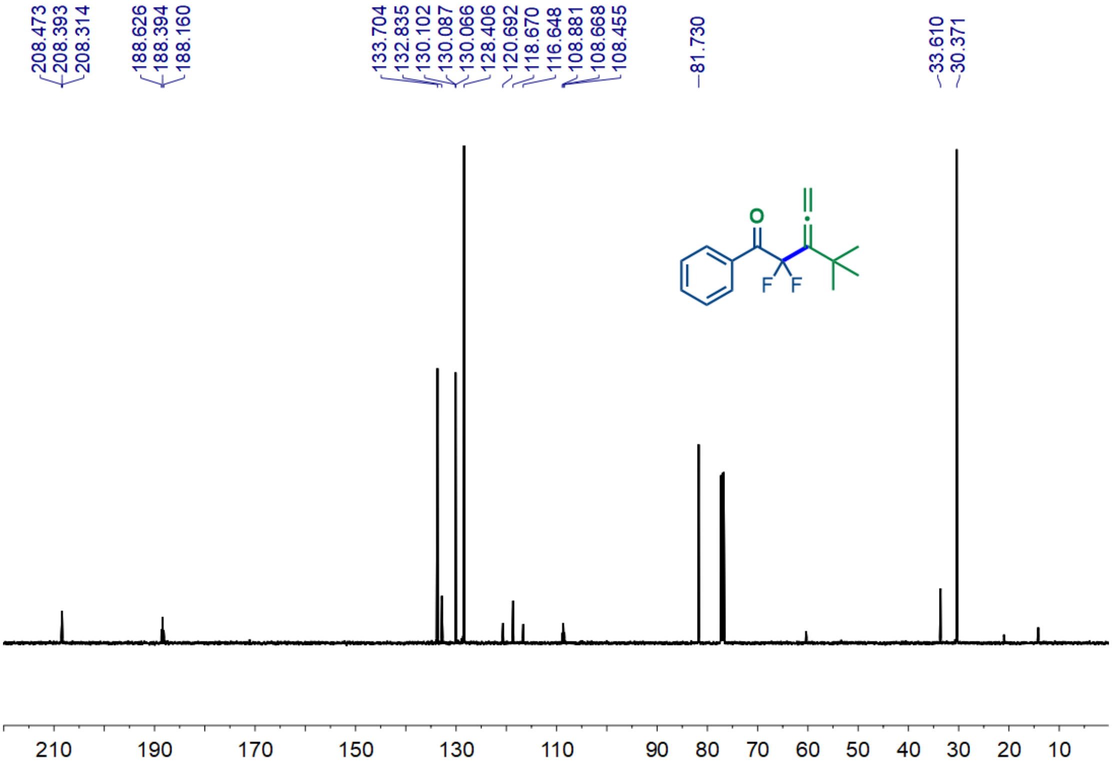

Supplementary Fig. 388 ^{13}C NMR (125 MHz, $CDCl_3$) spectrum of compound **129**

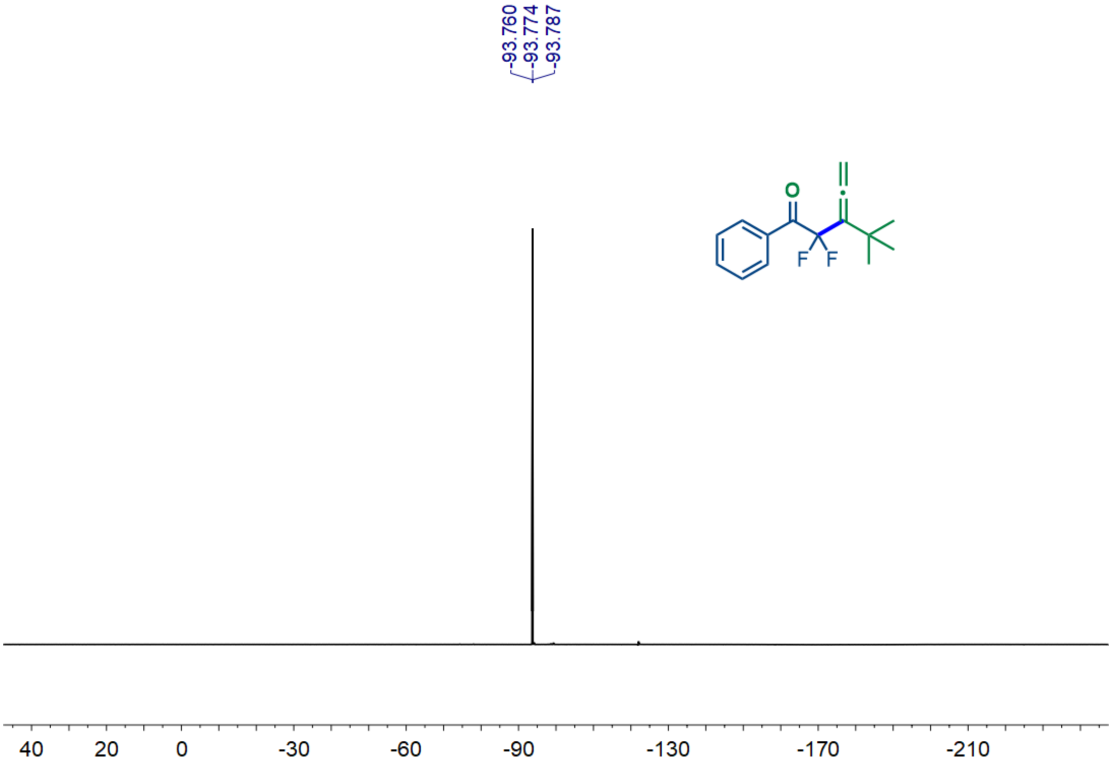

Supplementary Fig. 389 ^{19}F NMR (470 MHz, $CDCl_3$) spectrum of compound **129**

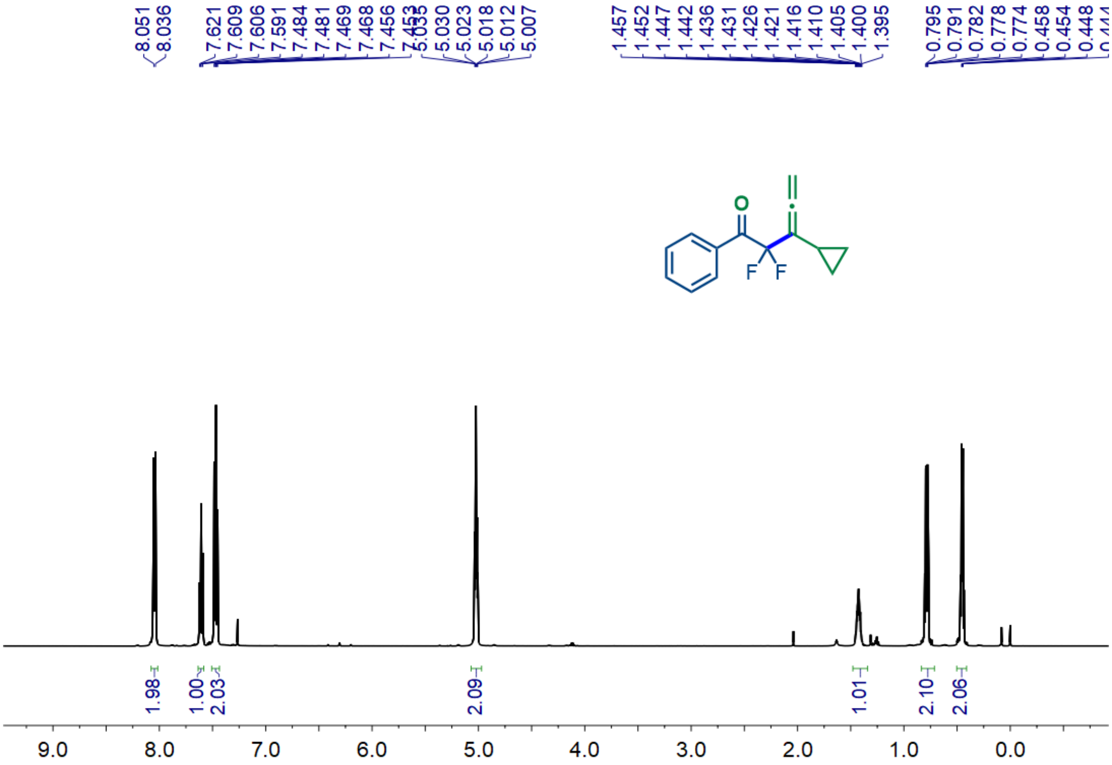

Supplementary Fig. 390 ^{1}H NMR (500 MHz, $CDCl_3$) spectrum of compound **130**

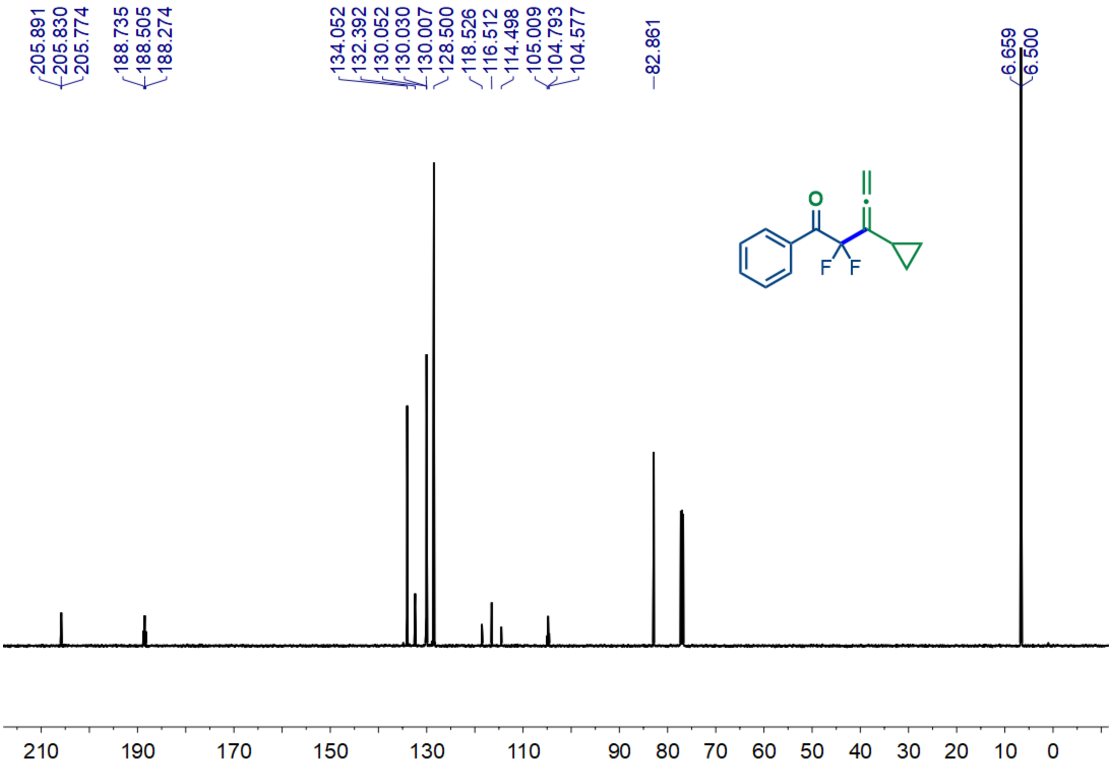

Supplementary Fig. 391 ^{13}C NMR (125 MHz, $CDCl_3$) spectrum of compound **130**

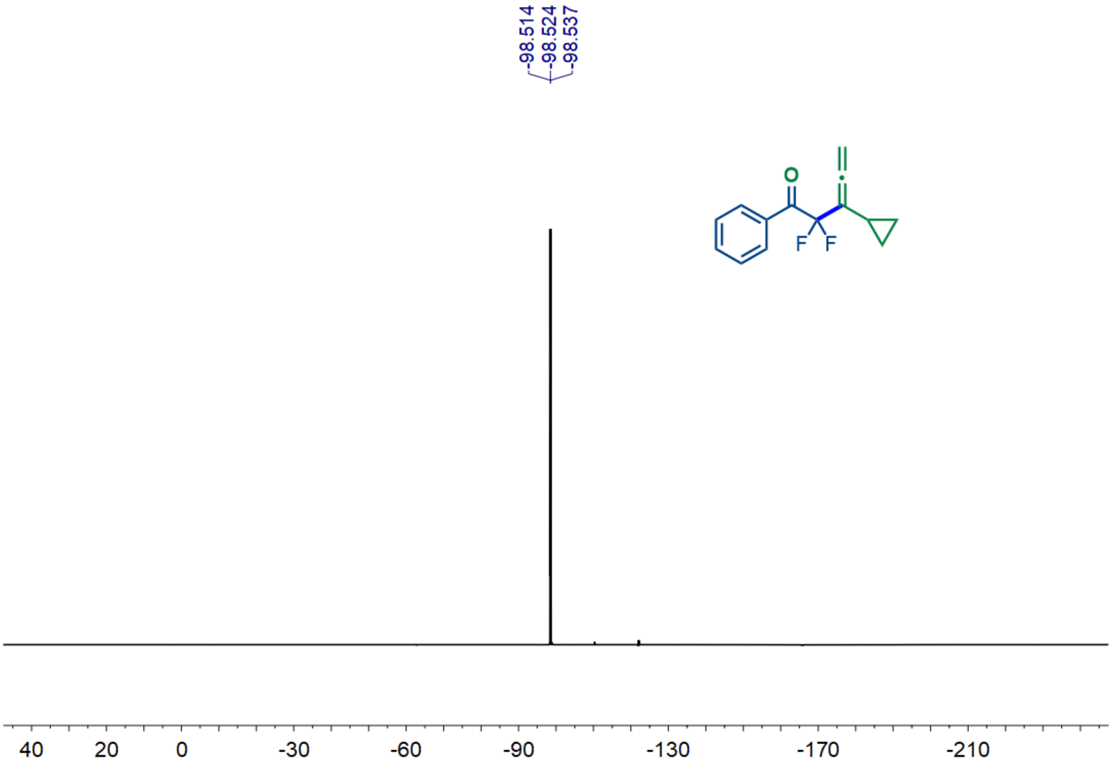

Supplementary Fig. 392 ^{19}F NMR (470 MHz, $CDCl_3$) spectrum of compound **130**

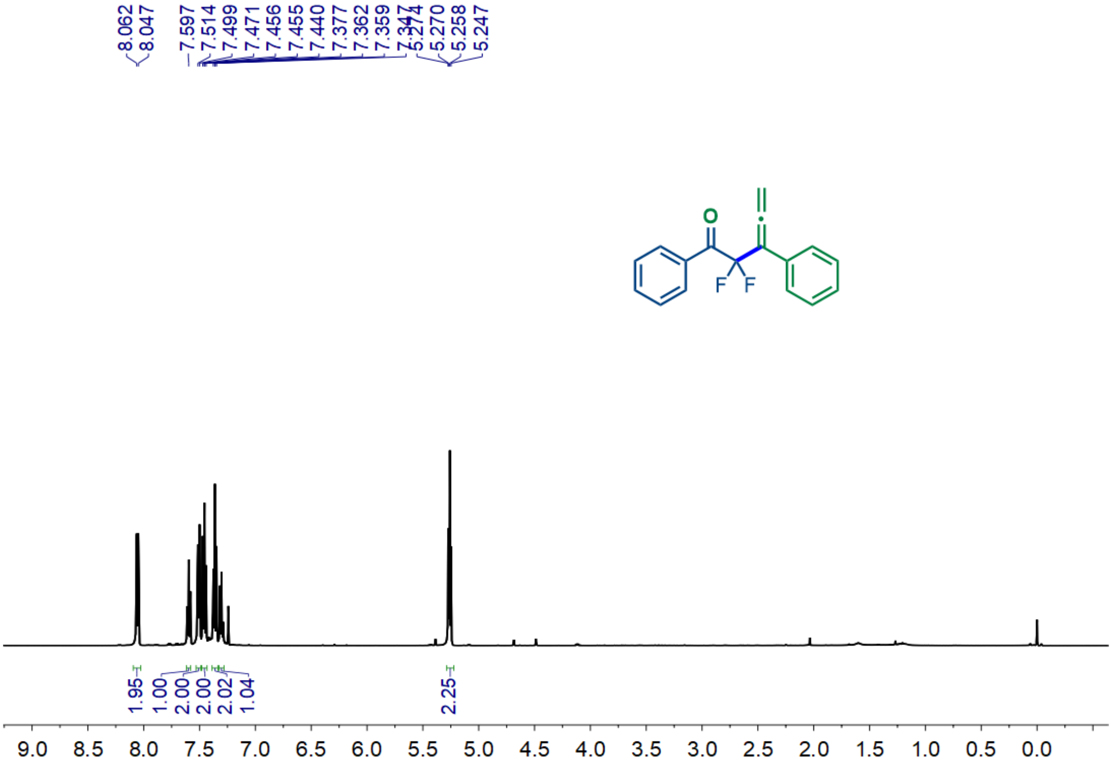

Supplementary Fig. 393 ^{1}H NMR (500 MHz, $CDCl_3$) spectrum of compound **131**

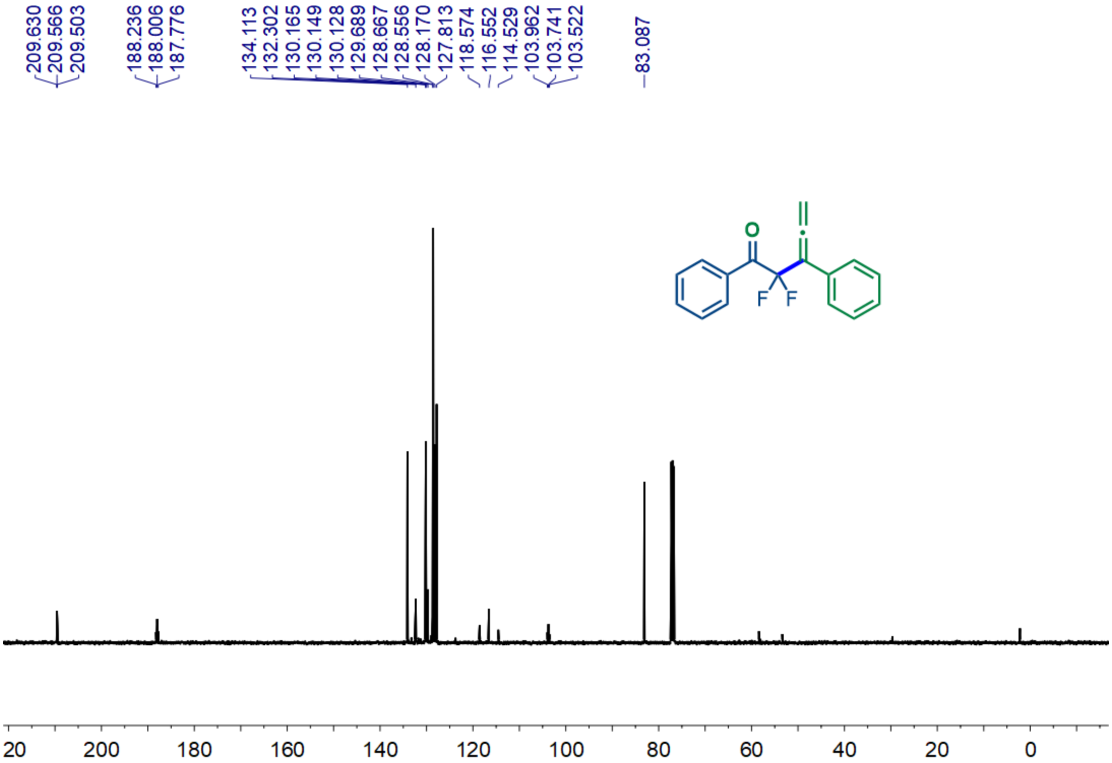

Supplementary Fig. 394 ^{13}C NMR (125 MHz, $CDCl_3$) spectrum of compound **131**

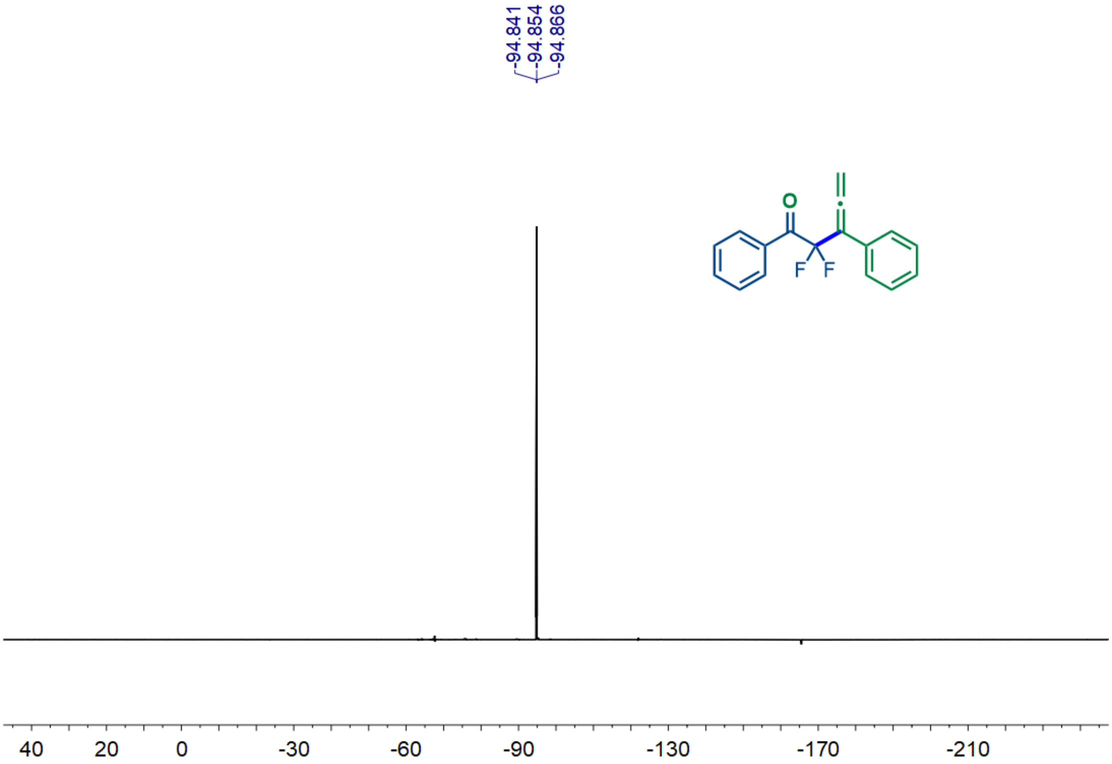

Supplementary Fig. 395 ^{19}F NMR (470 MHz, $CDCl_3$) spectrum of compound **131**

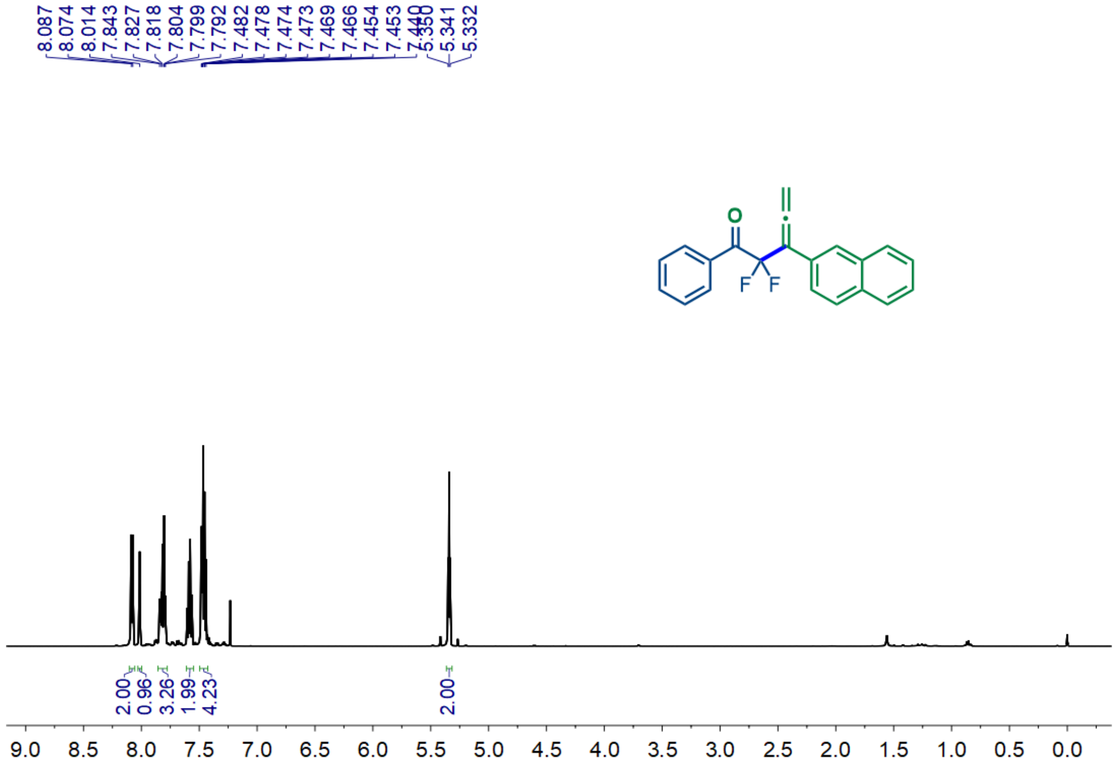

Supplementary Fig. 396 ^{1}H NMR (600 MHz, $CDCl_3$) spectrum of compound **132**

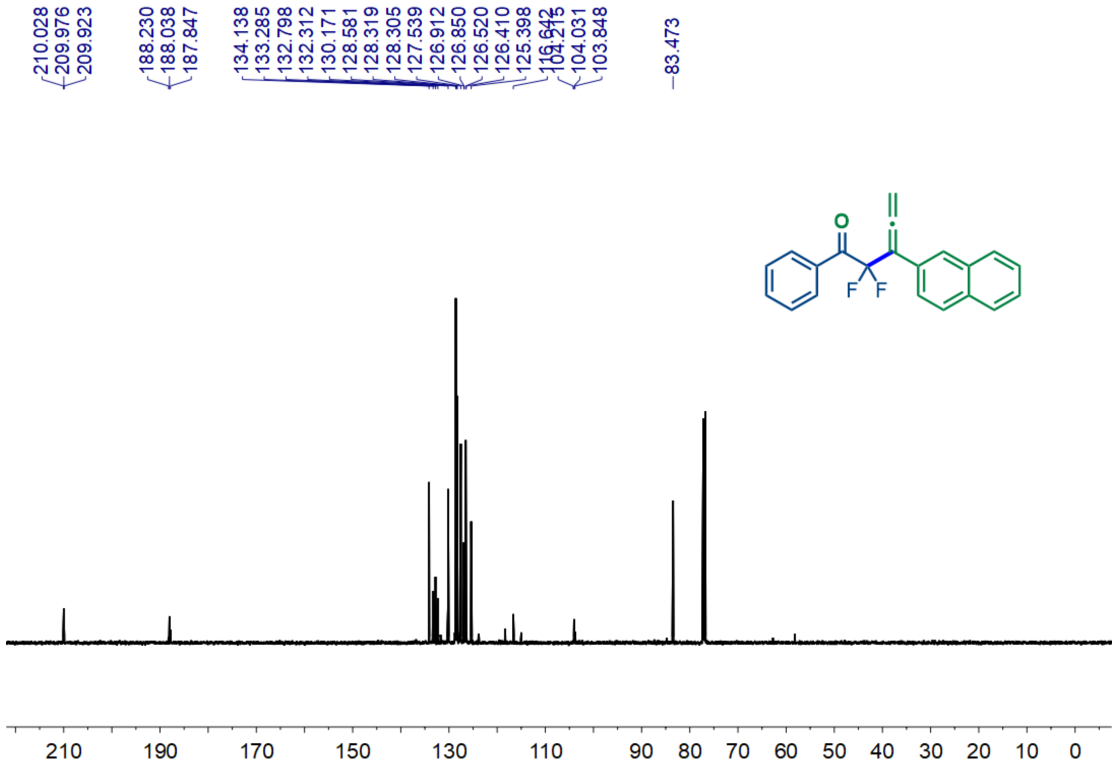

Supplementary Fig. 397 ^{13}C NMR (150 MHz, $CDCl_3$) spectrum of compound **132**

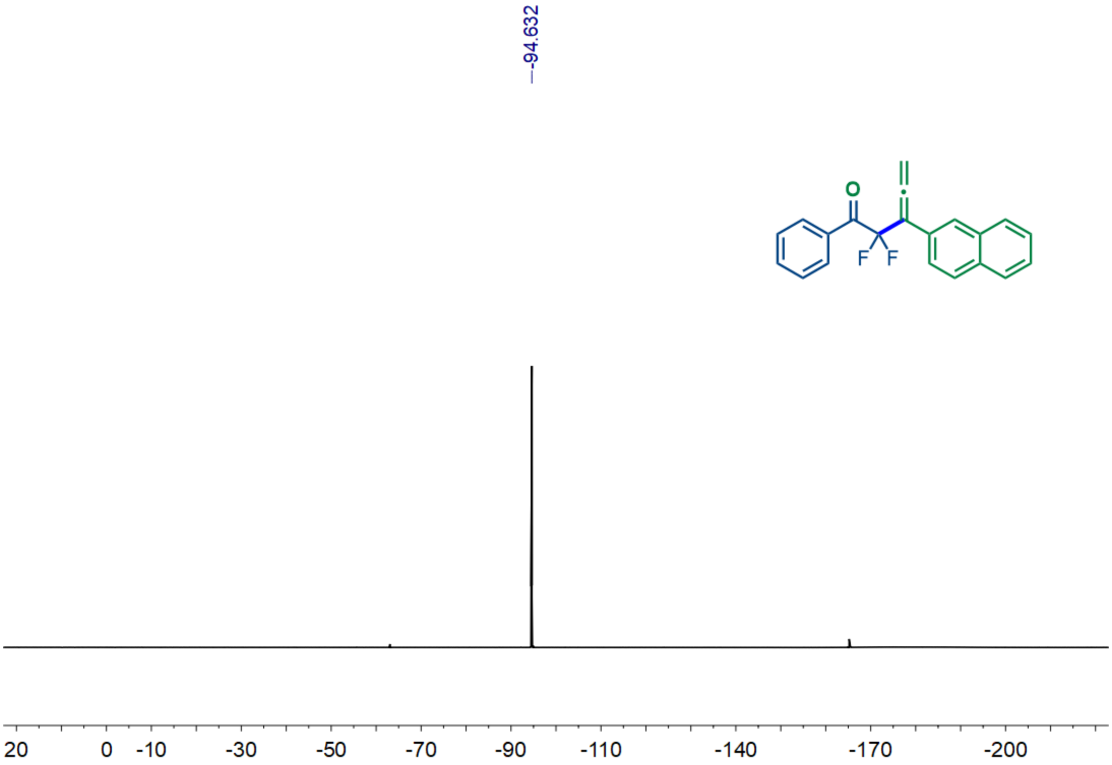

Supplementary Fig. 398 ^{19}F NMR (564 MHz, $CDCl_3$) spectrum of compound **132**

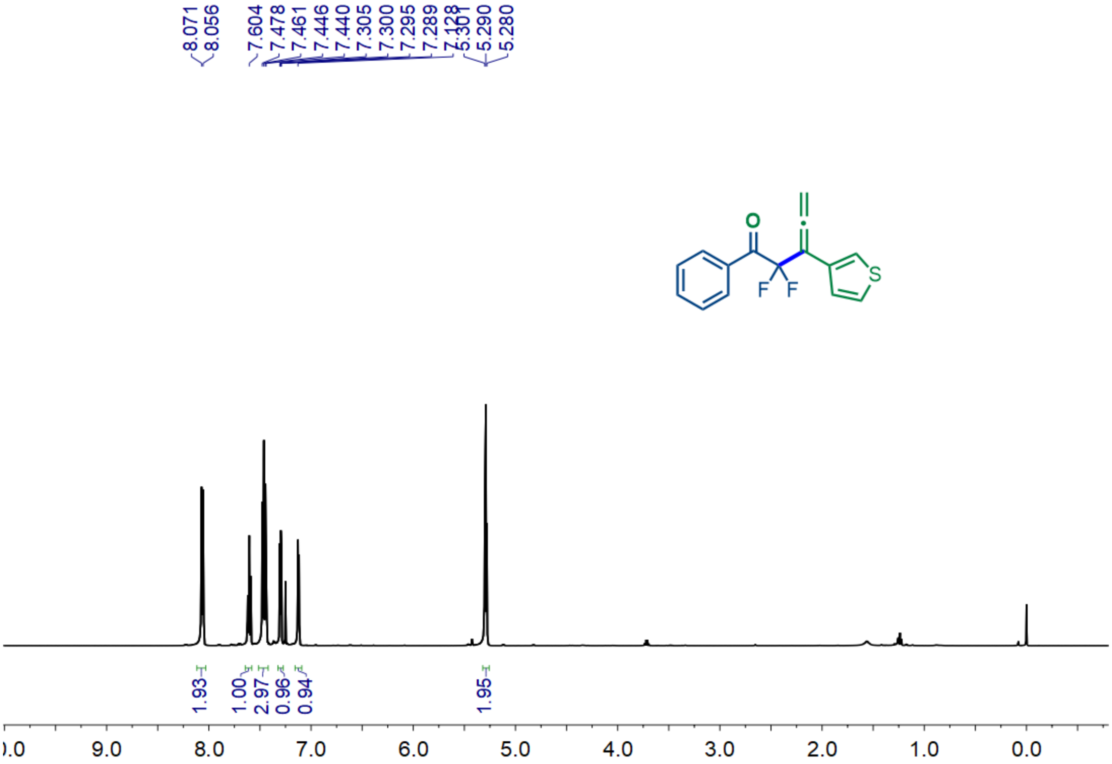

Supplementary Fig. 399 ^{1}H NMR (500 MHz, $CDCl_3$) spectrum of compound **133**

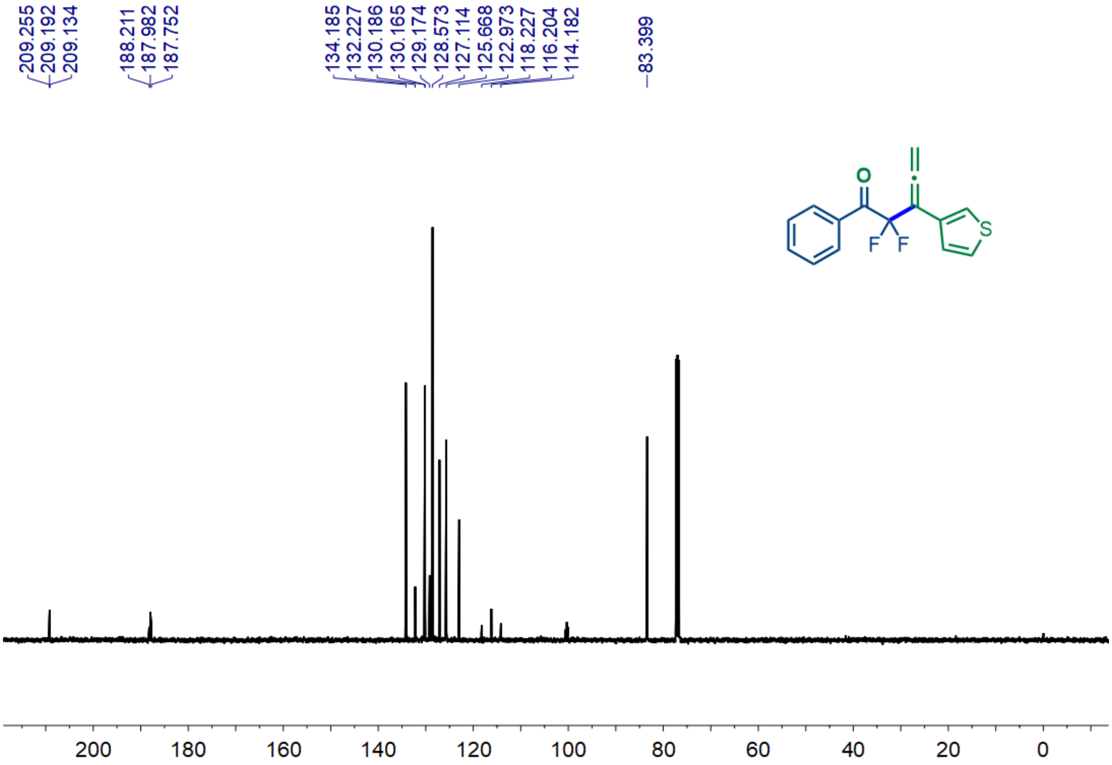

Supplementary Fig. 400 ^{13}C NMR (125 MHz, $CDCl_3$) spectrum of compound **133**

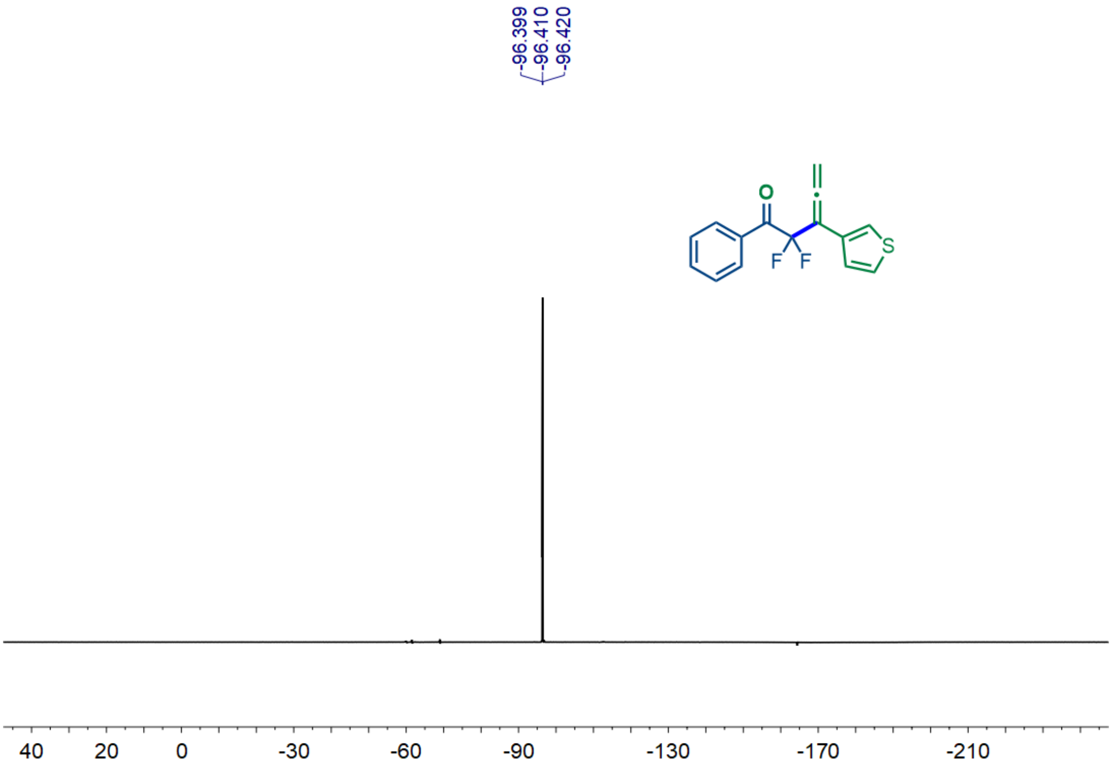

Supplementary Fig. 401 ^{19}F NMR (470 MHz, $CDCl_3$) spectrum of compound **133**

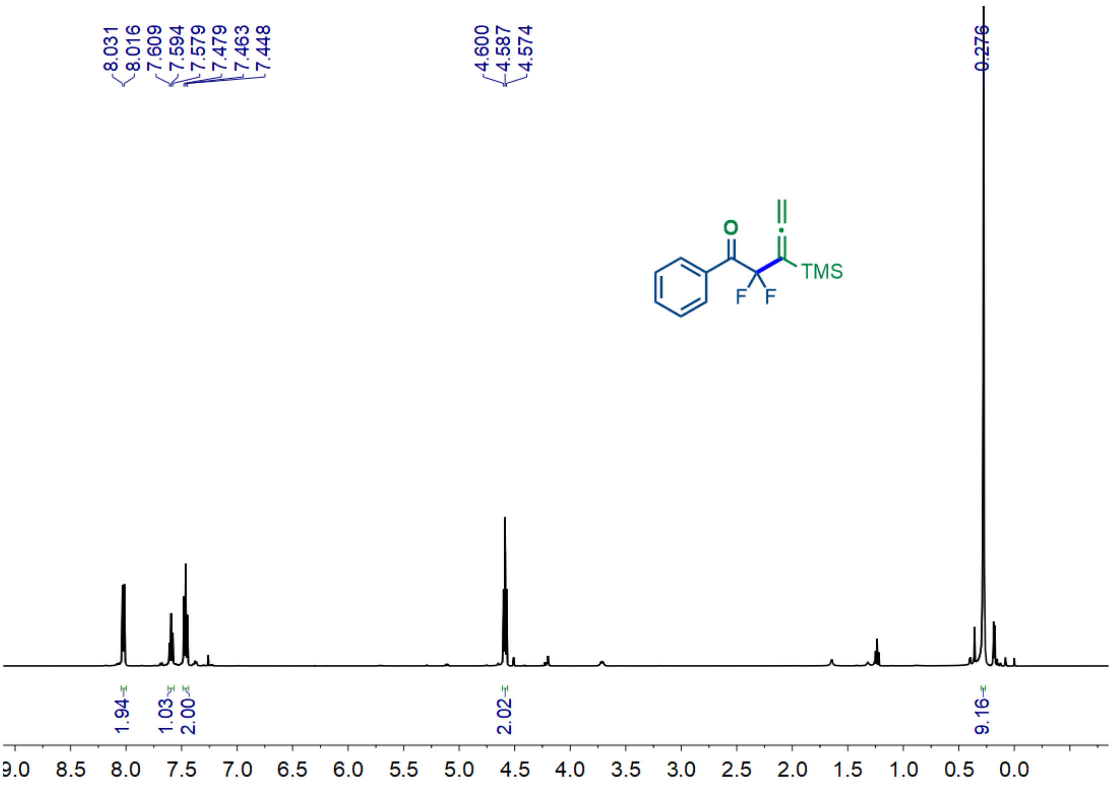

Supplementary Fig. 402 ^{1}H NMR (500 MHz, $CDCl_3$) spectrum of compound **134**

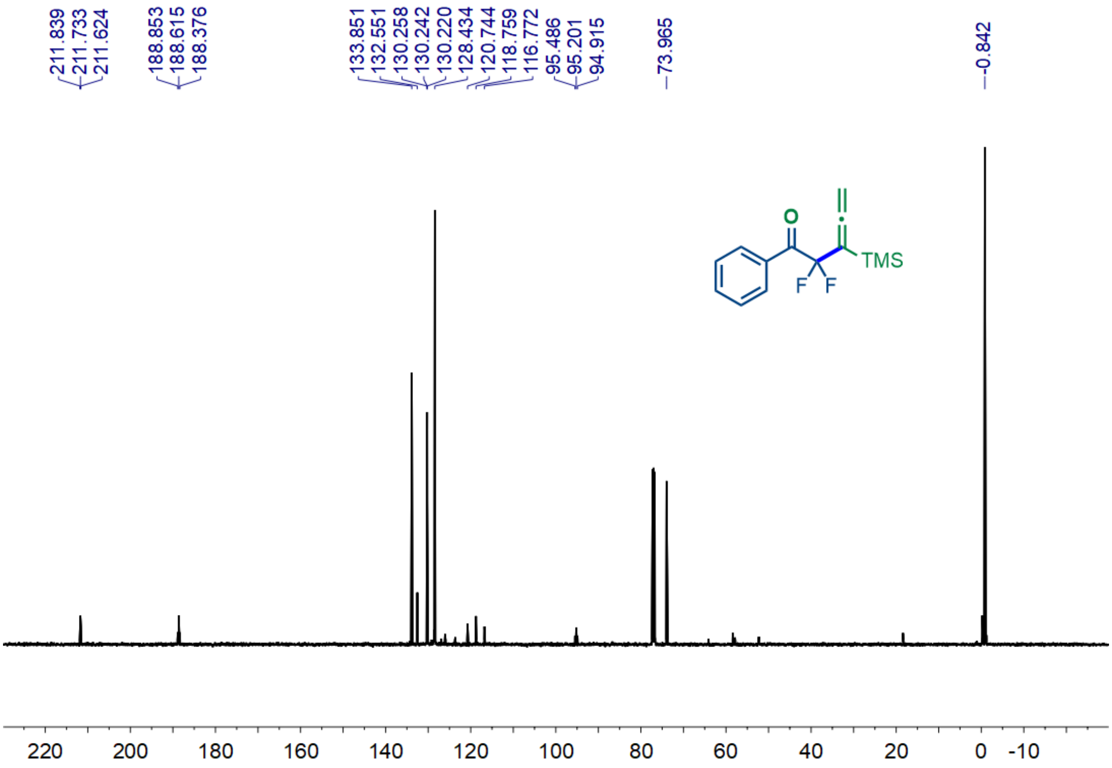

Supplementary Fig. 403 ^{13}C NMR (125 MHz, $CDCl_3$) spectrum of compound **134**

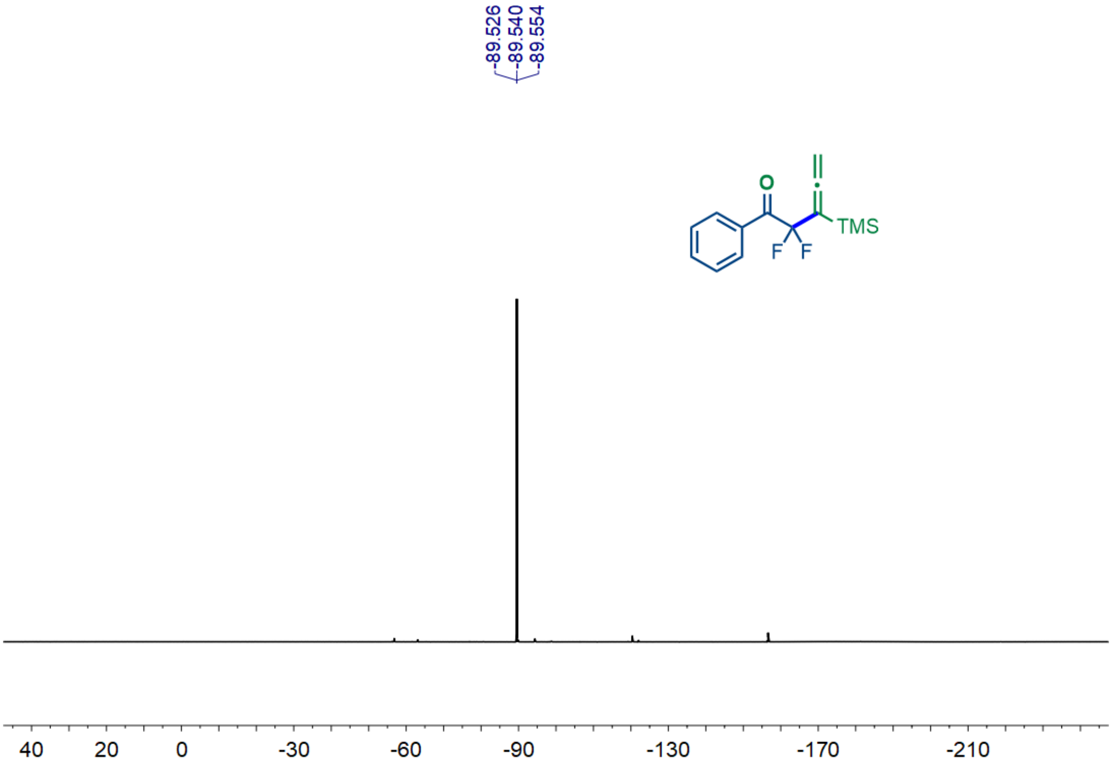

Supplementary Fig. 404 ^{19}F NMR (470 MHz, $CDCl_3$) spectrum of compound **134**

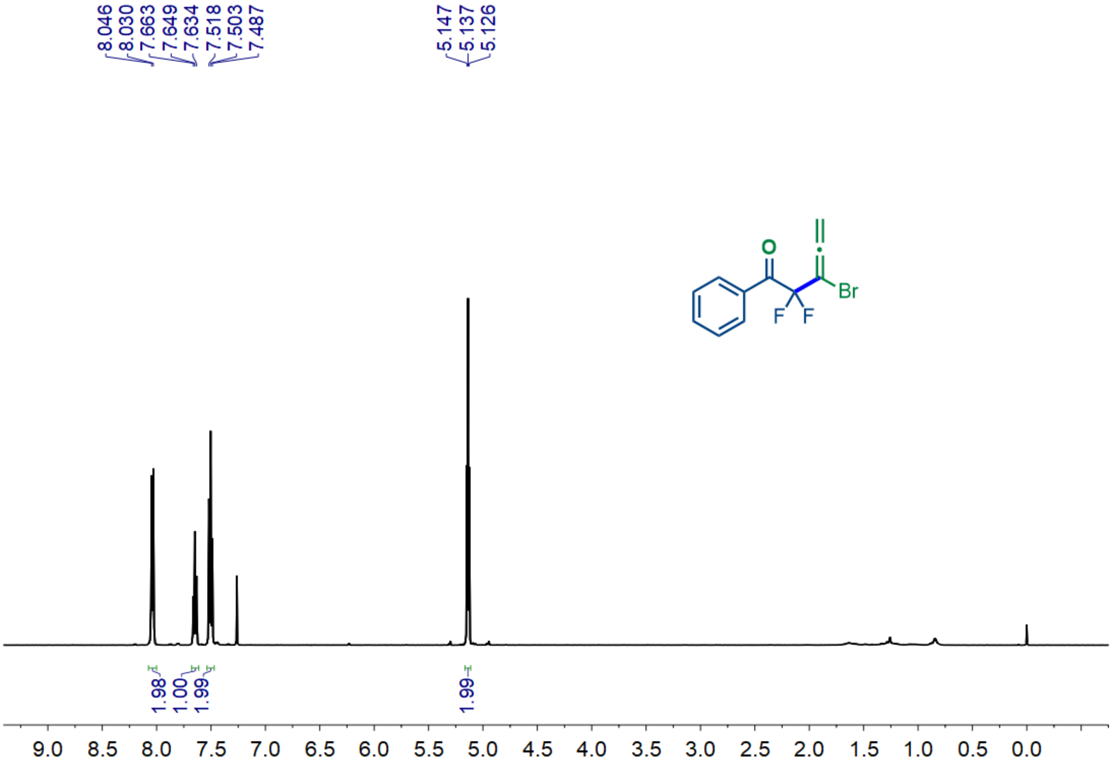

Supplementary Fig. 405 ^{1}H NMR (500 MHz, $CDCl_3$) spectrum of compound **135**

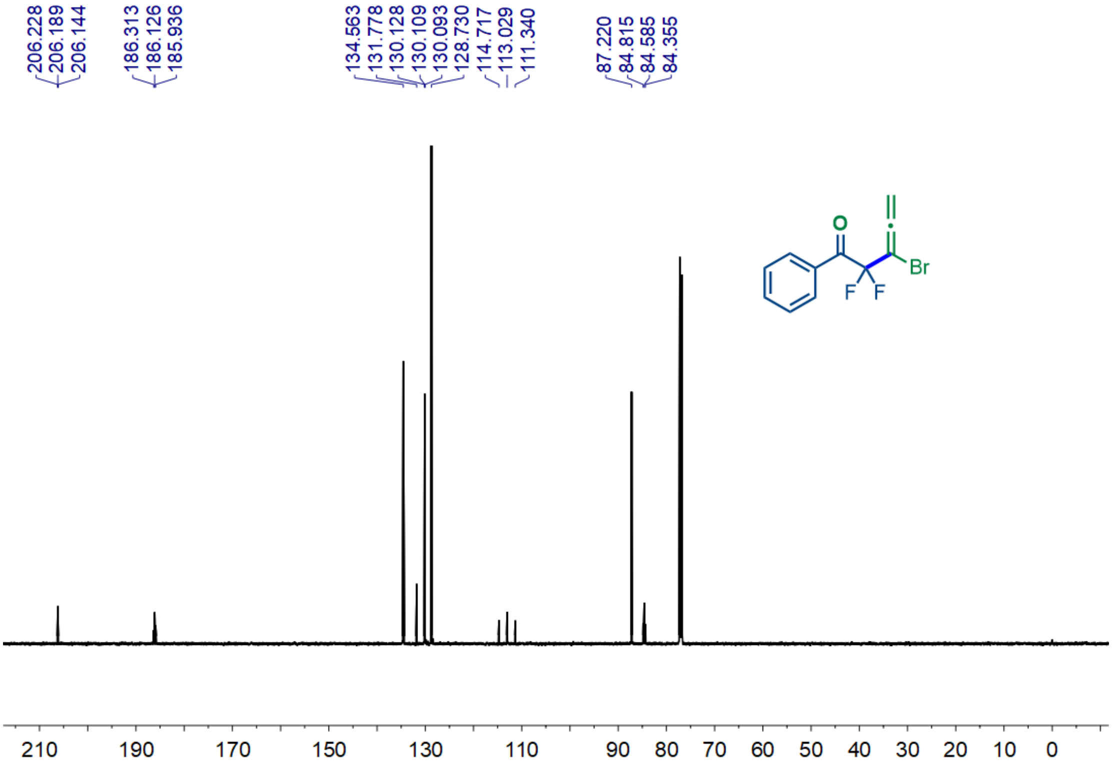

Supplementary Fig. 406 ^{13}C NMR (150 MHz, $CDCl_3$) spectrum of compound **135**

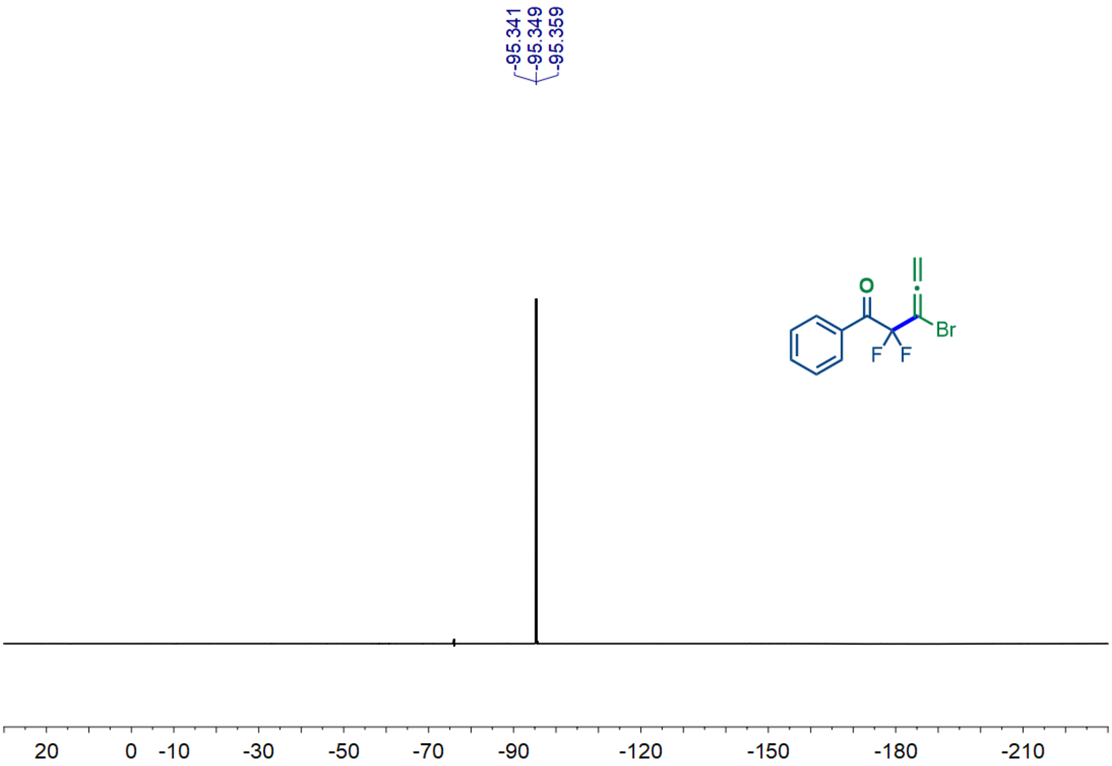

Supplementary Fig. 407 ^{19}F NMR (564 MHz, $CDCl_3$) spectrum of compound **135**

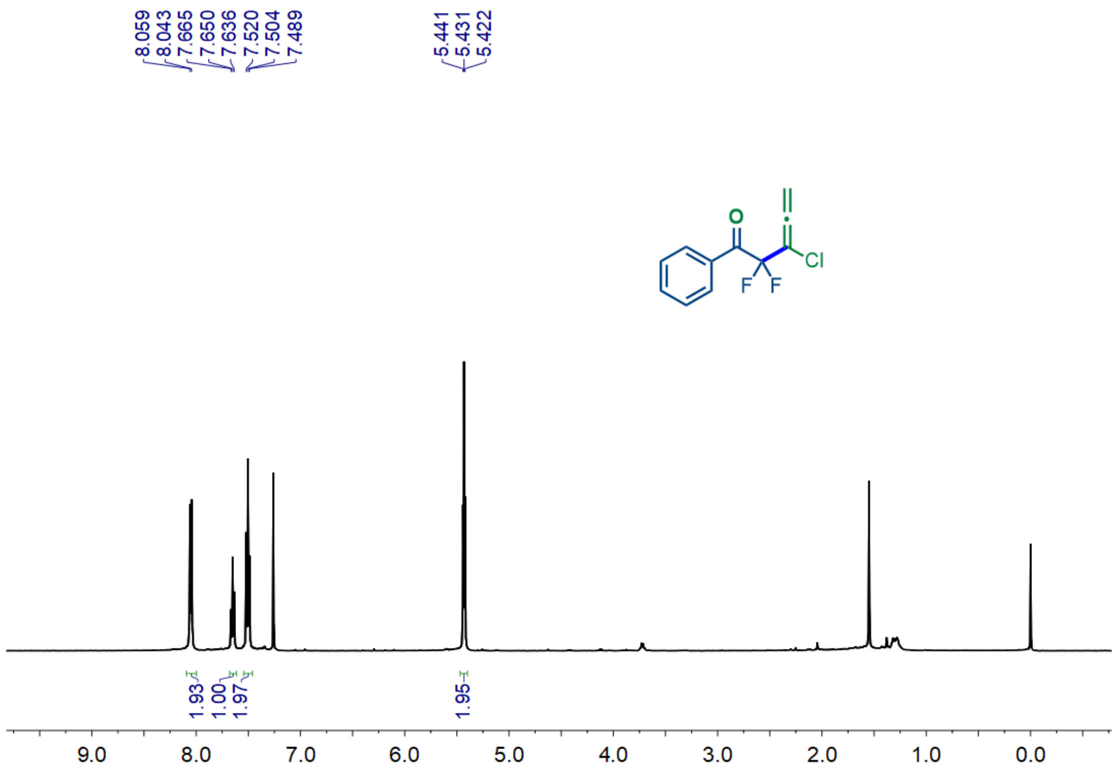

Supplementary Fig. 408 ^{1}H NMR (500 MHz, $CDCl_3$) spectrum of compound **136**

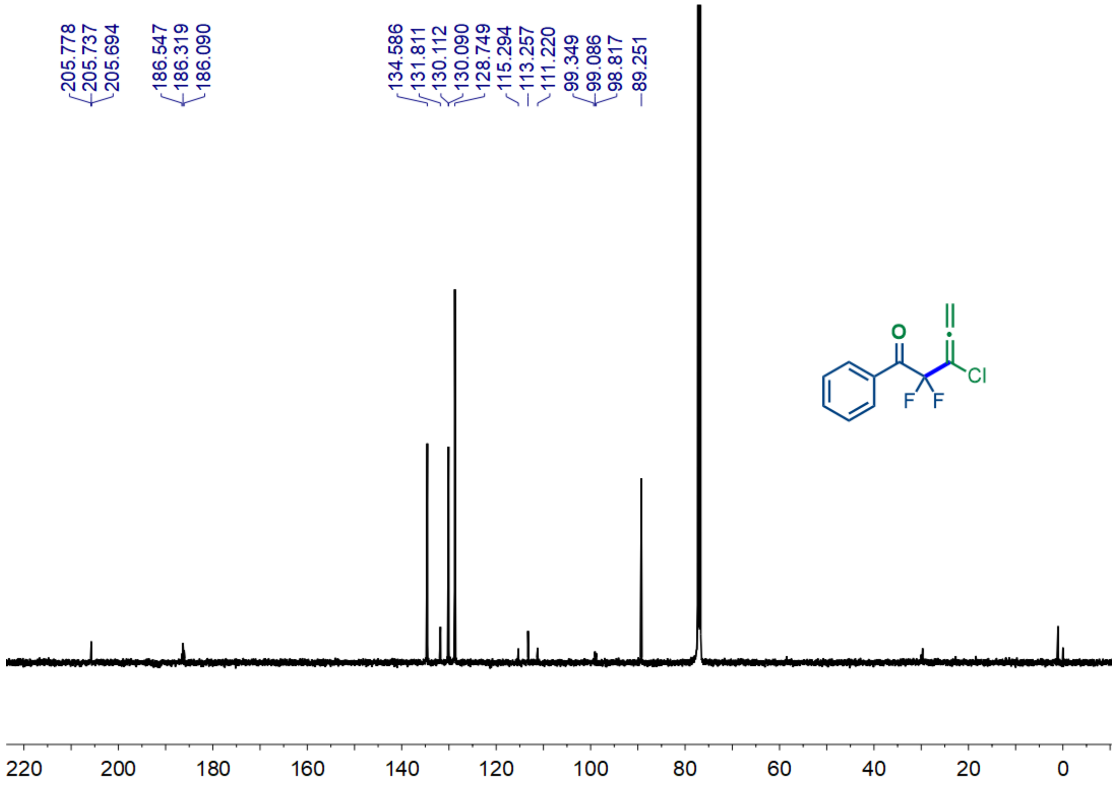

Supplementary Fig. 409 ^{13}C NMR (150 MHz, $CDCl_3$) spectrum of compound **136**

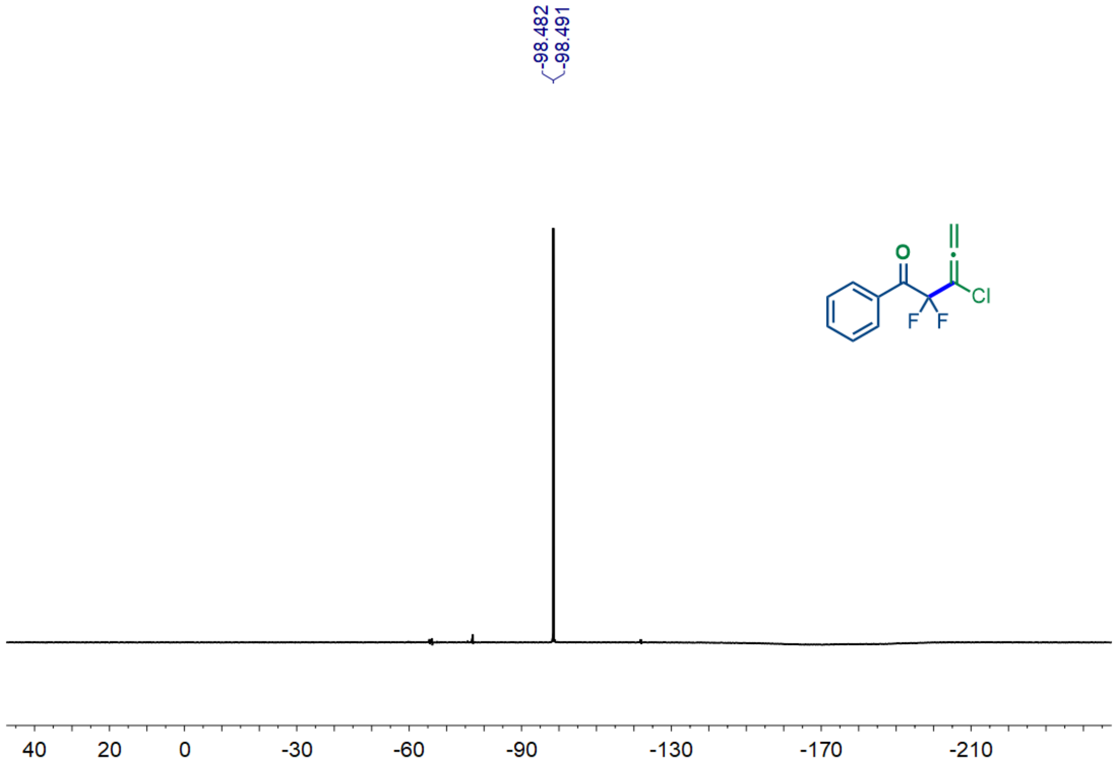

Supplementary Fig. 410 ^{19}F NMR (470 MHz, $CDCl_3$) spectrum of compound **136**

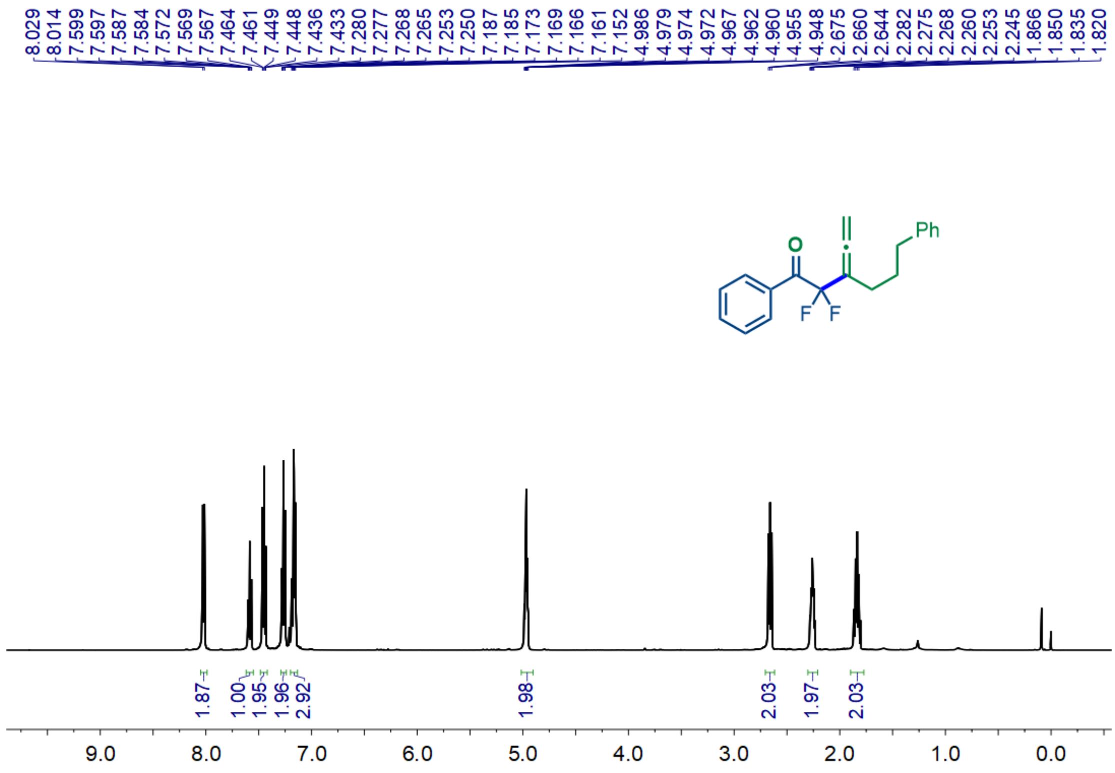

Supplementary Fig. 411 ^{1}H NMR (500 MHz, $CDCl_3$) spectrum of compound **137**

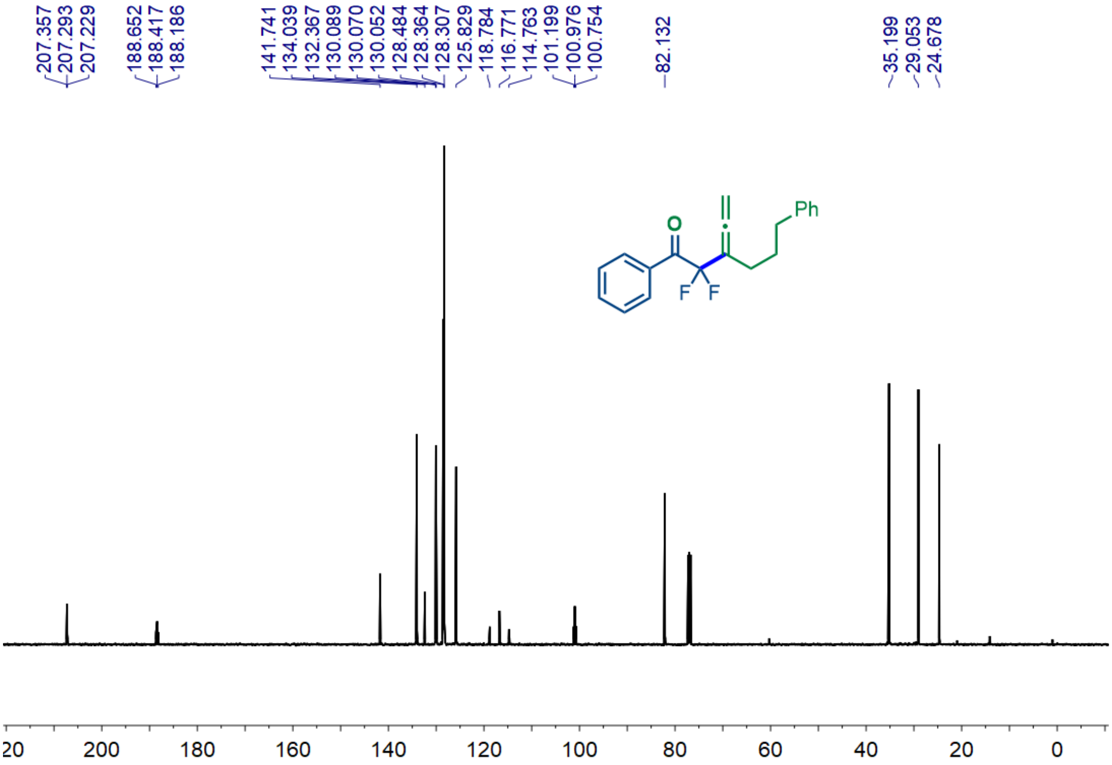

Supplementary Fig. 412 ^{13}C NMR (125 MHz, $CDCl_3$) spectrum of compound **137**

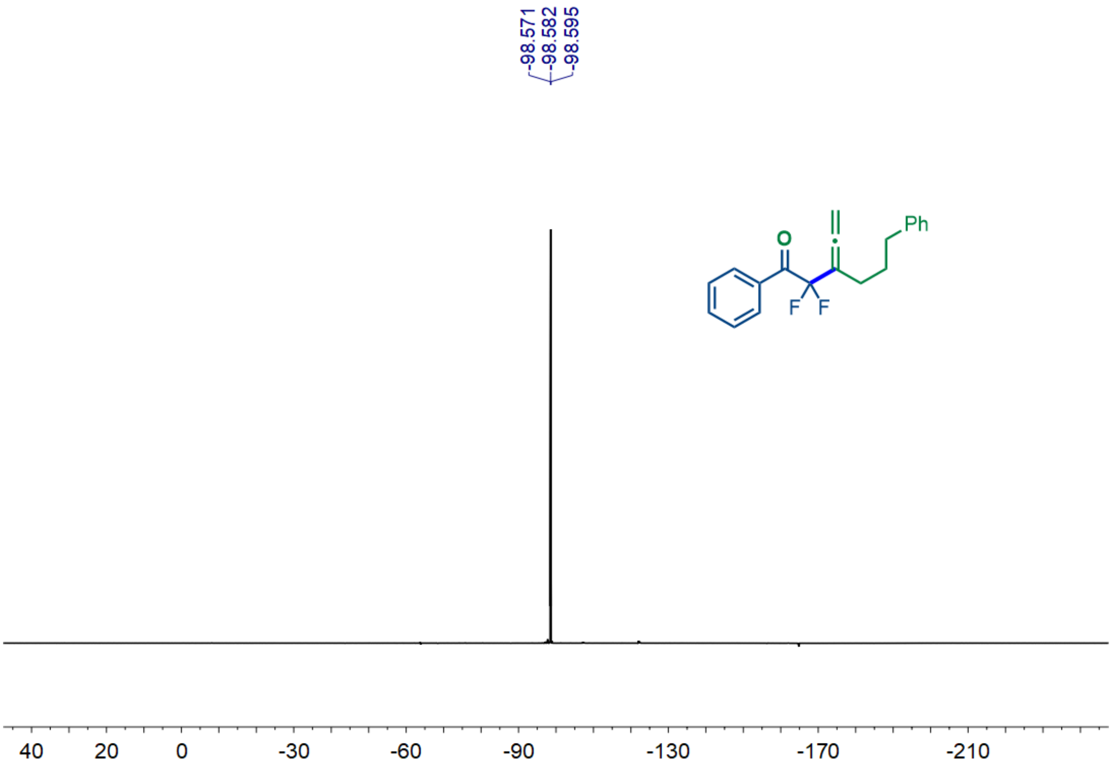

Supplementary Fig. 413 ^{19}F NMR (470 MHz, $CDCl_3$) spectrum of compound **137**

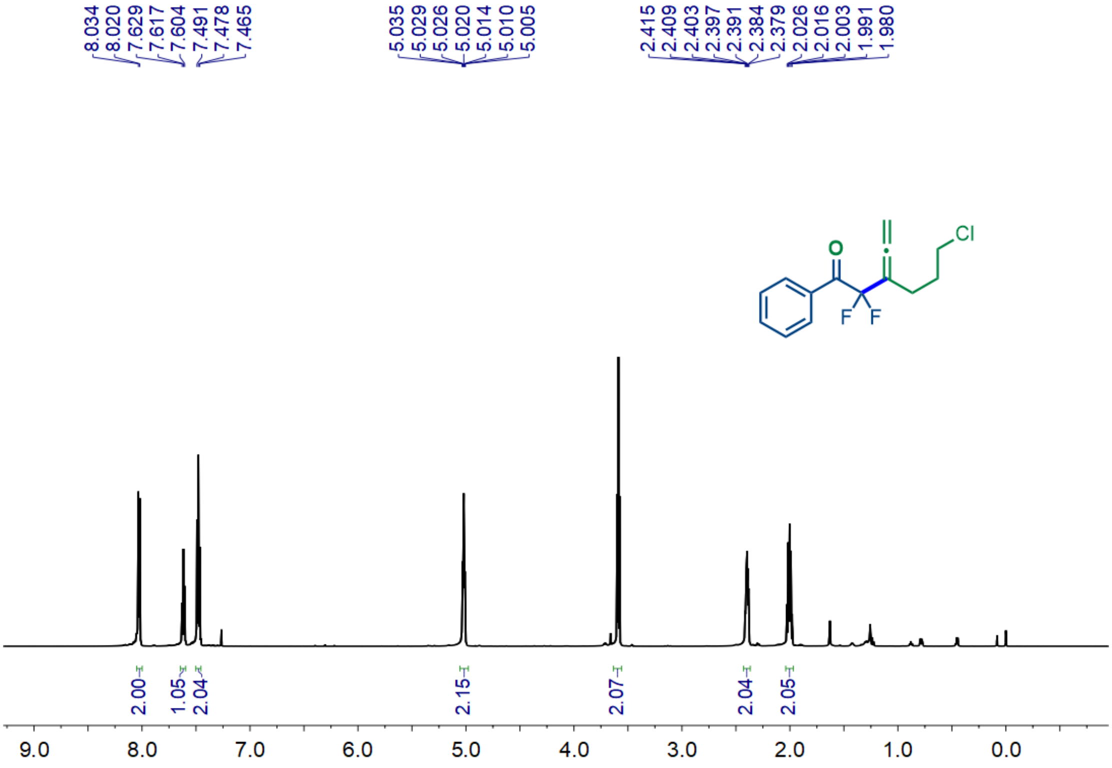

Supplementary Fig. 414 ^{1}H NMR (600 MHz, $CDCl_3$) spectrum of compound **138**

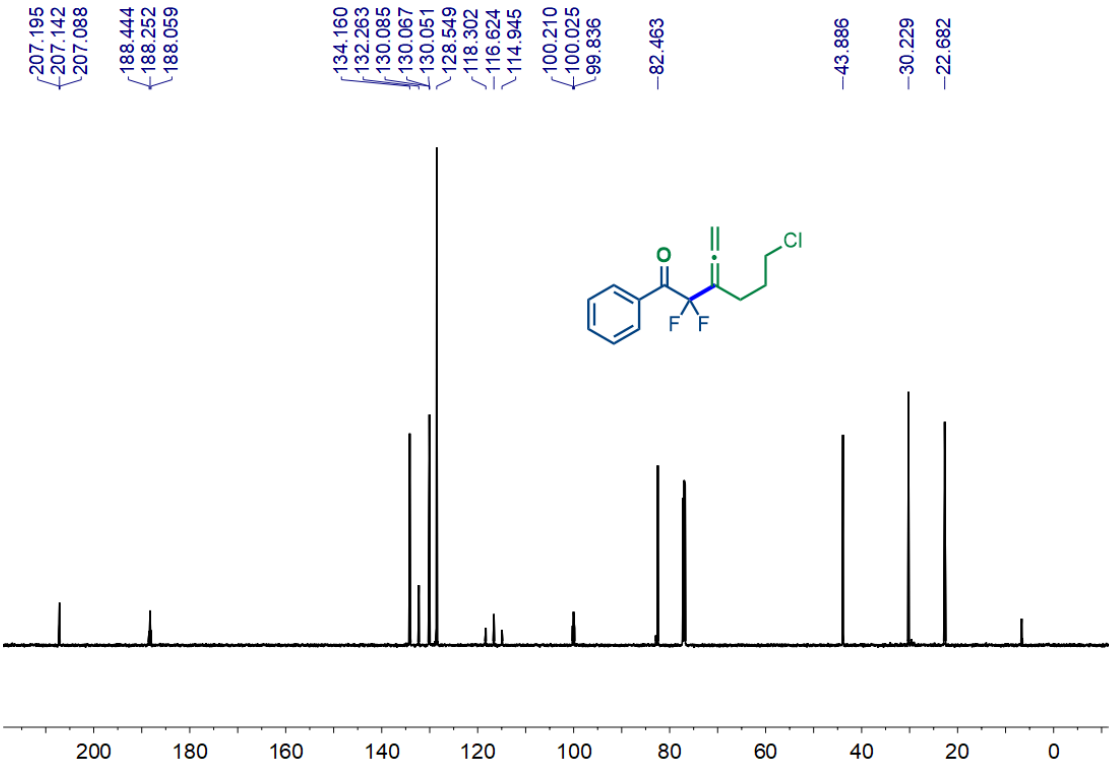

Supplementary Fig. 415 ^{13}C NMR (150 MHz, $CDCl_3$) spectrum of compound **138**

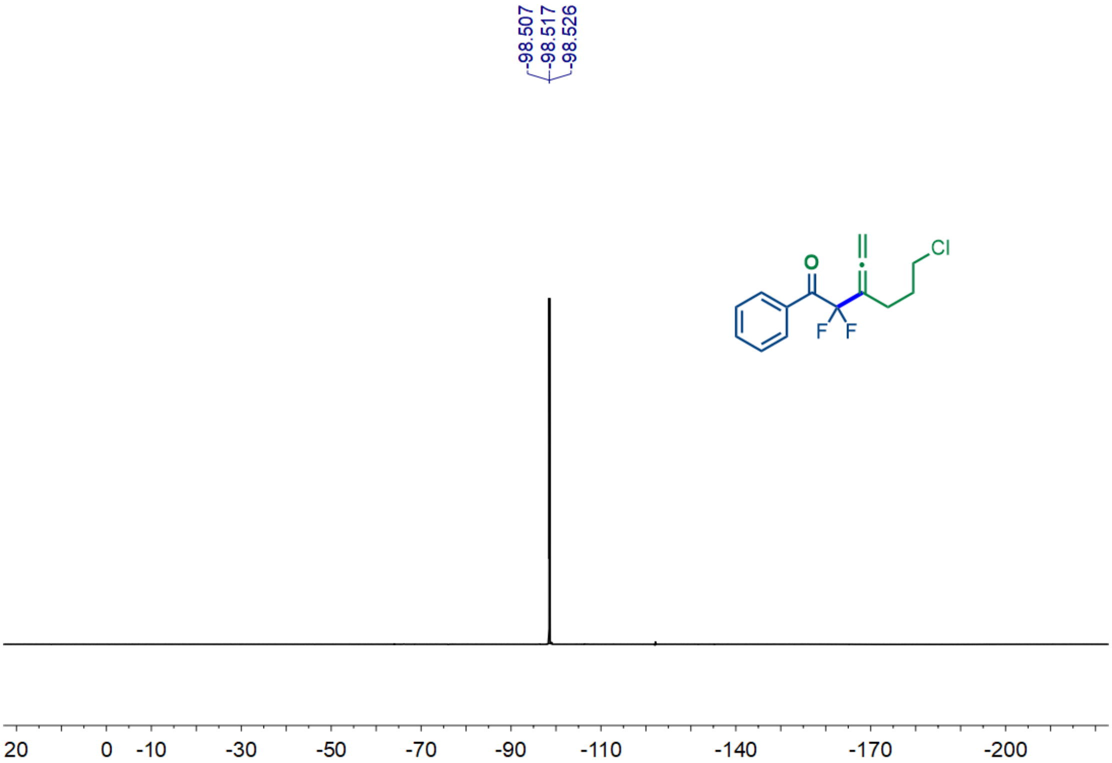

Supplementary Fig. 416 ^{19}F NMR (564 MHz, $CDCl_3$) spectrum of compound **138**

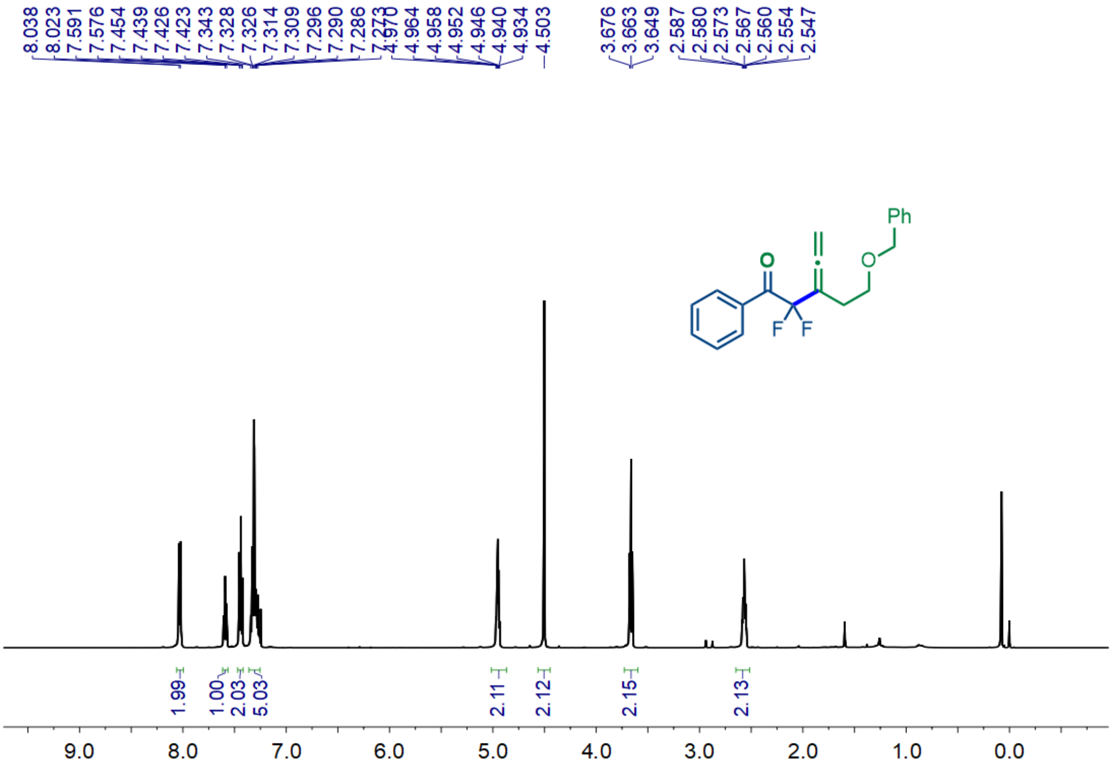

Supplementary Fig. 417 ^{1}H NMR (500 MHz, $CDCl_3$) spectrum of compound **139**

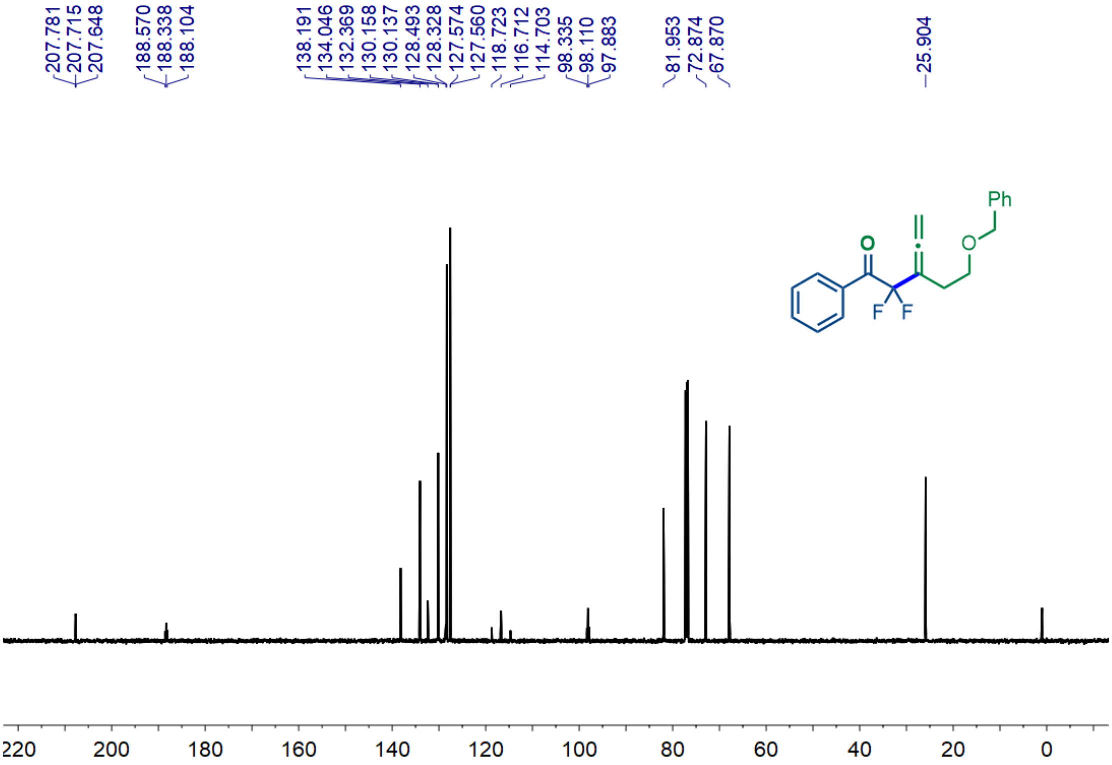

Supplementary Fig. 418 ^{13}C NMR (125 MHz, $CDCl_3$) spectrum of compound **139**

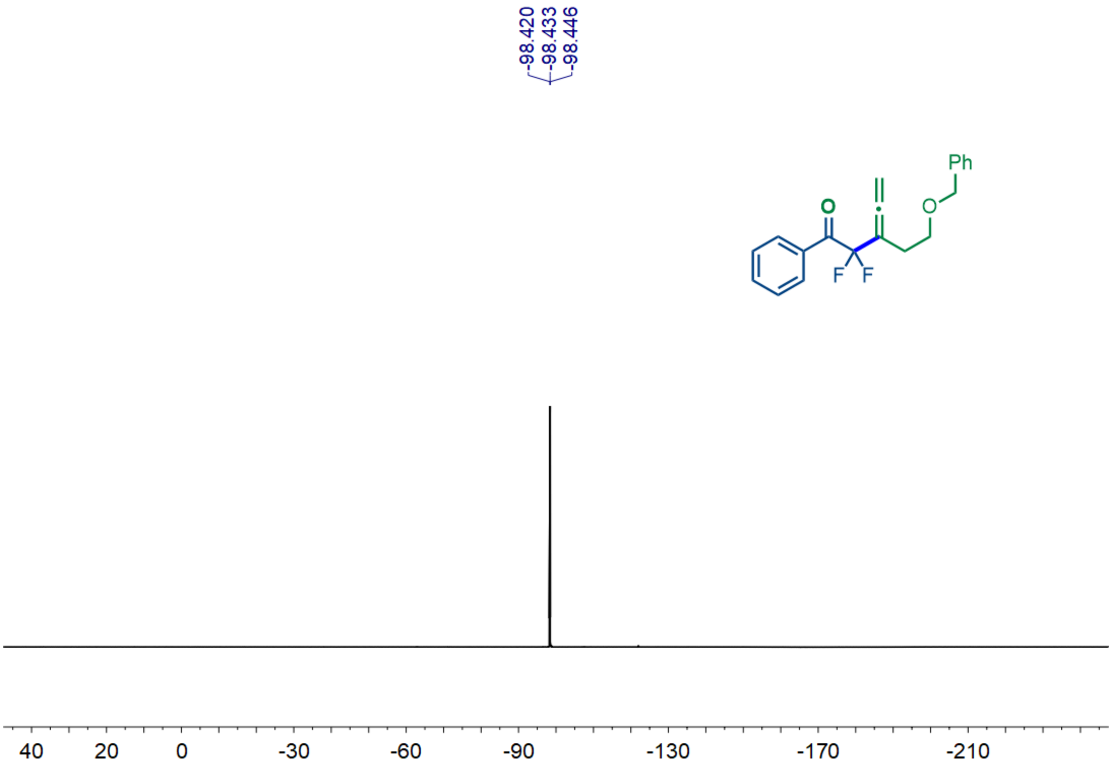

Supplementary Fig. 419 ^{19}F NMR (470 MHz, $CDCl_3$) spectrum of compound **139**

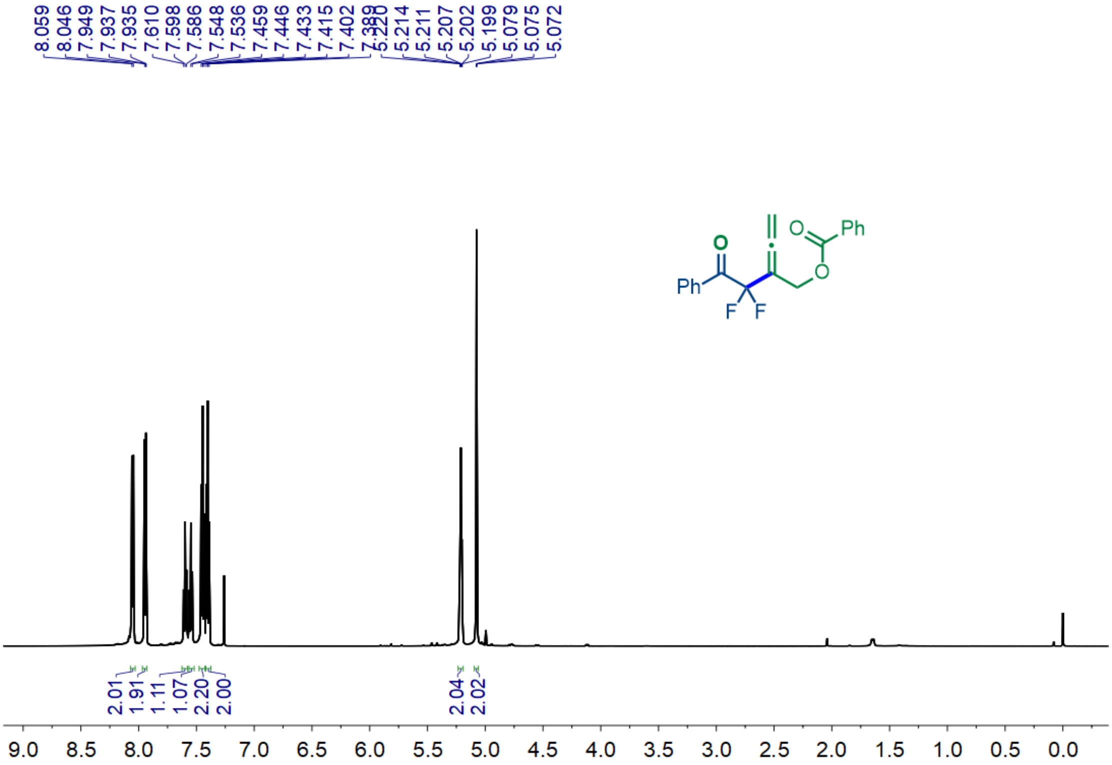

Supplementary Fig. 420 ^{1}H NMR (600 MHz, $CDCl_3$) spectrum of compound **140**

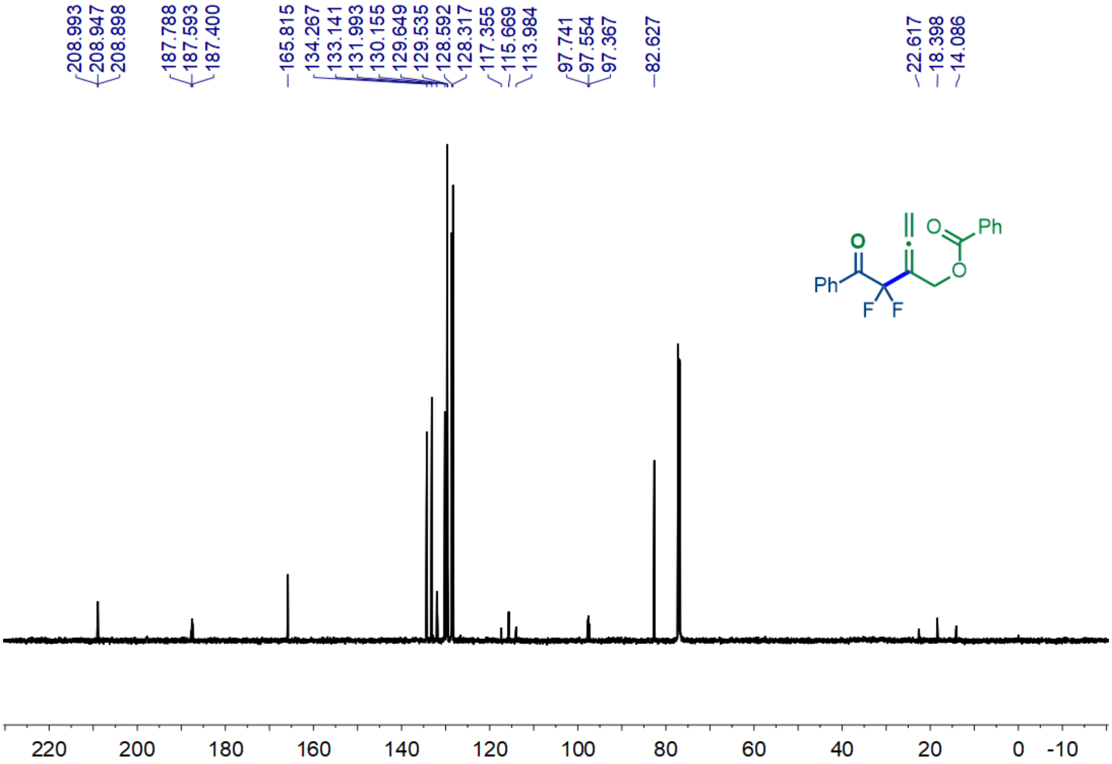

Supplementary Fig. 421 ^{13}C NMR (150 MHz, $CDCl_3$) spectrum of compound **140**

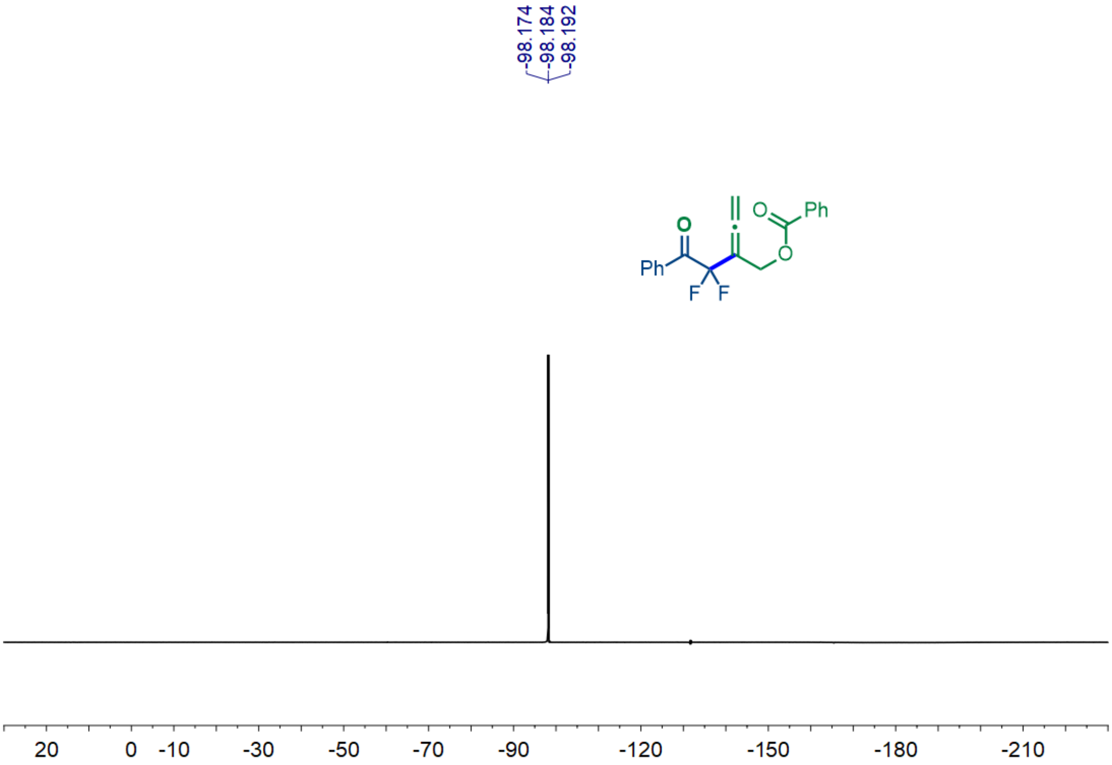

Supplementary Fig. 422 ^{19}F NMR (564 MHz, $CDCl_3$) spectrum of compound **140**

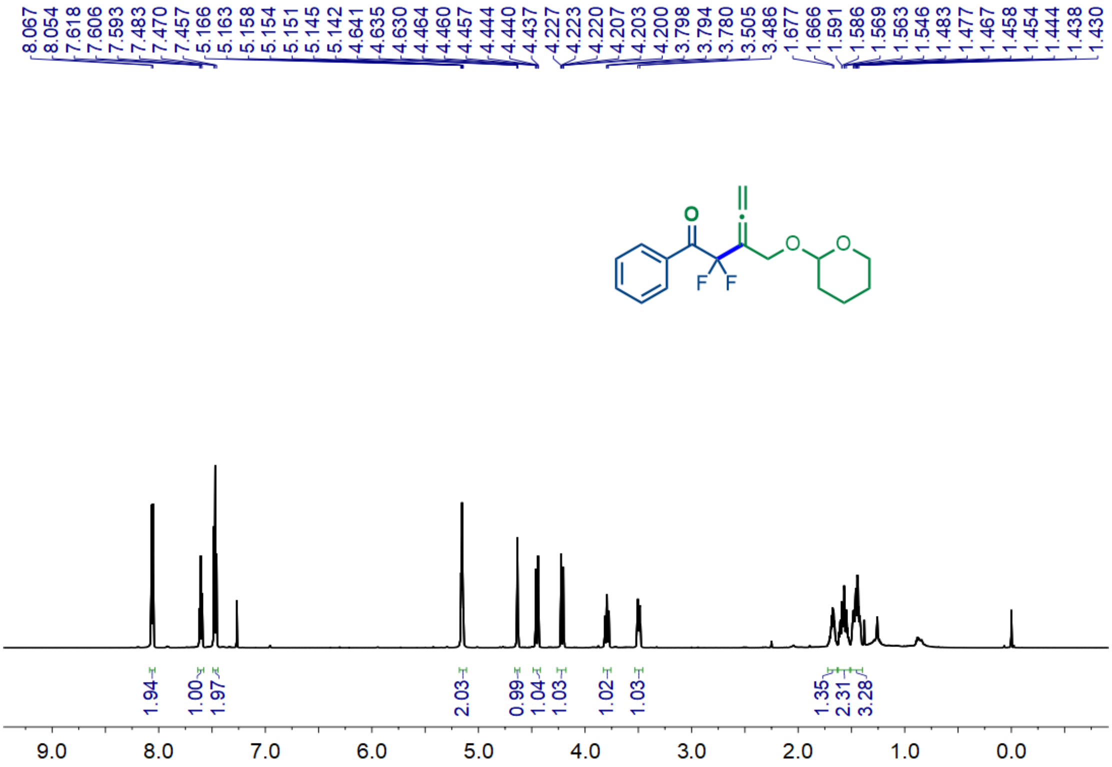

Supplementary Fig. 423 ^{1}H NMR (600 MHz, $CDCl_3$) spectrum of compound **141**

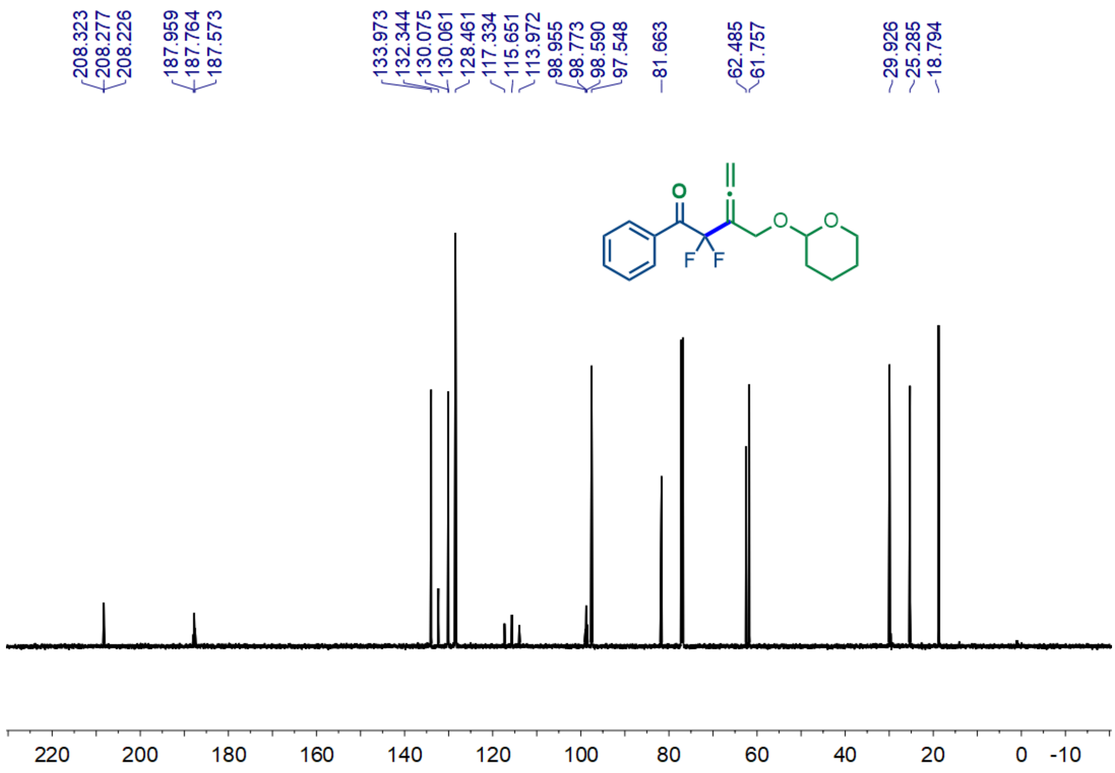

Supplementary Fig. 424 ^{13}C NMR (150 MHz, $CDCl_3$) spectrum of compound **141**

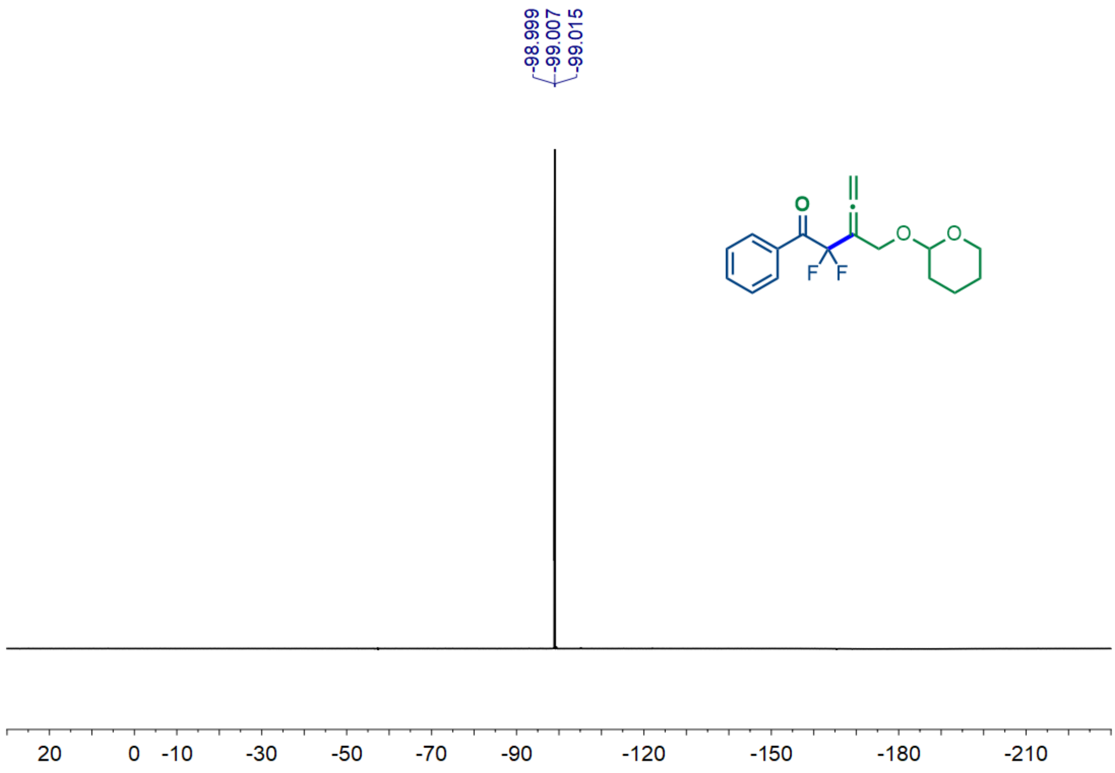

Supplementary Fig. 425 ^{19}F NMR (564 MHz, $CDCl_3$) spectrum of compound **141**

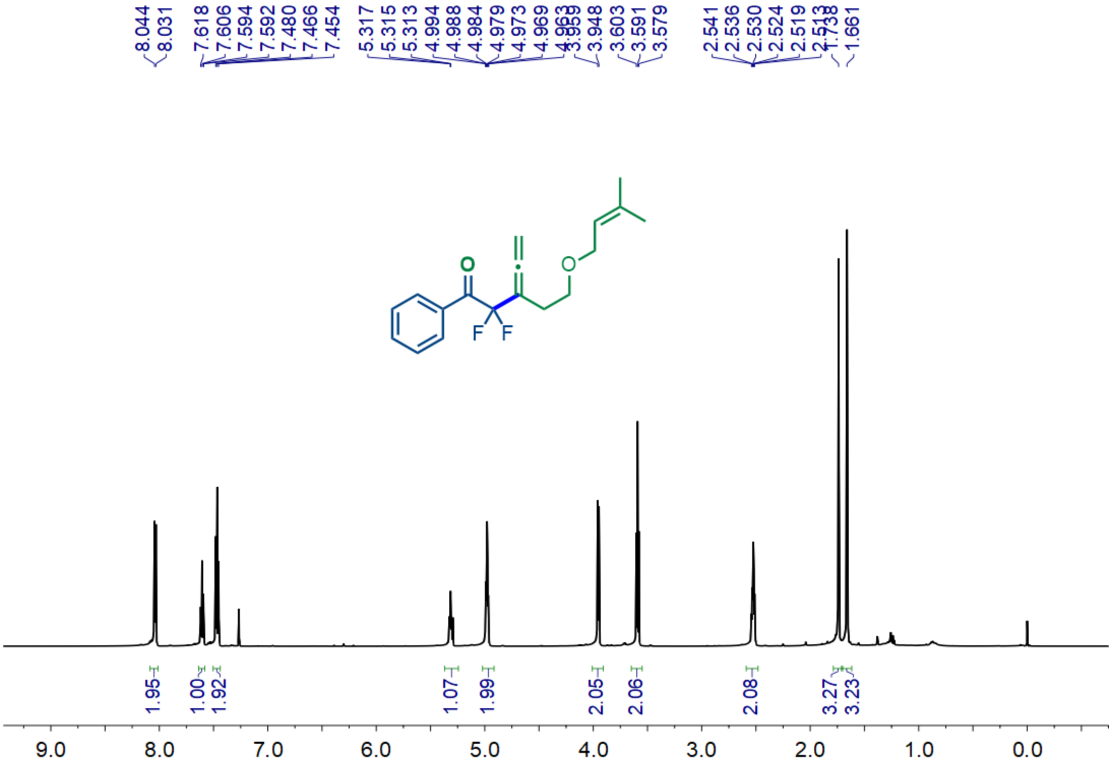

Supplementary Fig. 426 ^{1}H NMR (600 MHz, $CDCl_3$) spectrum of compound **142**

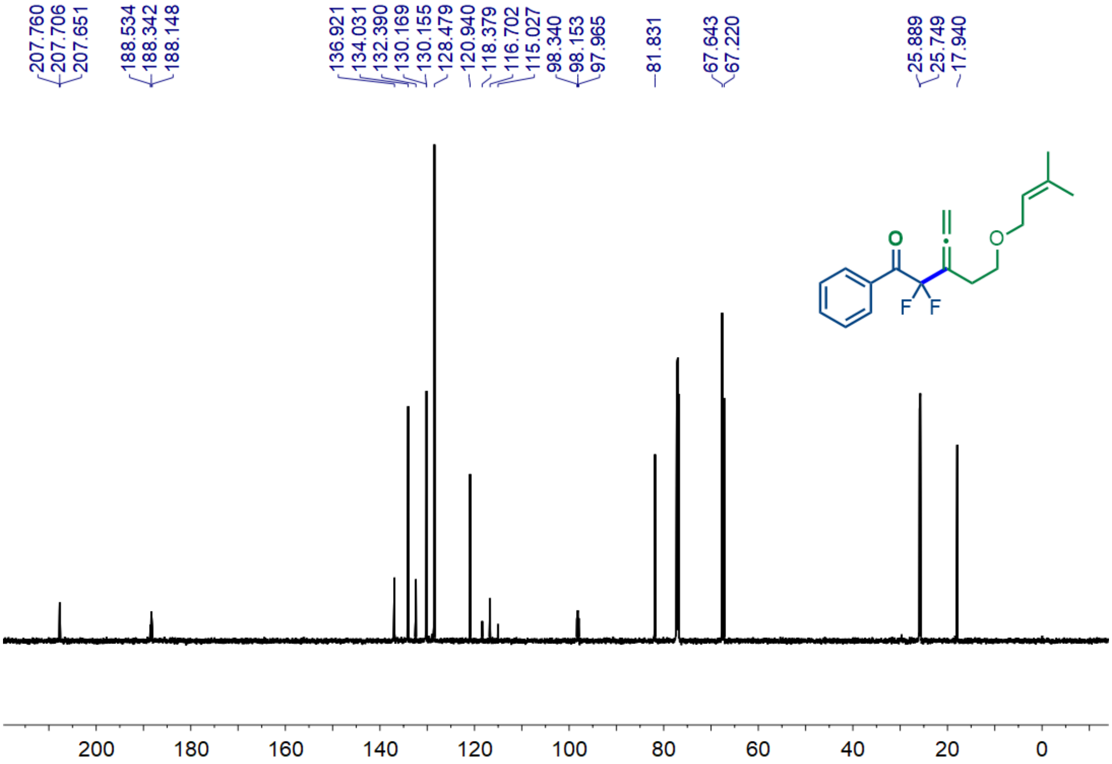

Supplementary Fig. 427 ^{13}C NMR (150 MHz, $CDCl_3$) spectrum of compound **142**

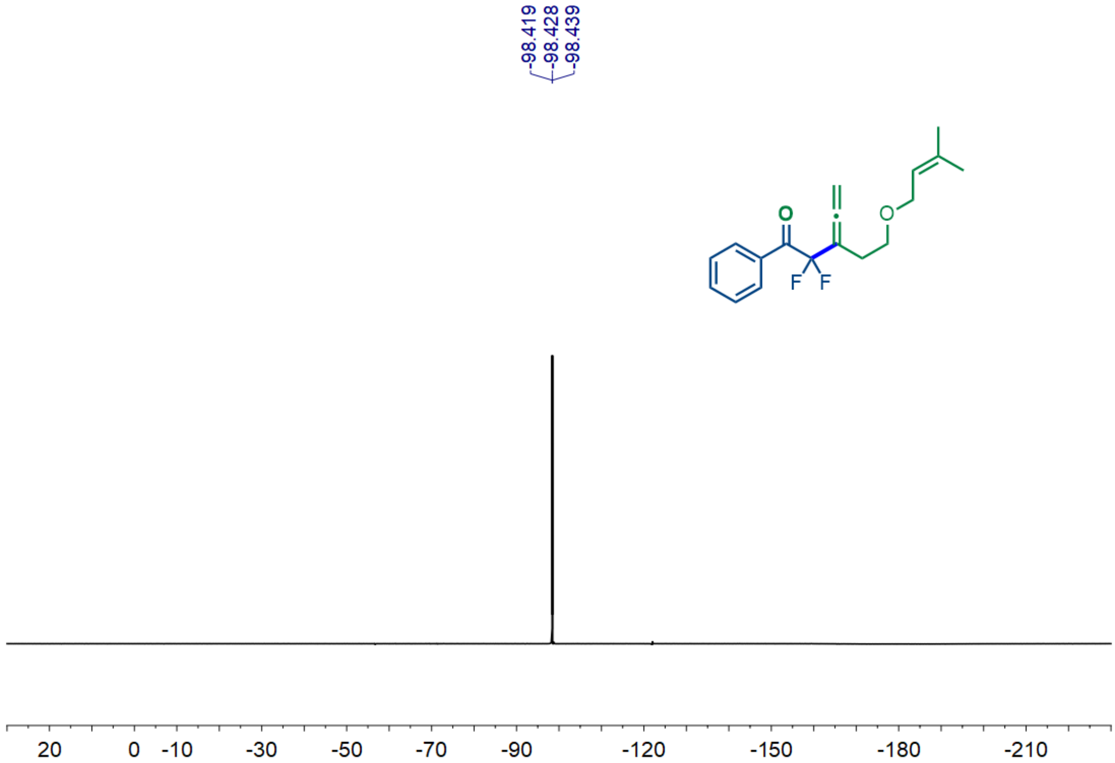

Supplementary Fig. 428 ^{19}F NMR (564 MHz, $CDCl_3$) spectrum of compound **142**

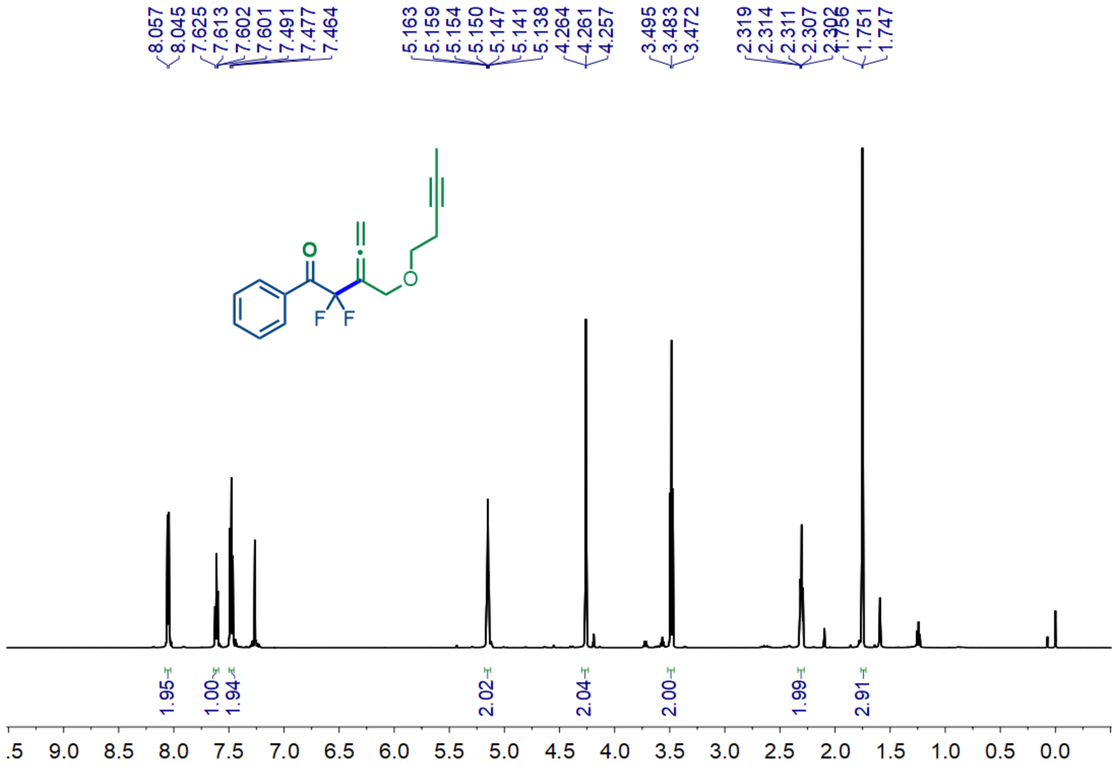

Supplementary Fig. 429 ^{1}H NMR (600 MHz, $CDCl_3$) spectrum of compound **143**

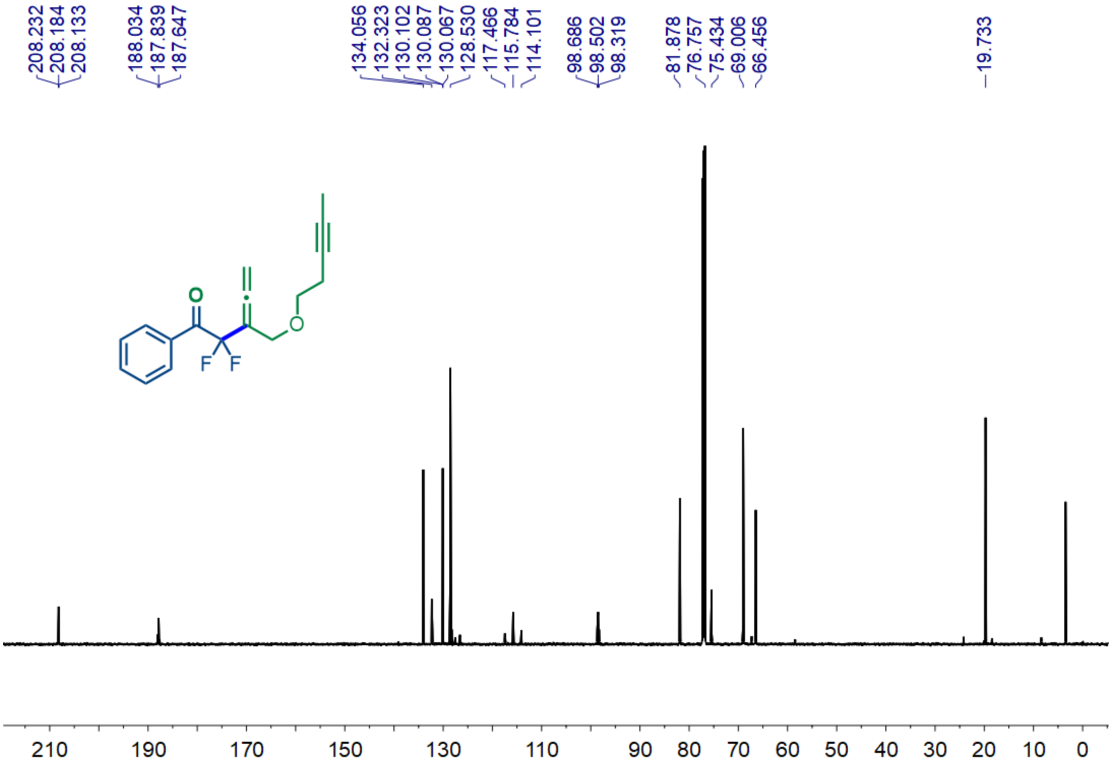

Supplementary Fig. 430 ^{13}C NMR (150 MHz, $CDCl_3$) spectrum of compound **143**

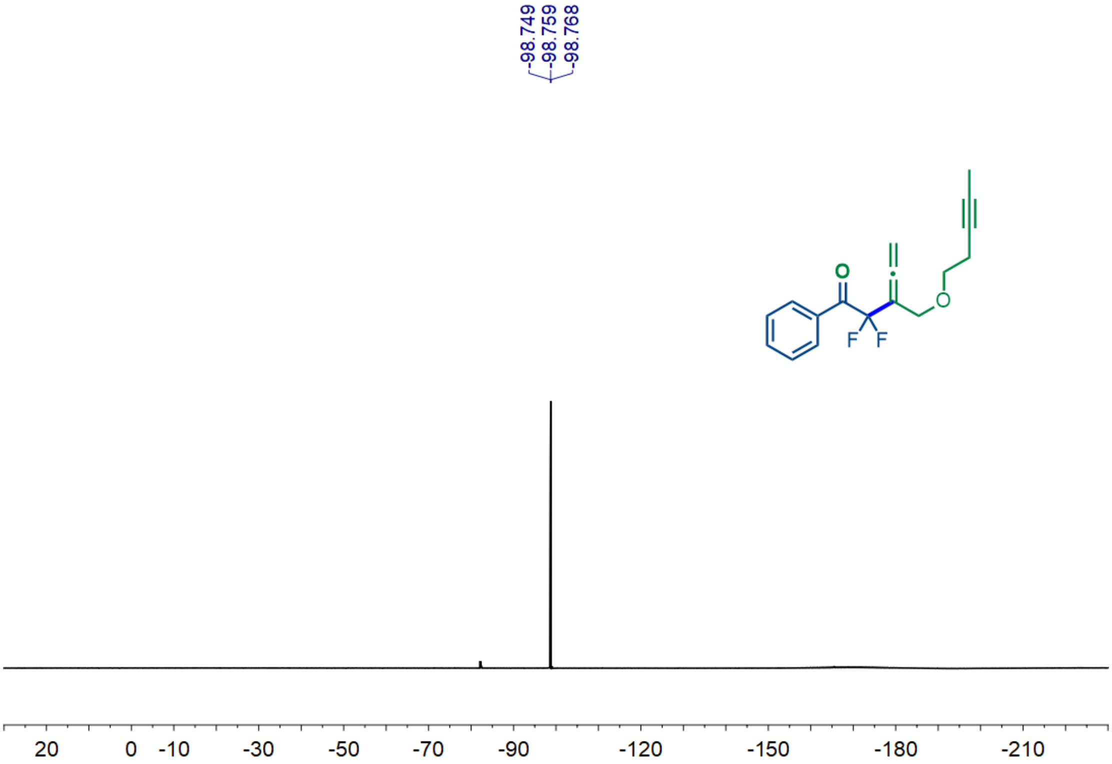

Supplementary Fig. 431 ^{19}F NMR (564 MHz, $CDCl_3$) spectrum of compound **143**

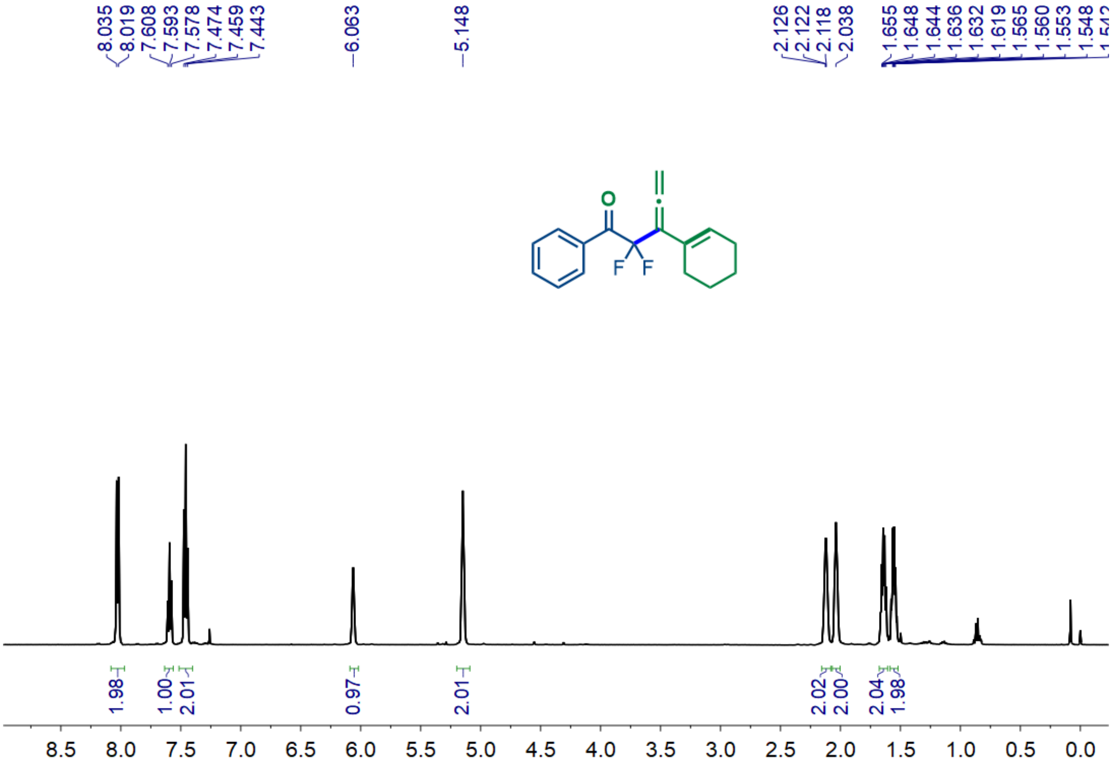

Supplementary Fig. 432 ^{1}H NMR (500 MHz, $CDCl_3$) spectrum of compound **144**

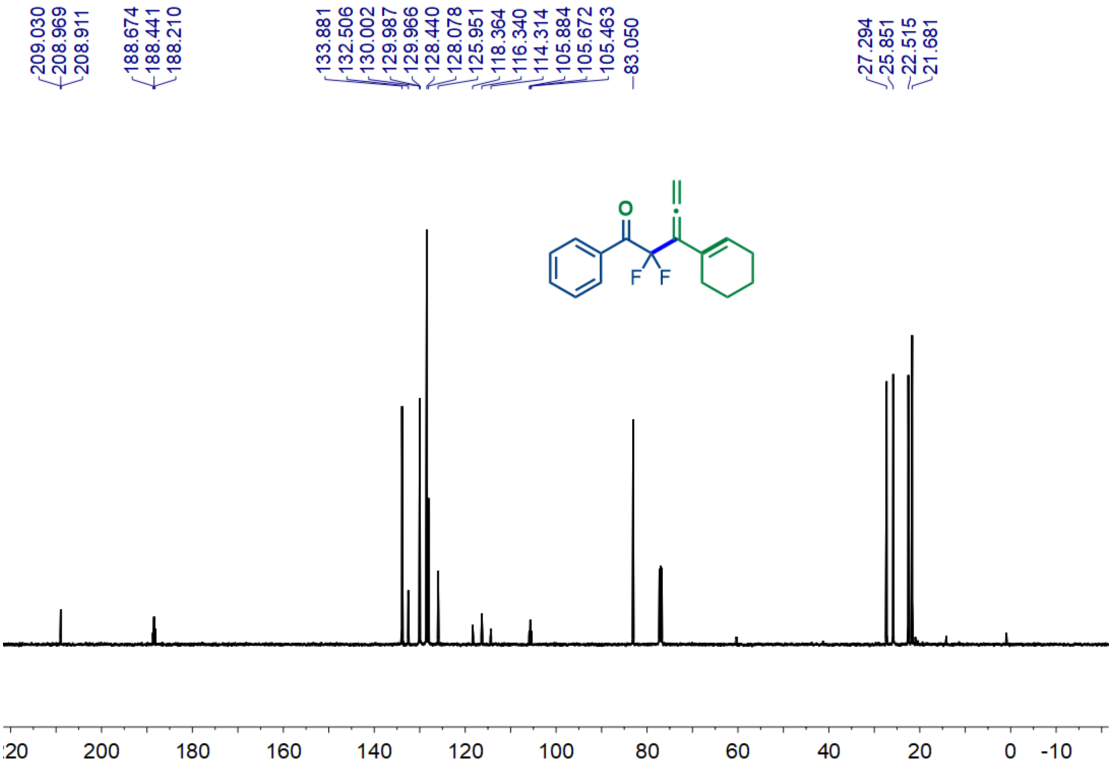

Supplementary Fig. 433 ^{13}C NMR (125 MHz, $CDCl_3$) spectrum of compound **144**

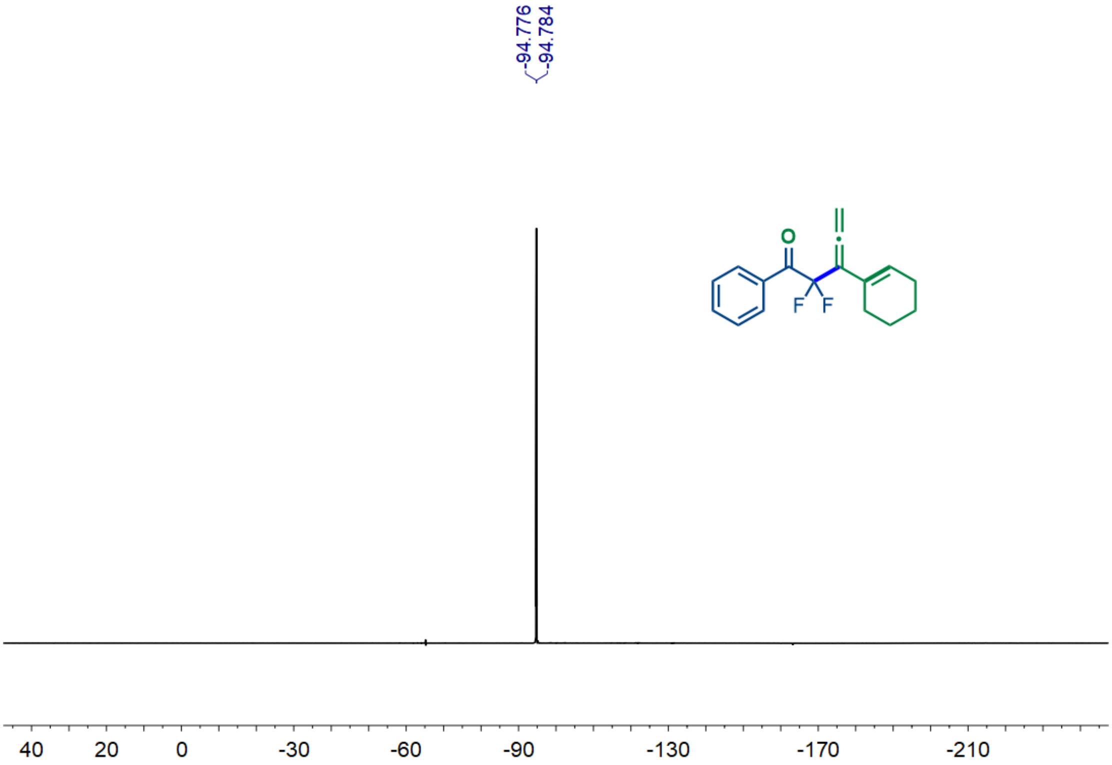

Supplementary Fig. 434 ^{19}F NMR (470 MHz, $CDCl_3$) spectrum of compound **144**

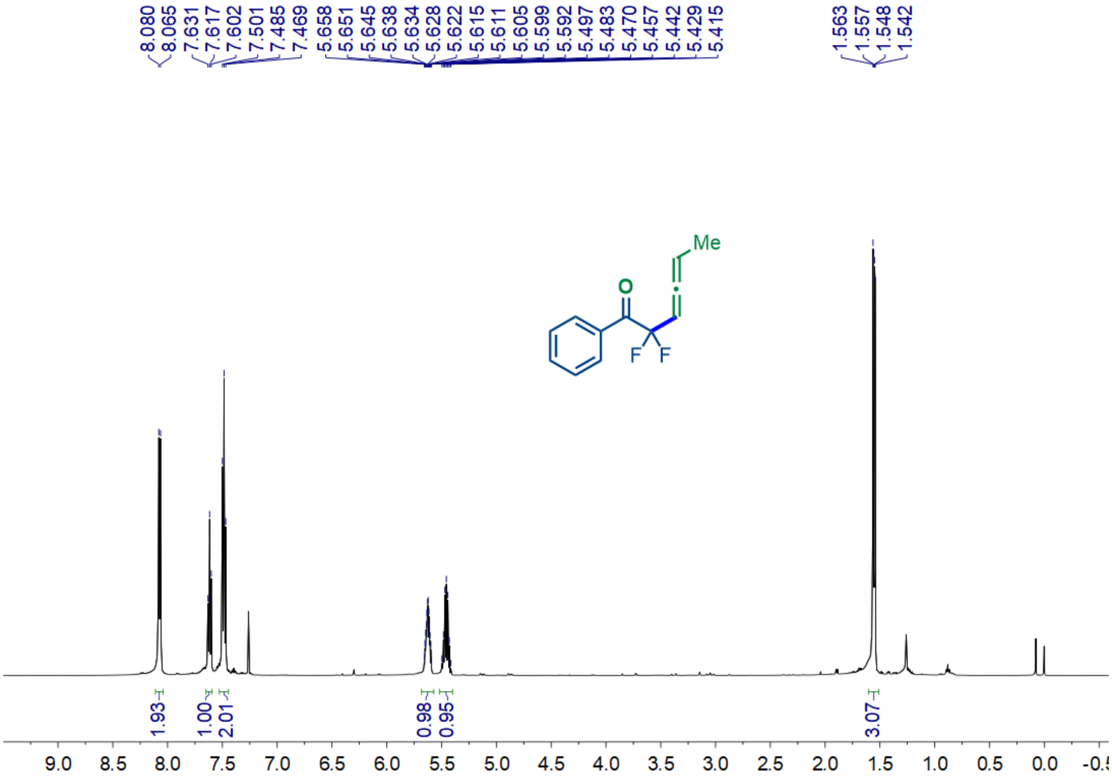

Supplementary Fig. 435 ^{1}H NMR (500 MHz, $CDCl_3$) spectrum of compound **145**

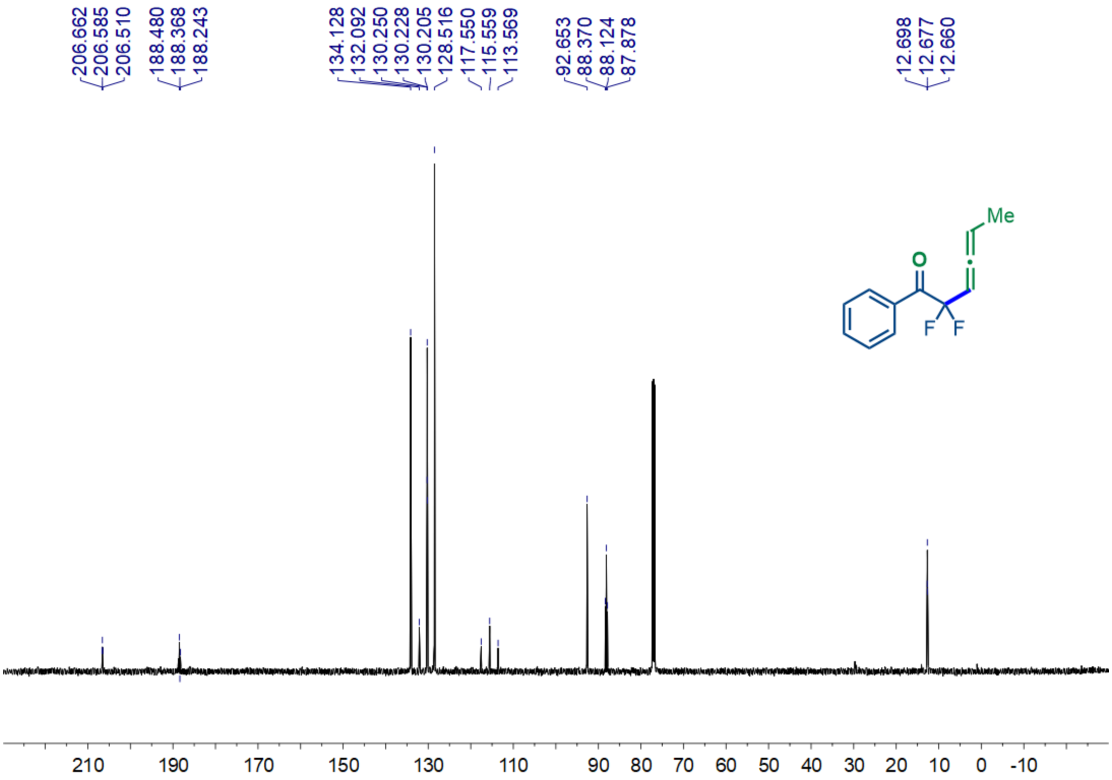

Supplementary Fig. 436 ^{13}C NMR (125 MHz, $CDCl_3$) spectrum of compound **145**

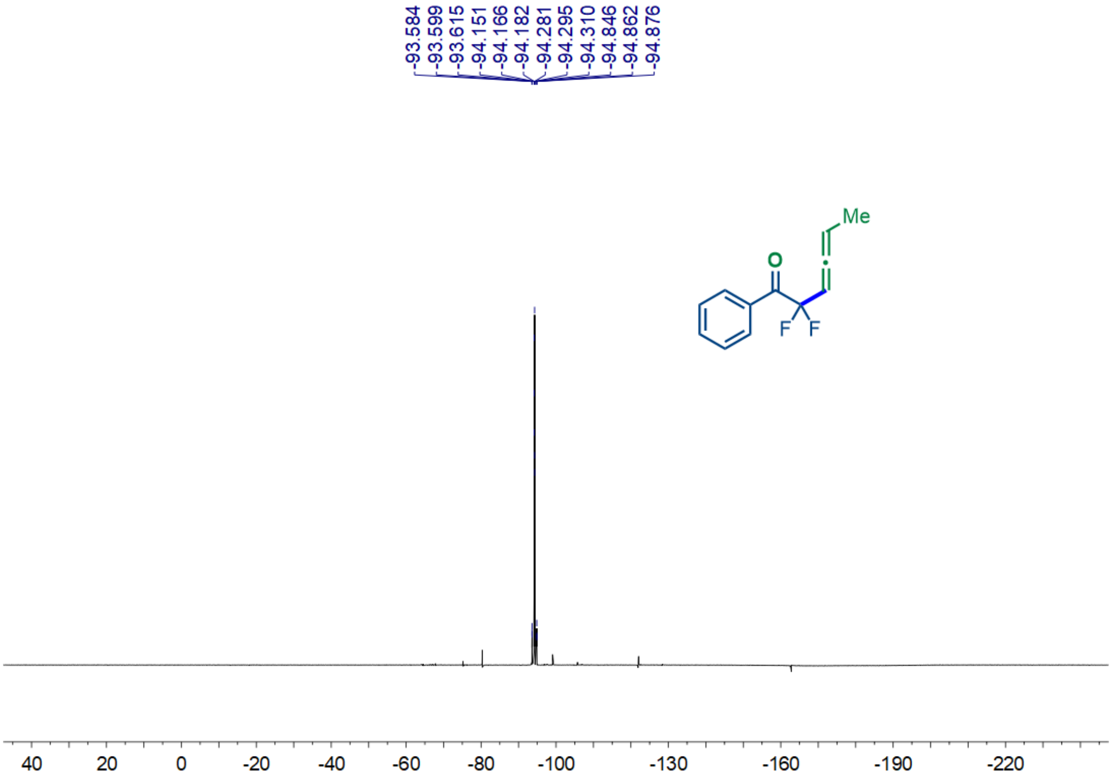

Supplementary Fig. 437 ^{19}F NMR (470 MHz, $CDCl_3$) spectrum of compound **145**

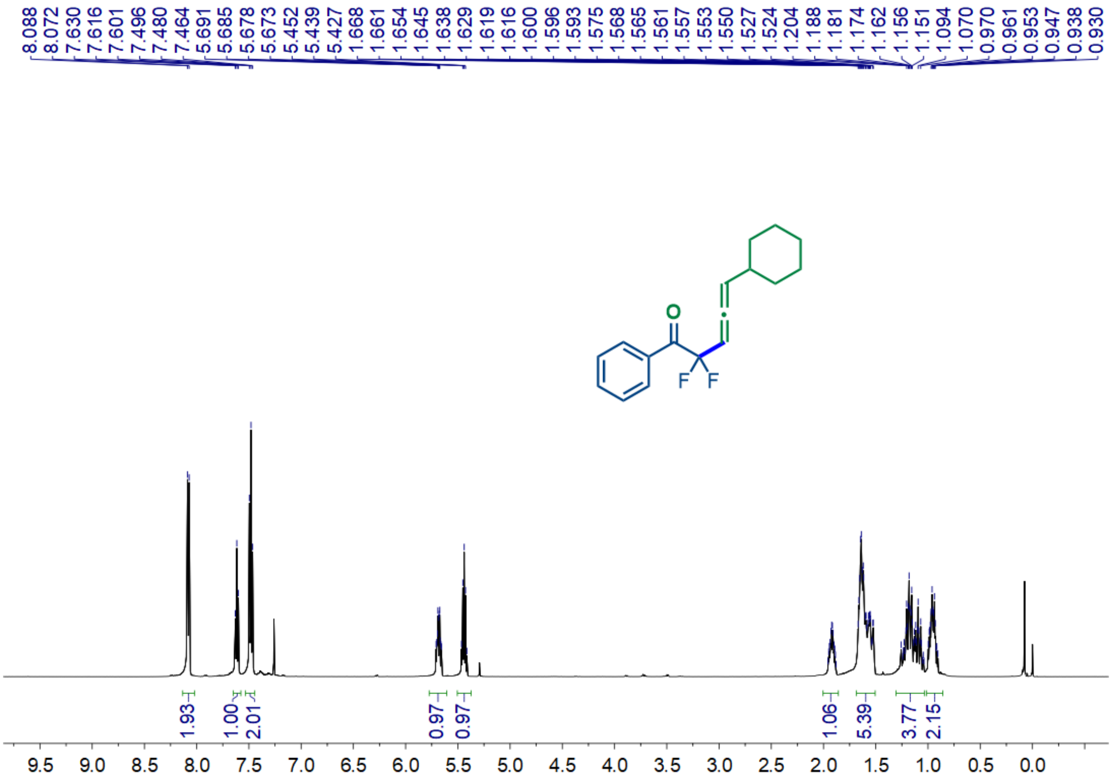

Supplementary Fig. 438 ^{1}H NMR (500 MHz, $CDCl_3$) spectrum of compound **146**

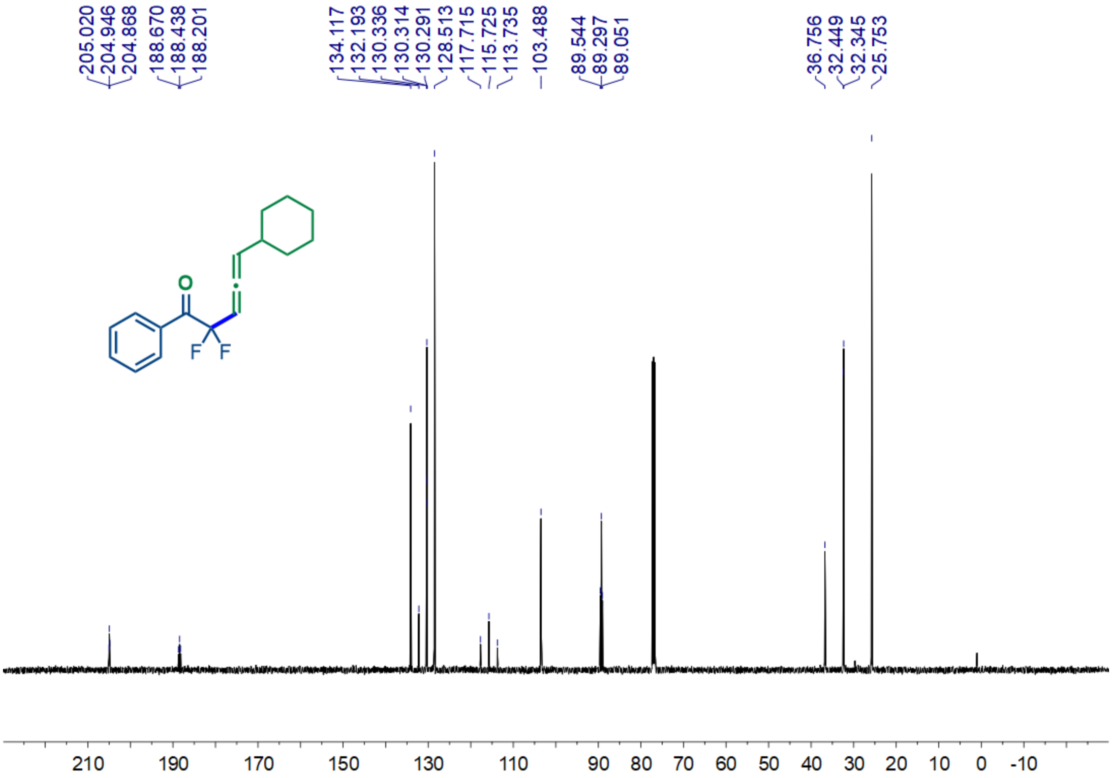

Supplementary Fig. 439 ^{13}C NMR (125 MHz, $CDCl_3$) spectrum of compound **146**

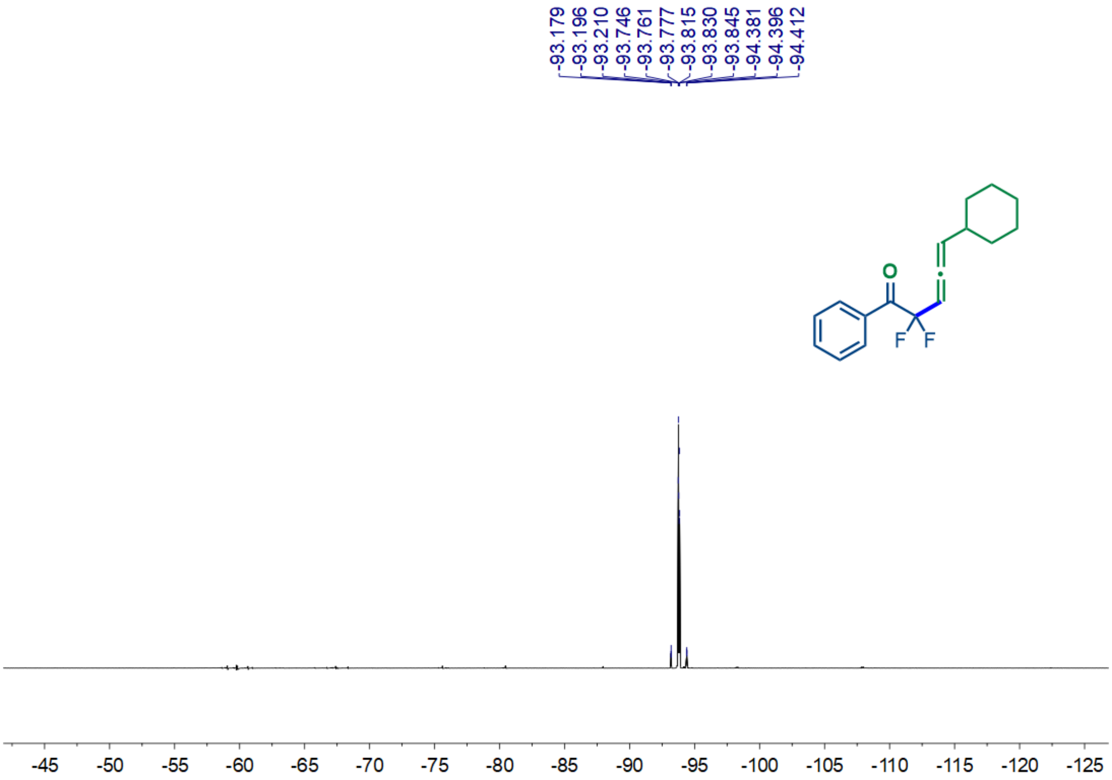

Supplementary Fig. 440 ^{19}F NMR (470 MHz, $CDCl_3$) spectrum of compound **146**

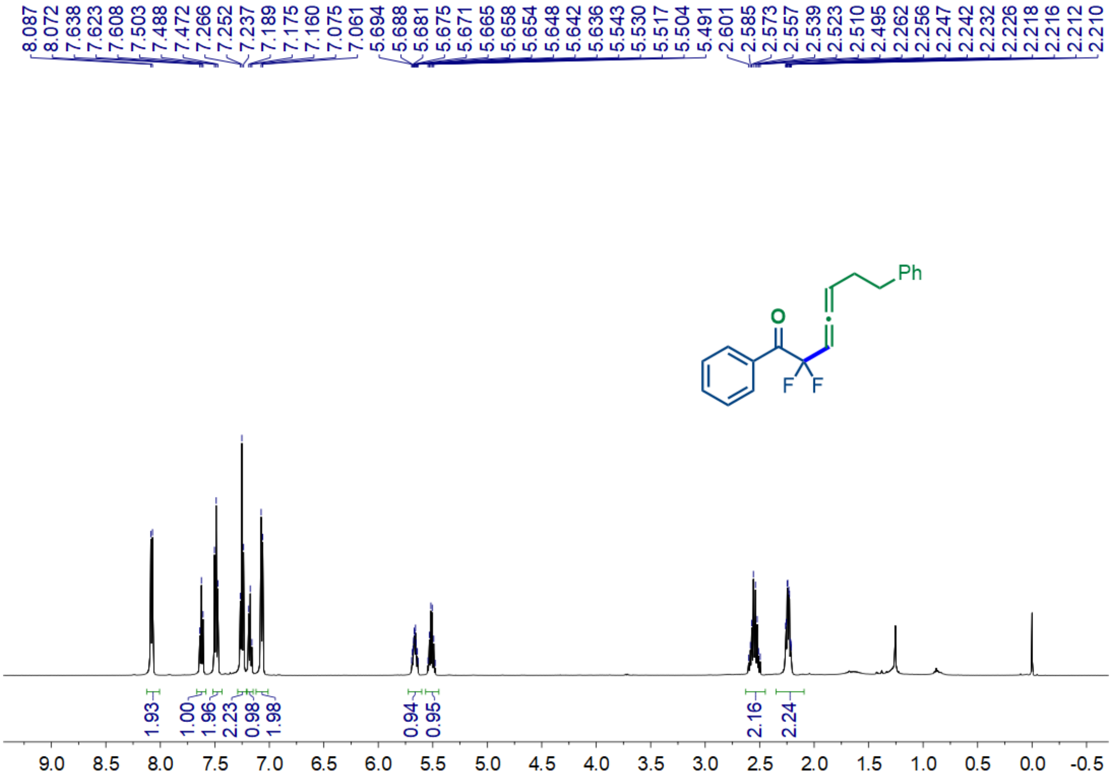

Supplementary Fig. 441 ^{1}H NMR (500 MHz, $CDCl_3$) spectrum of compound **147**

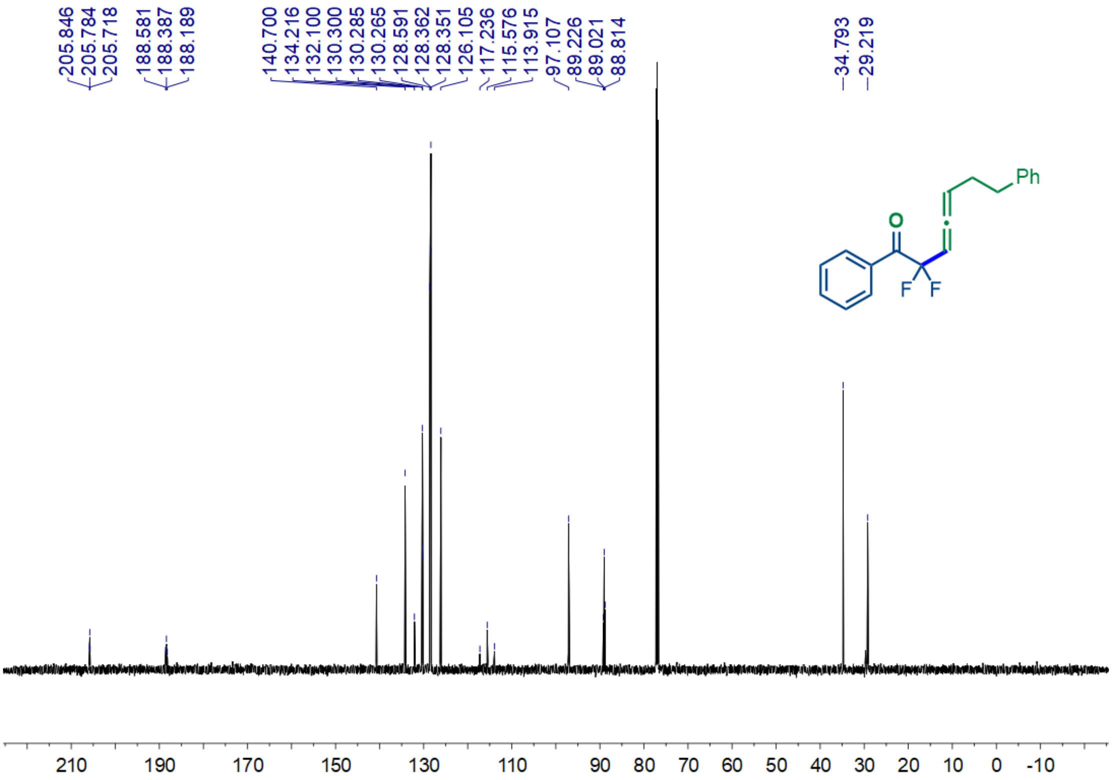

Supplementary Fig. 442 ^{13}C NMR (125 MHz, $CDCl_3$) spectrum of compound **147**

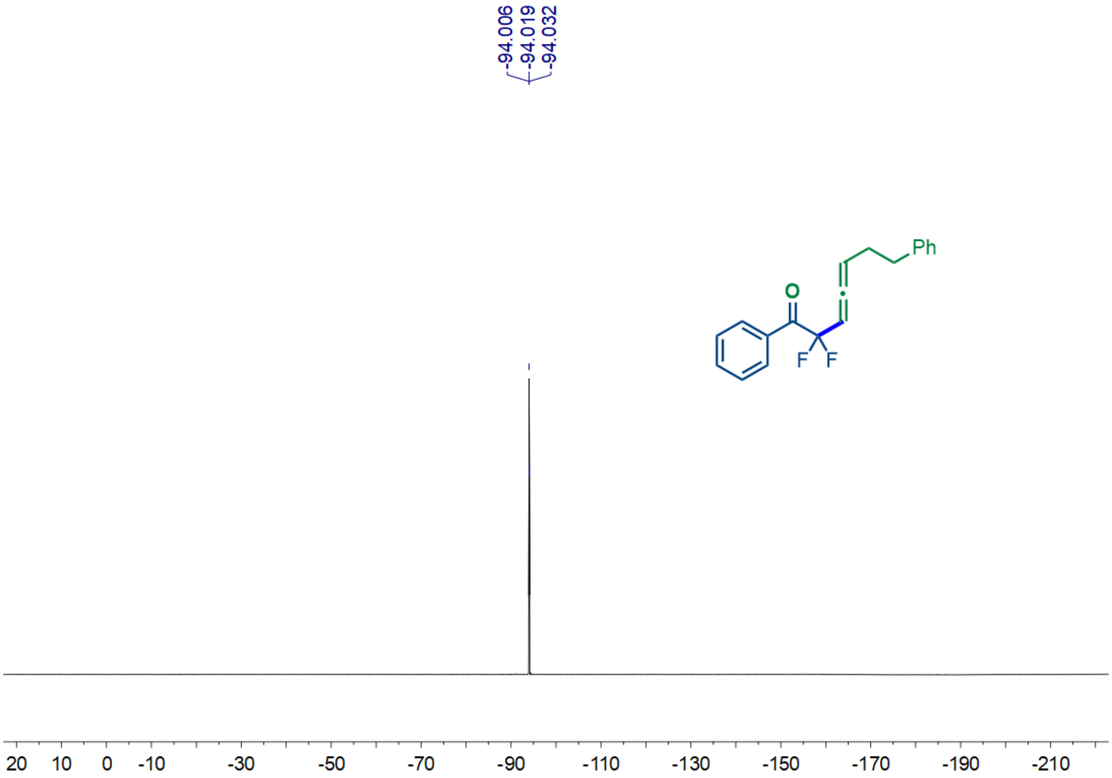

Supplementary Fig. 443 ^{19}F NMR (470 MHz, $CDCl_3$) spectrum of compound **147**

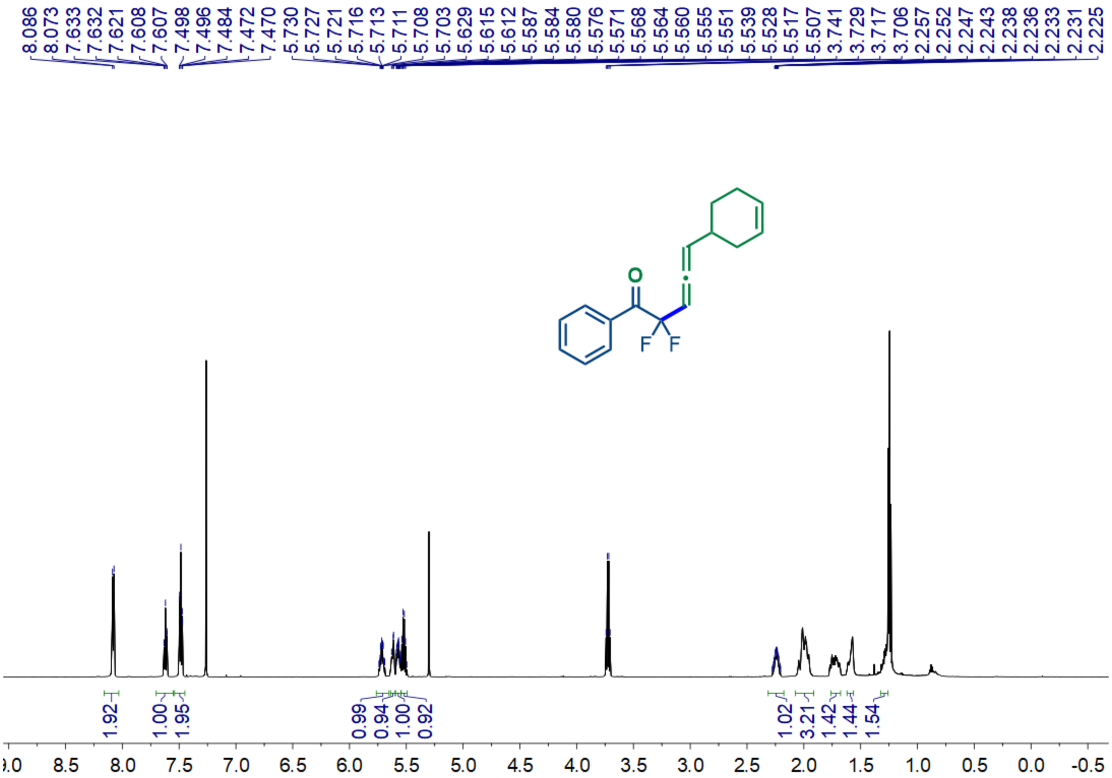

Supplementary Fig. 444 ^{1}H NMR (600 MHz, $CDCl_3$) spectrum of compound **148**

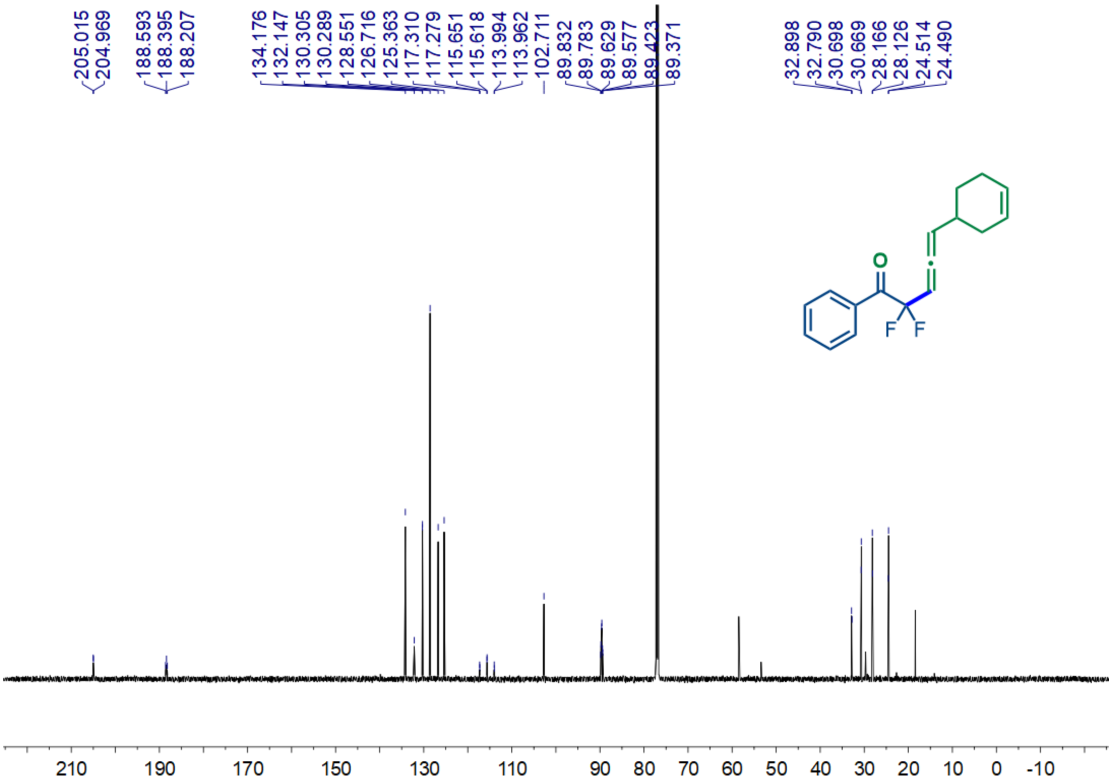

Supplementary Fig. 445 ^{13}C NMR (150 MHz, $CDCl_3$) spectrum of compound **148**

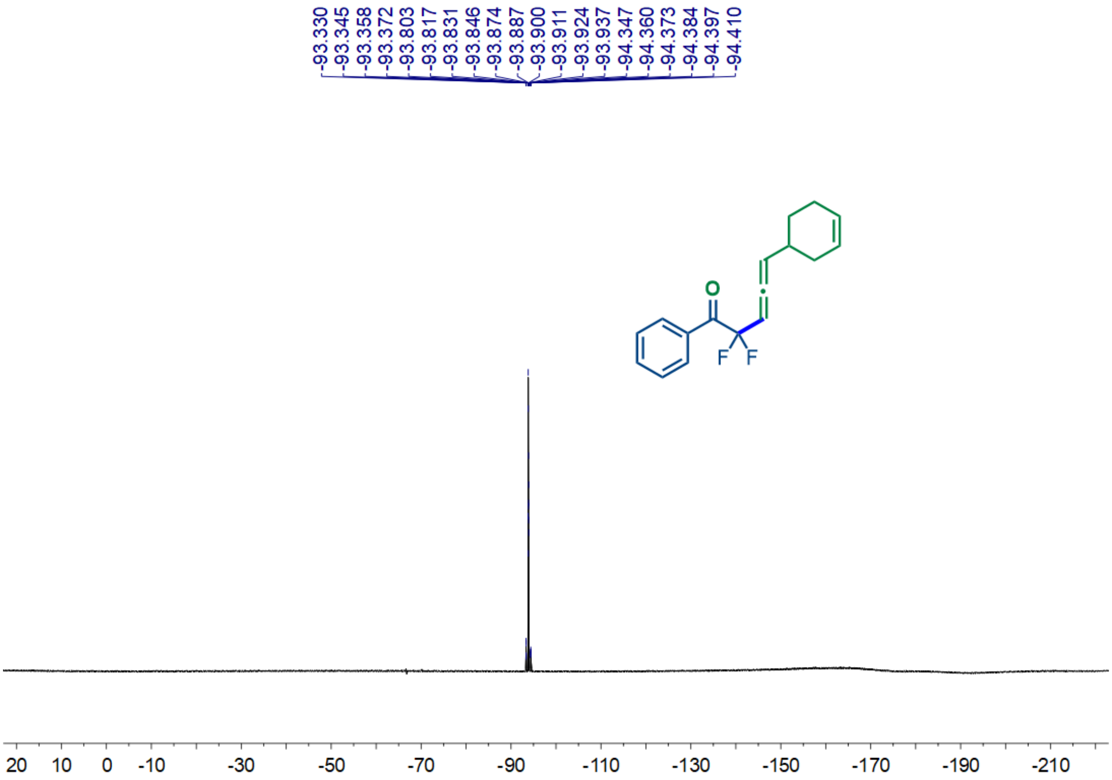

Supplementary Fig. 446 ^{19}F NMR (564 MHz, $CDCl_3$) spectrum of compound **148**

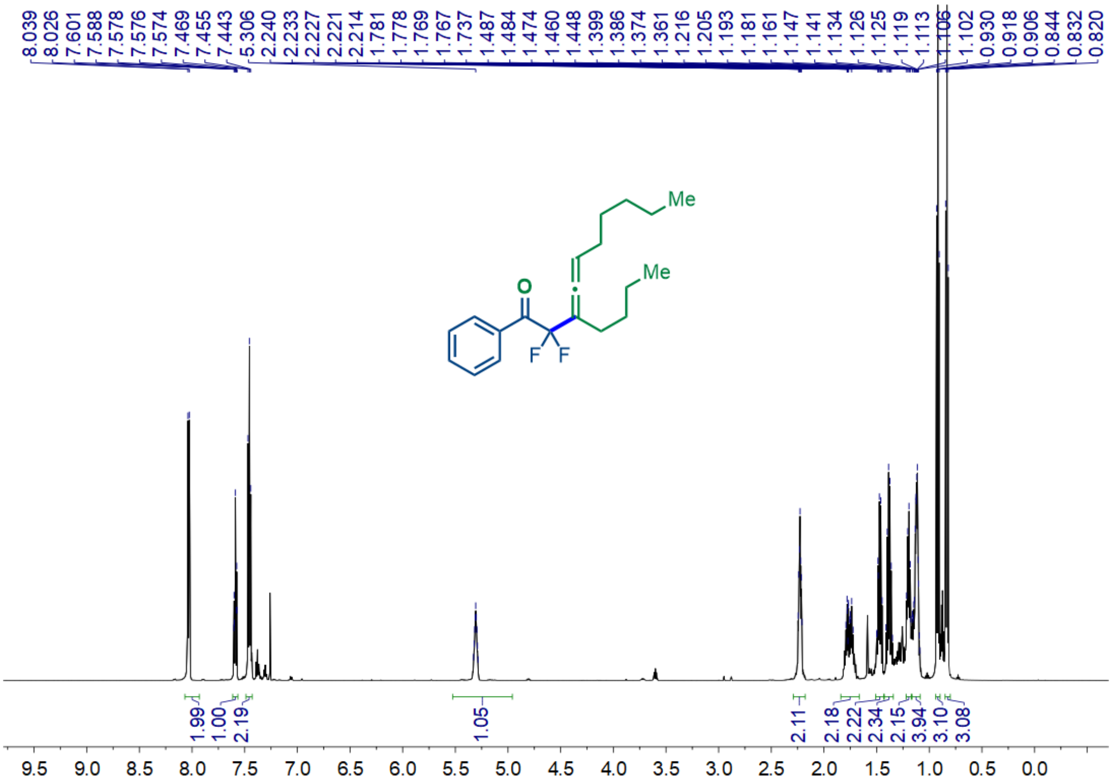

Supplementary Fig. 447 ^{1}H NMR (600 MHz, $CDCl_3$) spectrum of compound **149**

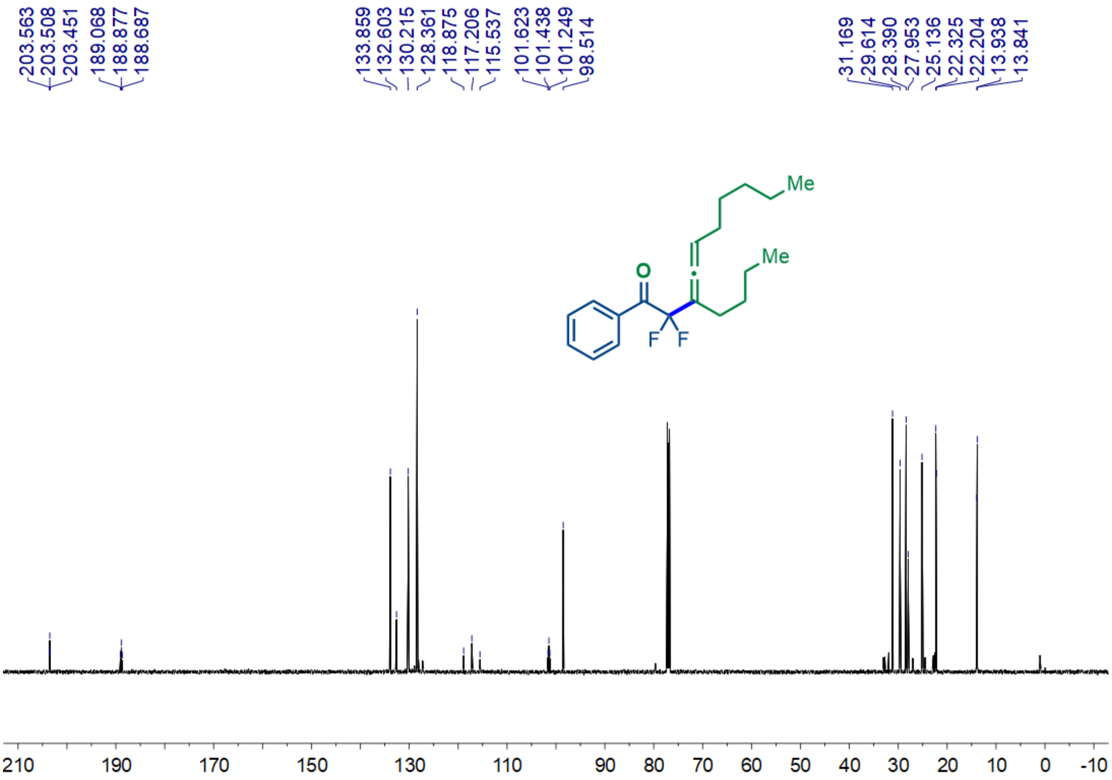

Supplementary Fig. 448 ^{13}C NMR (150 MHz, $CDCl_3$) spectrum of compound **149**

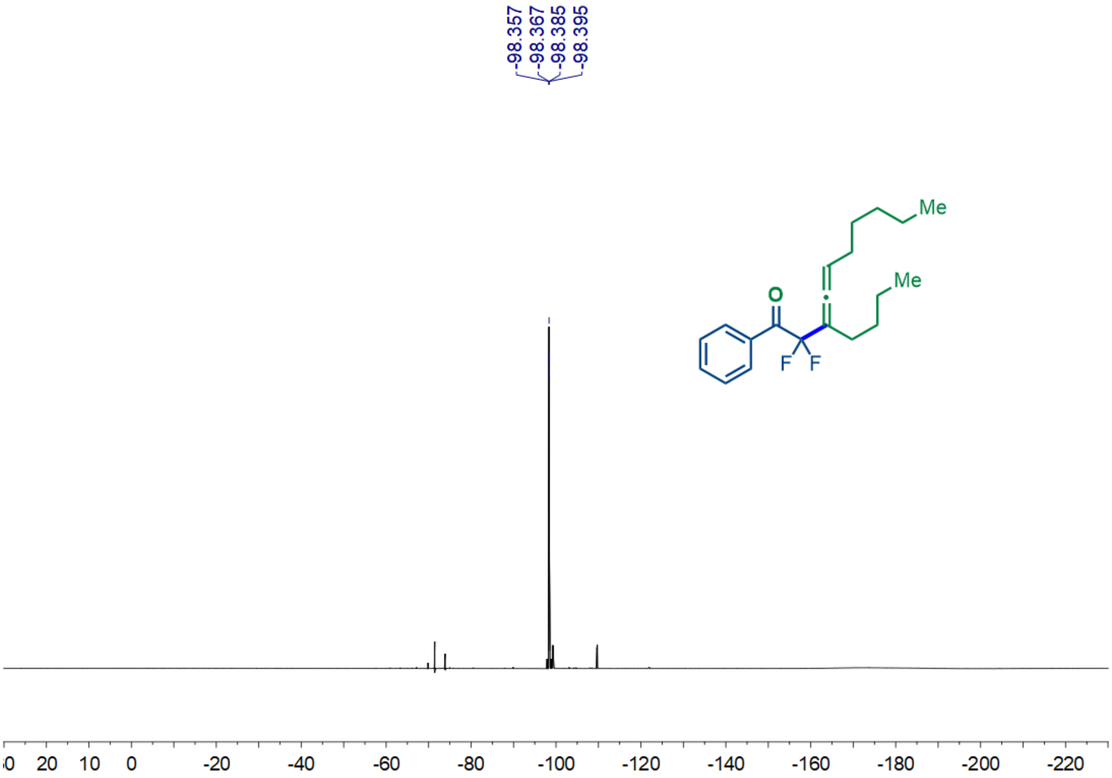

Supplementary Fig. 449 ^{19}F NMR (564 MHz, $CDCl_3$) spectrum of compound **149**

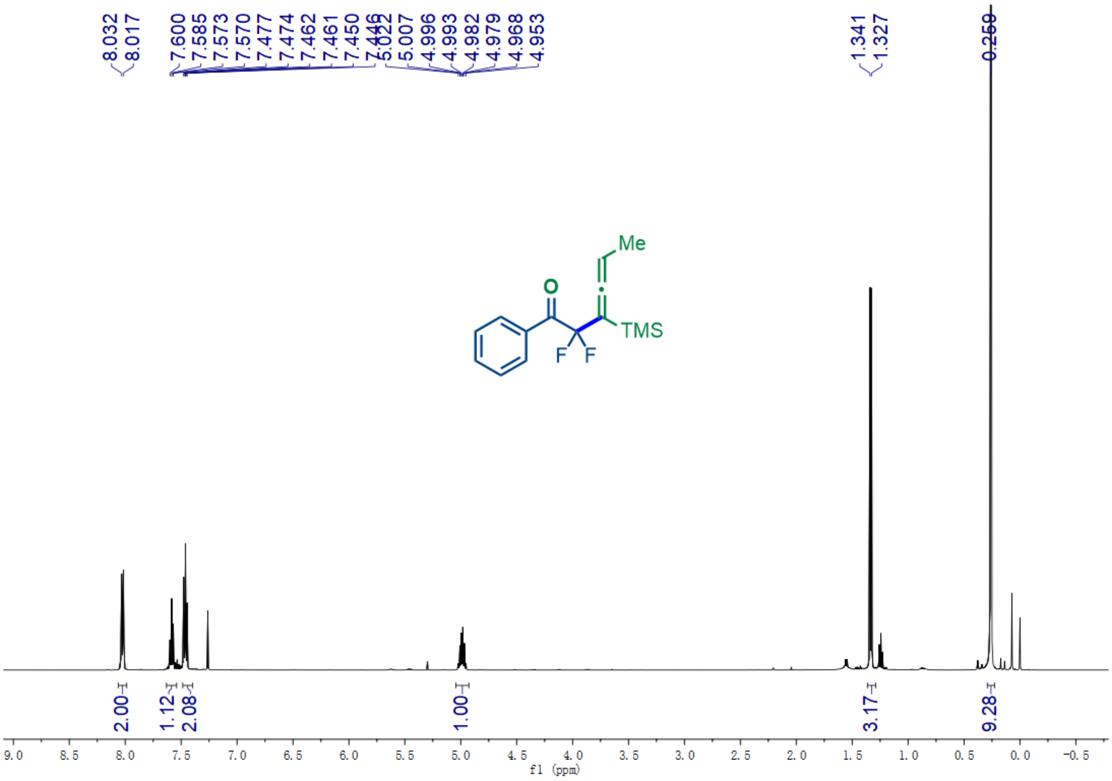

Supplementary Fig. 450 ^{1}H NMR (500 MHz, $CDCl_3$) spectrum of compound **150**

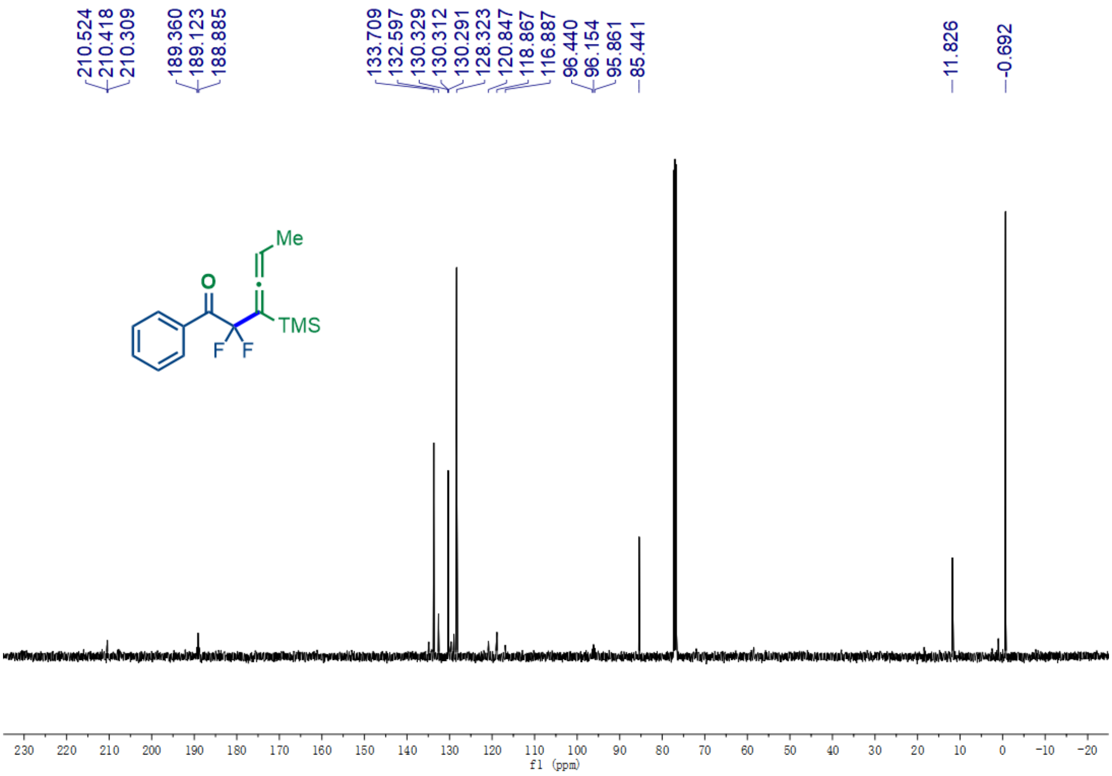

Supplementary Fig. 451 ^{13}C NMR (125 MHz, $CDCl_3$) spectrum of compound **150**

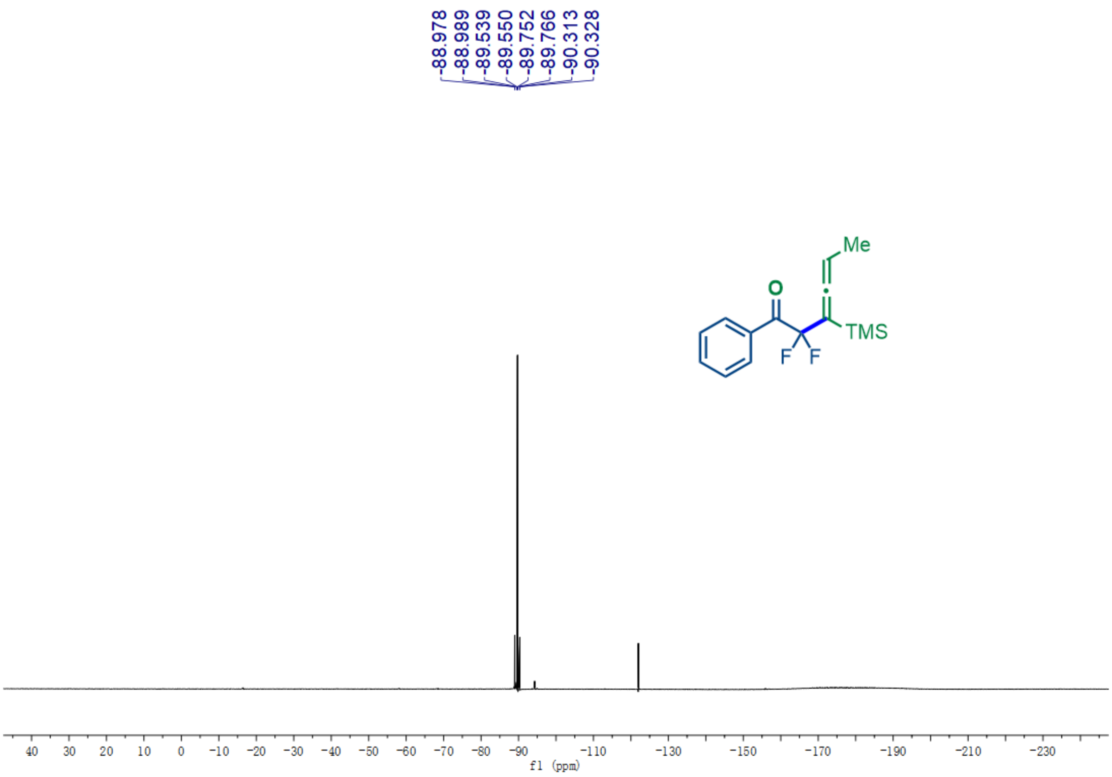

Supplementary Fig. 452 ^{19}F NMR (470 MHz, $CDCl_3$) spectrum of compound **150**

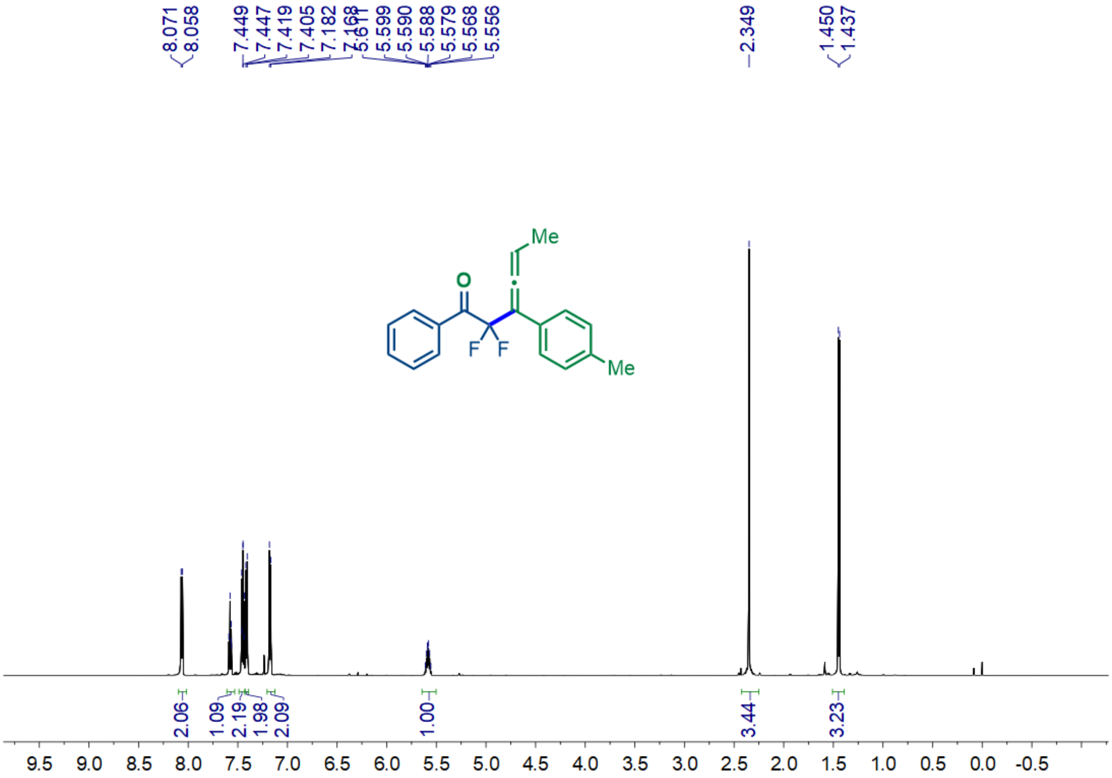

Supplementary Fig. 453 ^{1}H NMR (600 MHz, $CDCl_3$) spectrum of compound **151**

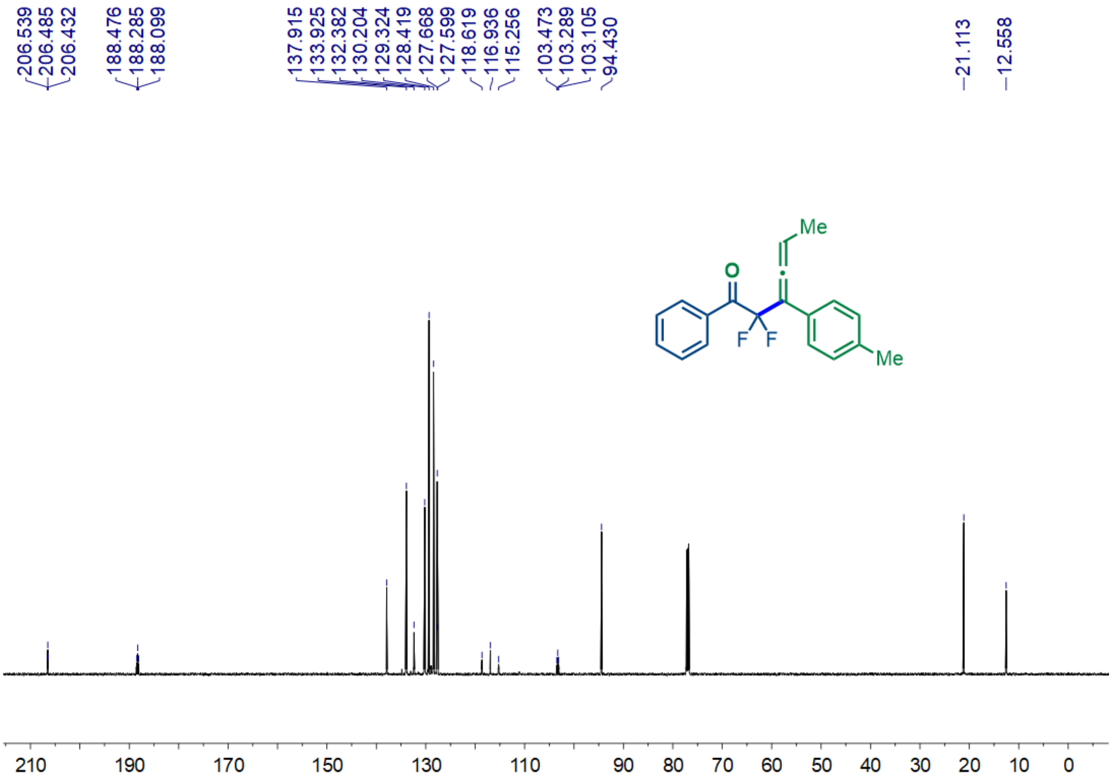

Supplementary Fig. 454 ^{13}C NMR (150 MHz, $CDCl_3$) spectrum of compound **151**

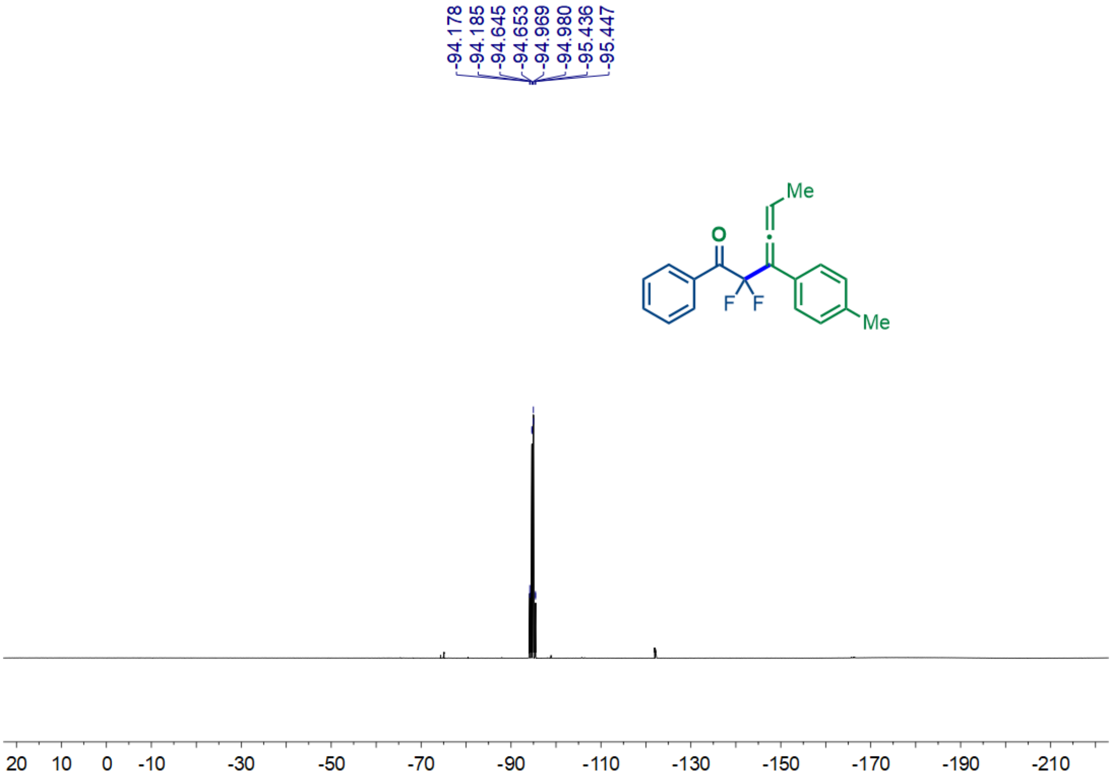

Supplementary Fig. 455 ^{19}F NMR (564 MHz, $CDCl_3$) spectrum of compound **151**

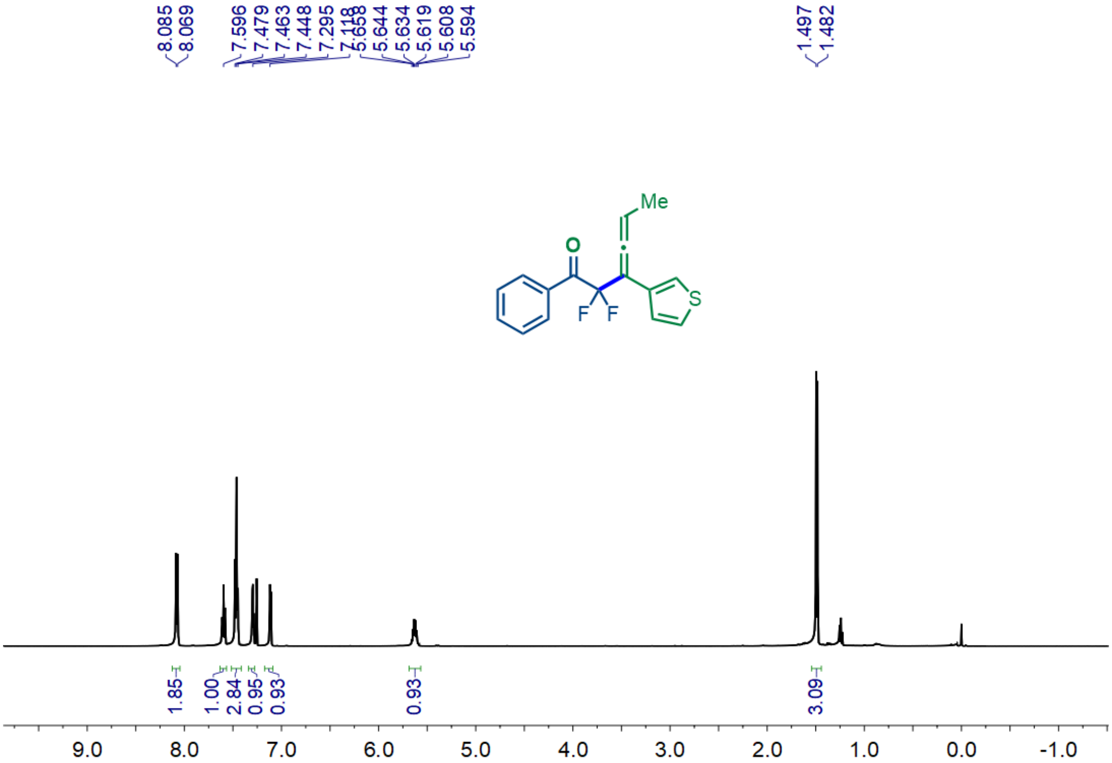

Supplementary Fig. 456 ^{1}H NMR (500 MHz, $CDCl_3$) spectrum of compound **152**

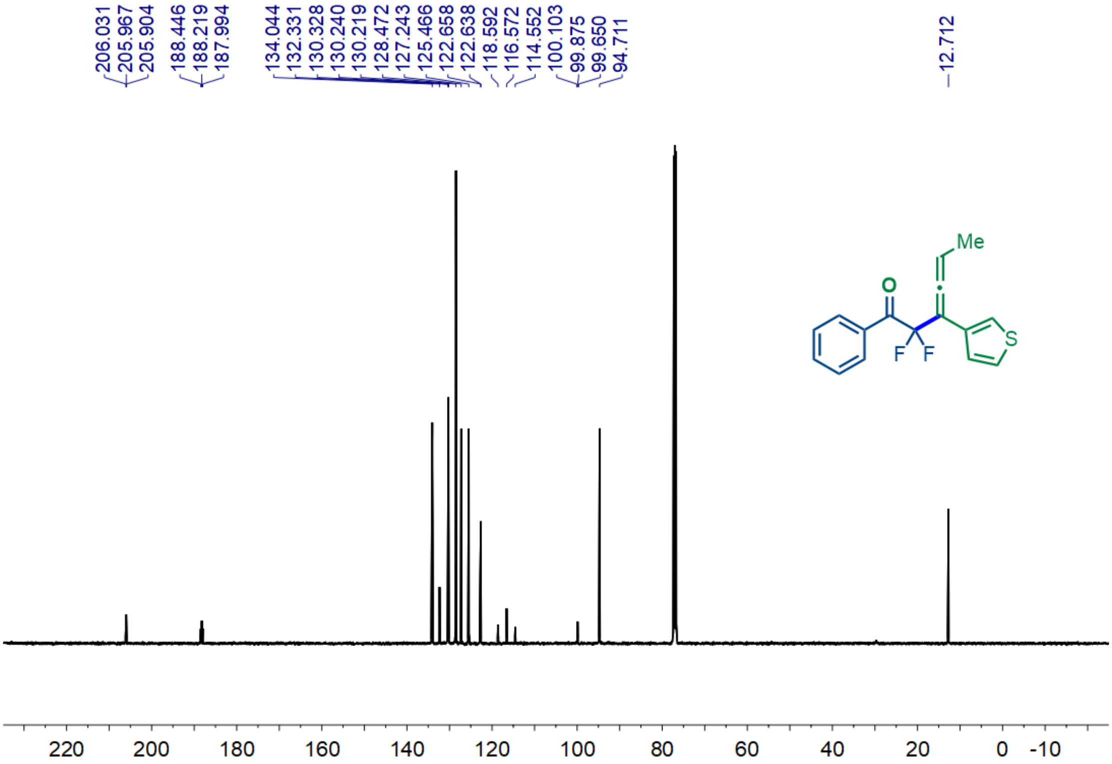

Supplementary Fig. 457 ^{13}C NMR (125 MHz, $CDCl_3$) spectrum of compound **152**

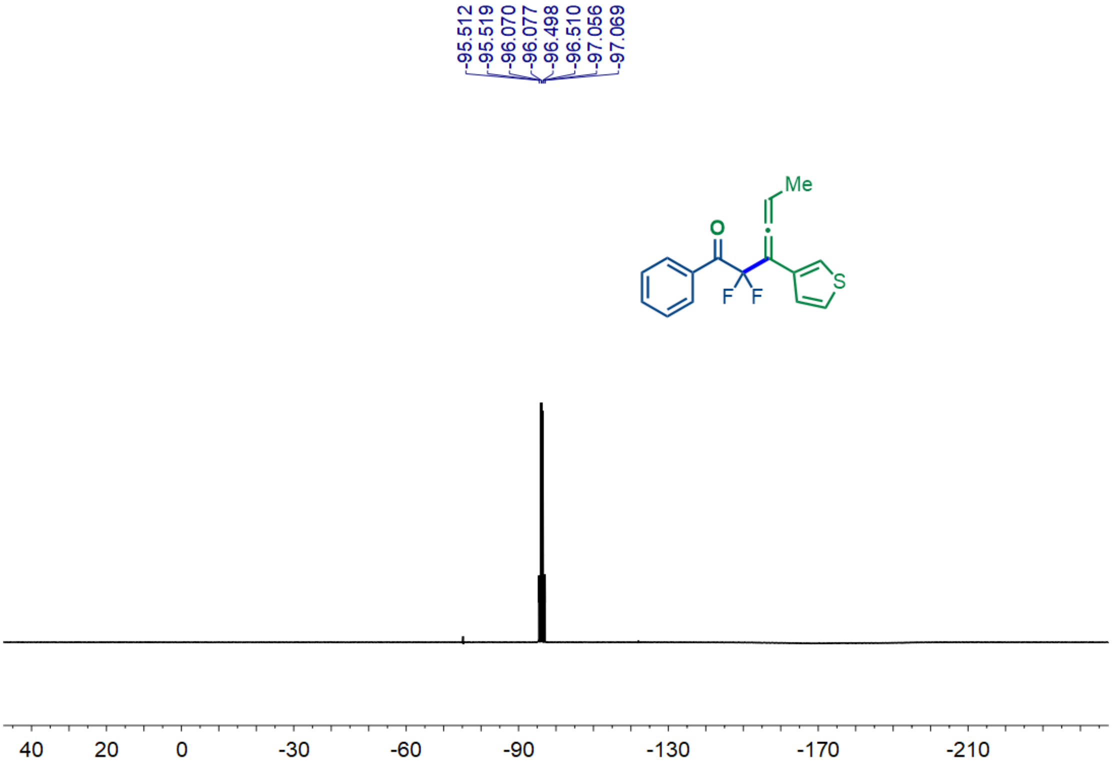

Supplementary Fig. 458 ^{19}F NMR (470 MHz, $CDCl_3$) spectrum of compound **152**

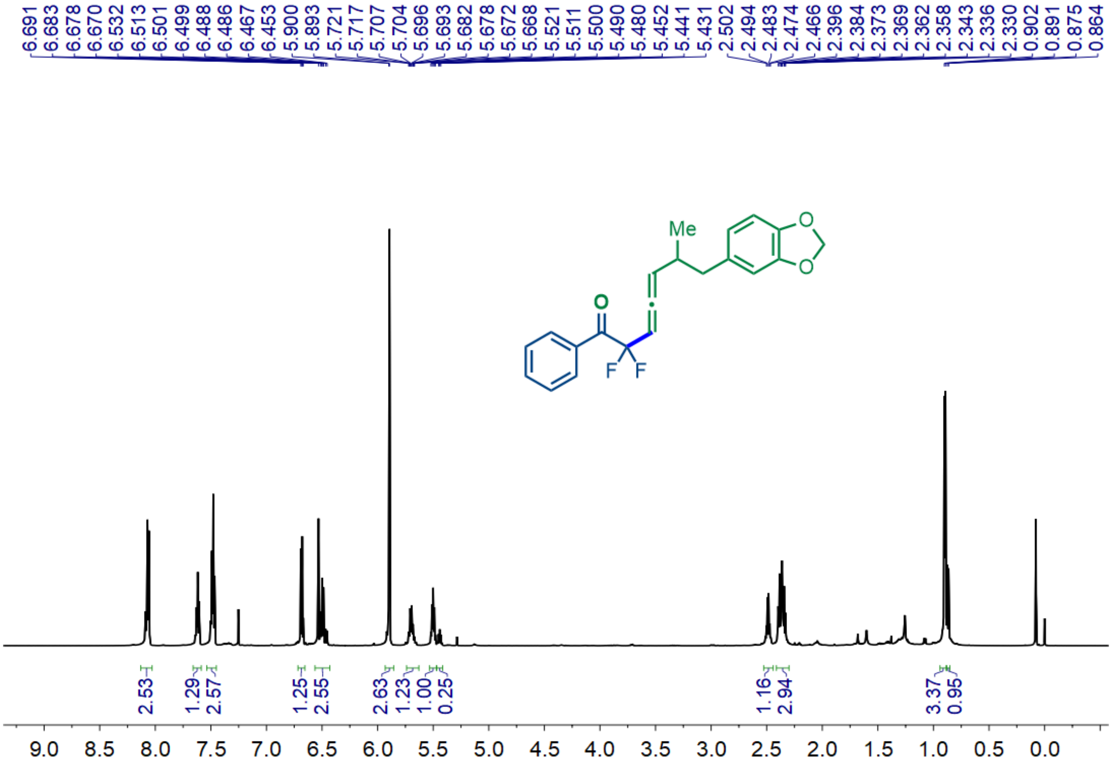

Supplementary Fig. 459 ^{1}H NMR (600 MHz, $CDCl_3$) spectrum of compound **153**

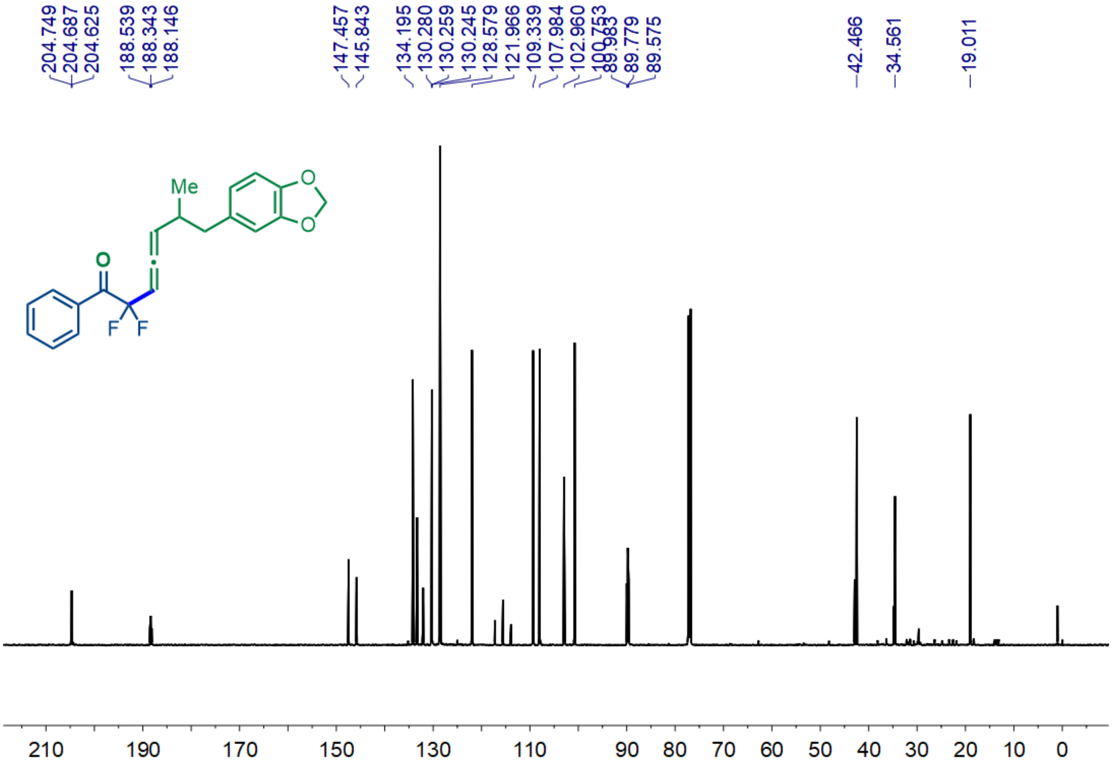

Supplementary Fig. 460 ^{13}C NMR (150 MHz, $CDCl_3$) spectrum of compound **153**

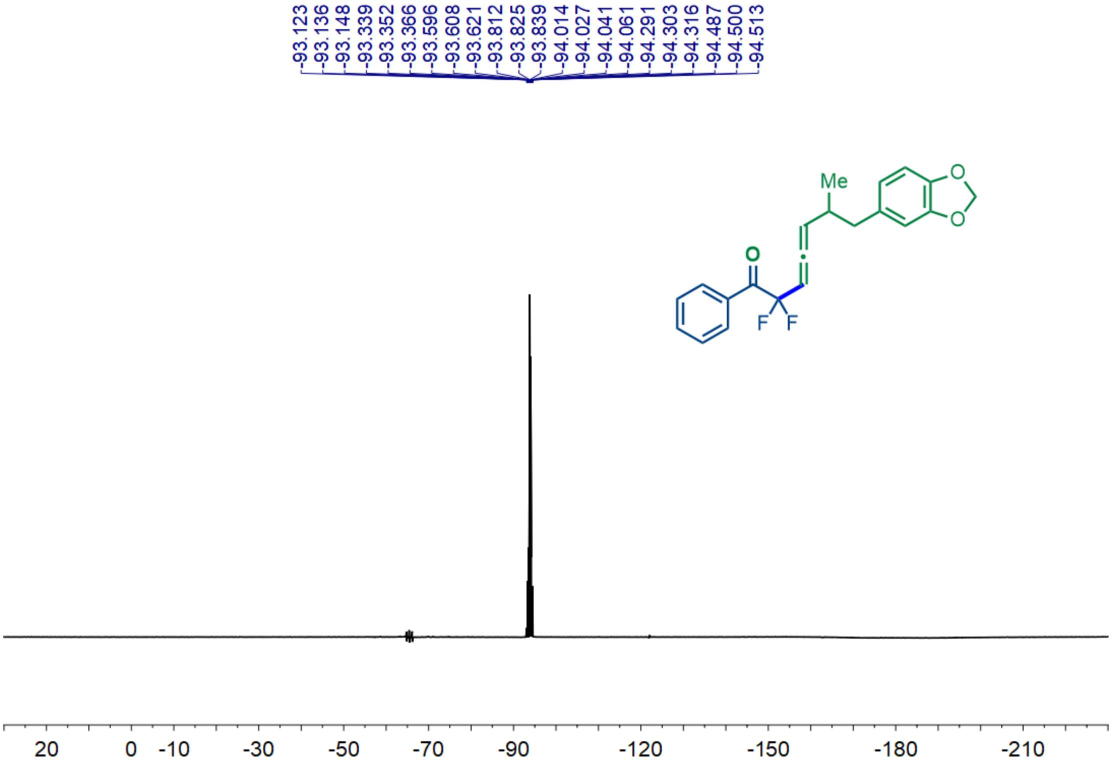

Supplementary Fig. 461 ^{19}F NMR (564 MHz, $CDCl_3$) spectrum of compound **153**

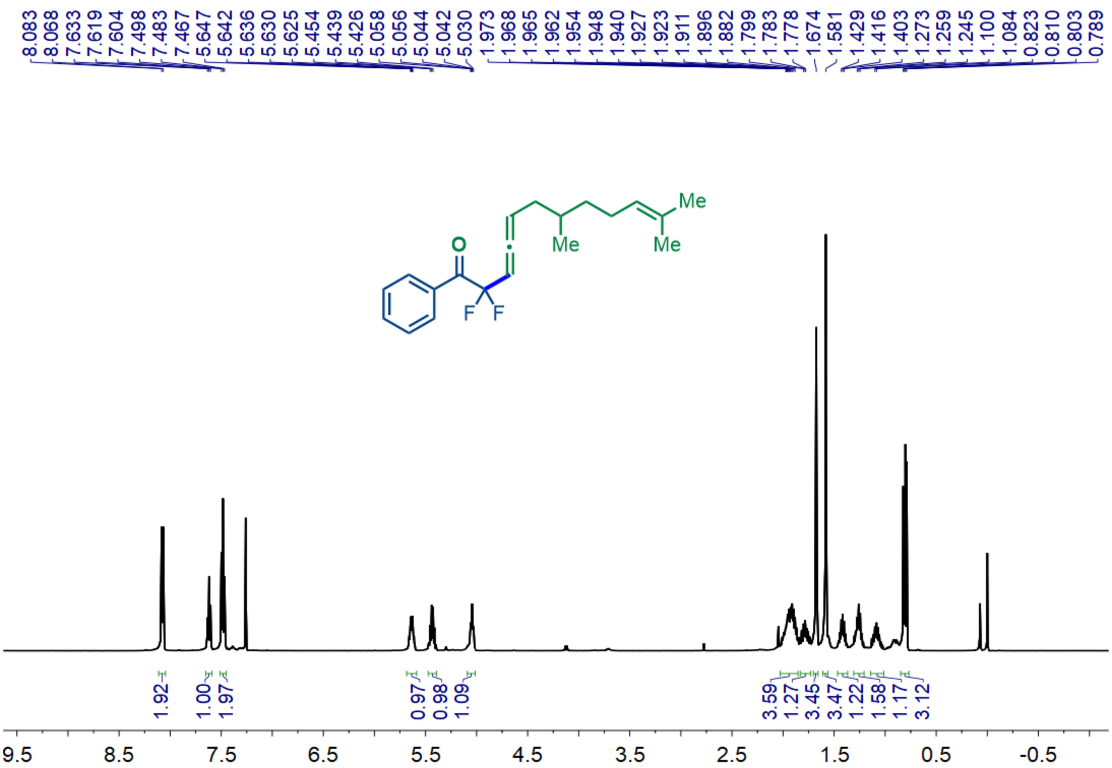

Supplementary Fig. 462 ^{1}H NMR (500 MHz, $CDCl_3$) spectrum of compound **154**

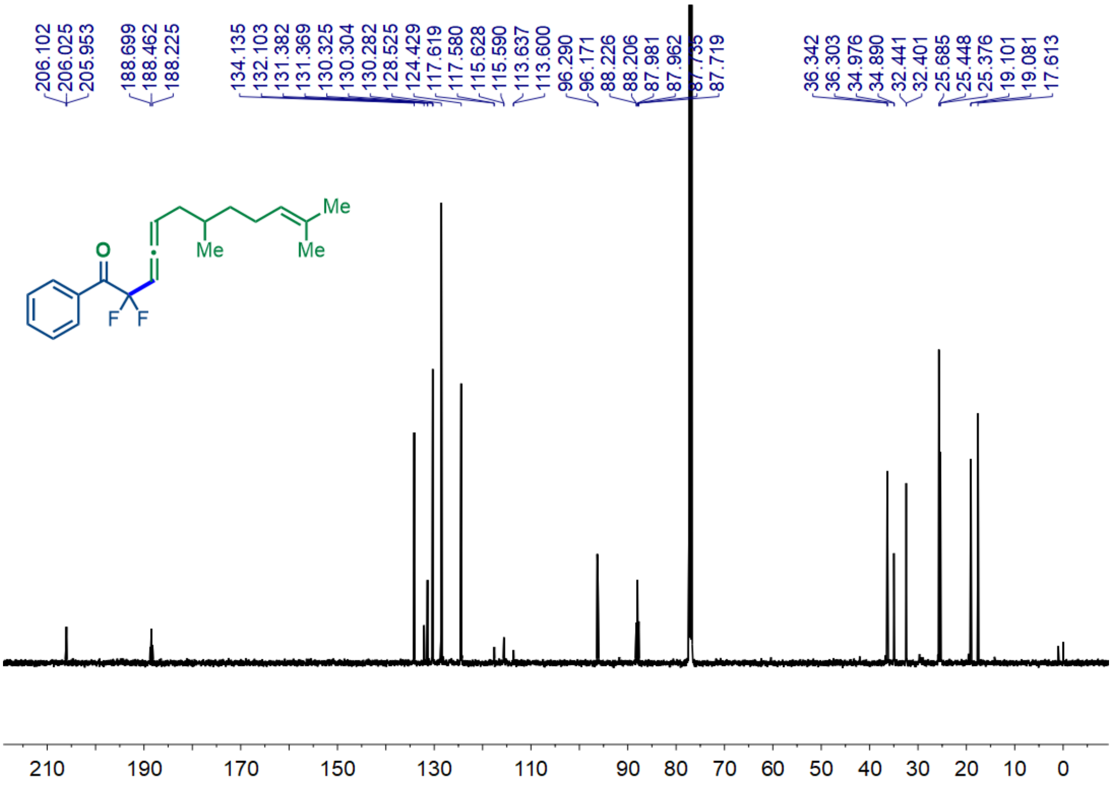

Supplementary Fig. 463 ^{13}C NMR (125 MHz, $CDCl_3$) spectrum of compound **154**

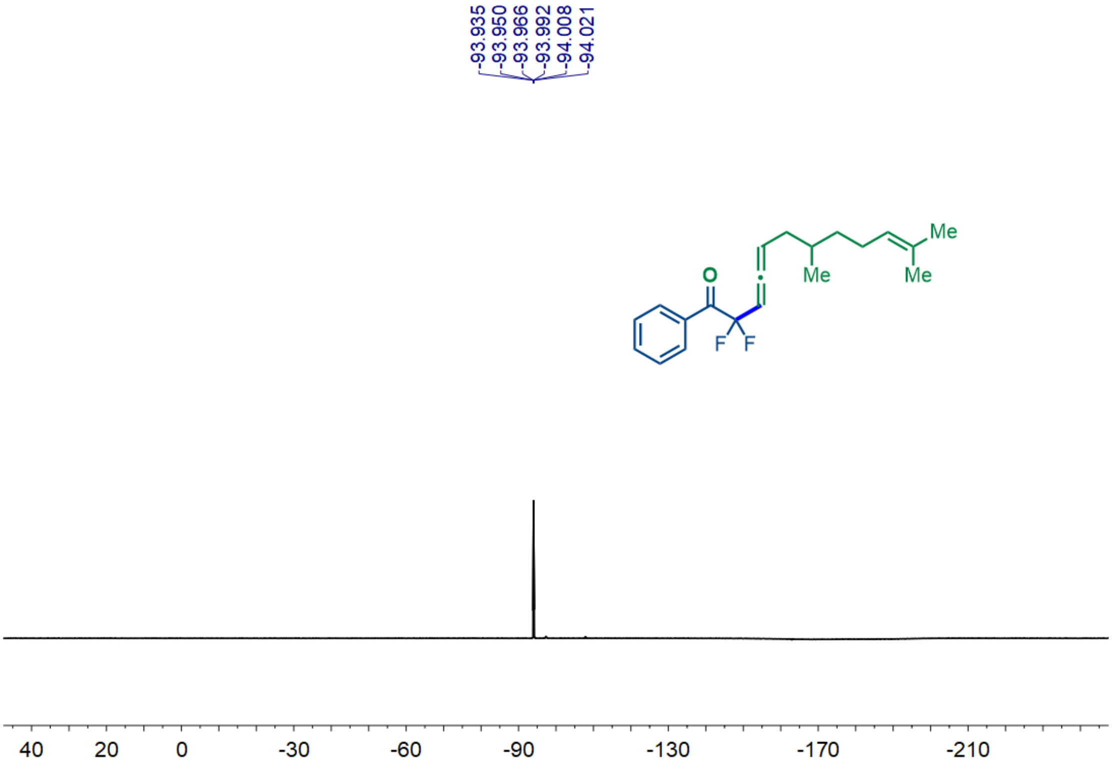

Supplementary Fig. 464 ^{19}F NMR (470 MHz, $CDCl_3$) spectrum of compound **154**

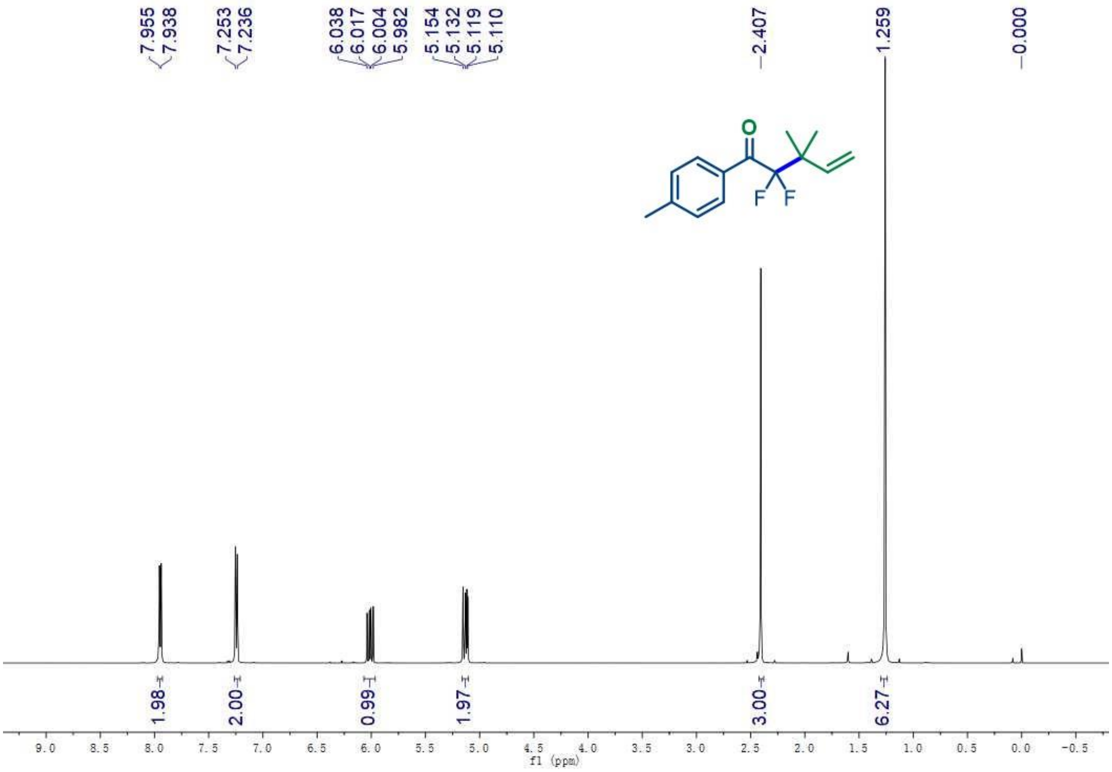

Supplementary Fig. 465 ^{1}H NMR (500 MHz, $CDCl_3$) spectrum of compound **155**

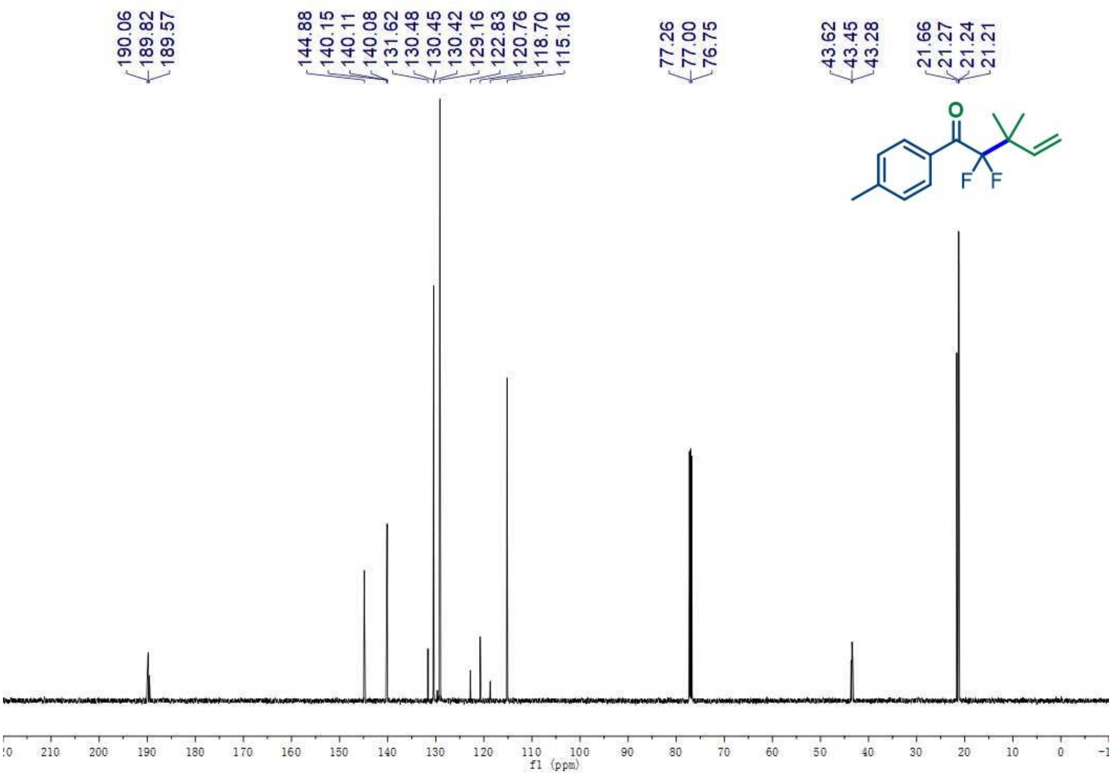

Supplementary Fig. 466 ^{13}C NMR (125 MHz, $CDCl_3$) spectrum of compound **155**

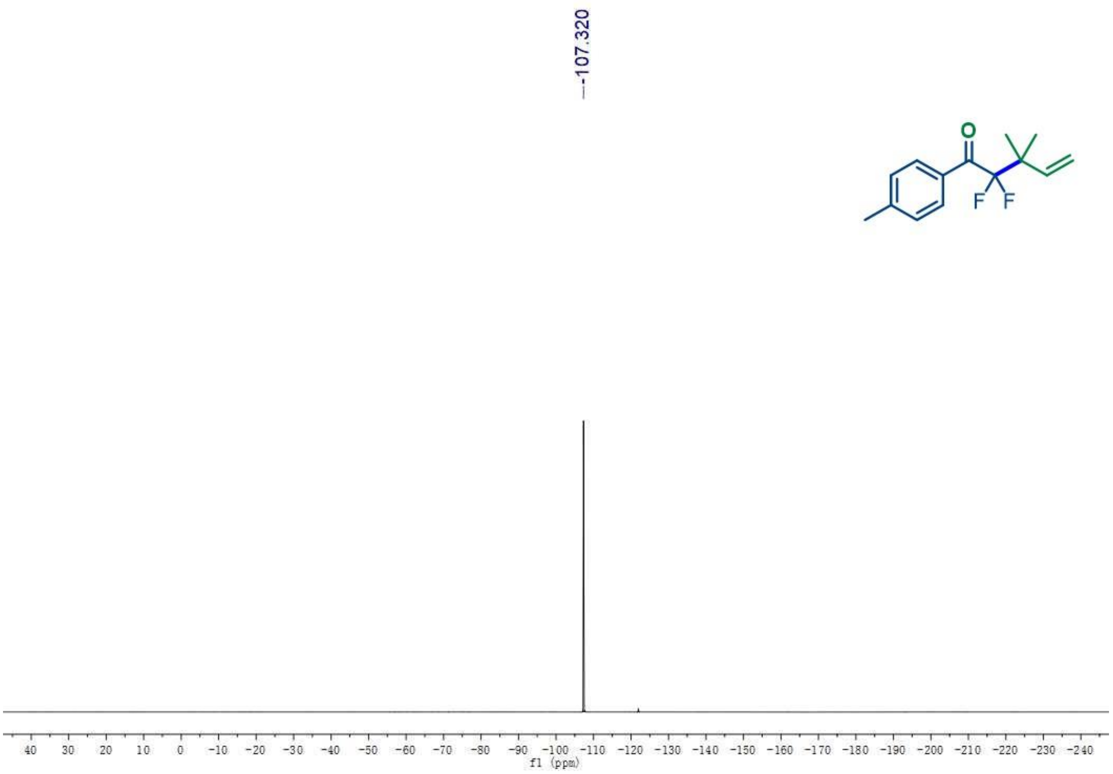

Supplementary Fig. 467 ^{19}F NMR (470 MHz, $CDCl_3$) spectrum of compound **155**

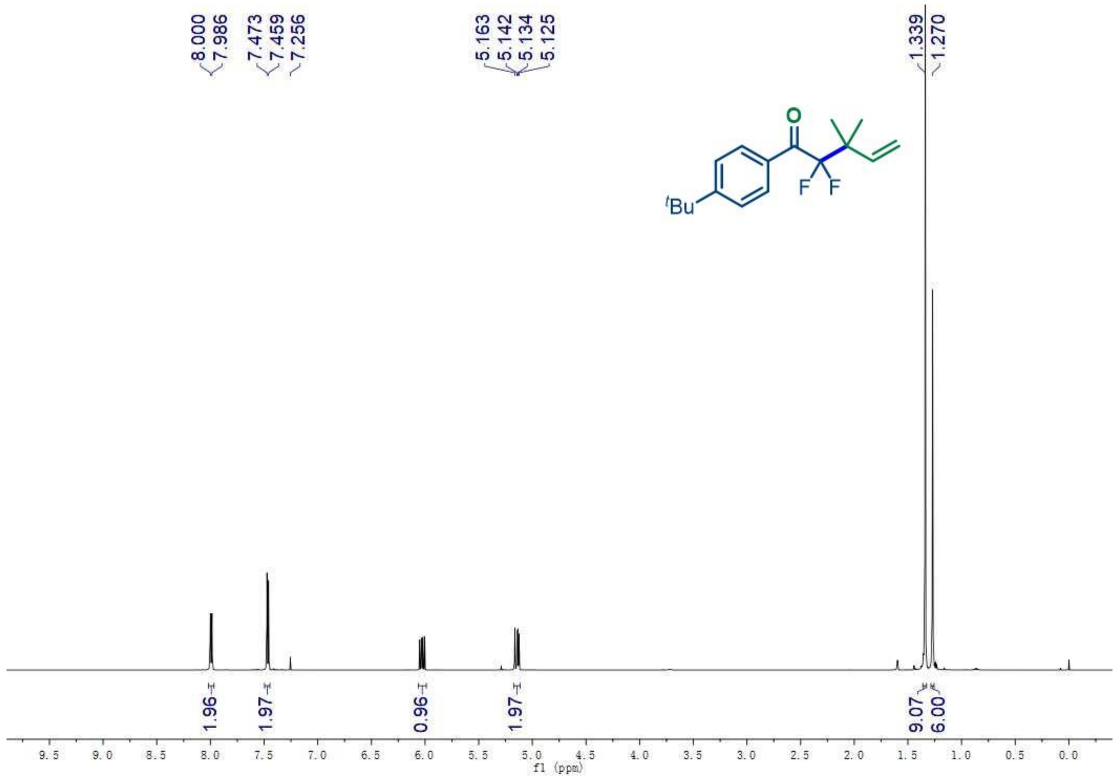

Supplementary Fig. 468 ^{1}H NMR (600 MHz, $CDCl_3$) spectrum of compound **156**

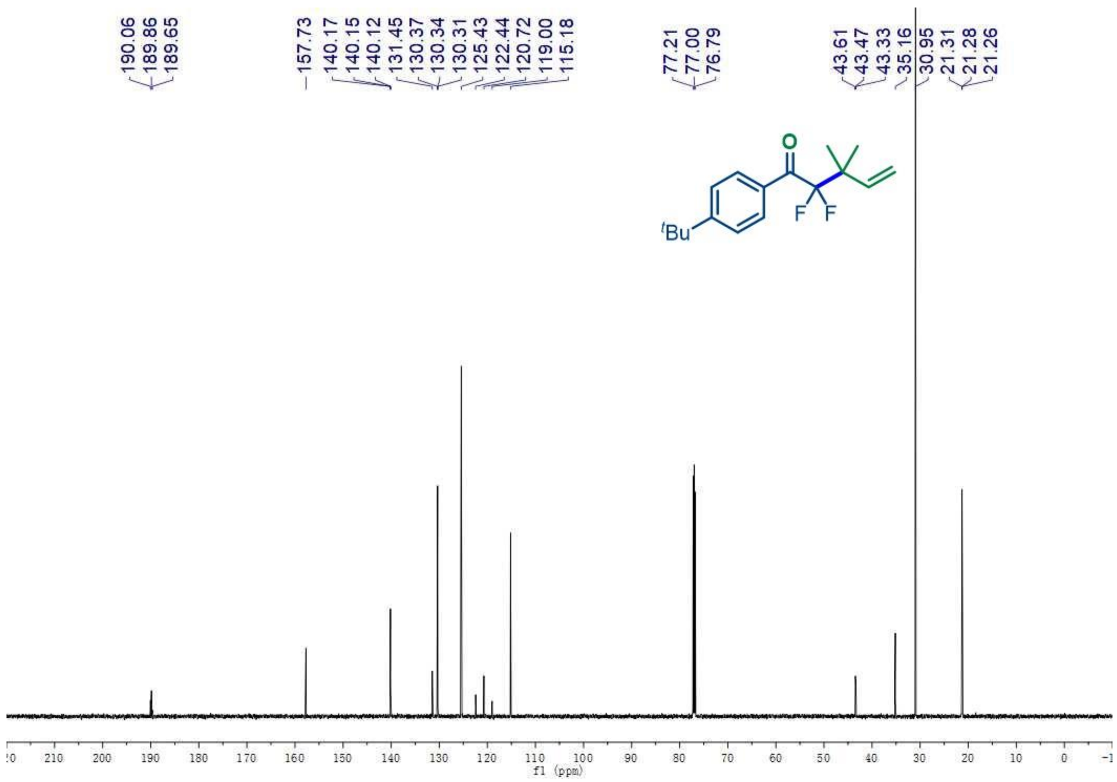

Supplementary Fig. 469 ^{13}C NMR (150 MHz, $CDCl_3$) spectrum of compound **156**

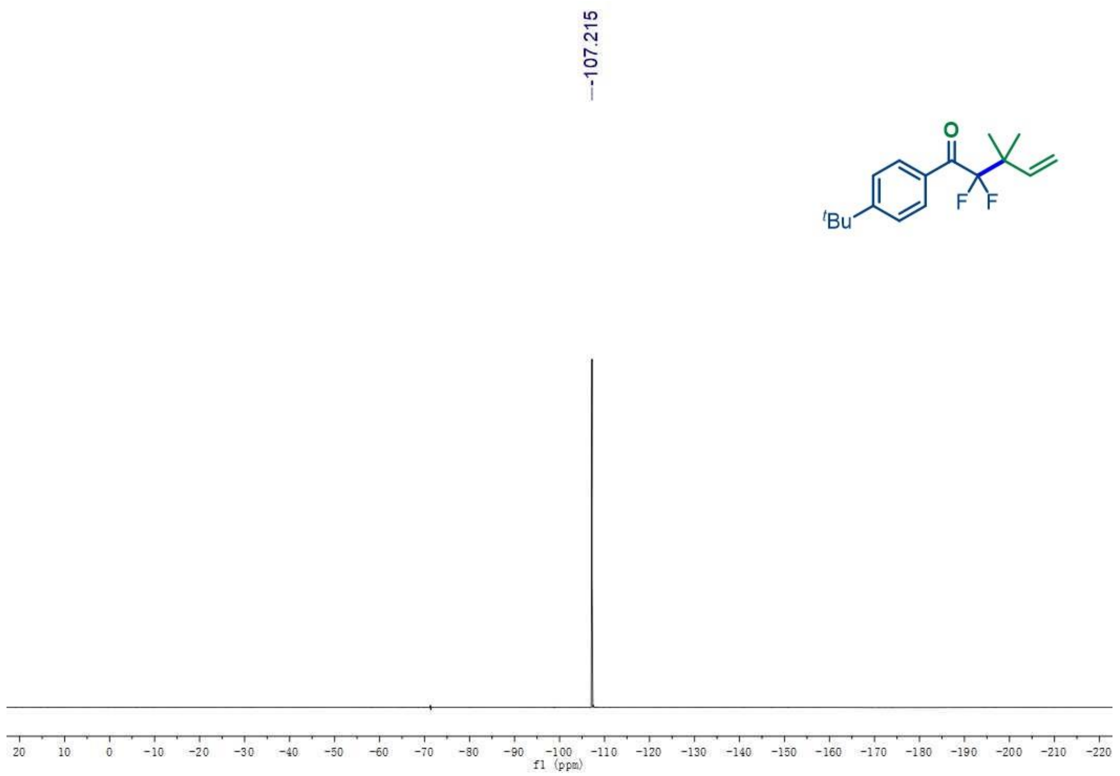

Supplementary Fig. 470 ^{19}F NMR (564 MHz, $CDCl_3$) spectrum of compound **156**

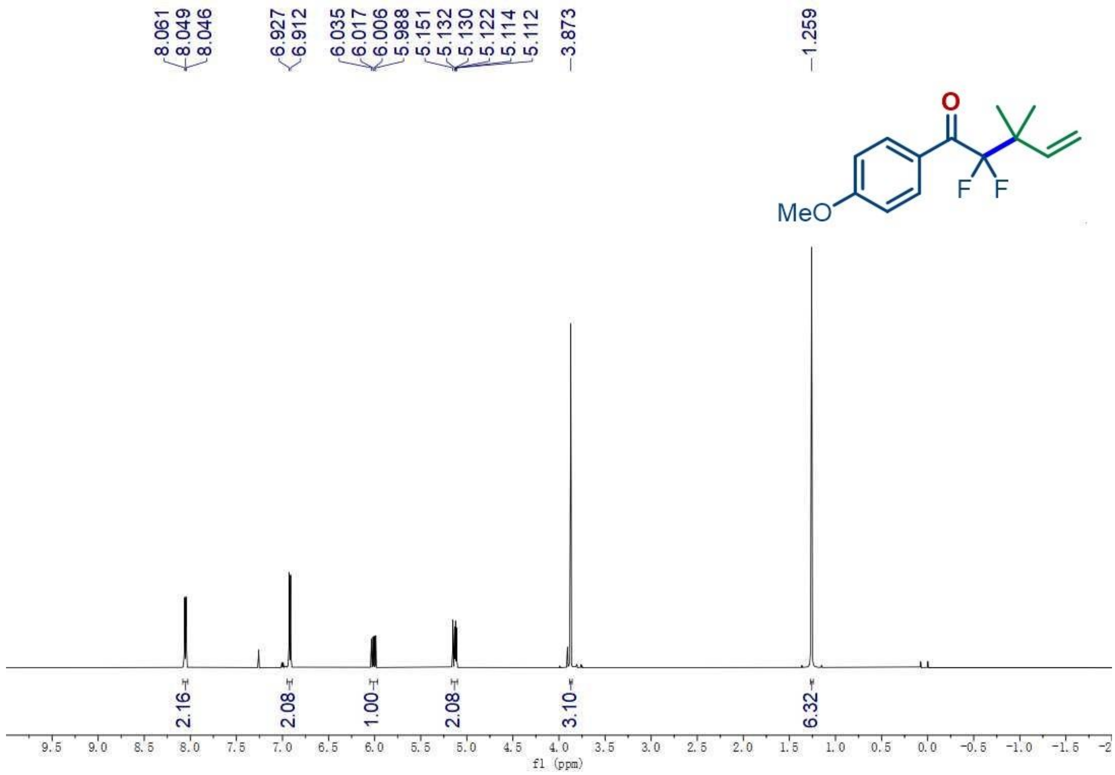

Supplementary Fig. 471 ^{1}H NMR (600 MHz, $CDCl_3$) spectrum of compound **157**

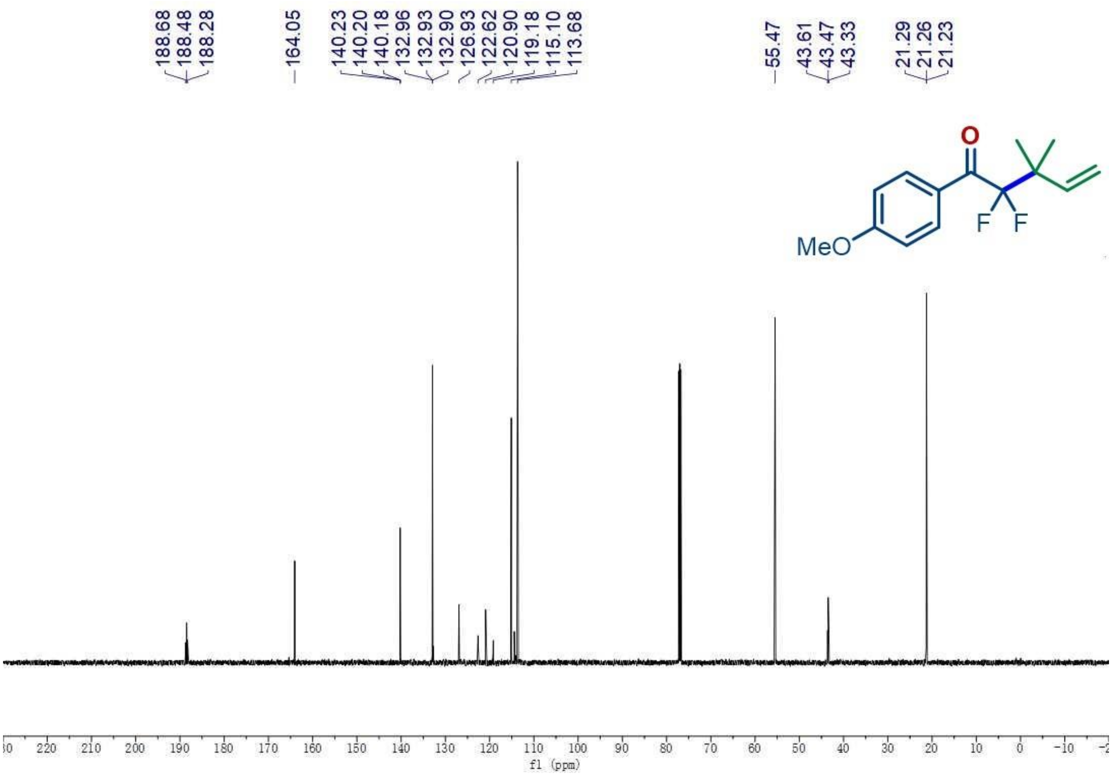

Supplementary Fig. 472 ^{13}C NMR (150 MHz, $CDCl_3$) spectrum of compound **157**

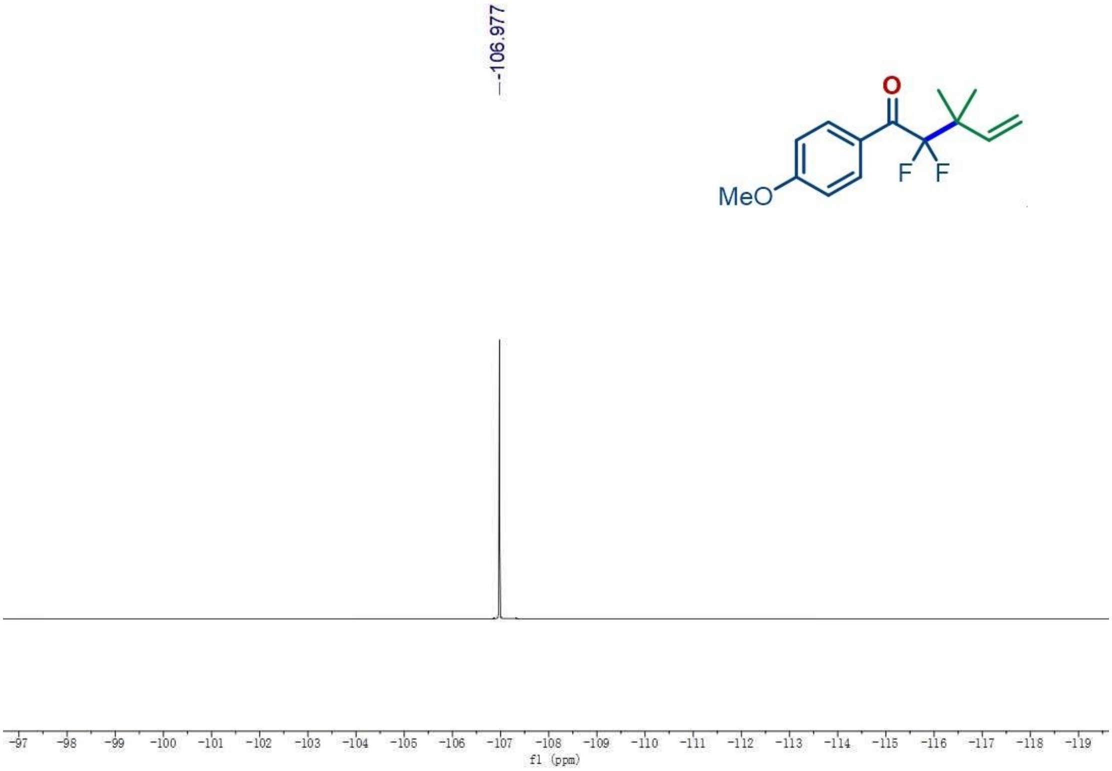

Supplementary Fig. 473 ^{19}F NMR (564 MHz, $CDCl_3$) spectrum of compound **157**

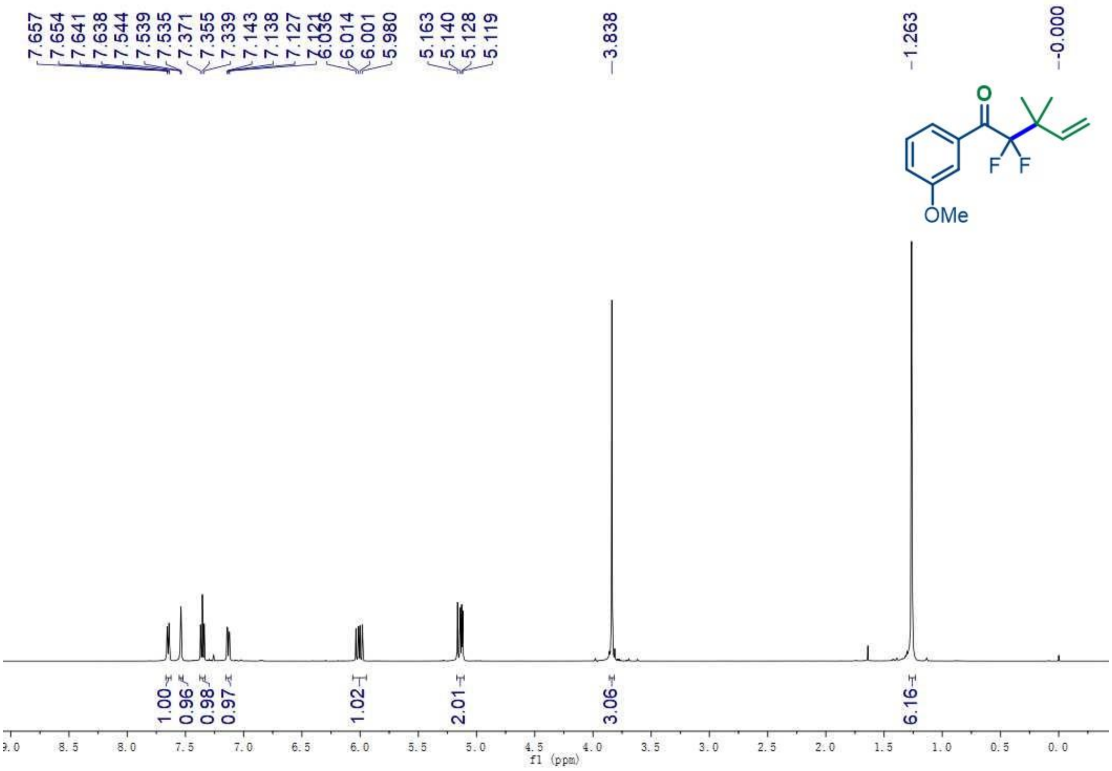

Supplementary Fig. 474 ^{1}H NMR (500 MHz, $CDCl_3$) spectrum of compound **158**

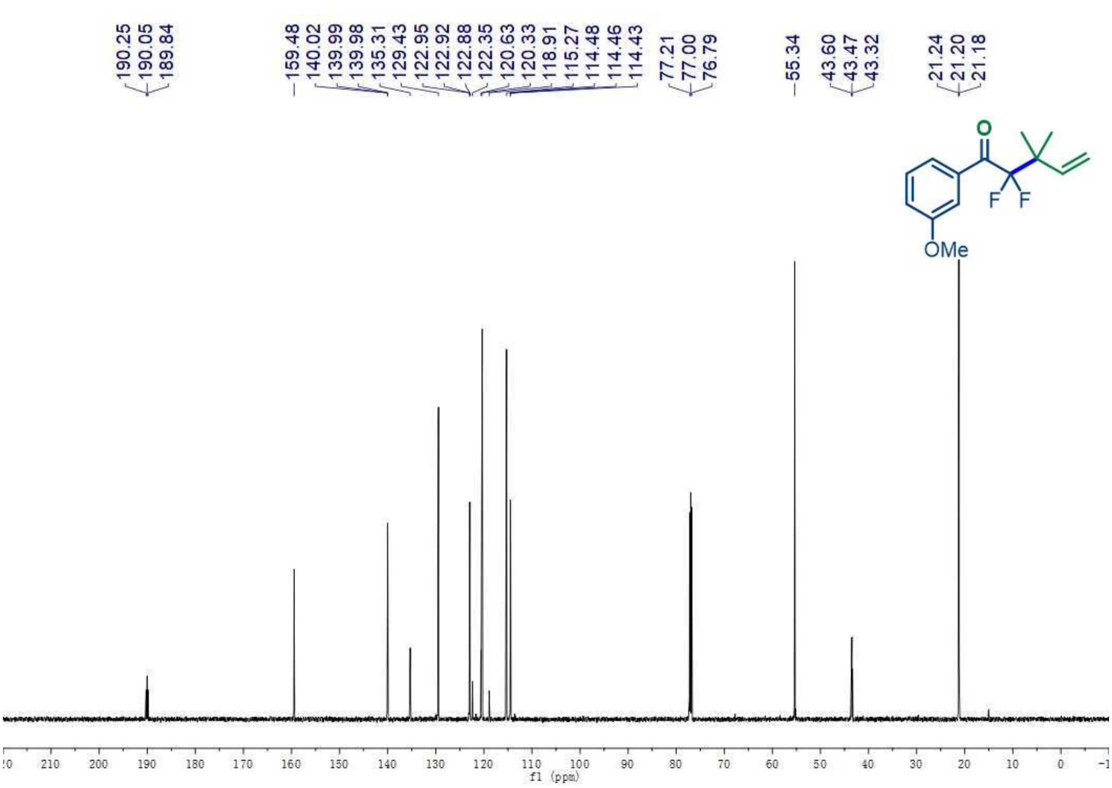

Supplementary Fig. 475 ^{13}C NMR (150 MHz, $CDCl_3$) spectrum of compound **158**

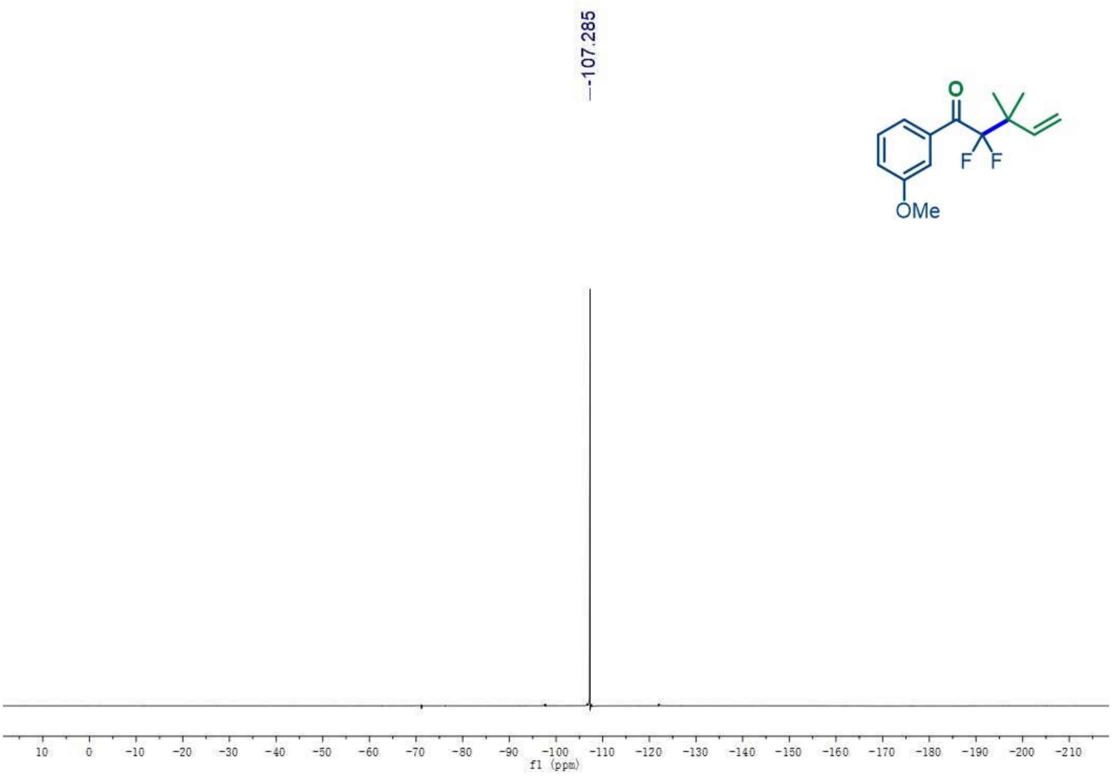

Supplementary Fig. 476 ^{19}F NMR (564 MHz, $CDCl_3$) spectrum of compound **158**

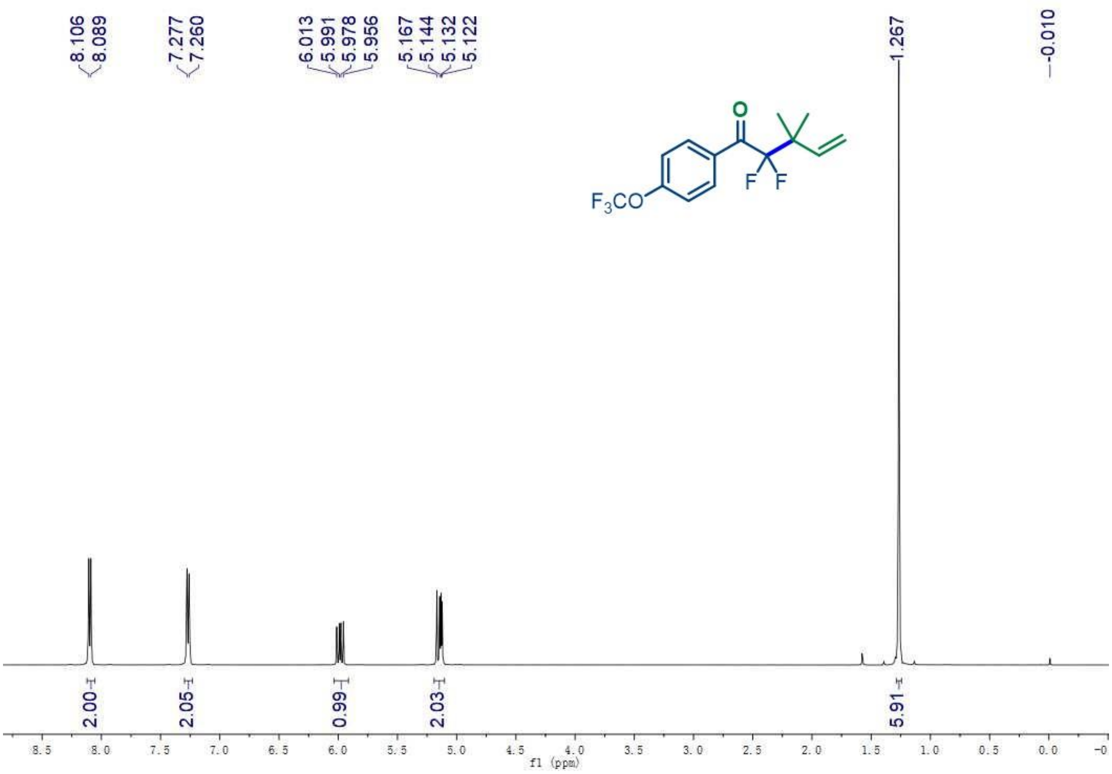

Supplementary Fig. 477 ^{1}H NMR (500 MHz, $CDCl_3$) spectrum of compound **159**

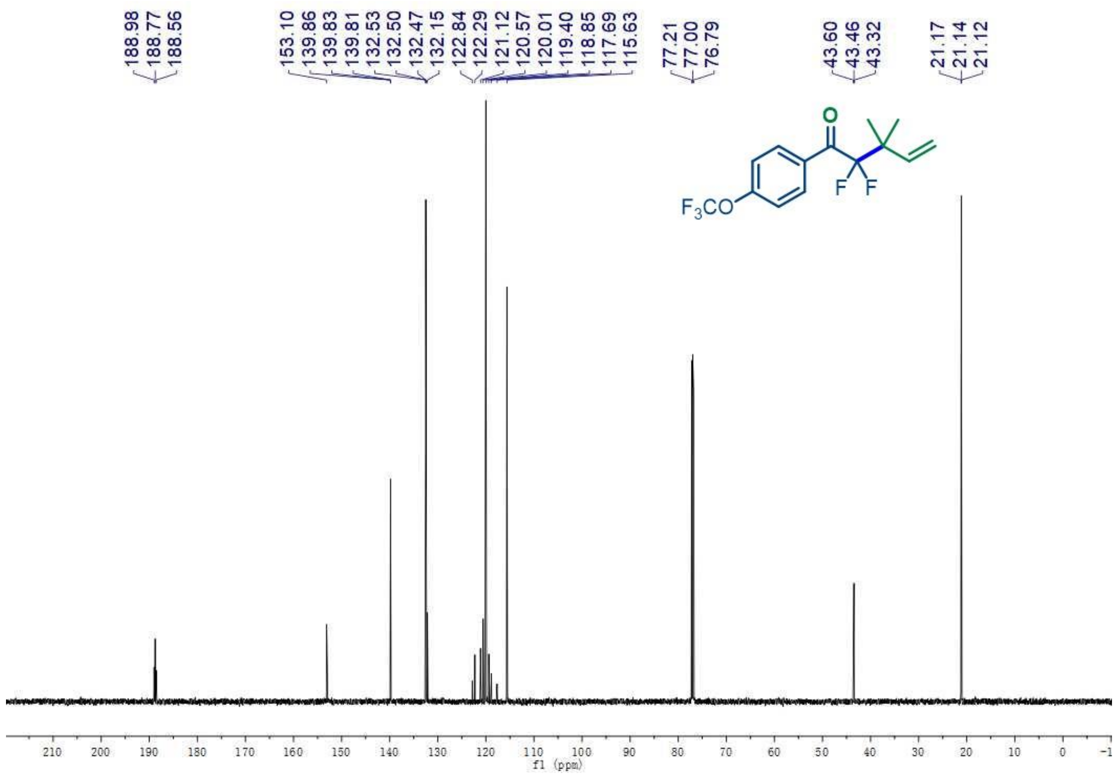

Supplementary Fig. 478 ^{13}C NMR (150 MHz, $CDCl_3$) spectrum of compound **159**

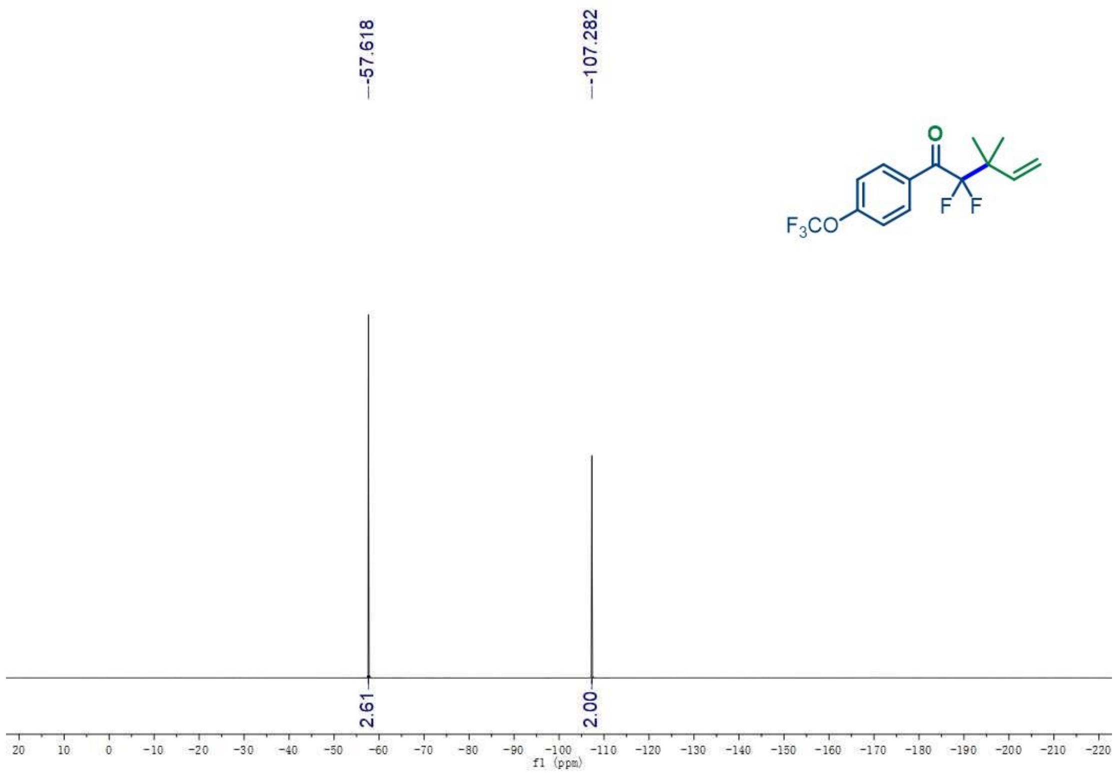

Supplementary Fig. 479 ^{19}F NMR (564 MHz, $CDCl_3$) spectrum of compound **159**

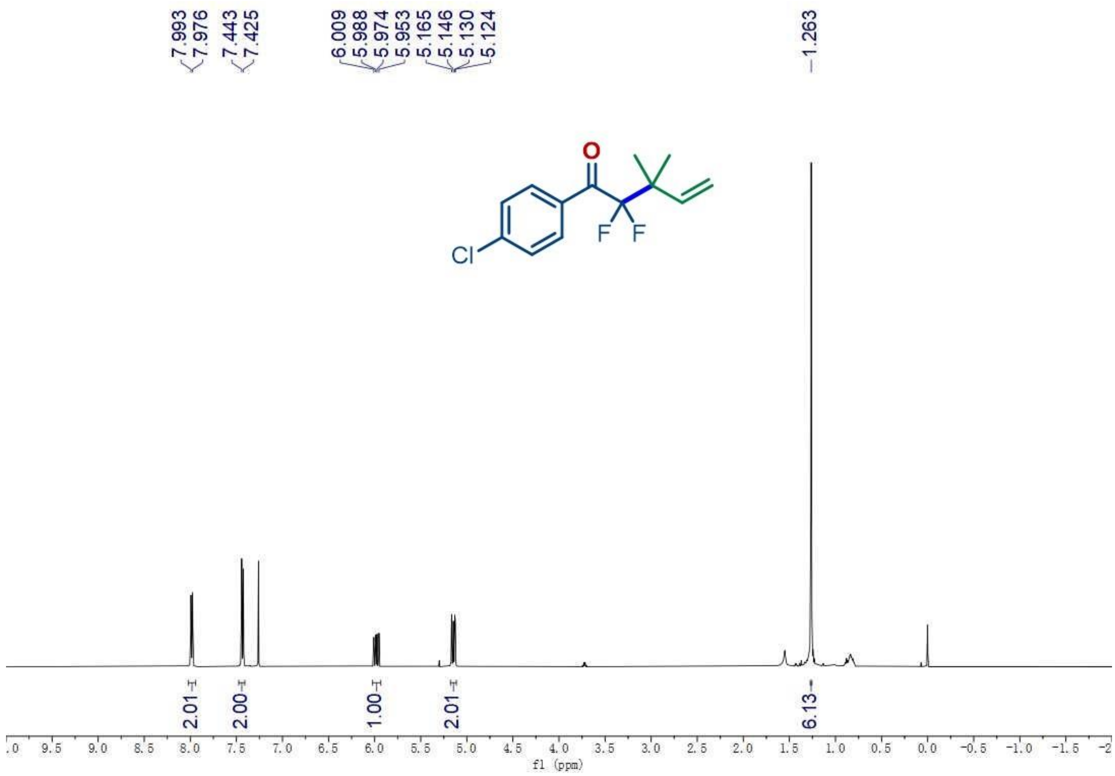

Supplementary Fig. 480 ^{1}H NMR (500 MHz, $CDCl_3$) spectrum of compound **160**

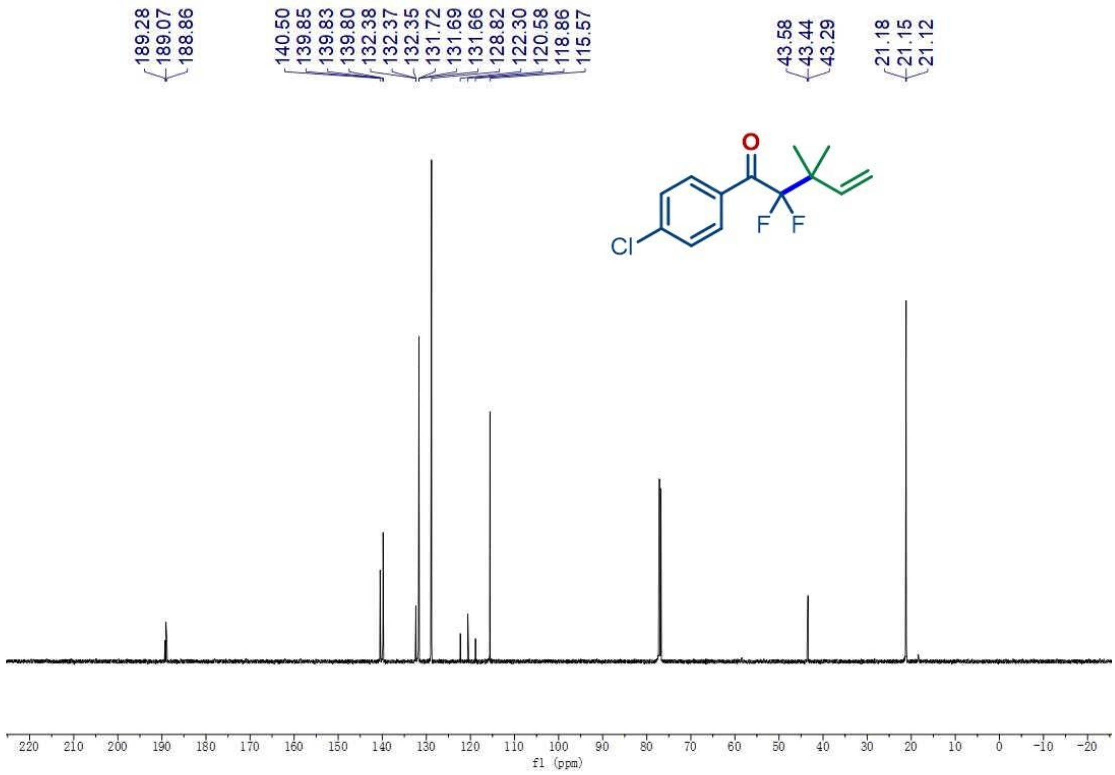

Supplementary Fig. 481 ^{13}C NMR (150 MHz, $CDCl_3$) spectrum of compound **160**

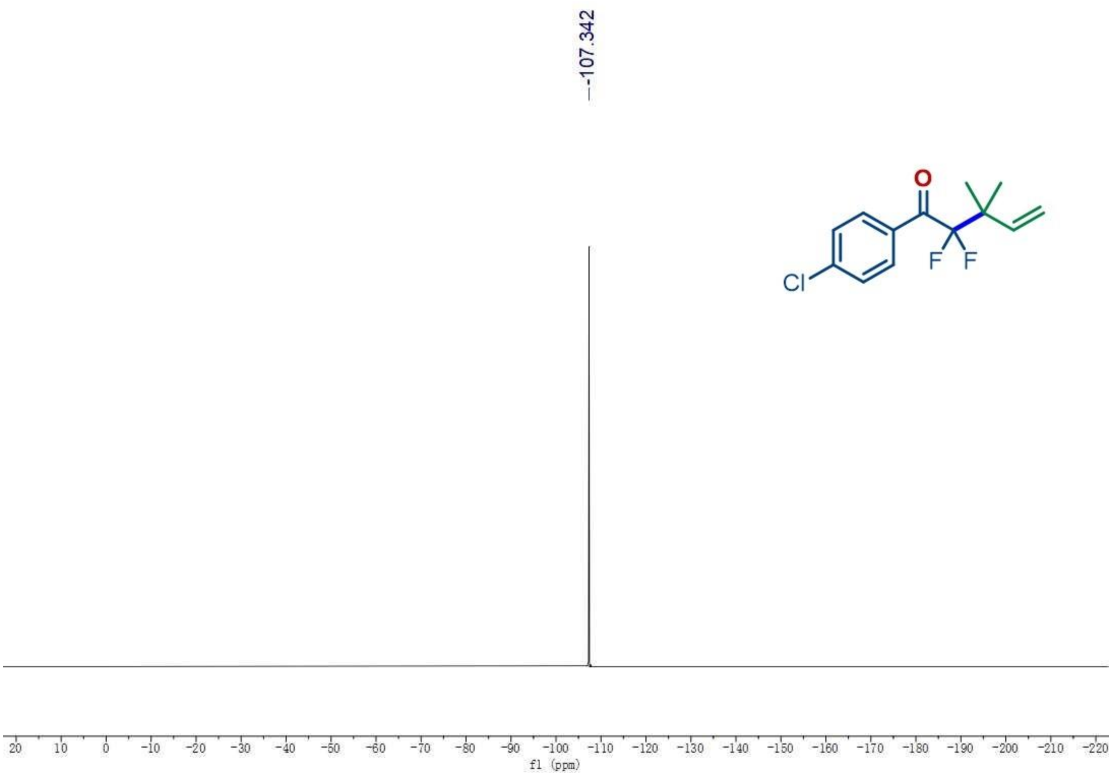

Supplementary Fig. 482 ^{19}F NMR (564 MHz, $CDCl_3$) spectrum of compound **160**

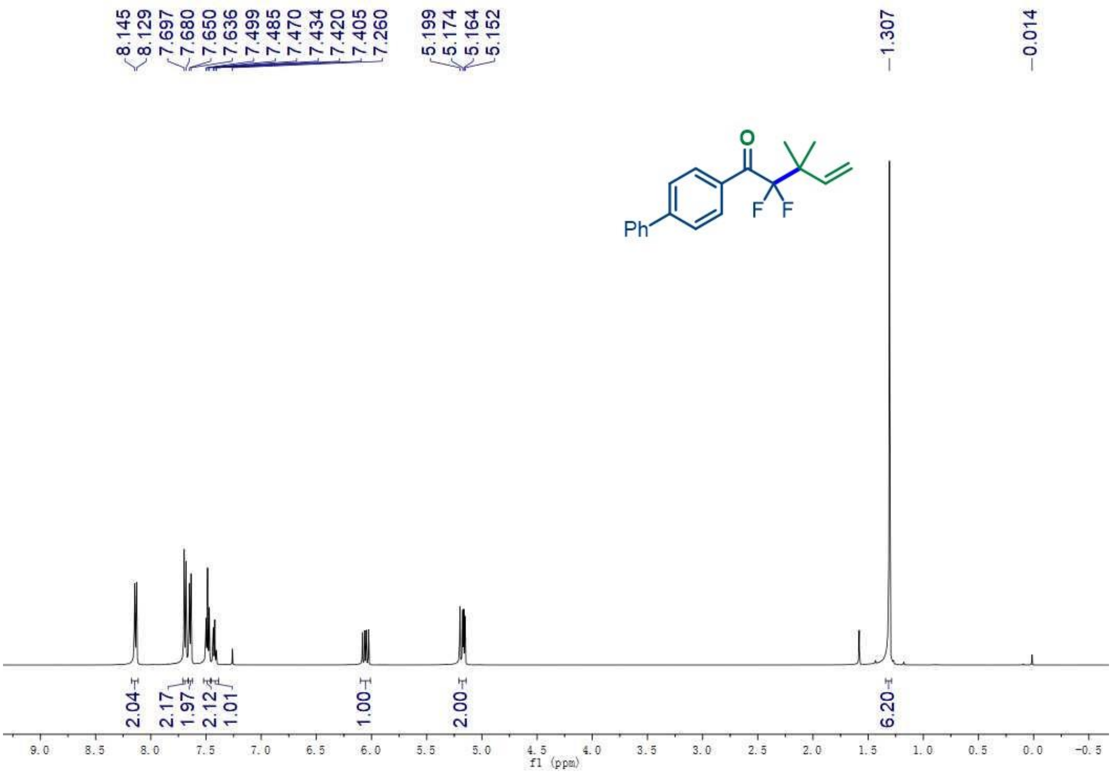

Supplementary Fig. 483 ^{1}H NMR (500 MHz, $CDCl_3$) spectrum of compound **161**

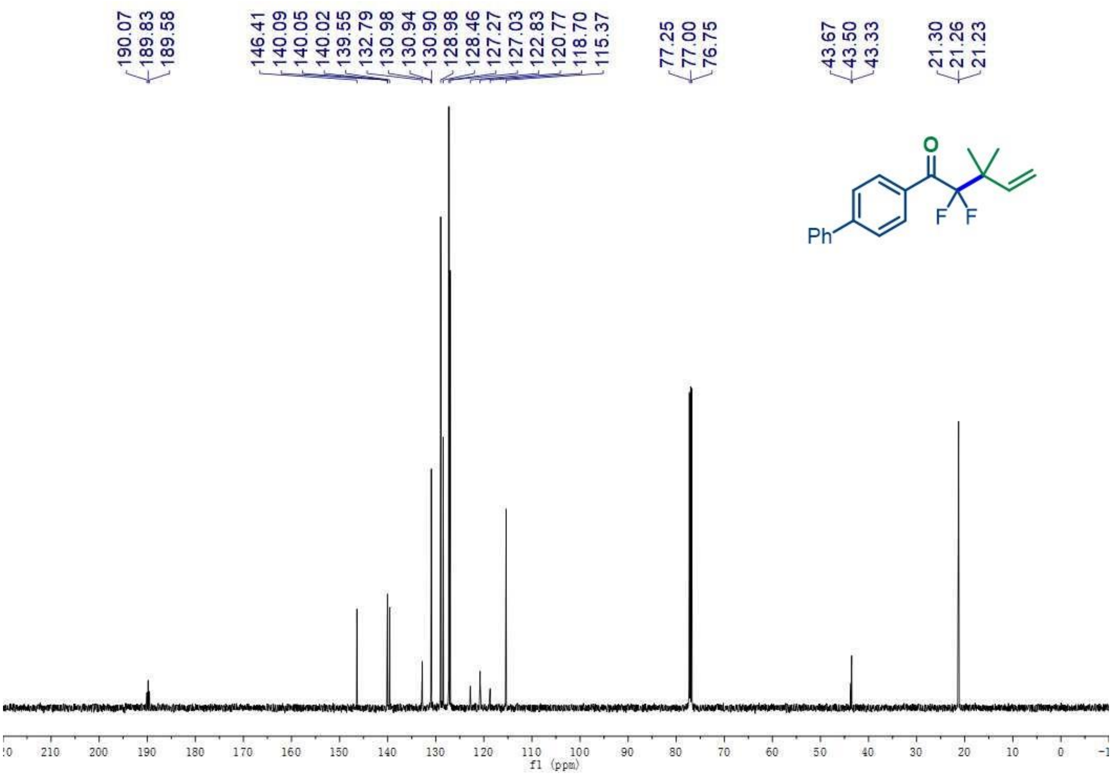

Supplementary Fig. 484 ^{13}C NMR (125 MHz, $CDCl_3$) spectrum of compound **161**

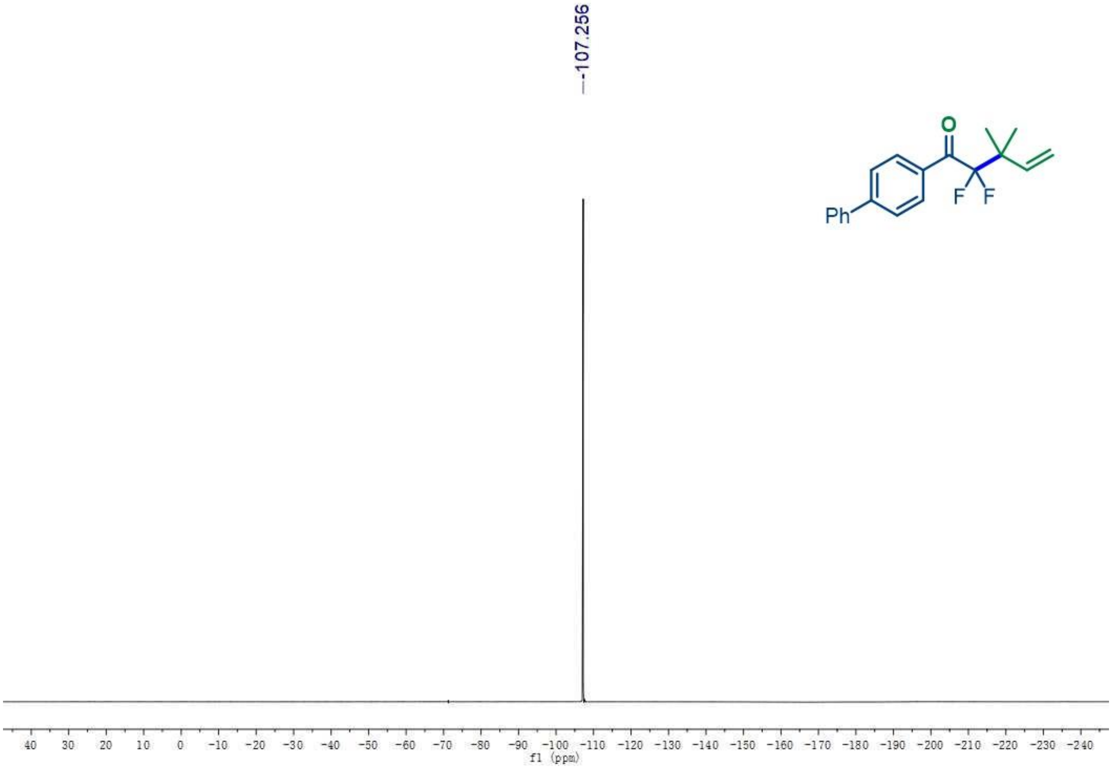

Supplementary Fig. 485 ^{19}F NMR (470 MHz, $CDCl_3$) spectrum of compound **161**

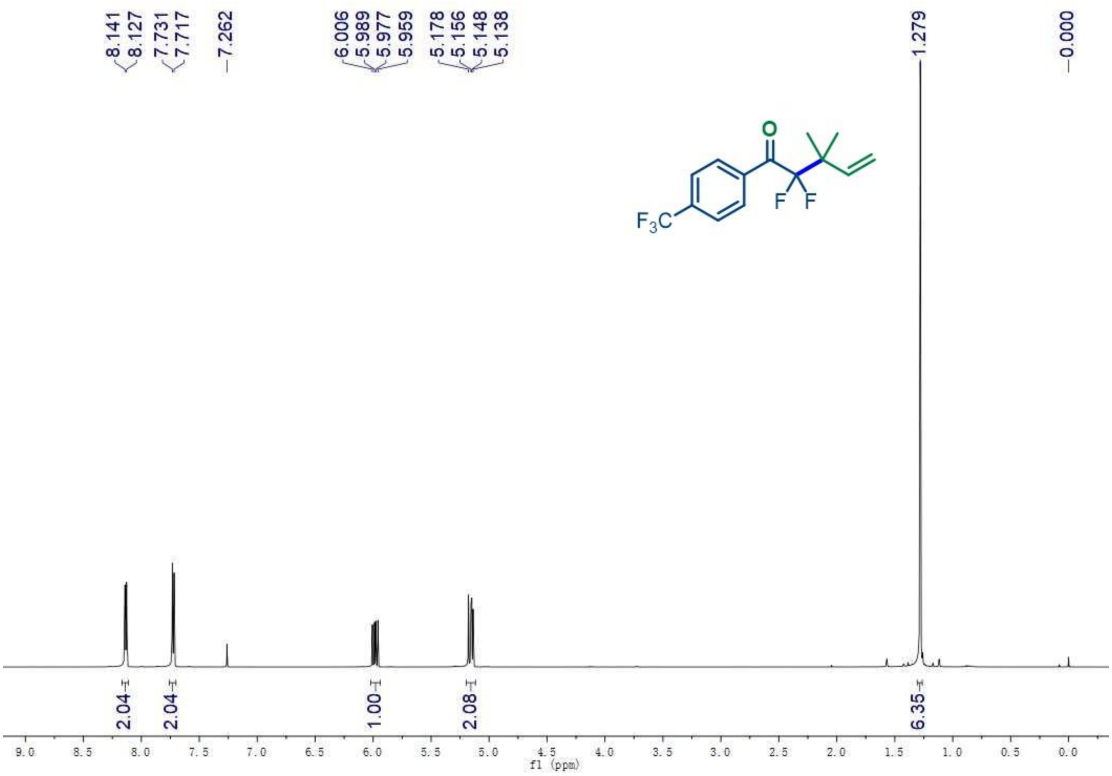

Supplementary Fig. 486 ^{1}H NMR (600 MHz, $CDCl_3$) spectrum of compound **162**

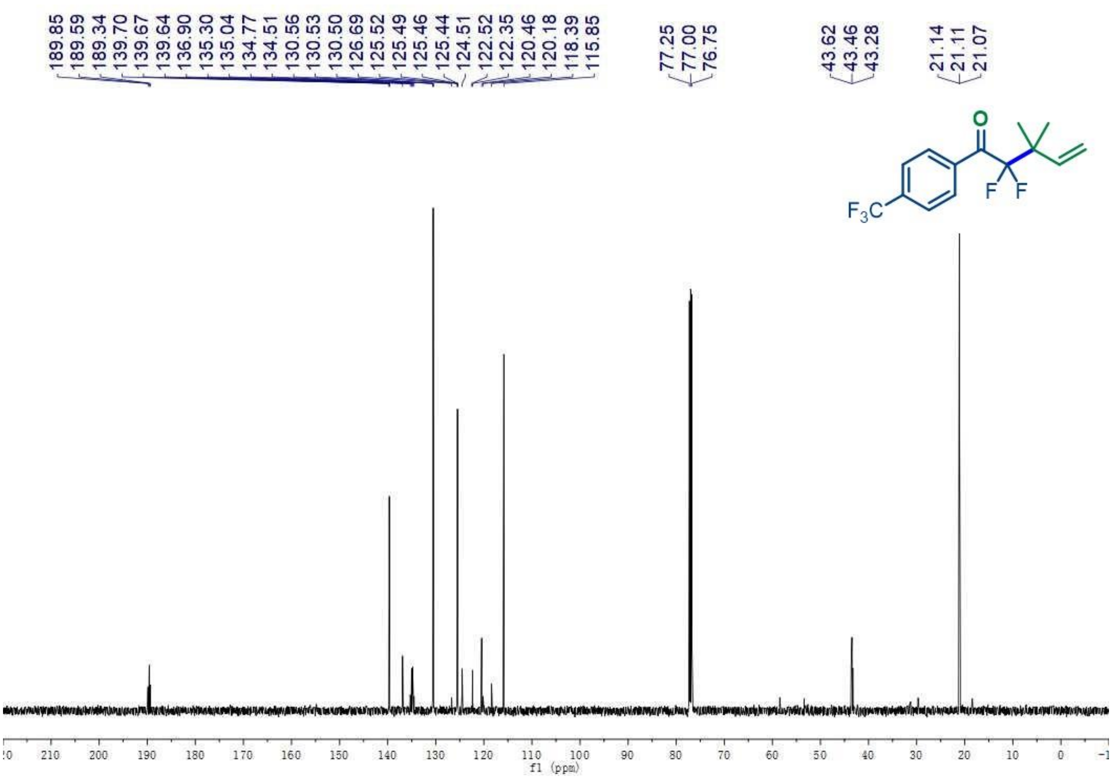

Supplementary Fig. 487 ^{13}C NMR (125 MHz, $CDCl_3$) spectrum of compound **162**

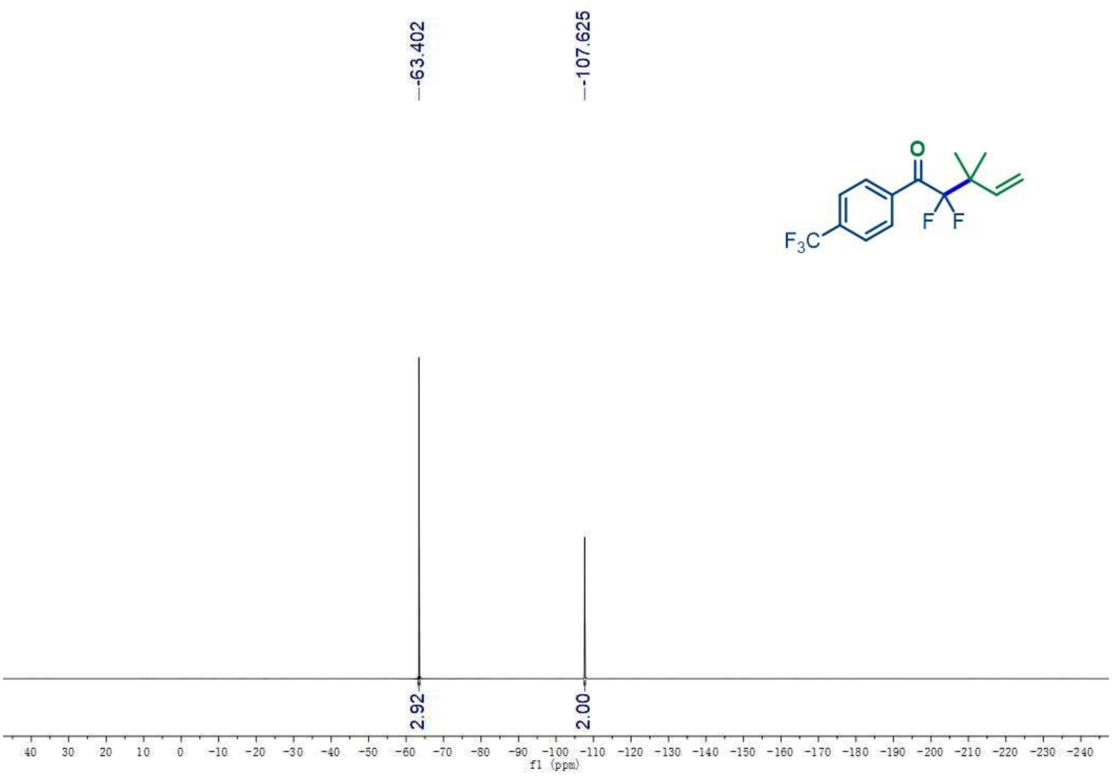

Supplementary Fig. 488 ^{19}F NMR (470 MHz, $CDCl_3$) spectrum of compound **162**

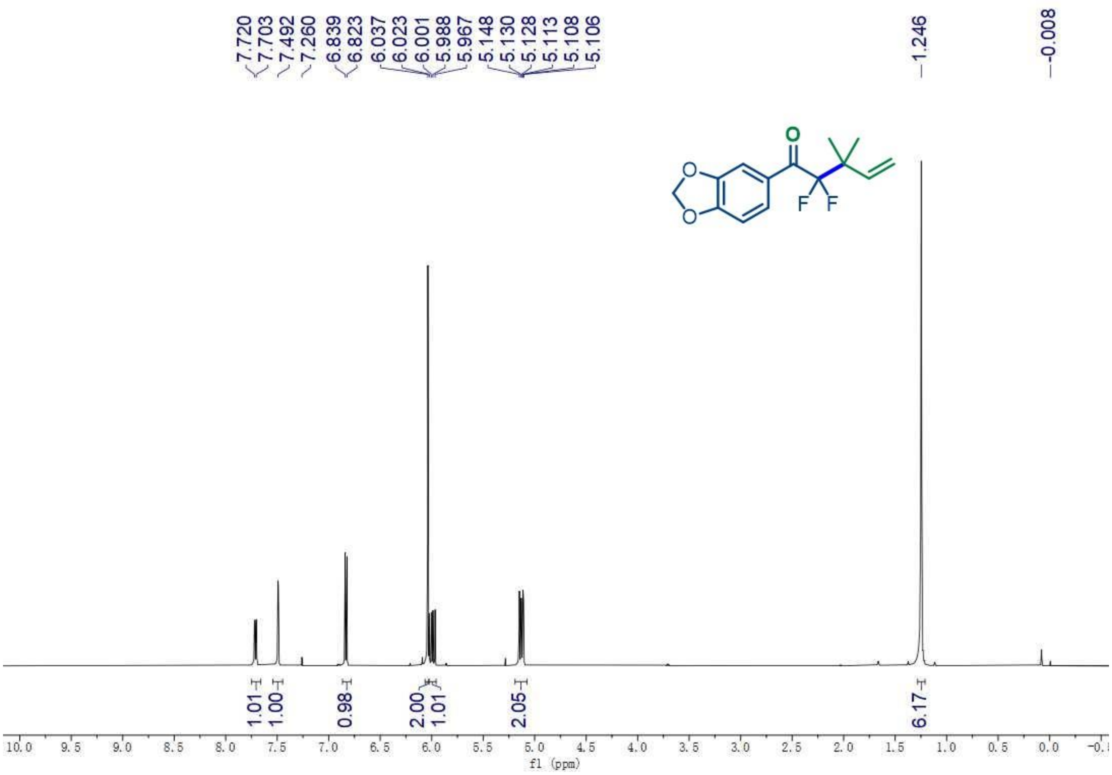

Supplementary Fig. 489 ^{1}H NMR (500 MHz, $CDCl_3$) spectrum of compound **163**

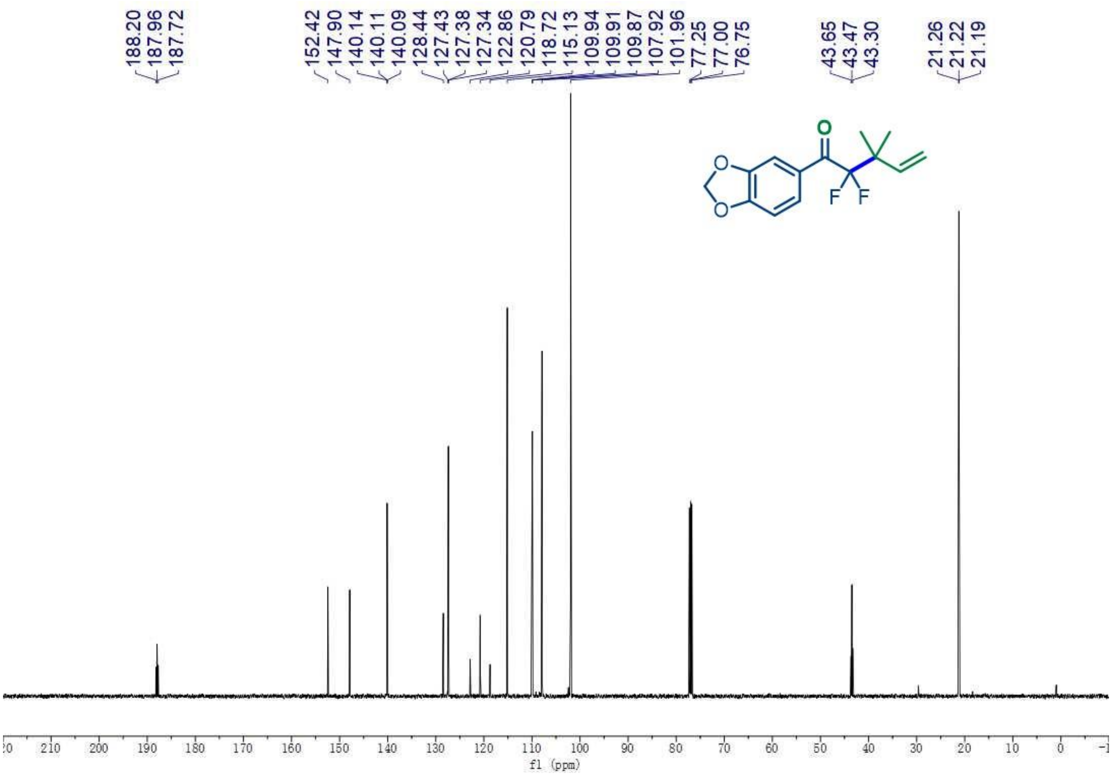

Supplementary Fig. 490 ^{13}C NMR (125 MHz, $CDCl_3$) spectrum of compound **163**

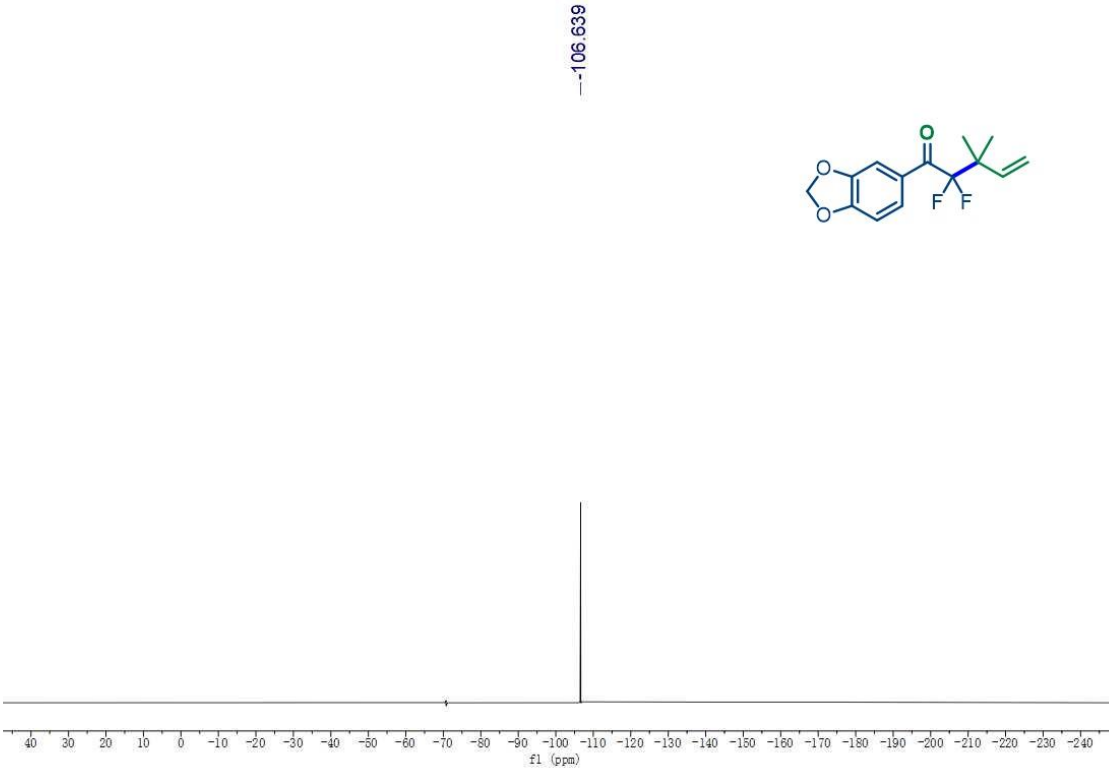

Supplementary Fig. 491 ^{19}F NMR (470 MHz, $CDCl_3$) spectrum of compound **163**

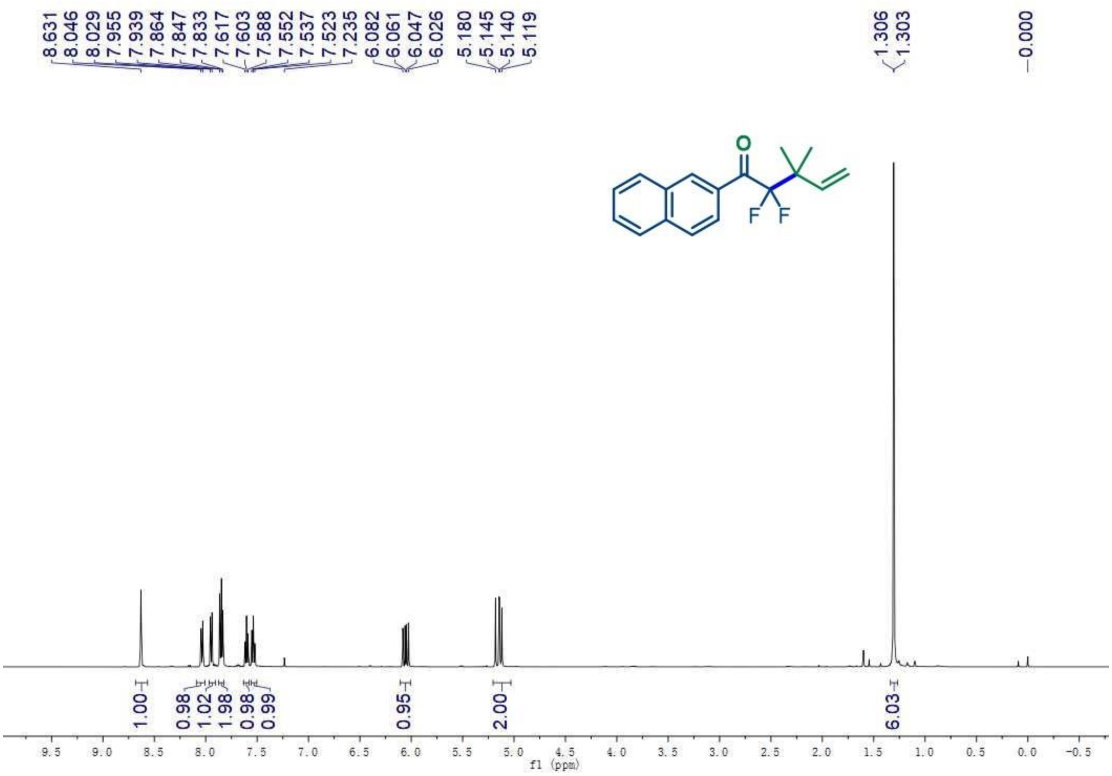

Supplementary Fig. 492 ^{1}H NMR (500 MHz, $CDCl_3$) spectrum of compound **164**

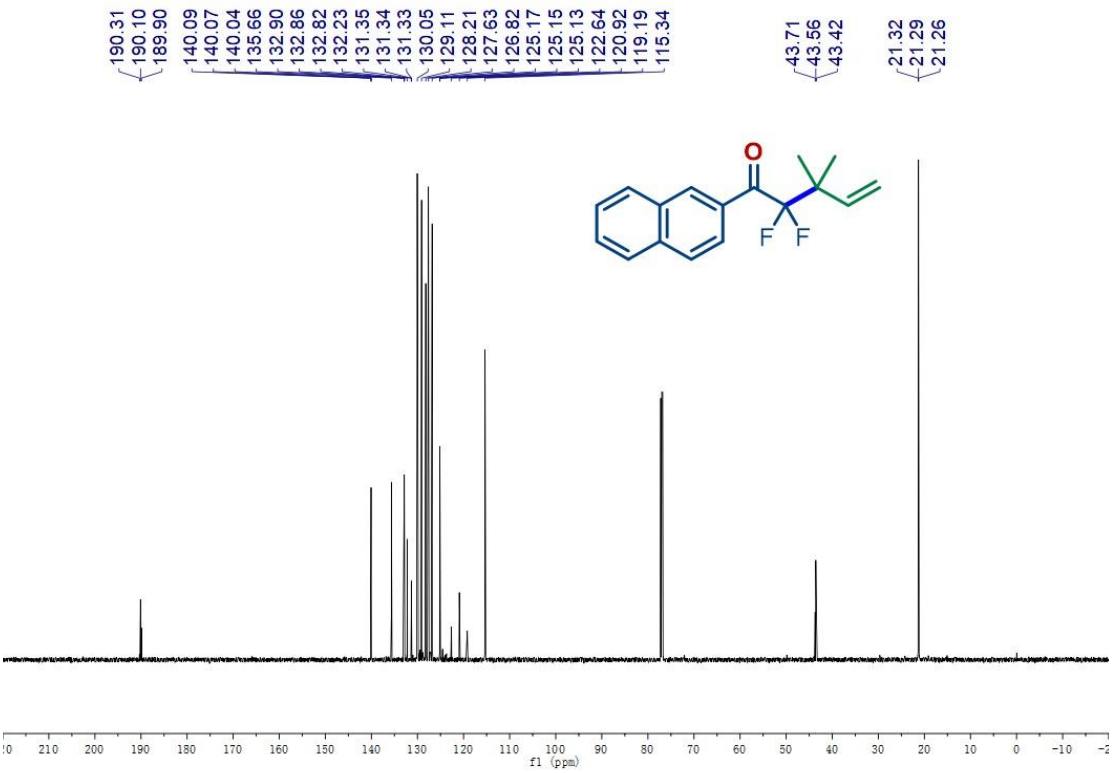

Supplementary Fig. 493 ^{13}C NMR (150 MHz, $CDCl_3$) spectrum of compound **164**

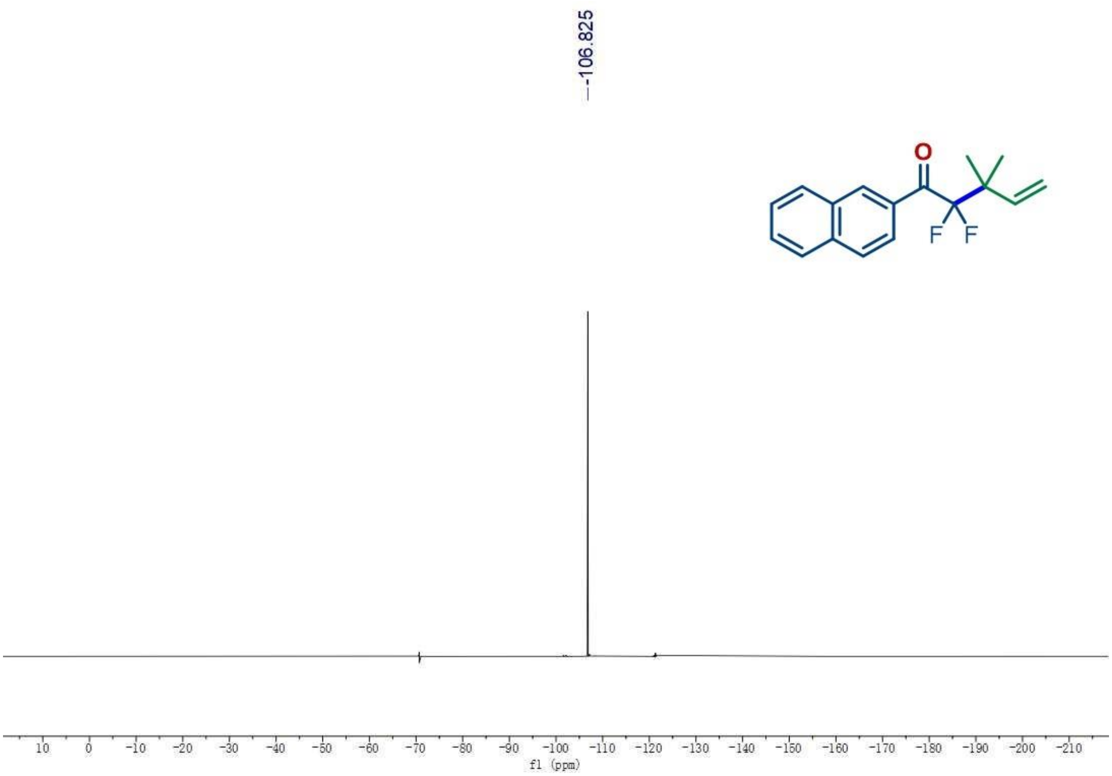

Supplementary Fig. 494 ^{19}F NMR (564 MHz, $CDCl_3$) spectrum of compound **164**

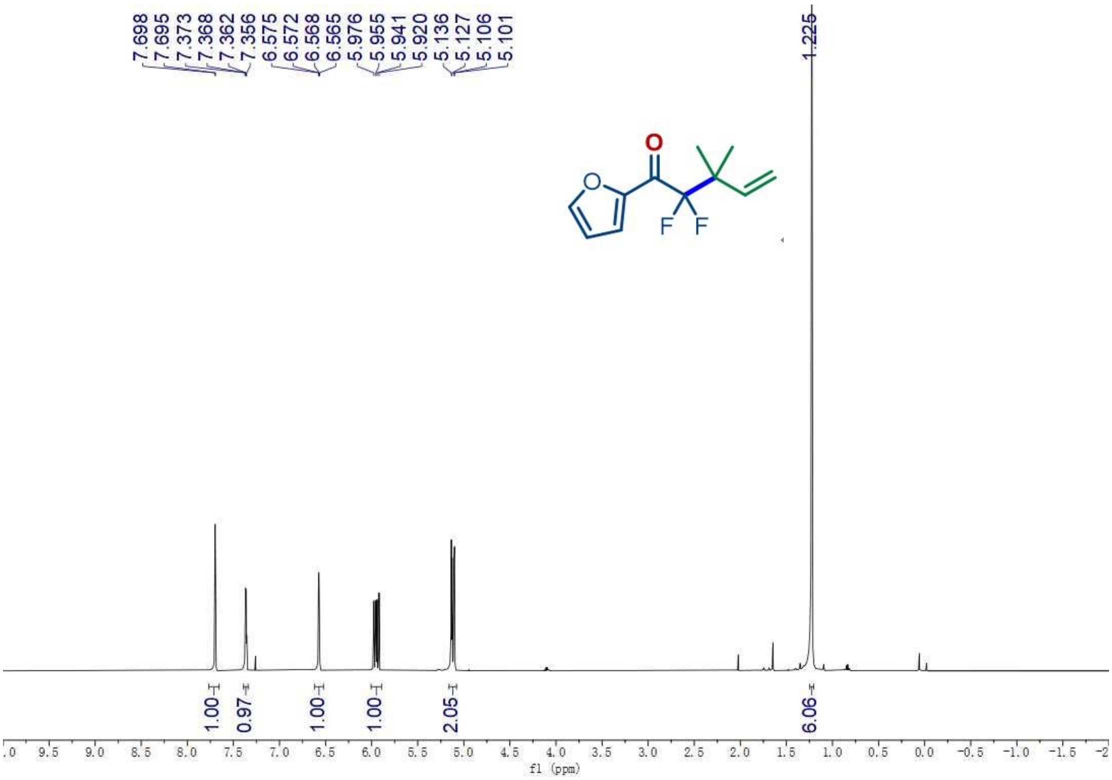

Supplementary Fig. 495 ^{1}H NMR (500 MHz, $CDCl_3$) spectrum of compound **165**

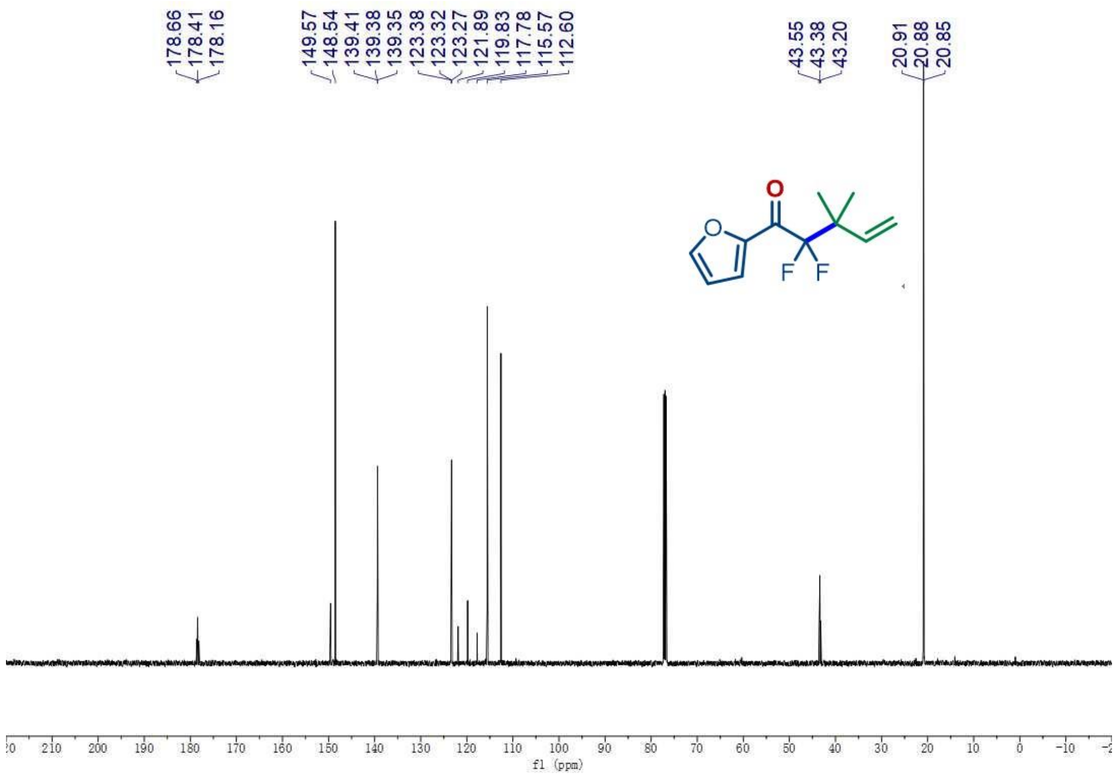

Supplementary Fig. 496 ^{13}C NMR (125 MHz, $CDCl_3$) spectrum of compound **165**

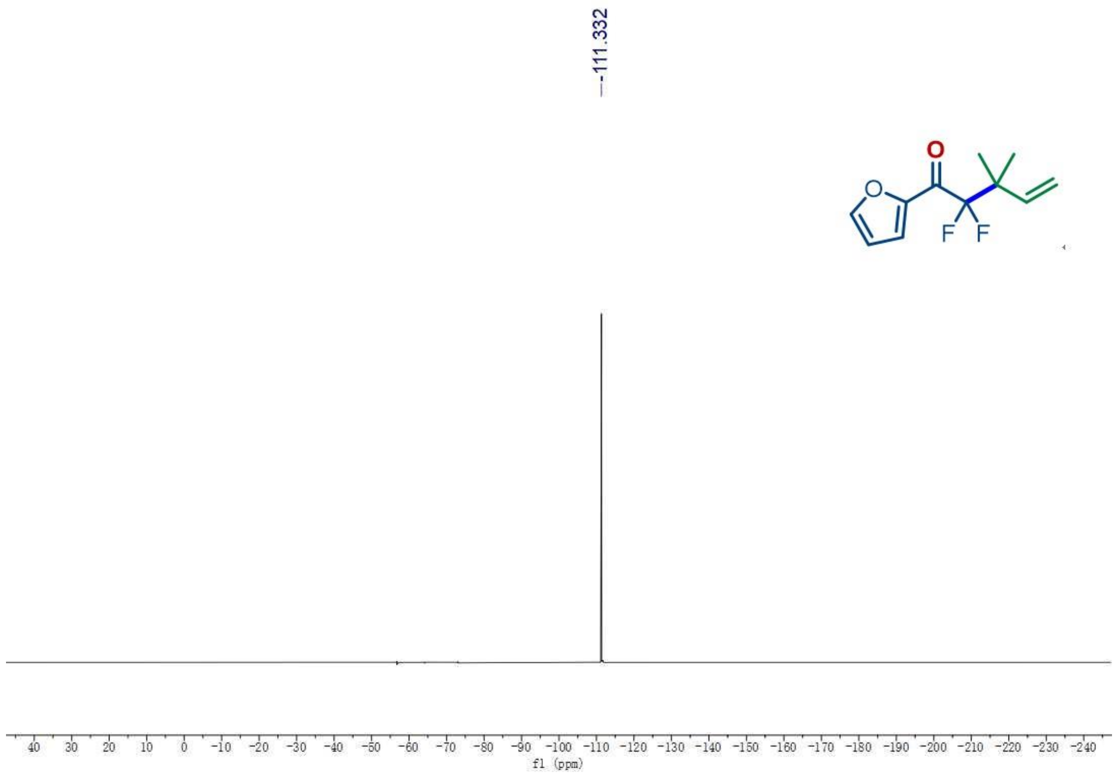

Supplementary Fig. 497 ^{19}F NMR (470 MHz, $CDCl_3$) spectrum of compound **165**

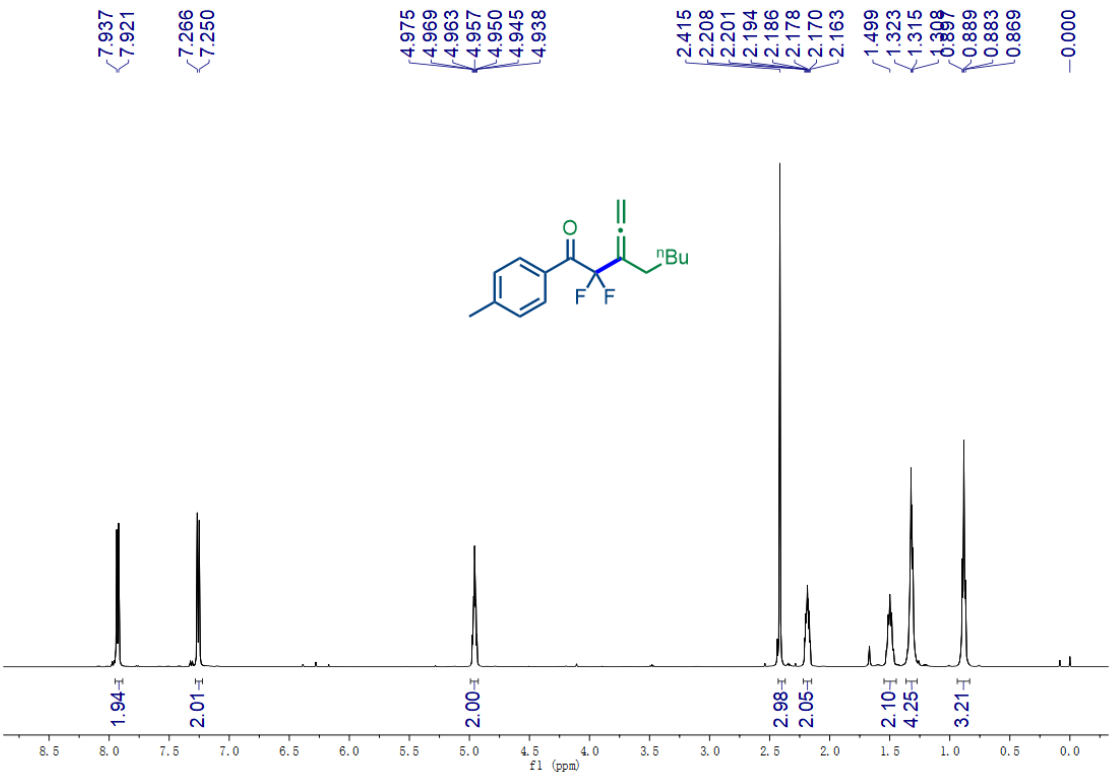

Supplementary Fig. 498 ^{1}H NMR (500 MHz, $CDCl_3$) spectrum of compound **166**

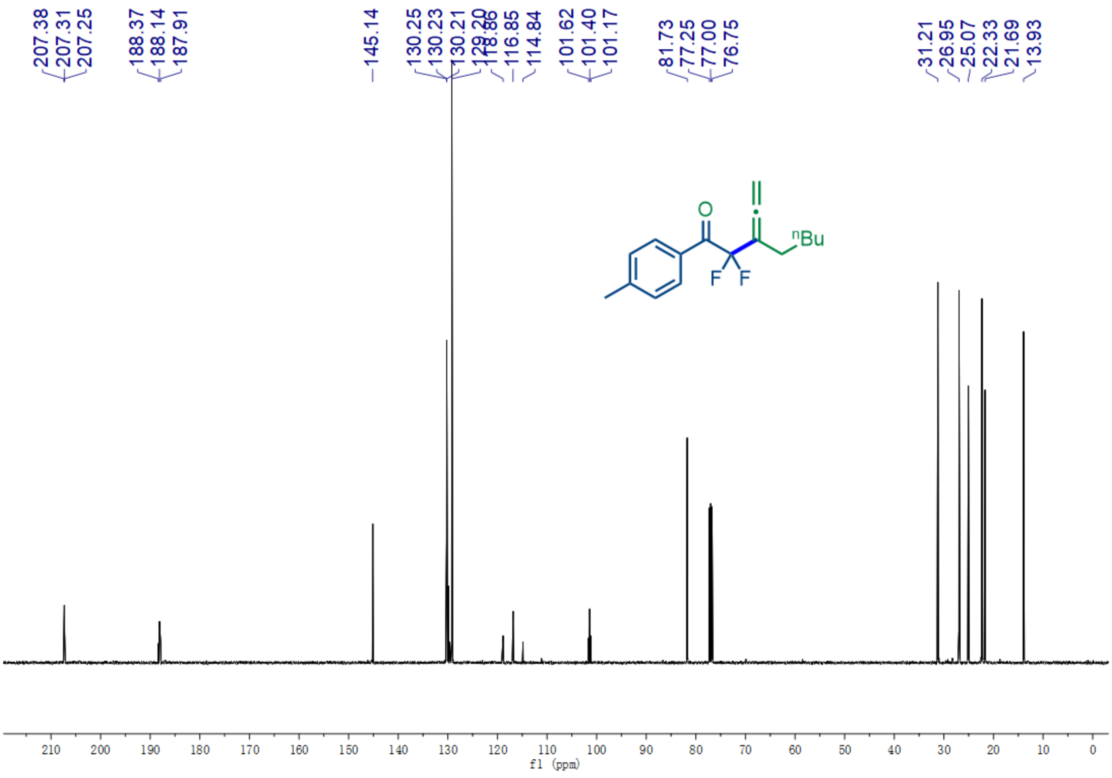

Supplementary Fig. 499 ^{13}C NMR (125 MHz, $CDCl_3$) spectrum of compound **166**

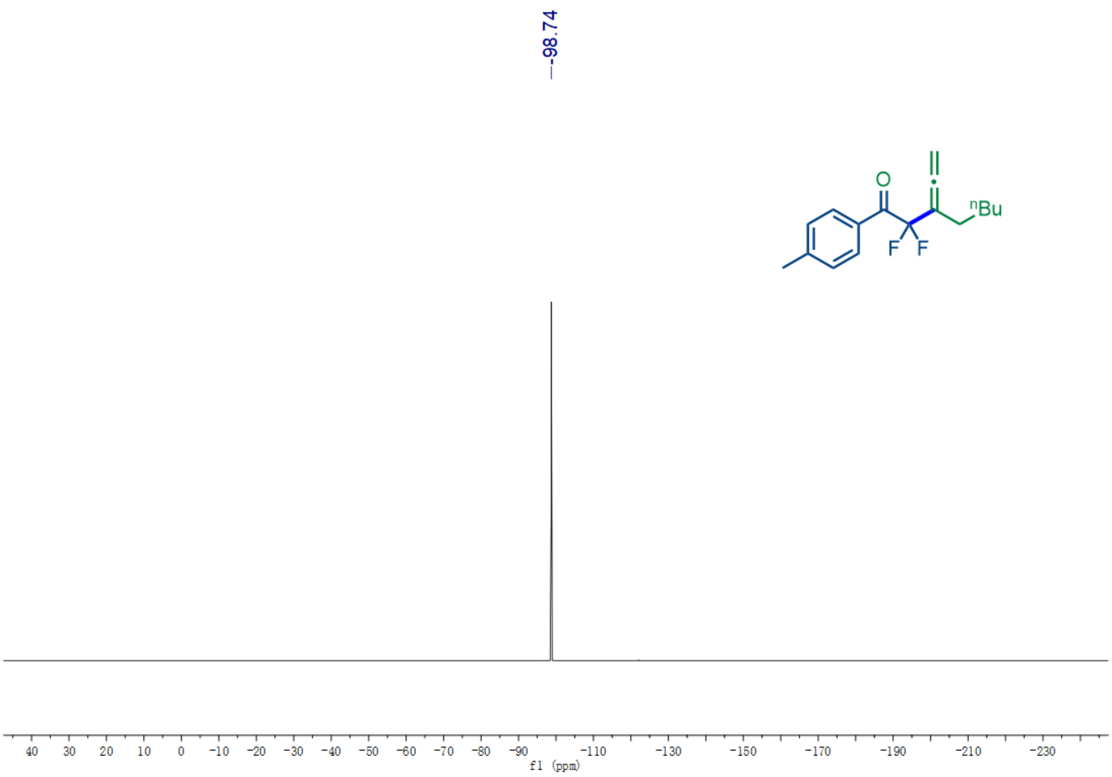

Supplementary Fig. 500 ^{19}F NMR (470 MHz, $CDCl_3$) spectrum of compound **166**

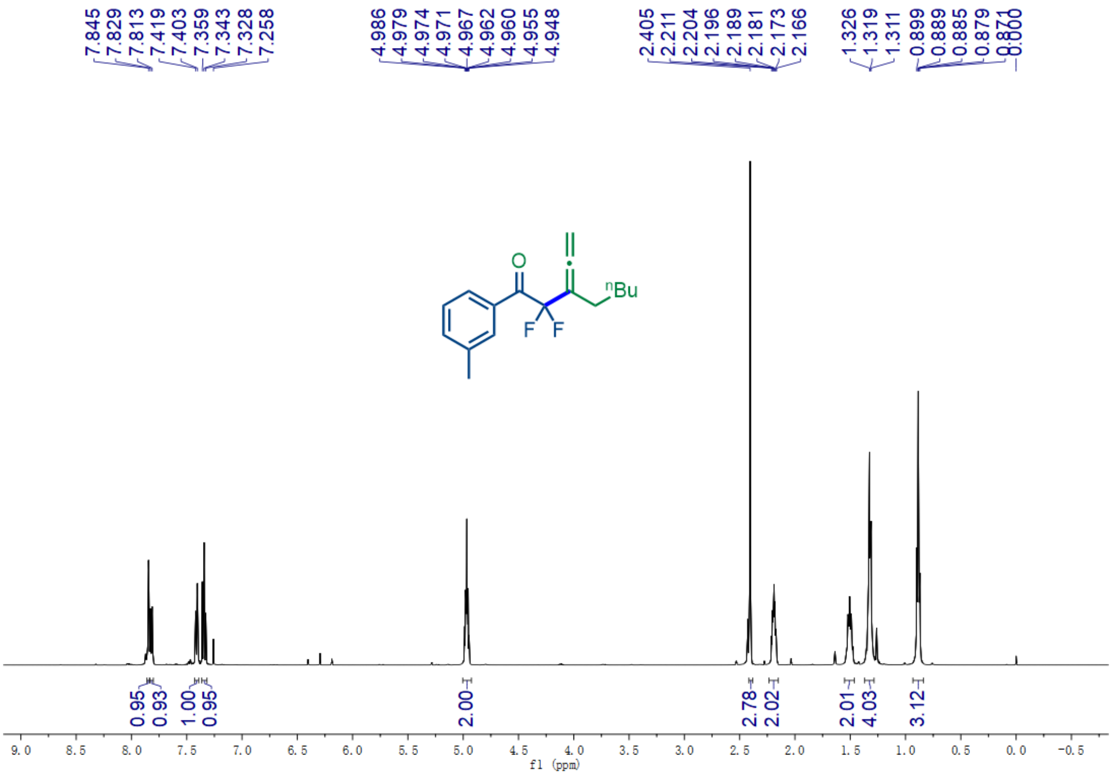

Supplementary Fig. 501 ^{1}H NMR (500 MHz, $CDCl_3$) spectrum of compound **167**

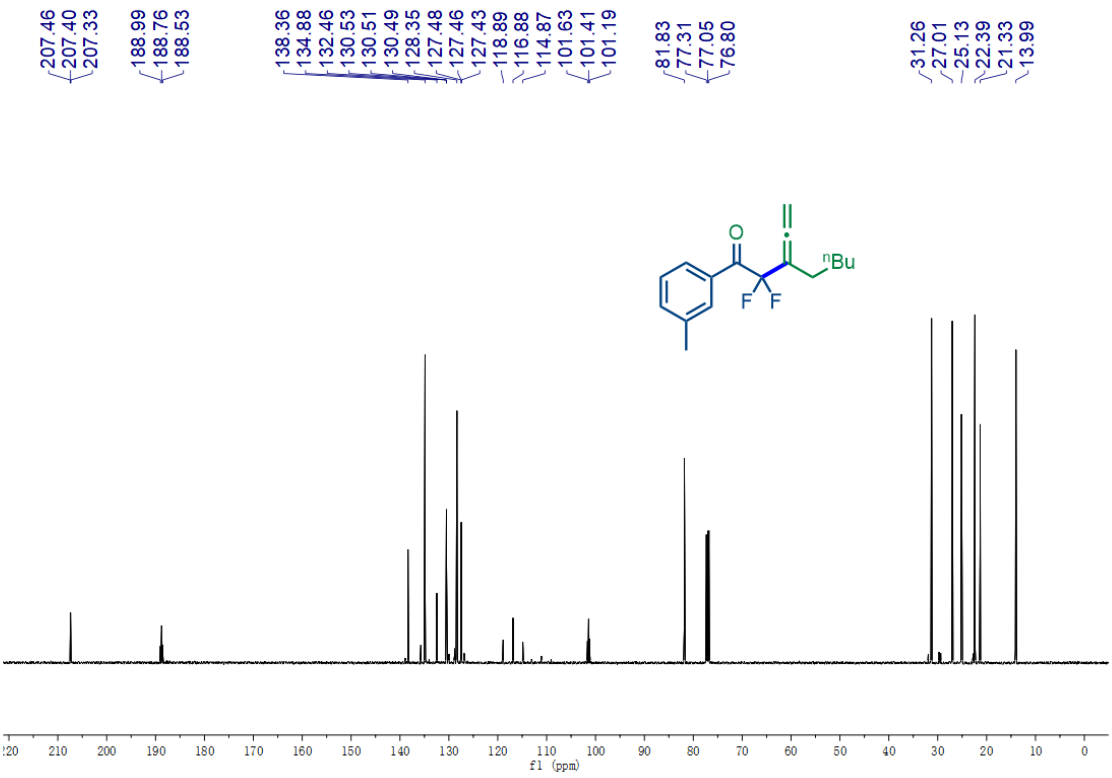

Supplementary Fig. 502 ^{13}C NMR (125 MHz, $CDCl_3$) spectrum of compound **167**

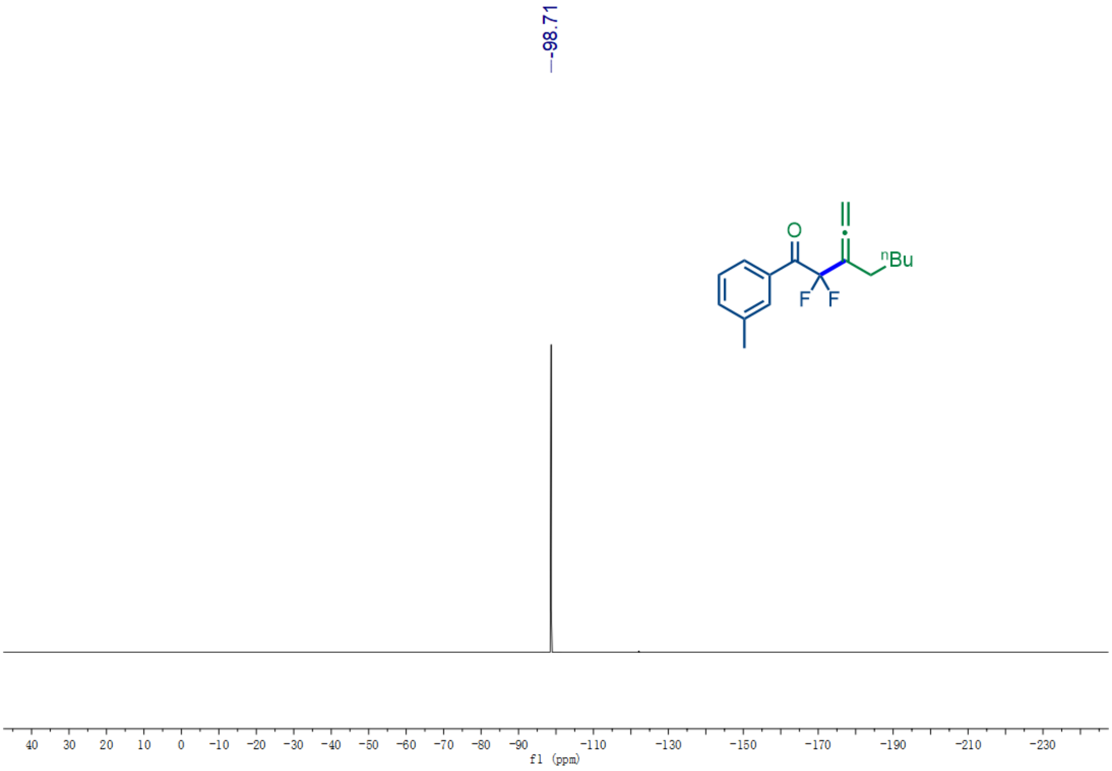

Supplementary Fig. 503 ^{19}F NMR (470 MHz, $CDCl_3$) spectrum of compound **167**

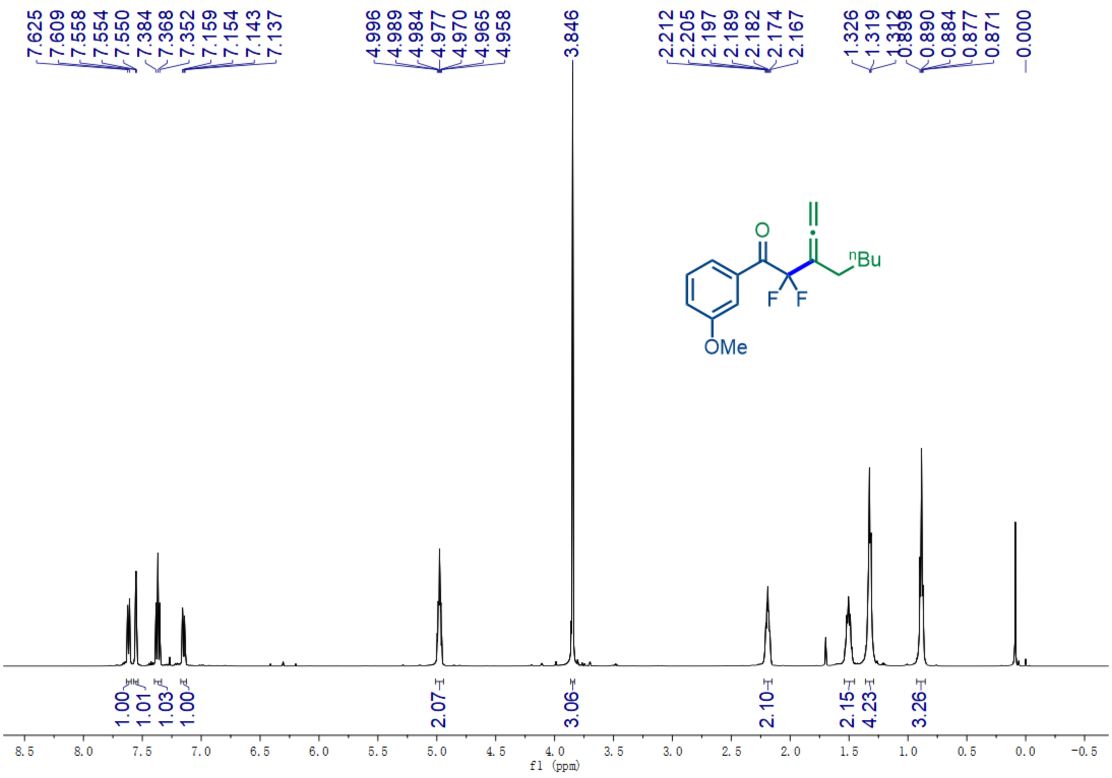

Supplementary Fig. 504 ^{1}H NMR (500 MHz, $CDCl_3$) spectrum of compound **168**

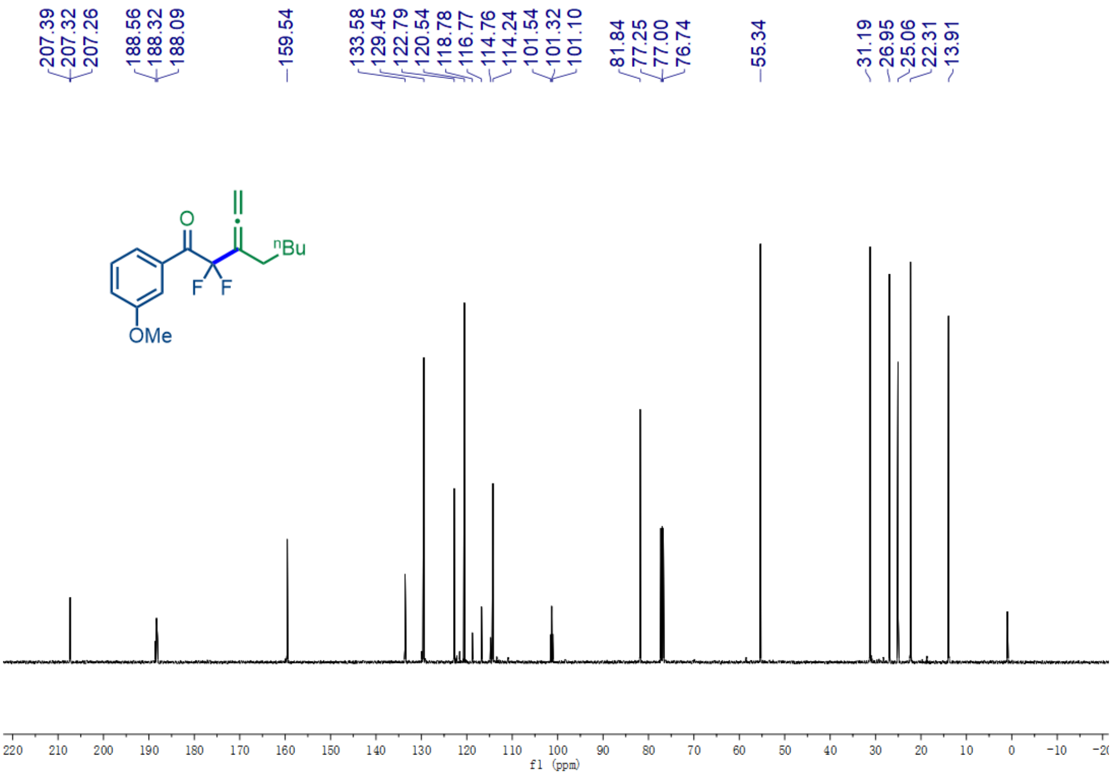

Supplementary Fig. 505 ^{13}C NMR (125 MHz, $CDCl_3$) spectrum of compound **168**

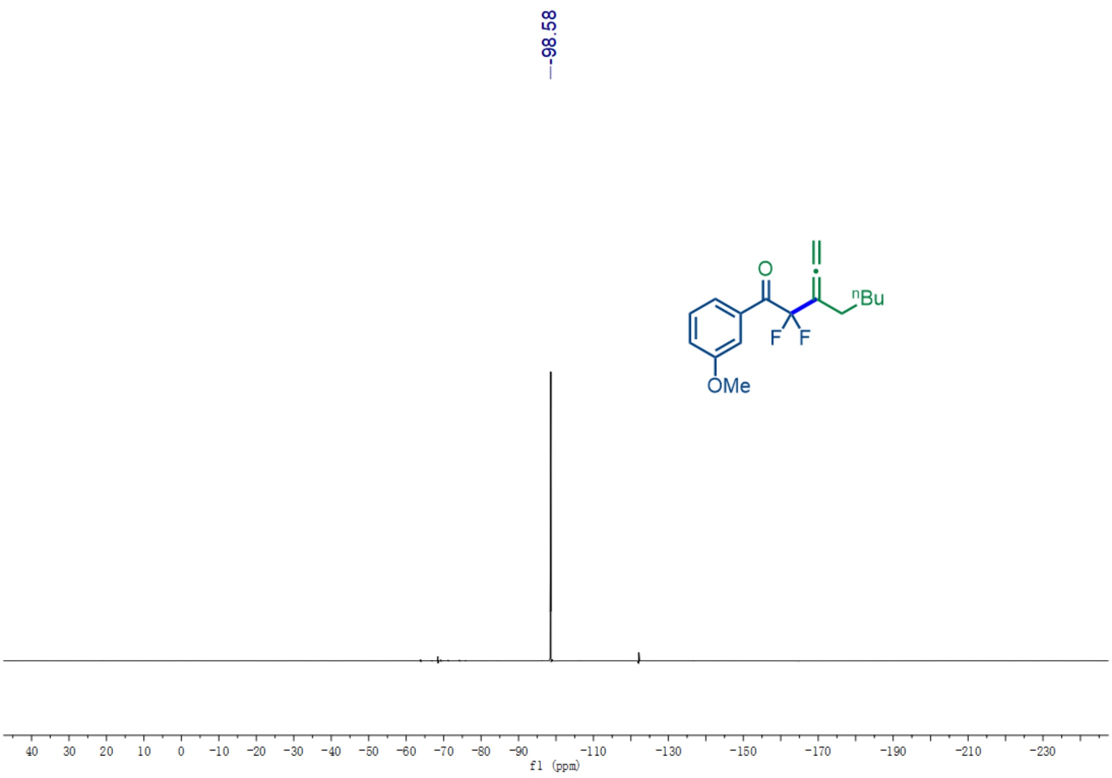

Supplementary Fig. 506 ^{19}F NMR (470 MHz, $CDCl_3$) spectrum of compound **168**

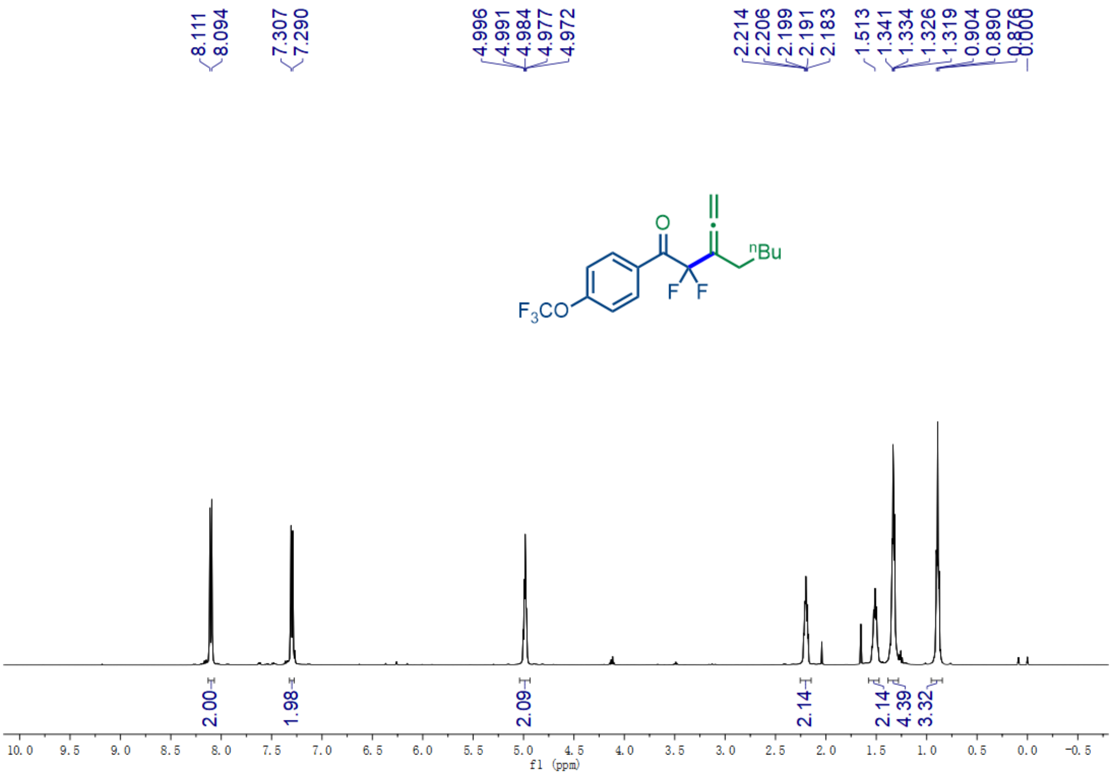

Supplementary Fig. 507 ^{1}H NMR (500 MHz, $CDCl_3$) spectrum of compound **169**

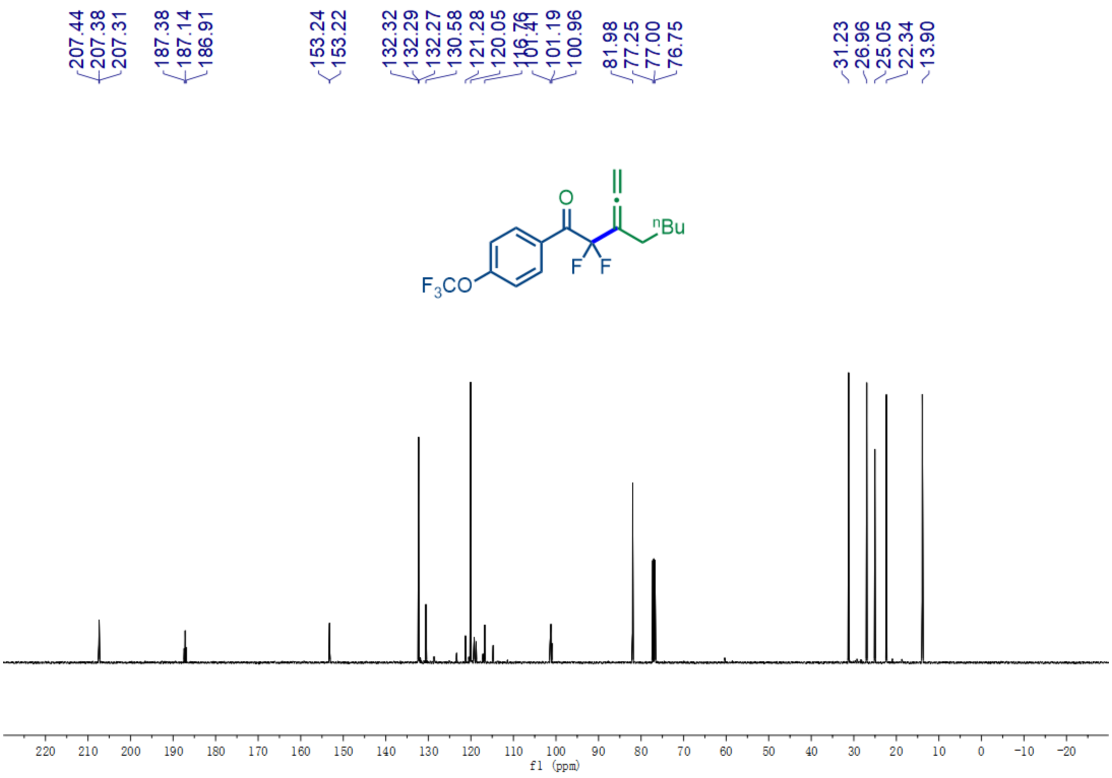

Supplementary Fig. 508 ^{13}C NMR (125 MHz, $CDCl_3$) spectrum of compound **169**

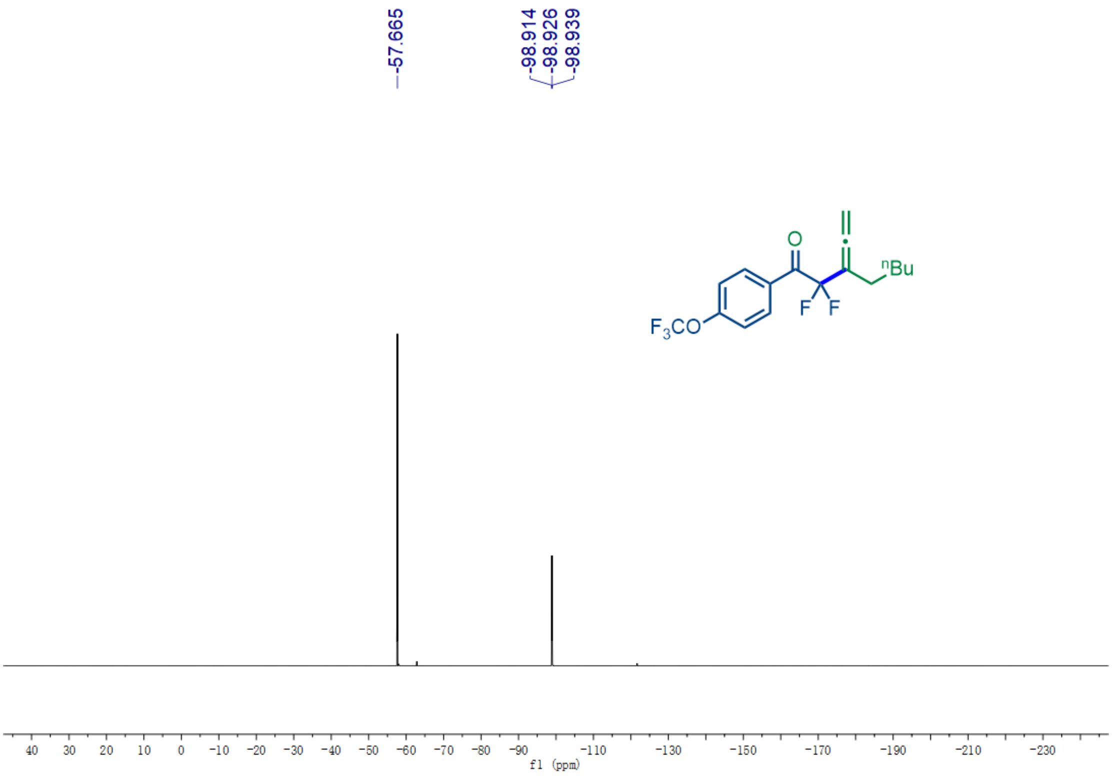

Supplementary Fig. 509 ^{19}F NMR (470 MHz, $CDCl_3$) spectrum of compound **169**

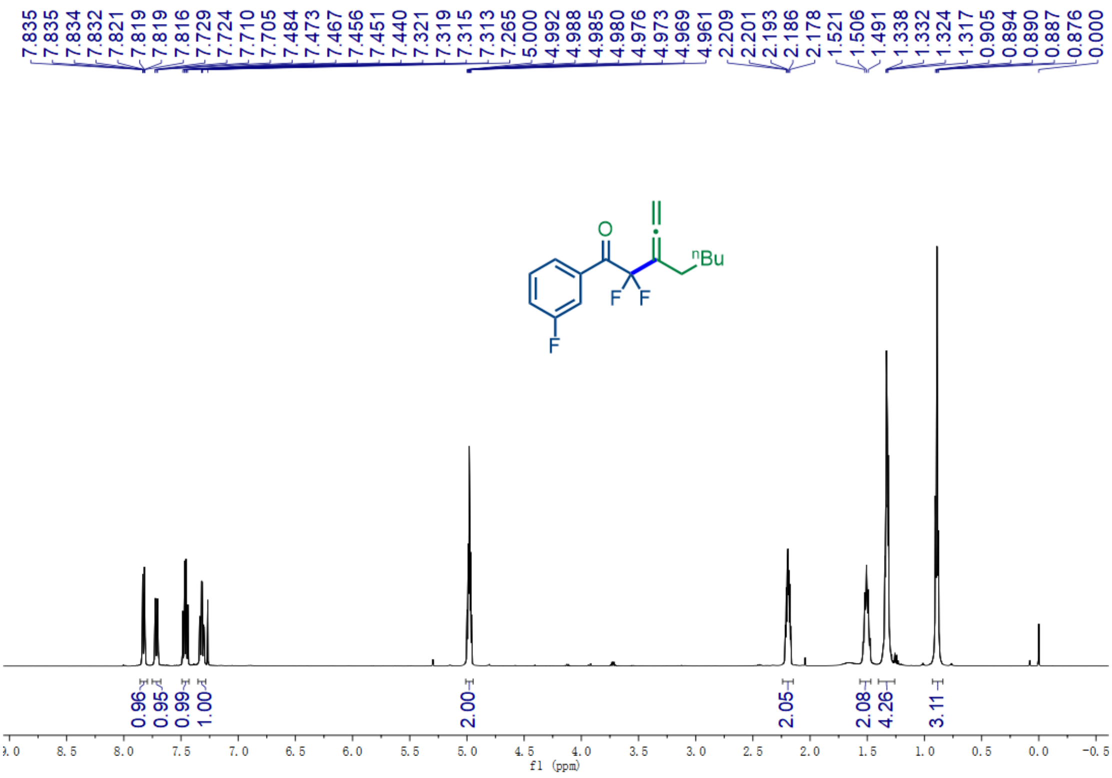

Supplementary Fig. 510 ^{1}H NMR (500 MHz, $CDCl_3$) spectrum of compound **170**

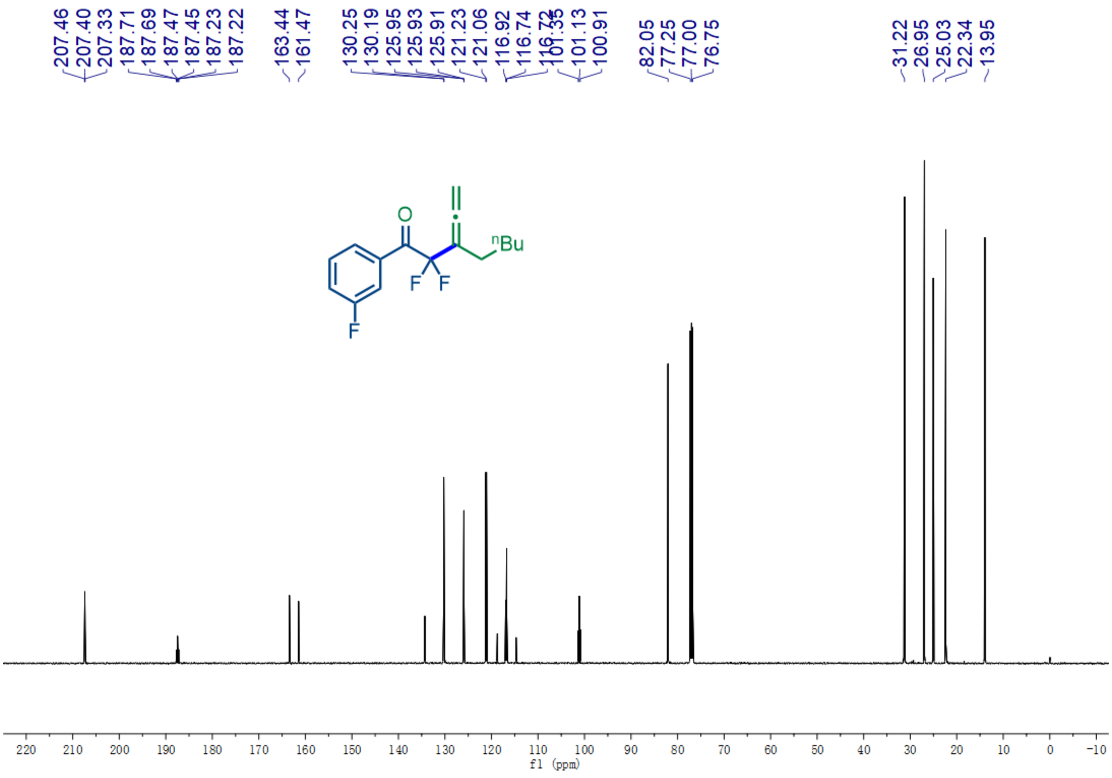

Supplementary Fig. 511 ^{13}C NMR (125 MHz, $CDCl_3$) spectrum of compound **170**

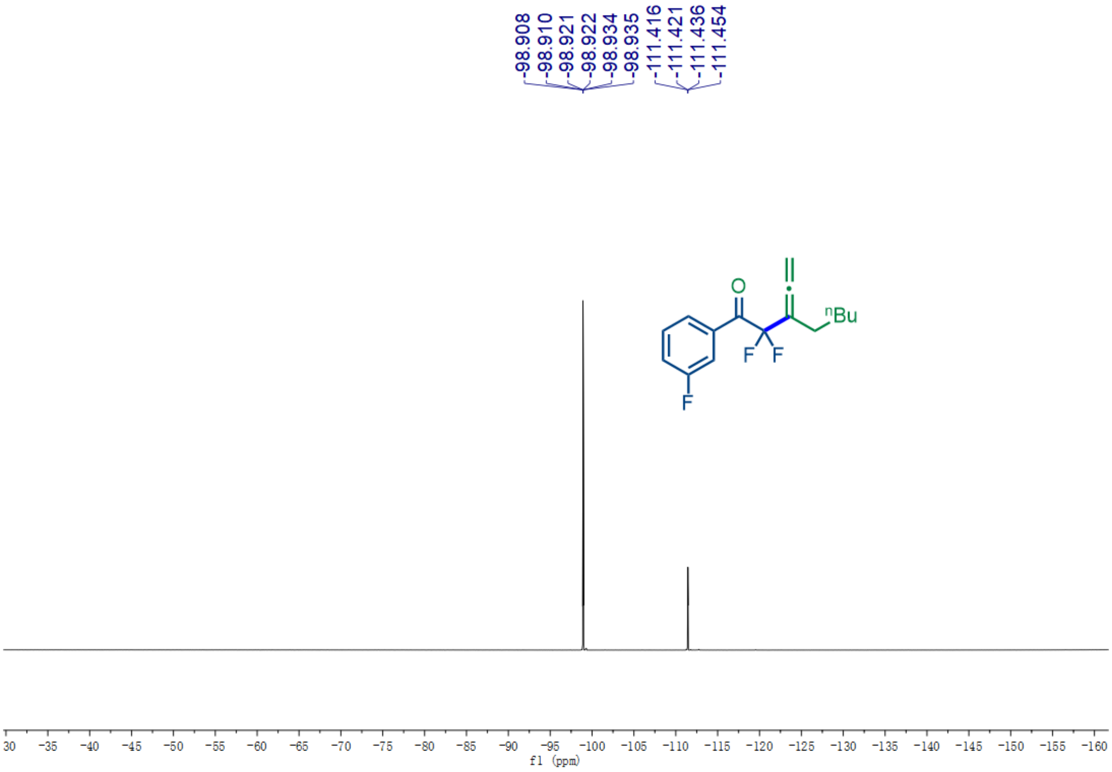

Supplementary Fig. 512 ^{19}F NMR (470 MHz, $CDCl_3$) spectrum of compound **170**

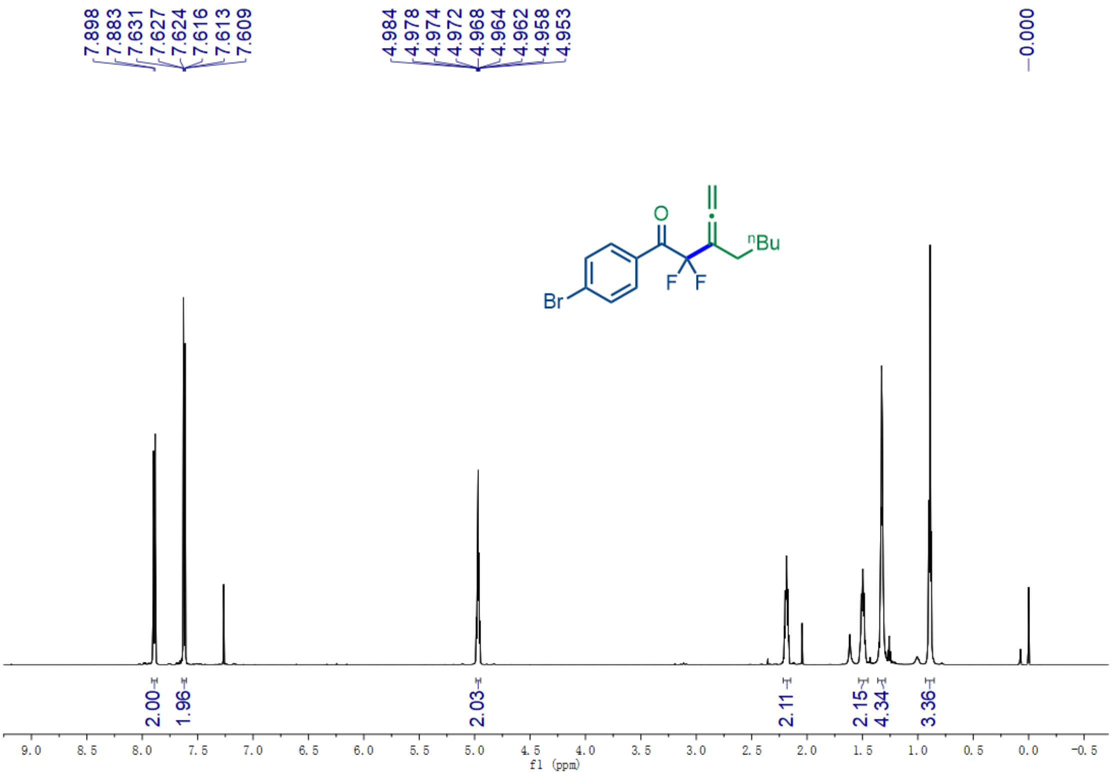

Supplementary Fig. 513 ^{1}H NMR (600 MHz, $CDCl_3$) spectrum of compound **171**

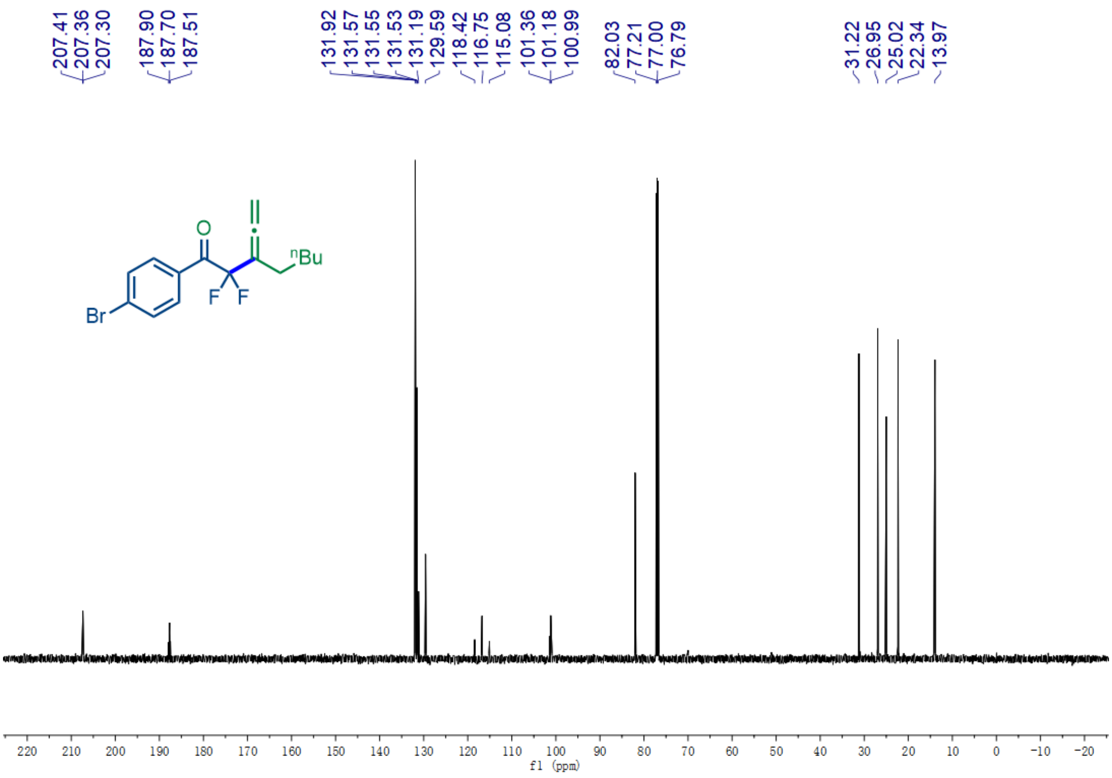

Supplementary Fig. 514 ^{13}C NMR (150 MHz, $CDCl_3$) spectrum of compound **171**

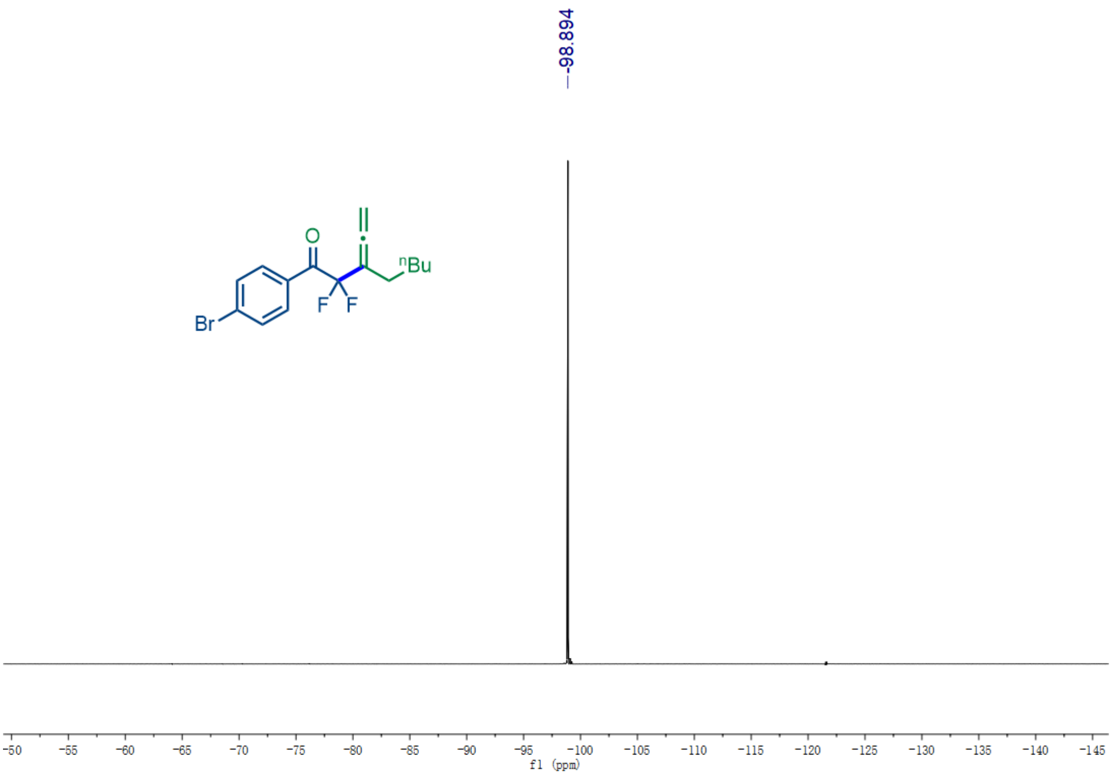

Supplementary Fig. 515 ^{19}F NMR (564 MHz, $CDCl_3$) spectrum of compound **171**

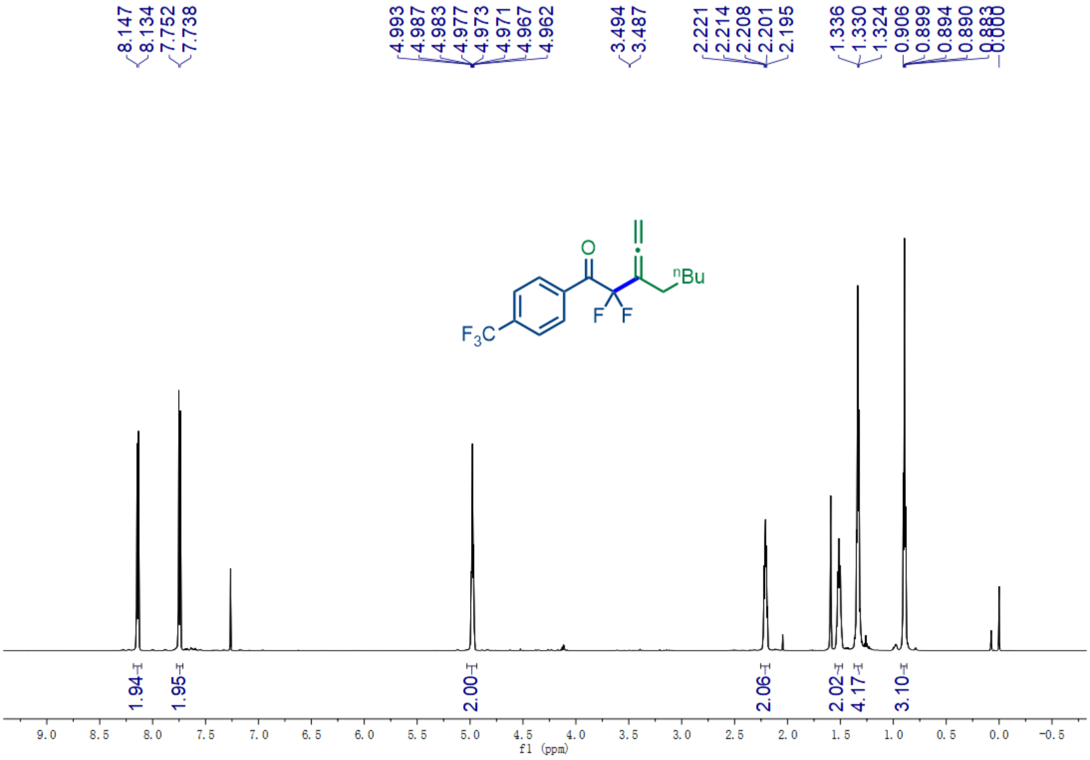

Supplementary Fig. 516 ^{1}H NMR (600 MHz, $CDCl_3$) spectrum of compound **172**

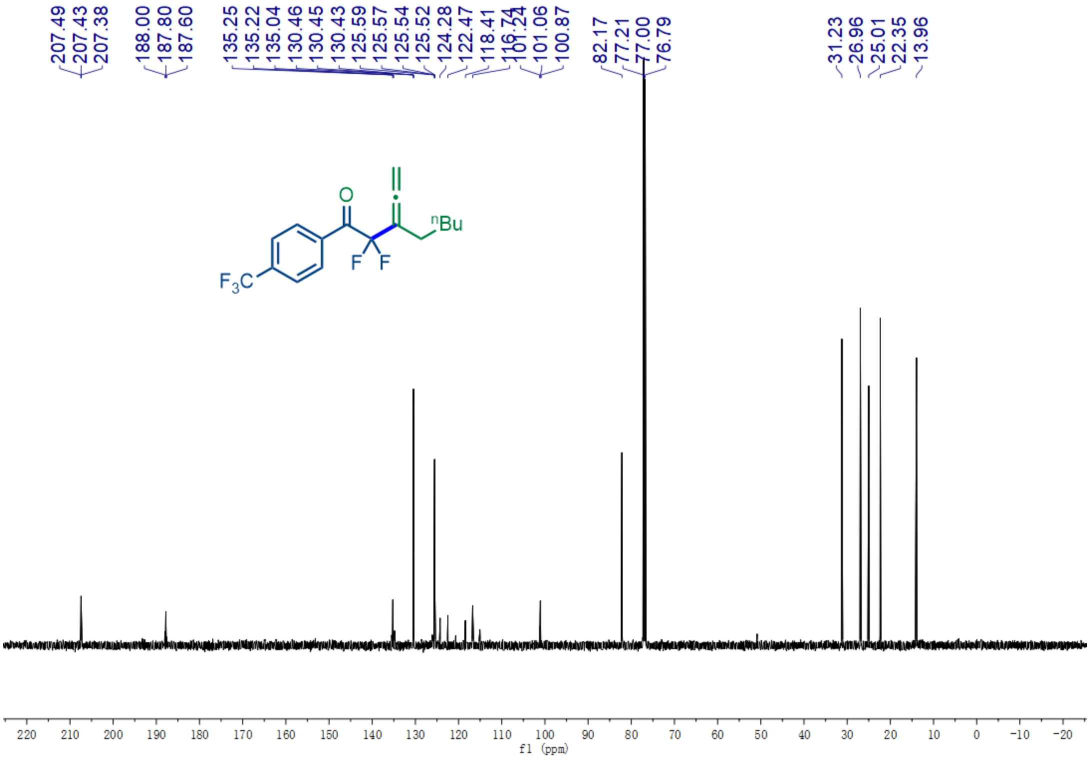

Supplementary Fig. 517 ^{13}C NMR (150 MHz, $CDCl_3$) spectrum of compound **172**

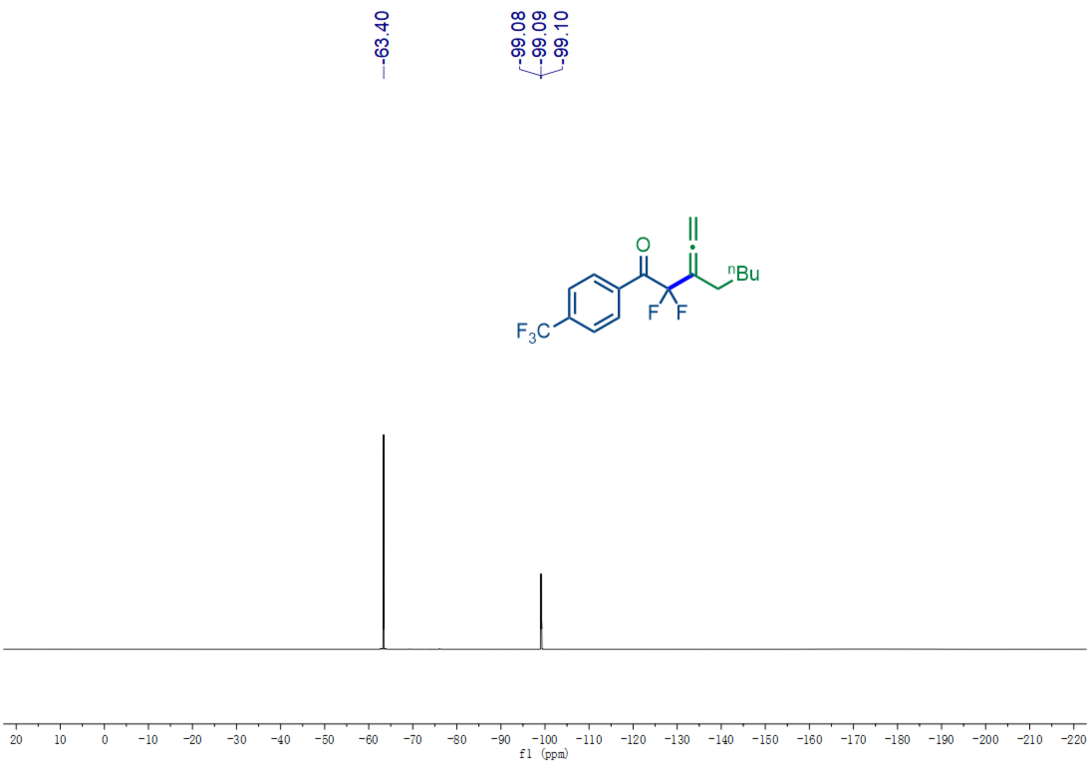

Supplementary Fig. 518 ^{19}F NMR (564 MHz, $CDCl_3$) spectrum of compound **172**

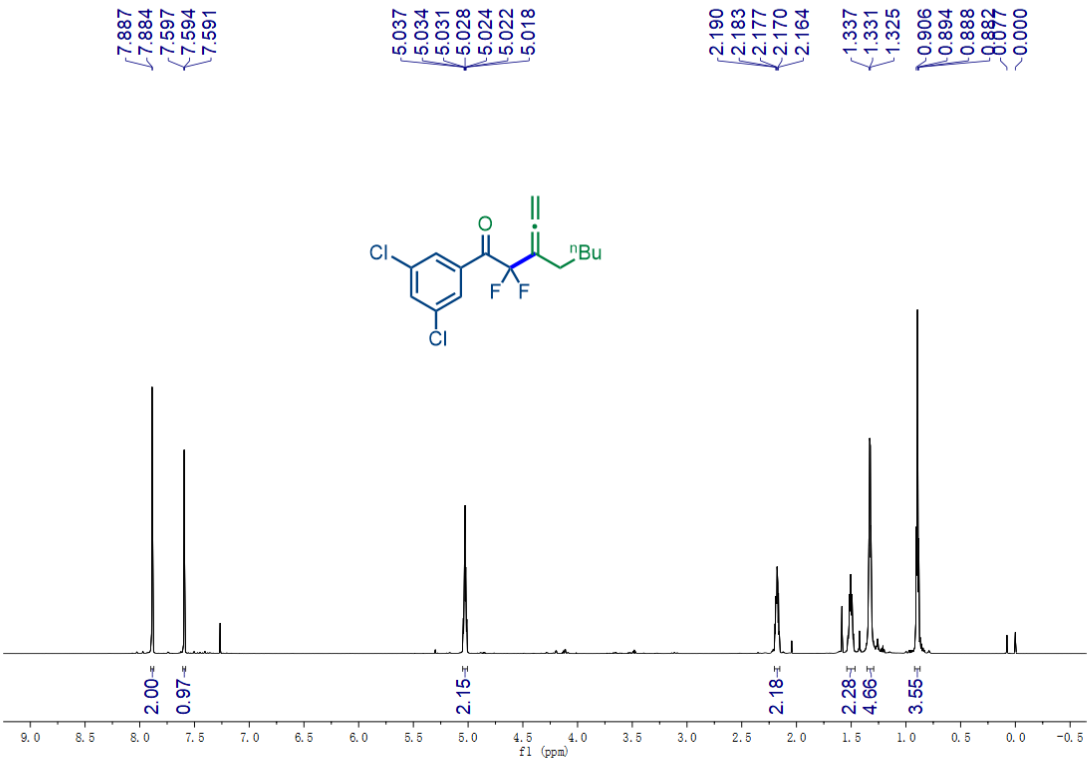

Supplementary Fig. 519 ^{1}H NMR (600 MHz, $CDCl_3$) spectrum of compound **173**

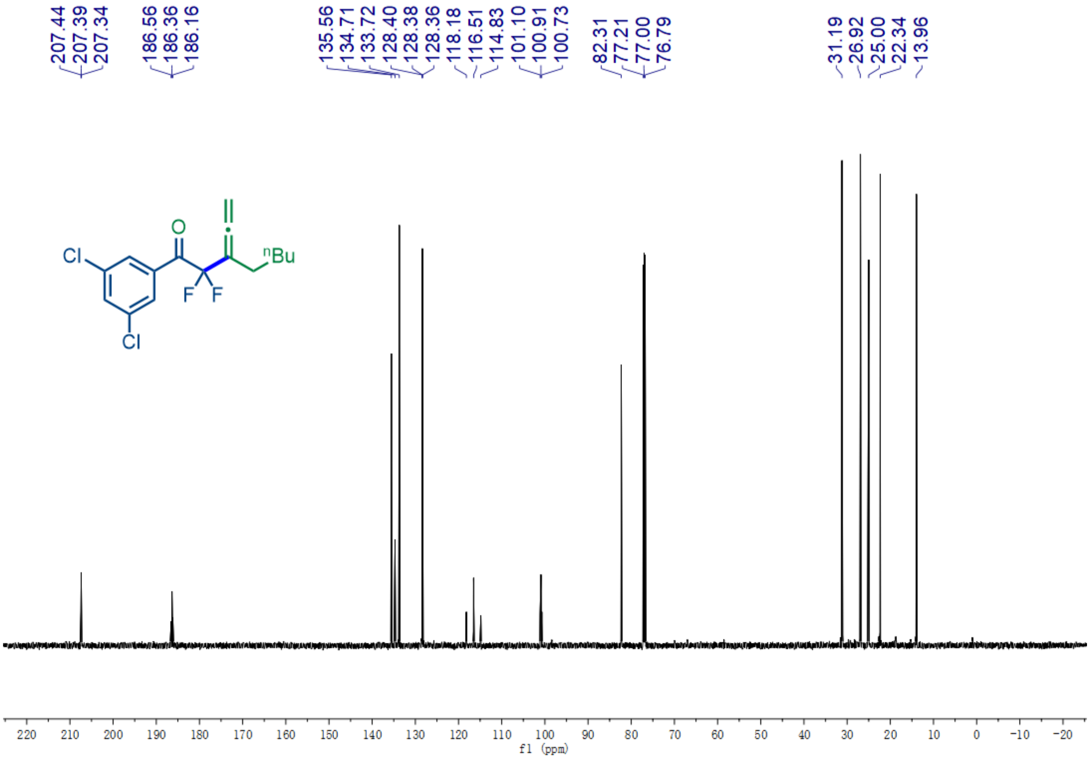

Supplementary Fig. 520 ^{13}C NMR (150 MHz, $CDCl_3$) spectrum of compound **173**

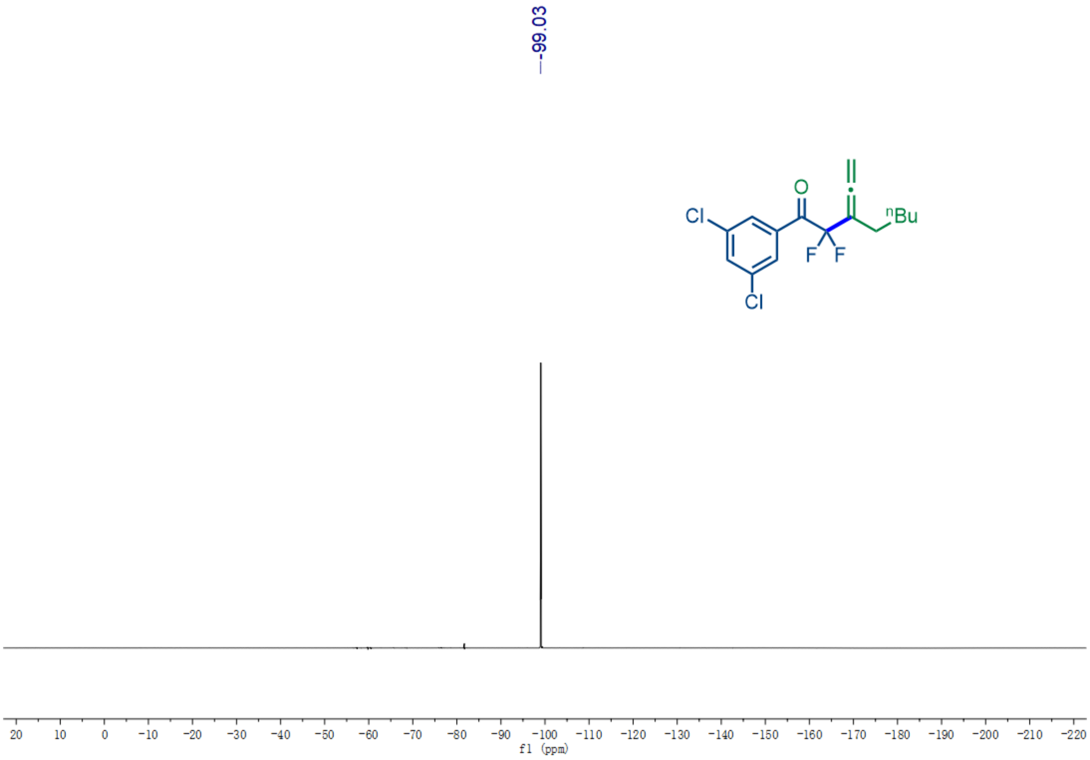

Supplementary Fig. 521 ^{19}F NMR (564 MHz, $CDCl_3$) spectrum of compound **173**

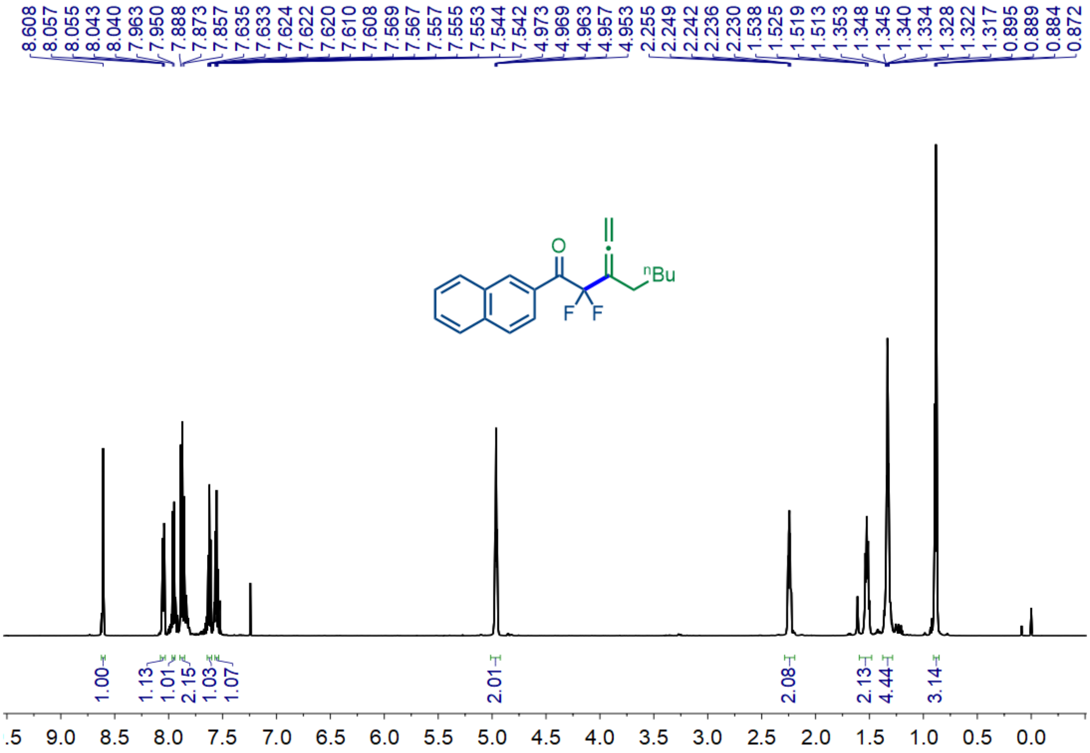

Supplementary Fig. 522 ^{1}H NMR (600 MHz, $CDCl_3$) spectrum of compound **174**

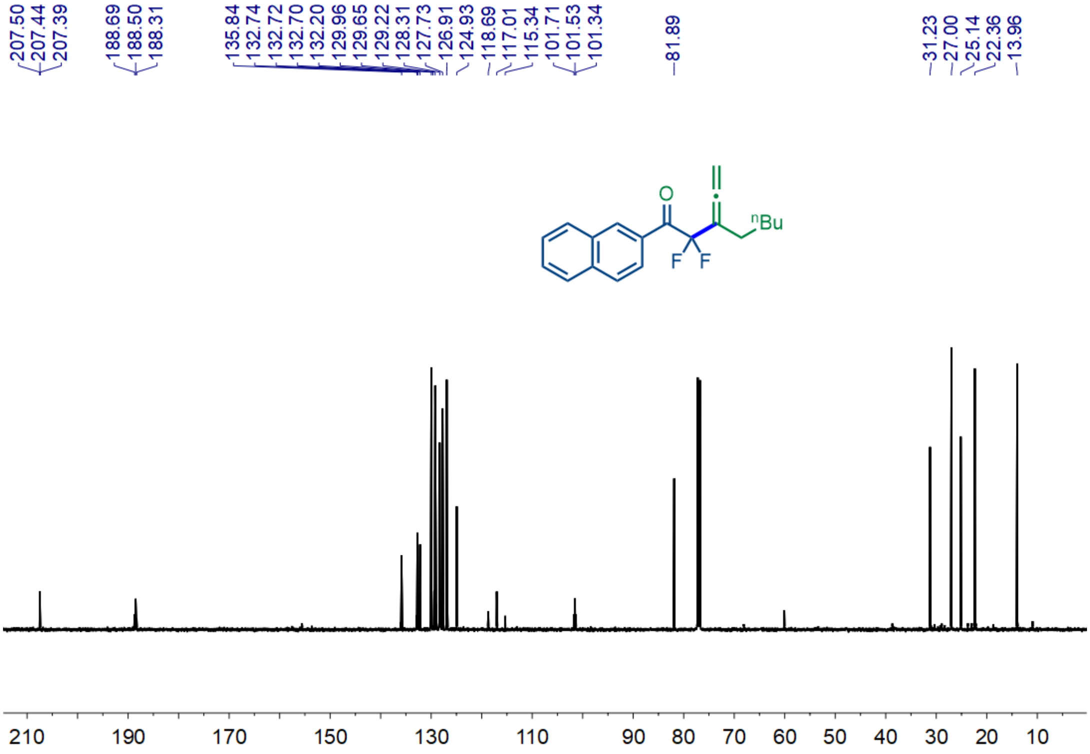

Supplementary Fig. 523 ^{13}C NMR (150 MHz, $CDCl_3$) spectrum of compound **174**

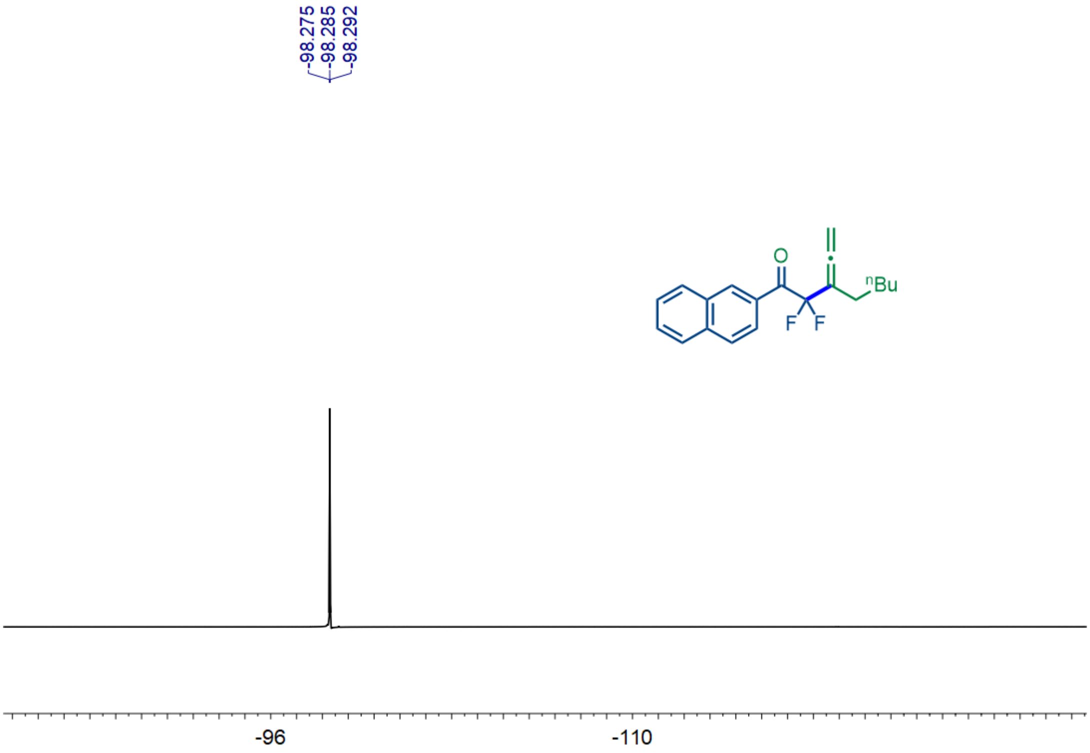

Supplementary Fig. 524 ^{19}F NMR (564 MHz, $CDCl_3$) spectrum of compound **174**

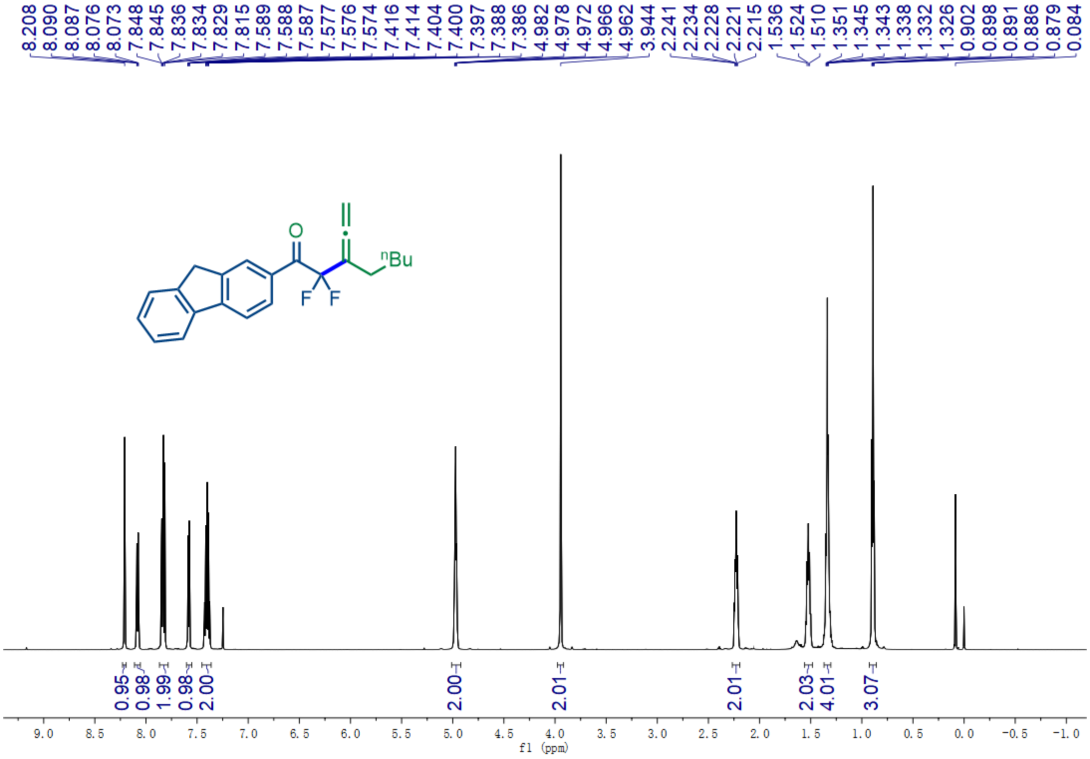

Supplementary Fig. 525 ^{1}H NMR (600 MHz, $CDCl_3$) spectrum of compound **175**

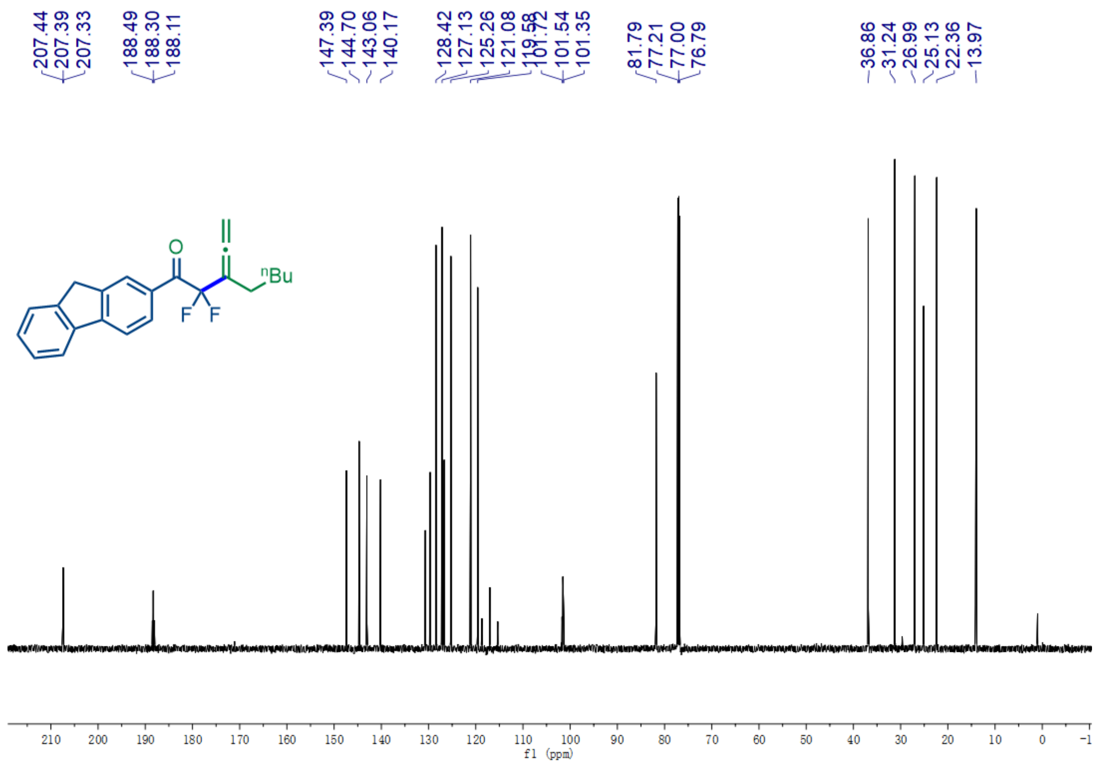

Supplementary Fig. 526 ^{13}C NMR (150 MHz, $CDCl_3$) spectrum of compound **175**

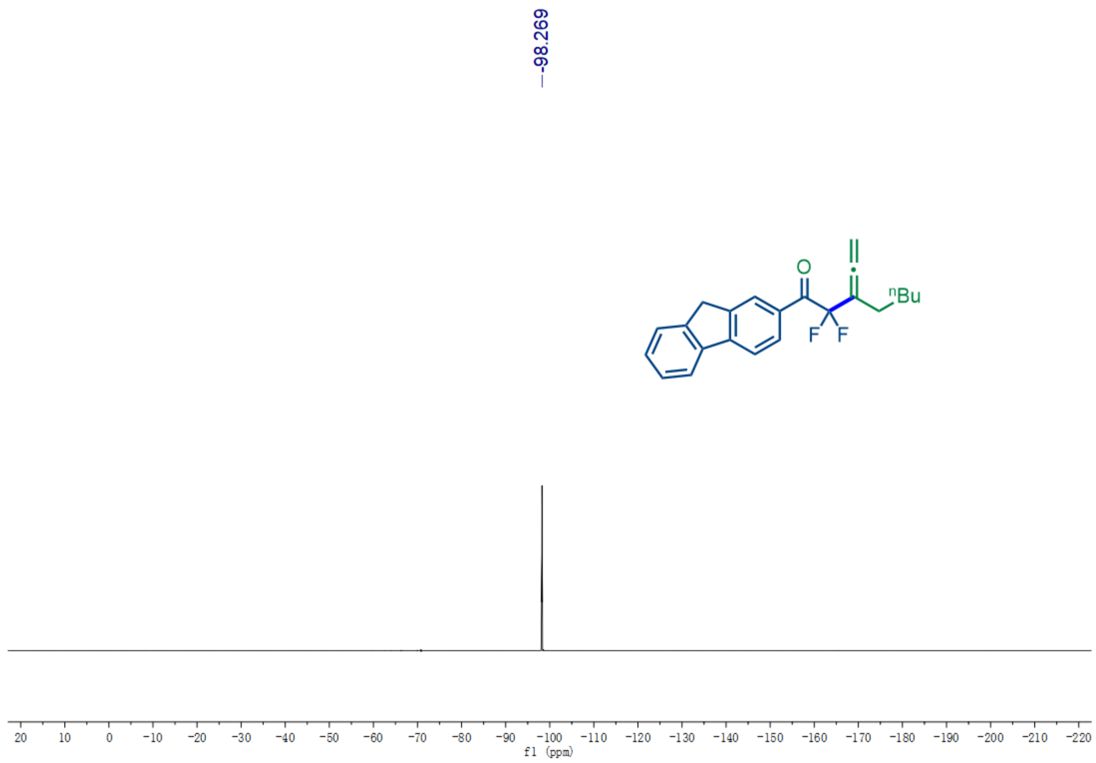

Supplementary Fig. 527 ^{19}F NMR (564 MHz, $CDCl_3$) spectrum of compound **175**

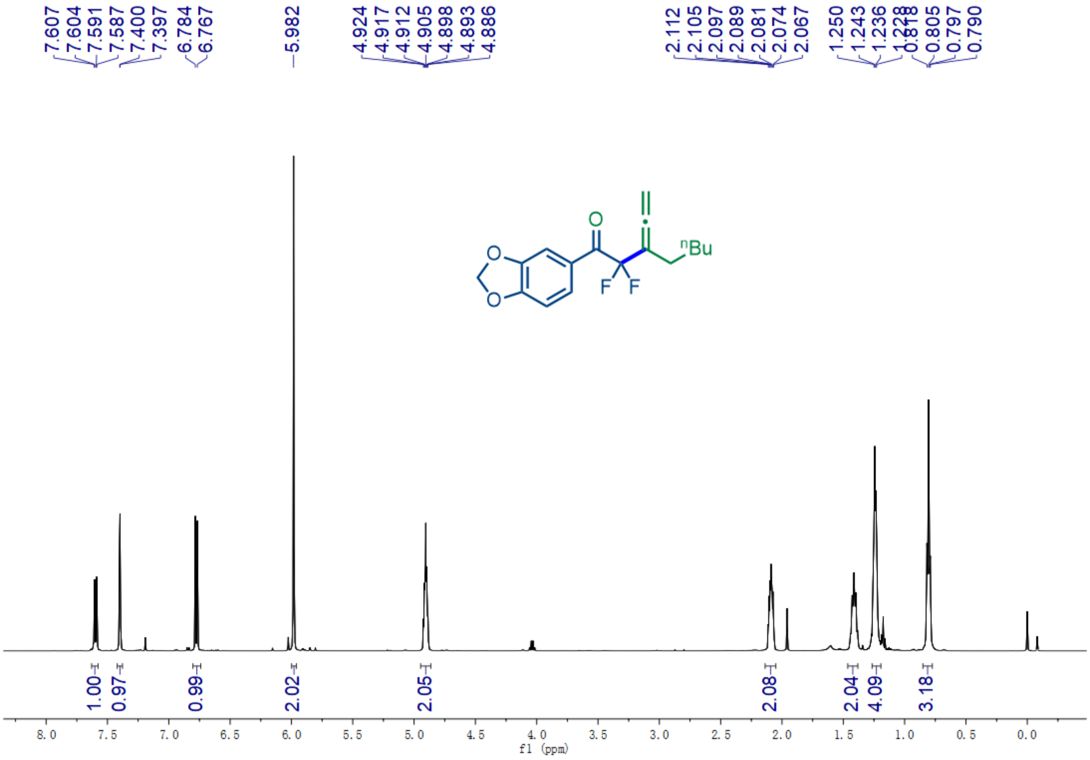

Supplementary Fig. 528 ^{1}H NMR (500 MHz, $CDCl_3$) spectrum of compound **176**

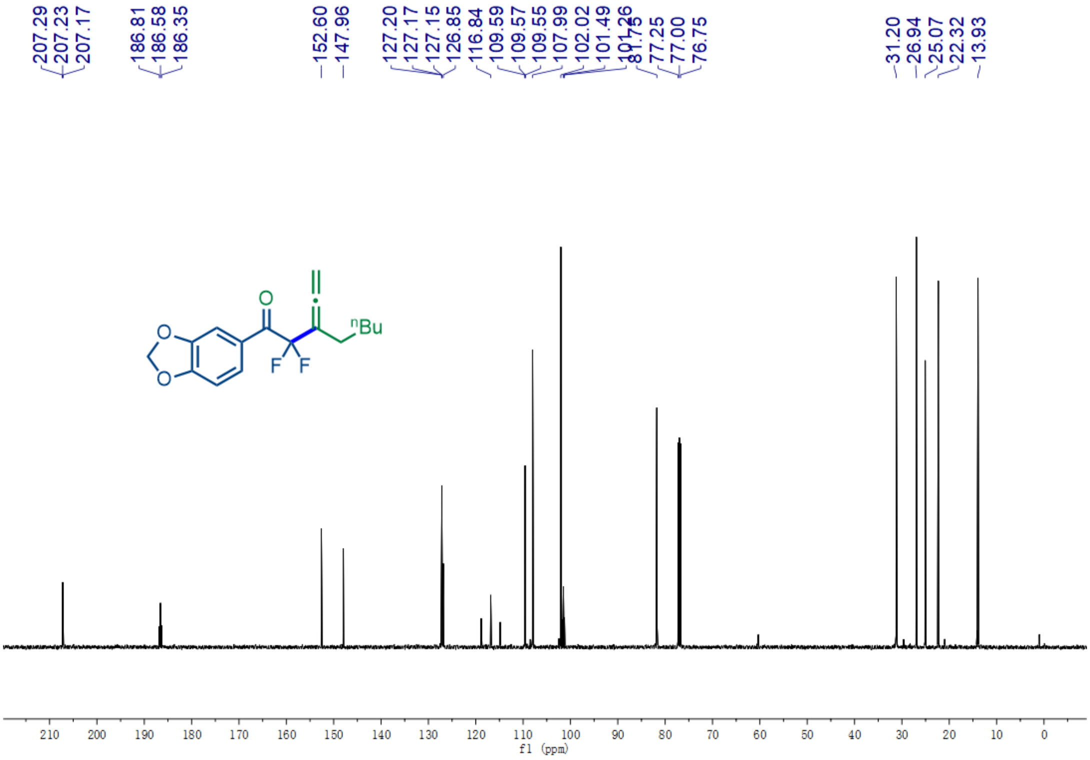

Supplementary Fig. 529 ^{13}C NMR (125 MHz, $CDCl_3$) spectrum of compound **176**

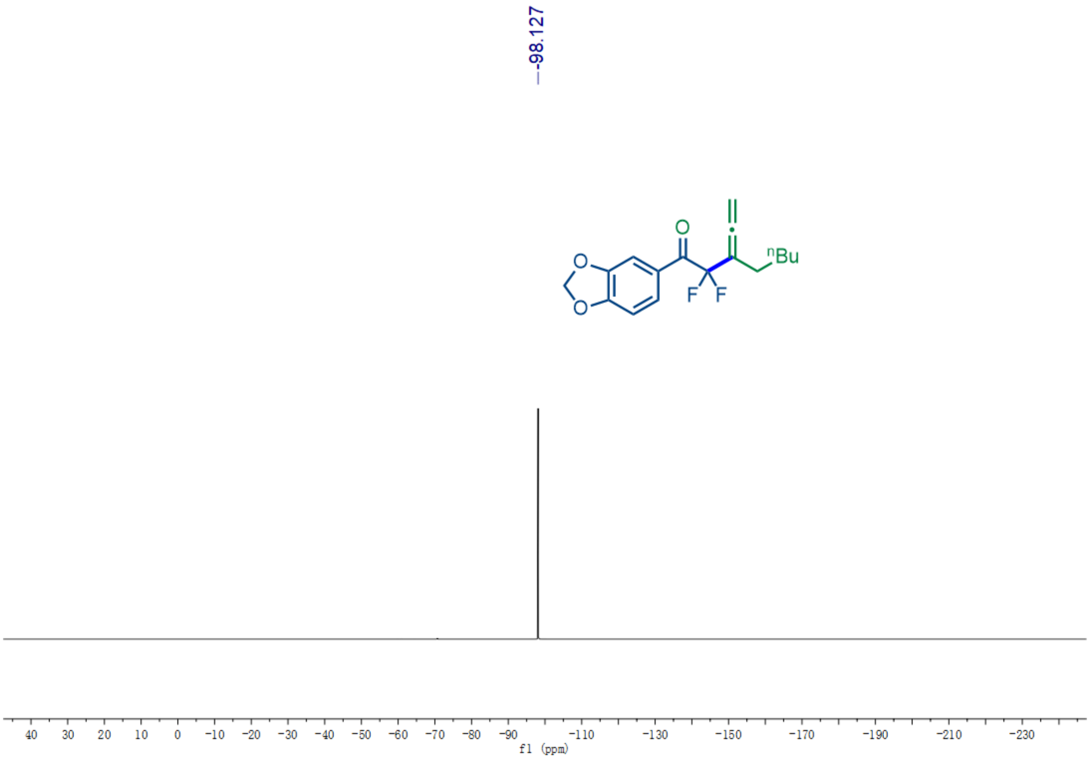

Supplementary Fig. 530 ^{19}F NMR (470 MHz, $CDCl_3$) spectrum of compound **176**

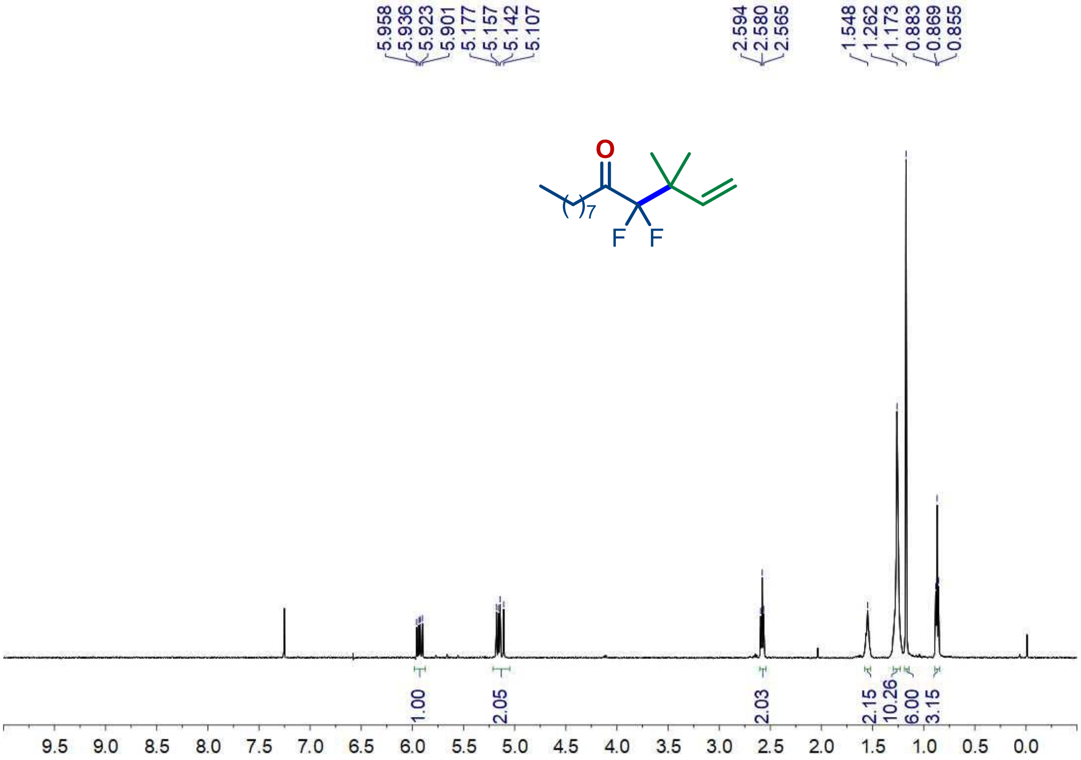

Supplementary Fig. 531 ^{1}H NMR (500 MHz, $CDCl_3$) spectrum of compound **177**

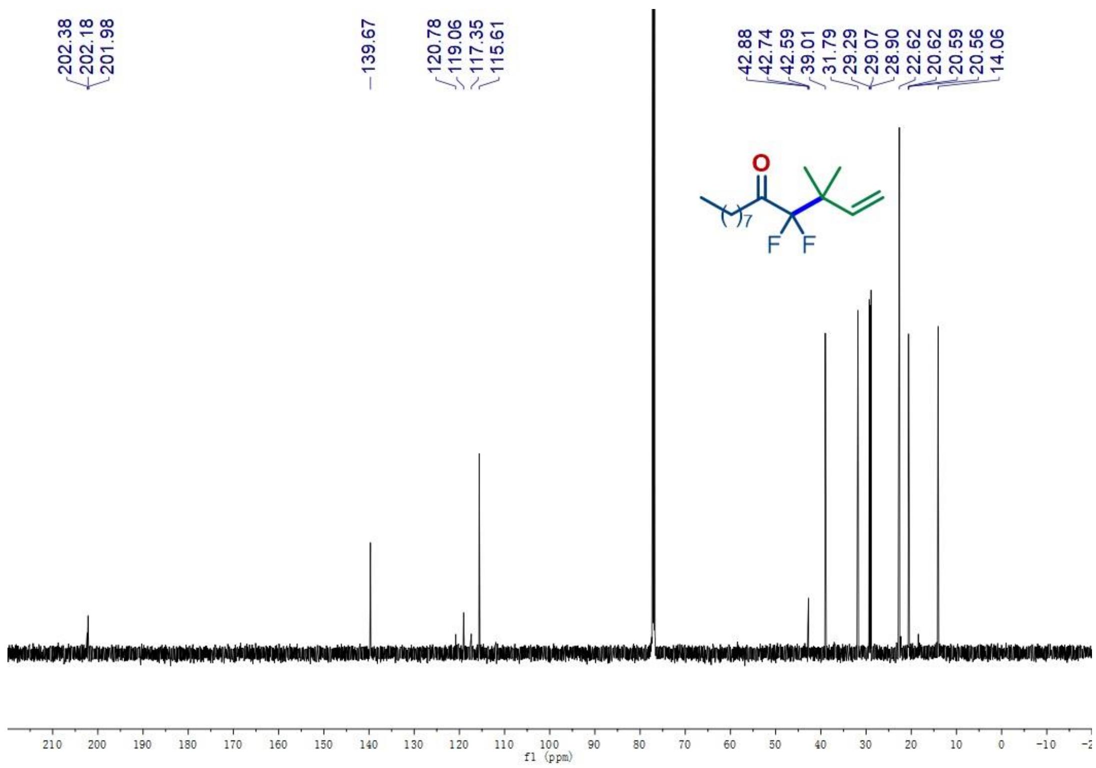

Supplementary Fig. 532 ^{13}C NMR (150 MHz, $CDCl_3$) spectrum of compound **177**

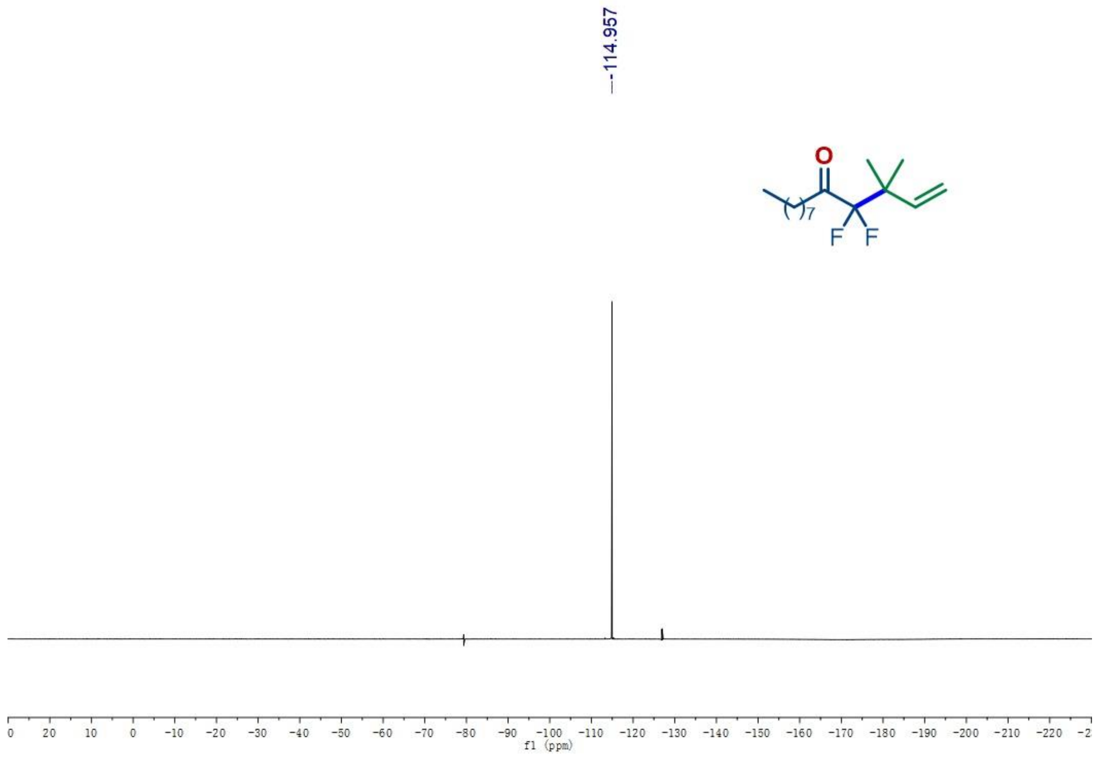

Supplementary Fig. 533 ^{19}F NMR (564 MHz, $CDCl_3$) spectrum of compound **177**

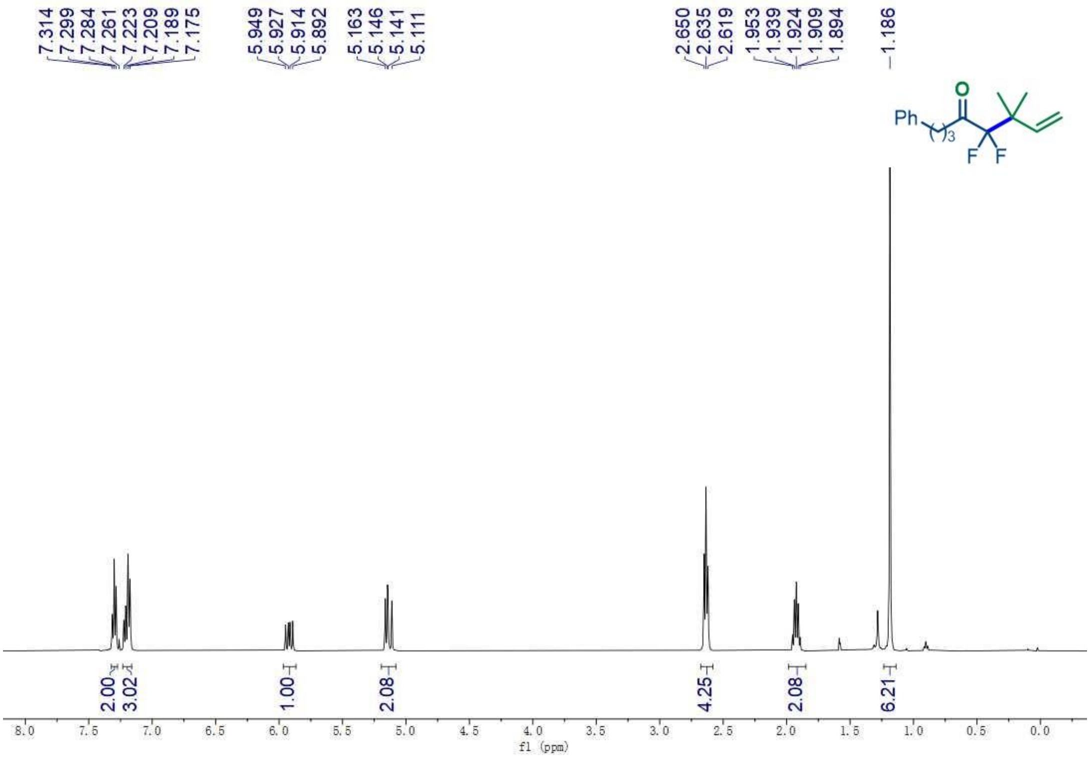

Supplementary Fig. 534 ^{1}H NMR (500 MHz, $CDCl_3$) spectrum of compound **178**

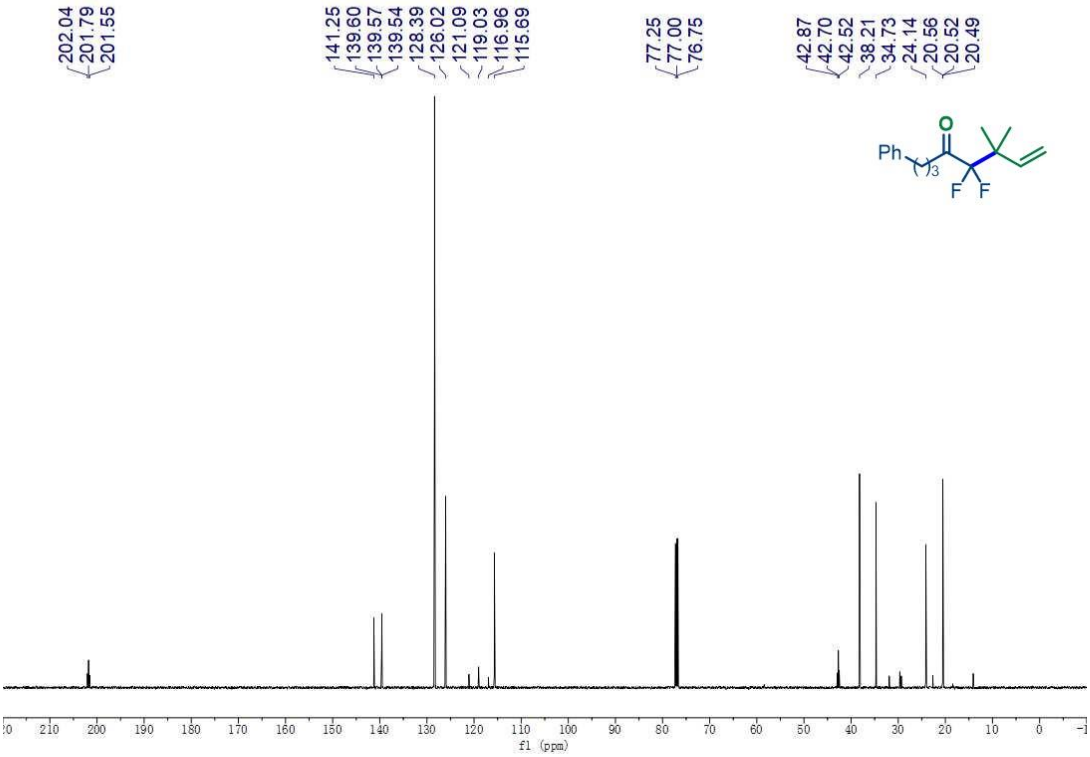

Supplementary Fig. 535 ^{13}C NMR (125 MHz, $CDCl_3$) spectrum of compound **178**

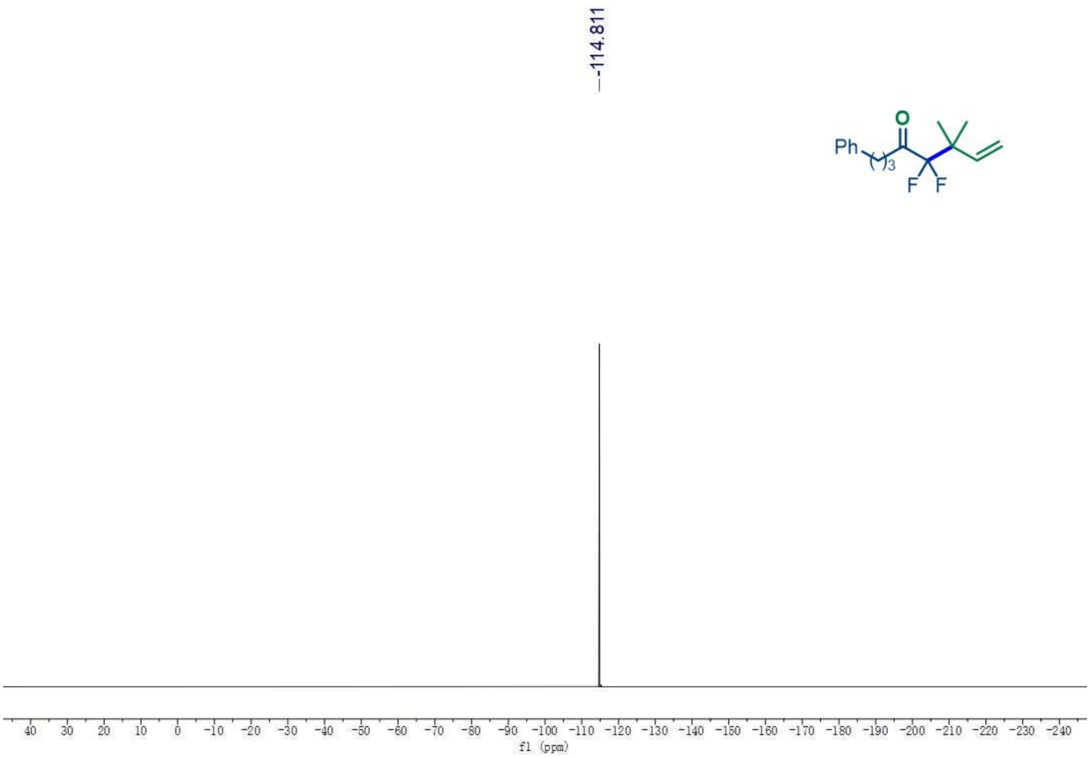

Supplementary Fig. 536 ^{19}F NMR (470 MHz, $CDCl_3$) spectrum of compound **178**

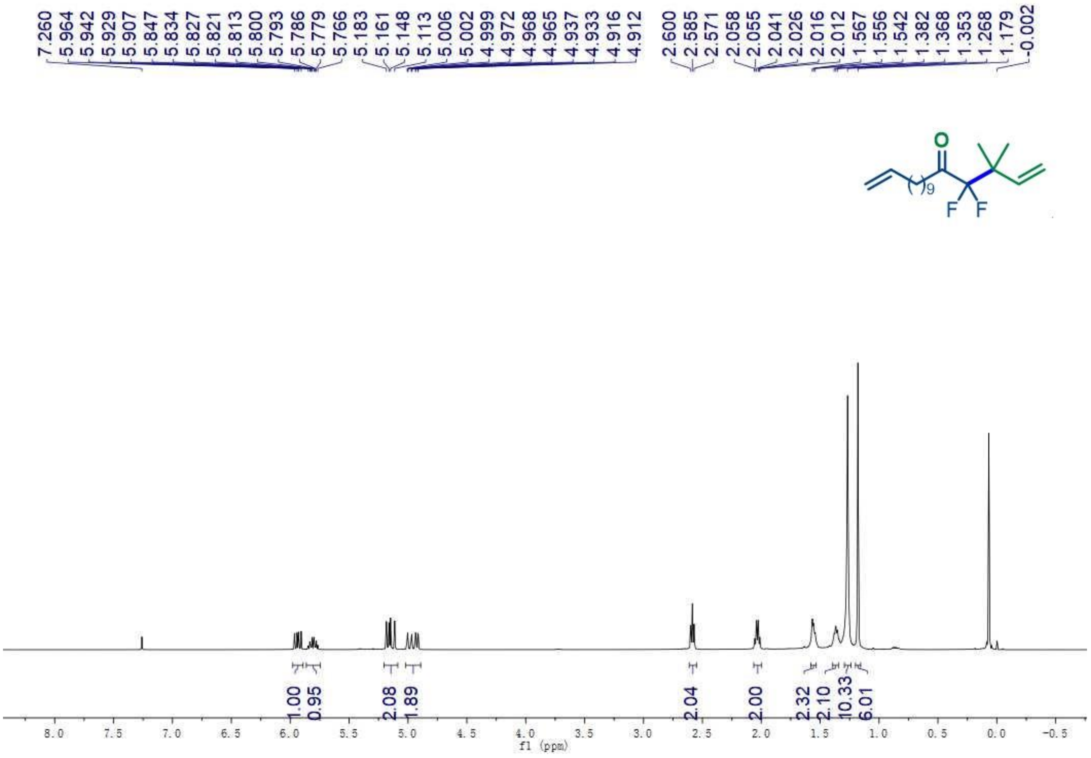

Supplementary Fig. 537 ^{1}H NMR (500 MHz, $CDCl_3$) spectrum of compound **179**

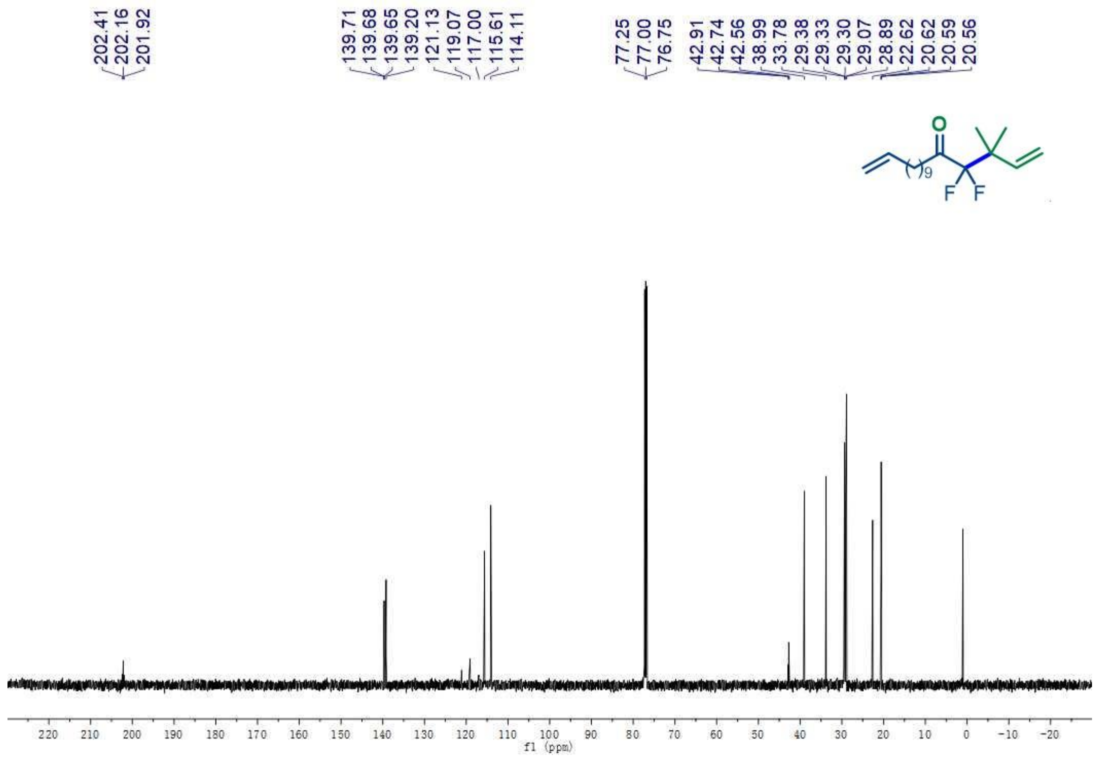

Supplementary Fig. 538 ^{13}C NMR (125 MHz, $CDCl_3$) spectrum of compound **179**

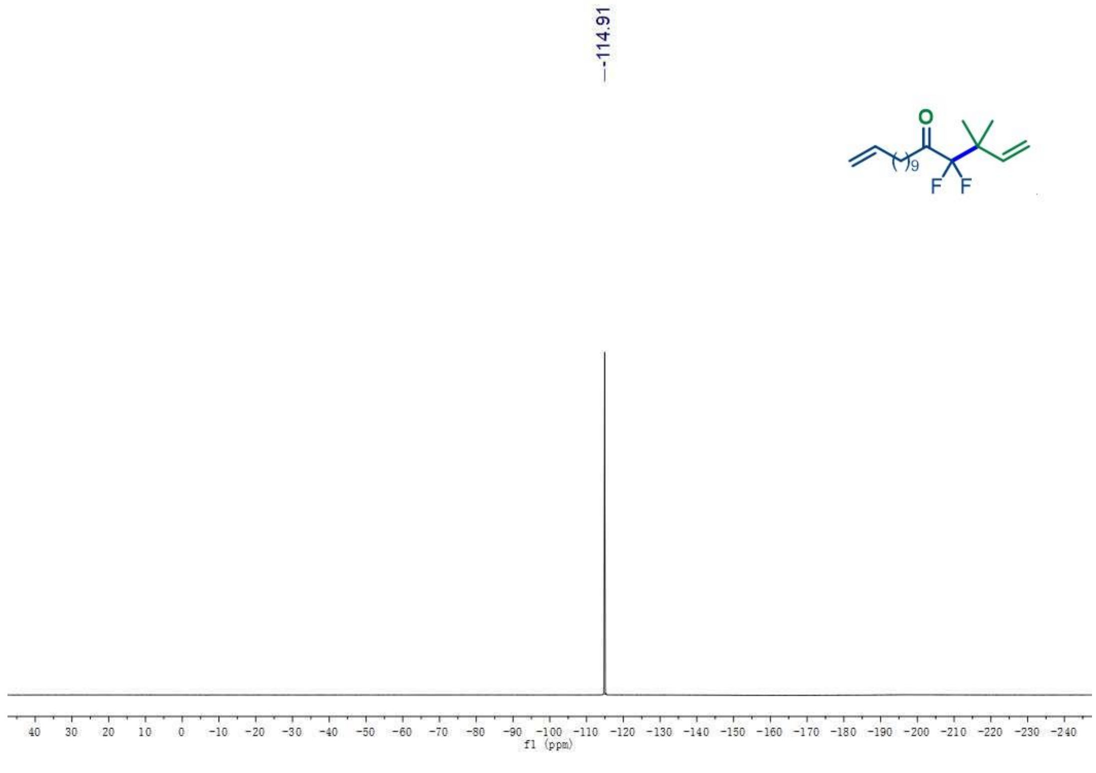

Supplementary Fig. 539 ^{19}F NMR (470 MHz, $CDCl_3$) spectrum of compound **179**

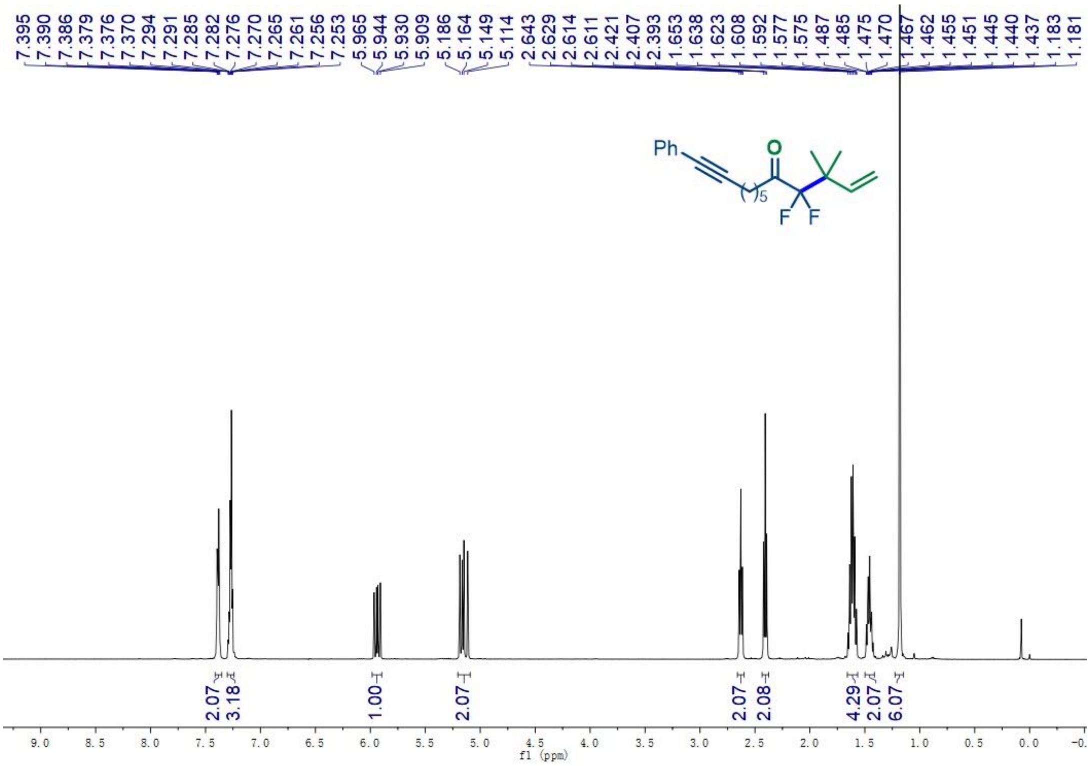

Supplementary Fig. 540 ^{1}H NMR (500 MHz, $CDCl_3$) spectrum of compound **180**

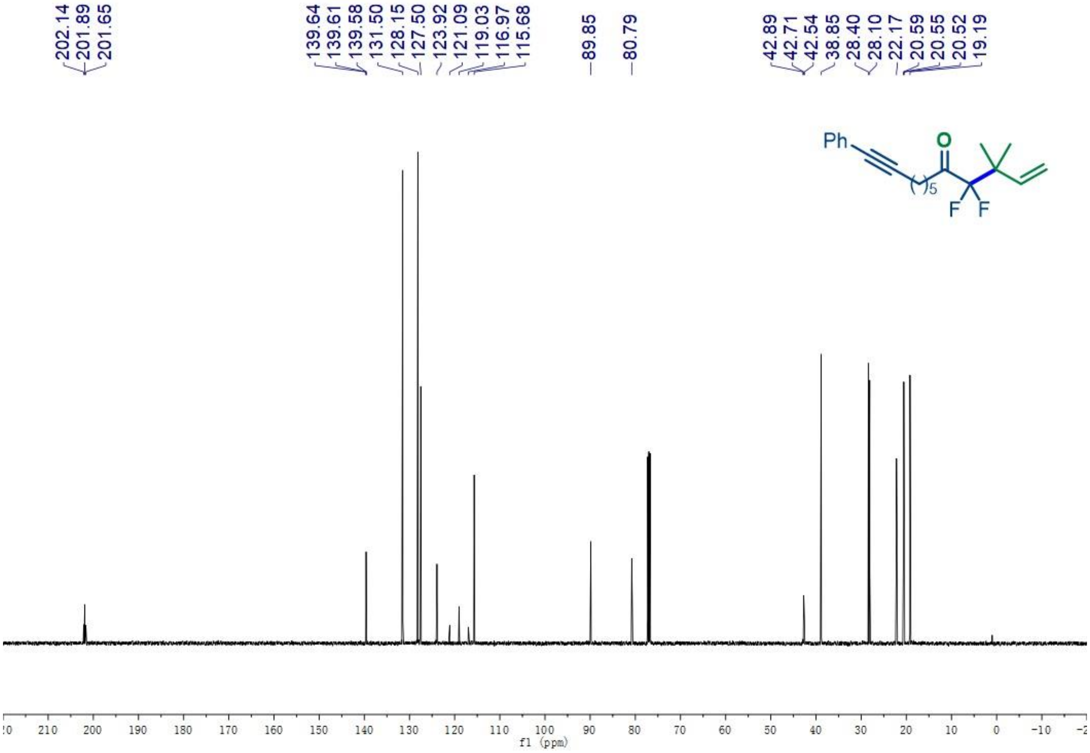

Supplementary Fig. 541 ^{13}C NMR (125 MHz, $CDCl_3$) spectrum of compound **180**

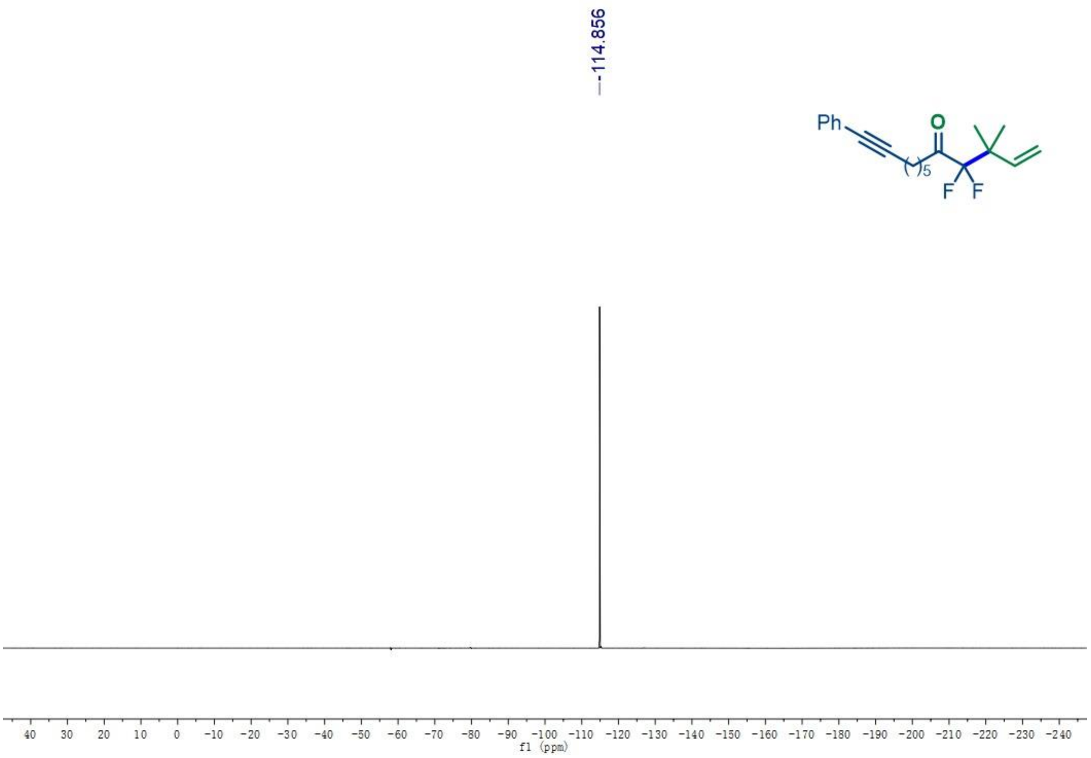

Supplementary Fig. 542 ^{19}F NMR (470 MHz, $CDCl_3$) spectrum of compound **180**

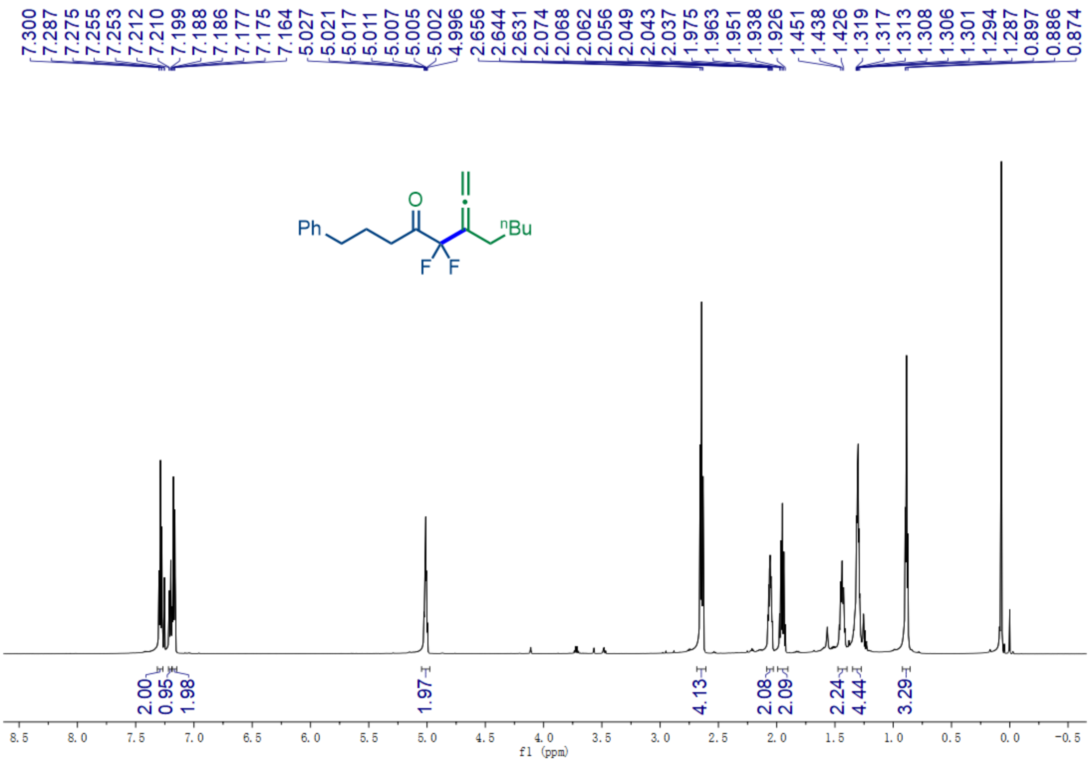

Supplementary Fig. 543 ^{1}H NMR (600 MHz, $CDCl_3$) spectrum of compound **181**

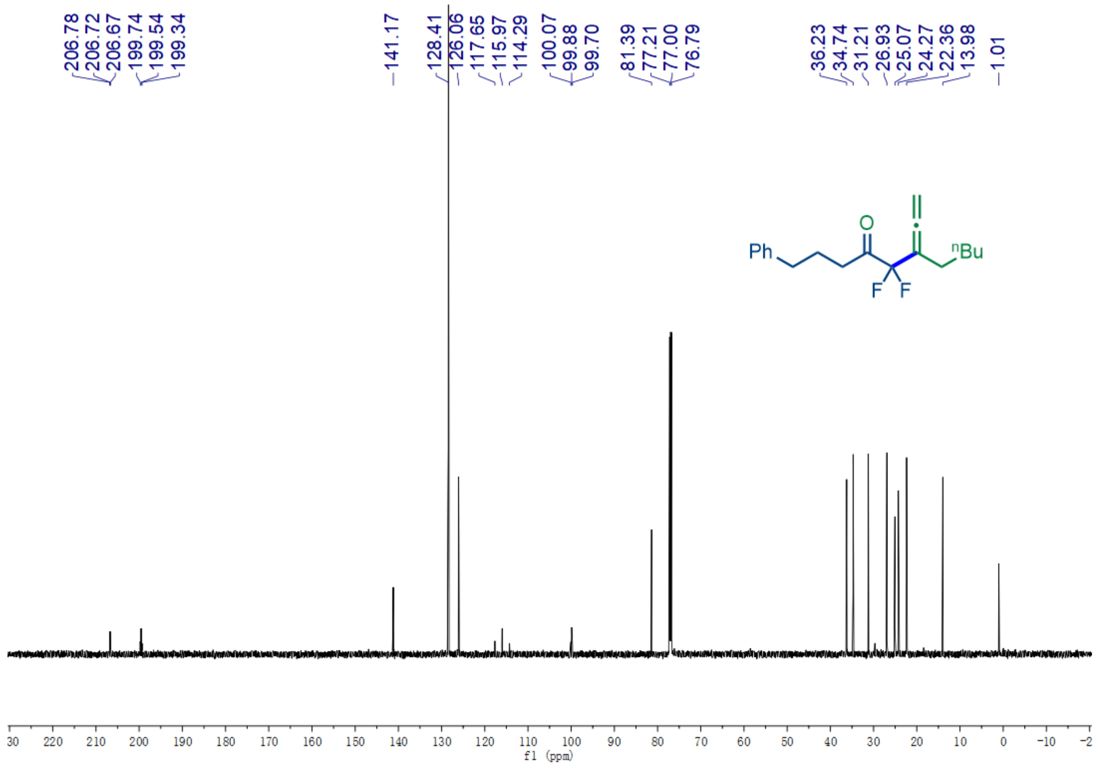

Supplementary Fig. 544 ^{13}C NMR (150 MHz, $CDCl_3$) spectrum of compound **181**

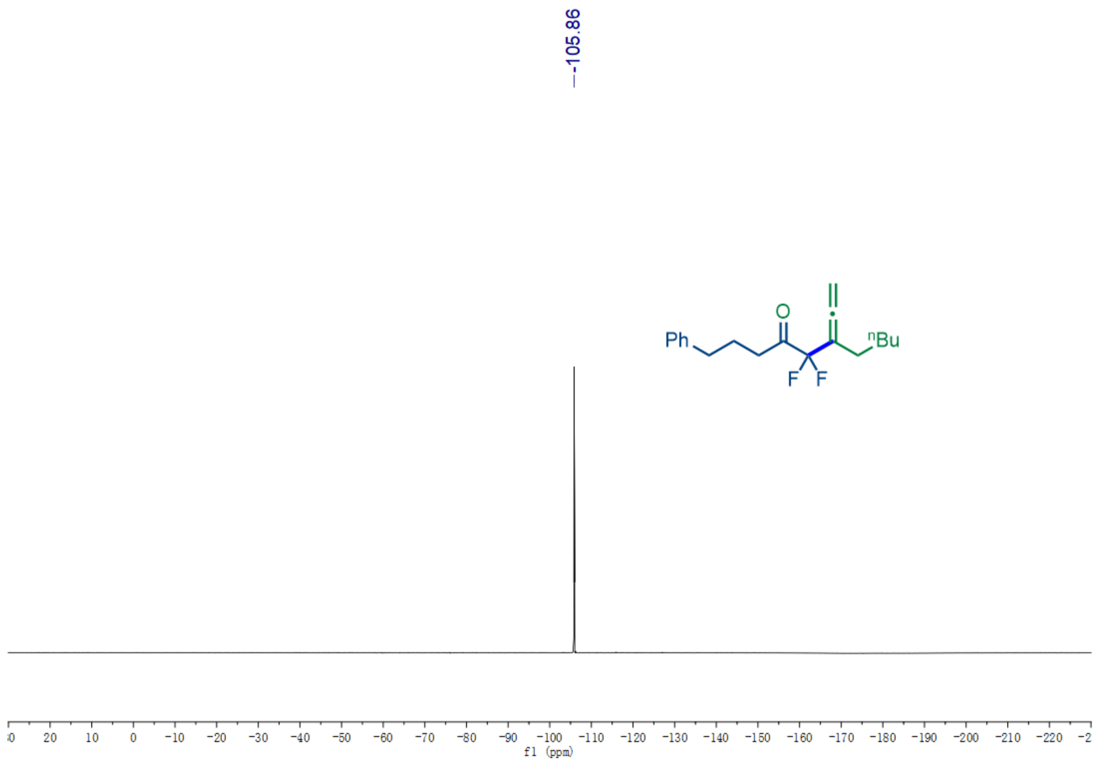

Supplementary Fig. 545 ^{19}F NMR (564 MHz, $CDCl_3$) spectrum of compound **181**

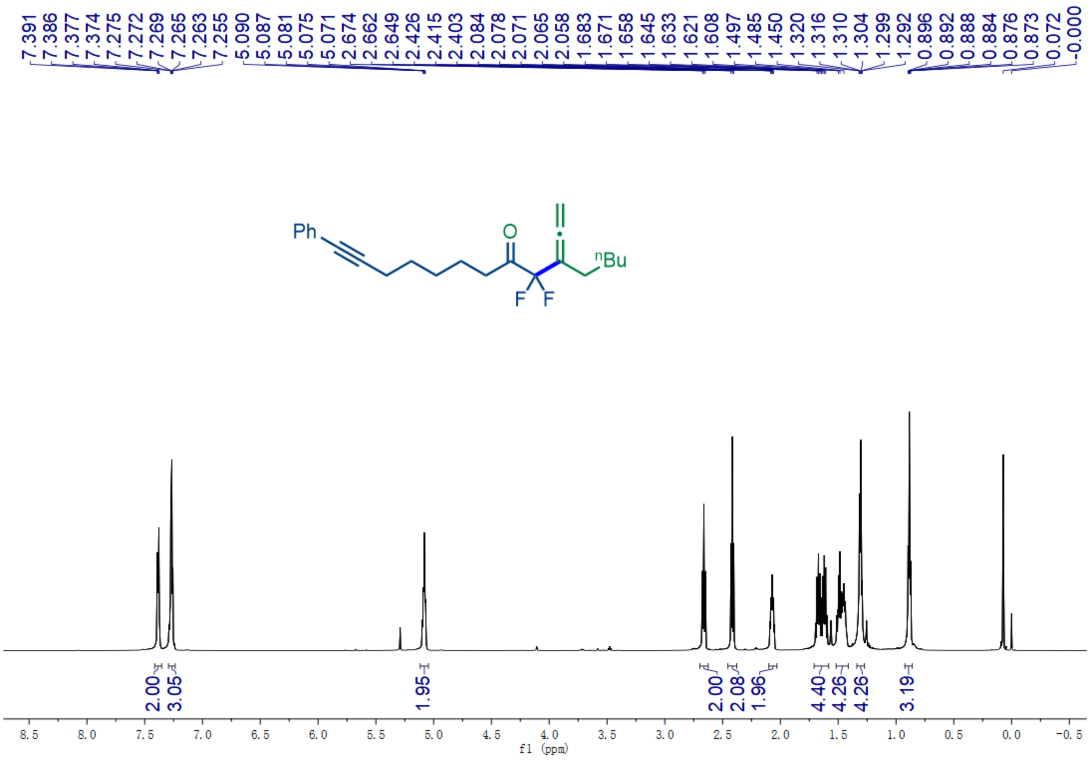

Supplementary Fig. 546 ^{1}H NMR (600 MHz, $CDCl_3$) spectrum of compound **182**

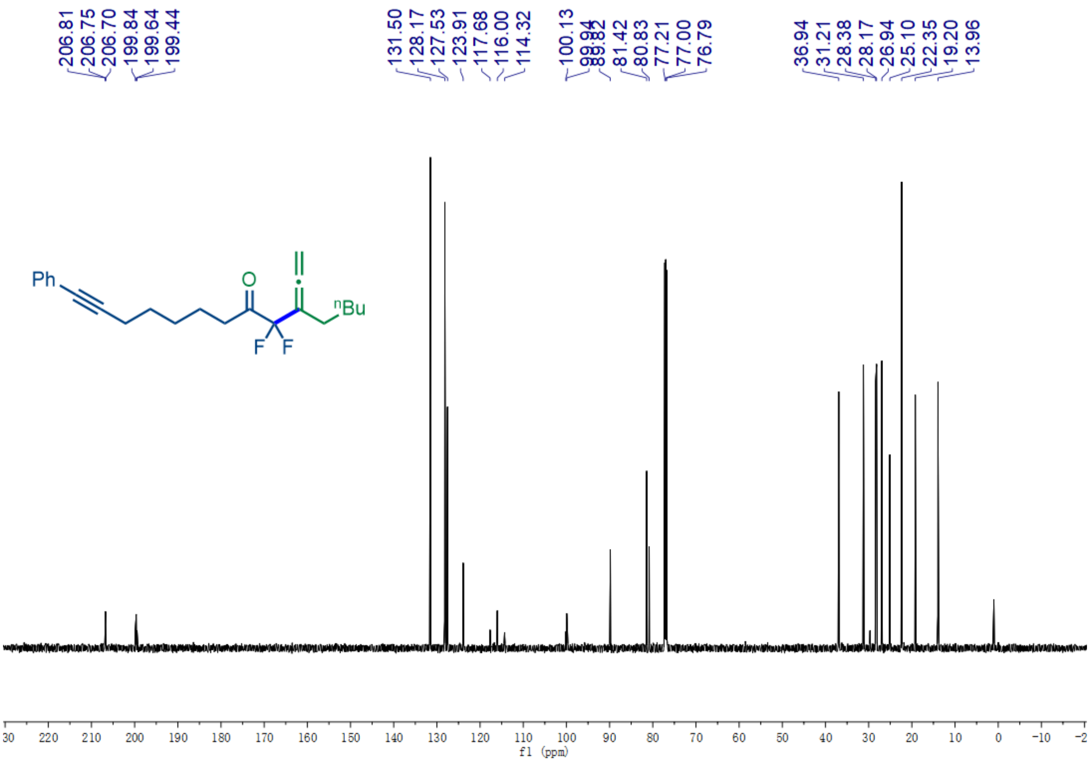

Supplementary Fig. 547 ^{13}C NMR (150 MHz, $CDCl_3$) spectrum of compound **182**

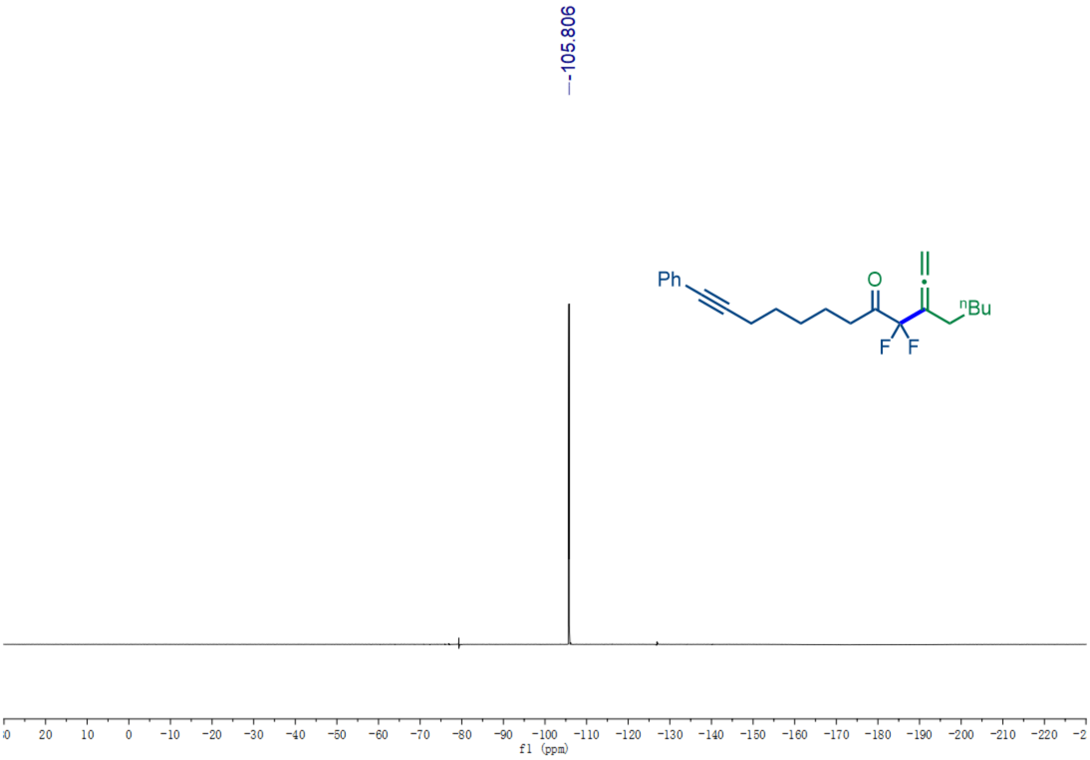

Supplementary Fig. 548 ^{19}F NMR (564 MHz, $CDCl_3$) spectrum of compound **182**

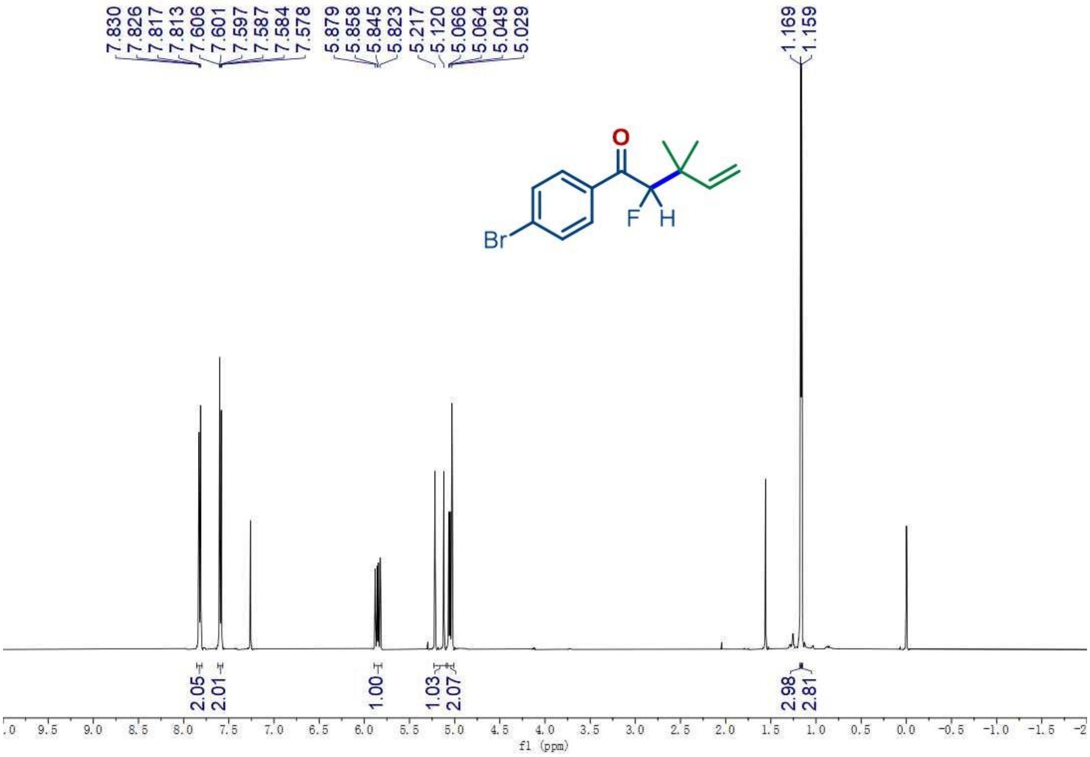

Supplementary Fig. 549 ^{1}H NMR (500 MHz, $CDCl_3$) spectrum of compound **183**

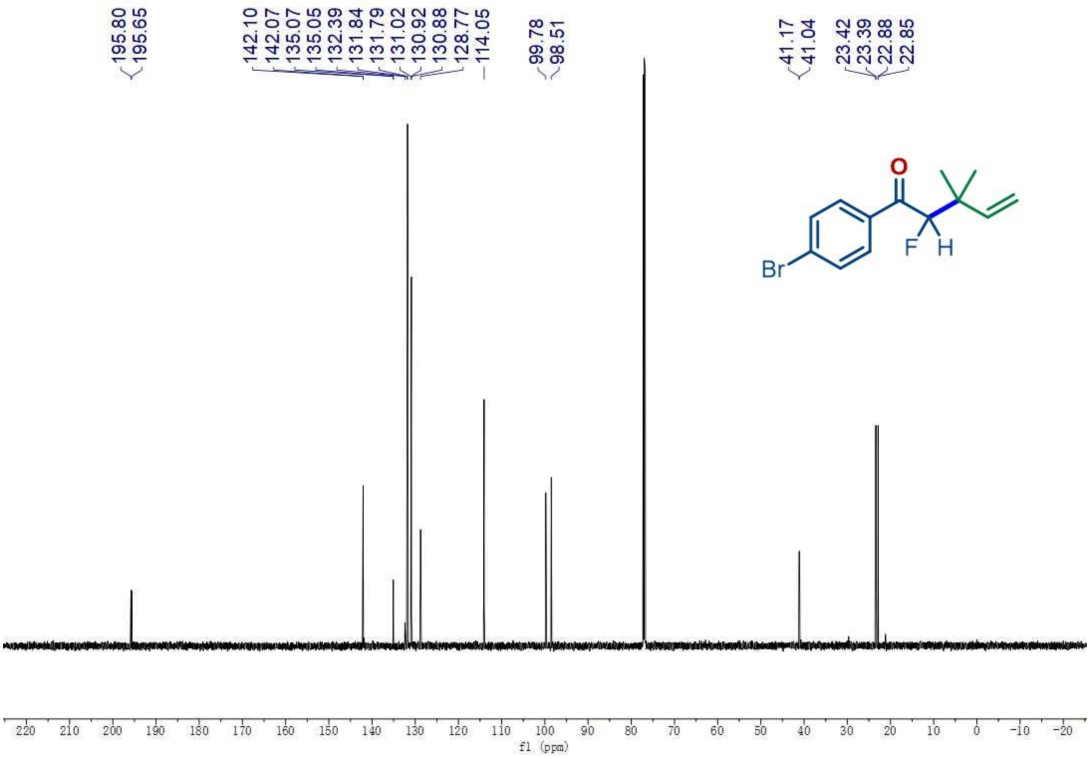

Supplementary Fig. 550 ^{13}C NMR (150 MHz, $CDCl_3$) spectrum of compound **183**

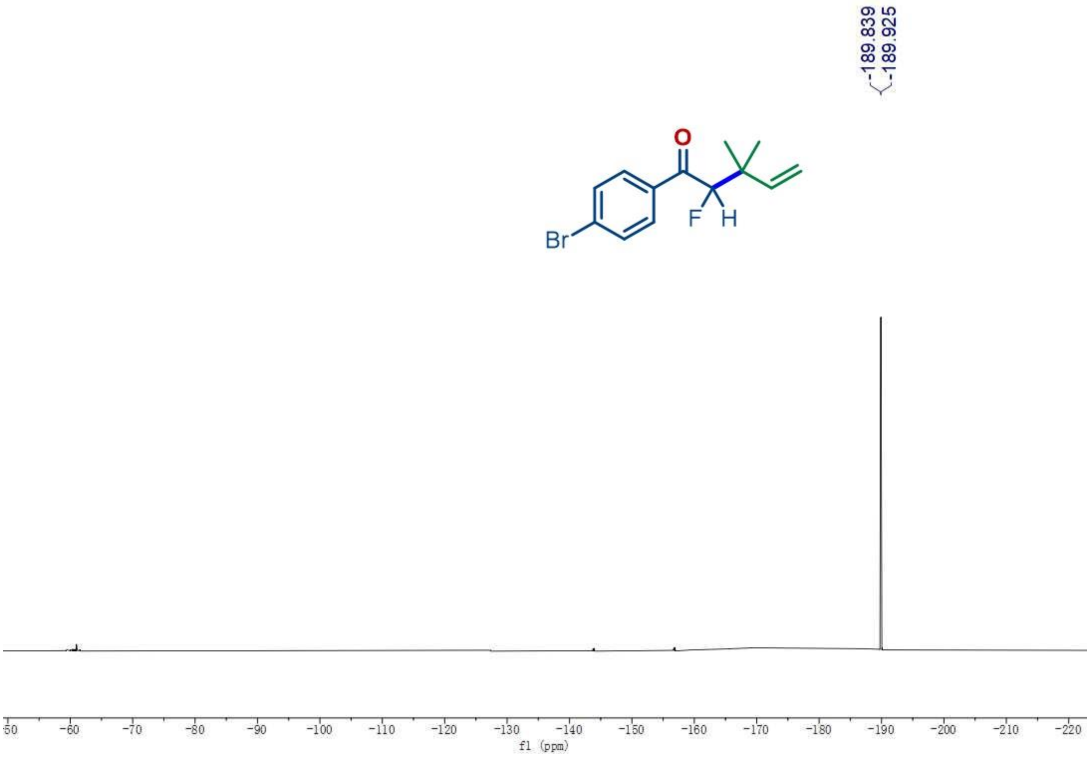

Supplementary Fig. 551 ^{19}F NMR (564 MHz, $CDCl_3$) spectrum of compound **183**

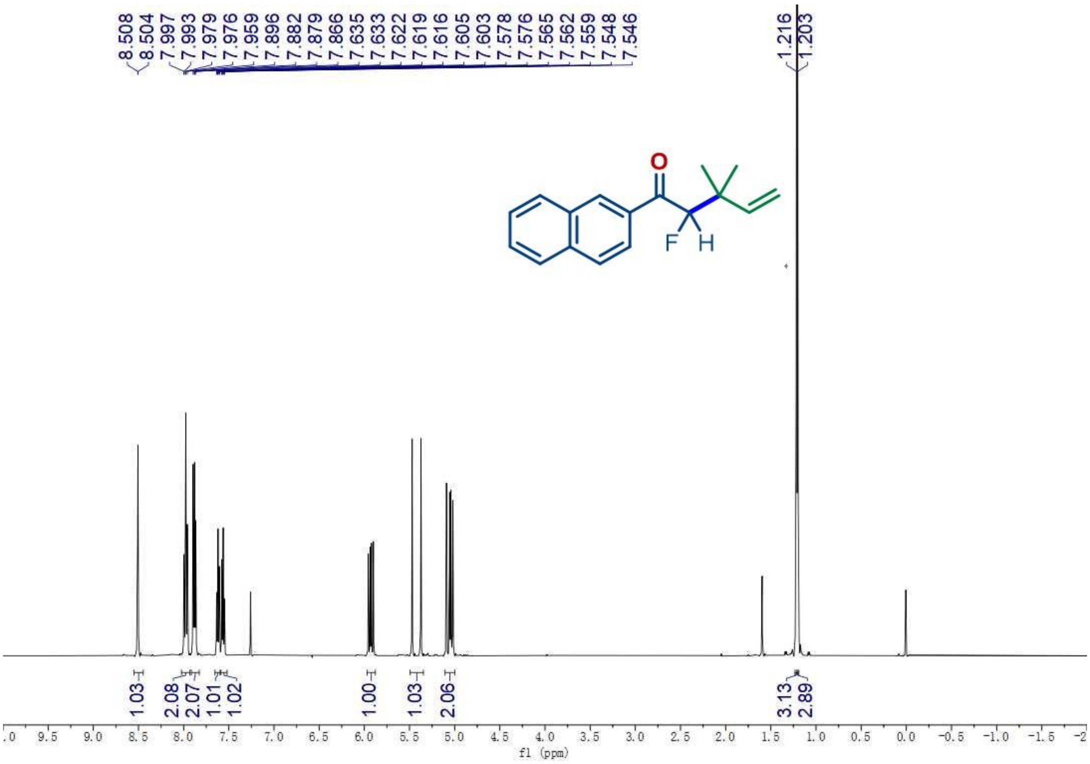

Supplementary Fig. 552 ^{1}H NMR (500 MHz, $CDCl_3$) spectrum of compound **184**

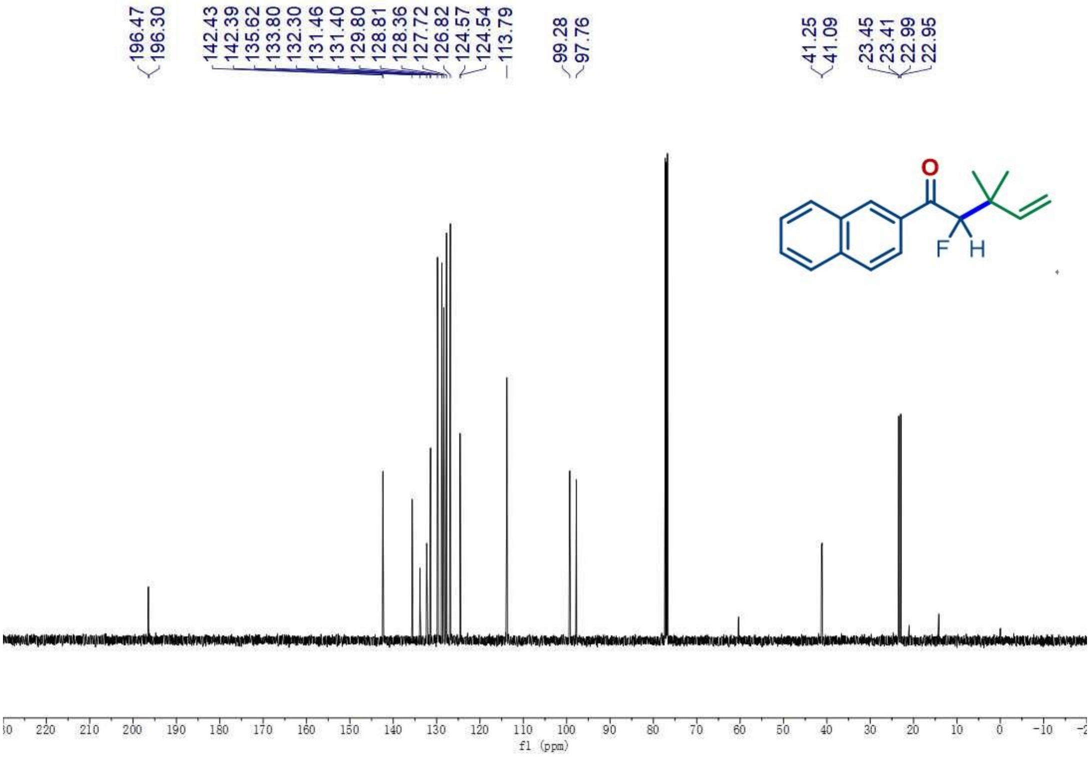

Supplementary Fig. 553 ^{13}C NMR (125 MHz, $CDCl_3$) spectrum of compound **184**

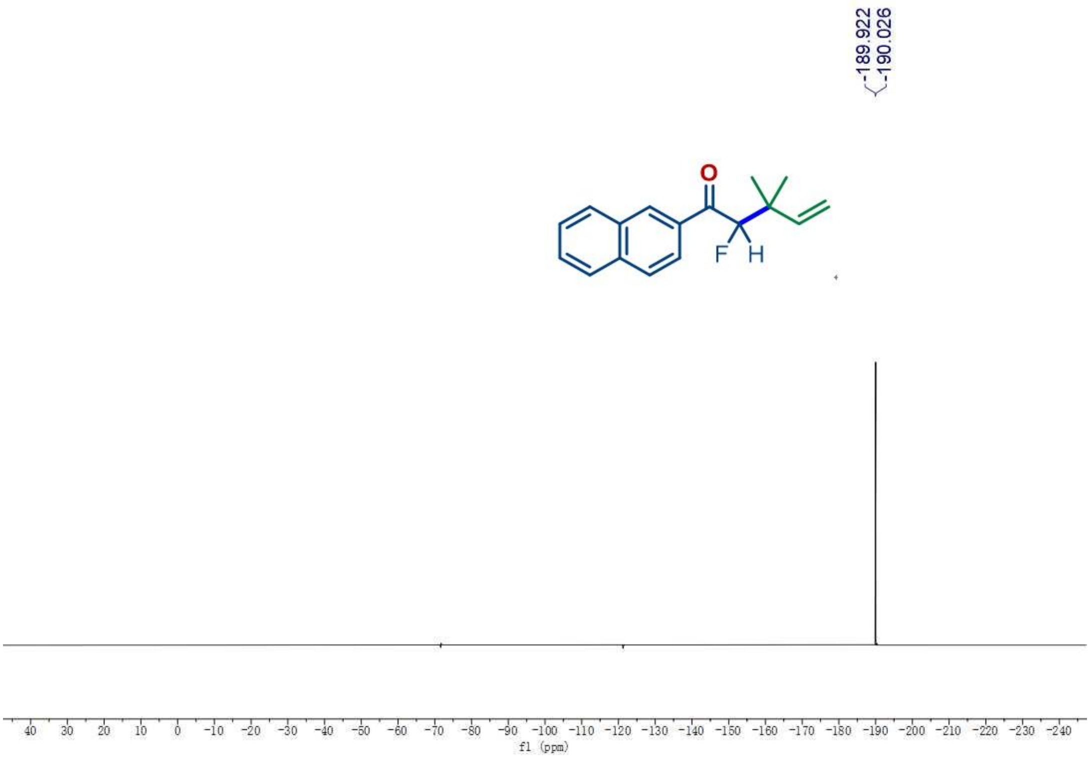

Supplementary Fig. 554 ^{19}F NMR (470 MHz, $CDCl_3$) spectrum of compound **184**

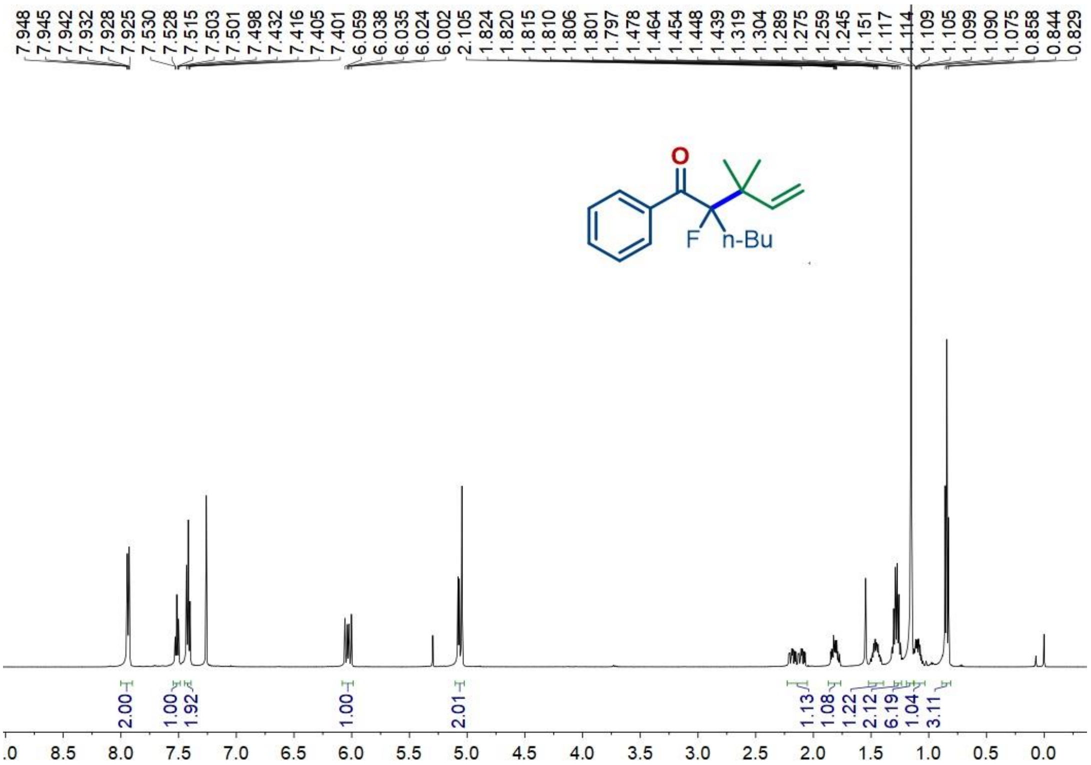

Supplementary Fig. 555 ^{1}H NMR (500 MHz, $CDCl_3$) spectrum of compound **185**

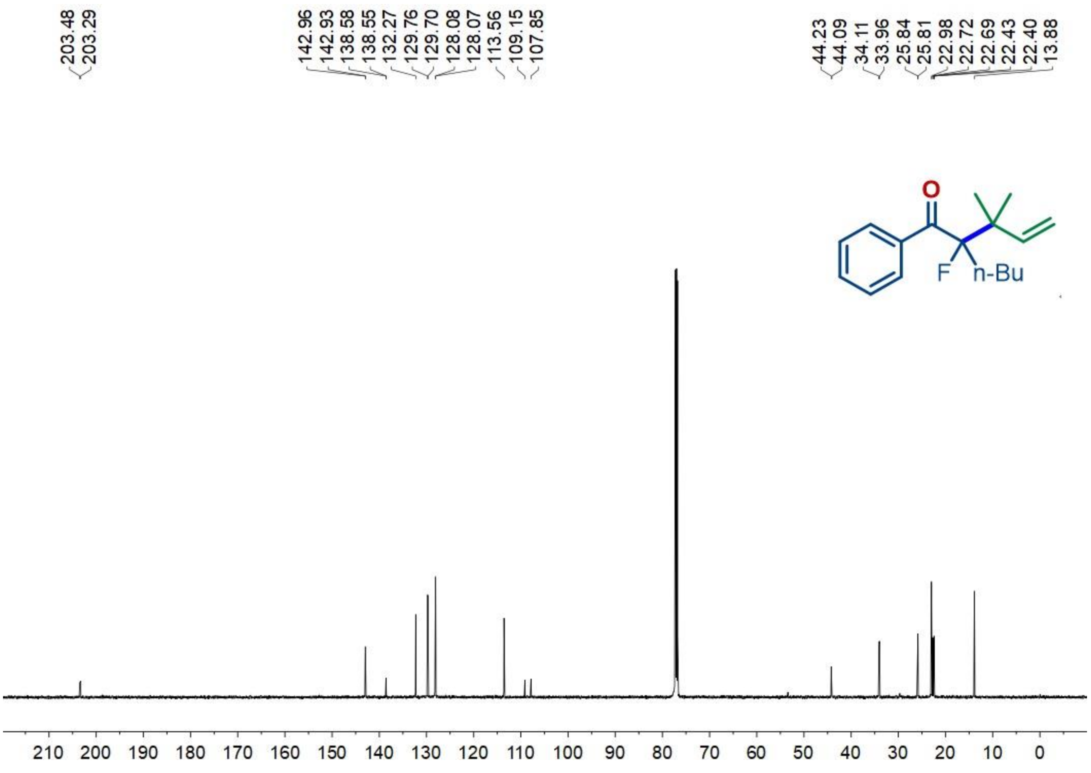

Supplementary Fig. 556 ^{13}C NMR (150 MHz, $CDCl_3$) spectrum of compound **185**

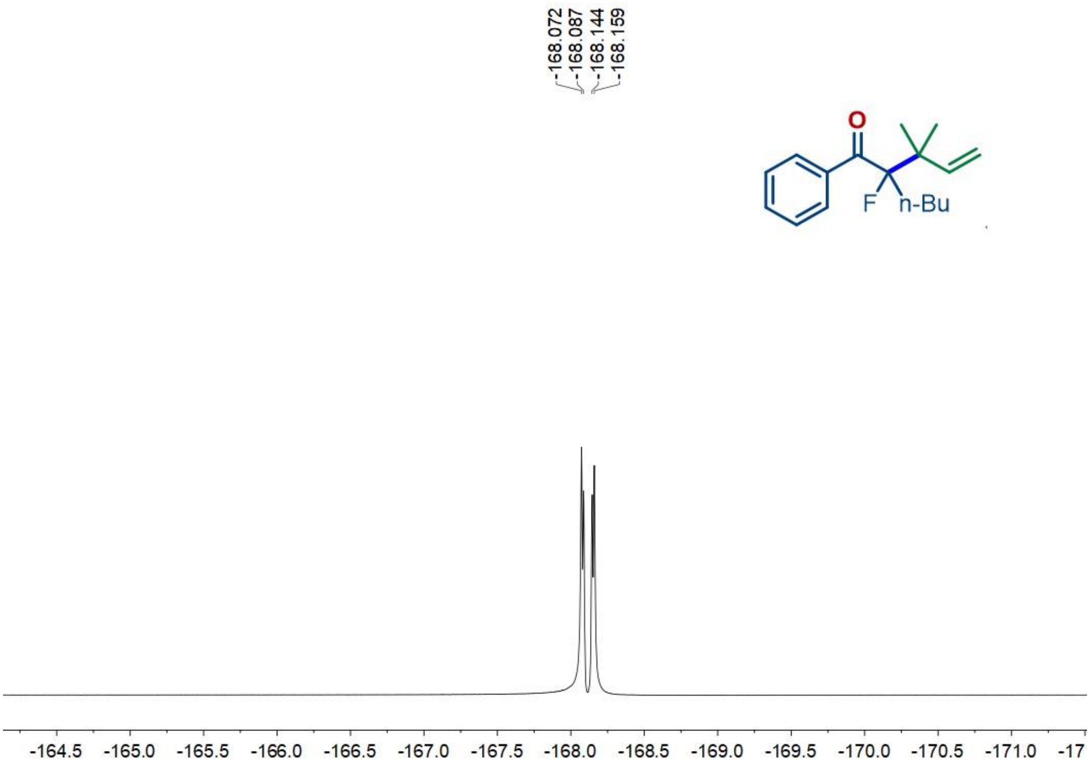

Supplementary Fig. 557 ^{19}F NMR (564 MHz, $CDCl_3$) spectrum of compound **185**

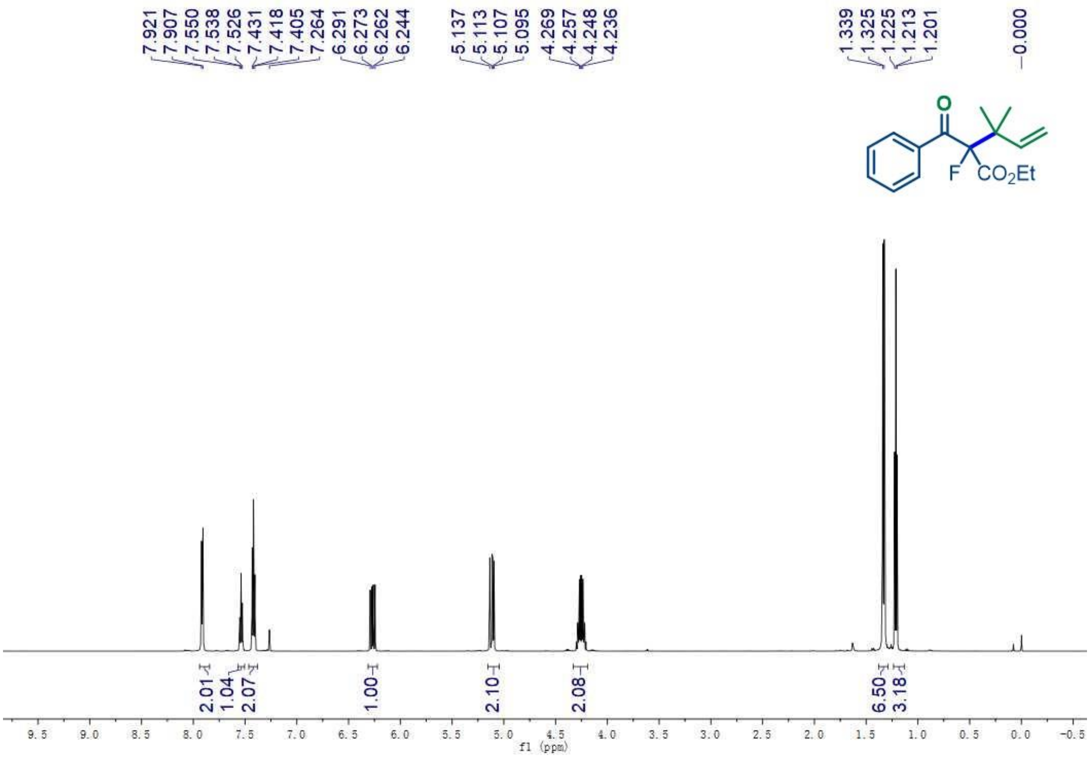

Supplementary Fig. 558^{1}H NMR (600 MHz, $CDCl_3$) spectrum of compound **186**

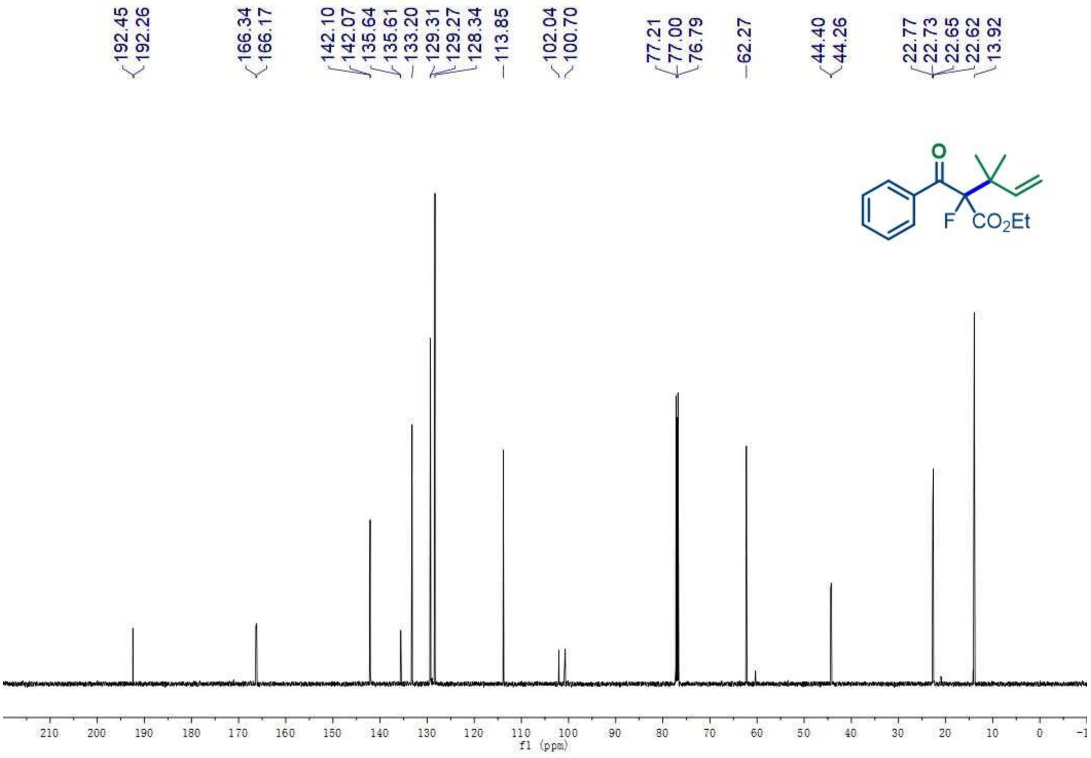

Supplementary Fig. 559 ^{13}C NMR (150 MHz, $CDCl_3$) spectrum of compound **186**

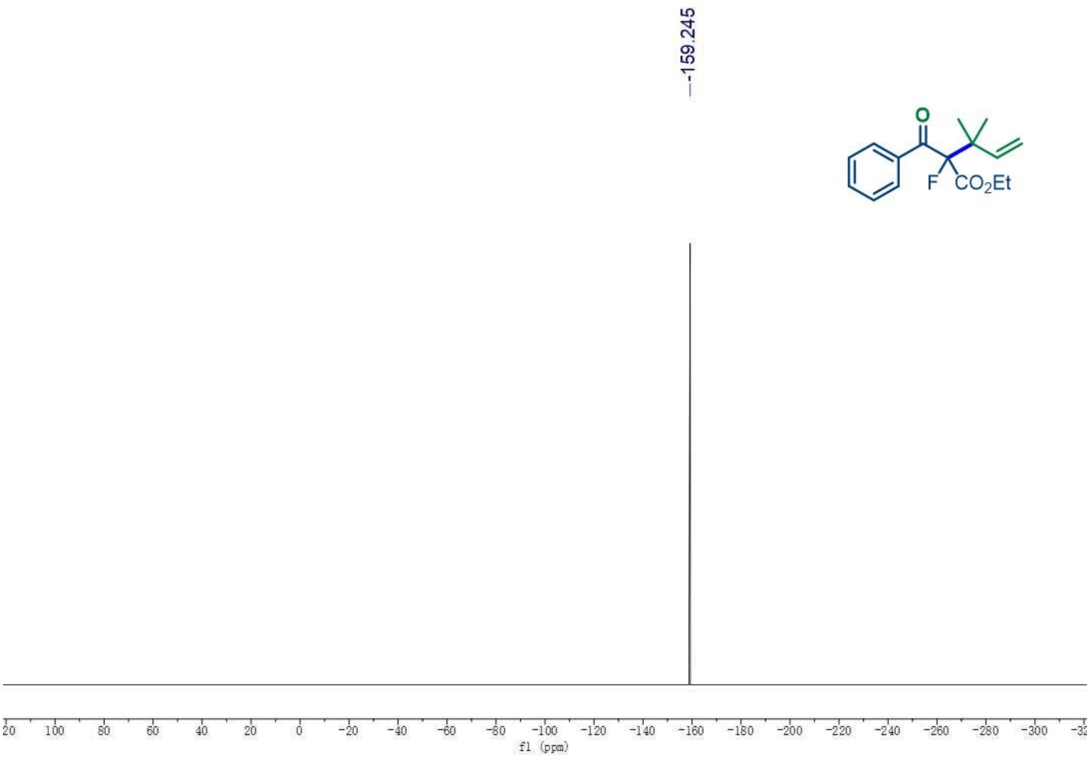

Supplementary Fig. 560 ^{19}F NMR (564 MHz, $CDCl_3$) spectrum of compound **186**

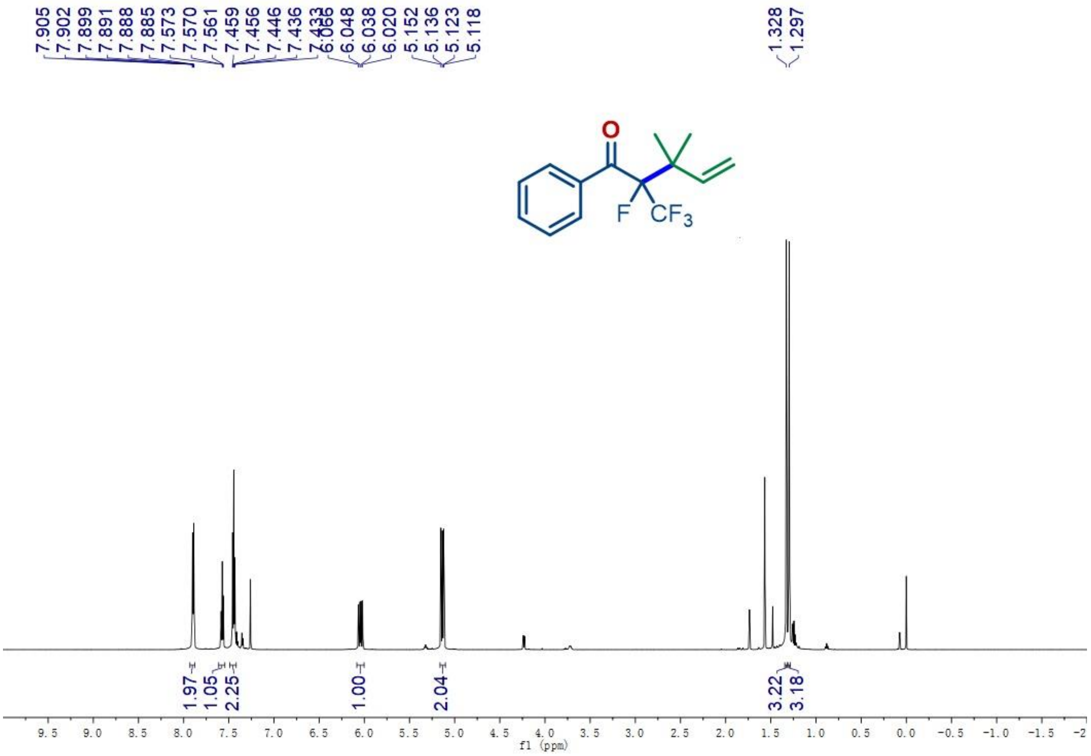

Supplementary Fig. 561 ^{1}H NMR (600 MHz, $CDCl_3$) spectrum of compound **187**

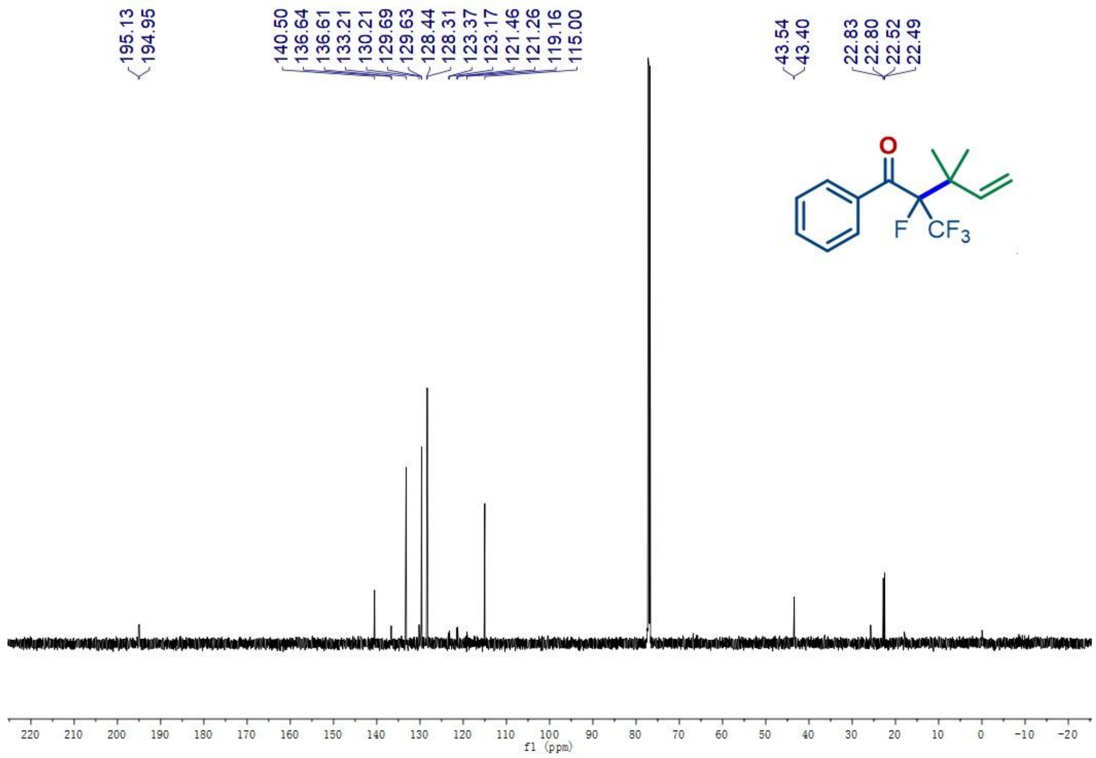

Supplementary Fig. 562 ^{13}C NMR (150 MHz, $CDCl_3$) spectrum of compound **187**

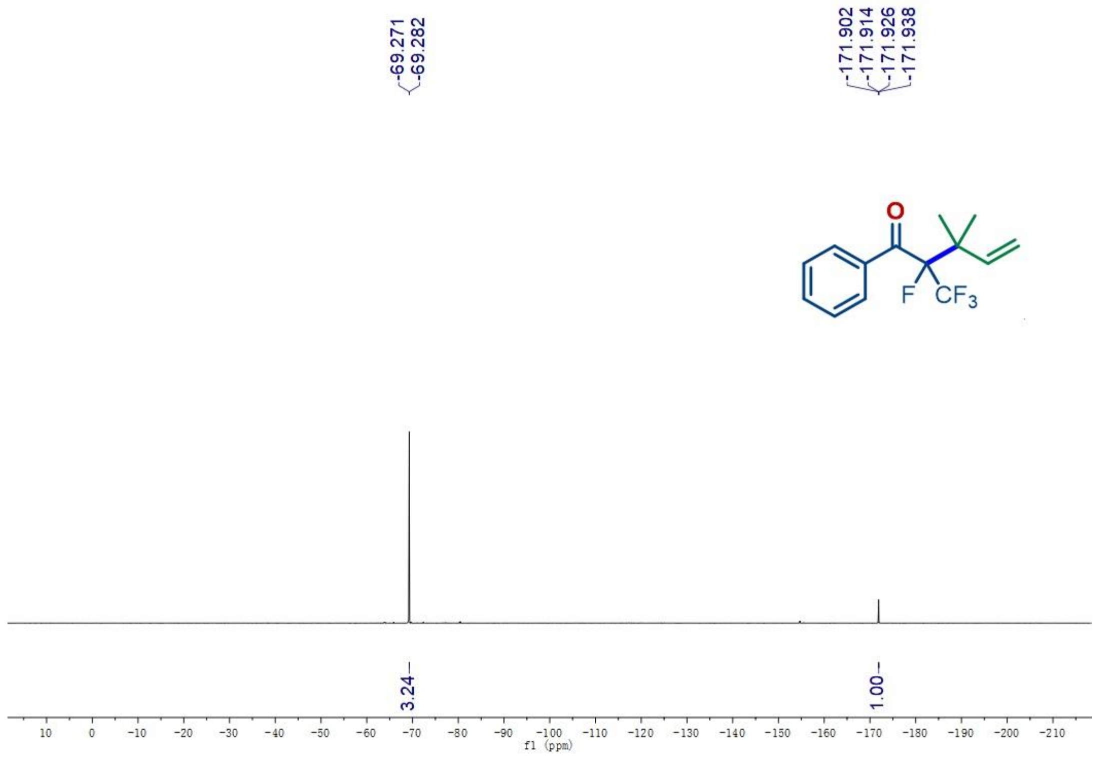

Supplementary Fig. 563 ^{19}F NMR (564 MHz, $CDCl_3$) spectrum of compound **187**

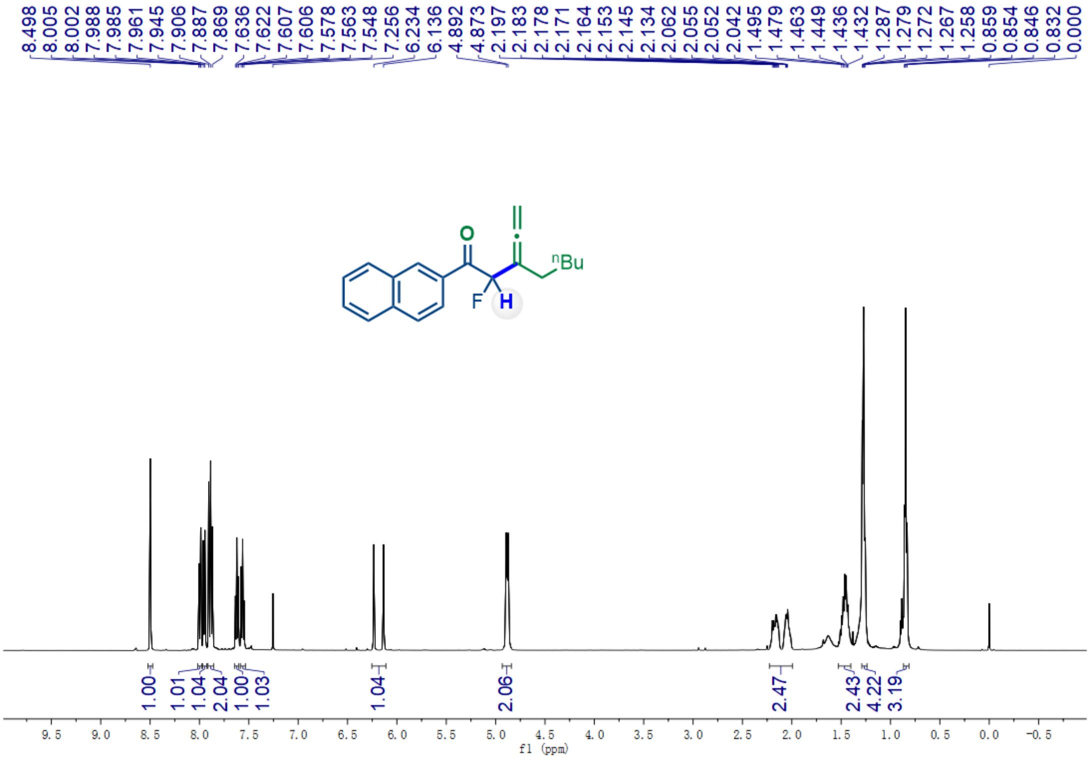

Supplementary Fig. 564 ^{1}H NMR (500 MHz, $CDCl_3$) spectrum of compound **188**

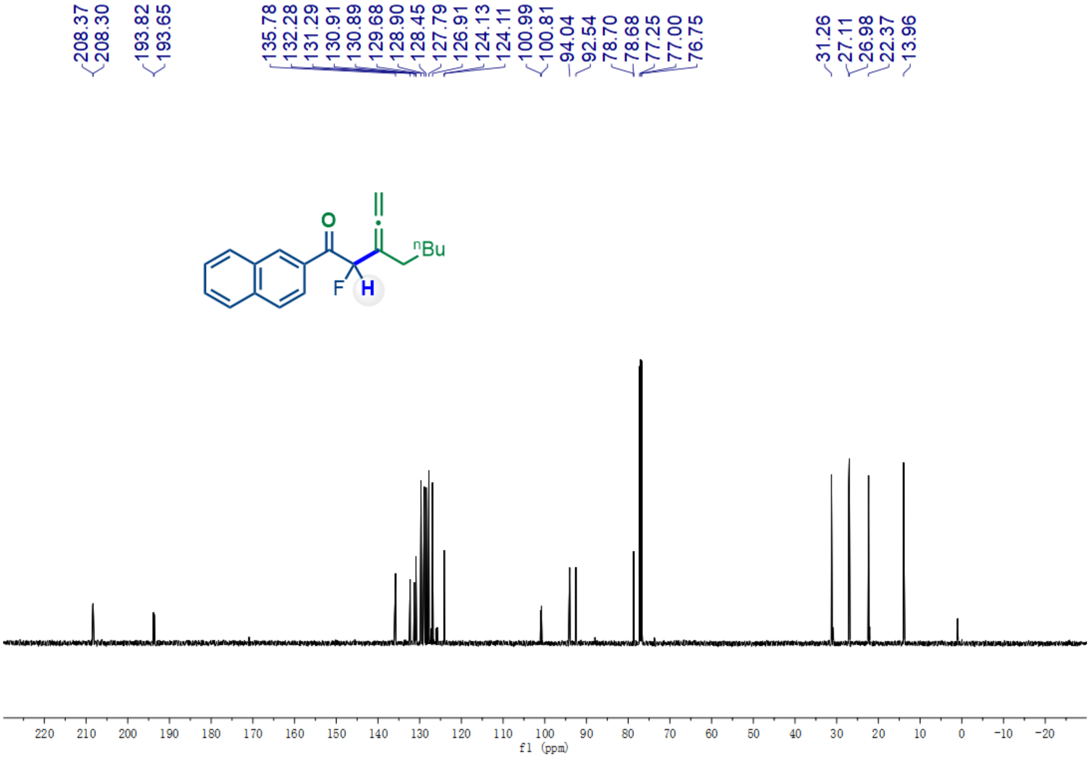

Supplementary Fig. 565 ^{13}C NMR (125 MHz, $CDCl_3$) spectrum of compound **188**

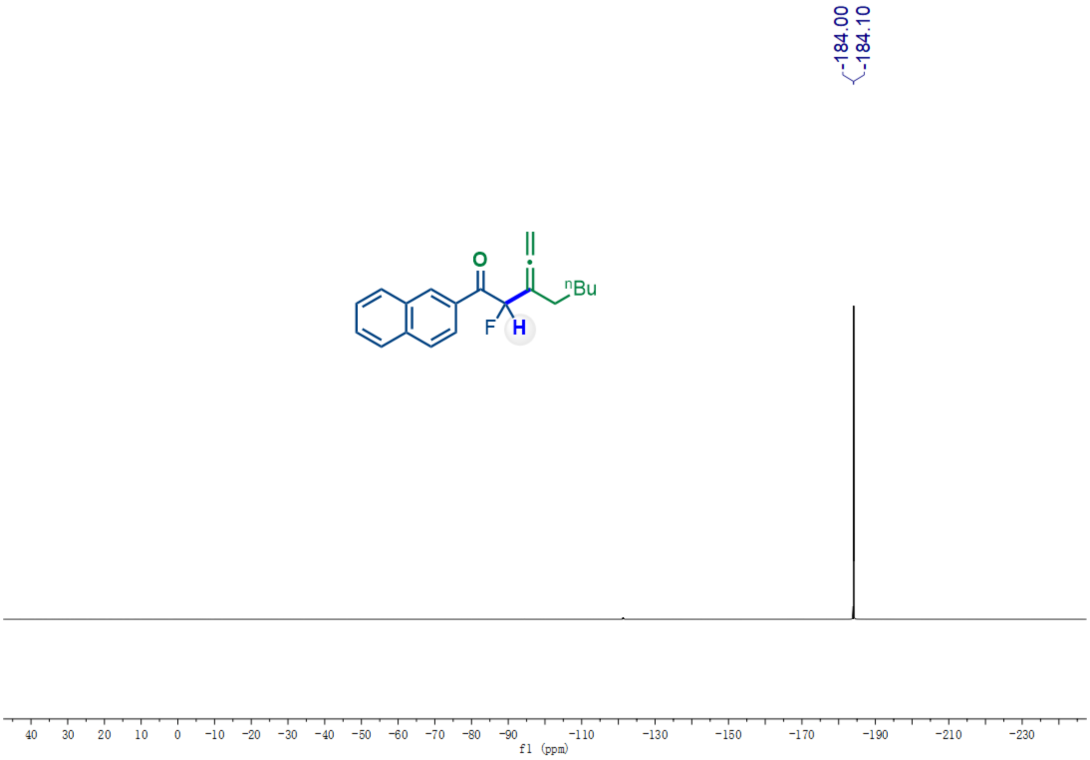

Supplementary Fig. 566 ^{19}F NMR (470 MHz, $CDCl_3$) spectrum of compound **188**

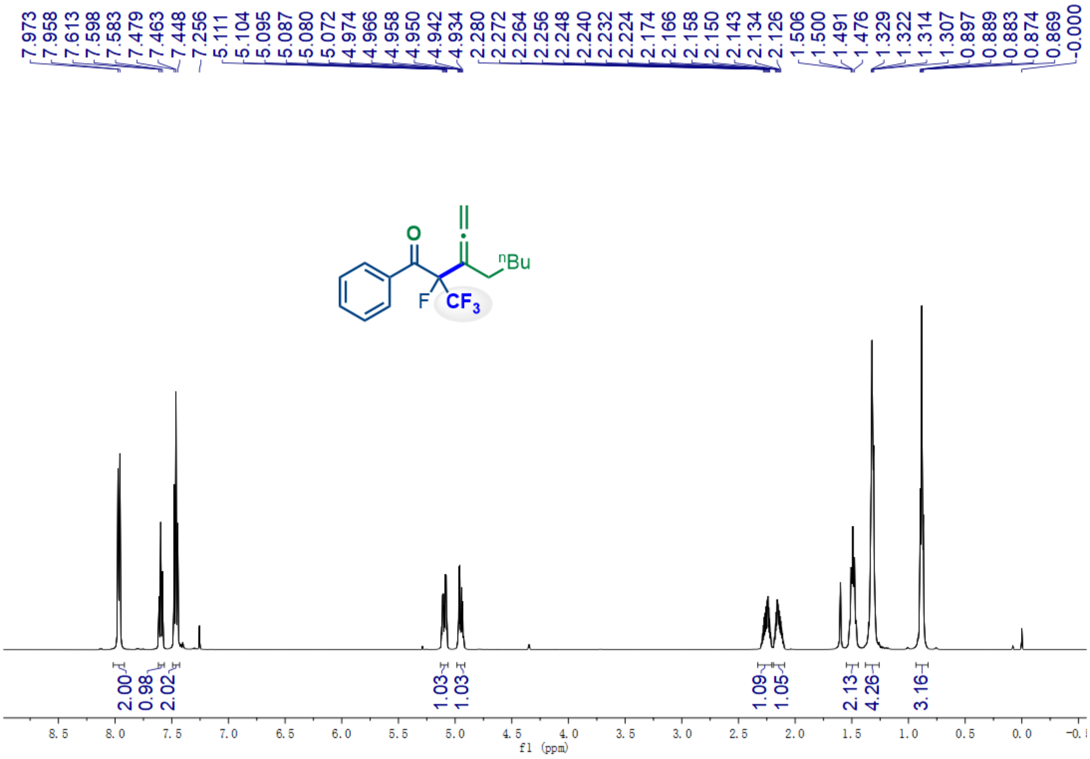

Supplementary Fig. 567 ^{1}H NMR (500 MHz, $CDCl_3$) spectrum of compound **189**

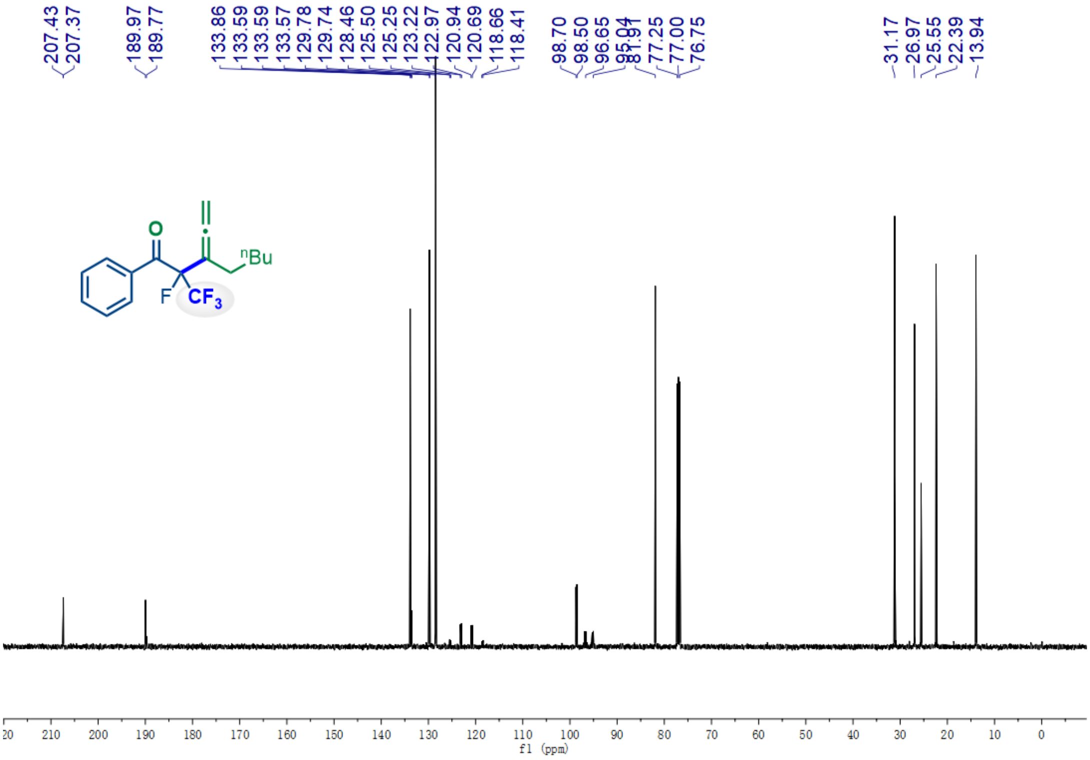

Supplementary Fig. 568 ^{13}C NMR (125 MHz, $CDCl_3$) spectrum of compound **189**

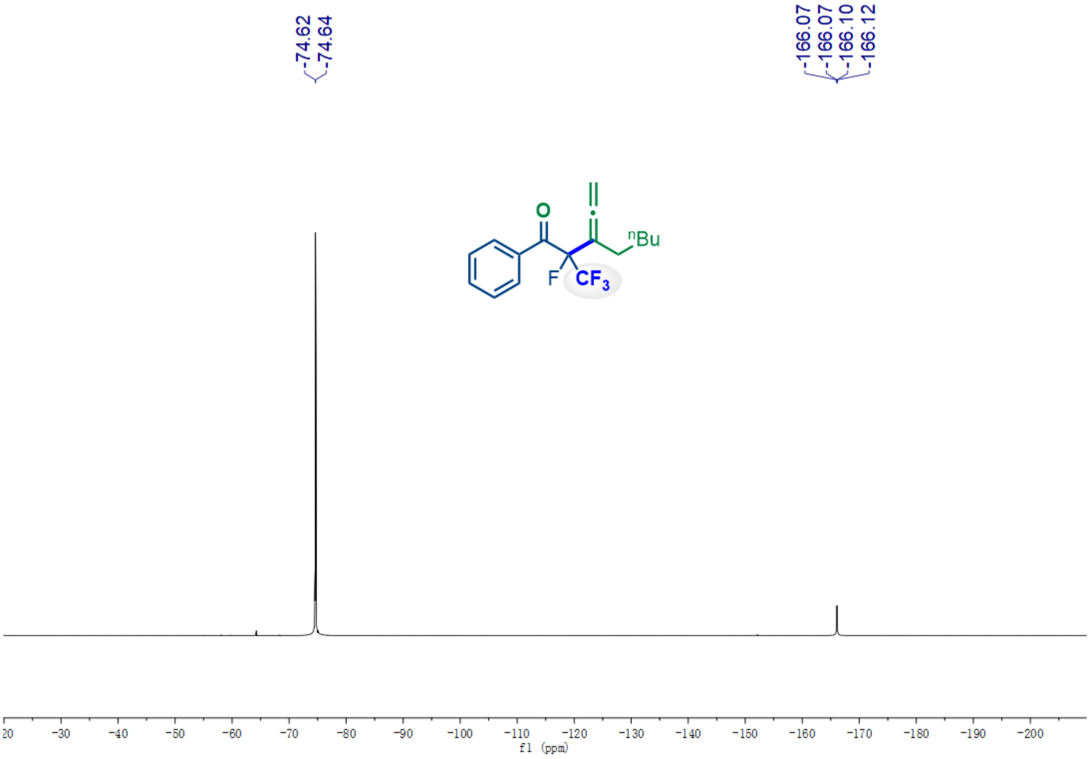

Supplementary Fig. 569 ^{19}F NMR (470 MHz, $CDCl_3$) spectrum of compound **189**

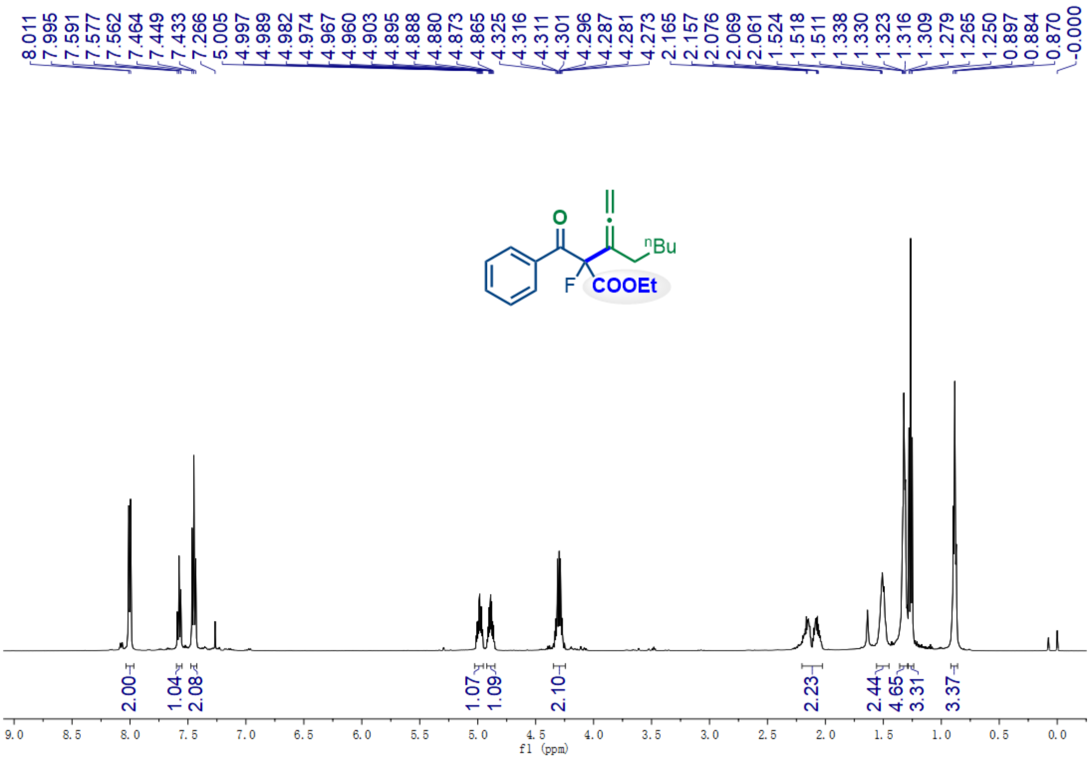

Supplementary Fig. 570 ^{1}H NMR (500 MHz, $CDCl_3$) spectrum of compound **190**

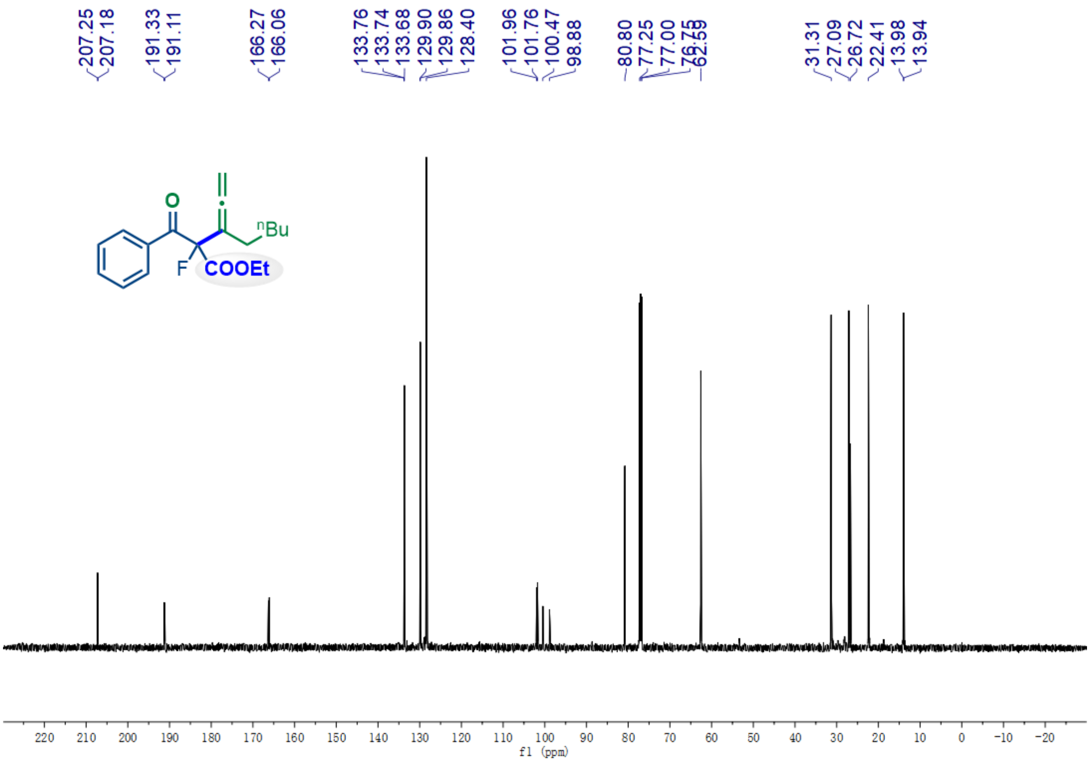

Supplementary Fig. 571 ^{13}C NMR (125 MHz, $CDCl_3$) spectrum of compound **190**

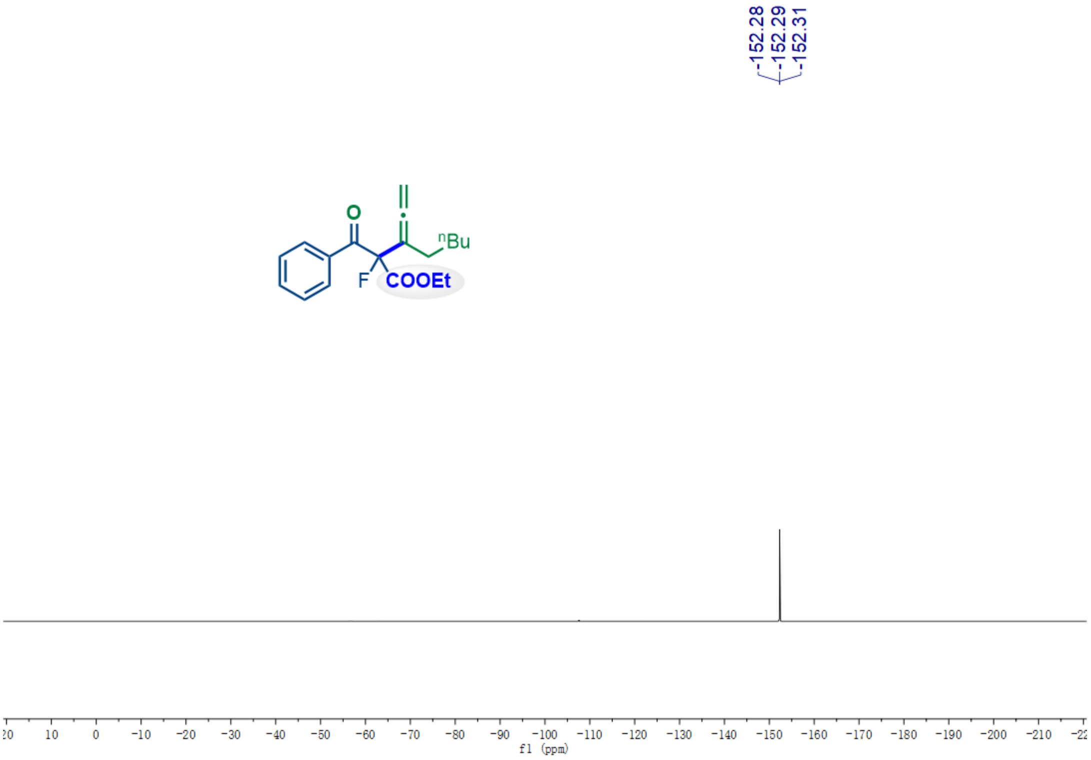

Supplementary Fig. 572 ^{19}F NMR (470 MHz, $CDCl_3$) spectrum of compound **190**

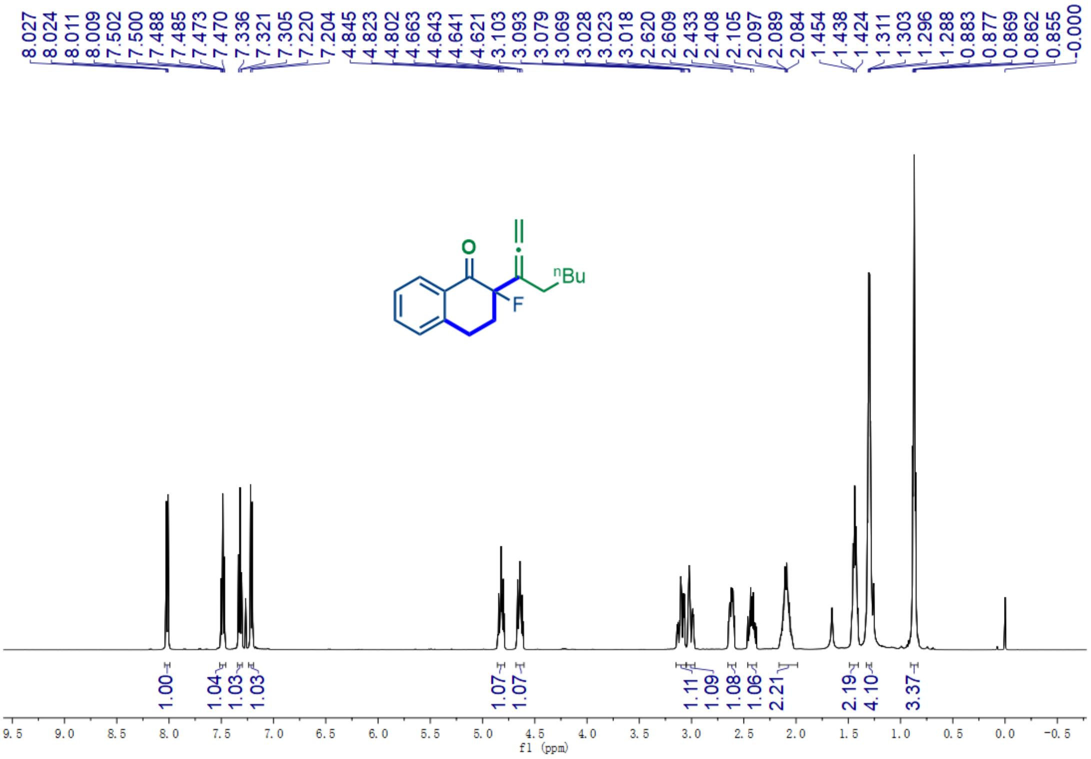

Supplementary Fig. 573 ^{1}H NMR (500 MHz, $CDCl_3$) spectrum of compound **191**

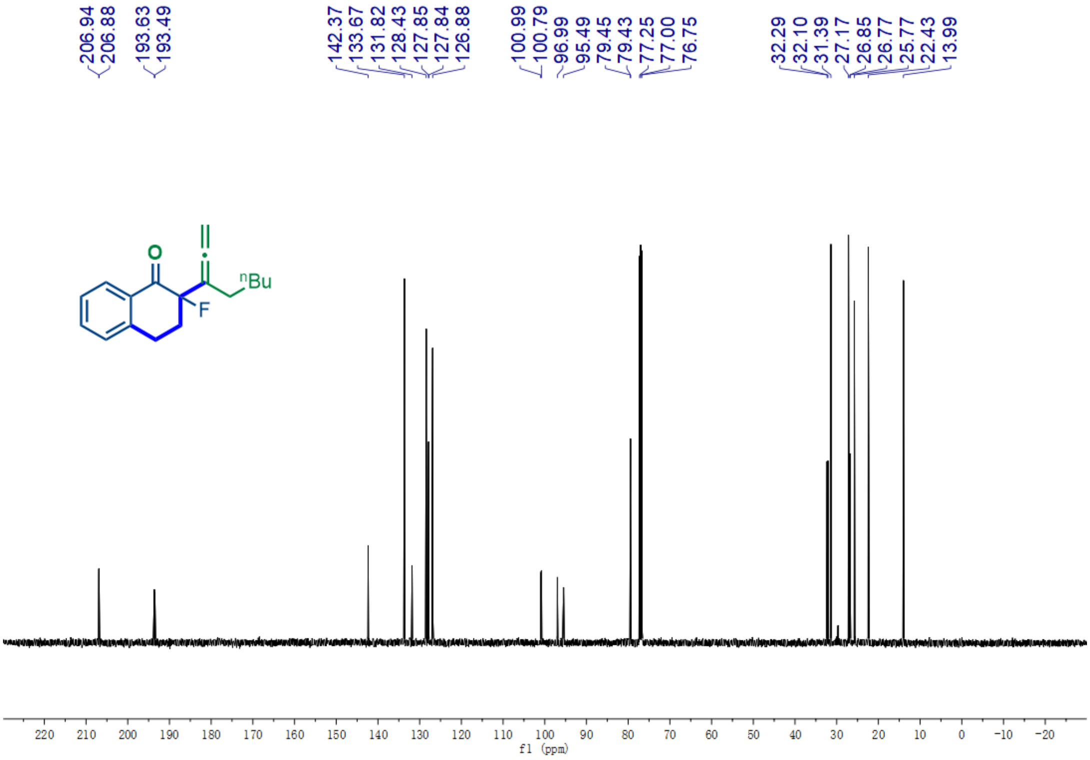

Supplementary Fig. 574 ^{13}C NMR (125 MHz, $CDCl_3$) spectrum of compound **191**

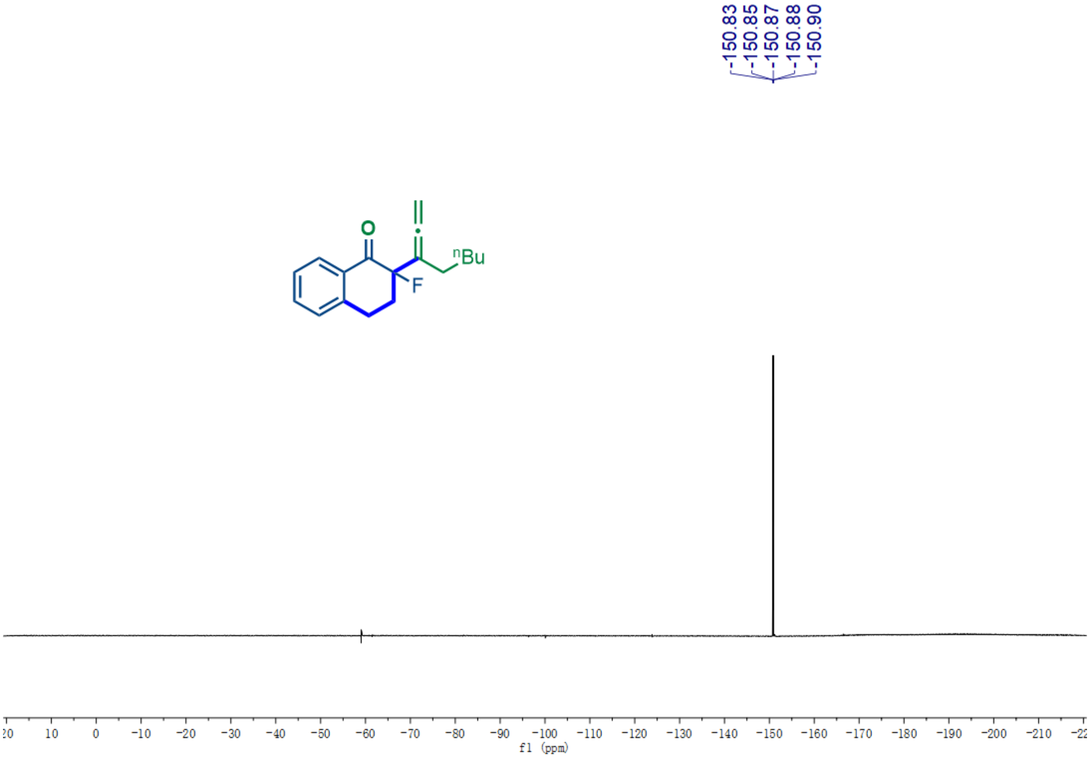

Supplementary Fig. 575 ^{19}F NMR (470 MHz, $CDCl_3$) spectrum of compound **191**

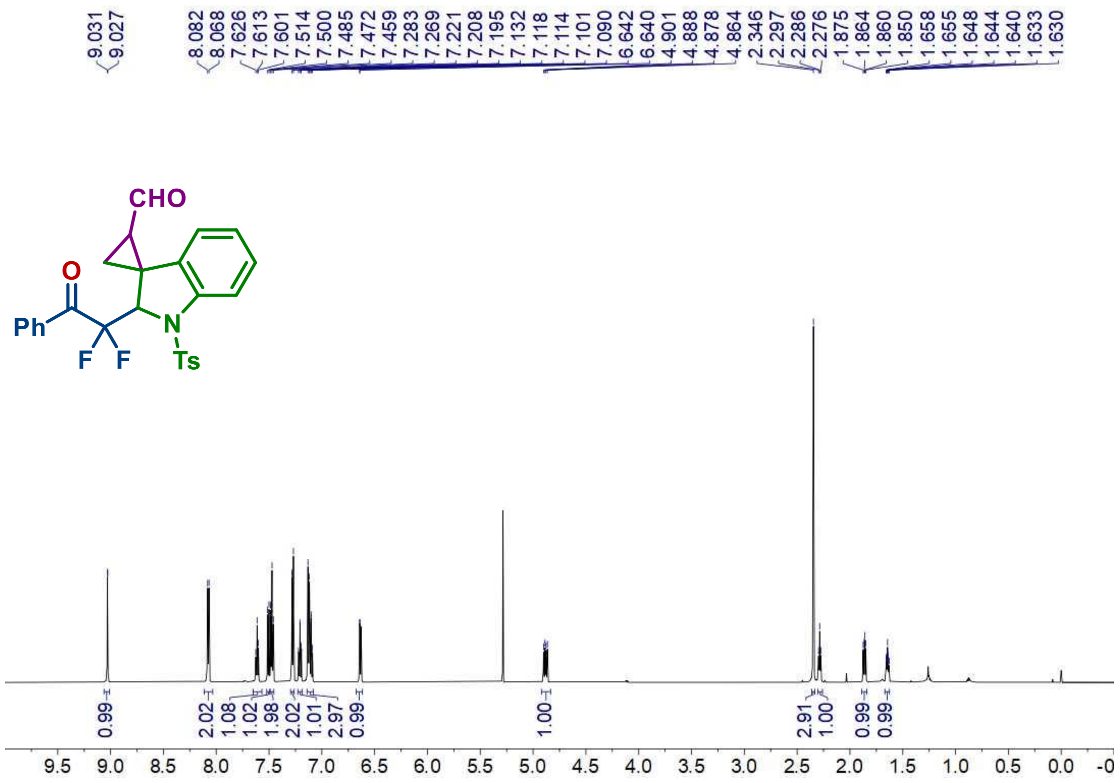

Supplementary Fig. 576 ^{1}H NMR (600 MHz, $CDCl_3$) spectrum of compound **192**

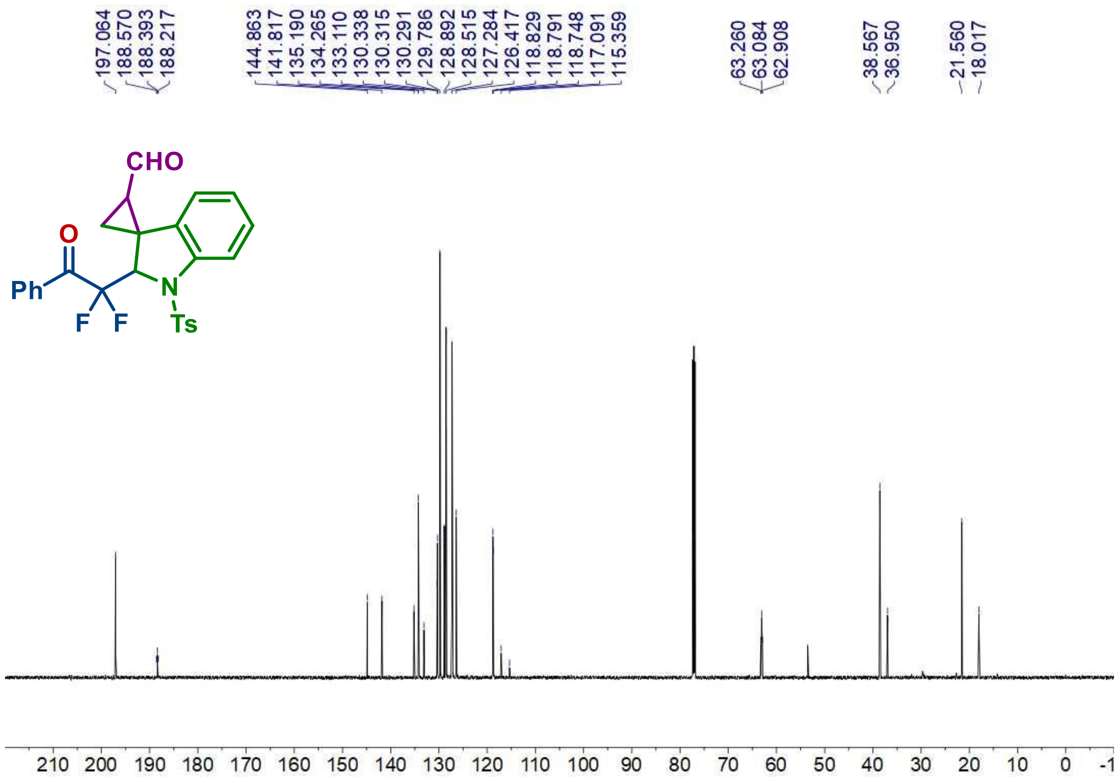

Supplementary Fig. 577 ^{13}C NMR (150 MHz, $CDCl_3$) spectrum of compound **192**

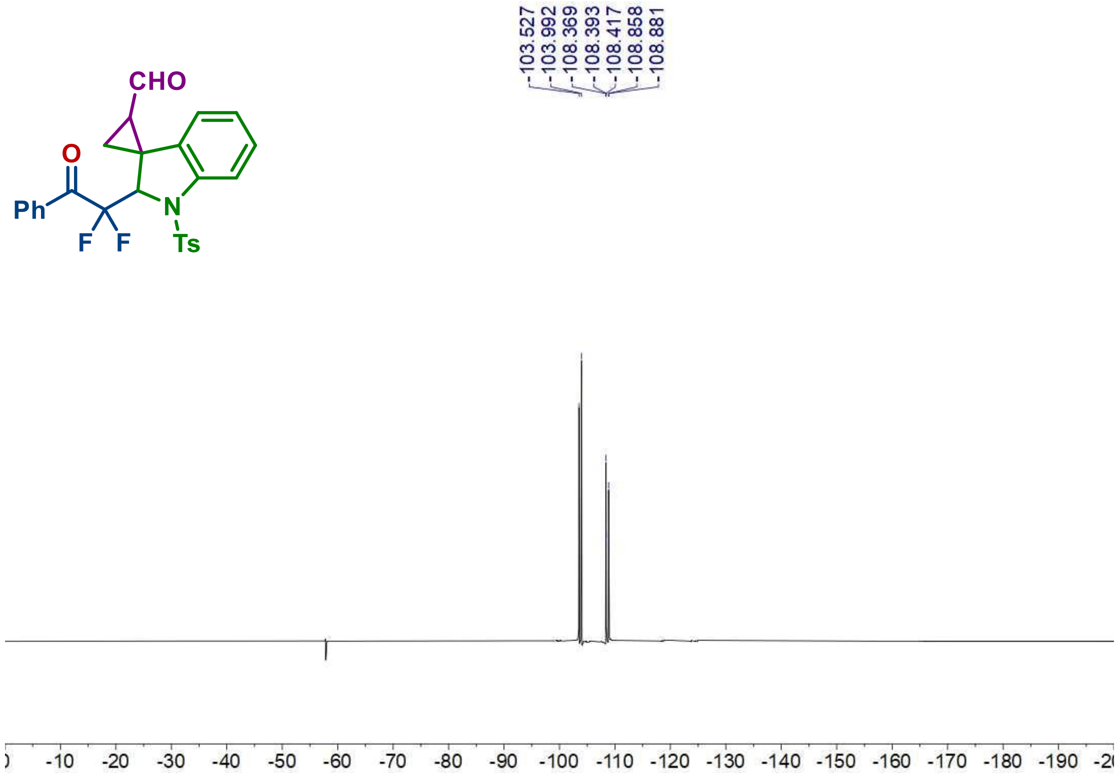

Supplementary Fig. 578 ^{19}F NMR (564 MHz, $CDCl_3$) spectrum of compound **192**

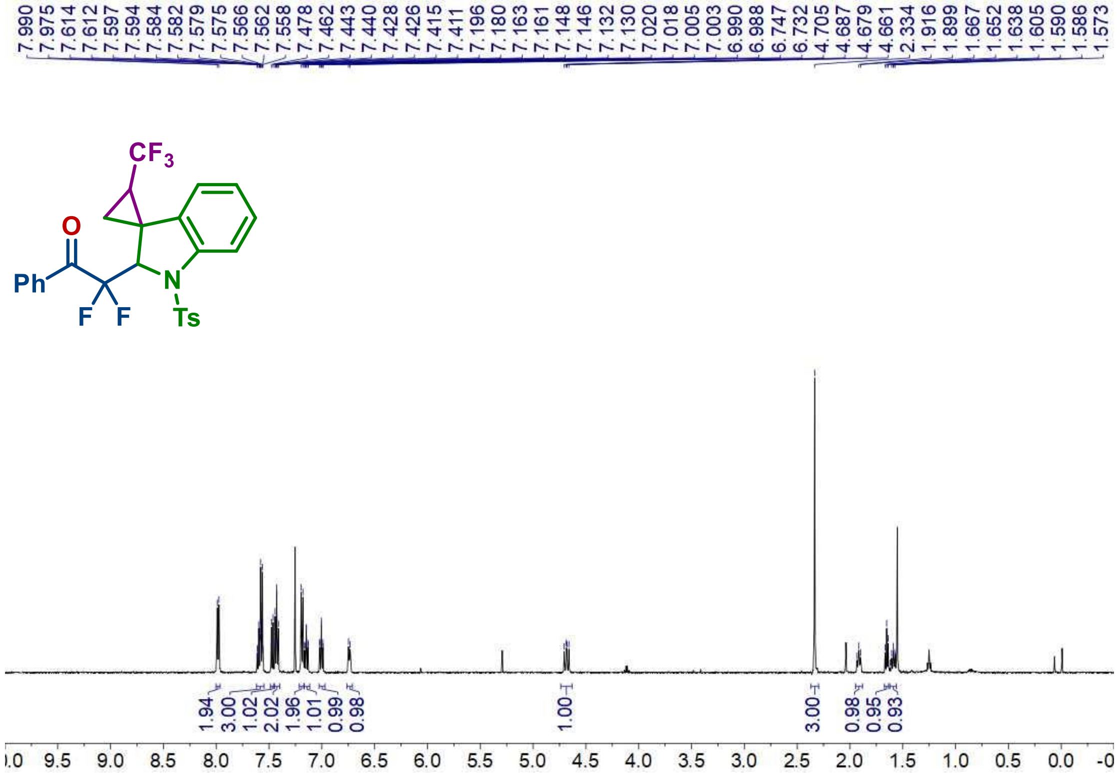

Supplementary Fig. 579 ^{1}H NMR (500 MHz, $CDCl_3$) spectrum of compound **193**

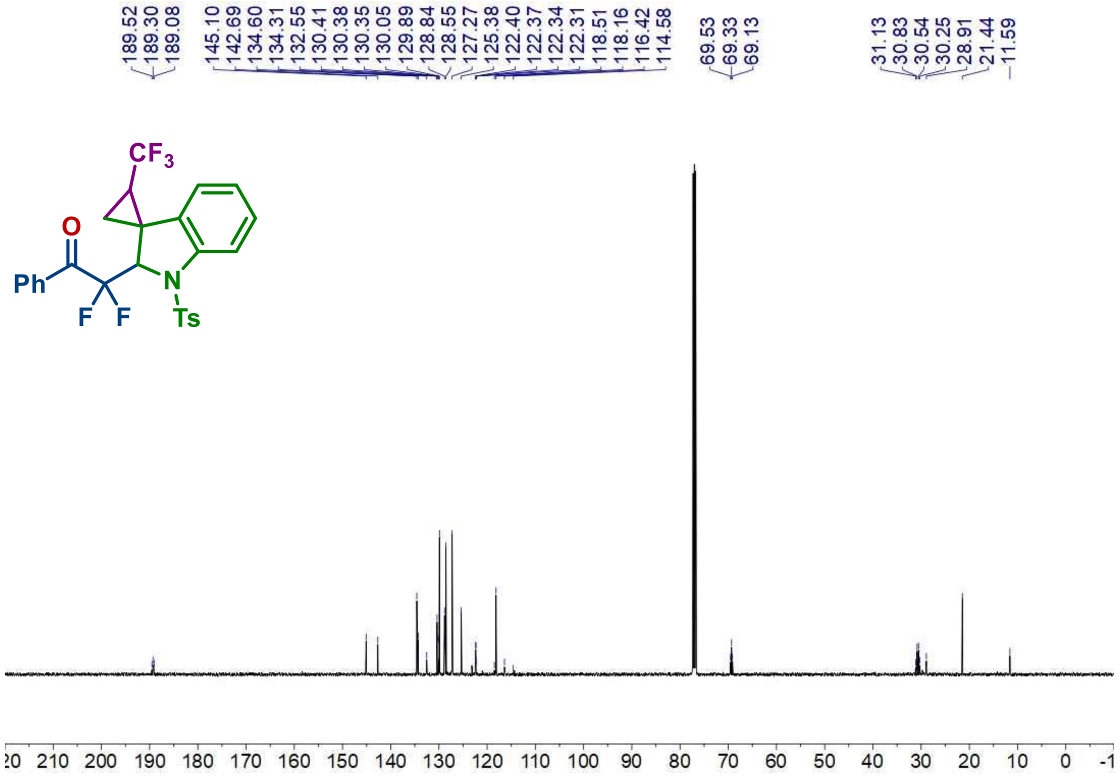

Supplementary Fig. 580 ^{13}C NMR (125 MHz, $CDCl_3$) spectrum of compound **193**

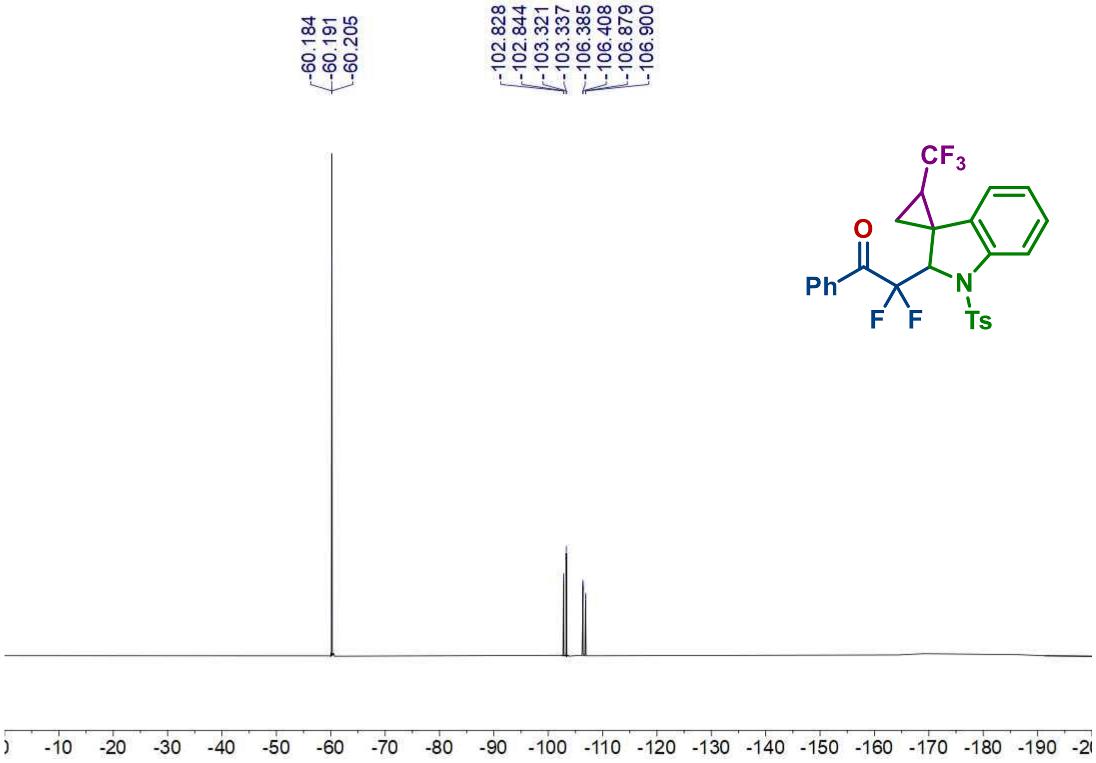

Supplementary Fig. 581 ^{19}F NMR (564 MHz, $CDCl_3$) spectrum of compound **193**

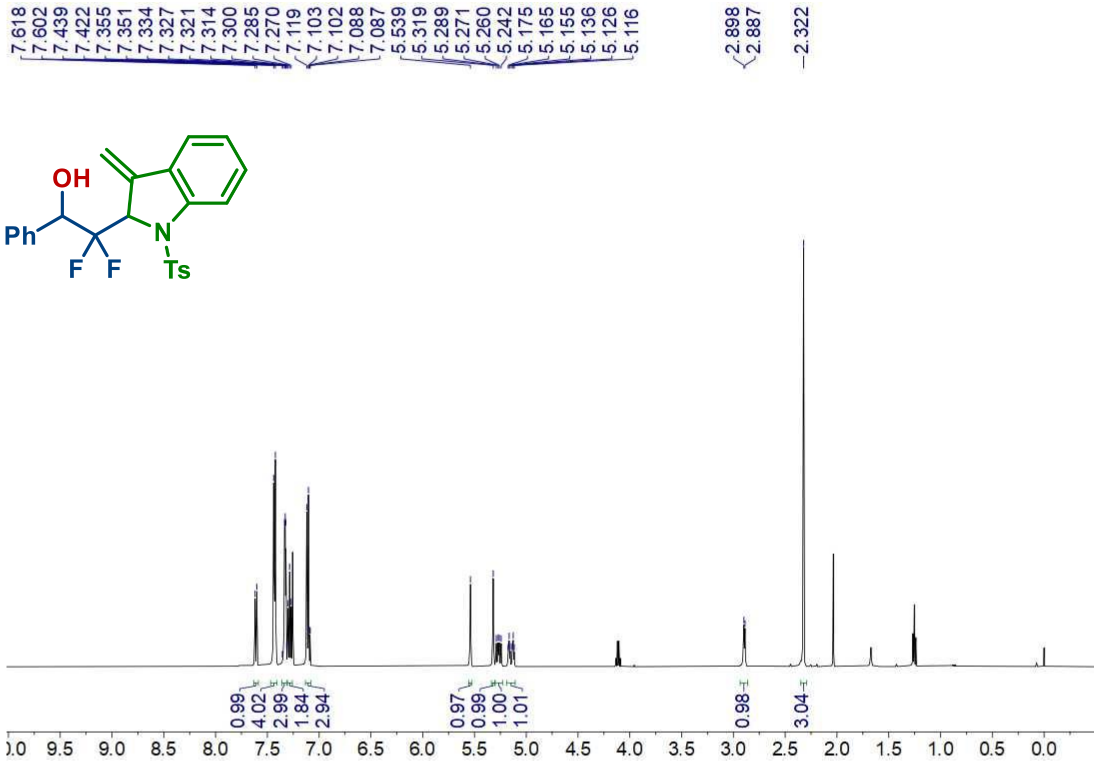

Supplementary Fig. 582 ^{1}H NMR (500 MHz, $CDCl_3$) spectrum of compound **194**

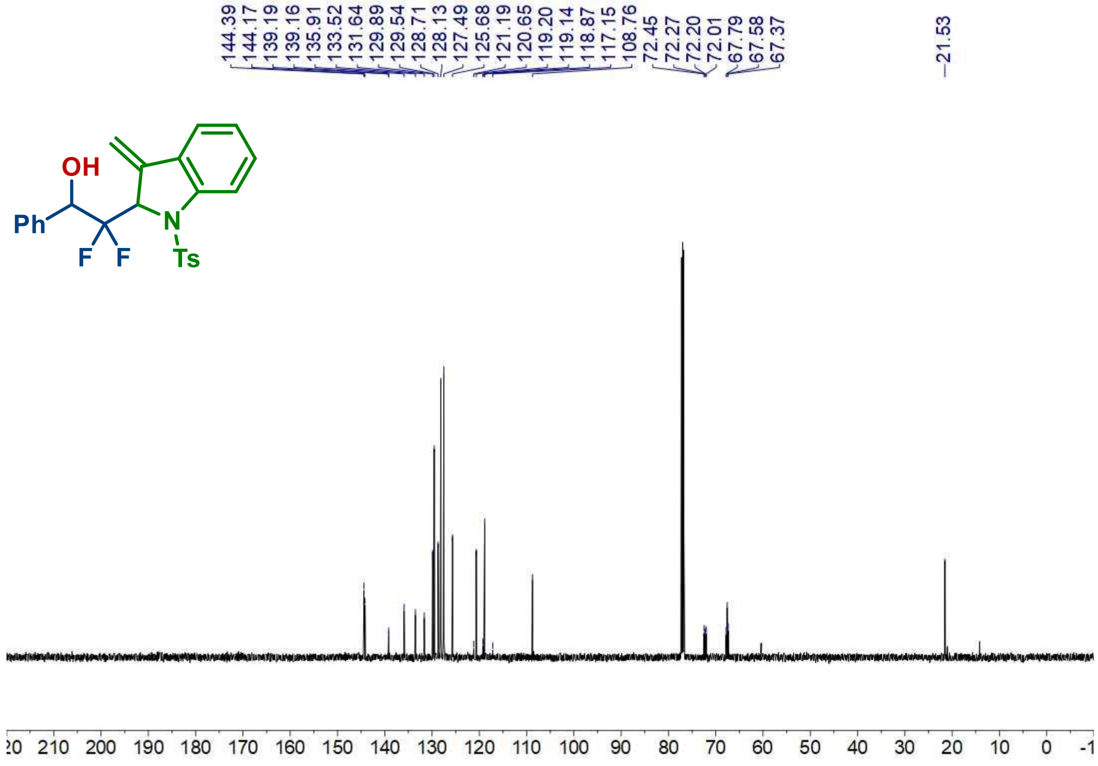

Supplementary Fig. 583 ^{13}C NMR (125 MHz, $CDCl_3$) spectrum of compound **194**

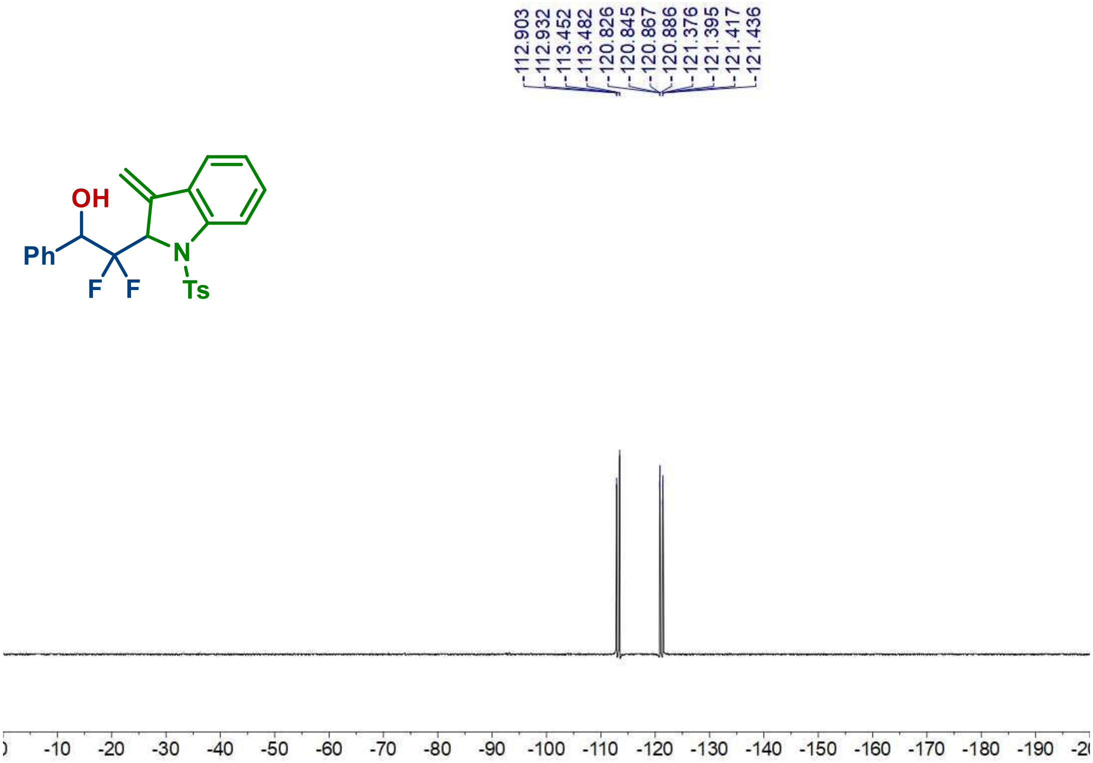

Supplementary Fig. 584 ^{19}F NMR (470 MHz, $CDCl_3$) spectrum of compound **194**

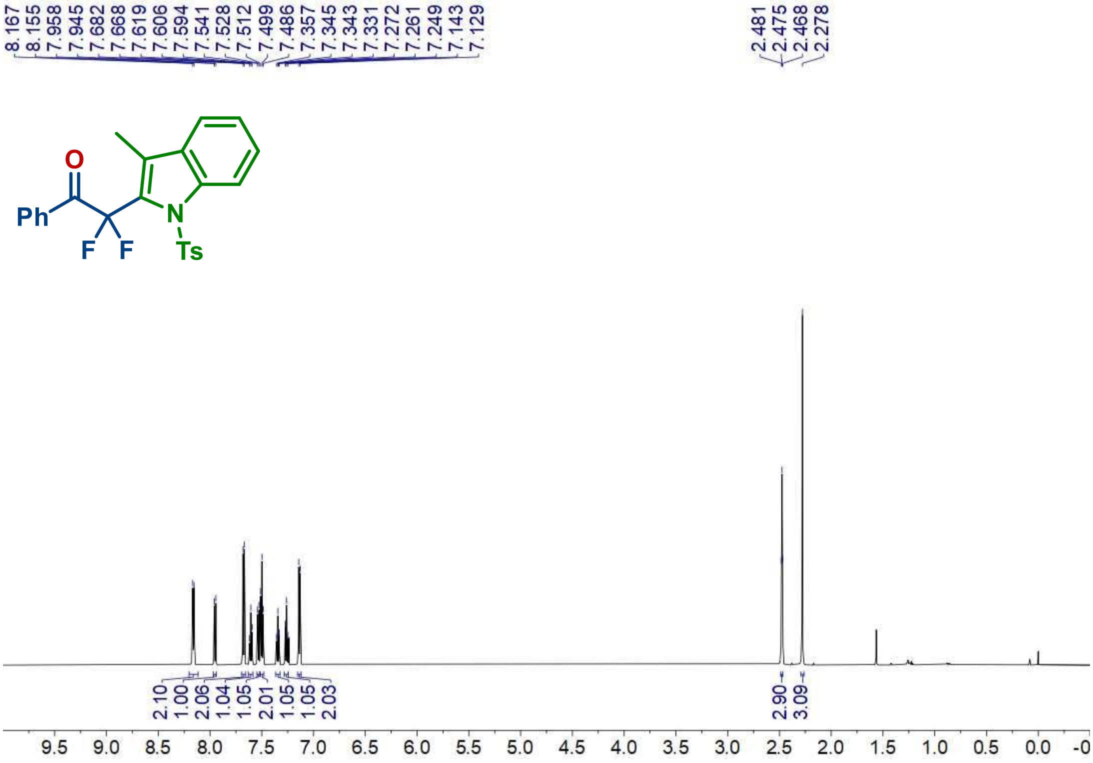

Supplementary Fig. 585 ^{1}H NMR (600 MHz, $CDCl_3$) spectrum of compound **195**

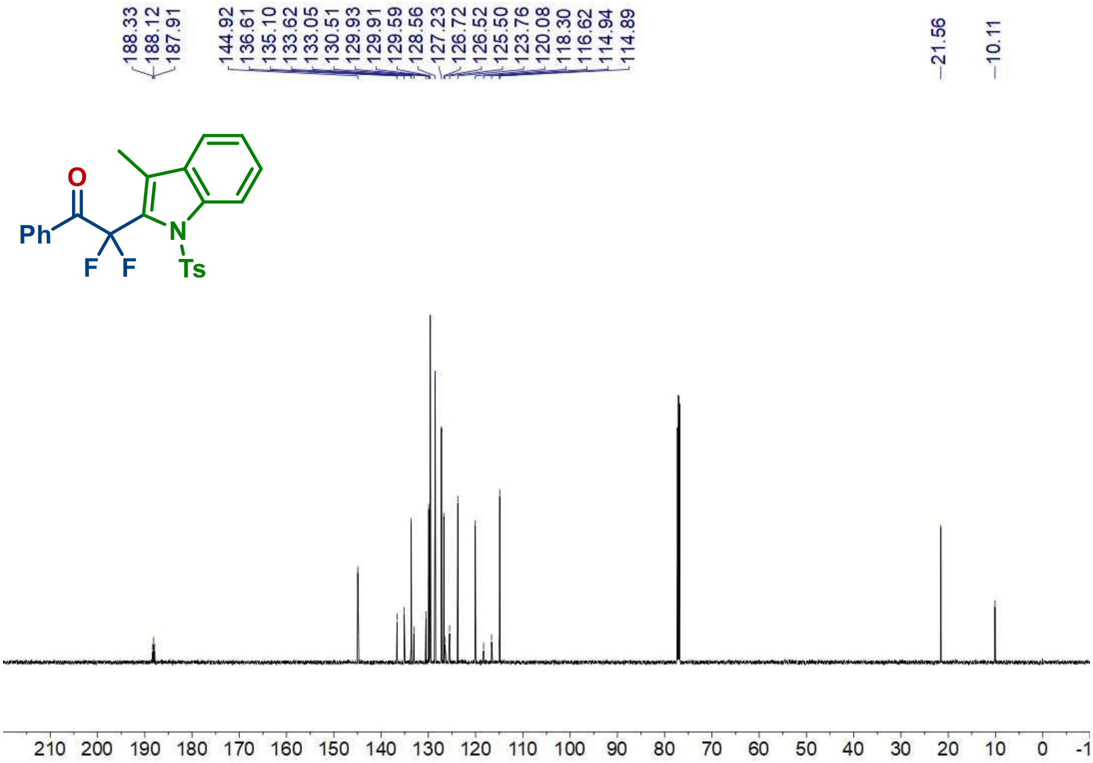

Supplementary Fig. 586 ^{13}C NMR (150 MHz, $CDCl_3$) spectrum of compound **195**

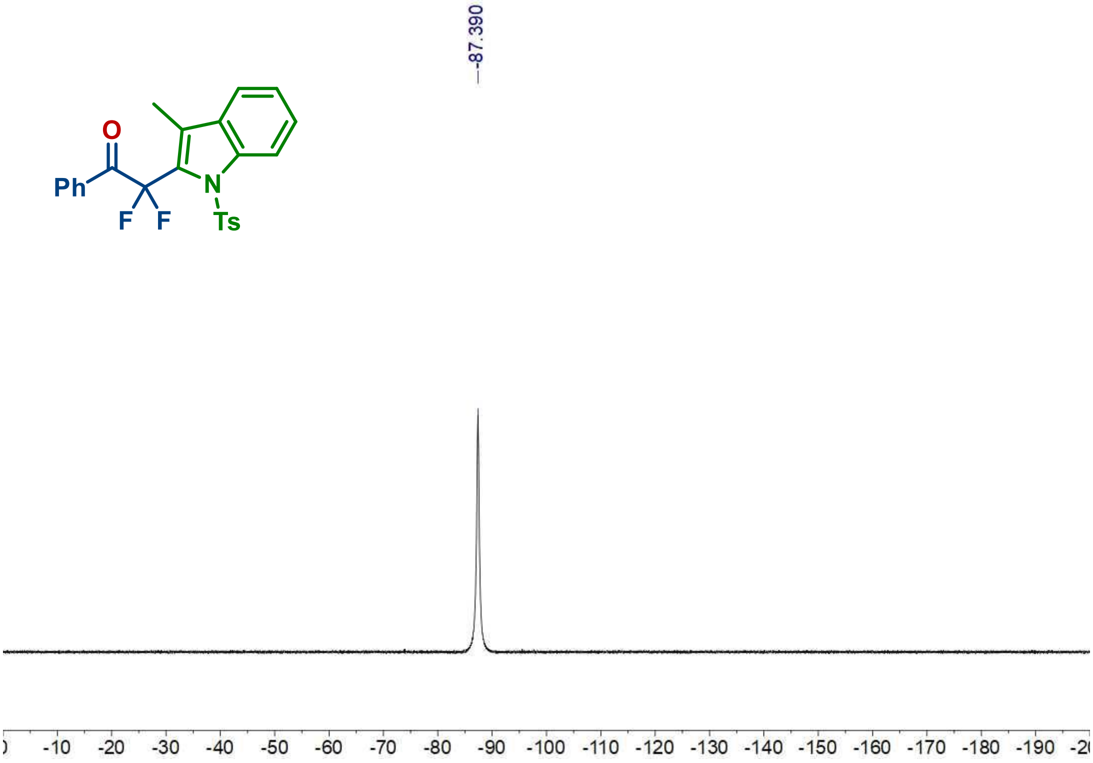

Supplementary Fig. 587 ^{13}C NMR (150 MHz, $CDCl_3$) spectrum of compound **195**

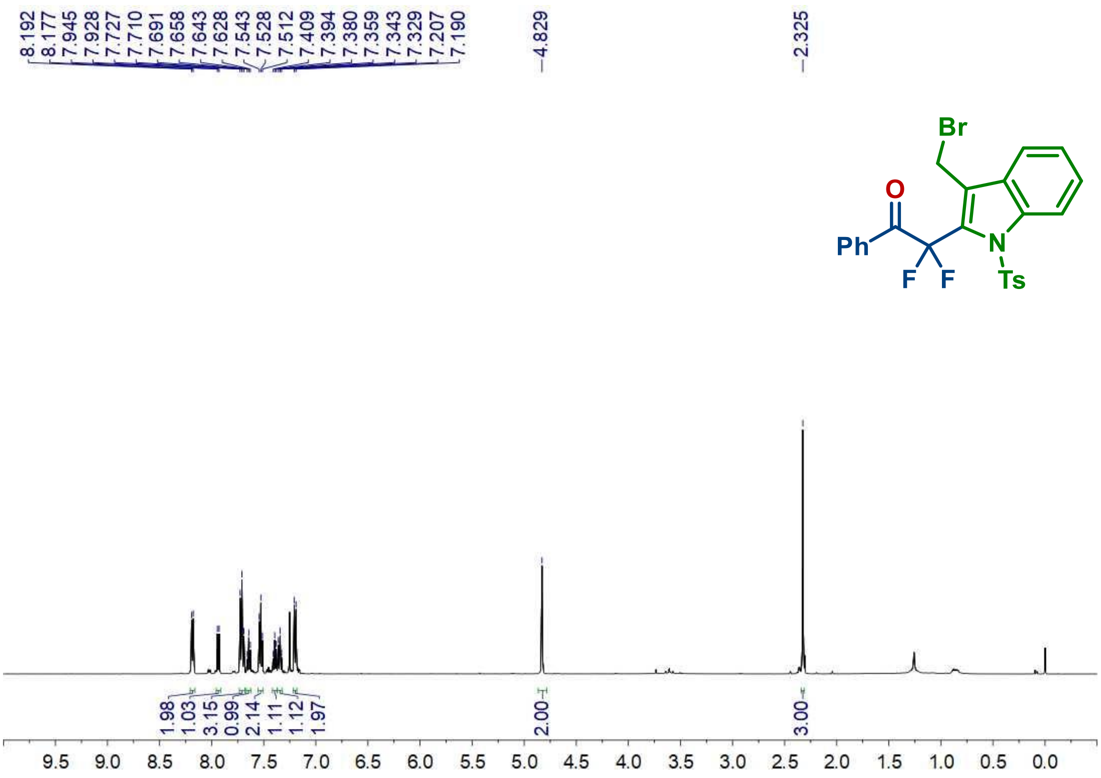

Supplementary Fig. 588 ^{1}H NMR (500 MHz, $CDCl_3$) spectrum of compound **196**

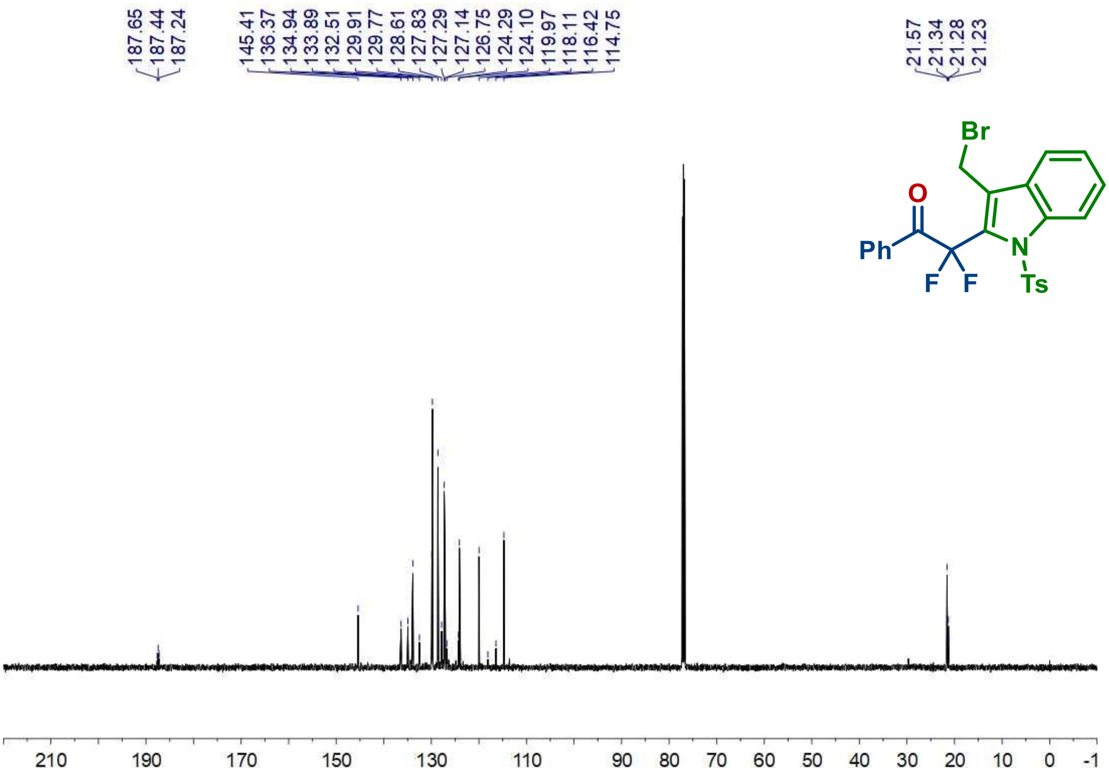

Supplementary Fig. 589 ^{13}C NMR (150 MHz, $CDCl_3$) spectrum of compound **196**

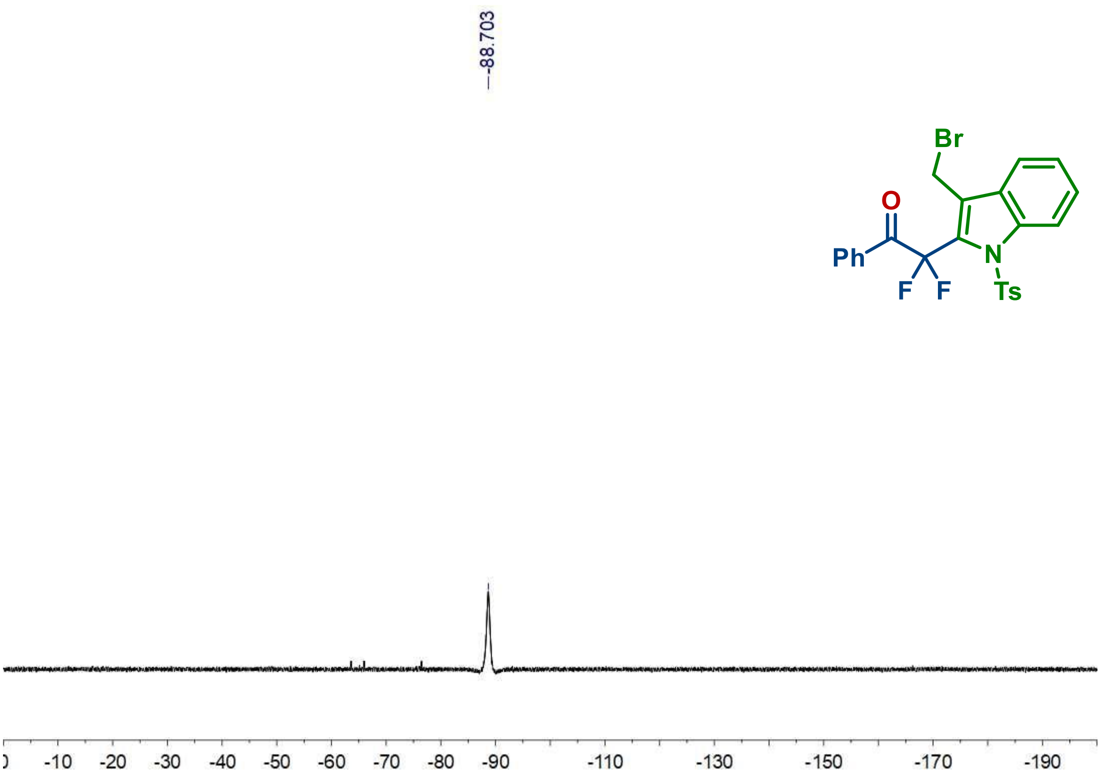

Supplementary Fig. 590 ^{19}F NMR (564 MHz, $CDCl_3$) spectrum of compound **196**

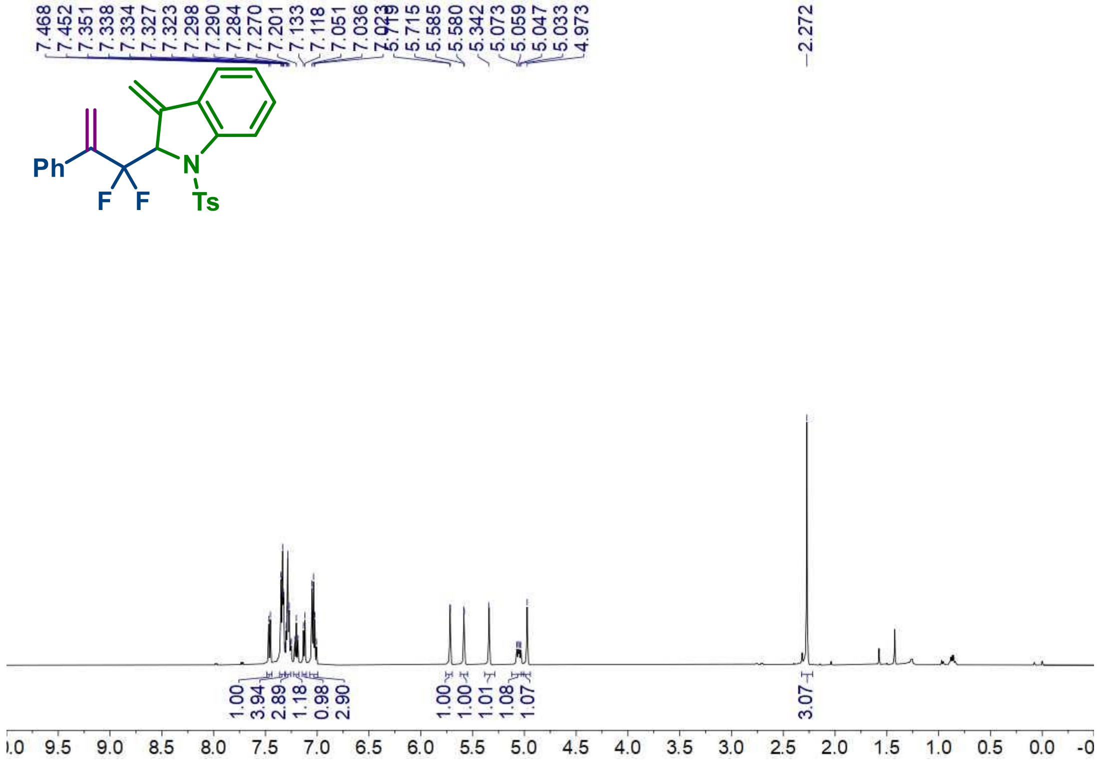

Supplementary Fig. 591 ^{1}H NMR (500 MHz, $CDCl_3$) spectrum of compound **197**

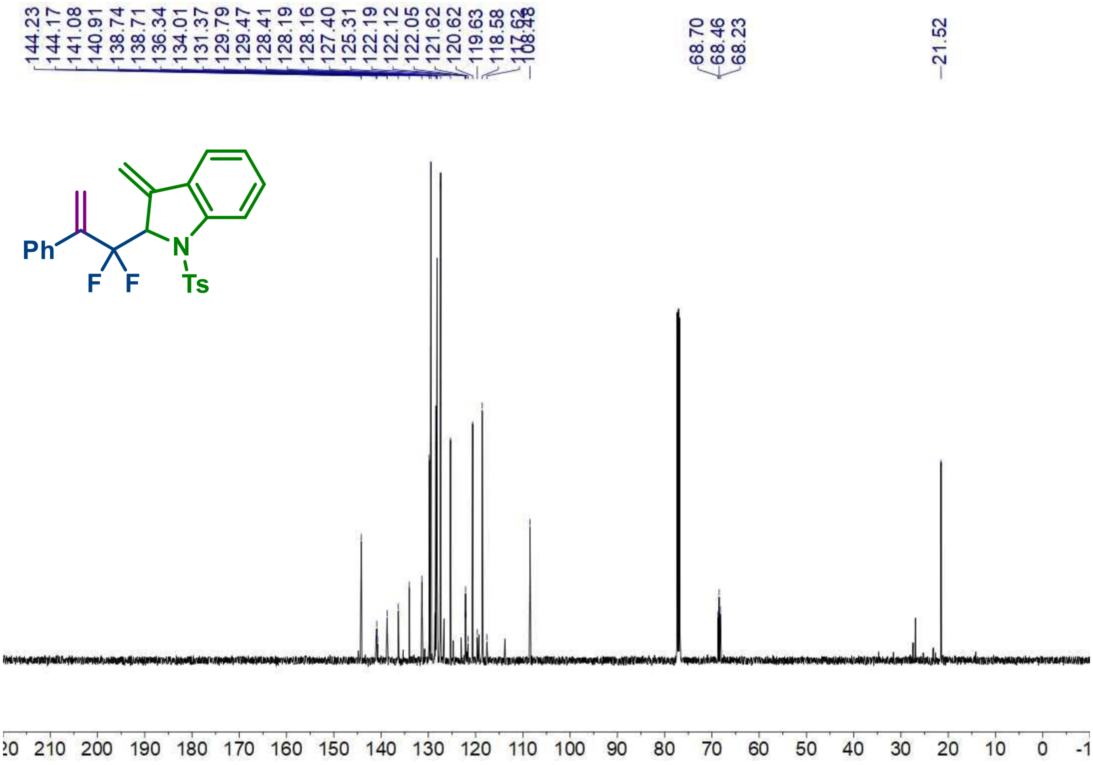

Supplementary Fig. 592 ^{13}C NMR (125 MHz, $CDCl_3$) spectrum of compound **197**

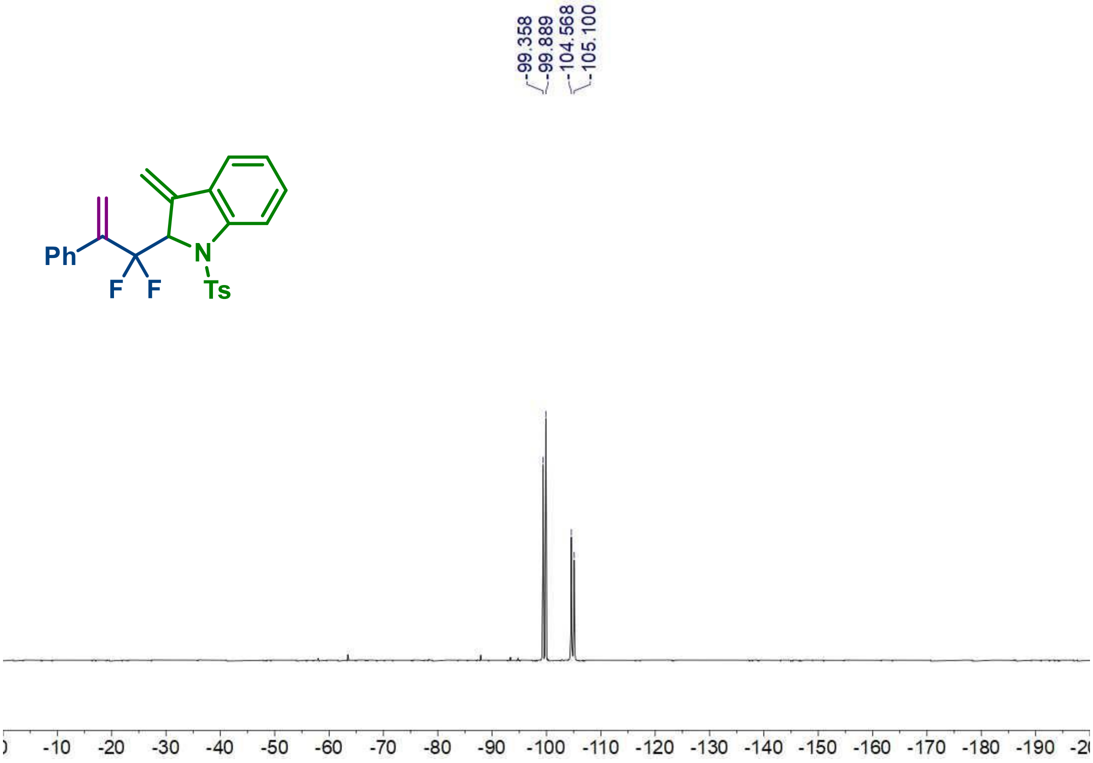

Supplementary Fig. 593 ^{19}F NMR (470 MHz, $CDCl_3$) spectrum of compound **197**

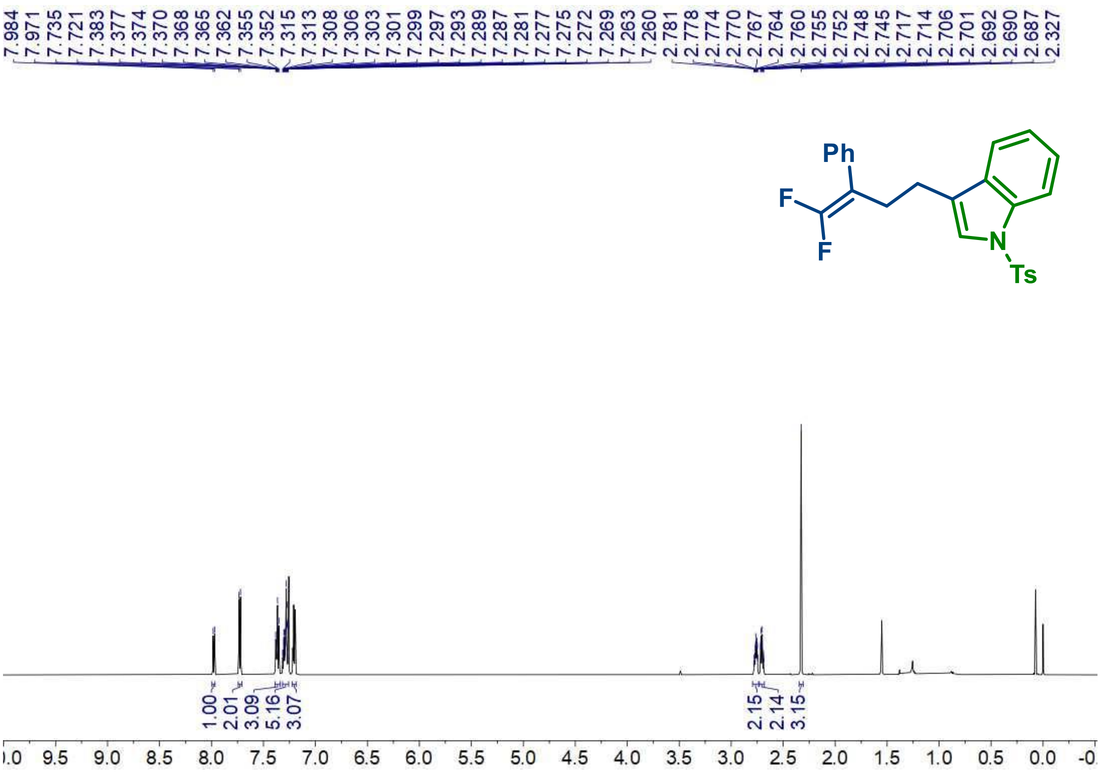

Supplementary Fig. 594 ^{1}H NMR (600 MHz, $CDCl_3$) spectrum of compound **198**

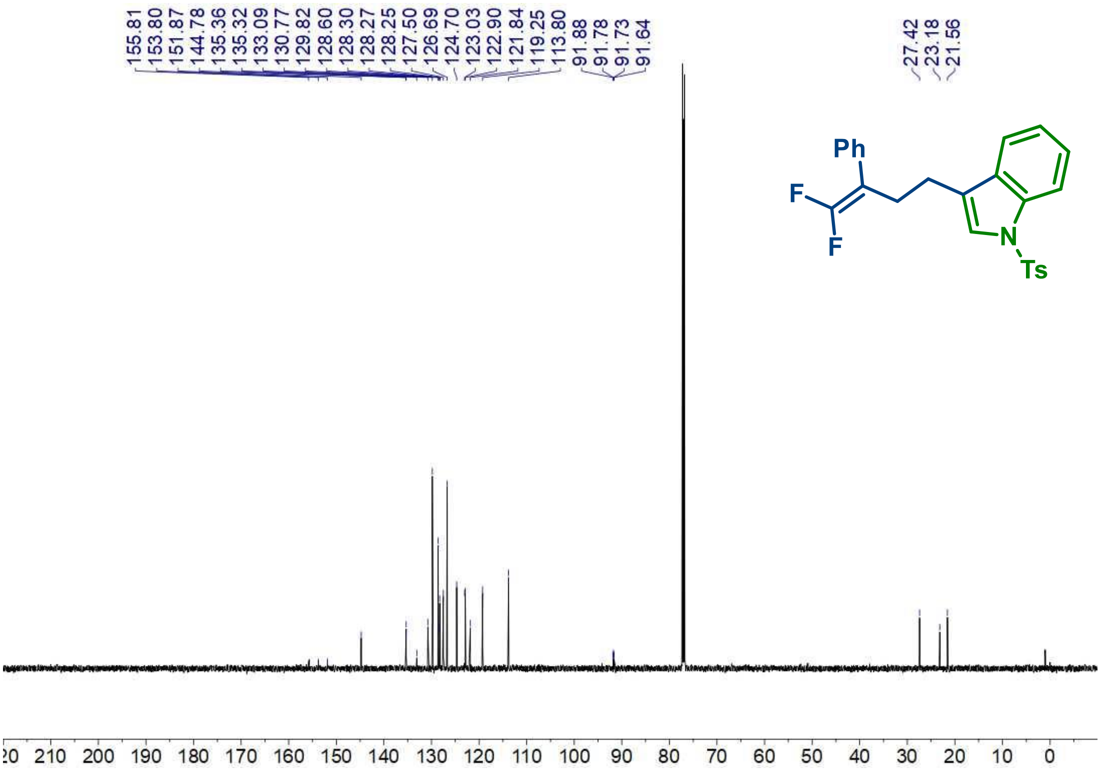

Supplementary Fig. 595 ^{13}C NMR (150 MHz, $CDCl_3$) spectrum of compound **198**

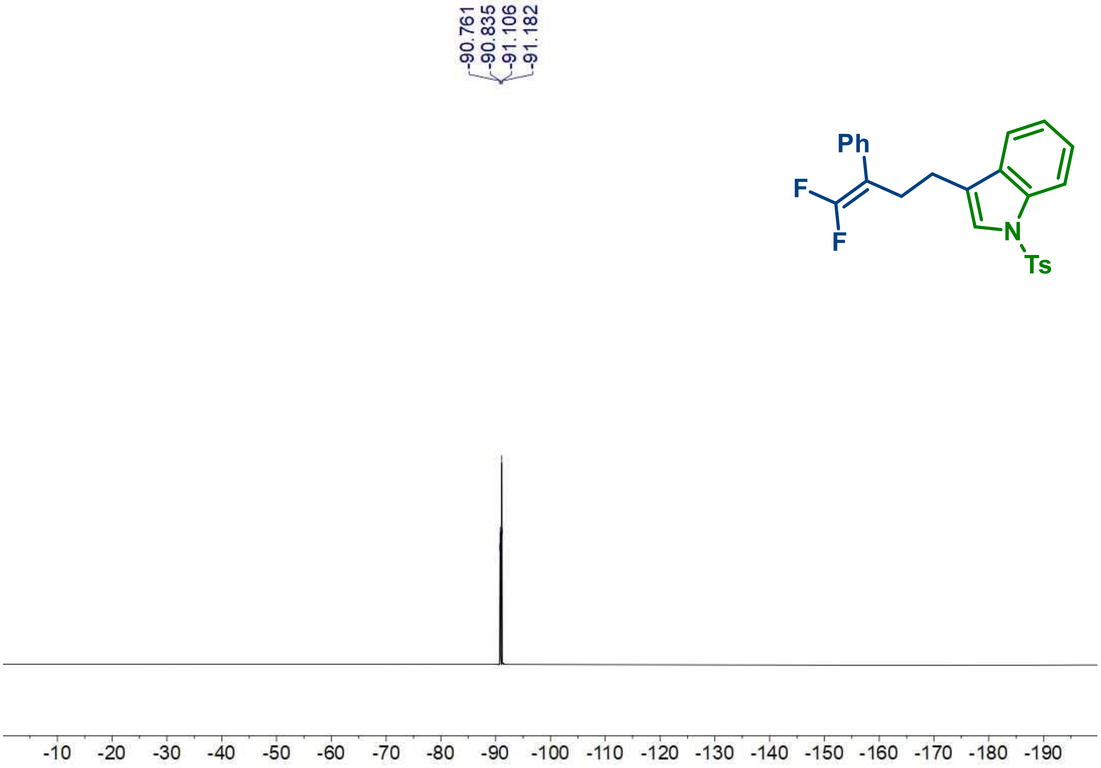

Supplementary Fig. 596 ^{19}F NMR (564 MHz, $CDCl_3$) spectrum of compound **198**

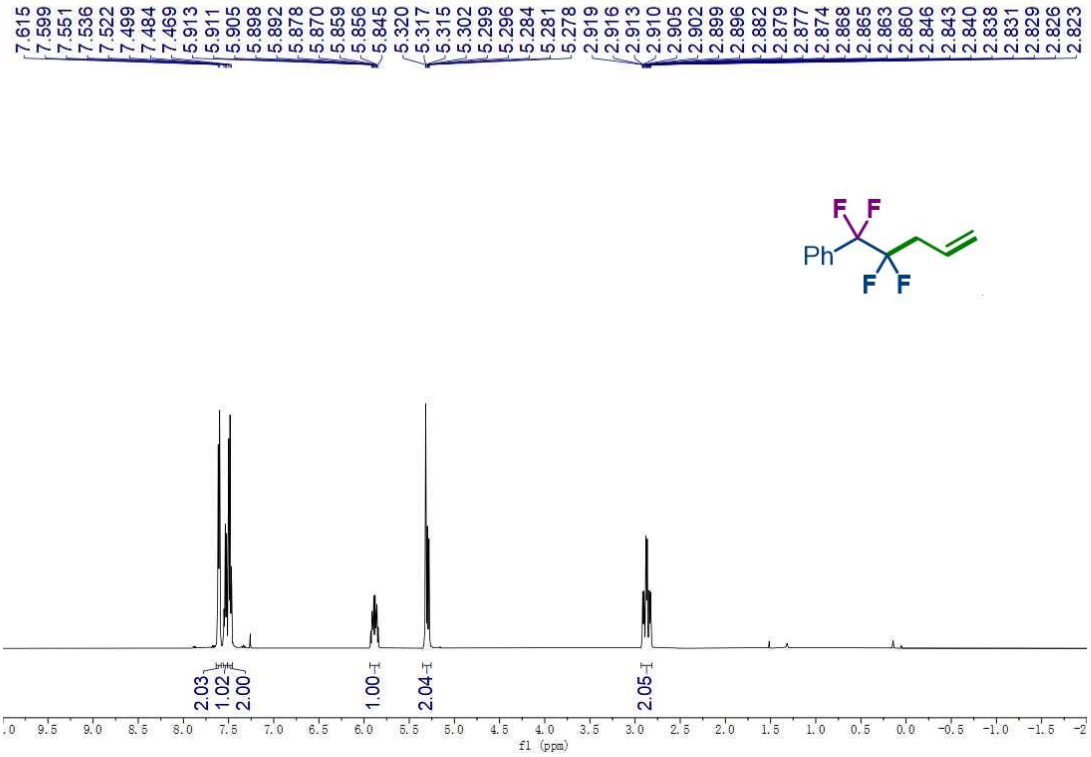

Supplementary Fig. 597 ^{1}H NMR (500 MHz, $CDCl_3$) spectrum of compound **199**

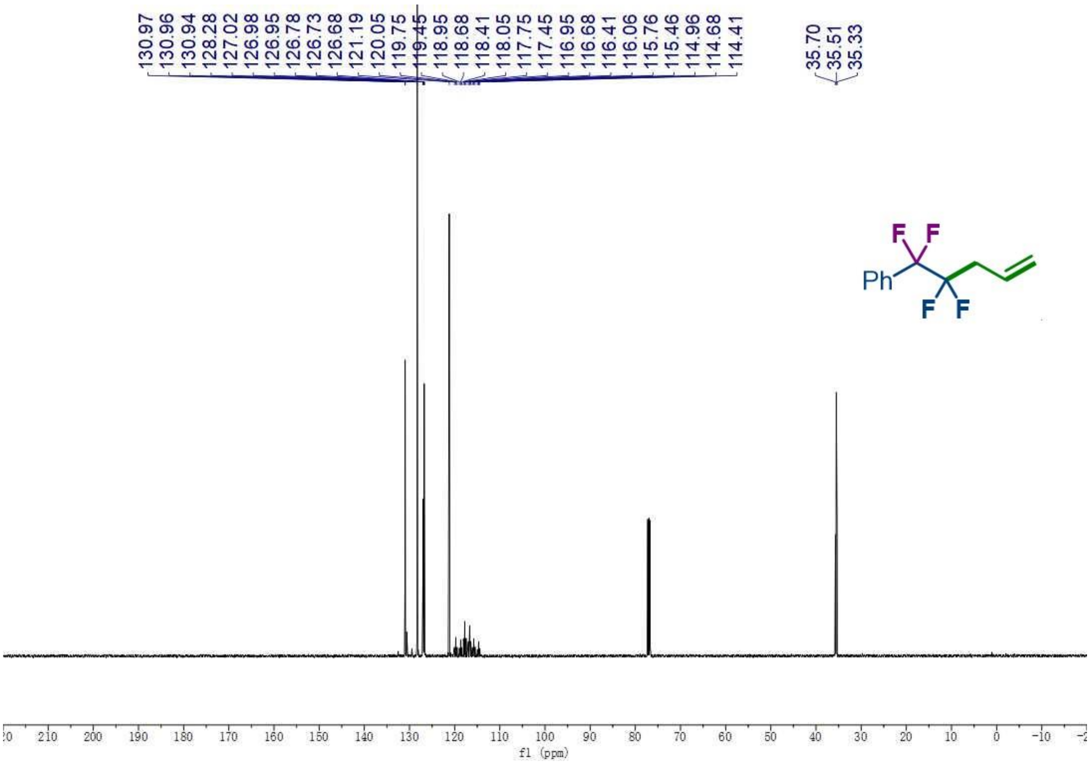

Supplementary Fig. 598 ^{13}C NMR (125 MHz, $CDCl_3$) spectrum of compound **199**

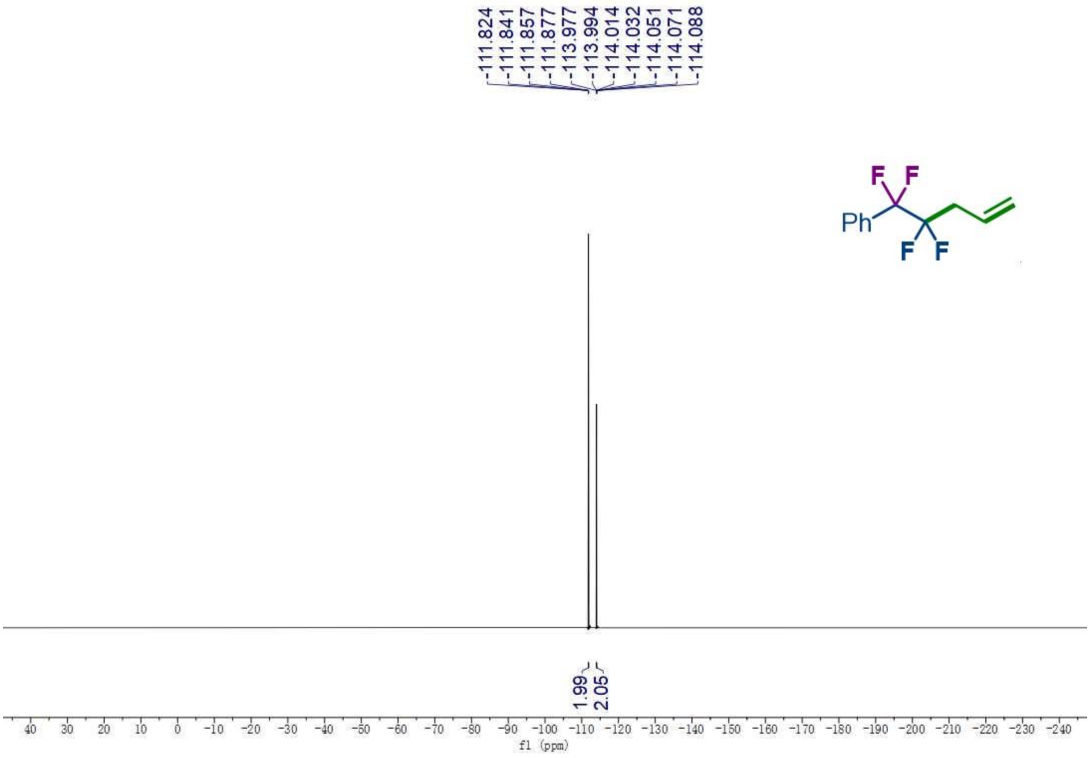

Supplementary Fig. 599 ^{19}F NMR (470 MHz, $CDCl_3$) spectrum of compound **199**

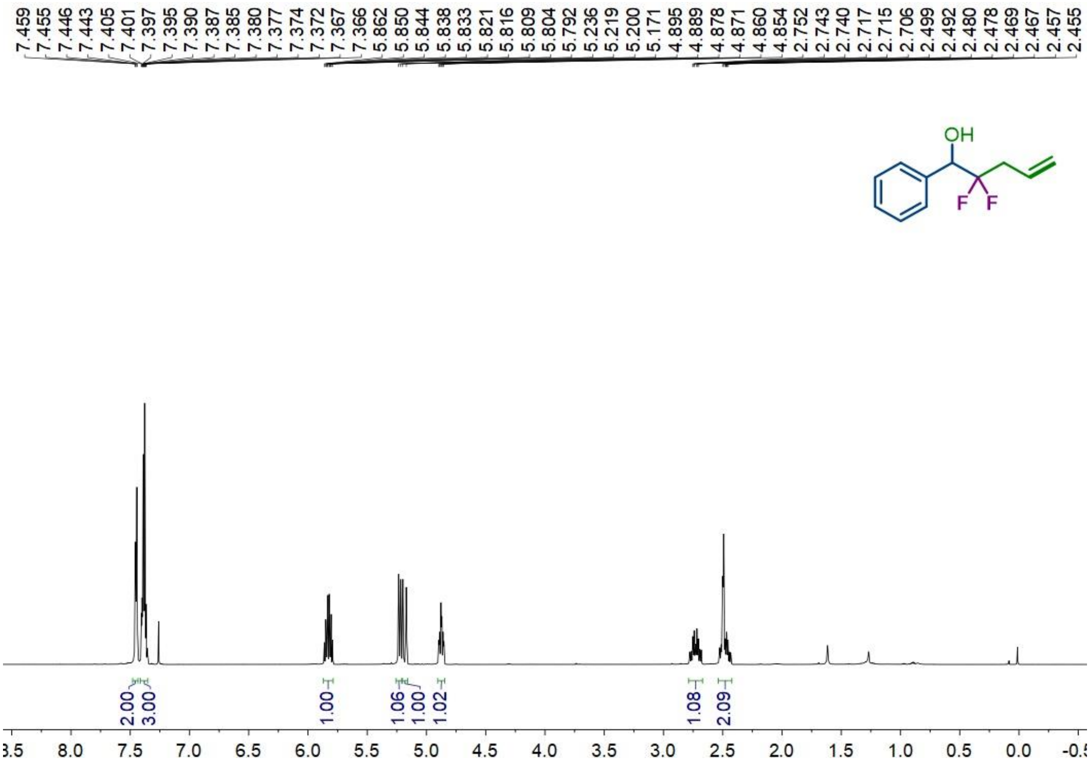

Supplementary Fig. 600 ^{1}H NMR (600 MHz, $CDCl_3$) spectrum of compound **200**

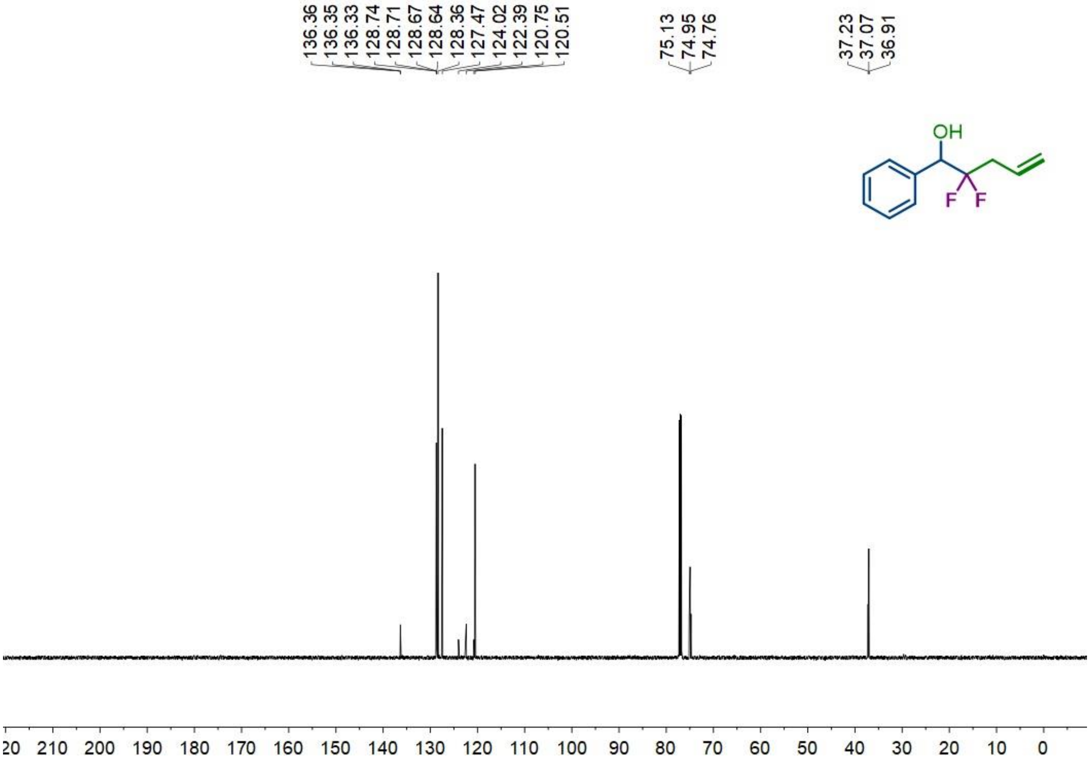

Supplementary Fig. 601 ^{13}C NMR (150 MHz, $CDCl_3$) spectrum of compound **200**

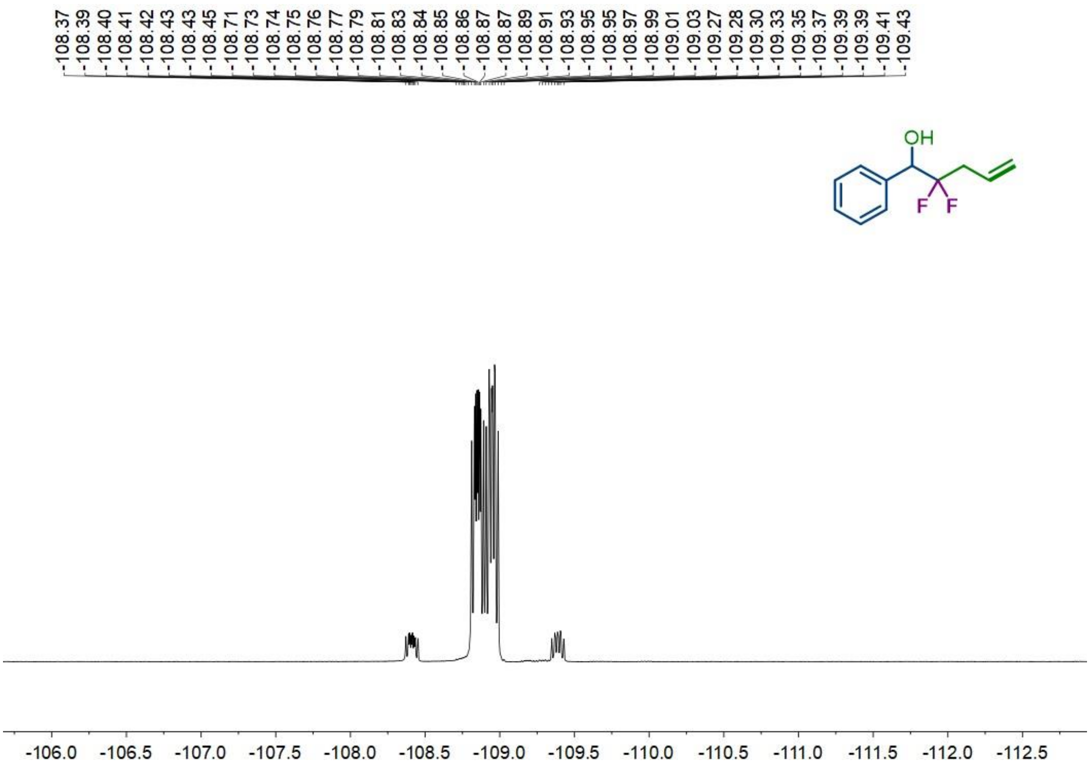

Supplementary Fig. 602 ^{19}F NMR (564 MHz, $CDCl_3$) spectrum of compound **200**

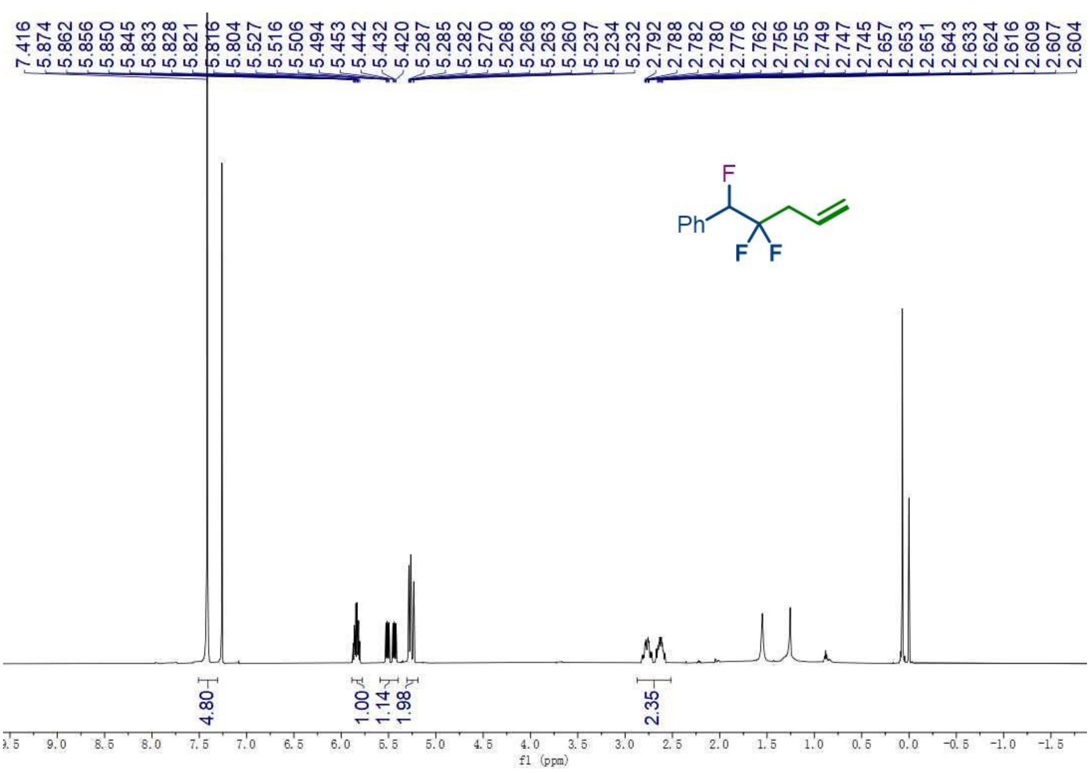

Supplementary Fig. 603 ^{1}H NMR (600 MHz, $CDCl_3$) spectrum of compound **201**

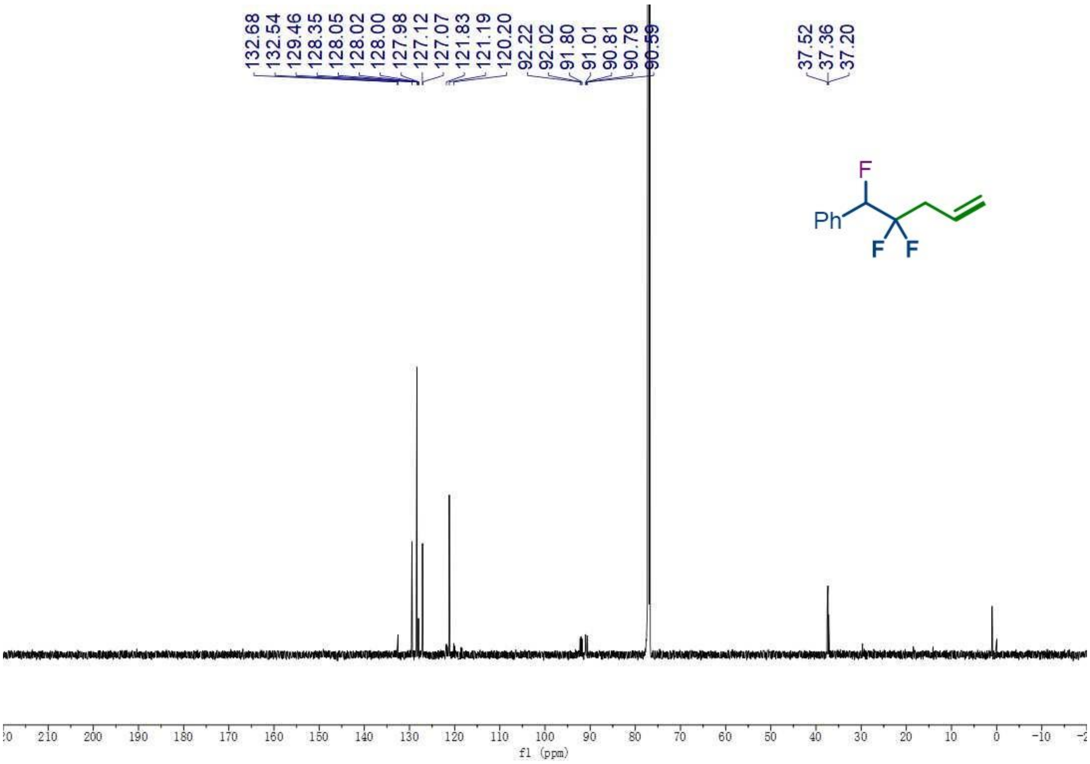

Supplementary Fig. 604 ^{13}C NMR (150 MHz, $CDCl_3$) spectrum of compound **201**

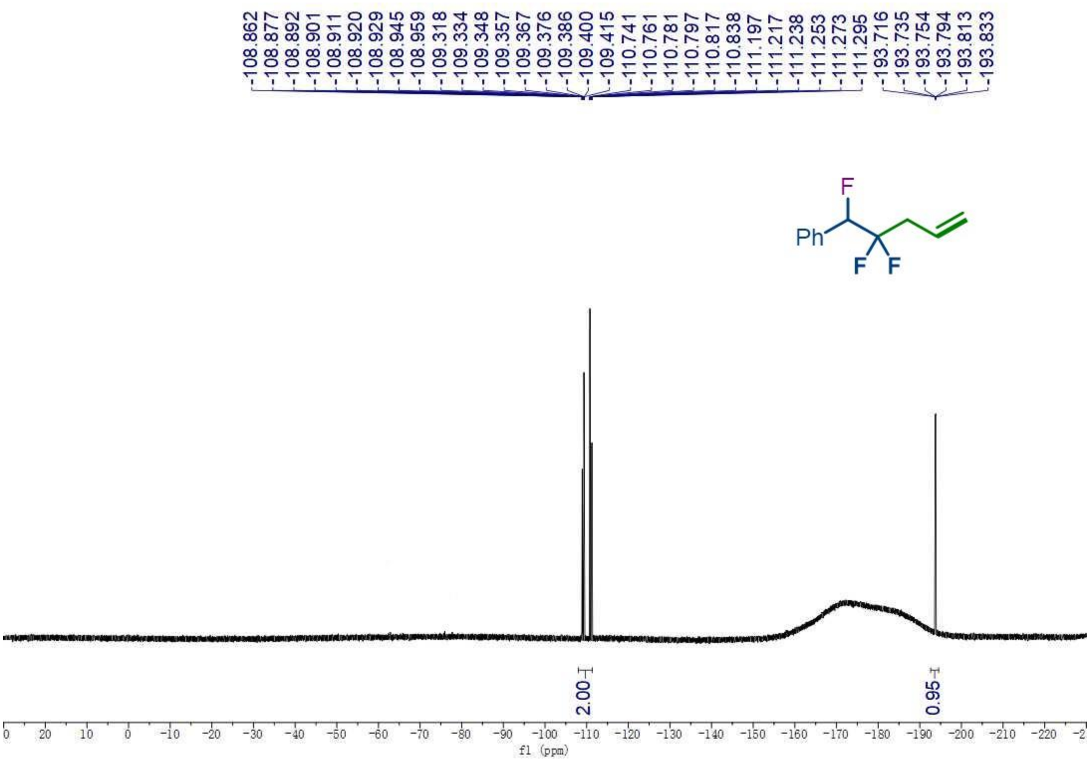

Supplementary Fig. 605 ^{19}F NMR (564 MHz, $CDCl_3$) spectrum of compound **201**

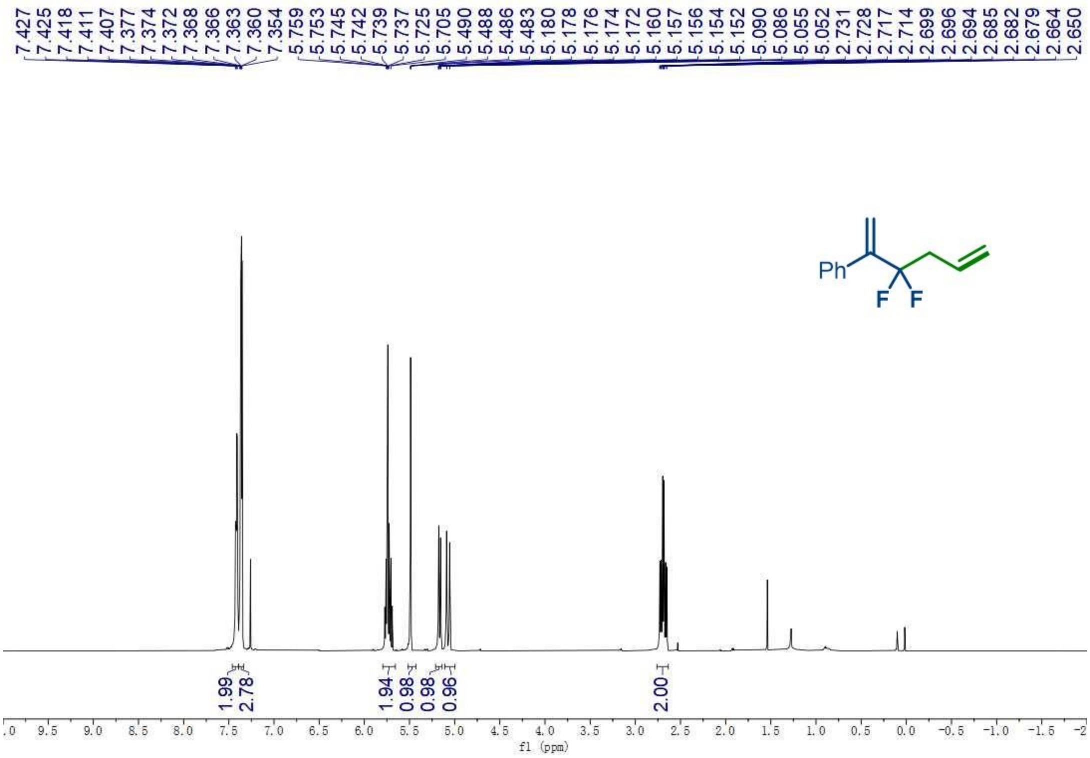

Supplementary Fig. 606 ^{1}H NMR (500 MHz, $CDCl_3$) spectrum of compound **202**

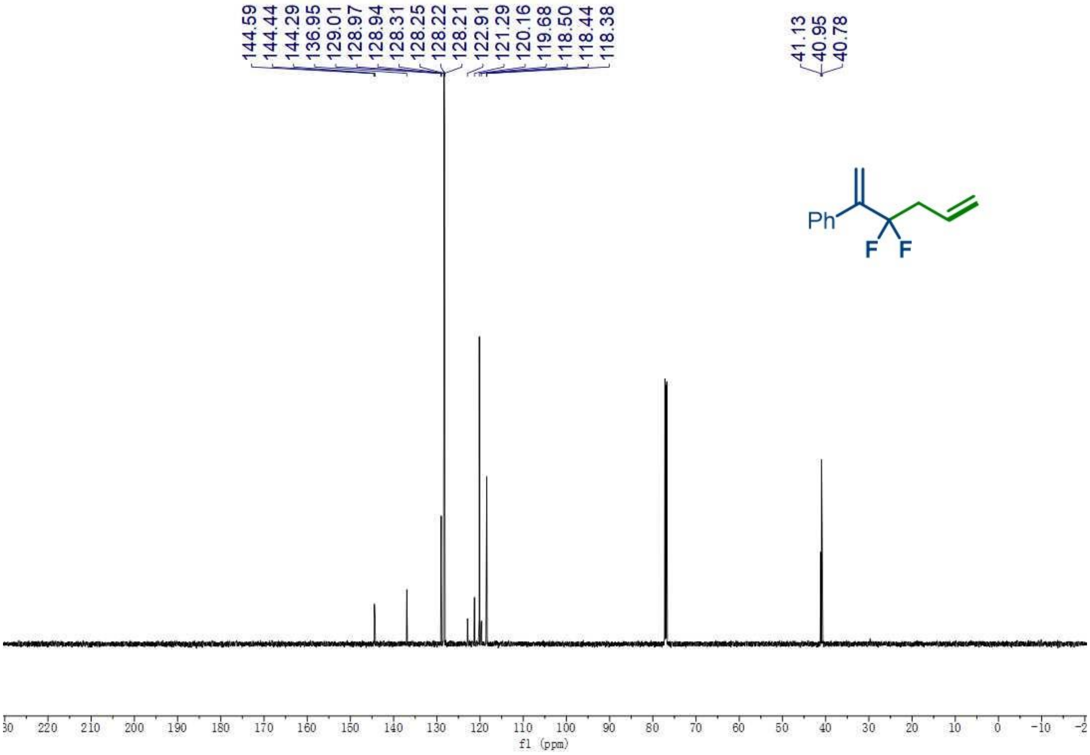

Supplementary Fig. 607 ^{13}C NMR (150 MHz, $CDCl_3$) spectrum of compound **202**

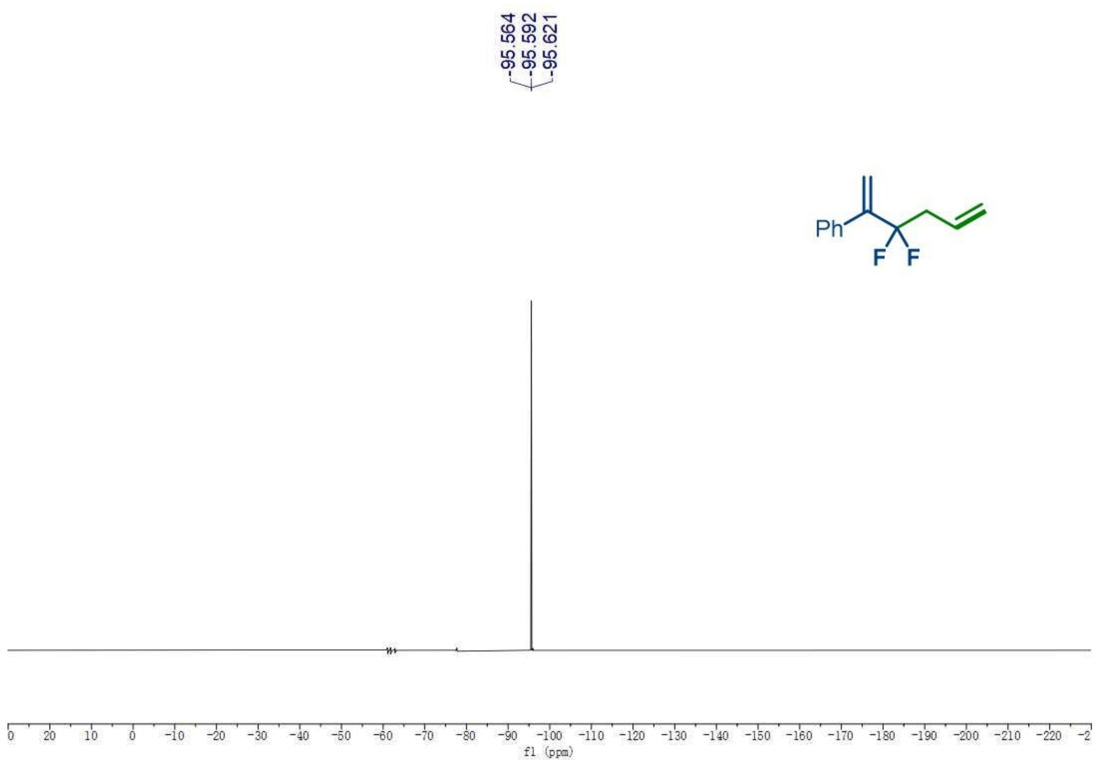

Supplementary Fig. 608 ^{19}F NMR (564 MHz, $CDCl_3$) spectrum of compound **202**

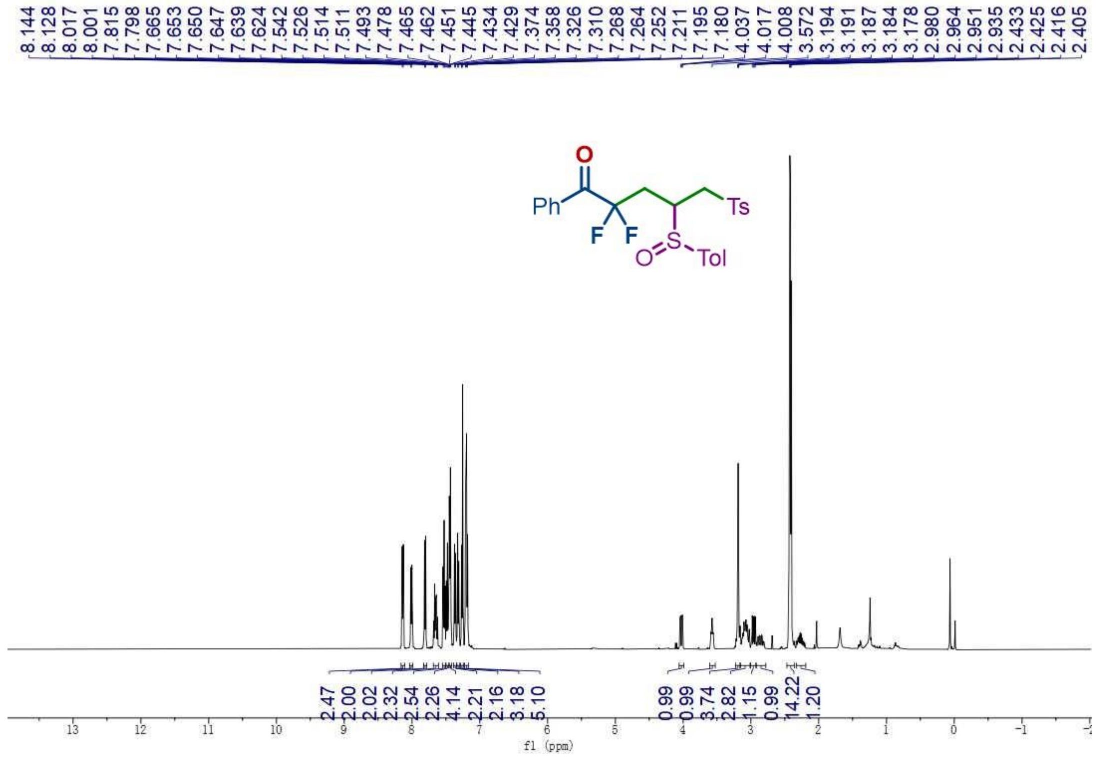

Supplementary Fig. 609 ^{1}H NMR (500 MHz, $CDCl_3$) spectrum of compound **203**

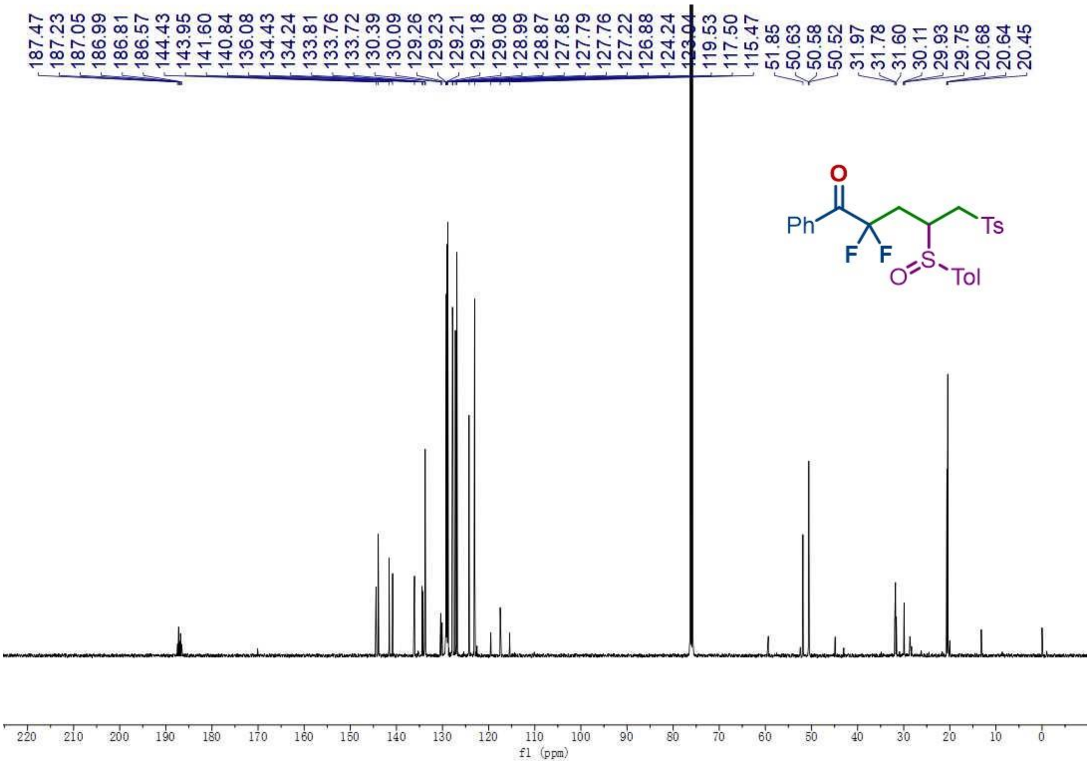

Supplementary Fig. 610 ^{13}C NMR (125 MHz, $CDCl_3$) spectrum of compound **203**

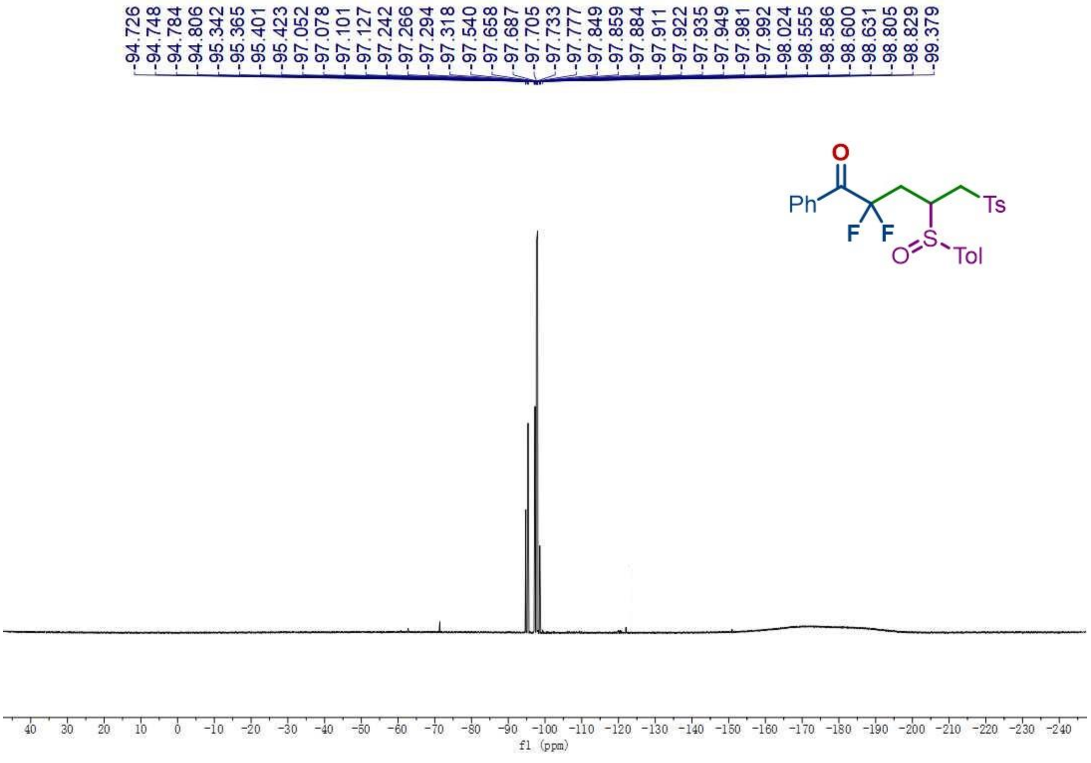

Supplementary Fig. 611 ^{19}F NMR (470 MHz, $CDCl_3$) spectrum of compound **203**

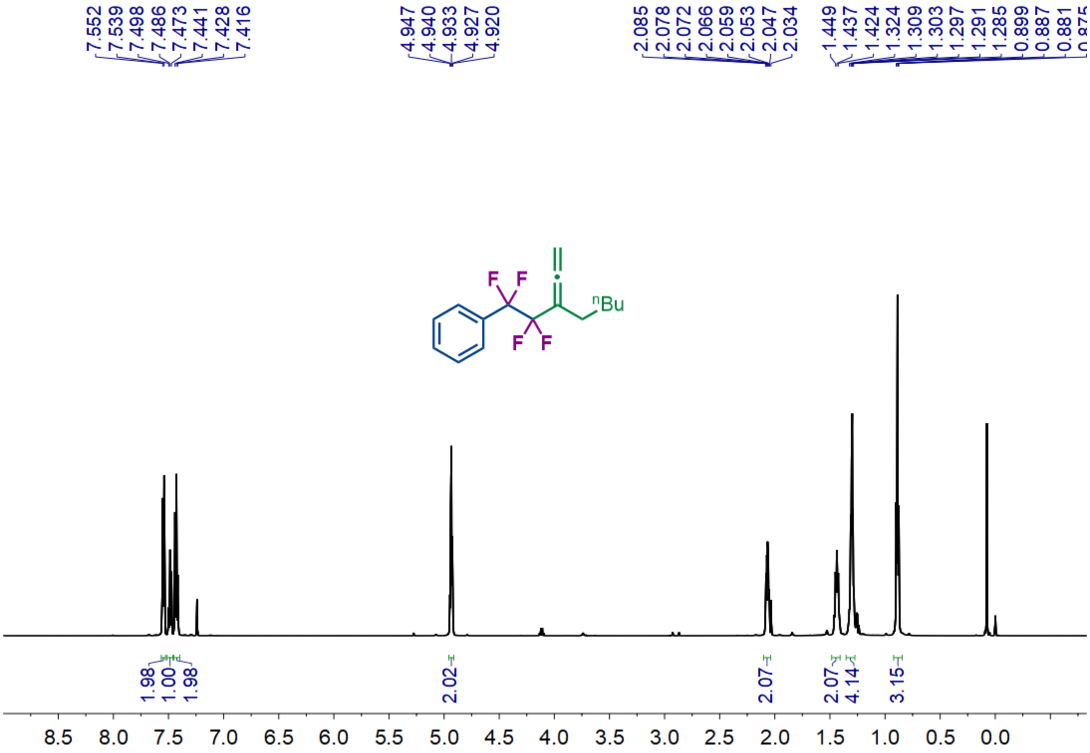

Supplementary Fig. 612 ^{1}H NMR (600 MHz, $CDCl_3$) spectrum of compound **204**

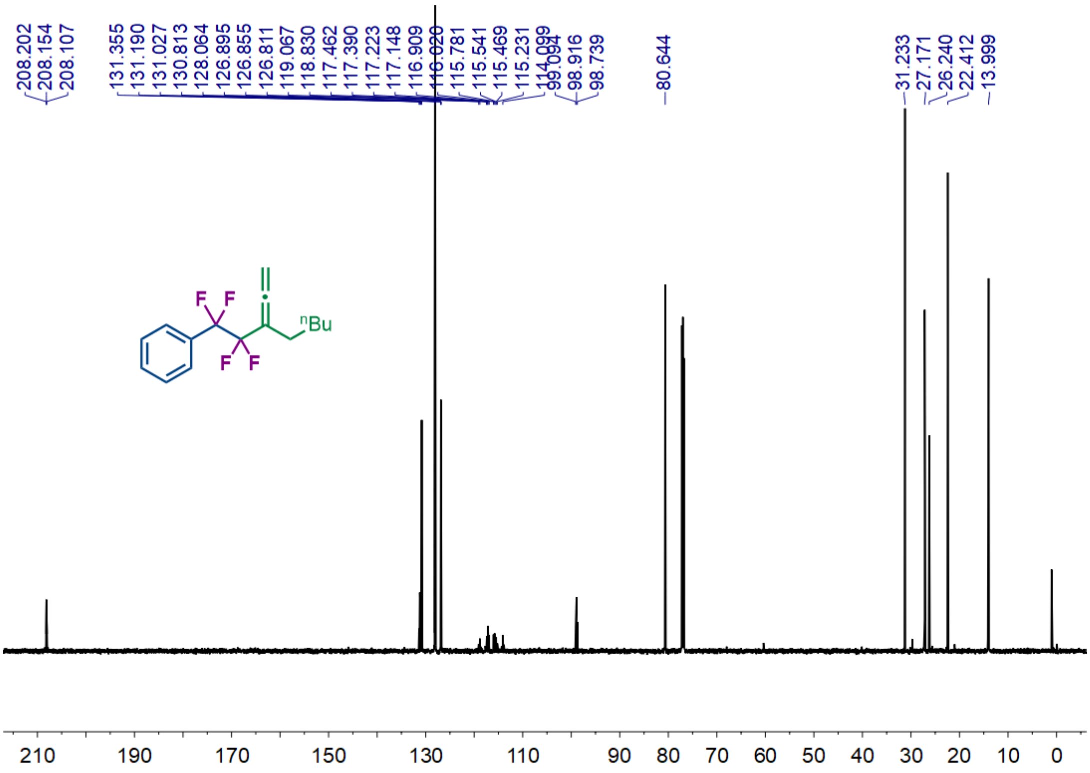

Supplementary Fig. 613 ^{13}C NMR (150 MHz, $CDCl_3$) spectrum of compound **204**

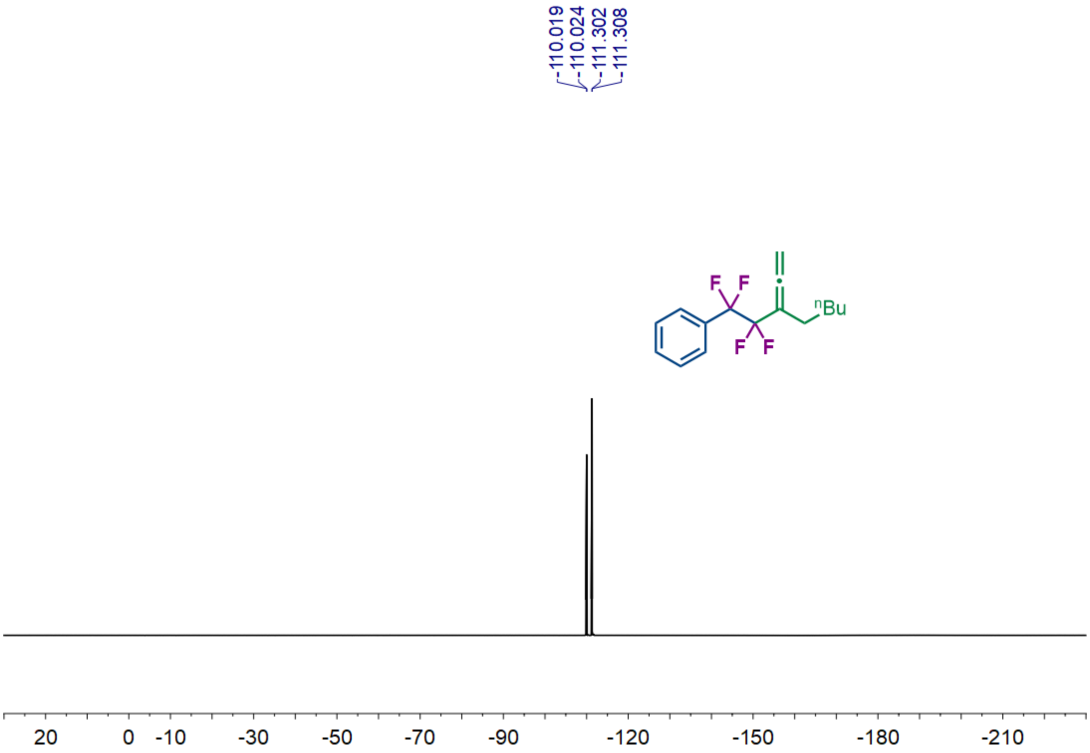

Supplementary Fig. 614 ^{19}F NMR (564 MHz, $CDCl_3$) spectrum of compound **204**

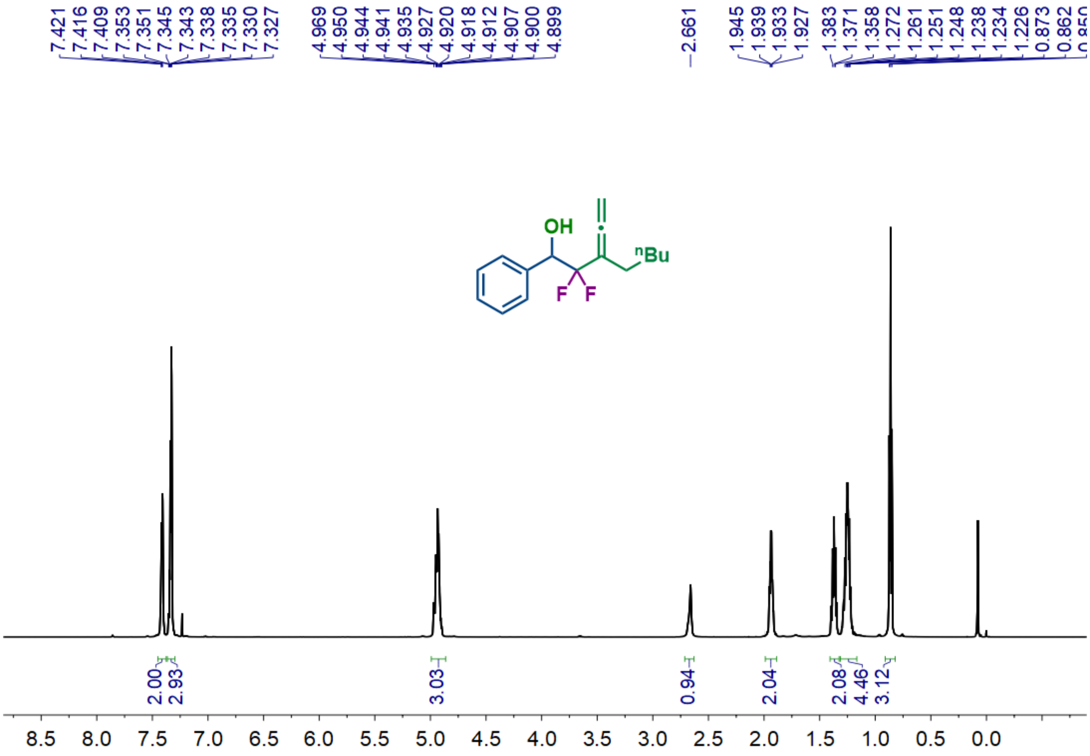

Supplementary Fig. 615 ^{1}H NMR (600 MHz, $CDCl_3$) spectrum of compound **205**

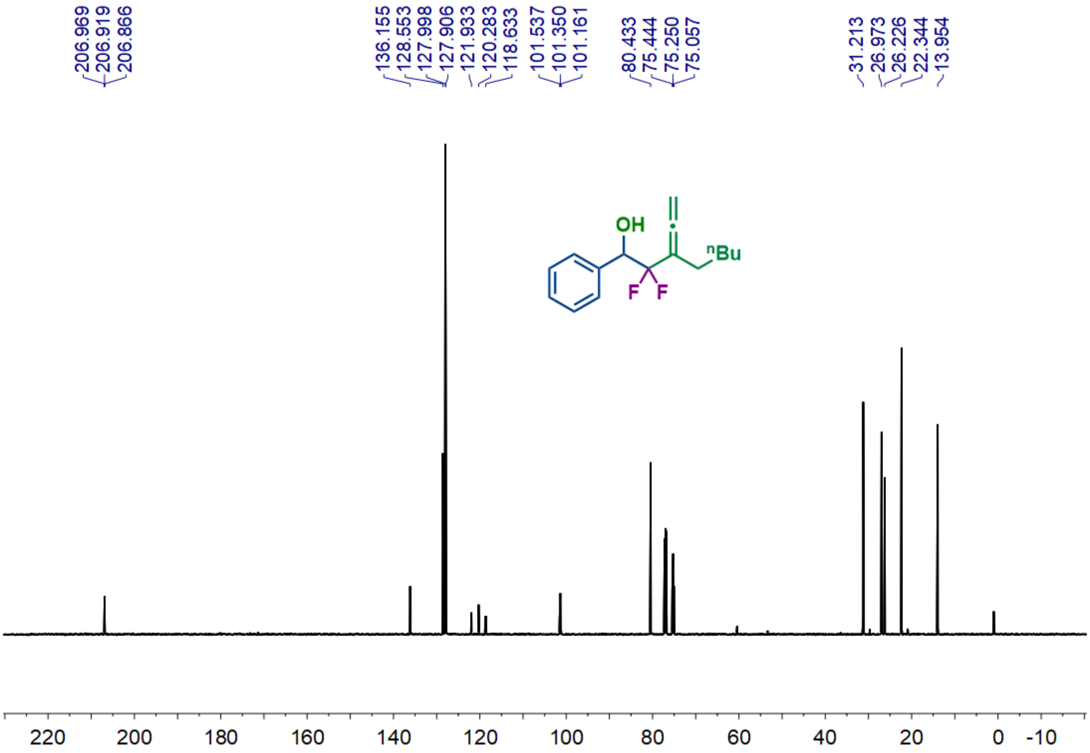

Supplementary Fig. 616 ^{13}C NMR (150 MHz, $CDCl_3$) spectrum of compound **205**

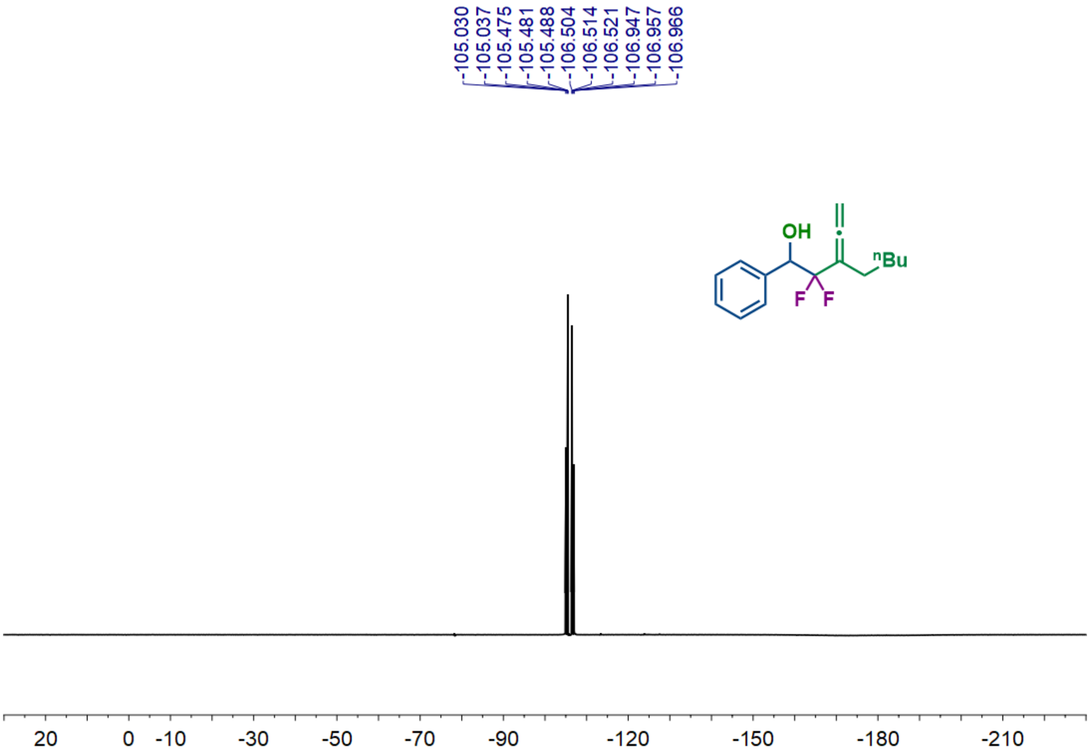

Supplementary Fig. 617 ^{19}F NMR (564 MHz, $CDCl_3$) spectrum of compound **205**

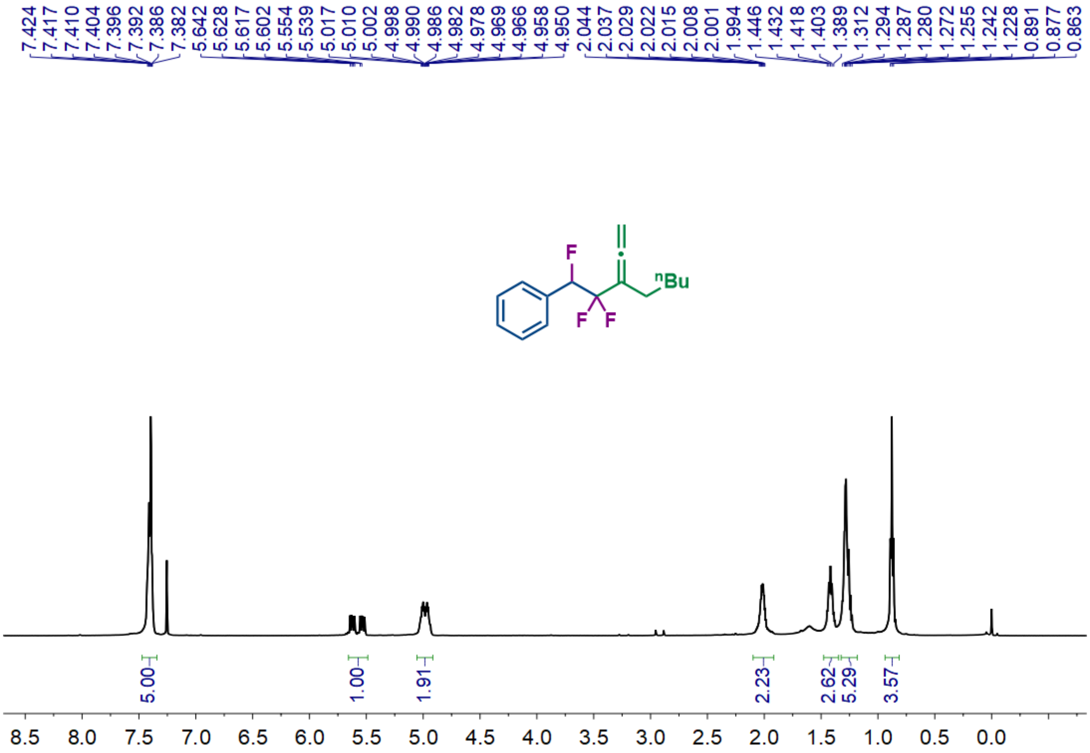

Supplementary Fig. 618 ^{1}H NMR (500 MHz, $CDCl_3$) spectrum of compound **206**

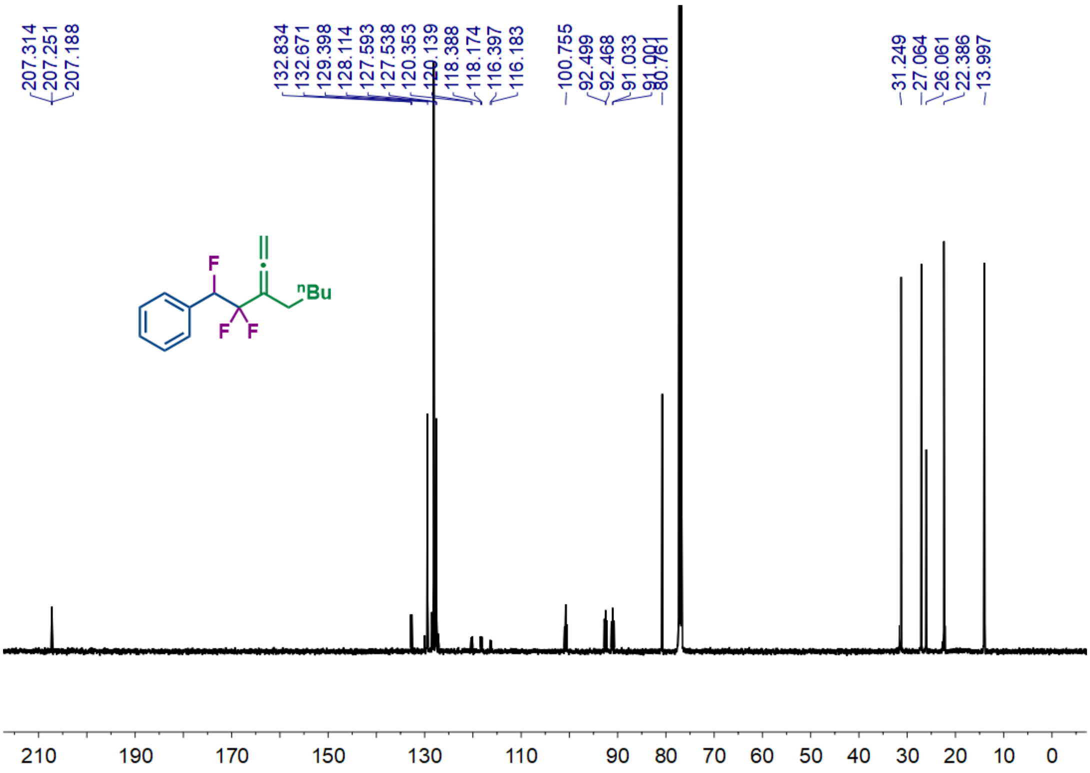

Supplementary Fig. 619 ^{13}C NMR (125 MHz, $CDCl_3$) spectrum of compound **206**

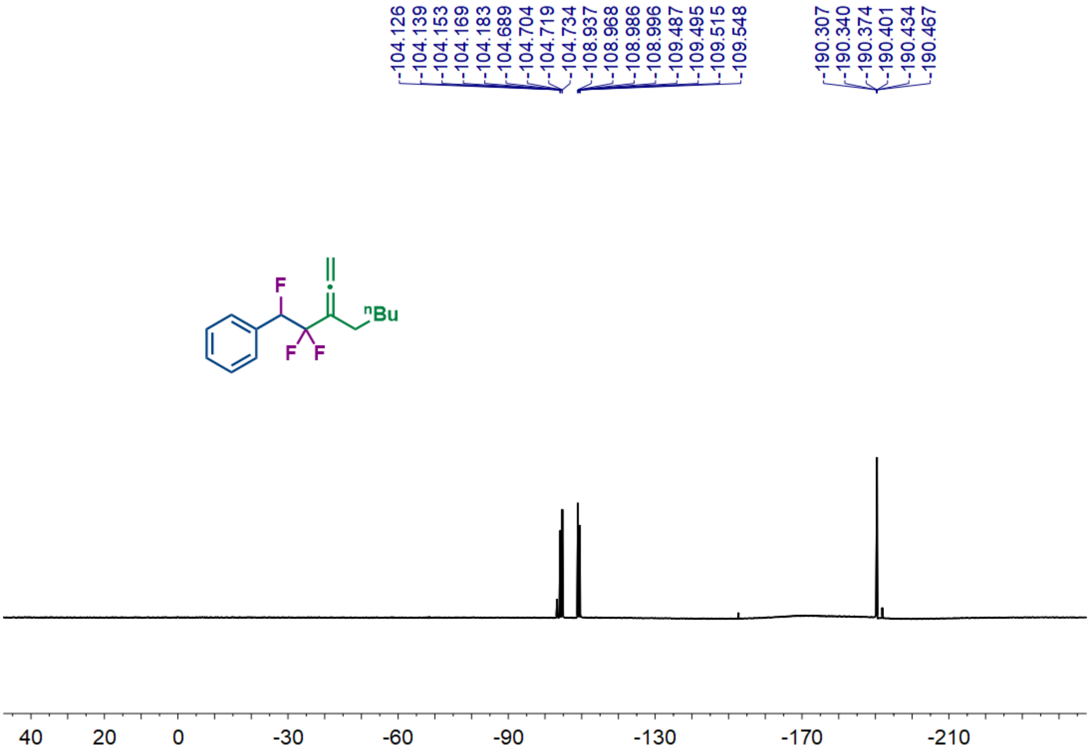

Supplementary Fig. 620 ^{19}F NMR (470 MHz, $CDCl_3$) spectrum of compound **206**

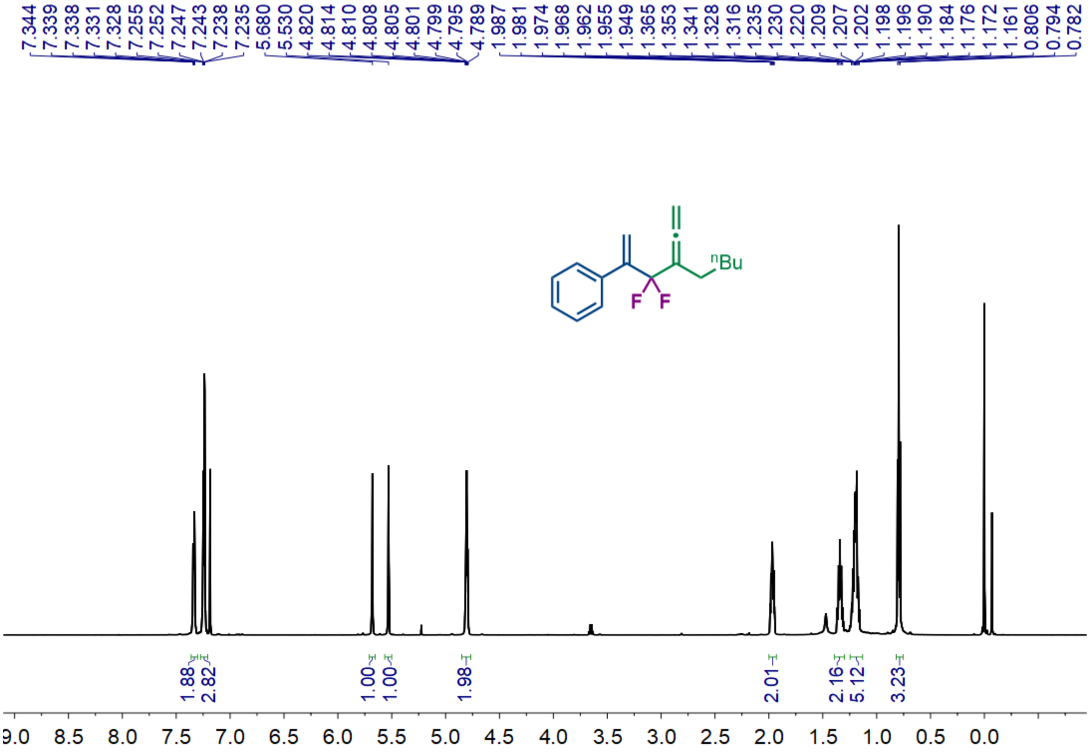

Supplementary Fig. 621 ^{1}H NMR (600 MHz, $CDCl_3$) spectrum of compound **207**

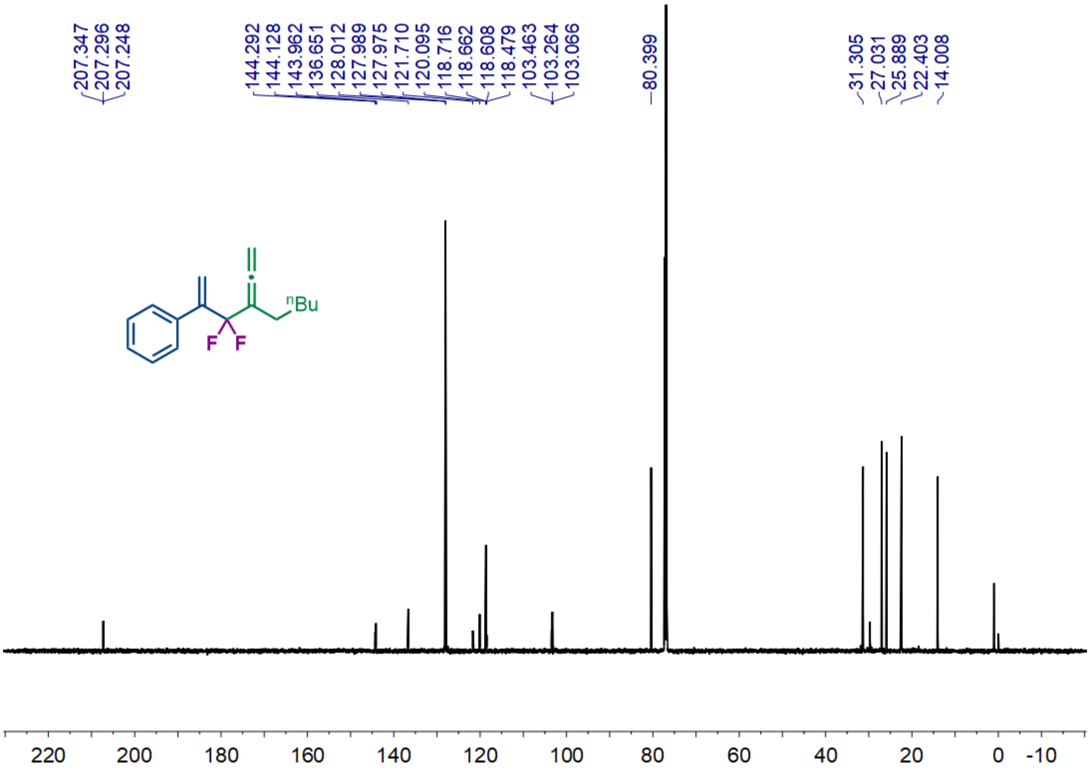

Supplementary Fig. 622 ^{13}C NMR (150 MHz, $CDCl_3$) spectrum of compound **207**

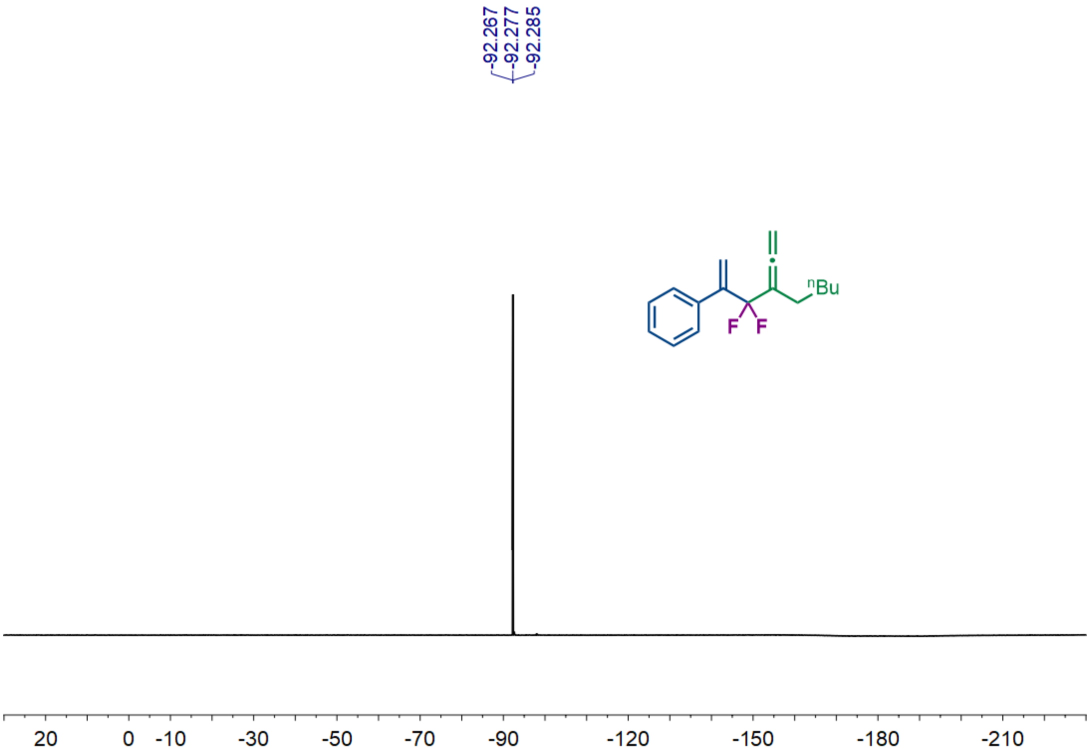

Supplementary Fig. 623 ^{19}F NMR (564 MHz, $CDCl_3$) spectrum of compound **207**

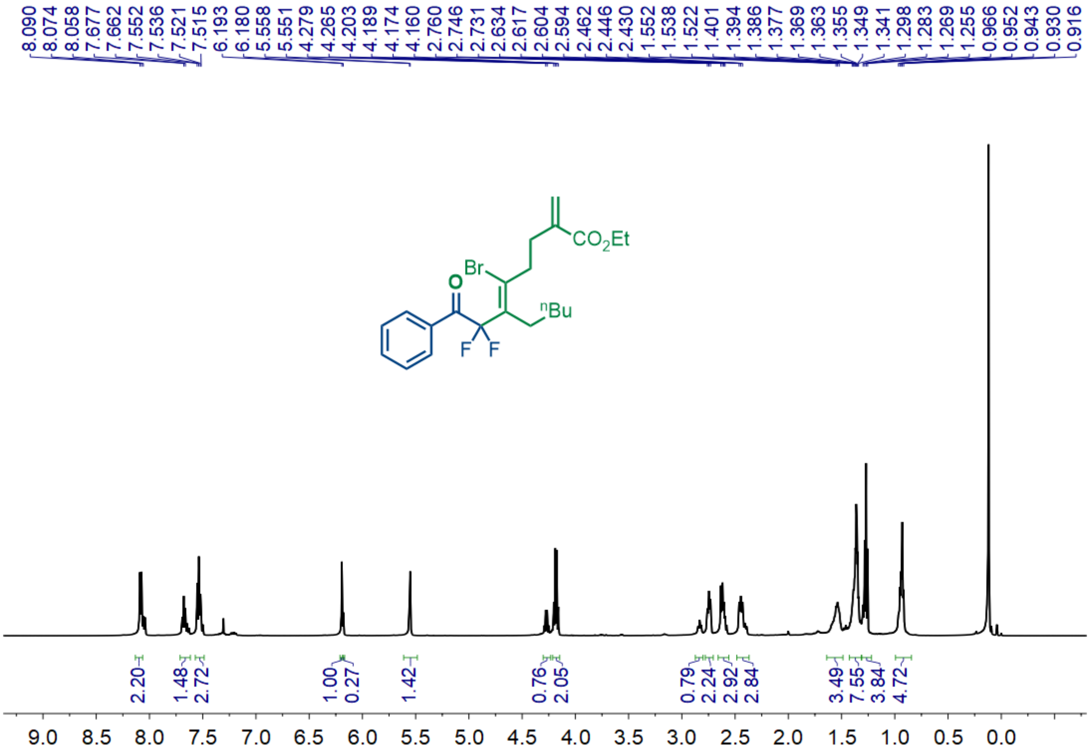

Supplementary Fig. 624 ^{1}H NMR (500 MHz, $CDCl_3$) spectrum of compound **208**

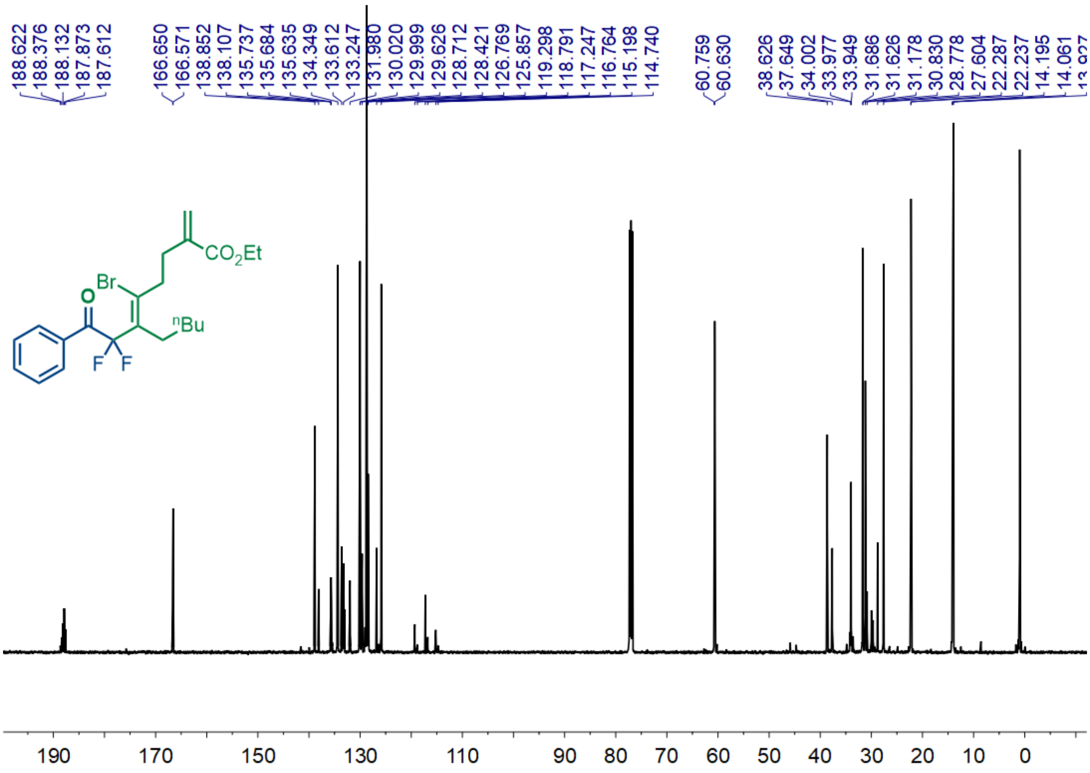

Supplementary Fig. 625 ^{13}C NMR (125 MHz, $CDCl_3$) spectrum of compound **208**

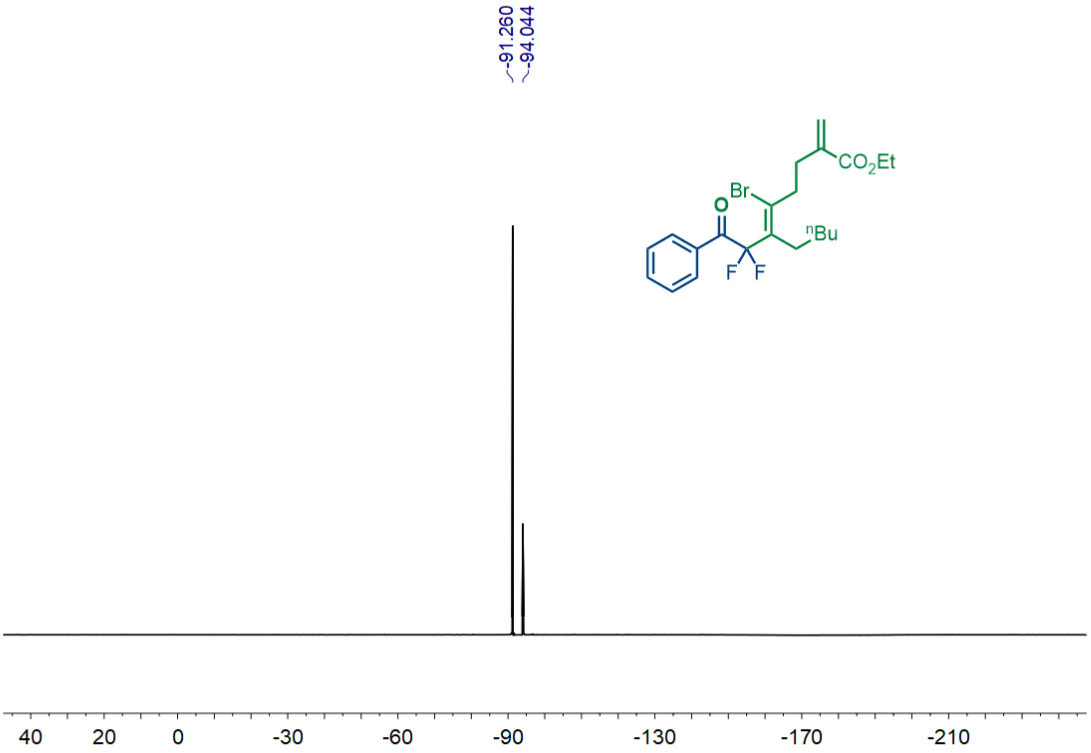

Supplementary Fig. 626 ^{19}F NMR (564 MHz, $CDCl_3$) spectrum of compound **208**

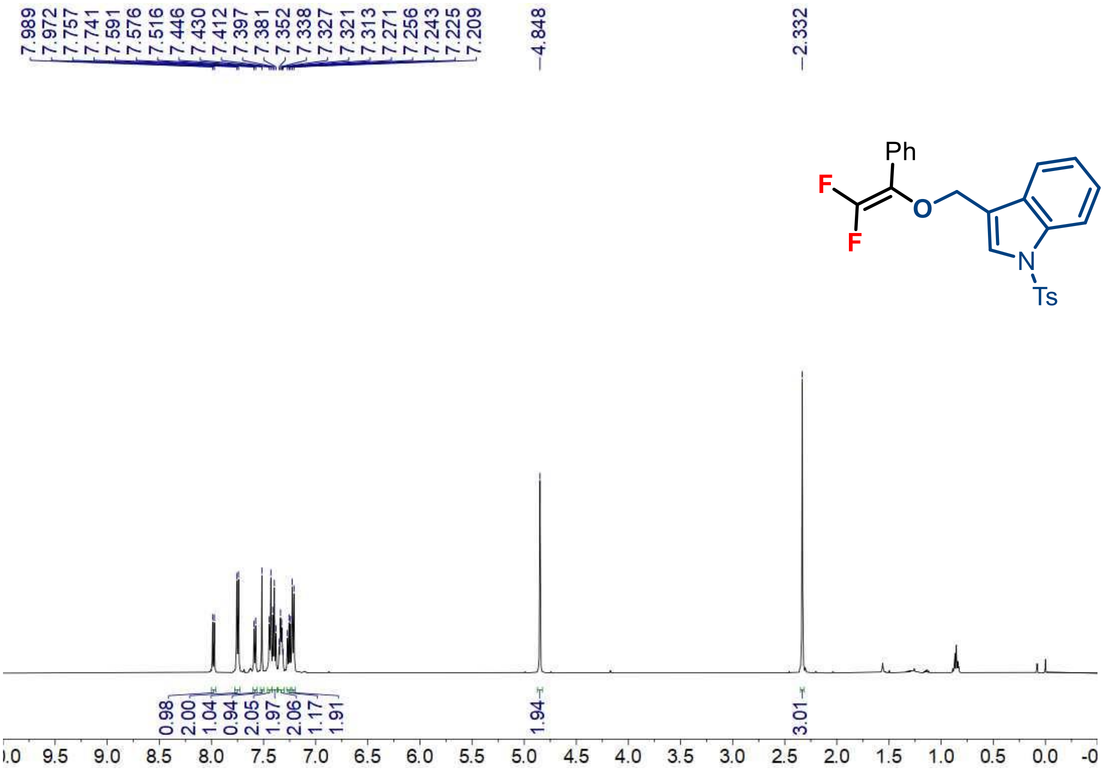

Supplementary Fig. 627 ^{1}H NMR (500 MHz, $CDCl_3$) spectrum of compound **209**

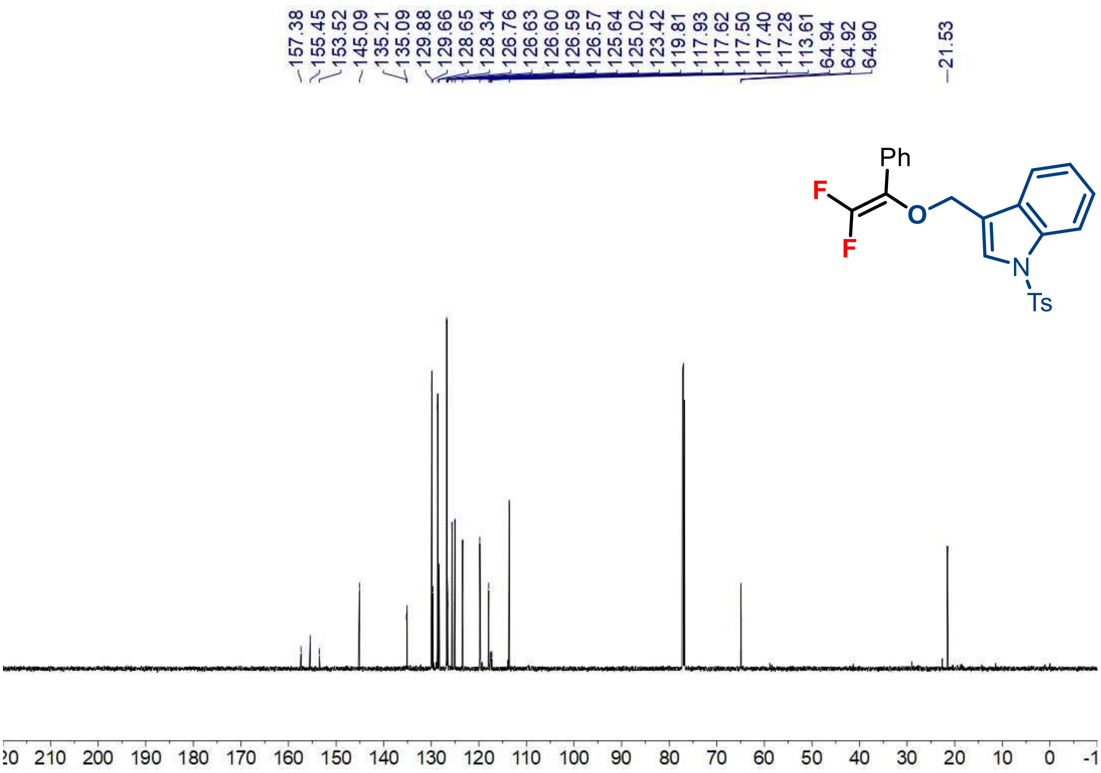

Supplementary Fig. 628 ^{13}C NMR (150 MHz, $CDCl_3$) spectrum of compound **209**

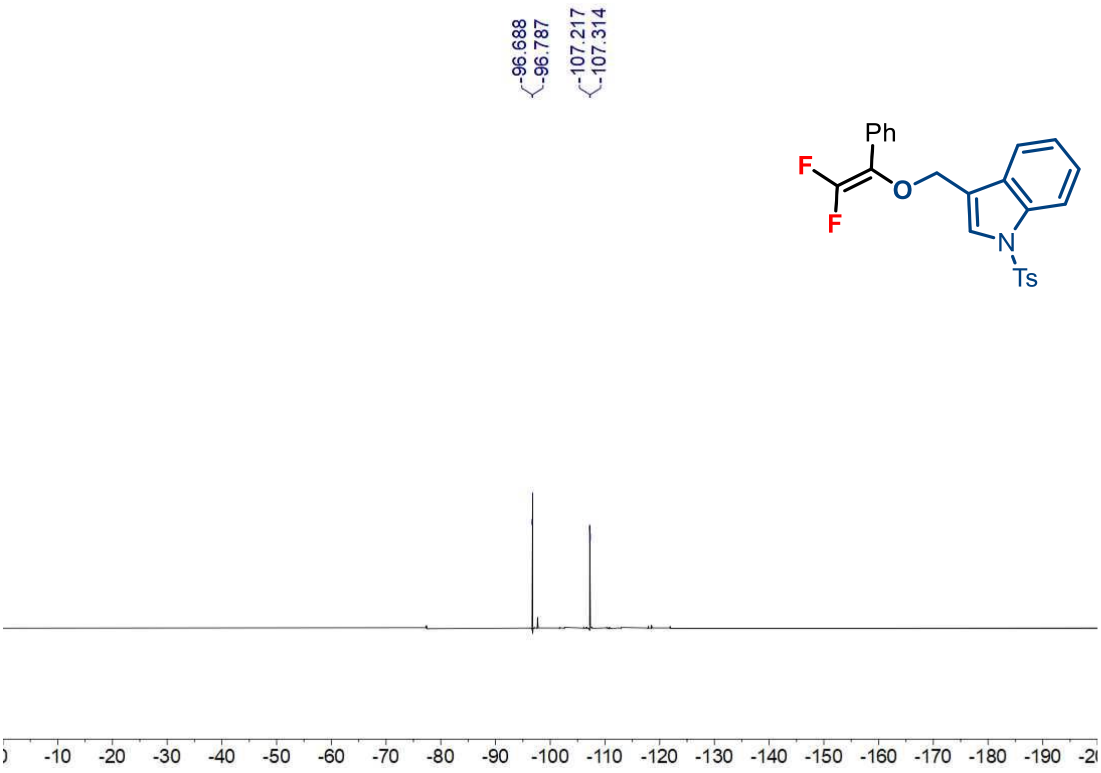

Supplementary Fig. 629 ^{19}F NMR (564 MHz, $CDCl_3$) spectrum of compound **209**

Supplementary References

1. Liu, Z. et al. Site-selective C–H benzylation of alkanes with *N*-triftosylhydrazones leading to alkyl aromatics. *Chem* **6**, 2110–2124 (2020).
2. Ning, Y. et al. Difluoroacetaldehyde *N*-triftosylhydrazone (DFHZ-Tfs) as a bench-stable crystalline diazo surrogate for diazoacetaldehyde and difluorodiazoethane. *Angew. Chem. Int. Ed*. **59**, 6473–6481 (2020).
3. Nair, V. N. et al. Catalyst-controlled regiodivergence in rearrangements of indole-based onium ylides. *J. Am. Chem. Soc.* **143**, 9016–9025 (2021).
4. Morimoto, T. & Sekiya, M. A new, general route to γ,δ-unsaturated α,α-dichloroketones from allyl 2,2,2-trichloroethyl ethers via the [3,3]sigmatropic rearrangement of intermediary 2,2-dichlorovinyl ethers. *Synthesis* **1981**, 308–310 (1981).
5. Frisch, M. J. et al. *Gaussian 16, Revision C.01*; Gaussian, Inc.: Wallingford, CT, 2019.
6. Becke, A. D. Density-functional thermochemistry. III. The role of exact exchange. *J. Chem. Phys.* **98**, 5648–5652 (1993).
7. Perdew, J. P. & Wang, Y. Accurate and simple analytic representation of the electron-gas correlation energy. *Phys. Rev. B* **45**, 13244 (1992).
8. Dolg, M., Wedig, U., Stoll, H. & Preuss, H. Energy-adjusted abinitio pseudopotentials for the first row transition elements. *J. Chem. Phys.* **86**, 866–872 (1987).
9. Andzelm, J. & Huzinaga, S. Gaussian Basis Sets for Molecular Calculations, Elsevier Science, New York, 1984.
10. Hehre, W. J., Ditchfield, R. & Pople, J. A. Self-consistent molecular orbital methods. XII. Further extensions of Gaussian-type basis sets for use in molecular orbital studies of organic molecules. *J. Chem. Phys*. **56**, 2257–2261 (1972).
11. Dill, J. D. & Pople, J. A. Self-consistent molecular orbital methods. XV. Extended Gaussian-type basis sets for lithium, beryllium, and boron. *J. Chem. Phys*. **62**, 2921–2923 (1975).
12. Weigend, F. & Ahlrichs, R. Balanced basis sets of split valence, triple zeta valence and quadruple zeta valence quality for H to Rn: Design and assessment of accuracy. *Phys. Chem. Chem. Phys.* **7***,* 3297–3305 (2005).
13. Grimme, S., Ehrlich, S. & Goerigk, L. Effect of the damping function in dispersion corrected density functional theory. *J. Comp. Chem.* **32**, 1456–1465 (2011).
14. Marenich, A. V., Cramer, C. J. & Truhlar, D. G. Universal solvation model based on solute electron density and on a continuum model of the solvent defined by the bulk dielectric constant and atomic surface tensions. *J. Phys. Chem. B* **113**, 6378–6396 (2009).
15. Fukui, K. Formulation of the reaction coordinate. *J. Phys. Chem.* **74**, 4161–4163 (1970).
16. Fukui, K. The path of chemical reactions - the IRC approach. *Acc. Chem. Res.* **14**, 363–368 (1981).
17. Legault, C. Y. CYLview, version 1.0b; Université de Sherbrooke, (2009) (http://www.cylview.org).
