# Supplementary material for: Carbodefluorination of fluoroalkyl ketones via a carbene-initiated rearrangement strategy
Source: Nat Commun. 2022 Jul 25;13:4280. doi: 10.1038/s41467-022-31976-z (PMC9314321; doi:10.1038/s41467-022-31976-z)
Supplement: Supplementary file 3 — Description of Additional Supplementary Files [file 41467_2022_31976_MOESM3_ESM.pdf]

Supplementary Data 1: Cartesian coordinates and energies of the computed structures
