# Supplementary material for: Carbodefluorination of fluoroalkyl ketones via a carbene-initiated rearrangement strategy
Source: Nat Commun. 2022 Jul 25;13:4280. doi: 10.1038/s41467-022-31976-z (PMC9314321; doi:10.1038/s41467-022-31976-z)
Supplement: Supplementary file 4 — Supplementary Data 1 [file 41467_2022_31976_MOESM4_ESM.pdf]

Cartesian coordinates and energies of the computed structures

carbene

Zero-point correction= 0.216204 (Hartree/Particle)

Thermal correction to Energy= 0.255384

Thermal correction to Enthalpy= 0.256328

Thermal correction to Gibbs Free Energy= 0.133808

Sum of electronic and zero-point Energies= -24619.430114

Sum of electronic and thermal Energies= -24619.390934

Sum of electronic and thermal Enthalpies= -24619.389990

Sum of electronic and thermal Free Energies= -24619.512510

C	0.55029800	-1.35706200	2.78937100
C	1.90861400	-1.34507200	3.13714700
C	2.51324900	-0.69217700	2.07454900
N	1.56523200	-0.34654100	1.16829600
N	0.34289200	-0.76201200	1.62345500
Br	4.37494300	-0.31285000	1.88113900
Br	2.73421900	-2.05903400	4.69997100
Br	-0.89225000	-2.12578700	3.77858500
C	-0.70374500	3.01244100	-0.22616800
C	0.40557400	3.80375200	-0.55125700
C	1.45390100	2.89574600	-0.59681200
N	0.98042100	1.65718400	-0.31646500
N	-0.36348900	1.73817000	-0.08822100
Br	3.28086400	3.28548600	-0.98785700
Br	0.46621500	5.68386100	-0.85753700
Br	-2.51211900	3.58552800	0.01755900
C	-0.12827900	-2.05360200	-2.28999500

C	1.09255900	-2.19850600	-2.96536200
C	1.92788800	-1.30490400	-2.31477100
N	1.22971900	-0.68373300	-1.33046800
N	-0.05597800	-1.15288400	-1.32396200
Br	3.76568100	-0.96612800	-2.71242000
Br	1.51017500	-3.34833100	-4.42812500
Br	-1.74954900	-3.02099800	-2.62311000
B	1.73764700	0.29876700	-0.23768900
Ag	-1.64439500	-0.13608800	0.45913800
C	-4.70979500	-0.47348300	-0.27805500
C	-4.30314400	-0.44253500	-1.64499000
C	-5.22016700	-0.59841100	-2.67201900
C	-6.56479300	-0.83226100	-2.36436500
C	-6.99664600	-0.89512100	-1.03079000
C	-6.09505800	-0.70384500	0.00014400
H	-3.25451500	-0.27248500	-1.86082000
H	-4.89506000	-0.55203100	-3.70601300
H	-7.28412900	-0.96936600	-3.16684100
H	-8.04172700	-1.08574900	-0.80945600
H	-6.44023400	-0.75060900	1.02442200
C	-3.70658000	-0.26371700	0.70936500
H	2.88588000	0.50556600	-0.41889100
C	-4.20082000	-0.04070000	2.12925100
F	-3.22855700	0.39585900	2.94823200
F	-5.19843800	0.87487600	2.20365100
F	-4.66612200	-1.20948100	2.63694600

Int1

Zero-point correction= 0.496297 (Hartree/Particle)

Thermal correction to Energy= 0.554790

Thermal correction to Enthalpy= 0.555734

Thermal correction to Gibbs Free Energy= 0.390414

Sum of electronic and zero-point Energies= -25916.972541

Sum of electronic and thermal Energies= -25916.914049

Sum of electronic and thermal Enthalpies= -25916.913104

Sum of electronic and thermal Free Energies= -25917.078424

C	-0.60935600	-1.33939900	-2.76803100
C	-1.04808900	-2.67149000	-2.80974000
C	-1.68637800	-2.84223500	-1.59142700
N	-1.61839200	-1.68364200	-0.88896800
N	-0.94339200	-0.74956900	-1.62921300
Br	-2.53549100	-4.43564000	-0.96866800
Br	-0.83153400	-3.94477500	-4.21088800
Br	0.34749300	-0.40219100	-4.12776100
C	-3.84652800	1.85446500	-0.01855400
C	-5.00950700	1.14468800	0.31367100
C	-4.54442300	-0.13454000	0.57481200
N	-3.19826500	-0.15762100	0.40663400
N	-2.76901900	1.08696400	0.03739600
Br	-5.59311500	-1.64408500	1.08953000
Br	-6.80348700	1.78119000	0.39456300
Br	-3.72834500	3.69895500	-0.51557500
C	0.75899500	-0.12756900	2.25528400
C	0.35691600	-1.03899700	3.24201100
C	-0.80698700	-1.59108100	2.72804300
N	-1.06397200	-1.03659700	1.51777000
N	-0.08655800	-0.12367500	1.23187600
Br	-1.90600900	-2.91101100	3.56184500

Br	1.20790400	-1.43055900	4.90142100
Br	2.30496200	0.98786900	2.25246400
B	-2.21012300	-1.35183800	0.51148000
Ag	-0.34306600	1.39746700	-0.57039200
C	0.07932300	4.46310800	-0.20624600
C	-0.12900600	4.21352500	1.18131900
C	-0.40458000	5.24476800	2.06603100
C	-0.52576200	6.55281000	1.58479800
C	-0.35092900	6.83113200	0.22131900
C	-0.03058200	5.81294700	-0.66240800
H	-0.03961700	3.19260900	1.53513200
H	-0.54126400	5.03505600	3.12171300
H	-0.76205900	7.36069800	2.27150600
H	-0.46196300	7.84780600	-0.14209900
H	0.09786000	6.04221700	-1.71164100
C	0.33283100	3.33107600	-1.04101500
H	-2.80115900	-2.29685900	0.90433900
C	0.76351600	3.57375700	-2.47337000
F	1.47087100	2.54531100	-2.96727900
F	-0.36107100	3.71021500	-3.21537500
F	1.52534200	4.68656200	-2.70369600
O	3.12563800	3.75771900	-0.56339100
H	2.88280900	4.65755700	-0.81744900
C	6.77512500	1.10038200	2.77787200
C	6.15978300	2.36613600	2.75667500
C	5.50923300	2.82487900	1.61597600
C	5.47002900	1.99974500	0.48075600
C	6.09069500	0.72589500	0.52730200
C	6.75528100	0.26253900	1.66557400
H	7.28542100	0.77043900	3.67775000

H	6.20133000	2.99366300	3.64212500
H	5.03044100	3.79861600	1.59674000
H	7.25553400	-0.69757900	1.67265100
C	4.89939300	2.16010900	-0.83950400
C	5.16302600	1.01547600	-1.53273600
H	4.93326400	0.74323600	-2.55179500
N	5.90565600	0.13853400	-0.73671700
C	4.24379700	3.37999100	-1.38992900
H	4.96451400	4.21183800	-1.41103000
H	3.91545800	3.19631200	-2.42021400
S	6.22969700	-1.48509600	-1.19735000
O	6.12885400	-1.47591700	-2.65458900
O	7.45186600	-1.86481900	-0.49382600
C	3.95729500	-3.82664500	1.19706000
C	2.74725100	-3.93186600	0.49563700
C	2.62961300	-3.27566700	-0.73970400
C	3.68140500	-2.52663600	-1.26155300
C	4.87007400	-2.43173500	-0.53286000
C	5.02096400	-3.08077700	0.69429000
H	4.07243500	-4.34337100	2.14588000
H	1.70674300	-3.36071900	-1.30656600
H	3.59128900	-2.04433700	-2.22865900
H	5.96210300	-3.02152600	1.22841700
C	1.59485600	-4.72266400	1.06407700
H	1.94663700	-5.57126500	1.65796000
H	0.98324100	-4.09539500	1.72376800
H	0.94013100	-5.10275100	0.27505400

TS1

Zero-point correction= 0.496436 (Hartree/Particle)

Thermal correction to Energy= 0.552938

Thermal correction to Enthalpy= 0.553882

Thermal correction to Gibbs Free Energy= 0.396312

Sum of electronic and zero-point Energies= -25916.974327

Sum of electronic and thermal Energies= -25916.917825

Sum of electronic and thermal Enthalpies= -25916.916881

Sum of electronic and thermal Free Energies= -25917.074451

C	-0.06097500	-1.02966100	-2.88546600
C	-0.04979100	-2.42899900	-2.96505200
C	-0.68170800	-2.82363200	-1.79531300
N	-1.03432000	-1.72440800	-1.08491900
N	-0.64018200	-0.60615600	-1.76928000
Br	-1.01466100	-4.61908700	-1.23634200
Br	0.64897500	-3.52904800	-4.35284000
Br	0.62740900	0.19901400	-4.17608000
C	-4.00488100	1.24355700	0.01517900
C	-5.02215800	0.27807800	0.01609000
C	-4.33146400	-0.92096800	0.09861800
N	-3.00181400	-0.66130600	0.14647400
N	-2.80281800	0.69069400	0.09145600
Br	-5.08355400	-2.67517500	0.13001700
Br	-6.90744100	0.54262700	-0.06293700
Br	-4.21627600	3.14222600	-0.06765100
C	0.81170700	-0.10874000	2.29576600
C	0.29112600	-0.93198500	3.30567200
C	-0.75766800	-1.58873300	2.68080200
N	-0.83445000	-1.16997900	1.39439200
N	0.13959400	-0.23880500	1.16047300

Br	-1.94004700	-2.87230700	3.45719900
Br	0.88326400	-1.11620600	5.10984000
Br	2.32900000	1.04936600	2.41073600
B	-1.80166600	-1.63737800	0.26833800
Ag	-0.57035000	1.49952400	-0.69347200
C	-0.35254200	4.43624200	0.45521000
C	-0.33989400	3.79551300	1.71809900
C	-0.55464800	4.50579800	2.89233200
C	-0.80737200	5.87916800	2.83476700
C	-0.83424300	6.53855800	1.60061500
C	-0.59727800	5.83582700	0.42586500
H	-0.15808100	2.72803900	1.75220800
H	-0.53732000	3.99089700	3.84747300
H	-0.98900900	6.43631300	3.74935800
H	-1.03980100	7.60372000	1.55855400
H	-0.62582400	6.36673700	-0.51651500
C	-0.15335600	3.60179500	-0.72060500
H	-2.21265400	-2.71489500	0.53692000
C	-0.34985500	4.26924700	-2.07020400
F	-0.00340600	3.46963400	-3.09892000
F	-1.65616500	4.57099800	-2.21066000
F	0.35464500	5.43258600	-2.26888000
O	1.95892500	3.83525000	-0.80749800
H	1.97705700	4.79670800	-0.92174300
C	6.89376700	2.31256400	1.58285400
C	6.14615700	3.50041000	1.47889600
C	5.09202600	3.60294500	0.57846000
C	4.77757000	2.49840400	-0.22870300
C	5.53926200	1.30880700	-0.10002500
C	6.60633500	1.20099400	0.79536100

H	7.71881100	2.26145100	2.28680800
H	6.40109600	4.34782100	2.10821100
H	4.51485900	4.51963100	0.50535100
H	7.20211400	0.29922500	0.85500400
C	3.77705600	2.26350400	-1.24706200
C	3.93962100	0.97863900	-1.68457500
H	3.41526900	0.43098200	-2.45316200
N	5.00810000	0.38609300	-1.01715200
C	2.82918000	3.24464100	-1.83455000
H	3.36248500	4.07629800	-2.31085600
H	2.19529900	2.77058000	-2.58547800
S	5.48800900	-1.25053300	-1.27861700
O	4.88235800	-1.59975400	-2.56058600
O	6.92968200	-1.28214600	-1.04983400
C	4.77035300	-3.26710600	2.15400900
C	3.44616700	-3.70324200	2.00437400
C	2.75893700	-3.36605700	0.82728800
C	3.36591900	-2.61036800	-0.17185400
C	4.68413200	-2.18328500	0.01192600
C	5.39726400	-2.50951500	1.16695700
H	5.32344900	-3.53358200	3.05032800
H	1.73888300	-3.71120500	0.68504100
H	2.83580200	-2.37717000	-1.08853400
H	6.43037100	-2.20004700	1.27445700
C	2.76985900	-4.50723800	3.08702200
H	3.49697300	-5.06031400	3.68811000
H	2.21308300	-3.85021200	3.76589300
H	2.05656100	-5.22220200	2.66681300

Int2

Zero-point correction= 0.497835 (Hartree/Particle)

Thermal correction to Energy= 0.555363

Thermal correction to Enthalpy= 0.556307

Thermal correction to Gibbs Free Energy= 0.395749

Sum of electronic and zero-point Energies= -25916.971301

Sum of electronic and thermal Energies= -25916.913774

Sum of electronic and thermal Enthalpies= -25916.912830

Sum of electronic and thermal Free Energies= -25917.073387

C	1.39696700	1.08807900	2.98748900
C	1.97787500	0.21381600	3.91574500
C	2.63335300	-0.71316700	3.12045100
N	2.44380400	-0.39290800	1.81757000
N	1.66526200	0.73016000	1.73928900
Br	3.64129000	-2.22057500	3.72028800
Br	1.90051400	0.28369800	5.81956100
Br	0.35384700	2.64551000	3.37339100
C	3.68973900	1.58991000	-1.82939800
C	5.02883900	1.23161900	-1.62012100
C	4.94995700	0.19664700	-0.70111900
N	3.64778200	-0.03138900	-0.40241400
N	2.86645600	0.84290300	-1.10704300
Br	6.41322900	-0.76774100	0.05976900
Br	6.59272500	1.98014700	-2.41438600
Br	3.02206400	2.95979800	-2.98358300
C	-0.07957500	-2.37383800	-0.85127200
C	0.70920700	-3.48363300	-1.18617100
C	1.96866300	-3.12726800	-0.72960400
N	1.90249000	-1.89415600	-0.17034200

N	0.62165800	-1.42306100	-0.25496400
Br	3.57053000	-4.16076900	-0.85510800
Br	0.18372000	-5.10486700	-2.04388100
Br	-1.96607500	-2.16969700	-1.14894300
B	3.02137000	-1.08657500	0.55082500
Ag	0.61488900	1.30326900	-0.36198300
C	-1.27116400	2.10702800	-2.79328400
C	-0.77109400	0.91695300	-3.35046000
C	-0.94251900	0.62159600	-4.70080500
C	-1.61327000	1.51918700	-5.53297000
C	-2.11451300	2.70758600	-5.00198300
C	-1.95445600	2.99692300	-3.64725300
H	-0.23308900	0.22485900	-2.71149200
H	-0.54078700	-0.30344600	-5.10327800
H	-1.73867200	1.29697800	-6.58871200
H	-2.63263000	3.41543800	-5.64242800
H	-2.35236800	3.92985800	-3.26627100
C	-1.08000200	2.35193500	-1.31611700
H	3.87227000	-1.83658100	0.88729000
C	-1.10132100	3.81639600	-0.92228100
F	-0.80650800	4.02159000	0.38491000
F	-0.23333100	4.52764600	-1.64954300
F	-2.34952700	4.40889400	-1.07811500
H	-3.19439400	2.37086800	-1.01232800
C	-7.61423300	3.46567700	1.30590300
C	-6.47002400	4.20938600	0.96600800
C	-5.22613600	3.59926600	0.86348700
C	-5.13265400	2.21724700	1.09601100
C	-6.29956000	1.48466200	1.43074900
C	-7.55027200	2.09696200	1.54891300

H	-8.57122000	3.97098700	1.39178300
H	-6.56012600	5.27684300	0.79041400
H	-4.34586200	4.18225400	0.60641100
H	-8.42662400	1.53391500	1.84301800
C	-4.02768200	1.27889800	1.07890800
C	-4.53691500	0.04583100	1.39023200
H	-4.03671300	-0.90388400	1.50881900
N	-5.89883400	0.14832200	1.62008700
C	-2.61214900	1.58290000	0.79666100
H	-2.24963400	2.48689300	1.28269100
H	-1.93607700	0.75355700	0.99427700
O	-2.45517000	1.81185700	-0.69851500
S	-6.90145000	-1.24889300	1.86560000
O	-6.00801300	-2.23348300	2.46591900
O	-8.10131600	-0.75239300	2.52930400
C	-8.71347100	-1.63244300	-1.73306200
C	-7.93958600	-2.60574200	-2.38245400
C	-6.84821600	-3.16051300	-1.69634900
C	-6.52802000	-2.75711300	-0.40289700
C	-7.31378300	-1.77865800	0.21218200
C	-8.41321200	-1.21532900	-0.43916800
H	-9.56743700	-1.19845000	-2.24540800
H	-6.24515400	-3.92553500	-2.17748500
H	-5.69768400	-3.20611700	0.13091700
H	-9.02823000	-0.47609100	0.06114200
C	-8.29382600	-3.07403100	-3.77102700
H	-8.82493600	-2.30136800	-4.33388700
H	-7.40323200	-3.35817500	-4.33901700
H	-8.94704900	-3.95466300	-3.72964900

TS2

Zero-point correction= 0.494385 (Hartree/Particle)

Thermal correction to Energy= 0.552290

Thermal correction to Enthalpy= 0.553234

Thermal correction to Gibbs Free Energy= 0.390003

Sum of electronic and zero-point Energies= -25916.950181

Sum of electronic and thermal Energies= -25916.892276

Sum of electronic and thermal Enthalpies= -25916.891332

Sum of electronic and thermal Free Energies= -25917.054562

C	-1.40159200	-3.11068400	-0.47792300
C	-2.03021100	-3.86105400	0.52511500
C	-2.70788500	-2.90615300	1.26686900
N	-2.48485000	-1.68349000	0.72562200
N	-1.66221100	-1.81629000	-0.36134400
Br	-3.78193400	-3.21622900	2.81556400
Br	-1.98069200	-5.74692500	0.80215400
Br	-0.31559600	-3.78392200	-1.90461500
C	-3.71714200	1.40290000	-2.02093000
C	-5.06195500	1.25819000	-1.65357700
C	-4.99739100	0.61195500	-0.42852700
N	-3.69748600	0.40084000	-0.10720500
N	-2.90393200	0.89656400	-1.10518000
Br	-6.47494700	0.08069600	0.65911300
Br	-6.61585000	1.82078000	-2.60503600
Br	-3.02936900	2.20122500	-3.61770100
C	-0.07678300	1.46763900	2.15763200
C	-0.87053700	1.86769600	3.24145100
C	-2.09691300	1.27660600	2.98014100

N	-2.01401500	0.58002300	1.82037300
N	-0.75137600	0.70672300	1.30699700
Br	-3.67758700	1.40646500	4.04468100
Br	-0.38401200	2.95356900	4.73219600
Br	1.76800900	1.89510000	1.86446400
B	-3.09208900	-0.31042700	1.13662200
Ag	-0.57647500	0.24408000	-1.16142100
C	1.38171800	2.45279700	-2.41395200
C	0.56090700	3.28570000	-1.64032700
C	0.72615100	4.67188100	-1.65612900
C	1.72392300	5.24775600	-2.44223200
C	2.55030800	4.43093300	-3.21780100
C	2.38059500	3.04824900	-3.20847500
H	-0.21649200	2.84950900	-1.02119500
H	0.07563800	5.29627100	-1.05088300
H	1.85548200	6.32589500	-2.45401900
H	3.32495200	4.87110700	-3.83905700
H	3.01386000	2.42724300	-3.83197900
C	1.21068600	0.94971800	-2.39020000
H	-3.96149100	-0.50406300	1.91345100
C	0.95949900	0.34711600	-3.67156500
F	0.77039200	-0.94527200	-3.81232300
F	0.35217100	1.00496800	-4.60938700
F	2.74530400	0.22886200	-4.26980000
H	2.92902900	0.23197900	-3.03144600
C	7.39867700	-2.61319700	-2.92549100
C	6.27252400	-2.36003200	-3.72940900
C	5.08079600	-1.91218600	-3.17177300
C	5.02040700	-1.70884100	-1.78319800
C	6.16979900	-1.96206800	-0.99217500

C	7.36755100	-2.42169300	-1.54696100
H	8.31388200	-2.97208700	-3.38673800
H	6.33524400	-2.52001100	-4.80154800
H	4.22322700	-1.70101800	-3.80325500
H	8.22833400	-2.64101500	-0.92824100
C	3.96964800	-1.26854000	-0.88608100
C	4.49143200	-1.26107300	0.37778400
H	4.02279100	-1.03025700	1.32281300
N	5.81205300	-1.69244300	0.34346300
C	2.56386100	-0.94764000	-1.22624600
H	2.10191700	-1.70461100	-1.86220700
H	1.94891500	-0.78507500	-0.34152500
O	2.52608900	0.34396400	-1.98198700
S	6.85161500	-1.58613400	1.72009000
O	5.95462200	-1.70453500	2.86724600
O	7.93003400	-2.53923000	1.47268700
C	9.11875100	1.65081800	0.84878700
C	8.48989800	2.68563700	1.55820900
C	7.36029500	2.37704100	2.33189100
C	6.85677500	1.08002400	2.38892100
C	7.49878600	0.07482500	1.66022400
C	8.63455900	0.34611800	0.89283900
H	10.00490600	1.86789000	0.25904500
H	6.87311900	3.16072900	2.90536700
H	5.99688900	0.84546700	3.00673800
H	9.13928700	-0.44772200	0.35419700
C	9.00573300	4.09969700	1.47818400
H	10.08468700	4.12387200	1.30026900
H	8.52606500	4.63986200	0.65203300
H	8.79477200	4.65573200	2.39619700

TS2''

Zero-point correction= 0.492651 (Hartree/Particle)

Thermal correction to Energy= 0.550850

Thermal correction to Enthalpy= 0.551795

Thermal correction to Gibbs Free Energy= 0.389560

Sum of electronic and zero-point Energies= -25916.937225

Sum of electronic and thermal Energies= -25916.879026

Sum of electronic and thermal Enthalpies= -25916.878082

Sum of electronic and thermal Free Energies= -25917.040317

C	1.51571900	-0.58644900	3.16610200
C	2.29933200	-1.68908300	3.53419900
C	2.93101300	-2.04766500	2.35479700
N	2.53832700	-1.20453100	1.36739000
N	1.65292900	-0.29334700	1.88080800
Br	4.15918500	-3.49231200	2.11885500
Br	2.46404800	-2.50464100	5.25012900
Br	0.36247000	0.42708900	4.30604000
C	3.65316900	2.29352000	-0.91231000
C	4.94211000	1.80710800	-1.16939600
C	4.83722600	0.44935700	-0.91443000
N	3.56627200	0.16636200	-0.53561200
N	2.83181200	1.32242100	-0.53526000
Br	6.23769800	-0.84186500	-1.05767500
Br	6.48072300	2.77893900	-1.73893800
Br	3.06252400	4.10410200	-1.05810100
C	-0.31359900	-1.55085300	-1.70867800
C	0.30151000	-2.61026700	-2.38923400

C	1.60635800	-2.58438200	-1.92090500
N	1.73579600	-1.57250700	-1.02766700
N	0.53556400	-0.92818400	-0.90427600
Br	3.02317200	-3.75810000	-2.43321700
Br	-0.46891200	-3.81119500	-3.65551200
Br	-2.14592800	-1.00920900	-1.81401800
B	2.95557600	-1.20465800	-0.13049400
Ag	0.56242700	1.23254400	0.27395700
C	-1.05568500	3.67381200	-1.11057100
C	-0.86693900	2.91932100	-2.28440000
C	-0.92405100	3.51660000	-3.54050800
C	-1.16013000	4.88842900	-3.64895100
C	-1.34749800	5.65325800	-2.49506400
C	-1.30741500	5.05566300	-1.23769200
H	-0.66472500	1.85580800	-2.20672200
H	-0.77353600	2.91370100	-4.43077100
H	-1.19568800	5.35991500	-4.62661900
H	-1.53222500	6.72046000	-2.57315300
H	-1.46867800	5.66962000	-0.36096700
C	-1.00729000	2.98538600	0.20025200
H	3.78338900	-2.03212600	-0.28415600
C	-0.75728200	3.82485300	1.43524600
F	-0.68877700	3.07608700	2.55796700
F	0.41769400	4.47206800	1.30952500
F	-1.71382600	4.76131700	1.66772200
H	-1.51415500	1.78078700	0.29265500
C	-8.24662400	2.96065000	-0.15796900
C	-7.17473100	3.87173800	-0.19027600
C	-5.92147800	3.51656400	0.29407600
C	-5.73893500	2.22602200	0.81373100

C	-6.82971900	1.32150400	0.83100200
C	-8.09529400	1.67489700	0.35447000
H	-9.21828300	3.26648900	-0.53438300
H	-7.33226500	4.86619800	-0.59719000
H	-5.09374400	4.21887000	0.26458500
H	-8.93049900	0.98776100	0.40311800
C	-4.60179400	1.54745400	1.39961800
C	-5.00900000	0.28501000	1.72989300
H	-4.47157300	-0.51977300	2.20916800
N	-6.35742900	0.12938000	1.41366900
C	-3.25827800	2.12606000	1.66159900
H	-3.33311100	3.10368800	2.14070200
H	-2.65750700	1.46138800	2.28751800
O	-2.60410100	2.30981100	0.35262300
S	-7.17711800	-1.38152300	1.53354700
O	-6.40715300	-2.13910900	2.51831300
O	-8.58664300	-1.05363000	1.73418800
C	-7.75592100	-2.51256400	-2.30936300
C	-6.65086200	-3.33308600	-2.57543400
C	-5.71366600	-3.54807300	-1.55073800
C	-5.86518800	-2.95945700	-0.29913200
C	-6.97566700	-2.14079200	-0.06802700
C	-7.92943800	-1.91508700	-1.06220900
H	-8.49575500	-2.34255800	-3.08630100
H	-4.85780800	-4.19213800	-1.73354800
H	-5.14664700	-3.14697400	0.49101300
H	-8.79782200	-1.29827500	-0.86205800
C	-6.47797100	-3.99513100	-3.91880000
H	-5.46283300	-3.85177100	-4.30426100
H	-6.64440300	-5.07674100	-3.84469700

H	-7.18187500	-3.59939000	-4.65552500

Int3

Zero-point correction= 0.483583 (Hartree/Particle)

Thermal correction to Energy= 0.540682

Thermal correction to Enthalpy= 0.541626

Thermal correction to Gibbs Free Energy= 0.380478

Sum of electronic and zero-point Energies= -25816.510332

Sum of electronic and thermal Energies= -25816.453233

Sum of electronic and thermal Enthalpies= -25816.452289

Sum of electronic and thermal Free Energies= -25816.613436

C	-1.45166000	-1.08330500	-3.02935100
C	-2.46025100	-2.01011500	-3.32669900
C	-3.16097300	-2.12087900	-2.13616500
N	-2.59153000	-1.31085600	-1.20950500
N	-1.52605500	-0.66311700	-1.77516500
Br	-4.69092000	-3.21852300	-1.81370900
Br	-2.78972000	-2.91385500	-4.97314200
Br	-0.08580200	-0.44923400	-4.20722400
C	-3.83413500	2.45022100	0.28015800
C	-4.99273300	2.00877700	0.93392000
C	-4.80398200	0.64025000	1.04155700
N	-3.61176800	0.31219700	0.48496200
N	-3.00769300	1.44825100	0.01491400
Br	-5.99916800	-0.61367700	1.84704900
Br	-6.48379200	3.03943000	1.52701400
Br	-3.42155900	4.24612300	-0.23554900
C	0.15060700	-0.90220100	2.13064700

C	-0.30590800	-2.09167200	2.71346600
C	-1.52701300	-2.31303100	2.09526900
N	-1.76359400	-1.31298900	1.21162700
N	-0.71253300	-0.43683800	1.23785600
Br	-2.70815200	-3.78149900	2.40397500
Br	0.54380500	-3.15535400	4.04940600
Br	1.78356400	0.01778800	2.49358400
B	-2.99125400	-1.09988300	0.27839700
Ag	-0.59252200	1.20512700	-0.48501300
C	1.62868100	3.59935200	0.73741700
C	0.44375100	4.05310600	1.34438500
C	0.49196800	4.76878900	2.53707300
C	1.71849600	5.04003200	3.14956900
C	2.89877300	4.58575600	2.55965200
C	2.85864500	3.86736500	1.36542700
H	-0.51845700	3.83061600	0.89895700
H	-0.43361900	5.10403100	2.99542100
H	1.75124700	5.59492700	4.08247600
H	3.85732100	4.78920900	3.02787900
H	3.77840500	3.51575500	0.91376600
C	1.65291400	2.86447500	-0.55037300
H	-3.80789900	-1.90516500	0.55973700
C	0.64535600	2.98453400	-1.49827200
F	0.79752000	2.56157800	-2.77028700
F	-0.19046600	4.03936100	-1.48097400
C	8.45286000	2.89885800	-0.92277500
C	7.39264100	3.81953600	-1.00993200
C	6.10058600	3.39492200	-1.29848300
C	5.86509400	2.02479400	-1.49378300
C	6.94878200	1.11493800	-1.40232800

C	8.25180700	1.53555600	-1.12291700
H	9.45456400	3.25739600	-0.70532400
H	7.58993600	4.87633100	-0.85612200
H	5.28721200	4.11107200	-1.37430700
H	9.07569000	0.83415100	-1.08581600
C	4.67901800	1.25702100	-1.81052700
C	5.05570900	-0.05289000	-1.89394800
H	4.47494200	-0.93299300	-2.12741800
N	6.42691900	-0.16484200	-1.67003800
C	3.30216100	1.77393400	-2.02320600
H	3.29870000	2.68456500	-2.62805500
H	2.67180800	1.02224800	-2.50222100
O	2.74182500	2.09259200	-0.70229200
S	7.20646800	-1.68549700	-1.44707400
O	6.35976800	-2.63723300	-2.16359700
O	8.60767000	-1.47129600	-1.80118500
C	8.02805800	-1.88748100	2.51068000
C	6.88313100	-2.49046800	3.04864000
C	5.84303900	-2.85308100	2.17547500
C	5.93370000	-2.61545100	0.80822300
C	7.08609000	-2.00231700	0.30417300
C	8.14036300	-1.63914100	1.14328000
H	8.84758400	-1.61253200	3.16843800
H	4.95461200	-3.33735500	2.57189500
H	5.13426400	-2.91835700	0.14112900
H	9.03730700	-1.18815200	0.73451600
C	6.76127500	-2.75380700	4.52803000
H	6.55220300	-3.81134300	4.72459600
H	7.67616500	-2.48401300	5.06181700
H	5.93545000	-2.17772700	4.96198900

Int4

Zero-point correction= 0.484207 (Hartree/Particle)

Thermal correction to Energy= 0.541249

Thermal correction to Enthalpy= 0.542194

Thermal correction to Gibbs Free Energy= 0.380958

Sum of electronic and zero-point Energies= -25816.518251

Sum of electronic and thermal Energies= -25816.461209

Sum of electronic and thermal Enthalpies= -25816.460264

Sum of electronic and thermal Free Energies= -25816.621500

C	1.32466300	-0.61623200	3.12471400
C	2.32401900	-1.45810300	3.63017300
C	3.19217300	-1.60814700	2.55899800
N	2.72266700	-0.89731600	1.50415100
N	1.55380800	-0.28319700	1.86388400
Br	4.79484100	-2.64660900	2.52881800
Br	2.46866700	-2.21155100	5.37618700
Br	-0.20855400	0.05455000	4.05811200
C	2.73091800	2.76797900	-0.88667900
C	4.11632000	2.90282600	-0.72045500
C	4.51988200	1.63972800	-0.31561300
N	3.43659800	0.82636600	-0.25336000
N	2.32341100	1.53932500	-0.60813800
Br	6.30049700	1.09227100	0.10793600
Br	5.19692400	4.45041700	-0.99391700
Br	1.48740800	4.10715300	-1.45169200
C	0.88485900	-2.10836800	-2.18037700

C	1.99899500	-2.76779300	-2.71802200
C	3.05258900	-2.32628400	-1.93333900
N	2.57952400	-1.46723000	-0.99692700
N	1.22526500	-1.33614300	-1.15759800
Br	4.88972100	-2.81927900	-2.11301100
Br	2.05569100	-3.99096100	-4.18024300
Br	-0.92911500	-2.23315500	-2.76253400
B	3.36509500	-0.68906300	0.09852500
Ag	0.20446500	0.49729100	-0.01670300
C	-2.51881500	4.00988000	0.08386300
C	-3.36639000	4.92178100	-0.57108000
C	-3.63547600	6.16567000	-0.00292300
C	-3.07797100	6.51705500	1.22860200
C	-2.24430100	5.61370100	1.89100500
C	-1.96526700	4.37026500	1.32607800
H	-3.81754100	4.65783800	-1.52121200
H	-4.29143200	6.85866300	-0.52175000
H	-3.29336000	7.48606200	1.66911900
H	-1.80406600	5.87775000	2.84821200
H	-1.30874800	3.67570700	1.84016200
C	-2.20127900	2.68559400	-0.48743400
H	4.46690300	-1.11393400	0.13394200
C	-2.13871500	2.39793400	-1.79344600
F	-2.28739400	3.27532200	-2.77403700
F	-1.82362200	1.19987600	-2.27094200
O	-1.85594900	1.65646100	0.38359600
C	-4.38712300	-3.83474400	-0.24465500
C	-3.30585800	-3.47544300	0.58107700
C	-3.04679100	-2.14251600	0.87903900
C	-3.89333600	-1.15273300	0.35446800

C	-4.98506000	-1.53940100	-0.46314400
C	-5.24091100	-2.87546900	-0.78355900
H	-4.56028600	-4.88243200	-0.47168200
H	-2.66134500	-4.25105800	0.98382700
H	-2.20440600	-1.87404800	1.50992900
H	-6.05869600	-3.15387400	-1.43628000
C	-3.93160000	0.29098900	0.47832700
C	-5.01730900	0.72667900	-0.23105000
H	-5.39771200	1.72503900	-0.38571100
N	-5.65712800	-0.35763800	-0.82825800
C	-2.97327300	1.15422000	1.21807300
H	-3.46645000	2.02760300	1.65096000
H	-2.47185500	0.60524000	2.01509300
S	-7.19888800	-0.22403100	-1.58665200
O	-7.30396700	1.18895300	-1.94740700
O	-7.24843400	-1.28769300	-2.58669300
C	-9.79333300	-2.14383500	0.85563500
C	-10.20387800	-1.14006600	1.74468100
C	-9.68384900	0.15405700	1.58047700
C	-8.77174200	0.44224100	0.57019100
C	-8.37221900	-0.58381700	-0.29171300
C	-8.88012200	-1.87815400	-0.16256500
H	-10.19783400	-3.14697800	0.95648100
H	-10.00461400	0.94863400	2.24845500
H	-8.39249400	1.44980000	0.43996100
H	-8.58362000	-2.65575900	-0.85683600
C	-11.16798500	-1.44433100	2.86266700
H	-11.81995800	-0.59127400	3.07311100
H	-11.79598500	-2.30801500	2.62687800
H	-10.62640700	-1.67577700	3.78870900

TS3

Zero-point correction= 0.481008 (Hartree/Particle)

Thermal correction to Energy= 0.537883

Thermal correction to Enthalpy= 0.538828

Thermal correction to Gibbs Free Energy= 0.378646

Sum of electronic and zero-point Energies= -25816.494504

Sum of electronic and thermal Energies= -25816.437629

Sum of electronic and thermal Enthalpies= -25816.436684

Sum of electronic and thermal Free Energies= -25816.596866

C	-2.28098400	-0.45404400	3.12614700
C	-3.52923200	0.01185400	3.56295200
C	-4.16277600	0.38789900	2.38835900
N	-3.33275000	0.15124200	1.34284300
N	-2.15931200	-0.37249600	1.81115200
Br	-5.91555100	1.13210200	2.22285200
Br	-4.20724400	0.09933000	5.34462600
Br	-0.85848100	-1.15337600	4.20262300
C	-1.91074300	-2.28397900	-2.02416200
C	-3.22164300	-2.72056900	-2.26446400
C	-4.00054400	-1.79462700	-1.58811400
N	-3.19072200	-0.88223000	-0.99702900
N	-1.88770100	-1.19681900	-1.26970400
Br	-5.90797000	-1.77220600	-1.46685000
Br	-3.80406200	-4.22151600	-3.28849700
Br	-0.28562800	-3.06135800	-2.67090800
C	-1.02361200	2.88604500	-0.95569300
C	-2.08689800	3.53806200	-1.59409400

C	-3.16263900	2.68719600	-1.38941800
N	-2.74918200	1.61223600	-0.67525000
N	-1.41288000	1.74265200	-0.41006400
Br	-4.95352900	2.94509200	-2.00370500
Br	-2.06620300	5.20635900	-2.51903200
Br	0.79003100	3.46435700	-0.80510700
B	-3.57031600	0.38489200	-0.17817500
Ag	-0.21289800	-0.18633600	0.26433900
C	2.73555200	-3.32168600	0.31504400
C	3.74565100	-4.05202400	-0.34271000
C	3.98633000	-5.38286800	-0.00270100
C	3.23175800	-6.01063200	0.99048800
C	2.22999800	-5.29464600	1.64966800
C	1.98562700	-3.96385300	1.31853600
H	4.35196900	-3.58827000	-1.11056200
H	4.77093900	-5.92939000	-0.51811800
H	3.42203600	-7.04876300	1.24718500
H	1.63386200	-5.77397000	2.42103400
H	1.20544200	-3.40600600	1.82388900
C	2.40669000	-1.89998000	0.02785500
H	-4.71853400	0.61237400	-0.34570900
C	2.99143300	-1.18127400	-1.00048300
F	3.62747600	-1.70750400	-2.04416300
F	2.60403000	0.06035700	-1.28401700
O	1.69722600	-1.22715600	0.89300600
C	5.04489200	4.01872600	0.06595500
C	4.13574200	3.96184700	1.13427300
C	3.73451700	2.73740800	1.66723800
C	4.26561000	1.56260600	1.12794700
C	5.19333500	1.64483400	0.06773000

C	5.58673200	2.85942000	-0.49071300
H	5.33447500	4.98340600	-0.33944700
H	3.73750500	4.88348100	1.54749900
H	3.02392200	2.69862200	2.48736900
H	6.26738400	2.90533700	-1.33128600
C	4.08424300	0.14635900	1.45161500
C	4.89271100	-0.57182900	0.53194000
H	5.18125400	-1.61166600	0.54941900
N	5.57973000	0.31911800	-0.25992600
C	3.19103800	-0.45239200	2.31738400
H	3.29834600	-1.49165100	2.59708900
H	2.53261400	0.14852700	2.93430000
S	6.86670900	-0.17055900	-1.32491900
O	6.71109800	-1.62038500	-1.41684900
O	6.77719900	0.70439000	-2.48889900
C	10.19376100	1.67312800	0.04834800
C	10.66824300	0.79283600	1.03026800
C	9.95458600	-0.39426700	1.26922700
C	8.79976600	-0.69675800	0.55728200
C	8.34705100	0.20827900	-0.40959000
C	9.03723200	1.39174400	-0.67722700
H	10.73754700	2.59078000	-0.15645000
H	10.31403600	-1.09308100	2.01951800
H	8.26864500	-1.62499900	0.73718100
H	8.68827900	2.07004500	-1.44712000
C	11.92909900	1.09184000	1.79951000
H	12.75233800	0.45113800	1.46019900
H	12.24204400	2.13110200	1.67076100
H	11.79551400	0.90346300	2.86983100

Int3'

Zero-point correction= 0.377809 (Hartree/Particle)

Thermal correction to Energy= 0.403809

Thermal correction to Enthalpy= 0.404753

Thermal correction to Gibbs Free Energy= 0.321310

Sum of electronic and zero-point Energies= -1804.575531

Sum of electronic and thermal Energies= -1804.549530

Sum of electronic and thermal Enthalpies= -1804.548586

Sum of electronic and thermal Free Energies= -1804.632030

C	4.30921100	1.00402300	0.03567900
C	5.66344700	1.24091200	-0.26623600
C	6.10464800	2.52758700	-0.56597800
C	5.21141200	3.60113200	-0.57725700
C	3.86581400	3.37523800	-0.28484300
C	3.41737900	2.09130500	0.01854700
H	6.37149100	0.42130400	-0.27505600
H	7.15324800	2.68989000	-0.79856900
H	5.56113000	4.60180100	-0.81341400
H	3.16066700	4.20164300	-0.28901300
H	2.37354000	1.91672300	0.25463500
C	3.77017400	-0.33259100	0.35987300
C	4.47906700	-1.40111200	0.74803800
F	3.94035600	-2.58700600	1.01330300
F	5.79411000	-1.43542500	0.93602800
C	-2.03837400	-3.78369000	1.28133400
C	-0.65906800	-3.87357300	1.54275600
C	0.24564100	-3.03497900	0.90274200
C	-0.24267000	-2.08837600	-0.01048600

C	-1.63603100	-2.00887200	-0.25436200
C	-2.55014900	-2.85547500	0.37826500
H	-2.72290800	-4.45698100	1.78865900
H	-0.29835100	-4.61089600	2.25375100
H	1.30935500	-3.09513700	1.10752000
H	-3.60938600	-2.80693500	0.15976200
C	0.40490900	-1.09303800	-0.83951000
C	-0.57383100	-0.44533900	-1.53115300
H	-0.50686100	0.34489000	-2.26348400
N	-1.82224800	-0.99663400	-1.21702200
C	1.87142800	-0.86291400	-0.96882600
H	2.38428100	-1.77349000	-1.30666000
H	2.07775400	-0.06791700	-1.69551400
O	2.39866100	-0.49875200	0.33263000
S	-3.29803500	-0.23960600	-1.65796000
O	-2.98275900	0.50492600	-2.87665800
O	-4.31169800	-1.29239900	-1.63502700
C	-4.63805600	1.43375800	1.77758200
C	-4.14738200	2.74600300	1.71131800
C	-3.39892100	3.12447400	0.58636000
C	-3.14141900	2.22596300	-0.44471400
C	-3.64105300	0.92477200	-0.34737800
C	-4.39357100	0.51878300	0.75690700
H	-5.22293600	1.12369000	2.63885200
H	-3.01558500	4.13842300	0.51474100
H	-2.57532000	2.53172300	-1.31732000
H	-4.78922600	-0.48862900	0.81170000
C	-4.44539100	3.73741100	2.80685800
H	-5.36775700	4.29067000	2.58920100
H	-4.58367500	3.23991900	3.77093700

H	-3.64226800	4.47269800	2.91114300

Int4'

Zero-point correction= 0.377913 (Hartree/Particle)

Thermal correction to Energy= 0.403819

Thermal correction to Enthalpy= 0.404764

Thermal correction to Gibbs Free Energy= 0.321621

Sum of electronic and zero-point Energies= -1804.576970

Sum of electronic and thermal Energies= -1804.551063

Sum of electronic and thermal Enthalpies= -1804.550119

Sum of electronic and thermal Free Energies= -1804.633262

C	4.33517400	-0.77696900	-0.25446900
C	4.65140200	-1.32544200	1.00156300
C	5.36885400	-2.51632200	1.09168000
C	5.77506100	-3.18870800	-0.06299600
C	5.45757000	-2.65768100	-1.31426800
C	4.74479800	-1.46416400	-1.41103100
H	4.32995400	-0.82460600	1.90738900
H	5.60268800	-2.92538400	2.07042900
H	6.33078500	-4.11874200	0.01240800
H	5.76931600	-3.17109500	-2.21949600
H	4.50757100	-1.04654000	-2.38381600
C	3.57655600	0.48210500	-0.41014300
C	3.56759300	1.49070100	0.47067100
F	4.27084700	1.52457900	1.59905300
F	2.88681400	2.61481800	0.30863700
O	2.89587500	0.69800200	-1.59283300
C	-1.69145500	3.88621800	-0.34489600

C	-0.68928100	3.94037000	-1.33033700
C	0.12471100	2.84239600	-1.58193100
C	-0.08258900	1.66482600	-0.84807200
C	-1.09452400	1.63322800	0.14337800
C	-1.90547600	2.73844100	0.41382400
H	-2.30409100	4.76302500	-0.15754400
H	-0.54222500	4.85870500	-1.89095100
H	0.91771800	2.89386700	-2.32106200
H	-2.65284400	2.71212900	1.19659900
C	0.56547200	0.36891100	-0.85645000
C	-0.04205500	-0.38909600	0.10037900
H	0.15452100	-1.40226600	0.41710600
N	-1.04298100	0.35524200	0.73530000
C	1.67664800	-0.07525300	-1.75050200
H	1.88620600	-1.13988700	-1.59854900
H	1.41398800	0.07300300	-2.80240500
S	-2.22427800	-0.36910900	1.74906100
O	-1.56270300	-1.54931000	2.30394000
O	-2.73606900	0.70340800	2.59998900
C	-5.58149800	-0.47049300	-0.51391400
C	-5.54214800	-1.74650800	-1.09475200
C	-4.46835700	-2.59265600	-0.77846500
C	-3.45923400	-2.18469300	0.08875700
C	-3.52511500	-0.90607700	0.64830500
C	-4.58327300	-0.04228100	0.35731500
H	-6.40724900	0.19703300	-0.74317900
H	-4.42408700	-3.58712500	-1.21350000
H	-2.64182300	-2.85126100	0.33965000
H	-4.63139900	0.93995900	0.81251000
C	-6.64498200	-2.21212300	-2.01071600

H	-7.43438600	-2.71883800	-1.44130300
H	-7.11021700	-1.37388200	-2.53704400
H	-6.27400700	-2.92279400	-2.75488100

TS3'

Zero-point correction= 0.375008 (Hartree/Particle)

Thermal correction to Energy= 0.400486

Thermal correction to Enthalpy= 0.401431

Thermal correction to Gibbs Free Energy= 0.320579

Sum of electronic and zero-point Energies= -1804.545814

Sum of electronic and thermal Energies= -1804.520335

Sum of electronic and thermal Enthalpies= -1804.519391

Sum of electronic and thermal Free Energies= -1804.600243

C	3.94684900	-0.52787600	-0.12547400
C	3.84307400	-1.67766400	0.68221900
C	4.60436400	-2.81042900	0.39769400
C	5.48321200	-2.82374900	-0.68676200
C	5.59682600	-1.68867500	-1.49250900
C	4.83798900	-0.55508700	-1.21550900
H	3.16555500	-1.69840300	1.52545000
H	4.50699700	-3.68850600	1.02973800
H	6.07502300	-3.70906600	-0.90064100
H	6.28084000	-1.68517800	-2.33654300
H	4.92917400	0.33453200	-1.82858200
C	3.19117400	0.73516000	0.08907000
C	2.19593500	0.87887800	1.07108700
F	2.10000700	0.09762200	2.15357400
F	1.71956300	2.08856400	1.36385000

O	3.20075000	1.65359000	-0.81831900
C	-2.43746800	3.78255800	-0.00020700
C	-1.68318500	4.21552700	-1.10137600
C	-0.63472900	3.44302400	-1.59469100
C	-0.34947200	2.21803700	-0.98249400
C	-1.12689900	1.79746200	0.11955800
C	-2.16682500	2.57043200	0.63465500
H	-3.24390400	4.40546300	0.37480100
H	-1.91437500	5.16857400	-1.56731300
H	-0.04588500	3.78606400	-2.44015600
H	-2.72712300	2.25355500	1.50512600
C	0.64565900	1.18687700	-1.25166400
C	0.49634400	0.21055700	-0.23165500
H	0.82112400	-0.81954500	-0.24549500
N	-0.61869800	0.54796200	0.55075900
C	1.75795900	1.23827300	-2.09086400
H	2.24088800	0.32041500	-2.40429100
H	1.87850000	2.07741900	-2.76721600
S	-1.43776300	-0.60340500	1.52916200
O	-0.45429600	-1.66170700	1.75375300
O	-2.03461200	0.14903600	2.63072000
C	-5.03788200	-1.21032600	-0.22633100
C	-4.78168300	-2.23613000	-1.14537400
C	-3.48076400	-2.76164100	-1.21666300
C	-2.46133000	-2.27528900	-0.40619200
C	-2.74606700	-1.24243800	0.49326400
C	-4.03044800	-0.70679000	0.59499900
H	-6.04102000	-0.80177200	-0.14556400
H	-3.26680000	-3.56876400	-1.91183800
H	-1.46621100	-2.70312100	-0.45543500

H	-4.24238300	0.07401200	1.31573800
C	-5.86887900	-2.76424900	-2.04599200
H	-5.87684700	-3.85910200	-2.06020000
H	-6.85638000	-2.41937300	-1.72884500
H	-5.71547200	-2.43013600	-3.07942600

3

Zero-point correction= 0.376802 (Hartree/Particle)

Thermal correction to Energy= 0.402647

Thermal correction to Enthalpy= 0.403591

Thermal correction to Gibbs Free Energy= 0.321721

Sum of electronic and zero-point Energies= -1804.602225

Sum of electronic and thermal Energies= -1804.576379

Sum of electronic and thermal Enthalpies= -1804.575435

Sum of electronic and thermal Free Energies= -1804.657305

C	-3.79968400	0.26538200	-0.21992200
C	-3.38116900	1.30955200	0.62590500
C	-4.04676400	2.53474300	0.59999100
C	-5.12938100	2.73268300	-0.25783700
C	-5.55400800	1.69847900	-1.09804900
C	-4.89539000	0.47515100	-1.07828400
H	-2.55553800	1.17331300	1.31319100
H	-3.72034400	3.33332900	1.25914100
H	-5.64410100	3.68907700	-0.27107900
H	-6.39720900	1.84911600	-1.76540800
H	-5.21150300	-0.33871000	-1.72166900
C	-3.15086800	-1.07671500	-0.28153700
C	-1.83327100	-1.34583600	0.50286800

F	-2.00648000	-0.94548500	1.80336700
F	-1.62412600	-2.68889100	0.53542900
O	-3.59643600	-1.98678000	-0.95690300
C	3.25902800	-3.37431000	0.06316800
C	2.89328100	-3.64677600	-1.25991500
C	1.78863300	-3.01400600	-1.83155100
C	1.06289000	-2.09808300	-1.06933500
C	1.44683000	-1.83093200	0.25583300
C	2.53611200	-2.46845200	0.84512300
H	4.11134200	-3.88530400	0.50096400
H	3.46333900	-4.36554200	-1.84032800
H	1.49226900	-3.23627600	-2.85231100
H	2.80488400	-2.27016100	1.87492200
C	-0.12267100	-1.29766300	-1.39897400
C	-0.57209800	-0.65647200	-0.08524800
H	-0.78429800	0.40815800	-0.18815400
N	0.55491500	-0.88846200	0.85145000
C	-0.71103300	-1.13062800	-2.58785900
H	-1.57177100	-0.48439500	-2.72192800
H	-0.34563400	-1.64322500	-3.47240300
S	1.14472200	0.43918300	1.75813600
O	-0.04245400	1.22585900	2.10352300
O	2.01116700	-0.13636800	2.78772200
C	4.27388200	1.88061000	-0.38645100
C	3.71661700	2.96140800	-1.08872400
C	2.36265700	3.25995900	-0.88730800
C	1.57766300	2.50384000	-0.01841900
C	2.15714200	1.43030800	0.66122600
C	3.50887400	1.11447900	0.48685200
H	5.32521700	1.64070800	-0.52079700

H	1.91581300	4.10055700	-1.41051600
H	0.53729000	2.75805200	0.14976300
H	3.95515800	0.29490700	1.03741500
C	4.56752300	3.78405200	-2.02275400
H	3.98112500	4.55679600	-2.52638300
H	5.38111000	4.27931400	-1.48009200
H	5.03136800	3.15564300	-2.79134200

2aa

Zero-point correction= 0.279500 (Hartree/Particle)

Thermal correction to Energy= 0.297565

Thermal correction to Enthalpy= 0.298509

Thermal correction to Gibbs Free Energy= 0.233538

Sum of electronic and zero-point Energies= -1297.524282

Sum of electronic and thermal Energies= -1297.506217

Sum of electronic and thermal Enthalpies= -1297.505273

Sum of electronic and thermal Free Energies= -1297.570244

O	-3.38715300	2.92617400	-0.82480700
H	-4.11977400	3.55750900	-0.83627900
C	-2.09335700	-2.42605900	-1.77190700
C	-3.11398400	-1.54930600	-2.18234900
C	-3.32374000	-0.34134600	-1.52757800
C	-2.49493600	-0.00460100	-0.44690600
C	-1.46815700	-0.89899100	-0.05501900
C	-1.25649400	-2.11889000	-0.70202200
H	-1.95515600	-3.36743600	-2.29543100
H	-3.74570000	-1.82311100	-3.02227700
H	-4.10031500	0.34489100	-1.84862100

H	-0.48769100	-2.80655800	-0.37295300
C	-2.44696800	1.13827500	0.44199200
C	-1.41844700	0.92848000	1.30902000
H	-1.06313300	1.52924400	2.13226000
N	-0.82080600	-0.31428300	1.05092400
C	-3.39540500	2.29332300	0.45859000
H	-4.40859000	1.93374000	0.69938500
H	-3.10423500	2.99507500	1.25496200
S	0.69218800	-0.76378800	1.71751100
O	0.79495500	0.00953600	2.95504000
O	0.71875300	-2.22570400	1.71665900
C	3.40686600	-0.56013000	-1.28043800
C	3.81716000	0.77908600	-1.25666800
C	3.25791700	1.63377800	-0.29216400
C	2.31170800	1.17397600	0.61655500
C	1.91523900	-0.16603100	0.56051500
C	2.45824900	-1.04180700	-0.37977500
H	3.83736800	-1.23950200	-2.01036800
H	3.57402000	2.67241000	-0.24984500
H	1.89928800	1.83844400	1.36756800
H	2.15844200	-2.08306000	-0.39426000
C	4.82836200	1.30186700	-2.24488100
H	5.31502600	0.48932700	-2.79044600
H	4.34991600	1.95860400	-2.98161600
H	5.60463400	1.89120800	-1.74565800
